# Causal relationship between plasma metabolites and hypertension: A Mendelian randomization study

**DOI:** 10.1097/MD.0000000000045077

**Published:** 2025-10-03

**Authors:** Fan Yang, Yuan Wu, Haixiang Zhu, Xiaojian Ye, Leiwen Tang

**Affiliations:** a Department of Cardiovascular Medicine, Sir Run-Run Shaw Hospital, Zhejiang University School of Medicine, Hangzhou, Zhejiang, China; b Department of Nursing, The Second Affiliated Hospital, Zhejiang University School of Medicine, Hangzhou, Zhejiang, China.

**Keywords:** causal effect, hypertension, Mendelian randomization, plasma metabolites

## Abstract

Observational studies have indicated an association between metabolites and hypertension. However, establishing a causal relationship remains challenging. This study aims to investigate this causal relationship using Mendelian randomization methodology. Genome-wide association study summary statistics for plasma metabolites were obtained from 3 studies, encompassing over 2000 varieties. Genome-wide association study summary statistics for hypertension were derived from 2 studies, with a sample size of 122,996 and 129,909, respectively. The main method used was the inverse variance weighting (IVW) method, and the results were adjusted using Bonferroni multiple correction. The IVW method estimated effects between the levels of taurochenodeoxycholate, 1-dihomo-linolenoyl-GPC (20:3n3 or 6), N-acetylphenylalanine, and dihomo-linolenate (20:3n3 or n6) and hypertension are 1.041 (95% confidence interval [CI] 1.022–1.059, *P* = 1.49e−05), 1.023 (95% CI 1.015–1.031, *P* = 5.32e−08), 1.029 (95% CI 1.021–1.037, *P* = 6.10e−12), and 1.019 (95% CI 1.010–1.028, *P* = 1.54e−05), respectively. The IVW method estimated effects between the levels of 2-hydroxyoctanoate, 1-docosapentaenoyl-GPC (22:5n3), X-11538, gamma-glutamyl-alpha-lysine, and 2-butenoylglycine and hypertension are 0.954 (95% CI 0.936–0.972, *P* = 8.87e−07), 0.981 (95% CI 0.973–0.989, *P* = 4.14e−06), 0.981 (95% CI 0.975–0.987, *P* = 7.53e−10), 0.942 (95% CI 0.922–0.963, *P* = 8.71e−08), and 0.930 (95% CI 0.902–0.958, *P* = 2.22e−06), respectively. In summary, there are indications that some metabolites have a causal relationship with hypertension. It is necessary to conduct further investigations to comprehend the underlying biological mechanisms that support these associations.

## 1. Introduction

Hypertension is a chronic pathological condition and a major risk factor for cardiovascular diseases. The incidence of hypertension in adults is approximately 24%, affecting around 1 billion individuals globally, with 9 million deaths attributed to hypertension each year.^[1–3]^

Hypertension is increasingly being recognized as a metabolic disorder.^[4,5]^ Animal studies have indicated that changes in several metabolites are associated with hypertension,^[6,7]^ and similar findings have been observed in human studies.^[8]^ For example, ceramides can mediate vascular dysfunction by inhibiting the endothelial nitric oxide synthase-serine/threonine protein kinase-heat shock protein 90 signal complex^[9]^; diacylglycerols 16:0/22:5 and diacylglycerols 16:0/22:6 species were significantly associated with systolic blood pressure (SBP), diastolic blood pressure (DBP), and mean arterial pressure in a high-risk Mexican American population^[10]^; higher levels of androgenic steroids were consistently associated with elevated systolic and DBP during both early and late adolescence.^[11]^ However, traditional observational studies are often hindered by reverse causality and confounding factors, which complicate the determination of causal relationships.

To overcome these limitations, Mendelian randomization (MR) is utilized in genetic epidemiology, which employs genetic variations such as single-nucleotide polymorphisms (SNPs) as instrumental variables (IVs) for mutable disease risk factors or exposures. This design strengthens the inference of causality in exposure-outcome associations. To examine the association between metabolites and hypertension, as well as to assess the directionality of these associations, we conducted a 2-sample MR study utilizing the latest genome-wide association studies (GWAS).

## 2. Method

### 2.1. Study design

The current study adopts an MR design to investigate the causal effects of plasma metabolites on the risk of hypertension. Plasma metabolite data from 3 sources were utilized as exposures, while hypertension data from the FinnGen cohort were used as the outcome. Additionally, the hypertension data from the UK Biobank cohort were employed for replication analysis. This investigation relies on publicly available summary data from GWAS, and the original studies provide information about the obtained ethical approvals. The overview of the research information is presented in Table 1.

**Table 1:** Overview of the research information.

Type	Trait	Source	Sample size	N case*	N control†	Population
Exposure	1091 metabolites	CLSA cohort	8299			European
Exposure	644 metabolites	Long et al	1920			European
Exposure	452 metabolites	Shin et al	7824			European
Outcome	Hypertension	FinnGen	412,113	122,996	289,117	European
Outcome	Hypertension	UKB	484,598	129,909	354,689	European

CLSA = Canadian Longitudinal Study on Aging, UKB = UK Biobank.

* Number of cases.

† Number of controls.

### 2.2. Genetic associations with plasma metabolites

The data for plasma metabolites were obtained from 3 studies. The first study identified a total of 1091 blood metabolites.^[12]^ This particular study included 8299 individuals from the Canadian Longitudinal Study on Aging, with an average age of 62.4 years. Women constituted 50.90% of the cohort. Out of the 1091 blood metabolites identified, 850 were categorized into 8 super pathways: lipid, amino acid, xenobiotics, nucleotide, cofactor and vitamins, carbohydrate, peptide, and energy.

The second study, conducted by Long et al. performed an analysis on genetic variations discovered through whole-genome sequencing of 1960 individuals, primarily consisting of females (96% of the sample). In this study, a total of 644 metabolites were identified.^[13]^

Lastly, Shin et al conducted the third study, which involved genetic analysis of 7824 adult individuals from 2 European populations. This study identified 486 metabolites.^[14]^

### 2.3. Genetic associations with hypertension

The first summary data of GWAS for hypertension we obtained was from FinnGen, which combines imputed genotype data generated from newly collected and legacy samples in the Finnish Biobank with digital health record data from the Finnish National Institute for Health and Welfare (https://www.finngen.fi/en). This integration provides new insights into the genetics of the disease. The case sample size was 122,996, and the control sample size was 289,117.^[15]^

The summary data of GWAS for hypertension we obtained for replication analysis was analyzed by Handan et al, with a case sample size of 129,909 and a control sample size of 354,689. The population is derived from the UK Biobank, which includes genetic and health-related data from nearly half a million participants.^[16]^

### 2.4. Selection of genetic instruments

In this study, we aimed to enhance statistical efficiency by acquiring additional IVs. To achieve this, we identified independent SNPs associated with the exposure at a significance level of 5e−6. Independence was defined based on an *r*^2^ value below 0.001 and a distance exceeding 10,000 kb. We then extracted the SNPs from the outcomes data. Any SNPs that were not available in the outcomes data were subsequently excluded.

### 2.5. Statistical analysis

The primary analytical approach utilized in this study was the inverse variance weighting (IVW) method using random effects. This method employs a meta-analytic approach to combine the estimated effects of each SNP on exposure and outcome risks.^[17]^ In addition, several sensitivity analyses were conducted to assess the robustness of the association. Firstly, the association was estimated using a weighted median method, which assumes that at least 50% of the SNPs are valid IVs.^[18]^ Then, the presence of directional pleiotropy was determined by examining whether there was a statistically significant difference between the intercept and zero, using MR-Egger regression.^[19]^ Lastly, the MR-pleiotropy residual sum and outlier (MR-PRESSO) test was employed to identify potential outliers, and the results were adjusted by IVW after removing the outliers.^[20]^ We also conducted Steiger filtering to ensure the directionality of the association.^[21]^

The relationship between metabolites and hypertension was evaluated through odds ratios along with their corresponding 95% confidence intervals (CIs). The analysis was conducted utilizing R version 4.2.2 and the TwoSampleMR package version 0.5.8. To account for multiple testing, a Bonferroni correction was applied with a significance threshold set at *P* < .05/(1091 + 644 + 452), indicating statistically significant evidence for causality.

## 3. Results

### 3.1. An overview of IVs for metabolites

For IVs used for metabolites, all *F* statistics were >10, ranging from 10.1 to 99.3, indicating sufficient IVs strength (Table S1, Supplemental Digital Content, https://links.lww.com/MD/Q274). The proportion of exposure explained by IVs, as well as the heterogeneity and pleiotropy analysis and statistical power and directionality of the association, are also shown in Table S1, Supplemental Digital Content, https://links.lww.com/MD/Q274. The leave-one-out plots were presented in Figures S1 to S9, Supplemental Digital Content, https://links.lww.com/MD/Q273.

### 3.2. Associations of metabolites with hypertension

**Figure 1.**
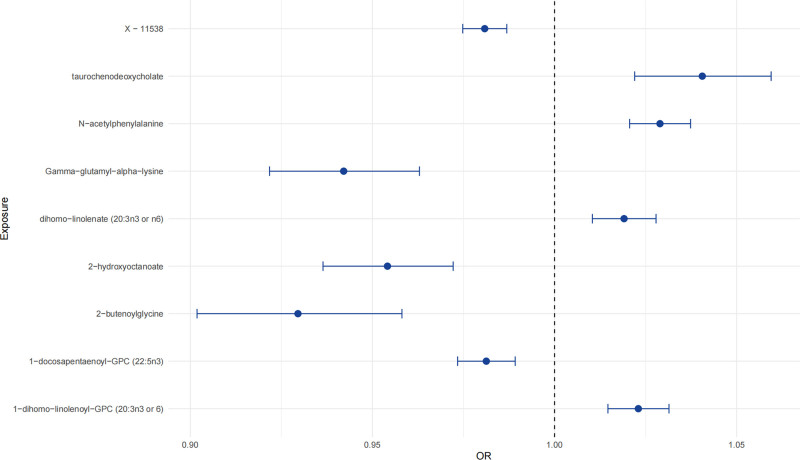
Forest plot for the effect of metabolites on hypertension. OR = odds ratio.

**Figure 2.**
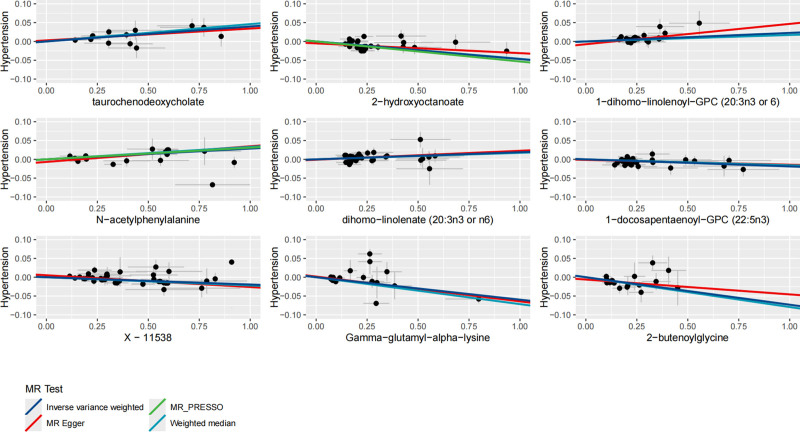
Scatterplot for the effect of metabolites on hypertension. MR = Mendelian randomization, MR-PRESSO = MR-pleiotropy residual sum and outlier.

After correcting the IVW *P*-values using the Bonferroni method in our analysis, we identified several statistically significant associations (Table 2). Notably, we did not observe any evidence of pleiotropy, and the statistical power of our analysis was sufficient. Furthermore, the identified associations were in the expected direction (Table 3; Table S2, Supplemental Digital Content, https://links.lww.com/MD/Q274). The forest plot and scatterplot were presented in Figure 1 and Figure 2, respectively.

**Table 2:** MR estimates for the effect of metabolites on hypertension.

Exposure	Outcome	nSNP	MR Egger		Weighted median		Inverse variance weighted		MR-PRESSO	
			OR (95% CI)	*P*	OR (95% CI)	*P*	OR (95% CI)	*P*	OR (95% CI)	*P*
Taurochenodeoxycholate	Hypertension	13	1.032 (0.990–1.076)	.165	1.047 (1.021–1.074)	3.43e−04	1.041 (1.022–1.059)	1.49e−05		
2-Hydroxyoctanoate	Hypertension	28	0.974 (0.917–1.035)	.408	0.955 (0.935–0.975)	9.82e−06	0.954 (0.936–0.972)	8.87e−07	0.948 (0.932–0.964)	1.42e−06
1-Dihomo-linolenoyl-GPC (20:3n3 or 6)	Hypertension	41	1.056 (0.998–1.116)	.064	1.017 (1.006–1.029)	.003	1.023 (1.015–1.031)	5.32e−08		
N-Acetylphenylalanine	Hypertension	24	1.042 (1.024–1.061)	1.77e−04	1.034 (1.025–1.043)	4.32e−13	1.029 (1.021–1.037)	6.1e−12	1.031 (1.025–1.038)	3.51e−09
Dihomo-linolenate (20:3n3 or n6)	Hypertension	51	1.024 (0.990–1.059)	.176	1.018 (1.005–1.030)	.005	1.019 (1.010–1.028)	1.54e-05		
1-Docosapentaenoyl-GPC (22:5n3)	Hypertension	45	0.987 (0.960–1.015)	.369	0.984 (0.974–0.995)	.005	0.981 (0.973–0.989)	4.14e−06		
X-11538	Hypertension	42	0.969 (0.956–0.983)	7.84e−05	0.978 (0.970–0.987)	1.02e−06	0.981 (0.975–0.987)	7.53e−10		
Gamma-glutamyl-alpha-lysine	Hypertension	18	0.936 (0.909–0.964)	4.89e−04	0.931 (0.894–0.969)	4.92e−04	0.942 (0.922–0.963)	8.71e−08		
2-Butenoylglycine	Hypertension	17	0.961 (0.889–1.039)	.335	0.924 (0.888–0.961)	6.87e−05	0.930 (0.902–0.958)	2.22e−06		

CI = confidence interval, MR = Mendelian randomization, MR-PRESSO = MR-pleiotropy residual sum and outlier, nSNP = number of single-nucleotide polymorphisms, OR = odds ratio.

**Table 3:** Instrumental variables strength and others used in MR analysis of the effect of metabolites on hypertension.

Exposure	Outcome	*R* ^2^	*F*	Heterogeneity	Pleiotropy	Power	Direction
Taurochenodeoxycholate	Hypertension	0.090	14	0.539	0.670	0.939	True
2-Hydroxyoctanoate	Hypertension	0.239	17	1.26e−05	0.479	1.000	True
1-Dihomo-linolenoyl-GPC (20:3n3 or 6)	Hypertension	0.485	23	1.000	0.272	0.996	True
N-acetylphenylalanine	Hypertension	0.912	80	0.029	0.131	1.000	True
Dihomo-linolenate (20:3n3 or n6)	Hypertension	0.434	17	0.943	0.773	0.955	True
1-Docosapentaenoyl-GPC (22:5n3)	Hypertension	0.436	19	0.987	0.652	0.956	True
X-11538	Hypertension	0.748	36	0.777	0.068	0.998	True
Gamma-glutamyl-alpha-lysine	Hypertension	0.053	25	0.446	0.533	0.982	True
2-Butenoylglycine	Hypertension	0.077	38	0.184	0.373	1.000	True

MR = Mendelian randomization.

#### 3.2.1. Positive associations of metabolites with hypertension

There is a positive correlation between the levels of taurochenodeoxycholate, 1-dihomo-linolenoyl-GPC (20:3n3 or 6), N-acetylphenylalanine, and dihomo-linolenate (20:3n3 or n6) and hypertension. The IVW method estimated effects are 1.041 (95% CI 1.022–1.059, *P* = 1.49e–05), 1.023 (95% CI 1.015–1.031, *P* = 5.32e–08), 1.029 (95% CI 1.021–1.037, *P* = 6.10e–12), and 1.019 (95% CI 1.010–1.028, *P* = 1.54e–05), respectively. The directions of the MR Egger and weighted median estimated effects are consistent with those of IVW. The MR PRESSO method detected outliers in the analysis of N-acetylphenylalanine to hypertension, but the results remained significant after correction (*P* = 3.51e–09).

#### 3.2.2. Negative associations of metabolites with hypertension

There is a negative correlation between the levels of 2-hydroxyoctanoate, 1-docosapentaenoyl-GPC (22:5n3), X-11538, gamma-glutamyl-alpha-lysine, and 2-butenoylglycine and hypertension. The IVW method estimated effects are 0.954 (95% CI 0.936–0.972, *P* = 8.87e–07), 0.981 (95% CI 0.973–0.989, *P* = 4.14e–06), 0.981 (95% CI 0.975–0.987, *P* = 7.53e–10), 0.942 (95% CI 0.922–0.963, *P* = 8.71e–08), and 0.930 (95% CI 0.902–0.958, *P* = 2.22e–06), respectively. The directions of the MR Egger and weighted median estimated effects are consistent with those of IVW. The MR PRESSO method detected outliers in the analysis of N-acetylphenylalanine to hypertension, but the results remained significant after correction (*P* = 1.42e–06). Unfortunately, the molecules X-11538 remain unidentified.

### 3.3. Replication analysis

The results of the replication analysis are presented in Tables S3 and S4, Supplemental Digital Content, https://links.lww.com/MD/Q274. The analysis of 1-dihomo-linolenoyl-GPC (20:3n3 or 6), N-acetylphenylalanine, and X-11538 in relation to hypertension is still significant (*P* < .05).

## 4. Discussion

This study employed an MR analysis to evaluate the causal association between blood metabolites and hypertension. Several metabolites, including 1-dihomo-linolenoyl-GPC (20:3n3 or 6), N-acetylphenylalanine, and X-11538, have been identified to possess a causal relationship with hypertension.

Previous observational studies have indicated the important role of metabolites in hypertension and have demonstrated that metabolites influence the development and progression of hypertension.^[22,23]^ However, these findings are susceptible to confounding factors, and the use of MR methods can greatly overcome these limitations. An MR study has analyzed the bidirectional causal relationship between several metabolites and DBP and SBP.^[24]^ Additionally, we conducted a replication analysis on hypertension and applied the more stringent Bonferroni correction compared to the false discovery rate, which yielded more comprehensive and rigorous findings.

N-acetylphenylalanine is the N-acetyl derivative of phenylalanine. Jenni et al reported a significant correlation between plasma levels of phenylalanine and N-acetylphenylalanine and the response of SBP and DBP to bisoprolol treatment compared to placebo before therapy, suggesting a potential role of N-acetylphenylalanine in hypertension.^[25]^ Parag et al discovered a positive association between N-acetylphenylalanine and higher levels of coronary artery calcium.^[26]^ These findings imply a possible involvement of N-acetylphenylalanine in hypertension.

One major strength of this study is its large-scale investigation into the causal relationship between metabolites and hypertension. This approach effectively reduces the potential problems associated with residual confounding and reverse causality inherent in observational studies. However, this study also has several limitations. Firstly, our analysis is restricted to cohorts primarily composed of European participants. Though this reduces potential population bias in our MR analysis, it restricts the generalizability of our findings to other populations. Secondly, the application of strict multiple testing correction may be excessively conservative, potentially overlooking some metabolites that are causally related to hypertension. Thirdly, the causal effects identified in the MR analysis represent cumulative effects over an individual’s lifetime, which may lead to an overestimation of the effect size when considering changes in exposure and outcomes in a clinical setting. Fourthly, the precise mechanisms through which these metabolites affect the risk of hypertension have not been thoroughly investigated in this study. Finally, the use of summary data from GWAS prevents us from conducting subgroup analysis or nonlinear MR analysis to improve the credibility of the findings.

## 5. Conclusions

In conclusion, there is evidence to suggest that several metabolites may be causally linked to hypertension. Further investigation is imperative to fully comprehend the underlying biological mechanisms that uphold these associations.

## Acknowledgments

We sincerely appreciate the original researchers of GWAS for sharing their data.

## Author contributions

**Data curation:** Fan Yang, Yuan Wu.

**Formal analysis:** Fan Yang, Xiaojian Ye.

**Software:** Yuan Wu, Haixiang Zhu, Xiaojian Ye.

**Visualization:** Haixiang Zhu.

**Writing – original draft:** Fan Yang.

**Writing – review & editing:** Leiwen Tang.

## Supplementary Material
