# Supplementary material for: Causal relationship between plasma metabolites and hypertension: A Mendelian randomization study
Source: Medicine (Baltimore). 2025 Oct 3;104(40):e45077. doi: 10.1097/MD.0000000000045077 (PMC12499832; doi:10.1097/MD.0000000000045077)

Supplementary Figure 1. Leave-one-out plot in MR analysis of the effect of taurochenodeoxycholate on hypertension.

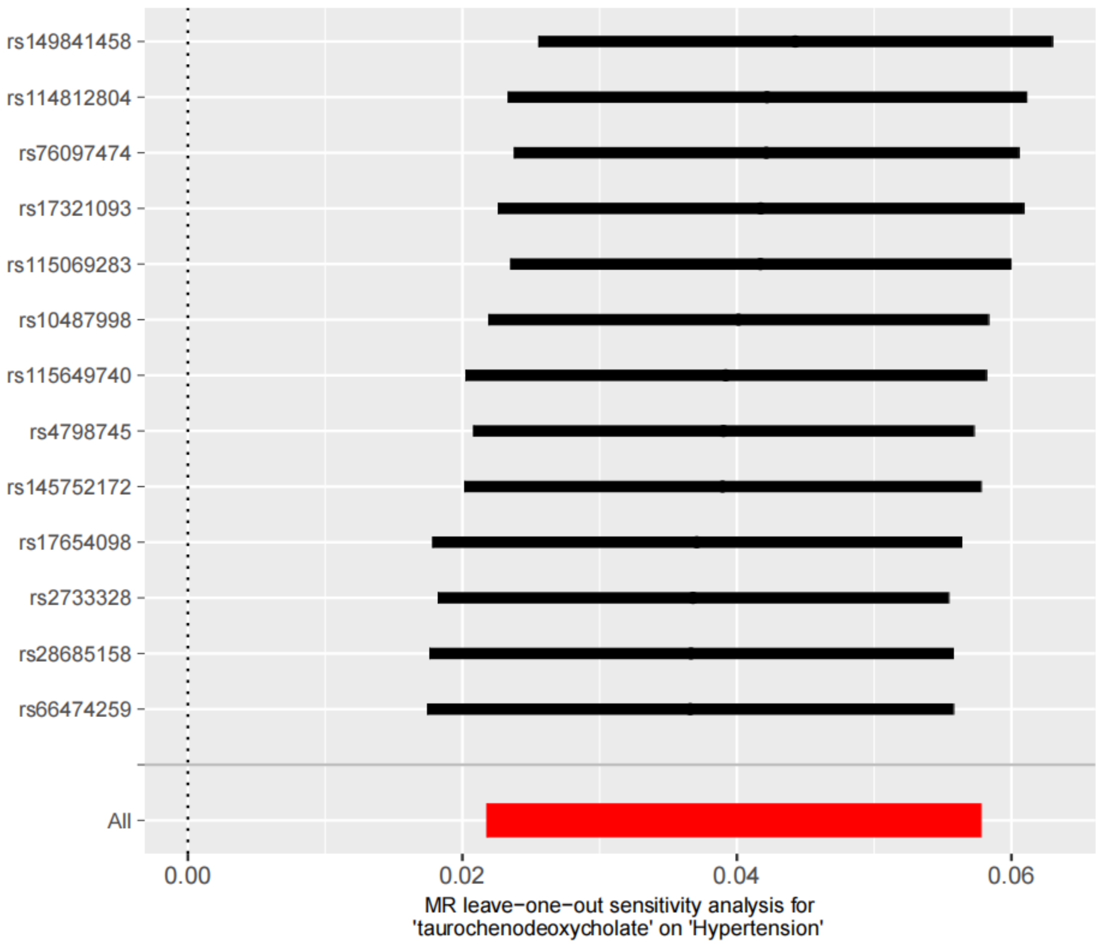

Supplementary Figure 2. Leave-one-out plot in MR analysis of the effect of 2-hydroxyoctanoate on hypertension.

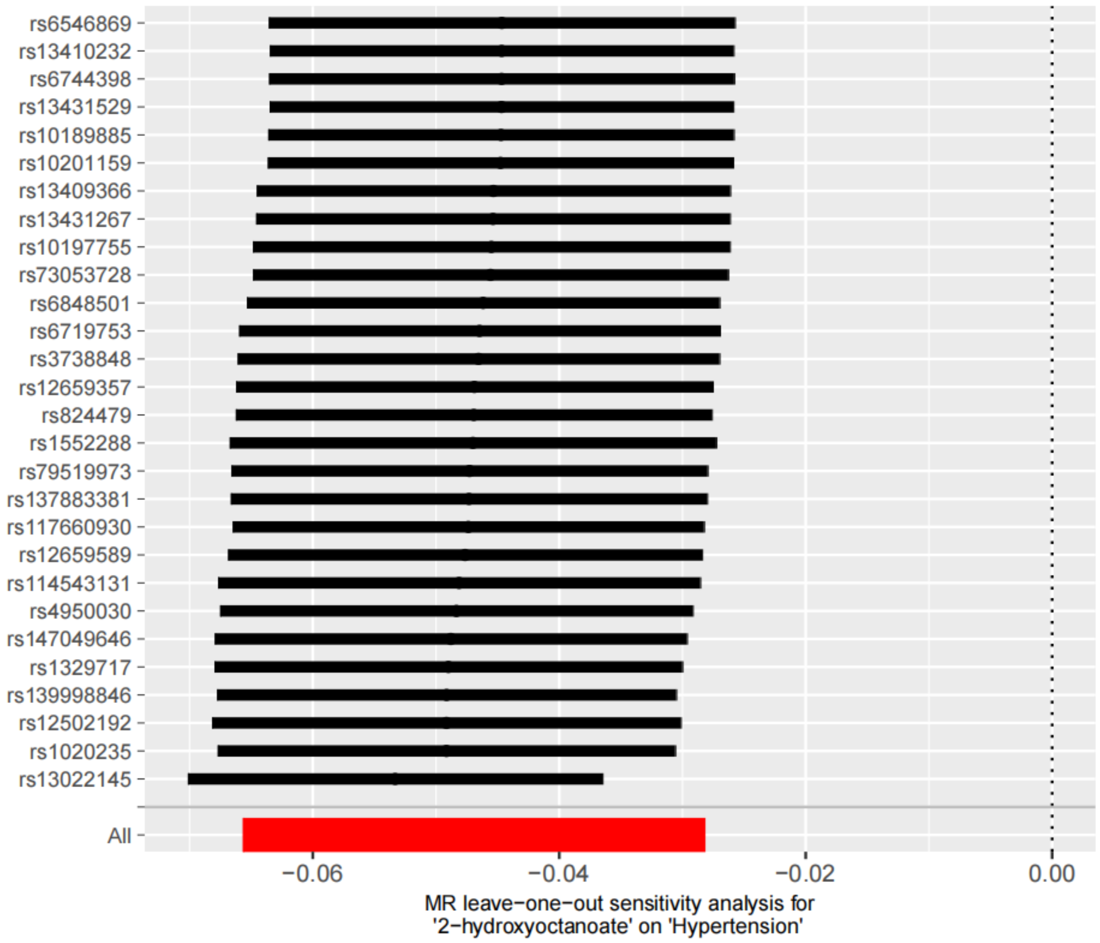

Supplementary Figure 3. Leave-one-out plot in MR analysis of the effect of 1-dihomo-linolenoyl-GPC (20:3n3 or 6) on hypertension.

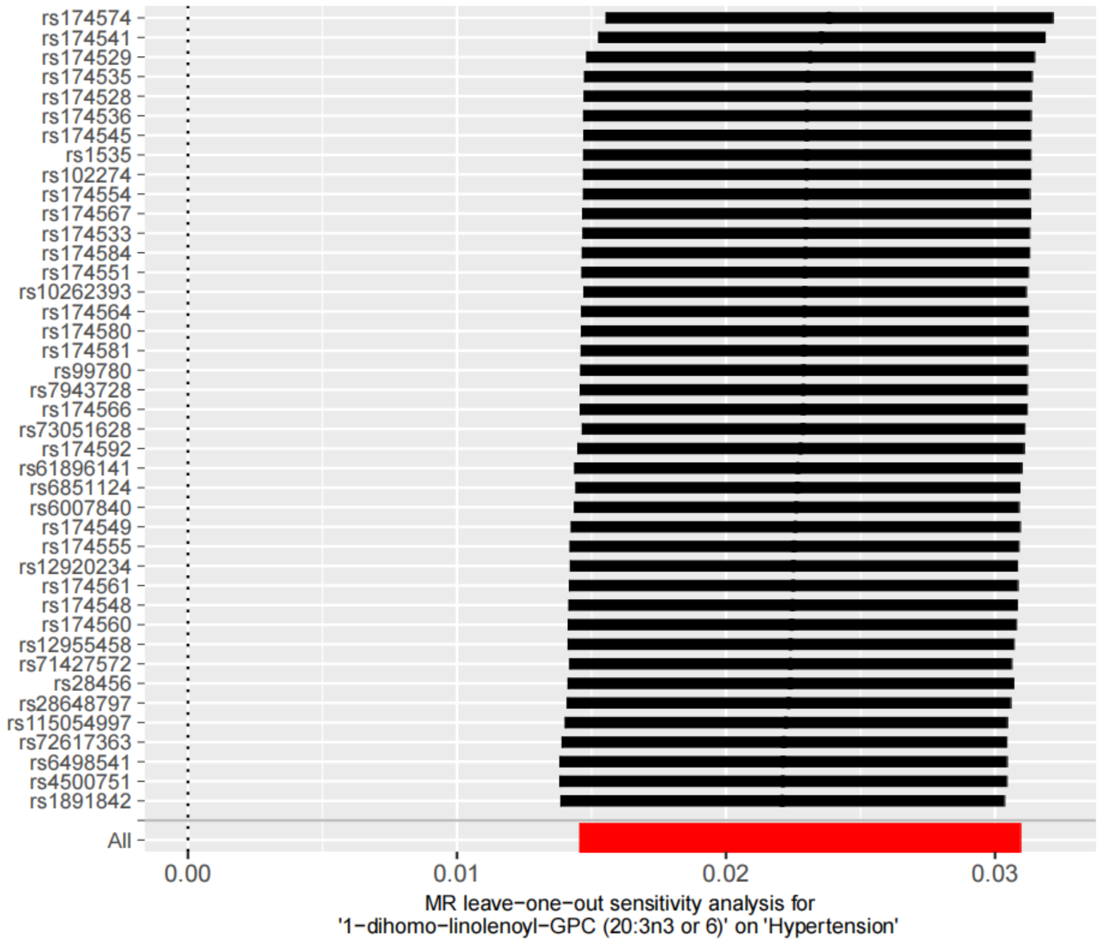

Supplementary Figure 4. Leave-one-out plot in MR analysis of the effect of N-acetylphenylalanine on hypertension.

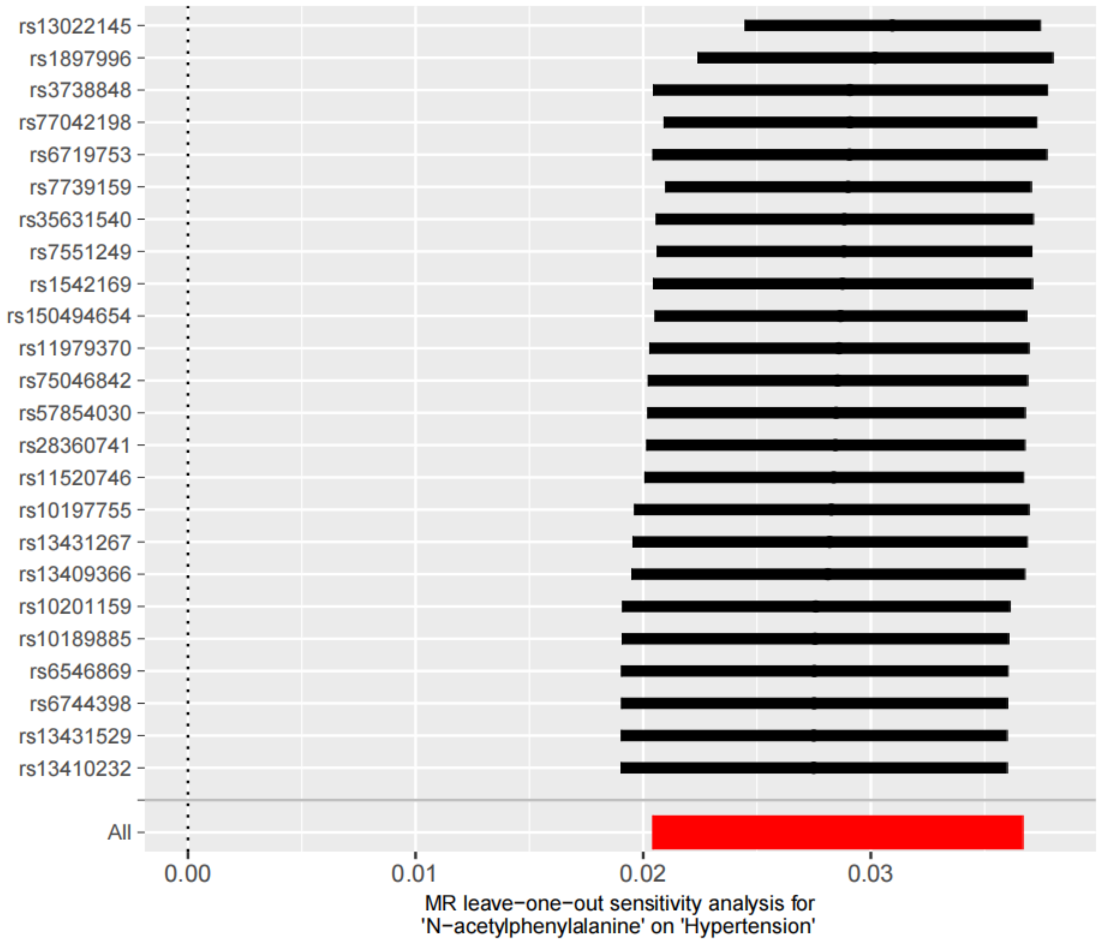

Supplementary Figure 5. Leave-one-out plot in MR analysis of the effect of dihomo-linolenate (20:3n3 or n6) on hypertension.

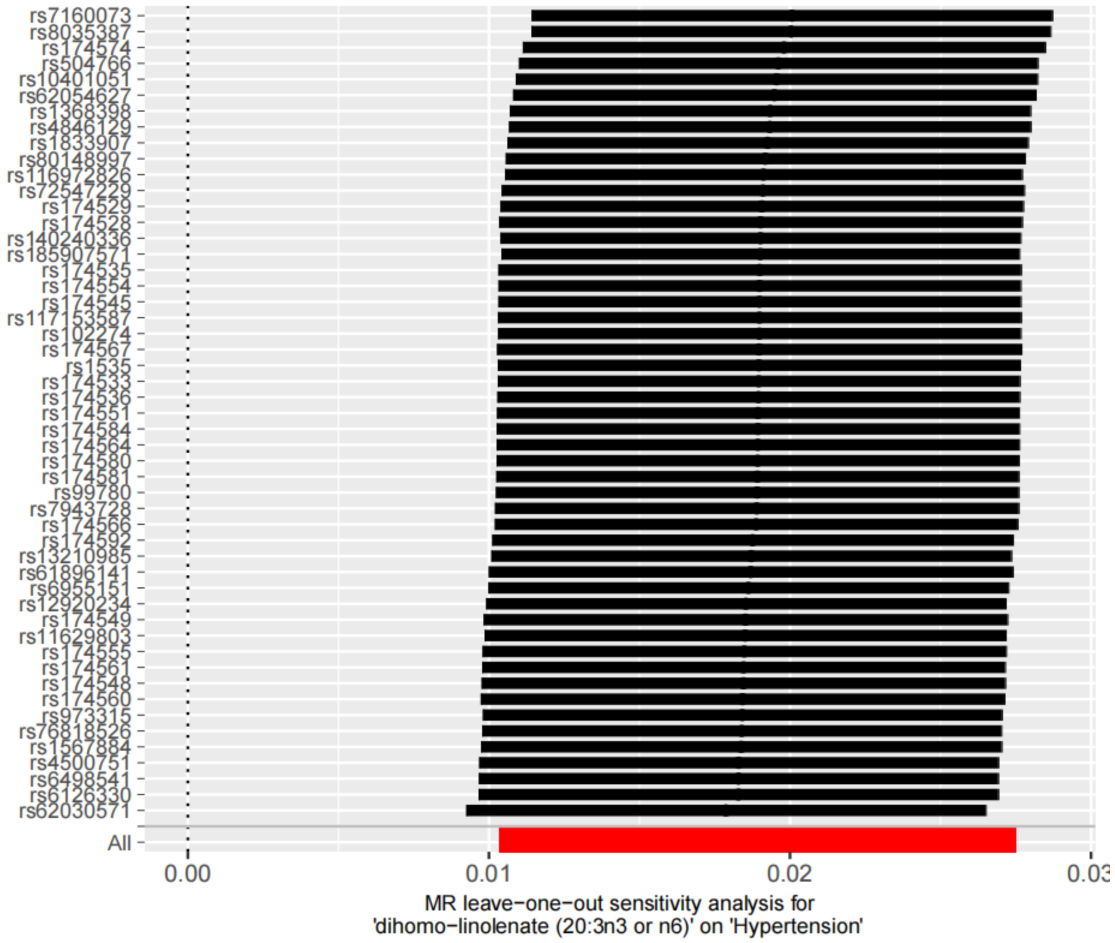

Supplementary Figure 6. Leave-one-out plot in MR analysis of the effect of 1-docosapentaenoyl-GPC (22:5n3) on hypertension.

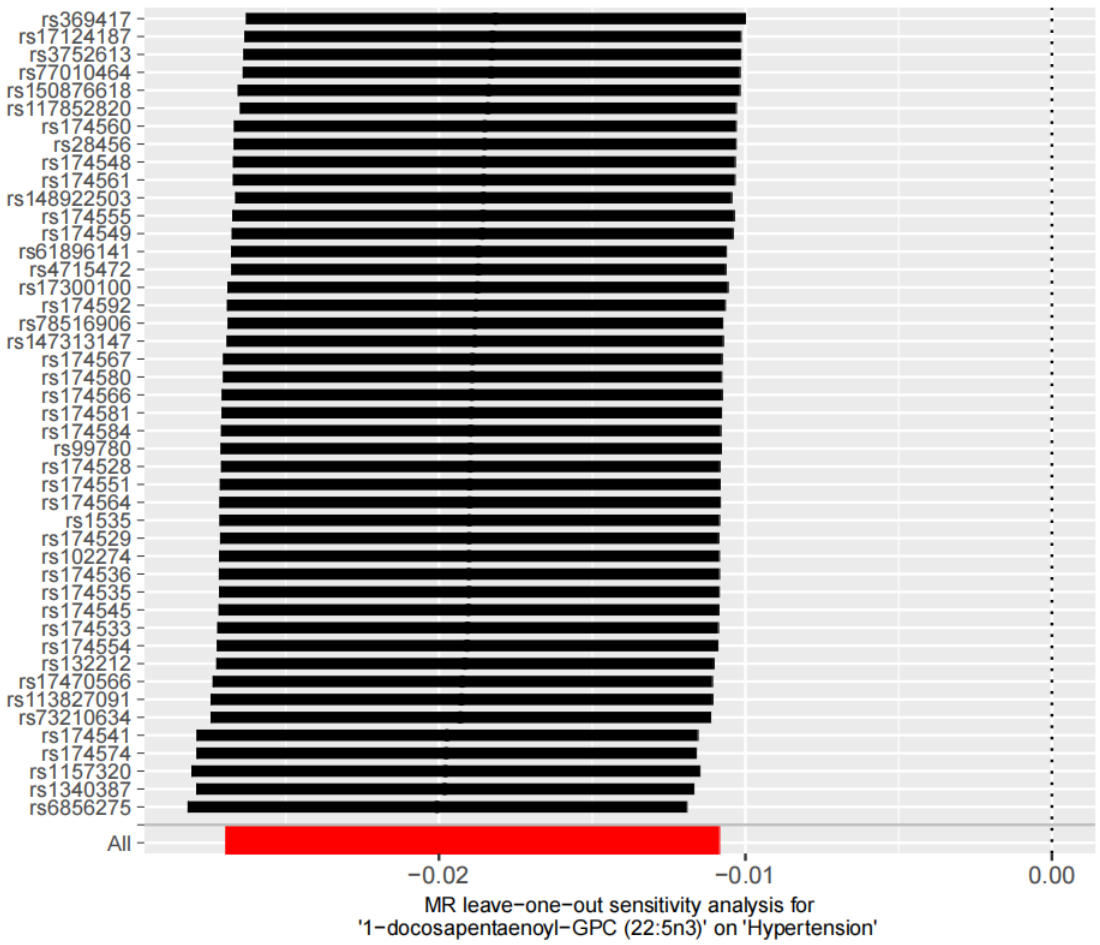

Supplementary Figure 7. Leave-one-out plot in MR analysis of the effect of X - 11538 on hypertension.

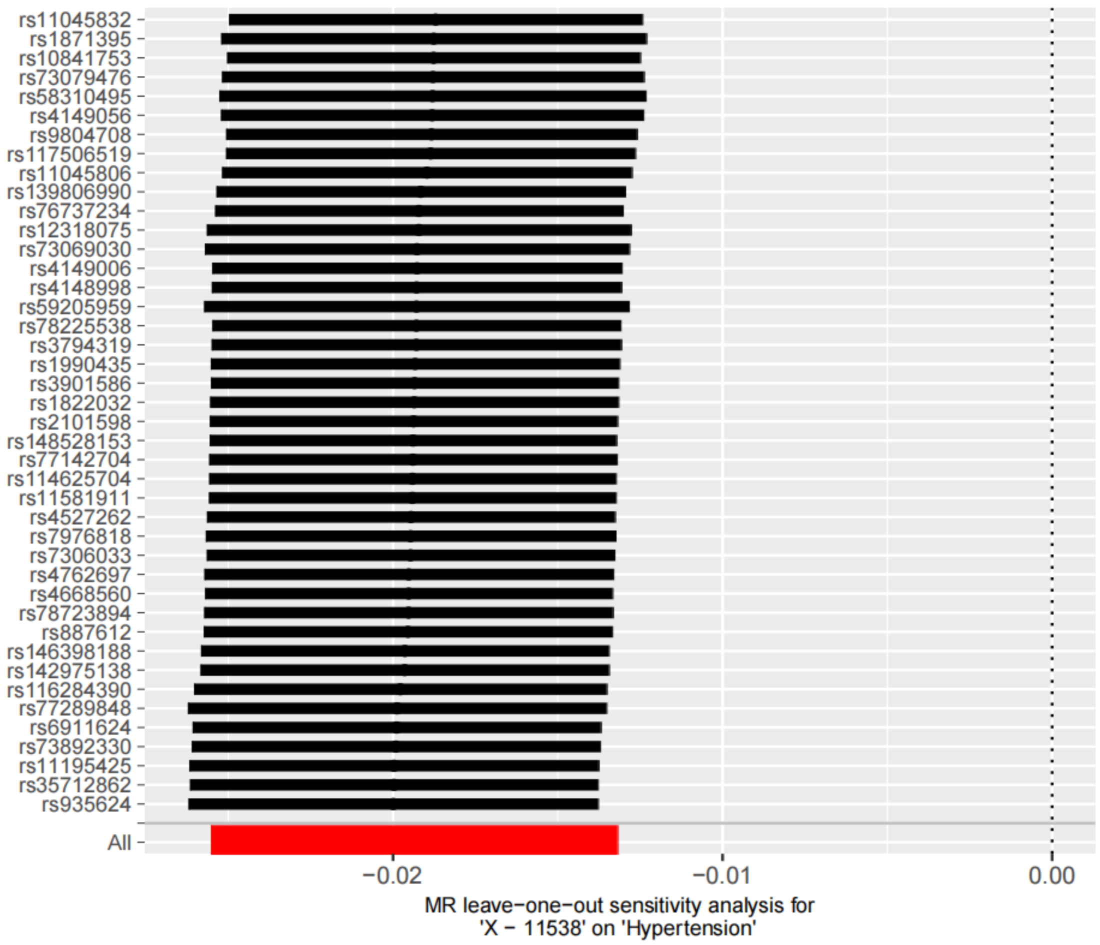

Supplementary Figure 8. Leave-one-out plot in MR analysis of the effect of Gamma-glutamyl-alpha-lysine on hypertension.

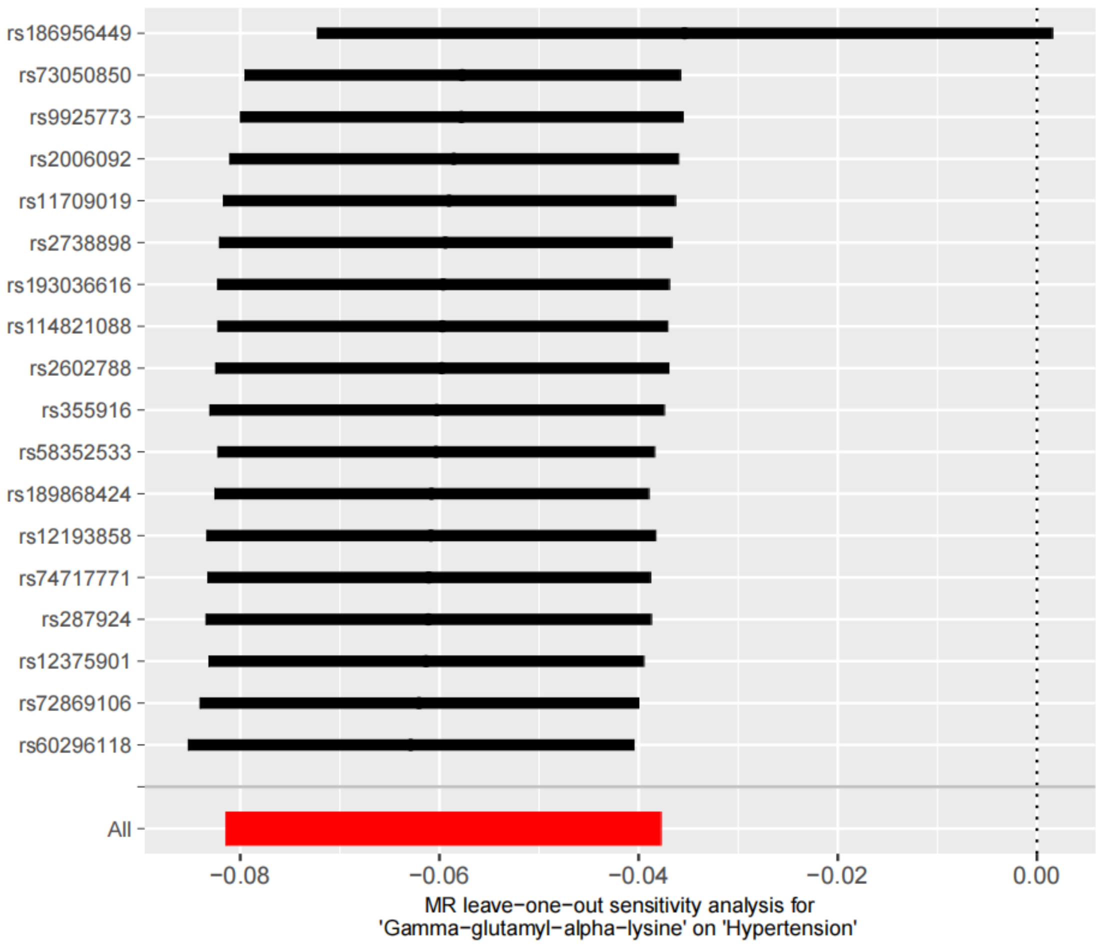

Supplementary Figure 9. Leave-one-out plot in MR analysis of the effect of 2-butenoylglycine on hypertension.

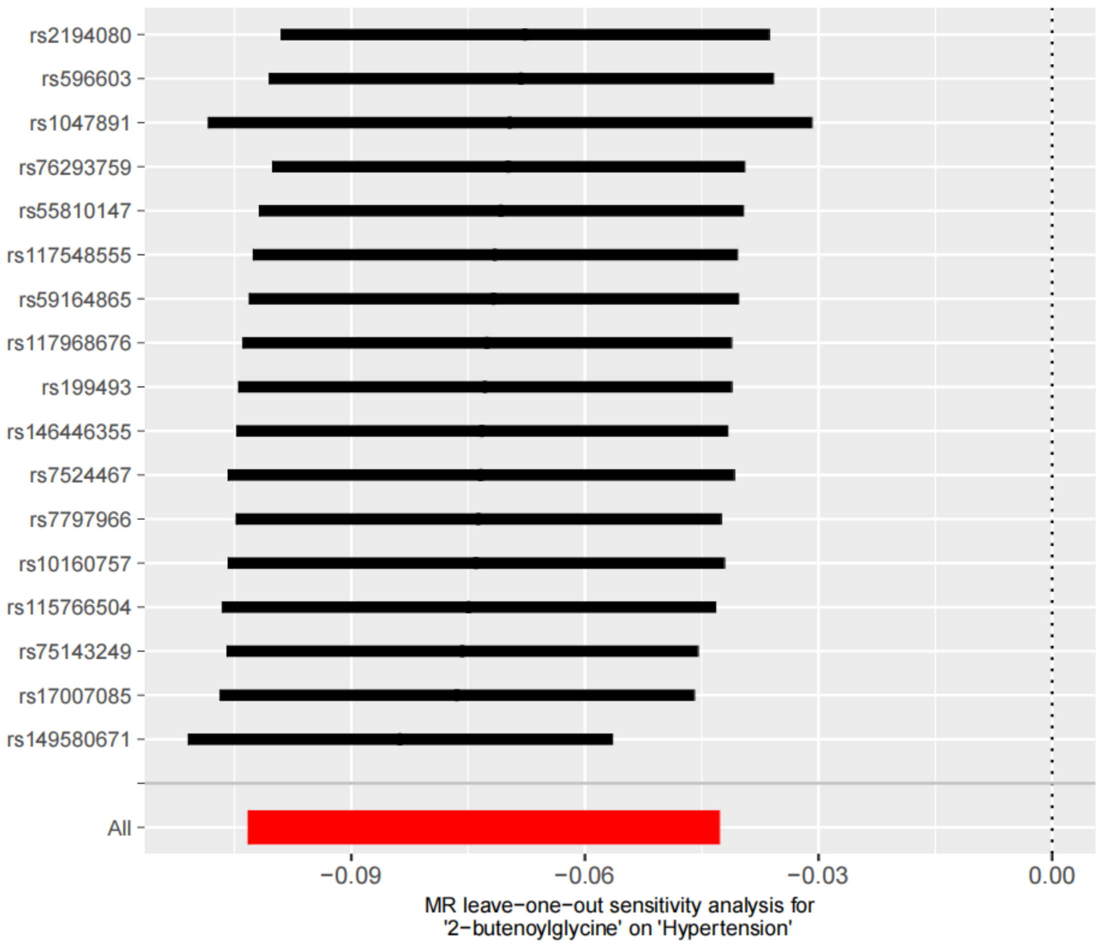

Supplement: Supplementary file 2 [file medi-104-e45077-s002.pdf]
