# Supplementary material for: Causal relationship between plasma metabolites and hypertension: A Mendelian randomization study
Source: Medicine (Baltimore). 2025 Oct 3;104(40):e45077. doi: 10.1097/MD.0000000000045077 (PMC12499832; doi:10.1097/MD.0000000000045077)
Supplement: Supplementary file 1 [file medi-104-e45077-s001.pdf]

Table S1. Instrumental variables strength and others used in MR analysis of the effect of metabolites on hypertension(FinnGen).

Exposure	Outcome	R2	F	Heterogeneity	Pleiotropy	power	Direction
arachidonate (20:4n6)	Hypertension	2	80	0.139	0.101	0.761	TRUE
deoxycholate	Hypertension	0.085	14	0.011	0.608	0.034	TRUE
arachidate (20:0)	Hypertension	0.097	14	0.012	0.428	0.047	TRUE
margarate (17:0)	Hypertension	0.073	13	0.089	0.654	0.142	TRUE
inosine	Hypertension	0.179	17	0.111	0.706	0.035	TRUE
myo-inositol	Hypertension	0.111	17	0.049	0.751	0.223	TRUE
isoleucine	Hypertension	0.097	15	0.674	0.549	0.077	TRUE
alanine	Hypertension	0.092	12	0.213	0.602	0.061	TRUE
3-methoxytyrosine	Hypertension	0.139	14	0.003	0.127	0.071	TRUE
beta-hydroxyisovalerate	Hypertension	0.180	15	0.615	0.253	0.214	TRUE
taurodeoxycholate	Hypertension	0.122	15	0.065	0.352	0.248	TRUE
threonine	Hypertension	0.107	16	3.28e-05	0.795	0.286	TRUE
tyrosine	Hypertension	0.110	14	0.005	0.666	0.073	TRUE
lysine	Hypertension	0.151	15	0.112	0.473	0.063	TRUE
malate	Hypertension	0.466	14	1.91e-04	0.080	0.167	TRUE
palmitate (16:0)	Hypertension	0.206	14	0.127	0.120	0.059	TRUE

Exposure	Outcome	R2	F	Heterogeneity	Pleiotropy	power	Direction
myristate (14:0)	Hypertension	0.208	16	0.503	0.904	0.081	TRUE
kynurenate	Hypertension	0.152	16	0.011	0.605	0.545	TRUE
5,6-dihydrothymine	Hypertension	0.213	17	0.571	0.536	0.202	TRUE
5-methylthioadenosine (MTA)	Hypertension	0.133	16	0.351	0.118	0.032	TRUE
2-hydroxyphenylacetate	Hypertension	0.154	15	4.36e-04	0.225	0.040	TRUE
succinate	Hypertension	0.170	16	0.044	0.783	0.047	TRUE
ornithine	Hypertension	0.136	15	0.037	0.387	0.057	TRUE
5-oxoproline	Hypertension	0.226	21	0.005	0.927	0.555	TRUE
N6,N6,N6-trimethyllysine	Hypertension	0.087	17	0.322	0.643	0.040	TRUE
orotate	Hypertension	0.302	35	0.013	0.793	0.118	TRUE
pantothenate	Hypertension	0.125	16	0.015	0.118	0.404	TRUE
glycerol	Hypertension	0.123	14	0.008	0.346	0.441	TRUE
kynurenine	Hypertension	0.120	15	1.9e-07	0.136	0.955	TRUE
sucrose	Hypertension	0.071	16	0.046	0.675	0.044	TRUE
tartarate	Hypertension	0.148	16	3.42e-04	0.775	0.080	TRUE
carnitine	Hypertension	0.304	17	0.031	0.054	0.200	TRUE
choline	Hypertension	0.152	16	0.535	0.787	0.055	TRUE

Exposure	Outcome	R2	F	Heterogeneity	Pleiotropy	power	Direction
4-acetamidobutanoate	Hypertension	0.187	22	0.160	0.778	0.083	TRUE
chenodeoxycholate	Hypertension	0.149	16	0.002	0.463	0.156	TRUE
citrate	Hypertension	0.119	15	6.48e-04	0.887	0.256	TRUE
N1-methyladenosine	Hypertension	0.104	13	0.174	0.662	0.053	TRUE
3-aminoisobutyrate	Hypertension	0.339	32	0.055	0.012	0.079	TRUE
2-isopropylmalate	Hypertension	0.153	16	0.027	0.589	0.250	TRUE
vanillylmandelate (VMA)	Hypertension	0.156	18	0.636	0.565	0.352	TRUE
3-methylhistidine	Hypertension	0.247	17	0.006	0.891	0.048	TRUE
xanthurenate	Hypertension	0.118	14	7.74e-07	0.057	0.177	TRUE
4-guanidinobutanoate	Hypertension	0.272	22	0.009	0.005	0.124	TRUE
5-hydroxylysine	Hypertension	0.304	19	0.001	0.736	0.027	TRUE
pimelate (heptanedioate)	Hypertension	0.125	16	0.386	0.240	0.078	TRUE
cystathionine	Hypertension	0.120	15	0.023	0.907	0.034	TRUE
imidazole lactate	Hypertension	0.610	60	8.8e-04	0.890	0.766	TRUE
glycerate	Hypertension	0.189	14	0.392	0.531	0.026	TRUE
guanosine	Hypertension	0.170	17	0.450	0.182	0.041	TRUE
suberate (octanedioate)	Hypertension	0.168	17	0.318	0.285	0.295	TRUE

Exposure	Outcome	R2	F	Heterogeneity	Pleiotropy	power	Direction
methylsuccinate	Hypertension	0.296	31	0.491	0.646	0.277	TRUE
hippurate	Hypertension	0.154	16	0.047	0.760	0.152	TRUE
ethylmalonate	Hypertension	2.7	67	0.027	0.821	0.129	TRUE
ribitol	Hypertension	0.272	19	0.015	0.113	0.909	TRUE
N-acetylalanine	Hypertension	0.145	14	0.438	0.652	0.033	TRUE
malonate	Hypertension	0.330	16	0.386	0.441	0.058	TRUE
N-acetylmethionine	Hypertension	0.149	15	6.58e-07	0.390	0.426	TRUE
phenylacetate	Hypertension	0.068	17	0.099	0.658	0.346	TRUE
urate	Hypertension	0.263	20	0.225	0.771	0.291	TRUE
ursodeoxycholate	Hypertension	0.144	17	0.012	0.473	0.031	TRUE
arginine	Hypertension	0.151	15	3.9e-08	0.807	0.416	TRUE
fumarate	Hypertension	0.126	14	0.001	0.620	0.028	TRUE
serine	Hypertension	0.166	19	0.039	0.435	0.470	TRUE
valine	Hypertension	0.122	15	0.110	0.848	0.037	TRUE
urea	Hypertension	0.099	15	0.316	0.423	0.065	TRUE
cortisol	Hypertension	0.109	14	0.066	0.679	0.182	TRUE
cortisone	Hypertension	0.201	17	5.76e-04	0.639	0.042	TRUE

Exposure	Outcome	R2	F	Heterogeneity	Pleiotropy	power	Direction
2-hydroxystearate	Hypertension	0.141	13	0.012	0.086	0.167	TRUE
retinol (Vitamin A)	Hypertension	0.131	15	0.131	0.611	0.627	TRUE
gamma-glutamylhistidine	Hypertension	0.117	15	0.967	0.533	0.064	TRUE
paraxanthine	Hypertension	0.195	18	0.610	0.948	0.772	TRUE
quinate	Hypertension	0.144	14	0.250	0.195	0.224	TRUE
indolelactate	Hypertension	0.246	15	7.4e-04	0.120	0.143	TRUE
gamma-glutamylleucine	Hypertension	0.191	17	0.075	0.055	0.085	TRUE
methionine sulfoxide	Hypertension	0.466	16	1.53e-04	0.635	0.147	TRUE
theobromine	Hypertension	0.138	15	3.99e-04	0.532	0.026	TRUE
theophylline	Hypertension	0.248	16	0.017	0.303	0.465	TRUE
eicosapentaenoate (EPA; 20:5n3)	Hypertension	0.428	17	0.417	0.092	0.758	TRUE
glycocholate	Hypertension	0.102	14	0.386	0.285	0.100	TRUE
taurochenodeoxycholate	Hypertension	0.090	14	0.539	0.670	0.939	TRUE
taurocholate	Hypertension	0.091	13	0.210	0.413	0.147	TRUE
proline	Hypertension	0.291	15	0.250	0.373	0.054	TRUE
1,2-dimyristoyl-GPC (14:0/14:0)	Hypertension	0.148	15	0.377	0.833	0.041	TRUE
quinolinate	Hypertension	0.227	14	2.16e-23	0.030	0.330	TRUE

Exposure	Outcome	R2	F	Heterogeneity	Pleiotropy	power	Direction
1,2-distearoyl-GPC (18:0/18:0)	Hypertension	0.098	8.7	3.12e-05	0.102	0.033	TRUE
1-myristoyl-2-palmitoyl-GPC (14:0/16:0)	Hypertension	0.128	13	0.002	0.635	0.029	TRUE
1-palmitoyl-2-oleoyl-GPE (16:0/18:1)	Hypertension	0.654	21	1.000	0.254	0.899	TRUE
2-arachidonoylglycerol (20:4)	Hypertension	0.491	24	0.636	0.766	0.401	TRUE
1-stearoyl-GPI (18:0)	Hypertension	0.240	15	0.009	0.078	0.065	TRUE
stearoyl sphingomyelin (d18:1/18:0)	Hypertension	0.204	14	0.032	0.677	0.094	TRUE
1-palmityl-GPC (O-16:0)	Hypertension	0.121	17	1.15e-04	0.218	0.219	TRUE
1,5-anhydroglucitol (1,5-AG)	Hypertension	0.208	14	6.84e-05	0.825	0.087	TRUE
oxalate (ethanedioate)	Hypertension	0.141	15	0.891	0.577	0.758	TRUE
erythritol	Hypertension	0.157	17	0.011	0.709	0.252	TRUE
glycylglycine	Hypertension	0.277	20	5.82e-06	0.899	0.271	TRUE
1-oleoylglycerol (18:1)	Hypertension	0.119	17	3.08e-09	0.900	0.110	TRUE
citrulline	Hypertension	0.098	13	0.433	0.813	0.092	TRUE
biliverdin	Hypertension	0.627	57	0.238	0.917	0.063	TRUE
3-hydroxyoctanoate	Hypertension	0.229	21	0.017	0.754	0.034	TRUE
2-hydroxyoctanoate	Hypertension	0.239	17	1.26e-05	0.479	1.000	TRUE
3-hydroxydecanoate	Hypertension	0.502	39	0.007	0.071	0.130	TRUE

Exposure	Outcome	R2	F	Heterogeneity	Pleiotropy	power	Direction
phenyllactate (PLA)	Hypertension	0.356	32	0.003	0.837	0.161	TRUE
alpha-hydroxyisocaproate	Hypertension	0.101	13	0.023	0.698	0.453	TRUE
homoarginine	Hypertension	0.286	29	6.59e-08	0.471	0.066	TRUE
homocitrulline	Hypertension	0.097	24	0.339	0.530	0.056	TRUE
N-acetylaspartate (NAA)	Hypertension	0.371	16	3.81e-06	0.815	0.155	TRUE
pyroglutamylglutamine	Hypertension	0.167	17	0.574	0.017	0.328	TRUE
cholate	Hypertension	0.076	11	1.9e-05	0.409	0.148	TRUE
gamma-glutamylglutamine	Hypertension	0.157	18	0.001	0.589	0.090	TRUE
gamma-glutamyltyrosine	Hypertension	0.095	14	0.199	0.956	0.118	TRUE
1-linoleoylglycerol (18:2)	Hypertension	0.212	16	0.012	0.163	0.781	TRUE
thyroxine	Hypertension	0.087	13	0.023	0.277	0.066	TRUE
1-methylnicotinamide	Hypertension	0.097	14	0.855	0.530	0.357	TRUE
3-indoxyl sulfate	Hypertension	0.058	13	0.085	0.790	0.102	TRUE
N-acetylglycine	Hypertension	0.294	31	1.05e-04	0.359	0.265	TRUE
creatine	Hypertension	0.165	19	0.099	0.108	0.944	TRUE
galactonate	Hypertension	0.151	15	3.98e-07	0.732	0.693	TRUE
1-methylhistidine	Hypertension	0.155	15	0.001	0.947	0.131	TRUE

Exposure	Outcome	R2	F	Heterogeneity	Pleiotropy	power	Direction
hypoxanthine	Hypertension	0.158	15	8.22e-21	0.799	0.500	TRUE
betaine	Hypertension	0.167	16	0.002	0.207	0.061	TRUE
xanthine	Hypertension	0.099	13	0.001	0.789	0.852	TRUE
pyroglutamylglycine	Hypertension	0.127	14	0.002	0.389	0.118	TRUE
DSGEGDFXAEGGGVR	Hypertension	0.253	15	2.36e-28	0.425	0.999	TRUE
3-ureidopropionate	Hypertension	0.066	10	0.093	0.651	0.036	TRUE
pyridoxate	Hypertension	0.179	14	0.214	0.119	0.110	TRUE
androsterone sulfate	Hypertension	0.762	41	1.22e-04	0.322	0.131	TRUE
2-methylbutyrylglycine	Hypertension	0.146	15	0.238	0.524	0.349	TRUE
propionylglycine	Hypertension	0.344	26	4.25e-05	0.074	0.742	TRUE
5-hydroxyhexanoate	Hypertension	0.104	16	0.605	0.552	0.132	TRUE
3-hydroxysebacate	Hypertension	0.371	26	0.043	0.947	0.062	TRUE
3-(4-hydroxyphenyl)lactate	Hypertension	0.134	16	0.002	0.054	0.039	TRUE
acetylcarnitine	Hypertension	0.198	14	0.022	0.502	0.029	TRUE
trans-4-hydroxyproline	Hypertension	0.120	14	0.006	0.233	0.693	TRUE
hexanoylcarnitine	Hypertension	0.536	35	3.53e-04	0.113	0.239	TRUE
glycochenodeoxycholate	Hypertension	0.089	15	0.093	0.253	0.471	TRUE

Exposure	Outcome	R2	F	Heterogeneity	Pleiotropy	power	Direction
4-imidazoleacetate	Hypertension	0.134	16	2.43e-04	0.280	0.141	TRUE
1-methylimidazoleacetate	Hypertension	0.285	25	0.729	0.679	0.397	TRUE
N-acetylneuraminate	Hypertension	0.130	15	0.004	0.403	0.821	TRUE
dodecanedioate	Hypertension	0.288	18	0.066	0.185	0.211	TRUE
N-acetyltyrosine	Hypertension	1.7	112	3.46e-08	0.528	1.000	TRUE
1,3-dimethylurate	Hypertension	0.185	16	0.029	0.155	0.142	TRUE
trigonelline (N'-methylnicotinate)	Hypertension	0.104	14	3.2e-04	0.483	0.031	TRUE
indolepropionate	Hypertension	0.167	16	4.49e-05	0.067	0.339	TRUE
butyrylcarnitine	Hypertension	2.7	92	0.002	0.232	0.025	TRUE
myristoleate (14:1n5)	Hypertension	0.124	13	0.003	0.739	0.253	TRUE
dehydroisoandrosterone sulfate (DHEA-S)	Hypertension	0.217	20	0.173	0.814	0.590	TRUE
3-methylxanthine	Hypertension	0.107	16	0.331	0.436	0.036	TRUE
guanidinosuccinate	Hypertension	0.168	14	4.33e-04	0.378	0.037	TRUE
caproate (6:0)	Hypertension	0.204	16	0.096	0.338	0.041	TRUE
caprylate (8:0)	Hypertension	0.312	17	0.002	0.101	0.070	TRUE
10-undecenoate (11:1n1)	Hypertension	0.434	30	0.201	0.435	0.040	TRUE
docosapentaenoate (n3 DPA; 22:5n3)	Hypertension	0.288	14	0.110	0.970	0.044	TRUE

Exposure	Outcome	R2	F	Heterogeneity	Pleiotropy	power	Direction
phenol sulfate	Hypertension	0.138	17	0.155	0.737	0.073	TRUE
pregnen-diol disulfate	Hypertension	0.717	65	0.158	0.624	0.095	TRUE
X - 11261	Hypertension	0.263	17	0.465	0.867	0.199	TRUE
bilirubin (E,E)	Hypertension	0.370	38	0.989	0.824	0.037	TRUE
glycocholenate sulfate	Hypertension	0.614	28	8.27e-04	0.083	0.525	TRUE
pregn steroid monosulfate	Hypertension	0.301	21	0.124	0.955	0.036	TRUE
glycolithocholate sulfate	Hypertension	0.190	18	0.002	0.685	0.050	TRUE
X - 11381	Hypertension	0.261	16	0.006	0.548	0.044	TRUE
taurocholenate sulfate	Hypertension	0.367	21	3.05e-04	0.402	0.027	TRUE
2-arachidonoyl-GPE (20:4)	Hypertension	0.780	29	0.995	0.234	0.579	TRUE
andro steroid monosulfate (1)	Hypertension	0.241	19	0.033	0.388	0.124	TRUE
HWESASXX	Hypertension	0.141	15	0.358	0.057	0.152	TRUE
X - 11540	Hypertension	0.128	13	7.64e-06	0.440	0.026	TRUE
adrenate (22:4n6)	Hypertension	0.307	15	0.444	0.127	0.597	TRUE
X - 11787	Hypertension	0.776	53	1.6e-11	0.800	0.988	TRUE
X - 11795	Hypertension	0.143	13	4.21e-04	0.089	0.190	TRUE
1-arachidonoyl-GPC (20:4n6)	Hypertension	2.8	139	0.933	0.285	0.792	TRUE

Exposure	Outcome	R2	F	Heterogeneity	Pleiotropy	power	Direction
1-palmitoleoyl-GPC (16:1)	Hypertension	0.069	14	7.23e-05	0.323	0.032	TRUE
gamma-glutamylthreonine	Hypertension	0.140	15	0.004	0.593	0.064	TRUE
2-arachidonoyl-GPC (20:4)	Hypertension	2.2	93	0.760	0.162	0.690	TRUE
gamma-glutamylphenylalanine	Hypertension	0.073	18	0.187	0.801	0.048	TRUE
isobutyrylcarnitine	Hypertension	0.358	26	1.83e-05	0.672	0.154	TRUE
pseudouridine	Hypertension	0.182	15	0.057	0.399	0.364	TRUE
palmitoleate (16:1n7)	Hypertension	0.208	14	0.001	0.617	0.044	TRUE
1-dihomo-linolenoyl-GPC (20:3n3 or 6)	Hypertension	0.485	23	1.000	0.272	0.996	TRUE
1-docosahexaenoyl-GPC (22:6)	Hypertension	0.183	16	0.810	0.065	0.035	TRUE
1-dihomo-linoleoyl-GPC (20:2)	Hypertension	1.3	56	0.988	0.886	0.770	TRUE
gamma-glutamyl-epsilon-lysine	Hypertension	0.173	15	0.005	0.233	0.150	TRUE
octanoylcarnitine	Hypertension	0.482	37	0.008	0.064	0.064	TRUE
decanoylcarnitine	Hypertension	0.360	30	0.002	0.550	0.028	TRUE
N-acetylasparagine	Hypertension	0.293	25	1.9e-15	6.39e-04	0.184	TRUE
N-acetylglutamine	Hypertension	1.6	136	2.75e-08	0.985	1.000	TRUE
N-acetylhistidine	Hypertension	0.747	43	3.75e-12	0.011	0.970	TRUE
gamma-glutamylglycine	Hypertension	0.251	18	0.004	0.057	0.115	TRUE

Exposure	Outcome	R2	F	Heterogeneity	Pleiotropy	power	Direction
N-acetylphenylalanine	Hypertension	0.912	80	0.029	0.131	1.000	TRUE
myristoylcarnitine	Hypertension	0.275	18	4.94e-04	0.635	0.973	TRUE
glycylphenylalanine	Hypertension	0.362	23	0.241	0.002	0.077	TRUE
1-stearoyl-GPC (18:0)	Hypertension	0.154	15	0.705	0.986	0.112	TRUE
5-dodecenoate (12:1n7)	Hypertension	0.107	13	0.024	0.636	0.062	TRUE
10-nonadecenoate (19:1n9)	Hypertension	0.054	12	0.728	0.772	0.032	TRUE
epiandrosterone sulfate	Hypertension	0.252	20	3.67e-05	0.484	0.027	TRUE
1-arachidonoyl-GPI (20:4)	Hypertension	0.447	18	0.118	0.065	0.251	TRUE
2-docosahexaenoyl-GPE (22:6)	Hypertension	0.133	13	0.023	0.018	0.081	TRUE
stachydrine	Hypertension	0.131	13	0.166	0.965	0.077	TRUE
1-methylxanthine	Hypertension	0.240	24	0.123	0.182	0.097	TRUE
7-methylxanthine	Hypertension	0.092	15	0.015	0.631	0.303	TRUE
1-linolenoylglycerol (18:3)	Hypertension	0.145	18	0.010	0.286	0.626	TRUE
1-methylurate	Hypertension	0.240	22	0.573	0.958	0.093	TRUE
choline phosphate	Hypertension	0.160	16	2.84e-04	0.960	0.090	TRUE
1,7-dimethylurate	Hypertension	0.220	16	0.014	0.624	0.111	TRUE
5-acetylamino-6-formylamino-3-methyluracil	Hypertension	0.482	45	0.001	0.969	0.092	TRUE

Exposure	Outcome	R2	F	Heterogeneity	Pleiotropy	power	Direction
1,3,7-trimethylurate	Hypertension	0.156	15	1.35e-05	0.673	0.346	TRUE
isovalerylcarnitine	Hypertension	0.278	21	9.52e-06	0.102	0.900	TRUE
stearoylcarnitine	Hypertension	0.164	15	0.010	0.066	0.518	TRUE
1-linoleoyl-GPC (18:2)	Hypertension	0.488	26	0.956	0.019	0.638	TRUE
5-acetylamino-6-amino-3-methyluracil	Hypertension	0.212	19	0.014	0.667	0.166	TRUE
1-palmitoyl-GPA (16:0)	Hypertension	0.148	15	0.043	0.179	0.477	TRUE
gamma-glutamylisoleucine	Hypertension	0.226	17	0.667	0.983	0.308	TRUE
laurylcarnitine	Hypertension	0.192	19	0.017	0.596	0.619	TRUE
7-methylguanine	Hypertension	0.180	15	0.009	0.145	0.608	TRUE
phenylacetylglutamine	Hypertension	0.092	16	5.63e-07	0.349	0.034	TRUE
pro-hydroxy-pro	Hypertension	0.175	16	1.38e-04	0.333	0.336	TRUE
5-methyluridine (ribothymidine)	Hypertension	0.350	30	0.531	0.378	0.176	TRUE
N2,N2-dimethylguanosine	Hypertension	0.085	15	0.145	0.799	0.264	TRUE
1-docosahexaenoylglycerol (22:6)	Hypertension	0.162	20	1.7e-04	0.942	0.118	TRUE
N6-carbamoylthreonyladenosine	Hypertension	0.112	15	4.54e-08	0.233	0.300	TRUE
oleoylcarnitine	Hypertension	0.179	14	1.52e-07	0.494	0.071	TRUE
orotidine	Hypertension	0.344	26	0.730	0.942	0.232	TRUE

Exposure	Outcome	R2	F	Heterogeneity	Pleiotropy	power	Direction
1-arachidonoyl-GPE (20:4n6)	Hypertension	0.739	20	0.036	0.614	0.080	TRUE
2-palmitoyl-GPC (16:0)	Hypertension	0.169	14	0.181	0.944	0.034	TRUE
2-linoleoyl-GPC (18:2)	Hypertension	0.099	13	0.391	0.186	0.214	TRUE
tiglylcarnitine	Hypertension	0.191	15	0.145	0.187	0.038	TRUE
hexanoylglycine	Hypertension	0.277	22	1.22e-05	0.896	0.157	TRUE
2-methylmalonyl carnitine	Hypertension	0.123	15	5.29e-04	0.572	0.437	TRUE
1-myristoylglycerol (14:0)	Hypertension	0.207	16	0.874	0.793	0.030	TRUE
1-oleoyl-GPE (18:1)	Hypertension	0.223	11	0.930	0.039	0.400	TRUE
1-palmitoyl-GPE (16:0)	Hypertension	0.512	25	0.611	0.079	0.049	TRUE
cysteinylglycine	Hypertension	0.164	16	3.39e-08	0.142	0.785	TRUE
tetradecanedioate	Hypertension	0.870	31	1.93e-05	0.003	0.164	TRUE
2-hydroxypalmitate	Hypertension	0.149	14	0.259	0.106	0.068	TRUE
hexadecanedioate	Hypertension	0.495	27	0.002	0.237	0.038	TRUE
dihomo-linolenate (20:3n3 or n6)	Hypertension	0.434	17	0.943	0.773	0.955	TRUE
4-vinylphenol sulfate	Hypertension	0.156	13	0.026	0.061	0.104	TRUE
p-cresol sulfate	Hypertension	0.084	12	0.342	0.719	0.422	TRUE
2-linoleoyl-GPE (18:2)	Hypertension	0.654	26	0.402	0.279	0.507	TRUE

Exposure	Outcome	R2	F	Heterogeneity	Pleiotropy	power	Direction
1-linoleoyl-GPI (18:2)	Hypertension	0.538	23	0.605	0.799	0.253	TRUE
1-linoleoyl-GPE (18:2)	Hypertension	1.2	46	0.374	0.053	0.468	TRUE
1-oleoyl-GPI (18:1)	Hypertension	0.177	15	0.002	0.817	0.097	TRUE
gamma-glutamylglutamate	Hypertension	0.244	14	0.297	0.632	0.073	TRUE
2-hydroxy-3-methylvalerate	Hypertension	0.169	12	0.017	0.655	0.038	TRUE
deoxycarnitine	Hypertension	0.287	21	0.019	0.016	0.338	TRUE
3-methyladipate	Hypertension	0.124	15	0.054	0.158	0.643	TRUE
N6-acetyllysine	Hypertension	0.141	17	0.265	0.853	0.111	TRUE
octadecanedioate	Hypertension	0.138	14	3.86e-06	0.160	0.153	TRUE
7-alpha-hydroxy-3-oxo-4-cholestenoate (7-Hoca)	Hypertension	0.099	13	0.011	0.304	0.111	TRUE
dimethylarginine (SDMA + ADMA)	Hypertension	0.149	18	3.17e-07	0.835	0.277	TRUE
o-cresol sulfate	Hypertension	0.093	14	0.212	0.326	0.037	TRUE
taurolithocholate 3-sulfate	Hypertension	0.118	15	0.032	0.460	0.297	TRUE
succinylcarnitine	Hypertension	0.258	23	1.4e-04	0.042	0.968	TRUE
gamma-glutamylalanine	Hypertension	0.148	15	7.44e-10	0.233	0.457	TRUE
N-acetylserine	Hypertension	0.155	15	0.234	0.762	0.153	TRUE
gamma-glutamyl-2-aminobutyrate	Hypertension	0.109	15	0.083	0.288	0.074	TRUE

Exposure	Outcome	R2	F	Heterogeneity	Pleiotropy	power	Direction
tryptophan betaine	Hypertension	0.088	14	0.216	0.791	0.029	TRUE
cyclo(leu-pro)	Hypertension	0.157	15	1.84e-04	0.295	0.377	TRUE
5alpha-androstan-3alpha,17beta-diol monosulfate (1)	Hypertension	0.211	18	0.033	0.016	0.033	TRUE
5alpha-androstan-3beta,17beta-diol disulfate	Hypertension	0.542	38	4.75e-05	0.392	0.140	TRUE
5alpha-pregnan-3beta,20alpha-diol disulfate	Hypertension	0.182	17	0.159	0.518	0.428	TRUE
5alpha-pregnan-3beta,20alpha-diol monosulfate (2)	Hypertension	0.084	15	0.199	0.105	0.041	TRUE
4-androsten-3beta,17beta-diol disulfate (1)	Hypertension	0.486	38	4.26e-04	0.079	0.123	TRUE
4-androsten-3beta,17beta-diol disulfate (2)	Hypertension	0.141	13	0.012	0.110	0.105	TRUE
4-androsten-3alpha,17alpha-diol monosulfate (2)	Hypertension	0.146	12	4.61e-04	0.993	0.236	TRUE
4-androsten-3alpha,17alpha-diol monosulfate (3)	Hypertension	0.546	27	0.049	0.787	0.510	TRUE
4-androsten-3beta,17beta-diol monosulfate (2)	Hypertension	0.260	19	0.009	0.833	0.939	TRUE
4-androsten-3beta,17beta-diol monosulfate (1)	Hypertension	0.165	16	0.323	0.548	0.263	TRUE
1-docosapentaenoyl-GPC (22:5n3)	Hypertension	0.436	19	0.987	0.652	0.956	TRUE
1-pentadecanoyl-GPC (15:0)	Hypertension	0.101	14	0.704	0.033	0.118	TRUE
N-methylproline	Hypertension	0.117	14	0.002	0.005	0.039	TRUE
N-acetyl-beta-alanine	Hypertension	0.175	17	0.004	0.955	0.218	TRUE
cysteine sulfinic acid	Hypertension	0.099	13	0.007	0.489	0.104	TRUE

Exposure	Outcome	R2	F	Heterogeneity	Pleiotropy	power	Direction
ergothioneine	Hypertension	0.114	14	0.781	0.574	0.126	TRUE
N-acetylputrescine	Hypertension	0.374	38	0.206	0.349	0.034	TRUE
palmitoyl sphingomyelin (d18:1/16:0)	Hypertension	0.075	15	0.173	0.867	0.046	TRUE
sphingomyelin (d18:1/18:1, d18:2/18:0)	Hypertension	0.347	15	0.090	0.065	0.467	TRUE
13-HODE + 9-HODE	Hypertension	0.334	16	0.012	0.780	0.657	TRUE
oleoyl ethanolamide	Hypertension	0.163	16	0.002	0.222	0.652	TRUE
S-methylmethionine	Hypertension	0.085	9.3	9.98e-06	0.999	0.219	TRUE
phenylalanylphenylalanine	Hypertension	0.141	16	0.025	0.825	0.030	TRUE
16a-hydroxy DHEA 3-sulfate	Hypertension	0.412	29	0.002	0.044	0.081	TRUE
pregnenolone sulfate	Hypertension	0.213	15	0.002	0.506	0.045	TRUE
cis-4-decenoyl carnitine	Hypertension	0.375	23	0.001	0.312	0.095	TRUE
17-methylstearate	Hypertension	0.122	13	0.102	0.119	0.449	TRUE
cinnamoylglycine	Hypertension	0.282	17	0.007	0.914	0.043	TRUE
3-methylglutaconate	Hypertension	0.289	25	0.002	0.371	0.508	TRUE
15-methylpalmitate	Hypertension	0.070	14	1.68e-04	0.913	0.041	TRUE
2-stearoyl-GPI (18:0)	Hypertension	0.214	15	0.001	0.109	0.152	TRUE
glycoursodeoxycholate	Hypertension	0.068	15	0.451	0.146	0.861	TRUE

Exposure	Outcome	R2	F	Heterogeneity	Pleiotropy	power	Direction
S-methylcysteine	Hypertension	0.177	23	0.293	0.527	0.062	TRUE
leucylalanine	Hypertension	0.243	16	5.37e-27	9.56e-04	0.943	TRUE
phenylalanylserine	Hypertension	0.464	36	4.38e-11	0.358	0.204	TRUE
alpha-glutamyltyrosine	Hypertension	0.407	29	6.05e-49	0.366	1.000	TRUE
isoleucylvaline	Hypertension	0.161	19	0.067	0.946	0.324	TRUE
X - 17299	Hypertension	0.157	15	0.066	0.822	0.035	TRUE
N1-Methyl-2-pyridone-5-carboxamide	Hypertension	0.080	13	0.214	0.294	0.131	TRUE
hydantoin-5-propionic acid	Hypertension	0.159	16	0.102	0.497	0.059	TRUE
dihydroferulic acid	Hypertension	0.095	16	0.122	0.049	0.052	TRUE
pregnanediol-3-glucuronide	Hypertension	0.131	16	0.052	0.034	0.028	TRUE
imidazole propionate	Hypertension	0.145	18	0.067	0.554	0.059	TRUE
prolylproline	Hypertension	0.301	39	0.529	0.065	0.161	TRUE
phenylalanyltryptophan	Hypertension	0.173	17	0.236	0.904	0.796	TRUE
alpha-glutamylglycine	Hypertension	0.384	22	8.45e-06	0.959	0.178	TRUE
histidyltryptophan	Hypertension	0.123	14	0.172	0.297	0.039	TRUE
serylalanine	Hypertension	0.243	17	8.97e-14	0.133	0.156	TRUE
valylarginine	Hypertension	0.260	17	2.91e-40	0.710	1.000	TRUE

Exposure	Outcome	R2	F	Heterogeneity	Pleiotropy	power	Direction
indoleacetylglutamine	Hypertension	0.187	16	0.603	0.954	0.409	TRUE
N-palmitoylglycine	Hypertension	0.318	21	0.003	0.148	0.158	TRUE
2-aminobutyrate	Hypertension	0.118	16	0.132	0.603	0.374	TRUE
1-stearoyl-GPE (18:0)	Hypertension	0.495	30	0.799	0.949	0.071	TRUE
erythronate	Hypertension	0.136	16	0.511	0.487	0.213	TRUE
1-palmitoyl-2-linoleoyl-GPC (16:0/18:2)	Hypertension	0.855	34	0.274	0.818	0.555	TRUE
1-stearoyl-2-oleoyl-GPE (18:0/18:1)	Hypertension	0.373	21	0.071	0.069	0.156	TRUE
1-palmitoyl-2-linoleoyl-GPE (16:0/18:2)	Hypertension	1.6	55	0.972	0.441	0.854	TRUE
1-stearoyl-2-arachidonoyl-GPC (18:0/20:4)	Hypertension	3.6	163	0.368	0.227	0.889	TRUE
sphingomyelin (d18:2/16:0, d18:1/16:1)	Hypertension	0.071	12	0.314	0.094	0.052	TRUE
sphingomyelin (d18:1/14:0, d16:1/16:0)	Hypertension	0.310	27	0.006	0.486	0.837	TRUE
2-hydroxydecanoate	Hypertension	0.097	16	0.094	0.373	0.027	TRUE
dimethylmalonic acid	Hypertension	0.120	13	0.008	0.594	0.160	TRUE
N-delta-acetylornithine	Hypertension	3.6	295	6.34e-08	0.340	1.000	TRUE
N-acetyl-1-methylhistidine	Hypertension	1.1	99	8.86e-15	0.115	0.762	TRUE
N-acetyl-3-methylhistidine	Hypertension	0.642	50	5.63e-12	0.091	0.998	TRUE
2-aminooctanoate	Hypertension	1.2	86	4.31e-11	0.973	1.000	TRUE

Exposure	Outcome	R2	F	Heterogeneity	Pleiotropy	power	Direction
N-acetylcarnosine	Hypertension	0.262	22	0.004	0.145	0.058	TRUE
5-hydroxyindoleacetate	Hypertension	0.152	18	0.107	0.707	0.098	TRUE
2-aminoheptanoate	Hypertension	0.115	16	0.696	0.921	0.098	TRUE
guanidinoacetate	Hypertension	0.253	17	1.38e-04	0.016	0.082	TRUE
bilirubin (Z,Z)	Hypertension	0.502	50	0.026	0.214	0.185	TRUE
gamma-glutamylvaline	Hypertension	0.186	16	0.173	0.817	0.069	TRUE
glycerol 3-phosphate	Hypertension	0.131	16	2.63e-04	0.256	0.073	TRUE
aspartate	Hypertension	0.149	16	9.24e-07	0.389	0.398	TRUE
1-eicosenoyl-GPC (20:1)	Hypertension	0.107	15	5.55e-04	0.959	0.049	TRUE
1-eicosapentaenoyl-GPC (20:5)	Hypertension	0.656	19	0.299	0.867	0.584	TRUE
4-acetylphenol sulfate	Hypertension	0.122	16	0.230	0.037	0.033	TRUE
1-dihomo-linolenoyl-GPE (20:3n3 or 6)	Hypertension	0.519	18	0.881	0.066	0.029	TRUE
1-docosahexaenoyl-GPE (22:6)	Hypertension	0.251	16	0.013	0.985	0.224	TRUE
isovalerate	Hypertension	0.404	16	1.82e-07	0.028	0.048	TRUE
glutarylcarnitine (C5)	Hypertension	0.284	18	8.47e-06	0.372	0.577	TRUE
docosahexaenoate (DHA; 22:6n3)	Hypertension	0.112	17	0.372	0.354	0.034	TRUE
palmitoylcarnitine	Hypertension	0.192	13	6.91e-06	0.019	0.184	TRUE

Exposure	Outcome	R2	F	Heterogeneity	Pleiotropy	power	Direction
1-margaroyl-GPC (17:0)	Hypertension	0.137	18	0.318	0.531	0.091	TRUE
gamma-glutamylmethionine	Hypertension	0.160	16	0.042	0.062	0.117	TRUE
gamma-CEHC	Hypertension	0.108	18	0.479	0.070	0.677	TRUE
N-palmitoyl-sphingosine (d18:1/16:0)	Hypertension	0.119	16	0.034	0.891	0.156	TRUE
methionine sulfone	Hypertension	0.982	63	3.4e-21	0.375	0.782	TRUE
2-methylbutyrylcarnitine (C5)	Hypertension	0.146	17	0.021	0.436	0.047	TRUE
O-sulfo-L-tyrosine	Hypertension	0.157	15	0.492	0.275	0.069	TRUE
3-(3-hydroxyphenyl)propionate sulfate	Hypertension	0.080	16	0.224	0.998	0.066	TRUE
2-palmitoyl-GPE (16:0)	Hypertension	0.452	27	0.046	0.350	0.201	TRUE
1-myristoyl-GPC (14:0)	Hypertension	0.101	13	0.477	0.197	0.722	TRUE
1-arachidoyl-GPC (20:0)	Hypertension	0.081	13	1.96e-05	0.109	0.089	TRUE
1-docosapentaenoyl-GPC (22:5n6)	Hypertension	0.332	14	0.140	0.586	0.821	TRUE
21-hydroxypregnenolone disulfate	Hypertension	0.269	20	0.001	0.143	0.251	TRUE
mannitol/sorbitol	Hypertension	0.120	17	0.110	0.698	0.055	TRUE
methyl glucopyranoside (alpha + beta)	Hypertension	0.459	33	0.007	0.195	0.033	TRUE
4-methylcatechol sulfate	Hypertension	0.169	13	0.012	0.833	0.107	TRUE
3-methyl catechol sulfate (1)	Hypertension	0.100	18	0.021	0.157	0.223	TRUE

Exposure	Outcome	R2	F	Heterogeneity	Pleiotropy	power	Direction
2-docosahexaenoyl-GPC (22:6)	Hypertension	0.098	16	0.242	0.107	0.089	TRUE
linoleoylcarnitine	Hypertension	0.232	21	0.003	0.204	0.403	TRUE
pyroglutamine	Hypertension	0.230	16	0.001	0.271	0.069	TRUE
X - 11564	Hypertension	0.158	14	7.38e-05	0.792	0.039	TRUE
X - 15486	Hypertension	0.227	17	1.34e-15	0.943	0.193	TRUE
X - 21286	Hypertension	0.136	13	0.158	0.856	0.127	TRUE
1-arachidonoyl-GPA (20:4)	Hypertension	0.117	14	0.185	0.293	0.650	TRUE
X - 11441	Hypertension	0.360	29	0.041	0.343	0.096	TRUE
X - 21319	Hypertension	0.168	15	0.053	0.397	0.112	TRUE
X - 18249	Hypertension	0.170	19	0.068	0.768	0.035	TRUE
X - 11905	Hypertension	0.639	30	4.25e-06	0.411	0.057	TRUE
X - 18914	Hypertension	0.192	19	0.015	0.073	0.101	TRUE
X - 21339	Hypertension	0.234	15	0.006	0.953	0.163	TRUE
X - 21341	Hypertension	0.084	14	0.508	0.577	0.033	TRUE
X - 21343	Hypertension	0.333	17	0.082	0.432	0.088	TRUE
X - 11308	Hypertension	0.165	16	1.33e-04	0.780	0.056	TRUE
X - 21353	Hypertension	0.443	28	0.002	0.580	0.084	TRUE

Exposure	Outcome	R2	F	Heterogeneity	Pleiotropy	power	Direction
X - 17178	Hypertension	0.538	25	4.57e-11	0.786	0.197	TRUE
X - 21365	Hypertension	0.189	19	8.79e-04	0.798	0.106	TRUE
X - 17189	Hypertension	0.181	13	4.55e-09	0.782	0.041	TRUE
X - 11442	Hypertension	0.449	30	6.96e-07	0.425	0.071	TRUE
X - 21411	Hypertension	0.280	18	1.55e-11	0.045	0.999	TRUE
X - 12511	Hypertension	0.992	90	1.28e-07	0.446	1.000	TRUE
X - 01911	Hypertension	0.097	14	0.003	0.637	0.146	TRUE
X - 11444	Hypertension	0.193	24	0.359	0.249	0.132	TRUE
X - 12824	Hypertension	0.445	30	1.55e-15	0.204	0.925	TRUE
X - 15497	Hypertension	0.105	14	0.115	0.799	0.051	TRUE
X - 21441	Hypertension	0.162	15	0.075	0.380	0.100	TRUE
X - 17185	Hypertension	0.128	14	0.027	0.122	0.074	TRUE
X - 21448	Hypertension	0.603	70	0.011	0.582	0.055	TRUE
X - 02269	Hypertension	0.288	21	0.117	0.073	0.162	TRUE
X - 02249	Hypertension	0.199	17	0.020	0.550	0.161	TRUE
X - 11850	Hypertension	0.064	13	0.961	0.690	0.239	TRUE
X - 16946	Hypertension	0.572	47	0.633	0.340	0.042	TRUE

Exposure	Outcome	R2	F	Heterogeneity	Pleiotropy	power	Direction
alpha-hydroxyisovalerate	Hypertension	0.254	13	0.038	0.009	0.087	TRUE
3-methylglutarylcarnitine (1)	Hypertension	0.140	20	0.634	0.393	0.086	TRUE
3-methylglutarylcarnitine (2)	Hypertension	0.213	22	0.008	0.246	0.265	TRUE
X - 12063	Hypertension	0.576	31	0.009	0.024	0.913	TRUE
X - 07765	Hypertension	0.066	14	0.669	0.438	0.126	TRUE
X - 11299	Hypertension	0.079	17	0.019	0.012	0.123	TRUE
X - 11305	Hypertension	0.090	14	0.120	0.697	0.051	TRUE
X - 11372	Hypertension	0.146	15	0.340	0.154	0.050	TRUE
X - 11429	Hypertension	0.136	15	0.360	0.003	0.074	TRUE
X - 11438	Hypertension	0.081	12	0.018	0.230	0.026	TRUE
X - 11440	Hypertension	0.626	46	0.026	0.064	0.035	TRUE
X - 11452	Hypertension	0.065	16	0.251	0.505	0.026	TRUE
X - 11470	Hypertension	0.278	17	0.536	0.628	0.045	TRUE
X - 11478	Hypertension	0.183	19	0.028	0.305	0.216	TRUE
X - 11530	Hypertension	0.420	45	0.122	0.728	0.493	TRUE
X - 11538	Hypertension	0.748	36	0.777	0.068	0.998	TRUE
X - 11880	Hypertension	0.150	13	0.001	0.372	0.185	TRUE

Exposure	Outcome	R2	F	Heterogeneity	Pleiotropy	power	Direction
X - 12007	Hypertension	0.119	16	0.395	0.778	0.120	TRUE
X - 12206	Hypertension	0.242	18	0.003	0.405	0.126	TRUE
X - 12216	Hypertension	0.084	14	0.146	0.570	0.194	TRUE
X - 12230	Hypertension	0.143	15	0.478	0.494	0.049	TRUE
X - 12411	Hypertension	0.190	16	0.359	0.360	0.089	TRUE
5,8-tetradecadienoate	Hypertension	0.258	16	0.190	0.184	0.048	TRUE
X - 12450	Hypertension	0.146	16	0.056	0.641	0.043	TRUE
X - 12729	Hypertension	0.136	13	0.002	0.026	0.035	TRUE
X - 12798	Hypertension	0.761	41	0.038	0.205	0.076	TRUE
X - 12472	Hypertension	0.234	17	0.220	0.826	0.300	TRUE
X - 12816	Hypertension	0.092	13	0.194	0.812	0.168	TRUE
X - 14056	Hypertension	0.146	15	0.019	0.774	0.175	TRUE
X - 12844	Hypertension	0.168	17	1.55e-05	0.508	0.357	TRUE
X - 12846	Hypertension	0.191	20	0.818	0.280	0.033	TRUE
X - 15469	Hypertension	0.113	17	3.66e-04	0.275	0.044	TRUE
X - 13529	Hypertension	0.153	15	0.133	0.146	0.086	TRUE
X - 16132	Hypertension	0.115	14	0.229	0.319	0.335	TRUE

Exposure	Outcome	R2	F	Heterogeneity	Pleiotropy	power	Direction
X - 14568	Hypertension	0.194	14	0.291	0.782	0.204	TRUE
X - 14939	Hypertension	0.266	20	0.047	0.715	0.758	TRUE
X - 15245	Hypertension	0.101	17	0.566	0.494	0.155	TRUE
X - 15492	Hypertension	0.225	17	0.142	0.775	0.252	TRUE
X - 15728	Hypertension	0.046	10	0.006	0.535	0.229	TRUE
X - 16123	Hypertension	0.180	14	0.110	0.387	0.222	TRUE
X - 16935	Hypertension	0.412	25	4.83e-04	0.437	0.045	TRUE
X - 16944	Hypertension	0.160	16	0.262	0.368	0.111	TRUE
X - 17145	Hypertension	0.094	12	0.017	0.403	0.146	TRUE
X - 18901	Hypertension	0.094	19	0.398	0.107	0.252	TRUE
X - 17166	Hypertension	0.284	21	3.15e-26	0.782	0.049	TRUE
X - 17167	Hypertension	0.169	18	0.008	0.505	0.425	TRUE
X - 18913	Hypertension	0.073	12	0.986	0.247	0.224	TRUE
X - 21628	Hypertension	0.237	16	0.285	0.887	0.426	TRUE
X - 16134	Hypertension	0.235	17	6.43e-04	0.342	0.089	TRUE
X - 17179	Hypertension	0.449	38	1.6e-04	0.882	0.480	TRUE
X - 19141	Hypertension	1.1	81	4.86e-12	0.199	0.120	TRUE

Exposure	Outcome	R2	F	Heterogeneity	Pleiotropy	power	Direction
X - 17438	Hypertension	0.082	16	0.019	0.619	0.032	TRUE
X - 21666	Hypertension	0.117	14	0.094	0.273	0.034	TRUE
X - 12855	Hypertension	0.123	14	0.059	0.792	0.153	TRUE
oleoyl-linoleoyl-glycerol (18:1/18:2) [1]	Hypertension	0.349	17	1.32e-07	0.997	0.324	TRUE
oleoyl-linoleoyl-glycerol (18:1/18:2) [2]	Hypertension	0.182	21	6.42e-10	0.552	0.330	TRUE
X - 12748	Hypertension	0.165	19	0.002	0.547	0.042	TRUE
X - 21735	Hypertension	0.338	21	0.003	0.002	0.716	TRUE
X - 21736	Hypertension	0.161	15	0.011	0.224	0.190	TRUE
X - 21737	Hypertension	0.087	17	0.234	0.372	0.035	TRUE
X - 11522	Hypertension	0.627	64	0.255	0.566	0.047	TRUE
gulonic acid	Hypertension	0.075	13	0.104	0.741	0.061	TRUE
sulfate	Hypertension	0.135	17	2.57e-04	0.122	0.045	TRUE
X - 15503	Hypertension	0.116	14	0.607	0.927	0.177	TRUE
X - 12822	Hypertension	0.272	24	2.74e-16	0.663	0.322	TRUE
X - 21821	Hypertension	0.124	16	0.466	0.720	0.053	TRUE
X - 17327	Hypertension	0.199	16	0.120	0.726	0.350	TRUE
X - 16570	Hypertension	0.260	29	0.053	0.904	0.789	TRUE

Exposure	Outcome	R2	F	Heterogeneity	Pleiotropy	power	Direction
X - 21849	Hypertension	0.218	21	0.070	0.041	0.125	TRUE
X - 21892	Hypertension	0.208	15	0.019	0.028	0.238	TRUE
1-nonadecanoyl-GPC (19:0)	Hypertension	0.123	13	0.045	0.694	0.451	TRUE
etiocholanolone glucuronide	Hypertension	0.322	27	0.043	0.252	0.171	TRUE
2-palmitoleoyl-GPC (16:1)	Hypertension	0.080	9.9	0.011	0.660	0.335	TRUE
sphingomyelin (d18:1/24:1, d18:2/24:0)	Hypertension	0.271	13	2.36e-10	0.010	0.050	TRUE
sphingomyelin (d18:2/14:0, d18:1/14:1)	Hypertension	0.135	18	0.147	0.500	0.032	TRUE
X - 12680	Hypertension	0.129	16	0.008	0.088	0.283	TRUE
X - 22147	Hypertension	0.106	15	0.440	0.661	0.050	TRUE
X - 22162	Hypertension	0.418	47	0.166	0.866	0.043	TRUE
X - 10458	Hypertension	0.037	12	0.562	0.810	0.080	TRUE
X - 11537	Hypertension	0.130	12	0.419	0.610	0.050	TRUE
X - 22379	Hypertension	0.521	36	0.187	0.401	0.177	TRUE
X - 11871	Hypertension	0.080	16	0.166	0.853	0.892	TRUE
X - 12101	Hypertension	0.269	20	0.003	0.406	0.088	TRUE
X - 11483	Hypertension	0.087	17	0.025	0.800	0.031	TRUE
X - 11491	Hypertension	1	51	2.07e-04	0.008	0.031	TRUE

Exposure	Outcome	R2	F	Heterogeneity	Pleiotropy	power	Direction
X - 12231	Hypertension	0.129	14	0.192	0.481	0.440	TRUE
X - 12830	Hypertension	0.094	15	0.071	0.299	0.360	TRUE
X - 18899	Hypertension	0.124	14	0.054	0.029	0.060	TRUE
5-hydroxytryptophan	Hypertension	0.180	14	1.81e-04	0.871	0.092	TRUE
X - 12127	Hypertension	0.142	13	0.250	0.676	0.541	TRUE
X - 12339	Hypertension	0.093	12	5.15e-09	0.209	0.410	TRUE
X - 12681	Hypertension	0.211	14	7.59e-05	0.891	0.766	TRUE
X - 12686	Hypertension	0.207	16	0.029	0.688	0.084	TRUE
X - 12688	Hypertension	0.218	20	0.033	0.499	0.070	TRUE
X - 14838	Hypertension	0.181	14	2.04e-04	0.118	0.883	TRUE
nonanoylcarnitine	Hypertension	0.367	37	0.071	0.202	0.054	TRUE
X - 15666	Hypertension	0.125	15	0.019	0.824	0.032	TRUE
X - 16071	Hypertension	0.173	29	0.011	0.859	0.226	TRUE
X - 16580	Hypertension	0.105	15	0.279	0.835	0.778	TRUE
X - 17337	Hypertension	0.273	19	0.035	0.597	0.184	TRUE
1-eicosapentaenoyl-GPE (20:5)	Hypertension	0.192	16	0.261	0.188	0.074	TRUE
X - 12026	Hypertension	0.132	15	0.122	0.341	0.104	TRUE

Exposure	Outcome	R2	F	Heterogeneity	Pleiotropy	power	Direction
X - 12543	Hypertension	0.089	11	0.141	0.426	0.103	TRUE
X - 17010	Hypertension	0.328	15	0.290	0.747	0.148	TRUE
X - 17351	Hypertension	0.099	15	0.088	0.603	0.942	TRUE
X - 22764	Hypertension	0.213	19	0.051	0.969	0.165	TRUE
glucose	Hypertension	0.169	13	0.047	0.342	0.039	TRUE
mannose	Hypertension	0.182	19	0.003	0.861	0.950	TRUE
X - 10358	Hypertension	0.099	14	0.439	0.077	0.165	TRUE
X - 22816	Hypertension	0.169	17	0.878	0.715	0.098	TRUE
X - 22822	Hypertension	0.102	14	0.001	0.942	0.073	TRUE
myristoleoylcarnitine	Hypertension	0.116	12	0.236	0.358	0.030	TRUE
N-acetyltaurine	Hypertension	0.148	15	0.313	0.491	0.104	TRUE
X - 12695	Hypertension	0.186	15	2.53e-05	0.325	0.031	TRUE
arabonate/xylonate	Hypertension	0.119	15	1.9e-05	0.489	0.107	TRUE
2-oleoyl-GPC (18:1)	Hypertension	0.137	14	0.110	0.564	0.128	TRUE
X - 24435	Hypertension	0.326	28	0.003	0.812	0.093	TRUE
dopamine sulfate (2)	Hypertension	0.287	17	0.025	0.248	0.117	TRUE
tyramine O-sulfate	Hypertension	0.127	16	0.034	0.635	0.128	TRUE

Exposure	Outcome	R2	F	Heterogeneity	Pleiotropy	power	Direction
methyl-4-hydroxybenzoate sulfate	Hypertension	0.050	16	0.110	0.220	0.041	TRUE
N-acetylcitrulline	Hypertension	3.3	301	7.28e-10	0.973	1.000	TRUE
4-hydroxychlorothalonil	Hypertension	0.176	17	0.051	0.369	0.034	TRUE
3-hydroxypyridine sulfate	Hypertension	0.122	12	1.47e-07	0.884	0.118	TRUE
sphingomyelin (d18:1/20:0, d16:1/22:0)	Hypertension	0.191	19	6.21e-05	0.042	0.039	TRUE
sphingomyelin (d18:1/20:1, d18:2/20:0)	Hypertension	0.199	16	0.067	0.162	0.105	TRUE
behenoyl sphingomyelin (d18:1/22:0)	Hypertension	0.137	15	4.87e-05	0.922	0.234	TRUE
X - 23026	Hypertension	0.175	16	0.016	0.608	0.665	TRUE
2-acetamidophenol sulfate	Hypertension	0.063	12	0.413	0.470	0.669	TRUE
1,2,3-benzenetriol sulfate (2)	Hypertension	0.085	19	0.043	0.337	0.208	TRUE
C-glycosyltryptophan	Hypertension	0.158	16	0.002	0.091	0.030	TRUE
p-cresol-glucuronide	Hypertension	0.077	13	0.002	0.360	0.078	TRUE
arabitol/xylitol	Hypertension	0.184	15	0.301	0.542	0.406	TRUE
X - 23293	Hypertension	0.847	49	0.370	0.476	0.033	TRUE
X - 23593	Hypertension	0.140	15	0.288	0.854	0.493	TRUE
X - 23639	Hypertension	0.118	14	0.369	0.106	0.176	TRUE
X - 23644	Hypertension	0.174	15	1.56e-04	0.948	0.030	TRUE

Exposure	Outcome	R2	F	Heterogeneity	Pleiotropy	power	Direction
X - 23705	Hypertension	1.3	56	1.000	0.200	0.763	TRUE
X - 11315	Hypertension	0.208	16	0.091	0.822	0.541	TRUE
X - 23765	Hypertension	0.146	15	0.108	0.791	0.126	TRUE
X - 23782	Hypertension	0.103	16	0.125	0.492	0.165	TRUE
X - 23788	Hypertension	0.105	14	0.472	0.839	0.104	TRUE
anthranilate	Hypertension	0.137	15	0.670	0.521	0.060	TRUE
X - 23314	Hypertension	0.288	22	0.019	0.107	0.032	TRUE
dimethylglycine	Hypertension	0.247	19	0.036	0.520	0.053	TRUE
creatinine	Hypertension	0.172	15	0.085	0.656	0.032	TRUE
X - 23997	Hypertension	0.129	13	0.068	0.607	0.075	TRUE
X - 24021	Hypertension	0.107	13	0.459	0.504	0.158	TRUE
X - 24061	Hypertension	0.181	14	0.701	0.720	0.439	TRUE
X - 24065	Hypertension	0.096	14	0.675	0.609	0.284	TRUE
X - 24071	Hypertension	0.201	17	0.007	0.782	0.043	TRUE
X - 24097	Hypertension	0.186	14	0.003	0.705	0.274	TRUE
X - 24125	Hypertension	0.208	17	1.53e-10	0.376	0.447	TRUE
X - 24240	Hypertension	0.237	15	0.002	0.744	0.025	TRUE

Exposure	Outcome	R2	F	Heterogeneity	Pleiotropy	power	Direction
X - 24241	Hypertension	0.343	26	0.246	0.373	0.043	TRUE
2-hydroxybutyrate/2-hydroxyisobutyrate	Hypertension	0.088	12	0.004	0.168	0.046	TRUE
2-methylcitrate/homocitrate	Hypertension	0.098	14	0.215	0.724	0.041	TRUE
X - 24242	Hypertension	0.101	12	0.004	0.878	0.057	TRUE
oleate/vaccenate (18:1)	Hypertension	0.208	13	0.084	0.294	0.038	TRUE
2-hydroxyglutarate	Hypertension	0.133	15	0.032	0.599	0.204	TRUE
X - 24293	Hypertension	0.075	11	0.676	0.243	0.104	TRUE
cis-aconitate	Hypertension	0.096	14	0.084	0.806	0.036	TRUE
X - 24278	Hypertension	0.639	25	9.49e-05	0.519	0.045	TRUE
leucylphenylalanine/isoleucylphenylalanine	Hypertension	0.338	41	0.058	0.807	0.379	TRUE
1-palmitoleoylglycerol (16:1)	Hypertension	0.124	15	0.548	0.549	0.029	TRUE
sphingomyelin (d18:1/15:0, d16:1/17:0)	Hypertension	0.197	19	0.091	0.952	0.450	TRUE
palmitoyl dihydrosphingomyelin (d18:0/16:0)	Hypertension	0.109	13	7.41e-04	0.620	0.129	TRUE
sphingomyelin (d18:2/23:0, d18:1/23:1, d17:1/24:1)	Hypertension	0.158	14	0.045	0.925	0.037	TRUE
sphingomyelin (d18:2/24:1, d18:1/24:2)	Hypertension	0.177	14	0.693	0.714	0.250	TRUE
1-stearoyl-2-oleoyl-GPC (18:0/18:1)	Hypertension	0.135	15	0.135	0.266	0.091	TRUE
1-stearoyl-2-linoleoyl-GPE (18:0/18:2)	Hypertension	1.4	43	0.733	0.258	0.859	TRUE

Exposure	Outcome	R2	F	Heterogeneity	Pleiotropy	power	Direction
1-stearoyl-2-arachidonoyl-GPE (18:0/20:4)	Hypertension	0.534	40	0.052	0.160	0.067	TRUE
1-stearoyl-2-arachidonoyl-GPI (18:0/20:4)	Hypertension	0.917	36	3.94e-05	0.825	0.263	TRUE
1-palmitoyl-2-linoleoyl-GPI (16:0/18:2)	Hypertension	0.638	24	0.419	0.481	0.554	TRUE
1-stearoyl-2-linoleoyl-GPC (18:0/18:2)	Hypertension	0.432	17	0.213	0.145	0.258	TRUE
1-oleoyl-2-linoleoyl-GPC (18:1/18:2)	Hypertension	0.534	24	0.883	0.888	0.881	TRUE
1-palmitoyl-2-dihomo-linolenoyl-GPC (16:0/20:3n3 or 6)	Hypertension	0.327	16	0.236	0.830	0.107	TRUE
1-palmitoyl-2-oleoyl-GPC (16:0/18:1)	Hypertension	0.117	13	0.292	0.731	0.301	TRUE
1-palmitoyl-2-arachidonoyl-GPC (16:0/20:4)	Hypertension	2.9	129	0.870	0.446	0.795	TRUE
1-palmitoyl-2-eicosapentaenoyl-GPC (16:0/20:5)	Hypertension	0.503	20	0.904	0.661	0.711	TRUE
1-palmitoyl-2-arachidonoyl-GPE (16:0/20:4)	Hypertension	0.340	27	0.135	0.542	0.135	TRUE
1-palmitoyl-2-docosahexaenoyl-GPE (16:0/22:6)	Hypertension	0.773	36	0.033	0.020	0.074	TRUE
1-stearoyl-2-docosahexaenoyl-GPE (18:0/22:6)	Hypertension	0.511	28	0.021	0.020	0.119	TRUE
1-palmitoyl-2-arachidonoyl-GPI (16:0/20:4)	Hypertension	0.320	17	0.287	0.439	0.169	TRUE
1-stearoyl-2-linoleoyl-GPI (18:0/18:2)	Hypertension	0.873	29	1.99e-04	0.546	0.109	TRUE
1-palmitoyl-2-palmitoleoyl-GPC (16:0/16:1)	Hypertension	0.130	13	0.233	0.406	0.046	TRUE
gamma-tocopherol/beta-tocopherol	Hypertension	0.182	16	0.384	0.016	0.036	TRUE
1-(1-enyl-stearoyl)-2-arachidonoyl-GPE (P-18:0/20:4)	Hypertension	0.712	33	0.995	0.123	0.741	TRUE

Exposure	Outcome	R2	F	Heterogeneity	Pleiotropy	power	Direction
1-(1-enyl-stearoyl)-2-docosahexaenoyl-GPE (P-18:0/22:6)	Hypertension	0.230	17	0.256	0.536	0.183	TRUE
1-(1-enyl-palmitoyl)-2-oleoyl-GPE (P-16:0/18:1)	Hypertension	0.183	13	0.007	0.934	0.572	TRUE
1-(1-enyl-palmitoyl)-2-oleoyl-GPC (P-16:0/18:1)	Hypertension	0.194	14	0.015	0.600	0.145	TRUE
sphingomyelin (d18:1/21:0, d17:1/22:0, d16:1/23:0)	Hypertension	0.335	23	0.349	0.186	0.539	TRUE
1-pentadecanoyl-2-linoleoyl-GPC (15:0/18:2)	Hypertension	0.413	17	0.274	0.229	0.827	TRUE
1-pentadecanoyl-2-oleoyl-GPC (15:0/18:1)	Hypertension	0.094	17	0.380	0.760	0.095	TRUE
1-margaroyl-2-oleoyl-GPC (17:0/18:1)	Hypertension	0.135	16	0.726	0.320	0.085	TRUE
1-margaroyl-2-linoleoyl-GPC (17:0/18:2)	Hypertension	0.226	15	0.198	0.220	0.467	TRUE
X - 24309	Hypertension	0.574	33	0.584	0.176	0.033	TRUE
2'-deoxyuridine	Hypertension	0.244	20	0.121	0.302	0.067	TRUE
1,2-dilinoleoyl-GPC (18:2/18:2)	Hypertension	0.726	29	0.533	0.778	0.709	TRUE
1-palmitoyl-2-docosahexaenoyl-GPC (16:0/22:6)	Hypertension	0.182	15	0.381	0.185	0.196	TRUE
1-stearoyl-2-docosahexaenoyl-GPC (18:0/22:6)	Hypertension	0.168	14	0.813	0.028	0.208	TRUE
1-(1-enyl-stearoyl)-2-oleoyl-GPC (P-18:0/18:1)	Hypertension	0.139	13	2.09e-05	0.242	0.049	TRUE
1-(1-enyl-stearoyl)-2-arachidonoyl-GPC (P-18:0/20:4)	Hypertension	1.6	68	1.000	0.116	0.797	TRUE
1-(1-enyl-stearoyl)-2-oleoyl-GPE (P-18:0/18:1)	Hypertension	0.098	11	0.138	0.279	0.042	TRUE
sphingomyelin (d18:1/17:0, d17:1/18:0, d19:1/16:0)	Hypertension	0.116	14	0.076	0.299	0.128	TRUE

Exposure	Outcome	R2	F	Heterogeneity	Pleiotropy	power	Direction
1-palmitoyl-2-stearoyl-GPC (16:0/18:0)	Hypertension	0.272	16	0.549	0.844	0.042	TRUE
1-stearoyl-2-dihomo-linolenoyl-GPC (18:0/20:3n3 or 6)	Hypertension	0.203	18	0.158	0.031	0.295	TRUE
1-(1-enyl-palmitoyl)-2-docosahexaenoyl-GPC (P-16:0/22:6)	Hypertension	0.193	15	0.804	0.897	0.382	TRUE
palmitoleoyl-oleoyl-glycerol (16:1/18:1) [2]	Hypertension	0.254	14	0.072	0.032	0.142	TRUE
palmitoleoyl-oleoyl-glycerol (16:1/18:1) [1]	Hypertension	0.228	17	0.372	0.550	0.034	TRUE
palmitoyl-linoleoyl-glycerol (16:0/18:2) [1]	Hypertension	0.228	17	1.16e-11	0.877	0.030	TRUE
palmitoyl-linoleoyl-glycerol (16:0/18:2) [2]	Hypertension	0.197	18	3.21e-12	0.570	0.121	TRUE
X - 24425	Hypertension	0.148	18	0.068	0.487	0.218	TRUE
1-palmitoyl-2-oleoyl-GPI (16:0/18:1)	Hypertension	0.318	19	0.649	0.121	0.108	TRUE
1-(1-enyl-palmitoyl)-2-docosahexaenoyl-GPE (P-16:0/22:6)	Hypertension	0.272	17	0.465	0.577	0.274	TRUE
1-(1-enyl-palmitoyl)-2-arachidonoyl-GPE (P-16:0/20:4)	Hypertension	0.657	26	0.968	0.337	0.820	TRUE
1-(1-enyl-palmitoyl)-2-linoleoyl-GPE (P-16:0/18:2)	Hypertension	0.439	18	0.821	0.776	0.390	TRUE
1-(1-enyl-palmitoyl)-2-linoleoyl-GPC (P-16:0/18:2)	Hypertension	0.125	15	0.177	0.040	0.065	TRUE
1-palmitoleoyl-2-linoleoyl-GPC (16:1/18:2)	Hypertension	0.874	40	0.784	0.080	0.627	TRUE
1-palmitoyl-2-alpha-linolenoyl-GPC (16:0/18:3n3)	Hypertension	0.854	34	0.796	0.741	0.657	TRUE
1-(1-enyl-palmitoyl)-2-arachidonoyl-GPC (P-16:0/20:4)	Hypertension	1.8	69	0.675	0.645	0.820	TRUE
1-oleoyl-2-docosahexaenoyl-GPC (18:1/22:6)	Hypertension	0.132	15	0.003	0.763	0.028	TRUE

Exposure	Outcome	R2	F	Heterogeneity	Pleiotropy	power	Direction
1-adrenoyl-GPC (22:4)	Hypertension	0.620	26	0.826	0.717	0.809	TRUE
1-stearoyl-2-docosapentaenoyl-GPC (18:0/22:5n3)	Hypertension	0.644	27	0.063	0.121	0.463	TRUE
lactate	Hypertension	0.146	14	3.13e-07	0.166	0.480	TRUE
1-stearoyl-2-docosapentaenoyl-GPC (18:0/22:5n6)	Hypertension	0.459	20	0.968	0.958	0.687	TRUE
1-(1-enyl-stearoyl)-GPC (P-18:0)	Hypertension	0.116	15	0.997	0.882	0.144	TRUE
1-stearyl-GPC (O-18:0)	Hypertension	0.119	17	1.02e-05	0.073	0.463	TRUE
1-(1-enyl-stearoyl)-2-docosahexaenoyl-GPC (P-18:0/22:6)	Hypertension	0.154	14	0.090	0.516	0.551	TRUE
1-oleoyl-2-dihomo-linolenoyl-GPC (18:1/20:3)	Hypertension	0.607	29	0.985	0.340	0.919	TRUE
1-palmitoyl-2-adrenoyl-GPC (16:0/22:4)	Hypertension	0.129	17	0.371	0.930	0.235	TRUE
1-myristoyl-2-linoleoyl-GPC (14:0/18:2)	Hypertension	0.469	19	0.732	0.977	0.567	TRUE
1-linoleoyl-2-arachidonoyl-GPC (18:2/20:4n6)	Hypertension	1.2	56	0.896	0.039	0.829	TRUE
1-oleoyl-2-eicosapentaenoyl-GPC (18:1/20:5)	Hypertension	0.541	22	0.534	0.575	0.521	TRUE
1-(1-enyl-palmitoyl)-2-palmitoyl-GPC (P-16:0/16:0)	Hypertension	0.183	20	0.549	0.440	0.536	TRUE
phosphatidylcholine (16:0/22:5n3, 18:1/20:4)	Hypertension	2.1	86	0.789	4.59e-04	0.568	TRUE
phosphatidylcholine (18:0/20:5, 16:0/22:5n6)	Hypertension	0.962	33	0.254	0.313	0.609	TRUE
1-palmitoyl-2-meadoyl-GPC (16:0/20:3n9)	Hypertension	0.947	42	0.879	0.260	0.681	TRUE
1-stearoyl-2-meadoyl-GPC (18:0/20:3n9)	Hypertension	1.1	55	0.962	0.318	0.827	TRUE

Exposure	Outcome	R2	F	Heterogeneity	Pleiotropy	power	Direction
glutamine	Hypertension	0.168	18	0.047	0.452	0.210	TRUE
3-hydroxy-3-methylglutarate	Hypertension	0.103	16	0.406	0.930	0.098	TRUE
tryptophan	Hypertension	0.145	14	0.093	0.709	0.200	TRUE
beta-alanine	Hypertension	0.147	17	0.353	0.813	0.070	TRUE
adenosine	Hypertension	0.256	15	0.411	0.307	0.063	TRUE
caffeine	Hypertension	0.234	15	0.002	0.745	0.352	TRUE
glutamate	Hypertension	0.124	17	0.322	0.984	0.044	TRUE
glycine	Hypertension	0.208	32	3.26e-04	0.705	0.195	TRUE
gluconate	Hypertension	0.162	18	0.015	0.404	0.037	TRUE
histidine	Hypertension	0.097	12	0.368	0.474	0.391	TRUE
nicotinamide	Hypertension	0.135	17	0.047	0.964	0.165	TRUE
dihydroorotate	Hypertension	0.224	17	4.53e-06	0.266	0.078	TRUE
uridine	Hypertension	0.124	16	0.061	0.178	0.081	TRUE
trans-urocanate	Hypertension	0.133	19	0.003	0.272	0.032	TRUE
2-aminoadipate	Hypertension	0.225	16	3.34e-05	0.302	0.035	TRUE
cholesterol	Hypertension	0.125	15	0.067	0.599	0.082	TRUE
Carnitine	Hypertension	0.126	68	0.458	0.891	0.367	TRUE

Exposure	Outcome	R2	F	Heterogeneity	Pleiotropy	power	Direction
Benzoate	Hypertension	0.024	22	0.083	0.022	0.405	TRUE
Maltotriose	Hypertension	0.041	23	0.308	0.200	0.584	TRUE
Hippurate	Hypertension	0.024	23	0.044	0.975	0.063	TRUE
Methionine sulfoxide	Hypertension	0.029	25	1.75e-04	0.236	0.563	TRUE
Xanthurenate	Hypertension	0.061	30	0.395	0.895	0.179	TRUE
N-alpha-acetylornithine	Hypertension	0.049	27	0.077	0.654	0.250	TRUE
3-methylhistidine	Hypertension	0.041	23	0.005	0.719	0.065	TRUE
Glycerophosphorylcholine (GPC)	Hypertension	0.035	25	0.613	0.271	0.040	TRUE
Phenylacetate	Hypertension	0.045	27	0.189	0.796	0.115	TRUE
3-methyl-2-oxovalerate	Hypertension	0.024	25	0.209	0.464	0.955	TRUE
Tartarate	Hypertension	0.037	22	0.025	0.654	0.047	TRUE
Suberate (C8-DC)	Hypertension	0.038	23	0.045	0.333	0.027	TRUE
5-hydroxylysine	Hypertension	0.102	74	0.085	0.103	0.782	TRUE
3-phenylpropionate hydrocinnamate	Hypertension	0.049	24	7.92e-04	0.525	0.050	TRUE
Kynurenine	Hypertension	0.067	40	6e-28	0.169	1.000	TRUE
N-acetylglutamate	Hypertension	0.037	26	0.012	0.559	0.215	TRUE
Glycerol 3-phosphate	Hypertension	0.071	33	0.046	0.714	0.035	TRUE

Exposure	Outcome	R2	F	Heterogeneity	Pleiotropy	power	Direction
Imidazole lactate	Hypertension	0.232	121	2.11e-04	0.554	0.933	TRUE
4-guanidinobutanoate	Hypertension	0.031	24	0.034	0.987	0.292	TRUE
Glucuronate	Hypertension	0.021	22	3.43e-05	0.906	0.456	TRUE
Ribitol	Hypertension	0.087	41	0.148	0.747	0.386	TRUE
Glycodeoxycholate	Hypertension	0.018	24	0.180	0.138	0.307	TRUE
Theobromine	Hypertension	0.040	25	0.002	0.114	0.760	TRUE
Quinate	Hypertension	0.036	23	0.012	0.968	0.398	TRUE
Gentisate	Hypertension	0.055	23	0.014	0.966	0.345	TRUE
Theophylline	Hypertension	0.033	23	0.130	0.424	0.947	TRUE
1,5-anhydroglucitol (1,5-ag)	Hypertension	0.079	30	4.23e-06	0.725	0.529	TRUE
1-stearoyl-2-arachidonoyl-GPI (18:0/20:4)	Hypertension	0.089	47	3.61e-10	0.464	0.195	TRUE
Indolelactate	Hypertension	0.052	36	0.002	0.757	0.587	TRUE
Docosahexaenoate DHA; 22:6n3	Hypertension	0.029	24	0.498	0.224	0.275	TRUE
1-stearoyl-2-oleoyl-GPS (18:0/18:1)	Hypertension	0.030	28	0.003	0.651	0.027	TRUE
1-myristoyl-2-palmitoyl-gpc (14:0/16:0)	Hypertension	0.022	23	0.769	0.498	0.482	TRUE
Paraxanthine	Hypertension	0.049	24	0.054	0.702	0.249	TRUE
4-methyl-2-oxopentanoate	Hypertension	0.029	24	0.386	0.803	0.622	TRUE

Exposure	Outcome	R2	F	Heterogeneity	Pleiotropy	power	Direction
3-indoxyl sulfate	Hypertension	0.027	23	0.959	0.734	0.111	TRUE
Sphingosine 1-phosphate	Hypertension	0.034	24	0.149	0.803	0.987	TRUE
Alpha-hydroxyisocaproate	Hypertension	0.040	24	6.28e-04	0.429	0.170	TRUE
1-stearoyl-GPI (18:0)	Hypertension	0.031	23	0.565	0.063	0.223	TRUE
1,2-dipalmitoyl-gpc (16:0/16:0)	Hypertension	0.076	46	4.04e-06	0.806	0.899	TRUE
Maleate	Hypertension	0.034	23	0.036	0.073	0.913	TRUE
Isovalerate (i5:0)	Hypertension	0.027	22	0.082	0.162	0.089	TRUE
4-acetylphenol sulfate	Hypertension	0.028	23	0.113	0.237	0.105	TRUE
Phenyllactate (PLA)	Hypertension	0.087	36	1.15e-05	0.456	0.064	TRUE
N-acetylaspartate (naa)	Hypertension	0.019	23	0.116	0.146	0.035	TRUE
Palmitoylcarnitine	Hypertension	0.055	27	0.112	0.401	0.190	TRUE
Erythritol	Hypertension	0.059	29	0.006	0.978	0.126	TRUE
Hexanoylcarnitine	Hypertension	0.139	88	0.421	0.800	0.389	TRUE
Acetylcarnitine	Hypertension	0.049	34	0.365	0.619	0.338	TRUE
Iminodiacetate (IDA)	Hypertension	0.026	22	0.062	0.887	0.486	TRUE
Cysteine s-sulfate	Hypertension	0.028	23	0.331	0.678	0.089	TRUE
Oxalate (ethanedioate)	Hypertension	0.046	24	1.34e-04	0.937	0.062	TRUE

Exposure	Outcome	R2	F	Heterogeneity	Pleiotropy	power	Direction
DHEAS	Hypertension	0.092	28	6.63e-10	0.578	0.187	TRUE
3-hydroxymyristate	Hypertension	0.021	22	0.025	0.043	0.028	TRUE
Homoarginine	Hypertension	0.140	67	8.85e-05	0.132	0.168	TRUE
1-oleoylglycerol (18:1)	Hypertension	0.058	25	1.6e-06	0.784	0.065	TRUE
3-methyl-2-oxobutyrate	Hypertension	0.041	26	0.019	0.632	0.778	TRUE
Tartronate (hydroxymalonate)	Hypertension	0.038	24	3.47e-08	0.818	0.083	TRUE
2,3-dihydroxypyridine	Hypertension	0.032	22	0.110	0.291	0.293	TRUE
3-hydroxyoctanoate	Hypertension	0.042	25	0.785	0.910	0.551	TRUE
2-hydroxyoctanoate	Hypertension	0.058	32	0.007	0.242	0.256	TRUE
Homocitrulline	Hypertension	0.078	41	7.95e-05	0.895	0.642	TRUE
Indoleacetate	Hypertension	0.058	24	0.253	0.516	0.076	TRUE
1-linoleoylglycerol (18:2)	Hypertension	0.019	23	2.47e-04	0.008	1.000	TRUE
2-linoleoylglycerol (18:2)	Hypertension	0.029	22	0.001	0.229	0.091	TRUE
Indolepropionate	Hypertension	0.030	36	0.004	0.237	0.553	TRUE
Threonate	Hypertension	0.038	24	0.001	0.519	0.180	TRUE
N-acetylglycine	Hypertension	0.108	67	0.232	0.331	0.986	TRUE
Glycolithocholate	Hypertension	0.032	22	0.273	0.600	0.040	TRUE

Exposure	Outcome	R2	F	Heterogeneity	Pleiotropy	power	Direction
EDTA	Hypertension	0.021	22	0.127	0.054	0.060	TRUE
1-methylhistidine	Hypertension	0.061	32	0.005	0.097	0.143	TRUE
Citramalate	Hypertension	0.030	23	8.73e-04	0.991	0.331	TRUE
3-hydroxydecanoate	Hypertension	0.043	36	0.002	0.614	0.074	TRUE
1,3-dimethylurate	Hypertension	0.039	25	0.050	0.770	0.404	TRUE
Galactonate	Hypertension	0.039	25	0.002	0.048	0.428	TRUE
Trigonelline	Hypertension	0.031	23	0.021	0.576	0.139	TRUE
Dodecanedioate	Hypertension	0.048	25	0.804	0.990	0.163	TRUE
Isobutyrylcarnitine (c4)	Hypertension	0.173	62	6.41e-05	0.980	0.069	TRUE
3-methylxanthine	Hypertension	0.048	25	0.004	0.891	0.973	TRUE
3-hydroxylaurate	Hypertension	0.025	26	0.121	0.141	0.149	TRUE
Pyridoxate	Hypertension	0.044	23	0.015	0.519	0.429	TRUE
5-hydroxyhexanoate	Hypertension	0.024	23	0.300	0.369	0.026	TRUE
Gamma-glutamylvaline	Hypertension	0.051	25	1.88e-04	0.207	0.137	TRUE
3-hydroxysebacate	Hypertension	0.046	32	1.98e-07	0.066	0.041	TRUE
Propionylglycine	Hypertension	0.091	52	0.128	0.809	0.949	TRUE
Butyrylglycine	Hypertension	0.161	110	0.052	0.357	0.802	TRUE

Exposure	Outcome	R2	F	Heterogeneity	Pleiotropy	power	Direction
Pro-hydroxy-pro	Hypertension	0.039	23	2.25e-04	0.481	0.036	TRUE
Propionylcarnitine (c3)	Hypertension	0.078	50	0.001	0.692	0.076	TRUE
Adrenate (22:4n6)	Hypertension	0.040	34	3.38e-04	0.418	0.701	TRUE
Docosatrienoate (22:3n3)	Hypertension	0.056	33	0.026	0.573	0.111	TRUE
Docosadienoate (22:2n6)	Hypertension	0.027	23	0.993	0.799	0.141	TRUE
3-carboxy-4-methyl-5-propyl-2-furanpropanoate (cmpf)	Hypertension	0.027	25	0.038	0.892	0.032	TRUE
Docosapentaenoate n3 DPA; 22:5n3	Hypertension	0.040	28	0.132	0.609	0.025	TRUE
3-hydroxy-2-ethylpropionate	Hypertension	0.066	26	3.48e-07	0.082	0.861	TRUE
Myristoleate (14:1n5)	Hypertension	0.034	24	0.024	0.643	0.086	TRUE
10-undecenoate (11:1n1)	Hypertension	0.082	59	0.407	0.359	0.269	TRUE
1-methyl-4-imidazoleacetate	Hypertension	0.107	54	0.578	0.308	0.122	TRUE
Sebacate (C10-DC)	Hypertension	0.032	30	0.516	0.522	0.047	TRUE
Tauro-beta-muricholate	Hypertension	0.094	44	0.026	0.538	0.130	TRUE
Octanoylcarnitine (c8)	Hypertension	0.118	94	0.006	0.149	0.094	TRUE
Stearidonate (18:4n3)	Hypertension	0.047	26	0.181	0.814	0.283	TRUE
Decanoylcarnitine (C10)	Hypertension	0.082	64	0.023	0.156	0.052	TRUE
5-dodecenoate (12:1n7)	Hypertension	0.025	26	0.458	0.829	0.201	TRUE

Exposure	Outcome	R2	F	Heterogeneity	Pleiotropy	power	Direction
N-acetylisoleucine	Hypertension	0.024	22	0.147	0.470	0.251	TRUE
Campesterol	Hypertension	0.072	51	0.112	0.784	0.550	TRUE
N-acetylthreonine	Hypertension	0.043	24	2.24e-10	0.653	0.334	TRUE
1-oleoyl-GPC (18:1)	Hypertension	0.028	29	0.899	0.446	0.512	TRUE
10-nonadecenoate (19:1n9)	Hypertension	0.022	23	0.002	0.301	0.291	TRUE
Piperine	Hypertension	0.041	23	0.001	0.728	0.038	TRUE
Carnitine C14	Hypertension	0.037	25	0.629	0.179	0.544	TRUE
10-heptadecenoate (17:1n7)	Hypertension	0.028	23	0.418	0.793	0.034	TRUE
1-stearoyl-gpc (18:0)	Hypertension	0.050	30	4.01e-04	0.545	0.035	TRUE
1-palmitoyl-GPC (16:0)	Hypertension	0.052	29	0.059	0.716	0.105	TRUE
Hyocholate	Hypertension	0.028	23	0.009	0.462	0.275	TRUE
N-acetylhistidine	Hypertension	0.156	113	0.025	0.541	0.026	TRUE
Alpha-hydroxyisovalerate	Hypertension	0.094	57	4.83e-04	0.459	0.052	TRUE
N-acetylproline	Hypertension	0.031	24	0.252	0.078	0.294	TRUE
Stachydrine	Hypertension	0.028	23	0.057	0.042	0.073	TRUE
Salicyluric glucuronide	Hypertension	0.013	22	0.383	0.838	0.145	TRUE
P-cresol sulfate	Hypertension	0.037	22	0.051	0.118	0.346	TRUE

Exposure	Outcome	R2	F	Heterogeneity	Pleiotropy	power	Direction
Gamma-glutamylglycine	Hypertension	0.160	80	0.012	0.536	1.000	TRUE
1-linoleoyl-gpc (18:2)	Hypertension	0.041	31	0.014	0.601	0.275	TRUE
Stearoylcarnitine	Hypertension	0.064	36	0.059	0.378	0.975	TRUE
Eicosenoate (20:1)	Hypertension	0.035	22	6.4e-06	0.108	0.428	TRUE
Aconitate [cis or trans]	Hypertension	0.040	24	0.043	0.102	0.101	TRUE
Erythronate	Hypertension	0.046	35	5.59e-08	0.748	0.054	TRUE
Isovalerylcarnitine (C5)	Hypertension	0.057	48	0.024	0.988	0.965	TRUE
Linolenate [alpha or gamma; (18:3n3 or 6)]	Hypertension	0.031	23	0.006	0.350	0.219	TRUE
Gamma-glutamylthreonine	Hypertension	0.051	24	2.5e-05	0.454	0.059	TRUE
Laurylcarnitine	Hypertension	0.038	23	0.339	0.115	0.265	TRUE
Gamma-glutamylmethionine	Hypertension	0.036	23	0.199	0.042	0.700	TRUE
Epiandrosterone sulfate	Hypertension	0.175	92	2.36e-08	0.035	0.084	TRUE
Gamma-glutamyltryptophan	Hypertension	0.046	25	4.41e-04	0.154	0.223	TRUE
7-methylxanthine	Hypertension	0.036	25	0.042	0.190	0.821	TRUE
3,7-dimethylurate	Hypertension	0.039	23	0.071	0.114	0.151	TRUE
1,7-dimethyluric acid	Hypertension	0.053	23	0.063	0.347	0.173	TRUE
1,3,7-trimethylurate	Hypertension	0.052	26	7.39e-05	0.988	0.137	TRUE

Exposure	Outcome	R2	F	Heterogeneity	Pleiotropy	power	Direction
N4-acetylcytidine	Hypertension	0.107	34	9.44e-04	0.138	0.713	TRUE
Indolebutyrate	Hypertension	0.046	27	0.044	0.841	0.144	TRUE
5-acetylamino-6-amino-3-methyluracil	Hypertension	0.103	46	0.034	0.972	0.222	TRUE
N1-methylinosine	Hypertension	0.049	24	0.764	0.999	0.399	TRUE
N6-carbamoylthreonyladenosine	Hypertension	0.048	23	7.04e-11	0.606	0.399	TRUE
1-methylxanthine	Hypertension	0.103	46	0.009	0.715	0.096	TRUE
N2,n2-dimethylguanosine	Hypertension	0.025	23	0.046	0.780	0.070	TRUE
4-hydroxyhippurate	Hypertension	0.032	22	0.122	0.995	0.265	TRUE
3-(3-amino-3-carboxypropyl)uridine	Hypertension	0.027	25	8.05e-06	0.819	0.635	TRUE
5,6-dihydrouridine	Hypertension	0.060	29	5.08e-07	0.621	0.031	TRUE
Phenylacetylglutamine	Hypertension	0.032	22	3.89e-04	0.129	0.045	TRUE
3-hydroxydodecanedioate	Hypertension	0.030	23	0.012	0.360	0.110	TRUE
1-arachidonylglycerol (20:4)	Hypertension	0.069	42	0.137	0.091	0.036	TRUE
7-methylguanine	Hypertension	0.036	25	0.162	0.676	0.116	TRUE
1-stearoyl-GPE (18:0)	Hypertension	0.115	37	3.49e-11	0.873	0.122	TRUE
Phenol sulfate	Hypertension	0.036	23	0.002	0.244	0.467	TRUE
Oleoylcarnitine	Hypertension	0.041	35	0.033	0.663	0.201	TRUE

Exposure	Outcome	R2	F	Heterogeneity	Pleiotropy	power	Direction
Gamma-glutamylisoleucine	Hypertension	0.031	24	0.414	0.045	0.231	TRUE
Malonylcarnitine	Hypertension	0.030	23	0.120	0.790	0.786	TRUE
1-palmitoleoyl-GPC (16:1)	Hypertension	0.068	28	5.5e-09	0.604	0.060	TRUE
N-methyl-2-pyridone-5-carboxamide	Hypertension	0.028	24	1.04e-05	0.422	0.102	TRUE
Gamma-glutamyl-2-aminobutyrate	Hypertension	0.052	24	0.113	0.376	0.286	TRUE
1-stearoyl-GPG (18:0)	Hypertension	0.051	30	1.1e-05	0.962	0.146	TRUE
Hexanoylglycine	Hypertension	0.104	52	4.55e-05	0.742	0.066	TRUE
Glutamine degradant	Hypertension	0.091	42	9.44e-05	0.149	1.000	TRUE
5-methyluridine (ribothymidine)	Hypertension	0.180	77	0.057	0.193	0.175	TRUE
Cysteine-glutathione disulfide	Hypertension	0.073	44	0.050	0.177	0.947	TRUE
Isovalerylglycine	Hypertension	0.055	33	0.001	0.549	0.852	TRUE
2-hydroxy-3-methylvalerate	Hypertension	0.052	31	0.030	0.445	0.030	TRUE
Homostachydrine	Hypertension	0.031	24	0.922	0.731	0.220	TRUE
1-arachidonoyl-gpc (20:4n6)	Hypertension	0.169	96	0.002	0.861	0.101	TRUE
2-palmitoyl-GPC (16:0)	Hypertension	0.031	25	1.73e-07	0.802	0.552	TRUE
2-palmitoleoyl-GPC (16:1)	Hypertension	0.054	24	0.010	0.884	0.145	TRUE
1-arachidonoyl-GPE (20:4n6)	Hypertension	0.142	58	0.504	0.273	0.229	TRUE

Exposure	Outcome	R2	F	Heterogeneity	Pleiotropy	power	Direction
1-palmitoyl-GPE (16:0)	Hypertension	0.064	45	3.73e-07	0.717	0.076	TRUE
1-linoleoyl-GPE (18:2)	Hypertension	0.093	41	0.238	0.799	0.292	TRUE
1-oleoyl-GPE (18:1)	Hypertension	0.065	26	0.002	0.966	0.193	TRUE
2-hydroxypalmitate	Hypertension	0.031	24	0.122	0.142	0.412	TRUE
Docosapentaenoate (n6 DPA; 22:5n6)	Hypertension	0.025	26	0.379	0.689	0.129	TRUE
Gulonate	Hypertension	0.037	24	0.380	0.843	0.289	TRUE
Isobutyrylglycine	Hypertension	0.076	33	2.76e-09	0.505	0.267	TRUE
Glutarylcarnitine (c5-dc)	Hypertension	0.180	61	2.53e-06	0.656	0.579	TRUE
Carnitine C5:1	Hypertension	0.047	26	3.58e-07	0.366	0.026	TRUE
Hydroquinone sulfate	Hypertension	0.027	23	0.337	0.906	0.064	TRUE
Beta-hydroxyisovaleroylcarnitine	Hypertension	0.077	41	6.3e-04	0.745	0.743	TRUE
Catechol sulfate	Hypertension	0.054	23	0.003	0.404	0.037	TRUE
7-alpha-hydroxy-3-oxo-4-cholestenoate (7-hoca)	Hypertension	0.040	24	0.116	0.921	0.124	TRUE
Octadecanedioate	Hypertension	0.073	32	0.009	0.466	0.448	TRUE
Tetradecanedioate (C14-DC)	Hypertension	0.113	109	0.705	0.113	0.547	TRUE
Hexadecanedioate (C16-DC)	Hypertension	0.126	92	0.034	0.144	0.600	TRUE
Glycerophosphoethanolamine	Hypertension	0.048	27	0.037	0.018	0.613	TRUE

Exposure	Outcome	R2	F	Heterogeneity	Pleiotropy	power	Direction
Taurolithocholate 3-sulfate	Hypertension	0.053	32	0.258	0.575	0.040	TRUE
1-palmitoyl-GPI (16:0)	Hypertension	0.041	23	0.001	0.076	0.085	TRUE
Glycolithocholate sulfate	Hypertension	0.043	45	0.010	0.431	0.036	TRUE
3-(3-hydroxyphenyl)propionate	Hypertension	0.035	23	5.46e-06	0.322	0.439	TRUE
Deoxycarnitine	Hypertension	0.134	58	0.126	0.013	0.751	TRUE
Alpha-hydroxycaproate	Hypertension	0.020	24	0.070	0.646	0.074	TRUE
1-ribosyl-imidazoleacetate	Hypertension	0.082	33	2.31e-06	0.043	0.554	TRUE
3,4-dihydroxybutyrate	Hypertension	0.048	33	0.003	0.328	0.542	TRUE
Indoleacetylglutamine	Hypertension	0.069	36	0.400	0.300	0.124	TRUE
Mannitol/sorbitol	Hypertension	0.042	23	0.140	0.753	0.029	TRUE
Tryptophan betaine	Hypertension	0.077	32	4.51e-14	0.046	1.000	TRUE
Dihomo-linolenate (20:3n3 or n6)	Hypertension	0.041	26	0.214	0.399	0.842	TRUE
1-linoleoyl-GPI (18:2)	Hypertension	0.070	35	1.83e-06	0.550	0.799	TRUE
1-oleoyl-GPI (18:1)	Hypertension	0.054	25	0.098	0.364	0.276	TRUE
O-cresol sulfate	Hypertension	0.019	23	0.176	0.673	0.084	TRUE
4-vinylphenol sulfate	Hypertension	0.034	24	0.260	0.914	0.041	TRUE
Pyrraline	Hypertension	0.022	23	0.108	0.626	0.932	TRUE

Exposure	Outcome	R2	F	Heterogeneity	Pleiotropy	power	Direction
N6-acetyllysine	Hypertension	0.091	65	5.34e-04	0.265	0.106	TRUE
Thymol sulfate	Hypertension	0.031	28	0.170	0.692	0.106	TRUE
N-acetylserine	Hypertension	0.035	27	4.8e-08	0.690	0.268	TRUE
3-methyladipate	Hypertension	0.026	24	0.015	0.971	0.085	TRUE
4-ethylphenylsulfate	Hypertension	0.026	24	0.001	0.856	0.080	TRUE
1-stearoyl-2-oleoyl-GPE (18:0/18:1)	Hypertension	0.100	47	0.018	0.916	0.987	TRUE
Dimethylarginine (sdma + adma)	Hypertension	0.039	32	0.008	0.186	0.254	TRUE
Hexanoylglutamine	Hypertension	0.123	81	8.9e-05	0.866	0.500	TRUE
Gamma-glutamylalanine	Hypertension	0.032	24	0.018	0.958	0.284	TRUE
5alpha-androstan-3beta,17beta-diol disulfate	Hypertension	0.098	52	0.030	0.725	0.794	TRUE
N-methylproline	Hypertension	0.028	23	0.965	0.824	0.887	TRUE
Carotenoid	Hypertension	0.041	23	0.020	0.684	0.098	TRUE
5alpha-pregnan-3beta,20alpha-diol disulfate	Hypertension	0.102	39	0.008	0.851	0.264	TRUE
Taurocholenate sulfate	Hypertension	0.146	44	9.91e-04	0.176	0.119	TRUE
Pregnenediol disulfate (C21H34O8S2)	Hypertension	0.183	69	7.7e-05	0.725	0.698	TRUE
Glycocholenate sulfate	Hypertension	0.226	83	0.029	0.030	0.135	TRUE
Androstenediol (3beta,17beta) disulfate (1)	Hypertension	0.104	52	0.019	0.242	0.654	TRUE

Exposure	Outcome	R2	F	Heterogeneity	Pleiotropy	power	Direction
Chiro-inositol	Hypertension	0.019	22	0.125	0.356	0.034	TRUE
1-palmitoyl-2-linoleoyl-GPE (16:0/18:2)	Hypertension	0.211	92	8.32e-07	0.538	0.401	TRUE
Sphinganine-1-phosphate	Hypertension	0.022	26	0.040	0.876	0.049	TRUE
Glycosyl-N-stearoyl-sphingosine (d18:1/18:0)	Hypertension	0.087	36	0.314	0.931	0.495	TRUE
4-allylphenol sulfate	Hypertension	0.037	26	5.07e-08	0.614	0.109	TRUE
Succinylcarnitine	Hypertension	0.154	63	1.99e-04	0.794	0.214	TRUE
21-hydroxypregnenolone disulfate	Hypertension	0.112	37	2.24e-14	0.067	0.792	TRUE
5alpha-androstan-3alpha,17alpha-diol monosulfate	Hypertension	0.098	48	8.93e-07	0.330	0.028	TRUE
Androstenediol (3beta,17beta) disulfate (2)	Hypertension	0.099	29	3.86e-08	0.234	0.998	TRUE
5alpha-androstan-3beta,17alpha-diol disulfate	Hypertension	0.048	31	0.044	0.063	0.071	TRUE
5alpha-pregnan-3beta,20beta-diol monosulfate (1)	Hypertension	0.119	64	0.183	0.106	0.475	TRUE
5alpha-pregnan-3beta,20alpha-diol monosulfate (2)	Hypertension	0.085	34	0.256	0.149	0.352	TRUE
5alpha-androstan-3beta,17beta-diol monosulfate (2)	Hypertension	0.110	44	0.069	0.153	0.112	TRUE
Androstenediol (3alpha, 17alpha) monosulfate (3)	Hypertension	0.195	76	0.008	0.561	0.233	TRUE
5alpha-pregnan-diol disulfate	Hypertension	0.053	34	0.345	0.817	0.030	TRUE
5alpha-androstan-3alpha,17beta-diol monosulfate (1)	Hypertension	0.161	78	0.004	0.088	0.233	TRUE
5alpha-androstan-3alpha,17beta-diol disulfate	Hypertension	0.066	28	0.144	0.085	0.738	TRUE

Exposure	Outcome	R2	F	Heterogeneity	Pleiotropy	power	Direction
Andro steroid monosulfate C19H28O6S (1)	Hypertension	0.121	44	0.009	0.615	0.824	TRUE
Pregnenediol sulfate (C21H34O5S)	Hypertension	0.107	35	2.68e-14	0.654	0.250	TRUE
4-hydroxycoumarin	Hypertension	0.040	24	0.046	0.410	0.232	TRUE
16a-hydroxy DHEA 3-sulfate	Hypertension	0.140	64	5.78e-04	0.666	0.799	TRUE
Gamma-CEHC	Hypertension	0.107	36	4.02e-04	0.981	0.400	TRUE
2-hydroxyglutarate	Hypertension	0.048	26	0.329	0.375	0.037	TRUE
N-acetyl-beta-alanine	Hypertension	0.063	33	0.253	0.393	0.100	TRUE
Tridecenedioate (C13:1-DC)	Hypertension	0.033	23	0.003	0.555	0.042	TRUE
N-acetyl-3-methylhistidine	Hypertension	0.102	49	0.451	0.098	0.832	TRUE
2-hydroxyhippurate	Hypertension	0.030	23	0.055	0.115	0.154	TRUE
4-cholesten-3-one	Hypertension	0.050	24	0.169	0.142	0.059	TRUE
Androstenediol (3beta,17beta) monosulfate (2)	Hypertension	0.058	27	1.3e-06	0.630	0.038	TRUE
Androstenediol (3beta,17beta) monosulfate (1)	Hypertension	0.062	29	3.84e-13	0.367	0.101	TRUE
Pregnenolone sulfate	Hypertension	0.079	33	1.34e-12	0.202	0.249	TRUE
S-methylmethionine	Hypertension	0.024	22	0.292	0.477	0.033	TRUE
13-HODE + 9-HODE	Hypertension	0.020	24	0.736	0.965	0.252	TRUE
Palmitoyl sphingomyelin (d18:1/16:0)	Hypertension	0.059	26	2.58e-06	0.532	0.081	TRUE

Exposure	Outcome	R2	F	Heterogeneity	Pleiotropy	power	Direction
Indole-3-carboxylate	Hypertension	0.027	25	0.155	0.987	0.136	TRUE
Sphingomyelin (d18:1/18:1, d18:2/18:0)	Hypertension	0.079	31	9.17e-25	0.008	0.589	TRUE
Ergothioneine	Hypertension	0.049	37	0.046	0.048	0.035	TRUE
Cis-4-decenoylcarnitine (C10:1)	Hypertension	0.115	77	0.170	0.023	0.050	TRUE
5-(galactosylhydroxy)-L-lysine	Hypertension	0.026	24	0.006	0.953	0.353	TRUE
2s,3R-dihydroxybutyrate	Hypertension	0.034	26	0.153	0.196	0.149	TRUE
Cysteinylglycine disulfide	Hypertension	0.070	75	0.615	0.858	0.966	TRUE
2,3-dihydroxyisovalerate	Hypertension	0.040	22	0.574	0.779	0.481	TRUE
Alpha-ketoglutaramate	Hypertension	0.035	42	6.73e-04	0.857	0.032	TRUE
3-methylglutaconate	Hypertension	0.147	98	0.001	0.726	0.494	TRUE
(16 or 17)-methylstearate (a19:0 or i19:0)	Hypertension	0.036	23	0.008	0.889	0.181	TRUE
Cinnamoylglycine	Hypertension	0.072	30	0.046	0.427	0.925	TRUE
2R,3R-dihydroxybutyrate	Hypertension	0.086	46	0.031	0.133	0.049	TRUE
4-hydroxyglutamate	Hypertension	0.050	25	0.012	0.218	0.260	TRUE
S-methylcysteine	Hypertension	0.057	44	0.976	0.905	0.283	TRUE
Isoursodeoxycholate	Hypertension	0.026	24	0.054	0.127	0.222	TRUE
Cis-4-decenoate (10:1n6)	Hypertension	0.155	105	0.106	0.285	0.152	TRUE

Exposure	Outcome	R2	F	Heterogeneity	Pleiotropy	power	Direction
4-oxo-retinoic acid	Hypertension	0.037	28	0.005	0.815	0.209	TRUE
1-lignoceroyl-GPC (24:0)	Hypertension	0.027	28	0.698	0.450	0.040	TRUE
Sulfate	Hypertension	0.055	23	0.004	0.136	0.909	TRUE
4-hydroxy-2-oxoglutaric acid	Hypertension	0.050	23	0.085	0.516	0.115	TRUE
Glycoursodeoxycholate	Hypertension	0.040	22	0.022	0.132	0.075	TRUE
1-(1-enyl-palmitoyl)-GPC (p-16:0)	Hypertension	0.051	33	0.119	0.259	0.094	TRUE
Argininate	Hypertension	0.069	41	0.107	0.734	0.830	TRUE
1-methyl-5-imidazoleacetate	Hypertension	0.036	25	0.003	0.689	0.473	TRUE
Hydantoin-5-propionate	Hypertension	0.026	31	0.727	0.822	0.433	TRUE
2-oxoarginine	Hypertension	0.035	27	4.2e-04	0.063	0.074	TRUE
Pantoate	Hypertension	0.036	23	0.003	0.206	0.400	TRUE
Formiminoglutamate	Hypertension	0.035	25	1.37e-09	0.499	0.964	TRUE
1-(1-enyl-palmitoyl)-GPE (p-16:0)	Hypertension	0.038	25	0.094	0.385	0.046	TRUE
S-methylcysteine sulfoxide	Hypertension	0.025	23	0.820	0.821	0.036	TRUE
2-o-methylascorbic acid	Hypertension	0.156	88	9.3e-04	0.115	0.930	TRUE
Eicosanedioate (C20-DC)	Hypertension	0.025	30	0.291	0.555	0.292	TRUE
1-(1-enyl-stearoyl)-GPE (p-18:0)	Hypertension	0.040	28	0.002	0.637	0.035	TRUE

Exposure	Outcome	R2	F	Heterogeneity	Pleiotropy	power	Direction
Beta-citrylglutamate	Hypertension	0.121	107	0.030	0.186	0.035	TRUE
N-oleoyltaurine	Hypertension	0.069	32	0.004	0.790	0.560	TRUE
Docosadioate (C22-DC)	Hypertension	0.028	23	4.71e-04	0.168	0.037	TRUE
Oleoyl-linoleoyl-glycerol (18:1/18:2) [2]	Hypertension	0.080	34	1.12e-12	0.405	0.988	TRUE
Imidazole propionate	Hypertension	0.047	26	1.54e-05	0.052	0.240	TRUE
Carnitine C18:2	Hypertension	0.083	47	0.015	0.938	0.548	TRUE
Pregnanediol-3-glucuronide	Hypertension	0.086	42	0.066	0.998	0.245	TRUE
Carboxyethyl-gaba	Hypertension	0.049	34	0.046	0.936	0.190	TRUE
Alliin	Hypertension	0.079	56	1.57e-04	0.097	0.188	TRUE
Trimethylamine n-oxide	Hypertension	0.016	22	0.138	0.652	0.144	TRUE
Dihydroferulate	Hypertension	0.013	22	0.332	0.417	0.106	TRUE
Prolylglycine	Hypertension	0.034	26	4.55e-05	0.362	0.076	TRUE
N-palmitoylglycine	Hypertension	0.098	64	0.027	0.817	0.883	TRUE
N-methyltaurine	Hypertension	0.050	23	0.177	0.915	0.063	TRUE
Succinimide	Hypertension	0.027	25	0.030	0.415	0.041	TRUE
N-acetylcarnosine	Hypertension	0.117	58	8.37e-12	0.018	0.731	TRUE
Histidine betaine (hercynine)	Hypertension	0.037	28	0.079	0.161	0.101	TRUE

Exposure	Outcome	R2	F	Heterogeneity	Pleiotropy	power	Direction
2-stearoyl-GPE (18:0)	Hypertension	0.061	27	0.215	0.060	0.567	TRUE
Margaroylcarnitine (C17)	Hypertension	0.037	26	0.004	0.823	0.152	TRUE
(R)-3-hydroxybutyrylcarnitine	Hypertension	0.031	25	0.013	0.295	0.052	TRUE
Lanthionine	Hypertension	0.043	22	1.27e-04	0.662	0.076	TRUE
Mannonate	Hypertension	0.052	40	0.010	0.132	0.778	TRUE
3b-hydroxy-5-cholenoic acid	Hypertension	0.041	26	8.28e-04	0.386	0.046	TRUE
2-hydroxydecanoate	Hypertension	0.038	26	0.139	0.383	0.604	TRUE
Glycohyocholate	Hypertension	0.037	28	0.085	0.271	0.337	TRUE
3-methyl catechol sulfate (2)	Hypertension	0.040	22	0.100	0.528	0.028	TRUE
2-aminooctanoate	Hypertension	0.148	83	0.024	0.190	0.825	TRUE
3-methyl catechol sulfate (1)	Hypertension	0.025	23	0.002	0.406	0.392	TRUE
4-methylcatechol sulfate	Hypertension	0.038	24	0.028	0.698	0.060	TRUE
Guaiacol sulfate	Hypertension	0.039	23	0.002	0.505	0.654	TRUE
Gamma-CEHC glucuronide	Hypertension	0.046	29	0.071	0.099	0.087	TRUE
Dimethyl sulfone	Hypertension	0.038	25	0.149	0.751	0.044	TRUE
2-piperidinone	Hypertension	0.039	23	0.907	0.575	0.057	TRUE
Indolin-2-one	Hypertension	0.035	32	0.054	0.906	0.345	TRUE

Exposure	Outcome	R2	F	Heterogeneity	Pleiotropy	power	Direction
Sphingomyelin (d18:1/14:0, d16:1/16:0)	Hypertension	0.107	51	0.014	0.384	0.042	TRUE
3-acetylphenol sulfate	Hypertension	0.032	22	2.28e-07	0.044	0.350	TRUE
Sphingomyelin (d18:2/16:0, d18:1/16:1)	Hypertension	0.039	23	0.269	0.782	0.240	TRUE
S-allylcysteine	Hypertension	0.028	26	0.522	0.562	0.030	TRUE
6-oxopiperidine-2-carboxylate	Hypertension	0.075	59	0.112	0.340	0.029	TRUE
2-aminophenol sulfate	Hypertension	0.048	22	0.001	0.018	0.241	TRUE
N-acetylalliin	Hypertension	0.039	23	0.564	0.704	0.427	TRUE
2-aminoheptanoate	Hypertension	0.027	23	0.009	0.441	0.866	TRUE
3-methoxytyramine sulfate	Hypertension	0.026	24	0.026	0.821	0.236	TRUE
N2,N5-diacetylornithine	Hypertension	0.111	56	9.05e-04	0.737	0.092	TRUE
Ferulic acid 4-sulfate	Hypertension	0.095	38	0.001	0.289	0.975	TRUE
1H-indole-7-acetic acid	Hypertension	0.035	22	0.070	0.979	0.061	TRUE
3-(3-hydroxyphenyl)propionate sulfate	Hypertension	0.029	22	0.225	0.664	0.043	TRUE
1-linolenoyl-GPC (18:3)	Hypertension	0.062	24	0.011	0.795	0.864	TRUE
O-sulfo-l-tyrosine	Hypertension	0.061	28	2.28e-14	0.378	0.166	TRUE
N-formylanthranilic acid	Hypertension	0.161	97	2.32e-04	0.804	0.802	TRUE
Methionine sulfone	Hypertension	0.169	61	3.85e-04	0.563	0.981	TRUE

Exposure	Outcome	R2	F	Heterogeneity	Pleiotropy	power	Direction
Acisoga	Hypertension	0.096	40	0.005	0.912	0.755	TRUE
17alpha-hydroxypregnanolone glucuronide	Hypertension	0.110	52	0.001	0.244	0.621	TRUE
Etiocholanolone glucuronide	Hypertension	0.127	86	0.048	0.244	0.292	TRUE
Fructosyllysine	Hypertension	0.042	23	2.31e-04	0.951	0.117	TRUE
1-(1-enyl-oleoyl)-GPE (p-18:1)	Hypertension	0.019	23	0.925	0.640	0.089	TRUE
N-acetyltaurine	Hypertension	0.056	36	1.02e-04	0.339	0.792	TRUE
1-palmitoyl-GPG (16:0)	Hypertension	0.062	31	0.069	0.993	0.197	TRUE
1-oleoyl-GPG (18:1)	Hypertension	0.091	60	7.48e-05	0.032	0.052	TRUE
Octadecenedioylcarnitine (C18:1-DC)	Hypertension	0.147	100	0.400	0.924	0.705	TRUE
3-methylglutarylcarnitine (2)	Hypertension	0.170	69	9.31e-08	0.016	0.161	TRUE
Sphingomyelin (d18:1/24:1, d18:2/24:0)	Hypertension	0.022	23	0.901	0.822	0.512	TRUE
Methyl glucopyranoside (alpha + beta)	Hypertension	0.114	58	0.564	0.817	0.201	TRUE
9-hydroxystearate	Hypertension	0.046	24	0.023	0.319	0.996	TRUE
Sphingomyelin (d18:2/14:0, d18:1/14:1)	Hypertension	0.042	25	5.49e-05	0.835	0.999	TRUE
1-dihomo-linolenylglycerol (20:3)	Hypertension	0.038	24	0.006	0.267	0.628	TRUE
4-hydroxychlorothalonil	Hypertension	0.088	58	0.023	0.265	0.036	TRUE
Vanillactate	Hypertension	0.046	25	0.008	0.472	0.479	TRUE

Exposure	Outcome	R2	F	Heterogeneity	Pleiotropy	power	Direction
Carnitine C14:1	Hypertension	0.044	23	1.95e-04	0.635	0.075	TRUE
N-formylphenylalanine	Hypertension	0.039	23	0.209	0.604	0.454	TRUE
2-acetamidophenol sulfate	Hypertension	0.027	23	0.022	0.749	0.203	TRUE
4-vinylguaiacol sulfate	Hypertension	0.017	23	0.001	0.695	0.789	TRUE
5alpha-androstan-3alpha,17beta-diol monosulfate (2)	Hypertension	0.070	33	0.002	0.729	0.337	TRUE
Vanillic alcohol sulfate	Hypertension	0.040	24	0.085	0.067	0.858	TRUE
Methyl-4-hydroxybenzoate sulfate	Hypertension	0.038	24	0.330	0.768	0.388	TRUE
Eugenol sulfate	Hypertension	0.030	25	0.917	0.641	0.110	TRUE
2-methoxyresorcinol sulfate	Hypertension	0.033	23	0.910	0.913	0.454	TRUE
Tyramine O-sulfate	Hypertension	0.025	23	0.928	0.846	0.032	TRUE
4-methylguaiacol sulfate	Hypertension	0.024	22	0.107	0.421	0.345	TRUE
3-hydroxypyridine sulfate	Hypertension	0.034	24	0.023	0.784	0.199	TRUE
Arabonate/xylonate	Hypertension	0.050	26	1.65e-09	0.743	0.968	TRUE
Octadecanedioylcarnitine (C18-DC)	Hypertension	0.149	81	0.184	0.563	0.558	TRUE
Phenylacetylcarnitine	Hypertension	0.060	31	0.085	0.378	0.265	TRUE
Ethylparaben sulfate	Hypertension	0.032	22	0.132	0.107	0.036	TRUE
Sphingomyelin (d18:1/20:2, d18:2/20:1, d16:1/22:2)	Hypertension	0.064	39	0.009	0.612	0.026	TRUE

Exposure	Outcome	R2	F	Heterogeneity	Pleiotropy	power	Direction
Sphingomyelin (d18:1/20:1, d18:2/20:0)	Hypertension	0.084	39	0.020	0.180	0.041	TRUE
Behenoyl sphingomyelin (d18:1/22:0)	Hypertension	0.028	23	0.131	0.320	0.162	TRUE
Propyl 4-hydroxybenzoate sulfate	Hypertension	0.027	23	1.73e-04	0.642	0.265	TRUE
4-methoxyphenol sulfate	Hypertension	0.037	24	0.005	0.126	0.029	TRUE
Umbelliferone sulfate	Hypertension	0.036	23	0.010	0.714	0.256	TRUE
Sphingomyelin (d18:1/20:0, d16:1/22:0)	Hypertension	0.110	51	1.25e-04	0.631	0.141	TRUE
6-hydroxyindole sulfate	Hypertension	0.027	28	0.513	0.173	0.253	TRUE
P-cresol glucuronide	Hypertension	0.043	26	0.514	0.384	0.122	TRUE
N-carbamoylalanine	Hypertension	0.026	22	0.444	0.548	0.058	TRUE
Citraconate/glutaconate	Hypertension	0.042	23	0.067	0.195	0.033	TRUE
1,2,3-benzenetriol sulfate (2)	Hypertension	0.029	22	0.072	0.415	0.355	TRUE
3-methoxycatechol sulfate (2)	Hypertension	0.042	23	0.009	0.219	0.477	TRUE
C-glycosyltryptophan	Hypertension	0.071	24	1.1e-07	0.256	0.821	TRUE
3-methoxycatechol sulfate (1)	Hypertension	0.040	22	0.004	0.194	0.115	TRUE
Sphingomyelin (d18:1/22:1, d18:2/22:0, d16:1/24:1)	Hypertension	0.063	33	0.022	0.505	0.033	TRUE
Arabitol/xylitol	Hypertension	0.045	25	1.98e-06	0.354	0.142	TRUE
3-hydroxyhexanoate	Hypertension	0.030	28	0.377	0.635	0.045	TRUE

Exposure	Outcome	R2	F	Heterogeneity	Pleiotropy	power	Direction
Sphingomyelin (d18:1/22:2, d18:2/22:1, d16:1/24:2)	Hypertension	0.061	36	0.004	0.409	0.827	TRUE
3beta-hydroxy-5-cholestenoate	Hypertension	0.103	48	4.52e-04	0.698	0.058	TRUE
N-acetylkynurenine (2)	Hypertension	0.150	122	0.214	0.044	0.653	TRUE
Dopamine 4-sulfate	Hypertension	0.046	24	0.017	0.528	0.027	TRUE
Sphingomyelin (d17:1/16:0, d18:1/15:0, d16:1/17:0)	Hypertension	0.075	42	0.001	0.992	0.360	TRUE
Adipoylcarnitine (C6-DC)	Hypertension	0.080	45	0.009	0.582	0.094	TRUE
Dopamine 3-o-sulfate	Hypertension	0.031	24	0.098	0.664	0.143	TRUE
Lignoceroyl sphingomyelin (d18:1/24:0)	Hypertension	0.042	25	0.017	0.178	0.078	TRUE
N-acetylglucosamine/n-acetylgalactosamine	Hypertension	0.071	50	0.043	0.496	0.530	TRUE
Glycochenodeoxycholate 3-sulfate	Hypertension	0.106	42	0.002	0.151	0.156	TRUE
Trans 3,4-methyleneheptanoate	Hypertension	0.025	23	0.007	0.744	0.147	TRUE
Taurodeoxycholic acid 3-sulfate	Hypertension	0.101	79	0.352	0.461	0.460	TRUE
Phenol glucuronide	Hypertension	0.036	23	0.355	0.262	0.121	TRUE
Glycodeoxycholate 3-sulfate	Hypertension	0.159	76	0.377	0.038	0.248	TRUE
4-hydroxyphenylacetoylcarnitine	Hypertension	0.064	32	0.625	0.461	0.088	TRUE
1-stearoyl-2-oleoyl-gpc (18:0/18:1)	Hypertension	0.060	25	0.637	0.068	0.078	TRUE
Linoleoyl ethanolamide	Hypertension	0.041	34	0.148	0.297	0.471	TRUE

Exposure	Outcome	R2	F	Heterogeneity	Pleiotropy	power	Direction
1,2-dilinoleoyl-GPC (18:2/18:2)	Hypertension	0.066	57	0.077	0.731	0.037	TRUE
Nonanoylcarnitine (C9)	Hypertension	0.102	88	0.011	0.006	0.247	TRUE
2-hydroxybutyrate/2-hydroxyisobutyrate	Hypertension	0.018	25	0.002	0.362	0.422	TRUE
Sphingomyelin (d18:2/23:0, d18:1/23:1, d17:1/24:1)	Hypertension	0.036	27	0.046	0.939	0.443	TRUE
Tricosanoyl sphingomyelin (d18:1/23:0)	Hypertension	0.040	24	9.13e-05	0.853	0.030	TRUE
Palmitoyl dihydrosphingomyelin (d18:0/16:0)	Hypertension	0.076	27	0.024	0.008	0.420	TRUE
1-palmitoleoylglycerol (16:1)	Hypertension	0.058	23	0.426	0.953	0.347	TRUE
1-stearoyl-2-linoleoyl-gpc (18:0/18:2)	Hypertension	0.069	36	0.013	0.918	0.213	TRUE
1-palmitoyl-2-palmitoleoyl-gpc (16:0/16:1)	Hypertension	0.038	22	0.010	0.352	0.037	TRUE
1-stearoyl-2-linoleoyl-GPE (18:0/18:2)	Hypertension	0.131	70	9.38e-08	0.892	0.730	TRUE
Sphingomyelin (d18:2/24:1, d18:1/24:2)	Hypertension	0.045	23	7.02e-09	0.244	1.000	TRUE
1-palmitoyl-2-stearoyl-gpc (16:0/18:0)	Hypertension	0.074	34	4.9e-06	0.754	0.242	TRUE
Oleate/vaccenate (18:1)	Hypertension	0.039	23	5.19e-06	0.342	0.026	TRUE
1-palmitoyl-2-docosahexaenoyl-gpc (16:0/22:6)	Hypertension	0.056	39	4.3e-06	0.738	0.402	TRUE
1-(1-enyl-stearoyl)-2-oleoyl-GPE (p-18:0/18:1)	Hypertension	0.032	27	0.113	0.169	0.046	TRUE
1-stearoyl-2-arachidonoyl-GPE (18:0/20:4)	Hypertension	0.182	82	4.17e-10	0.890	0.646	TRUE
1-stearoyl-2-docosahexaenoyl-gpc (18:0/22:6)	Hypertension	0.089	41	4.52e-04	0.074	0.561	TRUE

Exposure	Outcome	R2	F	Heterogeneity	Pleiotropy	power	Direction
Sphingomyelin (d18:1/17:0, d17:1/18:0, d19:1/16:0)	Hypertension	0.058	29	0.012	0.612	0.046	TRUE
1-(1-enyl-palmitoyl)-2-arachidonoyl-GPE (p-16:0/20:4)	Hypertension	0.037	31	0.397	0.414	0.147	TRUE
Myristoyl dihydrosphingomyelin (d18:0/14:0)	Hypertension	0.050	32	0.097	0.188	0.124	TRUE
5-hydroxyindole sulfate	Hypertension	0.030	25	0.046	0.211	0.464	TRUE
1-palmitoyl-2-dihomo-linolenoyl-GPC (16:0/20:3n3 or 6)	Hypertension	0.100	57	0.832	0.445	0.517	TRUE
1-(1-enyl-palmitoyl)-2-arachidonoyl-gpc (p-16:0/20:4)	Hypertension	0.185	71	0.003	0.377	0.288	TRUE
Sphingomyelin (d18:1/21:0, d17:1/22:0, d16:1/23:0)	Hypertension	0.075	49	0.004	0.139	0.122	TRUE
1-palmitoyl-2-arachidonoyl-GPE (16:0/20:4)	Hypertension	0.174	108	7.26e-07	0.714	0.081	TRUE
Lactosyl-N-palmitoyl-sphingosine (d18:1/16:0)	Hypertension	0.053	32	0.039	0.439	0.149	TRUE
Sphingomyelin (d18:0/18:0, d19:0/17:0)	Hypertension	0.059	26	0.010	0.091	0.188	TRUE
1-(1-enyl-palmitoyl)-2-oleoyl-gpc (p-16:0/18:1)	Hypertension	0.060	28	0.133	0.302	0.041	TRUE
1-(1-enyl-stearoyl)-2-arachidonoyl-GPE (p-18:0/20:4)	Hypertension	0.072	47	0.003	0.900	0.155	TRUE
Behenoyl dihydrosphingomyelin (d18:0/22:0)	Hypertension	0.078	28	1.4e-05	0.900	0.118	TRUE
1-(1-enyl-palmitoyl)-2-linoleoyl-GPC (p-16:0/18:2)	Hypertension	0.034	26	3.34e-06	0.741	0.999	TRUE
1-(1-enyl-palmitoyl)-2-oleoyl-GPE (p-16:0/18:1)	Hypertension	0.051	25	0.137	0.154	0.913	TRUE
1-palmitoyl-2-arachidonoyl-GPI (16:0/20:4)	Hypertension	0.049	31	0.690	0.441	0.286	TRUE
1-palmitoyl-2-docosahexaenoyl-GPE (16:0/22:6)	Hypertension	0.136	99	5.95e-07	0.282	0.812	TRUE

Exposure	Outcome	R2	F	Heterogeneity	Pleiotropy	power	Direction
N-palmitoyl-sphinganine (d18:0/16:0)	Hypertension	0.048	25	3.18e-10	0.693	0.155	TRUE
1-stearoyl-2-docosahexaenoyl-GPE (18:0/22:6)	Hypertension	0.105	49	2.98e-05	0.797	0.760	TRUE
Gamma-tocopherol/beta-tocopherol	Hypertension	0.042	23	0.393	0.283	0.152	TRUE
1-stearoyl-2-linoleoyl-GPI (18:0/18:2)	Hypertension	0.097	48	9.43e-12	0.345	0.105	TRUE
1,2-dilinoleoyl-GPE (18:2/18:2)	Hypertension	0.048	41	0.578	0.143	0.517	TRUE
Phenylacetylglutamate	Hypertension	0.046	27	0.353	0.200	0.211	TRUE
1-(1-enyl-palmitoyl)-2-palmitoleoyl-GPC (P-16:0/16:1)	Hypertension	0.140	50	5.02e-09	0.637	0.986	TRUE
Palmitoylcholine	Hypertension	0.052	23	0.125	0.209	0.044	TRUE
Thioproline	Hypertension	0.032	26	0.380	0.599	0.175	TRUE
1-linoleoyl-2-arachidonoyl-GPC (18:2/20:4n6)	Hypertension	0.109	46	0.047	0.694	0.879	TRUE
1-linoleoyl-GPG (18:2)	Hypertension	0.102	51	0.459	0.251	0.285	TRUE
1-(1-enyl-palmitoyl)-2-linoleoyl-GPE (p-16:0/18:2)	Hypertension	0.057	34	0.011	0.626	0.373	TRUE
1-(1-enyl-stearoyl)-2-linoleoyl-GPE (p-18:0/18:2)	Hypertension	0.053	34	0.039	0.297	0.654	TRUE
1-stearoyl-2-oleoyl-GPI (18:0/18:1)	Hypertension	0.084	44	3.88e-05	0.060	0.456	TRUE
1-myristoyl-2-arachidonoyl-GPC (14:0/20:4)	Hypertension	0.107	51	1.64e-05	0.679	0.723	TRUE
1-oleoyl-2-arachidonoyl-GPE (18:1/20:4)	Hypertension	0.164	79	5.49e-06	0.521	0.113	TRUE
1-palmitoyl-2-oleoyl-GPI (16:0/18:1)	Hypertension	0.063	38	0.105	0.393	0.587	TRUE

Exposure	Outcome	R2	F	Heterogeneity	Pleiotropy	power	Direction
1-oleoyl-2-docosahexaenoyl-GPC (18:1/22:6)	Hypertension	0.070	26	7.22e-08	0.083	0.049	TRUE
1-oleoyl-2-linoleoyl-GPE (18:1/18:2)	Hypertension	0.153	78	3.41e-09	0.676	0.135	TRUE
1-(1-enyl-palmitoyl)-2-palmitoyl-GPC (P-16:0/16:0)	Hypertension	0.143	58	0.014	0.437	0.176	TRUE
Oleoylcholine	Hypertension	0.022	23	0.170	0.948	0.131	TRUE
Glycosyl-N-palmitoyl-sphingosine (d18:1/16:0)	Hypertension	0.077	43	0.007	0.653	0.341	TRUE
Arachidonoylcholine	Hypertension	0.043	36	0.070	0.603	0.030	TRUE
2-methylserine	Hypertension	0.040	23	0.001	0.132	0.027	TRUE
(S)-3-hydroxybutyrylcarnitine	Hypertension	0.054	35	0.005	0.342	0.914	TRUE
Methylsuccinoylcarnitine	Hypertension	0.151	64	0.001	0.060	0.750	TRUE
Dihomo-linolenoyl-choline	Hypertension	0.047	23	1.14e-04	0.193	0.312	TRUE
Docosahexaenoylcholine	Hypertension	0.030	22	0.158	0.777	0.100	TRUE
Glycocholate glucuronide (1)	Hypertension	0.028	23	0.080	0.539	0.028	TRUE
Caffeic acid sulfate	Hypertension	0.047	23	0.128	0.195	0.160	TRUE
Ascorbic acid 2-sulfate	Hypertension	0.045	25	0.019	0.501	0.090	TRUE
1-linoleoyl-2-linolenoyl-GPC (18:2/18:3)	Hypertension	0.059	38	4.97e-04	0.468	0.027	TRUE
Catechol glucuronide	Hypertension	0.031	22	0.678	0.381	0.034	TRUE
Furaneol sulfate	Hypertension	0.045	38	0.302	0.764	0.450	TRUE

Exposure	Outcome	R2	F	Heterogeneity	Pleiotropy	power	Direction
Ceramide (d18:1/24:1)	Hypertension	0.109	49	0.009	0.937	0.044	TRUE
N-oleoylserine	Hypertension	0.055	39	0.585	0.637	0.046	TRUE
Perfluorooctanesulfonate (PFOS)	Hypertension	0.036	23	0.081	0.733	0.537	TRUE
Pimeloylcarnitine/3-methyladipoylcarnitine (C7-DC)	Hypertension	0.070	39	0.116	0.703	0.048	TRUE
1-palmitoleoyl-2-linolenoyl-GPC (16:1/18:3)	Hypertension	0.074	45	0.281	0.386	0.462	TRUE
Linoleoyl-arachidonoyl-glycerol (18:2/20:4) [1]	Hypertension	0.086	61	8.32e-12	0.840	0.391	TRUE
Hexadecadienoate (16:2n6)	Hypertension	0.019	22	0.181	0.709	0.792	TRUE
Glycosyl-N-behenoyl-sphingadienine (d18:2/22:0)	Hypertension	0.092	39	0.010	0.999	0.141	TRUE
4-hydroxyphenylacetylglutamine	Hypertension	0.042	25	6.25e-06	0.041	0.646	TRUE
Gamma-glutamyl-alpha-lysine	Hypertension	0.053	25	0.446	0.533	0.982	TRUE
N-palmitoyl-sphingadienine (d18:2/16:0)	Hypertension	0.030	23	0.065	0.378	0.773	TRUE
Linoleoyl-arachidonoyl-glycerol (18:2/20:4) [2]	Hypertension	0.119	44	3.79e-05	0.227	0.755	TRUE
Palmitoleoylcarnitine (C16:1)	Hypertension	0.038	26	0.002	0.345	0.769	TRUE
N-stearoyl-sphingadienine (d18:2/18:0)	Hypertension	0.037	25	0.025	0.022	0.091	TRUE
2,3-dihydroxy-2-methylbutyrate	Hypertension	0.022	23	0.439	0.790	0.522	TRUE
Stearoylcholine	Hypertension	0.028	23	0.160	0.692	0.963	TRUE
Glycosyl-N-tricosanoyl-sphingadienine (d18:2/23:0)	Hypertension	0.047	36	0.043	0.448	0.141	TRUE

Exposure	Outcome	R2	F	Heterogeneity	Pleiotropy	power	Direction
N-palmitoyl-heptadecasphingosine (d17:1/16:0)	Hypertension	0.060	31	2.33e-04	0.009	0.078	TRUE
Ceramide (d18:1/14:0, d16:1/16:0)	Hypertension	0.068	30	3.79e-04	0.777	0.707	TRUE
Glycosyl-N-(2-hydroxynervonoyl)-sphingosine (d18:1/24:1(2OH))	Hypertension	0.099	52	9.43e-08	0.376	0.703	TRUE
Glycosyl ceramide (d18:2/24:1, d18:1/24:2)	Hypertension	0.087	38	0.148	0.812	0.052	TRUE
Glycosyl ceramide (d18:1/23:1, d17:1/24:1)	Hypertension	0.038	29	0.020	0.517	0.052	TRUE
Sphingadienine	Hypertension	0.029	24	0.323	0.779	0.049	TRUE
Ceramide (d18:1/17:0, d17:1/18:0)	Hypertension	0.041	24	0.042	0.601	0.175	TRUE
Nisinate (24:6n3)	Hypertension	0.032	26	0.059	0.324	0.037	TRUE
Sphingomyelin (d18:0/20:0, d16:0/22:0)	Hypertension	0.129	39	0.013	0.553	0.087	TRUE
Sphingomyelin (d18:2/24:2)	Hypertension	0.040	24	1.91e-05	0.982	0.919	TRUE
Sphingomyelin (d17:2/16:0, d18:2/15:0)	Hypertension	0.062	32	0.063	0.258	0.062	TRUE
Sphingomyelin (d18:2/23:1)	Hypertension	0.049	27	0.002	0.601	0.104	TRUE
Behenoylcarnitine (C22)	Hypertension	0.069	30	0.005	0.443	0.537	TRUE
Sphingomyelin (d18:2/18:1)	Hypertension	0.027	22	0.207	0.453	0.084	TRUE
Sphingomyelin (d18:2/21:0, d16:2/23:0)	Hypertension	0.069	30	2.43e-04	0.812	0.721	TRUE
Linoleoylcholine	Hypertension	0.042	23	0.060	0.662	0.062	TRUE
Sphingomyelin (d18:1/19:0, d19:1/18:0)	Hypertension	0.094	49	0.002	0.687	0.649	TRUE

Exposure	Outcome	R2	F	Heterogeneity	Pleiotropy	power	Direction
Linolenoylcarnitine (C18:3)	Hypertension	0.061	28	0.039	0.872	0.666	TRUE
Heneicosapentaenoate (21:5n3)	Hypertension	0.024	23	0.259	0.364	0.058	TRUE
Sphingomyelin (d18:1/25:0, d19:0/24:1, d20:1/23:0, d19:1/24:0)	Hypertension	0.131	54	0.139	0.566	0.070	TRUE
Arachidoylcarnitine (C20)	Hypertension	0.060	29	0.152	0.715	0.532	TRUE
Cerotoylcarnitine (C26)	Hypertension	0.123	49	3.65e-06	0.436	0.950	TRUE
Lignoceroylcarnitine (C24)	Hypertension	0.093	56	0.008	0.961	0.818	TRUE
Docosatrienoate (22:3n6)	Hypertension	0.038	29	0.100	0.969	0.247	TRUE
Dihomo-linolenoylcarnitine (C20:3n3 or 6)	Hypertension	0.207	78	0.125	0.991	1.000	TRUE
Carotene diol (2)	Hypertension	0.070	37	0.586	0.373	0.136	TRUE
Ethyl alpha-glucopyranoside	Hypertension	0.043	23	0.150	0.789	0.148	TRUE
Carotene diol (1)	Hypertension	0.087	36	0.112	0.684	0.039	TRUE
Nervonoylcarnitine (C24:1)	Hypertension	0.081	45	0.062	0.053	0.615	TRUE
Dihomo-linoleoylcarnitine (C20:2)	Hypertension	0.100	47	0.008	0.530	0.436	TRUE
Glycosyl ceramide (d18:1/20:0, d16:1/22:0)	Hypertension	0.111	44	0.130	0.684	0.923	TRUE
Docosahexaenoylcarnitine (C22:6)	Hypertension	0.064	25	0.015	0.208	0.030	TRUE
N,N,N-trimethyl-5-aminovalerate	Hypertension	0.077	32	9.31e-08	0.555	0.331	TRUE
Eicosenoylcarnitine (C20:1)	Hypertension	0.074	28	0.034	0.750	0.677	TRUE

Exposure	Outcome	R2	F	Heterogeneity	Pleiotropy	power	Direction
Ximenoylcarnitine (C26:1)	Hypertension	0.124	55	0.002	0.890	0.974	TRUE
Arachidonoylcarnitine (C20:4)	Hypertension	0.235	85	0.006	0.477	0.999	TRUE
5-dodecenoylcarnitine (C12:1)	Hypertension	0.047	24	0.025	0.372	0.996	TRUE
2-butenoylglycine	Hypertension	0.077	38	0.184	0.373	1.000	TRUE
(N(1) + N(8))-acetylspermidine	Hypertension	0.134	51	7.15e-04	0.194	0.925	TRUE
Hydroxy-cmpf	Hypertension	0.033	25	0.170	0.842	0.206	TRUE
2-furoylcarnitine	Hypertension	0.024	23	0.723	0.324	0.040	TRUE
Cortolone glucuronide (1)	Hypertension	0.070	31	0.079	0.133	0.492	TRUE
Carotene diol (3)	Hypertension	0.061	32	0.004	0.078	0.242	TRUE
3-hydroxyoleoylcarnitine	Hypertension	0.049	25	3.92e-05	0.469	0.742	TRUE
Dodecenedioate (C12:1-DC)	Hypertension	0.061	34	0.334	0.504	0.734	TRUE
Trans-2-hexenoylglycine	Hypertension	0.062	37	0.043	0.838	0.978	TRUE
2-hydroxyarachidate	Hypertension	0.019	23	8.81e-05	0.208	0.065	TRUE
3-hydroxyphenylacetoylglutamine	Hypertension	0.014	23	0.746	0.837	0.048	TRUE
Heptenedioate (C7:1-DC)	Hypertension	0.021	22	0.526	0.365	0.144	TRUE
Hexadecenedioate (C16:1-DC)	Hypertension	0.182	86	0.327	0.561	0.690	TRUE
Octadecenedioate (C18:1-DC)	Hypertension	0.124	77	0.239	0.167	0.420	TRUE

Exposure	Outcome	R2	F	Heterogeneity	Pleiotropy	power	Direction
Dihydroferulic acid sulfate	Hypertension	0.054	24	0.021	0.692	0.047	TRUE
Glutamine conjugate of C7H12O2	Hypertension	0.039	25	0.252	0.352	0.055	TRUE
Hydroxyasparagine	Hypertension	0.037	24	1.38e-08	0.967	0.697	TRUE
Sphingomyelin (d17:1/14:0, d16:1/15:0)	Hypertension	0.058	29	0.014	0.588	0.223	TRUE
Octadecadienedioate (C18:2-DC)	Hypertension	0.158	86	0.525	0.262	0.553	TRUE
Perfluorooctanoate (PFOA)	Hypertension	0.032	24	0.005	0.356	0.029	TRUE
3-carboxy-4-methyl-5-pentyl-2-furanpropionate (3-CMPFP)	Hypertension	0.066	37	0.350	0.092	0.167	TRUE
Glutamine conjugate of C6H10O2 (2)	Hypertension	0.019	23	0.029	0.808	0.681	TRUE
3-hydroxybutyroylglycine	Hypertension	0.107	54	3.26e-04	0.331	0.828	TRUE
Glycine conjugate of C10H14O2 (1)	Hypertension	0.058	44	6.43e-04	0.751	0.127	TRUE
Glutamine conjugate of C6H10O2 (1)	Hypertension	0.022	23	0.002	0.370	0.996	TRUE
N-methylhydroxyproline	Hypertension	0.023	24	0.413	0.178	0.027	TRUE
Glucuronide of C10H18O2 (7)	Hypertension	0.035	24	0.465	0.256	0.175	TRUE
Glyco-beta-muricholate	Hypertension	0.094	53	0.096	0.632	0.444	TRUE
Glucuronide of C12H22O4 (1)	Hypertension	0.023	24	0.698	0.537	0.338	TRUE
N,n,n-trimethyl-alanylproline betaine (tmap)	Hypertension	0.037	24	1.24e-04	0.537	0.177	TRUE
Glycine conjugate of C10H12O2	Hypertension	0.040	26	1.44e-07	0.574	0.026	TRUE

Exposure	Outcome	R2	F	Heterogeneity	Pleiotropy	power	Direction
N-acetyl-2-aminooctanoate	Hypertension	0.147	88	3.47e-07	0.478	1.000	TRUE
3-formylindole	Hypertension	0.161	83	0.038	0.393	0.336	TRUE
Gamma-glutamylcitrulline	Hypertension	0.059	26	0.011	0.962	0.792	TRUE
8-methoxykynurenate	Hypertension	0.036	25	0.010	0.014	0.028	TRUE
Tetradecadienoate (14:2)	Hypertension	0.039	27	0.421	0.231	0.479	TRUE
3-amino-2-piperidone	Hypertension	0.036	27	0.230	0.248	0.046	TRUE
Glucuronide of piperine metabolite C17H21NO3 (3)	Hypertension	0.044	28	0.327	0.451	0.031	TRUE
Dodecadienoate (12:2)	Hypertension	0.074	41	4.98e-05	0.035	0.065	TRUE
N-lactoyl tyrosine	Hypertension	0.032	22	0.136	0.592	0.044	TRUE
N,N-dimethylalanine	Hypertension	0.062	30	0.024	0.229	0.046	TRUE
Ethyl beta-glucopyranoside	Hypertension	0.096	69	0.275	0.768	0.065	TRUE
N-lactoyl phenylalanine	Hypertension	0.022	22	0.009	0.432	0.050	TRUE
N-acetyl-isoputreanine	Hypertension	0.161	65	0.002	0.383	0.801	TRUE
2,2'-Methylenebis(6-tert-butyl-p-cresol)	Hypertension	0.031	24	0.556	0.776	0.057	TRUE
N-lactoyl isoleucine	Hypertension	0.021	22	0.295	0.304	0.584	TRUE
2-hydroxysebacate	Hypertension	0.046	35	0.126	0.797	0.070	TRUE
Ascorbic acid 3-sulfate	Hypertension	0.041	24	0.290	0.657	0.092	TRUE

Exposure	Outcome	R2	F	Heterogeneity	Pleiotropy	power	Direction
N-lactoyl valine	Hypertension	0.023	22	0.182	0.064	0.774	TRUE
6-bromotryptophan	Hypertension	0.115	61	0.090	0.456	0.111	TRUE
Delta-CEHC	Hypertension	0.029	24	0.002	0.387	0.072	TRUE
Indoleacetoylcarnitine	Hypertension	0.137	43	0.027	0.447	0.066	TRUE
N-acetyl leucine	Hypertension	0.027	22	0.124	0.308	0.838	TRUE
3-indoleglyoxylic acid	Hypertension	0.097	69	0.038	0.493	0.253	TRUE
Sulfate of piperine metabolite C18H21NO3 (1)	Hypertension	0.037	24	5.94e-15	0.164	1.000	TRUE
Methyl vanillate sulfate	Hypertension	0.027	23	0.106	0.955	0.104	TRUE
Glucuronide of piperine metabolite C17H21NO3 (4)	Hypertension	0.045	34	0.210	0.419	0.094	TRUE
Sulfate of piperine metabolite C16H19NO3 (3)	Hypertension	0.049	24	0.052	0.453	0.471	TRUE
Sulfate of piperine metabolite C16H19NO3 (2)	Hypertension	0.043	23	8.88e-05	0.272	0.356	TRUE
3-hydroxypyridine glucuronide	Hypertension	0.035	24	0.372	0.530	0.068	TRUE
Glucuronide of piperine metabolite C17H21NO3 (5)	Hypertension	0.039	30	0.009	0.988	0.286	TRUE
2-naphthol sulfate	Hypertension	0.034	23	0.751	0.698	0.032	TRUE
11beta-hydroxyandrosterone glucuronide	Hypertension	0.094	36	0.026	0.436	0.787	TRUE
4-ethylcatechol sulfate	Hypertension	0.031	23	0.677	0.068	0.030	TRUE
Sulfate of piperine metabolite C18H21NO3 (3)	Hypertension	0.039	25	0.018	0.967	0.030	TRUE

Exposure	Outcome	R2	F	Heterogeneity	Pleiotropy	power	Direction
(2,4 or 2,5)-dimethylphenol sulfate	Hypertension	0.028	23	0.743	0.845	0.283	TRUE
11beta-hydroxyetiocholanolone glucuronide	Hypertension	0.038	24	0.008	0.706	0.184	TRUE
Cholic acid glucuronide	Hypertension	0.045	34	2.52e-05	0.872	0.097	TRUE
4-allylcatechol sulfate	Hypertension	0.030	23	0.191	0.372	0.641	TRUE
Glycoursodeoxycholic acid sulfate (1)	Hypertension	0.047	28	0.288	0.970	0.571	TRUE
Dihydrocaffeate sulfate (2)	Hypertension	0.032	22	1.11e-04	0.909	0.483	TRUE
4-methylhexanoylglutamine	Hypertension	0.028	39	0.380	0.300	0.314	TRUE
2-ketocaprylate	Hypertension	0.089	40	1.48e-04	0.013	0.027	TRUE
3-hydroxy-2-methylpyridine sulfate	Hypertension	0.035	22	0.316	0.048	0.071	TRUE
5-hydroxy-2-methylpyridine sulfate	Hypertension	0.043	24	0.332	0.373	0.038	TRUE
3-ethylcatechol sulfate (2)	Hypertension	0.027	23	0.002	0.680	0.110	TRUE
3-hydroxyhexanoylcarnitine (1)	Hypertension	0.047	32	0.032	0.117	0.380	TRUE
2,6-dihydroxybenzoic acid	Hypertension	0.049	31	0.004	0.769	0.926	TRUE
3-ethylcatechol sulfate (1)	Hypertension	0.030	23	0.075	0.049	0.073	TRUE
Lithocholate sulfate (1)	Hypertension	0.077	34	0.177	0.102	0.626	TRUE
4-acetylcatechol sulfate (1)	Hypertension	0.016	22	0.017	0.977	0.039	TRUE
2,3-dihydroxy-5-methylthio-4-pentenoate (dmtpa)	Hypertension	0.056	25	1.1e-12	0.997	0.264	TRUE

Exposure	Outcome	R2	F	Heterogeneity	Pleiotropy	power	Direction
Picolinoylglycine	Hypertension	0.040	26	0.005	0.480	0.823	TRUE
Succinoyltaurine	Hypertension	0.150	76	0.861	0.271	0.187	TRUE
Undecenoylcarnitine (C11:1)	Hypertension	0.104	52	0.178	0.822	0.465	TRUE
Palmitoyl-sphingosine-phosphoethanolamine (d18:1/16:0)	Hypertension	0.045	25	0.009	0.038	0.325	TRUE
Pregnenetriol disulfate	Hypertension	0.156	77	0.099	0.308	0.117	TRUE
Tetradecadienedioate (C14:2-DC)	Hypertension	0.112	43	0.047	0.954	0.675	TRUE
Hydroxy-N6,N6,N6-trimethyllysine	Hypertension	0.041	23	1.04e-05	0.100	0.514	TRUE
Pregnenetriol sulfate	Hypertension	0.102	34	1.58e-09	0.074	0.276	TRUE
Taurochenodeoxycholic acid 3-sulfate	Hypertension	0.075	31	3.77e-07	0.143	0.175	TRUE
Hydroxypalmitoyl sphingomyelin (d18:1/16:0(OH))	Hypertension	0.094	40	5.87e-10	0.448	0.063	TRUE
5-hydroxymethyl-2-furoylcarnitine	Hypertension	0.030	23	0.003	0.985	0.225	TRUE
Eicosenedioate (C20:1-DC)	Hypertension	0.078	47	0.492	0.082	0.315	TRUE
3-hydroxydecanoylcarnitine	Hypertension	0.052	23	0.003	0.137	0.034	TRUE
Levulinoylcarnitine	Hypertension	0.054	35	0.157	0.280	0.052	TRUE
Cis 3,4-methyleneheptanoate	Hypertension	0.053	25	0.001	0.630	0.715	TRUE
Branched-chain, straight-chain, or cyclopropyl 12:1 fatty acid	Hypertension	0.057	30	3.56e-05	0.580	0.137	TRUE
Deoxycholic acid 12-sulfate	Hypertension	0.095	62	0.031	0.400	0.999	TRUE

Exposure	Outcome	R2	F	Heterogeneity	Pleiotropy	power	Direction
Branched-chain, straight-chain, or cyclopropyl 10:1 fatty acid (1)	Hypertension	0.024	22	0.051	0.341	0.136	TRUE
3,5-dichloro-2,6-dihydroxybenzoic acid	Hypertension	0.056	43	0.019	0.005	0.053	TRUE
Vanillic acid glycine	Hypertension	0.051	28	3.23e-04	0.138	0.025	TRUE
(S)-a-amino-omega-caprolactam	Hypertension	0.027	28	0.004	0.253	1.000	TRUE
3-bromo-5-chloro-2,6-dihydroxybenzoic acid	Hypertension	0.054	41	0.044	0.962	0.405	TRUE
Dibutyl sulfosuccinate	Hypertension	0.063	23	0.008	0.807	0.057	TRUE
Metabolonic lactone sulfate	Hypertension	0.251	123	1.41e-05	0.662	0.047	TRUE
2-hydroxy-4-(methylthio)butanoic acid	Hypertension	0.166	82	0.001	0.154	0.655	TRUE
Pentose acid	Hypertension	0.040	24	0.103	0.742	0.684	TRUE
Branched chain 14:0 dicarboxylic acid	Hypertension	0.025	23	0.099	0.271	0.723	TRUE
S-carboxyethylcysteine	Hypertension	0.042	25	0.147	0.916	0.363	TRUE
N-succinyl-phenylalanine	Hypertension	0.089	69	0.556	0.823	0.520	TRUE
(2 or 3)-decenoate (10:1n7 or n8)	Hypertension	0.063	29	0.002	0.743	0.967	TRUE
Bilirubin degradation product, C17H20N2O5 (2)	Hypertension	0.103	69	0.366	0.925	0.295	TRUE
Bilirubin degradation product, C16H18N2O5 (4)	Hypertension	0.130	118	0.281	0.371	0.039	TRUE
Cis-3,4-methyleneheptanoylcarnitine	Hypertension	0.079	37	3.61e-05	0.847	0.301	TRUE
Tetrahydrocortisol glucuronide	Hypertension	0.064	30	9.47e-08	0.290	0.939	TRUE

Exposure	Outcome	R2	F	Heterogeneity	Pleiotropy	power	Direction
N-acetyl-2-aminoadipate	Hypertension	0.091	52	5.48e-04	0.091	0.911	TRUE
Bilirubin degradation product, C17H20N2O5 (1)	Hypertension	0.106	62	0.481	0.342	0.043	TRUE
2-methoxyhydroquinone sulfate (1)	Hypertension	0.022	23	0.837	0.537	0.218	TRUE
Bilirubin degradation product, C16H18N2O5 (3)	Hypertension	0.160	86	0.121	0.759	0.069	TRUE
3-hydroxyoctanoylcarnitine (1)	Hypertension	0.035	24	0.057	0.552	0.469	TRUE
2-methoxyhydroquinone sulfate (2)	Hypertension	0.022	23	0.410	0.257	0.116	TRUE
Cis-3,4-methyleneheptanoylglycine	Hypertension	0.107	77	0.261	0.195	0.974	TRUE
Bilirubin degradation product, C16H18N2O5 (2)	Hypertension	0.141	85	0.046	0.842	0.146	TRUE
2,4-di-tert-butylphenol	Hypertension	0.036	23	0.508	0.716	0.115	TRUE
Bilirubin degradation product, C16H18N2O5 (1)	Hypertension	0.143	100	2.92e-06	0.142	0.062	TRUE
3-hydroxyoctanoylcarnitine (2)	Hypertension	0.036	25	0.105	0.941	0.147	TRUE
Pantothenate	Hypertension	0.051	24	1.47e-07	0.086	0.026	TRUE
5-oxoproline	Hypertension	0.133	100	0.040	0.620	0.133	TRUE
Phosphoethanolamine	Hypertension	0.045	25	0.013	0.976	0.265	TRUE
Picolinate	Hypertension	0.070	34	5.26e-04	0.665	0.896	TRUE
3-ureidopropionate	Hypertension	0.034	26	0.257	0.206	0.459	TRUE
Erucate (22:1n9)	Hypertension	0.031	24	0.003	0.843	0.936	TRUE

Exposure	Outcome	R2	F	Heterogeneity	Pleiotropy	power	Direction
Eicosenedioate (C20:1-DC)	Hypertension	0.027	23	0.308	0.961	0.100	TRUE
Allantoin	Hypertension	0.036	23	0.403	0.935	0.087	TRUE
N-acetylvaline	Hypertension	0.044	23	5.8e-06	0.178	0.628	TRUE
N-acetylleucine	Hypertension	0.162	99	0.262	0.782	0.434	TRUE
N-acetylmethionine	Hypertension	0.037	26	0.004	0.603	0.274	TRUE
Glycerate	Hypertension	0.048	24	1.43e-07	0.645	0.206	TRUE
Sarcosine	Hypertension	0.041	23	0.001	0.993	0.216	TRUE
Trans-4-hydroxyproline	Hypertension	0.032	27	0.725	0.873	0.262	TRUE
Thyroxine	Hypertension	0.038	24	0.003	0.875	0.609	TRUE
Pipecolate	Hypertension	0.042	24	0.644	0.873	0.037	TRUE
Gamma-glutamyltyrosine	Hypertension	0.049	29	0.530	0.379	0.055	TRUE
Methyl indole-3-acetate	Hypertension	0.022	23	0.525	0.122	0.197	TRUE
Ursodeoxycholate	Hypertension	0.038	24	0.369	0.354	0.218	TRUE
3-hydroxy-3-methylglutarate	Hypertension	0.029	22	0.973	0.707	0.383	TRUE
Oleoyl ethanolamide	Hypertension	0.057	37	0.231	0.374	0.041	TRUE
4-hydroxyphenylpyruvate	Hypertension	0.033	25	0.209	0.867	0.273	TRUE
Gamma-glutamylglutamine	Hypertension	0.081	29	0.006	0.246	0.939	TRUE

Exposure	Outcome	R2	F	Heterogeneity	Pleiotropy	power	Direction
Alpha-tocopherol	Hypertension	0.046	24	2.51e-05	0.801	0.351	TRUE
N-acetyl-L-alanine	Hypertension	0.051	25	6.58e-09	0.774	0.035	TRUE
Citrate	Hypertension	0.054	35	0.024	0.999	0.035	TRUE
2-aminobutyrate	Hypertension	0.040	37	0.380	0.763	0.080	TRUE
Butyrate/isobutyrate (4:0)	Hypertension	0.033	25	0.044	0.636	0.351	TRUE
Urate	Hypertension	0.094	40	0.220	0.715	0.236	TRUE
3-hydroxyisobutyrate	Hypertension	0.036	23	0.109	0.236	0.793	TRUE
Vanillylmandelate (VMA)	Hypertension	0.088	32	1.29e-11	0.456	0.908	TRUE
3-aminoisobutyrate	Hypertension	0.230	163	0.031	0.912	0.571	TRUE
4-acetamidobutanoate	Hypertension	0.069	53	0.516	0.323	0.047	TRUE
2-hydroxystearate	Hypertension	0.041	23	0.008	0.050	0.559	TRUE
N1-methyladenosine	Hypertension	0.058	24	1.51e-08	0.991	0.754	TRUE
N-acetylneuraminate	Hypertension	0.064	27	1.49e-04	0.594	0.781	TRUE
Gamma-glutamylhistidine	Hypertension	0.027	25	0.130	0.741	0.595	TRUE
N-acetylglucosaminylasparagine	Hypertension	0.119	68	0.014	0.587	0.214	TRUE
Homovanillate (hva)	Hypertension	0.042	23	1.11e-06	0.639	0.075	TRUE
Acetoacetate	Hypertension	0.036	23	0.386	0.300	0.060	TRUE

Exposure	Outcome	R2	F	Heterogeneity	Pleiotropy	power	Direction
Cys-gly, oxidized	Hypertension	0.119	70	0.018	0.825	0.967	TRUE
Dihomo-linoleate (20:2n6)	Hypertension	0.027	22	0.003	0.110	0.065	TRUE
Cholate	Hypertension	0.030	23	0.003	0.451	0.110	TRUE
Beta-hydroxyisovalerate	Hypertension	0.074	32	0.205	0.186	0.146	TRUE
Creatine	Hypertension	0.069	34	2.4e-04	0.605	0.207	TRUE
Anthranilate	Hypertension	0.039	23	0.280	0.459	0.216	TRUE
Glycerol	Hypertension	0.027	23	0.633	0.312	0.036	TRUE
Gamma-glutamylleucine	Hypertension	0.016	22	0.080	0.630	0.146	TRUE
Choline	Hypertension	0.045	27	0.012	0.771	0.026	TRUE
3-methoxytyrosine	Hypertension	0.081	42	2.56e-10	0.314	0.084	TRUE
3-phosphoglycerate	Hypertension	0.033	23	0.648	0.214	0.587	TRUE
1-palmitoyl-2-linoleoyl-gpc (16:0/18:2)	Hypertension	0.081	43	0.025	0.108	0.077	TRUE
1-palmitoyl-2-oleoyl-GPE (16:0/18:1)	Hypertension	0.128	52	1.02e-08	0.216	0.975	TRUE
1-palmitoyl-2-linoleoyl-GPI (16:0/18:2)	Hypertension	0.083	37	0.016	0.695	0.031	TRUE
Taurocholic acid	Hypertension	0.042	23	0.083	0.094	0.153	TRUE
Taurodeoxycholate	Hypertension	0.024	23	0.102	0.980	0.340	TRUE
Stearoyl sphingomyelin (d18:1/18:0)	Hypertension	0.100	33	3e-09	0.519	0.066	TRUE

Exposure	Outcome	R2	F	Heterogeneity	Pleiotropy	power	Direction
Ceramide (d18:1/16:0)	Hypertension	0.048	23	4.32e-06	0.585	0.780	TRUE
Taurochenodeoxycholate	Hypertension	0.031	23	0.197	0.623	0.120	TRUE
4-hydroxyphenylacetate	Hypertension	0.037	23	0.029	0.262	0.325	TRUE
5,6-dihydrothymine	Hypertension	0.023	22	0.504	0.283	0.064	TRUE
1-palmitoyl-2-oleoyl-gpc (16:0/18:1)	Hypertension	0.043	24	0.050	0.897	0.098	TRUE
N-stearoyl-sphingosine (d18:1/18:0)	Hypertension	0.060	36	1.23e-07	0.404	0.127	TRUE
Glycochenodeoxycholate	Hypertension	0.042	23	7.87e-05	0.204	0.190	TRUE
N-formylmethionine	Hypertension	0.057	32	0.005	0.634	0.498	TRUE
N6,n6,n6-trimethyllysine	Hypertension	0.043	40	0.883	0.642	0.863	TRUE
S-adenosylhomocysteine (SAH)	Hypertension	0.050	30	9.15e-14	0.254	0.724	TRUE
4-acetaminophen sulfate	Hypertension	0.032	22	0.145	0.915	0.029	TRUE
2-hydroxyhippurate (salicylurate)	Hypertension	0.030	23	0.056	0.850	0.084	TRUE
Quinolinate	Hypertension	0.050	28	4.79e-07	0.938	0.999	TRUE
Eicosapentaenoate (EPA; 20:5n3)	Hypertension	0.046	38	2.14e-04	0.052	0.031	TRUE
Laurate (12:0)	Hypertension	0.024	22	0.250	0.890	0.616	TRUE
N-acetylputrescine	Hypertension	0.178	89	0.007	0.882	0.032	TRUE
Methylsuccinate	Hypertension	0.093	62	0.595	0.705	0.048	TRUE

Exposure	Outcome	R2	F	Heterogeneity	Pleiotropy	power	Direction
9,10-DiHOME	Hypertension	0.036	23	2.14e-05	0.749	0.039	TRUE
Linoleate (18:2n6)	Hypertension	0.033	23	0.433	0.062	0.217	TRUE
Adenosine 5'-diphosphate (ADP)	Hypertension	0.041	23	0.089	0.104	0.176	TRUE
Adenosine 5'-monophosphate (AMP)	Hypertension	0.021	22	8.28e-04	0.884	0.661	TRUE
Adenosine 3',5'-cyclic monophosphate (camp)	Hypertension	0.052	27	0.161	0.854	0.104	TRUE
Arachidonate (20:4n6)	Hypertension	0.079	52	0.640	0.490	0.078	TRUE
5-methylthioadenosine (mta)	Hypertension	0.045	29	1.59e-04	0.284	0.236	TRUE
Cortisone	Hypertension	0.022	23	0.664	0.281	0.436	TRUE
Beta-alanine	Hypertension	0.047	35	3.04e-04	0.424	0.026	TRUE
Succinate	Hypertension	0.034	22	0.073	0.903	0.059	TRUE
Phosphocholine	Hypertension	0.061	27	0.854	0.629	0.042	TRUE
Cysteinylglycine	Hypertension	0.060	46	3.4e-07	0.713	0.344	TRUE
Phenylpyruvate	Hypertension	0.060	28	0.254	0.688	0.804	TRUE
Creatinine	Hypertension	0.033	23	2.26e-05	0.558	0.952	TRUE
3-Hydroxybutyrate	Hypertension	0.025	23	0.072	0.047	0.642	TRUE
Cholesterol	Hypertension	0.028	23	0.045	0.398	0.137	TRUE
2-hydroxyphenylacetate	Hypertension	0.046	32	2.01e-04	0.719	0.042	TRUE

Exposure	Outcome	R2	F	Heterogeneity	Pleiotropy	power	Direction
Aspartate	Hypertension	0.045	31	0.655	0.616	0.277	TRUE
3-(4-hydroxyphenyl)lactate	Hypertension	0.048	31	0.005	0.121	0.619	TRUE
Arginine	Hypertension	0.067	29	3.31e-04	0.257	0.324	TRUE
Gluconate	Hypertension	0.058	37	0.009	0.482	0.034	TRUE
Sphinganine	Hypertension	0.042	25	0.618	0.788	0.335	TRUE
Gamma-glutamylglutamate	Hypertension	0.049	26	3.07e-07	0.806	0.369	TRUE
Flavin adenine dinucleotide (FAD)	Hypertension	0.101	48	0.051	0.125	0.217	TRUE
Histidine	Hypertension	0.041	24	9.46e-10	0.856	0.090	TRUE
Cortisol	Hypertension	0.036	25	5.99e-04	0.056	0.043	TRUE
Hypotaurine	Hypertension	0.076	26	5.24e-06	0.455	0.165	TRUE
Glutarate (C5-DC)	Hypertension	0.042	24	0.863	0.721	0.162	TRUE
S-1-pyrroline-5-carboxylate	Hypertension	0.028	26	5.7e-04	0.039	0.048	TRUE
Cystine	Hypertension	0.021	22	0.324	0.171	0.129	TRUE
Cystathionine	Hypertension	0.062	31	0.435	0.213	0.930	TRUE
Guanidinoacetate	Hypertension	0.065	32	3.46e-09	0.117	1.000	TRUE
Sphingosine	Hypertension	0.026	31	0.613	0.802	0.026	TRUE
Deoxycholate	Hypertension	0.049	24	1.13e-04	0.417	0.185	TRUE

Exposure	Outcome	R2	F	Heterogeneity	Pleiotropy	power	Direction
Glycocholate	Hypertension	0.062	23	0.035	0.914	0.035	TRUE
Palmitoleate (16:1n7)	Hypertension	0.038	24	0.033	0.667	0.123	TRUE
Leucine	Hypertension	0.035	23	0.055	0.541	0.182	TRUE
Stearate (18:0)	Hypertension	0.022	23	4.66e-05	0.059	0.582	TRUE
Methionine	Hypertension	0.037	23	0.064	0.553	0.132	TRUE
Orotate	Hypertension	0.219	99	4.5e-05	0.550	0.052	TRUE
Phosphate	Hypertension	0.045	27	0.120	0.682	0.537	TRUE
Inosine	Hypertension	0.027	22	0.306	0.517	0.254	TRUE
Inosine 5'-monophosphate (IMP)	Hypertension	0.038	23	0.378	0.522	0.112	TRUE
Phenylalanine	Hypertension	0.041	28	0.484	0.452	0.032	TRUE
Palmitate (16:0)	Hypertension	0.034	23	0.004	0.423	0.586	TRUE
Malate	Hypertension	0.025	35	0.012	0.978	0.051	TRUE
Isoleucine	Hypertension	0.030	23	0.825	0.314	0.036	TRUE
Nicotinamide	Hypertension	0.033	23	2.84e-06	0.471	0.720	TRUE
Myo-inositol	Hypertension	0.050	25	3.88e-05	0.131	0.209	TRUE
Lysine	Hypertension	0.073	32	0.333	0.153	0.137	TRUE
Citrulline	Hypertension	0.046	25	0.276	0.356	0.072	TRUE

Exposure	Outcome	R2	F	Heterogeneity	Pleiotropy	power	Direction
Ornithine	Hypertension	0.047	26	0.077	0.977	0.498	TRUE
Retinol (Vitamin A)	Hypertension	0.038	24	0.242	0.538	0.660	TRUE
1-methylnicotinamide	Hypertension	0.039	23	0.004	0.051	0.882	TRUE
Uridine	Hypertension	0.035	36	0.045	0.662	0.900	TRUE
Plasma lactate	Hypertension	0.028	23	0.951	0.967	0.076	TRUE
Pristanate	Hypertension	0.017	23	0.486	0.882	0.119	TRUE
Phytanate	Hypertension	0.037	24	5.45e-05	0.183	0.115	TRUE
Plasma free proline	Hypertension	0.095	38	0.006	0.847	0.141	TRUE
Glutamate	Hypertension	0.036	25	0.819	0.823	0.900	TRUE
2'-deoxyuridine	Hypertension	0.082	43	1.51e-17	0.267	0.938	TRUE
Myristate (14:0)	Hypertension	0.030	23	0.417	0.239	0.095	TRUE
Serine	Hypertension	0.103	37	1.95e-05	0.532	0.496	TRUE
Trans-urocanate	Hypertension	0.074	57	0.003	0.009	0.046	TRUE
Urea	Hypertension	0.036	23	0.866	0.665	0.154	TRUE
Pyridoxal	Hypertension	0.062	23	0.451	0.932	0.293	TRUE
Glutamine	Hypertension	0.064	39	0.001	0.658	0.544	TRUE
Taurine	Hypertension	0.017	24	0.043	0.647	0.027	TRUE

Exposure	Outcome	R2	F	Heterogeneity	Pleiotropy	power	Direction
Salicylate	Hypertension	0.044	23	0.067	0.995	0.841	TRUE
Serotonin	Hypertension	0.040	22	0.005	0.923	0.039	TRUE
Spermidine	Hypertension	0.040	24	0.001	0.053	0.336	TRUE
Dimethylglycine	Hypertension	0.097	56	9.68e-07	0.345	0.137	TRUE
Betaine	Hypertension	0.099	55	0.004	0.988	0.050	TRUE
Xylose	Hypertension	0.029	24	0.009	0.742	0.438	TRUE
Tyrosine	Hypertension	0.050	28	0.178	1.000	0.336	TRUE
Caprate (10:0)	Hypertension	0.026	24	0.028	0.655	0.037	TRUE
Cytidine	Hypertension	0.048	23	0.555	0.792	0.075	TRUE
Pyruvate	Hypertension	0.032	22	0.004	0.318	0.910	TRUE
Alanine	Hypertension	0.027	23	0.050	0.702	0.064	TRUE
Threonine	Hypertension	0.063	29	2.62e-05	0.965	0.246	TRUE
Cytosine	Hypertension	0.021	22	0.231	0.561	0.260	TRUE
12,13-DiHOME	Hypertension	0.046	23	0.398	0.193	0.355	TRUE
Mannose	Hypertension	0.064	45	0.470	0.113	0.988	TRUE
Caffeine	Hypertension	0.034	24	0.002	0.279	0.176	TRUE
Fructose	Hypertension	0.038	23	0.630	0.692	0.073	TRUE

Exposure	Outcome	R2	F	Heterogeneity	Pleiotropy	power	Direction
Alpha-ketobutyrate	Hypertension	0.024	22	1.81e-06	0.384	0.095	TRUE
Cysteine	Hypertension	0.027	22	0.068	0.036	0.106	TRUE
Blood sugar	Hypertension	0.035	24	0.009	0.446	0.047	TRUE
Tryptophan	Hypertension	0.030	25	0.280	0.941	0.257	TRUE
Valine	Hypertension	0.022	23	0.511	0.603	0.900	TRUE
Pseudouridine	Hypertension	0.044	24	1.04e-06	0.596	0.986	TRUE
Dihydroorotate	Hypertension	0.115	46	6.22e-09	0.565	0.622	TRUE
Caprylate (8:0)	Hypertension	0.043	25	0.141	0.481	0.050	TRUE
Arachidate (20:0)	Hypertension	0.022	23	3.56e-05	0.208	0.659	TRUE
Maltose	Hypertension	0.031	23	0.690	0.568	0.363	TRUE
N-stearoyl-sphinganine (d18:0/18:0)	Hypertension	0.019	27	0.136	0.295	0.105	TRUE
Sucrose	Hypertension	0.023	24	0.128	0.542	0.060	TRUE
Caproate (6:0)	Hypertension	0.051	30	0.055	0.266	0.153	TRUE
Nonadecanoate (19:0)	Hypertension	0.038	23	0.800	0.376	0.071	TRUE
Plasma free asparagine	Hypertension	0.140	82	0.001	0.171	0.271	TRUE
X-07765	Hypertension	0.055	29	0.002	0.816	0.777	TRUE
Kynurenate	Hypertension	0.100	45	2.35e-04	0.012	0.950	TRUE

Exposure	Outcome	R2	F	Heterogeneity	Pleiotropy	power	Direction
Alpha-ketoglutarate	Hypertension	0.047	28	0.938	0.627	0.119	TRUE
X-11308	Hypertension	0.074	39	0.017	0.208	0.034	TRUE
Pentadecanoate (15:0)	Hypertension	0.025	23	5e-04	0.889	0.776	TRUE
X-11315	Hypertension	0.077	40	0.004	0.317	0.029	TRUE
Margarate (17:0)	Hypertension	0.030	25	4.92e-04	0.863	1.000	TRUE
X-11372	Hypertension	0.040	28	0.086	0.844	0.729	TRUE
X-10458	Hypertension	0.055	31	0.573	0.924	0.055	TRUE
X-11299	Hypertension	0.037	25	0.001	0.576	0.588	TRUE
X-11478	Hypertension	0.059	31	0.325	0.292	0.125	TRUE
X-11849	Hypertension	0.048	25	0.004	0.500	0.026	TRUE
X-11787	Hypertension	0.141	112	0.004	0.307	0.109	TRUE
X-11632	Hypertension	0.041	24	0.777	0.507	0.774	TRUE
X-11852	Hypertension	0.036	23	0.150	0.676	0.331	TRUE
X-11847	Hypertension	0.033	23	0.024	0.906	0.042	TRUE
X-11483	Hypertension	0.039	23	0.021	0.954	0.066	TRUE
X-11470	Hypertension	0.131	48	0.508	0.287	0.770	TRUE
X-11381	Hypertension	0.083	38	0.136	0.026	0.167	TRUE

Exposure	Outcome	R2	F	Heterogeneity	Pleiotropy	power	Direction
X-11850	Hypertension	0.032	22	0.032	0.119	0.051	TRUE
X-11795	Hypertension	0.036	34	0.001	0.742	0.329	TRUE
X-11444	Hypertension	0.126	51	0.051	0.520	0.064	TRUE
X-11858	Hypertension	0.031	23	2.66e-04	0.760	0.036	TRUE
X-11843	Hypertension	0.039	23	0.155	0.848	0.064	TRUE
X-12127	Hypertension	0.041	24	0.041	0.532	0.824	TRUE
X-12221	Hypertension	0.019	23	0.133	0.651	0.655	TRUE
X-12283	Hypertension	0.036	23	0.043	0.532	0.827	TRUE
X-12410	Hypertension	0.061	39	0.160	0.139	0.137	TRUE
X-12104	Hypertension	0.020	23	0.003	0.503	0.773	TRUE
X-12193	Hypertension	0.047	23	0.639	0.204	0.042	TRUE
X-12216	Hypertension	0.044	29	5.2e-06	0.264	0.592	TRUE
X-12306	Hypertension	0.036	23	1.15e-04	0.118	0.479	TRUE
X-12100	Hypertension	0.052	36	9.59e-25	0.127	1.000	TRUE
X-12261	Hypertension	0.019	22	0.008	0.468	0.029	TRUE
X-11880	Hypertension	0.059	29	0.292	0.854	0.777	TRUE
X-12117	Hypertension	0.120	41	0.002	0.110	0.635	TRUE

Exposure	Outcome	R2	F	Heterogeneity	Pleiotropy	power	Direction
X-12407	Hypertension	0.019	22	0.766	0.377	0.547	TRUE
X-12101	Hypertension	0.049	32	1.27e-04	0.006	0.056	TRUE
X-12026	Hypertension	0.055	24	0.009	0.099	0.040	TRUE
X-12411	Hypertension	0.042	39	0.128	0.802	0.199	TRUE
X-12013	Hypertension	0.035	24	0.085	0.168	0.046	TRUE
X-12007	Hypertension	0.025	24	0.501	0.859	0.120	TRUE
X-12462	Hypertension	0.019	22	0.478	0.291	0.038	TRUE
X-12714	Hypertension	0.043	40	4.32e-04	0.329	0.055	TRUE
X-12740	Hypertension	0.027	23	0.169	0.252	0.153	TRUE
X-12730	Hypertension	0.030	23	0.009	0.114	0.233	TRUE
X-12701	Hypertension	0.024	25	0.701	0.301	0.035	TRUE
X-12816	Hypertension	0.049	23	0.003	0.221	0.281	TRUE
X-12731	Hypertension	0.049	24	7.55e-04	0.581	0.666	TRUE
X-12707	Hypertension	0.066	34	0.039	0.266	0.378	TRUE
X-12729	Hypertension	0.044	23	0.566	0.489	0.124	TRUE
X-12830	Hypertension	0.044	23	0.008	0.009	0.151	TRUE
X-12818	Hypertension	0.020	23	0.042	0.344	0.059	TRUE

Exposure	Outcome	R2	F	Heterogeneity	Pleiotropy	power	Direction
X-12812	Hypertension	0.027	22	0.238	0.134	0.216	TRUE
X-12822	Hypertension	0.058	55	0.007	0.176	0.991	TRUE
X-12738	Hypertension	0.024	23	0.144	0.021	0.181	TRUE
X-12544	Hypertension	0.035	23	0.259	0.325	0.091	TRUE
X-12680	Hypertension	0.023	24	0.108	0.115	0.502	TRUE
X-12906	Hypertension	0.022	23	0.542	0.483	0.404	TRUE
X-13684	Hypertension	0.110	46	0.076	0.826	0.248	TRUE
X-13507	Hypertension	0.014	23	0.441	0.857	0.051	TRUE
X-12851	Hypertension	0.032	26	0.003	0.554	0.154	TRUE
X-12849	Hypertension	0.037	22	0.106	0.009	0.392	TRUE
X-12847	Hypertension	0.030	28	0.079	0.388	0.069	TRUE
X-13553	Hypertension	0.041	25	5.35e-09	0.782	0.926	TRUE
X-12839	Hypertension	0.045	25	0.298	0.363	0.150	TRUE
X-13729	Hypertension	0.055	23	0.135	0.364	0.161	TRUE
X-13007	Hypertension	0.049	23	0.127	0.144	0.050	TRUE
X-13844	Hypertension	0.022	23	0.471	0.428	0.192	TRUE
X-13728	Hypertension	0.031	23	0.151	0.984	0.047	TRUE

Exposure	Outcome	R2	F	Heterogeneity	Pleiotropy	power	Direction
X-12844	Hypertension	0.098	39	1.16e-06	0.794	0.776	TRUE
X-13695	Hypertension	0.025	23	0.989	0.750	0.079	TRUE
X-13723	Hypertension	0.019	23	0.307	0.302	0.259	TRUE
X-14056	Hypertension	0.019	22	0.035	0.437	0.054	TRUE
X-13866	Hypertension	0.044	24	7.27e-05	0.327	0.516	TRUE
X-17146	Hypertension	0.026	24	0.361	0.115	0.627	TRUE
X-16397	Hypertension	0.033	27	0.116	0.329	0.080	TRUE
X-17335	Hypertension	0.020	23	0.027	0.988	0.065	TRUE
X-16964	Hypertension	0.024	22	0.363	0.114	0.076	TRUE
X-16935	Hypertension	0.108	65	0.019	0.941	0.029	TRUE
X-15486	Hypertension	0.017	23	0.048	0.737	0.583	TRUE
X-16580	Hypertension	0.029	24	0.153	0.240	0.438	TRUE
X-17325	Hypertension	0.042	32	0.047	0.933	0.535	TRUE
X-17328	Hypertension	0.032	27	0.709	0.523	0.457	TRUE
X-16087	Hypertension	0.072	56	0.673	0.587	0.210	TRUE
X-17010	Hypertension	0.029	24	0.090	0.336	0.131	TRUE
X-14939	Hypertension	0.046	39	0.368	0.937	0.064	TRUE

Exposure	Outcome	R2	F	Heterogeneity	Pleiotropy	power	Direction
X-15461	Hypertension	0.033	25	0.313	0.352	0.042	TRUE
X-15523	Hypertension	0.165	24	4.31e-04	0.881	0.027	TRUE
X-15728	Hypertension	0.046	23	0.022	0.350	0.054	TRUE
X-15503	Hypertension	0.061	39	1.85e-24	0.163	0.228	TRUE
X-16124	Hypertension	0.053	23	0.004	0.127	0.259	TRUE
X-17438	Hypertension	0.030	23	0.425	0.127	0.106	TRUE
X-17354	Hypertension	0.016	22	0.974	0.603	0.033	TRUE
X-17654	Hypertension	0.128	78	0.364	0.394	0.735	TRUE
X-17676	Hypertension	0.074	29	1.49e-04	0.090	0.093	TRUE
X-17367	Hypertension	0.060	31	0.007	0.484	0.926	TRUE
X-17357	Hypertension	0.086	45	1.61e-05	0.674	0.899	TRUE
X-18886	Hypertension	0.024	25	0.007	0.784	0.112	TRUE
X-18887	Hypertension	0.019	23	0.204	0.257	0.264	TRUE
X-17653	Hypertension	0.079	42	0.164	0.356	0.637	TRUE
X-17346	Hypertension	0.038	22	0.172	0.231	0.069	TRUE
X-17690	Hypertension	0.070	29	3.47e-05	0.026	0.187	TRUE
X-18345	Hypertension	0.042	23	0.258	0.816	0.526	TRUE

Exposure	Outcome	R2	F	Heterogeneity	Pleiotropy	power	Direction
X-17685	Hypertension	0.038	24	0.121	0.330	0.074	TRUE
X-18888	Hypertension	0.042	27	0.132	0.742	0.168	TRUE
X-18901	Hypertension	0.042	24	0.025	0.102	0.752	TRUE
X-17351	Hypertension	0.034	24	0.044	0.165	0.445	TRUE
X-18779	Hypertension	0.014	23	0.489	0.474	0.736	TRUE
X-21286	Hypertension	0.062	29	2.94e-04	0.211	0.030	TRUE
X-21355	Hypertension	0.017	23	0.938	0.591	0.265	TRUE
X-21319	Hypertension	0.023	24	0.268	0.813	0.582	TRUE
X-21258	Hypertension	0.054	30	0.379	0.084	0.263	TRUE
X-21364	Hypertension	0.039	27	0.144	0.991	0.745	TRUE
X-21353	Hypertension	0.052	37	0.115	0.663	0.036	TRUE
X-19438	Hypertension	0.037	24	1.22e-05	0.640	0.145	TRUE
X-18913	Hypertension	0.034	29	0.010	0.464	0.034	TRUE
X-18921	Hypertension	0.126	68	0.033	0.929	0.039	TRUE
X-21339	Hypertension	0.040	28	0.069	0.850	0.504	TRUE
X-19299	Hypertension	0.024	23	0.513	0.742	0.224	TRUE
X-18935	Hypertension	0.050	23	0.844	0.545	0.258	TRUE

Exposure	Outcome	R2	F	Heterogeneity	Pleiotropy	power	Direction
X-21310	Hypertension	0.034	24	5.81e-05	0.174	0.098	TRUE
X-21283	Hypertension	0.096	76	1.35e-04	0.413	0.209	TRUE
X-21285	Hypertension	0.057	28	0.021	0.127	0.041	TRUE
X-21312	Hypertension	0.039	27	3.84e-07	0.271	0.379	TRUE
X-18922	Hypertension	0.100	42	0.325	0.136	0.984	TRUE
X-21442	Hypertension	0.026	27	0.011	0.536	0.039	TRUE
X-21796	Hypertension	0.053	32	4.58e-04	0.936	0.191	TRUE
X-21441	Hypertension	0.157	63	0.055	0.194	0.059	TRUE
X-21752	Hypertension	0.030	23	0.037	0.268	0.096	TRUE
X-21807	Hypertension	0.027	23	0.471	0.295	0.544	TRUE
X-21736	Hypertension	0.060	25	0.003	0.269	0.971	TRUE
X-21829	Hypertension	0.063	29	0.065	0.975	0.360	TRUE
X-21471	Hypertension	0.159	71	0.066	0.483	0.826	TRUE
X-21733	Hypertension	0.060	25	0.129	0.828	0.078	TRUE
X-21742	Hypertension	0.020	23	0.156	0.797	0.061	TRUE
X-21821	Hypertension	0.023	24	3.26e-04	0.205	0.388	TRUE
X-21470	Hypertension	0.091	51	0.004	0.099	0.072	TRUE

Exposure	Outcome	R2	F	Heterogeneity	Pleiotropy	power	Direction
X-21383	Hypertension	0.039	23	0.051	0.744	0.904	TRUE
X-21607	Hypertension	0.128	60	7.35e-06	0.911	0.545	TRUE
X-21834	Hypertension	0.040	26	0.822	0.705	0.269	TRUE
X-21467	Hypertension	0.157	58	0.257	0.170	0.746	TRUE
X-22776	Hypertension	0.025	23	0.627	0.972	0.033	TRUE
X-23655	Hypertension	0.038	22	0.190	0.920	0.171	TRUE
X-22834	Hypertension	0.031	23	0.038	0.627	0.408	TRUE
X-22771	Hypertension	0.016	22	1.84e-04	0.164	0.149	TRUE
X-23639	Hypertension	0.041	28	2.33e-06	0.698	0.222	TRUE
X-23648	Hypertension	0.022	23	0.104	0.604	0.305	TRUE
X-23654	Hypertension	0.096	40	0.441	0.052	0.425	TRUE
X-22520	Hypertension	0.033	31	0.008	0.113	0.063	TRUE
X-23641	Hypertension	0.089	34	0.286	0.448	0.083	TRUE
X-23276	Hypertension	0.040	23	0.433	0.069	0.191	TRUE
X-21845	Hypertension	0.038	22	0.001	0.414	0.028	TRUE
X-22509	Hypertension	0.027	22	0.464	0.918	0.051	TRUE
X-23644	Hypertension	0.028	29	7.09e-05	0.192	0.259	TRUE

Exposure	Outcome	R2	F	Heterogeneity	Pleiotropy	power	Direction
X-22162	Hypertension	0.179	74	7.59e-05	0.316	0.181	TRUE
X-23587	Hypertension	0.025	23	0.187	0.391	0.114	TRUE
X-23593	Hypertension	0.032	22	0.027	0.003	0.071	TRUE
X-23636	Hypertension	0.020	23	0.124	0.186	0.050	TRUE
X-23678	Hypertension	0.042	23	0.011	0.950	0.638	TRUE
X-23680	Hypertension	0.079	48	6.61e-09	0.244	0.045	TRUE
X-23780	Hypertension	0.059	33	0.422	0.469	0.474	TRUE
X-23974	Hypertension	0.032	26	0.034	0.977	0.050	TRUE
X-23659	Hypertension	0.072	35	0.103	0.888	0.160	TRUE
X-23665	Hypertension	0.022	23	0.066	0.641	0.051	TRUE
X-23782	Hypertension	0.032	24	0.098	0.880	0.131	TRUE
X-24295	Hypertension	0.036	23	5.12e-05	0.211	0.205	TRUE
X-24243	Hypertension	0.047	30	0.010	0.793	0.271	TRUE
X-23739	Hypertension	0.072	47	1.41e-06	0.031	0.114	TRUE
X-23997	Hypertension	0.031	24	0.349	0.430	0.917	TRUE
X-24241	Hypertension	0.047	33	0.250	0.929	0.205	TRUE
X-24328	Hypertension	0.080	29	0.785	0.354	0.037	TRUE

Exposure	Outcome	R2	F	Heterogeneity	Pleiotropy	power	Direction
X-24456	Hypertension	0.047	23	0.191	0.193	0.334	TRUE
X-24418	Hypertension	0.120	60	0.004	0.440	0.824	TRUE
X-24344	Hypertension	0.025	23	0.309	0.135	0.050	TRUE
X-24556	Hypertension	0.074	39	0.530	0.892	0.233	TRUE
X-24337	Hypertension	0.082	43	0.009	0.682	0.494	TRUE
X-24546	Hypertension	0.098	44	0.137	0.422	0.925	TRUE
X-24306	Hypertension	0.041	23	0.097	0.966	0.313	TRUE
X-24307	Hypertension	0.025	23	0.068	0.056	0.072	TRUE
X-24334	Hypertension	0.046	29	1.47e-04	0.318	0.039	TRUE
X-24518	Hypertension	0.141	81	0.009	0.216	0.055	TRUE
X-24494	Hypertension	0.060	42	0.341	0.961	0.085	TRUE
X-24544	Hypertension	0.175	84	7.23e-09	0.845	0.051	TRUE
X-24531	Hypertension	0.049	32	0.410	0.203	0.391	TRUE
X-24811	Hypertension	0.041	23	0.094	0.942	0.141	TRUE
X-24757	Hypertension	0.035	24	6.07e-04	0.358	0.275	TRUE
X-24978	Hypertension	0.036	25	0.022	0.143	0.033	TRUE
X-24970	Hypertension	0.037	22	0.064	0.840	0.244	TRUE

Exposure	Outcome	R2	F	Heterogeneity	Pleiotropy	power	Direction
X-24795	Hypertension	0.046	25	3.21e-07	0.240	0.037	TRUE
X-24951	Hypertension	0.031	23	0.030	0.978	0.686	TRUE
X-24728	Hypertension	0.044	23	0.025	0.174	0.401	TRUE
X-24565	Hypertension	0.057	24	0.237	0.536	0.039	TRUE
X-24801	Hypertension	0.105	33	0.004	0.548	0.442	TRUE
X-24736	Hypertension	0.096	39	0.029	0.744	0.582	TRUE
X-24585	Hypertension	0.030	25	0.134	0.252	0.026	TRUE
X-24588	Hypertension	0.136	48	0.160	0.953	0.588	TRUE
X-25109	Hypertension	0.053	31	0.521	0.667	0.492	TRUE
X-24947	Hypertension	0.201	94	7.27e-06	0.312	0.967	TRUE
X-24949	Hypertension	0.097	43	0.016	0.933	0.174	TRUE
X-24812	Hypertension	0.046	24	0.015	0.193	0.290	TRUE
X-25217	Hypertension	0.027	23	0.257	0.967	0.196	TRUE
X-25828	Hypertension	0.013	22	0.019	0.373	0.501	TRUE
X-25371	Hypertension	0.095	42	0.002	0.090	0.851	TRUE
X-25419	Hypertension	0.049	26	0.475	0.823	0.223	TRUE
X-25265	Hypertension	0.044	23	1.03e-04	0.163	0.417	TRUE

Exposure	Outcome	R2	F	Heterogeneity	Pleiotropy	power	Direction
X-25172	Hypertension	0.029	22	0.144	0.184	0.180	TRUE
X-25957	Hypertension	0.025	24	0.684	0.283	0.286	TRUE
X-25422	Hypertension	0.092	48	9.54e-05	0.213	0.926	TRUE
X-25790	Hypertension	0.035	26	0.096	0.764	0.121	TRUE
X-25420	Hypertension	0.037	31	0.422	0.277	0.293	TRUE
X-25810	Hypertension	0.069	25	0.001	0.740	0.715	TRUE
X-25433	Hypertension	0.075	39	2.89e-05	0.910	0.674	TRUE
X-25343	Hypertension	0.024	23	0.058	0.138	0.762	TRUE
X-25271	Hypertension	0.034	24	0.027	0.280	0.407	TRUE
X-25520	Hypertension	0.021	22	0.041	0.333	0.528	TRUE
X-25519	Hypertension	0.028	24	0.024	0.032	0.270	TRUE
X-26111	Hypertension	0.067	40	0.042	0.418	0.578	TRUE
X-26109	Hypertension	0.183	89	0.008	0.019	0.107	TRUE
X-26054	Hypertension	0.162	61	0.124	0.007	0.046	TRUE
Carnitine C4	Hypertension	0.123	40	2.43e-04	0.590	0.116	TRUE
Androsterone sulfate	Hypertension	0.238	90	4.09e-15	0.251	0.209	TRUE
N-acetyltyrosine	Hypertension	0.262	134	0.933	0.152	0.430	TRUE

Exposure	Outcome	R2	F	Heterogeneity	Pleiotropy	power	Direction
N-acetyl-L-glutamine	Hypertension	0.348	308	0.297	0.584	0.961	TRUE
N-acetylphenylalanine	Hypertension	0.215	156	0.251	0.656	0.979	TRUE
N-acetylasparagine	Hypertension	0.057	40	0.007	0.883	0.966	TRUE
N-acetylarginine	Hypertension	0.405	359	0.026	0.402	0.979	TRUE
5-acetylamino-6-formylamino-3-methyluracil	Hypertension	0.278	166	0.502	0.550	0.042	TRUE
Orotidine	Hypertension	0.199	93	2.16e-09	0.593	0.079	TRUE
3-methylcytidine	Hypertension	0.282	275	0.309	0.691	0.026	TRUE
N-acetylcitrulline	Hypertension	0.369	369	0.001	0.624	0.772	TRUE
N-acetyl-aspartyl-glutamate (naag)	Hypertension	0.117	152	0.039	0.032	0.481	TRUE
1-stearoyl-2-arachidonoyl-gpc (18:0/20:4)	Hypertension	0.213	104	3.46e-09	0.746	0.195	TRUE
Bilirubin (E,E)	Hypertension	0.169	103	9.28e-05	0.091	0.026	TRUE
Bilirubin (E,Z or Z,E)	Hypertension	0.154	106	0.015	0.295	0.294	TRUE
Androsterone glucuronide	Hypertension	0.231	109	6.14e-04	0.771	0.044	TRUE
N6-methyllysine	Hypertension	0.705	851	0.045	0.295	0.079	TRUE
N-acetyl-1-methylhistidine	Hypertension	0.282	114	0.001	0.311	0.982	TRUE
N-delta-acetylornithine	Hypertension	0.341	302	0.486	0.107	0.820	TRUE
1-palmitoyl-2-arachidonoyl-gpc (16:0/20:4n6)	Hypertension	0.197	114	3.53e-08	0.446	0.510	TRUE

Exposure	Outcome	R2	F	Heterogeneity	Pleiotropy	power	Direction
Glycochenodeoxycholate glucuronide (1)	Hypertension	0.294	223	0.324	0.312	0.619	TRUE
2'-o-methylcytidine	Hypertension	0.215	194	0.059	0.710	0.035	TRUE
2'-o-methyluridine	Hypertension	0.230	234	0.239	0.739	0.128	TRUE
N6,N6-dimethyllysine	Hypertension	0.528	502	0.344	0.450	0.089	TRUE
N2-acetyl,N6,N6-dimethyllysine	Hypertension	0.513	353	9.86e-06	0.785	0.032	TRUE
N2-acetyl,N6-methyllysine	Hypertension	0.469	252	0.002	0.531	0.151	TRUE
Deoxycholic acid glucuronide	Hypertension	0.233	140	0.049	0.253	0.269	TRUE
Decadienedioic acid (C10:2-DC)	Hypertension	0.206	110	0.008	0.748	0.032	TRUE
Bilirubin degradation product, C17H18N2O4 (1)	Hypertension	0.174	125	0.003	0.010	0.163	TRUE
Bilirubin degradation product, C17H18N2O4 (2)	Hypertension	0.159	120	0.031	0.868	0.060	TRUE
Bilirubin degradation product, C17H18N2O4 (3)	Hypertension	0.154	118	0.007	0.304	0.093	TRUE
Bilirubin (z,z)	Hypertension	0.220	127	0.001	0.337	0.114	TRUE
Ethylmalonate	Hypertension	0.230	88	3.82e-06	0.468	0.536	TRUE
Biliverdin	Hypertension	0.191	105	0.009	0.360	0.132	TRUE
Glycine	Hypertension	0.179	129	0.542	0.606	0.897	TRUE
X-12112	Hypertension	0.526	288	8.86e-04	0.845	0.358	TRUE
X-12798	Hypertension	0.183	86	8.36e-04	0.544	0.357	TRUE

Exposure	Outcome	R2	F	Heterogeneity	Pleiotropy	power	Direction
X-13431	Hypertension	0.071	35	0.052	0.950	0.548	TRUE
X-19141	Hypertension	0.223	124	0.025	0.031	0.364	TRUE
Glutamine	Hypertension	0.030	31	0.004	0.196	1	TRUE
Tryptophan	Hypertension	0.381	26	5.17e-11	0.386	1	TRUE
Histidine	Hypertension	0.024	29	0.038	0.109	1	TRUE
Leucine	Hypertension	0.417	26	1.22e-10	0.640	1	TRUE
Cholesterol	Hypertension	0.098	24	2.02e-11	0.560	1	TRUE
Phenylalanine	Hypertension	0.015	28	0.191	0.802	0.458	TRUE
Creatinine	Hypertension	0.047	25	3.1e-05	0.282	1	TRUE
Lactate	Hypertension	0.016	24	0.054	0.941	1	TRUE
3-hydroxybutyrate (BHBA)	Hypertension	0.016	23	0.704	0.325	0.638	TRUE
Arabinose	Hypertension	0.016	23	0.045	0.119	0.877	TRUE
Fructose	Hypertension	0.006	24	0.438		0.245	TRUE
Mannose	Hypertension	0.065	61	3.09e-04	0.012	1	TRUE
Pyruvate	Hypertension	0.034	22	1.72e-04	0.611	0.937	TRUE
Uridine	Hypertension	0.056	28	0.410	0.084	0.055	TRUE
Linoleate (18:2n6)	Hypertension	0.029	24	0.092	0.353	1.000	TRUE

Exposure	Outcome	R2	F	Heterogeneity	Pleiotropy	power	Direction
Allantoin	Hypertension	0.039	23	0.274	0.649	0.965	TRUE
Arachidonate (20:4n6)	Hypertension	0.081	56	0.334	0.847	1	TRUE
Deoxycholate	Hypertension	0.028	23	0.171	0.328	0.911	TRUE
Margarate (17:0)	Hypertension	0.024	29	0.002	0.396	0.999	TRUE
Inosine	Hypertension	0.053	28	0.369	0.258	0.849	TRUE
Isoleucine	Hypertension	0.034	23	0.384	0.255	1	TRUE
Threonine	Hypertension	0.051	22	2.42e-04	0.884	0.858	TRUE
Tyrosine	Hypertension	0.073	26	0.050	0.694	1	TRUE
Lysine	Hypertension	0.053	26	0.081	0.039	1	TRUE
Methionine	Hypertension	0.040	22	0.008	0.702	1	TRUE
Malate	Hypertension	0.036	25	0.805	0.767	1	TRUE
Palmitate (16:0)	Hypertension	0.061	24	0.009	0.225	1.000	TRUE
Nonadecanoate (19:0)	Hypertension	0.023	24	0.230	0.162	1	TRUE
Stearate (18:0)	Hypertension	0.090	26	0.017	0.863	1	TRUE
Oleate (18:1n9)	Hypertension	0.036	24	0.021	0.338	1	TRUE
Pentadecanoate (15:0)	Hypertension	0.033	23	8.58e-04	0.678	1	TRUE
Myristate (14:0)	Hypertension	0.037	23	0.008	0.386	0.547	TRUE

Exposure	Outcome	R2	F	Heterogeneity	Pleiotropy	power	Direction
Pipecolate	Hypertension	0.025	23	0.014	0.164	0.998	TRUE
Ornithine	Hypertension	0.019	24	0.042	0.434	0.401	TRUE
5-oxoproline	Hypertension	0.109	52	3.75e-05	0.907	1	TRUE
Pantothenate	Hypertension	0.050	24	6.32e-04	0.996	0.231	TRUE
4-acetamidobutanoate	Hypertension	0.093	39	9.55e-05	0.614	1	TRUE
Alpha-tocopherol	Hypertension	0.016	23	0.890	0.677	0.260	TRUE
Citrate	Hypertension	0.078	27	0.092	0.809	0.110	TRUE
Glycerate	Hypertension	0.028	23	0.127	0.091	0.821	TRUE
Guanosine	Hypertension	0.076	22	0.062	0.201	0.192	TRUE
N-acetylalanine	Hypertension	0.039	24	0.077	0.178	1	TRUE
Urate	Hypertension	0.079	67	0.595	0.142	0.039	TRUE
Ursodeoxycholate	Hypertension	0.018	23	0.005	0.184	0.442	TRUE
Arginine	Hypertension	0.032	23	0.002	0.304	1.000	TRUE
Ascorbate (Vitamin C)	Hypertension	0.111	23	3.82e-05	0.608	0.957	TRUE
Heptanoate (7:0)	Hypertension	0.066	24	0.059	0.549	1.000	TRUE
Laurate (12:0)	Hypertension	0.087	24	1.09e-06	0.563	1	TRUE
Valine	Hypertension	0.022	27	0.007	0.158	1	TRUE

Exposure	Outcome	R2	F	Heterogeneity	Pleiotropy	power	Direction
Urea	Hypertension	0.021	22	0.024	0.199	1.000	TRUE
Cortisol	Hypertension	0.016	24	0.010	0.828	0.981	TRUE
Cortisone	Hypertension	0.081	24	0.010	0.586	0.666	TRUE
Proline	Hypertension	0.093	35	4.26e-04	0.432	0.375	TRUE
Citrulline	Hypertension	0.078	25	0.046	0.640	1	TRUE
Biliverdin	Hypertension	0.151	85	0.002	0.380	0.948	TRUE
Serotonin (5HT)	Hypertension	0.043	23	0.003	0.045	1.000	TRUE
Gamma-glutamylglutamine	Hypertension	0.061	28	2.45e-05	0.231	1	TRUE
Gamma-glutamyltyrosine	Hypertension	0.085	26	5.4e-05	0.731	1.000	TRUE
Hypoxanthine	Hypertension	0.046	27	0.041	0.374	1.000	TRUE
Betaine	Hypertension	0.059	33	0.196	0.552	0.031	TRUE
Xanthine	Hypertension	0.011	24	0.102	0.293	0.977	TRUE
Phosphate	Hypertension	0.007	24	0.474		0.999	TRUE
3-methoxytyrosine	Hypertension	0.037	24	6.31e-08	0.728	1	TRUE
Pelargonate (9:0)	Hypertension	0.064	23	0.060	0.704	1.000	TRUE
Undecanoate (11:0)	Hypertension	0.073	25	0.227	0.304	0.191	TRUE
Beta-hydroxyisovalerate	Hypertension	0.060	24	0.051	0.966	0.999	TRUE

Exposure	Outcome	R2	F	Heterogeneity	Pleiotropy	power	Direction
Taurodeoxycholate	Hypertension	0.116	23	0.562	0.342	1.000	TRUE
X-02973	Hypertension	0.049	23	4.27e-05	0.457	1	TRUE
X-03003	Hypertension	0.009	21	0.157	0.718	1	TRUE
X-03088	Hypertension	0.022	22	0.521	0.868	0.655	TRUE
X-03094	Hypertension	0.069	32	1.18e-08	0.405	1	TRUE
Glycerol	Hypertension	0.039	24	0.003	0.484	0.999	TRUE
Kynurenine	Hypertension	0.103	29	8.39e-27	0.961	1	TRUE
Mannitol	Hypertension	0.024	22	0.435	0.347	0.208	TRUE
Glycerol 3-phosphate (G3P)	Hypertension	0.018	23	0.030	0.555	0.393	TRUE
Acetylphosphate	Hypertension	0.033	24	5.47e-05	0.422	1	TRUE
Carnitine	Hypertension	0.488	29	8.18e-13	0.200	1	TRUE
Choline	Hypertension	0.047	24	0.018	0.300	1.000	TRUE
N-acetylornithine	Hypertension	0.183	151	0.094	0.402	1	TRUE
N1-methyladenosine	Hypertension	0.010	25	0.185	0.434	1	TRUE
3-methyl-2-oxovalerate	Hypertension	0.079	27	1.26e-06	0.873	1	TRUE
3-methylhistidine	Hypertension	0.016	22	0.447	0.904	0.575	TRUE
3-phenylpropionate (hydrocinnamate)	Hypertension	0.020	29	0.071	0.332	0.151	TRUE

Exposure	Outcome	R2	F	Heterogeneity	Pleiotropy	power	Direction
Phenylacetate	Hypertension	0.023	21	0.378	0.332	1	TRUE
Glycerophosphorylcholine (GPC)	Hypertension	0.027	23	0.134	0.923	0.996	TRUE
Aspartate	Hypertension	0.009	23	0.387	0.485	1.000	TRUE
X-04357	Hypertension	0.006	22	0.360		0.090	TRUE
X-04494	Hypertension	0.037	22	0.197	0.566	0.385	TRUE
X-04495	Hypertension	0.023	23	0.432	0.319	0.993	TRUE
X-04498	Hypertension	0.048	23	0.012	0.723	1	TRUE
X-04499--3,4-dihydroxybutyrate	Hypertension	0.046	25	0.004	0.066	0.596	TRUE
X-04500	Hypertension	0.033	23	0.003	0.298	1.000	TRUE
Dihomo-linoleate (20:2n6)	Hypertension	0.009	23	0.102	0.280	1	TRUE
X-18601	Hypertension	0.049	28	4.73e-05	0.802	0.123	TRUE
2-hydroxystearate	Hypertension	0.070	23	7.41e-05	0.545	1.000	TRUE
X-05426	Hypertension	0.027	23	0.029	0.379	0.846	TRUE
Indolelactate	Hypertension	0.039	25	6.31e-05	0.814	1.000	TRUE
Glycylvaline	Hypertension	0.033	23	0.019	0.469	0.035	TRUE
Gamma-glutamylleucine	Hypertension	0.054	23	0.071	0.509	0.498	TRUE
Eicosapentaenoate (EPA; 20:5n3)	Hypertension	0.030	32	0.574	0.516	0.074	TRUE

Exposure	Outcome	R2	F	Heterogeneity	Pleiotropy	power	Direction
Estrone 3-sulfate	Hypertension	0.205	24	0.287	0.325	0.682	TRUE
Glycocholate	Hypertension	0.020	23	0.576	0.608	0.504	TRUE
Glycodeoxycholate	Hypertension	0.077	23	6e-05	0.216	0.102	TRUE
Taurochenodeoxycholate	Hypertension	0.025	23	0.742	0.451	0.031	TRUE
Taurocholate	Hypertension	0.065	23	0.629	0.195	0.243	TRUE
X-05907	Hypertension	0.030	24	0.853	0.947	1	TRUE
Docosahexaenoate (DHA; 22:6n3)	Hypertension	0.003	22			1.000	TRUE
1-stearoylglycerophosphoinositol	Hypertension	0.013	24	0.021	0.319	1.000	TRUE
X-06226	Hypertension	0.048	23	0.906	0.490	1	TRUE
X-06246	Hypertension	0.046	24	3.1e-04	0.584	1	TRUE
X-06267	Hypertension	0.024	23	0.005	0.877	0.956	TRUE
X-06307	Hypertension	0.019	25	0.014	0.171	0.999	TRUE
X-06350	Hypertension	0.033	23	0.386	0.310	1.000	TRUE
X-06351	Hypertension	0.020	23	0.735	0.423	1.000	TRUE
Myo-inositol	Hypertension	0.093	26	3.44e-07	0.038	1	TRUE
Glucose	Hypertension	0.083	24	7.71e-04	0.210	1	TRUE
1,5-anhydroglucitol (1,5-AG)	Hypertension	0.083	36	9.32e-07	0.557	1	TRUE

Exposure	Outcome	R2	F	Heterogeneity	Pleiotropy	power	Direction
2-hydroxybutyrate (AHB)	Hypertension	0.030	25	0.031	0.744	1	TRUE
3-methyl-2-oxobutyrate	Hypertension	0.035	23	0.015	0.085	0.752	TRUE
1,6-anhydroglucose	Hypertension	0.030	26	0.118	0.434	0.195	TRUE
1-palmitoylglycerol (1-monopalmitin)	Hypertension	0.023	23	0.003	0.193	1.000	TRUE
1-oleoylglycerol (1-monoolein)	Hypertension	0.045	24	3.21e-04	0.135	1	TRUE
1-stearoylglycerol (1-monostearin)	Hypertension	0.053	23	0.008	0.086	1	TRUE
X-08402	Hypertension	0.093	58	0.122	0.210	1	TRUE
2-hydroxyisobutyrate	Hypertension	0.055	28	2.82e-04	0.146	1	TRUE
X-08766	Hypertension	0.042	22	1.74e-05	0.030	0.086	TRUE
4-methyl-2-oxopentanoate	Hypertension	0.026	27	0.005	0.288	0.132	TRUE
Phenyllactate (PLA)	Hypertension	0.059	28	0.142	0.613	1	TRUE
Homocitrulline	Hypertension	0.034	34	0.020	0.866	0.028	TRUE
Aspartylphenylalanine	Hypertension	0.034	44	0.392	0.455	0.990	TRUE
Levulinate (4-oxovalerate)	Hypertension	0.090	26	0.007	0.047	1	TRUE
Palmitoylcarnitine	Hypertension	0.012	22	0.052	0.199	1	TRUE
X-08988	Hypertension	0.084	29	0.006	0.091	1.000	TRUE
X-09026	Hypertension	0.037	24	0.031	0.527	1	TRUE

Exposure	Outcome	R2	F	Heterogeneity	Pleiotropy	power	Direction
X-09108	Hypertension	0.021	23	0.015	0.890	1.000	TRUE
Cholate	Hypertension	0.008	22	0.441		0.994	TRUE
X-09706	Hypertension	0.035	23	0.016	0.244	1.000	TRUE
X-10395	Hypertension	0.073	27	0.077	0.144	1	TRUE
X-10429	Hypertension	0.058	25	0.632	0.490	1	TRUE
X-10500	Hypertension	0.075	23	0.024	0.193	0.107	TRUE
X-10506	Hypertension	0.019	23	0.115	0.202	0.591	TRUE
X-10510	Hypertension	0.070	32	1.68e-04	0.682	1	TRUE
1-linoleoylglycerol (1-monolinolein)	Hypertension	0.041	28	0.124	0.154	1.000	TRUE
Indoleacetate	Hypertension	0.041	25	0.042	0.986	0.595	TRUE
Hyodeoxycholate	Hypertension	0.043	24	0.647	0.521	0.644	TRUE
3-indoxyl sulfate	Hypertension	0.006	24	0.259		0.983	TRUE
N-acetylglycine	Hypertension	0.059	58	7.21e-04	0.779	1.000	TRUE
Bilirubin (Z,Z)	Hypertension	0.114	132	0.710	0.806	0.291	TRUE
Creatine	Hypertension	0.026	39	0.029	0.337	1	TRUE
Erythrose	Hypertension	0.010	22	0.840	0.682	0.129	TRUE
Threonate	Hypertension	0.027	22	0.159	0.628	0.683	TRUE

Exposure	Outcome	R2	F	Heterogeneity	Pleiotropy	power	Direction
X-10810	Hypertension	0.042	24	7.09e-04	0.965	0.987	TRUE
Cysteine	Hypertension	0.033	22	0.305	0.267	0.984	TRUE
Pyroglutamylglycine	Hypertension	0.028	22	0.980		1.000	TRUE
N-(2-furoyl)glycine	Hypertension	0.729	23	0.014	0.936	1.000	TRUE
DSGEGDFXAEGGGVR*	Hypertension	0.033	21	0.161	0.689	0.930	TRUE
Pyridoxate	Hypertension	0.018	22	4.3e-04	0.011	1	TRUE
Androsterone sulfate	Hypertension	0.118	60	0.003	0.725	0.156	TRUE
3-carboxy-4-methyl-5-propyl-2-furanpropanoate (CMPF)	Hypertension	0.033	25	0.012	0.022	0.089	TRUE
3-(4-hydroxyphenyl)lactate	Hypertension	0.044	25	1.37e-05	0.084	1.000	TRUE
Acetylcarnitine	Hypertension	0.036	38	0.048	0.898	1.000	TRUE
Serine	Hypertension	0.084	35	0.001	0.851	0.982	TRUE
Trans-4-hydroxyproline	Hypertension	0.006	21	0.043		0.996	TRUE
Glutamate	Hypertension	0.008	28	0.011		0.633	TRUE
Hexanoylcarnitine	Hypertension	0.098	68	0.090	0.970	1	TRUE
Glycine	Hypertension	0.121	79	5.11e-04	0.401	1	TRUE
Alanine	Hypertension	0.078	25	0.117	0.169	1	TRUE
Glycochenodeoxycholate	Hypertension	0.032	24	0.400	0.624	0.188	TRUE

Exposure	Outcome	R2	F	Heterogeneity	Pleiotropy	power	Direction
2-aminobutyrate	Hypertension	0.105	26	1.51e-04	0.571	1.000	TRUE
Scyllo-inositol	Hypertension	0.033	34	2.26e-04	0.909	1.000	TRUE
Dodecanedioate	Hypertension	0.024	24	0.160	0.287	1.000	TRUE
Gamma-glutamylvaline	Hypertension	0.026	24	0.127	0.624	1	TRUE
Indolepropionate	Hypertension	0.038	28	0.522	0.483	0.053	TRUE
Butyrylcarnitine	Hypertension	0.212	106	3.14e-04	0.193	1.000	TRUE
Myristoleate (14:1n5)	Hypertension	0.036	27	0.009	0.308	0.999	TRUE
Dehydroisoandrosterone sulfate (DHEA-S)	Hypertension	0.045	28	7.81e-06	0.611	1.000	TRUE
Propionylcarnitine	Hypertension	0.111	33	0.003	0.138	1	TRUE
Caproate (6:0)	Hypertension	0.053	23	1.64e-04	0.919	1	TRUE
Caprylate (8:0)	Hypertension	0.097	23	0.002	0.050	1.000	TRUE
10-undecenoate (11:1n1)	Hypertension	0.069	47	1.05e-04	0.813	0.993	TRUE
Docosapentaenoate (n3 DPA; 22:5n3)	Hypertension	0.017	32	0.618	0.331	1.000	TRUE
X-11204	Hypertension	0.074	24	1.14e-14	0.242	1	FALSE
X-02269	Hypertension	0.033	30	0.110	0.557	0.991	TRUE
Phenol sulfate	Hypertension	0.027	22	0.331	0.292	0.298	TRUE
X-06126	Hypertension	0.030	22	0.287	0.219	0.134	TRUE

Exposure	Outcome	R2	F	Heterogeneity	Pleiotropy	power	Direction
X-07765	Hypertension	0.095	22	0.066	0.796	0.893	TRUE
X-11247	Hypertension	0.039	25	0.004	0.905	0.265	TRUE
X-11261	Hypertension	0.038	40	0.005	0.824	0.298	TRUE
Bilirubin (E,E)*	Hypertension	0.077	74	0.315	0.380	0.179	TRUE
X-02249	Hypertension	0.028	30	0.723	0.831	1.000	TRUE
Heme*	Hypertension	0.016	24	0.873	0.982	1	TRUE
X-11299	Hypertension	0.019	22	0.090	0.963	0.353	TRUE
X-11315	Hypertension	0.061	26	0.002	0.770	0.968	TRUE
X-11317	Hypertension	0.095	25	1.77e-08	0.380	1.000	TRUE
1-linoleoylglycerophosphoethanolamine*	Hypertension	0.045	42	3.67e-04	0.393	1	TRUE
X-11327	Hypertension	0.082	25	0.008	0.042	1	TRUE
X-11334	Hypertension	0.064	24	6.06e-05	0.307	1.000	TRUE
3-dehydrocarnitine*	Hypertension	0.065	30	2.44e-04	0.024	1.000	TRUE
Pyroglutamine*	Hypertension	0.061	41	0.021	0.382	1	TRUE
C-glycosyltryptophan*	Hypertension	0.045	24	0.004	0.375	1	TRUE
X-11374	Hypertension	0.098	23	0.003	0.685	0.553	TRUE
X-11381	Hypertension	0.036	29	0.717	0.281	1.000	TRUE

Exposure	Outcome	R2	F	Heterogeneity	Pleiotropy	power	Direction
X-03056--N-[3-(2-Oxopyrrolidin-1-yl)propyl]acetamide	Hypertension	0.077	38	7.57e-04	0.454	0.087	TRUE
X-11412	Hypertension	0.094	24	3.28e-05	0.729	1	TRUE
X-01911	Hypertension	0.040	27	0.166	0.795	1	TRUE
X-11422--xanthine	Hypertension	0.012	24	0.705	0.618	1	TRUE
X-11423--O-sulfo-L-tyrosine	Hypertension	0.080	27	0.005	0.937	0.612	TRUE
X-09789	Hypertension	0.027	33	0.001	0.306	1.000	TRUE
X-11437	Hypertension	0.026	24	0.166	0.937	0.201	TRUE
X-11438	Hypertension	0.060	24	2.19e-05	0.375	0.677	TRUE
X-11440	Hypertension	0.113	87	3.72e-09	0.115	0.477	TRUE
X-11441	Hypertension	0.096	85	0.173	0.844	0.042	TRUE
X-11442	Hypertension	0.098	87	0.032	0.638	0.913	TRUE
X-11444	Hypertension	0.031	28	0.034	0.622	1	TRUE
X-11445--5-alpha-pregnan-3beta,20alpha-disulfate	Hypertension	0.097	28	6.39e-04	0.830	0.805	TRUE
X-11452	Hypertension	0.016	25	0.004	0.278	1	TRUE
X-11469	Hypertension	0.035	32	0.088	0.716	0.345	TRUE
X-11470	Hypertension	0.029	29	1.74e-14	0.660	0.130	TRUE
X-11478	Hypertension	0.045	35	0.027	0.525	0.125	TRUE

Exposure	Outcome	R2	F	Heterogeneity	Pleiotropy	power	Direction
X-11483	Hypertension	0.036	26	0.199	0.550	0.808	TRUE
X-11485	Hypertension	0.011	24	0.491		0.068	TRUE
X-11491	Hypertension	0.054	38	1.02e-05	0.085	1	TRUE
X-11497	Hypertension	0.017	23	0.303	0.859	0.909	TRUE
HWESASXX*	Hypertension	0.024	29	0.002	0.309	0.886	TRUE
X-11521	Hypertension	0.023	26	0.202	0.336	0.744	TRUE
X-11529	Hypertension	0.211	318	0.183	0.133	0.997	TRUE
X-11530	Hypertension	0.097	147	0.249	0.759	0.132	TRUE
X-11537	Hypertension	0.036	24	0.253	0.805	0.976	TRUE
X-11538	Hypertension	0.096	81	0.238	0.717	1.000	TRUE
X-11540	Hypertension	0.042	23	0.111	0.959	0.874	TRUE
X-11546	Hypertension	0.113	23	0.794	0.339	0.975	TRUE
X-11550	Hypertension	0.050	24	3.51e-06	0.889	1.000	TRUE
X-11552	Hypertension	0.084	23	0.042	0.777	0.806	TRUE
X-11593--O-methylascorbate*	Hypertension	0.185	60	0.001	0.431	1	TRUE
Adrenate (22:4n6)	Hypertension	0.027	39	0.467	0.211	0.028	TRUE
Homostachydrine*	Hypertension	0.053	22	0.409	0.718	1.000	TRUE

Exposure	Outcome	R2	F	Heterogeneity	Pleiotropy	power	Direction
ADSGEGDFXAEGGGVR*	Hypertension	0.036	33	0.001	0.997	0.766	TRUE
X-11786--methylcysteine	Hypertension	0.078	27	0.050	0.808	1.000	TRUE
X-11787	Hypertension	0.151	77	0.004	0.426	1.000	TRUE
X-11792	Hypertension	0.133	32	0.792	0.485	0.950	TRUE
X-11793--oxidized bilirubin*	Hypertension	0.092	78	0.506	0.396	0.053	TRUE
X-11795	Hypertension	0.022	23	0.021	0.204	0.950	TRUE
X-11799	Hypertension	0.049	60	0.101	0.939	0.998	TRUE
X-11805	Hypertension	0.025	23	0.540	0.515	0.043	TRUE
X-11818	Hypertension	0.048	24	0.147	0.908	0.884	TRUE
X-11820	Hypertension	0.028	29	0.008	0.194	0.198	TRUE
X-11843	Hypertension	0.025	23			0.613	TRUE
X-11845	Hypertension	0.101	22	0.253	0.861	0.983	TRUE
X-11847	Hypertension	0.027	22	0.069	0.829	0.601	TRUE
X-11849	Hypertension	0.031	23	0.314	0.283	0.864	TRUE
X-11850	Hypertension	0.019	22	0.028	0.105	0.962	TRUE
X-11852	Hypertension	0.055	23	1.16e-05	0.916	1.000	TRUE
X-11858	Hypertension	0.048	26	0.294	0.515	0.065	TRUE

Exposure	Outcome	R2	F	Heterogeneity	Pleiotropy	power	Direction
X-11859	Hypertension	0.048	22	0.037	0.816	1	TRUE
X-11876	Hypertension	0.038	22	0.030	0.234	0.928	TRUE
1-arachidonoylglycerophosphocholine*	Hypertension	0.104	54	0.004	0.374	0.060	TRUE
1-palmitoleoylglycerophosphocholine*	Hypertension	0.026	24	0.124	0.154	0.087	TRUE
X-11905	Hypertension	0.103	39	0.243	0.467	0.053	TRUE
X-12007	Hypertension	0.320	23	0.257	0.350	0.992	TRUE
X-12013	Hypertension	0.055	26	0.412	0.252	1.000	TRUE
Gamma-glutamylmethionine*	Hypertension	0.052	23	0.399	0.682	0.713	TRUE
Gamma-glutamylthreonine*	Hypertension	0.035	23	6.43e-05	0.939	1.000	TRUE
X-12029	Hypertension	0.081	22	0.031	0.711	0.075	TRUE
X-12038	Hypertension	0.112	23	2.96e-10	0.241	1	TRUE
X-12039	Hypertension	0.053	23	0.688	0.885	0.800	TRUE
X-12040	Hypertension	0.399	25	0.001	0.008	0.455	TRUE
X-12056	Hypertension	0.059	23	2.07e-04	0.930	0.026	TRUE
X-12063	Hypertension	0.155	100	1.15e-04	0.816	0.703	TRUE
Gamma-tocopherol	Hypertension	0.027	23	0.893	0.637	1	TRUE
Gamma-glutamylphenylalanine	Hypertension	0.069	24	3.78e-04	0.681	1.000	TRUE

Exposure	Outcome	R2	F	Heterogeneity	Pleiotropy	power	Direction
Isobutyrylcarnitine	Hypertension	0.065	48	2.17e-04	0.920	1.000	TRUE
Pseudouridine	Hypertension	0.061	24	0.250	0.359	1	TRUE
Valerate	Hypertension	0.006	22			1	TRUE
Palmitoleate (16:1n7)	Hypertension	0.017	31	0.203	0.551	1.000	TRUE
Alpha-ketoglutarate	Hypertension	0.041	23	3.75e-04	0.666	1.000	TRUE
Erythronate*	Hypertension	0.090	25	6.17e-19	0.810	1	FALSE
Lathosterol	Hypertension	0.043	25	0.562	0.488	0.998	TRUE
X-12092	Hypertension	0.332	291	0.001	0.961	0.058	TRUE
X-12093	Hypertension	0.192	81	0.005	0.572	1.000	TRUE
X-12094	Hypertension	0.018	25	1.39e-05	0.269	0.117	TRUE
X-12095--N1-methyl-3-pyridone-4-carboxamide	Hypertension	0.050	24	0.010	0.520	0.999	TRUE
X-12100--hydroxytryptophan*	Hypertension	0.110	25	7.87e-04	0.715	1	TRUE
X-12116	Hypertension	0.030	22	0.664	0.558	0.997	TRUE
Eicosenoate (20:1n9 or 11)	Hypertension	0.019	23	0.360	0.255	1	TRUE
X-12188	Hypertension	0.303	26	0.092	0.189	0.810	TRUE
X-12189	Hypertension	0.975	24	0.341	0.419	0.979	TRUE
X-12206	Hypertension	0.039	22	0.054	0.370	1	TRUE

Exposure	Outcome	R2	F	Heterogeneity	Pleiotropy	power	Direction
X-12212	Hypertension	0.027	23	0.400	0.674	0.986	TRUE
X-12216	Hypertension	0.019	22			1.000	TRUE
X-12217	Hypertension	0.025	22	8.8e-05	0.947	0.719	TRUE
X-12230	Hypertension	0.009	25	0.232		0.115	TRUE
X-12231	Hypertension	0.033	23	1.71e-06	0.853	1.000	TRUE
X-12236	Hypertension	0.099	23	0.033	0.578	1	TRUE
X-12244--N-acetylcarnosine	Hypertension	0.077	38	0.186	0.878	0.998	TRUE
X-12253	Hypertension	0.016	23	0.012	0.937	0.385	TRUE
X-12261	Hypertension	0.155	23	0.548	0.710	0.998	TRUE
X-12329	Hypertension	0.494	22	0.034	0.978	0.351	TRUE
X-10346	Hypertension	0.051	22	0.284	0.710	0.126	TRUE
ADpSGEGDFXAEGGGVR*	Hypertension	0.044	43	0.032	0.650	0.684	TRUE
1-eicosatrienoylglycerophosphocholine*	Hypertension	0.048	28	0.001	0.517	0.981	TRUE
1-docosahexaenoylglycerophosphocholine*	Hypertension	0.012	22	0.417	0.320	0.039	TRUE
X-12405	Hypertension	0.014	23	0.527	0.312	0.186	TRUE
X-12407	Hypertension	0.044	24	0.224	0.169	0.339	TRUE
X-12428	Hypertension	0.031	22	0.524		1	TRUE

Exposure	Outcome	R2	F	Heterogeneity	Pleiotropy	power	Direction
1-eicosadienoylglycerophosphocholine*	Hypertension	0.022	24	0.872	0.336	1.000	TRUE
X-12435	Hypertension	1.5	24	0.142	0.217	1.000	TRUE
X-12441--12-hydroxyeicosatetraenoate (12-HETE)	Hypertension	0.078	36	0.048	0.474	1.000	TRUE
X-12442--5,8-tetradecadienoate	Hypertension	0.030	37	0.360	0.971	0.930	TRUE
X-12443	Hypertension	0.031	22	0.102	0.891	1.000	TRUE
X-12450	Hypertension	0.060	23	0.005	0.915	1.000	TRUE
X-12456	Hypertension	0.105	37	0.056	0.044	0.130	TRUE
X-12465	Hypertension	0.024	22	0.097	0.252	0.665	TRUE
Octanoylcarnitine	Hypertension	0.076	96	0.007	0.398	1.000	TRUE
Alpha-hydroxyisovalerate	Hypertension	0.047	38	0.055	0.149	0.750	TRUE
N-acetylthreonine	Hypertension	0.021	23	0.596	0.209	0.993	TRUE
Decanoylcarnitine	Hypertension	0.071	59	0.068	0.434	0.028	TRUE
1-palmitoylglycerophosphocholine	Hypertension	0.064	24	0.025	0.231	0.619	TRUE
1-heptadecanoylglycerophosphocholine	Hypertension	0.013	23	0.399	0.467	0.217	TRUE
1-oleoylglycerophosphocholine	Hypertension	0.022	23	0.563	0.207	1	TRUE
1-stearoylglycerophosphocholine	Hypertension	0.012	23	0.114	0.600	1.000	TRUE
5-dodecenoate (12:1n7)	Hypertension	0.038	25	0.141	0.115	1	TRUE

Exposure	Outcome	R2	F	Heterogeneity	Pleiotropy	power	Direction
Stearidonate (18:4n3)	Hypertension	0.021	31	0.407	0.765	0.998	TRUE
10-heptadecenoate (17:1n7)	Hypertension	0.003	23			1.000	TRUE
10-nonadecenoate (19:1n9)	Hypertension	0.013	25	0.003	0.808	0.998	TRUE
Epiandrosterone sulfate	Hypertension	0.075	63	0.296	0.108	1.000	TRUE
Linolenate [alpha or gamma; (18:3n3 or 6)]	Hypertension	0.006	22	0.249		0.115	TRUE
X-12510--2-aminooctanoic acid	Hypertension	0.129	90	0.006	0.338	1	TRUE
X-12524	Hypertension	0.050	24	0.020	0.352	1	TRUE
Bilirubin (E,Z or Z,E)*	Hypertension	0.080	51	0.631	0.555	0.449	TRUE
X-12544	Hypertension	0.040	23	0.492	0.486	0.888	TRUE
X-12556	Hypertension	0.054	29	0.011	0.127	1	TRUE
1-arachidonoylglycerophosphoinositol*	Hypertension	0.073	42	0.026	0.108	1	TRUE
X-12627	Hypertension	0.025	22	0.015	0.480	0.179	TRUE
X-12644	Hypertension	0.047	26	0.247	0.582	0.996	TRUE
X-12645	Hypertension	0.047	23	0.099	0.028	1.000	TRUE
Asparagine	Hypertension	0.104	29	0.004	0.195	1.000	TRUE
X-12680	Hypertension	0.088	22	0.493	0.257	0.937	TRUE
X-12696	Hypertension	0.060	33	0.118	0.894	1	TRUE

Exposure	Outcome	R2	F	Heterogeneity	Pleiotropy	power	Direction
X-12704	Hypertension	0.058	26	0.028	0.838	1.000	TRUE
X-12712	Hypertension	1.8	25	0.151	0.866	0.471	TRUE
X-12717	Hypertension	0.040	22	0.768	0.761	0.109	TRUE
X-12719	Hypertension	0.057	22	0.379	0.233	0.174	TRUE
X-12726	Hypertension	0.056	24	0.502	0.804	0.997	TRUE
X-12728	Hypertension	1.5	26	0.285	0.334	0.412	TRUE
X-12729	Hypertension	0.158	24	0.280	0.757	0.106	TRUE
X-12734	Hypertension	0.019	25	0.062	0.190	0.058	TRUE
X-12740	Hypertension	0.068	24	0.051	0.606	0.697	TRUE
X-12749	Hypertension	0.091	25	1.23e-06	0.888	1.000	TRUE
Stachydrine	Hypertension	0.016	22	0.906		0.929	TRUE
Isovalerylcarnitine	Hypertension	0.084	45	0.015	0.845	0.114	TRUE
Stearoylcarnitine	Hypertension	0.016	28	0.007	0.759	1.000	TRUE
1-stearoylglycerophosphoethanolamine	Hypertension	0.033	26	6.73e-07	0.593	1.000	TRUE
1-linoleoylglycerophosphocholine	Hypertension	0.032	24	0.217	0.081	0.772	TRUE
Bradykinin, des-arg(9)	Hypertension	0.135	44	0.007	0.519	0.512	TRUE
X-12771	Hypertension	0.051	23	0.001	0.429	1.000	TRUE

Exposure	Outcome	R2	F	Heterogeneity	Pleiotropy	power	Direction
X-12776	Hypertension	0.026	23	0.083		1	TRUE
Gamma-glutamylisoleucine*	Hypertension	0.064	22	5.08e-05	0.671	0.773	TRUE
X-12786	Hypertension	0.019	24	9.84e-04	0.737	0.998	TRUE
X-12798	Hypertension	0.173	233	0.098	0.859	0.573	TRUE
X-12816	Hypertension	0.016	24	0.433	0.694	0.945	TRUE
X-12830	Hypertension	0.054	22	0.016	0.324	0.073	TRUE
X-12833	Hypertension	0.902	24	0.004	0.484	0.957	TRUE
X-12844	Hypertension	0.032	33	0.439	0.991	1	TRUE
X-12847	Hypertension	0.012	23	0.553		0.109	TRUE
X-12850	Hypertension	0.039	33	0.012	0.693	0.400	TRUE
Laurylcarnitine	Hypertension	0.034	24	0.032	0.585	0.236	TRUE
X-12851	Hypertension	0.021	24	0.014	0.130	0.056	TRUE
X-12855	Hypertension	0.060	24	0.022	0.852	0.999	TRUE
X-12990--docosapentaenoic acid (n6-DPA)	Hypertension	0.062	27	0.449	0.435	0.934	TRUE
Isovalerate	Hypertension	0.006	22	0.236		0.988	TRUE
X-13069	Hypertension	0.042	24	0.004	0.301	1	TRUE
X-13183--stearamide	Hypertension	0.080	22	0.073	0.346	0.950	TRUE

Exposure	Outcome	R2	F	Heterogeneity	Pleiotropy	power	Direction
X-13215	Hypertension	0.103	23	4.12e-05	0.013	1	TRUE
7-methylguanine	Hypertension	0.015	22	0.560	0.596	0.414	TRUE
Phenylacetylglutamine	Hypertension	0.036	24	0.342	0.639	1.000	TRUE
Pro-hydroxy-pro	Hypertension	0.038	23	0.249	0.887	1	TRUE
N2,N2-dimethylguanosine	Hypertension	0.166	24	0.004	0.523	0.963	TRUE
Cysteine-glutathione disulfide	Hypertension	0.091	26	0.682	0.336	1.000	TRUE
Oleoylcarnitine	Hypertension	0.013	23	0.621	0.362	1.000	TRUE
1-arachidonoylglycerophosphoethanolamine*	Hypertension	0.074	39	0.125	0.057	0.141	TRUE
X-13429	Hypertension	0.077	95	0.252	0.814	1.000	TRUE
X-13431--nonanoylcarnitine*	Hypertension	0.129	86	0.002	0.018	0.104	TRUE
X-13435	Hypertension	0.066	40	5.77e-05	0.872	1	TRUE
X-13477	Hypertension	0.021	30	0.011	0.399	1	TRUE
2-palmitoylglycerophosphocholine*	Hypertension	0.045	22	0.018	0.249	0.764	TRUE
2-oleoylglycerophosphocholine*	Hypertension	0.029	23	0.182	0.115	0.898	TRUE
2-stearoylglycerophosphocholine*	Hypertension	0.030	25	0.028	0.431	0.976	TRUE
2-linoleoylglycerophosphocholine*	Hypertension	0.046	23	0.001	0.741	0.993	TRUE
X-13496	Hypertension	0.094	24	1.59e-11	0.078	1	TRUE

Exposure	Outcome	R2	F	Heterogeneity	Pleiotropy	power	Direction
1-palmitoylglycerophosphoinositol*	Hypertension	0.019	22	0.041	0.458	0.125	TRUE
X-13548	Hypertension	0.112	24	0.003	0.778	1	TRUE
X-13549	Hypertension	0.037	23	0.161	0.835	1	TRUE
X-13553	Hypertension	0.015	23	0.575		1.000	TRUE
X-13619	Hypertension	0.057	23	0.002	0.944	1	TRUE
2-methylbutyroylcarnitine	Hypertension	0.050	23	0.015	0.199	1	TRUE
Hydroxyisovaleroyl carnitine	Hypertension	0.028	37	0.015	0.183	1.000	TRUE
Glutaroyl carnitine	Hypertension	0.145	51	0.005	0.263	0.230	TRUE
X-13658	Hypertension	0.163	26	0.727	0.402	0.033	TRUE
X-13671	Hypertension	0.037	26	7.26e-04	0.932	1	TRUE
2-tetradecenoyl carnitine	Hypertension	0.041	25	0.297	0.721	0.999	TRUE
4-hydroxyhippurate	Hypertension	0.051	23	0.008	0.578	1.000	TRUE
X-13741	Hypertension	0.040	25	2e-04	0.129	1.000	TRUE
1-myristoylglycerophosphocholine	Hypertension	0.007	24	0.038		1	TRUE
1-oleoylglycerophosphoethanolamine	Hypertension	0.015	27	1.17e-04	0.689	0.977	TRUE
1-palmitoylglycerophosphoethanolamine	Hypertension	0.055	29	3.17e-04	0.743	0.036	TRUE
3-(3-hydroxyphenyl)propionate	Hypertension	0.157	22	1.44e-10	0.267	0.075	TRUE

Exposure	Outcome	R2	F	Heterogeneity	Pleiotropy	power	Direction
Tetradecanedioate	Hypertension	0.110	45	0.330	0.358	1.000	TRUE
2-hydroxypalmitate	Hypertension	0.098	24	2.62e-04	0.437	0.526	TRUE
Hexadecanedioate	Hypertension	0.095	42	0.004	0.172	0.992	TRUE
Dihomo-linolenate (20:3n3 or n6)	Hypertension	0.055	29	0.146	0.491	0.999	TRUE
X-13859	Hypertension	0.102	24	0.012	0.395	1.000	TRUE
Threitol	Hypertension	0.011	24	0.777	0.977	1.000	TRUE
X-14056	Hypertension	0.017	24	0.455	0.348	1.000	TRUE
X-14057	Hypertension	0.020	23	0.015	0.703	1.000	TRUE
X-14086	Hypertension	0.086	26	0.120	0.271	0.923	TRUE
P-cresol sulfate	Hypertension	0.026	24	0.112	0.448	0.511	TRUE
X-14189--leucylalanine	Hypertension	0.094	32	0.054	0.241	0.172	TRUE
X-14205--alpha-glutamyltyrosine	Hypertension	0.113	29	0.173	0.487	0.989	TRUE
X-14208--phenylalanylserine	Hypertension	0.122	30	0.490	0.780	1.000	TRUE
X-14304--leucylalanine	Hypertension	0.115	28	0.865	0.708	1	TRUE
X-14374	Hypertension	0.018	22	0.010	0.946	0.055	TRUE
X-14450--phenylalanylleucine	Hypertension	0.037	23	0.582	0.653	0.999	TRUE
X-14473	Hypertension	0.032	26	0.384	0.148	1.000	TRUE

Exposure	Outcome	R2	F	Heterogeneity	Pleiotropy	power	Direction
X-14541	Hypertension	0.129	23	0.261	0.463	1.000	TRUE
X-14588	Hypertension	0.019	24	0.638	0.275	1	TRUE
X-14625	Hypertension	0.037	26	0.144	0.917	1	TRUE
X-14626	Hypertension	0.064	42	8.89e-04	0.576	1	TRUE
X-14632	Hypertension	0.175	22	0.111	0.264	1	TRUE
X-14658	Hypertension	0.046	22	0.060	0.236	0.995	TRUE
X-14662	Hypertension	0.060	24	0.130	0.755	0.777	TRUE
X-14745	Hypertension	0.026	23	0.017	0.811	0.710	TRUE
Gamma-glutamylglutamate	Hypertension	0.200	24	0.739	0.627	1.000	TRUE
Octadecanedioate	Hypertension	0.027	37	0.002	0.487	0.279	TRUE
Leucylleucine	Hypertension	0.056	23	0.071	0.705	1.000	TRUE
7-alpha-hydroxy-3-oxo-4-cholestenoate (7-Hoca)	Hypertension	0.025	23	0.597	0.949	1.000	TRUE
N-Butyl Oleate	Hypertension	0.025	22	0.074	0.180	0.946	TRUE
Dimethylarginine (SDMA + ADMA)	Hypertension	0.069	23	1.64e-04	0.550	1	TRUE
Taurolithocholate 3-sulfate	Hypertension	0.021	23	1.73e-04	0.270	0.945	TRUE
X-14977--vanillin	Hypertension	0.023	21	0.787		0.419	TRUE
Succinylcarnitine	Hypertension	0.153	43	3.4e-09	0.197	1.000	TRUE

Exposure	Outcome	R2	F	Heterogeneity	Pleiotropy	power	Direction
Tryptophan betaine	Hypertension	0.037	38	0.009	0.483	0.999	TRUE
Cyclo(leu-pro)	Hypertension	0.049	22	0.025	0.010	1.000	TRUE
Chiro-inositol	Hypertension	0.054	23	0.477	0.385	0.211	TRUE
5alpha-androstan-3beta,17beta-diol disulfate	Hypertension	0.061	43	0.009	0.311	0.060	TRUE
4-androsten-3beta,17beta-diol disulfate 1*	Hypertension	0.099	50	0.107	0.908	1.000	TRUE
4-androsten-3beta,17beta-diol disulfate 2*	Hypertension	0.053	30	0.111	0.201	1.000	TRUE
2-hydroxyglutarate	Hypertension	0.019	22	0.452	0.829	0.940	TRUE
Ergothioneine	Hypertension	0.012	22			0.146	TRUE
Palmitoyl sphingomyelin	Hypertension	0.102	24	2.15e-07	0.942	1	TRUE
Phenylalanylphenylalanine	Hypertension	0.014	23	0.038	0.711	1.000	TRUE
Cis-4-decenoyl carnitine	Hypertension	0.055	82	0.083	0.840	1.000	TRUE
15-methylpalmitate (isobar with 2-methylpalmitate)	Hypertension	0.030	23	0.350	0.789	1.000	TRUE

n: 2187

Table S2. Full result of MR estimates for the effect of metabolites on hypertension(FinnGen).

Exposure	Outcome	nSNP	MR Egger		Weighted median		Inverse variance weighted		MR_PRESSO	
			OR (95% CI)	pval	OR (95% CI)	pval	OR (95% CI)	pval	OR (95% CI)	pval
arachidonate (20:4n6)	Hypertension	52	0.983 (0.971 to 0.996)	0.013	0.993 (0.988 to 0.998)	0.010	0.994 (0.989 to 0.998)	0.004		
deoxycholate	Hypertension	12	1.026 (0.923 to 1.140)	0.647	1.007 (0.975 to 1.040)	0.678	0.998 (0.967 to 1.031)	0.919	1.008 (0.980 to 1.038)	0.576
arachidate (20:0)	Hypertension	14	1.018 (0.963 to 1.076)	0.546	0.993 (0.966 to 1.021)	0.622	0.997 (0.972 to 1.022)	0.809	1.004 (0.982 to 1.026)	0.740
margarate (17:0)	Hypertension	11	1.023 (0.967 to 1.083)	0.444	0.988 (0.958 to 1.019)	0.460	1.011 (0.986 to 1.037)	0.380		
inosine	Hypertension	21	0.997 (0.974 to 1.021)	0.830	1.002 (0.985	0.831	1.001 (0.988	0.864		

Exposure	Outcome	nSNP	MR Egger		Weighted median		Inverse variance weighted		MR_PRESSO	
			OR (95% CI)	pval	OR (95% CI)	pval	OR (95% CI)	pval	OR (95% CI)	pval
					to 1.019)		to 1.015)			
myo-inositol	Hypertension	13	1.019 (0.972 to 1.068)	0.448	1.020 (0.994 to 1.046)	0.141	1.012 (0.989 to 1.036)	0.305		
isoleucine	Hypertension	13	1.007 (0.962 to 1.055)	0.761	0.990 (0.964 to 1.017)	0.465	0.994 (0.975 to 1.014)	0.561		
alanine	Hypertension	15	0.986 (0.948 to 1.026)	0.507	1.011 (0.982 to 1.040)	0.467	0.995 (0.975 to 1.016)	0.657		
3-methoxytyrosine	Hypertension	20	0.996 (0.982 to 1.011)	0.634	0.998 (0.978 to 1.018)	0.837	1.004 (0.994 to 1.015)	0.417		

Exposure	Outcome	nSNP	MR Egger		Weighted median		Inverse variance weighted		MR_PRESSO	
			OR (95% CI)	pval	OR (95% CI)	pval	OR (95% CI)	pval	OR (95% CI)	pval
beta-hydroxyisovalerate	Hypertension	24	1.002 (0.987 to 1.017)	0.774	1.005 (0.993 to 1.018)	0.412	1.009 (1.000 to 1.019)	0.042		
taurodeoxycholate	Hypertension	16	0.970 (0.931 to 1.011)	0.172	0.978 (0.954 to 1.002)	0.068	0.988 (0.969 to 1.007)	0.208		
threonine	Hypertension	13	1.003 (0.918 to 1.097)	0.942	0.993 (0.963 to 1.024)	0.667	1.015 (0.981 to 1.049)	0.393	0.996 (0.974 to 1.019)	0.743
tyrosine	Hypertension	16	1.004 (0.957 to 1.054)	0.864	1.004 (0.982 to 1.027)	0.732	0.995 (0.973 to 1.017)	0.649	1.001 (0.984 to 1.019)	0.897
lysine	Hypertension	20	1.008 (0.994 to 1.022)	0.279	1.005 (0.996 to 1.015)	0.294	1.004 (0.995 to 1.012)	0.375		

Exposure	Outcome	nSNP	MR Egger		Weighted median		Inverse variance weighted		MR_PRESSO	
			OR (95% CI)	pval	OR (95% CI)	pval	OR (95% CI)	pval	OR (95% CI)	pval
malate	Hypertension	64	0.986 (0.973 to 0.999)	0.040	0.996 (0.986 to 1.007)	0.503	0.995 (0.986 to 1.004)	0.263	0.994 (0.985 to 1.002)	0.137
palmitate (16:0)	Hypertension	29	1.003 (0.994 to 1.012)	0.508	0.998 (0.992 to 1.005)	0.564	0.997 (0.992 to 1.002)	0.277		
myristate (14:0)	Hypertension	26	0.997 (0.968 to 1.028)	0.871	0.994 (0.975 to 1.014)	0.570	0.996 (0.982 to 1.009)	0.545		
kynurenate	Hypertension	19	1.011 (0.979 to 1.044)	0.515	1.007 (0.986 to 1.027)	0.525	1.018 (1.001 to 1.036)	0.040	1.014 (0.998 to 1.030)	0.094
5,6-dihydrothymine	Hypertension	25	1.002 (0.969 to 1.036)	0.923	0.992 (0.974 to 1.010)	0.382	0.992 (0.979 to 1.004)	0.192		

Exposure	Outcome	nSNP	MR Egger		Weighted median		Inverse variance weighted		MR_PRESSO	
			OR (95% CI)	pval	OR (95% CI)	pval	OR (95% CI)	pval	OR (95% CI)	pval
5-methylthioadenosine (MTA)	Hypertension	16	1.030 (0.990 to 1.071)	0.166	1.010 (0.987 to 1.035)	0.395	0.999 (0.982 to 1.016)	0.905		
2-hydroxyphenylacetate	Hypertension	20	1.038 (0.978 to 1.101)	0.239	0.998 (0.973 to 1.025)	0.892	1.002 (0.978 to 1.026)	0.878	0.994 (0.972 to 1.017)	0.629
succinate	Hypertension	21	1.001 (0.972 to 1.031)	0.948	0.990 (0.971 to 1.009)	0.288	0.998 (0.981 to 1.015)	0.786		
ornithine	Hypertension	18	1.030 (0.970 to 1.093)	0.356	1.018 (0.995 to 1.041)	0.136	1.003 (0.984 to 1.024)	0.733		
5-oxoproline	Hypertension	22	1.016 (0.986 to 1.048)	0.313	1.009 (0.992 to 1.026)	0.328	1.015 (0.999 to 1.032)	0.066	1.013 (0.999 to 1.028)	0.091

Exposure	Outcome	nSNP	MR Egger		Weighted median		Inverse variance weighted		MR_PRESSO	
			OR (95% CI)	pval	OR (95% CI)	pval	OR (95% CI)	pval	OR (95% CI)	pval
N6,N6,N6-trimethyllysine	Hypertension	10	0.989 (0.950 to 1.030)	0.612	1.006 (0.979 to 1.033)	0.675	0.998 (0.978 to 1.018)	0.814		
orotate	Hypertension	18	0.992 (0.964 to 1.020)	0.583	0.995 (0.980 to 1.010)	0.504	0.995 (0.981 to 1.010)	0.511	0.992 (0.980 to 1.004)	0.211
pantothenate	Hypertension	16	1.005 (0.973 to 1.037)	0.783	0.981 (0.960 to 1.003)	0.084	0.984 (0.963 to 1.004)	0.119	0.989 (0.977 to 1.001)	0.096
glycerol	Hypertension	17	0.967 (0.931 to 1.005)	0.105	0.961 (0.940 to 0.983)	5.19e-04	0.983 (0.962 to 1.003)	0.094	0.976 (0.958 to 0.994)	0.021
kynurenine	Hypertension	16	1.016 (0.946 to 1.090)	0.678	0.991 (0.964 to 1.017)	0.488	0.965 (0.933 to 0.997)	0.034	0.981 (0.959 to 1.003)	0.112

Exposure	Outcome	nSNP	MR Egger		Weighted median		Inverse variance weighted		MR_PRESSO	
			OR (95% CI)	pval	OR (95% CI)	pval	OR (95% CI)	pval	OR (95% CI)	pval
sucrose	Hypertension	9	0.999 (0.979 to 1.020)	0.958	1.011 (1.000 to 1.021)	0.040	1.003 (0.993 to 1.014)	0.551		
tartarate	Hypertension	18	1.012 (0.959 to 1.069)	0.667	1.002 (0.982 to 1.023)	0.824	1.005 (0.982 to 1.029)	0.679	0.996 (0.982 to 1.010)	0.551
carnitine	Hypertension	35	1.002 (0.991 to 1.013)	0.719	0.998 (0.989 to 1.006)	0.597	0.993 (0.986 to 1.000)	0.061	0.992 (0.987 to 0.998)	0.009
choline	Hypertension	19	1.000 (0.971 to 1.029)	0.982	1.003 (0.983 to 1.023)	0.797	1.003 (0.989 to 1.018)	0.661		
4-acetamidobutanoate	Hypertension	17	1.000 (0.968 to 1.033)	0.984	1.003 (0.983 to 1.023)	0.771	1.005 (0.989 to 1.020)	0.569		

Exposure	Outcome	nSNP	MR Egger		Weighted median		Inverse variance weighted		MR_PRESSO	
			OR (95% CI)	pval	OR (95% CI)	pval	OR (95% CI)	pval	OR (95% CI)	pval
chenodeoxycholate	Hypertension	18	1.023 (0.978 to 1.071)	0.336	1.009 (0.983 to 1.036)	0.500	1.008 (0.984 to 1.033)	0.500	1.002 (0.981 to 1.025)	0.835
citrate	Hypertension	16	0.991 (0.937 to 1.048)	0.749	0.986 (0.959 to 1.014)	0.324	0.987 (0.960 to 1.016)	0.374	0.987 (0.965 to 1.010)	0.280
N1-methyladenosine	Hypertension	16	1.013 (0.968 to 1.061)	0.587	1.009 (0.986 to 1.034)	0.438	1.004 (0.984 to 1.023)	0.716		
3-aminoisobutyrate	Hypertension	22	0.974 (0.955 to 0.993)	0.015	0.986 (0.972 to 1.000)	0.045	0.997 (0.985 to 1.009)	0.605		
2-isopropylmalate	Hypertension	19	1.001 (0.961 to 1.043)	0.954	1.008 (0.983 to 1.035)	0.527	1.011 (0.990 to 1.033)	0.293		

Exposure	Outcome	nSNP	MR Egger		Weighted median		Inverse variance weighted		MR_PRESSO	
			OR (95% CI)	pval	OR (95% CI)	pval	OR (95% CI)	pval	OR (95% CI)	pval
vanillylmandelate (VMA)	Hypertension	17	1.009 (0.990 to 1.029)	0.357	1.010 (0.992 to 1.029)	0.290	1.014 (1.002 to 1.026)	0.025		
3-methylhistidine	Hypertension	28	0.996 (0.969 to 1.024)	0.797	0.999 (0.983 to 1.016)	0.950	0.998 (0.983 to 1.013)	0.791	0.994 (0.980 to 1.007)	0.378
xanthurenate	Hypertension	17	0.981 (0.947 to 1.016)	0.298	0.999 (0.976 to 1.022)	0.908	1.010 (0.987 to 1.034)	0.387	1.000 (0.989 to 1.011)	0.993
4-guanidinobutanoate	Hypertension	25	0.956 (0.924 to 0.989)	0.015	0.987 (0.972 to 1.002)	0.091	1.005 (0.991 to 1.019)	0.460		
5-hydroxylysine	Hypertension	32	0.997 (0.974 to 1.020)	0.781	1.006 (0.990 to 1.022)	0.446	1.000 (0.986 to 1.014)	0.982	1.005 (0.991 to 1.020)	0.459

Exposure	Outcome	nSNP	MR Egger		Weighted median		Inverse variance weighted		MR_PRESSO	
			OR (95% CI)	pval	OR (95% CI)	pval	OR (95% CI)	pval	OR (95% CI)	pval
pimelate (heptanedioate)	Hypertension	15	1.028 (0.989 to 1.070)	0.188	1.004 (0.982 to 1.026)	0.713	1.005 (0.989 to 1.021)	0.522		
cystathionine	Hypertension	16	1.000 (0.966 to 1.034)	0.982	1.000 (0.979 to 1.023)	0.973	1.001 (0.982 to 1.021)	0.895	0.997 (0.981 to 1.013)	0.696
imidazole lactate	Hypertension	21	0.991 (0.951 to 1.033)	0.674	0.985 (0.970 to 0.999)	0.041	0.988 (0.975 to 1.002)	0.085	0.988 (0.976 to 1.001)	0.085
glycerate	Hypertension	26	1.007 (0.983 to 1.032)	0.589	1.000 (0.982 to 1.017)	0.972	1.000 (0.988 to 1.013)	0.986		
guanosine	Hypertension	20	1.010 (0.997 to 1.022)	0.146	1.002 (0.994 to 1.010)	0.672	1.002 (0.996 to 1.008)	0.532		

Exposure	Outcome	nSNP	MR Egger		Weighted median		Inverse variance weighted		MR_PRESSO	
			OR (95% CI)	pval	OR (95% CI)	pval	OR (95% CI)	pval	OR (95% CI)	pval
suberate (octanedioate)	Hypertension	19	0.996 (0.966 to 1.028)	0.825	1.027 (1.005 to 1.050)	0.015	1.012 (0.996 to 1.028)	0.137		
methylsuccinate	Hypertension	19	1.001 (0.961 to 1.042)	0.972	0.991 (0.977 to 1.006)	0.254	0.991 (0.980 to 1.003)	0.137		
hippurate	Hypertension	19	1.012 (0.981 to 1.044)	0.456	1.017 (0.996 to 1.038)	0.110	1.008 (0.991 to 1.026)	0.365		
ethylmalonate	Hypertension	86	0.997 (0.988 to 1.007)	0.573	1.000 (0.993 to 1.006)	0.963	0.998 (0.994 to 1.002)	0.421	0.998 (0.994 to 1.002)	0.296
ribitol	Hypertension	28	1.037 (1.015 to 1.059)	0.003	1.026 (1.011 to 1.041)	4.46e-04	1.022 (1.009 to 1.034)	6.77e-04		

Exposure	Outcome	nSNP	MR Egger		Weighted median		Inverse variance weighted		MR_PRESSO	
			OR (95% CI)	pval	OR (95% CI)	pval	OR (95% CI)	pval	OR (95% CI)	pval
N-acetylalanine	Hypertension	21	0.992 (0.959 to 1.026)	0.639	0.994 (0.973 to 1.016)	0.607	0.999 (0.984 to 1.013)	0.880		
malonate	Hypertension	41	0.997 (0.983 to 1.013)	0.742	1.003 (0.990 to 1.016)	0.667	1.002 (0.994 to 1.011)	0.610		
N-acetylmethionine	Hypertension	20	0.988 (0.923 to 1.057)	0.731	1.010 (0.984 to 1.037)	0.445	1.016 (0.988 to 1.045)	0.275	1.015 (0.992 to 1.039)	0.219
phenylacetate	Hypertension	8	0.970 (0.919 to 1.023)	0.302	0.976 (0.949 to 1.004)	0.093	0.980 (0.952 to 1.008)	0.162		
urate	Hypertension	26	1.005 (0.974 to 1.037)	0.748	1.007 (0.989 to 1.026)	0.435	1.009 (0.996 to 1.023)	0.159		

Exposure	Outcome	nSNP	MR Egger		Weighted median		Inverse variance weighted		MR_PRESSO	
			OR (95% CI)	pval	OR (95% CI)	pval	OR (95% CI)	pval	OR (95% CI)	pval
ursodeoxycholate	Hypertension	17	0.988 (0.949 to 1.028)	0.556	0.995 (0.976 to 1.013)	0.570	1.001 (0.982 to 1.020)	0.934	0.993 (0.978 to 1.009)	0.411
arginine	Hypertension	20	0.976 (0.902 to 1.056)	0.549	0.984 (0.957 to 1.012)	0.261	0.985 (0.957 to 1.013)	0.295	0.985 (0.960 to 1.011)	0.279
fumarate	Hypertension	18	0.991 (0.948 to 1.035)	0.681	0.995 (0.975 to 1.015)	0.618	1.000 (0.979 to 1.022)	0.966	0.996 (0.976 to 1.016)	0.698
serine	Hypertension	17	0.976 (0.951 to 1.001)	0.080	0.987 (0.970 to 1.005)	0.159	0.984 (0.971 to 0.998)	0.024		
valine	Hypertension	16	0.998 (0.955 to 1.043)	0.922	0.987 (0.962 to 1.012)	0.305	1.002 (0.982 to 1.022)	0.871		

Exposure	Outcome	nSNP	MR Egger		Weighted median		Inverse variance weighted		MR_PRESSO	
			OR (95% CI)	pval	OR (95% CI)	pval	OR (95% CI)	pval	OR (95% CI)	pval
urea	Hypertension	13	0.985 (0.955 to 1.015)	0.349	0.992 (0.970 to 1.014)	0.473	0.995 (0.977 to 1.013)	0.596		
cortisol	Hypertension	15	0.984 (0.952 to 1.016)	0.340	0.990 (0.968 to 1.013)	0.397	0.989 (0.971 to 1.008)	0.258		
cortisone	Hypertension	24	0.997 (0.971 to 1.023)	0.810	1.001 (0.985 to 1.018)	0.871	1.002 (0.986 to 1.018)	0.830	1.006 (0.991 to 1.021)	0.473
2-hydroxystearate	Hypertension	22	1.073 (1.001 to 1.149)	0.061	1.014 (0.990 to 1.038)	0.269	1.009 (0.988 to 1.030)	0.394	1.010 (0.994 to 1.026)	0.239
retinol (Vitamin A)	Hypertension	17	0.992 (0.938 to 1.050)	0.796	0.977 (0.955 to 1.000)	0.051	0.979 (0.960 to 0.997)	0.025		

Exposure	Outcome	nSNP	MR Egger		Weighted median		Inverse variance weighted		MR_PRESSO	
			OR (95% CI)	pval	OR (95% CI)	pval	OR (95% CI)	pval	OR (95% CI)	pval
gamma-glutamylhistidine	Hypertension	15	0.989 (0.964 to 1.015)	0.405	0.991 (0.971 to 1.011)	0.370	0.996 (0.982 to 1.010)	0.555		
paraxanthine	Hypertension	21	0.979 (0.957 to 1.001)	0.082	0.974 (0.958 to 0.991)	0.002	0.979 (0.967 to 0.992)	0.001		
quinate	Hypertension	20	1.026 (1.000 to 1.053)	0.065	1.016 (0.997 to 1.035)	0.106	1.011 (0.997 to 1.025)	0.123		
indolelactate	Hypertension	32	0.969 (0.935 to 1.003)	0.087	0.983 (0.964 to 1.001)	0.069	0.994 (0.978 to 1.010)	0.463	0.986 (0.973 to 1.000)	0.056
gamma-glutamylleucine	Hypertension	22	0.985 (0.964 to 1.008)	0.214	1.005 (0.988 to 1.022)	0.573	1.005 (0.991 to 1.018)	0.508		

Exposure	Outcome	nSNP	MR Egger		Weighted median		Inverse variance weighted		MR_PRESSO	
			OR (95% CI)	pval	OR (95% CI)	pval	OR (95% CI)	pval	OR (95% CI)	pval
methionine sulfoxide	Hypertension	56	1.009 (0.988 to 1.030)	0.402	1.013 (1.001 to 1.026)	0.036	1.005 (0.994 to 1.015)	0.394	1.001 (0.992 to 1.011)	0.779
theobromine	Hypertension	18	0.983 (0.927 to 1.042)	0.578	0.996 (0.970 to 1.022)	0.763	1.000 (0.975 to 1.026)	0.988	0.999 (0.978 to 1.021)	0.957
theophylline	Hypertension	30	0.977 (0.953 to 1.001)	0.072	0.978 (0.960 to 0.995)	0.014	0.987 (0.973 to 1.002)	0.079	0.983 (0.971 to 0.996)	0.015
eicosapentaenoate (EPA; 20:5n3)	Hypertension	51	0.962 (0.935 to 0.991)	0.013	0.984 (0.972 to 0.995)	0.006	0.986 (0.978 to 0.995)	0.001		
glycocholate	Hypertension	14	0.973 (0.935 to 1.012)	0.199	0.998 (0.973 to 1.023)	0.854	0.993 (0.975 to 1.011)	0.429		

Exposure	Outcome	nSNP	MR Egger		Weighted median		Inverse variance weighted		MR_PRESSO	
			OR (95% CI)	pval	OR (95% CI)	pval	OR (95% CI)	pval	OR (95% CI)	pval
taurochenodeoxycholate	Hypertension	13	1.032 (0.990 to 1.076)	0.165	1.047 (1.021 to 1.074)	3.43e-04	1.041 (1.022 to 1.059)	1.49e-05		
taurocholate	Hypertension	14	0.968 (0.915 to 1.023)	0.271	0.990 (0.967 to 1.014)	0.397	0.990 (0.971 to 1.009)	0.302		
proline	Hypertension	39	1.007 (0.983 to 1.032)	0.549	0.993 (0.977 to 1.010)	0.437	0.998 (0.986 to 1.010)	0.708		
1,2-dimyristoyl-GPC (14:0/14:0)	Hypertension	19	0.998 (0.960 to 1.038)	0.927	1.004 (0.983 to 1.027)	0.696	1.002 (0.986 to 1.018)	0.806		
quinolinate	Hypertension	31	1.034 (0.989 to 1.082)	0.153	0.992 (0.974 to 1.010)	0.391	0.989 (0.965 to 1.014)	0.397	0.995 (0.981 to 1.010)	0.519

Exposure	Outcome	nSNP	MR Egger		Weighted median		Inverse variance weighted		MR_PRESSO	
			OR (95% CI)	pval	OR (95% CI)	pval	OR (95% CI)	pval	OR (95% CI)	pval
1,2-distearoyl-GPC (18:0/18:0)	Hypertension	22	1.013 (0.996 to 1.030)	0.147	0.996 (0.984 to 1.009)	0.561	1.001 (0.991 to 1.012)	0.808	0.995 (0.980 to 1.010)	0.486
1-myristoyl-2-palmitoyl-GPC (14:0/16:0)	Hypertension	19	1.010 (0.962 to 1.060)	0.694	0.998 (0.976 to 1.021)	0.878	0.999 (0.977 to 1.022)	0.954		
1-palmitoyl-2-oleoyl-GPE (16:0/18:1)	Hypertension	61	1.004 (0.988 to 1.022)	0.608	1.011 (1.002 to 1.021)	0.019	1.014 (1.007 to 1.021)	8.84e-05		
2-arachidonoylglycerol (20:4)	Hypertension	41	0.985 (0.939 to 1.033)	0.528	0.987 (0.976 to 0.997)	0.012	0.992 (0.984 to 1.000)	0.039		
1-stearoyl-GPI (18:0)	Hypertension	31	1.022 (0.992 to 1.053)	0.163	1.001 (0.984 to 1.017)	0.950	0.997 (0.983 to 1.011)	0.665		

Exposure	Outcome	nSNP	MR Egger		Weighted median		Inverse variance weighted		MR_PRESSO	
			OR (95% CI)	pval	OR (95% CI)	pval	OR (95% CI)	pval	OR (95% CI)	pval
stearoyl sphingomyelin (d18:1/18:0)	Hypertension	28	1.002 (0.968 to 1.037)	0.923	0.993 (0.974 to 1.012)	0.483	0.995 (0.980 to 1.011)	0.541		
1-palmityl-GPC (O-16:0)	Hypertension	14	0.958 (0.907 to 1.011)	0.145	0.995 (0.971 to 1.019)	0.678	0.988 (0.963 to 1.015)	0.394	0.996 (0.972 to 1.020)	0.733
1,5-anhydroglucitol (1,5-AG)	Hypertension	29	1.001 (0.969 to 1.035)	0.940	1.001 (0.984 to 1.019)	0.920	1.004 (0.988 to 1.021)	0.600	1.012 (0.998 to 1.027)	0.096
oxalate (ethanedioate)	Hypertension	19	1.035 (0.996 to 1.075)	0.098	1.016 (0.994 to 1.038)	0.152	1.024 (1.009 to 1.040)	0.002		
erythritol	Hypertension	18	1.004 (0.961 to 1.048)	0.870	1.038 (1.013 to 1.063)	0.003	1.011 (0.991 to 1.032)	0.283		

Exposure	Outcome	nSNP	MR Egger		Weighted median		Inverse variance weighted		MR_PRESSO	
			OR (95% CI)	pval	OR (95% CI)	pval	OR (95% CI)	pval	OR (95% CI)	pval
glycylglycine	Hypertension	28	1.012 (0.966 to 1.060)	0.631	1.019 (1.004 to 1.034)	0.015	1.009 (0.991 to 1.026)	0.324	1.018 (1.010 to 1.027)	2.97e-04
1-oleoylglycerol (18:1)	Hypertension	14	1.003 (0.935 to 1.076)	0.928	1.015 (0.986 to 1.045)	0.325	1.007 (0.973 to 1.043)	0.680	1.041 (1.020 to 1.063)	0.003
citrulline	Hypertension	15	1.017 (0.937 to 1.104)	0.697	0.998 (0.967 to 1.029)	0.890	1.007 (0.987 to 1.027)	0.501		
biliverdin	Hypertension	23	0.999 (0.985 to 1.013)	0.860	0.999 (0.989 to 1.009)	0.854	0.998 (0.990 to 1.006)	0.637		
3-hydroxyoctanoate	Hypertension	22	1.002 (0.992 to 1.013)	0.688	1.003 (0.996 to 1.010)	0.453	1.001 (0.994 to 1.008)	0.794		

Exposure	Outcome	nSNP	MR Egger		Weighted median		Inverse variance weighted		MR_PRESSO	
			OR (95% CI)	pval	OR (95% CI)	pval	OR (95% CI)	pval	OR (95% CI)	pval
2-hydroxyoctanoate	Hypertension	28	0.974 (0.917 to 1.035)	0.408	0.955 (0.935 to 0.975)	9.82e-06	0.954 (0.936 to 0.972)	8.87e-07	0.948 (0.932 to 0.964)	1.42e-06
3-hydroxydecanoate	Hypertension	26	1.017 (1.001 to 1.034)	0.046	1.007 (0.995 to 1.018)	0.248	1.004 (0.995 to 1.013)	0.371		
phenyllactate (PLA)	Hypertension	22	0.998 (0.964 to 1.032)	0.896	0.995 (0.976 to 1.014)	0.590	0.994 (0.979 to 1.011)	0.504	0.988 (0.974 to 1.002)	0.112
alpha-hydroxyisocaproate	Hypertension	15	1.011 (0.962 to 1.062)	0.676	1.037 (1.011 to 1.063)	0.005	1.020 (0.998 to 1.042)	0.073		
homoarginine	Hypertension	20	0.990 (0.953 to 1.029)	0.632	0.984 (0.965 to 1.003)	0.094	1.003 (0.984 to 1.022)	0.770	0.993 (0.976 to 1.011)	0.463

Exposure	Outcome	nSNP	MR Egger		Weighted median		Inverse variance weighted		MR_PRESSO	
			OR (95% CI)	pval	OR (95% CI)	pval	OR (95% CI)	pval	OR (95% CI)	pval
homocitrulline	Hypertension	8	1.036 (0.942 to 1.139)	0.493	1.013 (0.983 to 1.043)	0.400	1.004 (0.982 to 1.027)	0.723		
N-acetylaspartate (NAA)	Hypertension	47	1.003 (0.984 to 1.023)	0.734	1.009 (0.995 to 1.023)	0.200	1.005 (0.994 to 1.017)	0.377	1.002 (0.992 to 1.012)	0.708
pyroglutamylglutamine	Hypertension	20	1.048 (1.018 to 1.079)	0.005	1.011 (0.992 to 1.031)	0.240	1.013 (0.999 to 1.026)	0.062		
cholate	Hypertension	13	1.078 (0.927 to 1.254)	0.350	0.999 (0.968 to 1.031)	0.968	1.011 (0.972 to 1.052)	0.572	0.989 (0.967 to 1.010)	0.325
gamma-glutamylglutamine	Hypertension	17	1.000 (0.977 to 1.024)	0.978	0.995 (0.984 to 1.005)	0.313	0.995 (0.984 to 1.006)	0.346	0.995 (0.988 to 1.002)	0.170

Exposure	Outcome	nSNP	MR Egger		Weighted median		Inverse variance weighted		MR_PRESSO	
			OR (95% CI)	pval	OR (95% CI)	pval	OR (95% CI)	pval	OR (95% CI)	pval
gamma-glutamyltyrosine	Hypertension	13	1.011 (0.941 to 1.085)	0.779	1.000 (0.968 to 1.033)	0.994	1.009 (0.984 to 1.034)	0.496		
1-linoleoylglycerol (18:2)	Hypertension	27	0.996 (0.961 to 1.033)	0.838	1.010 (0.991 to 1.030)	0.310	1.020 (1.004 to 1.037)	0.014		
thyroxine	Hypertension	13	0.967 (0.917 to 1.021)	0.252	0.987 (0.959 to 1.016)	0.373	0.995 (0.970 to 1.020)	0.680		
1-methylnicotinamide	Hypertension	14	0.969 (0.924 to 1.016)	0.216	0.973 (0.946 to 1.000)	0.048	0.983 (0.963 to 1.003)	0.093		
3-indoxyl sulfate	Hypertension	9	1.019 (0.949 to 1.094)	0.626	1.023 (0.986 to 1.061)	0.227	1.010 (0.977 to 1.044)	0.566		

Exposure	Outcome	nSNP	MR Egger		Weighted median		Inverse variance weighted		MR_PRESSO	
			OR (95% CI)	pval	OR (95% CI)	pval	OR (95% CI)	pval	OR (95% CI)	pval
N-acetylglycine	Hypertension	19	1.001 (0.979 to 1.024)	0.929	1.006 (0.994 to 1.018)	0.328	0.992 (0.980 to 1.003)	0.155	0.993 (0.983 to 1.004)	0.228
creatine	Hypertension	17	0.996 (0.962 to 1.031)	0.816	0.970 (0.946 to 0.995)	0.018	0.971 (0.953 to 0.989)	0.002		
galactonate	Hypertension	20	1.033 (0.964 to 1.108)	0.368	1.007 (0.982 to 1.033)	0.586	1.022 (0.993 to 1.052)	0.138	1.007 (0.985 to 1.030)	0.540
1-methylhistidine	Hypertension	20	1.005 (0.946 to 1.068)	0.864	1.002 (0.978 to 1.027)	0.850	1.007 (0.983 to 1.032)	0.555	1.001 (0.982 to 1.020)	0.917
hypoxanthine	Hypertension	21	0.996 (0.898 to 1.105)	0.939	0.987 (0.967 to 1.008)	0.234	0.983 (0.947 to 1.021)	0.381	1.000 (0.985 to 1.016)	0.987

Exposure	Outcome	nSNP	MR Egger		Weighted median		Inverse variance weighted		MR_PRESSO	
			OR (95% CI)	pval	OR (95% CI)	pval	OR (95% CI)	pval	OR (95% CI)	pval
betaine	Hypertension	21	1.007 (0.986 to 1.027)	0.528	0.999 (0.985 to 1.013)	0.906	0.997 (0.983 to 1.010)	0.629	0.999 (0.987 to 1.012)	0.915
xanthine	Hypertension	15	0.976 (0.911 to 1.047)	0.515	0.991 (0.963 to 1.020)	0.532	0.968 (0.940 to 0.997)	0.030	0.992 (0.976 to 1.009)	0.383
pyroglutamylglycine	Hypertension	18	0.990 (0.946 to 1.036)	0.660	1.005 (0.981 to 1.028)	0.705	1.007 (0.985 to 1.030)	0.513	1.011 (0.994 to 1.028)	0.233
DSGEGDFXAEGGGVR	Hypertension	33	0.987 (0.932 to 1.047)	0.673	0.988 (0.968 to 1.009)	0.251	0.967 (0.940 to 0.995)	0.020	0.993 (0.976 to 1.010)	0.440
3-ureidopropionate	Hypertension	13	0.986 (0.916 to 1.061)	0.714	0.983 (0.953 to 1.013)	0.256	1.002 (0.977 to 1.028)	0.871		

Exposure	Outcome	nSNP	MR Egger		Weighted median		Inverse variance weighted		MR_PRESSO	
			OR (95% CI)	pval	OR (95% CI)	pval	OR (95% CI)	pval	OR (95% CI)	pval
pyridoxate	Hypertension	25	0.976 (0.951 to 1.002)	0.083	0.993 (0.973 to 1.014)	0.509	0.994 (0.980 to 1.009)	0.421		
androsterone sulfate	Hypertension	38	1.000 (0.990 to 1.010)	0.934	1.002 (0.994 to 1.009)	0.636	1.003 (0.996 to 1.010)	0.360		
2-methylbutyrylglycine	Hypertension	19	1.026 (0.987 to 1.066)	0.209	1.020 (0.999 to 1.041)	0.061	1.014 (0.998 to 1.030)	0.083		
propionylglycine	Hypertension	26	0.959 (0.901 to 1.021)	0.207	1.001 (0.981 to 1.022)	0.900	1.015 (0.995 to 1.036)	0.149	1.010 (0.992 to 1.029)	0.289
5-hydroxyhexanoate	Hypertension	13	1.004 (0.961 to 1.048)	0.874	0.992 (0.970 to 1.014)	0.476	0.991 (0.975 to 1.007)	0.276		

Exposure	Outcome	nSNP	MR Egger		Weighted median		Inverse variance weighted		MR_PRESSO	
			OR (95% CI)	pval	OR (95% CI)	pval	OR (95% CI)	pval	OR (95% CI)	pval
3-hydroxysebacate	Hypertension	29	0.999 (0.970 to 1.028)	0.921	0.997 (0.982 to 1.011)	0.657	0.998 (0.986 to 1.009)	0.688		
3-(4-hydroxyphenyl)lactate	Hypertension	17	0.959 (0.918 to 1.002)	0.078	0.992 (0.970 to 1.015)	0.515	0.998 (0.974 to 1.022)	0.882	0.999 (0.980 to 1.019)	0.945
acetylcarnitine	Hypertension	27	1.011 (0.977 to 1.046)	0.532	1.001 (0.979 to 1.024)	0.943	1.001 (0.985 to 1.016)	0.946		
trans-4-hydroxyproline	Hypertension	17	1.020 (0.947 to 1.098)	0.609	0.962 (0.934 to 0.990)	0.009	0.976 (0.951 to 1.002)	0.068		
hexanoylcarnitine	Hypertension	31	0.986 (0.960 to 1.012)	0.297	1.007 (0.995 to 1.020)	0.257	1.006 (0.995 to 1.017)	0.308	1.004 (0.994 to 1.014)	0.450

Exposure	Outcome	nSNP	MR Egger		Weighted median		Inverse variance weighted		MR_PRESSO	
			OR (95% CI)	pval	OR (95% CI)	pval	OR (95% CI)	pval	OR (95% CI)	pval
glycochenodeoxycholate	Hypertension	12	0.994 (0.946 to 1.045)	0.831	1.013 (0.983 to 1.043)	0.402	1.022 (0.998 to 1.046)	0.076		
4-imidazoleacetate	Hypertension	17	0.970 (0.903 to 1.042)	0.423	1.003 (0.979 to 1.027)	0.820	1.008 (0.983 to 1.034)	0.520	1.006 (0.987 to 1.026)	0.538
1-methylimidazoleacetate	Hypertension	23	1.016 (0.991 to 1.041)	0.229	1.013 (0.996 to 1.030)	0.126	1.011 (0.999 to 1.023)	0.071		
N-acetylneuraminate	Hypertension	17	0.953 (0.904 to 1.005)	0.096	0.976 (0.954 to 0.999)	0.040	0.973 (0.950 to 0.997)	0.025	0.981 (0.964 to 0.998)	0.043
dodecanedioate	Hypertension	31	0.985 (0.950 to 1.021)	0.404	0.996 (0.979 to 1.013)	0.616	1.007 (0.993 to 1.022)	0.308		

Exposure	Outcome	nSNP	MR Egger		Weighted median		Inverse variance weighted		MR_PRESSO	
			OR (95% CI)	pval	OR (95% CI)	pval	OR (95% CI)	pval	OR (95% CI)	pval
N-acetyltyrosine	Hypertension	33	1.028 (0.996 to 1.060)	0.095	1.020 (1.011 to 1.030)	2.83e-05	1.018 (1.009 to 1.026)	4.03e-05	1.020 (1.012 to 1.029)	4.78e-05
1,3-dimethylurate	Hypertension	23	1.010 (0.984 to 1.037)	0.478	1.000 (0.985 to 1.016)	0.974	0.993 (0.979 to 1.007)	0.340		
trigonelline (N'-methylnicotinate)	Hypertension	15	1.013 (0.973 to 1.055)	0.548	1.015 (0.992 to 1.039)	0.207	1.001 (0.977 to 1.026)	0.937	1.006 (0.989 to 1.024)	0.482
indolepropionate	Hypertension	21	0.976 (0.936 to 1.018)	0.275	1.006 (0.985 to 1.028)	0.559	1.013 (0.992 to 1.034)	0.221	1.008 (0.991 to 1.027)	0.370
butyrylcarnitine	Hypertension	67	0.995 (0.985 to 1.004)	0.298	0.994 (0.989 to 1.000)	0.045	1.000 (0.995 to 1.005)	0.996	1.000 (0.995 to 1.004)	0.850

Exposure	Outcome	nSNP	MR Egger		Weighted median		Inverse variance weighted		MR_PRESSO	
			OR (95% CI)	pval	OR (95% CI)	pval	OR (95% CI)	pval	OR (95% CI)	pval
myristoleate (14:1n5)	Hypertension	19	0.995 (0.948 to 1.043)	0.825	0.988 (0.963 to 1.013)	0.340	0.988 (0.965 to 1.011)	0.295		
dehydroisoandrosterone sulfate (DHEA-S)	Hypertension	22	0.982 (0.957 to 1.006)	0.161	0.986 (0.971 to 1.002)	0.097	0.984 (0.972 to 0.997)	0.014		
3-methylxanthine	Hypertension	13	0.988 (0.957 to 1.019)	0.447	0.989 (0.966 to 1.012)	0.339	0.998 (0.982 to 1.015)	0.845		
guanidinosuccinate	Hypertension	24	1.006 (0.987 to 1.025)	0.561	0.999 (0.987 to 1.010)	0.798	0.999 (0.988 to 1.009)	0.792	0.996 (0.986 to 1.005)	0.390
caproate (6:0)	Hypertension	26	1.010 (0.993 to 1.027)	0.283	1.006 (0.997 to 1.015)	0.207	1.002 (0.995 to 1.008)	0.608		

Exposure	Outcome	nSNP	MR Egger		Weighted median		Inverse variance weighted		MR_PRESSO	
			OR (95% CI)	pval	OR (95% CI)	pval	OR (95% CI)	pval	OR (95% CI)	pval
caprylate (8:0)	Hypertension	36	0.985 (0.967 to 1.003)	0.110	0.984 (0.970 to 0.998)	0.023	0.997 (0.986 to 1.009)	0.613		
10-undecenoate (11:1n1)	Hypertension	29	1.008 (0.989 to 1.027)	0.438	1.003 (0.989 to 1.017)	0.686	1.001 (0.991 to 1.011)	0.829		
docosapentaenoate (n3 DPA; 22:5n3)	Hypertension	42	0.998 (0.993 to 1.003)	0.532	0.997 (0.994 to 1.001)	0.183	0.998 (0.995 to 1.002)	0.339		
phenol sulfate	Hypertension	16	0.999 (0.975 to 1.023)	0.911	0.995 (0.977 to 1.013)	0.554	0.995 (0.981 to 1.010)	0.545		
pregnen-diol disulfate	Hypertension	23	0.998 (0.980 to 1.017)	0.873	1.006 (0.995 to 1.017)	0.300	1.003 (0.994 to 1.011)	0.539		

Exposure	Outcome	nSNP	MR Egger		Weighted median		Inverse variance weighted		MR_PRESSO	
			OR (95% CI)	pval	OR (95% CI)	pval	OR (95% CI)	pval	OR (95% CI)	pval
X - 11261	Hypertension	30	0.990 (0.960 to 1.022)	0.542	0.995 (0.977 to 1.013)	0.568	0.993 (0.981 to 1.005)	0.229		
bilirubin (E,E)	Hypertension	20	1.002 (0.990 to 1.015)	0.735	1.000 (0.991 to 1.010)	0.917	1.001 (0.994 to 1.008)	0.781		
glycocholenate sulfate	Hypertension	44	1.011 (0.988 to 1.036)	0.355	0.986 (0.974 to 0.997)	0.017	0.991 (0.982 to 1.000)	0.053	0.987 (0.978 to 0.996)	0.008
pregn steroid monosulfate	Hypertension	29	1.000 (0.962 to 1.040)	0.997	1.000 (0.984 to 1.017)	0.977	0.999 (0.986 to 1.012)	0.879		
glycolithocholate sulfate	Hypertension	21	0.995 (0.981 to 1.010)	0.509	0.991 (0.981 to 1.001)	0.064	0.998 (0.989 to 1.006)	0.551	0.992 (0.983 to 1.001)	0.105

Exposure	Outcome	nSNP	MR Egger		Weighted median		Inverse variance weighted		MR_PRESSO	
			OR (95% CI)	pval	OR (95% CI)	pval	OR (95% CI)	pval	OR (95% CI)	pval
X - 11381	Hypertension	33	0.992 (0.959 to 1.027)	0.659	1.007 (0.989 to 1.025)	0.426	1.002 (0.987 to 1.017)	0.823		
taurocholenate sulfate	Hypertension	35	1.012 (0.981 to 1.045)	0.449	1.019 (1.005 to 1.034)	0.008	1.000 (0.987 to 1.013)	0.979		
2-arachidonoyl-GPE (20:4)	Hypertension	53	1.007 (0.982 to 1.033)	0.603	0.989 (0.981 to 0.998)	0.014	0.992 (0.986 to 0.998)	0.008		
andro steroid monosulfate (1)	Hypertension	25	1.013 (0.994 to 1.033)	0.202	1.004 (0.990 to 1.018)	0.593	1.006 (0.995 to 1.016)	0.278		
HWESASXX	Hypertension	18	1.035 (1.006 to 1.066)	0.032	1.018 (0.997 to 1.040)	0.098	1.008 (0.993 to 1.024)	0.272		

Exposure	Outcome	nSNP	MR Egger		Weighted median		Inverse variance weighted		MR_PRESSO	
			OR (95% CI)	pval	OR (95% CI)	pval	OR (95% CI)	pval	OR (95% CI)	pval
X - 11540	Hypertension	20	0.993 (0.973 to 1.014)	0.528	1.005 (0.992 to 1.018)	0.470	1.000 (0.988 to 1.012)	0.975	1.005 (0.992 to 1.018)	0.462
adrenate (22:4n6)	Hypertension	40	1.010 (0.979 to 1.041)	0.540	0.982 (0.969 to 0.995)	0.007	0.987 (0.977 to 0.996)	0.008		
X - 11787	Hypertension	30	0.979 (0.937 to 1.022)	0.334	0.982 (0.971 to 0.994)	0.003	0.984 (0.971 to 0.997)	0.014	0.980 (0.968 to 0.992)	0.003
X - 11795	Hypertension	21	0.968 (0.918 to 1.020)	0.237	1.006 (0.977 to 1.035)	0.688	1.010 (0.984 to 1.037)	0.465		
1-arachidonoyl-GPC (20:4n6)	Hypertension	44	0.988 (0.976 to 1.000)	0.056	0.994 (0.990 to 0.999)	0.012	0.994 (0.991 to 0.998)	9.7e-04		

Exposure	Outcome	nSNP	MR Egger		Weighted median		Inverse variance weighted		MR_PRESSO	
			OR (95% CI)	pval	OR (95% CI)	pval	OR (95% CI)	pval	OR (95% CI)	pval
1-palmitoleoyl-GPC (16:1)	Hypertension	10	0.951 (0.855 to 1.057)	0.380	1.003 (0.962 to 1.045)	0.897	1.001 (0.957 to 1.048)	0.955	1.006 (0.968 to 1.046)	0.758
gamma-glutamylthreonine	Hypertension	18	0.985 (0.916 to 1.059)	0.691	0.996 (0.970 to 1.023)	0.762	1.004 (0.979 to 1.029)	0.756	0.995 (0.974 to 1.017)	0.672
2-arachidonoyl-GPC (20:4)	Hypertension	50	0.985 (0.973 to 0.998)	0.032	0.994 (0.989 to 0.999)	0.015	0.994 (0.991 to 0.998)	0.003		
gamma-glutamylphenylalanine	Hypertension	8	0.997 (0.941 to 1.056)	0.921	1.002 (0.972 to 1.033)	0.902	1.004 (0.978 to 1.030)	0.773		
isobutyrylcarnitine	Hypertension	28	0.989 (0.961 to 1.018)	0.473	0.981 (0.966 to 0.996)	0.013	0.995 (0.980 to 1.010)	0.488	0.995 (0.983 to 1.007)	0.439

Exposure	Outcome	nSNP	MR Egger		Weighted median		Inverse variance weighted		MR_PRESSO	
			OR (95% CI)	pval	OR (95% CI)	pval	OR (95% CI)	pval	OR (95% CI)	pval
pseudouridine	Hypertension	24	0.997 (0.957 to 1.038)	0.875	1.014 (0.993 to 1.036)	0.199	1.013 (0.995 to 1.031)	0.157		
palmitoleate (16:1n7)	Hypertension	29	0.995 (0.981 to 1.009)	0.511	1.001 (0.992 to 1.009)	0.879	0.998 (0.990 to 1.007)	0.659		
1-dihomo-linolenoyl-GPC (20:3n3 or 6)	Hypertension	41	1.056 (0.998 to 1.116)	0.064	1.017 (1.006 to 1.029)	0.003	1.023 (1.015 to 1.031)	5.32e-08		
1-docosahexaenoyl-GPC (22:6)	Hypertension	22	0.975 (0.949 to 1.002)	0.089	1.005 (0.986 to 1.025)	0.592	0.999 (0.985 to 1.012)	0.863		
1-dihomo-linoleoyl-GPC (20:2)	Hypertension	46	1.007 (0.988 to 1.026)	0.486	1.008 (1.002 to 1.015)	0.013	1.008 (1.003 to 1.013)	0.001		

Exposure	Outcome	nSNP	MR Egger		Weighted median		Inverse variance weighted		MR_PRESSO	
			OR (95% CI)	pval	OR (95% CI)	pval	OR (95% CI)	pval	OR (95% CI)	pval
gamma-glutamyl-epsilon-lysine	Hypertension	23	0.980 (0.956 to 1.005)	0.132	0.989 (0.973 to 1.006)	0.211	0.992 (0.977 to 1.008)	0.334	0.989 (0.976 to 1.002)	0.112
octanoylcarnitine	Hypertension	26	1.035 (1.000 to 1.071)	0.061	1.005 (0.991 to 1.019)	0.490	1.002 (0.991 to 1.014)	0.717	1.005 (0.994 to 1.015)	0.408
decanoylcarnitine	Hypertension	24	1.018 (0.960 to 1.080)	0.557	1.001 (0.984 to 1.018)	0.935	1.000 (0.986 to 1.015)	0.972		
N-acetylasparagine	Hypertension	23	0.963 (0.938 to 0.989)	0.011	0.991 (0.972 to 1.011)	0.399	1.007 (0.987 to 1.027)	0.511	1.021 (0.995 to 1.048)	0.129
N-acetylglutamine	Hypertension	26	1.015 (0.990 to 1.041)	0.244	1.018 (1.010 to 1.026)	1.24e-05	1.016 (1.007 to 1.024)	4.86e-04	1.022 (1.014 to 1.030)	8.23e-06

Exposure	Outcome	nSNP	MR Egger		Weighted median		Inverse variance weighted		MR_PRESSO	
			OR (95% CI)	pval	OR (95% CI)	pval	OR (95% CI)	pval	OR (95% CI)	pval
N-acetylhistidine	Hypertension	35	1.048 (1.021 to 1.076)	0.001	1.020 (1.005 to 1.036)	0.010	1.015 (1.002 to 1.029)	0.022	1.016 (1.004 to 1.029)	0.017
gamma-glutamylglycine	Hypertension	28	1.016 (1.002 to 1.030)	0.030	1.008 (0.997 to 1.019)	0.176	1.005 (0.996 to 1.014)	0.262	1.006 (0.998 to 1.014)	0.154
N-acetylphenylalanine	Hypertension	24	1.042 (1.024 to 1.061)	1.77e-04	1.034 (1.025 to 1.043)	4.32e-13	1.029 (1.021 to 1.037)	6.1e-12	1.031 (1.025 to 1.038)	3.51e-09
myristoylcarnitine	Hypertension	31	1.011 (0.953 to 1.073)	0.713	1.034 (1.014 to 1.055)	9.85e-04	1.026 (1.009 to 1.043)	0.003		
glycylphenylalanine	Hypertension	32	0.962 (0.937 to 0.988)	0.008	1.000 (0.984 to 1.016)	0.990	1.003 (0.993 to 1.013)	0.564		

Exposure	Outcome	nSNP	MR Egger		Weighted median		Inverse variance weighted		MR_PRESSO	
			OR (95% CI)	pval	OR (95% CI)	pval	OR (95% CI)	pval	OR (95% CI)	pval
1-stearoyl-GPC (18:0)	Hypertension	20	1.007 (0.996 to 1.017)	0.234	1.008 (0.999 to 1.018)	0.081	1.006 (1.000 to 1.013)	0.065		
5-dodecenoate (12:1n7)	Hypertension	16	0.987 (0.943 to 1.032)	0.565	0.992 (0.967 to 1.019)	0.569	0.996 (0.971 to 1.020)	0.728		
10-nonadecenoate (19:1n9)	Hypertension	9	0.997 (0.982 to 1.011)	0.651	0.999 (0.989 to 1.009)	0.856	0.998 (0.991 to 1.006)	0.682		
epiandrosterone sulfate	Hypertension	25	0.990 (0.961 to 1.021)	0.539	1.002 (0.987 to 1.018)	0.805	1.000 (0.987 to 1.014)	0.975	0.997 (0.984 to 1.011)	0.715
1-arachidonoyl-GPI (20:4)	Hypertension	49	1.030 (0.991 to 1.072)	0.139	0.984 (0.972 to 0.997)	0.014	0.993 (0.984 to 1.003)	0.166		

Exposure	Outcome	nSNP	MR Egger		Weighted median		Inverse variance weighted		MR_PRESSO	
			OR (95% CI)	pval	OR (95% CI)	pval	OR (95% CI)	pval	OR (95% CI)	pval
2-docosahexaenoyl-GPE (22:6)	Hypertension	20	0.923 (0.870 to 0.979)	0.016	1.001 (0.978 to 1.025)	0.918	0.995 (0.975 to 1.015)	0.606	1.010 (0.994 to 1.026)	0.235
stachydrine	Hypertension	20	1.004 (0.972 to 1.038)	0.800	1.006 (0.982 to 1.030)	0.654	1.005 (0.987 to 1.023)	0.587		
1-methylxanthine	Hypertension	20	0.990 (0.968 to 1.013)	0.422	1.002 (0.987 to 1.016)	0.835	1.005 (0.993 to 1.016)	0.431		
7-methylxanthine	Hypertension	12	0.998 (0.936 to 1.064)	0.958	0.992 (0.965 to 1.020)	0.573	0.984 (0.957 to 1.012)	0.259	0.997 (0.976 to 1.018)	0.793
1-linolenoylglycerol (18:3)	Hypertension	16	1.041 (0.999 to 1.084)	0.074	1.026 (1.003 to 1.050)	0.026	1.021 (0.999 to 1.043)	0.061	1.029 (1.013 to 1.045)	0.003

Exposure	Outcome	nSNP	MR Egger		Weighted median		Inverse variance weighted		MR_PRESSO	
			OR (95% CI)	pval	OR (95% CI)	pval	OR (95% CI)	pval	OR (95% CI)	pval
1-methylurate	Hypertension	22	0.996 (0.984 to 1.008)	0.509	0.998 (0.989 to 1.008)	0.726	0.996 (0.989 to 1.002)	0.203		
choline phosphate	Hypertension	20	1.006 (0.975 to 1.038)	0.715	1.011 (0.992 to 1.030)	0.246	1.005 (0.987 to 1.024)	0.578	1.011 (0.996 to 1.026)	0.177
1,7-dimethylurate	Hypertension	28	0.990 (0.966 to 1.014)	0.415	0.986 (0.970 to 1.001)	0.073	0.995 (0.981 to 1.009)	0.464	0.989 (0.978 to 0.999)	0.043
5-acetylamino-6-formylamino-3-methyluracil	Hypertension	22	0.998 (0.955 to 1.042)	0.920	0.995 (0.982 to 1.008)	0.451	0.997 (0.983 to 1.011)	0.668	0.993 (0.981 to 1.006)	0.295
1,3,7-trimethylurate	Hypertension	20	0.978 (0.934 to 1.025)	0.364	0.987 (0.964 to 1.010)	0.255	0.987 (0.963 to 1.011)	0.281	0.976 (0.958 to 0.994)	0.018

Exposure	Outcome	nSNP	MR Egger		Weighted median		Inverse variance weighted		MR_PRESSO	
			OR (95% CI)	pval	OR (95% CI)	pval	OR (95% CI)	pval	OR (95% CI)	pval
isovalerylcarnitine	Hypertension	26	0.965 (0.902 to 1.032)	0.310	1.036 (1.014 to 1.058)	0.001	1.021 (1.002 to 1.040)	0.028	1.024 (1.006 to 1.042)	0.014
stearoylcarnitine	Hypertension	22	0.977 (0.934 to 1.022)	0.321	1.006 (0.982 to 1.030)	0.614	1.017 (0.996 to 1.039)	0.117	1.011 (0.992 to 1.031)	0.271
1-linoleoyl-GPC (18:2)	Hypertension	37	0.978 (0.951 to 1.006)	0.126	1.013 (1.002 to 1.023)	0.016	1.011 (1.004 to 1.019)	0.004		
5-acetylamino-6-amino-3-methyluracil	Hypertension	22	1.002 (0.974 to 1.031)	0.894	1.007 (0.990 to 1.023)	0.427	1.007 (0.993 to 1.022)	0.327	1.006 (0.993 to 1.018)	0.371
1-palmitoyl-GPA (16:0)	Hypertension	19	1.040 (1.005 to 1.075)	0.039	1.016 (0.997 to 1.036)	0.099	1.017 (1.002 to 1.032)	0.028		

Exposure	Outcome	nSNP	MR Egger		Weighted median		Inverse variance weighted		MR_PRESSO	
			OR (95% CI)	pval	OR (95% CI)	pval	OR (95% CI)	pval	OR (95% CI)	pval
gamma-glutamylisoleucine	Hypertension	27	0.989 (0.970 to 1.010)	0.310	0.997 (0.980 to 1.013)	0.691	0.990 (0.978 to 1.001)	0.078		
laurylcarnitine	Hypertension	20	0.969 (0.918 to 1.023)	0.267	0.987 (0.966 to 1.007)	0.201	0.983 (0.965 to 1.000)	0.051	0.979 (0.964 to 0.994)	0.013
7-methylguanine	Hypertension	23	0.994 (0.960 to 1.030)	0.746	1.003 (0.981 to 1.025)	0.798	1.018 (1.001 to 1.036)	0.043	1.010 (0.993 to 1.028)	0.252
phenylacetylglutamine	Hypertension	11	1.009 (0.979 to 1.041)	0.578	1.000 (0.987 to 1.013)	0.961	0.998 (0.976 to 1.021)	0.891	1.000 (0.986 to 1.015)	0.954
pro-hydroxy-pro	Hypertension	22	0.967 (0.923 to 1.013)	0.175	0.992 (0.969 to 1.016)	0.520	0.988 (0.967 to 1.009)	0.253	0.977 (0.959 to 0.995)	0.023

Exposure	Outcome	nSNP	MR Egger		Weighted median		Inverse variance weighted		MR_PRESSO	
			OR (95% CI)	pval	OR (95% CI)	pval	OR (95% CI)	pval	OR (95% CI)	pval
5-methyluridine (ribothymidine)	Hypertension	24	0.998 (0.978 to 1.018)	0.840	1.007 (0.993 to 1.021)	0.319	1.006 (0.996 to 1.016)	0.248		
N2,N2-dimethylguanosine	Hypertension	11	0.975 (0.902 to 1.054)	0.540	0.991 (0.960 to 1.023)	0.584	0.985 (0.959 to 1.011)	0.251		
1-docosahexaenoylglycerol (22:6)	Hypertension	16	1.005 (0.951 to 1.061)	0.868	1.008 (0.983 to 1.034)	0.549	1.007 (0.981 to 1.032)	0.612	1.023 (1.002 to 1.044)	0.050
N6-carbamoylthreonyladenosine	Hypertension	15	0.963 (0.880 to 1.054)	0.430	0.997 (0.970 to 1.026)	0.841	1.015 (0.976 to 1.055)	0.465	0.992 (0.972 to 1.012)	0.451
oleoylcarnitine	Hypertension	26	1.009 (0.989 to 1.030)	0.377	1.014 (1.000 to 1.029)	0.058	1.004 (0.991 to 1.018)	0.562	0.996 (0.979 to 1.013)	0.663

Exposure	Outcome	nSNP	MR Egger		Weighted median		Inverse variance weighted		MR_PRESSO	
			OR (95% CI)	pval	OR (95% CI)	pval	OR (95% CI)	pval	OR (95% CI)	pval
orotidine	Hypertension	28	1.007 (0.993 to 1.021)	0.345	1.003 (0.989 to 1.017)	0.691	1.007 (0.998 to 1.017)	0.141		
1-arachidonoyl-GPE (20:4n6)	Hypertension	75	1.001 (0.993 to 1.008)	0.865	1.003 (0.998 to 1.009)	0.227	1.002 (0.998 to 1.006)	0.271		
2-palmitoyl-GPC (16:0)	Hypertension	24	1.000 (0.973 to 1.027)	0.984	1.001 (0.986 to 1.017)	0.869	0.999 (0.987 to 1.011)	0.856		
2-linoleoyl-GPC (18:2)	Hypertension	15	1.028 (0.968 to 1.092)	0.381	0.987 (0.960 to 1.015)	0.358	0.987 (0.968 to 1.007)	0.211		
tiglylcarnitine	Hypertension	25	0.980 (0.947 to 1.015)	0.265	0.991 (0.972 to 1.011)	0.389	1.001 (0.986 to 1.017)	0.851		

Exposure	Outcome	nSNP	MR Egger		Weighted median		Inverse variance weighted		MR_PRESSO	
			OR (95% CI)	pval	OR (95% CI)	pval	OR (95% CI)	pval	OR (95% CI)	pval
hexanoylglycine	Hypertension	25	1.005 (0.987 to 1.024)	0.574	1.005 (0.995 to 1.015)	0.316	1.006 (0.995 to 1.018)	0.293	1.005 (0.994 to 1.016)	0.358
2-methylmalonyl carnitine	Hypertension	16	1.004 (0.929 to 1.084)	0.925	0.985 (0.956 to 1.015)	0.337	0.983 (0.956 to 1.010)	0.211	0.977 (0.952 to 1.002)	0.087
1-myristoylglycerol (14:0)	Hypertension	26	1.003 (0.976 to 1.030)	0.847	0.995 (0.977 to 1.014)	0.634	0.999 (0.987 to 1.012)	0.931		
1-oleoyl-GPE (18:1)	Hypertension	39	0.985 (0.957 to 1.013)	0.285	1.009 (0.992 to 1.025)	0.303	1.012 (1.001 to 1.024)	0.041		
1-palmitoyl-GPE (16:0)	Hypertension	41	0.987 (0.971 to 1.005)	0.159	1.002 (0.991 to 1.014)	0.679	1.001 (0.994 to 1.009)	0.716		

Exposure	Outcome	nSNP	MR Egger		Weighted median		Inverse variance weighted		MR_PRESSO	
			OR (95% CI)	pval	OR (95% CI)	pval	OR (95% CI)	pval	OR (95% CI)	pval
cysteinylglycine	Hypertension	20	0.985 (0.932 to 1.041)	0.599	1.008 (0.985 to 1.031)	0.514	1.023 (0.996 to 1.051)	0.092	1.006 (0.988 to 1.023)	0.542
tetradecanedioate	Hypertension	56	0.984 (0.975 to 0.994)	0.002	0.997 (0.988 to 1.006)	0.504	0.996 (0.990 to 1.003)	0.254	1.006 (0.999 to 1.014)	0.104
2-hydroxypalmitate	Hypertension	21	1.036 (0.996 to 1.077)	0.094	0.999 (0.977 to 1.022)	0.941	1.004 (0.988 to 1.021)	0.618		
hexadecanedioate	Hypertension	36	1.020 (0.985 to 1.055)	0.281	1.003 (0.990 to 1.016)	0.683	0.999 (0.989 to 1.010)	0.866		
dihomo-linolenate (20:3n3 or n6)	Hypertension	51	1.024 (0.990 to 1.059)	0.176	1.018 (1.005 to 1.030)	0.005	1.019 (1.010 to 1.028)	1.54e-05		

Exposure	Outcome	nSNP	MR Egger		Weighted median		Inverse variance weighted		MR_PRESSO	
			OR (95% CI)	pval	OR (95% CI)	pval	OR (95% CI)	pval	OR (95% CI)	pval
4-vinylphenol sulfate	Hypertension	23	1.046 (1.003 to 1.092)	0.050	1.015 (0.994 to 1.038)	0.168	1.006 (0.987 to 1.025)	0.530	1.012 (0.997 to 1.027)	0.143
p-cresol sulfate	Hypertension	14	1.025 (0.995 to 1.057)	0.124	1.011 (0.984 to 1.038)	0.429	1.021 (1.003 to 1.039)	0.023		
2-linoleoyl-GPE (18:2)	Hypertension	51	1.027 (0.993 to 1.062)	0.125	1.012 (1.003 to 1.022)	0.010	1.008 (1.001 to 1.015)	0.018		
1-linoleoyl-GPI (18:2)	Hypertension	46	1.012 (0.970 to 1.055)	0.599	1.014 (1.002 to 1.025)	0.018	1.006 (0.998 to 1.014)	0.120		
1-linoleoyl-GPE (18:2)	Hypertension	53	1.026 (1.005 to 1.046)	0.016	1.009 (1.002 to 1.016)	0.012	1.006 (1.001 to 1.011)	0.028		

Exposure	Outcome	nSNP	MR Egger		Weighted median		Inverse variance weighted		MR_PRESSO	
			OR (95% CI)	pval	OR (95% CI)	pval	OR (95% CI)	pval	OR (95% CI)	pval
1-oleoyl-GPI (18:1)	Hypertension	23	1.000 (0.950 to 1.053)	0.990	1.013 (0.991 to 1.035)	0.247	0.995 (0.976 to 1.014)	0.579		
gamma-glutamylglutamate	Hypertension	34	0.999 (0.989 to 1.009)	0.828	0.999 (0.994 to 1.004)	0.687	0.996 (0.992 to 1.001)	0.087		
2-hydroxy-3-methylvalerate	Hypertension	27	1.007 (0.980 to 1.034)	0.635	1.010 (0.992 to 1.030)	0.284	1.002 (0.986 to 1.017)	0.846		
deoxycarnitine	Hypertension	27	1.031 (0.997 to 1.066)	0.085	1.005 (0.986 to 1.023)	0.621	0.990 (0.976 to 1.005)	0.196	0.994 (0.981 to 1.008)	0.394
3-methyladipate	Hypertension	16	0.982 (0.927 to 1.040)	0.546	1.040 (1.015 to 1.067)	0.002	1.023 (1.000 to 1.046)	0.049		

Exposure	Outcome	nSNP	MR Egger		Weighted median		Inverse variance weighted		MR_PRESSO	
			OR (95% CI)	pval	OR (95% CI)	pval	OR (95% CI)	pval	OR (95% CI)	pval
N6-acetyllysine	Hypertension	16	0.997 (0.958 to 1.037)	0.874	0.996 (0.973 to 1.019)	0.740	0.993 (0.977 to 1.010)	0.440		
octadecanedioate	Hypertension	19	0.959 (0.911 to 1.009)	0.125	0.975 (0.953 to 0.999)	0.038	0.991 (0.966 to 1.017)	0.513	0.986 (0.962 to 1.010)	0.263
7-alpha-hydroxy-3-oxo-4-cholestenoate (7-Hoca)	Hypertension	15	0.988 (0.944 to 1.033)	0.601	1.018 (0.990 to 1.047)	0.202	1.008 (0.983 to 1.034)	0.541	1.018 (0.996 to 1.040)	0.135
dimethylarginine (SDMA + ADMA)	Hypertension	16	0.996 (0.918 to 1.081)	0.927	0.982 (0.959 to 1.005)	0.132	0.988 (0.957 to 1.020)	0.455	0.985 (0.964 to 1.007)	0.203
o-cresol sulfate	Hypertension	13	1.023 (0.972 to 1.077)	0.398	1.014 (0.989 to 1.039)	0.274	0.998 (0.979 to 1.018)	0.848		

Exposure	Outcome	nSNP	MR Egger		Weighted median		Inverse variance weighted		MR_PRESSO	
			OR (95% CI)	pval	OR (95% CI)	pval	OR (95% CI)	pval	OR (95% CI)	pval
taurolithocholate 3-sulfate	Hypertension	16	0.998 (0.951 to 1.046)	0.928	1.003 (0.980 to 1.027)	0.770	1.014 (0.993 to 1.036)	0.200		
succinylcarnitine	Hypertension	23	1.007 (0.974 to 1.042)	0.676	0.980 (0.963 to 0.999)	0.035	0.975 (0.958 to 0.992)	0.004	0.973 (0.958 to 0.989)	0.003
gamma-glutamylalanine	Hypertension	19	0.964 (0.882 to 1.055)	0.439	1.036 (1.009 to 1.064)	0.008	1.017 (0.983 to 1.051)	0.332	1.026 (1.001 to 1.052)	0.060
N-acetylserine	Hypertension	21	1.017 (0.960 to 1.076)	0.573	0.992 (0.971 to 1.013)	0.458	1.008 (0.992 to 1.025)	0.325		
gamma-glutamyl-2-aminobutyrate	Hypertension	14	1.021 (0.970 to 1.074)	0.449	0.981 (0.953 to 1.009)	0.180	0.995 (0.971 to 1.019)	0.663		

Exposure	Outcome	nSNP	MR Egger		Weighted median		Inverse variance weighted		MR_PRESSO	
			OR (95% CI)	pval	OR (95% CI)	pval	OR (95% CI)	pval	OR (95% CI)	pval
tryptophan betaine	Hypertension	12	0.993 (0.944 to 1.045)	0.790	1.004 (0.977 to 1.031)	0.777	0.999 (0.978 to 1.021)	0.944		
cyclo(leu-pro)	Hypertension	20	1.043 (0.986 to 1.104)	0.159	1.023 (1.000 to 1.047)	0.052	1.014 (0.991 to 1.039)	0.241	1.019 (1.000 to 1.040)	0.070
5alpha-androstan-3alpha,17beta-diol monosulfate (1)	Hypertension	23	0.981 (0.964 to 0.998)	0.039	0.991 (0.979 to 1.003)	0.156	0.999 (0.988 to 1.011)	0.884		
5alpha-androstan-3beta,17beta-diol disulfate	Hypertension	29	1.001 (0.988 to 1.015)	0.883	1.003 (0.995 to 1.011)	0.493	0.996 (0.989 to 1.003)	0.248	0.998 (0.990 to 1.006)	0.645
5alpha-pregnan-3beta,20alpha-diol disulfate	Hypertension	21	1.028 (0.984 to 1.073)	0.226	1.011 (0.990 to 1.032)	0.322	1.014 (0.998 to 1.031)	0.085		

Exposure	Outcome	nSNP	MR Egger		Weighted median		Inverse variance weighted		MR_PRESSO	
			OR (95% CI)	pval	OR (95% CI)	pval	OR (95% CI)	pval	OR (95% CI)	pval
5alpha-pregnan-3beta,20alpha-diol monosulfate (2)	Hypertension	11	0.973 (0.942 to 1.004)	0.122	0.994 (0.970 to 1.019)	0.619	0.997 (0.980 to 1.015)	0.775		
4-androsten-3beta,17beta-diol disulfate (1)	Hypertension	26	0.981 (0.955 to 1.008)	0.187	1.007 (0.994 to 1.021)	0.270	1.004 (0.991 to 1.017)	0.546	1.002 (0.991 to 1.012)	0.775
4-androsten-3beta,17beta-diol disulfate (2)	Hypertension	21	1.020 (1.002 to 1.038)	0.043	1.004 (0.994 to 1.015)	0.439	1.006 (0.997 to 1.016)	0.170		
4-androsten-3alpha,17alpha-diol monosulfate (2)	Hypertension	24	0.989 (0.934 to 1.048)	0.716	0.982 (0.959 to 1.005)	0.117	0.989 (0.968 to 1.010)	0.306	0.978 (0.966 to 0.991)	0.003
4-androsten-3alpha,17alpha-diol monosulfate (3)	Hypertension	41	1.008 (0.998 to 1.018)	0.116	1.011 (1.003 to 1.019)	0.007	1.009 (1.004 to 1.015)	0.001		

Exposure	Outcome	nSNP	MR Egger		Weighted median		Inverse variance weighted		MR_PRESSO	
			OR (95% CI)	pval	OR (95% CI)	pval	OR (95% CI)	pval	OR (95% CI)	pval
4-androsten-3beta,17beta-diol monosulfate (2)	Hypertension	27	0.981 (0.943 to 1.020)	0.340	0.979 (0.963 to 0.996)	0.016	0.977 (0.962 to 0.992)	0.003		
4-androsten-3beta,17beta-diol monosulfate (1)	Hypertension	20	0.983 (0.959 to 1.007)	0.172	0.989 (0.972 to 1.007)	0.250	0.989 (0.976 to 1.002)	0.089		
1-docosapentaenoyl-GPC (22:5n3)	Hypertension	45	0.987 (0.960 to 1.015)	0.369	0.984 (0.974 to 0.995)	0.005	0.981 (0.973 to 0.989)	4.14e-06		
1-pentadecanoyl-GPC (15:0)	Hypertension	14	0.962 (0.922 to 1.004)	0.100	1.011 (0.985 to 1.038)	0.418	1.008 (0.990 to 1.027)	0.379		
N-methylproline	Hypertension	16	1.096 (1.033 to 1.163)	0.009	1.009 (0.985 to 1.033)	0.466	0.998 (0.972 to 1.025)	0.883	1.006 (0.986 to 1.027)	0.553

Exposure	Outcome	nSNP	MR Egger		Weighted median		Inverse variance weighted		MR_PRESSO	
			OR (95% CI)	pval	OR (95% CI)	pval	OR (95% CI)	pval	OR (95% CI)	pval
N-acetyl-beta-alanine	Hypertension	20	1.009 (0.967 to 1.052)	0.700	1.017 (0.996 to 1.037)	0.110	1.010 (0.992 to 1.028)	0.288	1.013 (0.999 to 1.028)	0.085
cysteine sulfinic acid	Hypertension	15	0.991 (0.940 to 1.045)	0.747	1.010 (0.981 to 1.039)	0.504	1.008 (0.981 to 1.035)	0.577	1.016 (0.996 to 1.037)	0.139
ergothioneine	Hypertension	16	1.015 (0.988 to 1.042)	0.304	1.012 (0.988 to 1.035)	0.331	1.008 (0.992 to 1.025)	0.315		
N-acetylputrescine	Hypertension	20	1.011 (0.987 to 1.036)	0.375	1.003 (0.988 to 1.018)	0.696	1.001 (0.990 to 1.012)	0.897		
palmitoyl sphingomyelin (d18:1/16:0)	Hypertension	10	0.999 (0.942 to 1.059)	0.970	0.993 (0.965 to 1.022)	0.639	1.003 (0.979 to 1.029)	0.783		

Exposure	Outcome	nSNP	MR Egger		Weighted median		Inverse variance weighted		MR_PRESSO	
			OR (95% CI)	pval	OR (95% CI)	pval	OR (95% CI)	pval	OR (95% CI)	pval
sphingomyelin (d18:1/18:1, d18:2/18:0)	Hypertension	45	1.022 (0.986 to 1.058)	0.236	0.981 (0.967 to 0.995)	0.008	0.989 (0.978 to 1.000)	0.051		
13-HODE + 9-HODE	Hypertension	41	1.017 (0.995 to 1.040)	0.148	1.016 (1.003 to 1.029)	0.018	1.014 (1.003 to 1.026)	0.016	1.015 (1.007 to 1.024)	0.001
oleoyl ethanolamide	Hypertension	20	1.010 (0.960 to 1.062)	0.711	0.995 (0.973 to 1.018)	0.688	0.980 (0.959 to 1.002)	0.071	0.981 (0.964 to 1.000)	0.061
S-methylmethionine	Hypertension	18	0.986 (0.963 to 1.010)	0.277	0.995 (0.977 to 1.013)	0.580	0.986 (0.972 to 1.001)	0.071	0.975 (0.963 to 0.988)	0.002
phenylalanylphenylalanine	Hypertension	18	1.005 (0.962 to 1.050)	0.821	1.000 (0.976 to 1.023)	0.967	1.001 (0.980 to 1.022)	0.942		

Exposure	Outcome	nSNP	MR Egger		Weighted median		Inverse variance weighted		MR_PRESSO	
			OR (95% CI)	pval	OR (95% CI)	pval	OR (95% CI)	pval	OR (95% CI)	pval
16a-hydroxy DHEA 3-sulfate	Hypertension	29	1.015 (1.000 to 1.030)	0.059	1.011 (1.000 to 1.021)	0.050	1.003 (0.993 to 1.013)	0.568	1.004 (0.995 to 1.014)	0.391
pregnenolone sulfate	Hypertension	28	0.994 (0.981 to 1.008)	0.413	0.993 (0.984 to 1.003)	0.166	0.998 (0.990 to 1.006)	0.623	0.994 (0.986 to 1.002)	0.169
cis-4-decenoyl carnitine	Hypertension	32	1.023 (0.984 to 1.063)	0.259	1.010 (0.995 to 1.026)	0.192	1.004 (0.990 to 1.017)	0.597		
17-methylstearate	Hypertension	19	0.993 (0.958 to 1.028)	0.690	1.022 (0.998 to 1.047)	0.078	1.018 (0.999 to 1.038)	0.069		
cinnamoylglycine	Hypertension	33	1.002 (0.987 to 1.017)	0.776	0.999 (0.990 to 1.009)	0.897	1.002 (0.993 to 1.011)	0.737	1.000 (0.992 to 1.008)	0.985

Exposure	Outcome	nSNP	MR Egger		Weighted median		Inverse variance weighted		MR_PRESSO	
			OR (95% CI)	pval	OR (95% CI)	pval	OR (95% CI)	pval	OR (95% CI)	pval
3-methylglutaconate	Hypertension	23	0.974 (0.941 to 1.007)	0.139	0.969 (0.952 to 0.986)	3e-04	0.988 (0.972 to 1.003)	0.115	0.995 (0.979 to 1.012)	0.568
15-methylpalmitate	Hypertension	10	0.992 (0.896 to 1.099)	0.881	0.994 (0.963 to 1.026)	0.716	0.997 (0.957 to 1.040)	0.896	0.996 (0.977 to 1.016)	0.719
2-stearoyl-GPI (18:0)	Hypertension	29	1.017 (0.985 to 1.050)	0.309	0.998 (0.980 to 1.016)	0.797	0.993 (0.978 to 1.009)	0.392		
glycoursodeoxycholate	Hypertension	9	1.086 (1.027 to 1.149)	0.023	1.030 (0.998 to 1.063)	0.063	1.041 (1.018 to 1.064)	3.64e-04		
S-methylcysteine	Hypertension	15	1.004 (0.978 to 1.031)	0.770	0.999 (0.982 to 1.015)	0.872	0.997 (0.983 to 1.010)	0.614		

Exposure	Outcome	nSNP	MR Egger		Weighted median		Inverse variance weighted		MR_PRESSO	
			OR (95% CI)	pval	OR (95% CI)	pval	OR (95% CI)	pval	OR (95% CI)	pval
leucylalanine	Hypertension	31	1.053 (1.034 to 1.073)	6.74e-06	1.006 (0.992 to 1.020)	0.427	1.025 (1.011 to 1.039)	4.23e-04	1.020 (1.010 to 1.030)	6.72e-04
phenylalanylserine	Hypertension	26	1.028 (0.979 to 1.078)	0.279	1.018 (1.006 to 1.031)	0.005	1.006 (0.989 to 1.023)	0.512	1.018 (1.008 to 1.028)	0.002
alpha-glutamyltyrosine	Hypertension	28	1.002 (0.934 to 1.075)	0.952	1.016 (1.001 to 1.031)	0.032	0.972 (0.946 to 1.000)	0.050	1.000 (0.985 to 1.014)	0.970
isoleucylvaline	Hypertension	17	0.989 (0.948 to 1.031)	0.600	0.993 (0.973 to 1.013)	0.473	0.987 (0.971 to 1.004)	0.141		
X - 17299	Hypertension	20	1.006 (0.964 to 1.050)	0.797	1.000 (0.977 to 1.024)	0.972	1.001 (0.983 to 1.020)	0.897		

Exposure	Outcome	nSNP	MR Egger		Weighted median		Inverse variance weighted		MR_PRESSO	
			OR (95% CI)	pval	OR (95% CI)	pval	OR (95% CI)	pval	OR (95% CI)	pval
N1-Methyl-2-pyridone-5-carboxamide	Hypertension	12	0.982 (0.929 to 1.038)	0.535	1.001 (0.970 to 1.033)	0.955	1.010 (0.986 to 1.035)	0.410		
hydantoin-5-propionic acid	Hypertension	20	1.007 (0.993 to 1.022)	0.324	1.005 (0.994 to 1.015)	0.364	1.003 (0.995 to 1.012)	0.441		
dihydroferulic acid	Hypertension	12	1.048 (1.005 to 1.093)	0.051	1.012 (0.988 to 1.037)	0.335	1.004 (0.984 to 1.024)	0.720		
pregnanediol-3-glucuronide	Hypertension	16	0.969 (0.940 to 0.999)	0.065	0.991 (0.969 to 1.015)	0.463	1.000 (0.981 to 1.018)	0.967		
imidazole propionate	Hypertension	16	0.990 (0.965 to 1.016)	0.463	0.993 (0.975 to 1.011)	0.434	0.996 (0.981 to 1.012)	0.647		

Exposure	Outcome	nSNP	MR Egger		Weighted median		Inverse variance weighted		MR_PRESSO	
			OR (95% CI)	pval	OR (95% CI)	pval	OR (95% CI)	pval	OR (95% CI)	pval
prolylproline	Hypertension	16	0.974 (0.952 to 0.996)	0.039	0.985 (0.970 to 1.000)	0.048	0.994 (0.983 to 1.005)	0.278		
phenylalanyltryptophan	Hypertension	20	1.025 (0.993 to 1.058)	0.148	1.012 (0.991 to 1.033)	0.283	1.023 (1.008 to 1.039)	0.003		
alpha-glutamylglycine	Hypertension	35	1.007 (0.965 to 1.050)	0.756	1.015 (1.001 to 1.029)	0.032	1.006 (0.991 to 1.021)	0.449	1.012 (1.003 to 1.022)	0.012
histidyltryptophan	Hypertension	17	1.016 (0.979 to 1.054)	0.423	0.999 (0.974 to 1.023)	0.908	0.998 (0.979 to 1.018)	0.849		
serylalanine	Hypertension	28	0.954 (0.902 to 1.010)	0.116	1.012 (0.992 to 1.033)	0.246	0.993 (0.969 to 1.019)	0.610	1.008 (0.992 to 1.025)	0.329

Exposure	Outcome	nSNP	MR Egger		Weighted median		Inverse variance weighted		MR_PRESSO	
			OR (95% CI)	pval	OR (95% CI)	pval	OR (95% CI)	pval	OR (95% CI)	pval
valylarginine	Hypertension	31	1.024 (0.956 to 1.097)	0.500	1.021 (1.002 to 1.040)	0.031	1.036 (1.005 to 1.069)	0.024	1.009 (0.996 to 1.022)	0.203
indoleacetylglutamine	Hypertension	23	0.987 (0.965 to 1.010)	0.275	0.983 (0.968 to 0.999)	0.036	0.986 (0.976 to 0.998)	0.017		
N-palmitoylglycine	Hypertension	30	1.044 (0.992 to 1.099)	0.112	0.995 (0.975 to 1.015)	0.604	1.006 (0.990 to 1.022)	0.473	1.000 (0.985 to 1.015)	0.975
2-aminobutyrate	Hypertension	15	1.033 (0.971 to 1.098)	0.326	1.007 (0.982 to 1.033)	0.568	1.016 (0.997 to 1.036)	0.103		
1-stearoyl-GPE (18:0)	Hypertension	33	1.003 (0.975 to 1.033)	0.826	1.002 (0.991 to 1.014)	0.676	1.002 (0.994 to 1.011)	0.567		

Exposure	Outcome	nSNP	MR Egger		Weighted median		Inverse variance weighted		MR_PRESSO	
			OR (95% CI)	pval	OR (95% CI)	pval	OR (95% CI)	pval	OR (95% CI)	pval
erythronate	Hypertension	17	0.997 (0.971 to 1.025)	0.852	0.985 (0.962 to 1.008)	0.199	0.989 (0.974 to 1.005)	0.170		
1-palmitoyl-2-linoleoyl-GPC (16:0/18:2)	Hypertension	51	1.005 (0.979 to 1.031)	0.718	1.011 (1.002 to 1.019)	0.013	1.008 (1.001 to 1.014)	0.018		
1-stearoyl-2-oleoyl-GPE (18:0/18:1)	Hypertension	35	0.980 (0.952 to 1.008)	0.174	1.006 (0.993 to 1.021)	0.362	1.005 (0.994 to 1.017)	0.350		
1-palmitoyl-2-linoleoyl-GPE (16:0/18:2)	Hypertension	60	1.015 (0.997 to 1.033)	0.108	1.008 (1.002 to 1.014)	0.005	1.008 (1.004 to 1.012)	2.85e-04		
1-stearoyl-2-arachidonoyl-GPC (18:0/20:4)	Hypertension	49	1.001 (0.990 to 1.011)	0.912	0.995 (0.991 to 0.999)	0.012	0.994 (0.991 to 0.997)	3.13e-04		

Exposure	Outcome	nSNP	MR Egger		Weighted median		Inverse variance weighted		MR_PRESSO	
			OR (95% CI)	pval	OR (95% CI)	pval	OR (95% CI)	pval	OR (95% CI)	pval
sphingomyelin (d18:2/16:0, d18:1/16:1)	Hypertension	12	1.062 (0.996 to 1.133)	0.095	0.994 (0.960 to 1.028)	0.722	1.004 (0.979 to 1.030)	0.744		
sphingomyelin (d18:1/14:0, d16:1/16:0)	Hypertension	23	0.972 (0.942 to 1.004)	0.098	0.970 (0.954 to 0.986)	3.34e-04	0.982 (0.967 to 0.998)	0.023	0.979 (0.965 to 0.993)	0.008
2-hydroxydecanoate	Hypertension	12	0.983 (0.942 to 1.025)	0.438	0.988 (0.962 to 1.015)	0.378	1.000 (0.978 to 1.022)	0.977		
dimethylmalonic acid	Hypertension	18	1.007 (0.995 to 1.019)	0.279	1.005 (0.998 to 1.012)	0.142	1.010 (1.003 to 1.016)	0.003	1.010 (1.005 to 1.015)	7.61e-04
N-delta-acetylornithine	Hypertension	31	0.997 (0.980 to 1.015)	0.744	0.987 (0.982 to 0.993)	5.53e-06	0.989 (0.983 to 0.994)	4.24e-05	0.986 (0.982 to 0.991)	3.71e-06

Exposure	Outcome	nSNP	MR Egger		Weighted median		Inverse variance weighted		MR_PRESSO	
			OR (95% CI)	pval	OR (95% CI)	pval	OR (95% CI)	pval	OR (95% CI)	pval
N-acetyl-1-methylhistidine	Hypertension	25	1.041 (1.000 to 1.085)	0.061	1.016 (1.003 to 1.028)	0.012	1.009 (0.995 to 1.022)	0.208	1.023 (1.012 to 1.033)	3.48e-04
N-acetyl-3-methylhistidine	Hypertension	26	0.972 (0.918 to 1.028)	0.333	1.027 (1.012 to 1.043)	4.63e-04	1.021 (1.005 to 1.037)	0.010	1.033 (1.018 to 1.048)	2.22e-04
2-aminooctanoate	Hypertension	30	0.983 (0.945 to 1.022)	0.389	0.981 (0.971 to 0.991)	2.3e-04	0.983 (0.973 to 0.994)	0.002	0.980 (0.970 to 0.989)	3.35e-04
N-acetylcarnosine	Hypertension	24	0.968 (0.927 to 1.010)	0.152	1.002 (0.983 to 1.022)	0.818	0.997 (0.979 to 1.016)	0.787	1.004 (0.988 to 1.021)	0.630
5-hydroxyindoleacetate	Hypertension	17	1.014 (0.969 to 1.060)	0.556	1.003 (0.980 to 1.026)	0.808	1.006 (0.987 to 1.025)	0.536		

Exposure	Outcome	nSNP	MR Egger		Weighted median		Inverse variance weighted		MR_PRESSO	
			OR (95% CI)	pval	OR (95% CI)	pval	OR (95% CI)	pval	OR (95% CI)	pval
2-aminoheptanoate	Hypertension	14	0.992 (0.969 to 1.016)	0.542	0.997 (0.976 to 1.019)	0.813	0.993 (0.978 to 1.008)	0.381		
guanidinoacetate	Hypertension	29	0.963 (0.930 to 0.998)	0.047	0.998 (0.980 to 1.017)	0.867	1.004 (0.987 to 1.021)	0.658	0.999 (0.984 to 1.015)	0.900
bilirubin (Z,Z)	Hypertension	21	1.012 (0.982 to 1.043)	0.439	1.000 (0.984 to 1.016)	0.980	0.995 (0.980 to 1.010)	0.493	0.996 (0.984 to 1.008)	0.512
gamma-glutamylvaline	Hypertension	23	1.001 (0.974 to 1.029)	0.946	1.006 (0.986 to 1.025)	0.563	1.004 (0.990 to 1.018)	0.605		
glycerol 3-phosphate	Hypertension	16	1.009 (0.984 to 1.034)	0.514	0.997 (0.987 to 1.007)	0.582	0.995 (0.983 to 1.007)	0.443	0.996 (0.988 to 1.004)	0.361

Exposure	Outcome	nSNP	MR Egger		Weighted median		Inverse variance weighted		MR_PRESSO	
			OR (95% CI)	pval	OR (95% CI)	pval	OR (95% CI)	pval	OR (95% CI)	pval
aspartate	Hypertension	18	0.999 (0.959 to 1.042)	0.978	1.006 (0.988 to 1.025)	0.512	1.015 (0.992 to 1.039)	0.197	1.009 (0.996 to 1.022)	0.189
1-eicosenoyl-GPC (20:1)	Hypertension	14	0.995 (0.913 to 1.083)	0.905	1.006 (0.980 to 1.032)	0.654	0.997 (0.968 to 1.026)	0.832	0.997 (0.975 to 1.019)	0.777
1-eicosapentaenoyl-GPC (20:5)	Hypertension	68	0.989 (0.965 to 1.014)	0.381	0.986 (0.976 to 0.996)	0.007	0.991 (0.984 to 0.998)	0.014		
4-acetylphenol sulfate	Hypertension	15	0.979 (0.957 to 1.002)	0.097	0.995 (0.975 to 1.015)	0.616	1.001 (0.986 to 1.016)	0.883		
1-dihomo-linolenoyl-GPE (20:3n3 or 6)	Hypertension	57	0.997 (0.992 to 1.001)	0.179	0.997 (0.993 to 1.002)	0.261	1.000 (0.997 to 1.003)	0.847		

Exposure	Outcome	nSNP	MR Egger		Weighted median		Inverse variance weighted		MR_PRESSO	
			OR (95% CI)	pval	OR (95% CI)	pval	OR (95% CI)	pval	OR (95% CI)	pval
1-docosahexaenoyl-GPE (22:6)	Hypertension	31	0.991 (0.946 to 1.039)	0.723	1.000 (0.983 to 1.017)	0.961	0.992 (0.977 to 1.007)	0.278	0.992 (0.981 to 1.005)	0.232
isovalerate	Hypertension	51	1.028 (1.002 to 1.054)	0.042	1.010 (0.997 to 1.023)	0.145	1.002 (0.989 to 1.015)	0.811	1.007 (0.995 to 1.019)	0.257
glutarylcarnitine (C5)	Hypertension	32	0.998 (0.960 to 1.037)	0.906	1.018 (0.999 to 1.036)	0.063	1.014 (0.997 to 1.031)	0.109	1.016 (1.000 to 1.032)	0.063
docosahexaenoate (DHA; 22:6n3)	Hypertension	13	0.976 (0.930 to 1.025)	0.359	1.005 (0.982 to 1.029)	0.662	0.999 (0.981 to 1.016)	0.878		
palmitoylcarnitine	Hypertension	29	1.008 (0.991 to 1.026)	0.355	0.992 (0.977 to 1.008)	0.345	0.992 (0.980 to 1.004)	0.192	0.979 (0.967 to 0.991)	0.002

Exposure	Outcome	nSNP	MR Egger		Weighted median		Inverse variance weighted		MR_PRESSO	
			OR (95% CI)	pval	OR (95% CI)	pval	OR (95% CI)	pval	OR (95% CI)	pval
1-margaroyl-GPC (17:0)	Hypertension	15	1.006 (0.968 to 1.046)	0.765	0.987 (0.968 to 1.007)	0.197	0.994 (0.980 to 1.008)	0.422		
gamma-glutamylmethionine	Hypertension	20	0.942 (0.891 to 0.996)	0.049	0.999 (0.977 to 1.021)	0.921	0.993 (0.975 to 1.013)	0.503	0.988 (0.973 to 1.002)	0.111
gamma-CEHC	Hypertension	12	0.938 (0.900 to 0.977)	0.012	0.974 (0.950 to 0.998)	0.034	0.975 (0.959 to 0.992)	0.004		
N-palmitoyl-sphingosine (d18:1/16:0)	Hypertension	15	0.987 (0.934 to 1.043)	0.654	0.986 (0.961 to 1.011)	0.267	0.991 (0.969 to 1.013)	0.410		
methionine sulfone	Hypertension	32	1.001 (0.975 to 1.028)	0.926	1.002 (0.991 to 1.013)	0.748	0.991 (0.978 to 1.003)	0.144	0.985 (0.974 to 0.996)	0.014

Exposure	Outcome	nSNP	MR Egger		Weighted median		Inverse variance weighted		MR_PRESSO	
			OR (95% CI)	pval	OR (95% CI)	pval	OR (95% CI)	pval	OR (95% CI)	pval
2-methylbutyrylcarnitine (C5)	Hypertension	17	0.988 (0.959 to 1.019)	0.460	1.009 (0.987 to 1.032)	0.420	0.997 (0.978 to 1.018)	0.804	1.003 (0.988 to 1.017)	0.722
O-sulfo-L-tyrosine	Hypertension	21	1.015 (0.979 to 1.052)	0.428	0.998 (0.978 to 1.018)	0.825	0.996 (0.982 to 1.010)	0.577		
3-(3-hydroxyphenyl)propionate sulfate	Hypertension	10	1.005 (0.969 to 1.044)	0.782	1.003 (0.980 to 1.027)	0.783	1.005 (0.988 to 1.024)	0.554		
2-palmitoyl-GPE (16:0)	Hypertension	33	1.009 (0.978 to 1.041)	0.589	1.002 (0.990 to 1.014)	0.749	0.994 (0.985 to 1.004)	0.251		
1-myristoyl-GPC (14:0)	Hypertension	15	0.993 (0.959 to 1.027)	0.677	0.988 (0.962 to 1.016)	0.406	0.973 (0.955 to 0.992)	0.005		

Exposure	Outcome	nSNP	MR Egger		Weighted median		Inverse variance weighted		MR_PRESSO	
			OR (95% CI)	pval	OR (95% CI)	pval	OR (95% CI)	pval	OR (95% CI)	pval
1-arachidoyl-GPC (20:0)	Hypertension	12	1.010 (0.986 to 1.035)	0.434	0.999 (0.988 to 1.011)	0.911	0.993 (0.977 to 1.009)	0.381	0.996 (0.986 to 1.005)	0.395
1-docosapentaenoyl-GPC (22:5n6)	Hypertension	46	0.974 (0.941 to 1.008)	0.142	0.981 (0.969 to 0.994)	0.004	0.983 (0.973 to 0.993)	0.001		
21-hydroxypregnenolone disulfate	Hypertension	27	0.972 (0.924 to 1.022)	0.277	1.010 (0.992 to 1.028)	0.269	1.009 (0.993 to 1.025)	0.299	1.006 (0.992 to 1.020)	0.424
mannitol/sorbitol	Hypertension	14	1.012 (0.966 to 1.060)	0.626	1.014 (0.987 to 1.042)	0.322	1.004 (0.982 to 1.025)	0.743		
methyl glucopyranoside (alpha + beta)	Hypertension	29	1.008 (0.992 to 1.025)	0.331	1.002 (0.991 to 1.014)	0.687	0.999 (0.989 to 1.009)	0.900		

Exposure	Outcome	nSNP	MR Egger		Weighted median		Inverse variance weighted		MR_PRESSO	
			OR (95% CI)	pval	OR (95% CI)	pval	OR (95% CI)	pval	OR (95% CI)	pval
4-methylcatechol sulfate	Hypertension	26	0.990 (0.949 to 1.033)	0.648	0.995 (0.974 to 1.017)	0.654	0.994 (0.975 to 1.013)	0.539	0.989 (0.973 to 1.006)	0.230
3-methyl catechol sulfate (1)	Hypertension	11	0.947 (0.894 to 1.004)	0.101	0.986 (0.958 to 1.015)	0.336	0.987 (0.961 to 1.014)	0.343		
2-docosahexaenoyl-GPC (22:6)	Hypertension	12	0.981 (0.949 to 1.014)	0.286	1.009 (0.986 to 1.033)	0.447	1.007 (0.988 to 1.026)	0.481		
linoleoylcarnitine	Hypertension	22	0.996 (0.966 to 1.026)	0.770	1.015 (0.995 to 1.035)	0.143	1.012 (0.995 to 1.029)	0.154		
pyroglutamine	Hypertension	28	0.984 (0.946 to 1.023)	0.415	0.999 (0.978 to 1.019)	0.892	1.003 (0.986 to 1.022)	0.709	0.999 (0.982 to 1.015)	0.865

Exposure	Outcome	nSNP	MR Egger		Weighted median		Inverse variance weighted		MR_PRESSO	
			OR (95% CI)	pval	OR (95% CI)	pval	OR (95% CI)	pval	OR (95% CI)	pval
X - 11564	Hypertension	22	1.009 (0.951 to 1.071)	0.769	1.007 (0.984 to 1.030)	0.567	1.002 (0.977 to 1.027)	0.893	1.008 (0.985 to 1.032)	0.493
X - 15486	Hypertension	27	1.011 (0.933 to 1.095)	0.799	0.970 (0.951 to 0.990)	0.003	1.008 (0.980 to 1.037)	0.586	0.985 (0.971 to 0.999)	0.051
X - 21286	Hypertension	21	1.012 (0.964 to 1.062)	0.641	1.015 (0.992 to 1.040)	0.204	1.008 (0.989 to 1.026)	0.416		
1-arachidonoyl-GPA (20:4)	Hypertension	17	1.041 (1.005 to 1.079)	0.041	1.029 (1.004 to 1.054)	0.021	1.024 (1.006 to 1.042)	0.010		
X - 11441	Hypertension	25	0.993 (0.970 to 1.017)	0.592	1.000 (0.987 to 1.013)	0.992	1.004 (0.993 to 1.015)	0.516		

Exposure	Outcome	nSNP	MR Egger		Weighted median		Inverse variance weighted		MR_PRESSO	
			OR (95% CI)	pval	OR (95% CI)	pval	OR (95% CI)	pval	OR (95% CI)	pval
X - 21319	Hypertension	22	1.019 (0.985 to 1.054)	0.288	1.015 (0.995 to 1.035)	0.152	1.006 (0.988 to 1.024)	0.497		
X - 18249	Hypertension	18	0.996 (0.958 to 1.036)	0.843	1.004 (0.981 to 1.027)	0.753	1.001 (0.984 to 1.019)	0.888		
X - 11905	Hypertension	43	0.996 (0.982 to 1.011)	0.627	0.997 (0.989 to 1.005)	0.511	1.002 (0.994 to 1.009)	0.670	1.003 (0.996 to 1.009)	0.398
X - 18914	Hypertension	20	0.981 (0.952 to 1.011)	0.223	0.989 (0.968 to 1.009)	0.283	1.005 (0.988 to 1.023)	0.546		
X - 21339	Hypertension	30	1.007 (0.994 to 1.019)	0.318	1.011 (1.002 to 1.019)	0.012	1.007 (1.000 to 1.014)	0.049		

Exposure	Outcome	nSNP	MR Egger		Weighted median		Inverse variance weighted		MR_PRESSO	
			OR (95% CI)	pval	OR (95% CI)	pval	OR (95% CI)	pval	OR (95% CI)	pval
X - 21341	Hypertension	12	1.007 (0.974 to 1.041)	0.696	1.003 (0.977 to 1.030)	0.810	0.999 (0.981 to 1.017)	0.874		
X - 21343	Hypertension	38	0.987 (0.962 to 1.013)	0.335	0.990 (0.974 to 1.006)	0.201	0.996 (0.985 to 1.008)	0.550		
X - 11308	Hypertension	20	1.002 (0.989 to 1.014)	0.790	1.006 (1.000 to 1.013)	0.060	1.003 (0.995 to 1.011)	0.435	1.005 (0.998 to 1.013)	0.190
X - 21353	Hypertension	32	1.002 (0.982 to 1.023)	0.832	0.995 (0.984 to 1.006)	0.389	0.997 (0.987 to 1.007)	0.546		
X - 17178	Hypertension	44	1.001 (0.966 to 1.037)	0.974	1.014 (1.003 to 1.026)	0.014	1.005 (0.991 to 1.019)	0.464	1.011 (1.001 to 1.020)	0.035

Exposure	Outcome	nSNP	MR Egger		Weighted median		Inverse variance weighted		MR_PRESSO	
			OR (95% CI)	pval	OR (95% CI)	pval	OR (95% CI)	pval	OR (95% CI)	pval
X - 21365	Hypertension	20	0.999 (0.960 to 1.040)	0.971	0.996 (0.979 to 1.013)	0.666	0.994 (0.978 to 1.012)	0.523	0.999 (0.983 to 1.015)	0.879
X - 17189	Hypertension	28	1.004 (0.959 to 1.051)	0.871	1.005 (0.985 to 1.027)	0.616	0.998 (0.975 to 1.022)	0.885	1.008 (0.991 to 1.025)	0.364
X - 11442	Hypertension	30	0.980 (0.926 to 1.038)	0.500	1.001 (0.986 to 1.017)	0.851	1.002 (0.985 to 1.021)	0.785	1.010 (0.997 to 1.023)	0.143
X - 21411	Hypertension	31	1.026 (0.969 to 1.087)	0.383	0.996 (0.976 to 1.017)	0.697	0.968 (0.949 to 0.988)	0.001	0.965 (0.946 to 0.983)	9.71e-04
X - 12511	Hypertension	23	1.010 (0.973 to 1.047)	0.607	1.028 (1.018 to 1.039)	3e-08	1.024 (1.012 to 1.036)	1e-04	1.032 (1.023 to 1.042)	1.29e-06

Exposure	Outcome	nSNP	MR Egger		Weighted median		Inverse variance weighted		MR_PRESSO	
			OR (95% CI)	pval	OR (95% CI)	pval	OR (95% CI)	pval	OR (95% CI)	pval
X - 01911	Hypertension	14	1.001 (0.951 to 1.054)	0.964	0.992 (0.968 to 1.016)	0.514	0.990 (0.966 to 1.015)	0.426	0.998 (0.981 to 1.016)	0.847
X - 11444	Hypertension	16	0.993 (0.966 to 1.020)	0.608	1.005 (0.983 to 1.026)	0.674	1.007 (0.991 to 1.023)	0.419		
X - 12824	Hypertension	30	1.016 (0.963 to 1.072)	0.568	0.988 (0.972 to 1.003)	0.122	0.983 (0.964 to 1.003)	0.087	0.993 (0.981 to 1.006)	0.298
X - 15497	Hypertension	15	1.008 (0.967 to 1.051)	0.704	0.996 (0.973 to 1.019)	0.722	1.003 (0.984 to 1.024)	0.735		
X - 21441	Hypertension	22	1.019 (0.987 to 1.052)	0.261	1.005 (0.989 to 1.022)	0.520	1.006 (0.992 to 1.020)	0.426		

Exposure	Outcome	nSNP	MR Egger		Weighted median		Inverse variance weighted		MR_PRESSO	
			OR (95% CI)	pval	OR (95% CI)	pval	OR (95% CI)	pval	OR (95% CI)	pval
X - 17185	Hypertension	18	0.961 (0.917 to 1.007)	0.112	0.991 (0.967 to 1.016)	0.477	0.995 (0.974 to 1.016)	0.646	0.991 (0.973 to 1.009)	0.319
X - 21448	Hypertension	18	1.014 (0.971 to 1.058)	0.550	1.002 (0.991 to 1.013)	0.723	1.002 (0.990 to 1.014)	0.796	1.002 (0.993 to 1.012)	0.633
X - 02269	Hypertension	27	0.981 (0.953 to 1.010)	0.211	1.013 (0.996 to 1.030)	0.132	1.006 (0.994 to 1.019)	0.338		
X - 02249	Hypertension	23	1.004 (0.963 to 1.047)	0.851	0.992 (0.971 to 1.015)	0.504	0.993 (0.974 to 1.011)	0.435	0.998 (0.981 to 1.016)	0.847
X - 11850	Hypertension	10	0.992 (0.947 to 1.038)	0.733	0.989 (0.961 to 1.017)	0.427	0.983 (0.962 to 1.006)	0.142		

Exposure	Outcome	nSNP	MR Egger		Weighted median		Inverse variance weighted		MR_PRESSO	
			OR (95% CI)	pval	OR (95% CI)	pval	OR (95% CI)	pval	OR (95% CI)	pval
X - 16946	Hypertension	25	0.986 (0.959 to 1.014)	0.338	0.999 (0.985 to 1.013)	0.888	0.999 (0.989 to 1.009)	0.841		
alpha-hydroxyisovalerate	Hypertension	38	1.015 (0.998 to 1.032)	0.093	1.010 (0.995 to 1.024)	0.197	0.996 (0.985 to 1.007)	0.462	0.998 (0.989 to 1.008)	0.695
3-methylglutarylcarnitine (1)	Hypertension	14	0.977 (0.937 to 1.019)	0.303	0.995 (0.974 to 1.017)	0.650	0.995 (0.979 to 1.010)	0.493		
3-methylglutarylcarnitine (2)	Hypertension	19	0.978 (0.924 to 1.035)	0.443	1.006 (0.983 to 1.029)	0.636	1.010 (0.990 to 1.031)	0.344	1.009 (0.993 to 1.026)	0.277
X - 12063	Hypertension	37	1.007 (0.987 to 1.027)	0.491	0.975 (0.965 to 0.985)	6.52e-07	0.985 (0.977 to 0.994)	5.77e-04	0.976 (0.970 to 0.982)	8.49e-09

Exposure	Outcome	nSNP	MR Egger		Weighted median		Inverse variance weighted		MR_PRESSO	
			OR (95% CI)	pval	OR (95% CI)	pval	OR (95% CI)	pval	OR (95% CI)	pval
X - 07765	Hypertension	9	1.031 (0.979 to 1.085)	0.284	1.017 (0.989 to 1.046)	0.244	1.011 (0.989 to 1.033)	0.330		
X - 11299	Hypertension	9	1.058 (1.024 to 1.092)	0.011	1.014 (0.987 to 1.041)	0.322	1.010 (0.983 to 1.037)	0.475	1.019 (0.996 to 1.042)	0.155
X - 11305	Hypertension	13	0.992 (0.965 to 1.020)	0.594	0.993 (0.970 to 1.017)	0.578	0.996 (0.978 to 1.015)	0.696		
X - 11372	Hypertension	19	1.035 (0.990 to 1.082)	0.149	1.010 (0.988 to 1.033)	0.363	1.003 (0.986 to 1.020)	0.745		
X - 11429	Hypertension	18	0.957 (0.927 to 0.988)	0.016	1.019 (0.994 to 1.044)	0.137	1.005 (0.987 to 1.022)	0.595		

Exposure	Outcome	nSNP	MR Egger		Weighted median		Inverse variance weighted		MR_PRESSO	
			OR (95% CI)	pval	OR (95% CI)	pval	OR (95% CI)	pval	OR (95% CI)	pval
X - 11438	Hypertension	13	1.030 (0.976 to 1.088)	0.303	0.996 (0.962 to 1.031)	0.821	1.000 (0.972 to 1.029)	0.988		
X - 11440	Hypertension	28	1.015 (0.997 to 1.033)	0.122	1.007 (0.995 to 1.018)	0.258	0.999 (0.990 to 1.009)	0.901	1.000 (0.992 to 1.009)	0.934
X - 11452	Hypertension	8	1.020 (0.961 to 1.083)	0.540	1.008 (0.979 to 1.038)	0.604	1.000 (0.976 to 1.024)	0.991		
X - 11470	Hypertension	33	0.993 (0.971 to 1.016)	0.570	0.999 (0.982 to 1.015)	0.860	0.998 (0.987 to 1.010)	0.763		
X - 11478	Hypertension	19	1.026 (0.990 to 1.063)	0.174	1.015 (0.995 to 1.036)	0.151	1.009 (0.991 to 1.028)	0.325	1.016 (1.002 to 1.030)	0.043

Exposure	Outcome	nSNP	MR Egger		Weighted median		Inverse variance weighted		MR_PRESSO	
			OR (95% CI)	pval	OR (95% CI)	pval	OR (95% CI)	pval	OR (95% CI)	pval
X - 11530	Hypertension	19	1.007 (0.989 to 1.026)	0.445	1.007 (0.993 to 1.021)	0.315	1.010 (1.000 to 1.021)	0.051		
X - 11538	Hypertension	42	0.969 (0.956 to 0.983)	7.84e-05	0.978 (0.970 to 0.987)	1.02e-06	0.981 (0.975 to 0.987)	7.53e-10		
X - 11880	Hypertension	22	0.998 (0.969 to 1.028)	0.883	1.005 (0.986 to 1.025)	0.600	1.009 (0.994 to 1.025)	0.244		
X - 12007	Hypertension	15	0.988 (0.954 to 1.023)	0.501	0.993 (0.971 to 1.015)	0.540	0.992 (0.977 to 1.008)	0.339		
X - 12206	Hypertension	27	0.995 (0.967 to 1.024)	0.742	0.994 (0.977 to 1.012)	0.527	1.006 (0.990 to 1.021)	0.471		

Exposure	Outcome	nSNP	MR Egger		Weighted median		Inverse variance weighted		MR_PRESSO	
			OR (95% CI)	pval	OR (95% CI)	pval	OR (95% CI)	pval	OR (95% CI)	pval
X - 12216	Hypertension	12	0.996 (0.959 to 1.036)	0.860	0.993 (0.968 to 1.020)	0.618	0.987 (0.966 to 1.009)	0.251		
X - 12230	Hypertension	19	0.995 (0.970 to 1.021)	0.714	1.007 (0.987 to 1.027)	0.516	1.003 (0.989 to 1.017)	0.690		
X - 12411	Hypertension	24	0.987 (0.966 to 1.008)	0.236	0.997 (0.979 to 1.014)	0.695	0.995 (0.984 to 1.007)	0.426		
5,8-tetradecadienoate	Hypertension	32	1.014 (0.988 to 1.041)	0.299	0.988 (0.972 to 1.005)	0.163	0.998 (0.986 to 1.010)	0.752		
X - 12450	Hypertension	18	1.011 (0.970 to 1.054)	0.608	0.996 (0.973 to 1.018)	0.708	1.002 (0.983 to 1.022)	0.822		

Exposure	Outcome	nSNP	MR Egger		Weighted median		Inverse variance weighted		MR_PRESSO	
			OR (95% CI)	pval	OR (95% CI)	pval	OR (95% CI)	pval	OR (95% CI)	pval
X - 12729	Hypertension	20	0.985 (0.971 to 0.999)	0.047	0.992 (0.983 to 1.002)	0.116	0.999 (0.989 to 1.008)	0.779	0.997 (0.990 to 1.005)	0.495
X - 12798	Hypertension	38	1.005 (0.992 to 1.017)	0.476	1.005 (0.996 to 1.014)	0.292	0.998 (0.990 to 1.006)	0.595		
X - 12472	Hypertension	27	0.994 (0.954 to 1.037)	0.796	0.988 (0.971 to 1.006)	0.186	0.990 (0.977 to 1.003)	0.142		
X - 12816	Hypertension	14	0.994 (0.952 to 1.037)	0.772	0.982 (0.960 to 1.005)	0.123	0.989 (0.970 to 1.008)	0.244		
X - 14056	Hypertension	19	0.997 (0.919 to 1.083)	0.950	1.014 (0.991 to 1.037)	0.241	1.009 (0.988 to 1.031)	0.402		

Exposure	Outcome	nSNP	MR Egger		Weighted median		Inverse variance weighted		MR_PRESSO	
			OR (95% CI)	pval	OR (95% CI)	pval	OR (95% CI)	pval	OR (95% CI)	pval
X - 12844	Hypertension	20	0.998 (0.951 to 1.048)	0.952	1.018 (0.997 to 1.039)	0.097	1.013 (0.991 to 1.037)	0.253	1.009 (0.990 to 1.029)	0.367
X - 12846	Hypertension	19	1.028 (0.979 to 1.079)	0.286	0.999 (0.979 to 1.019)	0.921	1.001 (0.986 to 1.016)	0.901		
X - 15469	Hypertension	13	0.988 (0.963 to 1.014)	0.395	1.006 (0.997 to 1.014)	0.184	1.003 (0.994 to 1.012)	0.575	1.002 (0.993 to 1.011)	0.646
X - 13529	Hypertension	20	0.990 (0.967 to 1.014)	0.423	0.999 (0.982 to 1.016)	0.901	1.005 (0.991 to 1.019)	0.466		
X - 16132	Hypertension	16	1.004 (0.964 to 1.045)	0.859	0.999 (0.976 to 1.022)	0.903	0.985 (0.968 to 1.002)	0.083		

Exposure	Outcome	nSNP	MR Egger		Weighted median		Inverse variance weighted		MR_PRESSO	
			OR (95% CI)	pval	OR (95% CI)	pval	OR (95% CI)	pval	OR (95% CI)	pval
X - 14568	Hypertension	27	0.995 (0.967 to 1.023)	0.724	0.998 (0.980 to 1.017)	0.860	0.991 (0.979 to 1.004)	0.187		
X - 14939	Hypertension	27	1.025 (0.985 to 1.067)	0.240	1.005 (0.987 to 1.024)	0.563	1.018 (1.004 to 1.032)	0.014		
X - 15245	Hypertension	12	0.995 (0.952 to 1.041)	0.843	1.019 (0.992 to 1.047)	0.175	1.010 (0.991 to 1.029)	0.295		
X - 15492	Hypertension	26	1.005 (0.970 to 1.040)	0.800	1.008 (0.991 to 1.025)	0.370	1.009 (0.996 to 1.023)	0.182		
X - 15728	Hypertension	9	0.963 (0.901 to 1.029)	0.299	0.973 (0.940 to 1.008)	0.134	0.981 (0.947 to 1.016)	0.275	0.973 (0.946 to 1.000)	0.089

Exposure	Outcome	nSNP	MR Egger		Weighted median		Inverse variance weighted		MR_PRESSO	
			OR (95% CI)	pval	OR (95% CI)	pval	OR (95% CI)	pval	OR (95% CI)	pval
X - 16123	Hypertension	25	0.996 (0.963 to 1.031)	0.831	0.995 (0.974 to 1.015)	0.605	1.010 (0.993 to 1.026)	0.248		
X - 16935	Hypertension	33	1.007 (0.991 to 1.023)	0.399	1.001 (0.991 to 1.011)	0.831	1.001 (0.994 to 1.009)	0.726		
X - 16944	Hypertension	20	1.022 (0.985 to 1.061)	0.260	1.013 (0.992 to 1.036)	0.229	1.006 (0.990 to 1.023)	0.443		
X - 17145	Hypertension	15	0.995 (0.979 to 1.012)	0.566	0.996 (0.983 to 1.009)	0.520	0.990 (0.979 to 1.001)	0.083		
X - 18901	Hypertension	10	1.049 (1.007 to 1.093)	0.049	1.010 (0.985 to 1.037)	0.429	1.014 (0.996 to 1.034)	0.131		

Exposure	Outcome	nSNP	MR Egger		Weighted median		Inverse variance weighted		MR_PRESSO	
			OR (95% CI)	pval	OR (95% CI)	pval	OR (95% CI)	pval	OR (95% CI)	pval
X - 17166	Hypertension	27	1.002 (0.968 to 1.037)	0.906	1.003 (0.992 to 1.014)	0.624	0.998 (0.979 to 1.018)	0.846	0.998 (0.991 to 1.006)	0.666
X - 17167	Hypertension	19	1.003 (0.966 to 1.042)	0.862	1.006 (0.986 to 1.027)	0.556	1.015 (0.996 to 1.034)	0.128	1.019 (1.002 to 1.037)	0.039
X - 18913	Hypertension	12	1.002 (0.970 to 1.036)	0.889	0.988 (0.965 to 1.012)	0.318	0.985 (0.968 to 1.003)	0.096		
X - 21628	Hypertension	29	0.986 (0.956 to 1.017)	0.366	0.973 (0.958 to 0.989)	0.001	0.988 (0.976 to 1.000)	0.042		
X - 16134	Hypertension	28	0.989 (0.953 to 1.026)	0.545	0.997 (0.975 to 1.020)	0.816	1.004 (0.986 to 1.023)	0.643		

Exposure	Outcome	nSNP	MR Egger		Weighted median		Inverse variance weighted		MR_PRESSO	
			OR (95% CI)	pval	OR (95% CI)	pval	OR (95% CI)	pval	OR (95% CI)	pval
X - 17179	Hypertension	24	0.992 (0.967 to 1.018)	0.555	0.995 (0.984 to 1.005)	0.326	0.990 (0.979 to 1.002)	0.108	0.993 (0.984 to 1.003)	0.169
X - 19141	Hypertension	32	1.017 (0.993 to 1.042)	0.177	1.008 (1.000 to 1.015)	0.054	1.002 (0.992 to 1.013)	0.649	1.006 (0.998 to 1.014)	0.161
X - 17438	Hypertension	10	1.023 (0.937 to 1.116)	0.627	1.007 (0.976 to 1.040)	0.660	1.001 (0.969 to 1.034)	0.940	0.990 (0.961 to 1.019)	0.514
X - 21666	Hypertension	16	0.983 (0.952 to 1.015)	0.318	1.010 (0.988 to 1.034)	0.373	0.999 (0.981 to 1.016)	0.887		
X - 12855	Hypertension	17	0.984 (0.929 to 1.042)	0.583	0.997 (0.973 to 1.022)	0.834	0.991 (0.970 to 1.012)	0.393		

Exposure	Outcome	nSNP	MR Egger		Weighted median		Inverse variance weighted		MR_PRESSO	
			OR (95% CI)	pval	OR (95% CI)	pval	OR (95% CI)	pval	OR (95% CI)	pval
oleoyl-linoleoyl-glycerol (18:1/18:2) [1]	Hypertension	41	1.009 (0.968 to 1.051)	0.683	1.019 (1.005 to 1.034)	0.009	1.009 (0.993 to 1.024)	0.268	1.021 (1.011 to 1.031)	1.48e-04
oleoyl-linoleoyl-glycerol (18:1/18:2) [2]	Hypertension	17	1.029 (0.969 to 1.092)	0.372	1.042 (1.020 to 1.064)	1.1e-04	1.012 (0.983 to 1.043)	0.424	1.033 (1.015 to 1.052)	0.003
X - 12748	Hypertension	17	1.015 (0.956 to 1.078)	0.634	1.007 (0.980 to 1.035)	0.623	0.998 (0.972 to 1.025)	0.884	1.002 (0.978 to 1.027)	0.856
X - 21735	Hypertension	32	1.038 (1.005 to 1.072)	0.031	0.987 (0.972 to 1.003)	0.118	0.985 (0.972 to 0.999)	0.030		
X - 21736	Hypertension	21	0.971 (0.937 to 1.007)	0.133	0.974 (0.954 to 0.994)	0.012	0.991 (0.972 to 1.010)	0.347	0.984 (0.971 to 0.996)	0.020

Exposure	Outcome	nSNP	MR Egger		Weighted median		Inverse variance weighted		MR_PRESSO	
			OR (95% CI)	pval	OR (95% CI)	pval	OR (95% CI)	pval	OR (95% CI)	pval
X - 21737	Hypertension	10	1.020 (0.976 to 1.066)	0.398	0.996 (0.971 to 1.022)	0.745	1.002 (0.980 to 1.024)	0.881		
X - 11522	Hypertension	20	0.992 (0.959 to 1.026)	0.634	1.002 (0.990 to 1.015)	0.694	1.001 (0.991 to 1.012)	0.815		
gulonic acid	Hypertension	11	0.985 (0.925 to 1.049)	0.648	0.991 (0.963 to 1.021)	0.562	0.995 (0.971 to 1.019)	0.677		
sulfate	Hypertension	16	1.038 (0.990 to 1.089)	0.142	1.001 (0.980 to 1.022)	0.950	1.002 (0.979 to 1.026)	0.837	1.007 (0.990 to 1.025)	0.420
X - 15503	Hypertension	17	0.992 (0.946 to 1.040)	0.738	0.987 (0.963 to 1.012)	0.321	0.990 (0.972 to 1.008)	0.264		

Exposure	Outcome	nSNP	MR Egger		Weighted median		Inverse variance weighted		MR_PRESSO	
			OR (95% CI)	pval	OR (95% CI)	pval	OR (95% CI)	pval	OR (95% CI)	pval
X - 12822	Hypertension	23	1.029 (0.943 to 1.122)	0.529	0.972 (0.955 to 0.989)	0.001	1.010 (0.983 to 1.038)	0.480	0.981 (0.972 to 0.990)	6e-04
X - 21821	Hypertension	15	1.000 (0.979 to 1.023)	0.969	0.999 (0.986 to 1.012)	0.882	0.997 (0.988 to 1.005)	0.453		
X - 17327	Hypertension	24	0.994 (0.957 to 1.033)	0.769	0.983 (0.963 to 1.003)	0.100	0.988 (0.973 to 1.003)	0.114		
X - 16570	Hypertension	18	0.985 (0.933 to 1.040)	0.590	0.983 (0.964 to 1.003)	0.095	0.982 (0.967 to 0.997)	0.020		
X - 21849	Hypertension	21	1.055 (0.999 to 1.115)	0.072	1.004 (0.985 to 1.024)	0.659	0.994 (0.979 to 1.010)	0.455		

Exposure	Outcome	nSNP	MR Egger		Weighted median		Inverse variance weighted		MR_PRESSO	
			OR (95% CI)	pval	OR (95% CI)	pval	OR (95% CI)	pval	OR (95% CI)	pval
X - 21892	Hypertension	28	1.032 (1.009 to 1.056)	0.012	1.013 (0.995 to 1.031)	0.163	1.009 (0.995 to 1.024)	0.199		
1-nonadecanoyl-GPC (19:0)	Hypertension	18	0.990 (0.947 to 1.035)	0.668	0.988 (0.961 to 1.016)	0.389	0.982 (0.961 to 1.004)	0.113		
etiocholanolone glucuronide	Hypertension	24	1.007 (0.981 to 1.035)	0.591	1.007 (0.990 to 1.025)	0.399	0.994 (0.980 to 1.008)	0.411		
2-palmitoleoyl-GPC (16:1)	Hypertension	16	0.997 (0.925 to 1.075)	0.947	0.997 (0.966 to 1.029)	0.854	0.982 (0.955 to 1.010)	0.195	0.972 (0.948 to 0.996)	0.038
sphingomyelin (d18:1/24:1, d18:2/24:0)	Hypertension	42	1.032 (1.006 to 1.059)	0.022	0.995 (0.984 to 1.006)	0.382	0.998 (0.988 to 1.008)	0.689	0.998 (0.990 to 1.006)	0.597

Exposure	Outcome	nSNP	MR Egger		Weighted median		Inverse variance weighted		MR_PRESSO	
			OR (95% CI)	pval	OR (95% CI)	pval	OR (95% CI)	pval	OR (95% CI)	pval
sphingomyelin (d18:2/14:0, d18:1/14:1)	Hypertension	15	1.014 (0.972 to 1.058)	0.527	1.010 (0.985 to 1.035)	0.434	1.001 (0.981 to 1.022)	0.923		
X - 12680	Hypertension	16	1.016 (0.979 to 1.055)	0.410	0.999 (0.977 to 1.022)	0.946	0.987 (0.966 to 1.009)	0.237	0.983 (0.966 to 1.000)	0.065
X - 22147	Hypertension	14	0.986 (0.935 to 1.038)	0.594	1.008 (0.984 to 1.033)	0.517	0.997 (0.980 to 1.014)	0.704		
X - 22162	Hypertension	18	1.000 (0.983 to 1.017)	0.997	1.003 (0.994 to 1.013)	0.459	1.001 (0.993 to 1.010)	0.765		
X - 10458	Hypertension	6	0.986 (0.948 to 1.026)	0.535	0.995 (0.959 to 1.033)	0.809	0.990 (0.964 to 1.017)	0.470		

Exposure	Outcome	nSNP	MR Egger		Weighted median		Inverse variance weighted		MR_PRESSO	
			OR (95% CI)	pval	OR (95% CI)	pval	OR (95% CI)	pval	OR (95% CI)	pval
X - 11537	Hypertension	21	0.999 (0.978 to 1.020)	0.902	1.005 (0.987 to 1.024)	0.574	1.003 (0.990 to 1.016)	0.649		
X - 22379	Hypertension	30	1.011 (0.993 to 1.030)	0.239	1.008 (0.994 to 1.023)	0.264	1.005 (0.994 to 1.016)	0.383		
X - 11871	Hypertension	10	1.034 (0.975 to 1.097)	0.295	1.033 (0.999 to 1.068)	0.056	1.039 (1.012 to 1.067)	0.004		
X - 12101	Hypertension	27	0.970 (0.911 to 1.033)	0.358	0.987 (0.968 to 1.006)	0.178	0.996 (0.979 to 1.013)	0.654	0.992 (0.978 to 1.006)	0.271
X - 11483	Hypertension	10	1.008 (0.952 to 1.067)	0.802	1.015 (0.983 to 1.048)	0.351	1.001 (0.973 to 1.030)	0.940	0.985 (0.961 to 1.009)	0.245

Exposure	Outcome	nSNP	MR Egger		Weighted median		Inverse variance weighted		MR_PRESSO	
			OR (95% CI)	pval	OR (95% CI)	pval	OR (95% CI)	pval	OR (95% CI)	pval
X - 11491	Hypertension	42	1.016 (1.004 to 1.029)	0.016	1.001 (0.993 to 1.009)	0.788	1.000 (0.994 to 1.007)	0.929		
X - 12231	Hypertension	18	0.968 (0.924 to 1.013)	0.181	0.986 (0.964 to 1.007)	0.191	0.983 (0.966 to 1.000)	0.051		
X - 12830	Hypertension	12	1.001 (0.963 to 1.040)	0.974	0.999 (0.976 to 1.021)	0.901	0.982 (0.962 to 1.003)	0.091		
X - 18899	Hypertension	17	1.016 (0.996 to 1.037)	0.146	0.994 (0.977 to 1.011)	0.477	0.996 (0.982 to 1.010)	0.588		
5-hydroxytryptophan	Hypertension	26	1.009 (0.959 to 1.061)	0.734	1.001 (0.978 to 1.025)	0.936	1.005 (0.983 to 1.028)	0.655	0.992 (0.973 to 1.011)	0.391

Exposure	Outcome	nSNP	MR Egger		Weighted median		Inverse variance weighted		MR_PRESSO	
			OR (95% CI)	pval	OR (95% CI)	pval	OR (95% CI)	pval	OR (95% CI)	pval
X - 12127	Hypertension	21	1.006 (0.946 to 1.070)	0.855	1.024 (1.002 to 1.047)	0.036	1.019 (1.002 to 1.036)	0.028		
X - 12339	Hypertension	15	0.928 (0.803 to 1.073)	0.333	1.008 (0.969 to 1.050)	0.681	1.019 (0.977 to 1.064)	0.378	1.033 (1.001 to 1.067)	0.070
X - 12681	Hypertension	29	0.978 (0.938 to 1.019)	0.297	0.986 (0.965 to 1.008)	0.202	0.980 (0.961 to 1.000)	0.046	0.982 (0.965 to 0.999)	0.049
X - 12686	Hypertension	26	0.997 (0.956 to 1.038)	0.872	1.004 (0.984 to 1.024)	0.724	1.004 (0.987 to 1.022)	0.618		
X - 12688	Hypertension	22	1.016 (0.978 to 1.056)	0.426	1.003 (0.986 to 1.021)	0.717	1.004 (0.989 to 1.019)	0.647	1.000 (0.986 to 1.014)	0.967

Exposure	Outcome	nSNP	MR Egger		Weighted median		Inverse variance weighted		MR_PRESSO	
			OR (95% CI)	pval	OR (95% CI)	pval	OR (95% CI)	pval	OR (95% CI)	pval
X - 14838	Hypertension	25	0.943 (0.901 to 0.987)	0.020	0.972 (0.949 to 0.996)	0.021	0.975 (0.953 to 0.998)	0.032	0.969 (0.949 to 0.989)	0.006
nonanoylcarnitine	Hypertension	20	1.014 (0.993 to 1.036)	0.198	1.007 (0.994 to 1.021)	0.281	1.002 (0.991 to 1.013)	0.711		
X - 15666	Hypertension	16	1.003 (0.963 to 1.045)	0.887	0.998 (0.975 to 1.022)	0.891	0.999 (0.979 to 1.020)	0.921	0.990 (0.973 to 1.007)	0.278
X - 16071	Hypertension	12	0.986 (0.934 to 1.040)	0.611	0.994 (0.973 to 1.016)	0.588	0.990 (0.969 to 1.012)	0.371		
X - 16580	Hypertension	14	1.023 (0.962 to 1.088)	0.488	1.028 (0.995 to 1.062)	0.092	1.029 (1.005 to 1.054)	0.020		

Exposure	Outcome	nSNP	MR Egger		Weighted median		Inverse variance weighted		MR_PRESSO	
			OR (95% CI)	pval	OR (95% CI)	pval	OR (95% CI)	pval	OR (95% CI)	pval
X - 17337	Hypertension	29	1.003 (0.987 to 1.020)	0.712	1.001 (0.989 to 1.012)	0.892	1.007 (0.997 to 1.017)	0.165		
1-eicosapentaenoyl-GPE (20:5)	Hypertension	24	1.019 (0.984 to 1.055)	0.312	1.007 (0.989 to 1.026)	0.449	0.996 (0.983 to 1.009)	0.558		
X - 12026	Hypertension	17	1.032 (0.977 to 1.089)	0.275	0.993 (0.966 to 1.020)	0.609	1.007 (0.984 to 1.029)	0.568		
X - 12543	Hypertension	16	0.977 (0.936 to 1.019)	0.297	0.980 (0.954 to 1.006)	0.122	0.992 (0.972 to 1.013)	0.448		
X - 17010	Hypertension	44	0.997 (0.978 to 1.016)	0.778	1.000 (0.986 to 1.015)	0.989	0.995 (0.985 to 1.005)	0.284		

Exposure	Outcome	nSNP	MR Egger		Weighted median		Inverse variance weighted		MR_PRESSO	
			OR (95% CI)	pval	OR (95% CI)	pval	OR (95% CI)	pval	OR (95% CI)	pval
X - 17351	Hypertension	13	1.051 (1.001 to 1.104)	0.073	1.040 (1.008 to 1.073)	0.013	1.039 (1.015 to 1.064)	0.002		
X - 22764	Hypertension	22	1.007 (0.973 to 1.041)	0.702	0.994 (0.974 to 1.014)	0.531	1.007 (0.991 to 1.024)	0.395		
glucose	Hypertension	25	0.989 (0.966 to 1.013)	0.372	1.000 (0.983 to 1.017)	0.970	0.998 (0.984 to 1.013)	0.823		
mannose	Hypertension	19	1.034 (0.976 to 1.095)	0.268	1.046 (1.023 to 1.070)	9.69e-05	1.029 (1.007 to 1.052)	0.011		
X - 10358	Hypertension	14	1.062 (1.007 to 1.120)	0.047	1.007 (0.982 to 1.034)	0.576	1.011 (0.993 to 1.029)	0.240		

Exposure	Outcome	nSNP	MR Egger		Weighted median		Inverse variance weighted		MR_PRESSO	
			OR (95% CI)	pval	OR (95% CI)	pval	OR (95% CI)	pval	OR (95% CI)	pval
X - 22816	Hypertension	20	0.990 (0.965 to 1.016)	0.469	0.989 (0.972 to 1.006)	0.212	0.994 (0.982 to 1.007)	0.394		
X - 22822	Hypertension	14	1.007 (0.951 to 1.066)	0.807	0.988 (0.963 to 1.013)	0.335	1.005 (0.978 to 1.033)	0.700	0.992 (0.972 to 1.013)	0.475
myristoleoylcarnitine	Hypertension	19	0.993 (0.975 to 1.012)	0.485	1.001 (0.987 to 1.014)	0.917	1.001 (0.991 to 1.011)	0.886		
N-acetyltaurine	Hypertension	19	1.004 (0.971 to 1.039)	0.800	0.997 (0.976 to 1.017)	0.745	0.994 (0.978 to 1.010)	0.447		
X - 12695	Hypertension	24	1.016 (0.982 to 1.050)	0.370	0.995 (0.986 to 1.004)	0.257	0.999 (0.989 to 1.010)	0.896	0.997 (0.989 to 1.006)	0.586

Exposure	Outcome	nSNP	MR Egger		Weighted median		Inverse variance weighted		MR_PRESSO	
			OR (95% CI)	pval	OR (95% CI)	pval	OR (95% CI)	pval	OR (95% CI)	pval
arabonate/xylonate	Hypertension	16	0.980 (0.902 to 1.064)	0.633	0.993 (0.967 to 1.021)	0.642	1.007 (0.976 to 1.039)	0.657	0.987 (0.967 to 1.008)	0.251
2-oleoyl-GPC (18:1)	Hypertension	20	1.003 (0.963 to 1.045)	0.869	1.004 (0.982 to 1.025)	0.749	0.992 (0.975 to 1.010)	0.394		
X - 24435	Hypertension	23	0.999 (0.961 to 1.040)	0.973	1.011 (0.995 to 1.027)	0.190	1.004 (0.989 to 1.019)	0.623	1.007 (0.994 to 1.021)	0.299
dopamine sulfate (2)	Hypertension	33	1.001 (0.991 to 1.010)	0.920	1.002 (0.994 to 1.009)	0.670	1.005 (0.998 to 1.011)	0.135		
tyramine O-sulfate	Hypertension	16	0.986 (0.956 to 1.017)	0.390	0.990 (0.970 to 1.009)	0.299	0.992 (0.975 to 1.010)	0.386		

Exposure	Outcome	nSNP	MR Egger		Weighted median		Inverse variance weighted		MR_PRESSO	
			OR (95% CI)	pval	OR (95% CI)	pval	OR (95% CI)	pval	OR (95% CI)	pval
methyl-4-hydroxybenzoate sulfate	Hypertension	6	1.044 (0.969 to 1.126)	0.320	1.007 (0.961 to 1.055)	0.768	0.997 (0.953 to 1.042)	0.884		
N-acetylcitrulline	Hypertension	28	1.011 (0.993 to 1.029)	0.243	1.014 (1.008 to 1.021)	4.34e-05	1.011 (1.004 to 1.019)	0.002	1.016 (1.010 to 1.023)	3.35e-05
4-hydroxychlorothalonil	Hypertension	21	1.019 (0.977 to 1.064)	0.384	1.010 (0.987 to 1.033)	0.410	1.001 (0.984 to 1.019)	0.900		
3-hydroxypyridine sulfate	Hypertension	20	0.994 (0.971 to 1.018)	0.623	0.993 (0.983 to 1.002)	0.132	0.992 (0.980 to 1.005)	0.240	0.997 (0.987 to 1.006)	0.520
sphingomyelin (d18:1/20:0, d16:1/22:0)	Hypertension	20	0.939 (0.885 to 0.996)	0.050	0.995 (0.974 to 1.016)	0.630	0.998 (0.976 to 1.021)	0.893	0.992 (0.976 to 1.009)	0.391

Exposure	Outcome	nSNP	MR Egger		Weighted median		Inverse variance weighted		MR_PRESSO	
			OR (95% CI)	pval	OR (95% CI)	pval	OR (95% CI)	pval	OR (95% CI)	pval
sphingomyelin (d18:1/20:1, d18:2/20:0)	Hypertension	25	1.017 (0.984 to 1.051)	0.332	0.993 (0.977 to 1.011)	0.454	0.995 (0.981 to 1.009)	0.458		
behenoyl sphingomyelin (d18:1/22:0)	Hypertension	18	0.987 (0.947 to 1.028)	0.544	0.988 (0.968 to 1.009)	0.256	0.989 (0.965 to 1.013)	0.357	0.982 (0.963 to 1.001)	0.085
X - 23026	Hypertension	22	1.028 (0.992 to 1.066)	0.148	1.020 (1.000 to 1.041)	0.052	1.020 (1.002 to 1.038)	0.030		
2-acetamidophenol sulfate	Hypertension	10	0.993 (0.893 to 1.104)	0.895	1.029 (0.991 to 1.067)	0.132	1.033 (1.008 to 1.059)	0.010		
1,2,3-benzenetriol sulfate (2)	Hypertension	9	1.053 (0.973 to 1.140)	0.239	1.022 (0.991 to 1.055)	0.170	1.013 (0.984 to 1.044)	0.381		

Exposure	Outcome	nSNP	MR Egger		Weighted median		Inverse variance weighted		MR_PRESSO	
			OR (95% CI)	pval	OR (95% CI)	pval	OR (95% CI)	pval	OR (95% CI)	pval
C-glycosyltryptophan	Hypertension	19	0.977 (0.946 to 1.009)	0.171	0.994 (0.973 to 1.017)	0.618	0.999 (0.978 to 1.021)	0.946	1.006 (0.984 to 1.027)	0.613
p-cresol-glucuronide	Hypertension	12	0.959 (0.886 to 1.038)	0.328	0.998 (0.969 to 1.028)	0.902	0.993 (0.960 to 1.028)	0.705	0.984 (0.955 to 1.014)	0.325
arabitol/xylitol	Hypertension	25	1.026 (0.985 to 1.068)	0.228	1.009 (0.989 to 1.029)	0.379	1.014 (0.999 to 1.029)	0.071		
X - 23293	Hypertension	35	1.015 (0.976 to 1.056)	0.470	1.006 (0.994 to 1.017)	0.321	1.000 (0.993 to 1.008)	0.901		
X - 23593	Hypertension	18	1.014 (0.974 to 1.056)	0.501	1.023 (0.997 to 1.049)	0.078	1.018 (0.999 to 1.037)	0.059		

Exposure	Outcome	nSNP	MR Egger		Weighted median		Inverse variance weighted		MR_PRESSO	
			OR (95% CI)	pval	OR (95% CI)	pval	OR (95% CI)	pval	OR (95% CI)	pval
X - 23639	Hypertension	17	1.014 (0.982 to 1.048)	0.406	0.982 (0.957 to 1.007)	0.156	0.990 (0.972 to 1.008)	0.264		
X - 23644	Hypertension	23	0.999 (0.960 to 1.041)	0.977	0.998 (0.979 to 1.019)	0.868	1.001 (0.982 to 1.020)	0.951	0.992 (0.979 to 1.005)	0.261
X - 23705	Hypertension	48	0.981 (0.963 to 0.999)	0.040	0.992 (0.986 to 0.998)	0.009	0.992 (0.987 to 0.997)	0.001		
X - 11315	Hypertension	26	0.981 (0.951 to 1.013)	0.263	0.986 (0.967 to 1.006)	0.171	0.985 (0.970 to 0.999)	0.039		
X - 23765	Hypertension	19	0.998 (0.956 to 1.043)	0.934	1.009 (0.985 to 1.033)	0.467	0.993 (0.975 to 1.011)	0.443		

Exposure	Outcome	nSNP	MR Egger		Weighted median		Inverse variance weighted		MR_PRESSO	
			OR (95% CI)	pval	OR (95% CI)	pval	OR (95% CI)	pval	OR (95% CI)	pval
X - 23782	Hypertension	13	0.970 (0.912 to 1.030)	0.339	0.984 (0.957 to 1.011)	0.248	0.990 (0.969 to 1.011)	0.339		
X - 23788	Hypertension	15	1.005 (0.974 to 1.037)	0.776	0.997 (0.973 to 1.022)	0.819	1.007 (0.990 to 1.025)	0.408		
anthranilate	Hypertension	18	1.008 (0.969 to 1.048)	0.690	0.996 (0.973 to 1.020)	0.765	0.996 (0.980 to 1.013)	0.668		
X - 23314	Hypertension	26	1.016 (0.994 to 1.039)	0.157	1.008 (0.993 to 1.022)	0.302	1.001 (0.988 to 1.013)	0.916		
dimethylglycine	Hypertension	26	0.993 (0.963 to 1.024)	0.670	1.001 (0.984 to 1.018)	0.947	1.002 (0.988 to 1.017)	0.746		

Exposure	Outcome	nSNP	MR Egger		Weighted median		Inverse variance weighted		MR_PRESSO	
			OR (95% CI)	pval	OR (95% CI)	pval	OR (95% CI)	pval	OR (95% CI)	pval
creatinine	Hypertension	23	1.009 (0.969 to 1.051)	0.659	1.014 (0.992 to 1.035)	0.214	1.001 (0.983 to 1.019)	0.921		
X - 23997	Hypertension	19	1.003 (0.970 to 1.036)	0.876	0.993 (0.974 to 1.013)	0.501	0.995 (0.979 to 1.011)	0.546		
X - 24021	Hypertension	16	1.000 (0.969 to 1.032)	0.980	0.989 (0.967 to 1.010)	0.302	0.990 (0.975 to 1.006)	0.212		
X - 24061	Hypertension	25	0.990 (0.965 to 1.015)	0.418	0.990 (0.970 to 1.010)	0.321	0.986 (0.973 to 0.998)	0.027		
X - 24065	Hypertension	14	0.970 (0.913 to 1.030)	0.339	0.983 (0.957 to 1.009)	0.191	0.985 (0.968 to 1.002)	0.091		

Exposure	Outcome	nSNP	MR Egger		Weighted median		Inverse variance weighted		MR_PRESSO	
			OR (95% CI)	pval	OR (95% CI)	pval	OR (95% CI)	pval	OR (95% CI)	pval
X - 24071	Hypertension	23	1.004 (0.959 to 1.052)	0.859	0.993 (0.973 to 1.014)	0.539	0.998 (0.980 to 1.017)	0.846	0.994 (0.978 to 1.011)	0.504
X - 24097	Hypertension	27	0.996 (0.959 to 1.034)	0.821	0.996 (0.976 to 1.017)	0.720	0.989 (0.972 to 1.007)	0.245		
X - 24125	Hypertension	24	1.039 (0.979 to 1.103)	0.215	1.024 (1.002 to 1.046)	0.032	1.014 (0.990 to 1.038)	0.268	1.019 (1.000 to 1.038)	0.058
X - 24240	Hypertension	32	1.003 (0.978 to 1.030)	0.791	0.997 (0.980 to 1.015)	0.734	1.000 (0.986 to 1.015)	0.995	0.997 (0.984 to 1.010)	0.666
X - 24241	Hypertension	26	1.012 (0.987 to 1.038)	0.361	1.005 (0.991 to 1.020)	0.464	1.001 (0.991 to 1.012)	0.793		

Exposure	Outcome	nSNP	MR Egger		Weighted median		Inverse variance weighted		MR_PRESSO	
			OR (95% CI)	pval	OR (95% CI)	pval	OR (95% CI)	pval	OR (95% CI)	pval
2-hydroxybutyrate/2-hydroxyisobutyrate	Hypertension	14	0.968 (0.925 to 1.014)	0.193	0.987 (0.963 to 1.012)	0.300	0.997 (0.972 to 1.023)	0.810	0.991 (0.970 to 1.013)	0.447
2-methylcitrate/homocitrate	Hypertension	14	0.992 (0.956 to 1.029)	0.672	0.999 (0.977 to 1.022)	0.964	0.998 (0.979 to 1.017)	0.804		
X - 24242	Hypertension	16	0.998 (0.964 to 1.034)	0.923	0.998 (0.976 to 1.021)	0.890	0.996 (0.977 to 1.015)	0.676	1.009 (0.997 to 1.021)	0.172
oleate/vaccenate (18:1)	Hypertension	31	0.995 (0.979 to 1.010)	0.488	1.000 (0.989 to 1.011)	0.970	1.001 (0.993 to 1.010)	0.747		
2-hydroxyglutarate	Hypertension	18	0.998 (0.951 to 1.048)	0.951	1.001 (0.980 to 1.023)	0.897	1.011 (0.992 to 1.030)	0.272	1.006 (0.989 to 1.023)	0.508

Exposure	Outcome	nSNP	MR Egger		Weighted median		Inverse variance weighted		MR_PRESSO	
			OR (95% CI)	pval	OR (95% CI)	pval	OR (95% CI)	pval	OR (95% CI)	pval
X - 24293	Hypertension	13	1.036 (0.987 to 1.087)	0.177	1.002 (0.969 to 1.035)	0.917	1.009 (0.986 to 1.032)	0.451		
cis-aconitate	Hypertension	14	0.998 (0.961 to 1.037)	0.921	1.001 (0.972 to 1.030)	0.961	1.002 (0.979 to 1.025)	0.882		
X - 24278	Hypertension	52	1.002 (0.991 to 1.012)	0.750	1.002 (0.995 to 1.008)	0.630	0.999 (0.993 to 1.005)	0.709	0.999 (0.993 to 1.004)	0.625
leucylphenylalanine/isoleucylphenylalanine	Hypertension	17	0.993 (0.968 to 1.019)	0.613	0.989 (0.976 to 1.002)	0.103	0.990 (0.978 to 1.003)	0.137		
1-palmitoleoylglycerol (16:1)	Hypertension	16	0.987 (0.947 to 1.030)	0.563	0.995 (0.970 to 1.019)	0.660	0.999 (0.982 to 1.017)	0.940		

Exposure	Outcome	nSNP	MR Egger		Weighted median		Inverse variance weighted		MR_PRESSO	
			OR (95% CI)	pval	OR (95% CI)	pval	OR (95% CI)	pval	OR (95% CI)	pval
sphingomyelin (d18:1/15:0, d16:1/17:0)	Hypertension	21	0.985 (0.948 to 1.023)	0.446	0.976 (0.955 to 0.998)	0.031	0.986 (0.970 to 1.003)	0.098		
palmitoyl dihydrosphingomyelin (d18:0/16:0)	Hypertension	16	1.025 (0.957 to 1.097)	0.493	1.011 (0.979 to 1.044)	0.496	1.009 (0.980 to 1.038)	0.555	1.019 (0.997 to 1.042)	0.109
sphingomyelin (d18:2/23:0, d18:1/23:1, d17:1/24:1)	Hypertension	23	1.004 (0.953 to 1.058)	0.889	1.022 (0.998 to 1.045)	0.068	1.001 (0.981 to 1.023)	0.893	1.008 (0.990 to 1.027)	0.378
sphingomyelin (d18:2/24:1, d18:1/24:2)	Hypertension	25	1.014 (0.993 to 1.035)	0.215	1.003 (0.986 to 1.021)	0.729	1.010 (0.998 to 1.023)	0.098		
1-stearoyl-2-oleoyl-GPC (18:0/18:1)	Hypertension	18	0.994 (0.971 to 1.018)	0.640	1.002 (0.987 to 1.018)	0.774	1.006 (0.992 to 1.019)	0.396		

Exposure	Outcome	nSNP	MR Egger		Weighted median		Inverse variance weighted		MR_PRESSO	
			OR (95% CI)	pval	OR (95% CI)	pval	OR (95% CI)	pval	OR (95% CI)	pval
1-stearoyl-2-linoleoyl-GPE (18:0/18:2)	Hypertension	65	1.021 (1.000 to 1.042)	0.057	1.009 (1.003 to 1.015)	0.006	1.009 (1.004 to 1.014)	3e-04		
1-stearoyl-2-arachidonoyl-GPE (18:0/20:4)	Hypertension	27	1.027 (0.992 to 1.063)	0.142	1.002 (0.992 to 1.012)	0.720	1.002 (0.993 to 1.012)	0.657		
1-stearoyl-2-arachidonoyl-GPI (18:0/20:4)	Hypertension	51	0.989 (0.939 to 1.043)	0.694	0.989 (0.981 to 0.997)	0.009	0.995 (0.987 to 1.004)	0.265	0.997 (0.990 to 1.004)	0.377
1-palmitoyl-2-linoleoyl-GPI (16:0/18:2)	Hypertension	53	0.999 (0.970 to 1.028)	0.925	1.013 (1.003 to 1.024)	0.014	1.009 (1.002 to 1.016)	0.016		
1-stearoyl-2-linoleoyl-GPC (18:0/18:2)	Hypertension	49	0.989 (0.964 to 1.014)	0.389	1.016 (1.004 to 1.028)	0.009	1.007 (0.998 to 1.016)	0.128		

Exposure	Outcome	nSNP	MR Egger		Weighted median		Inverse variance weighted		MR_PRESSO	
			OR (95% CI)	pval	OR (95% CI)	pval	OR (95% CI)	pval	OR (95% CI)	pval
1-oleoyl-2-linoleoyl-GPC (18:1/18:2)	Hypertension	45	1.018 (0.978 to 1.058)	0.390	1.014 (1.003 to 1.025)	0.010	1.015 (1.007 to 1.023)	2.09e-04		
1-palmitoyl-2-dihomo-linolenoyl-GPC (16:0/20:3n3 or 6)	Hypertension	41	1.004 (0.995 to 1.012)	0.417	1.004 (0.996 to 1.011)	0.342	1.004 (0.999 to 1.009)	0.102		
1-palmitoyl-2-oleoyl-GPC (16:0/18:1)	Hypertension	18	1.007 (0.961 to 1.055)	0.778	1.005 (0.980 to 1.032)	0.675	1.014 (0.995 to 1.034)	0.138		
1-palmitoyl-2-arachidonoyl-GPC (16:0/20:4)	Hypertension	48	0.990 (0.977 to 1.002)	0.112	0.994 (0.990 to 0.999)	0.013	0.994 (0.991 to 0.998)	9.88e-04		
1-palmitoyl-2-eicosapentaenoyl-GPC (16:0/20:5)	Hypertension	50	0.983 (0.959 to 1.007)	0.170	0.986 (0.976 to 0.996)	0.007	0.988 (0.980 to 0.996)	0.002		

Exposure	Outcome	nSNP	MR Egger		Weighted median		Inverse variance weighted		MR_PRESSO	
			OR (95% CI)	pval	OR (95% CI)	pval	OR (95% CI)	pval	OR (95% CI)	pval
1-palmitoyl-2-arachidonoyl-GPE (16:0/20:4)	Hypertension	25	0.992 (0.979 to 1.004)	0.211	0.998 (0.988 to 1.008)	0.710	0.995 (0.988 to 1.002)	0.162		
1-palmitoyl-2-docosahexaenoyl-GPE (16:0/22:6)	Hypertension	44	0.986 (0.971 to 1.001)	0.071	1.003 (0.993 to 1.012)	0.584	1.002 (0.994 to 1.010)	0.610		
1-stearoyl-2-docosahexaenoyl-GPE (18:0/22:6)	Hypertension	36	0.969 (0.941 to 0.998)	0.046	1.004 (0.992 to 1.015)	0.530	1.004 (0.994 to 1.014)	0.473		
1-palmitoyl-2-arachidonoyl-GPI (16:0/20:4)	Hypertension	37	1.016 (0.989 to 1.045)	0.260	1.007 (0.992 to 1.022)	0.369	1.006 (0.995 to 1.017)	0.269		
1-stearoyl-2-linoleoyl-GPI (18:0/18:2)	Hypertension	61	0.991 (0.954 to 1.030)	0.650	1.012 (1.003 to 1.021)	0.010	1.003 (0.994 to 1.011)	0.525	1.006 (0.999 to 1.012)	0.084

Exposure	Outcome	nSNP	MR Egger		Weighted median		Inverse variance weighted		MR_PRESSO	
			OR (95% CI)	pval	OR (95% CI)	pval	OR (95% CI)	pval	OR (95% CI)	pval
1-palmitoyl-2-palmitoleoyl-GPC (16:0/16:1)	Hypertension	20	0.982 (0.943 to 1.022)	0.378	0.999 (0.977 to 1.022)	0.948	0.997 (0.981 to 1.014)	0.753		
gamma-tocopherol/beta-tocopherol	Hypertension	23	1.037 (1.007 to 1.068)	0.026	1.005 (0.985 to 1.025)	0.636	1.001 (0.987 to 1.015)	0.858		
1-(1-enyl-stearoyl)-2-arachidonoyl-GPE (P-18:0/20:4)	Hypertension	43	1.014 (0.983 to 1.046)	0.386	0.989 (0.981 to 0.998)	0.015	0.990 (0.983 to 0.996)	0.002		
1-(1-enyl-stearoyl)-2-docosahexaenoyl-GPE (P-18:0/22:6)	Hypertension	26	0.999 (0.976 to 1.022)	0.918	0.996 (0.980 to 1.012)	0.629	0.993 (0.981 to 1.004)	0.201		
1-(1-enyl-palmitoyl)-2-oleoyl-GPE (P-16:0/18:1)	Hypertension	28	0.985 (0.944 to 1.027)	0.474	0.986 (0.966 to 1.007)	0.192	0.983 (0.965 to 1.001)	0.070	0.981 (0.968 to 0.995)	0.011

Exposure	Outcome	nSNP	MR Egger		Weighted median		Inverse variance weighted		MR_PRESSO	
			OR (95% CI)	pval	OR (95% CI)	pval	OR (95% CI)	pval	OR (95% CI)	pval
1-(1-enyl-palmitoyl)-2-oleoyl-GPC (P-16:0/18:1)	Hypertension	27	1.002 (0.965 to 1.041)	0.911	0.984 (0.964 to 1.003)	0.104	0.993 (0.977 to 1.010)	0.415	0.988 (0.974 to 1.003)	0.139
sphingomyelin (d18:1/21:0, d17:1/22:0, d16:1/23:0)	Hypertension	29	0.972 (0.948 to 0.997)	0.040	0.983 (0.968 to 0.999)	0.039	0.988 (0.977 to 0.999)	0.032		
1-pentadecanoyl-2-linoleoyl-GPC (15:0/18:2)	Hypertension	47	1.001 (0.977 to 1.026)	0.922	1.015 (1.003 to 1.028)	0.012	1.015 (1.007 to 1.024)	5.43e-04		
1-pentadecanoyl-2-oleoyl-GPC (15:0/18:1)	Hypertension	11	0.998 (0.961 to 1.036)	0.914	0.987 (0.961 to 1.014)	0.352	0.993 (0.973 to 1.013)	0.481		
1-margaroyl-2-oleoyl-GPC (17:0/18:1)	Hypertension	17	1.041 (0.972 to 1.116)	0.269	1.004 (0.980 to 1.029)	0.741	1.005 (0.987 to 1.024)	0.556		

Exposure	Outcome	nSNP	MR Egger		Weighted median		Inverse variance weighted		MR_PRESSO	
			OR (95% CI)	pval	OR (95% CI)	pval	OR (95% CI)	pval	OR (95% CI)	pval
1-margaroyl-2-linoleoyl-GPC (17:0/18:2)	Hypertension	30	0.995 (0.963 to 1.027)	0.748	1.017 (1.001 to 1.034)	0.039	1.014 (1.001 to 1.027)	0.040		
X - 24309	Hypertension	35	0.997 (0.990 to 1.004)	0.368	0.998 (0.992 to 1.004)	0.524	1.001 (0.996 to 1.005)	0.807		
2'-deoxyuridine	Hypertension	24	0.985 (0.961 to 1.011)	0.265	1.011 (0.994 to 1.029)	0.193	0.997 (0.984 to 1.010)	0.640		
1,2-dilinoleoyl-GPC (18:2/18:2)	Hypertension	50	1.014 (0.985 to 1.045)	0.351	1.012 (1.002 to 1.021)	0.014	1.010 (1.003 to 1.017)	0.003		
1-palmitoyl-2-docosahexaenoyl-GPC (16:0/22:6)	Hypertension	24	1.010 (0.980 to 1.041)	0.532	0.994 (0.974 to 1.015)	0.586	0.991 (0.977 to 1.006)	0.233		

Exposure	Outcome	nSNP	MR Egger		Weighted median		Inverse variance weighted		MR_PRESSO	
			OR (95% CI)	pval	OR (95% CI)	pval	OR (95% CI)	pval	OR (95% CI)	pval
1-stearoyl-2-docosahexaenoyl-GPC (18:0/22:6)	Hypertension	23	1.017 (0.991 to 1.044)	0.210	0.992 (0.973 to 1.011)	0.405	0.991 (0.977 to 1.004)	0.174		
1-(1-enyl-stearoyl)-2-oleoyl-GPC (P-18:0/18:1)	Hypertension	21	0.976 (0.937 to 1.017)	0.262	0.992 (0.970 to 1.014)	0.472	0.997 (0.976 to 1.019)	0.806	0.988 (0.975 to 1.002)	0.102
1-(1-enyl-stearoyl)-2-arachidonoyl-GPC (P-18:0/20:4)	Hypertension	48	0.981 (0.967 to 0.996)	0.015	0.993 (0.987 to 0.998)	0.014	0.993 (0.988 to 0.997)	7.75e-04		
1-(1-enyl-stearoyl)-2-oleoyl-GPE (P-18:0/18:1)	Hypertension	17	0.990 (0.976 to 1.005)	0.207	0.998 (0.989 to 1.008)	0.742	0.997 (0.990 to 1.005)	0.492		
sphingomyelin (d18:1/17:0, d17:1/18:0, d19:1/16:0)	Hypertension	16	1.009 (0.972 to 1.047)	0.645	1.002 (0.977 to 1.028)	0.863	0.992 (0.972 to 1.012)	0.419		

Exposure	Outcome	nSNP	MR Egger		Weighted median		Inverse variance weighted		MR_PRESSO	
			OR (95% CI)	pval	OR (95% CI)	pval	OR (95% CI)	pval	OR (95% CI)	pval
1-palmitoyl-2-stearoyl-GPC (16:0/18:0)	Hypertension	34	1.002 (0.992 to 1.013)	0.666	1.003 (0.993 to 1.012)	0.563	1.001 (0.995 to 1.008)	0.631		
1-stearoyl-2-dihomo-linolenoyl-GPC (18:0/20:3n3 or 6)	Hypertension	23	0.973 (0.940 to 1.008)	0.145	1.026 (1.005 to 1.047)	0.015	1.011 (0.995 to 1.026)	0.173		
1-(1-enyl-palmitoyl)-2-docosahexaenoyl-GPC (P-16:0/22:6)	Hypertension	26	0.988 (0.968 to 1.009)	0.286	0.994 (0.977 to 1.012)	0.511	0.987 (0.975 to 0.999)	0.038		
palmitoleoyl-oleoyl-glycerol (16:1/18:1) [2]	Hypertension	37	0.984 (0.962 to 1.006)	0.171	1.018 (1.001 to 1.035)	0.038	1.006 (0.994 to 1.018)	0.331		
palmitoleoyl-oleoyl-glycerol (16:1/18:1) [1]	Hypertension	27	1.008 (0.977 to 1.040)	0.634	1.007 (0.987 to 1.027)	0.487	0.999 (0.985 to 1.013)	0.889		

Exposure	Outcome	nSNP	MR Egger		Weighted median		Inverse variance weighted		MR_PRESSO	
			OR (95% CI)	pval	OR (95% CI)	pval	OR (95% CI)	pval	OR (95% CI)	pval
palmitoyl-linoleoyl-glycerol (16:0/18:2) [1]	Hypertension	27	1.005 (0.950 to 1.063)	0.872	1.016 (0.997 to 1.036)	0.098	1.001 (0.978 to 1.024)	0.960	1.022 (1.007 to 1.036)	0.007
palmitoyl-linoleoyl-glycerol (16:0/18:2) [2]	Hypertension	22	1.003 (0.969 to 1.039)	0.858	1.005 (0.994 to 1.016)	0.365	0.994 (0.980 to 1.008)	0.412	1.002 (0.992 to 1.013)	0.680
X - 24425	Hypertension	16	0.999 (0.967 to 1.032)	0.971	0.989 (0.968 to 1.010)	0.312	0.990 (0.972 to 1.007)	0.248		
1-palmitoyl-2-oleoyl-GPI (16:0/18:1)	Hypertension	33	0.977 (0.944 to 1.012)	0.208	1.016 (1.002 to 1.031)	0.026	1.004 (0.995 to 1.014)	0.383		
1-(1-enyl-palmitoyl)-2-docosahexaenoyl-GPE (P-16:0/22:6)	Hypertension	31	0.986 (0.964 to 1.008)	0.214	0.991 (0.975 to 1.007)	0.282	0.991 (0.981 to 1.002)	0.106		

Exposure	Outcome	nSNP	MR Egger		Weighted median		Inverse variance weighted		MR_PRESSO	
			OR (95% CI)	pval	OR (95% CI)	pval	OR (95% CI)	pval	OR (95% CI)	pval
1-(1-enyl-palmitoyl)-2-arachidonoyl-GPE (P-16:0/20:4)	Hypertension	50	0.998 (0.976 to 1.021)	0.896	0.988 (0.980 to 0.997)	0.012	0.988 (0.981 to 0.995)	3.56e-04		
1-(1-enyl-palmitoyl)-2-linoleoyl-GPE (P-16:0/18:2)	Hypertension	49	1.005 (0.977 to 1.033)	0.737	1.016 (1.004 to 1.028)	0.007	1.009 (1.000 to 1.017)	0.043		
1-(1-enyl-palmitoyl)-2-linoleoyl-GPC (P-16:0/18:2)	Hypertension	16	1.039 (0.997 to 1.082)	0.088	0.998 (0.974 to 1.022)	0.850	0.996 (0.976 to 1.016)	0.675		
1-palmitoleoyl-2-linoleoyl-GPC (16:1/18:2)	Hypertension	44	0.983 (0.956 to 1.011)	0.247	1.010 (1.002 to 1.019)	0.017	1.008 (1.002 to 1.014)	0.007		
1-palmitoyl-2-alpha-linolenoyl-GPC (16:0/18:3n3)	Hypertension	50	1.004 (0.973 to 1.035)	0.818	1.011 (1.003 to 1.019)	0.010	1.009 (1.003 to 1.015)	0.005		

Exposure	Outcome	nSNP	MR Egger		Weighted median		Inverse variance weighted		MR_PRESSO	
			OR (95% CI)	pval	OR (95% CI)	pval	OR (95% CI)	pval	OR (95% CI)	pval
1-(1-enyl-palmitoyl)-2-arachidonoyl-GPC (P-16:0/20:4)	Hypertension	53	0.990 (0.977 to 1.003)	0.135	0.993 (0.987 to 0.999)	0.013	0.993 (0.989 to 0.997)	4.63e-04		
1-oleoyl-2-docosahexaenoyl-GPC (18:1/22:6)	Hypertension	18	1.006 (0.962 to 1.051)	0.809	0.998 (0.973 to 1.023)	0.861	1.000 (0.978 to 1.022)	0.971		
1-adrenoyl-GPC (22:4)	Hypertension	48	0.992 (0.968 to 1.016)	0.524	0.987 (0.978 to 0.996)	0.003	0.988 (0.981 to 0.995)	4.94e-04		
1-stearoyl-2-docosapentaenoyl-GPC (18:0/22:5n3)	Hypertension	47	0.963 (0.928 to 1.000)	0.056	0.988 (0.979 to 0.997)	0.011	0.992 (0.984 to 1.000)	0.055		
lactate	Hypertension	20	1.020 (0.964 to 1.079)	0.510	0.987 (0.964 to 1.010)	0.264	0.983 (0.956 to 1.011)	0.237	0.990 (0.976 to 1.005)	0.206

Exposure	Outcome	nSNP	MR Egger		Weighted median		Inverse variance weighted		MR_PRESSO	
			OR (95% CI)	pval	OR (95% CI)	pval	OR (95% CI)	pval	OR (95% CI)	pval
1-stearoyl-2-docosapentaenoyl-GPC (18:0/22:5n6)	Hypertension	46	0.989 (0.961 to 1.017)	0.432	0.986 (0.975 to 0.997)	0.009	0.988 (0.980 to 0.996)	0.002		
1-(1-enyl-stearoyl)-GPC (P-18:0)	Hypertension	15	1.012 (0.968 to 1.059)	0.605	1.008 (0.986 to 1.031)	0.492	1.009 (0.992 to 1.027)	0.310		
1-stearyl-GPC (O-18:0)	Hypertension	14	0.959 (0.898 to 1.025)	0.242	1.009 (0.983 to 1.036)	0.484	1.019 (0.987 to 1.051)	0.247	1.017 (0.999 to 1.036)	0.093
1-(1-enyl-stearoyl)-2-docosahexaenoyl-GPC (P-18:0/22:6)	Hypertension	21	0.994 (0.955 to 1.034)	0.758	0.994 (0.971 to 1.018)	0.618	0.982 (0.965 to 1.000)	0.044		
1-oleoyl-2-dihomo-linolenoyl-GPC (18:1/20:3)	Hypertension	42	1.000 (0.970 to 1.031)	0.997	1.014 (1.005 to 1.024)	0.002	1.015 (1.008 to 1.022)	4.78e-05		

Exposure	Outcome	nSNP	MR Egger		Weighted median		Inverse variance weighted		MR_PRESSO	
			OR (95% CI)	pval	OR (95% CI)	pval	OR (95% CI)	pval	OR (95% CI)	pval
1-palmitoyl-2-adrenoyl-GPC (16:0/22:4)	Hypertension	15	0.990 (0.955 to 1.026)	0.584	0.976 (0.950 to 1.002)	0.075	0.988 (0.971 to 1.006)	0.205		
1-myristoyl-2-linoleoyl-GPC (14:0/18:2)	Hypertension	50	1.010 (0.980 to 1.041)	0.516	1.016 (1.004 to 1.027)	0.006	1.011 (1.002 to 1.019)	0.012		
1-linoleoyl-2-arachidonoyl-GPC (18:2/20:4n6)	Hypertension	43	0.967 (0.945 to 0.990)	0.007	0.991 (0.985 to 0.998)	0.008	0.991 (0.986 to 0.996)	4.71e-04		
1-oleoyl-2-eicosapentaenoyl-GPC (18:1/20:5)	Hypertension	48	0.997 (0.974 to 1.020)	0.796	0.986 (0.976 to 0.996)	0.008	0.991 (0.983 to 0.998)	0.014		
1-(1-enyl-palmitoyl)-2-palmitoyl-GPC (P-16:0/16:0)	Hypertension	18	0.994 (0.966 to 1.023)	0.681	0.986 (0.968 to 1.004)	0.129	0.984 (0.971 to 0.996)	0.012		

Exposure	Outcome	nSNP	MR Egger		Weighted median		Inverse variance weighted		MR_PRESSO	
			OR (95% CI)	pval	OR (95% CI)	pval	OR (95% CI)	pval	OR (95% CI)	pval
phosphatidylcholine (16:0/22:5n3, 18:1/20:4)	Hypertension	51	0.971 (0.958 to 0.984)	7.35e-05	0.993 (0.988 to 0.999)	0.015	0.995 (0.991 to 0.999)	0.011		
phosphatidylcholine (18:0/20:5, 16:0/22:5n6)	Hypertension	58	0.981 (0.960 to 1.003)	0.101	0.990 (0.982 to 0.998)	0.014	0.992 (0.986 to 0.998)	0.012		
1-palmitoyl-2-meadoyl-GPC (16:0/20:3n9)	Hypertension	45	0.974 (0.945 to 1.005)	0.104	0.990 (0.983 to 0.998)	0.014	0.992 (0.986 to 0.997)	0.003		
1-stearoyl-2-meadoyl-GPC (18:0/20:3n9)	Hypertension	40	0.972 (0.936 to 1.009)	0.144	0.991 (0.984 to 0.998)	0.013	0.991 (0.985 to 0.996)	4.39e-04		
glutamine	Hypertension	18	0.968 (0.912 to 1.029)	0.311	0.987 (0.966 to 1.008)	0.211	0.990 (0.973 to 1.008)	0.294		

Exposure	Outcome	nSNP	MR Egger		Weighted median		Inverse variance weighted		MR_PRESSO	
			OR (95% CI)	pval	OR (95% CI)	pval	OR (95% CI)	pval	OR (95% CI)	pval
3-hydroxy-3-methylglutarate	Hypertension	13	1.005 (0.955 to 1.058)	0.854	1.007 (0.976 to 1.039)	0.658	1.007 (0.986 to 1.028)	0.510		
tryptophan	Hypertension	20	1.014 (0.989 to 1.039)	0.292	1.015 (0.994 to 1.035)	0.156	1.010 (0.994 to 1.026)	0.219		
beta-alanine	Hypertension	17	1.007 (0.983 to 1.032)	0.589	0.997 (0.980 to 1.015)	0.741	1.004 (0.993 to 1.016)	0.467		
adenosine	Hypertension	33	0.990 (0.976 to 1.004)	0.178	0.996 (0.990 to 1.003)	0.295	0.997 (0.992 to 1.002)	0.233		
caffeine	Hypertension	30	0.994 (0.961 to 1.028)	0.714	0.982 (0.962 to 1.002)	0.078	0.989 (0.972 to 1.007)	0.219	0.984 (0.969 to 0.998)	0.039

Exposure	Outcome	nSNP	MR Egger		Weighted median		Inverse variance weighted		MR_PRESSO	
			OR (95% CI)	pval	OR (95% CI)	pval	OR (95% CI)	pval	OR (95% CI)	pval
glutamate	Hypertension	14	1.003 (0.966 to 1.041)	0.884	0.999 (0.978 to 1.021)	0.925	1.002 (0.986 to 1.019)	0.767		
glycine	Hypertension	13	0.996 (0.931 to 1.065)	0.908	1.014 (0.991 to 1.039)	0.238	1.008 (0.984 to 1.033)	0.514	1.015 (0.992 to 1.039)	0.232
gluconate	Hypertension	18	0.983 (0.938 to 1.031)	0.498	0.992 (0.968 to 1.016)	0.508	1.001 (0.980 to 1.024)	0.894	0.993 (0.974 to 1.012)	0.485
histidine	Hypertension	16	1.007 (0.972 to 1.043)	0.713	1.013 (0.988 to 1.038)	0.323	1.019 (1.001 to 1.036)	0.036		
nicotinamide	Hypertension	16	0.992 (0.955 to 1.029)	0.667	1.005 (0.984 to 1.027)	0.638	0.991 (0.974 to 1.009)	0.310		

Exposure	Outcome	nSNP	MR Egger		Weighted median		Inverse variance weighted		MR_PRESSO	
			OR (95% CI)	pval	OR (95% CI)	pval	OR (95% CI)	pval	OR (95% CI)	pval
dihydroorotate	Hypertension	26	0.989 (0.973 to 1.005)	0.189	0.995 (0.987 to 1.004)	0.318	0.996 (0.986 to 1.006)	0.459	0.994 (0.987 to 1.001)	0.094
uridine	Hypertension	15	0.955 (0.887 to 1.028)	0.243	0.996 (0.971 to 1.021)	0.749	1.005 (0.984 to 1.027)	0.623		
trans-urocanate	Hypertension	14	1.022 (0.977 to 1.068)	0.359	1.003 (0.979 to 1.028)	0.794	0.999 (0.977 to 1.022)	0.935	0.998 (0.978 to 1.019)	0.872
2-aminoadipate	Hypertension	28	1.014 (0.981 to 1.049)	0.415	1.003 (0.984 to 1.022)	0.781	0.999 (0.981 to 1.017)	0.910	0.996 (0.983 to 1.010)	0.596
cholesterol	Hypertension	16	0.984 (0.939 to 1.030)	0.496	0.993 (0.967 to 1.020)	0.626	0.995 (0.972 to 1.017)	0.633		

Exposure	Outcome	nSNP	MR Egger		Weighted median		Inverse variance weighted		MR_PRESSO	
			OR (95% CI)	pval	OR (95% CI)	pval	OR (95% CI)	pval	OR (95% CI)	pval
Carnitine	Hypertension	16	1.018 (0.977 to 1.060)	0.405	1.017 (0.986 to 1.048)	0.289	1.016 (0.992 to 1.040)	0.199		
Benzoate	Hypertension	9	0.937 (0.910 to 0.965)	0.003	0.957 (0.924 to 0.991)	0.014	0.963 (0.934 to 0.992)	0.014		
Maltotriose	Hypertension	15	1.003 (0.949 to 1.061)	0.910	1.025 (0.985 to 1.067)	0.216	1.037 (1.007 to 1.068)	0.015		
Hippurate	Hypertension	9	1.007 (0.877 to 1.157)	0.920	1.035 (0.970 to 1.104)	0.302	1.009 (0.945 to 1.078)	0.780		
Methionine sulfoxide	Hypertension	10	0.914 (0.735 to 1.136)	0.441	1.050 (0.970 to 1.136)	0.231	1.043 (0.959 to 1.135)	0.329	1.073 (1.001 to 1.149)	0.086

Exposure	Outcome	nSNP	MR Egger		Weighted median		Inverse variance weighted		MR_PRESSO	
			OR (95% CI)	pval	OR (95% CI)	pval	OR (95% CI)	pval	OR (95% CI)	pval
Xanthurenate	Hypertension	17	1.021 (0.927 to 1.125)	0.684	1.007 (0.963 to 1.053)	0.768	1.014 (0.982 to 1.048)	0.384		
N-alpha-acetylornithine	Hypertension	15	1.004 (0.930 to 1.084)	0.923	1.030 (0.987 to 1.074)	0.172	1.020 (0.985 to 1.056)	0.266		
3-methylhistidine	Hypertension	15	1.037 (0.880 to 1.222)	0.673	1.029 (0.966 to 1.096)	0.373	1.007 (0.950 to 1.068)	0.803		
Glycerophosphorylcholine (GPC)	Hypertension	12	0.963 (0.890 to 1.042)	0.368	0.995 (0.948 to 1.045)	0.849	1.004 (0.968 to 1.040)	0.837		
Phenylacetate	Hypertension	14	0.996 (0.928 to 1.068)	0.904	0.966 (0.921 to 1.014)	0.161	0.988 (0.951 to 1.026)	0.529		

Exposure	Outcome	nSNP	MR Egger		Weighted median		Inverse variance weighted		MR_PRESSO	
			OR (95% CI)	pval	OR (95% CI)	pval	OR (95% CI)	pval	OR (95% CI)	pval
3-methyl-2-oxovalerate	Hypertension	8	1.001 (0.810 to 1.238)	0.991	1.088 (1.001 to 1.181)	0.047	1.084 (1.007 to 1.166)	0.032		
Tartarate	Hypertension	14	1.013 (0.926 to 1.107)	0.787	0.961 (0.915 to 1.010)	0.115	0.995 (0.950 to 1.043)	0.833	0.979 (0.944 to 1.015)	0.265
Suberate (C8-DC)	Hypertension	14	1.034 (0.958 to 1.115)	0.413	1.005 (0.956 to 1.055)	0.855	0.999 (0.961 to 1.040)	0.978		
5-hydroxylysine	Hypertension	12	1.001 (0.959 to 1.044)	0.978	1.011 (0.978 to 1.045)	0.528	1.030 (0.998 to 1.062)	0.063		
3-phenylpropionate hydrocinnamate	Hypertension	17	1.015 (0.943 to 1.093)	0.698	0.985 (0.943 to 1.028)	0.479	0.995 (0.953 to 1.039)	0.827	0.982 (0.948 to 1.018)	0.338

Exposure	Outcome	nSNP	MR Egger		Weighted median		Inverse variance weighted		MR_PRESSO	
			OR (95% CI)	pval	OR (95% CI)	pval	OR (95% CI)	pval	OR (95% CI)	pval
Kynurenine	Hypertension	14	0.846 (0.593 to 1.206)	0.373	1.011 (0.966 to 1.058)	0.642	1.090 (0.981 to 1.212)	0.109	1.060 (0.998 to 1.126)	0.094
N-acetylglutamate	Hypertension	12	0.969 (0.808 to 1.162)	0.741	1.010 (0.944 to 1.080)	0.776	1.021 (0.957 to 1.089)	0.528	0.990 (0.939 to 1.045)	0.725
Glycerol 3-phosphate	Hypertension	18	1.007 (0.951 to 1.066)	0.820	0.993 (0.955 to 1.034)	0.746	0.998 (0.965 to 1.033)	0.916		
Imidazole lactate	Hypertension	17	1.014 (0.969 to 1.061)	0.567	1.054 (1.022 to 1.088)	0.001	1.025 (0.996 to 1.055)	0.096	1.019 (0.980 to 1.058)	0.363
4-guanidinobutanoate	Hypertension	11	1.029 (0.889 to 1.191)	0.713	1.063 (0.990 to 1.141)	0.095	1.028 (0.963 to 1.097)	0.413	1.037 (0.985 to 1.092)	0.196

Exposure	Outcome	nSNP	MR Egger		Weighted median		Inverse variance weighted		MR_PRESSO	
			OR (95% CI)	pval	OR (95% CI)	pval	OR (95% CI)	pval	OR (95% CI)	pval
Glucuronate	Hypertension	8	1.029 (0.782 to 1.353)	0.847	1.036 (0.940 to 1.141)	0.478	1.044 (0.928 to 1.176)	0.473	1.060 (0.953 to 1.179)	0.343
Ribitol	Hypertension	18	1.016 (0.990 to 1.044)	0.250	1.011 (0.988 to 1.035)	0.359	1.019 (1.000 to 1.039)	0.045		
Glycodeoxycholate	Hypertension	6	1.127 (1.018 to 1.248)	0.082	1.060 (0.986 to 1.139)	0.115	1.038 (0.974 to 1.107)	0.252		
Theobromine	Hypertension	13	1.102 (0.927 to 1.311)	0.296	0.998 (0.942 to 1.058)	0.954	0.955 (0.897 to 1.018)	0.160	0.981 (0.936 to 1.028)	0.438
Quinate	Hypertension	13	0.971 (0.925 to 1.019)	0.253	0.974 (0.943 to 1.007)	0.117	0.970 (0.936 to 1.005)	0.093	0.964 (0.939 to 0.989)	0.018

Exposure	Outcome	nSNP	MR Egger		Weighted median		Inverse variance weighted		MR_PRESSO	
			OR (95% CI)	pval	OR (95% CI)	pval	OR (95% CI)	pval	OR (95% CI)	pval
Gentisate	Hypertension	20	1.025 (0.928 to 1.132)	0.633	1.025 (0.974 to 1.077)	0.343	1.023 (0.978 to 1.070)	0.325		
Theophylline	Hypertension	12	1.029 (0.929 to 1.141)	0.593	1.049 (0.987 to 1.115)	0.123	1.070 (1.018 to 1.124)	0.008		
1,5-anhydroglucitol (1,5-ag)	Hypertension	22	1.016 (0.956 to 1.080)	0.613	1.023 (0.988 to 1.059)	0.209	1.025 (0.987 to 1.064)	0.201	1.034 (1.007 to 1.061)	0.022
1-stearoyl-2-arachidonoyl-GPI (18:0/20:4)	Hypertension	16	0.943 (0.824 to 1.078)	0.403	0.973 (0.937 to 1.010)	0.156	0.987 (0.932 to 1.046)	0.669	0.973 (0.940 to 1.007)	0.137
Indolelactate	Hypertension	12	1.054 (0.918 to 1.209)	0.474	1.066 (1.005 to 1.130)	0.032	1.033 (0.970 to 1.100)	0.309	1.025 (0.975 to 1.077)	0.363

Exposure	Outcome	nSNP	MR Egger		Weighted median		Inverse variance weighted		MR_PRESSO	
			OR (95% CI)	pval	OR (95% CI)	pval	OR (95% CI)	pval	OR (95% CI)	pval
Docosahexaenoate DHA; 22:6n3	Hypertension	10	0.992 (0.930 to 1.059)	0.817	1.013 (0.962 to 1.066)	0.631	1.028 (0.989 to 1.068)	0.159		
1-stearoyl-2-oleoyl-GPS (18:0/18:1)	Hypertension	9	0.946 (0.737 to 1.213)	0.674	0.998 (0.927 to 1.075)	0.963	1.001 (0.924 to 1.083)	0.986		
1-myristoyl-2-palmitoyl-gpc (14:0/16:0)	Hypertension	8	0.924 (0.829 to 1.030)	0.203	0.951 (0.892 to 1.014)	0.123	0.957 (0.911 to 1.006)	0.086		
Paraxanthine	Hypertension	17	1.004 (0.921 to 1.095)	0.922	0.999 (0.955 to 1.044)	0.954	1.020 (0.981 to 1.061)	0.324		
4-methyl-2-oxopentanoate	Hypertension	10	1.026 (0.875 to 1.203)	0.762	1.051 (0.981 to 1.127)	0.160	1.046 (0.995 to 1.100)	0.075		

Exposure	Outcome	nSNP	MR Egger		Weighted median		Inverse variance weighted		MR_PRESSO	
			OR (95% CI)	pval	OR (95% CI)	pval	OR (95% CI)	pval	OR (95% CI)	pval
3-indoxyl sulfate	Hypertension	10	0.968 (0.867 to 1.080)	0.576	0.986 (0.927 to 1.049)	0.654	0.985 (0.936 to 1.036)	0.557		
Sphingosine 1-phosphate	Hypertension	12	0.919 (0.853 to 0.990)	0.050	0.896 (0.848 to 0.946)	8.39e-05	0.926 (0.888 to 0.966)	3.42e-04		
Alpha-hydroxyisocaproate	Hypertension	14	1.098 (0.904 to 1.333)	0.364	0.997 (0.937 to 1.061)	0.926	1.017 (0.953 to 1.086)	0.608	1.041 (0.988 to 1.097)	0.155
1-stearoyl-GPI (18:0)	Hypertension	11	0.906 (0.803 to 1.024)	0.148	1.052 (0.981 to 1.127)	0.154	1.023 (0.977 to 1.072)	0.328		
1,2-dipalmitoyl-gpc (16:0/16:0)	Hypertension	14	1.049 (0.971 to 1.133)	0.249	1.027 (0.985 to 1.070)	0.208	1.041 (0.995 to 1.088)	0.080	1.017 (0.989 to 1.047)	0.262

Exposure	Outcome	nSNP	MR Egger		Weighted median		Inverse variance weighted		MR_PRESSO	
			OR (95% CI)	pval	OR (95% CI)	pval	OR (95% CI)	pval	OR (95% CI)	pval
Maleate	Hypertension	12	1.185 (1.053 to 1.333)	0.018	1.046 (0.979 to 1.117)	0.180	1.064 (1.002 to 1.128)	0.041		
Isovalerate (i5:0)	Hypertension	10	0.901 (0.793 to 1.025)	0.151	0.975 (0.913 to 1.041)	0.449	0.987 (0.931 to 1.047)	0.670		
4-acetylphenol sulfate	Hypertension	10	1.026 (0.952 to 1.106)	0.517	1.006 (0.960 to 1.055)	0.791	0.986 (0.944 to 1.029)	0.513		
Phenyllactate (PLA)	Hypertension	20	1.026 (0.934 to 1.127)	0.598	0.960 (0.916 to 1.005)	0.082	0.995 (0.947 to 1.046)	0.844	0.984 (0.942 to 1.028)	0.485
N-acetylaspartate (naa)	Hypertension	7	0.938 (0.857 to 1.028)	0.231	0.984 (0.929 to 1.043)	0.590	1.004 (0.948 to 1.063)	0.904		

Exposure	Outcome	nSNP	MR Egger		Weighted median		Inverse variance weighted		MR_PRESSO	
			OR (95% CI)	pval	OR (95% CI)	pval	OR (95% CI)	pval	OR (95% CI)	pval
Palmitoylcarnitine	Hypertension	17	1.043 (0.973 to 1.119)	0.255	1.030 (0.986 to 1.076)	0.184	1.016 (0.981 to 1.052)	0.380		
Erythritol	Hypertension	17	1.010 (0.926 to 1.102)	0.818	0.997 (0.948 to 1.048)	0.900	1.012 (0.969 to 1.056)	0.605	0.966 (0.932 to 1.001)	0.076
Hexanoylcarnitine	Hypertension	14	1.020 (0.979 to 1.063)	0.366	1.022 (0.995 to 1.050)	0.114	1.015 (0.993 to 1.038)	0.179		
Acetylcarnitine	Hypertension	12	1.000 (0.905 to 1.106)	0.994	1.036 (0.979 to 1.097)	0.218	1.024 (0.980 to 1.071)	0.292		
Iminodiacetate (IDA)	Hypertension	10	1.054 (0.885 to 1.255)	0.570	1.062 (0.995 to 1.134)	0.069	1.041 (0.980 to 1.106)	0.189		

Exposure	Outcome	nSNP	MR Egger		Weighted median		Inverse variance weighted		MR_PRESSO	
			OR (95% CI)	pval	OR (95% CI)	pval	OR (95% CI)	pval	OR (95% CI)	pval
Cysteine s-sulfate	Hypertension	10	0.970 (0.881 to 1.068)	0.553	0.956 (0.894 to 1.022)	0.183	0.988 (0.941 to 1.037)	0.615		
Oxalate (ethanedioate)	Hypertension	16	0.990 (0.892 to 1.099)	0.850	0.982 (0.936 to 1.031)	0.474	0.993 (0.939 to 1.051)	0.815	0.973 (0.941 to 1.007)	0.137
DHEAS	Hypertension	28	0.967 (0.882 to 1.060)	0.480	0.961 (0.921 to 1.002)	0.061	0.988 (0.938 to 1.041)	0.652	0.973 (0.943 to 1.004)	0.096
3-hydroxymyristate	Hypertension	8	0.851 (0.739 to 0.980)	0.066	1.014 (0.926 to 1.111)	0.762	1.001 (0.916 to 1.094)	0.978	0.968 (0.898 to 1.042)	0.419
Homoarginine	Hypertension	18	0.962 (0.898 to 1.030)	0.284	0.971 (0.935 to 1.008)	0.126	1.009 (0.973 to 1.046)	0.623	1.005 (0.960 to 1.051)	0.838

Exposure	Outcome	nSNP	MR Egger		Weighted median		Inverse variance weighted		MR_PRESSO	
			OR (95% CI)	pval	OR (95% CI)	pval	OR (95% CI)	pval	OR (95% CI)	pval
1-oleoylglycerol (18:1)	Hypertension	19	1.019 (0.919 to 1.130)	0.726	0.988 (0.943 to 1.036)	0.620	1.006 (0.954 to 1.061)	0.816	0.976 (0.939 to 1.014)	0.227
3-methyl-2-oxobutyrate	Hypertension	13	1.012 (0.874 to 1.171)	0.879	1.038 (0.988 to 1.091)	0.137	1.047 (0.994 to 1.103)	0.084	1.064 (1.022 to 1.107)	0.011
Tartronate (hydroxymalonate)	Hypertension	13	1.015 (0.944 to 1.092)	0.687	1.015 (0.983 to 1.049)	0.367	1.010 (0.956 to 1.068)	0.722	1.010 (0.986 to 1.034)	0.433
2,3-dihydroxypyridine	Hypertension	12	1.142 (0.942 to 1.385)	0.207	1.014 (0.950 to 1.082)	0.683	1.027 (0.976 to 1.082)	0.305		
3-hydroxyoctanoate	Hypertension	14	1.032 (0.971 to 1.097)	0.334	1.045 (0.993 to 1.099)	0.091	1.035 (1.001 to 1.071)	0.046		

Exposure	Outcome	nSNP	MR Egger		Weighted median		Inverse variance weighted		MR_PRESSO	
			OR (95% CI)	pval	OR (95% CI)	pval	OR (95% CI)	pval	OR (95% CI)	pval
2-hydroxyoctanoate	Hypertension	15	1.006 (0.956 to 1.059)	0.825	0.997 (0.963 to 1.031)	0.851	0.982 (0.949 to 1.016)	0.285	0.996 (0.965 to 1.027)	0.794
Homocitrulline	Hypertension	16	1.035 (0.934 to 1.146)	0.520	1.027 (0.986 to 1.069)	0.196	1.029 (0.979 to 1.080)	0.258	1.027 (0.989 to 1.066)	0.193
Indoleacetate	Hypertension	20	0.984 (0.910 to 1.064)	0.688	1.000 (0.956 to 1.046)	0.994	1.007 (0.974 to 1.042)	0.670		
1-linoleoylglycerol (18:2)	Hypertension	7	0.735 (0.593 to 0.911)	0.037	1.071 (0.964 to 1.191)	0.201	1.139 (0.992 to 1.307)	0.065	1.078 (0.993 to 1.170)	0.149
2-linoleoylglycerol (18:2)	Hypertension	11	1.097 (0.922 to 1.306)	0.325	0.968 (0.903 to 1.039)	0.371	0.988 (0.919 to 1.061)	0.734		

Exposure	Outcome	nSNP	MR Egger		Weighted median		Inverse variance weighted		MR_PRESSO	
			OR (95% CI)	pval	OR (95% CI)	pval	OR (95% CI)	pval	OR (95% CI)	pval
Indolepropionate	Hypertension	7	1.073 (0.898 to 1.282)	0.473	0.988 (0.932 to 1.047)	0.675	0.960 (0.888 to 1.037)	0.298	0.960 (0.908 to 1.016)	0.231
Threonate	Hypertension	13	0.990 (0.896 to 1.092)	0.839	1.029 (0.982 to 1.079)	0.232	1.019 (0.969 to 1.070)	0.467	1.041 (1.003 to 1.081)	0.063
N-acetylglycine	Hypertension	14	0.939 (0.895 to 0.985)	0.023	0.951 (0.923 to 0.979)	7.76e-04	0.958 (0.932 to 0.985)	0.002		
Glycolithocholate	Hypertension	12	0.989 (0.928 to 1.054)	0.748	1.007 (0.963 to 1.054)	0.749	1.004 (0.970 to 1.039)	0.825		
EDTA	Hypertension	8	1.083 (0.991 to 1.183)	0.130	1.019 (0.948 to 1.095)	0.613	0.990 (0.929 to 1.056)	0.770		

Exposure	Outcome	nSNP	MR Egger		Weighted median		Inverse variance weighted		MR_PRESSO	
			OR (95% CI)	pval	OR (95% CI)	pval	OR (95% CI)	pval	OR (95% CI)	pval
1-methylhistidine	Hypertension	16	0.905 (0.793 to 1.033)	0.161	0.998 (0.948 to 1.050)	0.930	1.012 (0.962 to 1.065)	0.638	0.996 (0.958 to 1.035)	0.831
Citramalate	Hypertension	11	0.969 (0.795 to 1.183)	0.767	0.973 (0.910 to 1.040)	0.421	0.971 (0.899 to 1.048)	0.445	0.946 (0.890 to 1.004)	0.102
3-hydroxydecanoate	Hypertension	10	0.965 (0.857 to 1.087)	0.575	0.967 (0.922 to 1.014)	0.163	0.992 (0.934 to 1.053)	0.784	0.971 (0.936 to 1.006)	0.143
1,3-dimethylurate	Hypertension	13	1.014 (0.908 to 1.133)	0.805	1.037 (0.984 to 1.093)	0.178	1.030 (0.982 to 1.080)	0.222		
Galactonate	Hypertension	13	0.909 (0.805 to 1.027)	0.153	1.012 (0.952 to 1.077)	0.699	1.031 (0.974 to 1.091)	0.290	1.015 (0.974 to 1.057)	0.496

Exposure	Outcome	nSNP	MR Egger		Weighted median		Inverse variance weighted		MR_PRESSO	
			OR (95% CI)	pval	OR (95% CI)	pval	OR (95% CI)	pval	OR (95% CI)	pval
Trigonelline	Hypertension	11	1.010 (0.911 to 1.120)	0.849	0.972 (0.930 to 1.015)	0.200	0.983 (0.940 to 1.028)	0.457		
Dodecanedioate	Hypertension	16	0.984 (0.898 to 1.080)	0.743	0.991 (0.942 to 1.042)	0.718	0.985 (0.949 to 1.023)	0.427		
Isobutyrylcarnitine (c4)	Hypertension	24	0.997 (0.945 to 1.051)	0.904	0.984 (0.952 to 1.018)	0.350	0.996 (0.965 to 1.028)	0.807		
3-methylxanthine	Hypertension	16	0.933 (0.814 to 1.070)	0.337	0.922 (0.873 to 0.974)	0.004	0.941 (0.891 to 0.995)	0.032	0.923 (0.887 to 0.961)	0.002
3-hydroxylaurate	Hypertension	8	0.935 (0.833 to 1.049)	0.296	1.036 (0.972 to 1.105)	0.275	1.020 (0.957 to 1.087)	0.544		

Exposure	Outcome	nSNP	MR Egger		Weighted median		Inverse variance weighted		MR_PRESSO	
			OR (95% CI)	pval	OR (95% CI)	pval	OR (95% CI)	pval	OR (95% CI)	pval
Pyridoxate	Hypertension	16	0.991 (0.918 to 1.070)	0.825	0.980 (0.935 to 1.028)	0.416	0.971 (0.927 to 1.018)	0.226	0.986 (0.950 to 1.023)	0.465
5-hydroxyhexanoate	Hypertension	9	0.941 (0.822 to 1.077)	0.407	0.990 (0.921 to 1.064)	0.788	0.999 (0.946 to 1.056)	0.985		
Gamma-glutamylvaline	Hypertension	17	1.000 (0.964 to 1.036)	0.987	0.994 (0.970 to 1.018)	0.607	1.013 (0.983 to 1.045)	0.402		
3-hydroxysebacate	Hypertension	12	1.146 (0.987 to 1.331)	0.104	1.021 (0.970 to 1.076)	0.424	0.997 (0.922 to 1.077)	0.931	0.996 (0.944 to 1.050)	0.874
Propionylglycine	Hypertension	15	0.955 (0.908 to 1.004)	0.097	0.948 (0.919 to 0.977)	5.81e-04	0.960 (0.935 to 0.986)	0.003		

Exposure	Outcome	nSNP	MR Egger		Weighted median		Inverse variance weighted		MR_PRESSO	
			OR (95% CI)	pval	OR (95% CI)	pval	OR (95% CI)	pval	OR (95% CI)	pval
Butyrylglycine	Hypertension	13	0.989 (0.955 to 1.025)	0.561	0.985 (0.963 to 1.007)	0.175	0.976 (0.955 to 0.998)	0.034		
Pro-hydroxy-pro	Hypertension	14	1.034 (0.922 to 1.161)	0.577	0.971 (0.919 to 1.025)	0.289	0.997 (0.941 to 1.057)	0.924	0.975 (0.929 to 1.023)	0.323
Propionylcarnitine (c3)	Hypertension	13	0.991 (0.909 to 1.081)	0.850	1.003 (0.955 to 1.052)	0.918	1.006 (0.961 to 1.054)	0.783	0.998 (0.960 to 1.037)	0.906
Adrenate (22:4n6)	Hypertension	10	0.999 (0.887 to 1.124)	0.982	1.031 (0.977 to 1.088)	0.262	1.043 (0.981 to 1.110)	0.179	1.028 (0.979 to 1.079)	0.300
Docosatrienoate (22:3n3)	Hypertension	14	0.983 (0.886 to 1.091)	0.754	1.026 (0.977 to 1.078)	0.304	1.011 (0.967 to 1.057)	0.640		

Exposure	Outcome	nSNP	MR Egger		Weighted median		Inverse variance weighted		MR_PRESSO	
			OR (95% CI)	pval	OR (95% CI)	pval	OR (95% CI)	pval	OR (95% CI)	pval
Docosadienoate (22:2n6)	Hypertension	10	0.965 (0.839 to 1.109)	0.628	0.979 (0.924 to 1.038)	0.478	0.982 (0.937 to 1.029)	0.449		
3-carboxy-4-methyl-5-propyl-2-furanpropanoate (cmpf)	Hypertension	9	0.987 (0.827 to 1.178)	0.885	1.043 (0.967 to 1.126)	0.276	0.998 (0.927 to 1.074)	0.954		
Docosapentaenoate n3 DPA; 22:5n3	Hypertension	12	1.033 (0.906 to 1.177)	0.640	1.012 (0.954 to 1.073)	0.693	1.000 (0.950 to 1.053)	1.000		
3-hydroxy-2-ethylpropionate	Hypertension	21	0.925 (0.807 to 1.060)	0.278	1.022 (0.974 to 1.072)	0.385	1.041 (0.986 to 1.100)	0.146	1.025 (0.986 to 1.066)	0.231
Myristoleate (14:1n5)	Hypertension	12	0.947 (0.780 to 1.148)	0.590	0.994 (0.931 to 1.060)	0.844	0.989 (0.926 to 1.057)	0.747	0.966 (0.916 to 1.018)	0.221

Exposure	Outcome	nSNP	MR Egger		Weighted median		Inverse variance weighted		MR_PRESSO	
			OR (95% CI)	pval	OR (95% CI)	pval	OR (95% CI)	pval	OR (95% CI)	pval
10-undecenoate (11:1n1)	Hypertension	12	0.970 (0.931 to 1.010)	0.173	0.983 (0.951 to 1.016)	0.301	0.984 (0.957 to 1.012)	0.261		
1-methyl-4-imidazoleacetate	Hypertension	17	0.987 (0.941 to 1.035)	0.588	1.000 (0.970 to 1.032)	0.987	1.008 (0.983 to 1.034)	0.521		
Sebacate (C10-DC)	Hypertension	9	1.004 (0.991 to 1.016)	0.592	1.004 (0.991 to 1.018)	0.517	1.005 (0.994 to 1.017)	0.350		
Tauro-beta-muricholate	Hypertension	18	1.016 (0.986 to 1.046)	0.311	1.018 (0.998 to 1.039)	0.079	1.009 (0.988 to 1.031)	0.391		
Octanoylcarnitine (c8)	Hypertension	11	1.042 (0.972 to 1.118)	0.275	1.023 (0.992 to 1.055)	0.148	0.994 (0.955 to 1.034)	0.753		

Exposure	Outcome	nSNP	MR Egger		Weighted median		Inverse variance weighted		MR_PRESSO	
			OR (95% CI)	pval	OR (95% CI)	pval	OR (95% CI)	pval	OR (95% CI)	pval
Stearidonate (18:4n3)	Hypertension	15	1.013 (0.928 to 1.105)	0.780	1.016 (0.963 to 1.072)	0.561	1.022 (0.978 to 1.068)	0.329		
Decanoylcarnitine (C10)	Hypertension	11	1.043 (0.973 to 1.117)	0.264	1.025 (0.984 to 1.067)	0.232	0.996 (0.957 to 1.037)	0.846		
5-dodecenoate (12:1n7)	Hypertension	8	0.986 (0.889 to 1.093)	0.800	0.975 (0.911 to 1.044)	0.465	0.976 (0.929 to 1.025)	0.334		
N-acetylisoleucine	Hypertension	9	1.098 (0.920 to 1.310)	0.336	1.032 (0.962 to 1.107)	0.377	1.029 (0.970 to 1.092)	0.347		
Campesterol	Hypertension	12	1.019 (0.955 to 1.087)	0.589	1.035 (1.002 to 1.068)	0.036	1.027 (0.995 to 1.059)	0.096		

Exposure	Outcome	nSNP	MR Egger		Weighted median		Inverse variance weighted		MR_PRESSO	
			OR (95% CI)	pval	OR (95% CI)	pval	OR (95% CI)	pval	OR (95% CI)	pval
N-acetylthreonine	Hypertension	15	0.978 (0.786 to 1.218)	0.849	1.003 (0.945 to 1.065)	0.917	1.025 (0.939 to 1.120)	0.576	0.999 (0.944 to 1.058)	0.986
1-oleoyl-GPC (18:1)	Hypertension	8	0.978 (0.834 to 1.148)	0.796	1.042 (0.974 to 1.116)	0.230	1.042 (0.987 to 1.099)	0.137		
10-nonadecenoate (19:1n9)	Hypertension	8	1.171 (0.924 to 1.484)	0.241	1.008 (0.930 to 1.092)	0.851	1.033 (0.937 to 1.138)	0.516	1.014 (0.965 to 1.066)	0.610
Piperine	Hypertension	15	0.982 (0.859 to 1.122)	0.793	1.036 (0.974 to 1.102)	0.261	1.003 (0.945 to 1.065)	0.917	0.999 (0.952 to 1.049)	0.976
Carnitine C14	Hypertension	12	0.972 (0.883 to 1.070)	0.577	1.026 (0.971 to 1.084)	0.356	1.037 (0.998 to 1.078)	0.060		

Exposure	Outcome	nSNP	MR Egger		Weighted median		Inverse variance weighted		MR_PRESSO	
			OR (95% CI)	pval	OR (95% CI)	pval	OR (95% CI)	pval	OR (95% CI)	pval
10-heptadecenoate (17:1n7)	Hypertension	10	0.979 (0.849 to 1.129)	0.778	1.000 (0.938 to 1.066)	0.999	0.997 (0.950 to 1.047)	0.912		
1-stearoyl-gpc (18:0)	Hypertension	14	0.967 (0.865 to 1.081)	0.565	1.022 (0.972 to 1.074)	0.396	0.998 (0.948 to 1.050)	0.929	1.007 (0.966 to 1.050)	0.747
1-palmitoyl-GPC (16:0)	Hypertension	15	1.008 (0.907 to 1.119)	0.891	0.980 (0.933 to 1.030)	0.435	0.989 (0.948 to 1.032)	0.624		
Hyocholate	Hypertension	10	1.001 (0.914 to 1.097)	0.984	0.952 (0.906 to 1.001)	0.054	0.973 (0.922 to 1.026)	0.308	0.962 (0.918 to 1.009)	0.151
N-acetylhistidine	Hypertension	12	1.010 (0.970 to 1.053)	0.639	0.988 (0.963 to 1.013)	0.352	1.000 (0.974 to 1.027)	0.991		

Exposure	Outcome	nSNP	MR Egger		Weighted median		Inverse variance weighted		MR_PRESSO	
			OR (95% CI)	pval	OR (95% CI)	pval	OR (95% CI)	pval	OR (95% CI)	pval
Alpha-hydroxyisovalerate	Hypertension	14	0.969 (0.876 to 1.072)	0.553	0.996 (0.960 to 1.033)	0.825	1.004 (0.960 to 1.050)	0.869	0.993 (0.951 to 1.037)	0.748
N-acetylproline	Hypertension	11	0.969 (0.905 to 1.038)	0.395	1.018 (0.965 to 1.075)	0.513	1.028 (0.985 to 1.072)	0.203		
Stachydrine	Hypertension	10	1.109 (0.996 to 1.234)	0.095	1.032 (0.956 to 1.113)	0.421	0.990 (0.926 to 1.058)	0.761		
Salicyluric glucuronide	Hypertension	5	1.044 (0.891 to 1.222)	0.634	1.031 (0.952 to 1.116)	0.457	1.027 (0.966 to 1.091)	0.392		
P-cresol sulfate	Hypertension	14	1.072 (0.947 to 1.214)	0.294	0.974 (0.913 to 1.040)	0.434	0.973 (0.921 to 1.028)	0.327		

Exposure	Outcome	nSNP	MR Egger		Weighted median		Inverse variance weighted		MR_PRESSO	
			OR (95% CI)	pval	OR (95% CI)	pval	OR (95% CI)	pval	OR (95% CI)	pval
Gamma-glutamylglycine	Hypertension	18	0.960 (0.920 to 1.002)	0.079	0.957 (0.935 to 0.980)	2.52e-04	0.950 (0.924 to 0.977)	2.58e-04		
1-linoleoyl-gpc (18:2)	Hypertension	11	0.958 (0.877 to 1.046)	0.362	1.023 (0.971 to 1.078)	0.393	0.977 (0.933 to 1.024)	0.337		
Stearoylcarnitine	Hypertension	15	1.116 (0.981 to 1.270)	0.119	1.070 (1.020 to 1.122)	0.005	1.054 (1.013 to 1.097)	0.009		
Eicosenoate (20:1)	Hypertension	13	1.159 (0.935 to 1.438)	0.205	0.971 (0.907 to 1.040)	0.400	0.968 (0.892 to 1.050)	0.437	0.961 (0.908 to 1.016)	0.193
Aconitate [cis or trans]	Hypertension	14	1.107 (0.991 to 1.236)	0.096	1.023 (0.964 to 1.086)	0.450	1.012 (0.961 to 1.065)	0.657		

Exposure	Outcome	nSNP	MR Egger		Weighted median		Inverse variance weighted		MR_PRESSO	
			OR (95% CI)	pval	OR (95% CI)	pval	OR (95% CI)	pval	OR (95% CI)	pval
Erythronate	Hypertension	11	0.971 (0.771 to 1.222)	0.806	0.994 (0.940 to 1.052)	0.845	1.006 (0.917 to 1.102)	0.905	0.994 (0.948 to 1.041)	0.798
Isovalerylcarnitine (C5)	Hypertension	10	1.054 (0.886 to 1.253)	0.568	1.059 (1.008 to 1.112)	0.022	1.055 (1.006 to 1.107)	0.027		
Linolenate [alpha or gamma; (18:3n3 or 6)]	Hypertension	11	1.065 (0.881 to 1.287)	0.530	1.029 (0.952 to 1.111)	0.474	0.977 (0.901 to 1.060)	0.579	1.023 (0.965 to 1.084)	0.466
Gamma-glutamylthreonine	Hypertension	18	1.022 (0.938 to 1.113)	0.629	0.997 (0.956 to 1.040)	0.902	0.994 (0.947 to 1.044)	0.810	0.999 (0.962 to 1.037)	0.954
Laurylcarnitine	Hypertension	14	0.930 (0.869 to 0.995)	0.057	0.967 (0.919 to 1.018)	0.201	0.977 (0.941 to 1.015)	0.234		

Exposure	Outcome	nSNP	MR Egger		Weighted median		Inverse variance weighted		MR_PRESSO	
			OR (95% CI)	pval	OR (95% CI)	pval	OR (95% CI)	pval	OR (95% CI)	pval
Gamma-glutamylmethionine	Hypertension	13	1.037 (0.958 to 1.124)	0.389	0.979 (0.925 to 1.036)	0.464	0.956 (0.913 to 1.001)	0.057		
Epiandrosterone sulfate	Hypertension	17	0.984 (0.952 to 1.016)	0.344	0.996 (0.979 to 1.013)	0.660	1.005 (0.974 to 1.036)	0.764	0.997 (0.981 to 1.014)	0.761
Gamma-glutamyltryptophan	Hypertension	15	1.139 (0.976 to 1.329)	0.122	0.997 (0.940 to 1.058)	0.916	1.019 (0.962 to 1.080)	0.520	1.006 (0.952 to 1.063)	0.843
7-methylxanthine	Hypertension	12	1.059 (0.899 to 1.248)	0.510	0.920 (0.856 to 0.989)	0.024	0.950 (0.889 to 1.014)	0.122		
3,7-dimethylurate	Hypertension	14	1.069 (0.998 to 1.145)	0.082	1.010 (0.965 to 1.057)	0.679	1.016 (0.977 to 1.057)	0.426		

Exposure	Outcome	nSNP	MR Egger		Weighted median		Inverse variance weighted		MR_PRESSO	
			OR (95% CI)	pval	OR (95% CI)	pval	OR (95% CI)	pval	OR (95% CI)	pval
1,7-dimethyluric acid	Hypertension	19	1.051 (0.968 to 1.141)	0.254	1.009 (0.962 to 1.059)	0.705	1.015 (0.972 to 1.060)	0.495		
1,3,7-trimethylurate	Hypertension	17	1.014 (0.901 to 1.141)	0.824	1.072 (1.022 to 1.123)	0.004	1.013 (0.959 to 1.070)	0.643	1.045 (1.011 to 1.079)	0.020
N4-acetylcytidine	Hypertension	26	0.997 (0.952 to 1.045)	0.914	1.007 (0.977 to 1.038)	0.646	1.027 (0.997 to 1.057)	0.080	1.019 (0.995 to 1.043)	0.130
Indolebutyrate	Hypertension	14	1.003 (0.893 to 1.126)	0.963	1.017 (0.977 to 1.058)	0.413	1.014 (0.978 to 1.052)	0.444		
5-acetylamino-6-amino-3-methyluracil	Hypertension	19	1.012 (0.958 to 1.069)	0.679	0.997 (0.967 to 1.028)	0.845	1.013 (0.982 to 1.044)	0.418		

Exposure	Outcome	nSNP	MR Egger		Weighted median		Inverse variance weighted		MR_PRESSO	
			OR (95% CI)	pval	OR (95% CI)	pval	OR (95% CI)	pval	OR (95% CI)	pval
N1-methylinosine	Hypertension	17	0.974 (0.865 to 1.097)	0.670	0.980 (0.932 to 1.031)	0.436	0.974 (0.937 to 1.012)	0.178		
N6-carbamoylthreonyladenosine	Hypertension	17	1.005 (0.910 to 1.110)	0.920	1.008 (0.970 to 1.047)	0.683	1.027 (0.968 to 1.089)	0.375	1.009 (0.977 to 1.042)	0.596
1-methylxanthine	Hypertension	19	1.016 (0.962 to 1.072)	0.583	1.007 (0.979 to 1.036)	0.630	1.007 (0.977 to 1.037)	0.646	1.003 (0.977 to 1.030)	0.820
N2,n2-dimethylguanosine	Hypertension	9	1.016 (0.836 to 1.235)	0.877	1.011 (0.930 to 1.099)	0.794	0.990 (0.918 to 1.067)	0.789	1.022 (0.963 to 1.085)	0.497
4-hydroxyhippurate	Hypertension	12	0.975 (0.860 to 1.107)	0.707	0.990 (0.928 to 1.056)	0.762	0.975 (0.930 to 1.022)	0.295		

Exposure	Outcome	nSNP	MR Egger		Weighted median		Inverse variance weighted		MR_PRESSO	
			OR (95% CI)	pval	OR (95% CI)	pval	OR (95% CI)	pval	OR (95% CI)	pval
3-(3-amino-3-carboxypropyl)uridine	Hypertension	9	1.082 (0.818 to 1.430)	0.599	1.016 (0.936 to 1.102)	0.711	1.049 (0.938 to 1.173)	0.402	0.977 (0.913 to 1.046)	0.524
5,6-dihydrouridine	Hypertension	17	1.028 (0.903 to 1.170)	0.683	1.033 (0.984 to 1.084)	0.197	0.999 (0.938 to 1.064)	0.966	0.996 (0.956 to 1.037)	0.846
Phenylacetylglutamine	Hypertension	12	1.123 (0.955 to 1.321)	0.191	1.037 (0.956 to 1.124)	0.387	0.995 (0.917 to 1.080)	0.906	1.004 (0.941 to 1.071)	0.904
3-hydroxydodecanedioate	Hypertension	11	0.939 (0.841 to 1.049)	0.298	1.012 (0.966 to 1.061)	0.611	0.986 (0.936 to 1.038)	0.588	1.006 (0.977 to 1.035)	0.713
1-arachidonylglycerol (20:4)	Hypertension	14	1.048 (0.991 to 1.109)	0.128	0.998 (0.961 to 1.035)	0.897	1.002 (0.970 to 1.035)	0.898		

Exposure	Outcome	nSNP	MR Egger		Weighted median		Inverse variance weighted		MR_PRESSO	
			OR (95% CI)	pval	OR (95% CI)	pval	OR (95% CI)	pval	OR (95% CI)	pval
7-methylguanine	Hypertension	12	0.997 (0.915 to 1.088)	0.954	1.004 (0.953 to 1.058)	0.875	1.014 (0.971 to 1.058)	0.532		
1-stearoyl-GPE (18:0)	Hypertension	26	1.017 (0.905 to 1.143)	0.780	1.021 (0.986 to 1.056)	0.246	1.008 (0.963 to 1.056)	0.734	1.001 (0.969 to 1.035)	0.937
Phenol sulfate	Hypertension	13	0.896 (0.707 to 1.137)	0.387	0.983 (0.913 to 1.059)	0.652	1.034 (0.963 to 1.111)	0.351	1.012 (0.950 to 1.078)	0.719
Oleoylcarnitine	Hypertension	10	0.962 (0.868 to 1.066)	0.480	0.968 (0.911 to 1.028)	0.291	0.981 (0.931 to 1.034)	0.481	0.950 (0.906 to 0.997)	0.073
Gamma-glutamylisoleucine	Hypertension	11	1.119 (0.987 to 1.269)	0.112	0.961 (0.894 to 1.033)	0.279	0.977 (0.928 to 1.028)	0.364		

Exposure	Outcome	nSNP	MR Egger		Weighted median		Inverse variance weighted		MR_PRESSO	
			OR (95% CI)	pval	OR (95% CI)	pval	OR (95% CI)	pval	OR (95% CI)	pval
Malonylcarnitine	Hypertension	11	1.041 (0.936 to 1.158)	0.476	1.057 (1.006 to 1.112)	0.028	1.055 (1.011 to 1.102)	0.014		
1-palmitoleoyl-GPC (16:1)	Hypertension	20	0.940 (0.753 to 1.172)	0.589	0.972 (0.919 to 1.028)	0.327	0.995 (0.932 to 1.062)	0.874	0.974 (0.925 to 1.027)	0.348
N-methyl-2-pyridone-5-carboxamide	Hypertension	10	0.999 (0.944 to 1.056)	0.963	1.008 (0.983 to 1.034)	0.546	1.014 (0.972 to 1.058)	0.523		
Gamma-glutamyl-2-aminobutyrate	Hypertension	18	1.066 (0.963 to 1.179)	0.236	1.012 (0.962 to 1.064)	0.653	1.021 (0.979 to 1.064)	0.328		
1-stearoyl-GPG (18:0)	Hypertension	14	0.983 (0.848 to 1.140)	0.826	0.951 (0.909 to 0.994)	0.026	0.986 (0.930 to 1.047)	0.653	0.959 (0.933 to 0.986)	0.013

Exposure	Outcome	nSNP	MR Egger		Weighted median		Inverse variance weighted		MR_PRESSO	
			OR (95% CI)	pval	OR (95% CI)	pval	OR (95% CI)	pval	OR (95% CI)	pval
Hexanoylglycine	Hypertension	17	1.015 (0.950 to 1.084)	0.672	1.022 (0.990 to 1.054)	0.178	1.005 (0.973 to 1.038)	0.772	1.036 (1.012 to 1.061)	0.010
Glutamine degradant	Hypertension	18	1.000 (0.912 to 1.097)	0.999	1.076 (1.026 to 1.129)	0.003	1.063 (1.011 to 1.117)	0.016	1.052 (1.008 to 1.099)	0.034
5-methyluridine (ribothymidine)	Hypertension	21	0.975 (0.942 to 1.008)	0.151	0.992 (0.969 to 1.016)	0.511	0.992 (0.970 to 1.014)	0.476		
Cysteine-glutathione disulfide	Hypertension	14	0.999 (0.932 to 1.071)	0.978	0.965 (0.930 to 1.002)	0.063	0.956 (0.921 to 0.991)	0.015		
Isovalerylglycine	Hypertension	14	0.921 (0.807 to 1.051)	0.247	0.951 (0.905 to 0.999)	0.046	0.957 (0.912 to 1.006)	0.082		

Exposure	Outcome	nSNP	MR Egger		Weighted median		Inverse variance weighted		MR_PRESSO	
			OR (95% CI)	pval	OR (95% CI)	pval	OR (95% CI)	pval	OR (95% CI)	pval
2-hydroxy-3-methylvalerate	Hypertension	14	1.034 (0.938 to 1.139)	0.518	0.992 (0.942 to 1.046)	0.773	0.999 (0.954 to 1.046)	0.956	0.985 (0.948 to 1.023)	0.451
Homostachydrine	Hypertension	11	1.010 (0.926 to 1.100)	0.832	1.028 (0.968 to 1.092)	0.372	1.023 (0.978 to 1.070)	0.321		
1-arachidonoyl-gpc (20:4n6)	Hypertension	16	0.997 (0.955 to 1.042)	0.907	0.994 (0.974 to 1.015)	0.584	0.994 (0.967 to 1.022)	0.688	0.998 (0.974 to 1.023)	0.888
2-palmitoyl-GPC (16:0)	Hypertension	10	0.999 (0.716 to 1.394)	0.998	1.047 (0.972 to 1.127)	0.227	1.042 (0.932 to 1.163)	0.472	1.022 (0.956 to 1.093)	0.545
2-palmitoleoyl-GPC (16:1)	Hypertension	19	0.978 (0.862 to 1.109)	0.735	1.002 (0.951 to 1.056)	0.938	0.987 (0.945 to 1.031)	0.551		

Exposure	Outcome	nSNP	MR Egger		Weighted median		Inverse variance weighted		MR_PRESSO	
			OR (95% CI)	pval	OR (95% CI)	pval	OR (95% CI)	pval	OR (95% CI)	pval
1-arachidonoyl-GPE (20:4n6)	Hypertension	21	0.973 (0.941 to 1.007)	0.132	0.979 (0.954 to 1.005)	0.111	0.989 (0.971 to 1.008)	0.253		
1-palmitoyl-GPE (16:0)	Hypertension	12	1.053 (0.825 to 1.344)	0.687	1.017 (0.971 to 1.065)	0.475	1.007 (0.941 to 1.078)	0.837	1.007 (0.964 to 1.052)	0.756
1-linoleoyl-GPE (18:2)	Hypertension	19	0.989 (0.944 to 1.037)	0.666	0.997 (0.966 to 1.029)	0.843	0.984 (0.960 to 1.010)	0.222		
1-oleoyl-GPE (18:1)	Hypertension	21	0.983 (0.874 to 1.106)	0.781	0.997 (0.952 to 1.044)	0.895	0.986 (0.943 to 1.030)	0.517	0.976 (0.937 to 1.017)	0.264
2-hydroxypalmitate	Hypertension	11	1.025 (0.942 to 1.115)	0.583	0.990 (0.932 to 1.051)	0.736	0.967 (0.920 to 1.016)	0.188		

Exposure	Outcome	nSNP	MR Egger		Weighted median		Inverse variance weighted		MR_PRESSO	
			OR (95% CI)	pval	OR (95% CI)	pval	OR (95% CI)	pval	OR (95% CI)	pval
Docosapentaenoate (n6 DPA; 22:5n6)	Hypertension	8	1.048 (0.906 to 1.213)	0.551	1.017 (0.955 to 1.084)	0.595	1.018 (0.970 to 1.068)	0.468		
Gulonate	Hypertension	13	1.014 (0.904 to 1.137)	0.815	1.038 (0.978 to 1.103)	0.220	1.025 (0.979 to 1.073)	0.288		
Isobutyrylglycine	Hypertension	19	1.017 (0.911 to 1.136)	0.763	1.019 (0.972 to 1.067)	0.435	0.984 (0.934 to 1.036)	0.533	1.015 (0.978 to 1.054)	0.437
Glutarylcarnitine (c5-dc)	Hypertension	25	1.033 (0.959 to 1.113)	0.398	1.023 (0.992 to 1.055)	0.154	1.017 (0.985 to 1.051)	0.301	1.023 (0.995 to 1.051)	0.128
Carnitine C5:1	Hypertension	15	1.080 (0.906 to 1.286)	0.408	1.039 (0.977 to 1.104)	0.220	1.000 (0.930 to 1.076)	0.992	1.025 (0.975 to 1.077)	0.349

Exposure	Outcome	nSNP	MR Egger		Weighted median		Inverse variance weighted		MR_PRESSO	
			OR (95% CI)	pval	OR (95% CI)	pval	OR (95% CI)	pval	OR (95% CI)	pval
Hydroquinone sulfate	Hypertension	10	1.000 (0.852 to 1.175)	0.996	1.003 (0.939 to 1.071)	0.936	0.991 (0.942 to 1.043)	0.731		
Beta-hydroxyisovaleroylcarnitine	Hypertension	16	1.019 (0.931 to 1.116)	0.693	1.031 (0.992 to 1.072)	0.124	1.033 (0.988 to 1.079)	0.150	1.034 (1.002 to 1.068)	0.060
Catechol sulfate	Hypertension	19	1.042 (0.932 to 1.167)	0.479	1.004 (0.954 to 1.056)	0.884	0.997 (0.950 to 1.047)	0.919	1.012 (0.969 to 1.057)	0.590
7-alpha-hydroxy-3-oxo-4-cholestenoate (7-hoca)	Hypertension	14	1.009 (0.906 to 1.124)	0.876	1.026 (0.970 to 1.085)	0.365	1.014 (0.967 to 1.063)	0.566		
Octadecanedioate	Hypertension	19	0.945 (0.857 to 1.041)	0.268	0.954 (0.913 to 0.996)	0.033	0.977 (0.939 to 1.017)	0.252		

Exposure	Outcome	nSNP	MR Egger		Weighted median		Inverse variance weighted		MR_PRESSO	
			OR (95% CI)	pval	OR (95% CI)	pval	OR (95% CI)	pval	OR (95% CI)	pval
Tetradecanedioate (C14-DC)	Hypertension	9	0.953 (0.919 to 0.988)	0.036	0.980 (0.955 to 1.005)	0.122	0.979 (0.958 to 1.001)	0.057		
Hexadecanedioate (C16-DC)	Hypertension	12	0.957 (0.920 to 0.995)	0.052	0.971 (0.946 to 0.996)	0.022	0.979 (0.951 to 1.008)	0.150		
Glycerophosphoethanolamine	Hypertension	15	0.957 (0.896 to 1.022)	0.217	1.007 (0.968 to 1.048)	0.711	1.035 (0.995 to 1.077)	0.085	1.023 (0.991 to 1.056)	0.186
Taurolithocholate 3-sulfate	Hypertension	14	0.979 (0.909 to 1.054)	0.576	1.008 (0.957 to 1.061)	0.769	0.997 (0.960 to 1.035)	0.870		
1-palmitoyl-GPI (16:0)	Hypertension	15	0.925 (0.835 to 1.025)	0.161	1.009 (0.950 to 1.071)	0.778	1.010 (0.955 to 1.068)	0.729	0.999 (0.950 to 1.051)	0.970

Exposure	Outcome	nSNP	MR Egger		Weighted median		Inverse variance weighted		MR_PRESSO	
			OR (95% CI)	pval	OR (95% CI)	pval	OR (95% CI)	pval	OR (95% CI)	pval
Glycolithocholate sulfate	Hypertension	8	0.952 (0.831 to 1.090)	0.500	0.998 (0.946 to 1.053)	0.932	1.003 (0.945 to 1.064)	0.928	1.029 (0.981 to 1.078)	0.293
3-(3-hydroxyphenyl)propionate	Hypertension	13	0.895 (0.759 to 1.056)	0.216	1.038 (0.965 to 1.116)	0.318	0.968 (0.898 to 1.043)	0.393	0.966 (0.905 to 1.030)	0.313
Deoxycarnitine	Hypertension	20	0.977 (0.936 to 1.019)	0.286	1.002 (0.970 to 1.035)	0.905	1.025 (0.996 to 1.054)	0.091		
Alpha-hydroxycaproate	Hypertension	7	1.018 (0.893 to 1.161)	0.795	0.989 (0.950 to 1.030)	0.601	0.988 (0.947 to 1.030)	0.567		
1-ribosyl-imidazoleacetate	Hypertension	21	0.886 (0.771 to 1.019)	0.107	0.957 (0.912 to 1.005)	0.078	1.025 (0.974 to 1.080)	0.345	0.980 (0.940 to 1.021)	0.340

Exposure	Outcome	nSNP	MR Egger		Weighted median		Inverse variance weighted		MR_PRESSO	
			OR (95% CI)	pval	OR (95% CI)	pval	OR (95% CI)	pval	OR (95% CI)	pval
3,4-dihydroxybutyrate	Hypertension	12	0.951 (0.804 to 1.125)	0.571	1.060 (1.004 to 1.118)	0.034	1.033 (0.974 to 1.095)	0.283	1.047 (0.995 to 1.101)	0.108
Indoleacetylglutamine	Hypertension	16	1.055 (0.971 to 1.147)	0.228	1.002 (0.964 to 1.041)	0.918	1.010 (0.983 to 1.039)	0.457		
Mannitol/sorbitol	Hypertension	15	1.009 (0.950 to 1.071)	0.781	1.021 (0.974 to 1.070)	0.384	1.001 (0.965 to 1.039)	0.953		
Tryptophan betaine	Hypertension	20	0.954 (0.844 to 1.079)	0.464	1.009 (0.963 to 1.058)	0.703	1.071 (1.001 to 1.147)	0.047	1.052 (1.005 to 1.101)	0.047
Dihomo-linolenate (20:3n3 or n6)	Hypertension	13	0.991 (0.895 to 1.097)	0.861	0.989 (0.933 to 1.048)	0.709	0.951 (0.909 to 0.996)	0.034		

Exposure	Outcome	nSNP	MR Egger		Weighted median		Inverse variance weighted		MR_PRESSO	
			OR (95% CI)	pval	OR (95% CI)	pval	OR (95% CI)	pval	OR (95% CI)	pval
1-linoleoyl-GPI (18:2)	Hypertension	17	0.996 (0.889 to 1.115)	0.941	0.975 (0.929 to 1.023)	0.294	0.965 (0.918 to 1.014)	0.160	0.963 (0.924 to 1.005)	0.104
1-oleoyl-GPI (18:1)	Hypertension	18	0.922 (0.806 to 1.055)	0.257	0.987 (0.934 to 1.042)	0.632	0.980 (0.939 to 1.023)	0.362		
O-cresol sulfate	Hypertension	7	1.025 (0.962 to 1.093)	0.475	1.017 (0.976 to 1.060)	0.430	1.014 (0.975 to 1.055)	0.482		
4-vinylphenol sulfate	Hypertension	12	0.988 (0.858 to 1.138)	0.875	1.001 (0.943 to 1.063)	0.967	0.996 (0.951 to 1.043)	0.861		
Pyrraline	Hypertension	8	0.951 (0.835 to 1.084)	0.481	0.894 (0.831 to 0.961)	0.002	0.924 (0.867 to 0.984)	0.014		

Exposure	Outcome	nSNP	MR Egger		Weighted median		Inverse variance weighted		MR_PRESSO	
			OR (95% CI)	pval	OR (95% CI)	pval	OR (95% CI)	pval	OR (95% CI)	pval
N6-acetyllysine	Hypertension	12	1.042 (0.968 to 1.121)	0.297	1.011 (0.975 to 1.048)	0.545	1.008 (0.960 to 1.059)	0.750	1.020 (0.983 to 1.060)	0.320
Thymol sulfate	Hypertension	9	0.960 (0.838 to 1.101)	0.580	0.967 (0.910 to 1.027)	0.269	0.986 (0.939 to 1.036)	0.579		
N-acetylserine	Hypertension	11	0.972 (0.737 to 1.282)	0.845	0.975 (0.905 to 1.050)	0.498	1.025 (0.916 to 1.146)	0.670	0.982 (0.965 to 0.999)	0.076
3-methyladipate	Hypertension	9	0.985 (0.835 to 1.161)	0.861	0.959 (0.903 to 1.018)	0.171	0.988 (0.922 to 1.058)	0.722	0.947 (0.921 to 0.975)	0.008
4-ethylphenylsulfate	Hypertension	9	0.998 (0.844 to 1.180)	0.980	1.022 (0.956 to 1.092)	0.521	1.012 (0.936 to 1.094)	0.770	1.005 (0.960 to 1.053)	0.833

Exposure	Outcome	nSNP	MR Egger		Weighted median		Inverse variance weighted		MR_PRESSO	
			OR (95% CI)	pval	OR (95% CI)	pval	OR (95% CI)	pval	OR (95% CI)	pval
1-stearoyl-2-oleoyl-GPE (18:0/18:1)	Hypertension	18	1.050 (0.974 to 1.132)	0.219	1.015 (0.979 to 1.051)	0.417	1.046 (1.013 to 1.081)	0.007	1.027 (1.008 to 1.046)	0.014
Dimethylarginine (sdma + adma)	Hypertension	10	1.068 (0.933 to 1.222)	0.367	0.970 (0.914 to 1.029)	0.310	0.978 (0.916 to 1.044)	0.501		
Hexanoylglutamine	Hypertension	13	1.026 (0.941 to 1.118)	0.573	1.027 (0.997 to 1.058)	0.075	1.019 (0.977 to 1.063)	0.377	1.008 (0.979 to 1.037)	0.619
Gamma-glutamylalanine	Hypertension	11	0.979 (0.818 to 1.170)	0.818	0.992 (0.936 to 1.051)	0.780	0.974 (0.918 to 1.034)	0.388	0.990 (0.940 to 1.044)	0.731
5alpha-androstan-3beta,17beta-diol disulfate	Hypertension	16	0.965 (0.923 to 1.008)	0.135	0.974 (0.947 to 1.002)	0.067	0.970 (0.942 to 1.000)	0.048		

Exposure	Outcome	nSNP	MR Egger		Weighted median		Inverse variance weighted		MR_PRESSO	
			OR (95% CI)	pval	OR (95% CI)	pval	OR (95% CI)	pval	OR (95% CI)	pval
N-methylproline	Hypertension	10	1.078 (0.973 to 1.195)	0.190	1.068 (1.001 to 1.138)	0.046	1.067 (1.016 to 1.121)	0.010		
Carotenoid	Hypertension	15	0.963 (0.837 to 1.107)	0.602	0.996 (0.937 to 1.060)	0.912	0.989 (0.934 to 1.047)	0.701		
5alpha-pregnan-3beta,20alpha-diol disulfate	Hypertension	22	0.980 (0.912 to 1.053)	0.586	1.003 (0.967 to 1.040)	0.885	0.986 (0.953 to 1.020)	0.411	0.993 (0.963 to 1.024)	0.670
Taurocholenate sulfate	Hypertension	28	0.968 (0.926 to 1.012)	0.166	0.974 (0.949 to 0.999)	0.044	0.993 (0.967 to 1.020)	0.615	0.980 (0.959 to 1.002)	0.081
Pregnenediol disulfate ($C_{21}H_{34}O_8S_2$)	Hypertension	23	0.973 (0.921 to 1.027)	0.334	0.972 (0.946 to 1.000)	0.047	0.980 (0.949 to 1.013)	0.239	0.987 (0.961 to 1.014)	0.342

Exposure	Outcome	nSNP	MR Egger		Weighted median		Inverse variance weighted		MR_PRESSO	
			OR (95% CI)	pval	OR (95% CI)	pval	OR (95% CI)	pval	OR (95% CI)	pval
Glycocholenate sulfate	Hypertension	24	1.013 (0.991 to 1.035)	0.276	0.990 (0.968 to 1.013)	0.397	0.994 (0.977 to 1.011)	0.474	0.985 (0.970 to 1.000)	0.067
Androstenediol (3beta,17beta) disulfate (1)	Hypertension	17	0.950 (0.902 to 1.002)	0.077	0.964 (0.933 to 0.997)	0.033	0.975 (0.944 to 1.007)	0.131	0.968 (0.947 to 0.989)	0.011
Chiro-inositol	Hypertension	7	0.878 (0.674 to 1.143)	0.377	1.021 (0.956 to 1.090)	0.542	1.003 (0.949 to 1.061)	0.907		
1-palmitoyl-2-linoleoyl-GPE (16:0/18:2)	Hypertension	20	1.028 (0.972 to 1.086)	0.344	1.010 (0.989 to 1.032)	0.338	1.013 (0.983 to 1.044)	0.409	1.012 (0.995 to 1.029)	0.195
Sphinganine-1-phosphate	Hypertension	7	1.028 (0.797 to 1.325)	0.842	1.028 (0.957 to 1.105)	0.442	1.007 (0.935 to 1.085)	0.852		

Exposure	Outcome	nSNP	MR Egger		Weighted median		Inverse variance weighted		MR_PRESSO	
			OR (95% CI)	pval	OR (95% CI)	pval	OR (95% CI)	pval	OR (95% CI)	pval
Glycosyl-N-stearoyl-sphingosine (d18:1/18:0)	Hypertension	20	0.975 (0.904 to 1.051)	0.513	0.985 (0.945 to 1.027)	0.483	0.978 (0.950 to 1.007)	0.130		
4-allylphenol sulfate	Hypertension	12	1.060 (0.877 to 1.280)	0.563	0.970 (0.911 to 1.032)	0.335	1.013 (0.934 to 1.099)	0.757	0.991 (0.929 to 1.058)	0.797
Succinylcarnitine	Hypertension	21	1.002 (0.938 to 1.071)	0.943	1.025 (0.995 to 1.057)	0.108	1.010 (0.977 to 1.044)	0.549		
21-hydroxypregnenolone disulfate	Hypertension	25	0.866 (0.762 to 0.985)	0.038	0.950 (0.917 to 0.984)	0.005	0.972 (0.922 to 1.025)	0.297	0.951 (0.923 to 0.981)	0.004
5alpha-androstan-3alpha,17alpha-diol monosulfate	Hypertension	17	0.982 (0.937 to 1.029)	0.464	0.987 (0.964 to 1.011)	0.300	1.001 (0.971 to 1.031)	0.972	0.991 (0.968 to 1.015)	0.472

Exposure	Outcome	nSNP	MR Egger		Weighted median		Inverse variance weighted		MR_PRESSO	
			OR (95% CI)	pval	OR (95% CI)	pval	OR (95% CI)	pval	OR (95% CI)	pval
Androstenediol (3beta,17beta) disulfate (2)	Hypertension	29	0.895 (0.806 to 0.994)	0.049	0.950 (0.915 to 0.988)	0.009	0.949 (0.907 to 0.994)	0.027	0.967 (0.936 to 0.998)	0.046
5alpha-androstan-3beta,17alpha-diol disulfate	Hypertension	13	0.920 (0.850 to 0.996)	0.065	0.961 (0.922 to 1.001)	0.059	0.992 (0.954 to 1.032)	0.707		
5alpha-pregnan-3beta,20beta-diol monosulfate (1)	Hypertension	16	1.008 (0.970 to 1.048)	0.683	0.990 (0.961 to 1.019)	0.489	0.981 (0.957 to 1.006)	0.142		
5alpha-pregnan-3beta,20alpha-diol monosulfate (2)	Hypertension	21	1.022 (0.963 to 1.084)	0.485	1.009 (0.970 to 1.049)	0.655	0.982 (0.953 to 1.011)	0.215		
5alpha-androstan-3beta,17beta-diol monosulfate (2)	Hypertension	21	0.982 (0.960 to 1.005)	0.145	0.985 (0.968 to 1.002)	0.080	0.992 (0.973 to 1.012)	0.438		

Exposure	Outcome	nSNP	MR Egger		Weighted median		Inverse variance weighted		MR_PRESSO	
			OR (95% CI)	pval	OR (95% CI)	pval	OR (95% CI)	pval	OR (95% CI)	pval
Androstenediol (3alpha, 17alpha) monosulfate (3)	Hypertension	22	0.994 (0.974 to 1.015)	0.588	0.991 (0.973 to 1.009)	0.307	0.991 (0.975 to 1.006)	0.241		
5alpha-pregnan-diol disulfate	Hypertension	13	0.994 (0.930 to 1.062)	0.857	1.011 (0.978 to 1.045)	0.532	1.001 (0.977 to 1.026)	0.926		
5alpha-androstan-3alpha,17beta-diol monosulfate (1)	Hypertension	18	0.978 (0.956 to 0.999)	0.058	0.988 (0.973 to 1.002)	0.090	0.990 (0.972 to 1.008)	0.267		
5alpha-androstan-3alpha,17beta-diol disulfate	Hypertension	20	1.019 (0.956 to 1.086)	0.577	0.963 (0.924 to 1.004)	0.079	0.966 (0.937 to 0.997)	0.030		
Andro steroid monosulfate C19H28O6S (1)	Hypertension	23	1.022 (0.987 to 1.058)	0.232	1.023 (1.001 to 1.044)	0.040	1.029 (1.004 to 1.054)	0.022		

Exposure	Outcome	nSNP	MR Egger		Weighted median		Inverse variance weighted		MR_PRESSO	
			OR (95% CI)	pval	OR (95% CI)	pval	OR (95% CI)	pval	OR (95% CI)	pval
Pregnenediol sulfate (C21H34O5S)	Hypertension	26	0.983 (0.849 to 1.138)	0.822	1.010 (0.962 to 1.059)	0.697	1.013 (0.951 to 1.080)	0.682	1.002 (0.969 to 1.036)	0.914
4-hydroxycoumarin	Hypertension	14	1.001 (0.939 to 1.068)	0.965	0.982 (0.939 to 1.027)	0.429	0.979 (0.942 to 1.018)	0.290		
16a-hydroxy DHEA 3-sulfate	Hypertension	19	1.022 (0.990 to 1.054)	0.200	1.019 (1.000 to 1.038)	0.044	1.026 (1.001 to 1.052)	0.044		
Gamma-CEHC	Hypertension	25	0.982 (0.916 to 1.053)	0.609	0.964 (0.929 to 1.001)	0.054	0.982 (0.948 to 1.018)	0.333	0.981 (0.953 to 1.010)	0.219
2-hydroxyglutarate	Hypertension	15	1.035 (0.950 to 1.127)	0.450	0.988 (0.943 to 1.035)	0.605	0.997 (0.963 to 1.032)	0.881		

Exposure	Outcome	nSNP	MR Egger		Weighted median		Inverse variance weighted		MR_PRESSO	
			OR (95% CI)	pval	OR (95% CI)	pval	OR (95% CI)	pval	OR (95% CI)	pval
N-acetyl-beta-alanine	Hypertension	16	0.986 (0.965 to 1.006)	0.196	0.987 (0.968 to 1.006)	0.185	0.991 (0.974 to 1.008)	0.295		
Tridecenedioate (C13:1-DC)	Hypertension	12	1.043 (0.886 to 1.227)	0.627	0.986 (0.928 to 1.048)	0.645	0.996 (0.932 to 1.064)	0.897	0.997 (0.957 to 1.038)	0.872
N-acetyl-3-methylhistidine	Hypertension	18	1.068 (1.020 to 1.117)	0.012	1.053 (1.019 to 1.088)	0.002	1.032 (1.007 to 1.057)	0.011		
2-hydroxyhippurate	Hypertension	11	1.106 (0.994 to 1.230)	0.098	1.017 (0.954 to 1.085)	0.599	1.019 (0.961 to 1.080)	0.539		
4-cholesten-3-one	Hypertension	17	0.949 (0.876 to 1.029)	0.225	1.005 (0.960 to 1.052)	0.828	1.006 (0.972 to 1.041)	0.730		

Exposure	Outcome	nSNP	MR Egger		Weighted median		Inverse variance weighted		MR_PRESSO	
			OR (95% CI)	pval	OR (95% CI)	pval	OR (95% CI)	pval	OR (95% CI)	pval
Androstenediol (3beta,17beta) monosulfate (2)	Hypertension	18	0.975 (0.858 to 1.107)	0.698	0.963 (0.917 to 1.011)	0.126	1.003 (0.947 to 1.062)	0.926	0.998 (0.957 to 1.041)	0.944
Androstenediol (3beta,17beta) monosulfate (1)	Hypertension	18	0.946 (0.839 to 1.067)	0.381	0.967 (0.925 to 1.012)	0.148	0.991 (0.923 to 1.064)	0.796	0.968 (0.930 to 1.008)	0.141
Pregnenolone sulfate	Hypertension	20	0.929 (0.803 to 1.076)	0.339	0.982 (0.936 to 1.031)	0.465	1.016 (0.951 to 1.085)	0.643	0.998 (0.953 to 1.045)	0.929
S-methylmethionine	Hypertension	9	1.066 (0.901 to 1.261)	0.481	1.016 (0.953 to 1.083)	0.628	1.003 (0.953 to 1.054)	0.921		
13-HODE + 9-HODE	Hypertension	7	1.029 (0.914 to 1.159)	0.656	1.032 (0.960 to 1.110)	0.395	1.032 (0.973 to 1.093)	0.295		

Exposure	Outcome	nSNP	MR Egger		Weighted median		Inverse variance weighted		MR_PRESSO	
			OR (95% CI)	pval	OR (95% CI)	pval	OR (95% CI)	pval	OR (95% CI)	pval
Palmitoyl sphingomyelin (d18:1/16:0)	Hypertension	19	1.036 (0.894 to 1.202)	0.642	1.018 (0.964 to 1.076)	0.519	0.992 (0.933 to 1.055)	0.802	0.994 (0.945 to 1.045)	0.812
Indole-3-carboxylate	Hypertension	9	0.983 (0.926 to 1.043)	0.582	0.990 (0.952 to 1.030)	0.618	0.982 (0.947 to 1.018)	0.333		
Sphingomyelin (d18:1/18:1, d18:2/18:0)	Hypertension	21	1.221 (1.037 to 1.439)	0.027	1.005 (0.963 to 1.049)	0.818	0.974 (0.897 to 1.057)	0.526	0.994 (0.960 to 1.029)	0.735
Ergothioneine	Hypertension	11	1.009 (0.996 to 1.023)	0.207	1.007 (0.995 to 1.019)	0.266	1.002 (0.988 to 1.017)	0.749		
Cis-4-decenoylcarnitine (C10:1)	Hypertension	13	1.053 (1.008 to 1.100)	0.041	1.024 (0.993 to 1.055)	0.131	1.003 (0.974 to 1.033)	0.831		

Exposure	Outcome	nSNP	MR Egger		Weighted median		Inverse variance weighted		MR_PRESSO	
			OR (95% CI)	pval	OR (95% CI)	pval	OR (95% CI)	pval	OR (95% CI)	pval
5-(galactosylhydroxy)-L-lysine	Hypertension	9	1.030 (0.903 to 1.175)	0.672	1.022 (0.968 to 1.078)	0.436	1.034 (0.974 to 1.098)	0.275	1.040 (0.985 to 1.098)	0.209
2s,3R-dihydroxybutyrate	Hypertension	11	1.026 (0.954 to 1.104)	0.507	0.992 (0.943 to 1.043)	0.747	0.983 (0.941 to 1.027)	0.451		
Cysteinylglycine disulfide	Hypertension	8	1.045 (0.990 to 1.104)	0.160	1.055 (1.021 to 1.089)	0.001	1.050 (1.020 to 1.081)	0.001		
2,3-dihydroxyisovalerate	Hypertension	15	0.976 (0.910 to 1.047)	0.515	0.976 (0.926 to 1.028)	0.356	0.968 (0.934 to 1.003)	0.075		
Alpha-ketoglutaramate	Hypertension	7	0.992 (0.871 to 1.128)	0.903	0.999 (0.944 to 1.057)	0.974	1.002 (0.938 to 1.070)	0.953	0.970 (0.932 to 1.010)	0.213

Exposure	Outcome	nSNP	MR Egger		Weighted median		Inverse variance weighted		MR_PRESSO	
			OR (95% CI)	pval	OR (95% CI)	pval	OR (95% CI)	pval	OR (95% CI)	pval
3-methylglutaconate	Hypertension	13	0.975 (0.920 to 1.033)	0.402	0.963 (0.924 to 1.003)	0.068	0.983 (0.951 to 1.016)	0.311	0.992 (0.935 to 1.053)	0.807
(16 or 17)-methylstearate (a19:0 or i19:0)	Hypertension	13	0.983 (0.944 to 1.024)	0.427	0.977 (0.950 to 1.005)	0.108	0.981 (0.951 to 1.013)	0.246		
Cinnamoylglycine	Hypertension	20	0.986 (0.911 to 1.067)	0.731	0.953 (0.910 to 0.999)	0.046	0.958 (0.923 to 0.994)	0.022		
2R,3R-dihydroxybutyrate	Hypertension	16	1.071 (0.981 to 1.169)	0.146	1.018 (0.979 to 1.058)	0.372	1.004 (0.967 to 1.041)	0.849	0.984 (0.955 to 1.013)	0.288
4-hydroxyglutamate	Hypertension	17	0.961 (0.868 to 1.064)	0.453	0.996 (0.950 to 1.045)	0.867	1.020 (0.974 to 1.068)	0.393	1.010 (0.970 to 1.051)	0.631

Exposure	Outcome	nSNP	MR Egger		Weighted median		Inverse variance weighted		MR_PRESSO	
			OR (95% CI)	pval	OR (95% CI)	pval	OR (95% CI)	pval	OR (95% CI)	pval
S-methylcysteine	Hypertension	11	0.977 (0.921 to 1.037)	0.466	0.981 (0.943 to 1.021)	0.347	0.980 (0.952 to 1.010)	0.190		
Isoursodeoxycholate	Hypertension	9	1.083 (0.949 to 1.237)	0.276	0.975 (0.909 to 1.046)	0.481	0.975 (0.914 to 1.040)	0.443		
Cis-4-decenoate (10:1n6)	Hypertension	13	0.989 (0.949 to 1.031)	0.617	0.995 (0.966 to 1.025)	0.742	1.008 (0.982 to 1.034)	0.540		
4-oxo-retinoic acid	Hypertension	11	0.995 (0.864 to 1.145)	0.943	0.976 (0.919 to 1.036)	0.423	0.980 (0.919 to 1.044)	0.529		
1-lignoceroyl-GPC (24:0)	Hypertension	8	0.951 (0.844 to 1.072)	0.446	0.995 (0.935 to 1.058)	0.866	0.996 (0.950 to 1.043)	0.854		

Exposure	Outcome	nSNP	MR Egger		Weighted median		Inverse variance weighted		MR_PRESSO	
			OR (95% CI)	pval	OR (95% CI)	pval	OR (95% CI)	pval	OR (95% CI)	pval
Sulfate	Hypertension	20	0.973 (0.877 to 1.080)	0.612	1.035 (0.984 to 1.088)	0.180	1.049 (1.003 to 1.097)	0.038		
4-hydroxy-2-oxoglutaric acid	Hypertension	18	0.964 (0.885 to 1.050)	0.416	0.970 (0.920 to 1.023)	0.269	0.989 (0.947 to 1.032)	0.601		
Glycoursodeoxycholate	Hypertension	15	1.058 (0.984 to 1.137)	0.153	1.011 (0.959 to 1.066)	0.686	1.009 (0.963 to 1.056)	0.708		
1-(1-enyl-palmitoyl)-GPC (p-16:0)	Hypertension	13	1.065 (0.938 to 1.210)	0.351	0.977 (0.929 to 1.028)	0.372	0.990 (0.949 to 1.033)	0.651		
Argininate	Hypertension	14	0.979 (0.884 to 1.084)	0.689	0.950 (0.907 to 0.995)	0.031	0.963 (0.926 to 1.001)	0.059		

Exposure	Outcome	nSNP	MR Egger		Weighted median		Inverse variance weighted		MR_PRESSO	
			OR (95% CI)	pval	OR (95% CI)	pval	OR (95% CI)	pval	OR (95% CI)	pval
1-methyl-5-imidazoleacetate	Hypertension	12	1.063 (0.919 to 1.231)	0.428	1.026 (0.963 to 1.093)	0.424	1.035 (0.972 to 1.101)	0.285	1.006 (0.950 to 1.064)	0.851
Hydantoin-5-propionate	Hypertension	7	0.981 (0.838 to 1.148)	0.818	0.967 (0.914 to 1.024)	0.252	0.963 (0.922 to 1.006)	0.089		
2-oxoarginine	Hypertension	11	0.827 (0.680 to 1.006)	0.090	0.978 (0.923 to 1.037)	0.464	1.009 (0.936 to 1.089)	0.810	0.997 (0.943 to 1.054)	0.916
Pantoate	Hypertension	13	0.891 (0.777 to 1.022)	0.128	0.945 (0.893 to 1.000)	0.050	0.970 (0.911 to 1.032)	0.335	0.948 (0.906 to 0.993)	0.045
Formiminoglutamate	Hypertension	12	0.991 (0.816 to 1.204)	0.932	0.952 (0.893 to 1.016)	0.137	0.934 (0.847 to 1.031)	0.175	0.924 (0.876 to 0.975)	0.020

Exposure	Outcome	nSNP	MR Egger		Weighted median		Inverse variance weighted		MR_PRESSO	
			OR (95% CI)	pval	OR (95% CI)	pval	OR (95% CI)	pval	OR (95% CI)	pval
1-(1-enyl-palmitoyl)-GPE (p-16:0)	Hypertension	13	1.054 (0.921 to 1.207)	0.463	0.976 (0.919 to 1.037)	0.430	0.995 (0.944 to 1.049)	0.857		
S-methylcysteine sulfoxide	Hypertension	9	0.989 (0.914 to 1.070)	0.782	0.974 (0.923 to 1.028)	0.333	0.997 (0.956 to 1.038)	0.867		
2-o-methylascorbic acid	Hypertension	16	0.935 (0.886 to 0.987)	0.028	0.950 (0.926 to 0.973)	4.31e-05	0.971 (0.939 to 1.003)	0.077		
Eicosanedioate (C20-DC)	Hypertension	7	0.947 (0.869 to 1.032)	0.269	0.958 (0.911 to 1.007)	0.095	0.970 (0.932 to 1.010)	0.140		
1-(1-enyl-stearoyl)-GPE (p-18:0)	Hypertension	12	0.926 (0.679 to 1.261)	0.634	1.003 (0.945 to 1.065)	0.920	0.998 (0.934 to 1.065)	0.942	1.015 (0.967 to 1.065)	0.553

Exposure	Outcome	nSNP	MR Egger		Weighted median		Inverse variance weighted		MR_PRESSO	
			OR (95% CI)	pval	OR (95% CI)	pval	OR (95% CI)	pval	OR (95% CI)	pval
Beta-citrylglutamate	Hypertension	10	1.037 (0.981 to 1.097)	0.238	1.005 (0.979 to 1.032)	0.709	1.001 (0.970 to 1.034)	0.927		
N-oleoyltaurine	Hypertension	18	0.985 (0.896 to 1.082)	0.751	0.987 (0.950 to 1.026)	0.512	0.973 (0.936 to 1.011)	0.165	0.981 (0.948 to 1.016)	0.300
Docosadioate (C22-DC)	Hypertension	10	1.092 (0.960 to 1.242)	0.217	1.034 (0.968 to 1.105)	0.323	1.004 (0.933 to 1.080)	0.922	0.980 (0.918 to 1.047)	0.571
Oleoyl-linoleoyl-glycerol (18:1/18:2) [2]	Hypertension	20	1.112 (0.965 to 1.281)	0.159	1.058 (1.004 to 1.114)	0.033	1.052 (0.990 to 1.118)	0.103	1.075 (1.027 to 1.125)	0.007
Imidazole propionate	Hypertension	15	0.891 (0.777 to 1.022)	0.124	0.971 (0.924 to 1.020)	0.238	1.020 (0.954 to 1.090)	0.560	0.981 (0.945 to 1.019)	0.341

Exposure	Outcome	nSNP	MR Egger		Weighted median		Inverse variance weighted		MR_PRESSO	
			OR (95% CI)	pval	OR (95% CI)	pval	OR (95% CI)	pval	OR (95% CI)	pval
Carnitine C18:2	Hypertension	15	1.021 (0.929 to 1.123)	0.668	1.047 (1.003 to 1.094)	0.037	1.025 (0.983 to 1.069)	0.254	1.040 (1.002 to 1.080)	0.060
Pregnanediol-3-glucuronide	Hypertension	17	0.985 (0.905 to 1.072)	0.735	0.974 (0.935 to 1.014)	0.201	0.985 (0.952 to 1.019)	0.395		
Carboxyethyl-gaba	Hypertension	12	1.021 (0.915 to 1.139)	0.718	1.010 (0.961 to 1.062)	0.682	1.017 (0.975 to 1.060)	0.439	1.002 (0.966 to 1.040)	0.904
Alliin	Hypertension	12	0.938 (0.877 to 1.004)	0.094	0.946 (0.913 to 0.979)	0.002	0.987 (0.944 to 1.032)	0.562	1.040 (0.987 to 1.097)	0.172
Trimethylamine n-oxide	Hypertension	6	0.938 (0.786 to 1.120)	0.521	1.018 (0.941 to 1.101)	0.656	0.976 (0.910 to 1.048)	0.506		

Exposure	Outcome	nSNP	MR Egger		Weighted median		Inverse variance weighted		MR_PRESSO	
			OR (95% CI)	pval	OR (95% CI)	pval	OR (95% CI)	pval	OR (95% CI)	pval
Dihydroferulate	Hypertension	5	0.938 (0.839 to 1.049)	0.347	1.016 (0.937 to 1.102)	0.693	0.979 (0.916 to 1.047)	0.540		
Prolylglycine	Hypertension	11	0.923 (0.756 to 1.128)	0.455	0.951 (0.891 to 1.016)	0.135	1.010 (0.931 to 1.095)	0.815	0.966 (0.912 to 1.022)	0.259
N-palmitoylglycine	Hypertension	13	0.974 (0.907 to 1.045)	0.472	1.002 (0.963 to 1.042)	0.930	0.966 (0.934 to 1.000)	0.047		
N-methyltaurine	Hypertension	18	0.996 (0.947 to 1.047)	0.871	1.005 (0.970 to 1.042)	0.778	0.993 (0.967 to 1.020)	0.629		
Succinimide	Hypertension	9	0.975 (0.917 to 1.036)	0.436	0.998 (0.965 to 1.032)	0.902	0.995 (0.959 to 1.033)	0.808		

Exposure	Outcome	nSNP	MR Egger		Weighted median		Inverse variance weighted		MR_PRESSO	
			OR (95% CI)	pval	OR (95% CI)	pval	OR (95% CI)	pval	OR (95% CI)	pval
N-acetylcarnosine	Hypertension	17	1.166 (1.010 to 1.345)	0.054	1.010 (0.971 to 1.050)	0.630	0.975 (0.911 to 1.043)	0.455	0.996 (0.973 to 1.019)	0.740
Histidine betaine (hercynine)	Hypertension	11	1.070 (0.957 to 1.196)	0.267	1.015 (0.961 to 1.072)	0.588	0.988 (0.942 to 1.036)	0.618		
2-stearoyl-GPE (18:0)	Hypertension	19	0.960 (0.891 to 1.035)	0.303	1.022 (0.982 to 1.064)	0.281	1.030 (0.996 to 1.065)	0.088		
Margaroylcarnitine (C17)	Hypertension	12	1.043 (0.829 to 1.311)	0.727	0.980 (0.909 to 1.055)	0.585	1.017 (0.948 to 1.090)	0.644		
(R)-3-hydroxybutyrylcarnitine	Hypertension	10	0.878 (0.700 to 1.103)	0.296	0.987 (0.916 to 1.063)	0.722	0.994 (0.924 to 1.069)	0.861		

Exposure	Outcome	nSNP	MR Egger		Weighted median		Inverse variance weighted		MR_PRESSO	
			OR (95% CI)	pval	OR (95% CI)	pval	OR (95% CI)	pval	OR (95% CI)	pval
Lanthionine	Hypertension	16	0.940 (0.736 to 1.200)	0.627	0.962 (0.905 to 1.022)	0.213	0.991 (0.927 to 1.061)	0.801	0.966 (0.922 to 1.012)	0.168
Mannonate	Hypertension	11	0.969 (0.947 to 0.992)	0.025	0.967 (0.947 to 0.987)	0.002	0.960 (0.940 to 0.981)	2.53e-04		
3b-hydroxy-5-cholenoic acid	Hypertension	13	1.057 (0.935 to 1.194)	0.394	1.008 (0.960 to 1.058)	0.759	1.005 (0.953 to 1.060)	0.864	0.989 (0.945 to 1.035)	0.641
2-hydroxydecanoate	Hypertension	12	0.923 (0.835 to 1.021)	0.150	0.976 (0.920 to 1.037)	0.435	0.962 (0.917 to 1.009)	0.113		
Glycohyocholate	Hypertension	11	1.034 (0.923 to 1.159)	0.575	1.008 (0.954 to 1.066)	0.766	0.973 (0.924 to 1.025)	0.302		

Exposure	Outcome	nSNP	MR Egger		Weighted median		Inverse variance weighted		MR_PRESSO	
			OR (95% CI)	pval	OR (95% CI)	pval	OR (95% CI)	pval	OR (95% CI)	pval
3-methyl catechol sulfate (2)	Hypertension	15	0.984 (0.928 to 1.042)	0.586	0.999 (0.960 to 1.039)	0.946	0.999 (0.967 to 1.032)	0.960		
2-aminooctanoate	Hypertension	16	0.951 (0.910 to 0.994)	0.044	0.964 (0.940 to 0.989)	0.005	0.975 (0.947 to 1.003)	0.079		
3-methyl catechol sulfate (1)	Hypertension	9	1.075 (0.965 to 1.197)	0.232	1.050 (0.987 to 1.117)	0.119	1.037 (0.965 to 1.114)	0.323	1.024 (0.961 to 1.090)	0.491
4-methylcatechol sulfate	Hypertension	13	0.982 (0.855 to 1.127)	0.800	1.016 (0.956 to 1.080)	0.606	1.007 (0.952 to 1.066)	0.803	1.025 (0.980 to 1.072)	0.305
Guaiacol sulfate	Hypertension	14	0.923 (0.810 to 1.051)	0.249	1.007 (0.944 to 1.073)	0.840	0.960 (0.902 to 1.022)	0.203	0.977 (0.922 to 1.037)	0.460

Exposure	Outcome	nSNP	MR Egger		Weighted median		Inverse variance weighted		MR_PRESSO	
			OR (95% CI)	pval	OR (95% CI)	pval	OR (95% CI)	pval	OR (95% CI)	pval
Gamma-CEHC glucuronide	Hypertension	13	0.948 (0.895 to 1.004)	0.097	0.975 (0.936 to 1.014)	0.206	0.990 (0.956 to 1.026)	0.598		
Dimethyl sulfone	Hypertension	13	0.985 (0.911 to 1.066)	0.716	1.019 (0.966 to 1.074)	0.491	0.996 (0.953 to 1.041)	0.847		
2-piperidinone	Hypertension	14	0.974 (0.902 to 1.052)	0.514	0.990 (0.944 to 1.039)	0.687	0.993 (0.957 to 1.031)	0.728		
Indolin-2-one	Hypertension	9	1.036 (0.915 to 1.174)	0.593	1.003 (0.951 to 1.058)	0.913	1.029 (0.979 to 1.081)	0.257		
Sphingomyelin (d18:1/14:0, d16:1/16:0)	Hypertension	18	1.007 (0.988 to 1.026)	0.492	1.006 (0.992 to 1.021)	0.388	1.002 (0.986 to 1.019)	0.767		

Exposure	Outcome	nSNP	MR Egger		Weighted median		Inverse variance weighted		MR_PRESSO	
			OR (95% CI)	pval	OR (95% CI)	pval	OR (95% CI)	pval	OR (95% CI)	pval
3-acetylphenol sulfate	Hypertension	12	0.883 (0.762 to 1.025)	0.133	1.011 (0.960 to 1.065)	0.671	1.030 (0.948 to 1.119)	0.483	0.990 (0.946 to 1.036)	0.665
Sphingomyelin (d18:2/16:0, d18:1/16:1)	Hypertension	14	1.008 (0.906 to 1.122)	0.889	1.028 (0.966 to 1.093)	0.382	1.022 (0.976 to 1.070)	0.361		
S-allylcysteine	Hypertension	9	0.966 (0.854 to 1.093)	0.600	1.005 (0.952 to 1.062)	0.849	1.002 (0.961 to 1.044)	0.942		
6-oxopiperidine-2-carboxylate	Hypertension	11	0.980 (0.928 to 1.035)	0.491	0.980 (0.946 to 1.015)	0.263	1.001 (0.965 to 1.038)	0.970		
2-aminophenol sulfate	Hypertension	18	1.110 (1.030 to 1.198)	0.015	0.992 (0.942 to 1.045)	0.759	1.020 (0.972 to 1.070)	0.429	0.996 (0.949 to 1.046)	0.886

Exposure	Outcome	nSNP	MR Egger		Weighted median		Inverse variance weighted		MR_PRESSO	
			OR (95% CI)	pval	OR (95% CI)	pval	OR (95% CI)	pval	OR (95% CI)	pval
N-acetylalliin	Hypertension	14	0.954 (0.875 to 1.042)	0.316	0.960 (0.920 to 1.002)	0.063	0.970 (0.940 to 1.001)	0.056		
2-aminoheptanoate	Hypertension	10	1.017 (0.826 to 1.251)	0.878	0.938 (0.866 to 1.016)	0.114	0.939 (0.872 to 1.011)	0.095	0.968 (0.901 to 1.039)	0.391
3-methoxytyramine sulfate	Hypertension	9	0.985 (0.886 to 1.095)	0.785	0.976 (0.931 to 1.023)	0.308	0.974 (0.926 to 1.025)	0.308	0.955 (0.923 to 0.988)	0.032
N2,N5-diacetylornithine	Hypertension	17	1.002 (0.965 to 1.040)	0.914	0.989 (0.960 to 1.019)	0.473	1.006 (0.980 to 1.034)	0.639	1.021 (0.976 to 1.067)	0.381
Ferulic acid 4-sulfate	Hypertension	21	1.012 (0.948 to 1.081)	0.717	1.029 (0.995 to 1.064)	0.096	1.044 (1.009 to 1.081)	0.013	1.036 (1.006 to 1.066)	0.027

Exposure	Outcome	nSNP	MR Egger		Weighted median		Inverse variance weighted		MR_PRESSO	
			OR (95% CI)	pval	OR (95% CI)	pval	OR (95% CI)	pval	OR (95% CI)	pval
1H-indole-7-acetic acid	Hypertension	13	0.993 (0.918 to 1.075)	0.872	1.008 (0.958 to 1.061)	0.748	0.993 (0.951 to 1.036)	0.730		
3-(3-hydroxyphenyl)propionate sulfate	Hypertension	11	0.975 (0.881 to 1.078)	0.632	0.992 (0.943 to 1.044)	0.761	0.995 (0.954 to 1.038)	0.825		
1-linolenoyl-GPC (18:3)	Hypertension	21	1.059 (0.934 to 1.201)	0.379	1.053 (1.001 to 1.108)	0.047	1.043 (0.997 to 1.091)	0.067	1.027 (0.989 to 1.066)	0.187
O-sulfo-l-tyrosine	Hypertension	18	0.936 (0.775 to 1.131)	0.504	0.967 (0.922 to 1.014)	0.167	1.014 (0.937 to 1.097)	0.735	0.981 (0.941 to 1.023)	0.381
N-formylanthranilic acid	Hypertension	15	0.981 (0.930 to 1.035)	0.500	0.980 (0.956 to 1.005)	0.116	0.976 (0.942 to 1.012)	0.192	0.986 (0.960 to 1.014)	0.355

Exposure	Outcome	nSNP	MR Egger		Weighted median		Inverse variance weighted		MR_PRESSO	
			OR (95% CI)	pval	OR (95% CI)	pval	OR (95% CI)	pval	OR (95% CI)	pval
Methionine sulfone	Hypertension	24	0.954 (0.901 to 1.009)	0.114	0.948 (0.919 to 0.977)	5.96e-04	0.967 (0.938 to 0.997)	0.034	0.975 (0.950 to 1.000)	0.067
Acisoga	Hypertension	20	1.032 (0.973 to 1.095)	0.302	1.022 (0.989 to 1.057)	0.199	1.030 (0.998 to 1.062)	0.063	1.024 (0.996 to 1.053)	0.114
17alpha-hydroxypregnanolone glucuronide	Hypertension	18	0.993 (0.933 to 1.057)	0.839	1.012 (0.974 to 1.051)	0.547	1.024 (0.984 to 1.065)	0.249	1.015 (0.981 to 1.049)	0.405
Etiocholanolone glucuronide	Hypertension	13	1.008 (0.961 to 1.058)	0.742	1.005 (0.970 to 1.040)	0.795	0.987 (0.954 to 1.021)	0.438		
Fructosyllysine	Hypertension	15	0.984 (0.863 to 1.121)	0.809	0.959 (0.902 to 1.020)	0.187	0.987 (0.928 to 1.050)	0.685	0.994 (0.940 to 1.051)	0.834

Exposure	Outcome	nSNP	MR Egger		Weighted median		Inverse variance weighted		MR_PRESSO	
			OR (95% CI)	pval	OR (95% CI)	pval	OR (95% CI)	pval	OR (95% CI)	pval
1-(1-enyl-oleoyl)-GPE (p-18:1)	Hypertension	7	0.978 (0.937 to 1.020)	0.350	0.983 (0.947 to 1.021)	0.368	0.985 (0.955 to 1.016)	0.345		
N-acetyltaurine	Hypertension	13	0.996 (0.895 to 1.109)	0.946	1.015 (0.964 to 1.068)	0.575	1.041 (0.976 to 1.110)	0.220	1.015 (0.976 to 1.056)	0.466
1-palmitoyl-GPG (16:0)	Hypertension	17	0.985 (0.907 to 1.069)	0.717	0.977 (0.939 to 1.017)	0.255	0.985 (0.951 to 1.021)	0.403		
1-oleoyl-GPG (18:1)	Hypertension	13	0.936 (0.877 to 0.999)	0.071	0.970 (0.942 to 0.999)	0.046	1.004 (0.965 to 1.044)	0.851	1.029 (0.992 to 1.068)	0.162
Octadecenedioylcarnitine (C18:1-DC)	Hypertension	13	0.977 (0.946 to 1.008)	0.176	0.973 (0.950 to 0.996)	0.024	0.978 (0.959 to 0.998)	0.028		

Exposure	Outcome	nSNP	MR Egger		Weighted median		Inverse variance weighted		MR_PRESSO	
			OR (95% CI)	pval	OR (95% CI)	pval	OR (95% CI)	pval	OR (95% CI)	pval
3-methylglutarylcarnitine (2)	Hypertension	21	0.938 (0.881 to 0.999)	0.062	1.004 (0.966 to 1.044)	0.821	1.008 (0.970 to 1.048)	0.686	1.032 (0.997 to 1.069)	0.094
Sphingomyelin (d18:1/24:1, d18:2/24:0)	Hypertension	8	1.030 (0.890 to 1.192)	0.705	1.041 (0.972 to 1.115)	0.249	1.047 (0.991 to 1.106)	0.105		
Methyl glucopyranoside (alpha + beta)	Hypertension	17	0.986 (0.955 to 1.017)	0.388	0.988 (0.964 to 1.013)	0.347	0.989 (0.969 to 1.009)	0.270		
9-hydroxystearate	Hypertension	16	0.871 (0.763 to 0.995)	0.062	0.929 (0.876 to 0.984)	0.012	0.930 (0.884 to 0.978)	0.005		
Sphingomyelin (d18:2/14:0, d18:1/14:1)	Hypertension	14	0.900 (0.733 to 1.105)	0.335	0.961 (0.896 to 1.030)	0.262	0.918 (0.843 to 1.001)	0.052	0.969 (0.925 to 1.015)	0.212

Exposure	Outcome	nSNP	MR Egger		Weighted median		Inverse variance weighted		MR_PRESSO	
			OR (95% CI)	pval	OR (95% CI)	pval	OR (95% CI)	pval	OR (95% CI)	pval
1-dihomo-linolenylglycerol (20:3)	Hypertension	13	1.048 (0.897 to 1.225)	0.566	0.972 (0.921 to 1.027)	0.312	0.961 (0.909 to 1.015)	0.155		
4-hydroxychlorothalonil	Hypertension	13	0.969 (0.910 to 1.032)	0.353	0.968 (0.932 to 1.006)	0.097	0.998 (0.959 to 1.039)	0.931		
Vanillactate	Hypertension	15	0.987 (0.867 to 1.123)	0.843	1.060 (0.995 to 1.128)	0.069	1.031 (0.974 to 1.091)	0.297	1.064 (1.017 to 1.112)	0.018
Carnitine C14:1	Hypertension	16	1.015 (0.907 to 1.135)	0.801	1.014 (0.957 to 1.074)	0.636	0.992 (0.934 to 1.053)	0.785	0.969 (0.925 to 1.016)	0.215
N-formylphenylalanine	Hypertension	14	1.058 (0.956 to 1.171)	0.295	1.024 (0.971 to 1.080)	0.381	1.032 (0.990 to 1.076)	0.134		

Exposure	Outcome	nSNP	MR Egger		Weighted median		Inverse variance weighted		MR_PRESSO	
			OR (95% CI)	pval	OR (95% CI)	pval	OR (95% CI)	pval	OR (95% CI)	pval
2-acetamidophenol sulfate	Hypertension	10	1.009 (0.909 to 1.120)	0.876	1.027 (0.968 to 1.091)	0.376	1.024 (0.969 to 1.081)	0.401	1.008 (0.962 to 1.057)	0.738
4-vinylguaiacol sulfate	Hypertension	6	1.002 (0.706 to 1.424)	0.990	1.077 (0.988 to 1.174)	0.094	1.075 (0.958 to 1.208)	0.218	1.070 (1.025 to 1.117)	0.053
5alpha-androstan-3alpha,17beta-diol monosulfate (2)	Hypertension	18	1.003 (0.898 to 1.120)	0.959	1.053 (0.999 to 1.110)	0.052	1.020 (0.965 to 1.078)	0.483	1.037 (0.985 to 1.093)	0.184
Vanillic alcohol sulfate	Hypertension	14	1.011 (0.941 to 1.087)	0.763	0.964 (0.918 to 1.013)	0.147	0.950 (0.911 to 0.990)	0.015		
Methyl-4-hydroxybenzoate sulfate	Hypertension	13	0.960 (0.880 to 1.047)	0.375	0.969 (0.919 to 1.022)	0.253	0.971 (0.933 to 1.010)	0.148		

Exposure	Outcome	nSNP	MR Egger		Weighted median		Inverse variance weighted		MR_PRESSO	
			OR (95% CI)	pval	OR (95% CI)	pval	OR (95% CI)	pval	OR (95% CI)	pval
Eugenol sulfate	Hypertension	10	1.029 (0.961 to 1.101)	0.433	1.007 (0.963 to 1.053)	0.763	1.014 (0.979 to 1.051)	0.430		
2-methoxyresorcinol sulfate	Hypertension	12	0.961 (0.870 to 1.061)	0.448	0.963 (0.917 to 1.010)	0.120	0.966 (0.930 to 1.003)	0.073		
Tyramine O-sulfate	Hypertension	9	0.992 (0.924 to 1.064)	0.825	1.001 (0.949 to 1.057)	0.960	0.998 (0.957 to 1.040)	0.910		
4-methylguaiacol sulfate	Hypertension	9	1.011 (0.899 to 1.137)	0.858	0.955 (0.901 to 1.013)	0.124	0.966 (0.917 to 1.018)	0.200		
3-hydroxypyridine sulfate	Hypertension	12	0.996 (0.872 to 1.139)	0.959	0.956 (0.899 to 1.015)	0.142	0.980 (0.922 to 1.041)	0.504	0.962 (0.922 to 1.004)	0.103

Exposure	Outcome	nSNP	MR Egger		Weighted median		Inverse variance weighted		MR_PRESSO	
			OR (95% CI)	pval	OR (95% CI)	pval	OR (95% CI)	pval	OR (95% CI)	pval
Arabonate/xylonate	Hypertension	16	1.094 (0.893 to 1.340)	0.401	1.089 (1.028 to 1.153)	0.004	1.060 (0.977 to 1.149)	0.160	1.079 (1.038 to 1.122)	0.002
Octadecanedioylcarnitine (C18-DC)	Hypertension	16	0.973 (0.938 to 1.009)	0.165	0.971 (0.948 to 0.994)	0.016	0.982 (0.960 to 1.003)	0.096		
Phenylacetylcarnitine	Hypertension	16	0.980 (0.895 to 1.074)	0.677	1.032 (0.983 to 1.083)	0.204	1.019 (0.979 to 1.059)	0.355		
Ethylparaben sulfate	Hypertension	12	1.092 (0.976 to 1.222)	0.156	1.011 (0.948 to 1.079)	0.735	0.997 (0.945 to 1.052)	0.910		
Sphingomyelin (d18:1/20:2, d18:2/20:1, d16:1/22:2)	Hypertension	14	0.972 (0.868 to 1.089)	0.638	1.018 (0.972 to 1.066)	0.442	1.000 (0.957 to 1.045)	0.989	1.013 (0.975 to 1.052)	0.517

Exposure	Outcome	nSNP	MR Egger		Weighted median		Inverse variance weighted		MR_PRESSO	
			OR (95% CI)	pval	OR (95% CI)	pval	OR (95% CI)	pval	OR (95% CI)	pval
Sphingomyelin (d18:1/20:1, d18:2/20:0)	Hypertension	18	1.050 (0.968 to 1.138)	0.255	0.998 (0.959 to 1.040)	0.932	0.997 (0.960 to 1.036)	0.896	0.989 (0.959 to 1.021)	0.521
Behenoyl sphingomyelin (d18:1/22:0)	Hypertension	10	0.958 (0.843 to 1.089)	0.529	1.000 (0.937 to 1.068)	0.996	1.020 (0.966 to 1.077)	0.479		
Propyl 4-hydroxybenzoate sulfate	Hypertension	10	1.033 (0.794 to 1.345)	0.813	1.007 (0.938 to 1.081)	0.843	0.973 (0.888 to 1.066)	0.558	0.984 (0.930 to 1.041)	0.594
4-methoxyphenol sulfate	Hypertension	13	1.110 (0.968 to 1.272)	0.164	1.024 (0.962 to 1.089)	0.457	0.999 (0.941 to 1.061)	0.970	1.024 (0.977 to 1.074)	0.346
Umbelliferone sulfate	Hypertension	13	1.034 (0.969 to 1.105)	0.336	1.028 (0.992 to 1.064)	0.131	1.024 (0.987 to 1.061)	0.203	1.032 (1.001 to 1.064)	0.071

Exposure	Outcome	nSNP	MR Egger		Weighted median		Inverse variance weighted		MR_PRESSO	
			OR (95% CI)	pval	OR (95% CI)	pval	OR (95% CI)	pval	OR (95% CI)	pval
Sphingomyelin (d18:1/20:0, d16:1/22:0)	Hypertension	18	0.983 (0.879 to 1.100)	0.772	0.978 (0.941 to 1.017)	0.264	1.009 (0.969 to 1.051)	0.663	1.000 (0.965 to 1.036)	0.992
6-hydroxyindole sulfate	Hypertension	8	0.905 (0.815 to 1.005)	0.111	0.964 (0.902 to 1.031)	0.285	0.973 (0.926 to 1.023)	0.285		
P-cresol glucuronide	Hypertension	14	1.024 (0.937 to 1.119)	0.610	0.994 (0.940 to 1.052)	0.844	0.987 (0.950 to 1.026)	0.504		
N-carbamoylalanine	Hypertension	10	1.052 (0.914 to 1.210)	0.503	0.971 (0.909 to 1.038)	0.386	1.008 (0.961 to 1.058)	0.738		
Citraconate/glutaconate	Hypertension	15	1.060 (0.964 to 1.166)	0.250	1.009 (0.954 to 1.067)	0.763	1.002 (0.952 to 1.054)	0.940		

Exposure	Outcome	nSNP	MR Egger		Weighted median		Inverse variance weighted		MR_PRESSO	
			OR (95% CI)	pval	OR (95% CI)	pval	OR (95% CI)	pval	OR (95% CI)	pval
1,2,3-benzenetriol sulfate (2)	Hypertension	11	1.055 (0.988 to 1.128)	0.145	1.044 (0.997 to 1.094)	0.066	1.032 (0.990 to 1.076)	0.136		
3-methoxycatechol sulfate (2)	Hypertension	15	0.980 (0.893 to 1.076)	0.681	1.023 (0.971 to 1.077)	0.394	1.032 (0.980 to 1.087)	0.232	1.011 (0.977 to 1.046)	0.550
C-glycosyltryptophan	Hypertension	25	0.952 (0.814 to 1.112)	0.540	1.072 (1.019 to 1.129)	0.008	1.037 (0.979 to 1.099)	0.211	1.049 (1.010 to 1.089)	0.022
3-methoxycatechol sulfate (1)	Hypertension	15	0.905 (0.789 to 1.037)	0.175	0.988 (0.932 to 1.047)	0.685	0.987 (0.932 to 1.046)	0.660	0.986 (0.949 to 1.024)	0.471
Sphingomyelin (d18:1/22:1, d18:2/22:0, d16:1/24:1)	Hypertension	16	0.974 (0.887 to 1.068)	0.579	0.970 (0.926 to 1.017)	0.207	1.002 (0.960 to 1.045)	0.937		

Exposure	Outcome	nSNP	MR Egger		Weighted median		Inverse variance weighted		MR_PRESSO	
			OR (95% CI)	pval	OR (95% CI)	pval	OR (95% CI)	pval	OR (95% CI)	pval
Arabitol/xylitol	Hypertension	15	0.923 (0.750 to 1.136)	0.464	0.968 (0.909 to 1.032)	0.323	1.014 (0.937 to 1.098)	0.723	0.977 (0.925 to 1.032)	0.419
3-hydroxyhexanoate	Hypertension	9	1.027 (0.895 to 1.180)	0.712	1.016 (0.951 to 1.086)	0.637	0.995 (0.946 to 1.046)	0.835		
Sphingomyelin (d18:1/22:2, d18:2/22:1, d16:1/24:2)	Hypertension	14	0.918 (0.817 to 1.031)	0.176	0.973 (0.923 to 1.025)	0.296	0.961 (0.913 to 1.011)	0.127		
3beta-hydroxy-5-cholestenoate	Hypertension	18	0.984 (0.918 to 1.056)	0.668	0.983 (0.951 to 1.016)	0.309	0.996 (0.957 to 1.036)	0.839	0.983 (0.952 to 1.015)	0.304
N-acetylkynurenine (2)	Hypertension	11	1.060 (1.022 to 1.101)	0.013	1.032 (1.011 to 1.055)	0.003	1.021 (0.998 to 1.044)	0.070		

Exposure	Outcome	nSNP	MR Egger		Weighted median		Inverse variance weighted		MR_PRESSO	
			OR (95% CI)	pval	OR (95% CI)	pval	OR (95% CI)	pval	OR (95% CI)	pval
Dopamine 4-sulfate	Hypertension	16	1.022 (0.946 to 1.104)	0.586	1.010 (0.968 to 1.055)	0.642	1.001 (0.960 to 1.043)	0.977	1.011 (0.974 to 1.049)	0.582
Sphingomyelin (d17:1/16:0, d18:1/15:0, d16:1/17:0)	Hypertension	15	0.980 (0.889 to 1.081)	0.691	0.957 (0.915 to 1.001)	0.057	0.980 (0.932 to 1.031)	0.442		
Adipoylcarnitine (C6-DC)	Hypertension	15	0.963 (0.861 to 1.078)	0.524	0.988 (0.946 to 1.033)	0.599	0.992 (0.950 to 1.036)	0.723	1.003 (0.965 to 1.042)	0.893
Dopamine 3-o-sulfate	Hypertension	11	1.009 (0.961 to 1.060)	0.718	1.005 (0.968 to 1.043)	0.788	1.017 (0.984 to 1.052)	0.309		
Lignoceroyl sphingomyelin (d18:1/24:0)	Hypertension	14	1.103 (0.943 to 1.290)	0.244	0.958 (0.906 to 1.013)	0.130	0.991 (0.935 to 1.050)	0.763	0.968 (0.933 to 1.004)	0.104

Exposure	Outcome	nSNP	MR Egger		Weighted median		Inverse variance weighted		MR_PRESSO	
			OR (95% CI)	pval	OR (95% CI)	pval	OR (95% CI)	pval	OR (95% CI)	pval
N-acetylglucosamine/n-acetylgalactosamine	Hypertension	12	1.004 (0.932 to 1.080)	0.927	1.020 (0.984 to 1.058)	0.273	1.026 (0.988 to 1.067)	0.184		
Glycochenodeoxycholate 3-sulfate	Hypertension	21	1.027 (0.970 to 1.088)	0.373	1.008 (0.974 to 1.044)	0.641	0.990 (0.959 to 1.023)	0.548	0.996 (0.969 to 1.024)	0.783
Trans 3,4-methyleneheptanoate	Hypertension	9	0.949 (0.772 to 1.165)	0.630	0.972 (0.912 to 1.036)	0.387	0.981 (0.913 to 1.053)	0.589	0.952 (0.908 to 0.999)	0.084
Taurodeoxycholic acid 3-sulfate	Hypertension	11	0.969 (0.935 to 1.004)	0.119	0.977 (0.955 to 0.999)	0.041	0.980 (0.961 to 1.000)	0.054		
Phenol glucuronide	Hypertension	13	0.967 (0.927 to 1.008)	0.139	0.993 (0.958 to 1.029)	0.692	0.986 (0.960 to 1.012)	0.289		

Exposure	Outcome	nSNP	MR Egger		Weighted median		Inverse variance weighted		MR_PRESSO	
			OR (95% CI)	pval	OR (95% CI)	pval	OR (95% CI)	pval	OR (95% CI)	pval
Glycodeoxycholate 3-sulfate	Hypertension	18	0.971 (0.951 to 0.992)	0.015	0.988 (0.969 to 1.008)	0.230	0.989 (0.975 to 1.003)	0.132		
4-hydroxyphenylacetoylcarnitine	Hypertension	17	1.033 (0.962 to 1.109)	0.385	1.023 (0.976 to 1.072)	0.341	1.008 (0.976 to 1.042)	0.628		
1-stearoyl-2-oleoyl-gpc (18:0/18:1)	Hypertension	20	1.084 (0.986 to 1.192)	0.111	0.998 (0.958 to 1.040)	0.940	0.992 (0.962 to 1.024)	0.636		
Linoleoyl ethanolamide	Hypertension	10	0.976 (0.876 to 1.087)	0.669	1.009 (0.964 to 1.056)	0.692	1.032 (0.989 to 1.078)	0.148		
1,2-dilinoleoyl-GPC (18:2/18:2)	Hypertension	10	1.014 (0.941 to 1.092)	0.732	1.000 (0.966 to 1.034)	0.982	1.002 (0.963 to 1.043)	0.910		

Exposure	Outcome	nSNP	MR Egger		Weighted median		Inverse variance weighted		MR_PRESSO	
			OR (95% CI)	pval	OR (95% CI)	pval	OR (95% CI)	pval	OR (95% CI)	pval
Nonanoylcarnitine (C9)	Hypertension	10	1.061 (1.013 to 1.111)	0.037	1.000 (0.970 to 1.032)	0.989	0.986 (0.948 to 1.026)	0.501		
2-hydroxybutyrate/2-hydroxyisobutyrate	Hypertension	6	0.906 (0.675 to 1.215)	0.545	0.997 (0.918 to 1.084)	0.950	1.045 (0.939 to 1.164)	0.417	1.004 (0.921 to 1.093)	0.939
Sphingomyelin (d18:2/23:0, d18:1/23:1, d17:1/24:1)	Hypertension	11	0.972 (0.867 to 1.090)	0.636	0.973 (0.916 to 1.034)	0.379	0.968 (0.916 to 1.023)	0.252		
Tricosanoyl sphingomyelin (d18:1/23:0)	Hypertension	14	0.986 (0.846 to 1.149)	0.856	0.972 (0.913 to 1.035)	0.379	0.999 (0.932 to 1.070)	0.967	0.982 (0.923 to 1.045)	0.578
Palmitoyl dihydrosphingomyelin (d18:0/16:0)	Hypertension	23	1.140 (1.052 to 1.234)	0.004	1.038 (0.994 to 1.084)	0.089	1.022 (0.985 to 1.060)	0.248		

Exposure	Outcome	nSNP	MR Egger		Weighted median		Inverse variance weighted		MR_PRESSO	
			OR (95% CI)	pval	OR (95% CI)	pval	OR (95% CI)	pval	OR (95% CI)	pval
1-palmitoleoylglycerol (16:1)	Hypertension	21	0.977 (0.930 to 1.026)	0.361	0.979 (0.946 to 1.013)	0.219	0.978 (0.954 to 1.003)	0.083		
1-stearoyl-2-linoleoyl-gpc (18:0/18:2)	Hypertension	16	1.020 (0.917 to 1.135)	0.715	1.017 (0.974 to 1.062)	0.447	1.015 (0.972 to 1.061)	0.499		
1-palmitoyl-2-palmitoleoyl-gpc (16:0/16:1)	Hypertension	14	0.940 (0.825 to 1.071)	0.371	0.962 (0.910 to 1.017)	0.174	0.997 (0.945 to 1.052)	0.909	0.981 (0.939 to 1.024)	0.397
1-stearoyl-2-linoleoyl-GPE (18:0/18:2)	Hypertension	16	1.018 (0.923 to 1.123)	0.724	1.014 (0.987 to 1.041)	0.304	1.024 (0.982 to 1.069)	0.261	1.021 (0.999 to 1.044)	0.088
Sphingomyelin (d18:2/24:1, d18:1/24:2)	Hypertension	16	1.010 (0.845 to 1.207)	0.918	0.943 (0.881 to 1.009)	0.091	0.914 (0.843 to 0.991)	0.029	0.921 (0.863 to 0.983)	0.029

Exposure	Outcome	nSNP	MR Egger		Weighted median		Inverse variance weighted		MR_PRESSO	
			OR (95% CI)	pval	OR (95% CI)	pval	OR (95% CI)	pval	OR (95% CI)	pval
1-palmitoyl-2-stearoyl-gpc (16:0/18:0)	Hypertension	18	1.033 (0.920 to 1.160)	0.590	0.985 (0.945 to 1.027)	0.480	1.016 (0.965 to 1.069)	0.546	1.008 (0.962 to 1.055)	0.750
Oleate/vaccenate (18:1)	Hypertension	14	1.008 (0.980 to 1.036)	0.603	0.999 (0.985 to 1.013)	0.909	1.000 (0.977 to 1.024)	0.987	1.000 (0.985 to 1.015)	0.993
1-palmitoyl-2-docosahexaenoyl-gpc (16:0/22:6)	Hypertension	12	0.991 (0.808 to 1.215)	0.931	0.992 (0.948 to 1.038)	0.722	1.025 (0.960 to 1.094)	0.459	1.002 (0.962 to 1.044)	0.934
1-(1-enyl-stearoyl)-2-oleoyl-GPE (p-18:0/18:1)	Hypertension	10	0.938 (0.847 to 1.040)	0.260	1.020 (0.957 to 1.088)	0.542	1.005 (0.952 to 1.062)	0.851		
1-stearoyl-2-arachidonoyl-GPE (18:0/20:4)	Hypertension	19	1.023 (0.952 to 1.100)	0.540	1.008 (0.984 to 1.032)	0.523	1.019 (0.982 to 1.057)	0.325	1.011 (0.988 to 1.034)	0.369

Exposure	Outcome	nSNP	MR Egger		Weighted median		Inverse variance weighted		MR_PRESSO	
			OR (95% CI)	pval	OR (95% CI)	pval	OR (95% CI)	pval	OR (95% CI)	pval
1-stearoyl-2-docosahexaenoyl-gpc (18:0/22:6)	Hypertension	18	0.958 (0.886 to 1.036)	0.297	0.994 (0.958 to 1.031)	0.752	1.024 (0.985 to 1.066)	0.231	1.015 (0.982 to 1.049)	0.381
Sphingomyelin (d18:1/17:0, d17:1/18:0, d19:1/16:0)	Hypertension	17	1.035 (0.914 to 1.172)	0.599	0.988 (0.936 to 1.042)	0.651	1.004 (0.957 to 1.053)	0.872		
1-(1-enyl-palmitoyl)-2-arachidonoyl-GPE (p-16:0/20:4)	Hypertension	10	1.024 (0.926 to 1.131)	0.657	0.982 (0.928 to 1.041)	0.545	0.984 (0.943 to 1.027)	0.466		
Myristoyl dihydrosphingomyelin (d18:0/14:0)	Hypertension	13	0.947 (0.885 to 1.013)	0.144	0.975 (0.934 to 1.017)	0.243	0.988 (0.954 to 1.023)	0.491		
5-hydroxyindole sulfate	Hypertension	10	0.963 (0.854 to 1.086)	0.556	1.029 (0.967 to 1.095)	0.362	1.037 (0.981 to 1.098)	0.202		

Exposure	Outcome	nSNP	MR Egger		Weighted median		Inverse variance weighted		MR_PRESSO	
			OR (95% CI)	pval	OR (95% CI)	pval	OR (95% CI)	pval	OR (95% CI)	pval
1-palmitoyl-2-dihomo-linolenoyl-GPC (16:0/20:3n3 or 6)	Hypertension	15	1.005 (0.958 to 1.054)	0.840	1.012 (0.981 to 1.044)	0.457	1.022 (0.997 to 1.047)	0.084		
1-(1-enyl-palmitoyl)-2-arachidonoyl-gpc (p-16:0/20:4)	Hypertension	23	1.004 (0.963 to 1.046)	0.856	0.994 (0.973 to 1.015)	0.555	0.989 (0.964 to 1.014)	0.386	0.992 (0.971 to 1.014)	0.473
Sphingomyelin (d18:1/21:0, d17:1/22:0, d16:1/23:0)	Hypertension	13	0.930 (0.852 to 1.016)	0.137	0.975 (0.934 to 1.018)	0.249	0.990 (0.945 to 1.037)	0.678		
1-palmitoyl-2-arachidonoyl-GPE (16:0/20:4)	Hypertension	14	1.015 (0.949 to 1.087)	0.665	1.005 (0.983 to 1.028)	0.636	1.005 (0.969 to 1.042)	0.805	1.003 (0.984 to 1.022)	0.764
Lactosyl-N-palmitoyl-sphingosine (d18:1/16:0)	Hypertension	14	1.019 (0.932 to 1.114)	0.690	0.994 (0.945 to 1.045)	0.802	0.987 (0.946 to 1.028)	0.521	1.015 (0.982 to 1.049)	0.389

Exposure	Outcome	nSNP	MR Egger		Weighted median		Inverse variance weighted		MR_PRESSO	
			OR (95% CI)	pval	OR (95% CI)	pval	OR (95% CI)	pval	OR (95% CI)	pval
Sphingomyelin (d18:0/18:0, d19:0/17:0)	Hypertension	19	0.963 (0.900 to 1.030)	0.283	0.991 (0.953 to 1.031)	0.654	1.015 (0.979 to 1.052)	0.414		
1-(1-enyl-palmitoyl)-2-oleoyl-gpc (p-16:0/18:1)	Hypertension	18	1.023 (0.968 to 1.081)	0.438	1.007 (0.971 to 1.044)	0.699	0.997 (0.968 to 1.027)	0.843		
1-(1-enyl-stearoyl)-2-arachidonoyl-GPE (p-18:0/20:4)	Hypertension	13	0.983 (0.898 to 1.076)	0.719	0.990 (0.955 to 1.025)	0.557	0.988 (0.944 to 1.034)	0.606	0.998 (0.965 to 1.033)	0.926
Behenoyl dihydrosphingomyelin (d18:0/22:0)	Hypertension	23	1.004 (0.913 to 1.104)	0.936	1.010 (0.972 to 1.049)	0.610	1.010 (0.969 to 1.052)	0.654	1.028 (0.993 to 1.064)	0.133
1-(1-enyl-palmitoyl)-2-linoleoyl-GPC (p-16:0/18:2)	Hypertension	11	0.872 (0.679 to 1.119)	0.308	0.926 (0.864 to 0.993)	0.031	0.907 (0.831 to 0.991)	0.030	0.937 (0.872 to 1.007)	0.109

Exposure	Outcome	nSNP	MR Egger		Weighted median		Inverse variance weighted		MR_PRESSO	
			OR (95% CI)	pval	OR (95% CI)	pval	OR (95% CI)	pval	OR (95% CI)	pval
1-(1-enyl-palmitoyl)-2-oleoyl-GPE (p-16:0/18:1)	Hypertension	17	1.003 (0.927 to 1.085)	0.942	0.971 (0.923 to 1.021)	0.247	0.951 (0.914 to 0.989)	0.012		
1-palmitoyl-2-arachidonoyl-GPI (16:0/20:4)	Hypertension	13	0.985 (0.895 to 1.084)	0.766	1.030 (0.979 to 1.083)	0.260	1.022 (0.986 to 1.058)	0.232		
1-palmitoyl-2-docosahexaenoyl-GPE (16:0/22:6)	Hypertension	12	0.989 (0.914 to 1.070)	0.790	1.009 (0.983 to 1.034)	0.510	1.027 (0.981 to 1.075)	0.261	1.038 (0.993 to 1.085)	0.136
N-palmitoyl-sphinganine (d18:0/16:0)	Hypertension	16	1.071 (0.814 to 1.408)	0.633	0.995 (0.938 to 1.055)	0.854	1.015 (0.935 to 1.102)	0.726	1.017 (0.968 to 1.068)	0.515
1-stearoyl-2-docosahexaenoyl-GPE (18:0/22:6)	Hypertension	18	1.040 (0.947 to 1.141)	0.424	1.012 (0.979 to 1.047)	0.473	1.028 (0.986 to 1.072)	0.190	1.016 (0.991 to 1.042)	0.229

Exposure	Outcome	nSNP	MR Egger		Weighted median		Inverse variance weighted		MR_PRESSO	
			OR (95% CI)	pval	OR (95% CI)	pval	OR (95% CI)	pval	OR (95% CI)	pval
Gamma-tocopherol/beta-tocopherol	Hypertension	15	1.025 (0.946 to 1.111)	0.555	0.971 (0.922 to 1.021)	0.250	0.985 (0.947 to 1.024)	0.434		
1-stearoyl-2-linoleoyl-GPI (18:0/18:2)	Hypertension	17	1.070 (0.936 to 1.222)	0.340	1.019 (0.983 to 1.058)	0.306	1.008 (0.950 to 1.070)	0.798	0.996 (0.960 to 1.034)	0.840
1,2-dilinoleoyl-GPE (18:2/18:2)	Hypertension	10	0.974 (0.902 to 1.051)	0.513	1.018 (0.978 to 1.060)	0.375	1.031 (1.000 to 1.064)	0.052		
Phenylacetylglutamate	Hypertension	14	0.922 (0.837 to 1.016)	0.127	0.962 (0.915 to 1.012)	0.130	0.982 (0.947 to 1.018)	0.315		
1-(1-enyl-palmitoyl)-2-palmitoleoyl-GPC (P-16:0/16:1)	Hypertension	24	0.980 (0.901 to 1.066)	0.644	0.994 (0.964 to 1.025)	0.703	0.963 (0.924 to 1.003)	0.072	0.954 (0.920 to 0.990)	0.021

Exposure	Outcome	nSNP	MR Egger		Weighted median		Inverse variance weighted		MR_PRESSO	
			OR (95% CI)	pval	OR (95% CI)	pval	OR (95% CI)	pval	OR (95% CI)	pval
Palmitoylcholine	Hypertension	19	1.051 (0.972 to 1.137)	0.229	0.976 (0.931 to 1.023)	0.312	1.004 (0.967 to 1.042)	0.843		
Thioproline	Hypertension	10	0.966 (0.903 to 1.032)	0.334	0.983 (0.937 to 1.031)	0.486	0.981 (0.946 to 1.016)	0.284		
1-linoleoyl-2-arachidonoyl-GPC (18:2/20:4n6)	Hypertension	20	0.976 (0.931 to 1.023)	0.322	0.979 (0.949 to 1.011)	0.199	0.968 (0.943 to 0.994)	0.016		
1-linoleoyl-GPG (18:2)	Hypertension	17	0.973 (0.946 to 1.001)	0.075	0.988 (0.963 to 1.014)	0.365	0.985 (0.967 to 1.004)	0.124		
1-(1-enyl-palmitoyl)-2-linoleoyl-GPE (p-16:0/18:2)	Hypertension	14	1.021 (0.854 to 1.219)	0.826	1.013 (0.967 to 1.061)	0.591	0.977 (0.932 to 1.024)	0.333	0.987 (0.953 to 1.023)	0.492

Exposure	Outcome	nSNP	MR Egger		Weighted median		Inverse variance weighted		MR_PRESSO	
			OR (95% CI)	pval	OR (95% CI)	pval	OR (95% CI)	pval	OR (95% CI)	pval
1-(1-enyl-stearoyl)-2-linoleoyl-GPE (p-18:0/18:2)	Hypertension	13	0.908 (0.808 to 1.021)	0.136	0.986 (0.943 to 1.032)	0.553	0.966 (0.927 to 1.007)	0.099		
1-stearoyl-2-oleoyl-GPI (18:0/18:1)	Hypertension	16	0.912 (0.811 to 1.025)	0.144	1.014 (0.976 to 1.053)	0.487	1.022 (0.976 to 1.070)	0.354	1.006 (0.977 to 1.036)	0.701
1-myristoyl-2-arachidonoyl-GPC (14:0/20:4)	Hypertension	18	0.962 (0.898 to 1.031)	0.292	0.987 (0.957 to 1.017)	0.395	0.974 (0.935 to 1.014)	0.197	0.974 (0.941 to 1.008)	0.154
1-oleoyl-2-arachidonoyl-GPE (18:1/20:4)	Hypertension	18	1.023 (0.964 to 1.086)	0.465	1.008 (0.984 to 1.032)	0.534	1.006 (0.974 to 1.040)	0.709	1.001 (0.978 to 1.025)	0.917
1-palmitoyl-2-oleoyl-GPI (16:0/18:1)	Hypertension	14	0.986 (0.890 to 1.093)	0.798	1.048 (1.003 to 1.096)	0.038	1.030 (0.992 to 1.069)	0.123		

Exposure	Outcome	nSNP	MR Egger		Weighted median		Inverse variance weighted		MR_PRESSO	
			OR (95% CI)	pval	OR (95% CI)	pval	OR (95% CI)	pval	OR (95% CI)	pval
1-oleoyl-2-docosahexaenoyl-GPC (18:1/22:6)	Hypertension	22	0.870 (0.739 to 1.025)	0.111	0.978 (0.929 to 1.029)	0.393	1.004 (0.946 to 1.065)	0.897	0.980 (0.936 to 1.027)	0.413
1-oleoyl-2-linoleoyl-GPE (18:1/18:2)	Hypertension	17	0.994 (0.921 to 1.072)	0.872	1.011 (0.986 to 1.036)	0.410	1.007 (0.968 to 1.049)	0.718	1.006 (0.981 to 1.031)	0.653
1-(1-enyl-palmitoyl)-2-palmitoyl-GPC (P-16:0/16:0)	Hypertension	21	1.009 (0.957 to 1.065)	0.736	0.993 (0.966 to 1.022)	0.642	0.991 (0.964 to 1.019)	0.513		
Oleoylcholine	Hypertension	8	1.024 (0.882 to 1.189)	0.766	0.976 (0.912 to 1.045)	0.489	1.019 (0.962 to 1.080)	0.521		
Glycosyl-N-palmitoyl-sphingosine (d18:1/16:0)	Hypertension	15	0.998 (0.919 to 1.083)	0.955	0.996 (0.957 to 1.037)	0.854	0.981 (0.943 to 1.021)	0.353	0.988 (0.953 to 1.025)	0.539

Exposure	Outcome	nSNP	MR Egger		Weighted median		Inverse variance weighted		MR_PRESSO	
			OR (95% CI)	pval	OR (95% CI)	pval	OR (95% CI)	pval	OR (95% CI)	pval
Arachidonoylcholine	Hypertension	10	0.969 (0.858 to 1.093)	0.620	0.982 (0.936 to 1.029)	0.443	0.999 (0.953 to 1.046)	0.958		
2-methylserine	Hypertension	14	0.923 (0.824 to 1.034)	0.194	0.968 (0.913 to 1.027)	0.280	1.001 (0.940 to 1.064)	0.986	0.978 (0.934 to 1.025)	0.374
(S)-3-hydroxybutyrylcarnitine	Hypertension	13	0.960 (0.798 to 1.155)	0.674	1.060 (1.000 to 1.123)	0.051	1.050 (0.993 to 1.110)	0.084	1.061 (1.017 to 1.107)	0.021
Methylsuccinoylcarnitine	Hypertension	20	0.970 (0.913 to 1.030)	0.330	1.001 (0.971 to 1.032)	0.946	1.023 (0.992 to 1.056)	0.149	1.017 (0.992 to 1.043)	0.197
Dihomo-linolenoyl-choline	Hypertension	17	0.902 (0.794 to 1.023)	0.130	0.992 (0.946 to 1.039)	0.723	0.977 (0.927 to 1.030)	0.392	0.982 (0.949 to 1.017)	0.330

Exposure	Outcome	nSNP	MR Egger		Weighted median		Inverse variance weighted		MR_PRESSO	
			OR (95% CI)	pval	OR (95% CI)	pval	OR (95% CI)	pval	OR (95% CI)	pval
Docosahexaenoylcholine	Hypertension	11	1.027 (0.927 to 1.138)	0.620	0.993 (0.940 to 1.049)	0.804	1.014 (0.967 to 1.062)	0.572		
Glycocholate glucuronide (1)	Hypertension	10	0.975 (0.890 to 1.069)	0.606	1.018 (0.969 to 1.069)	0.481	1.001 (0.959 to 1.045)	0.958		
Caffeic acid sulfate	Hypertension	17	1.073 (0.981 to 1.174)	0.143	1.022 (0.967 to 1.080)	0.444	1.015 (0.974 to 1.058)	0.470		
Ascorbic acid 2-sulfate	Hypertension	15	0.944 (0.817 to 1.091)	0.451	1.015 (0.959 to 1.073)	0.614	0.990 (0.940 to 1.043)	0.711	1.005 (0.964 to 1.048)	0.820
1-linoleoyl-2-linolenoyl-GPC (18:2/18:3)	Hypertension	13	0.959 (0.847 to 1.085)	0.518	1.012 (0.970 to 1.056)	0.571	1.000 (0.949 to 1.055)	0.986	0.991 (0.944 to 1.040)	0.723

Exposure	Outcome	nSNP	MR Egger		Weighted median		Inverse variance weighted		MR_PRESSO	
			OR (95% CI)	pval	OR (95% CI)	pval	OR (95% CI)	pval	OR (95% CI)	pval
Catechol glucuronide	Hypertension	12	1.042 (0.943 to 1.151)	0.437	0.995 (0.950 to 1.042)	0.834	0.997 (0.964 to 1.032)	0.886		
Furaneol sulfate	Hypertension	10	0.959 (0.881 to 1.044)	0.365	0.960 (0.923 to 0.998)	0.039	0.971 (0.939 to 1.004)	0.084		
Ceramide (d18:1/24:1)	Hypertension	19	1.005 (0.936 to 1.079)	0.890	0.988 (0.952 to 1.025)	0.508	1.003 (0.969 to 1.038)	0.883		
N-oleoylserine	Hypertension	12	0.978 (0.905 to 1.057)	0.594	1.000 (0.958 to 1.043)	0.992	0.996 (0.968 to 1.025)	0.789		
Perfluorooctanesulfonate (PFOS)	Hypertension	13	0.986 (0.858 to 1.133)	0.846	0.945 (0.886 to 1.009)	0.089	0.964 (0.912 to 1.019)	0.193		

Exposure	Outcome	nSNP	MR Egger		Weighted median		Inverse variance weighted		MR_PRESSO	
			OR (95% CI)	pval	OR (95% CI)	pval	OR (95% CI)	pval	OR (95% CI)	pval
Pimeloylcarnitine/3-methyladipoylcarnitine (C7-DC)	Hypertension	15	1.009 (0.936 to 1.088)	0.814	0.986 (0.944 to 1.031)	0.534	0.996 (0.962 to 1.032)	0.831		
1-palmitoleoyl-2-linolenoyl-GPC (16:1/18:3)	Hypertension	14	0.998 (0.936 to 1.063)	0.941	1.015 (0.982 to 1.050)	0.368	1.024 (0.994 to 1.054)	0.120		
Linoleoyl-arachidonoyl-glycerol (18:2/20:4) [1]	Hypertension	12	0.975 (0.904 to 1.051)	0.519	0.980 (0.958 to 1.004)	0.101	0.981 (0.936 to 1.027)	0.407	0.988 (0.964 to 1.013)	0.388
Hexadecadienoate (16:2n6)	Hypertension	7	1.009 (0.678 to 1.503)	0.965	0.938 (0.859 to 1.025)	0.155	0.933 (0.863 to 1.009)	0.084		
Glycosyl-N-behenoyl-sphingadienine (d18:2/22:0)	Hypertension	20	1.010 (0.939 to 1.087)	0.795	0.992 (0.954 to 1.032)	0.686	1.010 (0.975 to 1.046)	0.584	1.021 (0.990 to 1.053)	0.199

Exposure	Outcome	nSNP	MR Egger		Weighted median		Inverse variance weighted		MR_PRESSO	
			OR (95% CI)	pval	OR (95% CI)	pval	OR (95% CI)	pval	OR (95% CI)	pval
4-hydroxyphenylacetylglutamine	Hypertension	14	0.855 (0.715 to 1.022)	0.111	1.063 (1.000 to 1.131)	0.050	1.040 (0.968 to 1.117)	0.287	1.070 (1.032 to 1.110)	0.004
Gamma-glutamyl-alpha-lysine	Hypertension	18	0.936 (0.909 to 0.964)	4.89e-04	0.931 (0.894 to 0.969)	4.92e-04	0.942 (0.922 to 0.963)	8.71e-08		
N-palmitoyl-sphingadienine (d18:2/16:0)	Hypertension	11	0.958 (0.774 to 1.185)	0.700	1.021 (0.955 to 1.093)	0.539	1.055 (0.994 to 1.119)	0.076		
Linoleoyl-arachidonoyl-glycerol (18:2/20:4) [2]	Hypertension	23	0.939 (0.878 to 1.004)	0.080	0.989 (0.959 to 1.020)	0.479	0.974 (0.942 to 1.008)	0.131	0.980 (0.951 to 1.010)	0.211
Palmitoleoylcarnitine (C16:1)	Hypertension	12	0.858 (0.688 to 1.071)	0.206	0.928 (0.865 to 0.995)	0.035	0.954 (0.887 to 1.026)	0.207	0.951 (0.902 to 1.002)	0.093

Exposure	Outcome	nSNP	MR Egger		Weighted median		Inverse variance weighted		MR_PRESSO	
			OR (95% CI)	pval	OR (95% CI)	pval	OR (95% CI)	pval	OR (95% CI)	pval
N-stearoyl-sphingadienine (d18:2/18:0)	Hypertension	12	1.021 (0.987 to 1.056)	0.260	1.003 (0.975 to 1.031)	0.848	0.989 (0.958 to 1.021)	0.490		
2,3-dihydroxy-2-methylbutyrate	Hypertension	8	0.974 (0.833 to 1.139)	0.755	0.998 (0.924 to 1.078)	0.959	0.954 (0.902 to 1.010)	0.107		
Stearoylcholine	Hypertension	10	1.050 (0.909 to 1.213)	0.528	1.069 (1.000 to 1.143)	0.051	1.080 (1.023 to 1.139)	0.005		
Glycosyl-N-tricosanoyl-sphingadienine (d18:2/23:0)	Hypertension	11	0.971 (0.865 to 1.091)	0.635	0.976 (0.931 to 1.022)	0.297	1.014 (0.968 to 1.062)	0.555		
N-palmitoyl-heptadecasphingosine (d17:1/16:0)	Hypertension	16	0.878 (0.797 to 0.968)	0.020	0.980 (0.933 to 1.029)	0.419	1.008 (0.959 to 1.059)	0.767	1.000 (0.957 to 1.044)	0.996

Exposure	Outcome	nSNP	MR Egger		Weighted median		Inverse variance weighted		MR_PRESSO	
			OR (95% CI)	pval	OR (95% CI)	pval	OR (95% CI)	pval	OR (95% CI)	pval
Ceramide (d18:1/14:0, d16:1/16:0)	Hypertension	19	0.953 (0.849 to 1.069)	0.425	0.963 (0.922 to 1.006)	0.093	0.968 (0.924 to 1.014)	0.172	0.980 (0.943 to 1.019)	0.331
Glycosyl-N-(2-hydroxynervonoyl)-sphingosine (d18:1/24:1(2OH))	Hypertension	16	1.017 (0.913 to 1.134)	0.759	1.002 (0.962 to 1.042)	0.940	0.973 (0.924 to 1.026)	0.314	0.986 (0.945 to 1.027)	0.506
Glycosyl ceramide (d18:2/24:1, d18:1/24:2)	Hypertension	19	1.003 (0.942 to 1.068)	0.929	0.994 (0.957 to 1.033)	0.775	0.996 (0.966 to 1.027)	0.808		
Glycosyl ceramide (d18:1/23:1, d17:1/24:1)	Hypertension	11	0.968 (0.859 to 1.092)	0.611	0.997 (0.953 to 1.043)	0.900	1.006 (0.961 to 1.053)	0.804	0.990 (0.958 to 1.023)	0.566
Sphingadienine	Hypertension	10	1.007 (0.915 to 1.108)	0.889	0.994 (0.951 to 1.040)	0.800	0.994 (0.960 to 1.029)	0.734		

Exposure	Outcome	nSNP	MR Egger		Weighted median		Inverse variance weighted		MR_PRESSO	
			OR (95% CI)	pval	OR (95% CI)	pval	OR (95% CI)	pval	OR (95% CI)	pval
Ceramide (d18:1/17:0, d17:1/18:0)	Hypertension	14	1.003 (0.942 to 1.068)	0.922	1.019 (0.980 to 1.060)	0.349	1.017 (0.982 to 1.054)	0.343		
Nisinate (24:6n3)	Hypertension	10	0.954 (0.860 to 1.057)	0.393	0.988 (0.945 to 1.033)	0.598	1.003 (0.963 to 1.045)	0.874		
Sphingomyelin (d18:0/20:0, d16:0/22:0)	Hypertension	28	1.020 (0.966 to 1.077)	0.475	1.021 (0.990 to 1.053)	0.190	1.006 (0.979 to 1.033)	0.679	1.013 (0.988 to 1.038)	0.320
Sphingomyelin (d18:2/24:2)	Hypertension	14	0.946 (0.764 to 1.172)	0.623	0.981 (0.913 to 1.053)	0.590	0.944 (0.868 to 1.028)	0.184	0.967 (0.915 to 1.021)	0.254
Sphingomyelin (d17:2/16:0, d18:2/15:0)	Hypertension	16	0.934 (0.833 to 1.048)	0.266	0.942 (0.887 to 1.001)	0.055	0.994 (0.945 to 1.046)	0.825		

Exposure	Outcome	nSNP	MR Egger		Weighted median		Inverse variance weighted		MR_PRESSO	
			OR (95% CI)	pval	OR (95% CI)	pval	OR (95% CI)	pval	OR (95% CI)	pval
Sphingomyelin (d18:2/23:1)	Hypertension	15	0.961 (0.852 to 1.084)	0.530	0.983 (0.934 to 1.035)	0.513	0.989 (0.935 to 1.047)	0.710	0.987 (0.948 to 1.028)	0.541
Behenoylcarnitine (C22)	Hypertension	19	1.065 (0.964 to 1.178)	0.233	1.025 (0.980 to 1.073)	0.284	1.027 (0.986 to 1.069)	0.197		
Sphingomyelin (d18:2/18:1)	Hypertension	10	0.941 (0.822 to 1.076)	0.399	0.965 (0.903 to 1.032)	0.300	0.988 (0.935 to 1.044)	0.669		
Sphingomyelin (d18:2/21:0, d16:2/23:0)	Hypertension	19	0.953 (0.829 to 1.095)	0.504	0.986 (0.931 to 1.045)	0.636	0.968 (0.913 to 1.026)	0.267	0.981 (0.929 to 1.037)	0.508
Linoleoylcholine	Hypertension	15	1.027 (0.931 to 1.133)	0.606	0.979 (0.928 to 1.033)	0.442	1.007 (0.960 to 1.057)	0.773		

Exposure	Outcome	nSNP	MR Egger		Weighted median		Inverse variance weighted		MR_PRESSO	
			OR (95% CI)	pval	OR (95% CI)	pval	OR (95% CI)	pval	OR (95% CI)	pval
Sphingomyelin (d18:1/19:0, d19:1/18:0)	Hypertension	16	1.006 (0.904 to 1.119)	0.916	1.026 (0.983 to 1.071)	0.237	1.026 (0.983 to 1.072)	0.242	1.042 (1.004 to 1.081)	0.049
Linolenoylcarnitine (C18:3)	Hypertension	18	1.043 (0.931 to 1.167)	0.481	1.033 (0.978 to 1.091)	0.248	1.034 (0.988 to 1.081)	0.147	1.050 (1.008 to 1.093)	0.031
Heneicosapentaenoate (21:5n3)	Hypertension	9	1.040 (0.936 to 1.155)	0.494	1.013 (0.958 to 1.072)	0.643	0.992 (0.948 to 1.037)	0.712		
Sphingomyelin (d18:1/25:0, d19:0/24:1, d20:1/23:0, d19:1/24:0)	Hypertension	21	1.012 (0.980 to 1.045)	0.476	1.004 (0.980 to 1.028)	0.754	1.005 (0.985 to 1.025)	0.658		
Arachidoylcarnitine (C20)	Hypertension	17	1.014 (0.932 to 1.103)	0.749	1.039 (0.996 to 1.085)	0.078	1.029 (0.994 to 1.065)	0.107		

Exposure	Outcome	nSNP	MR Egger		Weighted median		Inverse variance weighted		MR_PRESSO	
			OR (95% CI)	pval	OR (95% CI)	pval	OR (95% CI)	pval	OR (95% CI)	pval
Cerotoylcarnitine (C26)	Hypertension	21	1.062 (0.987 to 1.142)	0.123	1.027 (0.993 to 1.061)	0.119	1.036 (0.996 to 1.077)	0.078	1.046 (1.016 to 1.077)	0.007
Lignoceroylcarnitine (C24)	Hypertension	14	1.031 (0.951 to 1.117)	0.475	1.016 (0.982 to 1.052)	0.354	1.032 (0.997 to 1.069)	0.074	1.041 (1.010 to 1.073)	0.022
Docosatrienoate (22:3n6)	Hypertension	11	1.021 (0.920 to 1.132)	0.709	1.004 (0.959 to 1.051)	0.862	1.023 (0.984 to 1.062)	0.255		
Dihomo-linolenoylcarnitine (C20:3n3 or 6)	Hypertension	23	1.042 (1.000 to 1.085)	0.065	1.034 (1.008 to 1.062)	0.012	1.042 (1.020 to 1.064)	1.25e-04		
Carotene diol (2)	Hypertension	16	1.048 (0.965 to 1.139)	0.282	1.024 (0.981 to 1.069)	0.280	1.011 (0.981 to 1.042)	0.470		

Exposure	Outcome	nSNP	MR Egger		Weighted median		Inverse variance weighted		MR_PRESSO	
			OR (95% CI)	pval	OR (95% CI)	pval	OR (95% CI)	pval	OR (95% CI)	pval
Ethyl alpha-glucopyranoside	Hypertension	16	1.027 (0.936 to 1.127)	0.583	1.021 (0.973 to 1.071)	0.397	1.015 (0.977 to 1.054)	0.441		
Carotene diol (1)	Hypertension	20	1.012 (0.938 to 1.092)	0.759	1.023 (0.981 to 1.066)	0.288	0.998 (0.966 to 1.031)	0.890		
Nervonoylcarnitine (C24:1)	Hypertension	15	1.105 (1.027 to 1.190)	0.020	1.023 (0.984 to 1.064)	0.254	1.027 (0.993 to 1.062)	0.117		
Dihomo-linoleoylcarnitine (C20:2)	Hypertension	18	1.041 (0.968 to 1.119)	0.292	1.037 (0.997 to 1.078)	0.069	1.020 (0.986 to 1.055)	0.262	1.017 (0.987 to 1.049)	0.289
Glycosyl ceramide (d18:1/20:0, d16:1/22:0)	Hypertension	21	0.978 (0.917 to 1.043)	0.510	0.983 (0.950 to 1.017)	0.325	0.966 (0.940 to 0.992)	0.012		

Exposure	Outcome	nSNP	MR Egger		Weighted median		Inverse variance weighted		MR_PRESSO	
			OR (95% CI)	pval	OR (95% CI)	pval	OR (95% CI)	pval	OR (95% CI)	pval
Docosahexaenoylcarnitine (C22:6)	Hypertension	21	0.957 (0.890 to 1.029)	0.249	0.980 (0.942 to 1.019)	0.309	0.999 (0.966 to 1.033)	0.951		
N,N,N-trimethyl-5-aminovalerate	Hypertension	20	0.975 (0.834 to 1.139)	0.750	0.997 (0.948 to 1.049)	0.914	1.019 (0.962 to 1.079)	0.519	0.985 (0.945 to 1.027)	0.494
Eicosenoylcarnitine (C20:1)	Hypertension	22	1.014 (0.913 to 1.127)	0.794	1.027 (0.980 to 1.076)	0.262	1.031 (0.992 to 1.071)	0.117		
Ximenoylcarnitine (C26:1)	Hypertension	19	1.043 (0.975 to 1.115)	0.237	1.025 (0.989 to 1.063)	0.177	1.039 (1.005 to 1.074)	0.026		
Arachidonoylcarnitine (C20:4)	Hypertension	24	1.024 (0.982 to 1.068)	0.275	1.032 (1.004 to 1.060)	0.024	1.037 (1.014 to 1.062)	0.002	1.034 (1.013 to 1.056)	0.004

Exposure	Outcome	nSNP	MR Egger		Weighted median		Inverse variance weighted		MR_PRESSO	
			OR (95% CI)	pval	OR (95% CI)	pval	OR (95% CI)	pval	OR (95% CI)	pval
5-dodecenoylcarnitine (C12:1)	Hypertension	16	0.880 (0.773 to 1.002)	0.073	0.945 (0.888 to 1.007)	0.079	0.930 (0.882 to 0.981)	0.008	0.949 (0.907 to 0.993)	0.041
2-butenoylglycine	Hypertension	17	0.961 (0.889 to 1.039)	0.335	0.924 (0.888 to 0.961)	6.87e-05	0.930 (0.902 to 0.958)	2.22e-06		
(N(1) + N(8))-acetylspermidine	Hypertension	22	1.002 (0.945 to 1.062)	0.941	0.984 (0.951 to 1.018)	0.351	0.969 (0.939 to 1.000)	0.050	0.975 (0.948 to 1.002)	0.083
Hydroxy-cmpf	Hypertension	11	1.006 (0.861 to 1.175)	0.940	1.009 (0.945 to 1.077)	0.792	1.022 (0.969 to 1.077)	0.425		
2-furoylcarnitine	Hypertension	9	1.033 (0.969 to 1.100)	0.354	1.015 (0.965 to 1.069)	0.559	1.005 (0.967 to 1.043)	0.812		

Exposure	Outcome	nSNP	MR Egger		Weighted median		Inverse variance weighted		MR_PRESSO	
			OR (95% CI)	pval	OR (95% CI)	pval	OR (95% CI)	pval	OR (95% CI)	pval
Cortolone glucuronide (1)	Hypertension	19	0.972 (0.900 to 1.050)	0.480	1.010 (0.963 to 1.059)	0.684	1.025 (0.985 to 1.067)	0.223		
Carotene diol (3)	Hypertension	16	1.083 (1.005 to 1.168)	0.055	1.033 (0.991 to 1.078)	0.123	1.018 (0.976 to 1.060)	0.408		
3-hydroxyoleoylcarnitine	Hypertension	16	1.035 (0.840 to 1.276)	0.748	0.945 (0.891 to 1.003)	0.062	0.961 (0.899 to 1.026)	0.229	0.981 (0.930 to 1.034)	0.484
Dodecenedioate (C12:1-DC)	Hypertension	15	0.940 (0.865 to 1.021)	0.167	0.964 (0.917 to 1.012)	0.140	0.965 (0.932 to 0.999)	0.045		
Trans-2-hexenoylglycine	Hypertension	14	0.956 (0.868 to 1.052)	0.374	0.938 (0.898 to 0.980)	0.004	0.947 (0.911 to 0.984)	0.005		

Exposure	Outcome	nSNP	MR Egger		Weighted median		Inverse variance weighted		MR_PRESSO	
			OR (95% CI)	pval	OR (95% CI)	pval	OR (95% CI)	pval	OR (95% CI)	pval
2-hydroxyarachidate	Hypertension	7	0.999 (0.967 to 1.033)	0.973	1.009 (0.993 to 1.026)	0.262	1.011 (0.980 to 1.043)	0.491		
3-hydroxyphenylacetoylglutamine	Hypertension	5	1.009 (0.864 to 1.177)	0.918	0.980 (0.938 to 1.025)	0.382	0.992 (0.958 to 1.026)	0.632		
Heptenedioate (C7:1-DC)	Hypertension	8	0.966 (0.853 to 1.093)	0.599	1.023 (0.953 to 1.099)	0.524	1.021 (0.970 to 1.075)	0.416		
Hexadecenedioate (C16:1-DC)	Hypertension	19	0.986 (0.962 to 1.009)	0.251	0.978 (0.960 to 0.997)	0.022	0.981 (0.965 to 0.997)	0.019		
Octadecenedioate (C18:1-DC)	Hypertension	14	0.958 (0.919 to 0.999)	0.065	0.969 (0.944 to 0.994)	0.016	0.983 (0.960 to 1.007)	0.163		

Exposure	Outcome	nSNP	MR Egger		Weighted median		Inverse variance weighted		MR_PRESSO	
			OR (95% CI)	pval	OR (95% CI)	pval	OR (95% CI)	pval	OR (95% CI)	pval
Dihydroferulic acid sulfate	Hypertension	19	1.000 (0.973 to 1.028)	0.988	0.996 (0.975 to 1.018)	0.747	1.004 (0.985 to 1.023)	0.667		
Glutamine conjugate of C7H12O2	Hypertension	13	0.955 (0.850 to 1.071)	0.446	0.997 (0.940 to 1.058)	0.928	1.006 (0.962 to 1.052)	0.786		
Hydroxyasparagine	Hypertension	13	1.036 (0.703 to 1.528)	0.860	1.015 (0.932 to 1.104)	0.738	1.045 (0.929 to 1.174)	0.465	1.031 (0.953 to 1.114)	0.467
Sphingomyelin (d17:1/14:0, d16:1/15:0)	Hypertension	17	0.952 (0.840 to 1.079)	0.456	0.972 (0.916 to 1.032)	0.355	0.983 (0.933 to 1.037)	0.531		
Octadecadienedioate (C18:2-DC)	Hypertension	16	0.969 (0.942 to 0.997)	0.051	0.975 (0.952 to 0.997)	0.027	0.982 (0.964 to 1.000)	0.053		

Exposure	Outcome	nSNP	MR Egger		Weighted median		Inverse variance weighted		MR_PRESSO	
			OR (95% CI)	pval	OR (95% CI)	pval	OR (95% CI)	pval	OR (95% CI)	pval
Perfluorooctanoate (PFOA)	Hypertension	11	1.101 (0.893 to 1.359)	0.391	0.952 (0.880 to 1.030)	0.219	0.999 (0.929 to 1.074)	0.971		
3-carboxy-4-methyl-5-pentyl-2-furanpropionate (3-CMPFP)	Hypertension	15	0.970 (0.918 to 1.026)	0.312	0.984 (0.942 to 1.029)	0.486	1.013 (0.981 to 1.047)	0.432		
Glutamine conjugate of C6H10O2 (2)	Hypertension	7	1.091 (0.862 to 1.381)	0.500	1.029 (0.944 to 1.121)	0.518	1.061 (0.966 to 1.165)	0.213		
3-hydroxybutyroylglycine	Hypertension	17	0.945 (0.886 to 1.007)	0.103	0.940 (0.908 to 0.973)	4.78e-04	0.970 (0.934 to 1.008)	0.118	0.982 (0.945 to 1.020)	0.355
Glycine conjugate of C10H14O2 (1)	Hypertension	11	0.995 (0.883 to 1.120)	0.930	0.976 (0.930 to 1.024)	0.317	1.012 (0.955 to 1.072)	0.694	0.996 (0.953 to 1.041)	0.863

Exposure	Outcome	nSNP	MR Egger		Weighted median		Inverse variance weighted		MR_PRESSO	
			OR (95% CI)	pval	OR (95% CI)	pval	OR (95% CI)	pval	OR (95% CI)	pval
Glutamine conjugate of C6H10O2 (1)	Hypertension	8	0.949 (0.681 to 1.324)	0.770	1.080 (0.988 to 1.181)	0.089	1.110 (1.004 to 1.228)	0.042	1.062 (0.986 to 1.143)	0.162
N-methylhydroxyproline	Hypertension	8	0.901 (0.781 to 1.040)	0.206	1.012 (0.939 to 1.090)	0.764	0.999 (0.945 to 1.057)	0.980		
Glucuronide of C10H18O2 (7)	Hypertension	12	1.019 (0.950 to 1.092)	0.616	0.991 (0.943 to 1.040)	0.704	0.981 (0.948 to 1.016)	0.289		
Glyco-beta-muricholate	Hypertension	15	1.026 (0.996 to 1.056)	0.115	1.022 (1.001 to 1.043)	0.038	1.020 (1.000 to 1.042)	0.055		
Glucuronide of C12H22O4 (1)	Hypertension	8	1.008 (0.881 to 1.153)	0.912	0.959 (0.905 to 1.016)	0.157	0.966 (0.924 to 1.011)	0.133		

Exposure	Outcome	nSNP	MR Egger		Weighted median		Inverse variance weighted		MR_PRESSO	
			OR (95% CI)	pval	OR (95% CI)	pval	OR (95% CI)	pval	OR (95% CI)	pval
N,n,n-trimethyl-alanylproline betaine (tmap)	Hypertension	13	0.948 (0.748 to 1.201)	0.665	0.991 (0.927 to 1.059)	0.789	1.018 (0.938 to 1.106)	0.666	0.974 (0.934 to 1.016)	0.248
Glycine conjugate of C10H12O2	Hypertension	13	1.073 (0.833 to 1.383)	0.595	0.982 (0.929 to 1.037)	0.509	1.000 (0.924 to 1.081)	0.995	0.985 (0.942 to 1.030)	0.516
N-acetyl-2-aminooctanoate	Hypertension	15	1.030 (0.964 to 1.100)	0.399	1.037 (1.012 to 1.063)	0.003	1.050 (1.007 to 1.094)	0.023	1.040 (1.016 to 1.064)	0.006
3-formylindole	Hypertension	17	1.005 (0.957 to 1.056)	0.843	0.998 (0.971 to 1.027)	0.909	0.987 (0.959 to 1.015)	0.367		
Gamma-glutamylcitrulline	Hypertension	19	0.965 (0.828 to 1.126)	0.660	0.972 (0.922 to 1.024)	0.283	0.962 (0.920 to 1.006)	0.091	0.958 (0.926 to 0.990)	0.023

Exposure	Outcome	nSNP	MR Egger		Weighted median		Inverse variance weighted		MR_PRESSO	
			OR (95% CI)	pval	OR (95% CI)	pval	OR (95% CI)	pval	OR (95% CI)	pval
8-methoxykynurenate	Hypertension	12	0.819 (0.714 to 0.940)	0.017	1.013 (0.952 to 1.077)	0.691	0.999 (0.945 to 1.056)	0.975	1.021 (0.973 to 1.071)	0.413
Tetradecadienoate (14:2)	Hypertension	12	1.037 (0.925 to 1.163)	0.548	0.969 (0.915 to 1.026)	0.285	0.967 (0.927 to 1.009)	0.125		
3-amino-2-piperidone	Hypertension	11	0.953 (0.878 to 1.035)	0.280	0.994 (0.937 to 1.054)	0.838	0.995 (0.950 to 1.042)	0.832		
Glucuronide of piperine metabolite C17H21NO3 (3)	Hypertension	13	1.006 (0.987 to 1.025)	0.566	1.003 (0.985 to 1.021)	0.747	1.001 (0.986 to 1.017)	0.854		
Dodecadienoate (12:2)	Hypertension	15	0.991 (0.969 to 1.013)	0.415	1.000 (0.987 to 1.013)	0.984	1.006 (0.985 to 1.027)	0.601		

Exposure	Outcome	nSNP	MR Egger		Weighted median		Inverse variance weighted		MR_PRESSO	
			OR (95% CI)	pval	OR (95% CI)	pval	OR (95% CI)	pval	OR (95% CI)	pval
N-lactoyl tyrosine	Hypertension	12	1.015 (0.935 to 1.101)	0.732	0.985 (0.935 to 1.038)	0.574	0.995 (0.954 to 1.038)	0.820		
N,N-dimethylalanine	Hypertension	17	0.961 (0.888 to 1.040)	0.342	0.999 (0.949 to 1.051)	0.958	1.004 (0.963 to 1.046)	0.858	1.012 (0.976 to 1.050)	0.516
Ethyl beta-glucopyranoside	Hypertension	12	0.999 (0.961 to 1.039)	0.976	0.991 (0.963 to 1.019)	0.528	0.995 (0.969 to 1.022)	0.719		
N-lactoyl phenylalanine	Hypertension	8	1.112 (0.865 to 1.431)	0.438	1.028 (0.943 to 1.121)	0.532	1.007 (0.914 to 1.110)	0.884	1.044 (0.959 to 1.135)	0.359
N-acetyl-isoputreanine	Hypertension	21	1.048 (0.988 to 1.112)	0.133	1.035 (1.004 to 1.066)	0.024	1.024 (0.995 to 1.054)	0.104		

Exposure	Outcome	nSNP	MR Egger		Weighted median		Inverse variance weighted		MR_PRESSO	
			OR (95% CI)	pval	OR (95% CI)	pval	OR (95% CI)	pval	OR (95% CI)	pval
2,2'-Methylenebis(6-tert-butyl-p-cresol)	Hypertension	11	0.983 (0.915 to 1.057)	0.658	1.005 (0.959 to 1.054)	0.827	0.993 (0.960 to 1.027)	0.673		
N-lactoyl isoleucine	Hypertension	8	0.970 (0.834 to 1.128)	0.707	1.044 (0.973 to 1.120)	0.228	1.052 (0.996 to 1.111)	0.067		
2-hydroxysebacate	Hypertension	11	0.982 (0.896 to 1.076)	0.702	0.958 (0.912 to 1.007)	0.095	0.992 (0.951 to 1.036)	0.724		
Ascorbic acid 3-sulfate	Hypertension	14	0.984 (0.952 to 1.018)	0.372	0.984 (0.957 to 1.011)	0.244	0.989 (0.966 to 1.014)	0.387		
N-lactoyl valine	Hypertension	9	1.186 (0.958 to 1.469)	0.161	0.939 (0.869 to 1.015)	0.115	0.941 (0.883 to 1.004)	0.066		

Exposure	Outcome	nSNP	MR Egger		Weighted median		Inverse variance weighted		MR_PRESSO	
			OR (95% CI)	pval	OR (95% CI)	pval	OR (95% CI)	pval	OR (95% CI)	pval
6-bromotryptophan	Hypertension	16	0.980 (0.940 to 1.023)	0.379	0.990 (0.959 to 1.023)	0.565	0.993 (0.965 to 1.021)	0.606		
Delta-CEHC	Hypertension	10	0.913 (0.759 to 1.099)	0.364	0.943 (0.888 to 1.002)	0.057	0.990 (0.928 to 1.056)	0.762	0.970 (0.922 to 1.020)	0.265
Indoleacetoylcarnitine	Hypertension	27	1.017 (0.976 to 1.060)	0.422	1.014 (0.985 to 1.043)	0.353	1.004 (0.980 to 1.029)	0.738	1.012 (0.991 to 1.034)	0.273
N-acetyl leucine	Hypertension	10	1.178 (0.970 to 1.430)	0.136	1.072 (0.993 to 1.156)	0.075	1.063 (1.002 to 1.128)	0.043		
3-indoleglyoxylic acid	Hypertension	12	0.994 (0.931 to 1.061)	0.864	1.003 (0.973 to 1.035)	0.828	1.014 (0.980 to 1.049)	0.417		

Exposure	Outcome	nSNP	MR Egger		Weighted median		Inverse variance weighted		MR_PRESSO	
			OR (95% CI)	pval	OR (95% CI)	pval	OR (95% CI)	pval	OR (95% CI)	pval
Sulfate of piperine metabolite C18H21NO3 (1)	Hypertension	13	1.116 (0.816 to 1.527)	0.506	0.967 (0.907 to 1.031)	0.309	0.893 (0.795 to 1.004)	0.059	0.955 (0.908 to 1.005)	0.104
Methyl vanillate sulfate	Hypertension	10	0.983 (0.880 to 1.097)	0.764	0.979 (0.932 to 1.029)	0.409	0.986 (0.945 to 1.027)	0.495		
Glucuronide of piperine metabolite C17H21NO3 (4)	Hypertension	11	0.945 (0.841 to 1.061)	0.362	0.989 (0.939 to 1.041)	0.673	0.990 (0.948 to 1.033)	0.636		
Sulfate of piperine metabolite C16H19NO3 (3)	Hypertension	17	0.995 (0.905 to 1.095)	0.927	1.016 (0.967 to 1.066)	0.530	1.030 (0.988 to 1.073)	0.166		
Sulfate of piperine metabolite C16H19NO3 (2)	Hypertension	16	0.909 (0.731 to 1.131)	0.407	0.973 (0.914 to 1.037)	0.405	1.026 (0.959 to 1.098)	0.450	1.002 (0.946 to 1.061)	0.955

Exposure	Outcome	nSNP	MR Egger		Weighted median		Inverse variance weighted		MR_PRESSO	
			OR (95% CI)	pval	OR (95% CI)	pval	OR (95% CI)	pval	OR (95% CI)	pval
3-hydroxypyridine glucuronide	Hypertension	12	1.042 (0.939 to 1.156)	0.461	1.019 (0.967 to 1.073)	0.478	1.009 (0.972 to 1.047)	0.652		
Glucuronide of piperine metabolite C17H21NO3 (5)	Hypertension	11	0.977 (0.841 to 1.135)	0.772	0.950 (0.901 to 1.002)	0.057	0.976 (0.924 to 1.031)	0.390	0.963 (0.918 to 1.010)	0.155
2-naphthol sulfate	Hypertension	12	0.986 (0.920 to 1.057)	0.704	0.984 (0.936 to 1.035)	0.533	0.998 (0.962 to 1.036)	0.919		
11beta-hydroxyandrosterone glucuronide	Hypertension	22	1.005 (0.934 to 1.082)	0.896	1.025 (0.983 to 1.069)	0.250	1.031 (0.993 to 1.071)	0.111	1.020 (0.987 to 1.053)	0.252
4-ethylcatechol sulfate	Hypertension	11	1.014 (0.990 to 1.039)	0.289	1.004 (0.981 to 1.028)	0.720	0.998 (0.979 to 1.018)	0.868		

Exposure	Outcome	nSNP	MR Egger		Weighted median		Inverse variance weighted		MR_PRESSO	
			OR (95% CI)	pval	OR (95% CI)	pval	OR (95% CI)	pval	OR (95% CI)	pval
Sulfate of piperine metabolite C18H21NO3 (3)	Hypertension	13	0.998 (0.839 to 1.187)	0.982	0.992 (0.933 to 1.054)	0.786	1.001 (0.949 to 1.057)	0.957		
(2,4 or 2,5)-dimethylphenol sulfate	Hypertension	10	1.041 (0.919 to 1.181)	0.544	1.025 (0.978 to 1.074)	0.301	1.029 (0.993 to 1.065)	0.117		
11beta-hydroxyetiocholanolone glucuronide	Hypertension	13	1.009 (0.867 to 1.173)	0.912	0.974 (0.914 to 1.039)	0.426	0.982 (0.925 to 1.041)	0.537		
Cholic acid glucuronide	Hypertension	11	0.982 (0.885 to 1.089)	0.734	1.002 (0.970 to 1.034)	0.923	0.989 (0.946 to 1.035)	0.641	1.007 (0.977 to 1.039)	0.658
4-allylcatechol sulfate	Hypertension	11	1.007 (0.918 to 1.105)	0.888	1.059 (1.002 to 1.120)	0.042	1.047 (1.000 to 1.095)	0.048		

Exposure	Outcome	nSNP	MR Egger		Weighted median		Inverse variance weighted		MR_PRESSO	
			OR (95% CI)	pval	OR (95% CI)	pval	OR (95% CI)	pval	OR (95% CI)	pval
Glycoursodeoxycholic acid sulfate (1)	Hypertension	14	0.969 (0.868 to 1.081)	0.581	0.939 (0.896 to 0.985)	0.009	0.967 (0.933 to 1.002)	0.065		
Dihydrocaffeate sulfate (2)	Hypertension	12	0.959 (0.854 to 1.077)	0.492	0.952 (0.904 to 1.003)	0.063	0.964 (0.909 to 1.023)	0.231	0.961 (0.922 to 1.003)	0.100
4-methylhexanoylglutamine	Hypertension	6	0.984 (0.905 to 1.070)	0.725	1.031 (0.982 to 1.082)	0.214	1.030 (0.993 to 1.069)	0.115		
2-ketocaprylate	Hypertension	19	0.899 (0.827 to 0.977)	0.023	0.968 (0.925 to 1.012)	0.152	1.000 (0.961 to 1.042)	0.988	1.021 (0.989 to 1.055)	0.224
3-hydroxy-2-methylpyridine sulfate	Hypertension	13	1.081 (0.991 to 1.179)	0.106	0.988 (0.930 to 1.050)	0.703	0.991 (0.949 to 1.035)	0.685		

Exposure	Outcome	nSNP	MR Egger		Weighted median		Inverse variance weighted		MR_PRESSO	
			OR (95% CI)	pval	OR (95% CI)	pval	OR (95% CI)	pval	OR (95% CI)	pval
5-hydroxy-2-methylpyridine sulfate	Hypertension	15	1.038 (0.946 to 1.138)	0.448	0.987 (0.940 to 1.037)	0.613	0.997 (0.961 to 1.034)	0.866		
3-ethylcatechol sulfate (2)	Hypertension	10	1.063 (0.851 to 1.327)	0.604	1.028 (0.967 to 1.094)	0.376	1.015 (0.950 to 1.085)	0.657	1.038 (0.983 to 1.096)	0.216
3-hydroxyhexanoylcarnitine (1)	Hypertension	12	0.902 (0.773 to 1.054)	0.223	1.002 (0.943 to 1.064)	0.955	1.026 (0.974 to 1.081)	0.326		
2,6-dihydroxybenzoic acid	Hypertension	13	1.082 (0.903 to 1.296)	0.413	1.031 (0.977 to 1.089)	0.263	1.054 (0.994 to 1.117)	0.076	1.027 (0.988 to 1.068)	0.206
3-ethylcatechol sulfate (1)	Hypertension	11	1.126 (0.999 to 1.269)	0.084	1.003 (0.947 to 1.062)	0.924	0.990 (0.939 to 1.044)	0.715		

Exposure	Outcome	nSNP	MR Egger		Weighted median		Inverse variance weighted		MR_PRESSO	
			OR (95% CI)	pval	OR (95% CI)	pval	OR (95% CI)	pval	OR (95% CI)	pval
Lithocholate sulfate (1)	Hypertension	19	0.918 (0.854 to 0.987)	0.033	0.976 (0.936 to 1.018)	0.260	0.972 (0.942 to 1.004)	0.086		
4-acetylcatechol sulfate (1)	Hypertension	6	0.994 (0.950 to 1.040)	0.810	0.996 (0.975 to 1.017)	0.692	0.995 (0.967 to 1.023)	0.705		
2,3-dihydroxy-5-methylthio-4-pentenoate (dmtpa)	Hypertension	19	1.019 (0.837 to 1.241)	0.854	1.028 (0.967 to 1.092)	0.376	1.019 (0.941 to 1.104)	0.639	1.015 (0.964 to 1.068)	0.592
Picolinoylglycine	Hypertension	13	0.982 (0.890 to 1.083)	0.723	0.979 (0.935 to 1.026)	0.376	0.952 (0.905 to 1.001)	0.057	0.978 (0.953 to 1.005)	0.137
Succinoyltaurine	Hypertension	17	0.987 (0.975 to 0.999)	0.046	0.990 (0.977 to 1.002)	0.100	0.991 (0.981 to 1.001)	0.063		

Exposure	Outcome	nSNP	MR Egger		Weighted median		Inverse variance weighted		MR_PRESSO	
			OR (95% CI)	pval	OR (95% CI)	pval	OR (95% CI)	pval	OR (95% CI)	pval
Undecenoylcarnitine (C11:1)	Hypertension	17	1.013 (0.950 to 1.081)	0.700	1.031 (0.994 to 1.068)	0.098	1.020 (0.991 to 1.050)	0.185		
Palmitoyl-sphingosine-phosphoethanolamine (d18:1/16:0)	Hypertension	15	1.073 (0.979 to 1.175)	0.156	0.982 (0.932 to 1.036)	0.510	0.976 (0.928 to 1.027)	0.349		
Pregnenetriol disulfate	Hypertension	18	0.999 (0.981 to 1.018)	0.940	1.001 (0.983 to 1.020)	0.917	0.993 (0.978 to 1.009)	0.399		
Tetradecadienedioate (C14:2-DC)	Hypertension	22	0.975 (0.926 to 1.025)	0.331	0.976 (0.946 to 1.008)	0.137	0.976 (0.949 to 1.003)	0.080		
Hydroxy-N6,N6,N6-trimethyllysine	Hypertension	15	1.094 (0.941 to 1.271)	0.265	0.991 (0.935 to 1.049)	0.748	0.967 (0.902 to 1.036)	0.344	0.987 (0.942 to 1.034)	0.595

Exposure	Outcome	nSNP	MR Egger		Weighted median		Inverse variance weighted		MR_PRESSO	
			OR (95% CI)	pval	OR (95% CI)	pval	OR (95% CI)	pval	OR (95% CI)	pval
Pregnenetriol sulfate	Hypertension	25	0.921 (0.821 to 1.033)	0.175	0.976 (0.935 to 1.019)	0.276	1.015 (0.958 to 1.074)	0.618	0.983 (0.951 to 1.016)	0.319
Taurochenodeoxycholic acid 3-sulfate	Hypertension	20	0.958 (0.881 to 1.043)	0.338	0.995 (0.957 to 1.034)	0.799	1.013 (0.965 to 1.063)	0.602	0.994 (0.967 to 1.022)	0.689
Hydroxypalmitoyl sphingomyelin (d18:1/16:0(OH))	Hypertension	20	1.035 (0.924 to 1.159)	0.560	1.011 (0.975 to 1.048)	0.560	0.995 (0.942 to 1.051)	0.863	1.022 (0.985 to 1.060)	0.260
5-hydroxymethyl-2-furoylcarnitine	Hypertension	11	1.026 (0.828 to 1.271)	0.820	1.013 (0.959 to 1.070)	0.637	1.024 (0.965 to 1.086)	0.437	1.009 (0.956 to 1.065)	0.745
Eicosenedioate (C20:1-DC)	Hypertension	14	0.949 (0.908 to 0.991)	0.037	0.970 (0.940 to 1.000)	0.050	0.982 (0.957 to 1.008)	0.169		

Exposure	Outcome	nSNP	MR Egger		Weighted median		Inverse variance weighted		MR_PRESSO	
			OR (95% CI)	pval	OR (95% CI)	pval	OR (95% CI)	pval	OR (95% CI)	pval
3-hydroxydecanoylcarnitine	Hypertension	19	1.041 (0.973 to 1.113)	0.258	1.023 (0.974 to 1.075)	0.355	0.998 (0.956 to 1.042)	0.928	1.008 (0.968 to 1.050)	0.698
Levulinoylcarnitine	Hypertension	13	1.052 (0.947 to 1.169)	0.363	0.995 (0.944 to 1.048)	0.838	0.995 (0.954 to 1.038)	0.823		
Cis 3,4-methyleneheptanoate	Hypertension	18	1.010 (0.896 to 1.140)	0.869	1.022 (0.967 to 1.079)	0.440	1.038 (0.985 to 1.094)	0.164	1.061 (1.025 to 1.097)	0.004
Branched-chain, straight-chain, or cyclopropyl 12:1 fatty acid	Hypertension	16	0.958 (0.845 to 1.086)	0.513	0.962 (0.911 to 1.016)	0.163	0.988 (0.925 to 1.054)	0.711	0.985 (0.948 to 1.024)	0.452
Deoxycholic acid 12-sulfate	Hypertension	13	0.966 (0.909 to 1.027)	0.293	0.957 (0.929 to 0.986)	0.004	0.944 (0.914 to 0.975)	4.85e-04		

Exposure	Outcome	nSNP	MR Egger		Weighted median		Inverse variance weighted		MR_PRESSO	
			OR (95% CI)	pval	OR (95% CI)	pval	OR (95% CI)	pval	OR (95% CI)	pval
Branched-chain, straight-chain, or cyclopropyl 10:1 fatty acid (1)	Hypertension	9	1.031 (0.994 to 1.070)	0.146	1.016 (0.989 to 1.044)	0.237	1.019 (0.990 to 1.049)	0.203		
3,5-dichloro-2,6-dihydroxybenzoic acid	Hypertension	11	0.905 (0.846 to 0.969)	0.018	0.986 (0.936 to 1.038)	0.588	1.005 (0.951 to 1.062)	0.859	1.018 (0.971 to 1.067)	0.481
Vanillic acid glycine	Hypertension	15	1.032 (0.978 to 1.089)	0.275	1.008 (0.975 to 1.043)	0.620	1.000 (0.962 to 1.040)	0.998	1.000 (0.970 to 1.031)	0.997
(S)-a-amino-omega-caprolactam	Hypertension	8	0.985 (0.787 to 1.232)	0.896	1.093 (1.016 to 1.174)	0.016	1.125 (1.030 to 1.230)	0.009	1.077 (1.035 to 1.120)	0.011
3-bromo-5-chloro-2,6-dihydroxybenzoic acid	Hypertension	11	0.973 (0.881 to 1.075)	0.604	0.955 (0.909 to 1.003)	0.064	0.975 (0.928 to 1.025)	0.320		

Exposure	Outcome	nSNP	MR Egger		Weighted median		Inverse variance weighted		MR_PRESSO	
			OR (95% CI)	pval	OR (95% CI)	pval	OR (95% CI)	pval	OR (95% CI)	pval
Dibutyl sulfosuccinate	Hypertension	23	0.994 (0.979 to 1.009)	0.448	0.991 (0.977 to 1.005)	0.205	0.995 (0.982 to 1.008)	0.445		
Metabolonic lactone sulfate	Hypertension	18	1.005 (0.966 to 1.046)	0.800	1.001 (0.975 to 1.027)	0.965	0.998 (0.975 to 1.022)	0.873	0.977 (0.958 to 0.996)	0.031
2-hydroxy-4-(methylthio)butanoic acid	Hypertension	18	1.044 (1.003 to 1.086)	0.050	1.031 (1.002 to 1.060)	0.034	1.020 (0.993 to 1.047)	0.145	0.991 (0.960 to 1.023)	0.592
Pentose acid	Hypertension	14	1.025 (0.918 to 1.144)	0.672	1.020 (0.969 to 1.074)	0.454	1.042 (0.997 to 1.090)	0.067		
Branched chain 14:0 dicarboxylic acid	Hypertension	9	0.858 (0.721 to 1.021)	0.128	0.952 (0.880 to 1.031)	0.226	0.946 (0.884 to 1.013)	0.113		

Exposure	Outcome	nSNP	MR Egger		Weighted median		Inverse variance weighted		MR_PRESSO	
			OR (95% CI)	pval	OR (95% CI)	pval	OR (95% CI)	pval	OR (95% CI)	pval
S-carboxyethylcysteine	Hypertension	14	0.969 (0.871 to 1.077)	0.567	0.986 (0.932 to 1.044)	0.634	0.974 (0.930 to 1.020)	0.258		
N-succinyl-phenylalanine	Hypertension	11	0.982 (0.934 to 1.033)	0.505	0.977 (0.950 to 1.004)	0.090	0.977 (0.955 to 1.000)	0.054		
(2 or 3)-decenoate (10:1n7 or n8)	Hypertension	18	1.035 (0.925 to 1.158)	0.556	1.043 (0.990 to 1.098)	0.111	1.053 (1.002 to 1.106)	0.042	1.037 (0.992 to 1.084)	0.125
Bilirubin degradation product, C17H20N2O5 (2)	Hypertension	13	1.017 (0.972 to 1.064)	0.479	1.009 (0.982 to 1.038)	0.517	1.015 (0.990 to 1.041)	0.230		
Bilirubin degradation product, C16H18N2O5 (4)	Hypertension	10	0.990 (0.959 to 1.023)	0.576	0.997 (0.975 to 1.019)	0.776	1.002 (0.980 to 1.024)	0.869		

Exposure	Outcome	nSNP	MR Egger		Weighted median		Inverse variance weighted		MR_PRESSO	
			OR (95% CI)	pval	OR (95% CI)	pval	OR (95% CI)	pval	OR (95% CI)	pval
Cis-3,4-methyleneheptanoylcarnitine	Hypertension	18	1.012 (0.948 to 1.081)	0.718	1.019 (0.982 to 1.057)	0.322	1.018 (0.978 to 1.059)	0.386	1.018 (0.987 to 1.050)	0.276
Tetrahydrocortisol glucuronide	Hypertension	18	0.895 (0.786 to 1.020)	0.115	0.949 (0.899 to 1.003)	0.063	0.954 (0.895 to 1.017)	0.148	0.961 (0.907 to 1.018)	0.194
N-acetyl-2-aminoadipate	Hypertension	15	1.092 (1.021 to 1.167)	0.023	1.059 (1.023 to 1.096)	9.96e-04	1.038 (0.995 to 1.082)	0.081	1.038 (1.007 to 1.070)	0.033
Bilirubin degradation product, C17H20N2O5 (1)	Hypertension	15	0.991 (0.971 to 1.011)	0.389	0.992 (0.972 to 1.013)	0.454	0.998 (0.982 to 1.013)	0.751		
2-methoxyhydroquinone sulfate (1)	Hypertension	8	0.990 (0.878 to 1.116)	0.879	1.021 (0.958 to 1.089)	0.518	1.027 (0.977 to 1.079)	0.290		

Exposure	Outcome	nSNP	MR Egger		Weighted median		Inverse variance weighted		MR_PRESSO	
			OR (95% CI)	pval	OR (95% CI)	pval	OR (95% CI)	pval	OR (95% CI)	pval
Bilirubin degradation product, C16H18N2O5 (3)	Hypertension	17	1.000 (0.969 to 1.033)	0.982	1.001 (0.981 to 1.021)	0.936	1.004 (0.983 to 1.026)	0.709		
3-hydroxyoctanoylcarnitine (1)	Hypertension	12	0.923 (0.786 to 1.083)	0.349	0.941 (0.874 to 1.012)	0.101	0.966 (0.908 to 1.029)	0.284		
2-methoxyhydroquinone sulfate (2)	Hypertension	8	1.097 (0.962 to 1.252)	0.215	0.994 (0.919 to 1.076)	0.888	1.018 (0.960 to 1.079)	0.558		
Cis-3,4-methyleneheptanoylglycine	Hypertension	12	0.934 (0.891 to 0.978)	0.017	0.948 (0.922 to 0.976)	2.44e-04	0.960 (0.935 to 0.986)	0.002		
Bilirubin degradation product, C16H18N2O5 (2)	Hypertension	15	1.011 (0.973 to 1.051)	0.579	1.005 (0.982 to 1.027)	0.695	1.008 (0.983 to 1.034)	0.519		

Exposure	Outcome	nSNP	MR Egger		Weighted median		Inverse variance weighted		MR_PRESSO	
			OR (95% CI)	pval	OR (95% CI)	pval	OR (95% CI)	pval	OR (95% CI)	pval
2,4-di-tert-butylphenol	Hypertension	13	1.001 (0.920 to 1.088)	0.986	0.981 (0.930 to 1.034)	0.475	0.987 (0.951 to 1.023)	0.461		
Bilirubin degradation product, C16H18N2O5 (1)	Hypertension	13	1.028 (0.975 to 1.084)	0.322	0.997 (0.976 to 1.019)	0.774	0.996 (0.959 to 1.034)	0.845	0.993 (0.976 to 1.009)	0.396
3-hydroxyoctanoylcarnitine (2)	Hypertension	12	1.010 (0.843 to 1.210)	0.918	1.049 (0.984 to 1.119)	0.140	1.017 (0.966 to 1.070)	0.531		
Pantothenate	Hypertension	18	0.985 (0.958 to 1.014)	0.320	0.997 (0.981 to 1.013)	0.688	1.000 (0.975 to 1.025)	0.985	0.993 (0.978 to 1.007)	0.343
5-oxoproline	Hypertension	12	1.017 (0.972 to 1.064)	0.495	1.015 (0.990 to 1.042)	0.245	1.008 (0.978 to 1.039)	0.612		

Exposure	Outcome	nSNP	MR Egger		Weighted median		Inverse variance weighted		MR_PRESSO	
			OR (95% CI)	pval	OR (95% CI)	pval	OR (95% CI)	pval	OR (95% CI)	pval
Phosphoethanolamine	Hypertension	15	1.024 (0.892 to 1.174)	0.743	1.027 (0.975 to 1.082)	0.319	1.022 (0.973 to 1.073)	0.393		
Picolinate	Hypertension	17	0.977 (0.891 to 1.070)	0.620	0.967 (0.926 to 1.011)	0.140	0.959 (0.917 to 1.004)	0.075	0.964 (0.938 to 0.991)	0.022
3-ureidopropionate	Hypertension	11	0.919 (0.844 to 1.000)	0.080	0.955 (0.904 to 1.008)	0.097	0.966 (0.924 to 1.011)	0.133		
Erucate (22:1n9)	Hypertension	11	1.086 (0.916 to 1.288)	0.366	1.093 (1.012 to 1.180)	0.024	1.069 (0.991 to 1.154)	0.085	1.096 (1.023 to 1.174)	0.028
Eicosenedioate (C20:1-DC)	Hypertension	10	0.984 (0.878 to 1.101)	0.781	1.003 (0.945 to 1.066)	0.912	0.986 (0.942 to 1.032)	0.550		

Exposure	Outcome	nSNP	MR Egger		Weighted median		Inverse variance weighted		MR_PRESSO	
			OR (95% CI)	pval	OR (95% CI)	pval	OR (95% CI)	pval	OR (95% CI)	pval
Allantoin	Hypertension	13	0.993 (0.899 to 1.097)	0.894	0.990 (0.936 to 1.047)	0.720	0.989 (0.950 to 1.030)	0.604		
N-acetylvaline	Hypertension	16	0.978 (0.882 to 1.083)	0.672	1.026 (0.976 to 1.078)	0.322	1.038 (0.974 to 1.105)	0.250	1.016 (0.969 to 1.066)	0.513
N-acetylleucine	Hypertension	15	1.019 (0.987 to 1.051)	0.273	1.023 (1.001 to 1.046)	0.041	1.015 (0.993 to 1.038)	0.172		
N-acetylmethionine	Hypertension	12	0.998 (0.891 to 1.119)	0.978	1.036 (0.975 to 1.101)	0.253	1.024 (0.962 to 1.090)	0.452	1.039 (0.985 to 1.097)	0.191
Glycerate	Hypertension	17	0.991 (0.872 to 1.126)	0.893	0.995 (0.944 to 1.047)	0.836	1.018 (0.957 to 1.083)	0.576	0.977 (0.937 to 1.017)	0.275

Exposure	Outcome	nSNP	MR Egger		Weighted median		Inverse variance weighted		MR_PRESSO	
			OR (95% CI)	pval	OR (95% CI)	pval	OR (95% CI)	pval	OR (95% CI)	pval
Sarcosine	Hypertension	15	0.980 (0.827 to 1.161)	0.817	0.975 (0.921 to 1.033)	0.391	0.980 (0.921 to 1.044)	0.539	0.954 (0.912 to 0.998)	0.065
Trans-4-hydroxyproline	Hypertension	10	0.981 (0.906 to 1.061)	0.639	0.986 (0.931 to 1.044)	0.631	0.975 (0.934 to 1.018)	0.252		
Thyroxine	Hypertension	13	1.028 (0.882 to 1.198)	0.729	1.014 (0.951 to 1.081)	0.664	1.040 (0.974 to 1.110)	0.242	1.023 (0.966 to 1.085)	0.452
Pipecolate	Hypertension	15	1.002 (0.994 to 1.011)	0.589	1.003 (0.994 to 1.011)	0.571	1.003 (0.995 to 1.011)	0.493		
Gamma-glutamyltyrosine	Hypertension	14	1.025 (0.952 to 1.103)	0.523	0.989 (0.942 to 1.038)	0.660	0.994 (0.960 to 1.030)	0.755		

Exposure	Outcome	nSNP	MR Egger		Weighted median		Inverse variance weighted		MR_PRESSO	
			OR (95% CI)	pval	OR (95% CI)	pval	OR (95% CI)	pval	OR (95% CI)	pval
Methyl indole-3-acetate	Hypertension	8	0.965 (0.893 to 1.044)	0.412	1.017 (0.959 to 1.079)	0.574	1.026 (0.983 to 1.070)	0.240		
Ursodeoxycholate	Hypertension	13	0.975 (0.882 to 1.077)	0.630	1.011 (0.962 to 1.063)	0.655	1.021 (0.984 to 1.059)	0.272		
3-hydroxy-3-methylglutarate	Hypertension	11	1.049 (0.964 to 1.142)	0.296	1.031 (0.979 to 1.087)	0.249	1.034 (0.994 to 1.074)	0.093		
Oleoyl ethanolamide	Hypertension	13	1.030 (0.964 to 1.101)	0.394	1.004 (0.966 to 1.044)	0.833	1.003 (0.970 to 1.038)	0.856		
4-hydroxyphenylpyruvate	Hypertension	11	0.981 (0.908 to 1.060)	0.634	0.984 (0.935 to 1.034)	0.521	0.975 (0.939 to 1.013)	0.192		

Exposure	Outcome	nSNP	MR Egger		Weighted median		Inverse variance weighted		MR_PRESSO	
			OR (95% CI)	pval	OR (95% CI)	pval	OR (95% CI)	pval	OR (95% CI)	pval
Gamma-glutamylglutamine	Hypertension	23	0.995 (0.929 to 1.066)	0.888	0.971 (0.938 to 1.005)	0.090	0.959 (0.928 to 0.991)	0.012	0.968 (0.946 to 0.992)	0.016
Alpha-tocopherol	Hypertension	16	0.998 (0.806 to 1.237)	0.989	1.010 (0.957 to 1.066)	0.720	1.025 (0.957 to 1.099)	0.480	1.005 (0.968 to 1.044)	0.784
N-acetyl-L-alanine	Hypertension	17	0.971 (0.799 to 1.181)	0.774	0.974 (0.924 to 1.026)	0.322	0.998 (0.928 to 1.073)	0.952	0.989 (0.942 to 1.040)	0.680
Citrate	Hypertension	13	0.998 (0.881 to 1.130)	0.972	0.980 (0.932 to 1.029)	0.415	0.998 (0.950 to 1.048)	0.930		
2-aminobutyrate	Hypertension	9	0.976 (0.879 to 1.083)	0.656	0.999 (0.949 to 1.050)	0.954	0.991 (0.953 to 1.030)	0.635		

Exposure	Outcome	nSNP	MR Egger		Weighted median		Inverse variance weighted		MR_PRESSO	
			OR (95% CI)	pval	OR (95% CI)	pval	OR (95% CI)	pval	OR (95% CI)	pval
Butyrate/isobutyrate (4:0)	Hypertension	11	0.914 (0.711 to 1.174)	0.499	0.972 (0.911 to 1.038)	0.401	0.971 (0.915 to 1.031)	0.335		
Urate	Hypertension	20	1.025 (0.961 to 1.093)	0.470	1.061 (1.020 to 1.104)	0.003	1.014 (0.982 to 1.047)	0.400		
3-hydroxyisobutyrate	Hypertension	13	0.984 (0.877 to 1.103)	0.783	1.053 (0.996 to 1.112)	0.069	1.051 (1.001 to 1.104)	0.046		
Vanillylmandelate (VMA)	Hypertension	23	0.989 (0.858 to 1.139)	0.877	0.998 (0.950 to 1.049)	0.952	1.038 (0.975 to 1.105)	0.237	0.996 (0.961 to 1.033)	0.846
3-aminoisobutyrate	Hypertension	13	0.984 (0.951 to 1.018)	0.358	0.990 (0.971 to 1.009)	0.313	0.985 (0.963 to 1.007)	0.184		

Exposure	Outcome	nSNP	MR Egger		Weighted median		Inverse variance weighted		MR_PRESSO	
			OR (95% CI)	pval	OR (95% CI)	pval	OR (95% CI)	pval	OR (95% CI)	pval
4-acetamidobutanoate	Hypertension	11	1.018 (0.969 to 1.069)	0.496	1.004 (0.971 to 1.037)	0.824	0.996 (0.969 to 1.025)	0.796		
2-hydroxystearate	Hypertension	15	1.111 (0.969 to 1.274)	0.157	0.974 (0.916 to 1.036)	0.407	0.965 (0.913 to 1.021)	0.215		
N1-methyladenosine	Hypertension	20	1.039 (0.850 to 1.270)	0.712	1.015 (0.961 to 1.071)	0.596	1.038 (0.970 to 1.111)	0.280	1.015 (0.969 to 1.063)	0.544
N-acetylneuraminate	Hypertension	20	1.012 (0.912 to 1.124)	0.819	1.000 (0.947 to 1.055)	0.990	1.037 (0.983 to 1.095)	0.183	1.011 (0.972 to 1.052)	0.587
Gamma-glutamylhistidine	Hypertension	9	1.069 (0.935 to 1.222)	0.360	1.066 (1.000 to 1.137)	0.051	1.047 (0.989 to 1.108)	0.114		

Exposure	Outcome	nSNP	MR Egger		Weighted median		Inverse variance weighted		MR_PRESSO	
			OR (95% CI)	pval	OR (95% CI)	pval	OR (95% CI)	pval	OR (95% CI)	pval
N-acetylglucosaminylasparagine	Hypertension	15	0.978 (0.932 to 1.026)	0.385	0.990 (0.965 to 1.016)	0.453	0.989 (0.959 to 1.019)	0.451		
Homovanillate (hva)	Hypertension	15	0.958 (0.819 to 1.120)	0.599	1.023 (0.972 to 1.076)	0.386	0.991 (0.926 to 1.062)	0.803	1.016 (0.969 to 1.065)	0.521
Acetoacetate	Hypertension	13	0.966 (0.912 to 1.024)	0.270	0.998 (0.955 to 1.043)	0.914	0.993 (0.962 to 1.025)	0.653		
Cys-gly, oxidized	Hypertension	15	1.033 (0.981 to 1.089)	0.242	1.043 (1.015 to 1.072)	0.003	1.038 (1.007 to 1.070)	0.016		
Dihomo-linoleate (20:2n6)	Hypertension	10	1.141 (0.981 to 1.326)	0.126	1.003 (0.939 to 1.072)	0.926	1.009 (0.933 to 1.092)	0.818	0.973 (0.925 to 1.023)	0.318

Exposure	Outcome	nSNP	MR Egger		Weighted median		Inverse variance weighted		MR_PRESSO	
			OR (95% CI)	pval	OR (95% CI)	pval	OR (95% CI)	pval	OR (95% CI)	pval
Cholate	Hypertension	11	1.083 (0.908 to 1.292)	0.396	1.003 (0.941 to 1.069)	0.932	1.014 (0.951 to 1.082)	0.665	0.994 (0.940 to 1.053)	0.852
Beta-hydroxyisovalerate	Hypertension	19	0.970 (0.908 to 1.037)	0.383	1.022 (0.984 to 1.062)	0.263	1.011 (0.981 to 1.043)	0.465		
Creatine	Hypertension	17	0.996 (0.944 to 1.051)	0.883	0.993 (0.963 to 1.025)	0.683	0.985 (0.951 to 1.020)	0.407	0.993 (0.965 to 1.022)	0.659
Anthranilate	Hypertension	14	0.989 (0.904 to 1.082)	0.808	0.999 (0.948 to 1.052)	0.956	1.020 (0.982 to 1.061)	0.307		
Glycerol	Hypertension	10	1.095 (0.927 to 1.294)	0.317	1.002 (0.936 to 1.071)	0.961	1.003 (0.954 to 1.055)	0.900		

Exposure	Outcome	nSNP	MR Egger		Weighted median		Inverse variance weighted		MR_PRESSO	
			OR (95% CI)	pval	OR (95% CI)	pval	OR (95% CI)	pval	OR (95% CI)	pval
Gamma-glutamylleucine	Hypertension	6	1.045 (0.945 to 1.154)	0.440	1.035 (0.975 to 1.099)	0.258	1.025 (0.962 to 1.092)	0.451		
Choline	Hypertension	14	0.980 (0.849 to 1.133)	0.793	0.986 (0.929 to 1.046)	0.632	1.000 (0.944 to 1.060)	0.996	0.976 (0.933 to 1.021)	0.306
3-methoxytyrosine	Hypertension	16	1.098 (0.921 to 1.309)	0.316	1.041 (0.989 to 1.096)	0.121	1.007 (0.940 to 1.078)	0.843	0.993 (0.936 to 1.052)	0.807
3-phosphoglycerate	Hypertension	12	0.961 (0.846 to 1.091)	0.550	1.044 (0.984 to 1.107)	0.155	1.042 (0.997 to 1.089)	0.071		
1-palmitoyl-2-linoleoyl-gpc (16:0/18:2)	Hypertension	16	1.059 (0.990 to 1.132)	0.116	1.013 (0.978 to 1.049)	0.469	1.006 (0.972 to 1.043)	0.721	0.995 (0.966 to 1.025)	0.738

Exposure	Outcome	nSNP	MR Egger		Weighted median		Inverse variance weighted		MR_PRESSO	
			OR (95% CI)	pval	OR (95% CI)	pval	OR (95% CI)	pval	OR (95% CI)	pval
1-palmitoyl-2-oleoyl-GPE (16:0/18:1)	Hypertension	21	0.987 (0.904 to 1.078)	0.770	1.014 (0.984 to 1.045)	0.364	1.038 (0.995 to 1.083)	0.085	1.025 (0.997 to 1.054)	0.104
1-palmitoyl-2-linoleoyl-GPI (16:0/18:2)	Hypertension	19	0.986 (0.918 to 1.060)	0.707	1.025 (0.989 to 1.062)	0.181	0.999 (0.966 to 1.033)	0.945	1.007 (0.981 to 1.033)	0.606
Taurocholic acid	Hypertension	15	0.942 (0.859 to 1.032)	0.219	1.023 (0.975 to 1.073)	0.353	1.016 (0.973 to 1.060)	0.476		
Taurodeoxycholate	Hypertension	9	1.032 (0.875 to 1.218)	0.719	1.055 (0.988 to 1.128)	0.112	1.034 (0.976 to 1.096)	0.256		
Stearoyl sphingomyelin (d18:1/18:0)	Hypertension	25	1.024 (0.929 to 1.128)	0.638	1.020 (0.981 to 1.061)	0.321	0.995 (0.950 to 1.042)	0.836	1.009 (0.976 to 1.043)	0.598

Exposure	Outcome	nSNP	MR Egger		Weighted median		Inverse variance weighted		MR_PRESSO	
			OR (95% CI)	pval	OR (95% CI)	pval	OR (95% CI)	pval	OR (95% CI)	pval
Ceramide (d18:1/16:0)	Hypertension	17	1.087 (0.926 to 1.277)	0.324	1.022 (0.965 to 1.083)	0.453	1.043 (0.974 to 1.117)	0.224	1.024 (0.975 to 1.074)	0.362
Taurochenodeoxycholate	Hypertension	11	1.020 (0.883 to 1.178)	0.794	1.006 (0.949 to 1.067)	0.838	0.985 (0.938 to 1.035)	0.545		
4-hydroxyphenylacetate	Hypertension	13	1.062 (0.989 to 1.139)	0.126	1.026 (0.976 to 1.078)	0.317	1.027 (0.981 to 1.075)	0.249		
5,6-dihydrothymine	Hypertension	9	0.936 (0.815 to 1.074)	0.378	1.010 (0.943 to 1.082)	0.771	1.010 (0.960 to 1.063)	0.704		
1-palmitoyl-2-oleoyl-gpc (16:0/18:1)	Hypertension	15	0.983 (0.888 to 1.088)	0.747	1.034 (0.982 to 1.089)	0.208	0.989 (0.946 to 1.034)	0.629		

Exposure	Outcome	nSNP	MR Egger		Weighted median		Inverse variance weighted		MR_PRESSO	
			OR (95% CI)	pval	OR (95% CI)	pval	OR (95% CI)	pval	OR (95% CI)	pval
N-stearoyl-sphingosine (d18:1/18:0)	Hypertension	14	1.106 (0.893 to 1.371)	0.374	1.021 (0.970 to 1.074)	0.427	1.011 (0.946 to 1.082)	0.740	1.002 (0.959 to 1.047)	0.925
Glycochenodeoxycholate	Hypertension	15	0.957 (0.860 to 1.064)	0.432	0.995 (0.947 to 1.046)	0.851	1.018 (0.962 to 1.078)	0.536	1.013 (0.976 to 1.052)	0.507
N-formylmethionine	Hypertension	15	1.059 (0.930 to 1.206)	0.404	1.026 (0.974 to 1.082)	0.331	1.028 (0.976 to 1.084)	0.299	1.001 (0.969 to 1.034)	0.950
N6,n6,n6-trimethyllysine	Hypertension	9	0.961 (0.909 to 1.017)	0.211	0.948 (0.907 to 0.990)	0.016	0.951 (0.918 to 0.986)	0.006		
S-adenosylhomocysteine (SAH)	Hypertension	14	0.974 (0.857 to 1.107)	0.694	1.012 (0.970 to 1.056)	0.587	1.040 (0.967 to 1.118)	0.293	1.022 (0.982 to 1.063)	0.308

Exposure	Outcome	nSNP	MR Egger		Weighted median		Inverse variance weighted		MR_PRESSO	
			OR (95% CI)	pval	OR (95% CI)	pval	OR (95% CI)	pval	OR (95% CI)	pval
4-acetaminophen sulfate	Hypertension	12	0.998 (0.931 to 1.070)	0.954	0.998 (0.958 to 1.040)	0.924	1.001 (0.966 to 1.037)	0.947		
2-hydroxyhippurate (salicylurate)	Hypertension	11	0.977 (0.854 to 1.118)	0.745	1.017 (0.953 to 1.086)	0.607	0.989 (0.931 to 1.051)	0.714		
Quinolinate	Hypertension	15	1.086 (0.931 to 1.267)	0.314	1.036 (0.982 to 1.093)	0.199	1.080 (1.009 to 1.156)	0.027	1.048 (1.017 to 1.081)	0.012
Eicosapentaenoate (EPA; 20:5n3)	Hypertension	10	0.885 (0.785 to 0.996)	0.078	0.994 (0.946 to 1.044)	0.797	0.999 (0.931 to 1.071)	0.969	1.019 (0.971 to 1.069)	0.472
Laurate (12:0)	Hypertension	9	0.939 (0.778 to 1.133)	0.533	0.970 (0.896 to 1.049)	0.446	0.951 (0.892 to 1.014)	0.127		

Exposure	Outcome	nSNP	MR Egger		Weighted median		Inverse variance weighted		MR_PRESSO	
			OR (95% CI)	pval	OR (95% CI)	pval	OR (95% CI)	pval	OR (95% CI)	pval
N-acetylputrescine	Hypertension	18	1.001 (0.962 to 1.043)	0.944	1.001 (0.979 to 1.023)	0.960	0.999 (0.974 to 1.025)	0.946	1.001 (0.984 to 1.019)	0.894
Methylsuccinate	Hypertension	13	1.003 (0.963 to 1.045)	0.885	0.989 (0.959 to 1.019)	0.457	0.997 (0.972 to 1.021)	0.790		
9,10-DiHOME	Hypertension	13	0.986 (0.907 to 1.073)	0.755	1.009 (0.968 to 1.052)	0.660	0.997 (0.943 to 1.053)	0.902	0.992 (0.955 to 1.030)	0.684
Linoleate (18:2n6)	Hypertension	12	1.100 (0.976 to 1.239)	0.148	0.999 (0.937 to 1.064)	0.963	0.978 (0.933 to 1.025)	0.360		
Adenosine 5'-diphosphate (ADP)	Hypertension	15	0.944 (0.863 to 1.033)	0.231	1.028 (0.987 to 1.071)	0.189	1.018 (0.983 to 1.053)	0.320		

Exposure	Outcome	nSNP	MR Egger		Weighted median		Inverse variance weighted		MR_PRESSO	
			OR (95% CI)	pval	OR (95% CI)	pval	OR (95% CI)	pval	OR (95% CI)	pval
Adenosine 5'-monophosphate (AMP)	Hypertension	8	1.078 (0.817 to 1.423)	0.613	1.058 (0.971 to 1.152)	0.196	1.057 (0.954 to 1.172)	0.289	1.063 (0.999 to 1.131)	0.113
Adenosine 3',5'-cyclic monophosphate (camp)	Hypertension	16	0.999 (0.883 to 1.131)	0.990	1.029 (0.985 to 1.074)	0.200	1.011 (0.975 to 1.048)	0.569		
Arachidonate (20:4n6)	Hypertension	13	1.009 (0.960 to 1.060)	0.737	0.989 (0.958 to 1.022)	0.507	0.993 (0.967 to 1.020)	0.630		
5-methylthioadenosine (mta)	Hypertension	13	0.937 (0.798 to 1.101)	0.447	0.987 (0.929 to 1.048)	0.670	1.020 (0.955 to 1.089)	0.551	0.998 (0.938 to 1.060)	0.939
Cortisone	Hypertension	8	0.900 (0.799 to 1.014)	0.134	0.961 (0.888 to 1.040)	0.320	0.959 (0.907 to 1.014)	0.143		

Exposure	Outcome	nSNP	MR Egger		Weighted median		Inverse variance weighted		MR_PRESSO	
			OR (95% CI)	pval	OR (95% CI)	pval	OR (95% CI)	pval	OR (95% CI)	pval
Beta-alanine	Hypertension	11	0.952 (0.835 to 1.086)	0.486	0.999 (0.954 to 1.046)	0.961	1.000 (0.939 to 1.066)	0.996	0.974 (0.932 to 1.019)	0.289
Succinate	Hypertension	13	1.006 (0.983 to 1.031)	0.605	1.010 (0.992 to 1.029)	0.293	1.007 (0.988 to 1.027)	0.462		
Phosphocholine	Hypertension	19	1.006 (0.987 to 1.025)	0.537	1.006 (0.987 to 1.026)	0.540	1.003 (0.988 to 1.019)	0.676		
Cysteinylglycine	Hypertension	11	1.052 (0.893 to 1.239)	0.561	1.052 (0.998 to 1.109)	0.061	1.022 (0.956 to 1.093)	0.526	0.984 (0.907 to 1.066)	0.699
Phenylpyruvate	Hypertension	18	1.016 (0.902 to 1.144)	0.799	1.037 (0.985 to 1.091)	0.165	1.040 (1.001 to 1.081)	0.046		

Exposure	Outcome	nSNP	MR Egger		Weighted median		Inverse variance weighted		MR_PRESSO	
			OR (95% CI)	pval	OR (95% CI)	pval	OR (95% CI)	pval	OR (95% CI)	pval
Creatinine	Hypertension	12	1.042 (0.933 to 1.163)	0.483	1.056 (1.000 to 1.114)	0.048	1.070 (1.002 to 1.143)	0.044	1.061 (1.021 to 1.102)	0.014
3-Hydroxybutyrate	Hypertension	9	0.806 (0.697 to 0.931)	0.022	0.988 (0.922 to 1.059)	0.729	0.951 (0.891 to 1.015)	0.132		
Cholesterol	Hypertension	10	0.937 (0.829 to 1.059)	0.326	0.982 (0.920 to 1.050)	0.599	0.983 (0.923 to 1.046)	0.580		
2-hydroxyphenylacetate	Hypertension	12	1.018 (0.897 to 1.154)	0.793	1.008 (0.958 to 1.059)	0.764	0.996 (0.942 to 1.054)	0.895	0.980 (0.931 to 1.031)	0.450
Aspartate	Hypertension	12	0.996 (0.897 to 1.106)	0.947	1.003 (0.950 to 1.059)	0.906	1.022 (0.982 to 1.064)	0.284		

Exposure	Outcome	nSNP	MR Egger		Weighted median		Inverse variance weighted		MR_PRESSO	
			OR (95% CI)	pval	OR (95% CI)	pval	OR (95% CI)	pval	OR (95% CI)	pval
3-(4-hydroxyphenyl)lactate	Hypertension	13	1.077 (0.938 to 1.236)	0.315	0.982 (0.934 to 1.032)	0.465	0.965 (0.913 to 1.021)	0.216	0.984 (0.950 to 1.018)	0.370
Arginine	Hypertension	19	1.052 (0.927 to 1.194)	0.443	0.975 (0.929 to 1.022)	0.290	0.980 (0.936 to 1.027)	0.404	0.948 (0.913 to 0.984)	0.013
Gluconate	Hypertension	13	0.993 (0.967 to 1.020)	0.628	0.998 (0.982 to 1.016)	0.860	0.998 (0.976 to 1.021)	0.871		
Sphinganine	Hypertension	14	0.961 (0.865 to 1.069)	0.481	0.991 (0.944 to 1.040)	0.703	0.975 (0.940 to 1.011)	0.174		
Gamma-glutamylglutamate	Hypertension	16	1.001 (0.807 to 1.240)	0.996	1.023 (0.958 to 1.092)	0.495	0.975 (0.905 to 1.051)	0.513	1.002 (0.952 to 1.056)	0.927

Exposure	Outcome	nSNP	MR Egger		Weighted median		Inverse variance weighted		MR_PRESSO	
			OR (95% CI)	pval	OR (95% CI)	pval	OR (95% CI)	pval	OR (95% CI)	pval
Flavin adenine dinucleotide (FAD)	Hypertension	18	1.024 (0.971 to 1.080)	0.388	1.011 (0.977 to 1.046)	0.547	0.987 (0.958 to 1.018)	0.416		
Histidine	Hypertension	14	0.995 (0.918 to 1.078)	0.902	1.005 (0.972 to 1.039)	0.787	0.990 (0.935 to 1.047)	0.720	1.014 (0.971 to 1.058)	0.549
Cortisol	Hypertension	12	0.889 (0.784 to 1.007)	0.093	1.010 (0.953 to 1.071)	0.727	1.004 (0.941 to 1.072)	0.896	1.029 (0.983 to 1.077)	0.247
Hypotaurine	Hypertension	24	1.037 (0.907 to 1.187)	0.599	1.008 (0.964 to 1.054)	0.733	0.988 (0.942 to 1.036)	0.614	0.986 (0.952 to 1.022)	0.450
Glutarate (C5-DC)	Hypertension	15	0.996 (0.923 to 1.076)	0.928	0.961 (0.913 to 1.011)	0.126	0.984 (0.948 to 1.021)	0.393		

Exposure	Outcome	nSNP	MR Egger		Weighted median		Inverse variance weighted		MR_PRESSO	
			OR (95% CI)	pval	OR (95% CI)	pval	OR (95% CI)	pval	OR (95% CI)	pval
S-1-pyrroline-5-carboxylate	Hypertension	9	1.127 (1.004 to 1.264)	0.081	1.020 (0.957 to 1.088)	0.537	0.994 (0.917 to 1.077)	0.883	0.992 (0.928 to 1.061)	0.829
Cystine	Hypertension	8	0.942 (0.841 to 1.056)	0.343	1.009 (0.936 to 1.087)	0.817	1.020 (0.961 to 1.082)	0.518		
Cystathionine	Hypertension	17	0.995 (0.926 to 1.069)	0.886	0.972 (0.926 to 1.020)	0.252	0.954 (0.921 to 0.989)	0.010		
Guanidinoacetate	Hypertension	17	0.983 (0.870 to 1.111)	0.791	1.046 (1.000 to 1.095)	0.052	1.077 (1.014 to 1.145)	0.016	1.069 (1.025 to 1.114)	0.008
Sphingosine	Hypertension	7	0.979 (0.830 to 1.156)	0.814	0.990 (0.930 to 1.054)	0.757	1.000 (0.954 to 1.049)	0.987		

Exposure	Outcome	nSNP	MR Egger		Weighted median		Inverse variance weighted		MR_PRESSO	
			OR (95% CI)	pval	OR (95% CI)	pval	OR (95% CI)	pval	OR (95% CI)	pval
Deoxycholate	Hypertension	17	0.944 (0.847 to 1.053)	0.319	0.968 (0.925 to 1.013)	0.157	0.984 (0.935 to 1.035)	0.529	0.969 (0.932 to 1.008)	0.142
Glycocholate	Hypertension	22	1.002 (0.928 to 1.082)	0.964	0.991 (0.948 to 1.037)	0.706	0.998 (0.965 to 1.033)	0.908		
Palmitoleate (16:1n7)	Hypertension	13	1.064 (0.852 to 1.328)	0.595	1.014 (0.951 to 1.082)	0.662	1.014 (0.954 to 1.078)	0.652		
Leucine	Hypertension	13	0.929 (0.776 to 1.113)	0.441	0.974 (0.905 to 1.049)	0.487	0.981 (0.923 to 1.042)	0.538		
Stearate (18:0)	Hypertension	8	1.417 (1.084 to 1.853)	0.044	0.980 (0.900 to 1.066)	0.638	1.051 (0.936 to 1.180)	0.401	1.039 (0.957 to 1.129)	0.404

Exposure	Outcome	nSNP	MR Egger		Weighted median		Inverse variance weighted		MR_PRESSO	
			OR (95% CI)	pval	OR (95% CI)	pval	OR (95% CI)	pval	OR (95% CI)	pval
Methionine	Hypertension	13	0.979 (0.860 to 1.113)	0.751	1.053 (0.990 to 1.121)	0.102	1.015 (0.962 to 1.071)	0.583		
Orotate	Hypertension	20	0.988 (0.947 to 1.030)	0.576	0.994 (0.974 to 1.015)	0.570	0.998 (0.970 to 1.025)	0.861	0.994 (0.971 to 1.017)	0.607
Phosphate	Hypertension	14	1.056 (0.947 to 1.177)	0.350	1.023 (0.970 to 1.078)	0.408	1.033 (0.989 to 1.080)	0.143		
Inosine	Hypertension	10	1.054 (0.969 to 1.146)	0.257	1.019 (0.967 to 1.074)	0.482	1.027 (0.987 to 1.070)	0.190		
Inosine 5'-monophosphate (IMP)	Hypertension	14	1.032 (0.970 to 1.098)	0.339	1.020 (0.982 to 1.059)	0.314	1.013 (0.985 to 1.042)	0.361		

Exposure	Outcome	nSNP	MR Egger		Weighted median		Inverse variance weighted		MR_PRESSO	
			OR (95% CI)	pval	OR (95% CI)	pval	OR (95% CI)	pval	OR (95% CI)	pval
Phenylalanine	Hypertension	12	1.033 (0.949 to 1.125)	0.470	0.984 (0.935 to 1.035)	0.525	1.002 (0.966 to 1.039)	0.921		
Palmitate (16:0)	Hypertension	12	1.117 (0.934 to 1.335)	0.253	1.029 (0.962 to 1.099)	0.407	1.041 (0.972 to 1.116)	0.252	1.010 (0.955 to 1.068)	0.728
Malate	Hypertension	6	1.004 (0.780 to 1.292)	0.980	0.993 (0.932 to 1.058)	0.831	1.007 (0.924 to 1.098)	0.873		
Isoleucine	Hypertension	11	1.081 (0.935 to 1.251)	0.321	0.997 (0.936 to 1.062)	0.930	1.003 (0.957 to 1.052)	0.896		
Nicotinamide	Hypertension	12	1.038 (0.816 to 1.321)	0.769	1.049 (0.981 to 1.122)	0.160	0.953 (0.869 to 1.046)	0.311	0.992 (0.927 to 1.061)	0.816

Exposure	Outcome	nSNP	MR Egger		Weighted median		Inverse variance weighted		MR_PRESSO	
			OR (95% CI)	pval	OR (95% CI)	pval	OR (95% CI)	pval	OR (95% CI)	pval
Myo-inositol	Hypertension	17	1.118 (0.983 to 1.272)	0.109	0.981 (0.931 to 1.034)	0.476	1.018 (0.959 to 1.079)	0.561	0.980 (0.934 to 1.028)	0.414
Lysine	Hypertension	19	0.934 (0.861 to 1.013)	0.118	0.982 (0.936 to 1.030)	0.462	0.989 (0.958 to 1.021)	0.506		
Citrulline	Hypertension	15	0.978 (0.910 to 1.052)	0.565	0.994 (0.939 to 1.052)	0.833	1.008 (0.969 to 1.048)	0.693		
Ornithine	Hypertension	15	1.032 (0.949 to 1.123)	0.474	1.039 (0.983 to 1.097)	0.175	1.031 (0.986 to 1.079)	0.183		
Retinol (Vitamin A)	Hypertension	13	0.931 (0.842 to 1.030)	0.193	0.934 (0.883 to 0.988)	0.017	0.959 (0.920 to 1.000)	0.050		

Exposure	Outcome	nSNP	MR Egger		Weighted median		Inverse variance weighted		MR_PRESSO	
			OR (95% CI)	pval	OR (95% CI)	pval	OR (95% CI)	pval	OR (95% CI)	pval
1-methylnicotinamide	Hypertension	14	0.905 (0.780 to 1.050)	0.213	1.002 (0.943 to 1.064)	0.953	1.056 (0.993 to 1.122)	0.081	1.034 (0.984 to 1.087)	0.211
Uridine	Hypertension	8	0.976 (0.829 to 1.150)	0.782	0.963 (0.906 to 1.024)	0.231	0.942 (0.886 to 1.003)	0.062		
Plasma lactate	Hypertension	10	1.008 (0.869 to 1.168)	0.920	1.005 (0.935 to 1.080)	0.896	1.011 (0.954 to 1.070)	0.714		
Pristanate	Hypertension	6	0.971 (0.861 to 1.095)	0.658	0.987 (0.926 to 1.052)	0.683	0.980 (0.931 to 1.031)	0.428		
Phytanate	Hypertension	13	1.053 (0.943 to 1.175)	0.380	0.981 (0.925 to 1.040)	0.514	0.987 (0.923 to 1.055)	0.693	0.965 (0.914 to 1.019)	0.229

Exposure	Outcome	nSNP	MR Egger		Weighted median		Inverse variance weighted		MR_PRESSO	
			OR (95% CI)	pval	OR (95% CI)	pval	OR (95% CI)	pval	OR (95% CI)	pval
Plasma free proline	Hypertension	21	1.004 (0.930 to 1.083)	0.928	1.011 (0.965 to 1.059)	0.648	1.010 (0.967 to 1.054)	0.655	0.998 (0.961 to 1.037)	0.924
Glutamate	Hypertension	12	1.049 (0.949 to 1.159)	0.372	1.044 (0.985 to 1.107)	0.149	1.060 (1.017 to 1.105)	0.006		
2'-deoxyuridine	Hypertension	16	1.041 (0.887 to 1.222)	0.631	0.985 (0.946 to 1.025)	0.457	0.959 (0.885 to 1.039)	0.309	0.990 (0.966 to 1.015)	0.447
Myristate (14:0)	Hypertension	11	1.083 (0.968 to 1.211)	0.197	1.003 (0.947 to 1.062)	0.918	1.013 (0.970 to 1.057)	0.558		
Serine	Hypertension	23	0.952 (0.863 to 1.050)	0.334	0.975 (0.932 to 1.019)	0.263	0.980 (0.940 to 1.021)	0.324	0.997 (0.963 to 1.031)	0.853

Exposure	Outcome	nSNP	MR Egger		Weighted median		Inverse variance weighted		MR_PRESSO	
			OR (95% CI)	pval	OR (95% CI)	pval	OR (95% CI)	pval	OR (95% CI)	pval
Trans-urocanate	Hypertension	11	0.895 (0.834 to 0.961)	0.013	0.970 (0.937 to 1.005)	0.095	0.997 (0.952 to 1.043)	0.881	0.984 (0.945 to 1.023)	0.439
Urea	Hypertension	13	1.019 (1.002 to 1.037)	0.053	1.018 (0.999 to 1.037)	0.060	1.017 (1.002 to 1.032)	0.024		
Pyridoxal	Hypertension	22	0.983 (0.936 to 1.032)	0.492	0.978 (0.939 to 1.019)	0.290	0.981 (0.955 to 1.008)	0.160		
Glutamine	Hypertension	14	0.954 (0.866 to 1.052)	0.363	0.969 (0.927 to 1.012)	0.149	0.973 (0.926 to 1.022)	0.269	0.988 (0.949 to 1.029)	0.579
Taurine	Hypertension	6	1.053 (0.838 to 1.323)	0.682	0.977 (0.892 to 1.071)	0.625	0.999 (0.914 to 1.092)	0.983		

Exposure	Outcome	nSNP	MR Egger		Weighted median		Inverse variance weighted		MR_PRESSO	
			OR (95% CI)	pval	OR (95% CI)	pval	OR (95% CI)	pval	OR (95% CI)	pval
Salicylate	Hypertension	16	0.953 (0.852 to 1.065)	0.406	0.951 (0.895 to 1.011)	0.110	0.953 (0.906 to 1.002)	0.062		
Serotonin	Hypertension	15	1.007 (0.927 to 1.093)	0.875	1.010 (0.959 to 1.064)	0.710	1.003 (0.959 to 1.049)	0.884		
Spermidine	Hypertension	14	1.141 (0.977 to 1.331)	0.121	0.982 (0.930 to 1.036)	0.506	0.974 (0.915 to 1.037)	0.413	1.003 (0.968 to 1.038)	0.887
Dimethylglycine	Hypertension	15	1.024 (0.943 to 1.113)	0.582	1.011 (0.979 to 1.044)	0.499	0.991 (0.943 to 1.041)	0.706	0.992 (0.958 to 1.027)	0.648
Betaine	Hypertension	15	1.003 (0.890 to 1.129)	0.967	0.988 (0.951 to 1.026)	0.527	1.003 (0.966 to 1.042)	0.857	0.988 (0.956 to 1.020)	0.460

Exposure	Outcome	nSNP	MR Egger		Weighted median		Inverse variance weighted		MR_PRESSO	
			OR (95% CI)	pval	OR (95% CI)	pval	OR (95% CI)	pval	OR (95% CI)	pval
Xylose	Hypertension	10	0.949 (0.849 to 1.060)	0.382	0.970 (0.914 to 1.029)	0.310	0.964 (0.911 to 1.021)	0.214	0.989 (0.932 to 1.050)	0.735
Tyrosine	Hypertension	15	1.024 (0.960 to 1.091)	0.489	1.015 (0.974 to 1.058)	0.486	1.024 (0.989 to 1.060)	0.187		
Caprate (10:0)	Hypertension	9	1.034 (0.897 to 1.191)	0.661	1.021 (0.954 to 1.094)	0.546	1.004 (0.940 to 1.072)	0.913	0.979 (0.928 to 1.034)	0.473
Cytidine	Hypertension	17	1.001 (0.931 to 1.076)	0.988	0.995 (0.947 to 1.044)	0.828	0.992 (0.957 to 1.028)	0.656		
Pyruvate	Hypertension	12	0.972 (0.808 to 1.170)	0.771	1.059 (0.981 to 1.144)	0.142	1.065 (0.989 to 1.147)	0.096	1.094 (1.027 to 1.166)	0.020

Exposure	Outcome	nSNP	MR Egger		Weighted median		Inverse variance weighted		MR_PRESSO	
			OR (95% CI)	pval	OR (95% CI)	pval	OR (95% CI)	pval	OR (95% CI)	pval
Alanine	Hypertension	10	0.984 (0.852 to 1.135)	0.826	1.027 (0.959 to 1.101)	0.440	1.009 (0.947 to 1.075)	0.780		
Threonine	Hypertension	18	0.986 (0.848 to 1.146)	0.856	0.964 (0.917 to 1.014)	0.158	0.983 (0.933 to 1.036)	0.518	0.983 (0.944 to 1.025)	0.437
Cytosine	Hypertension	8	0.931 (0.807 to 1.074)	0.364	0.978 (0.913 to 1.048)	0.528	0.970 (0.916 to 1.027)	0.291		
12,13-DiHOME	Hypertension	17	0.981 (0.912 to 1.055)	0.616	1.035 (0.983 to 1.089)	0.192	1.025 (0.989 to 1.063)	0.173		
Mannose	Hypertension	12	0.896 (0.837 to 0.959)	0.010	0.928 (0.890 to 0.968)	5.36e-04	0.945 (0.914 to 0.977)	7.68e-04		

Exposure	Outcome	nSNP	MR Egger		Weighted median		Inverse variance weighted		MR_PRESSO	
			OR (95% CI)	pval	OR (95% CI)	pval	OR (95% CI)	pval	OR (95% CI)	pval
Caffeine	Hypertension	12	0.955 (0.841 to 1.085)	0.497	1.047 (0.988 to 1.109)	0.118	1.019 (0.957 to 1.086)	0.558	1.004 (0.945 to 1.067)	0.888
Fructose	Hypertension	14	1.006 (0.927 to 1.091)	0.892	1.002 (0.948 to 1.058)	0.957	0.991 (0.952 to 1.033)	0.675		
Alpha-ketobutyrate	Hypertension	9	0.931 (0.801 to 1.082)	0.384	0.968 (0.919 to 1.020)	0.230	0.986 (0.901 to 1.078)	0.755	0.959 (0.923 to 0.996)	0.080
Cysteine	Hypertension	10	0.949 (0.911 to 0.989)	0.039	0.974 (0.939 to 1.010)	0.148	0.985 (0.949 to 1.023)	0.438		
Blood sugar	Hypertension	12	1.005 (0.964 to 1.047)	0.825	1.001 (0.976 to 1.026)	0.939	0.995 (0.963 to 1.028)	0.755		

Exposure	Outcome	nSNP	MR Egger		Weighted median		Inverse variance weighted		MR_PRESSO	
			OR (95% CI)	pval	OR (95% CI)	pval	OR (95% CI)	pval	OR (95% CI)	pval
Tryptophan	Hypertension	10	0.980 (0.847 to 1.133)	0.790	0.972 (0.912 to 1.035)	0.372	0.975 (0.927 to 1.024)	0.308		
Valine	Hypertension	8	0.887 (0.746 to 1.056)	0.226	0.915 (0.847 to 0.989)	0.026	0.929 (0.877 to 0.984)	0.012		
Pseudouridine	Hypertension	15	1.158 (0.859 to 1.562)	0.353	1.060 (0.994 to 1.131)	0.076	1.070 (0.986 to 1.160)	0.103	1.077 (1.034 to 1.123)	0.004
Dihydroorotate	Hypertension	21	1.004 (0.908 to 1.109)	0.942	0.972 (0.938 to 1.006)	0.106	0.977 (0.935 to 1.022)	0.321	0.979 (0.941 to 1.018)	0.296
Caprylate (8:0)	Hypertension	14	1.002 (0.983 to 1.021)	0.879	1.002 (0.987 to 1.018)	0.770	1.005 (0.989 to 1.022)	0.531		

Exposure	Outcome	nSNP	MR Egger		Weighted median		Inverse variance weighted		MR_PRESSO	
			OR (95% CI)	pval	OR (95% CI)	pval	OR (95% CI)	pval	OR (95% CI)	pval
Arachidate (20:0)	Hypertension	8	1.053 (0.887 to 1.251)	0.577	0.968 (0.904 to 1.035)	0.340	0.947 (0.862 to 1.041)	0.258	0.947 (0.895 to 1.001)	0.112
Maltose	Hypertension	11	1.004 (0.909 to 1.108)	0.943	1.009 (0.958 to 1.062)	0.743	1.032 (0.994 to 1.071)	0.103		
N-stearoyl-sphinganine (d18:0/18:0)	Hypertension	6	0.957 (0.901 to 1.017)	0.228	0.970 (0.930 to 1.012)	0.160	0.983 (0.940 to 1.027)	0.441		
Sucrose	Hypertension	8	1.069 (0.887 to 1.288)	0.512	1.003 (0.936 to 1.075)	0.928	1.009 (0.945 to 1.078)	0.785		
Caproate (6:0)	Hypertension	14	0.966 (0.881 to 1.059)	0.469	1.034 (0.988 to 1.082)	0.147	1.014 (0.974 to 1.056)	0.495		

Exposure	Outcome	nSNP	MR Egger		Weighted median		Inverse variance weighted		MR_PRESSO	
			OR (95% CI)	pval	OR (95% CI)	pval	OR (95% CI)	pval	OR (95% CI)	pval
Nonadecanoate (19:0)	Hypertension	14	0.973 (0.893 to 1.061)	0.545	1.007 (0.954 to 1.062)	0.804	1.009 (0.970 to 1.049)	0.670		
Plasma free asparagine	Hypertension	15	1.038 (0.992 to 1.086)	0.131	1.026 (1.000 to 1.053)	0.054	1.012 (0.981 to 1.045)	0.442	1.000 (0.969 to 1.032)	0.997
X-07765	Hypertension	16	1.028 (0.917 to 1.151)	0.646	1.028 (0.969 to 1.091)	0.355	1.040 (0.990 to 1.093)	0.119	1.016 (0.976 to 1.058)	0.446
Kynurenate	Hypertension	19	0.960 (0.899 to 1.025)	0.241	1.009 (0.974 to 1.045)	0.621	1.040 (0.998 to 1.083)	0.062	1.035 (0.998 to 1.073)	0.085
Alpha-ketoglutarate	Hypertension	14	0.966 (0.880 to 1.061)	0.485	0.985 (0.941 to 1.031)	0.525	0.988 (0.955 to 1.022)	0.482		

Exposure	Outcome	nSNP	MR Egger		Weighted median		Inverse variance weighted		MR_PRESSO	
			OR (95% CI)	pval	OR (95% CI)	pval	OR (95% CI)	pval	OR (95% CI)	pval
X-11308	Hypertension	16	0.963 (0.903 to 1.027)	0.267	0.966 (0.926 to 1.008)	0.115	0.998 (0.963 to 1.035)	0.925	1.020 (0.982 to 1.058)	0.324
Pentadecanoate (15:0)	Hypertension	9	1.077 (0.852 to 1.362)	0.553	1.020 (0.953 to 1.092)	0.563	1.060 (0.972 to 1.157)	0.186	1.026 (1.001 to 1.052)	0.098
X-11315	Hypertension	16	1.032 (0.959 to 1.110)	0.419	0.995 (0.951 to 1.041)	0.838	0.999 (0.958 to 1.042)	0.973	0.988 (0.952 to 1.026)	0.550
Margarate (17:0)	Hypertension	10	1.134 (0.893 to 1.440)	0.334	1.102 (1.016 to 1.195)	0.019	1.111 (1.019 to 1.211)	0.016	1.122 (1.048 to 1.201)	0.013
X-11372	Hypertension	12	0.949 (0.861 to 1.045)	0.311	0.961 (0.917 to 1.007)	0.098	0.957 (0.917 to 0.999)	0.044		

Exposure	Outcome	nSNP	MR Egger		Weighted median		Inverse variance weighted		MR_PRESSO	
			OR (95% CI)	pval	OR (95% CI)	pval	OR (95% CI)	pval	OR (95% CI)	pval
X-10458	Hypertension	15	1.002 (0.923 to 1.087)	0.971	0.995 (0.949 to 1.044)	0.844	1.005 (0.973 to 1.039)	0.753		
X-11299	Hypertension	12	1.008 (0.895 to 1.136)	0.895	1.045 (0.990 to 1.103)	0.112	1.040 (0.982 to 1.101)	0.184	1.043 (1.006 to 1.081)	0.050
X-11478	Hypertension	16	0.974 (0.903 to 1.050)	0.499	1.019 (0.972 to 1.068)	0.437	1.011 (0.979 to 1.044)	0.489		
X-11849	Hypertension	16	1.043 (0.915 to 1.189)	0.537	1.004 (0.956 to 1.055)	0.875	1.000 (0.950 to 1.052)	0.993	1.033 (0.998 to 1.069)	0.090
X-11787	Hypertension	11	0.966 (0.908 to 1.028)	0.302	0.974 (0.934 to 1.016)	0.226	0.993 (0.958 to 1.030)	0.719		

Exposure	Outcome	nSNP	MR Egger		Weighted median		Inverse variance weighted		MR_PRESSO	
			OR (95% CI)	pval	OR (95% CI)	pval	OR (95% CI)	pval	OR (95% CI)	pval
X-11632	Hypertension	14	0.932 (0.861 to 1.009)	0.109	0.957 (0.911 to 1.005)	0.078	0.955 (0.922 to 0.990)	0.013		
X-11852	Hypertension	13	0.947 (0.828 to 1.083)	0.445	0.979 (0.922 to 1.040)	0.498	0.973 (0.925 to 1.023)	0.289		
X-11847	Hypertension	12	1.012 (0.879 to 1.165)	0.868	1.010 (0.953 to 1.070)	0.745	1.004 (0.950 to 1.062)	0.879		
X-11483	Hypertension	14	0.990 (0.902 to 1.086)	0.833	1.027 (0.972 to 1.085)	0.348	0.992 (0.945 to 1.042)	0.754	0.978 (0.938 to 1.021)	0.330
X-11470	Hypertension	23	1.045 (1.002 to 1.090)	0.051	1.005 (0.967 to 1.044)	0.817	1.026 (1.000 to 1.052)	0.046		

Exposure	Outcome	nSNP	MR Egger		Weighted median		Inverse variance weighted		MR_PRESSO	
			OR (95% CI)	pval	OR (95% CI)	pval	OR (95% CI)	pval	OR (95% CI)	pval
X-11381	Hypertension	18	1.001 (0.984 to 1.017)	0.944	1.004 (0.988 to 1.020)	0.646	1.012 (0.996 to 1.028)	0.148		
X-11850	Hypertension	12	1.095 (0.983 to 1.220)	0.132	1.017 (0.962 to 1.075)	0.552	1.006 (0.955 to 1.060)	0.818		
X-11795	Hypertension	9	0.993 (0.863 to 1.142)	0.920	0.996 (0.936 to 1.061)	0.912	0.973 (0.899 to 1.054)	0.505	1.007 (0.964 to 1.052)	0.773
X-11444	Hypertension	21	0.991 (0.943 to 1.043)	0.743	1.003 (0.966 to 1.041)	0.891	1.004 (0.972 to 1.038)	0.802		
X-11858	Hypertension	11	0.963 (0.766 to 1.211)	0.754	0.980 (0.913 to 1.052)	0.585	0.997 (0.929 to 1.070)	0.933		

Exposure	Outcome	nSNP	MR Egger		Weighted median		Inverse variance weighted		MR_PRESSO	
			OR (95% CI)	pval	OR (95% CI)	pval	OR (95% CI)	pval	OR (95% CI)	pval
X-11843	Hypertension	14	1.014 (0.936 to 1.099)	0.734	0.999 (0.948 to 1.054)	0.982	1.008 (0.966 to 1.051)	0.727		
X-12127	Hypertension	14	1.012 (0.897 to 1.142)	0.846	1.042 (0.995 to 1.092)	0.077	1.050 (1.004 to 1.098)	0.033	1.031 (1.002 to 1.062)	0.059
X-12221	Hypertension	7	1.020 (0.861 to 1.208)	0.830	1.052 (0.984 to 1.125)	0.135	1.060 (0.998 to 1.125)	0.057		
X-12283	Hypertension	13	0.989 (0.863 to 1.134)	0.880	0.972 (0.922 to 1.025)	0.296	0.949 (0.901 to 1.000)	0.050		
X-12410	Hypertension	13	1.037 (0.969 to 1.109)	0.318	0.997 (0.960 to 1.035)	0.875	0.988 (0.954 to 1.023)	0.504		

Exposure	Outcome	nSNP	MR Egger		Weighted median		Inverse variance weighted		MR_PRESSO	
			OR (95% CI)	pval	OR (95% CI)	pval	OR (95% CI)	pval	OR (95% CI)	pval
X-12104	Hypertension	7	0.999 (0.813 to 1.229)	0.996	1.025 (0.954 to 1.101)	0.507	1.068 (0.971 to 1.175)	0.177	1.031 (0.978 to 1.086)	0.306
X-12193	Hypertension	17	0.960 (0.899 to 1.024)	0.234	1.002 (0.957 to 1.048)	0.942	0.996 (0.963 to 1.031)	0.832		
X-12216	Hypertension	13	1.070 (0.889 to 1.287)	0.490	0.987 (0.939 to 1.038)	0.618	0.965 (0.898 to 1.037)	0.334	0.990 (0.944 to 1.037)	0.671
X-12306	Hypertension	13	1.054 (0.939 to 1.183)	0.390	1.003 (0.949 to 1.061)	0.907	0.966 (0.909 to 1.027)	0.269	0.985 (0.937 to 1.035)	0.561
X-12100	Hypertension	12	0.866 (0.633 to 1.183)	0.387	0.977 (0.927 to 1.030)	0.394	1.105 (0.968 to 1.261)	0.139	1.014 (0.983 to 1.047)	0.411

Exposure	Outcome	nSNP	MR Egger		Weighted median		Inverse variance weighted		MR_PRESSO	
			OR (95% CI)	pval	OR (95% CI)	pval	OR (95% CI)	pval	OR (95% CI)	pval
X-12261	Hypertension	7	0.953 (0.829 to 1.096)	0.528	0.981 (0.930 to 1.035)	0.488	1.001 (0.941 to 1.066)	0.963	0.983 (0.932 to 1.036)	0.549
X-11880	Hypertension	17	0.970 (0.888 to 1.059)	0.505	0.962 (0.915 to 1.012)	0.135	0.963 (0.928 to 0.999)	0.042		
X-12117	Hypertension	25	1.054 (1.007 to 1.103)	0.033	1.032 (1.002 to 1.063)	0.034	1.023 (0.993 to 1.053)	0.132		
X-12407	Hypertension	7	1.120 (0.978 to 1.283)	0.162	1.047 (0.977 to 1.122)	0.195	1.053 (0.999 to 1.111)	0.056		
X-12101	Hypertension	13	0.858 (0.778 to 0.946)	0.010	0.946 (0.899 to 0.997)	0.036	0.994 (0.931 to 1.062)	0.865	0.972 (0.924 to 1.022)	0.292

Exposure	Outcome	nSNP	MR Egger		Weighted median		Inverse variance weighted		MR_PRESSO	
			OR (95% CI)	pval	OR (95% CI)	pval	OR (95% CI)	pval	OR (95% CI)	pval
X-12026	Hypertension	19	0.931 (0.854 to 1.016)	0.128	0.998 (0.950 to 1.048)	0.923	0.997 (0.954 to 1.042)	0.890		
X-12411	Hypertension	9	0.976 (0.916 to 1.039)	0.469	0.988 (0.950 to 1.027)	0.536	0.982 (0.942 to 1.023)	0.374		
X-12013	Hypertension	12	0.922 (0.817 to 1.042)	0.223	1.017 (0.961 to 1.076)	0.557	1.005 (0.958 to 1.055)	0.839		
X-12007	Hypertension	9	0.989 (0.917 to 1.067)	0.790	0.995 (0.943 to 1.049)	0.849	0.983 (0.945 to 1.023)	0.409		
X-12462	Hypertension	7	0.961 (0.879 to 1.051)	0.422	1.004 (0.938 to 1.075)	0.905	1.005 (0.955 to 1.056)	0.861		

Exposure	Outcome	nSNP	MR Egger		Weighted median		Inverse variance weighted		MR_PRESSO	
			OR (95% CI)	pval	OR (95% CI)	pval	OR (95% CI)	pval	OR (95% CI)	pval
X-12714	Hypertension	9	0.942 (0.824 to 1.078)	0.416	1.029 (0.977 to 1.084)	0.282	1.006 (0.950 to 1.065)	0.837	1.025 (0.953 to 1.104)	0.527
X-12740	Hypertension	10	0.960 (0.863 to 1.068)	0.472	1.007 (0.948 to 1.069)	0.820	1.020 (0.971 to 1.070)	0.435		
X-12730	Hypertension	11	1.098 (0.998 to 1.207)	0.087	1.012 (0.950 to 1.078)	0.712	1.025 (0.964 to 1.089)	0.435	1.042 (0.991 to 1.097)	0.146
X-12701	Hypertension	8	0.922 (0.802 to 1.061)	0.303	0.996 (0.945 to 1.050)	0.894	0.997 (0.956 to 1.039)	0.873		
X-12816	Hypertension	18	0.987 (0.926 to 1.052)	0.692	1.016 (0.976 to 1.058)	0.431	1.021 (0.985 to 1.059)	0.254		

Exposure	Outcome	nSNP	MR Egger		Weighted median		Inverse variance weighted		MR_PRESSO	
			OR (95% CI)	pval	OR (95% CI)	pval	OR (95% CI)	pval	OR (95% CI)	pval
X-12731	Hypertension	17	1.000 (0.873 to 1.145)	0.997	0.976 (0.930 to 1.025)	0.329	0.964 (0.919 to 1.011)	0.134	0.976 (0.935 to 1.018)	0.275
X-12707	Hypertension	16	0.957 (0.910 to 1.006)	0.109	0.988 (0.953 to 1.025)	0.529	0.978 (0.946 to 1.012)	0.207		
X-12729	Hypertension	16	1.019 (0.995 to 1.044)	0.150	1.015 (0.990 to 1.040)	0.241	1.013 (0.995 to 1.032)	0.162		
X-12830	Hypertension	16	0.911 (0.859 to 0.966)	0.008	0.976 (0.938 to 1.015)	0.222	0.985 (0.950 to 1.022)	0.423		
X-12818	Hypertension	7	1.058 (0.922 to 1.214)	0.459	1.004 (0.944 to 1.068)	0.891	0.990 (0.932 to 1.053)	0.755		

Exposure	Outcome	nSNP	MR Egger		Weighted median		Inverse variance weighted		MR_PRESSO	
			OR (95% CI)	pval	OR (95% CI)	pval	OR (95% CI)	pval	OR (95% CI)	pval
X-12812	Hypertension	10	0.944 (0.849 to 1.050)	0.321	1.000 (0.939 to 1.066)	0.989	1.025 (0.975 to 1.077)	0.330		
X-12822	Hypertension	9	0.975 (0.860 to 1.104)	0.697	1.037 (0.996 to 1.080)	0.081	1.063 (1.005 to 1.124)	0.033		
X-12738	Hypertension	9	1.100 (1.005 to 1.205)	0.077	1.025 (0.965 to 1.088)	0.421	0.977 (0.925 to 1.033)	0.416		
X-12544	Hypertension	13	0.978 (0.907 to 1.055)	0.573	1.012 (0.958 to 1.070)	0.659	1.011 (0.972 to 1.053)	0.581		
X-12680	Hypertension	8	1.155 (0.938 to 1.421)	0.223	0.963 (0.899 to 1.032)	0.288	0.957 (0.897 to 1.021)	0.184		

Exposure	Outcome	nSNP	MR Egger		Weighted median		Inverse variance weighted		MR_PRESSO	
			OR (95% CI)	pval	OR (95% CI)	pval	OR (95% CI)	pval	OR (95% CI)	pval
X-12906	Hypertension	8	1.014 (0.871 to 1.179)	0.867	0.952 (0.882 to 1.027)	0.206	0.961 (0.906 to 1.020)	0.191		
X-13684	Hypertension	20	0.994 (0.929 to 1.063)	0.855	0.997 (0.960 to 1.035)	0.864	0.987 (0.957 to 1.018)	0.400		
X-13507	Hypertension	5	1.018 (0.921 to 1.125)	0.756	1.001 (0.935 to 1.072)	0.975	1.009 (0.957 to 1.065)	0.733		
X-12851	Hypertension	10	1.048 (0.938 to 1.170)	0.431	1.050 (0.995 to 1.109)	0.077	1.018 (0.959 to 1.081)	0.557	1.035 (0.981 to 1.092)	0.246
X-12849	Hypertension	14	1.127 (1.054 to 1.206)	0.004	1.063 (1.008 to 1.120)	0.024	1.030 (0.986 to 1.077)	0.180		

Exposure	Outcome	nSNP	MR Egger		Weighted median		Inverse variance weighted		MR_PRESSO	
			OR (95% CI)	pval	OR (95% CI)	pval	OR (95% CI)	pval	OR (95% CI)	pval
X-12847	Hypertension	9	1.046 (0.922 to 1.187)	0.510	0.993 (0.939 to 1.049)	0.797	0.991 (0.941 to 1.043)	0.721		
X-13553	Hypertension	14	1.083 (0.904 to 1.298)	0.405	1.027 (0.956 to 1.104)	0.461	1.059 (0.969 to 1.157)	0.209	1.063 (1.000 to 1.129)	0.080
X-12839	Hypertension	15	1.017 (0.946 to 1.092)	0.660	0.995 (0.957 to 1.034)	0.804	0.985 (0.956 to 1.015)	0.334		
X-13729	Hypertension	20	0.960 (0.899 to 1.025)	0.242	0.976 (0.932 to 1.021)	0.286	0.986 (0.952 to 1.021)	0.425		
X-13007	Hypertension	18	0.961 (0.900 to 1.026)	0.247	1.003 (0.964 to 1.044)	0.878	1.005 (0.973 to 1.038)	0.771		

Exposure	Outcome	nSNP	MR Egger		Weighted median		Inverse variance weighted		MR_PRESSO	
			OR (95% CI)	pval	OR (95% CI)	pval	OR (95% CI)	pval	OR (95% CI)	pval
X-13844	Hypertension	8	0.927 (0.815 to 1.055)	0.296	0.964 (0.893 to 1.040)	0.342	0.975 (0.922 to 1.032)	0.382		
X-13728	Hypertension	11	0.996 (0.855 to 1.161)	0.960	0.970 (0.908 to 1.037)	0.370	0.995 (0.942 to 1.050)	0.842		
X-12844	Hypertension	21	0.987 (0.866 to 1.126)	0.847	0.986 (0.944 to 1.030)	0.525	0.971 (0.926 to 1.018)	0.217	0.997 (0.961 to 1.035)	0.893
X-13695	Hypertension	9	1.024 (0.943 to 1.112)	0.593	1.006 (0.951 to 1.063)	0.846	1.012 (0.969 to 1.056)	0.593		
X-13723	Hypertension	7	1.021 (0.922 to 1.131)	0.702	0.966 (0.908 to 1.029)	0.286	0.968 (0.923 to 1.016)	0.187		

Exposure	Outcome	nSNP	MR Egger		Weighted median		Inverse variance weighted		MR_PRESSO	
			OR (95% CI)	pval	OR (95% CI)	pval	OR (95% CI)	pval	OR (95% CI)	pval
X-14056	Hypertension	7	0.943 (0.819 to 1.085)	0.447	0.970 (0.902 to 1.043)	0.413	0.991 (0.918 to 1.070)	0.820		
X-13866	Hypertension	15	0.955 (0.810 to 1.126)	0.594	1.025 (0.966 to 1.087)	0.411	1.033 (0.966 to 1.104)	0.340	1.004 (0.955 to 1.056)	0.885
X-17146	Hypertension	9	0.950 (0.842 to 1.072)	0.434	1.042 (0.970 to 1.119)	0.257	1.050 (0.994 to 1.109)	0.082		
X-16397	Hypertension	10	1.108 (0.889 to 1.381)	0.390	0.988 (0.925 to 1.056)	0.726	0.990 (0.932 to 1.050)	0.733		
X-17335	Hypertension	7	0.990 (0.850 to 1.154)	0.906	1.034 (0.958 to 1.117)	0.393	0.989 (0.911 to 1.075)	0.800		

Exposure	Outcome	nSNP	MR Egger		Weighted median		Inverse variance weighted		MR_PRESSO	
			OR (95% CI)	pval	OR (95% CI)	pval	OR (95% CI)	pval	OR (95% CI)	pval
X-16964	Hypertension	9	1.068 (0.971 to 1.173)	0.218	0.998 (0.939 to 1.060)	0.941	0.988 (0.944 to 1.035)	0.618		
X-16935	Hypertension	14	0.998 (0.935 to 1.066)	0.964	0.998 (0.961 to 1.036)	0.912	1.001 (0.968 to 1.035)	0.972	1.016 (0.984 to 1.048)	0.358
X-15486	Hypertension	6	0.968 (0.827 to 1.133)	0.709	0.961 (0.893 to 1.034)	0.288	0.945 (0.876 to 1.018)	0.138		
X-16580	Hypertension	10	0.867 (0.729 to 1.031)	0.145	0.964 (0.904 to 1.027)	0.255	0.965 (0.914 to 1.018)	0.192		
X-17325	Hypertension	11	0.960 (0.817 to 1.129)	0.634	0.964 (0.910 to 1.022)	0.221	0.967 (0.921 to 1.015)	0.172		

Exposure	Outcome	nSNP	MR Egger		Weighted median		Inverse variance weighted		MR_PRESSO	
			OR (95% CI)	pval	OR (95% CI)	pval	OR (95% CI)	pval	OR (95% CI)	pval
X-17328	Hypertension	10	1.021 (0.861 to 1.213)	0.814	0.992 (0.936 to 1.051)	0.785	0.965 (0.925 to 1.007)	0.101		
X-16087	Hypertension	11	1.002 (0.940 to 1.068)	0.958	0.973 (0.939 to 1.008)	0.126	0.986 (0.958 to 1.014)	0.315		
X-17010	Hypertension	10	1.043 (0.922 to 1.180)	0.526	1.005 (0.953 to 1.060)	0.851	0.983 (0.934 to 1.035)	0.523		
X-14939	Hypertension	10	1.009 (0.952 to 1.070)	0.768	1.018 (0.980 to 1.058)	0.365	1.007 (0.980 to 1.035)	0.616		
X-15461	Hypertension	11	0.946 (0.846 to 1.058)	0.357	0.984 (0.925 to 1.046)	0.600	0.996 (0.950 to 1.043)	0.851		

Exposure	Outcome	nSNP	MR Egger		Weighted median		Inverse variance weighted		MR_PRESSO	
			OR (95% CI)	pval	OR (95% CI)	pval	OR (95% CI)	pval	OR (95% CI)	pval
X-15523	Hypertension	56	1.000 (0.993 to 1.007)	0.995	0.998 (0.991 to 1.005)	0.598	1.000 (0.995 to 1.006)	0.904		
X-15728	Hypertension	17	1.061 (0.942 to 1.194)	0.346	1.032 (0.980 to 1.088)	0.234	1.006 (0.958 to 1.056)	0.821		
X-15503	Hypertension	13	0.836 (0.632 to 1.106)	0.235	1.002 (0.959 to 1.047)	0.936	1.017 (0.906 to 1.141)	0.776	0.976 (0.949 to 1.004)	0.126
X-16124	Hypertension	19	1.041 (1.004 to 1.079)	0.044	1.030 (1.001 to 1.061)	0.043	1.020 (0.992 to 1.048)	0.158	1.025 (1.002 to 1.048)	0.050
X-17438	Hypertension	11	1.044 (0.998 to 1.091)	0.091	1.029 (0.988 to 1.071)	0.170	1.014 (0.985 to 1.044)	0.348		

Exposure	Outcome	nSNP	MR Egger		Weighted median		Inverse variance weighted		MR_PRESSO	
			OR (95% CI)	pval	OR (95% CI)	pval	OR (95% CI)	pval	OR (95% CI)	pval
X-17354	Hypertension	6	0.962 (0.825 to 1.121)	0.645	0.998 (0.945 to 1.054)	0.945	1.003 (0.958 to 1.050)	0.893		
X-17654	Hypertension	14	0.961 (0.922 to 1.002)	0.084	0.974 (0.945 to 1.005)	0.097	0.976 (0.953 to 0.999)	0.040		
X-17676	Hypertension	21	1.036 (0.975 to 1.102)	0.264	1.019 (0.983 to 1.056)	0.304	0.992 (0.953 to 1.032)	0.694	1.008 (0.983 to 1.034)	0.535
X-17367	Hypertension	16	0.922 (0.835 to 1.019)	0.134	0.946 (0.907 to 0.987)	0.010	0.954 (0.917 to 0.992)	0.018	0.964 (0.930 to 0.999)	0.066
X-17357	Hypertension	16	0.986 (0.876 to 1.108)	0.812	0.937 (0.898 to 0.977)	0.002	0.963 (0.916 to 1.013)	0.142	0.946 (0.906 to 0.988)	0.027

Exposure	Outcome	nSNP	MR Egger		Weighted median		Inverse variance weighted		MR_PRESSO	
			OR (95% CI)	pval	OR (95% CI)	pval	OR (95% CI)	pval	OR (95% CI)	pval
X-18886	Hypertension	8	0.964 (0.822 to 1.131)	0.671	0.985 (0.924 to 1.050)	0.640	0.984 (0.912 to 1.061)	0.675	1.006 (0.946 to 1.069)	0.864
X-18887	Hypertension	7	0.860 (0.710 to 1.041)	0.181	0.982 (0.911 to 1.059)	0.639	0.968 (0.909 to 1.030)	0.304		
X-17653	Hypertension	16	0.948 (0.891 to 1.007)	0.106	0.952 (0.920 to 0.984)	0.004	0.972 (0.944 to 1.002)	0.068		
X-17346	Hypertension	14	0.961 (0.904 to 1.021)	0.223	0.981 (0.933 to 1.031)	0.449	0.992 (0.955 to 1.029)	0.656		
X-17690	Hypertension	20	1.127 (1.026 to 1.237)	0.023	0.997 (0.952 to 1.045)	0.913	1.014 (0.970 to 1.060)	0.540	0.981 (0.945 to 1.018)	0.321

Exposure	Outcome	nSNP	MR Egger		Weighted median		Inverse variance weighted		MR_PRESSO	
			OR (95% CI)	pval	OR (95% CI)	pval	OR (95% CI)	pval	OR (95% CI)	pval
X-18345	Hypertension	15	1.029 (0.979 to 1.082)	0.273	1.047 (1.005 to 1.091)	0.029	1.034 (1.003 to 1.066)	0.030		
X-17685	Hypertension	13	1.059 (0.953 to 1.178)	0.309	1.009 (0.948 to 1.073)	0.787	1.009 (0.960 to 1.060)	0.723		
X-18888	Hypertension	13	1.008 (0.946 to 1.074)	0.815	1.010 (0.968 to 1.053)	0.653	1.017 (0.980 to 1.055)	0.376		
X-18901	Hypertension	15	1.007 (0.939 to 1.081)	0.838	0.982 (0.934 to 1.033)	0.488	0.957 (0.916 to 1.000)	0.052		
X-17351	Hypertension	12	0.855 (0.723 to 1.011)	0.098	0.966 (0.915 to 1.021)	0.219	0.967 (0.919 to 1.017)	0.195	0.950 (0.909 to 0.993)	0.047

Exposure	Outcome	nSNP	MR Egger		Weighted median		Inverse variance weighted		MR_PRESSO	
			OR (95% CI)	pval	OR (95% CI)	pval	OR (95% CI)	pval	OR (95% CI)	pval
X-18779	Hypertension	5	1.025 (0.892 to 1.176)	0.753	1.055 (0.966 to 1.153)	0.234	1.077 (1.007 to 1.153)	0.031		
X-21286	Hypertension	18	1.105 (0.945 to 1.292)	0.231	1.010 (0.960 to 1.063)	0.703	1.001 (0.951 to 1.053)	0.969	1.014 (0.970 to 1.060)	0.547
X-21355	Hypertension	6	0.992 (0.848 to 1.161)	0.923	1.039 (0.964 to 1.119)	0.316	1.035 (0.975 to 1.100)	0.259		
X-21319	Hypertension	8	1.026 (0.843 to 1.247)	0.809	1.049 (0.976 to 1.128)	0.192	1.050 (0.993 to 1.110)	0.088		
X-21258	Hypertension	15	0.940 (0.890 to 0.992)	0.042	0.958 (0.918 to 0.998)	0.042	0.981 (0.950 to 1.012)	0.227		

Exposure	Outcome	nSNP	MR Egger		Weighted median		Inverse variance weighted		MR_PRESSO	
			OR (95% CI)	pval	OR (95% CI)	pval	OR (95% CI)	pval	OR (95% CI)	pval
X-21364	Hypertension	12	0.956 (0.841 to 1.087)	0.510	0.962 (0.909 to 1.018)	0.176	0.956 (0.911 to 1.002)	0.061		
X-21353	Hypertension	12	0.996 (0.979 to 1.013)	0.623	0.998 (0.985 to 1.011)	0.750	0.998 (0.983 to 1.012)	0.734		
X-19438	Hypertension	13	1.046 (0.912 to 1.199)	0.532	1.003 (0.942 to 1.069)	0.916	1.016 (0.951 to 1.086)	0.636	0.979 (0.929 to 1.030)	0.432
X-18913	Hypertension	10	0.955 (0.842 to 1.084)	0.498	0.978 (0.927 to 1.033)	0.428	0.997 (0.939 to 1.060)	0.935	0.977 (0.938 to 1.018)	0.296
X-18921	Hypertension	16	1.000 (0.945 to 1.059)	0.990	0.986 (0.958 to 1.016)	0.362	0.998 (0.969 to 1.028)	0.901		

Exposure	Outcome	nSNP	MR Egger		Weighted median		Inverse variance weighted		MR_PRESSO	
			OR (95% CI)	pval	OR (95% CI)	pval	OR (95% CI)	pval	OR (95% CI)	pval
X-21339	Hypertension	12	0.975 (0.885 to 1.074)	0.621	0.946 (0.898 to 0.996)	0.034	0.967 (0.923 to 1.013)	0.159		
X-19299	Hypertension	9	0.964 (0.895 to 1.037)	0.353	0.991 (0.940 to 1.045)	0.741	0.974 (0.938 to 1.011)	0.171		
X-18935	Hypertension	18	1.038 (0.977 to 1.102)	0.245	1.019 (0.983 to 1.055)	0.302	1.020 (0.994 to 1.047)	0.137		
X-21310	Hypertension	12	0.915 (0.787 to 1.064)	0.277	1.021 (0.966 to 1.080)	0.458	1.012 (0.943 to 1.087)	0.736	1.005 (0.962 to 1.050)	0.828
X-21283	Hypertension	11	0.993 (0.932 to 1.057)	0.824	1.006 (0.980 to 1.033)	0.659	1.013 (0.970 to 1.057)	0.561	1.002 (0.978 to 1.027)	0.856

Exposure	Outcome	nSNP	MR Egger		Weighted median		Inverse variance weighted		MR_PRESSO	
			OR (95% CI)	pval	OR (95% CI)	pval	OR (95% CI)	pval	OR (95% CI)	pval
X-21285	Hypertension	17	0.963 (0.909 to 1.021)	0.222	0.982 (0.947 to 1.019)	0.341	1.003 (0.972 to 1.035)	0.846		
X-21312	Hypertension	12	0.948 (0.814 to 1.103)	0.501	1.023 (0.976 to 1.071)	0.349	1.029 (0.967 to 1.095)	0.367	0.996 (0.954 to 1.041)	0.871
X-18922	Hypertension	20	0.997 (0.941 to 1.055)	0.907	0.950 (0.914 to 0.988)	0.011	0.957 (0.932 to 0.982)	8.67e-04		
X-21442	Hypertension	8	1.096 (0.837 to 1.435)	0.530	1.042 (0.980 to 1.109)	0.187	1.004 (0.940 to 1.073)	0.899	1.024 (0.970 to 1.081)	0.428
X-21796	Hypertension	14	1.008 (0.827 to 1.229)	0.937	0.995 (0.940 to 1.054)	0.875	1.016 (0.956 to 1.080)	0.608	1.000 (0.947 to 1.055)	0.993

Exposure	Outcome	nSNP	MR Egger		Weighted median		Inverse variance weighted		MR_PRESSO	
			OR (95% CI)	pval	OR (95% CI)	pval	OR (95% CI)	pval	OR (95% CI)	pval
X-21441	Hypertension	21	0.989 (0.969 to 1.008)	0.271	0.993 (0.975 to 1.010)	0.413	0.997 (0.981 to 1.013)	0.684		
X-21752	Hypertension	11	0.926 (0.788 to 1.088)	0.375	0.990 (0.925 to 1.060)	0.771	1.013 (0.950 to 1.080)	0.695		
X-21807	Hypertension	10	0.943 (0.906 to 0.981)	0.019	0.945 (0.907 to 0.984)	0.007	0.958 (0.932 to 0.985)	0.002		
X-21736	Hypertension	20	1.008 (0.920 to 1.105)	0.864	1.053 (1.001 to 1.108)	0.046	1.055 (1.005 to 1.107)	0.029	1.037 (0.999 to 1.077)	0.069
X-21829	Hypertension	18	1.023 (0.942 to 1.111)	0.594	0.992 (0.950 to 1.037)	0.727	1.022 (0.985 to 1.061)	0.254		

Exposure	Outcome	nSNP	MR Egger		Weighted median		Inverse variance weighted		MR_PRESSO	
			OR (95% CI)	pval	OR (95% CI)	pval	OR (95% CI)	pval	OR (95% CI)	pval
X-21471	Hypertension	19	0.964 (0.927 to 1.003)	0.092	0.966 (0.941 to 0.992)	0.012	0.976 (0.953 to 0.999)	0.041		
X-21733	Hypertension	20	1.016 (0.933 to 1.107)	0.713	0.992 (0.951 to 1.036)	0.727	1.007 (0.974 to 1.042)	0.664		
X-21742	Hypertension	7	1.023 (0.915 to 1.145)	0.706	1.042 (0.973 to 1.117)	0.237	1.010 (0.952 to 1.071)	0.736		
X-21821	Hypertension	8	1.065 (0.905 to 1.253)	0.475	1.016 (0.950 to 1.086)	0.650	0.963 (0.880 to 1.053)	0.408	0.978 (0.930 to 1.027)	0.411
X-21470	Hypertension	15	0.945 (0.887 to 1.008)	0.111	0.966 (0.934 to 0.999)	0.043	0.994 (0.961 to 1.029)	0.749	0.985 (0.955 to 1.017)	0.379

Exposure	Outcome	nSNP	MR Egger		Weighted median		Inverse variance weighted		MR_PRESSO	
			OR (95% CI)	pval	OR (95% CI)	pval	OR (95% CI)	pval	OR (95% CI)	pval
X-21383	Hypertension	14	0.959 (0.870 to 1.058)	0.424	0.955 (0.904 to 1.009)	0.101	0.945 (0.904 to 0.989)	0.015		
X-21607	Hypertension	18	1.025 (0.937 to 1.120)	0.600	1.004 (0.970 to 1.040)	0.820	1.020 (0.980 to 1.062)	0.338	1.004 (0.977 to 1.032)	0.754
X-21834	Hypertension	13	0.963 (0.885 to 1.047)	0.396	0.968 (0.921 to 1.017)	0.192	0.977 (0.942 to 1.014)	0.222		
X-21467	Hypertension	23	0.962 (0.934 to 0.990)	0.016	0.974 (0.951 to 0.997)	0.028	0.978 (0.960 to 0.996)	0.018		
X-22776	Hypertension	9	1.004 (0.902 to 1.119)	0.940	0.973 (0.909 to 1.043)	0.442	1.002 (0.952 to 1.056)	0.925		

Exposure	Outcome	nSNP	MR Egger		Weighted median		Inverse variance weighted		MR_PRESSO	
			OR (95% CI)	pval	OR (95% CI)	pval	OR (95% CI)	pval	OR (95% CI)	pval
X-23655	Hypertension	14	1.023 (0.917 to 1.142)	0.689	1.035 (0.982 to 1.090)	0.200	1.018 (0.978 to 1.059)	0.386		
X-22834	Hypertension	11	1.065 (0.939 to 1.208)	0.353	1.046 (0.989 to 1.107)	0.115	1.034 (0.983 to 1.089)	0.198		
X-22771	Hypertension	6	1.533 (0.952 to 2.469)	0.154	1.018 (0.937 to 1.105)	0.678	1.025 (0.904 to 1.163)	0.702	0.972 (0.902 to 1.048)	0.504
X-23639	Hypertension	12	1.027 (0.806 to 1.309)	0.835	0.967 (0.907 to 1.030)	0.296	0.980 (0.906 to 1.060)	0.616	0.982 (0.923 to 1.045)	0.584
X-23648	Hypertension	8	1.009 (0.853 to 1.194)	0.918	0.989 (0.917 to 1.067)	0.781	0.967 (0.904 to 1.035)	0.338		

Exposure	Outcome	nSNP	MR Egger		Weighted median		Inverse variance weighted		MR_PRESSO	
			OR (95% CI)	pval	OR (95% CI)	pval	OR (95% CI)	pval	OR (95% CI)	pval
X-23654	Hypertension	20	0.981 (0.937 to 1.028)	0.430	0.996 (0.958 to 1.035)	0.824	1.020 (0.991 to 1.050)	0.186		
X-22520	Hypertension	9	0.881 (0.755 to 1.028)	0.151	1.015 (0.958 to 1.076)	0.609	1.008 (0.952 to 1.068)	0.784	1.043 (0.987 to 1.102)	0.181
X-23641	Hypertension	22	1.033 (0.963 to 1.108)	0.377	1.014 (0.980 to 1.050)	0.422	1.007 (0.981 to 1.032)	0.611		
X-23276	Hypertension	14	1.078 (1.012 to 1.148)	0.039	1.027 (0.980 to 1.077)	0.263	1.019 (0.987 to 1.051)	0.246		
X-21845	Hypertension	14	0.975 (0.912 to 1.044)	0.484	1.005 (0.970 to 1.040)	0.800	0.999 (0.962 to 1.038)	0.962	1.006 (0.974 to 1.039)	0.711

Exposure	Outcome	nSNP	MR Egger		Weighted median		Inverse variance weighted		MR_PRESSO	
			OR (95% CI)	pval	OR (95% CI)	pval	OR (95% CI)	pval	OR (95% CI)	pval
X-22509	Hypertension	10	0.998 (0.912 to 1.092)	0.961	1.004 (0.953 to 1.058)	0.890	0.993 (0.957 to 1.031)	0.727		
X-23644	Hypertension	8	1.132 (0.906 to 1.413)	0.317	1.001 (0.917 to 1.092)	0.988	0.974 (0.879 to 1.079)	0.612	0.940 (0.868 to 1.018)	0.187
X-22162	Hypertension	21	0.991 (0.944 to 1.040)	0.712	0.996 (0.963 to 1.029)	0.796	1.008 (0.974 to 1.044)	0.636	1.016 (0.989 to 1.044)	0.271
X-23587	Hypertension	9	1.035 (0.914 to 1.173)	0.604	1.003 (0.934 to 1.076)	0.943	0.984 (0.927 to 1.044)	0.588		
X-23593	Hypertension	12	0.880 (0.814 to 0.950)	0.009	1.000 (0.948 to 1.055)	0.994	1.009 (0.960 to 1.061)	0.714	0.995 (0.955 to 1.035)	0.794

Exposure	Outcome	nSNP	MR Egger		Weighted median		Inverse variance weighted		MR_PRESSO	
			OR (95% CI)	pval	OR (95% CI)	pval	OR (95% CI)	pval	OR (95% CI)	pval
X-23636	Hypertension	7	0.857 (0.703 to 1.045)	0.187	0.949 (0.879 to 1.025)	0.180	0.992 (0.924 to 1.065)	0.832		
X-23678	Hypertension	15	0.960 (0.878 to 1.049)	0.386	0.993 (0.948 to 1.039)	0.759	0.962 (0.921 to 1.006)	0.089	0.963 (0.931 to 0.995)	0.042
X-23680	Hypertension	14	1.016 (0.969 to 1.066)	0.525	1.003 (0.982 to 1.024)	0.790	0.997 (0.960 to 1.035)	0.867	1.004 (0.987 to 1.021)	0.680
X-23780	Hypertension	15	1.049 (0.982 to 1.121)	0.180	1.067 (1.016 to 1.122)	0.010	1.027 (0.992 to 1.063)	0.130		
X-23974	Hypertension	10	1.003 (0.828 to 1.215)	0.975	1.029 (0.965 to 1.098)	0.381	1.006 (0.945 to 1.071)	0.851	1.036 (0.978 to 1.097)	0.261

Exposure	Outcome	nSNP	MR Egger		Weighted median		Inverse variance weighted		MR_PRESSO	
			OR (95% CI)	pval	OR (95% CI)	pval	OR (95% CI)	pval	OR (95% CI)	pval
X-23659	Hypertension	17	0.986 (0.952 to 1.021)	0.446	0.990 (0.964 to 1.018)	0.489	0.988 (0.963 to 1.013)	0.340		
X-23665	Hypertension	8	1.009 (0.929 to 1.096)	0.841	0.998 (0.952 to 1.046)	0.935	0.992 (0.946 to 1.041)	0.758		
X-23782	Hypertension	11	0.995 (0.859 to 1.151)	0.943	0.961 (0.898 to 1.029)	0.254	0.984 (0.927 to 1.044)	0.599		
X-24295	Hypertension	13	0.912 (0.761 to 1.094)	0.343	0.993 (0.927 to 1.064)	0.848	1.021 (0.946 to 1.102)	0.600	1.015 (0.954 to 1.080)	0.647
X-24243	Hypertension	13	1.006 (0.892 to 1.136)	0.919	0.997 (0.945 to 1.052)	0.914	1.021 (0.968 to 1.078)	0.439	1.031 (0.986 to 1.078)	0.204

Exposure	Outcome	nSNP	MR Egger		Weighted median		Inverse variance weighted		MR_PRESSO	
			OR (95% CI)	pval	OR (95% CI)	pval	OR (95% CI)	pval	OR (95% CI)	pval
X-23739	Hypertension	13	1.084 (0.993 to 1.182)	0.098	1.051 (1.009 to 1.095)	0.016	0.990 (0.933 to 1.051)	0.752	0.979 (0.931 to 1.028)	0.416
X-23997	Hypertension	11	0.886 (0.768 to 1.023)	0.133	0.957 (0.894 to 1.025)	0.210	0.938 (0.893 to 0.985)	0.010		
X-24241	Hypertension	12	1.013 (0.907 to 1.131)	0.820	1.019 (0.963 to 1.079)	0.508	1.018 (0.976 to 1.062)	0.411		
X-24328	Hypertension	23	1.039 (0.958 to 1.126)	0.367	1.001 (0.958 to 1.047)	0.957	1.002 (0.971 to 1.034)	0.899		
X-24456	Hypertension	17	1.075 (0.996 to 1.161)	0.084	1.022 (0.977 to 1.069)	0.338	1.024 (0.991 to 1.059)	0.156		

Exposure	Outcome	nSNP	MR Egger		Weighted median		Inverse variance weighted		MR_PRESSO	
			OR (95% CI)	pval	OR (95% CI)	pval	OR (95% CI)	pval	OR (95% CI)	pval
X-24418	Hypertension	17	1.004 (0.937 to 1.075)	0.917	1.009 (0.977 to 1.042)	0.585	1.029 (0.996 to 1.062)	0.082	1.009 (0.987 to 1.031)	0.443
X-24344	Hypertension	9	1.146 (0.980 to 1.341)	0.132	0.996 (0.937 to 1.060)	0.911	1.007 (0.959 to 1.057)	0.786		
X-24556	Hypertension	16	0.987 (0.942 to 1.035)	0.604	0.971 (0.937 to 1.006)	0.105	0.985 (0.960 to 1.010)	0.227		
X-24337	Hypertension	16	0.992 (0.916 to 1.073)	0.836	1.025 (0.984 to 1.067)	0.243	0.977 (0.940 to 1.016)	0.250	0.977 (0.933 to 1.023)	0.348
X-24546	Hypertension	19	0.982 (0.932 to 1.034)	0.494	0.962 (0.931 to 0.994)	0.021	0.964 (0.938 to 0.990)	0.007		

Exposure	Outcome	nSNP	MR Egger		Weighted median		Inverse variance weighted		MR_PRESSO	
			OR (95% CI)	pval	OR (95% CI)	pval	OR (95% CI)	pval	OR (95% CI)	pval
X-24306	Hypertension	15	0.974 (0.907 to 1.047)	0.489	0.964 (0.917 to 1.012)	0.140	0.976 (0.938 to 1.014)	0.213		
X-24307	Hypertension	9	1.173 (1.022 to 1.348)	0.058	0.968 (0.901 to 1.039)	0.362	1.011 (0.947 to 1.079)	0.748		
X-24334	Hypertension	13	1.073 (0.923 to 1.248)	0.379	0.972 (0.923 to 1.024)	0.288	0.997 (0.938 to 1.060)	0.920	0.984 (0.944 to 1.025)	0.460
X-24518	Hypertension	15	1.028 (0.983 to 1.074)	0.246	0.991 (0.964 to 1.018)	0.508	1.003 (0.978 to 1.029)	0.800		
X-24494	Hypertension	12	0.993 (0.926 to 1.066)	0.857	0.997 (0.953 to 1.042)	0.877	0.992 (0.958 to 1.027)	0.643		

Exposure	Outcome	nSNP	MR Egger		Weighted median		Inverse variance weighted		MR_PRESSO	
			OR (95% CI)	pval	OR (95% CI)	pval	OR (95% CI)	pval	OR (95% CI)	pval
X-24544	Hypertension	19	0.998 (0.940 to 1.060)	0.958	1.005 (0.981 to 1.030)	0.682	1.003 (0.962 to 1.045)	0.901	0.991 (0.966 to 1.016)	0.488
X-24531	Hypertension	13	0.932 (0.867 to 1.002)	0.083	0.961 (0.918 to 1.006)	0.087	0.975 (0.943 to 1.008)	0.129		
X-24811	Hypertension	15	1.012 (0.935 to 1.096)	0.768	0.995 (0.952 to 1.041)	0.838	1.015 (0.977 to 1.054)	0.447		
X-24757	Hypertension	12	0.911 (0.780 to 1.063)	0.262	0.958 (0.904 to 1.015)	0.149	0.976 (0.915 to 1.040)	0.449	0.978 (0.934 to 1.024)	0.360
X-24978	Hypertension	12	1.058 (0.973 to 1.151)	0.215	1.028 (0.977 to 1.081)	0.294	0.998 (0.954 to 1.043)	0.921	0.971 (0.929 to 1.016)	0.229

Exposure	Outcome	nSNP	MR Egger		Weighted median		Inverse variance weighted		MR_PRESSO	
			OR (95% CI)	pval	OR (95% CI)	pval	OR (95% CI)	pval	OR (95% CI)	pval
X-24970	Hypertension	14	0.960 (0.796 to 1.157)	0.674	0.959 (0.903 to 1.019)	0.173	0.978 (0.927 to 1.031)	0.410		
X-24795	Hypertension	15	1.047 (0.965 to 1.137)	0.292	1.007 (0.973 to 1.042)	0.688	1.003 (0.958 to 1.049)	0.907	0.997 (0.971 to 1.024)	0.841
X-24951	Hypertension	11	0.952 (0.813 to 1.114)	0.553	0.970 (0.904 to 1.042)	0.405	0.954 (0.893 to 1.018)	0.154		
X-24728	Hypertension	16	1.091 (0.994 to 1.197)	0.088	1.032 (0.983 to 1.084)	0.202	1.028 (0.981 to 1.078)	0.249	1.047 (1.020 to 1.074)	0.004
X-24565	Hypertension	20	1.005 (0.992 to 1.019)	0.464	1.005 (0.993 to 1.018)	0.422	1.003 (0.992 to 1.014)	0.622		

Exposure	Outcome	nSNP	MR Egger		Weighted median		Inverse variance weighted		MR_PRESSO	
			OR (95% CI)	pval	OR (95% CI)	pval	OR (95% CI)	pval	OR (95% CI)	pval
X-24801	Hypertension	27	1.002 (0.938 to 1.069)	0.958	1.003 (0.965 to 1.041)	0.894	1.019 (0.985 to 1.054)	0.273	1.019 (0.990 to 1.049)	0.219
X-24736	Hypertension	21	1.015 (0.957 to 1.076)	0.628	1.018 (0.991 to 1.046)	0.186	1.024 (1.000 to 1.049)	0.055	1.029 (1.007 to 1.052)	0.017
X-24585	Hypertension	10	0.975 (0.922 to 1.030)	0.393	0.994 (0.957 to 1.033)	0.766	1.000 (0.961 to 1.040)	0.987		
X-24588	Hypertension	24	1.022 (0.965 to 1.083)	0.468	1.028 (0.993 to 1.064)	0.116	1.020 (0.995 to 1.047)	0.123		
X-25109	Hypertension	14	1.011 (0.929 to 1.101)	0.800	1.033 (0.983 to 1.085)	0.200	1.029 (0.994 to 1.065)	0.101		

Exposure	Outcome	nSNP	MR Egger		Weighted median		Inverse variance weighted		MR_PRESSO	
			OR (95% CI)	pval	OR (95% CI)	pval	OR (95% CI)	pval	OR (95% CI)	pval
X-24947	Hypertension	19	1.009 (0.963 to 1.057)	0.703	1.038 (1.016 to 1.060)	6.8e-04	1.029 (1.001 to 1.059)	0.046	1.035 (1.011 to 1.058)	0.009
X-24949	Hypertension	19	0.987 (0.930 to 1.047)	0.665	0.979 (0.946 to 1.014)	0.235	0.989 (0.959 to 1.020)	0.476	0.996 (0.970 to 1.022)	0.766
X-24812	Hypertension	16	0.980 (0.909 to 1.056)	0.598	1.030 (0.985 to 1.077)	0.201	1.023 (0.979 to 1.068)	0.317	1.009 (0.972 to 1.048)	0.642
X-25217	Hypertension	10	0.976 (0.890 to 1.069)	0.613	0.994 (0.946 to 1.044)	0.799	0.977 (0.943 to 1.014)	0.219		
X-25828	Hypertension	5	0.776 (0.524 to 1.151)	0.297	0.919 (0.803 to 1.052)	0.222	0.944 (0.816 to 1.092)	0.437		

Exposure	Outcome	nSNP	MR Egger		Weighted median		Inverse variance weighted		MR_PRESSO	
			OR (95% CI)	pval	OR (95% CI)	pval	OR (95% CI)	pval	OR (95% CI)	pval
X-25371	Hypertension	19	1.028 (0.952 to 1.109)	0.490	0.967 (0.929 to 1.006)	0.096	0.967 (0.930 to 1.007)	0.103	0.977 (0.943 to 1.012)	0.220
X-25419	Hypertension	16	1.011 (0.945 to 1.082)	0.747	0.990 (0.948 to 1.034)	0.665	1.019 (0.988 to 1.050)	0.239		
X-25265	Hypertension	16	0.962 (0.935 to 0.989)	0.016	0.965 (0.941 to 0.989)	0.005	0.972 (0.948 to 0.997)	0.026		
X-25172	Hypertension	11	1.045 (1.001 to 1.091)	0.077	1.028 (0.993 to 1.064)	0.120	1.021 (0.990 to 1.053)	0.187		
X-25957	Hypertension	9	1.097 (0.977 to 1.231)	0.161	1.042 (0.976 to 1.111)	0.217	1.030 (0.983 to 1.080)	0.215		

Exposure	Outcome	nSNP	MR Egger		Weighted median		Inverse variance weighted		MR_PRESSO	
			OR (95% CI)	pval	OR (95% CI)	pval	OR (95% CI)	pval	OR (95% CI)	pval
X-25422	Hypertension	16	1.031 (0.921 to 1.153)	0.608	0.997 (0.957 to 1.039)	0.893	0.962 (0.918 to 1.009)	0.109	0.971 (0.929 to 1.014)	0.206
X-25790	Hypertension	11	1.018 (0.821 to 1.264)	0.873	0.932 (0.872 to 0.997)	0.041	0.986 (0.926 to 1.049)	0.650		
X-25420	Hypertension	10	0.939 (0.806 to 1.095)	0.447	1.018 (0.957 to 1.082)	0.578	1.025 (0.981 to 1.071)	0.266		
X-25810	Hypertension	23	0.957 (0.888 to 1.032)	0.266	0.954 (0.910 to 1.000)	0.048	0.968 (0.931 to 1.006)	0.096	0.979 (0.944 to 1.015)	0.259
X-25433	Hypertension	16	0.968 (0.912 to 1.027)	0.300	0.965 (0.939 to 0.991)	0.009	0.970 (0.937 to 1.006)	0.099	0.969 (0.947 to 0.992)	0.020

Exposure	Outcome	nSNP	MR Egger		Weighted median		Inverse variance weighted		MR_PRESSO	
			OR (95% CI)	pval	OR (95% CI)	pval	OR (95% CI)	pval	OR (95% CI)	pval
X-25343	Hypertension	9	0.928 (0.784 to 1.097)	0.409	1.029 (0.957 to 1.106)	0.435	1.060 (0.989 to 1.136)	0.098		
X-25271	Hypertension	12	0.868 (0.711 to 1.059)	0.193	1.022 (0.955 to 1.094)	0.528	0.969 (0.908 to 1.033)	0.333		
X-25520	Hypertension	8	0.983 (0.859 to 1.127)	0.818	1.020 (0.956 to 1.089)	0.547	1.048 (0.981 to 1.120)	0.164		
X-25519	Hypertension	10	0.933 (0.854 to 1.019)	0.162	0.986 (0.926 to 1.050)	0.658	1.028 (0.964 to 1.095)	0.402		
X-26111	Hypertension	14	0.945 (0.874 to 1.021)	0.178	0.951 (0.915 to 0.989)	0.011	0.972 (0.934 to 1.011)	0.161		

Exposure	Outcome	nSNP	MR Egger		Weighted median		Inverse variance weighted		MR_PRESSO	
			OR (95% CI)	pval	OR (95% CI)	pval	OR (95% CI)	pval	OR (95% CI)	pval
X-26109	Hypertension	18	1.030 (1.004 to 1.056)	0.037	1.017 (0.998 to 1.037)	0.075	1.006 (0.985 to 1.027)	0.593		
X-26054	Hypertension	23	1.038 (1.008 to 1.068)	0.021	1.012 (0.988 to 1.036)	0.319	1.002 (0.981 to 1.024)	0.830		
Carnitine C4	Hypertension	26	0.992 (0.929 to 1.059)	0.804	1.018 (0.982 to 1.055)	0.324	1.007 (0.976 to 1.040)	0.651	0.999 (0.971 to 1.029)	0.959
Androsterone sulfate	Hypertension	24	0.996 (0.960 to 1.033)	0.825	1.000 (0.985 to 1.015)	0.991	1.008 (0.978 to 1.039)	0.605	0.995 (0.964 to 1.027)	0.754
N-acetyltyrosine	Hypertension	19	1.025 (1.002 to 1.048)	0.049	1.019 (1.001 to 1.037)	0.039	1.012 (0.996 to 1.028)	0.134		

Exposure	Outcome	nSNP	MR Egger		Weighted median		Inverse variance weighted		MR_PRESSO	
			OR (95% CI)	pval	OR (95% CI)	pval	OR (95% CI)	pval	OR (95% CI)	pval
N-acetyl-L-glutamine	Hypertension	13	1.018 (0.998 to 1.038)	0.103	1.021 (1.006 to 1.036)	0.006	1.022 (1.007 to 1.037)	0.005		
N-acetylphenylalanine	Hypertension	13	1.024 (0.994 to 1.056)	0.147	1.030 (1.010 to 1.051)	0.003	1.030 (1.009 to 1.051)	0.005		
N-acetylasparagine	Hypertension	12	1.064 (0.940 to 1.205)	0.349	1.074 (1.020 to 1.130)	0.007	1.055 (0.997 to 1.117)	0.062	1.057 (1.027 to 1.088)	0.004
N-acetylarginine	Hypertension	14	1.016 (0.993 to 1.038)	0.196	1.019 (1.006 to 1.033)	0.005	1.022 (1.004 to 1.040)	0.016		
5-acetylamino-6-formylamino-3-methyluracil	Hypertension	17	0.995 (0.977 to 1.013)	0.579	0.997 (0.983 to 1.012)	0.730	0.998 (0.985 to 1.012)	0.829		

Exposure	Outcome	nSNP	MR Egger		Weighted median		Inverse variance weighted		MR_PRESSO	
			OR (95% CI)	pval	OR (95% CI)	pval	OR (95% CI)	pval	OR (95% CI)	pval
Orotidine	Hypertension	20	1.002 (0.983 to 1.022)	0.837	1.002 (0.993 to 1.012)	0.668	1.004 (0.987 to 1.022)	0.642	1.004 (0.994 to 1.013)	0.456
3-methylcytidine	Hypertension	11	1.003 (0.982 to 1.025)	0.791	0.999 (0.984 to 1.014)	0.894	1.000 (0.985 to 1.015)	0.991		
N-acetylcitrulline	Hypertension	12	1.011 (0.983 to 1.040)	0.479	1.016 (1.003 to 1.030)	0.021	1.015 (0.994 to 1.037)	0.163		
N-acetyl-aspartyl-glutamate (naag)	Hypertension	7	0.993 (0.967 to 1.020)	0.639	1.010 (0.988 to 1.032)	0.376	1.019 (0.988 to 1.051)	0.224		
1-stearoyl-2-arachidonoyl-gpc (18:0/20:4)	Hypertension	19	0.995 (0.962 to 1.029)	0.789	0.999 (0.983 to 1.016)	0.939	0.992 (0.966 to 1.018)	0.542	0.996 (0.981 to 1.010)	0.562

Exposure	Outcome	nSNP	MR Egger		Weighted median		Inverse variance weighted		MR_PRESSO	
			OR (95% CI)	pval	OR (95% CI)	pval	OR (95% CI)	pval	OR (95% CI)	pval
Bilirubin (E,E)	Hypertension	15	1.032 (0.987 to 1.079)	0.184	1.004 (0.983 to 1.025)	0.738	1.000 (0.970 to 1.032)	0.990	1.005 (0.976 to 1.034)	0.761
Bilirubin (E,Z or Z,E)	Hypertension	13	1.010 (0.962 to 1.061)	0.687	1.001 (0.978 to 1.024)	0.954	0.988 (0.961 to 1.016)	0.386		
Androsterone glucuronide	Hypertension	20	1.003 (0.956 to 1.053)	0.897	1.009 (0.980 to 1.039)	0.545	0.998 (0.965 to 1.033)	0.916	0.999 (0.971 to 1.027)	0.931
N6-methyllysine	Hypertension	17	1.007 (0.993 to 1.022)	0.362	1.002 (0.992 to 1.012)	0.678	1.002 (0.991 to 1.014)	0.711		
N-acetyl-1-methylhistidine	Hypertension	24	1.014 (0.982 to 1.047)	0.416	1.018 (0.999 to 1.037)	0.071	1.026 (1.003 to 1.050)	0.024	1.022 (1.004 to 1.040)	0.026

Exposure	Outcome	nSNP	MR Egger		Weighted median		Inverse variance weighted		MR_PRESSO	
			OR (95% CI)	pval	OR (95% CI)	pval	OR (95% CI)	pval	OR (95% CI)	pval
N-delta-acetylornithine	Hypertension	13	0.973 (0.956 to 0.991)	0.013	0.980 (0.966 to 0.993)	0.003	0.983 (0.970 to 0.997)	0.017		
1-palmitoyl-2-arachidonoyl-gpc (16:0/20:4n6)	Hypertension	16	0.971 (0.924 to 1.020)	0.255	0.989 (0.971 to 1.006)	0.210	0.985 (0.953 to 1.018)	0.360	0.983 (0.965 to 1.001)	0.084
Glycochenodeoxycholate glucuronide (1)	Hypertension	13	0.993 (0.975 to 1.011)	0.445	0.987 (0.973 to 1.000)	0.051	0.986 (0.973 to 0.999)	0.036		
2'-o-methylcytidine	Hypertension	11	1.003 (0.972 to 1.035)	0.849	0.997 (0.980 to 1.015)	0.753	0.999 (0.977 to 1.021)	0.922		
2'-o-methyluridine	Hypertension	10	0.997 (0.972 to 1.023)	0.829	0.998 (0.981 to 1.014)	0.792	0.994 (0.977 to 1.012)	0.521		

Exposure	Outcome	nSNP	MR Egger		Weighted median		Inverse variance weighted		MR_PRESSO	
			OR (95% CI)	pval	OR (95% CI)	pval	OR (95% CI)	pval	OR (95% CI)	pval
N6,N6-dimethyllysine	Hypertension	13	0.993 (0.977 to 1.009)	0.400	0.997 (0.983 to 1.011)	0.714	0.997 (0.986 to 1.009)	0.631		
N2-acetyl,N6,N6-dimethyllysine	Hypertension	15	1.004 (0.975 to 1.033)	0.814	0.996 (0.983 to 1.010)	0.611	1.001 (0.982 to 1.020)	0.956	0.991 (0.964 to 1.019)	0.538
N2-acetyl,N6-methyllysine	Hypertension	18	1.000 (0.982 to 1.019)	0.976	1.003 (0.990 to 1.016)	0.685	1.005 (0.992 to 1.018)	0.485	0.999 (0.986 to 1.012)	0.877
Deoxycholic acid glucuronide	Hypertension	15	1.008 (0.973 to 1.044)	0.659	1.008 (0.981 to 1.035)	0.569	0.991 (0.970 to 1.011)	0.365		
Decadienedioic acid (C10:2-DC)	Hypertension	17	1.006 (0.968 to 1.045)	0.771	1.013 (0.994 to 1.033)	0.189	1.001 (0.978 to 1.025)	0.945		

Exposure	Outcome	nSNP	MR Egger		Weighted median		Inverse variance weighted		MR_PRESSO	
			OR (95% CI)	pval	OR (95% CI)	pval	OR (95% CI)	pval	OR (95% CI)	pval
Bilirubin degradation product, C17H18N2O4 (1)	Hypertension	13	1.027 (0.995 to 1.060)	0.124	1.007 (0.987 to 1.028)	0.489	0.992 (0.963 to 1.022)	0.594		
Bilirubin degradation product, C17H18N2O4 (2)	Hypertension	12	1.007 (0.960 to 1.056)	0.789	1.004 (0.983 to 1.026)	0.696	1.003 (0.976 to 1.032)	0.810		
Bilirubin degradation product, C17H18N2O4 (3)	Hypertension	12	1.012 (0.969 to 1.056)	0.606	1.001 (0.980 to 1.024)	0.896	0.994 (0.965 to 1.025)	0.716		
Bilirubin (z,z)	Hypertension	16	1.013 (0.969 to 1.058)	0.588	1.005 (0.986 to 1.024)	0.631	0.995 (0.969 to 1.021)	0.682	0.997 (0.974 to 1.021)	0.829
Ethylmalonate	Hypertension	24	0.974 (0.935 to 1.015)	0.222	0.975 (0.957 to 0.994)	0.008	0.986 (0.960 to 1.012)	0.279	1.020 (0.981 to 1.060)	0.325

Exposure	Outcome	nSNP	MR Egger		Weighted median		Inverse variance weighted		MR_PRESSO	
			OR (95% CI)	pval	OR (95% CI)	pval	OR (95% CI)	pval	OR (95% CI)	pval
Biliverdin	Hypertension	17	1.006 (0.970 to 1.043)	0.750	1.003 (0.984 to 1.023)	0.736	0.993 (0.969 to 1.019)	0.611		
Glycine	Hypertension	13	0.970 (0.944 to 0.995)	0.042	0.973 (0.953 to 0.993)	0.007	0.974 (0.956 to 0.993)	0.007		
X-12112	Hypertension	21	0.994 (0.973 to 1.015)	0.582	0.994 (0.983 to 1.006)	0.354	0.993 (0.977 to 1.008)	0.338	0.994 (0.981 to 1.008)	0.409
X-12798	Hypertension	19	0.999 (0.953 to 1.047)	0.958	0.996 (0.973 to 1.020)	0.757	0.987 (0.958 to 1.017)	0.407	0.991 (0.964 to 1.019)	0.542
X-13431	Hypertension	17	1.024 (0.940 to 1.117)	0.593	1.022 (0.981 to 1.064)	0.298	1.027 (0.991 to 1.064)	0.140		

Exposure	Outcome	nSNP	MR Egger		Weighted median		Inverse variance weighted		MR_PRESSO	
			OR (95% CI)	pval	OR (95% CI)	pval	OR (95% CI)	pval	OR (95% CI)	pval
X-19141	Hypertension	16	1.028 (0.990 to 1.068)	0.177	0.992 (0.966 to 1.020)	0.572	0.988 (0.966 to 1.012)	0.329		
Glutamine	Hypertension	7	1.519 (0.466 to 4.952)	0.519	0.933 (0.528 to 1.652)	0.813	0.737 (0.341 to 1.592)	0.438		
Tryptophan	Hypertension	108	2.389 (0.232 to 24.618)	0.466	0.984 (0.676 to 1.433)	0.933	0.856 (0.613 to 1.195)	0.360	0.808 (0.602 to 1.086)	0.161
Histidine	Hypertension	6	0.279 (0.053 to 1.462)	0.205	0.801 (0.327 to 1.960)	0.627	1.270 (0.448 to 3.600)	0.653		
Leucine	Hypertension	120	1.581 (0.691 to 3.618)	0.281	1.348 (0.876 to 2.074)	0.175	1.314 (0.980 to 1.760)	0.068	1.213 (0.929 to 1.584)	0.159

Exposure	Outcome	nSNP	MR Egger		Weighted median		Inverse variance weighted		MR_PRESSO	
			OR (95% CI)	pval	OR (95% CI)	pval	OR (95% CI)	pval	OR (95% CI)	pval
Cholesterol	Hypertension	30	1.864 (0.441 to 7.877)	0.404	1.302 (0.822 to 2.064)	0.261	1.240 (0.759 to 2.027)	0.390	1.227 (0.848 to 1.776)	0.287
Phenylalanine	Hypertension	4	0.615 (0.012 to 31.635)	0.831	0.716 (0.219 to 2.335)	0.580	1.053 (0.340 to 3.264)	0.929		
Creatinine	Hypertension	14	0.258 (0.038 to 1.770)	0.193	0.472 (0.213 to 1.045)	0.064	0.700 (0.302 to 1.624)	0.406	0.758 (0.334 to 1.723)	0.522
Lactate	Hypertension	5	1.406 (0.165 to 11.972)	0.776	1.462 (0.867 to 2.466)	0.154	1.527 (0.837 to 2.785)	0.167		
3-hydroxybutyrate (BHBA)	Hypertension	5	1.012 (0.909 to 1.127)	0.838	1.060 (0.972 to 1.156)	0.189	1.065 (0.997 to 1.138)	0.060		

Exposure	Outcome	nSNP	MR Egger		Weighted median		Inverse variance weighted		MR_PRESSO	
			OR (95% CI)	pval	OR (95% CI)	pval	OR (95% CI)	pval	OR (95% CI)	pval
Arabinose	Hypertension	4	0.904 (0.760 to 1.075)	0.371	1.067 (0.944 to 1.206)	0.299	1.086 (0.912 to 1.294)	0.354		
Fructose	Hypertension	2					0.948 (0.595 to 1.509)	0.821		
Mannose	Hypertension	8	0.313 (0.190 to 0.516)	0.004	0.611 (0.478 to 0.781)	8.26e-05	0.685 (0.456 to 1.029)	0.068	0.656 (0.457 to 0.940)	0.083
Pyruvate	Hypertension	11	1.349 (0.530 to 3.435)	0.545	1.039 (0.793 to 1.361)	0.782	1.067 (0.778 to 1.462)	0.688	1.070 (0.823 to 1.392)	0.627
Uridine	Hypertension	15	0.301 (0.081 to 1.114)	0.095	1.163 (0.680 to 1.991)	0.581	0.995 (0.675 to 1.466)	0.979		

Exposure	Outcome	nSNP	MR Egger		Weighted median		Inverse variance weighted		MR_PRESSO	
			OR (95% CI)	pval	OR (95% CI)	pval	OR (95% CI)	pval	OR (95% CI)	pval
Linoleate (18:2n6)	Hypertension	9	2.728 (0.447 to 16.630)	0.313	1.179 (0.739 to 1.881)	0.490	1.117 (0.739 to 1.689)	0.599		
Allantoin	Hypertension	9	1.009 (0.706 to 1.443)	0.962	0.982 (0.785 to 1.228)	0.873	0.937 (0.784 to 1.121)	0.477		
Arachidonate (20:4n6)	Hypertension	11	0.825 (0.595 to 1.144)	0.279	0.853 (0.690 to 1.055)	0.142	0.847 (0.696 to 1.030)	0.096		
Deoxycholate	Hypertension	6	0.558 (0.221 to 1.405)	0.283	0.860 (0.721 to 1.026)	0.094	0.935 (0.788 to 1.109)	0.441		
Margarate (17:0)	Hypertension	6	2.970 (0.367 to 24.065)	0.365	1.352 (0.914 to 2.000)	0.131	1.119 (0.642 to 1.951)	0.692	1.446 (1.157 to 1.807)	0.032

Exposure	Outcome	nSNP	MR Egger		Weighted median		Inverse variance weighted		MR_PRESSO	
			OR (95% CI)	pval	OR (95% CI)	pval	OR (95% CI)	pval	OR (95% CI)	pval
Inosine	Hypertension	5	1.298 (0.948 to 1.777)	0.203	1.016 (0.914 to 1.130)	0.765	1.045 (0.965 to 1.132)	0.279		
Isoleucine	Hypertension	11	0.678 (0.239 to 1.922)	0.483	1.078 (0.610 to 1.903)	0.796	1.227 (0.805 to 1.870)	0.341		
Threonine	Hypertension	13	0.917 (0.151 to 5.577)	0.927	1.065 (0.669 to 1.695)	0.792	1.047 (0.636 to 1.724)	0.857	1.208 (0.777 to 1.879)	0.419
Tyrosine	Hypertension	21	0.634 (0.155 to 2.584)	0.532	0.903 (0.576 to 1.416)	0.656	0.836 (0.584 to 1.196)	0.326		
Lysine	Hypertension	15	0.104 (0.011 to 1.001)	0.072	1.265 (0.699 to 2.287)	0.438	1.390 (0.829 to 2.330)	0.212		

Exposure	Outcome	nSNP	MR Egger		Weighted median		Inverse variance weighted		MR_PRESSO	
			OR (95% CI)	pval	OR (95% CI)	pval	OR (95% CI)	pval	OR (95% CI)	pval
Methionine	Hypertension	13	0.833 (0.071 to 9.794)	0.887	0.563 (0.264 to 1.200)	0.137	0.521 (0.252 to 1.077)	0.079	0.558 (0.335 to 0.928)	0.048
Malate	Hypertension	10	0.589 (0.080 to 4.331)	0.617	0.737 (0.512 to 1.062)	0.101	0.803 (0.610 to 1.055)	0.115		
Palmitate (16:0)	Hypertension	19	0.689 (0.301 to 1.577)	0.391	0.904 (0.594 to 1.376)	0.638	1.111 (0.767 to 1.610)	0.577	1.283 (0.950 to 1.734)	0.123
Nonadecanoate (19:0)	Hypertension	7	4.269 (1.120 to 16.270)	0.087	1.545 (0.960 to 2.489)	0.073	1.448 (0.979 to 2.142)	0.064		
Stearate (18:0)	Hypertension	26	1.299 (0.557 to 3.029)	0.551	1.242 (0.878 to 1.756)	0.220	1.211 (0.899 to 1.631)	0.208	1.427 (1.174 to 1.734)	0.002

Exposure	Outcome	nSNP	MR Egger		Weighted median		Inverse variance weighted		MR_PRESSO	
			OR (95% CI)	pval	OR (95% CI)	pval	OR (95% CI)	pval	OR (95% CI)	pval
Oleate (18:1n9)	Hypertension	11	5.130 (0.324 to 81.241)	0.276	1.410 (0.882 to 2.255)	0.151	1.256 (0.802 to 1.967)	0.319	1.094 (0.741 to 1.615)	0.663
Pentadecanoate (15:0)	Hypertension	10	0.615 (0.176 to 2.152)	0.469	0.889 (0.574 to 1.375)	0.596	0.793 (0.503 to 1.249)	0.317	1.068 (0.733 to 1.557)	0.740
Myristate (14:0)	Hypertension	12	1.198 (0.681 to 2.105)	0.545	1.083 (0.811 to 1.445)	0.589	0.964 (0.706 to 1.315)	0.817	1.066 (0.838 to 1.356)	0.616
Pipecolate	Hypertension	8	1.766 (0.735 to 4.243)	0.250	1.012 (0.761 to 1.345)	0.937	0.902 (0.665 to 1.225)	0.509	1.003 (0.817 to 1.231)	0.979
Ornithine	Hypertension	6	1.758 (0.407 to 7.588)	0.491	1.111 (0.681 to 1.813)	0.672	0.959 (0.577 to 1.593)	0.872		

Exposure	Outcome	nSNP	MR Egger		Weighted median		Inverse variance weighted		MR_PRESSO	
			OR (95% CI)	pval	OR (95% CI)	pval	OR (95% CI)	pval	OR (95% CI)	pval
5-oxoproline	Hypertension	16	1.161 (0.589 to 2.287)	0.674	1.220 (0.910 to 1.637)	0.184	1.124 (0.745 to 1.696)	0.578	1.043 (0.745 to 1.460)	0.811
Pantothenate	Hypertension	15	0.982 (0.620 to 1.558)	0.941	0.905 (0.696 to 1.176)	0.453	0.981 (0.740 to 1.302)	0.897	0.881 (0.727 to 1.068)	0.219
4-acetamidobutanoate	Hypertension	16	1.027 (0.396 to 2.667)	0.957	1.075 (0.751 to 1.539)	0.692	1.283 (0.833 to 1.975)	0.257	1.218 (0.886 to 1.674)	0.248
Alpha-tocopherol	Hypertension	5	0.793 (0.329 to 1.908)	0.640	0.920 (0.668 to 1.265)	0.607	0.965 (0.743 to 1.254)	0.791		
Citrate	Hypertension	21	0.921 (0.418 to 2.029)	0.840	1.137 (0.800 to 1.616)	0.474	1.009 (0.758 to 1.343)	0.951		

Exposure	Outcome	nSNP	MR Egger		Weighted median		Inverse variance weighted		MR_PRESSO	
			OR (95% CI)	pval	OR (95% CI)	pval	OR (95% CI)	pval	OR (95% CI)	pval
Glycerate	Hypertension	9	2.319 (0.887 to 6.066)	0.130	0.830 (0.520 to 1.325)	0.435	0.943 (0.641 to 1.387)	0.765		
Guanosine	Hypertension	8	0.782 (0.557 to 1.099)	0.206	0.965 (0.844 to 1.104)	0.606	0.987 (0.865 to 1.125)	0.841		
N-acetylalanine	Hypertension	12	3558.841 (0.119 to 106346342.245)	0.151	1.507 (0.703 to 3.232)	0.292	1.788 (0.923 to 3.464)	0.085		
Urate	Hypertension	9	1.550 (0.842 to 2.853)	0.202	1.171 (0.810 to 1.692)	0.402	1.002 (0.724 to 1.387)	0.989		
Ursodeoxycholate	Hypertension	4	4.047 (0.956 to 17.137)	0.198	1.070 (0.815 to 1.405)	0.624	0.955 (0.643 to 1.416)	0.818	1.182 (0.926 to 1.508)	0.312

Exposure	Outcome	nSNP	MR Egger		Weighted median		Inverse variance weighted		MR_PRESSO	
			OR (95% CI)	pval	OR (95% CI)	pval	OR (95% CI)	pval	OR (95% CI)	pval
Arginine	Hypertension	10	2.422 (0.627 to 9.353)	0.235	1.359 (0.912 to 2.026)	0.132	1.187 (0.749 to 1.881)	0.466	1.374 (0.989 to 1.910)	0.095
Ascorbate (Vitamin C)	Hypertension	10	0.897 (0.514 to 1.565)	0.711	1.102 (0.986 to 1.232)	0.086	1.038 (0.906 to 1.190)	0.590	1.097 (0.979 to 1.230)	0.151
Heptanoate (7:0)	Hypertension	20	1.126 (0.494 to 2.567)	0.781	1.069 (0.746 to 1.533)	0.715	0.887 (0.657 to 1.199)	0.436		
Laurate (12:0)	Hypertension	27	1.890 (0.608 to 5.875)	0.282	1.433 (1.029 to 1.996)	0.033	1.373 (0.943 to 1.998)	0.098	1.195 (0.904 to 1.579)	0.223
Valine	Hypertension	6	1.177 (0.448 to 3.090)	0.757	1.632 (0.879 to 3.031)	0.121	2.099 (0.905 to 4.868)	0.084		

Exposure	Outcome	nSNP	MR Egger		Weighted median		Inverse variance weighted		MR_PRESSO	
			OR (95% CI)	pval	OR (95% CI)	pval	OR (95% CI)	pval	OR (95% CI)	pval
Urea	Hypertension	7	0.490 (0.137 to 1.753)	0.323	0.998 (0.621 to 1.602)	0.992	1.192 (0.701 to 2.028)	0.517		
Cortisol	Hypertension	5	1.003 (0.359 to 2.807)	0.995	1.023 (0.715 to 1.464)	0.900	1.114 (0.685 to 1.812)	0.663	0.935 (0.649 to 1.348)	0.744
Cortisone	Hypertension	24	1.116 (0.632 to 1.971)	0.709	1.044 (0.716 to 1.521)	0.823	0.972 (0.730 to 1.293)	0.844		
Proline	Hypertension	20	1.245 (0.690 to 2.246)	0.476	1.154 (0.842 to 1.583)	0.372	1.019 (0.735 to 1.411)	0.912	1.197 (0.908 to 1.578)	0.219
Citrulline	Hypertension	23	1.419 (0.597 to 3.373)	0.437	1.068 (0.728 to 1.566)	0.737	1.168 (0.851 to 1.605)	0.336		

Exposure	Outcome	nSNP	MR Egger		Weighted median		Inverse variance weighted		MR_PRESSO	
			OR (95% CI)	pval	OR (95% CI)	pval	OR (95% CI)	pval	OR (95% CI)	pval
Biliverdin	Hypertension	12	1.040 (0.850 to 1.272)	0.711	1.005 (0.913 to 1.107)	0.913	0.969 (0.849 to 1.106)	0.642		
Serotonin (5HT)	Hypertension	11	0.748 (0.508 to 1.100)	0.174	0.968 (0.782 to 1.199)	0.768	1.100 (0.858 to 1.411)	0.453	1.027 (0.877 to 1.203)	0.747
Gamma-glutamylglutamine	Hypertension	16	0.896 (0.498 to 1.612)	0.719	0.795 (0.592 to 1.069)	0.129	0.661 (0.466 to 0.938)	0.021	0.710 (0.514 to 0.981)	0.057
Gamma-glutamyltyrosine	Hypertension	23	1.117 (0.292 to 4.272)	0.873	0.779 (0.535 to 1.135)	0.194	0.889 (0.611 to 1.293)	0.539	0.825 (0.586 to 1.163)	0.285
Hypoxanthine	Hypertension	12	0.807 (0.398 to 1.635)	0.564	0.981 (0.732 to 1.315)	0.897	1.099 (0.837 to 1.443)	0.495		

Exposure	Outcome	nSNP	MR Egger		Weighted median		Inverse variance weighted		MR_PRESSO	
			OR (95% CI)	pval	OR (95% CI)	pval	OR (95% CI)	pval	OR (95% CI)	pval
Betaine	Hypertension	13	0.803 (0.377 to 1.707)	0.579	0.894 (0.639 to 1.251)	0.513	1.001 (0.775 to 1.294)	0.992		
Xanthine	Hypertension	3	3.271 (0.866 to 12.352)	0.331	1.004 (0.670 to 1.506)	0.983	0.880 (0.501 to 1.545)	0.656		
Phosphate	Hypertension	2					0.813 (0.368 to 1.796)	0.609		
3-methoxytyrosine	Hypertension	9	0.453 (0.038 to 5.410)	0.551	0.868 (0.551 to 1.368)	0.543	0.705 (0.382 to 1.301)	0.263	0.704 (0.443 to 1.119)	0.188
Pelargonate (9:0)	Hypertension	20	0.668 (0.158 to 2.824)	0.590	0.847 (0.546 to 1.313)	0.457	0.879 (0.631 to 1.226)	0.448		

Exposure	Outcome	nSNP	MR Egger		Weighted median		Inverse variance weighted		MR_PRESSO	
			OR (95% CI)	pval	OR (95% CI)	pval	OR (95% CI)	pval	OR (95% CI)	pval
Undecanoate (11:0)	Hypertension	21	0.731 (0.368 to 1.451)	0.381	1.069 (0.699 to 1.636)	0.759	1.014 (0.736 to 1.396)	0.933		
Beta-hydroxyisovalerate	Hypertension	17	1.062 (0.608 to 1.857)	0.835	1.027 (0.796 to 1.324)	0.839	1.074 (0.863 to 1.338)	0.521		
Taurodeoxycholate	Hypertension	8	0.787 (0.599 to 1.035)	0.137	0.944 (0.851 to 1.047)	0.273	0.903 (0.833 to 0.979)	0.013		
X-02973	Hypertension	16	0.384 (0.017 to 8.463)	0.554	1.342 (0.731 to 2.463)	0.343	1.244 (0.628 to 2.467)	0.531	0.931 (0.582 to 1.488)	0.770
X-03003	Hypertension	3	0.000 (0.000 to 2062303673221.323)	0.740	2.437 (0.978 to 6.073)	0.056	2.182 (0.941 to 5.057)	0.069		

Exposure	Outcome	nSNP	MR Egger		Weighted median		Inverse variance weighted		MR_PRESSO	
			OR (95% CI)	pval	OR (95% CI)	pval	OR (95% CI)	pval	OR (95% CI)	pval
X-03088	Hypertension	7	1.074 (0.817 to 1.411)	0.631	1.066 (0.845 to 1.344)	0.591	1.056 (0.870 to 1.281)	0.582		
X-03094	Hypertension	16	2.245 (0.714 to 7.058)	0.188	1.166 (0.807 to 1.683)	0.413	1.427 (0.876 to 2.322)	0.153	1.153 (0.928 to 1.434)	0.225
Glycerol	Hypertension	12	0.938 (0.565 to 1.558)	0.811	1.048 (0.823 to 1.335)	0.705	1.094 (0.820 to 1.459)	0.542	1.043 (0.817 to 1.331)	0.743
Kynurenine	Hypertension	26	1.357 (0.502 to 3.668)	0.553	0.982 (0.718 to 1.343)	0.910	1.386 (0.838 to 2.292)	0.203	1.070 (0.861 to 1.330)	0.550
Mannitol	Hypertension	6	0.904 (0.706 to 1.156)	0.466	1.026 (0.929 to 1.132)	0.615	1.026 (0.947 to 1.111)	0.532		

Exposure	Outcome	nSNP	MR Egger		Weighted median		Inverse variance weighted		MR_PRESSO	
			OR (95% CI)	pval	OR (95% CI)	pval	OR (95% CI)	pval	OR (95% CI)	pval
Glycerol 3-phosphate (G3P)	Hypertension	6	0.736 (0.220 to 2.457)	0.644	0.925 (0.592 to 1.445)	0.732	1.043 (0.613 to 1.777)	0.876		
Acetylphosphate	Hypertension	10	0.343 (0.005 to 25.524)	0.640	1.421 (0.666 to 3.036)	0.364	2.102 (0.816 to 5.416)	0.124	1.514 (0.706 to 3.246)	0.318
Carnitine	Hypertension	125	2.210 (0.832 to 5.867)	0.114	1.102 (0.749 to 1.621)	0.621	1.198 (0.891 to 1.612)	0.232	1.043 (0.811 to 1.342)	0.745
Choline	Hypertension	14	2.200 (0.396 to 12.223)	0.385	0.856 (0.447 to 1.640)	0.639	0.905 (0.498 to 1.646)	0.744		
N-acetylornithine	Hypertension	10	0.873 (0.765 to 0.997)	0.081	0.896 (0.828 to 0.969)	0.006	0.910 (0.827 to 1.001)	0.053		

Exposure	Outcome	nSNP	MR Egger		Weighted median		Inverse variance weighted		MR_PRESSO	
			OR (95% CI)	pval	OR (95% CI)	pval	OR (95% CI)	pval	OR (95% CI)	pval
N1-methyladenosine	Hypertension	3	0.022 (0.000 to 1.024)	0.302	0.248 (0.075 to 0.816)	0.022	0.228 (0.075 to 0.693)	0.009		
3-methyl-2-oxovalerate	Hypertension	22	1.123 (0.415 to 3.038)	0.822	1.294 (0.878 to 1.906)	0.193	1.210 (0.812 to 1.804)	0.348	1.204 (0.852 to 1.702)	0.307
3-methylhistidine	Hypertension	4	1.083 (0.771 to 1.522)	0.691	1.090 (0.955 to 1.243)	0.202	1.059 (0.953 to 1.177)	0.286		
3-phenylpropionate (hydrocinnamate)	Hypertension	4	0.869 (0.627 to 1.205)	0.489	1.002 (0.837 to 1.199)	0.985	1.023 (0.813 to 1.286)	0.848		
Phenylacetate	Hypertension	5	0.426 (0.161 to 1.129)	0.185	0.710 (0.479 to 1.053)	0.088	0.735 (0.539 to 1.002)	0.052		

Exposure	Outcome	nSNP	MR Egger		Weighted median		Inverse variance weighted		MR_PRESSO	
			OR (95% CI)	pval	OR (95% CI)	pval	OR (95% CI)	pval	OR (95% CI)	pval
Glycerophosphorylcholine (GPC)	Hypertension	8	0.913 (0.751 to 1.111)	0.398	0.913 (0.792 to 1.052)	0.207	0.907 (0.789 to 1.043)	0.172		
Aspartate	Hypertension	3	0.574 (0.274 to 1.201)	0.379	0.858 (0.608 to 1.211)	0.383	0.825 (0.619 to 1.101)	0.192		
X-04357	Hypertension	2					1.027 (0.712 to 1.482)	0.886		
X-04494	Hypertension	8	0.746 (0.247 to 2.252)	0.621	0.828 (0.536 to 1.279)	0.395	1.030 (0.723 to 1.466)	0.871		
X-04495	Hypertension	7	0.663 (0.366 to 1.200)	0.233	0.908 (0.678 to 1.216)	0.518	0.906 (0.731 to 1.123)	0.367		

Exposure	Outcome	nSNP	MR Egger		Weighted median		Inverse variance weighted		MR_PRESSO	
			OR (95% CI)	pval	OR (95% CI)	pval	OR (95% CI)	pval	OR (95% CI)	pval
X-04498	Hypertension	14	1.364 (0.746 to 2.494)	0.333	1.212 (0.903 to 1.628)	0.201	1.235 (0.949 to 1.606)	0.116	1.314 (1.076 to 1.604)	0.020
X-04499--3,4-dihydroxybutyrate	Hypertension	12	4.562 (1.002 to 20.760)	0.078	0.913 (0.621 to 1.341)	0.642	0.966 (0.655 to 1.424)	0.861	0.896 (0.677 to 1.185)	0.461
X-04500	Hypertension	5	0.966 (0.754 to 1.237)	0.800	1.055 (0.957 to 1.163)	0.283	1.108 (0.972 to 1.264)	0.126		
Dihomo-linoleate (20:2n6)	Hypertension	3	12.494 (1.898 to 82.246)	0.232	1.443 (0.825 to 2.521)	0.198	1.709 (0.911 to 3.208)	0.095		
X-18601	Hypertension	13	1.026 (0.732 to 1.437)	0.885	0.972 (0.837 to 1.128)	0.706	0.988 (0.834 to 1.170)	0.887	0.919 (0.810 to 1.043)	0.221

Exposure	Outcome	nSNP	MR Egger		Weighted median		Inverse variance weighted		MR_PRESSO	
			OR (95% CI)	pval	OR (95% CI)	pval	OR (95% CI)	pval	OR (95% CI)	pval
2-hydroxystearate	Hypertension	22	0.952 (0.558 to 1.624)	0.859	1.013 (0.759 to 1.354)	0.928	1.088 (0.789 to 1.499)	0.608	1.147 (0.851 to 1.544)	0.378
X-05426	Hypertension	7	0.726 (0.414 to 1.272)	0.314	0.860 (0.716 to 1.034)	0.109	0.940 (0.773 to 1.144)	0.538		
Indolelactate	Hypertension	11	1.060 (0.558 to 2.015)	0.862	1.116 (0.867 to 1.436)	0.394	1.132 (0.804 to 1.595)	0.478	1.052 (0.830 to 1.333)	0.685
Glycylvaline	Hypertension	3	0.510 (0.147 to 1.765)	0.480	1.052 (0.827 to 1.338)	0.681	1.003 (0.721 to 1.395)	0.987		
Gamma-glutamylleucine	Hypertension	17	0.842 (0.433 to 1.640)	0.621	0.887 (0.612 to 1.286)	0.528	1.029 (0.746 to 1.420)	0.861		

Exposure	Outcome	nSNP	MR Egger		Weighted median		Inverse variance weighted		MR_PRESSO	
			OR (95% CI)	pval	OR (95% CI)	pval	OR (95% CI)	pval	OR (95% CI)	pval
Eicosapentaenoate (EPA; 20:5n3)	Hypertension	7	0.833 (0.468 to 1.484)	0.562	0.935 (0.727 to 1.203)	0.601	1.010 (0.825 to 1.237)	0.923		
Estrone 3-sulfate	Hypertension	8	1.002 (0.952 to 1.054)	0.948	0.991 (0.950 to 1.034)	0.668	0.982 (0.947 to 1.017)	0.314		
Glycocholate	Hypertension	5	1.129 (0.849 to 1.500)	0.466	1.023 (0.871 to 1.201)	0.784	1.049 (0.920 to 1.195)	0.477		
Glycodeoxycholate	Hypertension	5	1.796 (0.859 to 3.758)	0.218	0.974 (0.884 to 1.074)	0.602	1.009 (0.857 to 1.186)	0.918	0.984 (0.913 to 1.060)	0.715
Taurochenodeoxycholate	Hypertension	6	0.924 (0.747 to 1.144)	0.509	0.952 (0.820 to 1.107)	0.524	0.998 (0.891 to 1.118)	0.971		

Exposure	Outcome	nSNP	MR Egger		Weighted median		Inverse variance weighted		MR_PRESSO	
			OR (95% CI)	pval	OR (95% CI)	pval	OR (95% CI)	pval	OR (95% CI)	pval
Taurocholate	Hypertension	11	0.949 (0.846 to 1.064)	0.393	1.006 (0.924 to 1.096)	0.883	1.017 (0.956 to 1.081)	0.591		
X-05907	Hypertension	9	1.287 (0.656 to 2.526)	0.487	1.205 (0.831 to 1.749)	0.326	1.260 (0.953 to 1.666)	0.105		
Docosahexaenoate (DHA; 22:6n3)	Hypertension	1								
1-stearoylglycerophosphoinositol	Hypertension	4	0.599 (0.356 to 1.007)	0.193	0.882 (0.669 to 1.164)	0.377	0.800 (0.579 to 1.105)	0.176		
X-06226	Hypertension	15	1.105 (0.596 to 2.049)	0.757	1.148 (0.780 to 1.689)	0.484	1.348 (1.015 to 1.790)	0.039		
X-06246	Hypertension	13	2.026 (0.470 to 8.732)	0.364	1.480 (1.042	0.028	1.351 (0.924	0.120	1.225 (0.886	0.245

Exposure	Outcome	nSNP	MR Egger		Weighted median		Inverse variance weighted		MR_PRESSO	
			OR (95% CI)	pval	OR (95% CI)	pval	OR (95% CI)	pval	OR (95% CI)	pval
					to 2.101)		to 1.975)		to 1.694)	
X-06267	Hypertension	7	1.361 (0.081 to 22.936)	0.839	0.788 (0.467 to 1.330)	0.373	1.084 (0.587 to 2.000)	0.797	0.879 (0.504 to 1.535)	0.670
X-06307	Hypertension	5	0.559 (0.244 to 1.282)	0.263	0.996 (0.787 to 1.260)	0.973	1.137 (0.785 to 1.647)	0.498		
X-06350	Hypertension	7	0.624 (0.342 to 1.139)	0.185	0.759 (0.529 to 1.089)	0.135	0.851 (0.646 to 1.120)	0.250		
X-06351	Hypertension	4	0.989 (0.609 to 1.607)	0.970	0.823 (0.584 to 1.161)	0.268	0.808 (0.612 to 1.067)	0.133		

Exposure	Outcome	nSNP	MR Egger		Weighted median		Inverse variance weighted		MR_PRESSO	
			OR (95% CI)	pval	OR (95% CI)	pval	OR (95% CI)	pval	OR (95% CI)	pval
Myo-inositol	Hypertension	27	0.672 (0.365 to 1.239)	0.215	1.032 (0.774 to 1.376)	0.832	1.217 (0.880 to 1.684)	0.235	1.125 (0.865 to 1.462)	0.389
Glucose	Hypertension	26	1.310 (0.633 to 2.710)	0.474	1.076 (0.732 to 1.581)	0.710	0.864 (0.601 to 1.243)	0.431		
1,5-anhydroglucitol (1,5-AG)	Hypertension	17	1.696 (0.833 to 3.451)	0.166	1.565 (1.197 to 2.045)	0.001	1.395 (1.023 to 1.902)	0.035	1.438 (1.125 to 1.839)	0.012
2-hydroxybutyrate (AHB)	Hypertension	9	1.196 (0.585 to 2.445)	0.639	1.260 (0.928 to 1.709)	0.138	1.334 (0.966 to 1.842)	0.080		
3-methyl-2-oxobutyrate	Hypertension	11	0.231 (0.047 to 1.134)	0.105	0.923 (0.530 to 1.608)	0.777	1.049 (0.640 to 1.719)	0.848	0.870 (0.554 to 1.365)	0.559

Exposure	Outcome	nSNP	MR Egger		Weighted median		Inverse variance weighted		MR_PRESSO	
			OR (95% CI)	pval	OR (95% CI)	pval	OR (95% CI)	pval	OR (95% CI)	pval
1,6-anhydroglucose	Hypertension	4	0.861 (0.644 to 1.151)	0.418	0.945 (0.849 to 1.053)	0.309	0.979 (0.859 to 1.114)	0.743		
1-palmitoylglycerol (1-monopalmitin)	Hypertension	7	0.078 (0.002 to 2.988)	0.228	1.010 (0.632 to 1.613)	0.968	1.233 (0.672 to 2.261)	0.499	0.919 (0.677 to 1.247)	0.610
1-oleoylglycerol (1-monoolein)	Hypertension	10	2.687 (1.016 to 7.106)	0.082	1.086 (0.891 to 1.322)	0.414	1.206 (0.936 to 1.553)	0.147	1.037 (0.917 to 1.174)	0.576
1-stearoylglycerol (1-monostearin)	Hypertension	15	2.950 (1.216 to 7.159)	0.033	1.243 (0.906 to 1.707)	0.178	1.327 (0.988 to 1.784)	0.060	1.286 (1.044 to 1.585)	0.036
X-08402	Hypertension	12	0.698 (0.497 to 0.979)	0.064	0.795 (0.633 to 0.997)	0.047	0.843 (0.688 to 1.032)	0.097		

Exposure	Outcome	nSNP	MR Egger		Weighted median		Inverse variance weighted		MR_PRESSO	
			OR (95% CI)	pval	OR (95% CI)	pval	OR (95% CI)	pval	OR (95% CI)	pval
2-hydroxyisobutyrate	Hypertension	12	0.616 (0.216 to 1.754)	0.385	1.099 (0.790 to 1.529)	0.576	1.348 (0.899 to 2.020)	0.148	1.312 (1.030 to 1.672)	0.056
X-08766	Hypertension	11	0.505 (0.280 to 0.909)	0.049	1.101 (0.812 to 1.495)	0.535	0.990 (0.691 to 1.418)	0.957	1.219 (1.065 to 1.395)	0.021
4-methyl-2-oxopentanoate	Hypertension	7	0.303 (0.037 to 2.467)	0.315	1.134 (0.602 to 2.138)	0.696	1.018 (0.527 to 1.969)	0.957	0.947 (0.509 to 1.762)	0.872
Phenyllactate (PLA)	Hypertension	12	0.698 (0.297 to 1.640)	0.428	0.735 (0.518 to 1.041)	0.083	0.864 (0.647 to 1.153)	0.320		
Homocitrulline	Hypertension	4	0.816 (0.093 to 7.129)	0.871	1.072 (0.777 to 1.480)	0.671	1.001 (0.642 to 1.561)	0.997		

Exposure	Outcome	nSNP	MR Egger		Weighted median		Inverse variance weighted		MR_PRESSO	
			OR (95% CI)	pval	OR (95% CI)	pval	OR (95% CI)	pval	OR (95% CI)	pval
Aspartylphenylalanine	Hypertension	3	1.608 (0.799 to 3.237)	0.410	1.070 (0.858 to 1.333)	0.549	1.083 (0.894 to 1.312)	0.416		
Levulinate (4-oxovalerate)	Hypertension	23	7.286 (0.922 to 57.577)	0.074	0.752 (0.511 to 1.106)	0.148	0.807 (0.573 to 1.135)	0.217		
Palmitoylcarnitine	Hypertension	4	0.245 (0.038 to 1.595)	0.279	0.979 (0.593 to 1.616)	0.935	1.424 (0.800 to 2.535)	0.229		
X-08988	Hypertension	21	0.600 (0.366 to 0.985)	0.058	0.884 (0.659 to 1.185)	0.410	0.890 (0.692 to 1.144)	0.362		
X-09026	Hypertension	11	0.518 (0.125 to 2.142)	0.387	0.735 (0.516 to 1.048)	0.089	0.821 (0.573 to 1.175)	0.281		

Exposure	Outcome	nSNP	MR Egger		Weighted median		Inverse variance weighted		MR_PRESSO	
			OR (95% CI)	pval	OR (95% CI)	pval	OR (95% CI)	pval	OR (95% CI)	pval
X-09108	Hypertension	6	4.079 (0.000 to 3270753523.098)	0.900	0.862 (0.450 to 1.651)	0.654	0.869 (0.412 to 1.831)	0.711	1.139 (0.544 to 2.382)	0.748
Cholate	Hypertension	2					0.843 (0.709 to 1.003)	0.054		
X-09706	Hypertension	11	2.194 (0.756 to 6.369)	0.182	1.149 (0.773 to 1.707)	0.492	1.155 (0.812 to 1.644)	0.422		
X-10395	Hypertension	20	0.979 (0.793 to 1.209)	0.844	0.888 (0.736 to 1.070)	0.212	0.878 (0.745 to 1.034)	0.120		
X-10429	Hypertension	15	0.892 (0.606 to 1.313)	0.574	0.783 (0.600 to 1.021)	0.071	0.789 (0.658 to 0.945)	0.010		

Exposure	Outcome	nSNP	MR Egger		Weighted median		Inverse variance weighted		MR_PRESSO	
			OR (95% CI)	pval	OR (95% CI)	pval	OR (95% CI)	pval	OR (95% CI)	pval
X-10500	Hypertension	24	2.163 (0.658 to 7.104)	0.217	1.319 (0.860 to 2.021)	0.204	0.991 (0.697 to 1.410)	0.960		
X-10506	Hypertension	6	0.664 (0.333 to 1.325)	0.310	0.946 (0.650 to 1.377)	0.772	1.056 (0.715 to 1.560)	0.784		
X-10510	Hypertension	16	0.695 (0.297 to 1.625)	0.415	0.756 (0.568 to 1.007)	0.056	0.819 (0.582 to 1.154)	0.254	0.834 (0.655 to 1.062)	0.165
1-linoleoylglycerol (1-monolinolein)	Hypertension	4	2.092 (1.195 to 3.662)	0.123	1.095 (0.888 to 1.351)	0.396	1.131 (0.909 to 1.408)	0.270		
Indoleacetate	Hypertension	12	0.962 (0.700 to 1.322)	0.815	0.999 (0.796 to 1.253)	0.991	0.964 (0.790 to 1.175)	0.716		

Exposure	Outcome	nSNP	MR Egger		Weighted median		Inverse variance weighted		MR_PRESSO	
			OR (95% CI)	pval	OR (95% CI)	pval	OR (95% CI)	pval	OR (95% CI)	pval
Hyodeoxycholate	Hypertension	10	1.005 (0.895 to 1.130)	0.930	1.074 (0.978 to 1.179)	0.135	1.039 (0.973 to 1.110)	0.256		
3-indoxyl sulfate	Hypertension	2					0.841 (0.601 to 1.176)	0.312		
N-acetylglycine	Hypertension	7	0.878 (0.574 to 1.343)	0.575	0.907 (0.799 to 1.029)	0.130	0.929 (0.767 to 1.125)	0.450	0.868 (0.638 to 1.180)	0.433
Bilirubin (Z,Z)	Hypertension	6	1.002 (0.903 to 1.113)	0.966	1.006 (0.946 to 1.071)	0.845	1.014 (0.959 to 1.073)	0.621		
Creatine	Hypertension	5	1.108 (0.516 to 2.380)	0.810	0.783 (0.624 to 0.984)	0.036	0.733 (0.544 to 0.988)	0.041		

Exposure	Outcome	nSNP	MR Egger		Weighted median		Inverse variance weighted		MR_PRESSO	
			OR (95% CI)	pval	OR (95% CI)	pval	OR (95% CI)	pval	OR (95% CI)	pval
Erythrose	Hypertension	3	0.929 (0.606 to 1.425)	0.793	1.008 (0.790 to 1.285)	0.950	1.029 (0.827 to 1.280)	0.796		
Threonate	Hypertension	9	0.903 (0.697 to 1.170)	0.466	0.945 (0.778 to 1.149)	0.572	0.951 (0.812 to 1.114)	0.533		
X-10810	Hypertension	12	1.067 (0.774 to 1.470)	0.702	1.101 (0.934 to 1.297)	0.252	1.072 (0.862 to 1.334)	0.533		
Cysteine	Hypertension	11	1.215 (0.934 to 1.581)	0.181	1.167 (0.925 to 1.472)	0.192	1.080 (0.902 to 1.292)	0.403		
Pyroglutamylglycine	Hypertension	2					0.877 (0.738 to 1.043)	0.137		

Exposure	Outcome	nSNP	MR Egger		Weighted median		Inverse variance weighted		MR_PRESSO	
			OR (95% CI)	pval	OR (95% CI)	pval	OR (95% CI)	pval	OR (95% CI)	pval
N-(2-furoyl)glycine	Hypertension	17	1.021 (0.934 to 1.116)	0.658	1.053 (1.004 to 1.104)	0.032	1.024 (0.981 to 1.068)	0.274	1.040 (1.000 to 1.081)	0.072
DSGEGDFXAEGGGVR*	Hypertension	8	0.809 (0.399 to 1.640)	0.578	0.891 (0.731 to 1.088)	0.257	0.937 (0.802 to 1.096)	0.416		
Pyridoxate	Hypertension	6	2.111 (1.669 to 2.671)	0.003	1.342 (0.989 to 1.822)	0.059	1.383 (1.014 to 1.887)	0.041		
Androsterone sulfate	Hypertension	15	0.999 (0.921 to 1.085)	0.989	1.007 (0.964 to 1.051)	0.765	1.009 (0.951 to 1.071)	0.756		
3-carboxy-4-methyl-5-propyl-2-furanpropanoate (CMPF)	Hypertension	10	1.228 (1.040 to 1.450)	0.041	1.017 (0.925 to 1.118)	0.731	0.989 (0.898 to 1.089)	0.817		

Exposure	Outcome	nSNP	MR Egger		Weighted median		Inverse variance weighted		MR_PRESSO	
			OR (95% CI)	pval	OR (95% CI)	pval	OR (95% CI)	pval	OR (95% CI)	pval
3-(4-hydroxyphenyl)lactate	Hypertension	13	0.353 (0.127 to 0.978)	0.070	0.694 (0.469 to 1.025)	0.067	0.876 (0.563 to 1.364)	0.558	0.764 (0.534 to 1.092)	0.167
Acetylcarnitine	Hypertension	7	1.111 (0.453 to 2.729)	0.827	1.045 (0.768 to 1.422)	0.779	1.176 (0.846 to 1.635)	0.334		
Serine	Hypertension	18	0.883 (0.370 to 2.106)	0.782	0.940 (0.641 to 1.381)	0.754	0.953 (0.672 to 1.353)	0.790		
Trans-4-hydroxyproline	Hypertension	2					0.813 (0.440 to 1.502)	0.509		
Glutamate	Hypertension	2					0.914 (0.300 to 2.784)	0.874		

Exposure	Outcome	nSNP	MR Egger		Weighted median		Inverse variance weighted		MR_PRESSO	
			OR (95% CI)	pval	OR (95% CI)	pval	OR (95% CI)	pval	OR (95% CI)	pval
Hexanoylcarnitine	Hypertension	11	1.170 (0.829 to 1.650)	0.395	1.170 (0.989 to 1.385)	0.068	1.163 (0.980 to 1.380)	0.084		
Glycine	Hypertension	12	0.796 (0.568 to 1.116)	0.216	0.821 (0.706 to 0.954)	0.010	0.889 (0.706 to 1.118)	0.314		
Alanine	Hypertension	23	2.285 (0.909 to 5.742)	0.093	1.132 (0.791 to 1.619)	0.498	1.207 (0.907 to 1.607)	0.197		
Glycochenodeoxycholate	Hypertension	9	1.017 (0.863 to 1.200)	0.844	0.996 (0.901 to 1.102)	0.942	0.980 (0.907 to 1.058)	0.604		
2-aminobutyrate	Hypertension	30	0.842 (0.322 to 2.203)	0.728	0.978 (0.697 to 1.371)	0.897	1.099 (0.807 to 1.495)	0.550	1.028 (0.777 to 1.360)	0.850

Exposure	Outcome	nSNP	MR Egger		Weighted median		Inverse variance weighted		MR_PRESSO	
			OR (95% CI)	pval	OR (95% CI)	pval	OR (95% CI)	pval	OR (95% CI)	pval
Scyllo-inositol	Hypertension	6	1.078 (0.391 to 2.974)	0.892	1.093 (0.847 to 1.410)	0.496	1.139 (0.745 to 1.742)	0.547	1.167 (0.872 to 1.562)	0.375
Dodecanedioate	Hypertension	6	1.823 (0.907 to 3.666)	0.167	1.233 (1.005 to 1.513)	0.044	1.196 (0.985 to 1.453)	0.071		
Gamma-glutamylvaline	Hypertension	8	0.804 (0.108 to 5.987)	0.838	1.291 (0.793 to 2.101)	0.305	1.348 (0.902 to 2.013)	0.145		
Indolepropionate	Hypertension	10	0.878 (0.596 to 1.296)	0.532	0.964 (0.792 to 1.172)	0.711	1.006 (0.871 to 1.162)	0.935		
Butyrylcarnitine	Hypertension	16	0.859 (0.724 to 1.021)	0.106	0.912 (0.843 to 0.988)	0.024	0.945 (0.848 to 1.053)	0.306	0.983 (0.842 to 1.148)	0.829

Exposure	Outcome	nSNP	MR Egger		Weighted median		Inverse variance weighted		MR_PRESSO	
			OR (95% CI)	pval	OR (95% CI)	pval	OR (95% CI)	pval	OR (95% CI)	pval
Myristoleate (14:1n5)	Hypertension	10	1.082 (0.749 to 1.563)	0.686	0.938 (0.782 to 1.126)	0.493	0.913 (0.742 to 1.125)	0.393	0.871 (0.738 to 1.028)	0.142
Dehydroisoandrosterone sulfate (DHEA-S)	Hypertension	12	0.953 (0.497 to 1.828)	0.888	1.072 (0.887 to 1.296)	0.471	1.122 (0.895 to 1.406)	0.319	1.069 (0.872 to 1.309)	0.538
Propionylcarnitine	Hypertension	25	0.580 (0.340 to 0.990)	0.058	0.820 (0.603 to 1.114)	0.204	0.845 (0.663 to 1.077)	0.173	0.983 (0.788 to 1.227)	0.881
Caproate (6:0)	Hypertension	17	0.670 (0.147 to 3.051)	0.612	0.732 (0.471 to 1.137)	0.165	0.723 (0.457 to 1.142)	0.164	0.592 (0.419 to 0.837)	0.010
Caprylate (8:0)	Hypertension	31	1.660 (0.893 to 3.086)	0.120	1.051 (0.765 to 1.443)	0.761	0.925 (0.701 to 1.219)	0.579	0.875 (0.678 to 1.129)	0.312

Exposure	Outcome	nSNP	MR Egger		Weighted median		Inverse variance weighted		MR_PRESSO	
			OR (95% CI)	pval	OR (95% CI)	pval	OR (95% CI)	pval	OR (95% CI)	pval
10-undecenoate (11:1n1)	Hypertension	11	0.992 (0.530 to 1.859)	0.981	1.020 (0.811 to 1.285)	0.863	1.059 (0.764 to 1.469)	0.730	1.046 (0.845 to 1.295)	0.689
Docosapentaenoate (n3 DPA; 22:5n3)	Hypertension	4	0.528 (0.262 to 1.064)	0.216	0.778 (0.585 to 1.035)	0.084	0.811 (0.640 to 1.027)	0.082		
X-11204	Hypertension	23	1.904 (0.214 to 16.939)	0.570	0.879 (0.569 to 1.359)	0.563	0.525 (0.280 to 0.983)	0.044	0.766 (0.523 to 1.122)	0.187
X-02269	Hypertension	8	0.935 (0.574 to 1.523)	0.797	1.117 (0.954 to 1.309)	0.168	1.084 (0.948 to 1.240)	0.237		
Phenol sulfate	Hypertension	9	1.288 (0.773 to 2.148)	0.364	1.002 (0.816 to 1.232)	0.983	0.971 (0.829 to 1.137)	0.714		

Exposure	Outcome	nSNP	MR Egger		Weighted median		Inverse variance weighted		MR_PRESSO	
			OR (95% CI)	pval	OR (95% CI)	pval	OR (95% CI)	pval	OR (95% CI)	pval
X-06126	Hypertension	10	1.479 (0.801 to 2.731)	0.246	1.064 (0.892 to 1.270)	0.489	0.983 (0.860 to 1.124)	0.806		
X-07765	Hypertension	9	1.012 (0.828 to 1.235)	0.913	1.008 (0.920 to 1.106)	0.859	1.036 (0.944 to 1.137)	0.456		
X-11247	Hypertension	11	0.994 (0.734 to 1.346)	0.970	0.913 (0.799 to 1.044)	0.184	0.977 (0.858 to 1.113)	0.730	0.946 (0.840 to 1.066)	0.387
X-11261	Hypertension	7	0.897 (0.422 to 1.908)	0.789	0.963 (0.763 to 1.217)	0.754	0.975 (0.746 to 1.276)	0.855	0.952 (0.781 to 1.159)	0.649
Bilirubin (E,E)*	Hypertension	8	1.047 (0.906 to 1.209)	0.557	0.989 (0.910 to 1.076)	0.798	0.987 (0.914 to 1.067)	0.748		

Exposure	Outcome	nSNP	MR Egger		Weighted median		Inverse variance weighted		MR_PRESSO	
			OR (95% CI)	pval	OR (95% CI)	pval	OR (95% CI)	pval	OR (95% CI)	pval
X-02249	Hypertension	7	0.905 (0.458 to 1.789)	0.785	0.866 (0.677 to 1.108)	0.253	0.839 (0.690 to 1.021)	0.080		
Heme*	Hypertension	4	0.724 (0.241 to 2.170)	0.622	0.755 (0.549 to 1.039)	0.085	0.734 (0.567 to 0.951)	0.019		
X-11299	Hypertension	6	0.978 (0.491 to 1.949)	0.953	0.981 (0.858 to 1.121)	0.778	0.962 (0.838 to 1.104)	0.579		
X-11315	Hypertension	17	0.914 (0.659 to 1.266)	0.595	0.956 (0.779 to 1.174)	0.667	0.949 (0.773 to 1.165)	0.615		
X-11317	Hypertension	28	1.476 (0.476 to 4.576)	0.506	1.283 (0.921 to 1.786)	0.141	0.911 (0.610 to 1.362)	0.650	1.063 (0.832 to 1.359)	0.629

Exposure	Outcome	nSNP	MR Egger		Weighted median		Inverse variance weighted		MR_PRESSO	
			OR (95% CI)	pval	OR (95% CI)	pval	OR (95% CI)	pval	OR (95% CI)	pval
1-linoleoylglycerophosphoethanolamine*	Hypertension	8	0.787 (0.302 to 2.055)	0.643	1.071 (0.860 to 1.335)	0.539	1.199 (0.856 to 1.679)	0.291	1.173 (0.931 to 1.477)	0.234
X-11327	Hypertension	24	3.751 (1.494 to 9.414)	0.010	1.549 (1.069 to 2.244)	0.021	1.438 (1.036 to 1.997)	0.030	1.243 (0.914 to 1.692)	0.180
X-11334	Hypertension	14	2.173 (0.585 to 8.073)	0.269	1.004 (0.726 to 1.389)	0.979	1.097 (0.749 to 1.607)	0.633	0.936 (0.761 to 1.152)	0.545
3-dehydrocarnitine*	Hypertension	16	3.696 (1.220 to 11.201)	0.037	1.106 (0.776 to 1.576)	0.578	0.925 (0.657 to 1.302)	0.654	0.790 (0.583 to 1.070)	0.151
Pyroglutamine*	Hypertension	11	1.420 (0.879 to 2.294)	0.185	1.194 (0.983 to 1.451)	0.074	1.154 (0.962 to 1.384)	0.123	1.199 (1.035 to 1.389)	0.039

Exposure	Outcome	nSNP	MR Egger		Weighted median		Inverse variance weighted		MR_PRESSO	
			OR (95% CI)	pval	OR (95% CI)	pval	OR (95% CI)	pval	OR (95% CI)	pval
C-glycosyltryptophan*	Hypertension	14	0.393 (0.088 to 1.750)	0.244	0.627 (0.366 to 1.074)	0.089	0.755 (0.440 to 1.296)	0.308	0.847 (0.534 to 1.344)	0.495
X-11374	Hypertension	11	0.908 (0.615 to 1.339)	0.637	0.945 (0.812 to 1.100)	0.465	0.978 (0.826 to 1.157)	0.792	0.938 (0.808 to 1.089)	0.423
X-11381	Hypertension	9	1.308 (0.596 to 2.869)	0.524	0.932 (0.635 to 1.370)	0.721	0.845 (0.636 to 1.122)	0.245		
X-03056--N-[3-(2-Oxopyrrolidin-1-yl)propyl]acetamide	Hypertension	15	0.881 (0.585 to 1.327)	0.555	1.034 (0.829 to 1.291)	0.766	1.007 (0.805 to 1.261)	0.948	1.000 (0.834 to 1.200)	0.996
X-11412	Hypertension	26	0.838 (0.086 to 8.214)	0.881	1.184 (0.815 to 1.720)	0.375	1.252 (0.846 to 1.853)	0.260	1.350 (1.037 to 1.758)	0.037

Exposure	Outcome	nSNP	MR Egger		Weighted median		Inverse variance weighted		MR_PRESSO	
			OR (95% CI)	pval	OR (95% CI)	pval	OR (95% CI)	pval	OR (95% CI)	pval
X-01911	Hypertension	9	0.852 (0.607 to 1.197)	0.387	0.894 (0.763 to 1.048)	0.167	0.817 (0.716 to 0.932)	0.003		
X-11422--xanthine	Hypertension	3	2.041 (0.072 to 58.213)	0.748	0.606 (0.333 to 1.100)	0.100	0.642 (0.385 to 1.072)	0.090		
X-11423--O-sulfo-L-tyrosine	Hypertension	22	1.072 (0.351 to 3.272)	0.904	1.088 (0.730 to 1.623)	0.679	1.027 (0.712 to 1.483)	0.885	1.123 (0.821 to 1.535)	0.477
X-09789	Hypertension	6	2.883 (0.668 to 12.439)	0.229	1.087 (0.889 to 1.329)	0.416	1.220 (0.935 to 1.592)	0.144	1.086 (0.934 to 1.262)	0.343
X-11437	Hypertension	7	0.980 (0.872 to 1.101)	0.746	0.975 (0.906 to 1.050)	0.509	0.976 (0.906 to 1.053)	0.536		

Exposure	Outcome	nSNP	MR Egger		Weighted median		Inverse variance weighted		MR_PRESSO	
			OR (95% CI)	pval	OR (95% CI)	pval	OR (95% CI)	pval	OR (95% CI)	pval
X-11438	Hypertension	17	0.793 (0.428 to 1.471)	0.473	0.892 (0.722 to 1.102)	0.289	1.034 (0.813 to 1.316)	0.785	1.033 (0.869 to 1.229)	0.717
X-11440	Hypertension	10	0.827 (0.637 to 1.074)	0.193	0.919 (0.843 to 1.002)	0.056	0.981 (0.802 to 1.199)	0.851	0.965 (0.857 to 1.088)	0.583
X-11441	Hypertension	8	1.012 (0.844 to 1.213)	0.902	1.010 (0.914 to 1.115)	0.852	0.998 (0.893 to 1.114)	0.965		
X-11442	Hypertension	8	0.991 (0.788 to 1.246)	0.940	1.013 (0.915 to 1.122)	0.801	1.037 (0.906 to 1.187)	0.601		
X-11444	Hypertension	8	1.139 (0.589 to 2.200)	0.713	1.330 (1.020 to 1.733)	0.035	1.328 (0.992 to 1.780)	0.057		

Exposure	Outcome	nSNP	MR Egger		Weighted median		Inverse variance weighted		MR_PRESSO	
			OR (95% CI)	pval	OR (95% CI)	pval	OR (95% CI)	pval	OR (95% CI)	pval
X-11445--5-alpha-pregnan-3beta,20alpha-disulfate	Hypertension	9	1.034 (0.577 to 1.855)	0.914	0.859 (0.762 to 0.969)	0.013	0.970 (0.843 to 1.115)	0.666	0.940 (0.829 to 1.065)	0.364
X-11452	Hypertension	4	0.612 (0.206 to 1.817)	0.469	1.274 (0.969 to 1.675)	0.083	1.341 (0.921 to 1.951)	0.126	1.316 (1.174 to 1.474)	0.133
X-11469	Hypertension	8	1.226 (0.493 to 3.046)	0.676	1.077 (0.936 to 1.239)	0.298	1.029 (0.901 to 1.175)	0.674		
X-11470	Hypertension	7	0.691 (0.115 to 4.163)	0.703	0.896 (0.628 to 1.277)	0.543	1.017 (0.497 to 2.078)	0.964	1.024 (0.741 to 1.415)	0.893
X-11478	Hypertension	8	1.052 (0.825 to 1.341)	0.697	0.970 (0.828 to 1.136)	0.708	0.987 (0.849 to 1.148)	0.865		

Exposure	Outcome	nSNP	MR Egger		Weighted median		Inverse variance weighted		MR_PRESSO	
			OR (95% CI)	pval	OR (95% CI)	pval	OR (95% CI)	pval	OR (95% CI)	pval
X-11483	Hypertension	6	0.974 (0.871 to 1.089)	0.668	0.957 (0.885 to 1.034)	0.266	0.950 (0.878 to 1.028)	0.205		
X-11485	Hypertension	2					1.015 (0.815 to 1.264)	0.892		
X-11491	Hypertension	9	1.115 (0.829 to 1.501)	0.495	0.853 (0.748 to 0.972)	0.017	0.859 (0.718 to 1.028)	0.097	0.834 (0.717 to 0.969)	0.056
X-11497	Hypertension	5	0.702 (0.045 to 11.017)	0.817	0.862 (0.493 to 1.508)	0.603	0.917 (0.589 to 1.427)	0.700		
HWESASXX*	Hypertension	6	1.837 (0.570 to 5.918)	0.366	0.962 (0.777 to 1.192)	0.726	0.933 (0.710 to 1.226)	0.619	0.957 (0.710 to 1.291)	0.794

Exposure	Outcome	nSNP	MR Egger		Weighted median		Inverse variance weighted		MR_PRESSO	
			OR (95% CI)	pval	OR (95% CI)	pval	OR (95% CI)	pval	OR (95% CI)	pval
X-11521	Hypertension	6	0.388 (0.077 to 1.959)	0.316	0.906 (0.652 to 1.259)	0.556	0.942 (0.706 to 1.258)	0.688		
X-11529	Hypertension	5	0.923 (0.868 to 0.981)	0.082	0.962 (0.921 to 1.004)	0.076	0.966 (0.915 to 1.019)	0.203		
X-11530	Hypertension	5	1.039 (0.843 to 1.281)	0.743	1.012 (0.921 to 1.112)	0.801	1.009 (0.907 to 1.123)	0.865		
X-11537	Hypertension	4	0.903 (0.704 to 1.158)	0.506	0.920 (0.827 to 1.024)	0.127	0.931 (0.839 to 1.034)	0.182		
X-11538	Hypertension	9	0.879 (0.730 to 1.059)	0.218	0.881 (0.799 to 0.970)	0.010	0.906 (0.821 to 0.999)	0.048		

Exposure	Outcome	nSNP	MR Egger		Weighted median		Inverse variance weighted		MR_PRESSO	
			OR (95% CI)	pval	OR (95% CI)	pval	OR (95% CI)	pval	OR (95% CI)	pval
X-11540	Hypertension	5	1.034 (0.531 to 2.014)	0.927	0.990 (0.798 to 1.229)	0.930	1.053 (0.860 to 1.289)	0.618		
X-11546	Hypertension	10	1.085 (0.987 to 1.194)	0.130	1.036 (0.965 to 1.112)	0.328	1.040 (0.991 to 1.093)	0.114		
X-11550	Hypertension	15	1.169 (0.032 to 42.757)	0.933	1.409 (0.722 to 2.748)	0.314	0.908 (0.392 to 2.100)	0.821	1.204 (0.674 to 2.151)	0.542
X-11552	Hypertension	8	0.818 (0.170 to 3.925)	0.810	0.953 (0.755 to 1.202)	0.682	1.034 (0.822 to 1.299)	0.776		
X-11593--O-methylascorbate*	Hypertension	24	0.626 (0.418 to 0.938)	0.033	0.633 (0.508 to 0.788)	4.43e-05	0.713 (0.557 to 0.913)	0.007	0.874 (0.605 to 1.263)	0.480

Exposure	Outcome	nSNP	MR Egger		Weighted median		Inverse variance weighted		MR_PRESSO	
			OR (95% CI)	pval	OR (95% CI)	pval	OR (95% CI)	pval	OR (95% CI)	pval
Adrenate (22:4n6)	Hypertension	5	0.540 (0.244 to 1.193)	0.225	0.915 (0.701 to 1.194)	0.512	1.001 (0.805 to 1.244)	0.994		
Homostachydrine*	Hypertension	4	1.207 (0.739 to 1.971)	0.531	1.081 (0.868 to 1.346)	0.486	1.096 (0.933 to 1.288)	0.265		
ADSGEGDFXAEGGGVR*	Hypertension	6	1.049 (0.645 to 1.705)	0.858	1.043 (0.914 to 1.190)	0.530	1.049 (0.853 to 1.292)	0.649	1.083 (0.895 to 1.309)	0.459
X-11786--methylcysteine	Hypertension	8	1.006 (0.429 to 2.354)	0.990	0.912 (0.756 to 1.099)	0.333	0.903 (0.748 to 1.091)	0.291		
X-11787	Hypertension	15	0.742 (0.391 to 1.407)	0.377	0.897 (0.584 to 1.378)	0.619	0.929 (0.658 to 1.311)	0.676	1.363 (0.972 to 1.912)	0.096

Exposure	Outcome	nSNP	MR Egger		Weighted median		Inverse variance weighted		MR_PRESSO	
			OR (95% CI)	pval	OR (95% CI)	pval	OR (95% CI)	pval	OR (95% CI)	pval
X-11792	Hypertension	10	1.099 (0.925 to 1.305)	0.314	1.066 (0.990 to 1.148)	0.092	1.034 (0.977 to 1.095)	0.250		
X-11793--oxidized bilirubin*	Hypertension	9	1.062 (0.895 to 1.260)	0.512	1.015 (0.908 to 1.134)	0.793	0.996 (0.902 to 1.101)	0.939		
X-11795	Hypertension	7	0.331 (0.080 to 1.367)	0.187	0.940 (0.664 to 1.330)	0.727	0.921 (0.621 to 1.366)	0.681		
X-11799	Hypertension	4	0.940 (0.700 to 1.262)	0.720	0.935 (0.857 to 1.021)	0.135	0.929 (0.829 to 1.041)	0.205		
X-11805	Hypertension	3	1.295 (0.749 to 2.239)	0.525	1.031 (0.842 to 1.261)	0.768	1.005 (0.848 to 1.192)	0.952		

Exposure	Outcome	nSNP	MR Egger		Weighted median		Inverse variance weighted		MR_PRESSO	
			OR (95% CI)	pval	OR (95% CI)	pval	OR (95% CI)	pval	OR (95% CI)	pval
X-11818	Hypertension	13	0.982 (0.552 to 1.746)	0.952	1.001 (0.745 to 1.344)	0.996	0.952 (0.747 to 1.213)	0.690		
X-11820	Hypertension	7	1.309 (0.841 to 2.037)	0.287	1.074 (0.893 to 1.292)	0.450	0.978 (0.763 to 1.253)	0.859	1.046 (0.922 to 1.188)	0.513
X-11843	Hypertension	1								
X-11845	Hypertension	12	1.018 (0.763 to 1.359)	0.904	1.035 (0.950 to 1.128)	0.429	1.045 (0.975 to 1.119)	0.213		
X-11847	Hypertension	7	1.003 (0.647 to 1.555)	0.990	0.917 (0.816 to 1.031)	0.148	0.955 (0.853 to 1.070)	0.427		
X-11849	Hypertension	7	0.989 (0.867 to 1.127)	0.871	1.074 (0.980	0.128	1.061 (0.993	0.078		

Exposure	Outcome	nSNP	MR Egger		Weighted median		Inverse variance weighted		MR_PRESSO	
			OR (95% CI)	pval	OR (95% CI)	pval	OR (95% CI)	pval	OR (95% CI)	pval
					to 1.177)		to 1.133)			
X-11850	Hypertension	4	0.978 (0.881 to 1.087)	0.723	1.069 (0.988 to 1.157)	0.096	1.096 (0.971 to 1.238)	0.139		
X-11852	Hypertension	7	0.906 (0.678 to 1.210)	0.532	0.982 (0.862 to 1.119)	0.788	0.918 (0.791 to 1.065)	0.260	0.823 (0.736 to 0.920)	0.041
X-11858	Hypertension	6	0.957 (0.850 to 1.077)	0.506	0.981 (0.914 to 1.054)	0.605	0.993 (0.938 to 1.051)	0.812		
X-11859	Hypertension	6	0.891 (0.180 to 4.400)	0.894	0.817 (0.572 to 1.168)	0.268	0.733 (0.494 to 1.088)	0.123		

Exposure	Outcome	nSNP	MR Egger		Weighted median		Inverse variance weighted		MR_PRESSO	
			OR (95% CI)	pval	OR (95% CI)	pval	OR (95% CI)	pval	OR (95% CI)	pval
X-11876	Hypertension	8	0.772 (0.458 to 1.302)	0.370	0.947 (0.743 to 1.207)	0.659	1.062 (0.838 to 1.345)	0.620		
1-arachidonoylglycerophosphocholine*	Hypertension	14	0.851 (0.561 to 1.292)	0.464	0.950 (0.791 to 1.141)	0.585	1.004 (0.803 to 1.257)	0.970	1.016 (0.877 to 1.177)	0.835
1-palmitoleoylglycerophosphocholine*	Hypertension	8	0.397 (0.124 to 1.272)	0.171	1.228 (0.839 to 1.798)	0.290	1.013 (0.725 to 1.416)	0.941		
X-11905	Hypertension	12	0.933 (0.767 to 1.135)	0.501	1.063 (0.936 to 1.206)	0.346	0.996 (0.907 to 1.094)	0.938		
X-12007	Hypertension	18	0.989 (0.905 to 1.081)	0.811	1.023 (0.964 to 1.085)	0.453	1.027 (0.981 to 1.074)	0.254		

Exposure	Outcome	nSNP	MR Egger		Weighted median		Inverse variance weighted		MR_PRESSO	
			OR (95% CI)	pval	OR (95% CI)	pval	OR (95% CI)	pval	OR (95% CI)	pval
X-12013	Hypertension	4	1.038 (0.957 to 1.126)	0.465	1.062 (0.983 to 1.147)	0.128	1.081 (1.013 to 1.153)	0.018		
Gamma-glutamylmethionine*	Hypertension	5	0.810 (0.369 to 1.780)	0.637	0.980 (0.727 to 1.321)	0.897	0.963 (0.771 to 1.202)	0.738		
Gamma-glutamylthreonine*	Hypertension	6	1.212 (0.315 to 4.659)	0.794	1.270 (0.874 to 1.845)	0.210	1.152 (0.682 to 1.947)	0.596	1.476 (1.097 to 1.988)	0.124
X-12029	Hypertension	26	1.205 (0.418 to 3.475)	0.733	1.158 (0.795 to 1.688)	0.445	0.994 (0.722 to 1.368)	0.970		
X-12038	Hypertension	35	0.308 (0.035 to 2.689)	0.294	1.209 (0.867 to 1.684)	0.263	1.129 (0.777 to 1.640)	0.524	1.181 (0.896 to 1.557)	0.245

Exposure	Outcome	nSNP	MR Egger		Weighted median		Inverse variance weighted		MR_PRESSO	
			OR (95% CI)	pval	OR (95% CI)	pval	OR (95% CI)	pval	OR (95% CI)	pval
X-12039	Hypertension	12	1.051 (0.924 to 1.195)	0.468	1.004 (0.912 to 1.104)	0.942	1.042 (0.972 to 1.118)	0.245		
X-12040	Hypertension	9	1.129 (1.058 to 1.204)	0.008	1.015 (0.982 to 1.050)	0.377	1.010 (0.967 to 1.055)	0.653	1.020 (0.993 to 1.048)	0.190
X-12056	Hypertension	10	0.988 (0.720 to 1.355)	0.941	1.017 (0.907 to 1.140)	0.775	1.000 (0.854 to 1.171)	0.998	1.055 (0.971 to 1.145)	0.243
X-12063	Hypertension	11	1.040 (0.862 to 1.255)	0.693	1.117 (1.003 to 1.244)	0.043	1.022 (0.910 to 1.147)	0.716	1.034 (0.846 to 1.263)	0.755
Gamma-tocopherol	Hypertension	7	1.029 (0.469 to 2.254)	0.947	1.168 (0.943 to 1.447)	0.154	1.252 (1.055 to 1.485)	0.010		

Exposure	Outcome	nSNP	MR Egger		Weighted median		Inverse variance weighted		MR_PRESSO	
			OR (95% CI)	pval	OR (95% CI)	pval	OR (95% CI)	pval	OR (95% CI)	pval
Gamma-glutamylphenylalanine	Hypertension	21	1.195 (0.340 to 4.200)	0.784	1.152 (0.746 to 1.779)	0.525	0.930 (0.603 to 1.436)	0.745	1.010 (0.673 to 1.516)	0.962
Isobutyrylcarnitine	Hypertension	10	0.931 (0.550 to 1.574)	0.795	0.878 (0.736 to 1.047)	0.146	0.908 (0.712 to 1.159)	0.439	0.937 (0.795 to 1.104)	0.462
Pseudouridine	Hypertension	19	3.046 (0.674 to 13.752)	0.166	1.930 (1.164 to 3.202)	0.011	1.512 (1.026 to 2.229)	0.036		
Valerate	Hypertension	1								
Palmitoleate (16:1n7)	Hypertension	4	0.594 (0.213 to 1.653)	0.424	0.894 (0.606 to 1.319)	0.571	0.838 (0.593 to 1.185)	0.318		
Alpha-ketoglutarate	Hypertension	10	1.002 (0.614 to 1.636)	0.993	1.080 (0.881	0.459	1.100 (0.848	0.473	1.170 (0.977	0.126

Exposure	Outcome	nSNP	MR Egger		Weighted median		Inverse variance weighted		MR_PRESSO	
			OR (95% CI)	pval	OR (95% CI)	pval	OR (95% CI)	pval	OR (95% CI)	pval
					to 1.325)		to 1.428)		to 1.401)	
Erythronate*	Hypertension	26	0.888 (0.012 to 66.204)	0.957	1.042 (0.699 to 1.555)	0.840	1.508 (0.813 to 2.797)	0.192	1.140 (0.823 to 1.578)	0.438
Lathosterol	Hypertension	9	0.947 (0.644 to 1.394)	0.792	1.046 (0.840 to 1.302)	0.688	1.082 (0.929 to 1.260)	0.314		
X-12092	Hypertension	11	0.996 (0.904 to 1.098)	0.939	0.990 (0.949 to 1.033)	0.651	0.998 (0.930 to 1.070)	0.949		
X-12093	Hypertension	7	1.125 (0.880 to 1.439)	0.390	1.129 (1.016 to 1.254)	0.024	1.053 (0.939 to 1.182)	0.375		

Exposure	Outcome	nSNP	MR Egger		Weighted median		Inverse variance weighted		MR_PRESSO	
			OR (95% CI)	pval	OR (95% CI)	pval	OR (95% CI)	pval	OR (95% CI)	pval
X-12094	Hypertension	5	0.289 (0.042 to 1.965)	0.294	0.743 (0.516 to 1.071)	0.112	1.020 (0.540 to 1.927)	0.951	0.932 (0.502 to 1.729)	0.844
X-12095--N1-methyl-3-pyridone-4-carboxamide	Hypertension	15	0.986 (0.695 to 1.398)	0.938	1.040 (0.841 to 1.286)	0.719	1.082 (0.878 to 1.334)	0.460	1.078 (0.955 to 1.217)	0.248
X-12100--hydroxytryptophan*	Hypertension	31	0.990 (0.250 to 3.912)	0.988	1.068 (0.762 to 1.497)	0.704	1.273 (0.937 to 1.729)	0.122		
X-12116	Hypertension	4	1.662 (0.303 to 9.117)	0.618	0.905 (0.717 to 1.143)	0.403	0.912 (0.741 to 1.121)	0.380		
Eicosenoate (20:1n9 or 11)	Hypertension	6	2.147 (1.088 to 4.238)	0.092	1.350 (0.966 to 1.887)	0.079	1.400 (1.077 to 1.820)	0.012		

Exposure	Outcome	nSNP	MR Egger		Weighted median		Inverse variance weighted		MR_PRESSO	
			OR (95% CI)	pval	OR (95% CI)	pval	OR (95% CI)	pval	OR (95% CI)	pval
X-12188	Hypertension	10	1.031 (0.957 to 1.111)	0.445	1.001 (0.964 to 1.038)	0.970	0.983 (0.947 to 1.020)	0.357		
X-12189	Hypertension	20	0.976 (0.943 to 1.010)	0.187	0.975 (0.943 to 1.008)	0.138	0.986 (0.963 to 1.010)	0.262		
X-12206	Hypertension	5	0.496 (0.077 to 3.196)	0.514	1.055 (0.695 to 1.603)	0.800	1.313 (0.853 to 2.021)	0.216		
X-12212	Hypertension	5	1.294 (0.614 to 2.729)	0.547	1.076 (0.866 to 1.337)	0.506	1.090 (0.925 to 1.284)	0.302		
X-12216	Hypertension	1								
X-12217	Hypertension	7	1.083 (0.461 to 2.544)	0.862	0.999 (0.712	0.996	1.056 (0.698	0.797	1.009 (0.730	0.961

Exposure	Outcome	nSNP	MR Egger		Weighted median		Inverse variance weighted		MR_PRESSO	
			OR (95% CI)	pval	OR (95% CI)	pval	OR (95% CI)	pval	OR (95% CI)	pval
					to 1.402)		to 1.597)		to 1.395)	
X-12230	Hypertension	2					1.027 (0.748 to 1.411)	0.869		
X-12231	Hypertension	7	0.859 (0.058 to 12.647)	0.916	1.068 (0.857 to 1.331)	0.557	1.118 (0.768 to 1.628)	0.559	1.142 (0.943 to 1.383)	0.245
X-12236	Hypertension	8	0.987 (0.671 to 1.450)	0.948	0.896 (0.781 to 1.027)	0.115	0.887 (0.771 to 1.021)	0.094		
X-12244--N-acetylcarnosine	Hypertension	13	1.133 (0.489 to 2.624)	0.776	1.041 (0.801 to 1.352)	0.764	1.062 (0.846 to 1.334)	0.604		

Exposure	Outcome	nSNP	MR Egger		Weighted median		Inverse variance weighted		MR_PRESSO	
			OR (95% CI)	pval	OR (95% CI)	pval	OR (95% CI)	pval	OR (95% CI)	pval
X-12253	Hypertension	4	1.100 (0.343 to 3.529)	0.887	1.002 (0.825 to 1.218)	0.980	1.046 (0.779 to 1.405)	0.765		
X-12261	Hypertension	7	1.030 (0.954 to 1.112)	0.485	1.041 (0.980 to 1.107)	0.193	1.043 (0.997 to 1.091)	0.070		
X-12329	Hypertension	19	1.007 (0.978 to 1.038)	0.636	1.008 (0.985 to 1.032)	0.503	1.008 (0.986 to 1.030)	0.500		
X-10346	Hypertension	9	0.969 (0.867 to 1.084)	0.602	0.964 (0.899 to 1.033)	0.294	0.988 (0.934 to 1.044)	0.663		
ADpSGEGDFXAEGGGVR*	Hypertension	4	1.297 (0.549 to 3.067)	0.614	1.042 (0.872 to 1.246)	0.648	1.041 (0.824 to 1.314)	0.738		

Exposure	Outcome	nSNP	MR Egger		Weighted median		Inverse variance weighted		MR_PRESSO	
			OR (95% CI)	pval	OR (95% CI)	pval	OR (95% CI)	pval	OR (95% CI)	pval
1-eicosatrienoylglycerophosphocholine*	Hypertension	13	0.826 (0.374 to 1.827)	0.647	0.987 (0.767 to 1.270)	0.921	1.064 (0.809 to 1.401)	0.656	1.000 (0.789 to 1.268)	0.997
1-docosahexaenoylglycerophosphocholine*	Hypertension	4	0.353 (0.071 to 1.754)	0.331	1.050 (0.698 to 1.578)	0.816	1.006 (0.714 to 1.418)	0.971		
X-12405	Hypertension	4	0.628 (0.297 to 1.326)	0.347	0.986 (0.795 to 1.223)	0.901	1.031 (0.860 to 1.236)	0.742		
X-12407	Hypertension	5	0.822 (0.637 to 1.060)	0.229	0.984 (0.883 to 1.096)	0.763	1.025 (0.926 to 1.135)	0.628		
X-12428	Hypertension	2					0.785 (0.646 to 0.953)	0.014		

Exposure	Outcome	nSNP	MR Egger		Weighted median		Inverse variance weighted		MR_PRESSO	
			OR (95% CI)	pval	OR (95% CI)	pval	OR (95% CI)	pval	OR (95% CI)	pval
1-eicosadienoylglycerophosphocholine*	Hypertension	6	0.728 (0.318 to 1.668)	0.495	1.129 (0.859 to 1.482)	0.384	1.138 (0.920 to 1.408)	0.233		
X-12435	Hypertension	16	1.000 (0.957 to 1.046)	0.990	0.971 (0.938 to 1.005)	0.093	0.977 (0.951 to 1.004)	0.091		
X-12441--12-hydroxyeicosatetraenoate (12-HETE)	Hypertension	6	0.985 (0.749 to 1.296)	0.920	1.041 (0.950 to 1.140)	0.392	1.092 (0.990 to 1.204)	0.078		
X-12442--5,8-tetradecadienoate	Hypertension	6	1.065 (0.820 to 1.384)	0.661	1.036 (0.889 to 1.207)	0.654	1.070 (0.938 to 1.221)	0.316		
X-12443	Hypertension	7	0.880 (0.681 to 1.138)	0.374	0.909 (0.802 to 1.030)	0.133	0.895 (0.804 to 0.996)	0.042		

Exposure	Outcome	nSNP	MR Egger		Weighted median		Inverse variance weighted		MR_PRESSO	
			OR (95% CI)	pval	OR (95% CI)	pval	OR (95% CI)	pval	OR (95% CI)	pval
X-12450	Hypertension	16	0.870 (0.319 to 2.374)	0.790	1.056 (0.770 to 1.448)	0.735	0.917 (0.684 to 1.230)	0.564		
X-12456	Hypertension	13	0.865 (0.736 to 1.017)	0.107	1.031 (0.909 to 1.169)	0.639	1.009 (0.906 to 1.124)	0.874		
X-12465	Hypertension	6	1.265 (0.908 to 1.762)	0.237	1.205 (0.974 to 1.492)	0.086	1.054 (0.853 to 1.301)	0.626		
Octanoylcarnitine	Hypertension	6	0.914 (0.608 to 1.375)	0.689	1.044 (0.901 to 1.210)	0.567	1.074 (0.853 to 1.354)	0.544		
Alpha-hydroxyisovalerate	Hypertension	9	0.651 (0.393 to 1.078)	0.139	0.963 (0.778 to 1.192)	0.729	0.959 (0.781 to 1.179)	0.693		

Exposure	Outcome	nSNP	MR Egger		Weighted median		Inverse variance weighted		MR_PRESSO	
			OR (95% CI)	pval	OR (95% CI)	pval	OR (95% CI)	pval	OR (95% CI)	pval
N-acetylthreonine	Hypertension	6	0.539 (0.256 to 1.134)	0.179	0.840 (0.557 to 1.267)	0.406	0.902 (0.658 to 1.237)	0.521		
Decanoylcarnitine	Hypertension	9	1.109 (0.830 to 1.482)	0.505	1.069 (0.923 to 1.238)	0.375	1.001 (0.858 to 1.168)	0.993		
1-palmitoylglycerophosphocholine	Hypertension	20	2.727 (0.509 to 14.614)	0.257	1.082 (0.699 to 1.674)	0.725	0.970 (0.652 to 1.443)	0.881	0.859 (0.617 to 1.195)	0.379
1-heptadecanoylglycerophosphocholine	Hypertension	4	1.505 (0.622 to 3.640)	0.460	0.903 (0.614 to 1.330)	0.607	1.036 (0.756 to 1.418)	0.827		
1-oleoylglycerophosphocholine	Hypertension	7	0.839 (0.447 to 1.577)	0.609	1.139 (0.792 to 1.640)	0.483	1.276 (0.969 to 1.680)	0.082		

Exposure	Outcome	nSNP	MR Egger		Weighted median		Inverse variance weighted		MR_PRESSO	
			OR (95% CI)	pval	OR (95% CI)	pval	OR (95% CI)	pval	OR (95% CI)	pval
1-stearoylglycerophosphocholine	Hypertension	4	2.776 (0.264 to 29.143)	0.484	1.716 (1.070 to 2.753)	0.025	1.350 (0.828 to 2.204)	0.229		
5-dodecenoate (12:1n7)	Hypertension	11	0.556 (0.349 to 0.886)	0.036	0.884 (0.712 to 1.097)	0.263	0.821 (0.691 to 0.976)	0.026		
Stearidonate (18:4n3)	Hypertension	5	0.775 (0.321 to 1.874)	0.612	0.859 (0.649 to 1.136)	0.286	0.893 (0.715 to 1.115)	0.318		
10-heptadecenoate (17:1n7)	Hypertension	1								
10-nonadecenoate (19:1n9)	Hypertension	4	0.923 (0.167 to 5.112)	0.935	1.212 (0.835 to 1.760)	0.311	1.154 (0.667 to 1.995)	0.609		
Epiandrosterone sulfate	Hypertension	9	1.014 (0.910 to 1.129)	0.810	1.053 (0.975	0.191	1.096 (1.018	0.015		

Exposure	Outcome	nSNP	MR Egger		Weighted median		Inverse variance weighted		MR_PRESSO	
			OR (95% CI)	pval	OR (95% CI)	pval	OR (95% CI)	pval	OR (95% CI)	pval
					to 1.138)		to 1.181)			
Linolenate [alpha or gamma; (18:3n3 or 6)]	Hypertension	2					1.034 (0.668 to 1.601)	0.880		
X-12510--2-aminooctanoic acid	Hypertension	11	0.787 (0.622 to 0.995)	0.076	0.817 (0.733 to 0.912)	2.91e-04	0.863 (0.743 to 1.003)	0.055		
X-12524	Hypertension	15	2.154 (0.758 to 6.120)	0.174	1.036 (0.599 to 1.791)	0.899	1.355 (0.862 to 2.129)	0.188		
Bilirubin (E,Z or Z,E)*	Hypertension	8	1.040 (0.837 to 1.293)	0.735	1.001 (0.885 to 1.132)	0.989	0.978 (0.884 to 1.083)	0.672		

Exposure	Outcome	nSNP	MR Egger		Weighted median		Inverse variance weighted		MR_PRESSO	
			OR (95% CI)	pval	OR (95% CI)	pval	OR (95% CI)	pval	OR (95% CI)	pval
X-12544	Hypertension	10	1.010 (0.866 to 1.176)	0.906	1.015 (0.876 to 1.177)	0.838	1.055 (0.959 to 1.162)	0.272		
X-12556	Hypertension	13	1.686 (0.586 to 4.852)	0.353	0.768 (0.538 to 1.096)	0.146	0.719 (0.520 to 0.993)	0.045		
1-arachidonoylglycerophosphoinositol*	Hypertension	13	0.535 (0.300 to 0.954)	0.058	0.727 (0.574 to 0.920)	0.008	0.862 (0.677 to 1.097)	0.228		
X-12627	Hypertension	8	0.737 (0.301 to 1.800)	0.527	0.944 (0.747 to 1.192)	0.628	1.022 (0.794 to 1.317)	0.863	0.952 (0.768 to 1.180)	0.671
X-12644	Hypertension	13	1.037 (0.681 to 1.579)	0.870	1.124 (0.880 to 1.435)	0.350	0.930 (0.775 to 1.114)	0.429		

Exposure	Outcome	nSNP	MR Egger		Weighted median		Inverse variance weighted		MR_PRESSO	
			OR (95% CI)	pval	OR (95% CI)	pval	OR (95% CI)	pval	OR (95% CI)	pval
X-12645	Hypertension	10	0.765 (0.566 to 1.033)	0.118	1.169 (0.973 to 1.405)	0.096	1.113 (0.952 to 1.302)	0.180		
Asparagine	Hypertension	26	0.788 (0.442 to 1.406)	0.428	1.096 (0.791 to 1.519)	0.582	1.113 (0.839 to 1.478)	0.458		
X-12680	Hypertension	9	1.172 (0.826 to 1.664)	0.404	0.944 (0.765 to 1.165)	0.593	0.961 (0.825 to 1.119)	0.606		
X-12696	Hypertension	13	1.581 (0.899 to 2.779)	0.140	1.412 (1.035 to 1.926)	0.030	1.526 (1.208 to 1.928)	3.95e-04		
X-12704	Hypertension	4	0.964 (0.502 to 1.850)	0.922	0.888 (0.769 to 1.026)	0.106	0.896 (0.746 to 1.077)	0.243		

Exposure	Outcome	nSNP	MR Egger		Weighted median		Inverse variance weighted		MR_PRESSO	
			OR (95% CI)	pval	OR (95% CI)	pval	OR (95% CI)	pval	OR (95% CI)	pval
X-12712	Hypertension	20	1.000 (0.944 to 1.058)	0.989	0.979 (0.947 to 1.011)	0.199	0.995 (0.969 to 1.022)	0.726		
X-12717	Hypertension	3	0.495 (0.016 to 15.400)	0.757	0.972 (0.859 to 1.099)	0.645	0.988 (0.887 to 1.100)	0.822		
X-12719	Hypertension	4	0.735 (0.512 to 1.055)	0.237	1.015 (0.864 to 1.193)	0.853	0.986 (0.870 to 1.116)	0.819		
X-12726	Hypertension	12	0.925 (0.825 to 1.038)	0.216	0.940 (0.846 to 1.044)	0.248	0.934 (0.856 to 1.019)	0.125		
X-12728	Hypertension	33	1.010 (0.998 to 1.021)	0.123	1.004 (0.994 to 1.014)	0.422	1.005 (0.998 to 1.012)	0.182		

Exposure	Outcome	nSNP	MR Egger		Weighted median		Inverse variance weighted		MR_PRESSO	
			OR (95% CI)	pval	OR (95% CI)	pval	OR (95% CI)	pval	OR (95% CI)	pval
X-12729	Hypertension	11	1.007 (0.912 to 1.113)	0.889	0.977 (0.913 to 1.046)	0.510	0.994 (0.942 to 1.048)	0.823		
X-12734	Hypertension	4	1.476 (0.995 to 2.190)	0.193	0.971 (0.858 to 1.100)	0.648	1.010 (0.875 to 1.166)	0.895		
X-12740	Hypertension	10	1.009 (0.845 to 1.205)	0.920	0.989 (0.900 to 1.085)	0.811	0.968 (0.887 to 1.057)	0.472		
X-12749	Hypertension	25	1.165 (0.401 to 3.381)	0.782	1.381 (1.001 to 1.904)	0.049	1.084 (0.745 to 1.577)	0.674	1.211 (0.912 to 1.610)	0.199
Stachydrine	Hypertension	2					1.098 (0.959 to 1.257)	0.174		

Exposure	Outcome	nSNP	MR Egger		Weighted median		Inverse variance weighted		MR_PRESSO	
			OR (95% CI)	pval	OR (95% CI)	pval	OR (95% CI)	pval	OR (95% CI)	pval
Isovalerylcarnitine	Hypertension	14	1.031 (0.784 to 1.355)	0.830	1.026 (0.855 to 1.230)	0.785	1.009 (0.855 to 1.190)	0.916		
Stearoylcarnitine	Hypertension	4	3.109 (0.023 to 423.952)	0.695	1.417 (0.921 to 2.181)	0.113	1.302 (0.692 to 2.450)	0.413		
1-stearoylglycerophosphoethanolamine	Hypertension	9	1.601 (0.467 to 5.486)	0.478	1.250 (0.976 to 1.601)	0.077	1.151 (0.767 to 1.726)	0.497	1.186 (1.020 to 1.381)	0.069
1-linoleoylglycerophosphocholine	Hypertension	10	0.379 (0.148 to 0.974)	0.079	0.743 (0.508 to 1.087)	0.126	0.950 (0.691 to 1.305)	0.751		
Bradykinin, des-arg(9)	Hypertension	14	0.979 (0.857 to 1.118)	0.758	1.033 (0.976 to 1.094)	0.256	1.019 (0.958 to 1.083)	0.553	1.035 (0.992 to 1.079)	0.136

Exposure	Outcome	nSNP	MR Egger		Weighted median		Inverse variance weighted		MR_PRESSO	
			OR (95% CI)	pval	OR (95% CI)	pval	OR (95% CI)	pval	OR (95% CI)	pval
X-12771	Hypertension	10	0.885 (0.492 to 1.591)	0.693	1.059 (0.870 to 1.288)	0.571	1.118 (0.913 to 1.369)	0.282		
X-12776	Hypertension	2					2.011 (0.536 to 7.540)	0.300		
Gamma-glutamylisoleucine*	Hypertension	15	1.264 (0.482 to 3.317)	0.641	0.864 (0.618 to 1.208)	0.393	1.037 (0.732 to 1.469)	0.838	0.917 (0.661 to 1.274)	0.616
X-12786	Hypertension	5	0.977 (0.526 to 1.812)	0.945	0.766 (0.581 to 1.009)	0.057	0.889 (0.645 to 1.224)	0.470		
X-12798	Hypertension	6	0.997 (0.806 to 1.233)	0.977	0.983 (0.879 to 1.098)	0.756	0.983 (0.856 to 1.128)	0.803		

Exposure	Outcome	nSNP	MR Egger		Weighted median		Inverse variance weighted		MR_PRESSO	
			OR (95% CI)	pval	OR (95% CI)	pval	OR (95% CI)	pval	OR (95% CI)	pval
X-12816	Hypertension	3	0.872 (0.722 to 1.054)	0.391	0.902 (0.809 to 1.005)	0.061	0.910 (0.832 to 0.994)	0.037		
X-12830	Hypertension	8	0.819 (0.541 to 1.237)	0.379	0.960 (0.833 to 1.107)	0.577	1.007 (0.853 to 1.190)	0.930		
X-12833	Hypertension	10	0.968 (0.907 to 1.032)	0.349	0.993 (0.961 to 1.026)	0.663	0.987 (0.951 to 1.024)	0.482	0.979 (0.950 to 1.009)	0.198
X-12844	Hypertension	7	0.756 (0.260 to 2.201)	0.630	0.771 (0.563 to 1.056)	0.105	0.761 (0.609 to 0.951)	0.016		
X-12847	Hypertension	2					1.023 (0.752 to 1.392)	0.886		

Exposure	Outcome	nSNP	MR Egger		Weighted median		Inverse variance weighted		MR_PRESSO	
			OR (95% CI)	pval	OR (95% CI)	pval	OR (95% CI)	pval	OR (95% CI)	pval
X-12850	Hypertension	7	1.032 (0.731 to 1.456)	0.865	0.958 (0.828 to 1.108)	0.564	0.971 (0.812 to 1.161)	0.746	1.020 (0.892 to 1.166)	0.784
Laurylcarnitine	Hypertension	7	0.599 (0.097 to 3.706)	0.606	0.870 (0.678 to 1.115)	0.272	1.023 (0.773 to 1.355)	0.873	0.887 (0.794 to 0.990)	0.086
X-12851	Hypertension	4	0.207 (0.060 to 0.713)	0.130	0.940 (0.794 to 1.111)	0.466	0.991 (0.784 to 1.254)	0.942		
X-12855	Hypertension	12	1.007 (0.442 to 2.294)	0.986	0.940 (0.742 to 1.191)	0.609	0.932 (0.761 to 1.141)	0.495		
X-12990--docosapentaenoic acid (n6-DPA)	Hypertension	6	1.496 (0.660 to 3.390)	0.389	1.006 (0.826 to 1.226)	0.952	1.049 (0.895 to 1.229)	0.558		

Exposure	Outcome	nSNP	MR Egger		Weighted median		Inverse variance weighted		MR_PRESSO	
			OR (95% CI)	pval	OR (95% CI)	pval	OR (95% CI)	pval	OR (95% CI)	pval
Isovalerate	Hypertension	2					1.195 (0.642 to 2.225)	0.575		
X-13069	Hypertension	11	0.522 (0.222 to 1.231)	0.172	0.717 (0.567 to 0.907)	0.005	0.825 (0.632 to 1.075)	0.154	0.846 (0.686 to 1.042)	0.154
X-13183--stearamide	Hypertension	9	1.087 (0.821 to 1.440)	0.577	1.022 (0.876 to 1.191)	0.783	0.958 (0.837 to 1.095)	0.527		
X-13215	Hypertension	26	2.473 (1.355 to 4.514)	0.007	1.363 (0.983 to 1.891)	0.063	1.178 (0.873 to 1.590)	0.284	0.946 (0.681 to 1.314)	0.743
7-methylguanine	Hypertension	4	0.796 (0.319 to 1.982)	0.672	0.956 (0.683 to 1.339)	0.795	1.049 (0.793 to 1.389)	0.737		

Exposure	Outcome	nSNP	MR Egger		Weighted median		Inverse variance weighted		MR_PRESSO	
			OR (95% CI)	pval	OR (95% CI)	pval	OR (95% CI)	pval	OR (95% CI)	pval
Phenylacetylglutamine	Hypertension	11	0.874 (0.734 to 1.040)	0.164	0.898 (0.777 to 1.038)	0.145	0.904 (0.812 to 1.006)	0.063		
Pro-hydroxy-pro	Hypertension	12	0.780 (0.333 to 1.831)	0.581	0.804 (0.588 to 1.099)	0.171	0.829 (0.649 to 1.060)	0.135		
N2,N2-dimethylguanosine	Hypertension	34	1.107 (0.828 to 1.480)	0.496	1.127 (0.905 to 1.404)	0.286	1.032 (0.850 to 1.252)	0.752		
Cysteine-glutathione disulfide	Hypertension	7	0.625 (0.316 to 1.233)	0.233	0.901 (0.773 to 1.050)	0.180	0.899 (0.797 to 1.013)	0.082		
Oleoylcarnitine	Hypertension	4	0.308 (0.027 to 3.526)	0.444	1.329 (0.916 to 1.930)	0.134	1.308 (0.964 to 1.774)	0.084		

Exposure	Outcome	nSNP	MR Egger		Weighted median		Inverse variance weighted		MR_PRESSO	
			OR (95% CI)	pval	OR (95% CI)	pval	OR (95% CI)	pval	OR (95% CI)	pval
1-arachidonoylglycerophosphoethanolamine*	Hypertension	14	0.618 (0.376 to 1.017)	0.083	0.960 (0.739 to 1.249)	0.763	1.011 (0.812 to 1.259)	0.921		
X-13429	Hypertension	5	0.946 (0.723 to 1.238)	0.713	0.909 (0.842 to 0.980)	0.013	0.915 (0.848 to 0.988)	0.023		
X-13431--nonanoylcarnitine*	Hypertension	10	1.271 (1.056 to 1.528)	0.035	1.064 (0.967 to 1.169)	0.202	1.007 (0.876 to 1.157)	0.926		
X-13435	Hypertension	11	1.112 (0.539 to 2.294)	0.780	1.228 (0.930 to 1.620)	0.148	1.173 (0.836 to 1.646)	0.357	1.296 (0.944 to 1.778)	0.143
X-13477	Hypertension	4	0.315 (0.064 to 1.542)	0.290	0.600 (0.332 to 1.083)	0.090	0.687 (0.346 to 1.364)	0.284		

Exposure	Outcome	nSNP	MR Egger		Weighted median		Inverse variance weighted		MR_PRESSO	
			OR (95% CI)	pval	OR (95% CI)	pval	OR (95% CI)	pval	OR (95% CI)	pval
2-palmitoylglycerophosphocholine*	Hypertension	15	0.507 (0.150 to 1.714)	0.294	0.915 (0.629 to 1.330)	0.640	1.044 (0.751 to 1.451)	0.799	0.963 (0.716 to 1.296)	0.808
2-oleoylglycerophosphocholine*	Hypertension	9	0.462 (0.177 to 1.207)	0.159	0.966 (0.633 to 1.474)	0.873	1.066 (0.759 to 1.499)	0.711		
2-stearoylglycerophosphocholine*	Hypertension	9	1.827 (0.362 to 9.216)	0.489	0.822 (0.621 to 1.088)	0.171	0.926 (0.698 to 1.228)	0.593		
2-linoleoylglycerophosphocholine*	Hypertension	13	1.102 (0.397 to 3.059)	0.856	0.963 (0.709 to 1.308)	0.810	0.932 (0.680 to 1.277)	0.663	0.862 (0.639 to 1.163)	0.353
X-13496	Hypertension	29	5.202 (0.979 to 27.654)	0.064	1.427 (0.905 to 2.250)	0.126	1.160 (0.718 to 1.874)	0.543	1.189 (0.818 to 1.727)	0.374

Exposure	Outcome	nSNP	MR Egger		Weighted median		Inverse variance weighted		MR_PRESSO	
			OR (95% CI)	pval	OR (95% CI)	pval	OR (95% CI)	pval	OR (95% CI)	pval
1-palmitoylglycerophosphoinositol*	Hypertension	5	0.776 (0.384 to 1.567)	0.530	0.992 (0.771 to 1.276)	0.951	1.020 (0.760 to 1.369)	0.893		
X-13548	Hypertension	27	1.418 (0.487 to 4.129)	0.527	1.198 (0.836 to 1.718)	0.326	1.224 (0.879 to 1.704)	0.232	1.144 (0.842 to 1.554)	0.399
X-13549	Hypertension	11	1.336 (0.319 to 5.593)	0.701	1.282 (0.729 to 2.253)	0.388	1.547 (0.969 to 2.470)	0.067		
X-13553	Hypertension	2					1.287 (0.942 to 1.759)	0.113		
X-13619	Hypertension	18	0.537 (0.045 to 6.423)	0.630	0.517 (0.295 to 0.905)	0.021	0.492 (0.287 to 0.842)	0.010		

Exposure	Outcome	nSNP	MR Egger		Weighted median		Inverse variance weighted		MR_PRESSO	
			OR (95% CI)	pval	OR (95% CI)	pval	OR (95% CI)	pval	OR (95% CI)	pval
2-methylbutyroylcarnitine	Hypertension	15	0.710 (0.228 to 2.213)	0.565	1.437 (0.997 to 2.070)	0.052	1.507 (1.079 to 2.106)	0.016		
Hydroxyisovaleroyl carnitine	Hypertension	4	0.670 (0.353 to 1.275)	0.347	1.003 (0.723 to 1.393)	0.983	1.190 (0.767 to 1.847)	0.437		
Glutaroyl carnitine	Hypertension	21	1.261 (0.803 to 1.982)	0.327	1.064 (0.854 to 1.326)	0.578	0.989 (0.821 to 1.192)	0.909		
X-13658	Hypertension	9	1.009 (0.981 to 1.037)	0.562	1.000 (0.978 to 1.022)	0.989	0.999 (0.981 to 1.017)	0.909		
X-13671	Hypertension	10	0.811 (0.063 to 10.368)	0.876	0.782 (0.518 to 1.182)	0.243	0.726 (0.435 to 1.212)	0.221	0.741 (0.515 to 1.066)	0.150

Exposure	Outcome	nSNP	MR Egger		Weighted median		Inverse variance weighted		MR_PRESSO	
			OR (95% CI)	pval	OR (95% CI)	pval	OR (95% CI)	pval	OR (95% CI)	pval
2-tetradecenoyl carnitine	Hypertension	11	1.043 (0.795 to 1.370)	0.766	1.060 (0.908 to 1.239)	0.460	1.092 (0.964 to 1.235)	0.166		
4-hydroxyhippurate	Hypertension	3	0.421 (0.059 to 3.006)	0.547	0.800 (0.609 to 1.052)	0.111	0.902 (0.609 to 1.336)	0.607		
X-13741	Hypertension	8	0.527 (0.223 to 1.242)	0.193	0.993 (0.851 to 1.158)	0.930	1.115 (0.894 to 1.389)	0.335	1.021 (0.902 to 1.155)	0.752
1-myristoylglycerophosphocholine	Hypertension	2					1.862 (0.693 to 4.998)	0.217		
1-oleoylglycerophosphoethanolamine	Hypertension	4	0.374 (0.003 to 41.315)	0.722	0.908 (0.624 to 1.321)	0.613	1.116 (0.531 to 2.344)	0.772	0.966 (0.739 to 1.264)	0.844

Exposure	Outcome	nSNP	MR Egger		Weighted median		Inverse variance weighted		MR_PRESSO	
			OR (95% CI)	pval	OR (95% CI)	pval	OR (95% CI)	pval	OR (95% CI)	pval
1-palmitoylglycerophosphoethanolamine	Hypertension	14	0.908 (0.488 to 1.689)	0.766	1.049 (0.828 to 1.330)	0.692	0.998 (0.755 to 1.318)	0.987	0.928 (0.750 to 1.148)	0.504
3-(3-hydroxyphenyl)propionate	Hypertension	8	0.791 (0.523 to 1.197)	0.310	0.980 (0.908 to 1.058)	0.607	1.004 (0.852 to 1.184)	0.958	0.973 (0.920 to 1.030)	0.388
Tetradecanedioate	Hypertension	14	0.886 (0.770 to 1.019)	0.115	0.931 (0.836 to 1.036)	0.190	0.936 (0.864 to 1.015)	0.111		
2-hydroxypalmitate	Hypertension	30	1.404 (0.538 to 3.662)	0.494	1.004 (0.701 to 1.437)	0.984	0.978 (0.702 to 1.363)	0.896	0.893 (0.664 to 1.200)	0.459
Hexadecanedioate	Hypertension	15	0.880 (0.758 to 1.023)	0.119	0.936 (0.846 to 1.035)	0.196	0.953 (0.855 to 1.062)	0.384	0.936 (0.863 to 1.015)	0.132

Exposure	Outcome	nSNP	MR Egger		Weighted median		Inverse variance weighted		MR_PRESSO	
			OR (95% CI)	pval	OR (95% CI)	pval	OR (95% CI)	pval	OR (95% CI)	pval
Dihomo-linolenate (20:3n3 or n6)	Hypertension	14	1.374 (0.669 to 2.821)	0.404	1.145 (0.844 to 1.553)	0.384	1.076 (0.840 to 1.378)	0.563		
X-13859	Hypertension	28	0.902 (0.570 to 1.426)	0.662	1.053 (0.774 to 1.432)	0.744	1.074 (0.854 to 1.351)	0.542		
Threitol	Hypertension	3	1.096 (0.001 to 971.663)	0.983	1.251 (0.807 to 1.941)	0.317	1.241 (0.860 to 1.791)	0.248		
X-14056	Hypertension	5	1.028 (0.723 to 1.461)	0.889	1.200 (0.937 to 1.538)	0.149	1.216 (1.007 to 1.469)	0.042		
X-14057	Hypertension	4	1.530 (0.497 to 4.709)	0.535	1.116 (0.829 to 1.502)	0.469	1.218 (0.807 to 1.839)	0.347		

Exposure	Outcome	nSNP	MR Egger		Weighted median		Inverse variance weighted		MR_PRESSO	
			OR (95% CI)	pval	OR (95% CI)	pval	OR (95% CI)	pval	OR (95% CI)	pval
X-14086	Hypertension	9	0.754 (0.436 to 1.306)	0.348	1.019 (0.853 to 1.217)	0.838	1.040 (0.887 to 1.219)	0.628		
P-cresol sulfate	Hypertension	8	0.900 (0.741 to 1.094)	0.330	0.986 (0.874 to 1.112)	0.815	0.959 (0.853 to 1.079)	0.484		
X-14189--leucylalanine	Hypertension	8	1.079 (0.921 to 1.263)	0.383	1.012 (0.934 to 1.097)	0.769	0.989 (0.902 to 1.084)	0.812		
X-14205--alpha-glutamyltyrosine	Hypertension	7	1.208 (0.800 to 1.822)	0.410	1.091 (0.921 to 1.292)	0.312	1.044 (0.898 to 1.214)	0.576		
X-14208--phenylalanylserine	Hypertension	10	1.055 (0.933 to 1.193)	0.419	1.046 (0.956 to 1.145)	0.324	1.071 (0.998 to 1.148)	0.056		

Exposure	Outcome	nSNP	MR Egger		Weighted median		Inverse variance weighted		MR_PRESSO	
			OR (95% CI)	pval	OR (95% CI)	pval	OR (95% CI)	pval	OR (95% CI)	pval
X-14304--leucylalanine	Hypertension	10	1.076 (0.728 to 1.591)	0.722	1.166 (1.002 to 1.358)	0.048	1.159 (1.036 to 1.297)	0.010		
X-14374	Hypertension	6	0.933 (0.106 to 8.241)	0.953	0.967 (0.694 to 1.347)	0.843	1.009 (0.675 to 1.508)	0.964		
X-14450--phenylalanylleucine	Hypertension	4	0.744 (0.338 to 1.636)	0.539	0.934 (0.777 to 1.124)	0.470	0.915 (0.791 to 1.059)	0.233		
X-14473	Hypertension	8	1.558 (1.082 to 2.244)	0.055	1.252 (1.044 to 1.503)	0.015	1.166 (1.024 to 1.327)	0.020		
X-14541	Hypertension	11	0.815 (0.583 to 1.140)	0.263	0.917 (0.810 to 1.038)	0.170	0.924 (0.840 to 1.016)	0.104		

Exposure	Outcome	nSNP	MR Egger		Weighted median		Inverse variance weighted		MR_PRESSO	
			OR (95% CI)	pval	OR (95% CI)	pval	OR (95% CI)	pval	OR (95% CI)	pval
X-14588	Hypertension	6	0.625 (0.153 to 2.550)	0.548	0.473 (0.163 to 1.371)	0.168	0.292 (0.136 to 0.627)	0.002		
X-14625	Hypertension	10	1.605 (0.529 to 4.873)	0.428	1.601 (0.967 to 2.651)	0.067	1.519 (0.978 to 2.358)	0.063		
X-14626	Hypertension	10	0.696 (0.385 to 1.259)	0.265	0.757 (0.620 to 0.924)	0.006	0.809 (0.602 to 1.087)	0.160	0.713 (0.598 to 0.851)	0.007
X-14632	Hypertension	13	1.162 (1.010 to 1.338)	0.060	1.143 (1.015 to 1.287)	0.028	1.092 (0.991 to 1.204)	0.074		
X-14658	Hypertension	7	0.686 (0.426 to 1.104)	0.181	0.900 (0.742 to 1.091)	0.284	0.931 (0.776 to 1.117)	0.439		

Exposure	Outcome	nSNP	MR Egger		Weighted median		Inverse variance weighted		MR_PRESSO	
			OR (95% CI)	pval	OR (95% CI)	pval	OR (95% CI)	pval	OR (95% CI)	pval
X-14662	Hypertension	10	1.055 (0.933 to 1.192)	0.422	1.033 (0.958 to 1.115)	0.400	1.039 (0.962 to 1.122)	0.334		
X-14745	Hypertension	6	0.464 (0.002 to 115.461)	0.799	0.838 (0.579 to 1.214)	0.349	0.949 (0.631 to 1.427)	0.801	0.793 (0.600 to 1.048)	0.178
Gamma-glutamylglutamate	Hypertension	8	1.127 (0.824 to 1.540)	0.482	1.073 (0.917 to 1.256)	0.381	1.045 (0.930 to 1.174)	0.461		
Octadecanedioate	Hypertension	5	1.475 (0.542 to 4.014)	0.502	0.975 (0.685 to 1.386)	0.886	1.029 (0.668 to 1.585)	0.897	0.775 (0.490 to 1.224)	0.472
Leucylleucine	Hypertension	8	0.916 (0.381 to 2.198)	0.850	1.165 (0.904 to 1.503)	0.238	1.085 (0.855 to 1.377)	0.502		

Exposure	Outcome	nSNP	MR Egger		Weighted median		Inverse variance weighted		MR_PRESSO	
			OR (95% CI)	pval	OR (95% CI)	pval	OR (95% CI)	pval	OR (95% CI)	pval
7-alpha-hydroxy-3-oxo-4-cholestenoate (7-Hoca)	Hypertension	8	0.842 (0.406 to 1.744)	0.659	0.973 (0.638 to 1.484)	0.899	0.861 (0.633 to 1.171)	0.339		
N-Butyl Oleate	Hypertension	5	12.781 (0.775 to 210.736)	0.173	1.205 (0.793 to 1.831)	0.382	1.080 (0.687 to 1.697)	0.739		
Dimethylarginine (SDMA + ADMA)	Hypertension	21	1.089 (0.290 to 4.090)	0.901	0.767 (0.498 to 1.182)	0.230	0.741 (0.470 to 1.169)	0.198	0.801 (0.523 to 1.226)	0.319
Taurolithocholate 3-sulfate	Hypertension	6	1.156 (0.765 to 1.748)	0.529	0.941 (0.792 to 1.118)	0.488	0.920 (0.730 to 1.159)	0.477	0.907 (0.754 to 1.091)	0.377
X-14977--vanillin	Hypertension	2					0.962 (0.728 to 1.270)	0.783		

Exposure	Outcome	nSNP	MR Egger		Weighted median		Inverse variance weighted		MR_PRESSO	
			OR (95% CI)	pval	OR (95% CI)	pval	OR (95% CI)	pval	OR (95% CI)	pval
Succinylcarnitine	Hypertension	24	1.465 (0.702 to 3.054)	0.320	1.276 (0.963 to 1.691)	0.090	0.947 (0.660 to 1.357)	0.766	0.693 (0.451 to 1.066)	0.110
Tryptophan betaine	Hypertension	7	0.804 (0.563 to 1.147)	0.282	0.950 (0.866 to 1.041)	0.269	0.916 (0.823 to 1.019)	0.106	0.956 (0.871 to 1.050)	0.392
Cyclo(leu-pro)	Hypertension	10	0.732 (0.550 to 0.973)	0.064	1.008 (0.863 to 1.177)	0.923	1.156 (0.988 to 1.352)	0.070	1.110 (0.976 to 1.261)	0.149
Chiro-inositol	Hypertension	6	1.056 (0.907 to 1.229)	0.521	1.002 (0.939 to 1.069)	0.954	0.983 (0.935 to 1.034)	0.509		
5alpha-androstan-3beta,17beta-diol disulfate	Hypertension	10	0.931 (0.786 to 1.103)	0.432	0.991 (0.919 to 1.070)	0.821	1.006 (0.913 to 1.107)	0.910		

Exposure	Outcome	nSNP	MR Egger		Weighted median		Inverse variance weighted		MR_PRESSO	
			OR (95% CI)	pval	OR (95% CI)	pval	OR (95% CI)	pval	OR (95% CI)	pval
4-androsten-3beta,17beta-diol disulfate 1*	Hypertension	15	0.911 (0.801 to 1.036)	0.178	0.919 (0.849 to 0.994)	0.035	0.905 (0.844 to 0.971)	0.005		
4-androsten-3beta,17beta-diol disulfate 2*	Hypertension	13	0.613 (0.354 to 1.061)	0.108	0.772 (0.639 to 0.933)	0.007	0.882 (0.747 to 1.041)	0.138		
2-hydroxyglutarate	Hypertension	5	0.982 (0.523 to 1.847)	0.960	0.957 (0.705 to 1.298)	0.776	0.916 (0.729 to 1.152)	0.455		
Ergothioneine	Hypertension	1								
Palmitoyl sphingomyelin	Hypertension	31	1.097 (0.254 to 4.728)	0.902	1.345 (0.923 to 1.958)	0.123	1.156 (0.785 to 1.703)	0.462	1.259 (0.898 to 1.766)	0.192
Phenylalanylphenylalanine	Hypertension	3	0.764 (0.101 to 5.812)	0.838	1.192 (0.855	0.300	1.238 (0.762	0.389		

Exposure	Outcome	nSNP	MR Egger		Weighted median		Inverse variance weighted		MR_PRESSO	
			OR (95% CI)	pval	OR (95% CI)	pval	OR (95% CI)	pval	OR (95% CI)	pval
					to 1.663)		to 2.011)			
Cis-4-decenoyl carnitine	Hypertension	5	1.223 (0.603 to 2.481)	0.616	1.166 (0.957 to 1.420)	0.127	1.137 (0.891 to 1.452)	0.302		
15-methylpalmitate (isobar with 2-methylpalmitate)	Hypertension	9	1.040 (0.398 to 2.716)	0.939	1.201 (0.836 to 1.726)	0.322	1.183 (0.893 to 1.568)	0.241		

n: 2187

Table S3. Instrumental variables strength and others used in MR analysis of the effect of metabolites on hypertension(UKB).

Exposure	Outcome	R2	F	Heterogeneity	Pleiotropy	power	Direction
arachidonate (20:4n6)	Hypertension	2	81	0.898	0.224	0.050	TRUE
deoxycholate	Hypertension	0.079	14	0.075	0.582	0.026	TRUE
arachidate (20:0)	Hypertension	0.107	13	0.160	0.402	0.050	TRUE
margarate (17:0)	Hypertension	0.067	13	0.082	0.359	0.034	TRUE
inosine	Hypertension	0.179	17	0.320	0.526	0.062	TRUE
myo-inositol	Hypertension	0.119	17	0.200	0.218	0.047	TRUE
isoleucine	Hypertension	0.097	15	0.012	0.059	0.043	TRUE
alanine	Hypertension	0.088	12	0.075	0.075	0.033	TRUE
3-methoxytyrosine	Hypertension	0.139	14	0.021	0.827	0.026	TRUE
beta-hydroxyisovalerate	Hypertension	0.199	15	0.003	0.537	0.029	TRUE
taurodeoxycholate	Hypertension	0.122	15	0.099	0.812	0.028	TRUE
threonine	Hypertension	0.115	16	2.02e-07	0.530	0.054	TRUE
tyrosine	Hypertension	0.111	13	0.033	0.230	0.027	TRUE
lysine	Hypertension	0.141	15	0.170	0.902	0.027	TRUE
malate	Hypertension	0.350	14	0.022	0.003	0.030	TRUE
palmitate (16:0)	Hypertension	0.206	14	0.332	0.701	0.026	TRUE

Exposure	Outcome	R2	F	Heterogeneity	Pleiotropy	power	Direction
myristate (14:0)	Hypertension	0.181	15	0.034	0.215	0.032	TRUE
kynurenate	Hypertension	0.146	16	0.727	0.977	0.035	TRUE
5,6-dihydrothymine	Hypertension	0.233	17	0.003	0.576	0.055	TRUE
5-methylthioadenosine (MTA)	Hypertension	0.136	16	2.31e-04	0.269	0.052	TRUE
2-hydroxyphenylacetate	Hypertension	0.150	16	0.099	0.459	0.026	TRUE
succinate	Hypertension	0.149	17	7.96e-05	0.755	0.032	TRUE
ornithine	Hypertension	0.158	15	0.777	0.756	0.026	TRUE
5-oxoproline	Hypertension	0.232	20	0.037	0.929	0.063	TRUE
N6,N6,N6-trimethyllysine	Hypertension	0.084	17	0.101	0.906	0.027	TRUE
orotate	Hypertension	0.287	37	0.096	0.869	0.027	TRUE
pantothenate	Hypertension	0.133	15	0.177	0.369	0.031	TRUE
glycerol	Hypertension	0.114	14	0.210	0.549	0.040	TRUE
kynurenine	Hypertension	0.135	15	2.91e-34	0.006	0.079	TRUE
sucrose	Hypertension	0.080	16	0.063	0.839	0.032	TRUE
tartarate	Hypertension	0.148	16	0.063	0.172	0.030	TRUE
carnitine	Hypertension	0.321	18	7.95e-05	0.028	0.030	TRUE
choline	Hypertension	0.141	15	0.439	0.072	0.032	TRUE

Exposure	Outcome	R2	F	Heterogeneity	Pleiotropy	power	Direction
4-acetamidobutanoate	Hypertension	0.178	22	0.054	0.741	0.067	TRUE
chenodeoxycholate	Hypertension	0.143	17	0.833	0.790	0.028	TRUE
citrate	Hypertension	0.127	15	0.810	0.666	0.026	TRUE
N1-methyladenosine	Hypertension	0.097	13	0.145	0.155	0.033	TRUE
3-aminoisobutyrate	Hypertension	0.339	32	0.671	0.555	0.041	TRUE
2-isopropylmalate	Hypertension	0.150	17	0.395	0.264	0.027	TRUE
vanillylmandelate (VMA)	Hypertension	0.153	18	0.257	0.054	0.037	TRUE
3-methylhistidine	Hypertension	0.228	17	1.42e-04	0.466	0.031	TRUE
xanthurenate	Hypertension	0.111	14	0.053	0.103	0.054	TRUE
4-guanidinobutanoate	Hypertension	0.272	22	6.24e-06	0.038	0.035	TRUE
5-hydroxylysine	Hypertension	0.278	19	1.87e-06	0.409	0.054	TRUE
pimelate (heptanedioate)	Hypertension	0.133	16	0.474	0.596	0.026	TRUE
cystathionine	Hypertension	0.103	15	0.271	0.331	0.032	TRUE
imidazole lactate	Hypertension	0.604	62	0.006	0.504	0.029	TRUE
glycerate	Hypertension	0.206	15	0.707	0.216	0.028	TRUE
guanosine	Hypertension	0.148	16	0.395	0.748	0.026	TRUE
suberate (octanedioate)	Hypertension	0.175	17	3.75e-05	0.213	0.052	TRUE

Exposure	Outcome	R2	F	Heterogeneity	Pleiotropy	power	Direction
methylsuccinate	Hypertension	0.285	32	0.064	0.638	0.040	TRUE
hippurate	Hypertension	0.158	16	0.076	0.238	0.038	TRUE
ethylmalonate	Hypertension	2.7	71	0.193	0.705	0.035	TRUE
ribitol	Hypertension	0.261	20	0.022	0.022	0.026	TRUE
N-acetylalanine	Hypertension	0.122	13	0.043	0.876	0.041	TRUE
malonate	Hypertension	0.310	16	0.007	0.165	0.040	TRUE
N-acetylmethionine	Hypertension	0.149	15	0.325	0.914	0.030	TRUE
phenylacetate	Hypertension	0.072	18	0.062	0.643	0.028	TRUE
urate	Hypertension	0.264	20	0.241	0.806	0.051	TRUE
ursodeoxycholate	Hypertension	0.149	16	0.136	0.012	0.032	TRUE
arginine	Hypertension	0.153	14	3.46e-14	0.336	0.035	TRUE
fumarate	Hypertension	0.128	14	0.243	0.906	0.028	TRUE
serine	Hypertension	0.180	20	1.59e-09	0.157	0.054	TRUE
valine	Hypertension	0.112	15	0.096	0.703	0.030	TRUE
urea	Hypertension	0.079	14	0.323	0.335	0.028	TRUE
cortisol	Hypertension	0.116	14	0.008	0.164	0.029	TRUE
cortisone	Hypertension	0.164	16	0.007	0.105	0.027	TRUE

Exposure	Outcome	R2	F	Heterogeneity	Pleiotropy	power	Direction
2-hydroxystearate	Hypertension	0.156	13	0.557	0.269	0.036	TRUE
retinol (Vitamin A)	Hypertension	0.124	15	0.007	0.008	0.056	TRUE
gamma-glutamylhistidine	Hypertension	0.117	15	0.372	0.543	0.035	TRUE
paraxanthine	Hypertension	0.190	18	0.489	0.649	0.047	TRUE
quinate	Hypertension	0.158	13	0.137	0.217	0.033	TRUE
indolelactate	Hypertension	0.225	15	0.632	0.616	0.034	TRUE
gamma-glutamylleucine	Hypertension	0.168	17	0.420	0.353	0.041	TRUE
methionine sulfoxide	Hypertension	0.463	16	0.368	0.708	0.030	TRUE
theobromine	Hypertension	0.129	15	5.98e-06	0.584	0.038	TRUE
theophylline	Hypertension	0.221	16	8.1e-04	0.993	0.031	TRUE
eicosapentaenoate (EPA; 20:5n3)	Hypertension	0.436	17	1.000	0.540	0.062	TRUE
glycocholate	Hypertension	0.088	14	0.234	0.120	0.032	TRUE
taurochenodeoxycholate	Hypertension	0.105	14	0.035	0.118	0.028	TRUE
taurocholate	Hypertension	0.093	13	0.009	0.376	0.029	TRUE
proline	Hypertension	0.282	15	2.72e-04	0.167	0.026	TRUE
1,2-dimyristoyl-GPC (14:0/14:0)	Hypertension	0.143	16	8.97e-04	0.024	0.029	TRUE
quinolinate	Hypertension	0.234	14	3.41e-36	0.013	0.051	TRUE

Exposure	Outcome	R2	F	Heterogeneity	Pleiotropy	power	Direction
1,2-distearoyl-GPC (18:0/18:0)	Hypertension	0.084	8.7	2.94e-04	0.154	0.032	TRUE
1-myristoyl-2-palmitoyl-GPC (14:0/16:0)	Hypertension	0.117	14	1.1e-05	0.362	0.036	TRUE
1-palmitoyl-2-oleoyl-GPE (16:0/18:1)	Hypertension	0.636	21	0.999	0.022	0.038	TRUE
2-arachidonoylglycerol (20:4)	Hypertension	0.525	23	0.689	0.121	0.048	TRUE
1-stearoyl-GPI (18:0)	Hypertension	0.234	15	0.325	0.145	0.031	TRUE
stearoyl sphingomyelin (d18:1/18:0)	Hypertension	0.213	15	0.045	0.352	0.039	TRUE
1-palmityl-GPC (O-16:0)	Hypertension	0.135	17	0.004	0.351	0.045	TRUE
1,5-anhydroglucitol (1,5-AG)	Hypertension	0.210	14	0.011	0.054	0.027	TRUE
oxalate (ethanedioate)	Hypertension	0.148	15	0.014	0.092	0.027	TRUE
erythritol	Hypertension	0.167	18	3.77e-05	0.468	0.028	TRUE
glycylglycine	Hypertension	0.276	20	7.46e-85	0.780	0.072	TRUE
1-oleoylglycerol (18:1)	Hypertension	0.112	17	0.003	0.187	0.026	TRUE
citrulline	Hypertension	0.120	14	0.111	0.618	0.027	TRUE
biliverdin	Hypertension	0.531	50	0.120	0.271	0.059	TRUE
3-hydroxyoctanoate	Hypertension	0.221	21	0.030	0.666	0.027	TRUE
2-hydroxyoctanoate	Hypertension	0.232	17	1.46e-08	0.741	0.068	TRUE
3-hydroxydecanoate	Hypertension	0.542	41	0.299	0.015	0.026	TRUE

Exposure	Outcome	R2	F	Heterogeneity	Pleiotropy	power	Direction
phenyllactate (PLA)	Hypertension	0.325	33	0.098	0.190	0.034	TRUE
alpha-hydroxyisocaproate	Hypertension	0.106	14	0.162	0.072	0.034	TRUE
homoarginine	Hypertension	0.320	27	5.93e-17	0.908	0.080	TRUE
homocitrulline	Hypertension	0.102	23	0.552	0.608	0.036	TRUE
N-acetylaspartate (NAA)	Hypertension	0.292	16	0.028	0.204	0.039	TRUE
pyroglutamylglutamine	Hypertension	0.174	16	0.026	0.261	0.029	TRUE
cholate	Hypertension	0.076	11	0.002	0.521	0.036	TRUE
gamma-glutamylglutamine	Hypertension	0.157	18	0.005	0.611	0.031	TRUE
gamma-glutamyltyrosine	Hypertension	0.112	15	0.956	0.558	0.037	TRUE
1-linoleoylglycerol (18:2)	Hypertension	0.205	16	1.91e-06	0.732	0.031	TRUE
thyroxine	Hypertension	0.090	14	0.144	0.838	0.030	TRUE
1-methylnicotinamide	Hypertension	0.091	14	0.147	0.588	0.039	TRUE
3-indoxyl sulfate	Hypertension	0.068	13	0.363	0.638	0.034	TRUE
N-acetylglycine	Hypertension	0.262	29	2.61e-13	0.395	0.040	TRUE
creatine	Hypertension	0.164	18	4.38e-07	0.009	0.102	TRUE
galactonate	Hypertension	0.148	15	5.16e-06	0.970	0.025	TRUE
1-methylhistidine	Hypertension	0.155	15	0.029	0.201	0.041	TRUE

Exposure	Outcome	R2	F	Heterogeneity	Pleiotropy	power	Direction
hypoxanthine	Hypertension	0.174	15	3.67e-43	0.523	0.067	TRUE
betaine	Hypertension	0.163	16	8.16e-10	0.074	0.032	TRUE
xanthine	Hypertension	0.102	13	1.22e-09	0.076	0.038	TRUE
pyroglutamylglycine	Hypertension	0.140	14	0.107	0.319	0.026	TRUE
DSGEGDFXAEGGGVR	Hypertension	0.236	15	1.59e-42	0.472	0.110	TRUE
3-ureidopropionate	Hypertension	0.075	9.9	0.702	0.255	0.035	TRUE
pyridoxate	Hypertension	0.176	14	0.034	0.667	0.027	TRUE
androsterone sulfate	Hypertension	0.576	34	2.91e-06	0.022	0.036	TRUE
2-methylbutyrylglycine	Hypertension	0.152	15	0.097	0.973	0.032	TRUE
propionylglycine	Hypertension	0.322	26	1.76e-13	0.023	0.079	TRUE
5-hydroxyhexanoate	Hypertension	0.104	16	0.013	0.745	0.028	TRUE
3-hydroxysebacate	Hypertension	0.368	26	0.995	0.561	0.047	TRUE
3-(4-hydroxyphenyl)lactate	Hypertension	0.133	15	0.019	0.508	0.039	TRUE
acetylcarnitine	Hypertension	0.198	14	0.003	0.049	0.031	TRUE
trans-4-hydroxyproline	Hypertension	0.118	14	0.020	0.649	0.069	TRUE
hexanoylcarnitine	Hypertension	0.507	37	0.001	0.011	0.049	TRUE
glycochenodeoxycholate	Hypertension	0.103	14	6.15e-07	0.697	0.034	TRUE

Exposure	Outcome	R2	F	Heterogeneity	Pleiotropy	power	Direction
4-imidazoleacetate	Hypertension	0.142	16	0.033	0.895	0.028	TRUE
1-methylimidazoleacetate	Hypertension	0.266	26	0.004	0.058	0.029	TRUE
N-acetylneuraminate	Hypertension	0.124	15	2.87e-05	0.302	0.028	TRUE
dodecanedioate	Hypertension	0.282	19	0.983	0.254	0.039	TRUE
N-acetyltyrosine	Hypertension	1.7	111	1.13e-06	0.793	0.080	TRUE
1,3-dimethylurate	Hypertension	0.174	16	1.33e-06	0.031	0.091	TRUE
trigonelline (N'-methylnicotinate)	Hypertension	0.109	13	0.072	0.783	0.026	TRUE
indolepropionate	Hypertension	0.171	16	7.3e-04	0.347	0.036	TRUE
butyrylcarnitine	Hypertension	2.2	83	3.17e-05	0.132	0.031	TRUE
myristoleate (14:1n5)	Hypertension	0.119	13	1.2e-06	0.518	0.092	TRUE
dehydroisoandrosterone sulfate (DHEA-S)	Hypertension	0.217	20	1.33e-06	0.216	0.036	TRUE
3-methylxanthine	Hypertension	0.107	16	0.568	0.223	0.031	TRUE
guanidinosuccinate	Hypertension	0.189	14	7.16e-16	0.733	0.033	TRUE
caproate (6:0)	Hypertension	0.187	15	0.386	0.405	0.027	TRUE
caprylate (8:0)	Hypertension	0.267	17	0.030	0.788	0.027	TRUE
10-undecenoate (11:1n1)	Hypertension	0.424	30	0.829	0.408	0.030	TRUE
docosapentaenoate (n3 DPA; 22:5n3)	Hypertension	0.301	14	0.990	0.972	0.026	TRUE

Exposure	Outcome	R2	F	Heterogeneity	Pleiotropy	power	Direction
phenol sulfate	Hypertension	0.107	16	0.373	0.629	0.040	TRUE
pregnen-diol disulfate	Hypertension	0.717	65	0.159	0.253	0.037	TRUE
X - 11261	Hypertension	0.258	17	0.078	0.546	0.028	TRUE
bilirubin (E,E)	Hypertension	0.425	41	0.354	0.554	0.025	TRUE
glycocholenate sulfate	Hypertension	0.626	29	0.005	0.741	0.027	TRUE
pregn steroid monosulfate	Hypertension	0.301	21	0.092	0.514	0.038	TRUE
glycolithocholate sulfate	Hypertension	0.183	18	0.594	0.921	0.027	TRUE
X - 11381	Hypertension	0.289	16	3.37e-10	0.148	0.100	TRUE
taurocholenate sulfate	Hypertension	0.334	20	0.141	0.521	0.039	TRUE
2-arachidonoyl-GPE (20:4)	Hypertension	0.783	30	0.250	0.164	0.038	TRUE
andro steroid monosulfate (1)	Hypertension	0.229	20	3.01e-04	0.103	0.038	TRUE
HWESASXX	Hypertension	0.158	16	0.073	0.037	0.026	TRUE
X - 11540	Hypertension	0.137	12	3.56e-15	0.555	0.033	TRUE
adrenate (22:4n6)	Hypertension	0.288	15	0.790	0.062	0.040	TRUE
X - 11787	Hypertension	0.776	53	3.93e-12	0.833	0.058	TRUE
X - 11795	Hypertension	0.123	13	0.124	0.930	0.025	TRUE
1-arachidonoyl-GPC (20:4n6)	Hypertension	2.8	137	0.985	0.461	0.051	TRUE

Exposure	Outcome	R2	F	Heterogeneity	Pleiotropy	power	Direction
1-palmitoleoyl-GPC (16:1)	Hypertension	0.087	14	2.99e-04	0.276	0.052	TRUE
gamma-glutamylthreonine	Hypertension	0.130	15	1.2e-09	0.115	0.058	TRUE
2-arachidonoyl-GPC (20:4)	Hypertension	2.2	94	0.991	0.330	0.051	TRUE
gamma-glutamylphenylalanine	Hypertension	0.072	18	0.585	0.527	0.025	TRUE
isobutyrylcarnitine	Hypertension	0.378	26	1.12e-07	0.864	0.058	TRUE
pseudouridine	Hypertension	0.192	15	0.092	0.691	0.028	TRUE
palmitoleate (16:1n7)	Hypertension	0.180	14	0.016	0.235	0.030	TRUE
1-dihomo-linolenoyl-GPC (20:3n3 or 6)	Hypertension	0.499	23	0.944	0.914	0.059	TRUE
1-docosahexaenoyl-GPC (22:6)	Hypertension	0.175	16	0.287	0.980	0.034	TRUE
1-dihomo-linoleoyl-GPC (20:2)	Hypertension	1.3	58	0.980	0.377	0.049	TRUE
gamma-glutamyl-epsilon-lysine	Hypertension	0.189	15	1.76e-05	0.517	0.050	TRUE
octanoylcarnitine	Hypertension	0.465	39	0.024	0.582	0.041	TRUE
decanoylcarnitine	Hypertension	0.365	29	0.015	0.285	0.055	TRUE
N-acetylasparagine	Hypertension	0.283	24	1.95e-06	1.7e-04	0.039	TRUE
N-acetylglutamine	Hypertension	1.8	144	0.002	0.277	0.110	TRUE
N-acetylhistidine	Hypertension	0.738	44	2.59e-19	2.81e-04	0.046	TRUE
gamma-glutamylglycine	Hypertension	0.234	18	3.79e-10	0.312	0.033	TRUE

Exposure	Outcome	R2	F	Heterogeneity	Pleiotropy	power	Direction
N-acetylphenylalanine	Hypertension	0.918	77	0.021	0.001	0.130	TRUE
myristoylcarnitine	Hypertension	0.298	17	4.14e-21	0.820	0.179	TRUE
glycylphenylalanine	Hypertension	0.362	23	5.05e-33	0.033	0.027	TRUE
1-stearoyl-GPC (18:0)	Hypertension	0.154	15	0.043	0.172	0.026	TRUE
5-dodecenoate (12:1n7)	Hypertension	0.088	13	1.67e-06	0.838	0.040	TRUE
10-nonadecenoate (19:1n9)	Hypertension	0.051	11	0.149	0.134	0.025	TRUE
epiandrosterone sulfate	Hypertension	0.220	18	1.92e-04	0.201	0.055	TRUE
1-arachidonoyl-GPI (20:4)	Hypertension	0.447	18	0.768	0.377	0.044	TRUE
2-docosahexaenoyl-GPE (22:6)	Hypertension	0.133	13	0.473	0.882	0.026	TRUE
stachydrine	Hypertension	0.115	13	1.3e-04	0.234	0.033	TRUE
1-methylxanthine	Hypertension	0.249	24	0.039	0.102	0.056	TRUE
7-methylxanthine	Hypertension	0.092	15	0.008	0.561	0.059	TRUE
1-linolenoylglycerol (18:3)	Hypertension	0.145	18	3.3e-08	0.032	0.027	TRUE
1-methylurate	Hypertension	0.251	22	0.002	0.387	0.029	TRUE
choline phosphate	Hypertension	0.152	16	0.002	0.393	0.036	TRUE
1,7-dimethylurate	Hypertension	0.209	15	0.064	0.538	0.028	TRUE
5-acetylamino-6-formylamino-3-methyluracil	Hypertension	0.450	42	5.17e-08	0.630	0.059	TRUE

Exposure	Outcome	R2	F	Heterogeneity	Pleiotropy	power	Direction
1,3,7-trimethylurate	Hypertension	0.164	15	1.89e-05	0.723	0.025	TRUE
isovalerylcarnitine	Hypertension	0.299	21	1.45e-27	0.203	0.175	TRUE
stearoylcarnitine	Hypertension	0.159	15	2.73e-07	0.137	0.030	TRUE
1-linoleoyl-GPC (18:2)	Hypertension	0.478	26	0.904	0.245	0.047	TRUE
5-acetylamino-6-amino-3-methyluracil	Hypertension	0.205	19	0.004	0.336	0.026	TRUE
1-palmitoyl-GPA (16:0)	Hypertension	0.141	16	0.661	0.552	0.031	TRUE
gamma-glutamylisoleucine	Hypertension	0.213	16	0.152	0.872	0.031	TRUE
laurylcarnitine	Hypertension	0.212	19	0.162	0.118	0.039	TRUE
7-methylguanine	Hypertension	0.169	15	0.209	0.704	0.040	TRUE
phenylacetylglutamine	Hypertension	0.103	16	7.7e-04	0.885	0.027	TRUE
pro-hydroxy-pro	Hypertension	0.178	15	0.438	0.549	0.031	TRUE
5-methyluridine (ribothymidine)	Hypertension	0.348	31	0.034	0.619	0.041	TRUE
N2,N2-dimethylguanosine	Hypertension	0.077	15	0.064	0.281	0.025	TRUE
1-docosahexaenoylglycerol (22:6)	Hypertension	0.175	20	0.017	0.302	0.029	TRUE
N6-carbamoylthreonyladenosine	Hypertension	0.108	14	9.73e-07	0.431	0.027	TRUE
oleoylcarnitine	Hypertension	0.185	14	4.32e-31	0.267	0.035	TRUE
orotidine	Hypertension	0.321	27	0.057	0.407	0.043	TRUE

Exposure	Outcome	R2	F	Heterogeneity	Pleiotropy	power	Direction
1-arachidonoyl-GPE (20:4n6)	Hypertension	0.735	19	0.970	0.681	0.026	TRUE
2-palmitoyl-GPC (16:0)	Hypertension	0.175	14	2.41e-05	0.968	0.029	TRUE
2-linoleoyl-GPC (18:2)	Hypertension	0.098	13	0.117	0.292	0.030	TRUE
tiglylcarnitine	Hypertension	0.190	15	0.064	0.210	0.062	TRUE
hexanoylglycine	Hypertension	0.281	22	1.72e-10	0.238	0.037	TRUE
2-methylmalonyl carnitine	Hypertension	0.123	15	0.021	0.570	0.028	TRUE
1-myristoylglycerol (14:0)	Hypertension	0.200	16	7.69e-05	0.616	0.031	TRUE
1-oleoyl-GPE (18:1)	Hypertension	0.225	11	0.960	0.093	0.031	TRUE
1-palmitoyl-GPE (16:0)	Hypertension	0.541	25	0.743	0.008	0.028	TRUE
cysteinylglycine	Hypertension	0.164	16	4.11e-28	0.217	0.058	TRUE
tetradecanedioate	Hypertension	0.863	32	0.827	0.551	0.037	TRUE
2-hydroxypalmitate	Hypertension	0.133	14	0.666	0.529	0.033	TRUE
hexadecanedioate	Hypertension	0.493	28	0.200	0.470	0.028	TRUE
dihomo-linolenate (20:3n3 or n6)	Hypertension	0.439	17	0.861	0.419	0.039	TRUE
4-vinylphenol sulfate	Hypertension	0.156	13	0.931	0.677	0.026	TRUE
p-cresol sulfate	Hypertension	0.088	12	5.8e-09	0.724	0.033	TRUE
2-linoleoyl-GPE (18:2)	Hypertension	0.663	25	0.542	0.941	0.045	TRUE

Exposure	Outcome	R2	F	Heterogeneity	Pleiotropy	power	Direction
1-linoleoyl-GPI (18:2)	Hypertension	0.547	23	0.119	0.005	0.029	TRUE
1-linoleoyl-GPE (18:2)	Hypertension	1.2	45	0.898	0.179	0.042	TRUE
1-oleoyl-GPI (18:1)	Hypertension	0.169	15	0.031	0.243	0.028	TRUE
gamma-glutamylglutamate	Hypertension	0.246	14	0.154	0.403	0.026	TRUE
2-hydroxy-3-methylvalerate	Hypertension	0.155	13	0.233	0.430	0.038	TRUE
deoxycarnitine	Hypertension	0.289	22	5.41e-05	0.392	0.031	TRUE
3-methyladipate	Hypertension	0.136	16	4.94e-07	5.93e-06	0.119	TRUE
N6-acetyllysine	Hypertension	0.145	18	0.175	0.208	0.031	TRUE
octadecanedioate	Hypertension	0.122	14	2.43e-05	0.837	0.027	TRUE
7-alpha-hydroxy-3-oxo-4-cholestenoate (7-Hoca)	Hypertension	0.095	13	0.089	0.563	0.030	TRUE
dimethylarginine (SDMA + ADMA)	Hypertension	0.167	18	0.025	0.038	0.028	TRUE
o-cresol sulfate	Hypertension	0.102	14	0.984	0.714	0.039	TRUE
taurolithocholate 3-sulfate	Hypertension	0.118	15	6.1e-07	0.252	0.037	TRUE
succinylcarnitine	Hypertension	0.255	23	0.007	0.051	0.068	TRUE
gamma-glutamylalanine	Hypertension	0.148	15	3.27e-17	0.061	0.060	TRUE
N-acetylserine	Hypertension	0.132	15	0.947	0.488	0.041	TRUE
gamma-glutamyl-2-aminobutyrate	Hypertension	0.104	15	0.021	0.156	0.054	TRUE

Exposure	Outcome	R2	F	Heterogeneity	Pleiotropy	power	Direction
tryptophan betaine	Hypertension	0.096	15	0.181	0.936	0.039	TRUE
cyclo(leu-pro)	Hypertension	0.156	15	7.1e-10	0.321	0.041	TRUE
5alpha-androstan-3alpha,17beta-diol monosulfate (1)	Hypertension	0.220	18	0.068	0.014	0.072	TRUE
5alpha-androstan-3beta,17beta-diol disulfate	Hypertension	0.552	38	5.55e-10	0.120	0.033	TRUE
5alpha-pregnan-3beta,20alpha-diol disulfate	Hypertension	0.180	17	0.475	0.204	0.044	TRUE
5alpha-pregnan-3beta,20alpha-diol monosulfate (2)	Hypertension	0.078	15	9.43e-05	0.418	0.030	TRUE
4-androsten-3beta,17beta-diol disulfate (1)	Hypertension	0.477	39	7.92e-06	0.886	0.039	TRUE
4-androsten-3beta,17beta-diol disulfate (2)	Hypertension	0.134	13	0.831	0.466	0.025	TRUE
4-androsten-3alpha,17alpha-diol monosulfate (2)	Hypertension	0.134	13	9.36e-04	0.776	0.026	TRUE
4-androsten-3alpha,17alpha-diol monosulfate (3)	Hypertension	0.553	27	7.03e-09	0.021	0.036	TRUE
4-androsten-3beta,17beta-diol monosulfate (2)	Hypertension	0.230	20	7.55e-18	0.275	0.050	TRUE
4-androsten-3beta,17beta-diol monosulfate (1)	Hypertension	0.145	16	0.016	0.288	0.077	TRUE
1-docosapentaenoyl-GPC (22:5n3)	Hypertension	0.417	19	0.975	0.064	0.038	TRUE
1-pentadecanoyl-GPC (15:0)	Hypertension	0.107	14	0.062	0.732	0.073	TRUE
N-methylproline	Hypertension	0.115	15	0.651	0.762	0.030	TRUE
N-acetyl-beta-alanine	Hypertension	0.152	18	0.023	0.972	0.029	TRUE
cysteine sulfinic acid	Hypertension	0.108	13	7.49e-07	0.572	0.039	TRUE

Exposure	Outcome	R2	F	Heterogeneity	Pleiotropy	power	Direction
ergothioneine	Hypertension	0.092	14	0.685	0.616	0.045	TRUE
N-acetylputrescine	Hypertension	0.348	39	3.26e-05	0.604	0.034	TRUE
palmitoyl sphingomyelin (d18:1/16:0)	Hypertension	0.089	16	0.209	0.157	0.050	TRUE
sphingomyelin (d18:1/18:1, d18:2/18:0)	Hypertension	0.356	15	0.959	0.029	0.034	TRUE
13-HODE + 9-HODE	Hypertension	0.323	16	2.46e-04	0.103	0.033	TRUE
oleoyl ethanolamide	Hypertension	0.153	16	0.363	0.466	0.039	TRUE
S-methylmethionine	Hypertension	0.098	10	0.055	0.083	0.030	TRUE
phenylalanylphenylalanine	Hypertension	0.141	16	0.428	0.333	0.033	TRUE
16a-hydroxy DHEA 3-sulfate	Hypertension	0.403	29	0.012	0.022	0.088	TRUE
pregnenolone sulfate	Hypertension	0.225	15	0.379	0.163	0.033	TRUE
cis-4-decenoyl carnitine	Hypertension	0.381	23	0.008	0.188	0.060	TRUE
17-methylstearate	Hypertension	0.114	13	0.053	0.474	0.030	TRUE
cinnamoylglycine	Hypertension	0.282	17	5.26e-11	0.443	0.026	TRUE
3-methylglutaconate	Hypertension	0.313	24	5.27e-08	0.032	0.062	TRUE
15-methylpalmitate	Hypertension	0.079	14	2.97e-04	0.603	0.025	TRUE
2-stearoyl-GPI (18:0)	Hypertension	0.205	14	0.320	0.172	0.026	TRUE
glycoursodeoxycholate	Hypertension	0.061	15	0.019	0.549	0.040	TRUE

Exposure	Outcome	R2	F	Heterogeneity	Pleiotropy	power	Direction
S-methylcysteine	Hypertension	0.203	22	0.023	0.844	0.031	TRUE
leucylalanine	Hypertension	0.242	15	1.06e-109	0.044	0.117	TRUE
phenylalanylserine	Hypertension	0.450	37	1.49e-70	0.018	0.080	TRUE
alpha-glutamyltyrosine	Hypertension	0.386	29	2.1e-114	0.090	0.039	TRUE
isoleucylvaline	Hypertension	0.156	19	9.83e-05	0.501	0.072	TRUE
X - 17299	Hypertension	0.156	15	4.58e-05	0.830	0.046	TRUE
N1-Methyl-2-pyridone-5-carboxamide	Hypertension	0.080	13	0.240	0.292	0.028	TRUE
hydantoin-5-propionic acid	Hypertension	0.155	15	0.021	0.340	0.028	TRUE
dihydroferulic acid	Hypertension	0.095	16	0.506	0.731	0.025	TRUE
pregnanediol-3-glucuronide	Hypertension	0.122	16	0.383	0.080	0.032	TRUE
imidazole propionate	Hypertension	0.121	17	0.043	0.187	0.026	TRUE
prolylproline	Hypertension	0.287	42	2.68e-05	0.717	0.124	TRUE
phenylalanyltryptophan	Hypertension	0.191	17	0.649	0.440	0.033	TRUE
alpha-glutamylglycine	Hypertension	0.382	22	6.88e-83	0.469	0.041	TRUE
histidyltryptophan	Hypertension	0.137	14	0.260	0.353	0.040	TRUE
serylalanine	Hypertension	0.247	17	2.41e-38	0.193	0.042	TRUE
valylarginine	Hypertension	0.268	17	7.76e-92	0.233	0.264	TRUE

Exposure	Outcome	R2	F	Heterogeneity	Pleiotropy	power	Direction
indoleacetylglutamine	Hypertension	0.152	14	0.020	0.828	0.051	TRUE
N-palmitoylglycine	Hypertension	0.311	21	4.33e-12	0.824	0.092	TRUE
2-aminobutyrate	Hypertension	0.118	16	0.026	0.237	0.055	TRUE
1-stearoyl-GPE (18:0)	Hypertension	0.519	29	0.296	0.040	0.026	TRUE
erythronate	Hypertension	0.141	16	0.056	0.709	0.055	TRUE
1-palmitoyl-2-linoleoyl-GPC (16:0/18:2)	Hypertension	0.845	34	0.999	0.077	0.053	TRUE
1-stearoyl-2-oleoyl-GPE (18:0/18:1)	Hypertension	0.378	21	0.718	0.556	0.026	TRUE
1-palmitoyl-2-linoleoyl-GPE (16:0/18:2)	Hypertension	1.6	56	0.994	0.093	0.047	TRUE
1-stearoyl-2-arachidonoyl-GPC (18:0/20:4)	Hypertension	3.6	160	0.996	0.870	0.053	TRUE
sphingomyelin (d18:2/16:0, d18:1/16:1)	Hypertension	0.065	12	0.021	0.847	0.026	TRUE
sphingomyelin (d18:1/14:0, d16:1/16:0)	Hypertension	0.318	28	2.02e-04	0.023	0.068	TRUE
2-hydroxydecanoate	Hypertension	0.089	16	0.084	0.201	0.032	TRUE
dimethylmalonic acid	Hypertension	0.121	14	0.016	0.594	0.031	TRUE
N-delta-acetylornithine	Hypertension	3.7	291	4.46e-07	0.411	0.080	TRUE
N-acetyl-1-methylhistidine	Hypertension	1.1	95	1.9e-06	0.094	0.046	TRUE
N-acetyl-3-methylhistidine	Hypertension	0.642	50	0.006	0.029	0.114	TRUE
2-aminooctanoate	Hypertension	1.2	85	4.42e-04	0.736	0.093	TRUE

Exposure	Outcome	R2	F	Heterogeneity	Pleiotropy	power	Direction
N-acetylcarnosine	Hypertension	0.291	22	0.659	0.243	0.031	TRUE
5-hydroxyindoleacetate	Hypertension	0.152	18	0.328	0.949	0.033	TRUE
2-aminoheptanoate	Hypertension	0.085	15	0.002	0.935	0.036	TRUE
guanidinoacetate	Hypertension	0.227	17	0.009	0.091	0.063	TRUE
bilirubin (Z,Z)	Hypertension	0.392	43	0.205	0.489	0.032	TRUE
gamma-glutamylvaline	Hypertension	0.177	16	0.014	0.535	0.035	TRUE
glycerol 3-phosphate	Hypertension	0.131	16	0.058	0.453	0.033	TRUE
aspartate	Hypertension	0.163	16	1.14e-11	0.177	0.037	TRUE
1-eicosenoyl-GPC (20:1)	Hypertension	0.096	15	2.62e-06	0.252	0.040	TRUE
1-eicosapentaenoyl-GPC (20:5)	Hypertension	0.640	19	0.589	0.694	0.045	TRUE
4-acetylphenol sulfate	Hypertension	0.106	16	8e-06	0.028	0.029	TRUE
1-dihomo-linolenoyl-GPE (20:3n3 or 6)	Hypertension	0.515	18	0.814	0.161	0.027	TRUE
1-docosahexaenoyl-GPE (22:6)	Hypertension	0.251	16	0.208	0.274	0.034	TRUE
isovalerate	Hypertension	0.377	16	7.94e-06	0.774	0.053	TRUE
glutarylcarnitine (C5)	Hypertension	0.290	17	1.81e-11	0.237	0.038	TRUE
docosahexaenoate (DHA; 22:6n3)	Hypertension	0.124	16	0.949	0.519	0.032	TRUE
palmitoylcarnitine	Hypertension	0.192	13	3.12e-23	0.022	0.057	TRUE

Exposure	Outcome	R2	F	Heterogeneity	Pleiotropy	power	Direction
1-margaroyl-GPC (17:0)	Hypertension	0.141	16	0.211	0.779	0.030	TRUE
gamma-glutamylmethionine	Hypertension	0.179	16	0.051	0.497	0.060	TRUE
gamma-CEHC	Hypertension	0.098	18	0.008	0.453	0.027	TRUE
N-palmitoyl-sphingosine (d18:1/16:0)	Hypertension	0.119	16	0.033	0.835	0.036	TRUE
methionine sulfone	Hypertension	0.973	63	6.03e-39	0.345	0.058	TRUE
2-methylbutyrylcarnitine (C5)	Hypertension	0.126	17	0.144	0.166	0.051	TRUE
O-sulfo-L-tyrosine	Hypertension	0.171	15	0.177	0.063	0.059	TRUE
3-(3-hydroxyphenyl)propionate sulfate	Hypertension	0.080	16	0.197	0.705	0.030	TRUE
2-palmitoyl-GPE (16:0)	Hypertension	0.452	27	0.792	0.722	0.025	TRUE
1-myristoyl-GPC (14:0)	Hypertension	0.087	13	0.195	0.391	0.027	TRUE
1-arachidoyl-GPC (20:0)	Hypertension	0.081	13	6.71e-08	0.025	0.028	TRUE
1-docosapentaenoyl-GPC (22:5n6)	Hypertension	0.342	14	0.048	0.780	0.029	TRUE
21-hydroxypregnenolone disulfate	Hypertension	0.267	20	2.18e-12	0.183	0.057	TRUE
mannitol/sorbitol	Hypertension	0.125	16	0.006	0.326	0.040	TRUE
methyl glucopyranoside (alpha + beta)	Hypertension	0.414	35	0.145	0.282	0.031	TRUE
4-methylcatechol sulfate	Hypertension	0.153	13	2.9e-04	0.948	0.044	TRUE
3-methyl catechol sulfate (1)	Hypertension	0.100	17	0.003	0.457	0.039	TRUE

Exposure	Outcome	R2	F	Heterogeneity	Pleiotropy	power	Direction
2-docosahexaenoyl-GPC (22:6)	Hypertension	0.092	17	0.421	0.408	0.028	TRUE
linoleoylcarnitine	Hypertension	0.209	22	1.96e-06	0.326	0.031	TRUE
pyroglutamine	Hypertension	0.238	16	1.66e-09	0.425	0.051	TRUE
X - 11564	Hypertension	0.158	14	0.011	0.236	0.032	TRUE
X - 15486	Hypertension	0.223	17	1.38e-23	0.990	0.086	TRUE
X - 21286	Hypertension	0.140	13	2.49e-09	0.109	0.048	TRUE
1-arachidonoyl-GPA (20:4)	Hypertension	0.127	15	0.164	0.752	0.032	TRUE
X - 11441	Hypertension	0.347	28	0.084	0.755	0.031	TRUE
X - 21319	Hypertension	0.179	15	0.149	0.863	0.033	TRUE
X - 18249	Hypertension	0.170	19	0.250	0.928	0.030	TRUE
X - 11905	Hypertension	0.631	30	0.327	0.188	0.027	TRUE
X - 18914	Hypertension	0.202	19	0.016	0.356	0.032	TRUE
X - 21339	Hypertension	0.235	16	0.003	0.580	0.028	TRUE
X - 21341	Hypertension	0.078	14	0.172	0.414	0.048	TRUE
X - 21343	Hypertension	0.315	18	8.93e-09	0.688	0.026	TRUE
X - 11308	Hypertension	0.166	16	0.358	0.272	0.028	TRUE
X - 21353	Hypertension	0.499	29	0.550	0.562	0.043	TRUE

Exposure	Outcome	R2	F	Heterogeneity	Pleiotropy	power	Direction
X - 17178	Hypertension	0.530	25	1.7e-88	0.324	0.095	TRUE
X - 21365	Hypertension	0.174	19	3.1e-08	0.981	0.050	TRUE
X - 17189	Hypertension	0.147	13	8.7e-37	0.317	0.090	TRUE
X - 11442	Hypertension	0.434	29	5.85e-13	0.572	0.031	TRUE
X - 21411	Hypertension	0.279	18	1.26e-04	0.065	0.077	TRUE
X - 12511	Hypertension	1	89	1.82e-04	0.465	0.119	TRUE
X - 01911	Hypertension	0.094	14	0.029	0.536	0.031	TRUE
X - 11444	Hypertension	0.199	23	0.302	0.694	0.033	TRUE
X - 12824	Hypertension	0.448	29	3.63e-30	0.098	0.092	TRUE
X - 15497	Hypertension	0.099	14	0.683	0.951	0.038	TRUE
X - 21441	Hypertension	0.162	14	0.216	0.453	0.034	TRUE
X - 17185	Hypertension	0.135	15	0.515	0.776	0.053	TRUE
X - 21448	Hypertension	0.619	68	3.93e-04	0.201	0.030	TRUE
X - 02269	Hypertension	0.280	21	4.86e-06	0.146	0.105	TRUE
X - 02249	Hypertension	0.192	17	0.002	0.645	0.031	TRUE
X - 11850	Hypertension	0.070	13	0.212	0.830	0.033	TRUE
X - 16946	Hypertension	0.565	48	0.559	0.664	0.038	TRUE

Exposure	Outcome	R2	F	Heterogeneity	Pleiotropy	power	Direction
alpha-hydroxyisovalerate	Hypertension	0.247	14	0.127	0.013	0.028	TRUE
3-methylglutarylcarnitine (1)	Hypertension	0.160	20	2.28e-05	0.698	0.037	TRUE
3-methylglutarylcarnitine (2)	Hypertension	0.218	23	5.47e-16	0.596	0.032	TRUE
X - 12063	Hypertension	0.570	32	3e-06	0.015	0.025	TRUE
X - 07765	Hypertension	0.066	14	0.058	0.784	0.028	TRUE
X - 11299	Hypertension	0.064	18	0.551	0.530	0.031	TRUE
X - 11305	Hypertension	0.077	14	0.025	0.453	0.026	TRUE
X - 11372	Hypertension	0.165	15	0.388	0.976	0.053	TRUE
X - 11429	Hypertension	0.151	15	0.152	0.022	0.039	TRUE
X - 11438	Hypertension	0.089	13	0.566	0.187	0.048	TRUE
X - 11440	Hypertension	0.618	47	6e-04	0.026	0.043	TRUE
X - 11452	Hypertension	0.065	16	0.066	0.157	0.052	TRUE
X - 11470	Hypertension	0.285	16	0.144	0.098	0.061	TRUE
X - 11478	Hypertension	0.197	18	0.084	0.767	0.055	TRUE
X - 11530	Hypertension	0.445	48	0.088	0.257	0.038	TRUE
X - 11538	Hypertension	0.759	36	0.367	0.704	0.098	TRUE
X - 11880	Hypertension	0.150	13	0.184	0.736	0.029	TRUE

Exposure	Outcome	R2	F	Heterogeneity	Pleiotropy	power	Direction
X - 12007	Hypertension	0.123	15	0.234	0.818	0.026	TRUE
X - 12206	Hypertension	0.242	18	0.128	0.009	0.030	TRUE
X - 12216	Hypertension	0.075	13	0.007	0.327	0.025	TRUE
X - 12230	Hypertension	0.138	15	0.095	0.152	0.059	TRUE
X - 12411	Hypertension	0.166	16	0.004	0.346	0.028	TRUE
5,8-tetradecadienoate	Hypertension	0.249	16	0.334	0.602	0.031	TRUE
X - 12450	Hypertension	0.120	16	9.35e-05	0.718	0.035	TRUE
X - 12729	Hypertension	0.118	13	0.041	0.147	0.026	TRUE
X - 12798	Hypertension	0.662	40	0.119	0.278	0.052	TRUE
X - 12472	Hypertension	0.233	17	0.029	0.905	0.032	TRUE
X - 12816	Hypertension	0.086	13	0.018	0.586	0.029	TRUE
X - 14056	Hypertension	0.146	15	0.340	0.114	0.028	TRUE
X - 12844	Hypertension	0.178	17	0.029	0.653	0.030	TRUE
X - 12846	Hypertension	0.206	19	0.980	0.946	0.039	TRUE
X - 15469	Hypertension	0.133	18	0.231	0.577	0.027	TRUE
X - 13529	Hypertension	0.146	15	0.016	0.282	0.030	TRUE
X - 16132	Hypertension	0.107	14	0.173	0.511	0.033	TRUE

Exposure	Outcome	R2	F	Heterogeneity	Pleiotropy	power	Direction
X - 14568	Hypertension	0.182	15	0.088	0.300	0.035	TRUE
X - 14939	Hypertension	0.258	20	0.478	0.629	0.031	TRUE
X - 15245	Hypertension	0.097	17	0.473	0.131	0.031	TRUE
X - 15492	Hypertension	0.216	17	0.570	0.284	0.027	TRUE
X - 15728	Hypertension	0.042	10	0.049	0.260	0.039	TRUE
X - 16123	Hypertension	0.183	14	0.011	0.538	0.050	TRUE
X - 16935	Hypertension	0.422	25	0.194	0.182	0.026	TRUE
X - 16944	Hypertension	0.172	15	0.020	0.568	0.030	TRUE
X - 17145	Hypertension	0.068	12	0.761	0.152	0.030	TRUE
X - 18901	Hypertension	0.094	19	0.015	0.913	0.034	TRUE
X - 17166	Hypertension	0.277	21	1.8e-37	0.834	0.041	TRUE
X - 17167	Hypertension	0.160	18	0.947	0.488	0.038	TRUE
X - 18913	Hypertension	0.059	12	0.788	0.704	0.034	TRUE
X - 21628	Hypertension	0.234	16	8.97e-18	0.050	0.120	TRUE
X - 16134	Hypertension	0.244	17	0.226	0.365	0.027	TRUE
X - 17179	Hypertension	0.488	41	0.066	0.708	0.032	TRUE
X - 19141	Hypertension	1.2	87	9.65e-17	0.019	0.030	TRUE

Exposure	Outcome	R2	F	Heterogeneity	Pleiotropy	power	Direction
X - 17438	Hypertension	0.082	16	0.954	0.282	0.039	TRUE
X - 21666	Hypertension	0.089	15	0.273	0.347	0.029	TRUE
X - 12855	Hypertension	0.127	15	0.062	0.631	0.041	TRUE
oleoyl-linoleoyl-glycerol (18:1/18:2) [1]	Hypertension	0.359	17	5.27e-11	0.771	0.028	TRUE
oleoyl-linoleoyl-glycerol (18:1/18:2) [2]	Hypertension	0.175	20	1.8e-08	0.562	0.035	TRUE
X - 12748	Hypertension	0.165	19	0.022	0.288	0.025	TRUE
X - 21735	Hypertension	0.331	21	9.6e-06	0.011	0.038	TRUE
X - 21736	Hypertension	0.158	15	0.006	0.845	0.030	TRUE
X - 21737	Hypertension	0.087	17	0.164	0.492	0.027	TRUE
X - 11522	Hypertension	0.619	63	0.311	0.524	0.040	TRUE
gulonic acid	Hypertension	0.083	14	0.055	0.130	0.043	TRUE
sulfate	Hypertension	0.146	17	0.081	0.682	0.029	TRUE
X - 15503	Hypertension	0.111	14	0.983	0.954	0.032	TRUE
X - 12822	Hypertension	0.255	24	5.76e-27	0.597	0.066	TRUE
X - 21821	Hypertension	0.109	15	0.026	0.063	0.034	TRUE
X - 17327	Hypertension	0.219	17	0.105	0.553	0.040	TRUE
X - 16570	Hypertension	0.253	30	9.1e-10	0.711	0.105	TRUE

Exposure	Outcome	R2	F	Heterogeneity	Pleiotropy	power	Direction
X - 21849	Hypertension	0.242	20	0.481	0.234	0.027	TRUE
X - 21892	Hypertension	0.182	14	0.033	0.121	0.033	TRUE
1-nonadecanoyl-GPC (19:0)	Hypertension	0.123	13	0.024	0.199	0.032	TRUE
etiocholanolone glucuronide	Hypertension	0.328	26	1.08e-07	0.911	0.037	TRUE
2-palmitoleoyl-GPC (16:1)	Hypertension	0.084	9.7	7.29e-04	0.716	0.062	TRUE
sphingomyelin (d18:1/24:1, d18:2/24:0)	Hypertension	0.233	13	1.19e-04	0.008	0.028	TRUE
sphingomyelin (d18:2/14:0, d18:1/14:1)	Hypertension	0.131	18	0.010	0.130	0.025	TRUE
X - 12680	Hypertension	0.130	16	5.92e-06	0.036	0.038	TRUE
X - 22147	Hypertension	0.106	15	0.050	0.460	0.051	TRUE
X - 22162	Hypertension	0.426	46	0.252	0.267	0.027	TRUE
X - 10458	Hypertension	0.028	14	0.104	0.750	0.028	TRUE
X - 11537	Hypertension	0.110	12	0.087	0.032	0.041	TRUE
X - 22379	Hypertension	0.530	35	0.005	0.148	0.025	TRUE
X - 11871	Hypertension	0.072	16	0.055	0.277	0.026	TRUE
X - 12101	Hypertension	0.262	20	2.58e-04	0.450	0.040	TRUE
X - 11483	Hypertension	0.087	17	0.674	0.864	0.045	TRUE
X - 11491	Hypertension	1.1	54	0.003	0.744	0.036	TRUE

Exposure	Outcome	R2	F	Heterogeneity	Pleiotropy	power	Direction
X - 12231	Hypertension	0.129	14	0.025	0.530	0.031	TRUE
X - 12830	Hypertension	0.094	15	0.515	0.117	0.030	TRUE
X - 18899	Hypertension	0.125	15	3.4e-05	0.073	0.034	TRUE
5-hydroxytryptophan	Hypertension	0.174	14	8.88e-04	0.938	0.034	TRUE
X - 12127	Hypertension	0.137	14	0.046	0.412	0.055	TRUE
X - 12339	Hypertension	0.098	12	0.016	0.042	0.037	TRUE
X - 12681	Hypertension	0.197	14	2.33e-24	0.717	0.094	TRUE
X - 12686	Hypertension	0.195	15	0.103	0.004	0.041	TRUE
X - 12688	Hypertension	0.237	20	0.072	0.101	0.053	TRUE
X - 14838	Hypertension	0.188	14	0.003	0.359	0.045	TRUE
nonanoylcarnitine	Hypertension	0.367	37	0.122	0.786	0.030	TRUE
X - 15666	Hypertension	0.131	15	0.340	0.901	0.044	TRUE
X - 16071	Hypertension	0.173	29	0.897	0.160	0.037	TRUE
X - 16580	Hypertension	0.125	15	0.014	0.667	0.047	TRUE
X - 17337	Hypertension	0.289	18	0.106	0.440	0.030	TRUE
1-eicosapentaenoyl-GPE (20:5)	Hypertension	0.192	16	0.706	0.888	0.036	TRUE
X - 12026	Hypertension	0.109	14	0.727	0.455	0.030	TRUE

Exposure	Outcome	R2	F	Heterogeneity	Pleiotropy	power	Direction
X - 12543	Hypertension	0.089	11	2.48e-08	0.775	0.032	TRUE
X - 17010	Hypertension	0.313	15	0.115	0.138	0.030	TRUE
X - 17351	Hypertension	0.094	15	0.575	0.914	0.038	TRUE
X - 22764	Hypertension	0.206	20	0.026	0.441	0.064	TRUE
glucose	Hypertension	0.146	13	0.232	0.155	0.034	TRUE
mannose	Hypertension	0.194	20	0.003	0.893	0.025	TRUE
X - 10358	Hypertension	0.127	15	0.304	0.468	0.032	TRUE
X - 22816	Hypertension	0.165	16	0.289	0.212	0.033	TRUE
X - 22822	Hypertension	0.097	15	5.79e-06	0.960	0.025	TRUE
myristoleoylcarnitine	Hypertension	0.109	12	0.783	0.436	0.028	TRUE
N-acetyltaurine	Hypertension	0.151	15	0.027	0.644	0.034	TRUE
X - 12695	Hypertension	0.191	16	3.87e-04	0.004	0.035	TRUE
arabonate/xylonate	Hypertension	0.119	15	0.960	0.733	0.043	TRUE
2-oleoyl-GPC (18:1)	Hypertension	0.137	14	0.358	0.558	0.028	TRUE
X - 24435	Hypertension	0.326	28	0.024	0.563	0.035	TRUE
dopamine sulfate (2)	Hypertension	0.250	18	0.003	0.742	0.043	TRUE
tyramine O-sulfate	Hypertension	0.115	15	0.238	0.026	0.029	TRUE

Exposure	Outcome	R2	F	Heterogeneity	Pleiotropy	power	Direction
methyl-4-hydroxybenzoate sulfate	Hypertension	0.055	18	0.452	0.600	0.033	TRUE
N-acetylcitrulline	Hypertension	3.4	298	9.98e-05	0.517	0.079	TRUE
4-hydroxychlorothalonil	Hypertension	0.181	17	0.201	0.385	0.026	TRUE
3-hydroxypyridine sulfate	Hypertension	0.122	12	5.91e-04	0.770	0.026	TRUE
sphingomyelin (d18:1/20:0, d16:1/22:0)	Hypertension	0.210	19	1.56e-17	0.084	0.031	TRUE
sphingomyelin (d18:1/20:1, d18:2/20:0)	Hypertension	0.230	17	0.710	0.942	0.029	TRUE
behenoyl sphingomyelin (d18:1/22:0)	Hypertension	0.146	15	1.13e-09	0.808	0.028	TRUE
X - 23026	Hypertension	0.156	15	0.003	0.577	0.054	TRUE
2-acetamidophenol sulfate	Hypertension	0.063	12	0.278	0.465	0.028	TRUE
1,2,3-benzenetriol sulfate (2)	Hypertension	0.085	19	0.023	0.417	0.051	TRUE
C-glycosyltryptophan	Hypertension	0.149	16	0.013	0.902	0.049	TRUE
p-cresol-glucuronide	Hypertension	0.089	12	0.142	0.638	0.029	TRUE
arabitol/xylitol	Hypertension	0.176	14	0.020	0.096	0.037	TRUE
X - 23293	Hypertension	0.841	52	0.188	0.391	0.028	TRUE
X - 23593	Hypertension	0.140	15	0.060	0.383	0.039	TRUE
X - 23639	Hypertension	0.124	14	0.795	0.440	0.028	TRUE
X - 23644	Hypertension	0.179	15	0.364	0.685	0.029	TRUE

Exposure	Outcome	R2	F	Heterogeneity	Pleiotropy	power	Direction
X - 23705	Hypertension	1.3	61	0.997	0.035	0.048	TRUE
X - 11315	Hypertension	0.193	16	0.626	0.382	0.034	TRUE
X - 23765	Hypertension	0.146	15	0.006	0.490	0.030	TRUE
X - 23782	Hypertension	0.103	16	0.043	0.853	0.031	TRUE
X - 23788	Hypertension	0.088	13	0.192	0.079	0.033	TRUE
anthranilate	Hypertension	0.121	15	0.145	0.386	0.027	TRUE
X - 23314	Hypertension	0.282	23	0.047	0.223	0.026	TRUE
dimethylglycine	Hypertension	0.262	19	0.008	0.862	0.027	TRUE
creatinine	Hypertension	0.154	14	0.002	0.981	0.035	TRUE
X - 23997	Hypertension	0.116	13	4.73e-04	0.849	0.032	TRUE
X - 24021	Hypertension	0.096	13	0.723	0.316	0.044	TRUE
X - 24061	Hypertension	0.174	14	0.175	0.004	0.042	TRUE
X - 24065	Hypertension	0.103	14	0.604	0.094	0.025	TRUE
X - 24071	Hypertension	0.199	17	4.8e-09	0.102	0.053	TRUE
X - 24097	Hypertension	0.160	14	0.061	0.300	0.042	TRUE
X - 24125	Hypertension	0.208	17	2.46e-10	0.605	0.027	TRUE
X - 24240	Hypertension	0.211	14	3.03e-06	0.734	0.049	TRUE

Exposure	Outcome	R2	F	Heterogeneity	Pleiotropy	power	Direction
X - 24241	Hypertension	0.343	26	4e-04	0.722	0.031	TRUE
2-hydroxybutyrate/2-hydroxyisobutyrate	Hypertension	0.094	12	0.095	0.040	0.034	TRUE
2-methylcitrate/homocitrate	Hypertension	0.098	14	0.007	0.397	0.030	TRUE
X - 24242	Hypertension	0.101	12	0.013	0.935	0.046	TRUE
oleate/vaccenate (18:1)	Hypertension	0.188	13	0.116	0.936	0.026	TRUE
2-hydroxyglutarate	Hypertension	0.133	15	8.22e-07	0.466	0.025	TRUE
X - 24293	Hypertension	0.071	12	0.693	0.883	0.039	TRUE
cis-aconitate	Hypertension	0.085	12	0.015	0.938	0.031	TRUE
X - 24278	Hypertension	0.639	25	1.19e-06	0.476	0.027	TRUE
leucylphenylalanine/isoleucylphenylalanine	Hypertension	0.326	42	2.56e-09	0.307	0.105	TRUE
1-palmitoleoylglycerol (16:1)	Hypertension	0.123	14	0.010	0.858	0.041	TRUE
sphingomyelin (d18:1/15:0, d16:1/17:0)	Hypertension	0.187	18	0.012	0.088	0.064	TRUE
palmitoyl dihydrosphingomyelin (d18:0/16:0)	Hypertension	0.104	14	2.53e-06	0.703	0.045	TRUE
sphingomyelin (d18:2/23:0, d18:1/23:1, d17:1/24:1)	Hypertension	0.146	14	0.824	0.480	0.033	TRUE
sphingomyelin (d18:2/24:1, d18:1/24:2)	Hypertension	0.163	14	0.793	0.092	0.025	TRUE
1-stearoyl-2-oleoyl-GPC (18:0/18:1)	Hypertension	0.135	15	0.003	0.252	0.030	TRUE
1-stearoyl-2-linoleoyl-GPE (18:0/18:2)	Hypertension	1.4	43	0.314	0.887	0.054	TRUE

Exposure	Outcome	R2	F	Heterogeneity	Pleiotropy	power	Direction
1-stearoyl-2-arachidonoyl-GPE (18:0/20:4)	Hypertension	0.547	38	0.954	0.777	0.032	TRUE
1-stearoyl-2-arachidonoyl-GPI (18:0/20:4)	Hypertension	0.908	36	0.943	0.754	0.043	TRUE
1-palmitoyl-2-linoleoyl-GPI (16:0/18:2)	Hypertension	0.638	24	0.777	0.022	0.047	TRUE
1-stearoyl-2-linoleoyl-GPC (18:0/18:2)	Hypertension	0.434	18	0.994	0.412	0.044	TRUE
1-oleoyl-2-linoleoyl-GPC (18:1/18:2)	Hypertension	0.554	22	0.145	0.029	0.041	TRUE
1-palmitoyl-2-dihomo-linolenoyl-GPC (16:0/20:3n3 or 6)	Hypertension	0.330	16	0.928	0.436	0.029	TRUE
1-palmitoyl-2-oleoyl-GPC (16:0/18:1)	Hypertension	0.124	14	0.420	0.055	0.034	TRUE
1-palmitoyl-2-arachidonoyl-GPC (16:0/20:4)	Hypertension	2.9	124	1.000	0.810	0.051	TRUE
1-palmitoyl-2-eicosapentaenoyl-GPC (16:0/20:5)	Hypertension	0.487	20	0.870	0.729	0.047	TRUE
1-palmitoyl-2-arachidonoyl-GPE (16:0/20:4)	Hypertension	0.362	28	0.347	0.585	0.026	TRUE
1-palmitoyl-2-docosahexaenoyl-GPE (16:0/22:6)	Hypertension	0.777	34	0.003	0.343	0.026	TRUE
1-stearoyl-2-docosahexaenoyl-GPE (18:0/22:6)	Hypertension	0.500	29	0.002	0.978	0.033	TRUE
1-palmitoyl-2-arachidonoyl-GPI (16:0/20:4)	Hypertension	0.336	17	0.296	0.884	0.026	TRUE
1-stearoyl-2-linoleoyl-GPI (18:0/18:2)	Hypertension	0.869	29	0.450	0.390	0.043	TRUE
1-palmitoyl-2-palmitoleoyl-GPC (16:0/16:1)	Hypertension	0.130	13	0.004	0.953	0.034	TRUE
gamma-tocopherol/beta-tocopherol	Hypertension	0.151	16	0.010	0.600	0.036	TRUE
1-(1-enyl-stearoyl)-2-arachidonoyl-GPE (P-18:0/20:4)	Hypertension	0.712	33	0.893	0.662	0.046	TRUE

Exposure	Outcome	R2	F	Heterogeneity	Pleiotropy	power	Direction
1-(1-enyl-stearoyl)-2-docosahexaenoyl-GPE (P-18:0/22:6)	Hypertension	0.223	18	0.790	0.650	0.050	TRUE
1-(1-enyl-palmitoyl)-2-oleoyl-GPE (P-16:0/18:1)	Hypertension	0.187	13	3.39e-07	0.128	0.035	TRUE
1-(1-enyl-palmitoyl)-2-oleoyl-GPC (P-16:0/18:1)	Hypertension	0.200	14	1.48e-04	0.922	0.039	TRUE
sphingomyelin (d18:1/21:0, d17:1/22:0, d16:1/23:0)	Hypertension	0.325	22	5.94e-05	0.089	0.051	TRUE
1-pentadecanoyl-2-linoleoyl-GPC (15:0/18:2)	Hypertension	0.429	17	0.970	0.347	0.053	TRUE
1-pentadecanoyl-2-oleoyl-GPC (15:0/18:1)	Hypertension	0.091	16	0.006	0.494	0.038	TRUE
1-margaroyl-2-oleoyl-GPC (17:0/18:1)	Hypertension	0.140	15	0.211	0.021	0.029	TRUE
1-margaroyl-2-linoleoyl-GPC (17:0/18:2)	Hypertension	0.224	15	0.965	0.353	0.033	TRUE
X - 24309	Hypertension	0.551	35	7.19e-06	0.748	0.033	TRUE
2'-deoxyuridine	Hypertension	0.244	20	0.331	0.440	0.044	TRUE
1,2-dilinoleoyl-GPC (18:2/18:2)	Hypertension	0.723	29	0.486	0.012	0.048	TRUE
1-palmitoyl-2-docosahexaenoyl-GPC (16:0/22:6)	Hypertension	0.174	15	0.226	0.801	0.034	TRUE
1-stearoyl-2-docosahexaenoyl-GPC (18:0/22:6)	Hypertension	0.157	15	0.206	0.900	0.038	TRUE
1-(1-enyl-stearoyl)-2-oleoyl-GPC (P-18:0/18:1)	Hypertension	0.139	13	6.28e-05	0.621	0.040	TRUE
1-(1-enyl-stearoyl)-2-arachidonoyl-GPC (P-18:0/20:4)	Hypertension	1.6	73	0.998	0.753	0.057	TRUE
1-(1-enyl-stearoyl)-2-oleoyl-GPE (P-18:0/18:1)	Hypertension	0.085	11	0.004	0.262	0.025	TRUE
sphingomyelin (d18:1/17:0, d17:1/18:0, d19:1/16:0)	Hypertension	0.128	14	0.672	0.409	0.032	TRUE

Exposure	Outcome	R2	F	Heterogeneity	Pleiotropy	power	Direction
1-palmitoyl-2-stearoyl-GPC (16:0/18:0)	Hypertension	0.305	15	0.924	0.893	0.030	TRUE
1-stearoyl-2-dihomo-linolenoyl-GPC (18:0/20:3n3 or 6)	Hypertension	0.203	17	0.571	0.837	0.033	TRUE
1-(1-enyl-palmitoyl)-2-docosahexaenoyl-GPC (P-16:0/22:6)	Hypertension	0.173	14	0.078	0.630	0.039	TRUE
palmitoleoyl-oleoyl-glycerol (16:1/18:1) [2]	Hypertension	0.246	14	0.025	0.347	0.058	TRUE
palmitoleoyl-oleoyl-glycerol (16:1/18:1) [1]	Hypertension	0.221	17	0.601	0.450	0.025	TRUE
palmitoyl-linoleoyl-glycerol (16:0/18:2) [1]	Hypertension	0.221	16	2.14e-19	0.318	0.055	TRUE
palmitoyl-linoleoyl-glycerol (16:0/18:2) [2]	Hypertension	0.197	18	2.31e-11	0.487	0.042	TRUE
X - 24425	Hypertension	0.149	18	0.133	0.113	0.027	TRUE
1-palmitoyl-2-oleoyl-GPI (16:0/18:1)	Hypertension	0.318	19	0.797	0.010	0.027	TRUE
1-(1-enyl-palmitoyl)-2-docosahexaenoyl-GPE (P-16:0/22:6)	Hypertension	0.254	17	0.602	0.725	0.035	TRUE
1-(1-enyl-palmitoyl)-2-arachidonoyl-GPE (P-16:0/20:4)	Hypertension	0.657	26	0.997	0.691	0.042	TRUE
1-(1-enyl-palmitoyl)-2-linoleoyl-GPE (P-16:0/18:2)	Hypertension	0.446	18	0.915	0.138	0.048	TRUE
1-(1-enyl-palmitoyl)-2-linoleoyl-GPC (P-16:0/18:2)	Hypertension	0.135	15	0.023	0.424	0.026	TRUE
1-palmitoleoyl-2-linoleoyl-GPC (16:1/18:2)	Hypertension	0.874	40	0.325	0.579	0.042	TRUE
1-palmitoyl-2-alpha-linolenoyl-GPC (16:0/18:3n3)	Hypertension	0.864	34	0.996	0.367	0.042	TRUE
1-(1-enyl-palmitoyl)-2-arachidonoyl-GPC (P-16:0/20:4)	Hypertension	1.7	73	0.979	0.061	0.049	TRUE
1-oleoyl-2-docosahexaenoyl-GPC (18:1/22:6)	Hypertension	0.111	15	0.071	0.515	0.027	TRUE

Exposure	Outcome	R2	F	Heterogeneity	Pleiotropy	power	Direction
1-adrenoyl-GPC (22:4)	Hypertension	0.607	26	0.979	0.357	0.055	TRUE
1-stearoyl-2-docosapentaenoyl-GPC (18:0/22:5n3)	Hypertension	0.654	27	0.533	0.087	0.036	TRUE
lactate	Hypertension	0.153	14	9.52e-04	0.049	0.059	TRUE
1-stearoyl-2-docosapentaenoyl-GPC (18:0/22:5n6)	Hypertension	0.480	20	0.764	0.698	0.042	TRUE
1-(1-enyl-stearoyl)-GPC (P-18:0)	Hypertension	0.116	15	0.021	0.179	0.047	TRUE
1-stearyl-GPC (O-18:0)	Hypertension	0.119	17	8.57e-06	0.371	0.063	TRUE
1-(1-enyl-stearoyl)-2-docosahexaenoyl-GPC (P-18:0/22:6)	Hypertension	0.135	14	0.003	0.992	0.042	TRUE
1-oleoyl-2-dihomo-linolenoyl-GPC (18:1/20:3)	Hypertension	0.625	28	1.000	0.444	0.053	TRUE
1-palmitoyl-2-adrenoyl-GPC (16:0/22:4)	Hypertension	0.143	18	3.85e-08	0.139	0.058	TRUE
1-myristoyl-2-linoleoyl-GPC (14:0/18:2)	Hypertension	0.470	19	0.987	0.667	0.060	TRUE
1-linoleoyl-2-arachidonoyl-GPC (18:2/20:4n6)	Hypertension	1.2	56	0.999	0.086	0.046	TRUE
1-oleoyl-2-eicosapentaenoyl-GPC (18:1/20:5)	Hypertension	0.532	22	0.837	0.723	0.047	TRUE
1-(1-enyl-palmitoyl)-2-palmitoyl-GPC (P-16:0/16:0)	Hypertension	0.183	20	0.065	0.029	0.031	TRUE
phosphatidylcholine (16:0/22:5n3, 18:1/20:4)	Hypertension	2.1	88	0.873	0.042	0.044	TRUE
phosphatidylcholine (18:0/20:5, 16:0/22:5n6)	Hypertension	0.940	34	0.798	0.092	0.049	TRUE
1-palmitoyl-2-meadoyl-GPC (16:0/20:3n9)	Hypertension	0.955	43	0.791	0.338	0.050	TRUE
1-stearoyl-2-meadoyl-GPC (18:0/20:3n9)	Hypertension	1.1	55	0.979	0.490	0.050	TRUE

Exposure	Outcome	R2	F	Heterogeneity	Pleiotropy	power	Direction
glutamine	Hypertension	0.159	19	0.005	0.613	0.035	TRUE
3-hydroxy-3-methylglutarate	Hypertension	0.091	15	0.497	0.264	0.029	TRUE
tryptophan	Hypertension	0.136	14	1.14e-04	0.172	0.056	TRUE
beta-alanine	Hypertension	0.134	18	0.247	0.403	0.040	TRUE
adenosine	Hypertension	0.187	14	0.617	0.762	0.025	TRUE
caffeine	Hypertension	0.202	15	1.05e-07	0.693	0.027	TRUE
glutamate	Hypertension	0.126	17	0.003	0.891	0.044	TRUE
glycine	Hypertension	0.188	29	2.07e-17	0.163	0.031	TRUE
gluconate	Hypertension	0.166	17	0.101	0.897	0.055	TRUE
histidine	Hypertension	0.092	12	0.727	0.649	0.025	TRUE
nicotinamide	Hypertension	0.133	16	0.358	0.101	0.034	TRUE
dihydroorotate	Hypertension	0.203	17	6.81e-06	0.423	0.027	TRUE
uridine	Hypertension	0.124	16	0.002	0.891	0.029	TRUE
trans-urocanate	Hypertension	0.124	19	0.573	0.188	0.038	TRUE
2-aminoadipate	Hypertension	0.212	16	2.09e-04	0.921	0.040	TRUE
cholesterol	Hypertension	0.136	15	0.005	0.344	0.034	TRUE
Carnitine	Hypertension	0.122	71	8.73e-06	0.121	0.099	TRUE

Exposure	Outcome	R2	F	Heterogeneity	Pleiotropy	power	Direction
Benzoate	Hypertension	0.018	22	0.772	0.649	0.027	TRUE
Maltotriose	Hypertension	0.044	23	0.727	0.589	0.032	TRUE
Hippurate	Hypertension	0.027	23	0.645	0.610	0.027	TRUE
Methionine sulfoxide	Hypertension	0.029	25	1.69e-08	0.179	0.057	TRUE
Xanthurenate	Hypertension	0.061	30	2.86e-05	0.928	0.033	TRUE
N-alpha-acetylornithine	Hypertension	0.049	27	0.057	0.642	0.049	TRUE
3-methylhistidine	Hypertension	0.041	23	0.124	0.423	0.033	TRUE
Glycerophosphorylcholine (GPC)	Hypertension	0.038	25	0.119	0.441	0.031	TRUE
Phenylacetate	Hypertension	0.045	27	0.162	0.361	0.063	TRUE
3-methyl-2-oxovalerate	Hypertension	0.024	25	0.311	0.342	0.028	TRUE
Tartarate	Hypertension	0.037	22	0.132	0.951	0.037	TRUE
Suberate (C8-DC)	Hypertension	0.038	23	0.005	0.302	0.030	TRUE
5-hydroxylysine	Hypertension	0.097	84	0.007	0.031	0.076	TRUE
3-phenylpropionate hydrocinnamate	Hypertension	0.043	24	0.017	0.633	0.047	TRUE
Kynurenine	Hypertension	0.067	40	3.97e-58	0.061	0.446	TRUE
N-acetylglutamate	Hypertension	0.037	26	5.11e-04	0.597	0.053	TRUE
Glycerol 3-phosphate	Hypertension	0.068	33	0.023	0.629	0.038	TRUE

Exposure	Outcome	R2	F	Heterogeneity	Pleiotropy	power	Direction
Imidazole lactate	Hypertension	0.230	127	1.95e-07	0.744	0.035	TRUE
4-guanidinobutanoate	Hypertension	0.031	24	4.9e-27	0.708	0.142	TRUE
Glucuronate	Hypertension	0.018	22	0.485	0.470	0.043	TRUE
Ribitol	Hypertension	0.081	43	0.439	0.616	0.037	TRUE
Glycodeoxycholate	Hypertension	0.018	24	0.047	0.051	0.027	TRUE
Theobromine	Hypertension	0.040	25	0.157	0.809	0.042	TRUE
Quinate	Hypertension	0.031	23	1.7e-06	0.600	0.058	TRUE
Gentisate	Hypertension	0.052	23	0.028	0.849	0.026	TRUE
Theophylline	Hypertension	0.033	23	0.386	0.820	0.045	TRUE
1,5-anhydroglucitol (1,5-ag)	Hypertension	0.076	30	7.12e-07	0.440	0.030	TRUE
1-stearoyl-2-arachidonoyl-GPI (18:0/20:4)	Hypertension	0.105	55	6.22e-05	0.714	0.039	TRUE
Indolelactate	Hypertension	0.052	36	0.632	0.357	0.034	TRUE
Docosahexaenoate DHA; 22:6n3	Hypertension	0.026	24	0.200	0.187	0.047	TRUE
1-stearoyl-2-oleoyl-GPS (18:0/18:1)	Hypertension	0.030	28	4.48e-04	0.173	0.114	TRUE
1-myristoyl-2-palmitoyl-gpc (14:0/16:0)	Hypertension	0.025	23	0.006	0.607	0.041	TRUE
Paraxanthine	Hypertension	0.049	24	0.620	0.218	0.034	TRUE
4-methyl-2-oxopentanoate	Hypertension	0.029	24	0.044	0.094	0.037	TRUE

Exposure	Outcome	R2	F	Heterogeneity	Pleiotropy	power	Direction
3-indoxyl sulfate	Hypertension	0.030	23	0.059	0.950	0.030	TRUE
Sphingosine 1-phosphate	Hypertension	0.032	24	0.156	0.286	0.036	TRUE
Alpha-hydroxyisocaproate	Hypertension	0.040	24	0.165	0.596	0.028	TRUE
1-stearoyl-GPI (18:0)	Hypertension	0.031	23	0.203	0.906	0.062	TRUE
1,2-dipalmitoyl-gpc (16:0/16:0)	Hypertension	0.073	48	5.22e-07	0.804	0.076	TRUE
Maleate	Hypertension	0.034	24	1.8e-05	0.704	0.051	TRUE
Isovalerate (i5:0)	Hypertension	0.024	23	0.031	0.599	0.037	TRUE
4-acetylphenol sulfate	Hypertension	0.028	23	0.014	0.637	0.043	TRUE
Phenyllactate (PLA)	Hypertension	0.087	36	0.297	0.320	0.040	TRUE
N-acetylaspartate (naa)	Hypertension	0.016	23	0.008	0.566	0.039	TRUE
Palmitoylcarnitine	Hypertension	0.057	27	0.103	0.549	0.029	TRUE
Erythritol	Hypertension	0.059	29	2.05e-14	0.196	0.042	TRUE
Hexanoylcarnitine	Hypertension	0.137	93	0.606	0.491	0.069	TRUE
Acetylcarnitine	Hypertension	0.049	34	0.065	0.376	0.031	TRUE
Iminodiacetate (IDA)	Hypertension	0.026	22	0.567	0.458	0.029	TRUE
Cysteine s-sulfate	Hypertension	0.030	23	0.060	0.570	0.039	TRUE
Oxalate (ethanedioate)	Hypertension	0.041	24	0.270	0.326	0.035	TRUE

Exposure	Outcome	R2	F	Heterogeneity	Pleiotropy	power	Direction
DHEAS	Hypertension	0.089	28	4.28e-17	0.753	0.043	TRUE
3-hydroxymyristate	Hypertension	0.018	22	0.330	0.374	0.035	TRUE
Homoarginine	Hypertension	0.140	67	1.97e-32	0.702	0.026	TRUE
1-oleoylglycerol (18:1)	Hypertension	0.054	26	1.55e-07	0.266	0.068	TRUE
3-methyl-2-oxobutyrate	Hypertension	0.041	26	0.854	0.603	0.042	TRUE
Tartronate (hydroxymalonate)	Hypertension	0.032	24	0.223	0.221	0.039	TRUE
2,3-dihydroxypyridine	Hypertension	0.029	22	7.22e-04	0.290	0.042	TRUE
3-hydroxyoctanoate	Hypertension	0.040	26	0.657	0.039	0.086	TRUE
2-hydroxyoctanoate	Hypertension	0.055	33	0.068	0.241	0.034	TRUE
Homocitrulline	Hypertension	0.078	41	0.053	0.640	0.026	TRUE
Indoleacetate	Hypertension	0.061	24	0.742	0.115	0.026	TRUE
1-linoleoylglycerol (18:2)	Hypertension	0.019	23	0.111	0.039	0.053	TRUE
2-linoleoylglycerol (18:2)	Hypertension	0.032	22	6.84e-05	0.320	0.038	TRUE
Indolepropionate	Hypertension	0.027	38	0.402	0.305	0.058	TRUE
Threonate	Hypertension	0.041	24	0.405	0.706	0.028	TRUE
N-acetylglycine	Hypertension	0.114	61	4.13e-05	0.009	0.117	TRUE
Glycolithocholate	Hypertension	0.033	23	0.534	0.034	0.028	TRUE

Exposure	Outcome	R2	F	Heterogeneity	Pleiotropy	power	Direction
EDTA	Hypertension	0.022	22	0.144	0.651	0.049	TRUE
1-methylhistidine	Hypertension	0.070	31	0.019	0.906	0.027	TRUE
Citramalate	Hypertension	0.030	23	8.54e-11	0.121	0.029	TRUE
3-hydroxydecanoate	Hypertension	0.038	40	0.050	0.715	0.032	TRUE
1,3-dimethylurate	Hypertension	0.037	26	0.958	0.425	0.065	TRUE
Galactonate	Hypertension	0.034	26	0.003	0.789	0.032	TRUE
Trigonelline	Hypertension	0.025	23	0.340	0.769	0.056	TRUE
Dodecanedioate	Hypertension	0.048	25	0.466	0.768	0.029	TRUE
Isobutyrylcarnitine (c4)	Hypertension	0.163	69	0.146	0.134	0.058	TRUE
3-methylxanthine	Hypertension	0.045	25	0.111	0.827	0.032	TRUE
3-hydroxylaurate	Hypertension	0.023	27	0.424	0.782	0.040	TRUE
Pyridoxate	Hypertension	0.038	23	1.91e-04	0.131	0.027	TRUE
5-hydroxyhexanoate	Hypertension	0.030	23	0.599	0.206	0.038	TRUE
Gamma-glutamylvaline	Hypertension	0.045	25	0.264	0.098	0.026	TRUE
3-hydroxysebacate	Hypertension	0.046	32	0.008	0.187	0.047	TRUE
Propionylglycine	Hypertension	0.089	54	0.052	0.131	0.140	TRUE
Butyrylglycine	Hypertension	0.158	117	1.2e-07	0.080	0.155	TRUE

Exposure	Outcome	R2	F	Heterogeneity	Pleiotropy	power	Direction
Pro-hydroxy-pro	Hypertension	0.039	23	0.169	0.016	0.034	TRUE
Propionylcarnitine (c3)	Hypertension	0.072	55	0.009	0.244	0.091	TRUE
Adrenate (22:4n6)	Hypertension	0.040	34	0.026	0.341	0.029	TRUE
Docosatrienoate (22:3n3)	Hypertension	0.056	33	0.001	0.235	0.069	TRUE
Docosadienoate (22:2n6)	Hypertension	0.027	23	0.117	0.744	0.071	TRUE
3-carboxy-4-methyl-5-propyl-2-furanpropanoate (cmpf)	Hypertension	0.029	25	0.022	0.904	0.028	TRUE
Docosapentaenoate n3 DPA; 22:5n3	Hypertension	0.040	28	1.34e-12	0.302	0.027	TRUE
3-hydroxy-2-ethylpropionate	Hypertension	0.063	26	5.77e-25	0.578	0.070	TRUE
Myristoleate (14:1n5)	Hypertension	0.034	24	1.77e-13	0.912	0.075	TRUE
10-undecenoate (11:1n1)	Hypertension	0.079	62	0.580	0.279	0.039	TRUE
1-methyl-4-imidazoleacetate	Hypertension	0.104	56	0.325	0.124	0.026	TRUE
Sebacate (C10-DC)	Hypertension	0.033	31	0.476	0.963	0.032	TRUE
Tauro-beta-muricholate	Hypertension	0.083	51	0.153	0.074	0.049	TRUE
Octanoylcarnitine (c8)	Hypertension	0.116	101	0.651	0.135	0.052	TRUE
Stearidonate (18:4n3)	Hypertension	0.044	26	6.26e-04	0.716	0.068	TRUE
Decanoylcarnitine (C10)	Hypertension	0.082	64	0.101	0.100	0.056	TRUE
5-dodecenoate (12:1n7)	Hypertension	0.025	26	2.45e-04	0.973	0.085	TRUE

Exposure	Outcome	R2	F	Heterogeneity	Pleiotropy	power	Direction
N-acetylisoleucine	Hypertension	0.024	22	2.57e-04	0.491	0.026	TRUE
Campesterol	Hypertension	0.081	49	3.36e-13	0.700	0.044	TRUE
N-acetylthreonine	Hypertension	0.043	24	2.2e-37	0.620	0.154	TRUE
1-oleoyl-GPC (18:1)	Hypertension	0.028	29	0.547	0.684	0.031	TRUE
10-nonadecenoate (19:1n9)	Hypertension	0.022	23	1.11e-11	0.329	0.052	TRUE
Piperine	Hypertension	0.038	22	0.001	0.352	0.030	TRUE
Carnitine C14	Hypertension	0.034	26	0.028	0.715	0.104	TRUE
10-heptadecenoate (17:1n7)	Hypertension	0.028	23	1.49e-07	0.535	0.082	TRUE
1-stearoyl-gpc (18:0)	Hypertension	0.058	29	0.050	0.349	0.034	TRUE
1-palmitoyl-GPC (16:0)	Hypertension	0.052	29	0.208	0.362	0.030	TRUE
Hyocholate	Hypertension	0.030	23	0.014	0.019	0.037	TRUE
N-acetylhistidine	Hypertension	0.160	107	0.002	0.878	0.042	TRUE
Alpha-hydroxyisovalerate	Hypertension	0.094	57	0.002	0.618	0.085	TRUE
N-acetylproline	Hypertension	0.026	24	0.133	0.271	0.040	TRUE
Stachydrine	Hypertension	0.028	23	0.308	0.652	0.040	TRUE
Salicyluric glucuronide	Hypertension	0.013	22	0.099	0.863	0.030	TRUE
P-cresol sulfate	Hypertension	0.035	22	0.005	0.595	0.041	TRUE

Exposure	Outcome	R2	F	Heterogeneity	Pleiotropy	power	Direction
Gamma-glutamylglycine	Hypertension	0.160	80	0.013	0.035	0.197	TRUE
1-linoleoyl-gpc (18:2)	Hypertension	0.041	31	0.036	0.381	0.033	TRUE
Stearoylcarnitine	Hypertension	0.064	36	3.3e-06	0.436	0.132	TRUE
Eicosenoate (20:1)	Hypertension	0.035	22	6.71e-09	0.067	0.072	TRUE
Aconitate [cis or trans]	Hypertension	0.040	24	0.491	0.920	0.033	TRUE
Erythronate	Hypertension	0.045	34	8.57e-36	0.722	0.031	TRUE
Isovalerylcarnitine (C5)	Hypertension	0.057	48	1.1e-06	0.949	0.085	TRUE
Linolenate [alpha or gamma; (18:3n3 or 6)]	Hypertension	0.028	23	0.004	0.307	0.030	TRUE
Gamma-glutamylthreonine	Hypertension	0.054	24	2.06e-06	0.455	0.026	TRUE
Laurylcarnitine	Hypertension	0.037	24	0.134	0.665	0.026	TRUE
Gamma-glutamylmethionine	Hypertension	0.036	23	0.195	0.158	0.035	TRUE
Epiandrosterone sulfate	Hypertension	0.175	92	2.79e-06	0.719	0.073	TRUE
Gamma-glutamyltryptophan	Hypertension	0.050	26	1.05e-05	0.833	0.079	TRUE
7-methylxanthine	Hypertension	0.031	25	1.44e-05	0.095	0.071	TRUE
3,7-dimethylurate	Hypertension	0.046	24	2.67e-05	0.477	0.026	TRUE
1,7-dimethyluric acid	Hypertension	0.048	23	0.055	0.974	0.028	TRUE
1,3,7-trimethylurate	Hypertension	0.044	26	0.123	0.244	0.028	TRUE

Exposure	Outcome	R2	F	Heterogeneity	Pleiotropy	power	Direction
N4-acetylcytidine	Hypertension	0.104	35	0.123	0.175	0.029	TRUE
Indolebutyrate	Hypertension	0.049	27	0.039	0.502	0.028	TRUE
5-acetylamino-6-amino-3-methyluracil	Hypertension	0.103	46	7.87e-08	0.416	0.027	TRUE
N1-methylinosine	Hypertension	0.049	24	0.031	0.628	0.033	TRUE
N6-carbamoylthreonyladenosine	Hypertension	0.040	24	3.28e-33	0.659	0.030	TRUE
1-methylxanthine	Hypertension	0.094	50	7.43e-07	0.547	0.080	TRUE
N2,n2-dimethylguanosine	Hypertension	0.025	24	0.006	0.925	0.026	TRUE
4-hydroxyhippurate	Hypertension	0.029	22	0.928	0.840	0.031	TRUE
3-(3-amino-3-carboxypropyl)uridine	Hypertension	0.023	24	3.24e-28	0.520	0.151	TRUE
5,6-dihydrouridine	Hypertension	0.060	29	1.09e-10	0.824	0.034	TRUE
Phenylacetylglutamine	Hypertension	0.032	22	0.180	0.662	0.033	TRUE
3-hydroxydodecanedioate	Hypertension	0.028	23	0.550	0.637	0.035	TRUE
1-arachidonylglycerol (20:4)	Hypertension	0.064	45	0.141	0.680	0.040	TRUE
7-methylguanine	Hypertension	0.036	25	0.502	0.155	0.057	TRUE
1-stearoyl-GPE (18:0)	Hypertension	0.115	37	6.35e-09	0.224	0.066	TRUE
Phenol sulfate	Hypertension	0.036	23	0.502	0.220	0.026	TRUE
Oleoylcarnitine	Hypertension	0.039	36	0.374	0.833	0.025	TRUE

Exposure	Outcome	R2	F	Heterogeneity	Pleiotropy	power	Direction
Gamma-glutamylisoleucine	Hypertension	0.031	24	0.548	0.771	0.028	TRUE
Malonylcarnitine	Hypertension	0.033	23	0.001	0.300	0.072	TRUE
1-palmitoleoyl-GPC (16:1)	Hypertension	0.062	29	6.88e-13	0.485	0.042	TRUE
N-methyl-2-pyridone-5-carboxamide	Hypertension	0.026	24	0.103	0.895	0.032	TRUE
Gamma-glutamyl-2-aminobutyrate	Hypertension	0.048	24	0.809	0.913	0.045	TRUE
1-stearoyl-GPG (18:0)	Hypertension	0.048	31	0.032	0.449	0.027	TRUE
Hexanoylglycine	Hypertension	0.101	53	8.42e-07	0.224	0.046	TRUE
Glutamine degradant	Hypertension	0.091	42	3.23e-04	0.684	0.071	TRUE
5-methyluridine (ribothymidine)	Hypertension	0.177	79	0.004	0.815	0.055	TRUE
Cysteine-glutathione disulfide	Hypertension	0.073	44	0.005	0.803	0.046	TRUE
Isovalerylglycine	Hypertension	0.053	34	2.16e-04	0.106	0.100	TRUE
2-hydroxy-3-methylvalerate	Hypertension	0.046	32	0.039	0.675	0.053	TRUE
Homostachydrine	Hypertension	0.049	34	1.54e-04	0.445	0.045	TRUE
1-arachidonoyl-gpc (20:4n6)	Hypertension	0.169	96	4.12e-07	0.501	0.034	TRUE
2-palmitoyl-GPC (16:0)	Hypertension	0.031	25	0.033	0.358	0.048	TRUE
2-palmitoleoyl-GPC (16:1)	Hypertension	0.054	24	7.01e-05	0.957	0.041	TRUE
1-arachidonoyl-GPE (20:4n6)	Hypertension	0.137	62	6.98e-06	0.271	0.026	TRUE

Exposure	Outcome	R2	F	Heterogeneity	Pleiotropy	power	Direction
1-palmitoyl-GPE (16:0)	Hypertension	0.064	45	9.02e-05	0.534	0.046	TRUE
1-linoleoyl-GPE (18:2)	Hypertension	0.087	43	0.055	0.300	0.025	TRUE
1-oleoyl-GPE (18:1)	Hypertension	0.065	26	0.197	0.280	0.037	TRUE
2-hydroxypalmitate	Hypertension	0.039	23	0.204	0.714	0.034	TRUE
Docosapentaenoate (n6 DPA; 22:5n6)	Hypertension	0.031	26	0.265	0.852	0.030	TRUE
Gulonate	Hypertension	0.039	25	5.06e-04	0.820	0.049	TRUE
Isobutyrylglycine	Hypertension	0.073	34	2.86e-06	0.294	0.052	TRUE
Glutarylcarnitine (c5-dc)	Hypertension	0.180	61	1.97e-07	0.749	0.096	TRUE
Carnitine C5:1	Hypertension	0.042	27	2.96e-08	0.698	0.031	TRUE
Hydroquinone sulfate	Hypertension	0.027	23	0.006	0.180	0.025	TRUE
Beta-hydroxyisovaleroylcarnitine	Hypertension	0.072	43	1.44e-05	0.199	0.160	TRUE
Catechol sulfate	Hypertension	0.054	23	0.003	0.869	0.030	TRUE
7-alpha-hydroxy-3-oxo-4-cholestenoate (7-hoca)	Hypertension	0.045	25	0.002	0.519	0.029	TRUE
Octadecanedioate	Hypertension	0.073	32	0.004	0.600	0.049	TRUE
Tetradecanedioate (C14-DC)	Hypertension	0.113	109	0.047	0.908	0.036	TRUE
Hexadecanedioate (C16-DC)	Hypertension	0.123	98	0.014	0.414	0.050	TRUE
Glycerophosphoethanolamine	Hypertension	0.046	27	0.235	0.638	0.034	TRUE

Exposure	Outcome	R2	F	Heterogeneity	Pleiotropy	power	Direction
Taurolithocholate 3-sulfate	Hypertension	0.053	32	0.043	0.330	0.043	TRUE
1-palmitoyl-GPI (16:0)	Hypertension	0.036	23	0.196	0.501	0.036	TRUE
Glycolithocholate sulfate	Hypertension	0.041	49	0.005	0.170	0.030	TRUE
3-(3-hydroxyphenyl)propionate	Hypertension	0.038	22	0.184	0.966	0.062	TRUE
Deoxycarnitine	Hypertension	0.131	60	7.41e-04	0.241	0.032	TRUE
Alpha-hydroxycaproate	Hypertension	0.017	24	0.232	0.419	0.042	TRUE
1-ribosyl-imidazoleacetate	Hypertension	0.079	33	2.29e-11	0.012	0.061	TRUE
3,4-dihydroxybutyrate	Hypertension	0.045	34	0.041	0.823	0.030	TRUE
Indoleacetylglutamine	Hypertension	0.071	35	0.227	0.067	0.026	TRUE
Mannitol/sorbitol	Hypertension	0.035	23	0.893	0.357	0.046	TRUE
Tryptophan betaine	Hypertension	0.076	32	4.61e-27	0.157	0.084	TRUE
Dihomo-linolenate (20:3n3 or n6)	Hypertension	0.044	26	0.271	0.352	0.033	TRUE
1-linoleoyl-GPI (18:2)	Hypertension	0.073	34	0.034	0.018	0.040	TRUE
1-oleoyl-GPI (18:1)	Hypertension	0.059	26	0.581	0.194	0.041	TRUE
O-cresol sulfate	Hypertension	0.020	23	0.026	0.885	0.050	TRUE
4-vinylphenol sulfate	Hypertension	0.034	24	0.128	0.282	0.027	TRUE
Pyrraline	Hypertension	0.019	23	5.12e-07	0.314	0.028	TRUE

Exposure	Outcome	R2	F	Heterogeneity	Pleiotropy	power	Direction
N6-acetyllysine	Hypertension	0.088	69	4.44e-06	0.264	0.041	TRUE
Thymol sulfate	Hypertension	0.033	28	9.38e-04	0.442	0.043	TRUE
N-acetylserine	Hypertension	0.035	27	3.03e-36	0.777	0.047	TRUE
3-methyladipate	Hypertension	0.026	24	0.017	0.407	0.060	TRUE
4-ethylphenylsulfate	Hypertension	0.023	24	2.76e-04	0.884	0.039	TRUE
1-stearoyl-2-oleoyl-GPE (18:0/18:1)	Hypertension	0.100	47	0.409	0.936	0.043	TRUE
Dimethylarginine (sdma + adma)	Hypertension	0.036	33	0.342	0.645	0.033	TRUE
Hexanoylglutamine	Hypertension	0.123	81	1.42e-12	0.407	0.072	TRUE
Gamma-glutamylalanine	Hypertension	0.029	24	4.27e-12	0.680	0.042	TRUE
5alpha-androstan-3beta,17beta-diol disulfate	Hypertension	0.098	52	2.69e-04	0.667	0.055	TRUE
N-methylproline	Hypertension	0.028	23	0.443	0.770	0.044	TRUE
Carotenoid	Hypertension	0.038	23	0.104	0.272	0.049	TRUE
5alpha-pregnan-3beta,20alpha-diol disulfate	Hypertension	0.102	39	0.037	0.604	0.050	TRUE
Taurocholenate sulfate	Hypertension	0.147	46	3.55e-10	0.397	0.031	TRUE
Pregnenediol disulfate (C21H34O8S2)	Hypertension	0.179	71	4.79e-05	0.223	0.060	TRUE
Glycocholenate sulfate	Hypertension	0.226	83	7.01e-05	0.304	0.067	TRUE
Androstenediol (3beta,17beta) disulfate (1)	Hypertension	0.095	58	0.171	0.857	0.054	TRUE

Exposure	Outcome	R2	F	Heterogeneity	Pleiotropy	power	Direction
Chiro-inositol	Hypertension	0.019	22	0.717	0.945	0.028	TRUE
1-palmitoyl-2-linoleoyl-GPE (16:0/18:2)	Hypertension	0.211	92	4.51e-07	0.636	0.050	TRUE
Sphinganine-1-phosphate	Hypertension	0.024	25	1.81e-05	0.370	0.046	TRUE
Glycosyl-N-stearoyl-sphingosine (d18:1/18:0)	Hypertension	0.084	37	0.005	0.336	0.035	TRUE
4-allylphenol sulfate	Hypertension	0.037	26	0.008	0.492	0.038	TRUE
Succinylcarnitine	Hypertension	0.154	63	0.090	0.197	0.081	TRUE
21-hydroxypregnenolone disulfate	Hypertension	0.112	37	2.71e-23	0.107	0.040	TRUE
5alpha-androstan-3alpha,17alpha-diol monosulfate	Hypertension	0.088	53	9.18e-04	0.528	0.030	TRUE
Androstenediol (3beta,17beta) disulfate (2)	Hypertension	0.097	29	1.26e-10	0.833	0.123	TRUE
5alpha-androstan-3beta,17alpha-diol disulfate	Hypertension	0.048	31	0.038	0.316	0.078	TRUE
5alpha-pregnan-3beta,20beta-diol monosulfate (1)	Hypertension	0.119	64	0.013	0.373	0.058	TRUE
5alpha-pregnan-3beta,20alpha-diol monosulfate (2)	Hypertension	0.083	35	3.37e-07	0.926	0.035	TRUE
5alpha-androstan-3beta,17beta-diol monosulfate (2)	Hypertension	0.107	46	0.608	0.253	0.059	TRUE
Androstenediol (3alpha, 17alpha) monosulfate (3)	Hypertension	0.183	87	6.81e-10	0.839	0.046	TRUE
5alpha-pregnan-diol disulfate	Hypertension	0.051	36	0.133	0.819	0.030	TRUE
5alpha-androstan-3alpha,17beta-diol monosulfate (1)	Hypertension	0.155	84	0.113	0.990	0.061	TRUE
5alpha-androstan-3alpha,17beta-diol disulfate	Hypertension	0.066	28	0.463	0.347	0.096	TRUE

Exposure	Outcome	R2	F	Heterogeneity	Pleiotropy	power	Direction
Andro steroid monosulfate C19H28O6S (1)	Hypertension	0.118	45	2.51e-05	0.084	0.035	TRUE
Pregnenediol sulfate (C21H34O5S)	Hypertension	0.101	35	7.51e-33	0.102	0.026	TRUE
4-hydroxycoumarin	Hypertension	0.032	24	0.058	0.398	0.031	TRUE
16a-hydroxy DHEA 3-sulfate	Hypertension	0.134	65	3.14e-07	0.802	0.060	TRUE
Gamma-CEHC	Hypertension	0.102	37	0.033	0.223	0.047	TRUE
2-hydroxyglutarate	Hypertension	0.045	27	0.199	0.444	0.087	TRUE
N-acetyl-beta-alanine	Hypertension	0.058	35	0.099	0.005	0.035	TRUE
Tridecenedioate (C13:1-DC)	Hypertension	0.041	27	0.114	0.295	0.029	TRUE
N-acetyl-3-methylhistidine	Hypertension	0.102	49	0.569	0.058	0.040	TRUE
2-hydroxyhippurate	Hypertension	0.030	23	0.082	0.055	0.030	TRUE
4-cholesten-3-one	Hypertension	0.047	24	0.030	0.225	0.044	TRUE
Androstenediol (3beta,17beta) monosulfate (2)	Hypertension	0.064	27	8.24e-04	0.300	0.050	TRUE
Androstenediol (3beta,17beta) monosulfate (1)	Hypertension	0.063	28	7.62e-12	0.569	0.062	TRUE
Pregnenolone sulfate	Hypertension	0.077	32	8.56e-16	0.082	0.038	TRUE
S-methylmethionine	Hypertension	0.024	22	0.182	0.083	0.045	TRUE
13-HODE + 9-HODE	Hypertension	0.017	24	0.608	0.262	0.030	TRUE
Palmitoyl sphingomyelin (d18:1/16:0)	Hypertension	0.059	26	3.78e-04	0.387	0.037	TRUE

Exposure	Outcome	R2	F	Heterogeneity	Pleiotropy	power	Direction
Indole-3-carboxylate	Hypertension	0.022	26	0.183	0.900	0.031	TRUE
Sphingomyelin (d18:1/18:1, d18:2/18:0)	Hypertension	0.073	32	2.26e-46	0.048	0.093	TRUE
Ergothioneine	Hypertension	0.050	38	0.007	0.627	0.077	TRUE
Cis-4-decenoylcarnitine (C10:1)	Hypertension	0.109	87	0.315	0.106	0.046	TRUE
5-(galactosylhydroxy)-L-lysine	Hypertension	0.023	24	0.150	0.035	0.031	TRUE
2s,3R-dihydroxybutyrate	Hypertension	0.044	30	0.036	0.531	0.037	TRUE
Cysteinylglycine disulfide	Hypertension	0.086	74	2.17e-04	0.396	0.171	TRUE
2,3-dihydroxyisovalerate	Hypertension	0.042	22	0.127	0.191	0.040	TRUE
Alpha-ketoglutaramate	Hypertension	0.032	45	0.745	0.580	0.089	TRUE
3-methylglutaconate	Hypertension	0.147	98	5.16e-08	0.165	0.049	TRUE
(16 or 17)-methylstearate (a19:0 or i19:0)	Hypertension	0.031	23	8.71e-12	0.086	0.089	TRUE
Cinnamoylglycine	Hypertension	0.066	30	0.011	0.961	0.120	TRUE
2R,3R-dihydroxybutyrate	Hypertension	0.102	43	1.38e-08	0.041	0.046	TRUE
4-hydroxyglutamate	Hypertension	0.050	25	0.452	0.345	0.056	TRUE
S-methylcysteine	Hypertension	0.051	48	0.059	0.179	0.028	TRUE
Isoursodeoxycholate	Hypertension	0.023	24	0.002	0.400	0.052	TRUE
Cis-4-decenoate (10:1n6)	Hypertension	0.153	112	0.012	0.206	0.031	TRUE

Exposure	Outcome	R2	F	Heterogeneity	Pleiotropy	power	Direction
4-oxo-retinoic acid	Hypertension	0.037	28	2.69e-06	0.472	0.040	TRUE
1-lignoceroyl-GPC (24:0)	Hypertension	0.027	28	0.565	0.106	0.041	TRUE
Sulfate	Hypertension	0.058	23	4.94e-04	0.368	0.058	TRUE
4-hydroxy-2-oxoglutaric acid	Hypertension	0.047	23	0.491	0.318	0.041	TRUE
Glycoursodeoxycholate	Hypertension	0.029	22	0.034	0.934	0.025	TRUE
1-(1-enyl-palmitoyl)-GPC (p-16:0)	Hypertension	0.054	32	0.198	0.435	0.036	TRUE
Argininate	Hypertension	0.069	41	7.02e-24	0.490	0.201	TRUE
1-methyl-5-imidazoleacetate	Hypertension	0.036	25	0.031	0.616	0.028	TRUE
Hydantoin-5-propionate	Hypertension	0.026	31	0.157	0.347	0.026	TRUE
2-oxoarginine	Hypertension	0.035	27	1.17e-29	0.431	0.075	TRUE
Pantoate	Hypertension	0.033	23	0.017	0.242	0.055	TRUE
Formiminoglutamate	Hypertension	0.035	25	1.99e-08	0.070	0.068	TRUE
1-(1-enyl-palmitoyl)-GPE (p-16:0)	Hypertension	0.038	25	0.337	0.391	0.032	TRUE
S-methylcysteine sulfoxide	Hypertension	0.022	23	0.132	0.872	0.030	TRUE
2-o-methylascorbic acid	Hypertension	0.158	84	0.990	0.742	0.090	TRUE
Eicosanedioate (C20-DC)	Hypertension	0.025	30	0.570	0.400	0.031	TRUE
1-(1-enyl-stearoyl)-GPE (p-18:0)	Hypertension	0.040	28	2.33e-11	0.647	0.029	TRUE

Exposure	Outcome	R2	F	Heterogeneity	Pleiotropy	power	Direction
Beta-citrylglutamate	Hypertension	0.121	107	0.050	0.166	0.038	TRUE
N-oleoyltaurine	Hypertension	0.069	32	0.515	0.157	0.030	TRUE
Docosadioate (C22-DC)	Hypertension	0.028	23	0.027	0.230	0.045	TRUE
Oleoyl-linoleoyl-glycerol (18:1/18:2) [2]	Hypertension	0.083	33	1.1e-07	0.629	0.091	TRUE
Imidazole propionate	Hypertension	0.047	26	2.49e-05	0.076	0.032	TRUE
Carnitine C18:2	Hypertension	0.085	48	0.001	0.870	0.058	TRUE
Pregnanediol-3-glucuronide	Hypertension	0.086	42	4.49e-05	0.886	0.048	TRUE
Carboxyethyl-gaba	Hypertension	0.049	34	1.02e-06	0.691	0.079	TRUE
Alliin	Hypertension	0.076	59	3.14e-04	0.023	0.034	TRUE
Trimethylamine n-oxide	Hypertension	0.016	22	0.880	0.705	0.039	TRUE
Dihydroferulate	Hypertension	0.008	21	0.558	0.535	0.028	TRUE
Prolylglycine	Hypertension	0.036	25	5.43e-16	0.576	0.094	TRUE
N-palmitoylglycine	Hypertension	0.098	64	9.46e-06	0.777	0.080	TRUE
N-methyltaurine	Hypertension	0.042	23	0.017	0.214	0.044	TRUE
Succinimide	Hypertension	0.024	25	0.448	0.992	0.028	TRUE
N-acetylcarnosine	Hypertension	0.114	60	2.26e-07	0.194	0.056	TRUE
Histidine betaine (hercynine)	Hypertension	0.042	27	0.014	0.798	0.059	TRUE

Exposure	Outcome	R2	F	Heterogeneity	Pleiotropy	power	Direction
2-stearoyl-GPE (18:0)	Hypertension	0.061	27	0.023	0.714	0.028	TRUE
Margaroylcarnitine (C17)	Hypertension	0.037	26	1.11e-04	0.344	0.038	TRUE
(R)-3-hydroxybutyrylcarnitine	Hypertension	0.031	25	0.002	0.289	0.057	TRUE
Lanthionine	Hypertension	0.043	22	7.41e-11	0.343	0.029	TRUE
Mannonate	Hypertension	0.049	41	0.006	0.175	0.083	TRUE
3b-hydroxy-5-cholenoic acid	Hypertension	0.041	26	1e-07	0.941	0.027	TRUE
2-hydroxydecanoate	Hypertension	0.035	27	0.032	0.664	0.033	TRUE
Glycohyocholate	Hypertension	0.035	29	3.97e-07	0.096	0.106	TRUE
3-methyl catechol sulfate (2)	Hypertension	0.035	22	1.89e-09	0.515	0.045	TRUE
2-aminooctanoate	Hypertension	0.152	80	3.47e-28	0.742	0.036	TRUE
3-methyl catechol sulfate (1)	Hypertension	0.020	24	1.3e-05	0.664	0.046	TRUE
4-methylcatechol sulfate	Hypertension	0.040	24	1.14e-08	0.516	0.069	TRUE
Guaiacol sulfate	Hypertension	0.039	23	4.83e-04	0.605	0.034	TRUE
Gamma-CEHC glucuronide	Hypertension	0.048	29	0.004	0.526	0.025	TRUE
Dimethyl sulfone	Hypertension	0.036	25	0.329	0.856	0.051	TRUE
2-piperidinone	Hypertension	0.034	24	0.013	0.106	0.053	TRUE
Indolin-2-one	Hypertension	0.032	33	0.745	0.622	0.032	TRUE

Exposure	Outcome	R2	F	Heterogeneity	Pleiotropy	power	Direction
Sphingomyelin (d18:1/14:0, d16:1/16:0)	Hypertension	0.107	51	0.004	0.267	0.028	TRUE
3-acetylphenol sulfate	Hypertension	0.030	22	0.005	0.163	0.052	TRUE
Sphingomyelin (d18:2/16:0, d18:1/16:1)	Hypertension	0.039	23	0.062	0.175	0.036	TRUE
S-allylcysteine	Hypertension	0.031	26	0.155	0.305	0.025	TRUE
6-oxopiperidine-2-carboxylate	Hypertension	0.092	60	0.007	0.124	0.060	TRUE
2-aminophenol sulfate	Hypertension	0.037	22	0.260	0.317	0.028	TRUE
N-acetylalliin	Hypertension	0.053	30	0.065	0.375	0.029	TRUE
2-aminoheptanoate	Hypertension	0.027	23	0.113	0.840	0.026	TRUE
3-methoxytyramine sulfate	Hypertension	0.026	24	0.109	0.299	0.029	TRUE
N2,N5-diacetylornithine	Hypertension	0.108	58	1.35e-07	0.958	0.044	TRUE
Ferulic acid 4-sulfate	Hypertension	0.087	41	0.002	0.663	0.080	TRUE
1H-indole-7-acetic acid	Hypertension	0.032	22	0.069	0.294	0.032	TRUE
3-(3-hydroxyphenyl)propionate sulfate	Hypertension	0.029	22	0.005	0.399	0.033	TRUE
1-linolenoyl-GPC (18:3)	Hypertension	0.055	24	0.036	0.848	0.035	TRUE
O-sulfo-l-tyrosine	Hypertension	0.059	29	1.89e-46	0.573	0.101	TRUE
N-formylanthranilic acid	Hypertension	0.163	93	0.215	0.160	0.039	TRUE
Methionine sulfone	Hypertension	0.169	61	1.43e-14	0.916	0.134	TRUE

Exposure	Outcome	R2	F	Heterogeneity	Pleiotropy	power	Direction
Acisoga	Hypertension	0.094	41	0.171	0.878	0.078	TRUE
17alpha-hydroxypregnanolone glucuronide	Hypertension	0.110	53	0.023	0.382	0.034	TRUE
Etiocholanolone glucuronide	Hypertension	0.134	84	0.098	0.943	0.029	TRUE
Fructosyllysine	Hypertension	0.042	23	0.026	0.970	0.085	TRUE
1-(1-enyl-oleoyl)-GPE (p-18:1)	Hypertension	0.016	22	0.377	0.099	0.043	TRUE
N-acetyltaurine	Hypertension	0.053	37	2.38e-29	0.553	0.071	TRUE
1-palmitoyl-GPG (16:0)	Hypertension	0.062	31	0.615	0.122	0.036	TRUE
1-oleoyl-GPG (18:1)	Hypertension	0.091	60	0.002	0.096	0.030	TRUE
Octadecenedioylcarnitine (C18:1-DC)	Hypertension	0.150	95	0.008	0.815	0.053	TRUE
3-methylglutarylcarnitine (2)	Hypertension	0.171	69	1.31e-11	0.020	0.048	TRUE
Sphingomyelin (d18:1/24:1, d18:2/24:0)	Hypertension	0.022	23	0.117	0.970	0.025	TRUE
Methyl glucopyranoside (alpha + beta)	Hypertension	0.111	60	0.043	0.271	0.033	TRUE
9-hydroxystearate	Hypertension	0.040	24	4.55e-08	0.502	0.142	TRUE
Sphingomyelin (d18:2/14:0, d18:1/14:1)	Hypertension	0.042	25	2.46e-09	0.469	0.030	TRUE
1-dihomo-linolenylglycerol (20:3)	Hypertension	0.038	24	0.146	0.809	0.026	TRUE
4-hydroxychlorothalonil	Hypertension	0.085	61	0.747	0.113	0.025	TRUE
Vanillactate	Hypertension	0.049	25	0.230	0.183	0.042	TRUE

Exposure	Outcome	R2	F	Heterogeneity	Pleiotropy	power	Direction
Carnitine C14:1	Hypertension	0.039	23	0.049	0.176	0.037	TRUE
N-formylphenylalanine	Hypertension	0.039	23	6.24e-04	0.682	0.033	TRUE
2-acetamidophenol sulfate	Hypertension	0.022	23	0.325	0.140	0.026	TRUE
4-vinylguaiacol sulfate	Hypertension	0.017	23	0.276	0.144	0.050	TRUE
5alpha-androstan-3alpha,17beta-diol monosulfate (2)	Hypertension	0.064	34	2.35e-04	9.31e-04	0.030	TRUE
Vanillic alcohol sulfate	Hypertension	0.038	24	0.243	0.571	0.040	TRUE
Methyl-4-hydroxybenzoate sulfate	Hypertension	0.038	24	0.417	0.970	0.041	TRUE
Eugenol sulfate	Hypertension	0.030	25	0.441	0.869	0.028	TRUE
2-methoxyresorcinol sulfate	Hypertension	0.035	23	0.421	0.151	0.034	TRUE
Tyramine O-sulfate	Hypertension	0.027	23	0.809	0.612	0.032	TRUE
4-methylguaiacol sulfate	Hypertension	0.024	22	0.026	0.185	0.029	TRUE
3-hydroxypyridine sulfate	Hypertension	0.034	24	2.04e-06	0.918	0.051	TRUE
Arabonate/xylonate	Hypertension	0.047	26	1.79e-08	0.886	0.025	TRUE
Octadecanedioylcarnitine (C18-DC)	Hypertension	0.162	88	0.098	0.424	0.040	TRUE
Phenylacetylcarnitine	Hypertension	0.058	32	0.102	0.905	0.063	TRUE
Ethylparaben sulfate	Hypertension	0.030	22	0.034	0.629	0.037	TRUE
Sphingomyelin (d18:1/20:2, d18:2/20:1, d16:1/22:2)	Hypertension	0.062	40	0.039	0.141	0.035	TRUE

Exposure	Outcome	R2	F	Heterogeneity	Pleiotropy	power	Direction
Sphingomyelin (d18:1/20:1, d18:2/20:0)	Hypertension	0.084	39	6.37e-07	0.455	0.042	TRUE
Behenoyl sphingomyelin (d18:1/22:0)	Hypertension	0.028	24	0.028	0.104	0.031	TRUE
Propyl 4-hydroxybenzoate sulfate	Hypertension	0.027	23	3.25e-05	0.811	0.026	TRUE
4-methoxyphenol sulfate	Hypertension	0.034	24	0.095	0.049	0.042	TRUE
Umbelliferone sulfate	Hypertension	0.028	23	0.999	0.974	0.061	TRUE
Sphingomyelin (d18:1/20:0, d16:1/22:0)	Hypertension	0.110	51	4.38e-15	0.847	0.030	TRUE
6-hydroxyindole sulfate	Hypertension	0.029	27	4.29e-04	0.927	0.036	TRUE
P-cresol glucuronide	Hypertension	0.045	25	0.007	0.392	0.047	TRUE
N-carbamoylalanine	Hypertension	0.024	22	0.380	0.836	0.040	TRUE
Citraconate/glutaconate	Hypertension	0.034	24	0.009	0.455	0.050	TRUE
1,2,3-benzenetriol sulfate (2)	Hypertension	0.026	22	0.339	0.503	0.040	TRUE
3-methoxycatechol sulfate (2)	Hypertension	0.036	23	0.106	0.227	0.026	TRUE
C-glycosyltryptophan	Hypertension	0.066	24	1.31e-32	0.828	0.048	TRUE
3-methoxycatechol sulfate (1)	Hypertension	0.037	22	0.667	0.684	0.030	TRUE
Sphingomyelin (d18:1/22:1, d18:2/22:0, d16:1/24:1)	Hypertension	0.059	33	6.59e-05	0.299	0.027	TRUE
Arabitol/xylitol	Hypertension	0.045	25	3.23e-25	0.410	0.125	TRUE
3-hydroxyhexanoate	Hypertension	0.027	28	0.052	0.627	0.049	TRUE

Exposure	Outcome	R2	F	Heterogeneity	Pleiotropy	power	Direction
Sphingomyelin (d18:1/22:2, d18:2/22:1, d16:1/24:2)	Hypertension	0.061	36	0.030	0.220	0.101	TRUE
3beta-hydroxy-5-cholestenoate	Hypertension	0.100	50	0.637	0.607	0.026	TRUE
N-acetylkynurenine (2)	Hypertension	0.163	104	0.103	0.810	0.073	TRUE
Dopamine 4-sulfate	Hypertension	0.046	24	0.620	0.878	0.036	TRUE
Sphingomyelin (d17:1/16:0, d18:1/15:0, d16:1/17:0)	Hypertension	0.075	42	0.006	0.760	0.056	TRUE
Adipoylcarnitine (C6-DC)	Hypertension	0.077	46	0.051	0.547	0.044	TRUE
Dopamine 3-o-sulfate	Hypertension	0.026	24	0.330	0.753	0.052	TRUE
Lignoceroyl sphingomyelin (d18:1/24:0)	Hypertension	0.042	25	9.97e-11	0.497	0.040	TRUE
N-acetylglucosamine/n-acetylgalactosamine	Hypertension	0.074	48	0.513	0.868	0.039	TRUE
Glycochenodeoxycholate 3-sulfate	Hypertension	0.100	44	4.44e-08	0.156	0.068	TRUE
Trans 3,4-methyleneheptanoate	Hypertension	0.030	25	0.085	0.943	0.028	TRUE
Taurodeoxycholic acid 3-sulfate	Hypertension	0.098	84	0.114	0.819	0.040	TRUE
Phenol glucuronide	Hypertension	0.030	23	0.918	0.490	0.034	TRUE
Glycodeoxycholate 3-sulfate	Hypertension	0.156	79	0.344	0.285	0.030	TRUE
4-hydroxyphenylacetoylcarnitine	Hypertension	0.070	33	0.009	0.545	0.026	TRUE
1-stearoyl-2-oleoyl-gpc (18:0/18:1)	Hypertension	0.062	25	0.216	0.351	0.036	TRUE
Linoleoyl ethanolamide	Hypertension	0.044	33	0.666	0.811	0.047	TRUE

Exposure	Outcome	R2	F	Heterogeneity	Pleiotropy	power	Direction
1,2-dilinoleoyl-GPC (18:2/18:2)	Hypertension	0.066	57	0.109	0.296	0.032	TRUE
Nonanoylcarnitine (C9)	Hypertension	0.107	77	0.394	0.322	0.060	TRUE
2-hydroxybutyrate/2-hydroxyisobutyrate	Hypertension	0.018	25	0.072	0.416	0.049	TRUE
Sphingomyelin (d18:2/23:0, d18:1/23:1, d17:1/24:1)	Hypertension	0.036	27	4.23e-04	0.822	0.043	TRUE
Tricosanoyl sphingomyelin (d18:1/23:0)	Hypertension	0.043	24	2.85e-06	0.586	0.028	TRUE
Palmitoyl dihydrosphingomyelin (d18:0/16:0)	Hypertension	0.076	27	2.04e-10	0.285	0.077	TRUE
1-palmitoleoylglycerol (16:1)	Hypertension	0.055	23	3.33e-05	0.587	0.057	TRUE
1-stearoyl-2-linoleoyl-gpc (18:0/18:2)	Hypertension	0.071	35	5.98e-06	0.441	0.031	TRUE
1-palmitoyl-2-palmitoleoyl-gpc (16:0/16:1)	Hypertension	0.038	22	0.013	0.004	0.028	TRUE
1-stearoyl-2-linoleoyl-GPE (18:0/18:2)	Hypertension	0.131	70	0.015	0.578	0.034	TRUE
Sphingomyelin (d18:2/24:1, d18:1/24:2)	Hypertension	0.045	24	3.57e-07	0.260	0.048	TRUE
1-palmitoyl-2-stearoyl-gpc (16:0/18:0)	Hypertension	0.084	35	0.048	0.481	0.028	TRUE
Oleate/vaccenate (18:1)	Hypertension	0.036	23	1.12e-10	0.109	0.033	TRUE
1-palmitoyl-2-docosahexaenoyl-gpc (16:0/22:6)	Hypertension	0.056	39	4.28e-06	0.136	0.075	TRUE
1-(1-enyl-stearoyl)-2-oleoyl-GPE (p-18:0/18:1)	Hypertension	0.032	27	1.26e-05	0.873	0.039	TRUE
1-stearoyl-2-arachidonoyl-GPE (18:0/20:4)	Hypertension	0.180	86	2.13e-05	0.049	0.057	TRUE
1-stearoyl-2-docosahexaenoyl-gpc (18:0/22:6)	Hypertension	0.089	41	4.28e-04	0.129	0.046	TRUE

Exposure	Outcome	R2	F	Heterogeneity	Pleiotropy	power	Direction
Sphingomyelin (d18:1/17:0, d17:1/18:0, d19:1/16:0)	Hypertension	0.058	29	6.35e-05	0.790	0.051	TRUE
1-(1-enyl-palmitoyl)-2-arachidonoyl-GPE (p-16:0/20:4)	Hypertension	0.040	30	0.237	0.110	0.047	TRUE
Myristoyl dihydrosphingomyelin (d18:0/14:0)	Hypertension	0.050	32	0.395	0.110	0.027	TRUE
5-hydroxyindole sulfate	Hypertension	0.032	27	0.392	0.493	0.036	TRUE
1-palmitoyl-2-dihomo-linolenoyl-GPC (16:0/20:3n3 or 6)	Hypertension	0.100	57	0.501	0.853	0.030	TRUE
1-(1-enyl-palmitoyl)-2-arachidonoyl-gpc (p-16:0/20:4)	Hypertension	0.186	71	1.11e-10	0.451	0.036	TRUE
Sphingomyelin (d18:1/21:0, d17:1/22:0, d16:1/23:0)	Hypertension	0.072	51	1.03e-05	0.655	0.093	TRUE
1-palmitoyl-2-arachidonoyl-GPE (16:0/20:4)	Hypertension	0.174	108	0.002	0.872	0.038	TRUE
Lactosyl-N-palmitoyl-sphingosine (d18:1/16:0)	Hypertension	0.053	32	0.122	0.603	0.036	TRUE
Sphingomyelin (d18:0/18:0, d19:0/17:0)	Hypertension	0.059	26	1.95e-07	0.112	0.072	TRUE
1-(1-enyl-palmitoyl)-2-oleoyl-gpc (p-16:0/18:1)	Hypertension	0.055	28	0.003	0.393	0.035	TRUE
1-(1-enyl-stearoyl)-2-arachidonoyl-GPE (p-18:0/20:4)	Hypertension	0.072	47	1.31e-10	0.463	0.101	TRUE
Behenoyl dihydrosphingomyelin (d18:0/22:0)	Hypertension	0.077	28	1.5e-12	0.888	0.058	TRUE
1-(1-enyl-palmitoyl)-2-linoleoyl-GPC (p-16:0/18:2)	Hypertension	0.034	26	0.002	0.418	0.059	TRUE
1-(1-enyl-palmitoyl)-2-oleoyl-GPE (p-16:0/18:1)	Hypertension	0.053	25	4.42e-07	0.144	0.053	TRUE
1-palmitoyl-2-arachidonoyl-GPI (16:0/20:4)	Hypertension	0.052	31	0.607	0.346	0.026	TRUE
1-palmitoyl-2-docosahexaenoyl-GPE (16:0/22:6)	Hypertension	0.138	93	5.81e-07	0.172	0.069	TRUE

Exposure	Outcome	R2	F	Heterogeneity	Pleiotropy	power	Direction
N-palmitoyl-sphinganine (d18:0/16:0)	Hypertension	0.051	25	6.14e-13	0.855	0.031	TRUE
1-stearoyl-2-docosahexaenoyl-GPE (18:0/22:6)	Hypertension	0.102	51	8.59e-07	0.373	0.067	TRUE
Gamma-tocopherol/beta-tocopherol	Hypertension	0.034	24	5.29e-04	0.804	0.031	TRUE
1-stearoyl-2-linoleoyl-GPI (18:0/18:2)	Hypertension	0.094	49	1.99e-07	0.181	0.028	TRUE
1,2-dilinoleoyl-GPE (18:2/18:2)	Hypertension	0.048	41	0.006	0.278	0.042	TRUE
Phenylacetylglutamate	Hypertension	0.043	28	0.034	0.329	0.048	TRUE
1-(1-enyl-palmitoyl)-2-palmitoleoyl-GPC (P-16:0/16:1)	Hypertension	0.137	51	9e-13	0.294	0.029	TRUE
Palmitoylcholine	Hypertension	0.052	23	0.067	0.616	0.040	TRUE
Thioproline	Hypertension	0.032	26	0.056	0.055	0.028	TRUE
1-linoleoyl-2-arachidonoyl-GPC (18:2/20:4n6)	Hypertension	0.111	47	0.004	0.809	0.098	TRUE
1-linoleoyl-GPG (18:2)	Hypertension	0.097	55	0.451	0.603	0.031	TRUE
1-(1-enyl-palmitoyl)-2-linoleoyl-GPE (p-16:0/18:2)	Hypertension	0.054	35	8.02e-08	0.216	0.076	TRUE
1-(1-enyl-stearoyl)-2-linoleoyl-GPE (p-18:0/18:2)	Hypertension	0.050	35	2.06e-08	0.828	0.066	TRUE
1-stearoyl-2-oleoyl-GPI (18:0/18:1)	Hypertension	0.079	47	2.51e-06	0.800	0.026	TRUE
1-myristoyl-2-arachidonoyl-GPC (14:0/20:4)	Hypertension	0.107	51	7.21e-06	0.535	0.051	TRUE
1-oleoyl-2-arachidonoyl-GPE (18:1/20:4)	Hypertension	0.164	79	0.009	0.417	0.047	TRUE
1-palmitoyl-2-oleoyl-GPI (16:0/18:1)	Hypertension	0.066	37	1.08e-04	0.513	0.032	TRUE

Exposure	Outcome	R2	F	Heterogeneity	Pleiotropy	power	Direction
1-oleoyl-2-docosahexaenoyl-GPC (18:1/22:6)	Hypertension	0.067	27	4.13e-09	0.376	0.050	TRUE
1-oleoyl-2-linoleoyl-GPE (18:1/18:2)	Hypertension	0.153	78	0.010	0.629	0.031	TRUE
1-(1-enyl-palmitoyl)-2-palmitoyl-GPC (P-16:0/16:0)	Hypertension	0.143	58	1.23e-06	0.060	0.034	TRUE
Oleoylcholine	Hypertension	0.022	23	0.033	0.264	0.034	TRUE
Glycosyl-N-palmitoyl-sphingosine (d18:1/16:0)	Hypertension	0.077	43	0.081	0.908	0.050	TRUE
Arachidonoylcholine	Hypertension	0.043	36	0.310	0.992	0.032	TRUE
2-methylserine	Hypertension	0.034	23	3.1e-09	0.424	0.067	TRUE
(S)-3-hydroxybutyrylcarnitine	Hypertension	0.054	35	0.001	0.540	0.035	TRUE
Methylsuccinoylcarnitine	Hypertension	0.151	64	0.003	0.654	0.025	TRUE
Dihomo-linolenoyl-choline	Hypertension	0.041	23	4.63e-09	0.888	0.034	TRUE
Docosahexaenoylcholine	Hypertension	0.027	22	0.005	0.723	0.038	TRUE
Glycocholate glucuronide (1)	Hypertension	0.028	23	0.244	0.916	0.025	TRUE
Caffeic acid sulfate	Hypertension	0.044	23	0.069	0.697	0.073	TRUE
Ascorbic acid 2-sulfate	Hypertension	0.042	25	0.155	0.897	0.026	TRUE
1-linoleoyl-2-linolenoyl-GPC (18:2/18:3)	Hypertension	0.059	38	0.013	0.410	0.027	TRUE
Catechol glucuronide	Hypertension	0.037	22	0.239	0.958	0.032	TRUE
Furaneol sulfate	Hypertension	0.042	39	0.030	0.754	0.054	TRUE

Exposure	Outcome	R2	F	Heterogeneity	Pleiotropy	power	Direction
Ceramide (d18:1/24:1)	Hypertension	0.109	49	1.98e-04	0.612	0.038	TRUE
N-oleoylserine	Hypertension	0.053	40	0.270	0.695	0.037	TRUE
Perfluorooctanesulfonate (PFOS)	Hypertension	0.036	23	0.002	0.066	0.028	TRUE
Pimeloylcarnitine/3-methyladipoylcarnitine (C7-DC)	Hypertension	0.067	40	0.014	0.309	0.026	TRUE
1-palmitoleoyl-2-linolenoyl-GPC (16:1/18:3)	Hypertension	0.074	45	0.079	0.487	0.031	TRUE
Linoleoyl-arachidonoyl-glycerol (18:2/20:4) [1]	Hypertension	0.081	69	1.3e-10	0.223	0.040	TRUE
Hexadecadienoate (16:2n6)	Hypertension	0.019	22	0.181	0.511	0.025	TRUE
Glycosyl-N-behenoyl-sphingadienine (d18:2/22:0)	Hypertension	0.095	38	0.354	0.633	0.042	TRUE
4-hydroxyphenylacetylglutamine	Hypertension	0.042	25	2.22e-30	0.942	0.085	TRUE
Gamma-glutamyl-alpha-lysine	Hypertension	0.051	25	0.972	0.695	0.028	TRUE
N-palmitoyl-sphingadienine (d18:2/16:0)	Hypertension	0.030	23	1.61e-04	0.148	0.042	TRUE
Linoleoyl-arachidonoyl-glycerol (18:2/20:4) [2]	Hypertension	0.122	43	0.024	0.145	0.028	TRUE
Palmitoleoylcarnitine (C16:1)	Hypertension	0.035	27	2.49e-08	0.897	0.028	TRUE
N-stearoyl-sphingadienine (d18:2/18:0)	Hypertension	0.037	25	0.221	0.107	0.050	TRUE
2,3-dihydroxy-2-methylbutyrate	Hypertension	0.022	23	0.546	0.273	0.031	TRUE
Stearoylcholine	Hypertension	0.028	23	1.62e-05	0.024	0.090	TRUE
Glycosyl-N-tricosanoyl-sphingadienine (d18:2/23:0)	Hypertension	0.047	36	0.132	0.831	0.049	TRUE

Exposure	Outcome	R2	F	Heterogeneity	Pleiotropy	power	Direction
N-palmitoyl-heptadecasphingosine (d17:1/16:0)	Hypertension	0.063	31	5.63e-08	0.475	0.063	TRUE
Ceramide (d18:1/14:0, d16:1/16:0)	Hypertension	0.066	30	0.097	0.213	0.088	TRUE
Glycosyl-N-(2-hydroxynervonoyl)-sphingosine (d18:1/24:1(2OH))	Hypertension	0.099	52	8.43e-14	0.195	0.029	TRUE
Glycosyl ceramide (d18:2/24:1, d18:1/24:2)	Hypertension	0.088	39	0.351	0.276	0.051	TRUE
Glycosyl ceramide (d18:1/23:1, d17:1/24:1)	Hypertension	0.035	29	0.044	0.502	0.030	TRUE
Sphingadienine	Hypertension	0.031	24	0.127	0.127	0.045	TRUE
Ceramide (d18:1/17:0, d17:1/18:0)	Hypertension	0.039	25	0.024	0.997	0.027	TRUE
Nisinate (24:6n3)	Hypertension	0.032	26	0.091	0.411	0.041	TRUE
Sphingomyelin (d18:0/20:0, d16:0/22:0)	Hypertension	0.127	40	1.03e-07	0.276	0.064	TRUE
Sphingomyelin (d18:2/24:2)	Hypertension	0.040	24	0.485	0.459	0.072	TRUE
Sphingomyelin (d17:2/16:0, d18:2/15:0)	Hypertension	0.065	32	6e-04	0.196	0.042	TRUE
Sphingomyelin (d18:2/23:1)	Hypertension	0.049	27	0.023	0.731	0.052	TRUE
Behenoylcarnitine (C22)	Hypertension	0.069	30	2.15e-05	0.452	0.040	TRUE
Sphingomyelin (d18:2/18:1)	Hypertension	0.022	22	0.848	0.396	0.033	TRUE
Sphingomyelin (d18:2/21:0, d16:2/23:0)	Hypertension	0.080	30	1.55e-06	0.828	0.147	TRUE
Linoleoylcholine	Hypertension	0.042	23	0.288	0.395	0.026	TRUE
Sphingomyelin (d18:1/19:0, d19:1/18:0)	Hypertension	0.091	51	6.63e-07	0.277	0.029	TRUE

Exposure	Outcome	R2	F	Heterogeneity	Pleiotropy	power	Direction
Linolenoylcarnitine (C18:3)	Hypertension	0.058	29	0.060	0.726	0.026	TRUE
Heneicosapentaenoate (21:5n3)	Hypertension	0.022	23	0.360	0.416	0.029	TRUE
Sphingomyelin (d18:1/25:0, d19:0/24:1, d20:1/23:0, d19:1/24:0)	Hypertension	0.128	55	0.123	0.177	0.040	TRUE
Arachidoylcarnitine (C20)	Hypertension	0.056	29	1.65e-04	0.535	0.056	TRUE
Cerotoylcarnitine (C26)	Hypertension	0.126	48	3.92e-09	0.275	0.051	TRUE
Lignoceroylcarnitine (C24)	Hypertension	0.099	52	0.009	0.095	0.030	TRUE
Docosatrienoate (22:3n6)	Hypertension	0.038	29	0.740	0.890	0.048	TRUE
Dihomo-linolenoylcarnitine (C20:3n3 or 6)	Hypertension	0.205	81	0.006	0.960	0.074	TRUE
Carotene diol (2)	Hypertension	0.065	39	1.06e-08	0.218	0.028	TRUE
Ethyl alpha-glucopyranoside	Hypertension	0.041	23	0.078	0.012	0.038	TRUE
Carotene diol (1)	Hypertension	0.078	39	9.48e-09	0.262	0.032	TRUE
Nervonoylcarnitine (C24:1)	Hypertension	0.081	45	2.25e-05	0.630	0.042	TRUE
Dihomo-linoleoylcarnitine (C20:2)	Hypertension	0.100	47	0.007	0.462	0.032	TRUE
Glycosyl ceramide (d18:1/20:0, d16:1/22:0)	Hypertension	0.111	44	0.027	0.016	0.046	TRUE
Docosahexaenoylcarnitine (C22:6)	Hypertension	0.061	25	4.43e-08	0.609	0.045	TRUE
N,N,N-trimethyl-5-aminovalerate	Hypertension	0.089	32	5.16e-17	0.240	0.102	TRUE
Eicosenoylcarnitine (C20:1)	Hypertension	0.071	28	3.89e-07	0.787	0.085	TRUE

Exposure	Outcome	R2	F	Heterogeneity	Pleiotropy	power	Direction
Ximenoylcarnitine (C26:1)	Hypertension	0.127	53	0.008	0.960	0.045	TRUE
Arachidonoylcarnitine (C20:4)	Hypertension	0.233	88	3.12e-06	0.163	0.096	TRUE
5-dodecenoylcarnitine (C12:1)	Hypertension	0.041	24	2.08e-05	0.592	0.063	TRUE
2-butenoylglycine	Hypertension	0.071	40	2.16e-06	0.160	0.081	TRUE
(N(1) + N(8))-acetylspermidine	Hypertension	0.131	53	3.07e-06	0.391	0.028	TRUE
Hydroxy-cmpf	Hypertension	0.033	25	0.003	0.680	0.026	TRUE
2-furoylcarnitine	Hypertension	0.019	23	0.004	0.103	0.066	TRUE
Cortolone glucuronide (1)	Hypertension	0.051	25	1.17e-07	0.688	0.048	TRUE
Carotene diol (3)	Hypertension	0.058	32	0.010	0.242	0.037	TRUE
3-hydroxyoleoylcarnitine	Hypertension	0.051	25	1.31e-04	0.802	0.037	TRUE
Dodecenedioate (C12:1-DC)	Hypertension	0.058	35	0.003	0.467	0.028	TRUE
Trans-2-hexenoylglycine	Hypertension	0.062	37	7.1e-06	0.130	0.135	TRUE
2-hydroxyarachidate	Hypertension	0.017	23	0.013	0.354	0.031	TRUE
3-hydroxyphenylacetoylglutamine	Hypertension	0.005	22	0.591		0.028	TRUE
Heptenedioate (C7:1-DC)	Hypertension	0.021	22	0.979	0.635	0.044	TRUE
Hexadecenedioate (C16:1-DC)	Hypertension	0.188	80	0.079	0.666	0.037	TRUE
Octadecenedioate (C18:1-DC)	Hypertension	0.127	73	0.434	0.721	0.052	TRUE

Exposure	Outcome	R2	F	Heterogeneity	Pleiotropy	power	Direction
Dihydroferulic acid sulfate	Hypertension	0.045	24	0.207	0.158	0.028	TRUE
Glutamine conjugate of C7H12O2	Hypertension	0.042	25	0.137	0.260	0.030	TRUE
Hydroxyasparagine	Hypertension	0.035	24	5.79e-38	0.937	0.042	TRUE
Sphingomyelin (d17:1/14:0, d16:1/15:0)	Hypertension	0.058	28	2.09e-06	0.353	0.062	TRUE
Octadecadienedioate (C18:2-DC)	Hypertension	0.158	86	0.362	0.734	0.052	TRUE
Perfluorooctanoate (PFOA)	Hypertension	0.034	24	0.006	0.170	0.079	TRUE
3-carboxy-4-methyl-5-pentyl-2-furanpropionate (3-CMPFP)	Hypertension	0.063	38	0.075	0.771	0.032	TRUE
Glutamine conjugate of C6H10O2 (2)	Hypertension	0.028	29	0.026	0.707	0.051	TRUE
3-hydroxybutyroylglycine	Hypertension	0.102	58	0.002	0.047	0.183	TRUE
Glycine conjugate of C10H14O2 (1)	Hypertension	0.058	44	0.015	0.240	0.026	TRUE
Glutamine conjugate of C6H10O2 (1)	Hypertension	0.032	29	3.24e-04	0.355	0.041	TRUE
N-methylhydroxyproline	Hypertension	0.023	24	0.147	0.703	0.050	TRUE
Glucuronide of C10H18O2 (7)	Hypertension	0.028	24	0.608	0.212	0.031	TRUE
Glyco-beta-muricholate	Hypertension	0.088	58	0.443	0.578	0.081	TRUE
Glucuronide of C12H22O4 (1)	Hypertension	0.026	24	0.932	0.590	0.031	TRUE
N,n,n-trimethyl-alanylproline betaine (tmap)	Hypertension	0.037	24	1.48e-25	0.752	0.067	TRUE
Glycine conjugate of C10H12O2	Hypertension	0.037	26	1.73e-06	0.431	0.030	TRUE

Exposure	Outcome	R2	F	Heterogeneity	Pleiotropy	power	Direction
N-acetyl-2-aminooctanoate	Hypertension	0.153	81	6.34e-11	0.173	0.141	TRUE
3-formylindole	Hypertension	0.161	83	0.607	0.667	0.027	TRUE
Gamma-glutamylcitrulline	Hypertension	0.059	26	7.39e-06	0.946	0.046	TRUE
8-methoxykynurenate	Hypertension	0.034	26	2.68e-04	0.090	0.063	TRUE
Tetradecadienoate (14:2)	Hypertension	0.041	26	2.46e-06	0.551	0.027	TRUE
3-amino-2-piperidone	Hypertension	0.027	29	0.435	0.184	0.042	TRUE
Glucuronide of piperine metabolite C17H21NO3 (3)	Hypertension	0.038	29	3.4e-04	0.216	0.041	TRUE
Dodecadienoate (12:2)	Hypertension	0.074	41	0.020	0.609	0.062	TRUE
N-lactoyl tyrosine	Hypertension	0.027	23	0.687	0.835	0.027	TRUE
N,N-dimethylalanine	Hypertension	0.062	30	2.56e-05	0.464	0.033	TRUE
Ethyl beta-glucopyranoside	Hypertension	0.090	79	0.564	0.517	0.040	TRUE
N-lactoyl phenylalanine	Hypertension	0.022	22	0.224	0.319	0.043	TRUE
N-acetyl-isoputreanine	Hypertension	0.166	61	0.040	0.509	0.139	TRUE
2,2'-Methylenebis(6-tert-butyl-p-cresol)	Hypertension	0.031	24	0.186	0.451	0.050	TRUE
N-lactoyl isoleucine	Hypertension	0.021	22	0.726	0.983	0.040	TRUE
2-hydroxysebacate	Hypertension	0.046	35	0.207	0.708	0.031	TRUE
Ascorbic acid 3-sulfate	Hypertension	0.039	25	0.261	0.435	0.043	TRUE

Exposure	Outcome	R2	F	Heterogeneity	Pleiotropy	power	Direction
N-lactoyl valine	Hypertension	0.029	22	0.001	0.817	0.029	TRUE
6-bromotryptophan	Hypertension	0.115	61	0.247	0.981	0.025	TRUE
Delta-CEHC	Hypertension	0.029	24	2.28e-08	0.886	0.026	TRUE
Indoleacetoylcarnitine	Hypertension	0.137	43	0.007	0.953	0.039	TRUE
N-acetyl leucine	Hypertension	0.024	22	0.670	0.913	0.034	TRUE
3-indoleglyoxylic acid	Hypertension	0.097	69	0.690	0.190	0.025	TRUE
Sulfate of piperine metabolite C18H21NO3 (1)	Hypertension	0.035	24	1.75e-05	0.128	0.059	TRUE
Methyl vanillate sulfate	Hypertension	0.027	23	0.096	0.354	0.029	TRUE
Glucuronide of piperine metabolite C17H21NO3 (4)	Hypertension	0.042	35	0.105	0.690	0.031	TRUE
Sulfate of piperine metabolite C16H19NO3 (3)	Hypertension	0.046	24	0.193	0.841	0.056	TRUE
Sulfate of piperine metabolite C16H19NO3 (2)	Hypertension	0.043	23	0.265	0.148	0.054	TRUE
3-hydroxypyridine glucuronide	Hypertension	0.037	24	2.2e-04	0.218	0.026	TRUE
Glucuronide of piperine metabolite C17H21NO3 (5)	Hypertension	0.037	31	0.052	0.730	0.035	TRUE
2-naphthol sulfate	Hypertension	0.034	23	0.512	0.613	0.040	TRUE
11beta-hydroxyandrosterone glucuronide	Hypertension	0.087	36	1.24e-12	0.004	0.062	TRUE
4-ethylcatechol sulfate	Hypertension	0.028	23	0.010	0.965	0.112	TRUE
Sulfate of piperine metabolite C18H21NO3 (3)	Hypertension	0.039	25	0.327	0.223	0.026	TRUE

Exposure	Outcome	R2	F	Heterogeneity	Pleiotropy	power	Direction
(2,4 or 2,5)-dimethylphenol sulfate	Hypertension	0.028	23	0.980	0.974	0.040	TRUE
11beta-hydroxyetiocholanolone glucuronide	Hypertension	0.035	24	0.349	0.773	0.050	TRUE
Cholic acid glucuronide	Hypertension	0.045	34	1.01e-04	0.022	0.033	TRUE
4-allylcatechol sulfate	Hypertension	0.030	23	0.244	0.184	0.051	TRUE
Glycoursodeoxycholic acid sulfate (1)	Hypertension	0.049	27	0.538	0.215	0.028	TRUE
Dihydrocaffeate sulfate (2)	Hypertension	0.027	22	0.091	0.176	0.035	TRUE
4-methylhexanoylglutamine	Hypertension	0.025	43	6.78e-04	0.094	0.044	TRUE
2-ketocaprylate	Hypertension	0.085	40	0.013	0.026	0.031	TRUE
3-hydroxy-2-methylpyridine sulfate	Hypertension	0.032	22	0.001	0.934	0.067	TRUE
5-hydroxy-2-methylpyridine sulfate	Hypertension	0.046	24	0.007	0.299	0.045	TRUE
3-ethylcatechol sulfate (2)	Hypertension	0.027	23	0.103	0.523	0.043	TRUE
3-hydroxyhexanoylcarnitine (1)	Hypertension	0.047	32	0.125	0.758	0.053	TRUE
2,6-dihydroxybenzoic acid	Hypertension	0.052	31	0.010	0.605	0.087	TRUE
3-ethylcatechol sulfate (1)	Hypertension	0.036	23	8.33e-04	0.429	0.061	TRUE
Lithocholate sulfate (1)	Hypertension	0.074	34	0.060	0.133	0.038	TRUE
4-acetylcatechol sulfate (1)	Hypertension	0.013	22	0.638	0.284	0.038	TRUE
2,3-dihydroxy-5-methylthio-4-pentenoate (dmtpa)	Hypertension	0.054	25	1.63e-39	0.684	0.103	TRUE

Exposure	Outcome	R2	F	Heterogeneity	Pleiotropy	power	Direction
Picolinoylglycine	Hypertension	0.034	26	8.31e-14	0.539	0.095	TRUE
Succinoyltaurine	Hypertension	0.148	79	0.003	0.130	0.045	TRUE
Undecenoylcarnitine (C11:1)	Hypertension	0.103	51	0.007	0.492	0.041	TRUE
Palmitoyl-sphingosine-phosphoethanolamine (d18:1/16:0)	Hypertension	0.048	25	5.53e-06	0.258	0.044	TRUE
Pregnenetriol disulfate	Hypertension	0.139	104	0.362	0.406	0.027	TRUE
Tetradecadienedioate (C14:2-DC)	Hypertension	0.106	45	1.07e-04	0.685	0.049	TRUE
Hydroxy-N6,N6,N6-trimethyllysine	Hypertension	0.041	23	6.12e-07	0.320	0.102	TRUE
Pregnenetriol sulfate	Hypertension	0.089	34	7.55e-19	0.607	0.080	TRUE
Taurochenodeoxycholic acid 3-sulfate	Hypertension	0.074	32	1.9e-04	0.124	0.071	TRUE
Hydroxypalmitoyl sphingomyelin (d18:1/16:0(OH))	Hypertension	0.094	40	7.63e-06	0.192	0.046	TRUE
5-hydroxymethyl-2-furoylcarnitine	Hypertension	0.028	23	0.002	0.312	0.029	TRUE
Eicosenedioate (C20:1-DC)	Hypertension	0.069	54	0.546	0.641	0.031	TRUE
3-hydroxydecanoylcarnitine	Hypertension	0.048	24	0.001	0.397	0.044	TRUE
Levulinoylcarnitine	Hypertension	0.055	35	0.070	0.212	0.026	TRUE
Cis 3,4-methyleneheptanoate	Hypertension	0.053	25	6.74e-08	0.106	0.100	TRUE
Branched-chain, straight-chain, or cyclopropyl 12:1 fatty acid	Hypertension	0.054	30	1.71e-04	0.339	0.067	TRUE
Deoxycholic acid 12-sulfate	Hypertension	0.095	62	4.68e-04	0.809	0.039	TRUE

Exposure	Outcome	R2	F	Heterogeneity	Pleiotropy	power	Direction
Branched-chain, straight-chain, or cyclopropyl 10:1 fatty acid (1)	Hypertension	0.016	22	0.035	0.627	0.037	TRUE
3,5-dichloro-2,6-dihydroxybenzoic acid	Hypertension	0.056	43	0.554	0.978	0.039	TRUE
Vanillic acid glycine	Hypertension	0.043	28	1.11e-06	0.014	0.111	TRUE
(S)-a-amino-omega-caprolactam	Hypertension	0.027	28	1.41e-28	0.797	0.129	TRUE
3-bromo-5-chloro-2,6-dihydroxybenzoic acid	Hypertension	0.054	41	0.400	0.207	0.029	TRUE
Dibutyl sulfosuccinate	Hypertension	0.052	23	0.108	0.683	0.025	TRUE
Metabolonic lactone sulfate	Hypertension	0.245	136	2.5e-05	0.173	0.046	TRUE
2-hydroxy-4-(methylthio)butanoic acid	Hypertension	0.158	94	5.17e-05	0.765	0.077	TRUE
Pentose acid	Hypertension	0.040	24	0.656	0.418	0.069	TRUE
Branched chain 14:0 dicarboxylic acid	Hypertension	0.022	23	0.157	0.063	0.028	TRUE
S-carboxyethylcysteine	Hypertension	0.039	25	0.081	0.298	0.033	TRUE
N-succinyl-phenylalanine	Hypertension	0.089	69	2.6e-04	0.194	0.058	TRUE
(2 or 3)-decenoate (10:1n7 or n8)	Hypertension	0.063	29	0.212	0.461	0.064	TRUE
Bilirubin degradation product, C17H20N2O5 (2)	Hypertension	0.103	69	0.215	0.744	0.052	TRUE
Bilirubin degradation product, C16H18N2O5 (4)	Hypertension	0.124	142	0.016	0.770	0.027	TRUE
Cis-3,4-methyleneheptanoylcarnitine	Hypertension	0.078	36	1.06e-12	0.438	0.089	TRUE
Tetrahydrocortisol glucuronide	Hypertension	0.067	29	0.954	0.720	0.035	TRUE

Exposure	Outcome	R2	F	Heterogeneity	Pleiotropy	power	Direction
N-acetyl-2-aminoadipate	Hypertension	0.094	50	0.004	0.095	0.050	TRUE
Bilirubin degradation product, C17H20N2O5 (1)	Hypertension	0.104	65	0.424	0.263	0.044	TRUE
2-methoxyhydroquinone sulfate (1)	Hypertension	0.025	23	0.049	0.675	0.071	TRUE
Bilirubin degradation product, C16H18N2O5 (3)	Hypertension	0.155	94	0.019	0.665	0.040	TRUE
3-hydroxyoctanoylcarnitine (1)	Hypertension	0.037	26	0.001	0.480	0.026	TRUE
2-methoxyhydroquinone sulfate (2)	Hypertension	0.022	23	0.280	0.455	0.036	TRUE
Cis-3,4-methyleneheptanoylglycine	Hypertension	0.107	77	0.005	0.019	0.155	TRUE
Bilirubin degradation product, C16H18N2O5 (2)	Hypertension	0.136	95	0.006	0.869	0.039	TRUE
2,4-di-tert-butylphenol	Hypertension	0.036	23	0.950	0.808	0.034	TRUE
Bilirubin degradation product, C16H18N2O5 (1)	Hypertension	0.138	114	0.015	0.294	0.035	TRUE
3-hydroxyoctanoylcarnitine (2)	Hypertension	0.040	26	0.028	0.776	0.030	TRUE
Pantothenate	Hypertension	0.044	23	0.008	0.318	0.064	TRUE
5-oxoproline	Hypertension	0.131	108	0.021	0.128	0.059	TRUE
Phosphoethanolamine	Hypertension	0.045	25	6.92e-04	0.968	0.047	TRUE
Picolinate	Hypertension	0.062	32	4.04e-24	0.941	0.061	TRUE
3-ureidopropionate	Hypertension	0.031	26	0.330	0.852	0.029	TRUE
Erucate (22:1n9)	Hypertension	0.029	24	0.084	0.839	0.028	TRUE

Exposure	Outcome	R2	F	Heterogeneity	Pleiotropy	power	Direction
Eicosenedioate (C20:1-DC)	Hypertension	0.027	23	0.249	0.078	0.034	TRUE
Allantoin	Hypertension	0.036	23	0.126	0.020	0.034	TRUE
N-acetylvaline	Hypertension	0.036	23	2.67e-26	0.465	0.038	TRUE
N-acetylleucine	Hypertension	0.164	94	0.250	0.362	0.045	TRUE
N-acetylmethionine	Hypertension	0.032	27	6e-04	0.181	0.047	TRUE
Glycerate	Hypertension	0.048	24	0.808	0.274	0.029	TRUE
Sarcosine	Hypertension	0.041	23	0.005	0.815	0.038	TRUE
Trans-4-hydroxyproline	Hypertension	0.032	27	0.061	0.175	0.044	TRUE
Thyroxine	Hypertension	0.038	24	1.3e-06	0.289	0.117	TRUE
Pipecolate	Hypertension	0.039	23	0.553	0.787	0.026	TRUE
Gamma-glutamyltyrosine	Hypertension	0.044	30	0.012	0.288	0.038	TRUE
Methyl indole-3-acetate	Hypertension	0.022	23	0.399	0.205	0.035	TRUE
Ursodeoxycholate	Hypertension	0.038	24	2.55e-07	0.642	0.027	TRUE
3-hydroxy-3-methylglutarate	Hypertension	0.032	22	0.025	0.531	0.072	TRUE
Oleoyl ethanolamide	Hypertension	0.057	37	0.549	0.746	0.042	TRUE
4-hydroxyphenylpyruvate	Hypertension	0.028	26	0.027	0.415	0.027	TRUE
Gamma-glutamylglutamine	Hypertension	0.076	30	7.42e-04	0.856	0.052	TRUE

Exposure	Outcome	R2	F	Heterogeneity	Pleiotropy	power	Direction
Alpha-tocopherol	Hypertension	0.049	24	5.34e-04	0.900	0.055	TRUE
N-acetyl-L-alanine	Hypertension	0.051	25	1.02e-36	0.667	0.042	TRUE
Citrate	Hypertension	0.057	34	0.005	0.725	0.045	TRUE
2-aminobutyrate	Hypertension	0.040	37	0.763	0.598	0.044	TRUE
Butyrate/isobutyrate (4:0)	Hypertension	0.033	25	0.120	0.899	0.046	TRUE
Urate	Hypertension	0.094	40	0.032	0.169	0.053	TRUE
3-hydroxyisobutyrate	Hypertension	0.033	23	0.003	0.363	0.036	TRUE
Vanillylmandelate (VMA)	Hypertension	0.085	32	4.85e-39	0.442	0.224	TRUE
3-aminoisobutyrate	Hypertension	0.233	153	0.379	0.492	0.037	TRUE
4-acetamidobutanoate	Hypertension	0.069	53	0.058	0.203	0.054	TRUE
2-hydroxystearate	Hypertension	0.042	23	4.59e-04	0.660	0.043	TRUE
N1-methyladenosine	Hypertension	0.058	24	6.71e-27	0.461	0.124	TRUE
N-acetylneuraminate	Hypertension	0.061	27	2.93e-26	0.229	0.145	TRUE
Gamma-glutamylhistidine	Hypertension	0.032	25	7.15e-07	0.182	0.102	TRUE
N-acetylglucosaminylasparagine	Hypertension	0.122	65	2.42e-04	0.138	0.042	TRUE
Homovanillate (hva)	Hypertension	0.042	23	3.24e-11	0.620	0.038	TRUE
Acetoacetate	Hypertension	0.031	23	0.906	0.420	0.034	TRUE

Exposure	Outcome	R2	F	Heterogeneity	Pleiotropy	power	Direction
Cys-gly, oxidized	Hypertension	0.141	77	0.004	0.014	0.173	TRUE
Dihomo-linoleate (20:2n6)	Hypertension	0.027	22	5.91e-12	0.257	0.037	TRUE
Cholate	Hypertension	0.030	23	0.036	0.110	0.038	TRUE
Beta-hydroxyisovalerate	Hypertension	0.069	34	0.109	0.523	0.056	TRUE
Creatine	Hypertension	0.062	35	7.34e-08	0.386	0.131	TRUE
Anthranilate	Hypertension	0.033	23	0.901	0.658	0.035	TRUE
Glycerol	Hypertension	0.027	23	0.057	0.300	0.032	TRUE
Gamma-glutamylleucine	Hypertension	0.011	23	0.039	0.869	0.032	TRUE
Choline	Hypertension	0.045	27	1.53e-05	0.265	0.100	TRUE
3-methoxytyrosine	Hypertension	0.081	42	7.45e-04	0.326	0.036	TRUE
3-phosphoglycerate	Hypertension	0.036	23	0.005	0.074	0.029	TRUE
1-palmitoyl-2-linoleoyl-gpc (16:0/18:2)	Hypertension	0.078	44	0.127	0.965	0.031	TRUE
1-palmitoyl-2-oleoyl-GPE (16:0/18:1)	Hypertension	0.128	52	2.93e-04	0.925	0.052	TRUE
1-palmitoyl-2-linoleoyl-GPI (16:0/18:2)	Hypertension	0.078	38	9.76e-11	0.729	0.052	TRUE
Taurocholic acid	Hypertension	0.042	23	0.053	0.155	0.049	TRUE
Taurodeoxycholate	Hypertension	0.024	23	0.220	0.452	0.040	TRUE
Stearoyl sphingomyelin (d18:1/18:0)	Hypertension	0.091	34	2.06e-17	0.495	0.044	TRUE

Exposure	Outcome	R2	F	Heterogeneity	Pleiotropy	power	Direction
Ceramide (d18:1/16:0)	Hypertension	0.048	23	0.020	0.291	0.027	TRUE
Taurochenodeoxycholate	Hypertension	0.031	23	3.69e-04	0.249	0.032	TRUE
4-hydroxyphenylacetate	Hypertension	0.034	24	0.534	0.828	0.030	TRUE
5,6-dihydrothymine	Hypertension	0.023	22	1.64e-04	0.603	0.030	TRUE
1-palmitoyl-2-oleoyl-gpc (16:0/18:1)	Hypertension	0.043	24	0.053	0.250	0.026	TRUE
N-stearoyl-sphingosine (d18:1/18:0)	Hypertension	0.062	35	1.87e-05	0.570	0.040	TRUE
Glycochenodeoxycholate	Hypertension	0.039	23	6.97e-05	0.254	0.055	TRUE
N-formylmethionine	Hypertension	0.054	32	2.9e-25	0.373	0.170	TRUE
N6,n6,n6-trimethyllysine	Hypertension	0.046	38	0.937	0.597	0.032	TRUE
S-adenosylhomocysteine (SAH)	Hypertension	0.047	30	6e-42	0.625	0.105	TRUE
4-acetaminophen sulfate	Hypertension	0.032	22	0.198	0.617	0.029	TRUE
2-hydroxyhippurate (salicylurate)	Hypertension	0.027	23	0.803	0.505	0.026	TRUE
Quinolinate	Hypertension	0.045	29	7.58e-26	0.576	0.130	TRUE
Eicosapentaenoate (EPA; 20:5n3)	Hypertension	0.052	36	0.016	0.137	0.049	TRUE
Laurate (12:0)	Hypertension	0.024	22	0.582	0.331	0.055	TRUE
N-acetylputrescine	Hypertension	0.174	93	0.025	0.465	0.077	TRUE
Methylsuccinate	Hypertension	0.093	62	0.002	0.680	0.036	TRUE

Exposure	Outcome	R2	F	Heterogeneity	Pleiotropy	power	Direction
9,10-DiHOME	Hypertension	0.033	23	0.062	0.024	0.053	TRUE
Linoleate (18:2n6)	Hypertension	0.033	23	1.5e-11	0.473	0.040	TRUE
Adenosine 5'-diphosphate (ADP)	Hypertension	0.041	23	0.242	0.328	0.059	TRUE
Adenosine 5'-monophosphate (AMP)	Hypertension	0.021	22	0.622	0.757	0.036	TRUE
Adenosine 3',5'-cyclic monophosphate (camp)	Hypertension	0.055	27	0.411	0.999	0.034	TRUE
Arachidonate (20:4n6)	Hypertension	0.082	50	0.023	0.991	0.025	TRUE
5-methylthioadenosine (mta)	Hypertension	0.048	28	7.17e-05	0.998	0.060	TRUE
Cortisone	Hypertension	0.022	23	0.821	0.702	0.032	TRUE
Beta-alanine	Hypertension	0.044	36	0.010	0.434	0.034	TRUE
Succinate	Hypertension	0.023	22	0.203	0.228	0.082	TRUE
Phosphocholine	Hypertension	0.055	27	0.204	0.342	0.033	TRUE
Cysteinylglycine	Hypertension	0.063	44	9.98e-07	0.050	0.104	TRUE
Phenylpyruvate	Hypertension	0.057	28	0.261	0.413	0.055	TRUE
Creatinine	Hypertension	0.028	23	5.76e-27	0.696	0.145	TRUE
3-Hydroxybutyrate	Hypertension	0.025	23	0.161	0.747	0.030	TRUE
Cholesterol	Hypertension	0.036	23	0.468	0.038	0.036	TRUE
2-hydroxyphenylacetate	Hypertension	0.046	32	0.314	0.233	0.026	TRUE

Exposure	Outcome	R2	F	Heterogeneity	Pleiotropy	power	Direction
Aspartate	Hypertension	0.045	31	0.097	0.365	0.053	TRUE
3-(4-hydroxyphenyl)lactate	Hypertension	0.045	31	4.44e-10	0.063	0.100	TRUE
Arginine	Hypertension	0.067	29	1.79e-09	0.023	0.060	TRUE
Gluconate	Hypertension	0.052	40	0.308	0.977	0.032	TRUE
Sphinganine	Hypertension	0.040	25	0.001	0.921	0.028	TRUE
Gamma-glutamylglutamate	Hypertension	0.052	25	5.06e-08	0.736	0.046	TRUE
Flavin adenine dinucleotide (FAD)	Hypertension	0.104	46	3.22e-05	0.020	0.041	TRUE
Histidine	Hypertension	0.038	25	1.26e-07	0.287	0.038	TRUE
Cortisol	Hypertension	0.036	25	0.136	0.797	0.045	TRUE
Hypotaurine	Hypertension	0.074	27	9.69e-17	0.516	0.049	TRUE
Glutarate (C5-DC)	Hypertension	0.040	24	0.211	0.461	0.033	TRUE
S-1-pyrroline-5-carboxylate	Hypertension	0.028	26	0.049	0.670	0.031	TRUE
Cystine	Hypertension	0.026	22	0.718	0.731	0.035	TRUE
Cystathionine	Hypertension	0.045	23	0.087	0.154	0.032	TRUE
Guanidinoacetate	Hypertension	0.071	33	3.58e-16	0.241	0.058	TRUE
Sphingosine	Hypertension	0.026	31	0.004	0.194	0.038	TRUE
Deoxycholate	Hypertension	0.043	24	3.87e-08	0.106	0.029	TRUE

Exposure	Outcome	R2	F	Heterogeneity	Pleiotropy	power	Direction
Glycocholate	Hypertension	0.062	23	0.002	0.093	0.029	TRUE
Palmitoleate (16:1n7)	Hypertension	0.040	24	4.46e-12	0.511	0.060	TRUE
Leucine	Hypertension	0.038	23	0.006	0.151	0.091	TRUE
Stearate (18:0)	Hypertension	0.022	23	2.12e-18	0.208	0.053	TRUE
Methionine	Hypertension	0.034	24	0.685	0.299	0.052	TRUE
Orotate	Hypertension	0.216	102	0.256	0.625	0.025	TRUE
Phosphate	Hypertension	0.045	27	9.86e-04	0.306	0.054	TRUE
Inosine	Hypertension	0.027	22	0.429	0.111	0.042	TRUE
Inosine 5'-monophosphate (IMP)	Hypertension	0.035	23	0.429	0.068	0.029	TRUE
Phenylalanine	Hypertension	0.043	28	0.339	0.268	0.041	TRUE
Palmitate (16:0)	Hypertension	0.034	23	2.03e-10	0.063	0.030	TRUE
Malate	Hypertension	0.043	45	0.130	0.725	0.038	TRUE
Isoleucine	Hypertension	0.030	23	0.118	0.554	0.044	TRUE
Nicotinamide	Hypertension	0.033	23	0.005	0.657	0.043	TRUE
Myo-inositol	Hypertension	0.053	24	0.015	0.118	0.026	TRUE
Lysine	Hypertension	0.070	32	0.057	0.924	0.040	TRUE
Citrulline	Hypertension	0.044	26	1.12e-04	0.063	0.026	TRUE

Exposure	Outcome	R2	F	Heterogeneity	Pleiotropy	power	Direction
Ornithine	Hypertension	0.044	26	0.251	0.665	0.045	TRUE
Retinol (Vitamin A)	Hypertension	0.038	24	2.41e-07	0.220	0.049	TRUE
1-methylnicotinamide	Hypertension	0.042	23	0.009	0.567	0.035	TRUE
Uridine	Hypertension	0.032	38	0.368	0.067	0.048	TRUE
Plasma lactate	Hypertension	0.022	23	0.996	0.866	0.026	TRUE
Pristanate	Hypertension	0.017	23	0.213	0.500	0.026	TRUE
Phytanate	Hypertension	0.034	24	0.123	0.577	0.038	TRUE
Plasma free proline	Hypertension	0.095	38	0.206	0.940	0.031	TRUE
Glutamate	Hypertension	0.038	25	4.27e-05	0.291	0.042	TRUE
2'-deoxyuridine	Hypertension	0.082	43	1.62e-30	0.241	0.084	TRUE
Myristate (14:0)	Hypertension	0.030	23	1.36e-08	0.512	0.050	TRUE
Serine	Hypertension	0.100	38	2.79e-08	0.610	0.088	TRUE
Trans-urocanate	Hypertension	0.071	61	0.421	0.672	0.033	TRUE
Urea	Hypertension	0.033	23	0.007	0.898	0.029	TRUE
Pyridoxal	Hypertension	0.053	23	0.617	0.841	0.030	TRUE
Glutamine	Hypertension	0.064	39	0.098	0.530	0.099	TRUE
Taurine	Hypertension	0.017	24	0.019	0.942	0.026	TRUE

Exposure	Outcome	R2	F	Heterogeneity	Pleiotropy	power	Direction
Salicylate	Hypertension	0.039	23	0.741	0.432	0.033	TRUE
Serotonin	Hypertension	0.032	22	0.433	0.188	0.047	TRUE
Spermidine	Hypertension	0.040	24	7.19e-04	0.603	0.028	TRUE
Dimethylglycine	Hypertension	0.097	56	4.33e-05	0.486	0.030	TRUE
Betaine	Hypertension	0.099	55	1.14e-07	0.763	0.064	TRUE
Xylose	Hypertension	0.029	24	0.188	0.286	0.029	TRUE
Tyrosine	Hypertension	0.045	29	0.533	0.183	0.037	TRUE
Caprate (10:0)	Hypertension	0.031	24	0.810	0.557	0.046	TRUE
Cytidine	Hypertension	0.048	24	0.625	0.758	0.035	TRUE
Pyruvate	Hypertension	0.032	22	0.005	0.345	0.055	TRUE
Alanine	Hypertension	0.027	23	0.002	0.744	0.027	TRUE
Threonine	Hypertension	0.069	29	1.02e-11	0.210	0.043	TRUE
Cytosine	Hypertension	0.021	22	0.005	0.250	0.027	TRUE
12,13-DiHOME	Hypertension	0.041	23	0.710	0.828	0.036	TRUE
Mannose	Hypertension	0.062	48	0.040	0.405	0.044	TRUE
Caffeine	Hypertension	0.034	24	0.027	0.681	0.030	TRUE
Fructose	Hypertension	0.035	23	0.053	0.129	0.034	TRUE

Exposure	Outcome	R2	F	Heterogeneity	Pleiotropy	power	Direction
Alpha-ketobutyrate	Hypertension	0.019	22	0.004	0.326	0.048	TRUE
Cysteine	Hypertension	0.024	22	0.034	0.319	0.049	TRUE
Blood sugar	Hypertension	0.031	24	0.708	0.430	0.033	TRUE
Tryptophan	Hypertension	0.035	26	0.065	0.089	0.031	TRUE
Valine	Hypertension	0.022	23	0.752	0.400	0.054	TRUE
Pseudouridine	Hypertension	0.044	24	4.49e-39	0.481	0.088	TRUE
Dihydroorotate	Hypertension	0.115	46	4.49e-17	0.320	0.047	TRUE
Caprylate (8:0)	Hypertension	0.043	25	0.079	0.437	0.027	TRUE
Arachidate (20:0)	Hypertension	0.019	23	0.132	0.069	0.042	TRUE
Maltose	Hypertension	0.031	23	0.057	0.979	0.029	TRUE
N-stearoyl-sphinganine (d18:0/18:0)	Hypertension	0.016	27	0.439	0.232	0.027	TRUE
Sucrose	Hypertension	0.023	24	0.058	0.598	0.043	TRUE
Caproate (6:0)	Hypertension	0.051	31	0.965	0.849	0.049	TRUE
Nonadecanoate (19:0)	Hypertension	0.035	23	0.051	0.530	0.056	TRUE
Plasma free asparagine	Hypertension	0.135	92	1.82e-10	0.545	0.061	TRUE
X-07765	Hypertension	0.055	29	0.004	0.463	0.043	TRUE
Kynurenate	Hypertension	0.100	45	3.05e-29	0.154	0.075	TRUE

Exposure	Outcome	R2	F	Heterogeneity	Pleiotropy	power	Direction
Alpha-ketoglutarate	Hypertension	0.047	28	0.339	0.123	0.027	TRUE
X-11308	Hypertension	0.077	38	3.77e-05	0.202	0.027	TRUE
Pentadecanoate (15:0)	Hypertension	0.025	23	5.14e-11	0.277	0.026	TRUE
X-11315	Hypertension	0.074	41	0.990	1.000	0.035	TRUE
Margarate (17:0)	Hypertension	0.032	24	7.79e-12	0.689	0.037	TRUE
X-11372	Hypertension	0.048	28	0.085	0.637	0.028	TRUE
X-10458	Hypertension	0.049	32	0.534	0.059	0.027	TRUE
X-11299	Hypertension	0.034	26	0.240	0.182	0.048	TRUE
X-11478	Hypertension	0.059	31	4.66e-06	0.286	0.034	TRUE
X-11849	Hypertension	0.048	25	0.006	0.368	0.072	TRUE
X-11787	Hypertension	0.138	121	0.024	0.878	0.049	TRUE
X-11632	Hypertension	0.045	25	0.015	0.637	0.026	TRUE
X-11852	Hypertension	0.034	23	2.73e-04	0.640	0.028	TRUE
X-11847	Hypertension	0.033	23	0.384	0.535	0.038	TRUE
X-11483	Hypertension	0.036	23	0.175	0.473	0.039	TRUE
X-11470	Hypertension	0.129	49	0.128	0.685	0.050	TRUE
X-11381	Hypertension	0.080	39	0.007	0.486	0.095	TRUE

Exposure	Outcome	R2	F	Heterogeneity	Pleiotropy	power	Direction
X-11850	Hypertension	0.035	22	0.013	0.322	0.061	TRUE
X-11795	Hypertension	0.023	24	0.437	0.616	0.034	TRUE
X-11444	Hypertension	0.127	51	6.92e-05	0.116	0.056	TRUE
X-11858	Hypertension	0.031	23	0.242	0.681	0.050	TRUE
X-11843	Hypertension	0.042	23	0.278	0.826	0.033	TRUE
X-12127	Hypertension	0.041	24	1.76e-05	0.271	0.050	TRUE
X-12221	Hypertension	0.019	23	0.448	0.168	0.028	TRUE
X-12283	Hypertension	0.039	23	0.194	0.081	0.025	TRUE
X-12410	Hypertension	0.061	39	0.007	0.133	0.028	TRUE
X-12104	Hypertension	0.017	23	0.882	0.277	0.041	TRUE
X-12193	Hypertension	0.044	23	0.595	0.787	0.041	TRUE
X-12216	Hypertension	0.042	29	5.27e-11	0.314	0.066	TRUE
X-12306	Hypertension	0.036	23	0.081	0.951	0.032	TRUE
X-12100	Hypertension	0.049	37	7.77e-71	0.273	0.373	TRUE
X-12261	Hypertension	0.022	23	0.766	0.570	0.027	TRUE
X-11880	Hypertension	0.057	29	0.093	0.523	0.027	TRUE
X-12117	Hypertension	0.120	41	0.001	0.145	0.051	TRUE

Exposure	Outcome	R2	F	Heterogeneity	Pleiotropy	power	Direction
X-12407	Hypertension	0.019	22	0.345	0.110	0.049	TRUE
X-12101	Hypertension	0.049	32	5.12e-06	0.211	0.031	TRUE
X-12026	Hypertension	0.052	24	0.007	0.555	0.030	TRUE
X-12411	Hypertension	0.042	39	0.070	0.729	0.047	TRUE
X-12013	Hypertension	0.035	24	0.129	0.015	0.026	TRUE
X-12007	Hypertension	0.028	23	0.037	0.876	0.031	TRUE
X-12462	Hypertension	0.016	22	0.619	0.472	0.044	TRUE
X-12714	Hypertension	0.037	45	0.805	0.659	0.046	TRUE
X-12740	Hypertension	0.027	23	0.495	0.349	0.046	TRUE
X-12730	Hypertension	0.022	23	0.186	0.960	0.026	TRUE
X-12701	Hypertension	0.024	25	0.435	0.393	0.031	TRUE
X-12816	Hypertension	0.046	23	6.47e-10	0.240	0.028	TRUE
X-12731	Hypertension	0.049	24	0.656	0.500	0.057	TRUE
X-12707	Hypertension	0.063	35	0.016	0.402	0.036	TRUE
X-12729	Hypertension	0.039	23	0.374	0.496	0.027	TRUE
X-12830	Hypertension	0.042	23	0.278	0.026	0.034	TRUE
X-12818	Hypertension	0.022	23	0.330	0.606	0.034	TRUE

Exposure	Outcome	R2	F	Heterogeneity	Pleiotropy	power	Direction
X-12812	Hypertension	0.027	22	0.014	0.913	0.028	TRUE
X-12822	Hypertension	0.058	55	1.12e-04	0.206	0.134	TRUE
X-12738	Hypertension	0.022	23	0.889	0.753	0.028	TRUE
X-12544	Hypertension	0.040	24	0.565	0.341	0.034	TRUE
X-12680	Hypertension	0.023	24	0.081	0.633	0.034	TRUE
X-12906	Hypertension	0.024	23	0.982	0.733	0.039	TRUE
X-13684	Hypertension	0.112	45	0.530	0.211	0.025	TRUE
X-13507	Hypertension	0.017	23	0.140	0.117	0.030	TRUE
X-12851	Hypertension	0.029	27	9.86e-10	0.153	0.045	TRUE
X-12849	Hypertension	0.037	22	0.197	0.400	0.038	TRUE
X-12847	Hypertension	0.030	28	0.061	0.115	0.056	TRUE
X-13553	Hypertension	0.036	25	5.43e-09	0.376	0.070	TRUE
X-12839	Hypertension	0.051	25	8.36e-04	0.979	0.026	TRUE
X-13729	Hypertension	0.055	23	0.129	0.252	0.028	TRUE
X-13007	Hypertension	0.046	22	0.326	0.499	0.027	TRUE
X-13844	Hypertension	0.024	23	0.043	0.768	0.030	TRUE
X-13728	Hypertension	0.028	23	0.016	0.106	0.038	TRUE

Exposure	Outcome	R2	F	Heterogeneity	Pleiotropy	power	Direction
X-12844	Hypertension	0.100	38	0.004	0.924	0.043	TRUE
X-13695	Hypertension	0.025	23	0.557	0.937	0.032	TRUE
X-13723	Hypertension	0.019	23	0.321	0.996	0.034	TRUE
X-14056	Hypertension	0.013	22	0.598	0.688	0.053	TRUE
X-13866	Hypertension	0.044	24	8.91e-08	0.724	0.040	TRUE
X-17146	Hypertension	0.025	24	0.035	0.420	0.029	TRUE
X-16397	Hypertension	0.033	28	0.294	0.850	0.028	TRUE
X-17335	Hypertension	0.017	23	0.350	0.136	0.035	TRUE
X-16964	Hypertension	0.029	22	1.33e-04	0.662	0.051	TRUE
X-16935	Hypertension	0.101	72	2.14e-07	0.121	0.065	TRUE
X-15486	Hypertension	0.034	41	1.96e-04	0.083	0.065	TRUE
X-16580	Hypertension	0.029	24	0.803	0.422	0.031	TRUE
X-17325	Hypertension	0.042	32	5.31e-04	0.640	0.109	TRUE
X-17328	Hypertension	0.038	26	0.035	0.345	0.063	TRUE
X-16087	Hypertension	0.072	55	0.079	0.971	0.106	TRUE
X-17010	Hypertension	0.026	24	0.065	0.632	0.029	TRUE
X-14939	Hypertension	0.049	37	2.82e-06	0.410	0.029	TRUE

Exposure	Outcome	R2	F	Heterogeneity	Pleiotropy	power	Direction
X-15461	Hypertension	0.030	25	0.035	0.286	0.040	TRUE
X-15523	Hypertension	0.111	24	0.023	0.833	0.028	TRUE
X-15728	Hypertension	0.043	23	0.490	0.927	0.038	TRUE
X-15503	Hypertension	0.061	39	1.39e-43	0.105	0.107	TRUE
X-16124	Hypertension	0.042	23	0.094	0.432	0.038	TRUE
X-17438	Hypertension	0.028	23	0.925	0.954	0.039	TRUE
X-17354	Hypertension	0.016	22	0.351	0.271	0.035	TRUE
X-17654	Hypertension	0.126	82	0.082	0.988	0.041	TRUE
X-17676	Hypertension	0.071	30	0.024	0.150	0.026	TRUE
X-17367	Hypertension	0.060	31	1.14e-06	0.122	0.080	TRUE
X-17357	Hypertension	0.086	45	0.127	0.544	0.078	TRUE
X-18886	Hypertension	0.026	27	1.55e-05	0.391	0.055	TRUE
X-18887	Hypertension	0.019	23	0.731	0.480	0.035	TRUE
X-17653	Hypertension	0.077	43	2.44e-07	0.127	0.035	TRUE
X-17346	Hypertension	0.032	22	0.932	0.424	0.027	TRUE
X-17690	Hypertension	0.072	29	0.042	0.432	0.032	TRUE
X-18345	Hypertension	0.036	23	0.401	0.063	0.030	TRUE

Exposure	Outcome	R2	F	Heterogeneity	Pleiotropy	power	Direction
X-17685	Hypertension	0.029	24	0.437	0.184	0.048	TRUE
X-18888	Hypertension	0.036	27	0.432	0.852	0.061	TRUE
X-18901	Hypertension	0.040	24	0.630	0.861	0.027	TRUE
X-17351	Hypertension	0.037	23	0.056	0.531	0.030	TRUE
X-18779	Hypertension	0.014	23	0.001	0.254	0.106	TRUE
X-21286	Hypertension	0.059	29	4.54e-05	0.164	0.031	TRUE
X-21355	Hypertension	0.017	23	0.202	0.476	0.033	TRUE
X-21319	Hypertension	0.030	28	0.006	0.019	0.032	TRUE
X-21258	Hypertension	0.049	31	0.012	0.938	0.061	TRUE
X-21364	Hypertension	0.036	27	0.018	0.246	0.069	TRUE
X-21353	Hypertension	0.052	36	0.238	0.763	0.031	TRUE
X-19438	Hypertension	0.035	24	0.124	0.739	0.027	TRUE
X-18913	Hypertension	0.037	28	0.034	0.553	0.047	TRUE
X-18921	Hypertension	0.123	71	0.070	0.539	0.035	TRUE
X-21339	Hypertension	0.046	27	1.85e-11	0.247	0.034	TRUE
X-19299	Hypertension	0.030	23	0.063	0.357	0.030	TRUE
X-18935	Hypertension	0.047	23	0.391	0.648	0.029	TRUE

Exposure	Outcome	R2	F	Heterogeneity	Pleiotropy	power	Direction
X-21310	Hypertension	0.036	23	0.082	0.929	0.035	TRUE
X-21283	Hypertension	0.096	76	5.65e-29	0.310	0.075	TRUE
X-21285	Hypertension	0.059	27	0.314	0.899	0.032	TRUE
X-21312	Hypertension	0.039	27	0.091	0.204	0.028	TRUE
X-18922	Hypertension	0.094	44	0.502	0.300	0.047	TRUE
X-21442	Hypertension	0.028	26	0.004	0.150	0.053	TRUE
X-21796	Hypertension	0.056	31	1.36e-07	0.217	0.037	TRUE
X-21441	Hypertension	0.153	65	0.001	0.209	0.050	TRUE
X-21752	Hypertension	0.028	23	0.585	0.297	0.035	TRUE
X-21807	Hypertension	0.027	22	0.114	0.114	0.048	TRUE
X-21736	Hypertension	0.058	25	0.156	0.064	0.048	TRUE
X-21829	Hypertension	0.060	30	0.103	0.710	0.032	TRUE
X-21471	Hypertension	0.155	73	0.008	0.559	0.041	TRUE
X-21733	Hypertension	0.060	25	6.04e-05	0.914	0.036	TRUE
X-21742	Hypertension	0.020	23	5.05e-05	0.959	0.026	TRUE
X-21821	Hypertension	0.023	24	0.486	0.355	0.032	TRUE
X-21470	Hypertension	0.086	56	0.006	0.230	0.028	TRUE

Exposure	Outcome	R2	F	Heterogeneity	Pleiotropy	power	Direction
X-21383	Hypertension	0.036	23	0.405	0.359	0.029	TRUE
X-21607	Hypertension	0.130	58	7.71e-07	0.013	0.096	TRUE
X-21834	Hypertension	0.037	26	0.292	0.104	0.035	TRUE
X-21467	Hypertension	0.148	63	8.82e-08	0.323	0.079	TRUE
X-22776	Hypertension	0.028	24	0.363	0.149	0.030	TRUE
X-23655	Hypertension	0.035	22	3.79e-05	0.624	0.050	TRUE
X-22834	Hypertension	0.030	23	0.148	0.060	0.061	TRUE
X-22771	Hypertension	0.019	22	0.307	0.530	0.032	TRUE
X-23639	Hypertension	0.044	28	5.97e-08	0.613	0.052	TRUE
X-23648	Hypertension	0.028	23	9.5e-11	0.476	0.061	TRUE
X-23654	Hypertension	0.101	40	0.318	0.570	0.031	TRUE
X-22520	Hypertension	0.036	30	0.109	0.208	0.032	TRUE
X-23641	Hypertension	0.089	34	0.005	0.426	0.034	TRUE
X-23276	Hypertension	0.036	23	0.153	0.324	0.041	TRUE
X-21845	Hypertension	0.035	23	0.387	0.143	0.039	TRUE
X-22509	Hypertension	0.027	22	0.421	0.822	0.027	TRUE
X-23644	Hypertension	0.028	29	2.51e-04	0.482	0.025	TRUE

Exposure	Outcome	R2	F	Heterogeneity	Pleiotropy	power	Direction
X-22162	Hypertension	0.174	76	4.53e-27	0.089	0.032	TRUE
X-23587	Hypertension	0.022	23	0.418	0.354	0.043	TRUE
X-23593	Hypertension	0.032	22	0.005	0.858	0.055	TRUE
X-23636	Hypertension	0.020	23	0.225	0.630	0.025	TRUE
X-23678	Hypertension	0.042	23	0.001	0.724	0.037	TRUE
X-23680	Hypertension	0.073	52	4.05e-06	0.472	0.035	TRUE
X-23780	Hypertension	0.062	33	0.844	0.904	0.031	TRUE
X-23974	Hypertension	0.032	26	0.482	0.481	0.039	TRUE
X-23659	Hypertension	0.069	36	0.033	0.162	0.059	TRUE
X-23665	Hypertension	0.019	23	0.146	0.823	0.047	TRUE
X-23782	Hypertension	0.034	24	0.175	0.400	0.029	TRUE
X-24295	Hypertension	0.036	23	5.68e-05	0.039	0.025	TRUE
X-24243	Hypertension	0.044	31	3.62e-04	0.117	0.031	TRUE
X-23739	Hypertension	0.074	45	7.91e-04	0.612	0.059	TRUE
X-23997	Hypertension	0.029	24	0.029	0.011	0.025	TRUE
X-24241	Hypertension	0.044	33	0.007	0.869	0.098	TRUE
X-24328	Hypertension	0.081	29	3.8e-05	0.339	0.044	TRUE

Exposure	Outcome	R2	F	Heterogeneity	Pleiotropy	power	Direction
X-24456	Hypertension	0.047	23	0.401	0.389	0.027	TRUE
X-24418	Hypertension	0.125	59	1.01e-07	0.674	0.029	TRUE
X-24344	Hypertension	0.027	23	0.035	0.522	0.043	TRUE
X-24556	Hypertension	0.066	42	2.76e-05	0.031	0.026	TRUE
X-24337	Hypertension	0.082	43	2.48e-09	0.340	0.048	TRUE
X-24546	Hypertension	0.096	45	0.456	0.856	0.044	TRUE
X-24306	Hypertension	0.036	23	0.037	0.122	0.035	TRUE
X-24307	Hypertension	0.023	24	0.083	0.808	0.027	TRUE
X-24334	Hypertension	0.046	29	6.82e-08	0.942	0.044	TRUE
X-24518	Hypertension	0.141	81	0.625	0.214	0.059	TRUE
X-24494	Hypertension	0.057	44	0.223	0.157	0.025	TRUE
X-24544	Hypertension	0.181	78	2.97e-14	0.293	0.049	TRUE
X-24531	Hypertension	0.049	32	7.22e-06	0.025	0.029	TRUE
X-24811	Hypertension	0.038	23	1.32e-07	0.145	0.033	TRUE
X-24757	Hypertension	0.035	24	3.99e-07	0.946	0.034	TRUE
X-24978	Hypertension	0.036	25	1.41e-06	0.969	0.036	TRUE
X-24970	Hypertension	0.037	22	0.452	0.864	0.026	TRUE

Exposure	Outcome	R2	F	Heterogeneity	Pleiotropy	power	Direction
X-24795	Hypertension	0.043	26	1.42e-06	0.281	0.051	TRUE
X-24951	Hypertension	0.031	23	0.920	0.248	0.032	TRUE
X-24728	Hypertension	0.044	23	6.27e-04	0.102	0.035	TRUE
X-24565	Hypertension	0.048	23	0.065	0.943	0.030	TRUE
X-24801	Hypertension	0.097	34	1.7e-04	0.595	0.043	TRUE
X-24736	Hypertension	0.094	40	0.052	0.015	0.057	TRUE
X-24585	Hypertension	0.019	27	0.439	0.647	0.046	TRUE
X-24588	Hypertension	0.136	48	4.98e-06	0.431	0.033	TRUE
X-25109	Hypertension	0.053	31	0.031	0.792	0.034	TRUE
X-24947	Hypertension	0.208	97	0.044	0.813	0.078	TRUE
X-24949	Hypertension	0.091	45	4.69e-05	0.061	0.031	TRUE
X-24812	Hypertension	0.035	24	3.7e-06	0.102	0.096	TRUE
X-25217	Hypertension	0.027	23	0.043	0.007	0.036	TRUE
X-25828	Hypertension	0.013	22	0.323	0.624	0.040	TRUE
X-25371	Hypertension	0.098	41	5.39e-07	0.281	0.065	TRUE
X-25419	Hypertension	0.049	26	0.038	0.960	0.028	TRUE
X-25265	Hypertension	0.039	23	2.41e-08	0.467	0.041	TRUE

Exposure	Outcome	R2	F	Heterogeneity	Pleiotropy	power	Direction
X-25172	Hypertension	0.024	22	0.205	0.061	0.034	TRUE
X-25957	Hypertension	0.023	24	0.150	0.030	0.030	TRUE
X-25422	Hypertension	0.092	48	2.32e-06	0.159	0.056	TRUE
X-25790	Hypertension	0.032	27	8.01e-04	0.096	0.038	TRUE
X-25420	Hypertension	0.037	31	3.07e-09	0.153	0.077	TRUE
X-25810	Hypertension	0.064	25	0.157	0.836	0.025	TRUE
X-25433	Hypertension	0.072	40	7.38e-06	0.530	0.056	TRUE
X-25343	Hypertension	0.022	23	0.710	0.410	0.056	TRUE
X-25271	Hypertension	0.034	24	0.357	0.870	0.037	TRUE
X-25520	Hypertension	0.021	22	0.757	0.832	0.034	TRUE
X-25519	Hypertension	0.025	23	1.56e-08	0.497	0.071	TRUE
X-26111	Hypertension	0.067	40	0.001	0.891	0.047	TRUE
X-26109	Hypertension	0.180	93	2.2e-04	0.008	0.038	TRUE
X-26054	Hypertension	0.167	66	8.38e-16	0.539	0.097	TRUE
Carnitine C4	Hypertension	0.246	92	6.82e-12	0.026	0.065	TRUE
Androsterone sulfate	Hypertension	0.238	90	5.6e-11	0.680	0.089	TRUE
N-acetyltyrosine	Hypertension	0.267	123	8.25e-04	0.780	0.050	TRUE

Exposure	Outcome	R2	F	Heterogeneity	Pleiotropy	power	Direction
N-acetyl-L-glutamine	Hypertension	0.350	310	0.005	0.150	0.091	TRUE
N-acetylphenylalanine	Hypertension	0.212	167	0.149	0.891	0.077	TRUE
N-acetylasparagine	Hypertension	0.448	486	0.003	0.095	0.082	TRUE
N-acetylarginine	Hypertension	0.410	338	1.32e-04	0.021	0.108	TRUE
5-acetylamino-6-formylamino-3-methyluracil	Hypertension	0.275	176	2.63e-07	0.025	0.041	TRUE
Orotidine	Hypertension	0.193	100	1.16e-38	0.591	0.027	TRUE
3-methylcytidine	Hypertension	0.277	332	0.017	0.356	0.041	TRUE
N-acetylcitrulline	Hypertension	0.368	369	8.41e-05	0.010	0.090	TRUE
N-acetyl-aspartyl-glutamate (naag)	Hypertension	0.192	193	3.07e-10	0.693	0.034	TRUE
1-stearoyl-2-arachidonoyl-gpc (18:0/20:4)	Hypertension	0.211	109	2.66e-04	0.881	0.047	TRUE
Bilirubin (E,E)	Hypertension	0.169	103	0.007	0.370	0.040	TRUE
Bilirubin (E,Z or Z,E)	Hypertension	0.154	106	0.098	0.570	0.043	TRUE
Androsterone glucuronide	Hypertension	0.246	110	4.61e-08	0.577	0.039	TRUE
N6-methyllysine	Hypertension	0.703	903	0.197	0.550	0.044	TRUE
N-acetyl-1-methylhistidine	Hypertension	0.282	114	2.22e-05	0.377	0.039	TRUE
N-delta-acetylornithine	Hypertension	0.341	303	0.416	0.626	0.067	TRUE
1-palmitoyl-2-arachidonoyl-gpc (16:0/20:4n6)	Hypertension	0.195	120	2.19e-04	0.660	0.043	TRUE

Exposure	Outcome	R2	F	Heterogeneity	Pleiotropy	power	Direction
Glycochenodeoxycholate glucuronide (1)	Hypertension	0.294	223	0.063	0.301	0.056	TRUE
2'-o-methylcytidine	Hypertension	0.216	194	0.099	0.694	0.050	TRUE
2'-o-methyluridine	Hypertension	0.238	201	0.401	0.122	0.037	TRUE
N6,N6-dimethyllysine	Hypertension	0.528	502	0.079	0.625	0.044	TRUE
N2-acetyl,N6,N6-dimethyllysine	Hypertension	0.515	333	0.017	0.886	0.038	TRUE
N2-acetyl,N6-methyllysine	Hypertension	0.466	265	0.062	0.385	0.044	TRUE
Deoxycholic acid glucuronide	Hypertension	0.237	133	0.098	0.812	0.073	TRUE
Decadienedioic acid (C10:2-DC)	Hypertension	0.203	115	2.04e-05	0.137	0.051	TRUE
Bilirubin degradation product, C17H18N2O4 (1)	Hypertension	0.177	118	6.06e-17	0.922	0.043	TRUE
Bilirubin degradation product, C17H18N2O4 (2)	Hypertension	0.162	112	6.04e-15	0.786	0.037	TRUE
Bilirubin degradation product, C17H18N2O4 (3)	Hypertension	0.154	118	3.21e-13	0.624	0.067	TRUE
Bilirubin (z,z)	Hypertension	0.223	121	0.180	0.554	0.049	TRUE
Ethylmalonate	Hypertension	0.224	94	0.265	0.241	0.035	TRUE
Biliverdin	Hypertension	0.191	105	0.215	0.320	0.071	TRUE
Glycine	Hypertension	0.179	129	0.047	0.836	0.206	TRUE
X-12112	Hypertension	0.523	302	0.218	0.134	0.047	TRUE
X-12798	Hypertension	0.181	89	0.004	0.582	0.065	TRUE

Exposure	Outcome	R2	F	Heterogeneity	Pleiotropy	power	Direction
X-13431	Hypertension	0.068	36	0.045	0.452	0.038	TRUE
X-19141	Hypertension	0.223	124	2.68e-07	0.588	0.032	TRUE
Glutamine	Hypertension	0.030	31	0.188	0.463	1.000	TRUE
Tryptophan	Hypertension	0.383	26	4.69e-12	0.910	0.998	TRUE
Histidine	Hypertension	0.020	30	1.35e-06	0.333	0.999	TRUE
Leucine	Hypertension	0.423	26	1.22e-16	0.803	0.737	TRUE
Cholesterol	Hypertension	0.098	24	1.92e-13	0.953	1.000	TRUE
Phenylalanine	Hypertension	0.015	28	0.107	0.234	0.530	TRUE
Creatinine	Hypertension	0.047	25	0.384	0.943	0.083	TRUE
Lactate	Hypertension	0.016	24	0.001	0.542	0.032	TRUE
3-hydroxybutyrate (BHBA)	Hypertension	0.016	23	0.453	0.745	0.027	TRUE
Arabinose	Hypertension	0.016	23	0.017	0.372	0.039	TRUE
Fructose	Hypertension	0.006	24	0.701		0.066	TRUE
Mannose	Hypertension	0.065	61	0.002	0.028	0.417	TRUE
Pyruvate	Hypertension	0.034	22	0.003	0.473	0.076	TRUE
Uridine	Hypertension	0.056	28	0.035	0.809	0.997	TRUE
Linoleate (18:2n6)	Hypertension	0.029	24	0.435	0.856	0.082	TRUE

Exposure	Outcome	R2	F	Heterogeneity	Pleiotropy	power	Direction
Allantoin	Hypertension	0.039	23	0.425	0.324	0.076	TRUE
Arachidonate (20:4n6)	Hypertension	0.081	56	0.006	0.254	0.029	TRUE
Deoxycholate	Hypertension	0.028	23	0.289	0.792	0.085	TRUE
Margarate (17:0)	Hypertension	0.024	29	3.03e-06	0.660	0.210	TRUE
Inosine	Hypertension	0.053	28	0.716	0.426	0.034	TRUE
Isoleucine	Hypertension	0.034	23	0.002	0.597	0.077	TRUE
Threonine	Hypertension	0.051	22	1.15e-08	0.421	0.546	TRUE
Tyrosine	Hypertension	0.073	26	0.106	0.866	0.132	TRUE
Lysine	Hypertension	0.053	26	0.469	0.694	0.425	TRUE
Methionine	Hypertension	0.040	22	0.482	0.410	0.051	TRUE
Malate	Hypertension	0.036	25	0.694	0.759	0.577	TRUE
Palmitate (16:0)	Hypertension	0.061	24	9.92e-07	0.753	0.655	TRUE
Nonadecanoate (19:0)	Hypertension	0.023	24	7.29e-04	0.679	0.931	TRUE
Stearate (18:0)	Hypertension	0.090	26	3.64e-05	0.412	0.065	TRUE
Oleate (18:1n9)	Hypertension	0.036	24	0.259	0.519	0.593	TRUE
Pentadecanoate (15:0)	Hypertension	0.033	23	1.48e-12	0.445	0.511	TRUE
Myristate (14:0)	Hypertension	0.037	23	6.81e-08	0.594	0.903	TRUE

Exposure	Outcome	R2	F	Heterogeneity	Pleiotropy	power	Direction
Pipecolate	Hypertension	0.025	23	0.004	0.037	0.228	TRUE
Ornithine	Hypertension	0.019	24	0.381	0.879	0.241	TRUE
5-oxoproline	Hypertension	0.109	52	7.82e-04	0.488	1.000	TRUE
Pantothenate	Hypertension	0.046	24	5.66e-09	0.504	0.027	TRUE
4-acetamidobutanoate	Hypertension	0.093	39	2.12e-07	0.571	0.574	TRUE
Alpha-tocopherol	Hypertension	0.016	23	0.169	0.616	0.085	TRUE
Citrate	Hypertension	0.074	27	0.290	0.758	0.580	TRUE
Glycerate	Hypertension	0.028	23	0.077	0.557	0.061	TRUE
Guanosine	Hypertension	0.076	22	0.848	0.879	0.043	TRUE
N-acetylalanine	Hypertension	0.039	24	0.003	0.140	0.933	TRUE
Urate	Hypertension	0.079	67	0.066	0.018	0.117	TRUE
Ursodeoxycholate	Hypertension	0.018	23	0.004	0.128	0.032	TRUE
Arginine	Hypertension	0.036	23	5.96e-06	0.603	0.405	TRUE
Ascorbate (Vitamin C)	Hypertension	0.111	23	0.084	0.546	0.054	TRUE
Heptanoate (7:0)	Hypertension	0.061	24	0.015	0.497	0.372	TRUE
Laurate (12:0)	Hypertension	0.087	24	5.15e-04	0.878	0.099	TRUE
Valine	Hypertension	0.022	27	0.018	0.114	0.297	TRUE

Exposure	Outcome	R2	F	Heterogeneity	Pleiotropy	power	Direction
Urea	Hypertension	0.024	22	0.035	0.490	0.138	TRUE
Cortisol	Hypertension	0.016	24	0.256	0.762	0.043	TRUE
Cortisone	Hypertension	0.081	24	0.046	0.414	0.195	TRUE
Proline	Hypertension	0.126	41	0.032	0.451	0.029	TRUE
Citrulline	Hypertension	0.078	25	5.19e-04	0.617	0.091	TRUE
Biliverdin	Hypertension	0.151	85	1.12e-04	0.679	0.091	TRUE
Serotonin (5HT)	Hypertension	0.043	23	8.72e-04	0.328	0.460	TRUE
Gamma-glutamylglutamine	Hypertension	0.061	28	1.72e-06	0.756	0.545	TRUE
Gamma-glutamyltyrosine	Hypertension	0.085	26	1.06e-08	0.947	0.805	TRUE
Hypoxanthine	Hypertension	0.049	26	0.041	0.814	0.033	TRUE
Betaine	Hypertension	0.065	34	1.1e-06	0.514	0.999	TRUE
Xanthine	Hypertension	0.011	24	0.578	0.574	0.403	TRUE
Phosphate	Hypertension	0.007	24	0.691		0.065	TRUE
3-methoxytyrosine	Hypertension	0.037	24	0.005	0.956	0.188	TRUE
Pelargonate (9:0)	Hypertension	0.064	23	0.035	0.450	0.085	TRUE
Undecanoate (11:0)	Hypertension	0.073	25	0.026	0.671	0.993	TRUE
Beta-hydroxyisovalerate	Hypertension	0.060	24	0.002	0.196	0.948	TRUE

Exposure	Outcome	R2	F	Heterogeneity	Pleiotropy	power	Direction
Taurodeoxycholate	Hypertension	0.116	23	0.128	0.233	0.127	TRUE
X-02973	Hypertension	0.049	23	0.008	0.663	0.031	TRUE
X-03003	Hypertension	0.009	21	0.025	0.239	0.309	TRUE
X-03088	Hypertension	0.022	22	0.019	0.643	0.119	TRUE
X-03094	Hypertension	0.072	31	9.87e-04	0.205	0.186	TRUE
Glycerol	Hypertension	0.039	24	0.120	0.873	0.671	TRUE
Kynurenine	Hypertension	0.103	29	1.8e-50	0.768	1.000	TRUE
Mannitol	Hypertension	0.024	22	3.53e-04	0.524	0.031	TRUE
Glycerol 3-phosphate (G3P)	Hypertension	0.018	23	0.277	0.657	0.123	TRUE
Acetylphosphate	Hypertension	0.037	25	0.001	0.476	0.999	TRUE
Carnitine	Hypertension	0.498	29	7.02e-20	0.604	1.000	TRUE
Choline	Hypertension	0.050	24	1.83e-08	0.256	0.812	TRUE
N-acetylornithine	Hypertension	0.183	151	0.090	0.811	0.559	TRUE
N1-methyladenosine	Hypertension	0.010	25	0.234	0.998	0.283	TRUE
3-methyl-2-oxovalerate	Hypertension	0.076	27	0.046	0.323	0.103	TRUE
3-methylhistidine	Hypertension	0.016	22	1.68e-05	0.483	0.109	TRUE
3-phenylpropionate (hydrocinnamate)	Hypertension	0.020	29	0.316	0.969	0.140	TRUE

Exposure	Outcome	R2	F	Heterogeneity	Pleiotropy	power	Direction
Phenylacetate	Hypertension	0.019	22	0.326	0.830	0.380	TRUE
Glycerophosphorylcholine (GPC)	Hypertension	0.027	23	0.003	0.739	0.108	TRUE
Aspartate	Hypertension	0.009	23	0.639	0.777	0.088	TRUE
X-04357	Hypertension	0.006	22	0.299		0.066	TRUE
X-04494	Hypertension	0.037	22	0.229	0.581	0.669	TRUE
X-04495	Hypertension	0.023	23	0.405	0.649	0.035	TRUE
X-04498	Hypertension	0.048	23	0.022	0.919	0.162	TRUE
X-04499--3,4-dihydroxybutyrate	Hypertension	0.046	25	0.250	0.529	0.167	TRUE
X-04500	Hypertension	0.033	23	0.235	0.633	0.098	TRUE
Dihomo-linoleate (20:2n6)	Hypertension	0.009	23	0.015	0.253	0.059	TRUE
X-18601	Hypertension	0.049	28	3.46e-13	0.374	0.025	TRUE
2-hydroxystearate	Hypertension	0.070	23	0.019	0.311	0.091	TRUE
X-05426	Hypertension	0.027	23	0.001	0.926	0.426	TRUE
Indolelactate	Hypertension	0.039	25	4.87e-14	0.957	0.150	TRUE
Glycylvaline	Hypertension	0.033	23	0.127	0.539	0.075	TRUE
Gamma-glutamylleucine	Hypertension	0.054	23	6.8e-05	0.352	0.868	TRUE
Eicosapentaenoate (EPA; 20:5n3)	Hypertension	0.030	32	0.324	0.239	0.028	TRUE

Exposure	Outcome	R2	F	Heterogeneity	Pleiotropy	power	Direction
Estrone 3-sulfate	Hypertension	0.205	24	0.727	0.233	0.091	TRUE
Glycocholate	Hypertension	0.024	22	0.302	0.623	0.067	TRUE
Glycodeoxycholate	Hypertension	0.077	23	2.79e-11	0.153	0.203	TRUE
Taurochenodeoxycholate	Hypertension	0.025	23	0.612	0.729	0.054	TRUE
Taurocholate	Hypertension	0.065	23	0.478	0.125	0.049	TRUE
X-05907	Hypertension	0.030	24	0.983	0.855	0.165	TRUE
Docosahexaenoate (DHA; 22:6n3)	Hypertension	0.003	22			0.480	TRUE
1-stearoylglycerophosphoinositol	Hypertension	0.013	24	0.992	0.863	0.026	TRUE
X-06226	Hypertension	0.048	23	0.554	0.936	0.782	TRUE
X-06246	Hypertension	0.046	24	0.007	0.832	0.661	TRUE
X-06267	Hypertension	0.024	23	0.107	0.710	0.256	TRUE
X-06307	Hypertension	0.019	25	0.035	0.485	0.182	TRUE
X-06350	Hypertension	0.033	23	0.800	0.380	0.043	TRUE
X-06351	Hypertension	0.020	23	0.325	0.252	0.081	TRUE
Myo-inositol	Hypertension	0.093	26	6.46e-06	0.461	0.594	TRUE
Glucose	Hypertension	0.083	24	1.48e-05	0.889	0.767	TRUE
1,5-anhydroglucitol (1,5-AG)	Hypertension	0.080	37	7.71e-04	0.625	0.103	TRUE

Exposure	Outcome	R2	F	Heterogeneity	Pleiotropy	power	Direction
2-hydroxybutyrate (AHB)	Hypertension	0.030	25	0.002	0.268	0.063	TRUE
3-methyl-2-oxobutyrate	Hypertension	0.035	23	0.518	0.652	0.042	TRUE
1,6-anhydroglucose	Hypertension	0.030	26	0.348	0.586	0.113	TRUE
1-palmitoylglycerol (1-monopalmitin)	Hypertension	0.026	23	7.91e-04	0.136	0.026	TRUE
1-oleoylglycerol (1-monoolein)	Hypertension	0.045	24	4.31e-04	0.628	0.037	TRUE
1-stearoylglycerol (1-monostearin)	Hypertension	0.057	24	2.27e-04	0.159	0.028	TRUE
X-08402	Hypertension	0.093	58	0.077	0.855	0.997	TRUE
2-hydroxyisobutyrate	Hypertension	0.055	28	4.97e-06	0.922	0.136	TRUE
X-08766	Hypertension	0.042	22	0.159	0.910	0.420	TRUE
4-methyl-2-oxopentanoate	Hypertension	0.026	27	0.934	0.986	0.584	TRUE
Phenyllactate (PLA)	Hypertension	0.059	28	0.002	0.642	0.456	TRUE
Homocitrulline	Hypertension	0.034	34	0.214	0.787	0.029	TRUE
Aspartylphenylalanine	Hypertension	0.034	44	5.11e-05	0.995	1.000	TRUE
Levulinate (4-oxovalerate)	Hypertension	0.097	25	1.58e-07	0.258	0.990	TRUE
Palmitoylcarnitine	Hypertension	0.018	26	0.035	0.831	0.933	TRUE
X-08988	Hypertension	0.087	29	0.021	0.354	1.000	TRUE
X-09026	Hypertension	0.037	24	0.035	0.872	0.656	TRUE

Exposure	Outcome	R2	F	Heterogeneity	Pleiotropy	power	Direction
X-09108	Hypertension	0.021	23	0.108	0.655	0.939	TRUE
Cholate	Hypertension	0.008	22	0.800		0.078	TRUE
X-09706	Hypertension	0.038	23	6.51e-04	0.621	0.038	TRUE
X-10395	Hypertension	0.076	27	0.422	0.517	0.140	TRUE
X-10429	Hypertension	0.058	25	1.32e-04	0.001	0.673	TRUE
X-10500	Hypertension	0.075	23	1.58e-07	0.840	0.909	TRUE
X-10506	Hypertension	0.019	23	0.589	0.530	0.032	TRUE
X-10510	Hypertension	0.070	32	3.1e-09	0.308	0.748	TRUE
1-linoleoylglycerol (1-monolinolein)	Hypertension	0.048	27	0.092	0.809	0.341	TRUE
Indoleacetate	Hypertension	0.041	25	0.161	0.210	0.039	TRUE
Hyodeoxycholate	Hypertension	0.043	24	0.054	0.322	0.102	TRUE
3-indoxyl sulfate	Hypertension	0.006	24	0.035		0.108	TRUE
N-acetylglycine	Hypertension	0.059	58	0.013	0.605	0.660	TRUE
Bilirubin (Z,Z)	Hypertension	0.114	132	0.022	0.287	0.034	TRUE
Creatine	Hypertension	0.026	39	0.146	0.766	0.995	TRUE
Erythrose	Hypertension	0.010	22	0.077	0.313	0.025	TRUE
Threonate	Hypertension	0.027	22	0.322	0.533	0.026	TRUE

Exposure	Outcome	R2	F	Heterogeneity	Pleiotropy	power	Direction
X-10810	Hypertension	0.042	24	0.012	0.804	0.045	TRUE
Cysteine	Hypertension	0.033	22	6e-06	0.379	0.090	TRUE
Pyroglutamylglycine	Hypertension	0.028	22	0.045		0.089	TRUE
N-(2-furoyl)glycine	Hypertension	0.729	23	0.006	0.863	0.108	TRUE
DSGEGDFXAEGGGVR*	Hypertension	0.033	21	0.015	0.093	0.052	TRUE
Pyridoxate	Hypertension	0.018	22	0.046	0.264	0.119	TRUE
Androsterone sulfate	Hypertension	0.118	60	7.67e-04	0.857	0.219	TRUE
3-carboxy-4-methyl-5-propyl-2-furanpropanoate (CMPF)	Hypertension	0.033	25	0.077	0.254	0.057	TRUE
3-(4-hydroxyphenyl)lactate	Hypertension	0.044	25	0.177	0.804	0.416	TRUE
Acetylcarnitine	Hypertension	0.039	36	0.011	0.718	0.994	TRUE
Serine	Hypertension	0.084	35	6.7e-07	0.311	0.969	TRUE
Trans-4-hydroxyproline	Hypertension	0.006	21	0.762		0.069	TRUE
Glutamate	Hypertension	0.008	28	0.158		0.052	TRUE
Hexanoylcarnitine	Hypertension	0.098	68	0.011	0.598	0.826	TRUE
Glycine	Hypertension	0.121	79	0.016	0.184	1.000	TRUE
Alanine	Hypertension	0.078	25	0.012	0.789	0.552	TRUE
Glycochenodeoxycholate	Hypertension	0.032	24	2.23e-04	0.393	0.038	TRUE

Exposure	Outcome	R2	F	Heterogeneity	Pleiotropy	power	Direction
2-aminobutyrate	Hypertension	0.105	26	0.089	0.746	0.955	TRUE
Scyllo-inositol	Hypertension	0.036	32	0.096	0.181	0.133	TRUE
Dodecanedioate	Hypertension	0.024	24	0.053	0.169	0.123	TRUE
Gamma-glutamylvaline	Hypertension	0.026	24	6.39e-05	0.888	0.837	TRUE
Indolepropionate	Hypertension	0.038	28	0.123	0.737	0.083	TRUE
Butyrylcarnitine	Hypertension	0.212	106	1.25e-06	0.171	0.240	TRUE
Myristoleate (14:1n5)	Hypertension	0.036	27	1.12e-04	0.417	0.295	TRUE
Dehydroisoandrosterone sulfate (DHEA-S)	Hypertension	0.045	28	2.47e-08	0.981	0.657	TRUE
Propionylcarnitine	Hypertension	0.107	33	1.68e-07	0.627	0.076	TRUE
Caproate (6:0)	Hypertension	0.053	23	2.95e-06	0.609	0.226	TRUE
Caprylate (8:0)	Hypertension	0.094	23	1.16e-05	0.307	0.096	TRUE
10-undecenoate (11:1n1)	Hypertension	0.072	45	0.004	0.360	0.110	TRUE
Docosapentaenoate (n3 DPA; 22:5n3)	Hypertension	0.017	32	0.698	0.761	0.043	TRUE
X-11204	Hypertension	0.077	24	4.25e-31	0.309	1.000	TRUE
X-02269	Hypertension	0.033	30	1.04e-04	0.403	0.056	TRUE
Phenol sulfate	Hypertension	0.027	22	2.55e-04	0.789	0.056	TRUE
X-06126	Hypertension	0.030	22	0.004	0.940	0.044	TRUE

Exposure	Outcome	R2	F	Heterogeneity	Pleiotropy	power	Direction
X-07765	Hypertension	0.095	22	0.002	0.836	0.065	TRUE
X-11247	Hypertension	0.039	25	0.408	0.183	0.130	TRUE
X-11261	Hypertension	0.038	40	8.34e-06	0.494	0.373	TRUE
Bilirubin (E,E)*	Hypertension	0.077	74	0.665	0.583	0.105	TRUE
X-02249	Hypertension	0.028	30	0.051	0.757	0.277	TRUE
Heme*	Hypertension	0.019	23	0.403	0.275	0.136	TRUE
X-11299	Hypertension	0.019	22	0.084	0.847	0.050	TRUE
X-11315	Hypertension	0.061	26	0.001	0.580	0.030	TRUE
X-11317	Hypertension	0.095	25	2.28e-05	0.036	0.233	TRUE
1-linoleoylglycerophosphoethanolamine*	Hypertension	0.045	42	0.012	0.785	0.098	TRUE
X-11327	Hypertension	0.082	25	0.009	0.074	0.258	TRUE
X-11334	Hypertension	0.064	24	4.94e-05	0.827	0.029	TRUE
3-dehydrocarnitine*	Hypertension	0.065	30	7.16e-05	0.066	0.996	TRUE
Pyroglutamine*	Hypertension	0.061	41	1.65e-06	0.792	0.096	TRUE
C-glycosyltryptophan*	Hypertension	0.048	24	0.001	0.446	0.040	TRUE
X-11374	Hypertension	0.108	23	0.222	0.146	0.026	TRUE
X-11381	Hypertension	0.036	29	0.668	0.317	0.084	TRUE

Exposure	Outcome	R2	F	Heterogeneity	Pleiotropy	power	Direction
X-03056--N-[3-(2-Oxopyrrolidin-1-yl)propyl]acetamide	Hypertension	0.083	36	0.152	0.188	0.736	TRUE
X-11412	Hypertension	0.094	24	0.003	0.962	0.053	TRUE
X-01911	Hypertension	0.040	27	0.002	0.795	0.055	TRUE
X-11422--xanthine	Hypertension	0.012	24	0.632	0.532	0.064	TRUE
X-11423--O-sulfo-L-tyrosine	Hypertension	0.083	27	3.57e-08	0.439	0.863	TRUE
X-09789	Hypertension	0.018	27	0.016	0.507	0.077	TRUE
X-11437	Hypertension	0.026	24	0.764	0.582	0.029	TRUE
X-11438	Hypertension	0.060	24	6.51e-04	0.535	0.092	TRUE
X-11440	Hypertension	0.120	77	5.16e-09	0.366	0.255	TRUE
X-11441	Hypertension	0.096	85	0.019	0.493	0.041	TRUE
X-11442	Hypertension	0.098	87	0.037	0.592	0.051	TRUE
X-11444	Hypertension	0.031	28	9e-07	0.102	0.149	TRUE
X-11445--5-alpha-pregnan-3beta,20alpha-disulfate	Hypertension	0.097	28	0.392	0.948	0.205	TRUE
X-11452	Hypertension	0.020	24	5.51e-04	0.919	0.561	TRUE
X-11469	Hypertension	0.035	32	2.36e-04	0.498	0.053	TRUE
X-11470	Hypertension	0.029	29	2.61e-18	0.587	0.351	TRUE
X-11478	Hypertension	0.045	35	0.012	0.217	0.161	TRUE

Exposure	Outcome	R2	F	Heterogeneity	Pleiotropy	power	Direction
X-11483	Hypertension	0.041	25	0.368	0.758	0.046	TRUE
X-11485	Hypertension	0.011	24	0.417		0.047	TRUE
X-11491	Hypertension	0.058	36	1.09e-04	0.133	0.254	TRUE
X-11497	Hypertension	0.017	23	0.205	0.147	0.812	TRUE
HWESASXX*	Hypertension	0.024	29	8.89e-04	0.265	0.036	TRUE
X-11521	Hypertension	0.023	26	0.211	0.608	0.079	TRUE
X-11529	Hypertension	0.211	318	0.376	0.410	0.053	TRUE
X-11530	Hypertension	0.097	147	0.528	0.839	0.047	TRUE
X-11537	Hypertension	0.028	25	0.949	0.936	0.080	TRUE
X-11538	Hypertension	0.102	70	0.035	0.075	0.354	TRUE
X-11540	Hypertension	0.042	23	0.253	0.216	0.235	TRUE
X-11546	Hypertension	0.127	23	0.411	0.511	0.069	TRUE
X-11550	Hypertension	0.050	24	4.51e-08	0.514	0.379	TRUE
X-11552	Hypertension	0.084	23	0.575	0.606	0.158	TRUE
X-11593--O-methylascorbate*	Hypertension	0.185	60	0.532	0.449	1.000	TRUE
Adrenate (22:4n6)	Hypertension	0.027	39	0.029	0.544	0.087	TRUE
Homostachydrine*	Hypertension	0.053	22	0.531	0.305	0.094	TRUE

Exposure	Outcome	R2	F	Heterogeneity	Pleiotropy	power	Direction
ADSGEGDFXAEGGGVR*	Hypertension	0.036	33	6.81e-05	0.828	0.338	TRUE
X-11786--methylcysteine	Hypertension	0.078	27	0.439	0.816	0.041	TRUE
X-11787	Hypertension	0.151	77	0.006	0.835	0.898	TRUE
X-11792	Hypertension	0.133	32	1.41e-07	0.078	0.127	TRUE
X-11793--oxidized bilirubin*	Hypertension	0.092	78	6.07e-05	0.596	0.088	TRUE
X-11795	Hypertension	0.022	23	0.478	0.684	0.573	TRUE
X-11799	Hypertension	0.049	60	0.580	0.569	0.042	TRUE
X-11805	Hypertension	0.025	23	0.338	0.456	0.037	TRUE
X-11818	Hypertension	0.048	24	0.175	0.430	0.046	TRUE
X-11820	Hypertension	0.028	29	0.417	0.582	0.037	TRUE
X-11843	Hypertension	0.025	23			0.060	TRUE
X-11845	Hypertension	0.101	22	0.435	0.549	0.072	TRUE
X-11847	Hypertension	0.027	22	0.015	0.122	0.063	TRUE
X-11849	Hypertension	0.031	23	0.015	0.150	0.059	TRUE
X-11850	Hypertension	0.024	22	0.324	0.790	0.029	TRUE
X-11852	Hypertension	0.055	23	0.191	0.067	0.050	TRUE
X-11858	Hypertension	0.048	26	0.087	0.910	0.144	TRUE

Exposure	Outcome	R2	F	Heterogeneity	Pleiotropy	power	Direction
X-11859	Hypertension	0.056	22	0.474	0.439	0.585	TRUE
X-11876	Hypertension	0.038	22	0.002	0.803	0.040	TRUE
1-arachidonoylglycerophosphocholine*	Hypertension	0.104	54	0.185	0.123	0.071	TRUE
1-palmitoleoylglycerophosphocholine*	Hypertension	0.026	24	3.6e-06	0.076	0.111	TRUE
X-11905	Hypertension	0.103	39	0.381	0.957	0.150	TRUE
X-12007	Hypertension	0.320	23	0.074	0.462	0.148	TRUE
X-12013	Hypertension	0.055	26	0.104	0.222	0.030	TRUE
Gamma-glutamylmethionine*	Hypertension	0.062	23	0.626	0.267	0.113	TRUE
Gamma-glutamylthreonine*	Hypertension	0.035	23	0.193	0.525	0.800	TRUE
X-12029	Hypertension	0.079	22	0.164	0.564	0.925	TRUE
X-12038	Hypertension	0.118	23	1.25e-05	0.232	0.086	TRUE
X-12039	Hypertension	0.053	23	0.008	0.886	0.029	TRUE
X-12040	Hypertension	0.399	25	2.22e-04	0.015	0.161	TRUE
X-12056	Hypertension	0.059	23	0.007	0.733	0.030	TRUE
X-12063	Hypertension	0.155	100	1.64e-05	0.642	0.084	TRUE
Gamma-tocopherol	Hypertension	0.027	23	0.405	0.165	0.153	TRUE
Gamma-glutamylphenylalanine	Hypertension	0.069	24	0.004	0.892	0.461	TRUE

Exposure	Outcome	R2	F	Heterogeneity	Pleiotropy	power	Direction
Isobutyrylcarnitine	Hypertension	0.065	48	0.002	0.459	0.055	TRUE
Pseudouridine	Hypertension	0.061	24	0.097	0.669	0.923	TRUE
Valerate	Hypertension	0.006	22			0.181	TRUE
Palmitoleate (16:1n7)	Hypertension	0.017	31	2.99e-05	0.777	0.603	TRUE
Alpha-ketoglutarate	Hypertension	0.045	23	1.02e-07	0.806	0.036	TRUE
Erythronate*	Hypertension	0.090	25	2.42e-28	0.415	1.000	TRUE
Lathosterol	Hypertension	0.048	24	0.223	0.281	0.083	TRUE
X-12092	Hypertension	0.332	291	0.363	0.633	0.103	TRUE
X-12093	Hypertension	0.192	81	0.082	0.236	0.084	TRUE
X-12094	Hypertension	0.018	25	0.100	0.541	0.053	TRUE
X-12095--N1-methyl-3-pyridone-4-carboxamide	Hypertension	0.050	24	0.120	0.694	0.177	TRUE
X-12100--hydroxytryptophan*	Hypertension	0.110	25	0.003	0.442	0.503	TRUE
X-12116	Hypertension	0.030	22	0.020	0.997	0.031	TRUE
Eicosenoate (20:1n9 or 11)	Hypertension	0.019	23	0.061	0.751	0.133	TRUE
X-12188	Hypertension	0.330	25	0.556	0.855	0.053	TRUE
X-12189	Hypertension	0.975	24	0.592	0.417	0.025	TRUE
X-12206	Hypertension	0.039	22	3.43e-05	0.906	0.054	TRUE

Exposure	Outcome	R2	F	Heterogeneity	Pleiotropy	power	Direction
X-12212	Hypertension	0.027	23	0.952	0.529	0.055	TRUE
X-12216	Hypertension	0.019	22			0.080	TRUE
X-12217	Hypertension	0.025	22	4.54e-07	0.663	0.122	TRUE
X-12230	Hypertension	0.009	25	0.937		0.042	TRUE
X-12231	Hypertension	0.033	23	0.005	0.774	0.142	TRUE
X-12236	Hypertension	0.099	23	0.072	0.386	0.315	TRUE
X-12244--N-acetylcarnosine	Hypertension	0.080	36	0.063	0.062	0.032	TRUE
X-12253	Hypertension	0.016	23	0.559	0.587	0.072	TRUE
X-12261	Hypertension	0.155	23	0.905	0.636	0.039	TRUE
X-12329	Hypertension	0.469	22	0.162	0.691	0.040	TRUE
X-10346	Hypertension	0.051	22	0.229	0.827	0.037	TRUE
ADpSGEGDFXAEGGGVR*	Hypertension	0.044	43	2.51e-23	0.461	0.309	TRUE
1-eicosatrienoylglycerophosphocholine*	Hypertension	0.048	28	0.075	0.173	0.455	TRUE
1-docosahexaenoylglycerophosphocholine*	Hypertension	0.012	22	0.198	0.295	0.431	TRUE
X-12405	Hypertension	0.014	23	0.424	0.259	0.030	TRUE
X-12407	Hypertension	0.044	24	0.319	0.928	0.051	TRUE
X-12428	Hypertension	0.031	22	0.491		0.185	TRUE

Exposure	Outcome	R2	F	Heterogeneity	Pleiotropy	power	Direction
1-eicosadienoylglycerophosphocholine*	Hypertension	0.022	24	0.260	0.274	0.029	TRUE
X-12435	Hypertension	1.5	24	0.132	0.923	0.035	TRUE
X-12441--12-hydroxyeicosatetraenoate (12-HETE)	Hypertension	0.078	36	0.674	0.573	0.073	TRUE
X-12442--5,8-tetradecadienoate	Hypertension	0.030	37	0.934	0.849	0.078	TRUE
X-12443	Hypertension	0.036	22	0.609	0.852	0.034	TRUE
X-12450	Hypertension	0.060	23	0.380	0.801	0.105	TRUE
X-12456	Hypertension	0.101	39	0.632	0.552	0.036	TRUE
X-12465	Hypertension	0.024	22	0.144	0.094	0.067	TRUE
Octanoylcarnitine	Hypertension	0.081	88	6.77e-04	0.165	0.637	TRUE
Alpha-hydroxyisovalerate	Hypertension	0.047	38	0.268	0.741	0.224	TRUE
N-acetylthreonine	Hypertension	0.021	23	0.293	0.864	0.123	TRUE
Decanoylcarnitine	Hypertension	0.071	59	0.001	0.591	0.248	TRUE
1-palmitoylglycerophosphocholine	Hypertension	0.061	24	0.016	0.141	0.752	TRUE
1-heptadecanoylglycerophosphocholine	Hypertension	0.013	23	0.036	0.396	0.025	TRUE
1-oleoylglycerophosphocholine	Hypertension	0.022	23	0.001	0.405	0.047	TRUE
1-stearoylglycerophosphocholine	Hypertension	0.012	23	0.109	0.674	0.137	TRUE
5-dodecenoate (12:1n7)	Hypertension	0.038	25	0.004	0.951	0.249	TRUE

Exposure	Outcome	R2	F	Heterogeneity	Pleiotropy	power	Direction
Stearidonate (18:4n3)	Hypertension	0.021	31	0.018	0.724	0.067	TRUE
10-heptadecenoate (17:1n7)	Hypertension	0.006	22	0.665		0.122	TRUE
10-nonadecenoate (19:1n9)	Hypertension	0.013	25	0.449	0.344	0.029	TRUE
Epiandrosterone sulfate	Hypertension	0.075	63	0.047	0.760	0.266	TRUE
Linolenate [alpha or gamma; (18:3n3 or 6)]	Hypertension	0.009	22	3.61e-05	0.474	0.476	TRUE
X-12510--2-aminooctanoic acid	Hypertension	0.129	90	0.183	0.445	0.615	TRUE
X-12524	Hypertension	0.050	24	0.438	0.462	0.845	TRUE
Bilirubin (E,Z or Z,E)*	Hypertension	0.080	51	0.326	0.214	0.035	TRUE
X-12544	Hypertension	0.040	23	0.526	0.891	0.051	TRUE
X-12556	Hypertension	0.054	29	1.01e-04	0.400	0.650	TRUE
1-arachidonoylglycerophosphoinositol*	Hypertension	0.077	41	0.013	0.291	0.034	TRUE
X-12627	Hypertension	0.025	22	0.554	0.619	0.111	TRUE
X-12644	Hypertension	0.047	26	0.721	0.554	0.054	TRUE
X-12645	Hypertension	0.047	23	0.005	0.979	0.336	TRUE
Asparagine	Hypertension	0.101	30	0.004	0.722	0.117	TRUE
X-12680	Hypertension	0.088	22	0.026	0.939	0.279	TRUE
X-12696	Hypertension	0.060	33	0.472	0.495	0.214	TRUE

Exposure	Outcome	R2	F	Heterogeneity	Pleiotropy	power	Direction
X-12704	Hypertension	0.058	26	0.526	0.539	0.292	TRUE
X-12712	Hypertension	1.8	25	0.034	0.578	0.084	TRUE
X-12717	Hypertension	0.056	23	0.091	0.534	0.054	TRUE
X-12719	Hypertension	0.057	22	0.243	0.481	0.239	TRUE
X-12726	Hypertension	0.056	24	0.100	0.640	0.060	TRUE
X-12728	Hypertension	1.5	26	0.185	0.770	0.025	TRUE
X-12729	Hypertension	0.184	24	0.606	0.281	0.053	TRUE
X-12734	Hypertension	0.019	25	0.571	0.888	0.033	TRUE
X-12740	Hypertension	0.068	24	6.05e-05	0.167	0.094	TRUE
X-12749	Hypertension	0.094	25	3.74e-06	0.808	0.360	TRUE
Stachydrine	Hypertension	0.016	22	0.050		0.057	TRUE
Isovalerylcarnitine	Hypertension	0.084	45	3.29e-05	0.118	0.534	TRUE
Stearoylcarnitine	Hypertension	0.016	28	0.007	0.279	0.899	TRUE
1-stearoylglycerophosphoethanolamine	Hypertension	0.033	26	1.05e-06	0.688	0.076	TRUE
1-linoleoylglycerophosphocholine	Hypertension	0.032	24	0.207	0.203	0.337	TRUE
Bradykinin, des-arg(9)	Hypertension	0.135	44	4.59e-07	0.520	0.062	TRUE
X-12771	Hypertension	0.046	23	0.598	0.715	0.044	TRUE

Exposure	Outcome	R2	F	Heterogeneity	Pleiotropy	power	Direction
X-12776	Hypertension	0.026	23	0.875		1.000	TRUE
Gamma-glutamylisoleucine*	Hypertension	0.064	22	0.151	0.016	0.085	TRUE
X-12786	Hypertension	0.023	24	2.38e-04	0.504	0.209	TRUE
X-12798	Hypertension	0.173	233	0.003	0.140	0.273	TRUE
X-12816	Hypertension	0.016	24	0.628	0.520	0.050	TRUE
X-12830	Hypertension	0.054	22	0.585	0.756	0.038	TRUE
X-12833	Hypertension	0.820	24	0.078	0.200	0.046	TRUE
X-12844	Hypertension	0.032	33	0.742	0.777	0.147	TRUE
X-12847	Hypertension	0.012	23	0.443		0.050	TRUE
X-12850	Hypertension	0.042	31	0.021	0.034	0.026	TRUE
Laurylcarnitine	Hypertension	0.034	24	0.002	0.539	0.658	TRUE
X-12851	Hypertension	0.021	24	0.012	0.578	0.392	TRUE
X-12855	Hypertension	0.060	24	0.004	0.996	0.093	TRUE
X-12990--docosapentaenoic acid (n6-DPA)	Hypertension	0.062	27	0.945	0.888	0.046	TRUE
Isovalerate	Hypertension	0.006	22	0.678		0.033	TRUE
X-13069	Hypertension	0.042	24	6.38e-04	0.374	0.400	TRUE
X-13183--stearamide	Hypertension	0.080	22	0.401	0.741	0.150	TRUE

Exposure	Outcome	R2	F	Heterogeneity	Pleiotropy	power	Direction
X-13215	Hypertension	0.107	23	0.384	0.616	0.993	TRUE
7-methylguanine	Hypertension	0.015	22	0.476	0.269	0.045	TRUE
Phenylacetylglutamine	Hypertension	0.036	24	3.39e-07	0.393	0.038	TRUE
Pro-hydroxy-pro	Hypertension	0.035	24	0.070	0.855	0.039	TRUE
N2,N2-dimethylguanosine	Hypertension	0.171	24	0.003	0.497	0.956	TRUE
Cysteine-glutathione disulfide	Hypertension	0.091	26	0.306	0.402	0.057	TRUE
Oleoylcarnitine	Hypertension	0.019	27	0.002	0.929	0.576	TRUE
1-arachidonoylglycerophosphoethanolamine*	Hypertension	0.074	39	0.384	0.074	0.047	TRUE
X-13429	Hypertension	0.077	95	0.713	0.398	0.039	TRUE
X-13431--nonanoylcarnitine*	Hypertension	0.129	86	5.31e-08	0.301	0.083	TRUE
X-13435	Hypertension	0.066	40	2.8e-04	0.093	0.111	TRUE
X-13477	Hypertension	0.021	30	2.39e-04	0.904	0.047	TRUE
2-palmitoylglycerophosphocholine*	Hypertension	0.045	22	0.002	0.113	0.106	TRUE
2-oleoylglycerophosphocholine*	Hypertension	0.033	23	0.026	0.033	0.315	TRUE
2-stearoylglycerophosphocholine*	Hypertension	0.030	25	0.239	0.543	0.512	TRUE
2-linoleoylglycerophosphocholine*	Hypertension	0.046	23	0.731	0.594	0.444	TRUE
X-13496	Hypertension	0.094	24	0.053	0.200	1.000	TRUE

Exposure	Outcome	R2	F	Heterogeneity	Pleiotropy	power	Direction
1-palmitoylglycerophosphoinositol*	Hypertension	0.019	22	0.175	0.940	0.086	TRUE
X-13548	Hypertension	0.116	24	0.085	0.787	0.437	TRUE
X-13549	Hypertension	0.037	23	0.079	0.493	0.147	TRUE
X-13553	Hypertension	0.015	23	0.678		0.057	TRUE
X-13619	Hypertension	0.053	23	0.006	0.503	0.320	TRUE
2-methylbutyroylcarnitine	Hypertension	0.053	24	1.02e-14	0.033	1.000	TRUE
Hydroxyisovaleroyl carnitine	Hypertension	0.028	37	3.21e-04	0.055	0.361	TRUE
Glutaroyl carnitine	Hypertension	0.145	51	1.09e-05	0.363	0.589	TRUE
X-13658	Hypertension	0.163	26	0.227	0.319	0.030	TRUE
X-13671	Hypertension	0.037	26	0.975	0.696	0.048	TRUE
2-tetradecenoyl carnitine	Hypertension	0.041	25	0.001	0.244	0.032	TRUE
4-hydroxyhippurate	Hypertension	0.051	23	0.633	0.515	0.412	TRUE
X-13741	Hypertension	0.040	25	2.11e-07	0.097	0.218	TRUE
1-myristoylglycerophosphocholine	Hypertension	0.010	23	0.008	0.363	0.071	TRUE
1-oleoylglycerophosphoethanolamine	Hypertension	0.015	27	0.009	0.744	0.115	TRUE
1-palmitoylglycerophosphoethanolamine	Hypertension	0.055	29	3.14e-05	0.019	0.384	TRUE
3-(3-hydroxyphenyl)propionate	Hypertension	0.176	22	1.22e-13	0.653	0.033	TRUE

Exposure	Outcome	R2	F	Heterogeneity	Pleiotropy	power	Direction
Tetradecanedioate	Hypertension	0.106	47	0.133	0.203	0.038	TRUE
2-hydroxypalmitate	Hypertension	0.098	24	0.058	0.605	0.064	TRUE
Hexadecanedioate	Hypertension	0.091	43	2.2e-05	0.065	0.189	TRUE
Dihomo-linolenate (20:3n3 or n6)	Hypertension	0.055	29	0.228	0.955	0.067	TRUE
X-13859	Hypertension	0.102	24	0.001	0.712	0.156	TRUE
Threitol	Hypertension	0.011	24	0.370	0.393	0.053	TRUE
X-14056	Hypertension	0.017	24	0.017	0.661	0.373	TRUE
X-14057	Hypertension	0.025	23	0.108	0.927	0.269	TRUE
X-14086	Hypertension	0.086	26	1.85e-05	0.061	0.252	TRUE
P-cresol sulfate	Hypertension	0.026	24	0.035	0.493	0.063	TRUE
X-14189--leucylalanine	Hypertension	0.094	32	2.92e-18	0.436	0.363	TRUE
X-14205--alpha-glutamyltyrosine	Hypertension	0.113	29	5.76e-05	0.407	0.997	TRUE
X-14208--phenylalanylserine	Hypertension	0.122	30	2.05e-10	0.514	0.800	TRUE
X-14304--leucylalanine	Hypertension	0.115	28	0.039	0.966	0.717	TRUE
X-14374	Hypertension	0.018	22	0.220	0.290	0.045	TRUE
X-14450--phenylalanylleucine	Hypertension	0.037	23	0.849	0.593	0.056	TRUE
X-14473	Hypertension	0.036	26	3.92e-04	0.719	0.071	TRUE

Exposure	Outcome	R2	F	Heterogeneity	Pleiotropy	power	Direction
X-14541	Hypertension	0.129	23	0.290	0.938	0.041	TRUE
X-14588	Hypertension	0.019	24	0.655	0.233	0.057	TRUE
X-14625	Hypertension	0.037	26	1.21e-05	0.013	0.352	TRUE
X-14626	Hypertension	0.064	42	0.001	0.454	0.390	TRUE
X-14632	Hypertension	0.175	22	0.066	0.067	0.044	TRUE
X-14658	Hypertension	0.046	22	0.014	0.767	0.170	TRUE
X-14662	Hypertension	0.065	24	0.049	0.924	0.044	TRUE
X-14745	Hypertension	0.031	23	0.975	0.808	0.079	TRUE
Gamma-glutamylglutamate	Hypertension	0.200	24	0.088	0.418	0.256	TRUE
Octadecanedioate	Hypertension	0.027	37	7.68e-04	0.764	0.096	TRUE
Leucylleucine	Hypertension	0.056	23	0.010	0.576	0.027	TRUE
7-alpha-hydroxy-3-oxo-4-cholestenoate (7-Hoca)	Hypertension	0.025	23	0.055	0.219	0.071	TRUE
N-Butyl Oleate	Hypertension	0.025	22	0.149	0.382	0.297	TRUE
Dimethylarginine (SDMA + ADMA)	Hypertension	0.069	23	1.09e-06	0.500	0.040	TRUE
Taurolithocholate 3-sulfate	Hypertension	0.021	23	0.421	0.739	0.071	TRUE
X-14977--vanillin	Hypertension	0.023	21	0.385		0.300	TRUE
Succinylcarnitine	Hypertension	0.173	43	2.91e-07	0.699	1.000	TRUE

Exposure	Outcome	R2	F	Heterogeneity	Pleiotropy	power	Direction
Tryptophan betaine	Hypertension	0.037	38	0.002	0.725	0.214	TRUE
Cyclo(leu-pro)	Hypertension	0.049	22	0.001	0.392	0.040	TRUE
Chiro-inositol	Hypertension	0.054	23	0.378	0.124	0.041	TRUE
5alpha-androstan-3beta,17beta-diol disulfate	Hypertension	0.061	43	0.001	0.691	0.105	TRUE
4-androsten-3beta,17beta-diol disulfate 1*	Hypertension	0.096	52	0.011	0.323	0.044	TRUE
4-androsten-3beta,17beta-diol disulfate 2*	Hypertension	0.053	30	1.9e-07	0.753	0.507	TRUE
2-hydroxyglutarate	Hypertension	0.019	22	0.124	0.149	0.188	TRUE
Ergothioneine	Hypertension	0.012	22			0.087	TRUE
Palmitoyl sphingomyelin	Hypertension	0.098	24	1.48e-04	0.283	0.999	TRUE
Phenylalanylphenylalanine	Hypertension	0.014	23	0.202	0.893	0.148	TRUE
Cis-4-decenoyl carnitine	Hypertension	0.059	73	0.871	0.594	0.297	TRUE
15-methylpalmitate (isobar with 2-methylpalmitate)	Hypertension	0.030	23	0.431	0.180	0.209	TRUE

n: 2187

Table S4. Full result of MR estimates for the effect of metabolites on hypertension(UKB).

Exposure	Outcome	nSNP	MR Egger		Weighted median		Inverse variance weighted		MR_PRESSO	
			OR (95% CI)	pval	OR (95% CI)	pval	OR (95% CI)	pval	OR (95% CI)	pval
arachidonate (20:4n6)	Hypertension	51	0.998 (0.996 to 1.000)	0.059	0.999 (0.998 to 1.000)	0.022	0.999 (0.999 to 1.000)	0.021		
deoxycholate	Hypertension	11	1.003 (0.991 to 1.015)	0.626	1.000 (0.995 to 1.004)	0.888	1.000 (0.996 to 1.004)	0.935		
arachidate (20:0)	Hypertension	16	0.994 (0.988 to 1.001)	0.115	0.996 (0.992 to 1.000)	0.074	0.997 (0.994 to 1.000)	0.050		
margarate (17:0)	Hypertension	10	0.994 (0.983 to 1.004)	0.269	0.998 (0.993 to 1.003)	0.422	0.998 (0.994 to 1.003)	0.466		
inosine	Hypertension	21	1.002 (0.997 to 1.007)	0.429	1.004 (1.001 to 1.007)	0.005	1.003 (1.001 to 1.005)	0.002		
myo-inositol	Hypertension	14	0.999 (0.993 to 1.005)	0.799	1.001 (0.997 to 1.004)	0.619	1.003 (1.000 to 1.006)	0.064		

Exposure	Outcome	nSNP	MR Egger		Weighted median		Inverse variance weighted		MR_PRESSO	
			OR (95% CI)	pval	OR (95% CI)	pval	OR (95% CI)	pval	OR (95% CI)	pval
isoleucine	Hypertension	13	1.005 (0.997 to 1.013)	0.234	0.999 (0.995 to 1.003)	0.609	0.997 (0.993 to 1.002)	0.219	0.999 (0.995 to 1.002)	0.467
alanine	Hypertension	14	1.002 (0.998 to 1.007)	0.326	1.001 (0.997 to 1.005)	0.552	0.999 (0.996 to 1.002)	0.391		
3-methoxytyrosine	Hypertension	20	1.000 (0.998 to 1.002)	0.765	1.000 (0.999 to 1.002)	0.569	1.000 (0.998 to 1.001)	0.831		
beta-hydroxyisovalerate	Hypertension	26	0.999 (0.995 to 1.002)	0.466	0.999 (0.997 to 1.001)	0.275	1.000 (0.998 to 1.002)	0.686	0.999 (0.997 to 1.000)	0.183
taurodeoxycholate	Hypertension	16	0.999 (0.992 to 1.006)	0.744	0.998 (0.994 to 1.001)	0.221	1.000 (0.997 to 1.003)	0.786		
threonine	Hypertension	14	1.008 (0.993 to 1.022)	0.310	1.000 (0.996 to 1.004)	0.956	1.003 (0.998 to 1.009)	0.213	1.002 (0.999 to 1.006)	0.190

Exposure	Outcome	nSNP	MR Egger		Weighted median		Inverse variance weighted		MR_PRESSO	
			OR (95% CI)	pval	OR (95% CI)	pval	OR (95% CI)	pval	OR (95% CI)	pval
tyrosine	Hypertension	17	1.004 (0.996 to 1.012)	0.315	1.002 (0.998 to 1.006)	0.293	1.000 (0.996 to 1.003)	0.858	1.001 (0.998 to 1.004)	0.590
lysine	Hypertension	19	1.000 (0.998 to 1.002)	0.740	1.000 (0.998 to 1.001)	0.902	1.000 (0.999 to 1.001)	0.687		
malate	Hypertension	51	1.003 (1.001 to 1.006)	0.006	1.001 (0.999 to 1.003)	0.259	1.000 (0.999 to 1.002)	0.553		
palmitate (16:0)	Hypertension	29	1.000 (0.999 to 1.002)	0.670	1.000 (0.999 to 1.001)	0.479	1.000 (0.999 to 1.001)	0.845		
myristate (14:0)	Hypertension	24	0.997 (0.991 to 1.003)	0.365	1.003 (0.999 to 1.006)	0.141	1.001 (0.998 to 1.003)	0.556		
kynurenate	Hypertension	18	1.001 (0.997 to 1.006)	0.620	1.001 (0.998 to 1.004)	0.686	1.001 (0.999 to 1.004)	0.261		

Exposure	Outcome	nSNP	MR Egger		Weighted median		Inverse variance weighted		MR_PRESSO	
			OR (95% CI)	pval	OR (95% CI)	pval	OR (95% CI)	pval	OR (95% CI)	pval
5,6-dihydrothymine	Hypertension	27	0.999 (0.993 to 1.006)	0.826	0.998 (0.995 to 1.001)	0.124	0.998 (0.995 to 1.000)	0.056	0.997 (0.994 to 0.999)	0.011
5-methylthioadenosine (MTA)	Hypertension	17	1.002 (0.993 to 1.010)	0.710	0.998 (0.994 to 1.001)	0.242	0.997 (0.993 to 1.001)	0.132	0.998 (0.995 to 1.001)	0.156
2-hydroxyphenylacetate	Hypertension	19	0.998 (0.991 to 1.005)	0.529	1.002 (0.998 to 1.005)	0.308	1.000 (0.998 to 1.003)	0.883		
succinate	Hypertension	17	1.000 (0.992 to 1.008)	0.969	0.999 (0.996 to 1.003)	0.749	1.001 (0.997 to 1.005)	0.617	1.001 (0.998 to 1.004)	0.521
ornithine	Hypertension	21	1.001 (0.995 to 1.007)	0.791	1.001 (0.998 to 1.004)	0.407	1.000 (0.998 to 1.002)	0.941		
5-oxoproline	Hypertension	23	1.003 (0.998 to 1.007)	0.264	1.004 (1.001 to 1.007)	0.006	1.003 (1.001 to 1.005)	0.013		

Exposure	Outcome	nSNP	MR Egger		Weighted median		Inverse variance weighted		MR_PRESSO	
			OR (95% CI)	pval	OR (95% CI)	pval	OR (95% CI)	pval	OR (95% CI)	pval
N6,N6,N6-trimethyllysine	Hypertension	10	0.999 (0.992 to 1.006)	0.828	1.000 (0.996 to 1.004)	0.990	1.000 (0.996 to 1.003)	0.814		
orotate	Hypertension	16	1.000 (0.996 to 1.003)	0.820	1.000 (0.997 to 1.002)	0.711	1.000 (0.998 to 1.002)	0.861		
pantothenate	Hypertension	17	1.001 (0.996 to 1.006)	0.649	1.000 (0.996 to 1.003)	0.784	0.999 (0.996 to 1.002)	0.568		
glycerol	Hypertension	16	1.000 (0.994 to 1.006)	0.903	1.003 (1.000 to 1.007)	0.060	1.002 (0.999 to 1.005)	0.166		
kynurenine	Hypertension	18	1.016 (1.001 to 1.031)	0.046	1.001 (0.998 to 1.005)	0.522	0.995 (0.987 to 1.003)	0.242	1.000 (0.997 to 1.003)	0.874
sucrose	Hypertension	10	0.999 (0.996 to 1.001)	0.320	0.999 (0.997 to 1.000)	0.068	0.999 (0.997 to 1.000)	0.097		

Exposure	Outcome	nSNP	MR Egger		Weighted median		Inverse variance weighted		MR_PRESSO	
			OR (95% CI)	pval	OR (95% CI)	pval	OR (95% CI)	pval	OR (95% CI)	pval
tartarate	Hypertension	18	1.005 (0.998 to 1.012)	0.150	1.000 (0.997 to 1.004)	0.879	1.001 (0.998 to 1.004)	0.623		
carnitine	Hypertension	36	1.001 (0.999 to 1.003)	0.224	1.000 (0.999 to 1.001)	0.857	1.000 (0.998 to 1.001)	0.391	1.000 (0.999 to 1.001)	0.503
choline	Hypertension	18	0.997 (0.992 to 1.002)	0.217	1.000 (0.997 to 1.004)	0.766	1.001 (0.999 to 1.003)	0.394		
4-acetamidobutanoate	Hypertension	16	0.997 (0.991 to 1.003)	0.385	0.996 (0.992 to 0.999)	0.025	0.996 (0.994 to 0.999)	0.010		
chenodeoxycholate	Hypertension	17	1.001 (0.996 to 1.005)	0.684	1.000 (0.997 to 1.002)	0.748	1.000 (0.998 to 1.003)	0.719		
citrate	Hypertension	17	1.000 (0.996 to 1.003)	0.797	1.000 (0.997 to 1.003)	0.869	1.000 (0.998 to 1.002)	0.866		

Exposure	Outcome	nSNP	MR Egger		Weighted median		Inverse variance weighted		MR_PRESSO	
			OR (95% CI)	pval	OR (95% CI)	pval	OR (95% CI)	pval	OR (95% CI)	pval
N1-methyladenosine	Hypertension	15	1.008 (0.996 to 1.020)	0.232	0.998 (0.994 to 1.002)	0.258	0.999 (0.995 to 1.002)	0.444		
3-aminoisobutyrate	Hypertension	22	1.002 (0.999 to 1.005)	0.187	1.001 (0.999 to 1.004)	0.192	1.001 (1.000 to 1.003)	0.111		
2-isopropylmalate	Hypertension	18	1.002 (0.998 to 1.006)	0.425	0.999 (0.996 to 1.002)	0.525	1.000 (0.997 to 1.002)	0.790		
vanillylmandelate (VMA)	Hypertension	17	1.006 (1.001 to 1.011)	0.030	1.003 (1.000 to 1.006)	0.083	1.001 (0.999 to 1.004)	0.281		
3-methylhistidine	Hypertension	26	1.002 (0.997 to 1.007)	0.401	1.001 (0.998 to 1.003)	0.652	1.001 (0.998 to 1.003)	0.665	1.000 (0.998 to 1.002)	0.746
xanthurenate	Hypertension	16	1.001 (0.997 to 1.004)	0.640	1.003 (0.998 to 1.007)	0.228	1.003 (1.001 to 1.006)	0.003		

Exposure	Outcome	nSNP	MR Egger		Weighted median		Inverse variance weighted		MR_PRESSO	
			OR (95% CI)	pval	OR (95% CI)	pval	OR (95% CI)	pval	OR (95% CI)	pval
4-guanidinobutanoate	Hypertension	25	0.993 (0.986 to 1.000)	0.079	0.999 (0.997 to 1.001)	0.423	1.001 (0.998 to 1.004)	0.529	0.999 (0.998 to 1.001)	0.542
5-hydroxylysine	Hypertension	29	1.000 (0.995 to 1.004)	0.859	1.000 (0.997 to 1.002)	0.794	0.998 (0.995 to 1.001)	0.117	0.997 (0.995 to 1.000)	0.057
pimelate (heptanedioate)	Hypertension	16	1.001 (0.995 to 1.008)	0.647	1.000 (0.996 to 1.003)	0.785	1.000 (0.998 to 1.002)	0.936		
cystathionine	Hypertension	14	0.995 (0.988 to 1.003)	0.240	0.999 (0.995 to 1.002)	0.457	0.999 (0.996 to 1.002)	0.439		
imidazole lactate	Hypertension	20	0.998 (0.993 to 1.003)	0.467	0.998 (0.996 to 1.000)	0.051	1.000 (0.998 to 1.002)	0.762		
glycerate	Hypertension	28	0.998 (0.994 to 1.002)	0.362	1.000 (0.997 to 1.002)	0.808	1.000 (0.999 to 1.002)	0.675		

Exposure	Outcome	nSNP	MR Egger		Weighted median		Inverse variance weighted		MR_PRESSO	
			OR (95% CI)	pval	OR (95% CI)	pval	OR (95% CI)	pval	OR (95% CI)	pval
guanosine	Hypertension	18	1.001 (0.998 to 1.003)	0.655	1.000 (0.998 to 1.001)	0.968	1.000 (0.999 to 1.001)	0.713		
suberate (octanedioate)	Hypertension	20	1.007 (1.000 to 1.014)	0.081	1.004 (1.001 to 1.008)	0.018	1.003 (0.999 to 1.006)	0.149	1.002 (0.998 to 1.005)	0.385
methylsuccinate	Hypertension	18	0.997 (0.990 to 1.004)	0.431	0.998 (0.996 to 1.000)	0.110	0.999 (0.997 to 1.001)	0.211		
hippurate	Hypertension	20	1.001 (0.996 to 1.007)	0.615	0.999 (0.996 to 1.002)	0.419	0.999 (0.996 to 1.001)	0.278		
ethylmalonate	Hypertension	81	1.000 (0.999 to 1.001)	0.903	1.000 (0.999 to 1.001)	0.513	1.000 (1.000 to 1.001)	0.309		
ribitol	Hypertension	26	1.004 (1.000 to 1.008)	0.041	0.999 (0.997 to 1.002)	0.695	1.000 (0.998 to 1.002)	0.947	1.000 (0.998 to 1.002)	0.750

Exposure	Outcome	nSNP	MR Egger		Weighted median		Inverse variance weighted		MR_PRESSO	
			OR (95% CI)	pval	OR (95% CI)	pval	OR (95% CI)	pval	OR (95% CI)	pval
N-acetylalanine	Hypertension	18	1.001 (0.991 to 1.011)	0.803	1.002 (0.999 to 1.006)	0.145	1.002 (0.999 to 1.005)	0.213	1.003 (1.000 to 1.005)	0.033
malonate	Hypertension	38	1.001 (0.997 to 1.004)	0.639	0.998 (0.996 to 1.000)	0.101	0.999 (0.997 to 1.001)	0.214	0.999 (0.998 to 1.001)	0.355
N-acetylmethionine	Hypertension	20	0.999 (0.993 to 1.005)	0.732	0.998 (0.995 to 1.001)	0.206	0.999 (0.997 to 1.002)	0.561		
phenylacetate	Hypertension	8	0.998 (0.990 to 1.006)	0.612	0.998 (0.994 to 1.003)	0.483	0.999 (0.995 to 1.004)	0.800		
urate	Hypertension	26	1.002 (0.997 to 1.006)	0.455	1.003 (1.000 to 1.005)	0.027	1.002 (1.000 to 1.004)	0.026		
ursodeoxycholate	Hypertension	18	1.007 (1.002 to 1.011)	0.012	1.001 (0.998 to 1.004)	0.619	1.001 (0.998 to 1.003)	0.511		

Exposure	Outcome	nSNP	MR Egger		Weighted median		Inverse variance weighted		MR_PRESSO	
			OR (95% CI)	pval	OR (95% CI)	pval	OR (95% CI)	pval	OR (95% CI)	pval
arginine	Hypertension	21	0.993 (0.980 to 1.006)	0.282	0.996 (0.993 to 1.000)	0.037	0.999 (0.994 to 1.004)	0.610	0.997 (0.994 to 1.000)	0.034
fumarate	Hypertension	18	0.999 (0.992 to 1.006)	0.811	0.998 (0.994 to 1.001)	0.184	1.000 (0.997 to 1.002)	0.718		
serine	Hypertension	18	0.994 (0.988 to 0.999)	0.045	0.998 (0.995 to 1.001)	0.155	0.997 (0.994 to 1.000)	0.095	0.999 (0.997 to 1.001)	0.273
valine	Hypertension	15	1.001 (0.993 to 1.009)	0.858	0.999 (0.995 to 1.002)	0.526	0.999 (0.996 to 1.002)	0.657		
urea	Hypertension	11	1.005 (0.996 to 1.013)	0.318	1.000 (0.996 to 1.005)	0.888	1.001 (0.997 to 1.004)	0.755		
cortisol	Hypertension	16	0.995 (0.987 to 1.003)	0.262	1.000 (0.996 to 1.004)	0.975	1.001 (0.997 to 1.004)	0.732	1.000 (0.997 to 1.002)	0.834

Exposure	Outcome	nSNP	MR Egger		Weighted median		Inverse variance weighted		MR_PRESSO	
			OR (95% CI)	pval	OR (95% CI)	pval	OR (95% CI)	pval	OR (95% CI)	pval
cortisone	Hypertension	20	0.996 (0.991 to 1.002)	0.177	0.999 (0.996 to 1.002)	0.674	1.000 (0.997 to 1.003)	0.890	0.999 (0.997 to 1.001)	0.405
2-hydroxystearate	Hypertension	24	1.006 (0.998 to 1.014)	0.168	1.002 (0.999 to 1.005)	0.236	1.001 (0.999 to 1.004)	0.226		
retinol (Vitamin A)	Hypertension	16	1.008 (1.000 to 1.016)	0.057	0.997 (0.993 to 1.000)	0.084	0.997 (0.993 to 1.000)	0.053	0.997 (0.994 to 1.000)	0.107
gamma-glutamylhistidine	Hypertension	15	1.003 (0.997 to 1.010)	0.349	1.003 (1.000 to 1.007)	0.055	1.001 (0.999 to 1.004)	0.323		
paraxanthine	Hypertension	21	0.999 (0.995 to 1.002)	0.459	0.999 (0.996 to 1.002)	0.519	0.998 (0.996 to 1.000)	0.034		
quinate	Hypertension	24	1.001 (0.997 to 1.005)	0.545	0.999 (0.996 to 1.002)	0.383	0.999 (0.997 to 1.001)	0.374		

Exposure	Outcome	nSNP	MR Egger		Weighted median		Inverse variance weighted		MR_PRESSO	
			OR (95% CI)	pval	OR (95% CI)	pval	OR (95% CI)	pval	OR (95% CI)	pval
indolelactate	Hypertension	30	1.000 (0.995 to 1.004)	0.957	1.000 (0.998 to 1.003)	0.745	1.001 (0.999 to 1.003)	0.296		
gamma-glutamylleucine	Hypertension	20	1.000 (0.996 to 1.004)	0.970	1.001 (0.998 to 1.004)	0.618	1.002 (1.000 to 1.004)	0.072		
methionine sulfoxide	Hypertension	57	1.000 (0.997 to 1.003)	0.975	1.001 (0.999 to 1.003)	0.375	1.000 (0.999 to 1.002)	0.565		
theobromine	Hypertension	17	0.999 (0.989 to 1.009)	0.864	1.001 (0.997 to 1.004)	0.727	1.002 (0.997 to 1.006)	0.455	1.001 (0.998 to 1.004)	0.499
theophylline	Hypertension	27	1.001 (0.996 to 1.005)	0.786	1.002 (0.999 to 1.005)	0.164	1.001 (0.998 to 1.003)	0.626	1.001 (0.999 to 1.002)	0.514
eicosapentaenoate (EPA; 20:5n3)	Hypertension	52	0.996 (0.991 to 1.002)	0.183	0.998 (0.996 to 0.999)	0.007	0.998 (0.997 to 0.999)	0.002		

Exposure	Outcome	nSNP	MR Egger		Weighted median		Inverse variance weighted		MR_PRESSO	
			OR (95% CI)	pval	OR (95% CI)	pval	OR (95% CI)	pval	OR (95% CI)	pval
glycocholate	Hypertension	12	0.995 (0.988 to 1.003)	0.236	1.001 (0.996 to 1.005)	0.759	1.001 (0.998 to 1.004)	0.488		
taurochenodeoxycholate	Hypertension	15	0.995 (0.987 to 1.002)	0.192	0.999 (0.995 to 1.002)	0.466	1.000 (0.997 to 1.004)	0.785	1.000 (0.997 to 1.003)	0.821
taurocholate	Hypertension	14	0.995 (0.984 to 1.006)	0.349	0.999 (0.995 to 1.003)	0.623	0.999 (0.995 to 1.003)	0.743	0.998 (0.996 to 1.001)	0.151
proline	Hypertension	38	1.003 (0.998 to 1.008)	0.211	1.000 (0.998 to 1.003)	0.718	1.000 (0.998 to 1.002)	0.947	1.001 (0.999 to 1.002)	0.479
1,2-dimyristoyl-GPC (14:0/14:0)	Hypertension	18	0.990 (0.982 to 0.998)	0.027	0.999 (0.996 to 1.002)	0.564	0.999 (0.996 to 1.003)	0.747	0.998 (0.996 to 1.001)	0.303
quinolinate	Hypertension	32	1.007 (0.999 to 1.014)	0.102	1.000 (0.998 to 1.003)	0.817	0.998 (0.993 to 1.002)	0.321	0.999 (0.998 to 1.000)	0.194

Exposure	Outcome	nSNP	MR Egger		Weighted median		Inverse variance weighted		MR_PRESSO	
			OR (95% CI)	pval	OR (95% CI)	pval	OR (95% CI)	pval	OR (95% CI)	pval
1,2-distearoyl-GPC (18:0/18:0)	Hypertension	19	1.003 (1.000 to 1.005)	0.051	1.001 (0.999 to 1.003)	0.298	1.001 (1.000 to 1.003)	0.149	1.000 (0.999 to 1.001)	0.788
1-myristoyl-2-palmitoyl-GPC (14:0/16:0)	Hypertension	17	0.997 (0.986 to 1.008)	0.571	1.002 (0.998 to 1.006)	0.317	1.002 (0.997 to 1.006)	0.502	1.003 (1.000 to 1.006)	0.066
1-palmitoyl-2-oleoyl-GPE (16:0/18:1)	Hypertension	59	0.998 (0.995 to 1.000)	0.100	1.001 (1.000 to 1.003)	0.160	1.001 (1.000 to 1.002)	0.175		
2-arachidonoylglycerol (20:4)	Hypertension	45	1.003 (0.998 to 1.008)	0.297	0.998 (0.996 to 0.999)	0.007	0.999 (0.998 to 1.000)	0.024		
1-stearoyl-GPI (18:0)	Hypertension	30	1.004 (0.999 to 1.009)	0.108	1.000 (0.998 to 1.003)	0.916	1.001 (0.999 to 1.003)	0.478		
stearoyl sphingomyelin (d18:1/18:0)	Hypertension	29	1.004 (0.999 to 1.009)	0.177	1.001 (0.999 to 1.004)	0.352	1.001 (0.999 to 1.004)	0.231		

Exposure	Outcome	nSNP	MR Egger		Weighted median		Inverse variance weighted		MR_PRESSO	
			OR (95% CI)	pval	OR (95% CI)	pval	OR (95% CI)	pval	OR (95% CI)	pval
1-palmityl-GPC (O-16:0)	Hypertension	16	0.994 (0.987 to 1.002)	0.160	0.999 (0.996 to 1.002)	0.577	0.998 (0.994 to 1.001)	0.171	0.998 (0.996 to 1.000)	0.079
1,5-anhydroglucitol (1,5-AG)	Hypertension	29	0.996 (0.992 to 1.001)	0.117	0.999 (0.997 to 1.002)	0.502	1.000 (0.998 to 1.003)	0.809	1.000 (0.998 to 1.001)	0.666
oxalate (ethanedioate)	Hypertension	20	1.006 (0.999 to 1.013)	0.109	1.004 (1.000 to 1.007)	0.048	1.000 (0.997 to 1.003)	0.840		
erythritol	Hypertension	18	1.003 (0.995 to 1.012)	0.463	1.001 (0.997 to 1.004)	0.673	1.000 (0.997 to 1.004)	0.839	1.002 (0.999 to 1.005)	0.223
glycylglycine	Hypertension	28	1.006 (0.985 to 1.027)	0.583	1.006 (1.002 to 1.010)	0.005	1.003 (0.996 to 1.010)	0.381	1.002 (0.999 to 1.005)	0.243
1-oleoylglycerol (18:1)	Hypertension	13	1.005 (0.998 to 1.012)	0.221	1.000 (0.997 to 1.004)	0.850	1.000 (0.997 to 1.004)	0.903	1.001 (0.998 to 1.004)	0.480

Exposure	Outcome	nSNP	MR Egger		Weighted median		Inverse variance weighted		MR_PRESSO	
			OR (95% CI)	pval	OR (95% CI)	pval	OR (95% CI)	pval	OR (95% CI)	pval
citrulline	Hypertension	17	1.003 (0.993 to 1.012)	0.596	1.001 (0.997 to 1.005)	0.601	1.000 (0.997 to 1.003)	0.849		
biliverdin	Hypertension	22	0.999 (0.997 to 1.001)	0.531	0.999 (0.997 to 1.001)	0.232	0.998 (0.997 to 0.999)	0.006		
3-hydroxyoctanoate	Hypertension	21	0.999 (0.998 to 1.001)	0.511	1.000 (0.999 to 1.001)	0.628	1.000 (0.999 to 1.001)	0.598		
2-hydroxyoctanoate	Hypertension	27	0.995 (0.981 to 1.008)	0.442	0.992 (0.989 to 0.995)	5.98e-07	0.997 (0.994 to 1.000)	0.060	0.995 (0.992 to 0.997)	9.38e-04
3-hydroxydecanoate	Hypertension	27	1.002 (1.000 to 1.004)	0.027	1.000 (0.998 to 1.001)	0.777	1.000 (0.999 to 1.001)	0.853		
phenyllactate (PLA)	Hypertension	20	0.998 (0.994 to 1.002)	0.422	1.001 (0.999 to 1.004)	0.269	1.001 (0.999 to 1.003)	0.454		

Exposure	Outcome	nSNP	MR Egger		Weighted median		Inverse variance weighted		MR_PRESSO	
			OR (95% CI)	pval	OR (95% CI)	pval	OR (95% CI)	pval	OR (95% CI)	pval
alpha-hydroxyisocaproate	Hypertension	15	0.996 (0.989 to 1.002)	0.199	1.001 (0.997 to 1.005)	0.594	1.001 (0.998 to 1.005)	0.404		
homoarginine	Hypertension	24	1.003 (0.995 to 1.011)	0.522	1.001 (0.998 to 1.004)	0.412	1.003 (1.000 to 1.007)	0.085	1.001 (0.999 to 1.004)	0.406
homocitrulline	Hypertension	9	1.001 (0.990 to 1.013)	0.817	1.000 (0.995 to 1.004)	0.815	0.998 (0.996 to 1.001)	0.280		
N-acetylaspartate (NAA)	Hypertension	36	0.997 (0.993 to 1.000)	0.088	0.997 (0.995 to 1.000)	0.021	0.999 (0.997 to 1.001)	0.212		
pyroglutamylglutamine	Hypertension	21	1.004 (0.998 to 1.010)	0.241	1.003 (0.999 to 1.006)	0.125	1.001 (0.998 to 1.003)	0.692		
cholate	Hypertension	13	0.995 (0.976 to 1.015)	0.657	1.001 (0.996 to 1.005)	0.822	1.002 (0.997 to 1.007)	0.472	0.999 (0.996 to 1.003)	0.771

Exposure	Outcome	nSNP	MR Egger		Weighted median		Inverse variance weighted		MR_PRESSO	
			OR (95% CI)	pval	OR (95% CI)	pval	OR (95% CI)	pval	OR (95% CI)	pval
gamma-glutamylglutamine	Hypertension	17	1.002 (0.998 to 1.005)	0.385	1.001 (0.999 to 1.002)	0.512	1.001 (0.999 to 1.002)	0.347	1.002 (1.001 to 1.003)	0.004
gamma-glutamyltyrosine	Hypertension	15	1.004 (0.997 to 1.011)	0.332	1.002 (0.998 to 1.005)	0.367	1.002 (0.999 to 1.004)	0.236		
1-linoleoylglycerol (18:2)	Hypertension	26	1.002 (0.994 to 1.010)	0.627	1.000 (0.997 to 1.003)	0.900	1.001 (0.997 to 1.004)	0.670	1.001 (0.998 to 1.003)	0.546
thyroxine	Hypertension	13	1.000 (0.988 to 1.011)	0.952	0.998 (0.993 to 1.002)	0.302	1.001 (0.997 to 1.004)	0.667		
1-methylnicotinamide	Hypertension	13	1.004 (0.996 to 1.012)	0.335	1.000 (0.996 to 1.004)	0.997	1.002 (0.999 to 1.006)	0.250		
3-indoxyl sulfate	Hypertension	10	0.997 (0.989 to 1.004)	0.418	0.999 (0.994 to 1.004)	0.662	0.998 (0.995 to 1.002)	0.335		

Exposure	Outcome	nSNP	MR Egger		Weighted median		Inverse variance weighted		MR_PRESSO	
			OR (95% CI)	pval	OR (95% CI)	pval	OR (95% CI)	pval	OR (95% CI)	pval
N-acetylglycine	Hypertension	18	1.001 (0.996 to 1.006)	0.830	1.000 (0.998 to 1.002)	0.917	0.999 (0.996 to 1.001)	0.289	1.000 (0.999 to 1.001)	0.925
creatine	Hypertension	18	1.004 (0.997 to 1.012)	0.269	0.998 (0.994 to 1.002)	0.328	0.994 (0.990 to 0.999)	0.017	0.999 (0.996 to 1.001)	0.205
galactonate	Hypertension	20	1.000 (0.989 to 1.010)	0.968	1.000 (0.997 to 1.004)	0.811	1.000 (0.996 to 1.004)	0.989	0.999 (0.997 to 1.001)	0.224
1-methylhistidine	Hypertension	20	0.994 (0.987 to 1.001)	0.101	0.997 (0.994 to 1.000)	0.074	0.998 (0.995 to 1.001)	0.224	0.997 (0.995 to 1.000)	0.064
hypoxanthine	Hypertension	23	1.003 (0.982 to 1.024)	0.784	1.001 (0.998 to 1.004)	0.394	0.996 (0.989 to 1.003)	0.313	0.999 (0.996 to 1.002)	0.507
betaine	Hypertension	20	1.002 (0.998 to 1.006)	0.357	1.000 (0.998 to 1.003)	0.749	0.999 (0.996 to 1.002)	0.537	1.000 (0.999 to 1.002)	0.546

Exposure	Outcome	nSNP	MR Egger		Weighted median		Inverse variance weighted		MR_PRESSO	
			OR (95% CI)	pval	OR (95% CI)	pval	OR (95% CI)	pval	OR (95% CI)	pval
xanthine	Hypertension	16	1.009 (0.997 to 1.022)	0.170	1.003 (0.999 to 1.007)	0.107	0.998 (0.992 to 1.004)	0.528	1.001 (0.997 to 1.004)	0.708
pyroglutamylglycine	Hypertension	20	1.002 (0.997 to 1.007)	0.434	0.999 (0.996 to 1.003)	0.687	1.000 (0.997 to 1.003)	0.881		
DSGEGDFXAEGGGVR	Hypertension	31	0.999 (0.987 to 1.012)	0.915	0.999 (0.997 to 1.002)	0.726	0.995 (0.990 to 1.001)	0.077	1.001 (0.999 to 1.003)	0.574
3-ureidopropionate	Hypertension	15	0.993 (0.984 to 1.002)	0.158	0.998 (0.994 to 1.003)	0.441	0.998 (0.995 to 1.001)	0.272		
pyridoxate	Hypertension	25	0.999 (0.994 to 1.004)	0.640	0.999 (0.996 to 1.002)	0.672	1.000 (0.997 to 1.002)	0.843		
androsterone sulfate	Hypertension	34	0.999 (0.998 to 1.001)	0.429	1.001 (1.000 to 1.002)	0.229	1.001 (1.000 to 1.002)	0.248		

Exposure	Outcome	nSNP	MR Egger		Weighted median		Inverse variance weighted		MR_PRESSO	
			OR (95% CI)	pval	OR (95% CI)	pval	OR (95% CI)	pval	OR (95% CI)	pval
2-methylbutyrylglycine	Hypertension	20	1.001 (0.994 to 1.008)	0.780	1.000 (0.996 to 1.003)	0.813	1.001 (0.998 to 1.003)	0.513		
propionylglycine	Hypertension	25	0.988 (0.975 to 1.001)	0.077	0.999 (0.995 to 1.002)	0.475	1.003 (0.999 to 1.008)	0.166	1.002 (0.999 to 1.005)	0.253
5-hydroxyhexanoate	Hypertension	13	0.998 (0.987 to 1.008)	0.695	0.997 (0.994 to 1.001)	0.160	1.000 (0.996 to 1.003)	0.792	0.999 (0.995 to 1.002)	0.427
3-hydroxysebacate	Hypertension	28	0.999 (0.996 to 1.003)	0.734	0.999 (0.997 to 1.001)	0.300	0.998 (0.997 to 1.000)	0.028		
3-(4-hydroxyphenyl)lactate	Hypertension	17	0.999 (0.992 to 1.007)	0.874	1.002 (0.999 to 1.006)	0.145	1.002 (0.999 to 1.005)	0.285	1.001 (0.998 to 1.003)	0.597
acetylcarnitine	Hypertension	27	1.005 (1.000 to 1.009)	0.047	1.002 (0.999 to 1.005)	0.284	1.001 (0.998 to 1.003)	0.569		

Exposure	Outcome	nSNP	MR Egger		Weighted median		Inverse variance weighted		MR_PRESSO	
			OR (95% CI)	pval	OR (95% CI)	pval	OR (95% CI)	pval	OR (95% CI)	pval
trans-4-hydroxyproline	Hypertension	17	0.994 (0.986 to 1.002)	0.154	0.994 (0.991 to 0.998)	0.006	0.996 (0.992 to 0.999)	0.008		
hexanoylcarnitine	Hypertension	28	0.996 (0.991 to 1.000)	0.061	1.002 (1.000 to 1.004)	0.019	1.001 (1.000 to 1.003)	0.115	1.001 (1.000 to 1.003)	0.121
glycochenodeoxycholate	Hypertension	14	0.999 (0.986 to 1.012)	0.876	1.001 (0.996 to 1.005)	0.796	1.001 (0.996 to 1.007)	0.646	1.000 (0.995 to 1.004)	0.890
4-imidazoleacetate	Hypertension	18	1.000 (0.992 to 1.008)	0.989	1.001 (0.998 to 1.005)	0.526	1.000 (0.997 to 1.004)	0.764	1.001 (0.999 to 1.004)	0.302
1-methylimidazoleacetate	Hypertension	21	1.004 (0.999 to 1.009)	0.118	1.002 (0.999 to 1.005)	0.134	1.000 (0.997 to 1.002)	0.766		
N-acetylneuraminate	Hypertension	16	0.995 (0.985 to 1.006)	0.399	0.998 (0.995 to 1.002)	0.374	1.001 (0.996 to 1.005)	0.821	0.999 (0.995 to 1.002)	0.357

Exposure	Outcome	nSNP	MR Egger		Weighted median		Inverse variance weighted		MR_PRESSO	
			OR (95% CI)	pval	OR (95% CI)	pval	OR (95% CI)	pval	OR (95% CI)	pval
dodecanedioate	Hypertension	30	1.001 (0.997 to 1.005)	0.596	0.999 (0.997 to 1.001)	0.250	0.999 (0.997 to 1.000)	0.157		
N-acetyltyrosine	Hypertension	33	1.002 (0.998 to 1.006)	0.360	1.002 (1.001 to 1.004)	5.92e-05	1.001 (1.000 to 1.003)	0.016	1.002 (1.001 to 1.003)	0.003
1,3-dimethylurate	Hypertension	22	1.002 (0.995 to 1.009)	0.521	0.998 (0.995 to 1.001)	0.172	0.995 (0.992 to 0.999)	0.008	0.997 (0.994 to 1.000)	0.033
trigonelline (N'-methylnicotinate)	Hypertension	16	1.001 (0.994 to 1.007)	0.846	1.000 (0.997 to 1.004)	0.846	1.000 (0.997 to 1.003)	0.930		
indolepropionate	Hypertension	21	1.002 (0.999 to 1.005)	0.124	1.002 (1.000 to 1.004)	0.064	1.001 (0.999 to 1.003)	0.178	1.001 (1.000 to 1.003)	0.113
butyrylcarnitine	Hypertension	60	0.999 (0.998 to 1.001)	0.288	1.000 (0.999 to 1.001)	0.461	1.000 (0.999 to 1.001)	0.606	1.000 (1.000 to 1.001)	0.505

Exposure	Outcome	nSNP	MR Egger		Weighted median		Inverse variance weighted		MR_PRESSO	
			OR (95% CI)	pval	OR (95% CI)	pval	OR (95% CI)	pval	OR (95% CI)	pval
myristoleate (14:1n5)	Hypertension	18	0.997 (0.987 to 1.007)	0.575	0.994 (0.990 to 0.999)	0.009	0.994 (0.989 to 0.999)	0.012	0.993 (0.989 to 0.996)	9.87e-04
dehydroisoandrosterone sulfate (DHEA-S)	Hypertension	22	1.005 (0.998 to 1.011)	0.167	1.000 (0.996 to 1.004)	0.962	1.001 (0.998 to 1.004)	0.519	1.002 (0.998 to 1.005)	0.337
3-methylxanthine	Hypertension	13	0.998 (0.992 to 1.003)	0.439	1.000 (0.996 to 1.003)	0.891	1.001 (0.998 to 1.004)	0.503		
guanidinosuccinate	Hypertension	26	0.999 (0.995 to 1.002)	0.469	0.999 (0.997 to 1.001)	0.186	0.999 (0.997 to 1.001)	0.420	0.999 (0.998 to 1.001)	0.361
caproate (6:0)	Hypertension	24	1.001 (0.999 to 1.003)	0.353	1.000 (0.999 to 1.002)	0.454	1.000 (0.999 to 1.001)	0.663		
caprylate (8:0)	Hypertension	32	1.000 (0.995 to 1.004)	0.896	1.001 (0.998 to 1.003)	0.610	1.000 (0.998 to 1.002)	0.822		

Exposure	Outcome	nSNP	MR Egger		Weighted median		Inverse variance weighted		MR_PRESSO	
			OR (95% CI)	pval	OR (95% CI)	pval	OR (95% CI)	pval	OR (95% CI)	pval
10-undecenoate (11:1n1)	Hypertension	28	0.999 (0.997 to 1.002)	0.668	1.001 (0.999 to 1.002)	0.471	1.000 (0.999 to 1.002)	0.583		
docosapentaenoate (n3 DPA; 22:5n3)	Hypertension	43	1.000 (0.999 to 1.001)	0.852	1.000 (0.999 to 1.000)	0.221	1.000 (0.999 to 1.000)	0.803		
phenol sulfate	Hypertension	13	0.999 (0.994 to 1.004)	0.717	0.998 (0.995 to 1.002)	0.344	0.998 (0.995 to 1.001)	0.138		
pregnen-diol disulfate	Hypertension	23	1.001 (0.998 to 1.003)	0.579	0.999 (0.998 to 1.001)	0.333	0.999 (0.998 to 1.001)	0.310		
X - 11261	Hypertension	30	0.999 (0.993 to 1.004)	0.644	0.999 (0.997 to 1.002)	0.488	1.000 (0.998 to 1.002)	0.788		
bilirubin (E,E)	Hypertension	21	0.999 (0.997 to 1.002)	0.612	1.000 (0.998 to 1.001)	0.726	1.000 (0.999 to 1.001)	0.982		

Exposure	Outcome	nSNP	MR Egger		Weighted median		Inverse variance weighted		MR_PRESSO	
			OR (95% CI)	pval	OR (95% CI)	pval	OR (95% CI)	pval	OR (95% CI)	pval
glycocholenate sulfate	Hypertension	44	0.999 (0.996 to 1.003)	0.705	0.999 (0.998 to 1.001)	0.372	1.000 (0.999 to 1.001)	0.842		
pregn steroid monosulfate	Hypertension	29	1.001 (0.995 to 1.007)	0.798	1.000 (0.997 to 1.002)	0.742	0.999 (0.997 to 1.001)	0.293		
glycolithocholate sulfate	Hypertension	20	1.000 (0.998 to 1.001)	0.665	0.999 (0.997 to 1.000)	0.086	1.000 (0.999 to 1.001)	0.533		
X - 11381	Hypertension	36	0.999 (0.993 to 1.006)	0.882	1.002 (0.999 to 1.005)	0.149	1.004 (1.001 to 1.007)	0.005	1.002 (1.000 to 1.004)	0.050
taurocholenate sulfate	Hypertension	33	1.000 (0.996 to 1.005)	0.912	1.000 (0.997 to 1.002)	0.692	0.999 (0.997 to 1.001)	0.193		
2-arachidonoyl-GPE (20:4)	Hypertension	53	1.002 (0.998 to 1.006)	0.305	0.998 (0.997 to 1.000)	0.024	0.999 (0.998 to 1.000)	0.175		

Exposure	Outcome	nSNP	MR Egger		Weighted median		Inverse variance weighted		MR_PRESSO	
			OR (95% CI)	pval	OR (95% CI)	pval	OR (95% CI)	pval	OR (95% CI)	pval
andro steroid monosulfate (1)	Hypertension	23	1.001 (0.998 to 1.005)	0.477	0.999 (0.997 to 1.001)	0.320	0.999 (0.997 to 1.001)	0.183		
HWESASXX	Hypertension	20	1.005 (1.000 to 1.010)	0.065	1.001 (0.998 to 1.004)	0.563	1.000 (0.997 to 1.003)	0.943		
X - 11540	Hypertension	22	1.000 (0.996 to 1.004)	0.908	1.001 (1.000 to 1.003)	0.154	1.001 (0.999 to 1.003)	0.360	1.001 (1.000 to 1.002)	0.031
adrenate (22:4n6)	Hypertension	38	1.005 (0.998 to 1.011)	0.155	0.997 (0.995 to 1.000)	0.018	0.999 (0.997 to 1.000)	0.117		
X - 11787	Hypertension	30	0.999 (0.993 to 1.006)	0.807	0.997 (0.995 to 0.998)	8.07e-05	0.999 (0.997 to 1.000)	0.143	0.998 (0.996 to 1.000)	0.021
X - 11795	Hypertension	19	1.000 (0.993 to 1.006)	0.937	1.000 (0.997 to 1.004)	0.805	1.000 (0.997 to 1.003)	0.997		

Exposure	Outcome	nSNP	MR Egger		Weighted median		Inverse variance weighted		MR_PRESSO	
			OR (95% CI)	pval	OR (95% CI)	pval	OR (95% CI)	pval	OR (95% CI)	pval
1-arachidonoyl-GPC (20:4n6)	Hypertension	45	0.999 (0.997 to 1.000)	0.165	0.999 (0.999 to 1.000)	0.018	0.999 (0.999 to 1.000)	0.017		
1-palmitoleoyl-GPC (16:1)	Hypertension	12	1.003 (0.991 to 1.015)	0.673	1.001 (0.996 to 1.006)	0.719	0.996 (0.991 to 1.002)	0.175	0.999 (0.996 to 1.003)	0.682
gamma-glutamylthreonine	Hypertension	17	0.993 (0.981 to 1.006)	0.329	1.001 (0.997 to 1.004)	0.706	1.003 (0.998 to 1.009)	0.185	1.000 (0.998 to 1.003)	0.989
2-arachidonoyl-GPC (20:4)	Hypertension	49	0.998 (0.996 to 1.000)	0.108	0.999 (0.998 to 1.000)	0.017	0.999 (0.999 to 1.000)	0.017		
gamma-glutamylphenylalanine	Hypertension	8	0.997 (0.990 to 1.005)	0.552	0.999 (0.994 to 1.004)	0.697	1.000 (0.997 to 1.003)	0.961		
isobutyrylcarnitine	Hypertension	29	1.002 (0.998 to 1.007)	0.303	1.001 (0.998 to 1.003)	0.608	1.002 (1.000 to 1.004)	0.088	1.002 (1.000 to 1.004)	0.118

Exposure	Outcome	nSNP	MR Egger		Weighted median		Inverse variance weighted		MR_PRESSO	
			OR (95% CI)	pval	OR (95% CI)	pval	OR (95% CI)	pval	OR (95% CI)	pval
pseudouridine	Hypertension	25	1.001 (0.994 to 1.008)	0.784	1.000 (0.997 to 1.003)	0.940	1.000 (0.997 to 1.002)	0.790		
palmitoleate (16:1n7)	Hypertension	25	1.000 (0.998 to 1.002)	0.699	1.000 (0.999 to 1.001)	0.880	0.999 (0.998 to 1.001)	0.361	1.000 (0.999 to 1.000)	0.320
1-dihomo-linolenoyl-GPC (20:3n3 or 6)	Hypertension	43	1.002 (0.996 to 1.008)	0.497	1.002 (1.001 to 1.004)	0.009	1.002 (1.001 to 1.003)	0.003		
1-docosahexaenoyl-GPC (22:6)	Hypertension	21	0.999 (0.994 to 1.004)	0.674	0.996 (0.993 to 0.999)	0.015	0.999 (0.997 to 1.001)	0.342		
1-dihomo-linoleoyl-GPC (20:2)	Hypertension	44	1.002 (0.999 to 1.005)	0.163	1.001 (1.000 to 1.002)	0.012	1.001 (1.000 to 1.002)	0.026		
gamma-glutamyl-epsilon-lysine	Hypertension	25	1.000 (0.994 to 1.007)	0.895	1.002 (0.999 to 1.006)	0.175	1.002 (0.999 to 1.006)	0.156	1.002 (0.999 to 1.004)	0.162

Exposure	Outcome	nSNP	MR Egger		Weighted median		Inverse variance weighted		MR_PRESSO	
			OR (95% CI)	pval	OR (95% CI)	pval	OR (95% CI)	pval	OR (95% CI)	pval
octanoylcarnitine	Hypertension	24	1.000 (0.995 to 1.005)	0.917	1.002 (1.000 to 1.004)	0.056	1.001 (0.999 to 1.003)	0.204		
decanoylcarnitine	Hypertension	25	0.999 (0.992 to 1.005)	0.663	1.003 (1.001 to 1.005)	0.013	1.002 (1.000 to 1.004)	0.047		
N-acetylasparagine	Hypertension	23	0.996 (0.994 to 0.999)	0.011	0.999 (0.996 to 1.001)	0.173	1.001 (0.999 to 1.003)	0.251	1.005 (1.003 to 1.008)	2.57e-04
N-acetylglutamine	Hypertension	27	1.000 (0.998 to 1.003)	0.795	1.003 (1.002 to 1.004)	8.02e-07	1.002 (1.001 to 1.003)	1.97e-04	1.002 (1.001 to 1.003)	1.22e-05
N-acetylhistidine	Hypertension	34	1.009 (1.005 to 1.013)	2.36e-04	1.005 (1.003 to 1.007)	1.23e-09	1.001 (0.999 to 1.003)	0.375	1.003 (1.001 to 1.005)	0.001
gamma-glutamylglycine	Hypertension	26	1.000 (0.997 to 1.004)	0.768	1.000 (0.998 to 1.001)	0.554	0.999 (0.997 to 1.001)	0.404	1.000 (0.999 to 1.001)	0.747

Exposure	Outcome	nSNP	MR Egger		Weighted median		Inverse variance weighted		MR_PRESSO	
			OR (95% CI)	pval	OR (95% CI)	pval	OR (95% CI)	pval	OR (95% CI)	pval
N-acetylphenylalanine	Hypertension	25	1.007 (1.004 to 1.009)	8.11e-06	1.004 (1.003 to 1.005)	1e-10	1.003 (1.002 to 1.004)	2.84e-06	1.003 (1.002 to 1.004)	4.98e-06
myristoylcarnitine	Hypertension	34	1.005 (0.992 to 1.018)	0.480	1.003 (0.999 to 1.006)	0.117	1.006 (1.003 to 1.010)	0.001	1.005 (1.001 to 1.008)	0.013
glycylphenylalanine	Hypertension	32	0.989 (0.979 to 1.000)	0.050	0.996 (0.993 to 1.000)	0.027	1.000 (0.996 to 1.004)	0.929	0.999 (0.996 to 1.001)	0.298
1-stearoyl-GPC (18:0)	Hypertension	20	0.999 (0.997 to 1.001)	0.217	0.999 (0.998 to 1.001)	0.391	1.000 (0.999 to 1.001)	0.790	1.000 (0.999 to 1.002)	0.438
5-dodecenoate (12:1n7)	Hypertension	13	0.997 (0.985 to 1.009)	0.595	1.001 (0.996 to 1.006)	0.701	0.998 (0.992 to 1.004)	0.453	0.998 (0.994 to 1.003)	0.464
10-nonadecenoate (19:1n9)	Hypertension	9	0.998 (0.996 to 1.000)	0.159	1.000 (0.999 to 1.001)	0.719	1.000 (0.999 to 1.001)	0.858		

Exposure	Outcome	nSNP	MR Egger		Weighted median		Inverse variance weighted		MR_PRESSO	
			OR (95% CI)	pval	OR (95% CI)	pval	OR (95% CI)	pval	OR (95% CI)	pval
epiandrosterone sulfate	Hypertension	24	1.005 (1.001 to 1.010)	0.037	1.004 (1.001 to 1.006)	0.002	1.002 (1.000 to 1.005)	0.020		
1-arachidonoyl-GPI (20:4)	Hypertension	49	1.001 (0.996 to 1.007)	0.655	0.998 (0.996 to 1.000)	0.014	0.999 (0.998 to 1.000)	0.062		
2-docosahexaenoyl-GPE (22:6)	Hypertension	20	1.000 (0.993 to 1.006)	0.909	1.000 (0.997 to 1.003)	0.930	1.000 (0.998 to 1.002)	0.943		
stachydrine	Hypertension	18	0.995 (0.988 to 1.002)	0.196	1.001 (0.998 to 1.005)	0.538	0.999 (0.995 to 1.003)	0.574	1.000 (0.997 to 1.004)	0.793
1-methylxanthine	Hypertension	21	1.000 (0.996 to 1.003)	0.922	1.001 (0.999 to 1.003)	0.373	1.002 (1.001 to 1.004)	0.008		
7-methylxanthine	Hypertension	12	0.998 (0.989 to 1.008)	0.752	0.998 (0.993 to 1.002)	0.309	0.996 (0.992 to 1.000)	0.053	0.997 (0.994 to 1.001)	0.179

Exposure	Outcome	nSNP	MR Egger		Weighted median		Inverse variance weighted		MR_PRESSO	
			OR (95% CI)	pval	OR (95% CI)	pval	OR (95% CI)	pval	OR (95% CI)	pval
1-linolenoylglycerol (18:3)	Hypertension	16	1.009 (1.001 to 1.017)	0.051	1.004 (1.000 to 1.007)	0.038	1.000 (0.996 to 1.005)	0.889	1.003 (1.000 to 1.005)	0.078
1-methylurate	Hypertension	23	0.999 (0.997 to 1.002)	0.676	1.000 (0.998 to 1.002)	0.867	1.000 (0.999 to 1.002)	0.573	0.999 (0.998 to 1.001)	0.281
choline phosphate	Hypertension	19	0.996 (0.990 to 1.003)	0.257	0.999 (0.996 to 1.002)	0.549	0.999 (0.995 to 1.002)	0.413	1.000 (0.997 to 1.002)	0.949
1,7-dimethylurate	Hypertension	27	0.999 (0.995 to 1.002)	0.490	0.999 (0.996 to 1.002)	0.530	1.000 (0.997 to 1.002)	0.739		
5-acetylamino-6-formylamino-3-methyluracil	Hypertension	22	0.996 (0.989 to 1.004)	0.348	0.997 (0.995 to 1.000)	0.041	0.998 (0.995 to 1.001)	0.154	0.996 (0.994 to 0.998)	3.61e-04
1,3,7-trimethylurate	Hypertension	21	1.001 (0.994 to 1.009)	0.746	0.999 (0.995 to 1.003)	0.579	1.000 (0.997 to 1.004)	0.983	0.999 (0.996 to 1.002)	0.591

Exposure	Outcome	nSNP	MR Egger		Weighted median		Inverse variance weighted		MR_PRESSO	
			OR (95% CI)	pval	OR (95% CI)	pval	OR (95% CI)	pval	OR (95% CI)	pval
isovalerylcarnitine	Hypertension	28	0.995 (0.979 to 1.012)	0.581	1.006 (1.002 to 1.011)	0.005	1.006 (1.002 to 1.010)	0.006	1.007 (1.002 to 1.011)	0.006
stearoylcarnitine	Hypertension	21	0.994 (0.985 to 1.003)	0.220	0.998 (0.995 to 1.001)	0.281	1.001 (0.997 to 1.005)	0.777	0.998 (0.996 to 1.001)	0.229
1-linoleoyl-GPC (18:2)	Hypertension	36	0.998 (0.992 to 1.004)	0.491	1.002 (1.000 to 1.004)	0.012	1.001 (1.000 to 1.003)	0.035		
5-acetylamino-6-amino-3-methyluracil	Hypertension	22	1.001 (0.998 to 1.004)	0.461	1.000 (0.998 to 1.002)	0.963	1.000 (0.998 to 1.002)	0.942		
1-palmitoyl-GPA (16:0)	Hypertension	18	1.002 (0.998 to 1.006)	0.358	1.002 (0.999 to 1.004)	0.149	1.001 (0.999 to 1.003)	0.379		
gamma-glutamylisoleucine	Hypertension	26	1.001 (0.997 to 1.005)	0.666	1.001 (0.998 to 1.003)	0.639	1.001 (0.999 to 1.003)	0.566		

Exposure	Outcome	nSNP	MR Egger		Weighted median		Inverse variance weighted		MR_PRESSO	
			OR (95% CI)	pval	OR (95% CI)	pval	OR (95% CI)	pval	OR (95% CI)	pval
laurylcarnitine	Hypertension	22	0.996 (0.990 to 1.003)	0.279	1.001 (0.998 to 1.003)	0.695	1.001 (0.999 to 1.004)	0.218		
7-methylguanine	Hypertension	22	1.001 (0.995 to 1.006)	0.832	1.001 (0.998 to 1.004)	0.663	1.002 (0.999 to 1.004)	0.170		
phenylacetylglutamine	Hypertension	13	1.001 (0.997 to 1.004)	0.750	1.000 (0.998 to 1.002)	0.938	1.000 (0.998 to 1.003)	0.745	1.001 (0.999 to 1.003)	0.434
pro-hydroxy-pro	Hypertension	23	0.998 (0.993 to 1.003)	0.417	0.999 (0.996 to 1.002)	0.472	0.999 (0.997 to 1.001)	0.514		
5-methyluridine (ribothymidine)	Hypertension	23	1.002 (0.998 to 1.006)	0.298	1.002 (1.000 to 1.004)	0.080	1.001 (0.999 to 1.003)	0.205		
N2,N2-dimethylguanosine	Hypertension	10	1.007 (0.994 to 1.020)	0.311	0.997 (0.992 to 1.002)	0.275	1.000 (0.996 to 1.004)	0.970		

Exposure	Outcome	nSNP	MR Egger		Weighted median		Inverse variance weighted		MR_PRESSO	
			OR (95% CI)	pval	OR (95% CI)	pval	OR (95% CI)	pval	OR (95% CI)	pval
1-docosahexaenoylglycerol (22:6)	Hypertension	17	0.998 (0.992 to 1.004)	0.455	1.001 (0.998 to 1.004)	0.378	1.001 (0.998 to 1.003)	0.722	1.000 (0.998 to 1.002)	0.675
N6-carbamoylthreonyladenosine	Hypertension	15	1.004 (0.992 to 1.016)	0.513	1.001 (0.997 to 1.005)	0.653	1.000 (0.994 to 1.005)	0.894	1.001 (0.998 to 1.004)	0.593
oleoylcarnitine	Hypertension	27	1.003 (0.999 to 1.008)	0.199	0.999 (0.996 to 1.002)	0.472	1.001 (0.998 to 1.004)	0.476	0.999 (0.998 to 1.001)	0.588
orotidine	Hypertension	25	0.998 (0.995 to 1.000)	0.112	0.998 (0.996 to 1.000)	0.100	0.999 (0.997 to 1.000)	0.132		
1-arachidonoyl-GPE (20:4n6)	Hypertension	75	1.000 (0.999 to 1.001)	0.618	1.000 (0.999 to 1.001)	0.577	1.000 (1.000 to 1.001)	0.775		
2-palmitoyl-GPC (16:0)	Hypertension	25	0.999 (0.993 to 1.006)	0.848	0.999 (0.996 to 1.002)	0.411	0.999 (0.997 to 1.002)	0.714	1.000 (0.998 to 1.002)	0.872

Exposure	Outcome	nSNP	MR Egger		Weighted median		Inverse variance weighted		MR_PRESSO	
			OR (95% CI)	pval	OR (95% CI)	pval	OR (95% CI)	pval	OR (95% CI)	pval
2-linoleoyl-GPC (18:2)	Hypertension	15	0.994 (0.984 to 1.004)	0.257	0.999 (0.995 to 1.003)	0.505	0.999 (0.996 to 1.003)	0.651		
tiglylcarnitine	Hypertension	25	1.000 (0.994 to 1.006)	0.909	1.003 (1.001 to 1.006)	0.021	1.003 (1.001 to 1.006)	0.010		
hexanoylglycine	Hypertension	26	1.000 (0.996 to 1.003)	0.793	1.000 (0.999 to 1.002)	0.737	1.001 (0.999 to 1.003)	0.313	1.000 (0.999 to 1.002)	0.593
2-methylmalonyl carnitine	Hypertension	16	1.002 (0.993 to 1.011)	0.663	1.000 (0.996 to 1.004)	0.860	1.000 (0.996 to 1.003)	0.812	1.001 (0.998 to 1.004)	0.514
1-myristoylglycerol (14:0)	Hypertension	25	1.002 (0.995 to 1.010)	0.522	1.001 (0.998 to 1.004)	0.491	1.001 (0.998 to 1.004)	0.648	1.000 (0.998 to 1.003)	0.691
1-oleoyl-GPE (18:1)	Hypertension	40	0.998 (0.994 to 1.001)	0.237	1.002 (0.999 to 1.004)	0.189	1.001 (0.999 to 1.002)	0.456		

Exposure	Outcome	nSNP	MR Egger		Weighted median		Inverse variance weighted		MR_PRESSO	
			OR (95% CI)	pval	OR (95% CI)	pval	OR (95% CI)	pval	OR (95% CI)	pval
1-palmitoyl-GPE (16:0)	Hypertension	44	0.997 (0.994 to 0.999)	0.024	1.001 (0.999 to 1.002)	0.510	1.000 (0.999 to 1.001)	0.721		
cysteinylglycine	Hypertension	20	0.993 (0.977 to 1.010)	0.430	0.997 (0.994 to 1.000)	0.084	1.003 (0.997 to 1.010)	0.350	0.999 (0.996 to 1.001)	0.347
tetradecanedioate	Hypertension	55	1.000 (0.999 to 1.001)	0.574	1.000 (0.999 to 1.001)	0.853	1.001 (1.000 to 1.001)	0.076		
2-hydroxypalmitate	Hypertension	19	0.999 (0.993 to 1.005)	0.828	1.001 (0.998 to 1.004)	0.614	1.001 (0.999 to 1.003)	0.342		
hexadecanedioate	Hypertension	35	1.001 (0.997 to 1.006)	0.555	1.000 (0.999 to 1.002)	0.631	1.000 (0.999 to 1.001)	0.719		
dihomo-linolenate (20:3n3 or n6)	Hypertension	52	0.999 (0.994 to 1.004)	0.675	1.002 (1.001 to 1.004)	0.010	1.001 (1.000 to 1.002)	0.147		

Exposure	Outcome	nSNP	MR Egger		Weighted median		Inverse variance weighted		MR_PRESSO	
			OR (95% CI)	pval	OR (95% CI)	pval	OR (95% CI)	pval	OR (95% CI)	pval
4-vinylphenol sulfate	Hypertension	23	1.001 (0.996 to 1.006)	0.736	1.001 (0.998 to 1.004)	0.509	1.000 (0.998 to 1.002)	0.930		
p-cresol sulfate	Hypertension	14	1.001 (0.989 to 1.013)	0.932	1.001 (0.996 to 1.006)	0.738	0.999 (0.993 to 1.005)	0.656	1.001 (0.997 to 1.005)	0.596
2-linoleoyl-GPE (18:2)	Hypertension	52	1.001 (0.996 to 1.006)	0.723	1.002 (1.000 to 1.003)	0.017	1.001 (1.000 to 1.002)	0.045		
1-linoleoyl-GPI (18:2)	Hypertension	47	0.991 (0.984 to 0.997)	0.007	1.002 (1.000 to 1.003)	0.017	1.000 (0.999 to 1.002)	0.690		
1-linoleoyl-GPE (18:2)	Hypertension	54	1.003 (1.000 to 1.006)	0.080	1.001 (1.000 to 1.002)	0.016	1.001 (1.000 to 1.001)	0.079		
1-oleoyl-GPI (18:1)	Hypertension	22	0.995 (0.987 to 1.004)	0.303	1.000 (0.997 to 1.003)	0.756	1.000 (0.998 to 1.003)	0.776	1.001 (0.999 to 1.003)	0.361

Exposure	Outcome	nSNP	MR Egger		Weighted median		Inverse variance weighted		MR_PRESSO	
			OR (95% CI)	pval	OR (95% CI)	pval	OR (95% CI)	pval	OR (95% CI)	pval
gamma-glutamylglutamate	Hypertension	34	1.000 (0.999 to 1.002)	0.567	1.000 (0.999 to 1.001)	0.898	1.000 (0.999 to 1.001)	0.668		
2-hydroxy-3-methylvalerate	Hypertension	24	0.996 (0.991 to 1.002)	0.217	0.998 (0.994 to 1.001)	0.154	0.998 (0.996 to 1.001)	0.209		
deoxycarnitine	Hypertension	27	1.002 (0.996 to 1.009)	0.530	1.002 (0.999 to 1.005)	0.151	0.999 (0.997 to 1.002)	0.676		
3-methyladipate	Hypertension	17	0.989 (0.983 to 0.995)	0.002	1.000 (0.995 to 1.006)	0.972	1.007 (1.002 to 1.012)	0.005	1.008 (1.004 to 1.013)	0.004
N6-acetyllysine	Hypertension	16	1.003 (0.997 to 1.009)	0.369	0.998 (0.995 to 1.002)	0.347	0.999 (0.997 to 1.002)	0.529		
octadecanedioate	Hypertension	17	0.999 (0.990 to 1.009)	0.914	0.999 (0.996 to 1.003)	0.653	1.000 (0.996 to 1.005)	0.873	1.000 (0.996 to 1.004)	0.988

Exposure	Outcome	nSNP	MR Egger		Weighted median		Inverse variance weighted		MR_PRESSO	
			OR (95% CI)	pval	OR (95% CI)	pval	OR (95% CI)	pval	OR (95% CI)	pval
7-alpha-hydroxy-3-oxo-4-cholestenoate (7-Hoca)	Hypertension	15	0.998 (0.991 to 1.004)	0.476	0.997 (0.993 to 1.001)	0.188	0.999 (0.996 to 1.003)	0.655		
dimethylarginine (SDMA + ADMA)	Hypertension	18	0.994 (0.989 to 1.000)	0.074	1.000 (0.998 to 1.003)	0.756	1.000 (0.998 to 1.003)	0.743	1.000 (0.998 to 1.001)	0.663
o-cresol sulfate	Hypertension	14	0.999 (0.992 to 1.007)	0.866	0.999 (0.995 to 1.002)	0.394	0.998 (0.995 to 1.001)	0.128		
taurolithocholate 3-sulfate	Hypertension	16	0.995 (0.984 to 1.007)	0.436	0.998 (0.994 to 1.002)	0.262	1.002 (0.997 to 1.007)	0.529	0.998 (0.996 to 1.001)	0.179
succinylcarnitine	Hypertension	22	1.002 (0.997 to 1.008)	0.422	0.997 (0.994 to 1.000)	0.031	0.997 (0.995 to 0.999)	0.015	0.996 (0.994 to 0.999)	0.005
gamma-glutamylalanine	Hypertension	19	0.990 (0.976 to 1.004)	0.182	1.001 (0.997 to 1.004)	0.796	1.003 (0.998 to 1.009)	0.248	1.001 (0.998 to 1.004)	0.406

Exposure	Outcome	nSNP	MR Egger		Weighted median		Inverse variance weighted		MR_PRESSO	
			OR (95% CI)	pval	OR (95% CI)	pval	OR (95% CI)	pval	OR (95% CI)	pval
N-acetylserine	Hypertension	18	1.006 (0.994 to 1.018)	0.326	1.002 (0.999 to 1.005)	0.201	1.002 (1.000 to 1.004)	0.093		
gamma-glutamyl-2-aminobutyrate	Hypertension	14	0.999 (0.992 to 1.006)	0.754	1.003 (0.999 to 1.007)	0.141	1.004 (1.000 to 1.007)	0.058	1.003 (1.000 to 1.006)	0.104
tryptophan betaine	Hypertension	13	0.998 (0.991 to 1.005)	0.522	0.999 (0.995 to 1.003)	0.604	0.998 (0.995 to 1.001)	0.197		
cyclo(leu-pro)	Hypertension	20	1.007 (0.996 to 1.019)	0.231	1.000 (0.997 to 1.004)	0.908	1.002 (0.997 to 1.006)	0.445	1.001 (0.999 to 1.004)	0.360
5alpha-androstan-3alpha,17beta-diol monosulfate (1)	Hypertension	24	1.007 (1.004 to 1.010)	2.13e-04	1.006 (1.003 to 1.008)	7.12e-06	1.003 (1.002 to 1.005)	4.03e-04		
5alpha-androstan-3beta,17beta-diol disulfate	Hypertension	29	0.998 (0.995 to 1.000)	0.076	1.000 (0.999 to 1.001)	0.768	0.999 (0.998 to 1.001)	0.379	1.001 (1.000 to 1.002)	0.319

Exposure	Outcome	nSNP	MR Egger		Weighted median		Inverse variance weighted		MR_PRESSO	
			OR (95% CI)	pval	OR (95% CI)	pval	OR (95% CI)	pval	OR (95% CI)	pval
5alpha-pregnan-3beta,20alpha-diol disulfate	Hypertension	21	0.993 (0.986 to 1.001)	0.096	0.997 (0.994 to 1.000)	0.044	0.998 (0.996 to 1.000)	0.096		
5alpha-pregnan-3beta,20alpha-diol monosulfate (2)	Hypertension	10	1.003 (0.993 to 1.013)	0.591	0.997 (0.993 to 1.001)	0.171	0.999 (0.994 to 1.004)	0.738	1.000 (0.996 to 1.004)	0.969
4-androsten-3beta,17beta-diol disulfate (1)	Hypertension	25	1.001 (0.996 to 1.005)	0.814	0.999 (0.997 to 1.001)	0.333	1.001 (0.999 to 1.003)	0.412	1.001 (0.999 to 1.003)	0.563
4-androsten-3beta,17beta-diol disulfate (2)	Hypertension	20	1.001 (0.999 to 1.003)	0.501	1.001 (0.999 to 1.002)	0.270	1.000 (0.999 to 1.001)	0.919		
4-androsten-3alpha,17alpha-diol monosulfate (2)	Hypertension	21	1.001 (0.992 to 1.011)	0.773	1.000 (0.997 to 1.004)	0.819	1.000 (0.997 to 1.003)	0.946	1.000 (0.998 to 1.002)	0.843
4-androsten-3alpha,17alpha-diol monosulfate (3)	Hypertension	41	1.003 (1.001 to 1.005)	0.012	1.002 (1.000 to 1.003)	0.024	1.001 (0.999 to 1.002)	0.272	1.001 (1.000 to 1.002)	0.094

Exposure	Outcome	nSNP	MR Egger		Weighted median		Inverse variance weighted		MR_PRESSO	
			OR (95% CI)	pval	OR (95% CI)	pval	OR (95% CI)	pval	OR (95% CI)	pval
4-androsten-3beta,17beta-diol monosulfate (2)	Hypertension	23	1.005 (0.992 to 1.019)	0.462	1.001 (0.998 to 1.005)	0.472	0.998 (0.993 to 1.002)	0.359	1.000 (0.997 to 1.003)	0.941
4-androsten-3beta,17beta-diol monosulfate (1)	Hypertension	18	1.008 (1.001 to 1.015)	0.038	1.002 (0.998 to 1.007)	0.254	1.005 (1.001 to 1.008)	0.007	1.006 (1.003 to 1.009)	0.002
1-docosapentaenoyl-GPC (22:5n3)	Hypertension	43	1.005 (0.999 to 1.011)	0.131	0.998 (0.996 to 1.000)	0.018	0.999 (0.998 to 1.000)	0.160		
1-pentadecanoyl-GPC (15:0)	Hypertension	15	1.004 (0.995 to 1.013)	0.459	1.004 (1.000 to 1.008)	0.052	1.005 (1.001 to 1.009)	0.005		
N-methylproline	Hypertension	15	1.002 (0.995 to 1.009)	0.617	1.001 (0.997 to 1.004)	0.764	1.001 (0.998 to 1.003)	0.531		
N-acetyl-beta-alanine	Hypertension	17	1.000 (0.993 to 1.008)	0.902	1.001 (0.998 to 1.005)	0.446	1.001 (0.998 to 1.003)	0.686		

Exposure	Outcome	nSNP	MR Egger		Weighted median		Inverse variance weighted		MR_PRESSO	
			OR (95% CI)	pval	OR (95% CI)	pval	OR (95% CI)	pval	OR (95% CI)	pval
cysteine sulfinic acid	Hypertension	16	0.996 (0.988 to 1.005)	0.375	0.998 (0.994 to 1.002)	0.329	0.998 (0.993 to 1.003)	0.427	0.998 (0.995 to 1.001)	0.155
ergothioneine	Hypertension	13	0.998 (0.993 to 1.004)	0.567	0.998 (0.994 to 1.002)	0.315	0.997 (0.994 to 1.000)	0.049		
N-acetylputrescine	Hypertension	18	0.998 (0.992 to 1.004)	0.479	1.001 (0.998 to 1.003)	0.557	0.999 (0.997 to 1.002)	0.578	1.000 (0.998 to 1.002)	0.951
palmitoyl sphingomyelin (d18:1/16:0)	Hypertension	11	0.999 (0.993 to 1.005)	0.771	1.003 (0.999 to 1.008)	0.180	1.003 (1.000 to 1.007)	0.056		
sphingomyelin (d18:1/18:1, d18:2/18:0)	Hypertension	46	1.005 (1.000 to 1.010)	0.071	0.998 (0.996 to 1.000)	0.030	0.999 (0.998 to 1.001)	0.309		
13-HODE + 9-HODE	Hypertension	40	1.002 (0.998 to 1.007)	0.261	1.000 (0.998 to 1.003)	0.945	0.999 (0.997 to 1.001)	0.539	1.000 (0.998 to 1.002)	0.909

Exposure	Outcome	nSNP	MR Egger		Weighted median		Inverse variance weighted		MR_PRESSO	
			OR (95% CI)	pval	OR (95% CI)	pval	OR (95% CI)	pval	OR (95% CI)	pval
oleoyl ethanolamide	Hypertension	19	0.996 (0.990 to 1.002)	0.240	0.998 (0.995 to 1.002)	0.311	0.998 (0.996 to 1.001)	0.169		
S-methylmethionine	Hypertension	19	0.998 (0.995 to 1.000)	0.039	0.999 (0.997 to 1.001)	0.415	0.999 (0.998 to 1.001)	0.229		
phenylalanylphenylalanine	Hypertension	18	0.997 (0.993 to 1.001)	0.194	0.999 (0.996 to 1.002)	0.654	0.999 (0.997 to 1.001)	0.337		
16a-hydroxy DHEA 3-sulfate	Hypertension	28	0.994 (0.992 to 0.997)	2.45e-04	0.995 (0.993 to 0.997)	1.35e-06	0.997 (0.995 to 0.999)	6.64e-04		
pregnenolone sulfate	Hypertension	29	1.000 (0.998 to 1.001)	0.948	1.001 (0.999 to 1.002)	0.290	1.001 (1.000 to 1.002)	0.068		
cis-4-decenoyl carnitine	Hypertension	33	0.999 (0.993 to 1.004)	0.645	1.003 (1.001 to 1.005)	0.007	1.002 (1.000 to 1.004)	0.024	1.002 (1.001 to 1.004)	0.010

Exposure	Outcome	nSNP	MR Egger		Weighted median		Inverse variance weighted		MR_PRESSO	
			OR (95% CI)	pval	OR (95% CI)	pval	OR (95% CI)	pval	OR (95% CI)	pval
17-methylstearate	Hypertension	18	0.997 (0.988 to 1.005)	0.411	0.998 (0.995 to 1.002)	0.440	0.999 (0.996 to 1.003)	0.666		
cinnamoylglycine	Hypertension	33	0.999 (0.996 to 1.002)	0.507	1.000 (0.998 to 1.001)	0.521	1.000 (0.998 to 1.002)	0.925	1.000 (0.999 to 1.001)	0.821
3-methylglutaconate	Hypertension	26	0.992 (0.987 to 0.997)	0.008	0.998 (0.995 to 1.002)	0.337	0.998 (0.995 to 1.000)	0.086	1.001 (1.000 to 1.003)	0.106
15-methylpalmitate	Hypertension	11	1.004 (0.989 to 1.019)	0.636	0.998 (0.993 to 1.004)	0.540	1.000 (0.994 to 1.006)	0.983	1.002 (0.996 to 1.007)	0.561
2-stearoyl-GPI (18:0)	Hypertension	28	1.004 (0.998 to 1.010)	0.183	1.000 (0.997 to 1.002)	0.796	1.000 (0.998 to 1.002)	0.896		
glycoursodeoxycholate	Hypertension	8	0.997 (0.980 to 1.015)	0.783	1.002 (0.997 to 1.008)	0.426	1.003 (0.997 to 1.009)	0.347	1.004 (1.000 to 1.009)	0.118

Exposure	Outcome	nSNP	MR Egger		Weighted median		Inverse variance weighted		MR_PRESSO	
			OR (95% CI)	pval	OR (95% CI)	pval	OR (95% CI)	pval	OR (95% CI)	pval
S-methylcysteine	Hypertension	18	1.000 (0.995 to 1.005)	0.923	0.999 (0.996 to 1.002)	0.353	0.999 (0.997 to 1.002)	0.577		
leucylalanine	Hypertension	31	1.010 (1.004 to 1.016)	0.002	1.005 (1.003 to 1.008)	3.53e-05	1.005 (1.001 to 1.009)	0.006	1.006 (1.004 to 1.008)	4.25e-06
phenylalanylserine	Hypertension	25	1.020 (1.006 to 1.034)	0.011	1.010 (1.008 to 1.012)	4.85e-21	1.003 (0.997 to 1.008)	0.313	1.000 (0.998 to 1.003)	0.746
alpha-glutamyltyrosine	Hypertension	27	1.017 (0.996 to 1.038)	0.126	1.010 (1.008 to 1.013)	1.66e-14	0.999 (0.992 to 1.006)	0.757	1.001 (0.999 to 1.003)	0.198
isoleucylvaline	Hypertension	16	0.993 (0.982 to 1.003)	0.177	0.999 (0.996 to 1.003)	0.711	0.996 (0.992 to 1.000)	0.029	0.999 (0.997 to 1.001)	0.394
X - 17299	Hypertension	20	0.999 (0.989 to 1.008)	0.789	0.998 (0.994 to 1.002)	0.303	0.998 (0.994 to 1.002)	0.253	1.000 (0.997 to 1.003)	0.928

Exposure	Outcome	nSNP	MR Egger		Weighted median		Inverse variance weighted		MR_PRESSO	
			OR (95% CI)	pval	OR (95% CI)	pval	OR (95% CI)	pval	OR (95% CI)	pval
N1-Methyl-2-pyridone-5-carboxamide	Hypertension	12	0.996 (0.990 to 1.003)	0.290	0.999 (0.995 to 1.003)	0.648	0.999 (0.996 to 1.003)	0.767		
hydantoin-5-propionic acid	Hypertension	20	1.001 (0.999 to 1.003)	0.281	1.001 (0.999 to 1.002)	0.267	1.000 (0.999 to 1.002)	0.584	1.001 (1.000 to 1.002)	0.263
dihydroferulic acid	Hypertension	12	1.001 (0.995 to 1.007)	0.742	1.002 (0.998 to 1.005)	0.337	1.000 (0.997 to 1.003)	0.964		
pregnanediol-3-glucuronide	Hypertension	15	0.996 (0.990 to 1.002)	0.189	1.000 (0.997 to 1.004)	0.795	1.001 (0.998 to 1.004)	0.486		
imidazole propionate	Hypertension	14	0.996 (0.990 to 1.002)	0.225	0.999 (0.996 to 1.003)	0.676	1.000 (0.997 to 1.003)	0.895		
prolylproline	Hypertension	14	0.996 (0.990 to 1.003)	0.292	0.992 (0.989 to 0.995)	2.24e-07	0.995 (0.992 to 0.998)	0.001	0.993 (0.990 to 0.996)	0.001

Exposure	Outcome	nSNP	MR Egger		Weighted median		Inverse variance weighted		MR_PRESSO	
			OR (95% CI)	pval	OR (95% CI)	pval	OR (95% CI)	pval	OR (95% CI)	pval
phenylalanyltryptophan	Hypertension	22	1.000 (0.996 to 1.003)	0.816	1.001 (0.998 to 1.004)	0.395	1.001 (0.999 to 1.003)	0.360		
alpha-glutamylglycine	Hypertension	35	1.006 (0.992 to 1.019)	0.403	1.001 (0.998 to 1.004)	0.625	1.001 (0.996 to 1.006)	0.657	0.999 (0.997 to 1.001)	0.172
histidyltryptophan	Hypertension	19	1.000 (0.995 to 1.005)	0.914	0.998 (0.995 to 1.002)	0.353	0.998 (0.996 to 1.001)	0.142		
serylalanine	Hypertension	29	0.994 (0.983 to 1.006)	0.350	0.999 (0.996 to 1.002)	0.720	1.002 (0.996 to 1.007)	0.565	1.000 (0.997 to 1.002)	0.723
valylarginine	Hypertension	32	0.998 (0.981 to 1.016)	0.864	1.002 (0.998 to 1.006)	0.375	1.008 (1.001 to 1.015)	0.017	1.006 (1.001 to 1.011)	0.030
indoleacetylglutamine	Hypertension	21	1.003 (0.997 to 1.009)	0.298	1.004 (1.001 to 1.007)	0.007	1.003 (1.000 to 1.005)	0.030	1.003 (1.001 to 1.005)	0.009

Exposure	Outcome	nSNP	MR Egger		Weighted median		Inverse variance weighted		MR_PRESSO	
			OR (95% CI)	pval	OR (95% CI)	pval	OR (95% CI)	pval	OR (95% CI)	pval
N-palmitoylglycine	Hypertension	29	1.002 (0.990 to 1.015)	0.714	1.001 (0.998 to 1.004)	0.380	1.004 (1.000 to 1.007)	0.040	1.002 (0.999 to 1.005)	0.167
2-aminobutyrate	Hypertension	15	0.997 (0.987 to 1.008)	0.608	1.003 (1.000 to 1.007)	0.069	1.003 (1.000 to 1.007)	0.052	1.002 (1.000 to 1.005)	0.095
1-stearoyl-GPE (18:0)	Hypertension	36	0.996 (0.991 to 1.000)	0.046	1.000 (0.999 to 1.002)	0.635	1.000 (0.999 to 1.001)	0.941		
erythronate	Hypertension	18	1.002 (0.997 to 1.008)	0.400	1.000 (0.997 to 1.004)	0.781	1.003 (1.000 to 1.006)	0.028		
1-palmitoyl-2-linoleoyl-GPC (16:0/18:2)	Hypertension	50	1.004 (1.001 to 1.008)	0.021	1.002 (1.000 to 1.003)	0.014	1.001 (1.000 to 1.002)	0.012		
1-stearoyl-2-oleoyl-GPE (18:0/18:1)	Hypertension	35	0.999 (0.995 to 1.002)	0.540	0.999 (0.997 to 1.001)	0.568	1.000 (0.998 to 1.001)	0.854		

Exposure	Outcome	nSNP	MR Egger		Weighted median		Inverse variance weighted		MR_PRESSO	
			OR (95% CI)	pval	OR (95% CI)	pval	OR (95% CI)	pval	OR (95% CI)	pval
1-palmitoyl-2-linoleoyl-GPE (16:0/18:2)	Hypertension	59	1.003 (1.000 to 1.006)	0.033	1.001 (1.000 to 1.002)	0.009	1.001 (1.000 to 1.001)	0.033		
1-stearoyl-2-arachidonoyl-GPC (18:0/20:4)	Hypertension	50	1.000 (0.998 to 1.001)	0.536	0.999 (0.999 to 1.000)	0.020	0.999 (0.999 to 1.000)	0.015		
sphingomyelin (d18:2/16:0, d18:1/16:1)	Hypertension	11	1.001 (0.988 to 1.015)	0.837	1.001 (0.996 to 1.006)	0.767	1.000 (0.995 to 1.005)	0.936	1.002 (0.997 to 1.006)	0.467
sphingomyelin (d18:1/14:0, d16:1/16:0)	Hypertension	23	0.992 (0.988 to 0.997)	0.004	0.996 (0.993 to 0.998)	0.002	0.997 (0.995 to 1.000)	0.034		
2-hydroxydecanoate	Hypertension	11	0.997 (0.990 to 1.004)	0.407	1.000 (0.996 to 1.004)	0.846	1.001 (0.998 to 1.005)	0.505		
dimethylmalonic acid	Hypertension	17	1.001 (1.000 to 1.003)	0.153	1.001 (1.000 to 1.002)	0.231	1.001 (1.000 to 1.002)	0.049		

Exposure	Outcome	nSNP	MR Egger		Weighted median		Inverse variance weighted		MR_PRESSO	
			OR (95% CI)	pval	OR (95% CI)	pval	OR (95% CI)	pval	OR (95% CI)	pval
N-delta-acetylornithine	Hypertension	32	1.000 (0.998 to 1.003)	0.956	0.998 (0.997 to 0.999)	1.16e-06	0.999 (0.998 to 1.000)	0.014	0.999 (0.998 to 1.000)	0.004
N-acetyl-1-methylhistidine	Hypertension	25	1.005 (1.000 to 1.009)	0.055	1.001 (0.999 to 1.003)	0.278	1.001 (0.999 to 1.002)	0.265	1.001 (1.000 to 1.003)	0.110
N-acetyl-3-methylhistidine	Hypertension	26	0.997 (0.992 to 1.002)	0.286	1.004 (1.003 to 1.006)	5.97e-07	1.003 (1.002 to 1.005)	4.66e-05	1.004 (1.003 to 1.005)	1.54e-06
2-aminooctanoate	Hypertension	29	0.997 (0.993 to 1.002)	0.253	0.997 (0.996 to 0.999)	1.21e-04	0.998 (0.997 to 0.999)	0.002	0.998 (0.997 to 0.999)	4.41e-04
N-acetylcarnosine	Hypertension	26	1.003 (0.998 to 1.008)	0.199	1.002 (0.999 to 1.005)	0.277	1.001 (0.999 to 1.003)	0.576		
5-hydroxyindoleacetate	Hypertension	17	0.999 (0.994 to 1.004)	0.744	0.999 (0.996 to 1.002)	0.578	0.999 (0.997 to 1.001)	0.380		

Exposure	Outcome	nSNP	MR Egger		Weighted median		Inverse variance weighted		MR_PRESSO	
			OR (95% CI)	pval	OR (95% CI)	pval	OR (95% CI)	pval	OR (95% CI)	pval
2-aminoheptanoate	Hypertension	11	0.998 (0.988 to 1.008)	0.694	1.001 (0.996 to 1.005)	0.752	0.998 (0.993 to 1.003)	0.475	1.000 (0.995 to 1.004)	0.893
guanidinoacetate	Hypertension	26	0.998 (0.992 to 1.004)	0.515	1.001 (0.998 to 1.004)	0.399	1.003 (1.000 to 1.005)	0.018		
bilirubin (Z,Z)	Hypertension	19	0.998 (0.994 to 1.002)	0.378	0.999 (0.997 to 1.002)	0.603	0.999 (0.998 to 1.001)	0.544		
gamma-glutamylvaline	Hypertension	22	1.000 (0.994 to 1.005)	0.870	1.000 (0.997 to 1.003)	0.942	1.001 (0.998 to 1.004)	0.410	1.000 (0.998 to 1.003)	0.781
glycerol 3-phosphate	Hypertension	16	1.000 (0.997 to 1.003)	0.994	0.999 (0.997 to 1.000)	0.107	0.999 (0.997 to 1.000)	0.121		
aspartate	Hypertension	20	0.997 (0.990 to 1.004)	0.438	1.001 (0.998 to 1.004)	0.627	1.001 (0.997 to 1.005)	0.491	1.001 (0.999 to 1.003)	0.416

Exposure	Outcome	nSNP	MR Egger		Weighted median		Inverse variance weighted		MR_PRESSO	
			OR (95% CI)	pval	OR (95% CI)	pval	OR (95% CI)	pval	OR (95% CI)	pval
1-eicosenoyl-GPC (20:1)	Hypertension	13	0.992 (0.975 to 1.009)	0.388	1.002 (0.998 to 1.006)	0.425	1.002 (0.997 to 1.008)	0.448	1.000 (0.998 to 1.003)	0.831
1-eicosapentaenoyl-GPC (20:5)	Hypertension	66	0.998 (0.994 to 1.002)	0.367	0.998 (0.996 to 0.999)	0.007	0.999 (0.998 to 1.000)	0.052		
4-acetylphenol sulfate	Hypertension	13	0.995 (0.990 to 1.000)	0.095	1.000 (0.997 to 1.003)	0.948	1.001 (0.997 to 1.004)	0.742	1.003 (1.001 to 1.004)	0.015
1-dihomo-linolenoyl-GPE (20:3n3 or 6)	Hypertension	57	1.000 (0.999 to 1.000)	0.483	1.000 (0.999 to 1.000)	0.309	1.000 (1.000 to 1.001)	0.505		
1-docosahexaenoyl-GPE (22:6)	Hypertension	31	0.996 (0.990 to 1.002)	0.194	0.999 (0.996 to 1.002)	0.427	0.999 (0.997 to 1.001)	0.389		
isovalerate	Hypertension	48	1.002 (0.998 to 1.007)	0.314	1.002 (1.000 to 1.004)	0.095	1.002 (1.000 to 1.004)	0.078	1.002 (1.001 to 1.004)	0.008

Exposure	Outcome	nSNP	MR Egger		Weighted median		Inverse variance weighted		MR_PRESSO	
			OR (95% CI)	pval	OR (95% CI)	pval	OR (95% CI)	pval	OR (95% CI)	pval
glutarylcarnitine (C5)	Hypertension	33	0.997 (0.990 to 1.004)	0.426	0.999 (0.996 to 1.001)	0.326	1.001 (0.998 to 1.004)	0.445	1.000 (0.998 to 1.002)	0.641
docosahexaenoate (DHA; 22:6n3)	Hypertension	15	1.001 (0.994 to 1.008)	0.743	0.998 (0.995 to 1.002)	0.327	0.999 (0.997 to 1.002)	0.457		
palmitoylcarnitine	Hypertension	29	1.001 (0.997 to 1.005)	0.639	0.999 (0.997 to 1.002)	0.622	0.997 (0.994 to 1.000)	0.055	0.998 (0.997 to 1.000)	0.021
1-margaroyl-GPC (17:0)	Hypertension	17	1.000 (0.994 to 1.006)	0.974	1.001 (0.999 to 1.004)	0.366	1.001 (0.999 to 1.003)	0.513		
gamma-glutamylmethionine	Hypertension	22	1.001 (0.994 to 1.008)	0.830	1.003 (1.000 to 1.006)	0.059	1.003 (1.001 to 1.006)	0.016		
gamma-CEHC	Hypertension	11	0.996 (0.985 to 1.006)	0.456	1.001 (0.997 to 1.006)	0.501	1.000 (0.996 to 1.004)	0.880	1.000 (0.997 to 1.003)	0.888

Exposure	Outcome	nSNP	MR Egger		Weighted median		Inverse variance weighted		MR_PRESSO	
			OR (95% CI)	pval	OR (95% CI)	pval	OR (95% CI)	pval	OR (95% CI)	pval
N-palmitoyl-sphingosine (d18:1/16:0)	Hypertension	15	1.002 (0.994 to 1.011)	0.587	1.000 (0.996 to 1.004)	0.983	1.002 (0.998 to 1.005)	0.383		
methionine sulfone	Hypertension	32	1.001 (0.996 to 1.006)	0.745	1.000 (0.999 to 1.002)	0.696	0.999 (0.996 to 1.001)	0.271	0.999 (0.997 to 1.000)	0.174
2-methylbutyrylcarnitine (C5)	Hypertension	15	1.000 (0.995 to 1.005)	0.959	0.998 (0.994 to 1.001)	0.204	0.997 (0.994 to 1.000)	0.056		
O-sulfo-L-tyrosine	Hypertension	23	1.002 (0.996 to 1.008)	0.486	0.998 (0.994 to 1.001)	0.111	0.997 (0.995 to 0.999)	0.010		
3-(3-hydroxyphenyl)propionate sulfate	Hypertension	10	1.000 (0.994 to 1.006)	0.955	1.001 (0.997 to 1.005)	0.504	1.001 (0.998 to 1.004)	0.572		
2-palmitoyl-GPE (16:0)	Hypertension	33	0.999 (0.995 to 1.004)	0.724	1.000 (0.998 to 1.002)	0.999	1.000 (0.999 to 1.001)	0.963		

Exposure	Outcome	nSNP	MR Egger		Weighted median		Inverse variance weighted		MR_PRESSO	
			OR (95% CI)	pval	OR (95% CI)	pval	OR (95% CI)	pval	OR (95% CI)	pval
1-myristoyl-GPC (14:0)	Hypertension	13	1.003 (0.997 to 1.009)	0.401	1.000 (0.996 to 1.004)	0.925	1.000 (0.997 to 1.004)	0.825		
1-arachidoyl-GPC (20:0)	Hypertension	12	1.003 (1.000 to 1.007)	0.100	1.001 (0.999 to 1.002)	0.470	1.000 (0.997 to 1.002)	0.740	1.000 (0.999 to 1.001)	0.899
1-docosapentaenoyl-GPC (22:5n6)	Hypertension	47	1.000 (0.995 to 1.006)	0.877	0.997 (0.995 to 1.000)	0.019	1.000 (0.998 to 1.001)	0.702	0.999 (0.998 to 1.001)	0.264
21-hydroxypregnenolone disulfate	Hypertension	27	0.994 (0.982 to 1.006)	0.363	0.999 (0.997 to 1.002)	0.658	1.002 (0.999 to 1.006)	0.196	1.003 (0.999 to 1.006)	0.144
mannitol/sorbitol	Hypertension	15	1.005 (0.998 to 1.013)	0.176	1.000 (0.997 to 1.004)	0.901	1.002 (0.999 to 1.005)	0.264	1.001 (0.998 to 1.004)	0.600
methyl glucopyranoside (alpha + beta)	Hypertension	25	1.002 (0.999 to 1.004)	0.221	1.001 (0.999 to 1.003)	0.168	1.000 (0.999 to 1.002)	0.537		

Exposure	Outcome	nSNP	MR Egger		Weighted median		Inverse variance weighted		MR_PRESSO	
			OR (95% CI)	pval	OR (95% CI)	pval	OR (95% CI)	pval	OR (95% CI)	pval
4-methylcatechol sulfate	Hypertension	23	1.002 (0.994 to 1.010)	0.649	1.002 (0.999 to 1.006)	0.231	1.002 (0.999 to 1.005)	0.217	1.003 (1.000 to 1.006)	0.034
3-methyl catechol sulfate (1)	Hypertension	12	0.995 (0.985 to 1.004)	0.297	0.998 (0.994 to 1.002)	0.229	0.998 (0.994 to 1.002)	0.335	0.996 (0.993 to 0.999)	0.036
2-docosahexaenoyl-GPC (22:6)	Hypertension	11	1.002 (0.996 to 1.009)	0.545	0.999 (0.995 to 1.002)	0.451	0.999 (0.997 to 1.002)	0.730		
linoleoylcarnitine	Hypertension	19	0.997 (0.990 to 1.005)	0.488	1.001 (0.998 to 1.004)	0.407	1.001 (0.997 to 1.004)	0.713	1.000 (0.998 to 1.002)	0.862
pyroglutamine	Hypertension	30	1.000 (0.993 to 1.007)	0.910	1.002 (0.999 to 1.005)	0.235	1.002 (0.999 to 1.006)	0.203	1.000 (0.999 to 1.002)	0.611
X - 11564	Hypertension	22	0.995 (0.988 to 1.002)	0.189	0.999 (0.996 to 1.003)	0.612	0.999 (0.996 to 1.002)	0.559	0.999 (0.997 to 1.002)	0.528

Exposure	Outcome	nSNP	MR Egger		Weighted median		Inverse variance weighted		MR_PRESSO	
			OR (95% CI)	pval	OR (95% CI)	pval	OR (95% CI)	pval	OR (95% CI)	pval
X - 15486	Hypertension	26	0.996 (0.982 to 1.010)	0.575	0.995 (0.991 to 0.998)	0.003	0.996 (0.991 to 1.001)	0.096	0.993 (0.990 to 0.996)	6.39e-05
X - 21286	Hypertension	22	0.994 (0.982 to 1.005)	0.281	1.000 (0.996 to 1.004)	0.939	1.003 (0.998 to 1.007)	0.290	1.000 (0.997 to 1.004)	0.912
1-arachidonoyl-GPA (20:4)	Hypertension	17	1.000 (0.992 to 1.007)	0.978	1.002 (0.998 to 1.005)	0.371	1.001 (0.998 to 1.004)	0.469		
X - 11441	Hypertension	25	1.000 (0.996 to 1.003)	0.988	0.999 (0.996 to 1.001)	0.210	0.999 (0.998 to 1.001)	0.517		
X - 21319	Hypertension	24	0.999 (0.994 to 1.003)	0.578	0.998 (0.995 to 1.001)	0.203	0.999 (0.997 to 1.001)	0.409		
X - 18249	Hypertension	18	0.999 (0.994 to 1.004)	0.751	0.999 (0.996 to 1.002)	0.375	0.999 (0.997 to 1.002)	0.582		

Exposure	Outcome	nSNP	MR Egger		Weighted median		Inverse variance weighted		MR_PRESSO	
			OR (95% CI)	pval	OR (95% CI)	pval	OR (95% CI)	pval	OR (95% CI)	pval
X - 11905	Hypertension	42	0.999 (0.997 to 1.001)	0.323	0.999 (0.998 to 1.001)	0.291	1.000 (0.999 to 1.001)	0.709		
X - 18914	Hypertension	21	0.997 (0.992 to 1.002)	0.274	0.999 (0.996 to 1.002)	0.411	0.999 (0.997 to 1.002)	0.539	1.000 (0.998 to 1.002)	0.850
X - 21339	Hypertension	29	1.001 (0.999 to 1.003)	0.469	1.001 (0.999 to 1.002)	0.340	1.000 (0.999 to 1.001)	0.621		
X - 21341	Hypertension	11	1.001 (0.995 to 1.007)	0.758	1.003 (0.999 to 1.007)	0.111	1.003 (1.000 to 1.007)	0.052		
X - 21343	Hypertension	35	0.999 (0.993 to 1.004)	0.689	0.998 (0.995 to 1.000)	0.065	1.000 (0.997 to 1.002)	0.917	0.998 (0.996 to 1.000)	0.086
X - 11308	Hypertension	20	1.000 (0.999 to 1.001)	0.795	1.000 (0.999 to 1.002)	0.413	1.000 (1.000 to 1.001)	0.360		

Exposure	Outcome	nSNP	MR Egger		Weighted median		Inverse variance weighted		MR_PRESSO	
			OR (95% CI)	pval	OR (95% CI)	pval	OR (95% CI)	pval	OR (95% CI)	pval
X - 21353	Hypertension	34	0.999 (0.997 to 1.002)	0.623	0.999 (0.998 to 1.001)	0.335	0.999 (0.998 to 1.000)	0.027		
X - 17178	Hypertension	42	1.008 (0.997 to 1.019)	0.161	1.009 (1.007 to 1.011)	1.08e-14	1.003 (0.999 to 1.007)	0.192	1.000 (0.998 to 1.002)	0.982
X - 21365	Hypertension	18	0.998 (0.987 to 1.008)	0.657	0.998 (0.995 to 1.001)	0.246	0.998 (0.994 to 1.001)	0.204	0.999 (0.997 to 1.001)	0.335
X - 17189	Hypertension	23	0.995 (0.974 to 1.016)	0.642	1.002 (0.998 to 1.006)	0.441	1.005 (0.998 to 1.013)	0.162	1.002 (1.000 to 1.005)	0.105
X - 11442	Hypertension	30	1.003 (0.992 to 1.014)	0.645	1.000 (0.997 to 1.002)	0.893	1.000 (0.996 to 1.003)	0.781	1.001 (0.999 to 1.002)	0.595
X - 21411	Hypertension	30	1.004 (0.996 to 1.011)	0.351	0.996 (0.993 to 0.998)	0.001	0.997 (0.994 to 0.999)	0.006	0.996 (0.994 to 0.998)	0.002

Exposure	Outcome	nSNP	MR Egger		Weighted median		Inverse variance weighted		MR_PRESSO	
			OR (95% CI)	pval	OR (95% CI)	pval	OR (95% CI)	pval	OR (95% CI)	pval
X - 12511	Hypertension	24	1.001 (0.996 to 1.005)	0.709	1.004 (1.002 to 1.005)	5.51e-08	1.003 (1.001 to 1.004)	6.23e-04	1.003 (1.002 to 1.005)	5.08e-05
X - 01911	Hypertension	13	1.001 (0.993 to 1.010)	0.734	1.002 (0.998 to 1.005)	0.390	0.999 (0.996 to 1.003)	0.593	1.000 (0.997 to 1.003)	0.911
X - 11444	Hypertension	17	1.000 (0.997 to 1.004)	0.893	1.001 (0.998 to 1.003)	0.620	1.001 (0.999 to 1.003)	0.403		
X - 12824	Hypertension	31	1.004 (0.995 to 1.012)	0.391	1.000 (0.998 to 1.003)	0.822	0.997 (0.994 to 1.000)	0.083	0.996 (0.994 to 0.999)	0.011
X - 15497	Hypertension	14	0.998 (0.992 to 1.004)	0.529	0.999 (0.996 to 1.003)	0.662	0.998 (0.995 to 1.001)	0.172		
X - 21441	Hypertension	23	1.000 (0.996 to 1.004)	0.901	0.999 (0.996 to 1.001)	0.399	0.999 (0.997 to 1.001)	0.257		

Exposure	Outcome	nSNP	MR Egger		Weighted median		Inverse variance weighted		MR_PRESSO	
			OR (95% CI)	pval	OR (95% CI)	pval	OR (95% CI)	pval	OR (95% CI)	pval
X - 17185	Hypertension	18	0.996 (0.991 to 1.002)	0.190	0.998 (0.994 to 1.001)	0.136	0.997 (0.995 to 0.999)	0.010		
X - 21448	Hypertension	19	0.996 (0.988 to 1.003)	0.253	0.999 (0.997 to 1.001)	0.167	1.000 (0.998 to 1.002)	0.744	1.000 (0.998 to 1.001)	0.682
X - 02269	Hypertension	26	0.999 (0.992 to 1.006)	0.869	1.001 (0.997 to 1.004)	0.725	1.004 (1.002 to 1.007)	0.002		
X - 02249	Hypertension	23	1.002 (0.996 to 1.009)	0.532	1.001 (0.998 to 1.004)	0.462	1.001 (0.998 to 1.004)	0.628	1.001 (0.999 to 1.003)	0.368
X - 11850	Hypertension	11	0.998 (0.989 to 1.006)	0.600	0.997 (0.992 to 1.002)	0.249	0.999 (0.995 to 1.002)	0.459		
X - 16946	Hypertension	24	0.998 (0.994 to 1.002)	0.437	0.998 (0.996 to 1.001)	0.182	0.999 (0.998 to 1.001)	0.299		

Exposure	Outcome	nSNP	MR Egger		Weighted median		Inverse variance weighted		MR_PRESSO	
			OR (95% CI)	pval	OR (95% CI)	pval	OR (95% CI)	pval	OR (95% CI)	pval
alpha-hydroxyisovalerate	Hypertension	36	1.004 (1.000 to 1.007)	0.044	1.000 (0.998 to 1.003)	0.909	1.000 (0.998 to 1.002)	0.764		
3-methylglutarylcarnitine (1)	Hypertension	16	0.996 (0.985 to 1.008)	0.551	0.996 (0.992 to 0.999)	0.013	0.999 (0.995 to 1.002)	0.473	0.997 (0.994 to 1.000)	0.064
3-methylglutarylcarnitine (2)	Hypertension	19	0.997 (0.983 to 1.011)	0.698	1.002 (0.999 to 1.005)	0.254	1.001 (0.995 to 1.006)	0.783	1.001 (0.997 to 1.004)	0.749
X - 12063	Hypertension	36	1.005 (1.001 to 1.009)	0.026	0.998 (0.996 to 1.000)	0.085	1.000 (0.998 to 1.002)	0.995	1.000 (0.999 to 1.002)	0.729
X - 07765	Hypertension	9	1.001 (0.991 to 1.011)	0.892	1.003 (0.998 to 1.007)	0.221	0.999 (0.995 to 1.004)	0.796		
X - 11299	Hypertension	7	0.998 (0.986 to 1.009)	0.685	1.003 (0.998 to 1.008)	0.188	1.001 (0.998 to 1.005)	0.506		

Exposure	Outcome	nSNP	MR Egger		Weighted median		Inverse variance weighted		MR_PRESSO	
			OR (95% CI)	pval	OR (95% CI)	pval	OR (95% CI)	pval	OR (95% CI)	pval
X - 11305	Hypertension	11	0.997 (0.989 to 1.006)	0.543	1.000 (0.996 to 1.005)	0.884	1.000 (0.996 to 1.005)	0.957		
X - 11372	Hypertension	22	0.997 (0.992 to 1.003)	0.357	0.997 (0.994 to 1.000)	0.060	0.997 (0.995 to 0.999)	0.014		
X - 11429	Hypertension	20	0.997 (0.992 to 1.001)	0.152	1.002 (0.998 to 1.005)	0.317	1.002 (0.999 to 1.004)	0.210		
X - 11438	Hypertension	14	1.000 (0.995 to 1.006)	0.960	0.997 (0.993 to 1.002)	0.222	0.997 (0.994 to 1.000)	0.033		
X - 11440	Hypertension	27	1.002 (0.999 to 1.005)	0.172	0.999 (0.997 to 1.000)	0.163	0.999 (0.997 to 1.001)	0.257	0.999 (0.998 to 1.001)	0.292
X - 11452	Hypertension	8	1.012 (1.002 to 1.022)	0.060	1.002 (0.997 to 1.006)	0.451	1.004 (1.000 to 1.009)	0.061		

Exposure	Outcome	nSNP	MR Egger		Weighted median		Inverse variance weighted		MR_PRESSO	
			OR (95% CI)	pval	OR (95% CI)	pval	OR (95% CI)	pval	OR (95% CI)	pval
X - 11470	Hypertension	35	1.000 (0.997 to 1.003)	0.891	0.998 (0.996 to 1.001)	0.163	0.997 (0.996 to 0.999)	0.004		
X - 11478	Hypertension	22	0.998 (0.993 to 1.002)	0.382	0.997 (0.994 to 1.000)	0.029	0.997 (0.995 to 1.000)	0.026		
X - 11530	Hypertension	19	1.002 (1.000 to 1.005)	0.123	1.001 (0.999 to 1.003)	0.385	1.001 (0.999 to 1.002)	0.250		
X - 11538	Hypertension	43	0.998 (0.996 to 1.000)	0.080	0.998 (0.996 to 0.999)	9.35e-04	0.998 (0.997 to 0.999)	1.55e-06		
X - 11880	Hypertension	22	1.000 (0.997 to 1.003)	0.957	1.001 (0.998 to 1.003)	0.547	1.001 (0.999 to 1.002)	0.555		
X - 12007	Hypertension	16	1.000 (0.994 to 1.005)	0.891	0.999 (0.996 to 1.003)	0.689	1.000 (0.998 to 1.003)	0.897		

Exposure	Outcome	nSNP	MR Egger		Weighted median		Inverse variance weighted		MR_PRESSO	
			OR (95% CI)	pval	OR (95% CI)	pval	OR (95% CI)	pval	OR (95% CI)	pval
X - 12206	Hypertension	27	0.996 (0.992 to 1.000)	0.039	1.001 (0.998 to 1.003)	0.672	1.001 (0.999 to 1.003)	0.595		
X - 12216	Hypertension	11	1.005 (0.994 to 1.016)	0.383	0.998 (0.993 to 1.002)	0.351	1.000 (0.995 to 1.005)	0.983	0.998 (0.994 to 1.002)	0.322
X - 12230	Hypertension	18	1.000 (0.994 to 1.005)	0.952	1.002 (0.999 to 1.006)	0.200	1.003 (1.001 to 1.006)	0.016		
X - 12411	Hypertension	21	1.004 (0.996 to 1.011)	0.340	1.000 (0.997 to 1.004)	0.837	1.000 (0.997 to 1.003)	0.801	1.002 (0.999 to 1.004)	0.203
5,8-tetradecadienoate	Hypertension	31	1.002 (0.998 to 1.005)	0.444	1.000 (0.998 to 1.003)	0.857	1.001 (0.999 to 1.002)	0.496		
X - 12450	Hypertension	15	0.996 (0.984 to 1.009)	0.583	1.003 (0.999 to 1.007)	0.207	0.999 (0.994 to 1.003)	0.523	1.000 (0.996 to 1.003)	0.886

Exposure	Outcome	nSNP	MR Egger		Weighted median		Inverse variance weighted		MR_PRESSO	
			OR (95% CI)	pval	OR (95% CI)	pval	OR (95% CI)	pval	OR (95% CI)	pval
X - 12729	Hypertension	18	0.999 (0.997 to 1.001)	0.320	1.000 (0.998 to 1.002)	0.924	1.000 (0.999 to 1.002)	0.775		
X - 12798	Hypertension	34	0.999 (0.998 to 1.001)	0.544	0.999 (0.998 to 1.001)	0.492	0.999 (0.998 to 1.000)	0.013		
X - 12472	Hypertension	27	0.999 (0.990 to 1.008)	0.789	0.998 (0.995 to 1.001)	0.153	0.999 (0.997 to 1.002)	0.545		
X - 12816	Hypertension	13	0.998 (0.987 to 1.009)	0.706	0.999 (0.995 to 1.002)	0.388	1.001 (0.997 to 1.004)	0.690	0.999 (0.997 to 1.001)	0.404
X - 14056	Hypertension	19	0.995 (0.988 to 1.002)	0.161	1.001 (0.997 to 1.004)	0.732	1.000 (0.998 to 1.003)	0.745		
X - 12844	Hypertension	21	0.998 (0.992 to 1.004)	0.555	1.000 (0.996 to 1.003)	0.782	0.999 (0.997 to 1.002)	0.660		

Exposure	Outcome	nSNP	MR Egger		Weighted median		Inverse variance weighted		MR_PRESSO	
			OR (95% CI)	pval	OR (95% CI)	pval	OR (95% CI)	pval	OR (95% CI)	pval
X - 12846	Hypertension	21	0.998 (0.994 to 1.003)	0.542	0.998 (0.996 to 1.001)	0.192	0.999 (0.997 to 1.000)	0.139		
X - 15469	Hypertension	15	1.001 (0.999 to 1.003)	0.406	1.001 (1.000 to 1.002)	0.291	1.000 (1.000 to 1.001)	0.397		
X - 13529	Hypertension	19	0.999 (0.995 to 1.003)	0.586	1.000 (0.998 to 1.003)	0.831	1.001 (0.998 to 1.003)	0.584	1.000 (0.998 to 1.002)	0.989
X - 16132	Hypertension	15	0.999 (0.992 to 1.006)	0.789	1.001 (0.997 to 1.004)	0.621	1.001 (0.998 to 1.004)	0.399		
X - 14568	Hypertension	24	1.001 (0.996 to 1.006)	0.632	0.999 (0.996 to 1.002)	0.492	0.999 (0.997 to 1.001)	0.358		
X - 14939	Hypertension	26	0.999 (0.994 to 1.004)	0.820	1.001 (0.999 to 1.003)	0.351	1.001 (0.999 to 1.002)	0.488		

Exposure	Outcome	nSNP	MR Egger		Weighted median		Inverse variance weighted		MR_PRESSO	
			OR (95% CI)	pval	OR (95% CI)	pval	OR (95% CI)	pval	OR (95% CI)	pval
X - 15245	Hypertension	11	1.007 (1.000 to 1.014)	0.101	1.001 (0.997 to 1.005)	0.553	1.001 (0.998 to 1.004)	0.450		
X - 15492	Hypertension	25	0.998 (0.992 to 1.003)	0.362	0.999 (0.997 to 1.002)	0.653	1.000 (0.998 to 1.002)	0.815		
X - 15728	Hypertension	8	0.997 (0.986 to 1.008)	0.602	1.000 (0.995 to 1.006)	0.881	1.003 (0.998 to 1.009)	0.263		
X - 16123	Hypertension	26	0.999 (0.993 to 1.005)	0.816	0.997 (0.994 to 1.000)	0.090	0.998 (0.995 to 1.000)	0.077		
X - 16935	Hypertension	34	1.001 (0.999 to 1.003)	0.211	1.000 (0.999 to 1.001)	0.890	1.000 (0.999 to 1.001)	0.839		
X - 16944	Hypertension	22	0.998 (0.992 to 1.004)	0.475	0.998 (0.995 to 1.001)	0.174	0.999 (0.997 to 1.002)	0.636		

Exposure	Outcome	nSNP	MR Egger		Weighted median		Inverse variance weighted		MR_PRESSO	
			OR (95% CI)	pval	OR (95% CI)	pval	OR (95% CI)	pval	OR (95% CI)	pval
X - 17145	Hypertension	11	1.000 (0.998 to 1.002)	0.997	0.999 (0.998 to 1.001)	0.359	0.999 (0.998 to 1.000)	0.111		
X - 18901	Hypertension	10	0.998 (0.990 to 1.007)	0.690	0.999 (0.995 to 1.003)	0.534	0.999 (0.994 to 1.003)	0.520	0.997 (0.994 to 1.000)	0.133
X - 17166	Hypertension	26	0.998 (0.992 to 1.004)	0.549	0.999 (0.997 to 1.000)	0.111	0.999 (0.995 to 1.002)	0.443	0.999 (0.998 to 1.000)	0.120
X - 17167	Hypertension	18	0.997 (0.992 to 1.002)	0.228	0.999 (0.996 to 1.002)	0.455	0.999 (0.996 to 1.001)	0.181		
X - 18913	Hypertension	10	0.997 (0.989 to 1.004)	0.442	0.998 (0.993 to 1.002)	0.347	0.998 (0.995 to 1.002)	0.326		
X - 21628	Hypertension	29	1.005 (0.994 to 1.015)	0.375	1.001 (0.997 to 1.004)	0.714	0.995 (0.991 to 0.999)	0.010	0.999 (0.996 to 1.002)	0.516

Exposure	Outcome	nSNP	MR Egger		Weighted median		Inverse variance weighted		MR_PRESSO	
			OR (95% CI)	pval	OR (95% CI)	pval	OR (95% CI)	pval	OR (95% CI)	pval
X - 16134	Hypertension	29	1.002 (0.998 to 1.005)	0.372	0.999 (0.997 to 1.002)	0.553	1.000 (0.998 to 1.002)	0.813		
X - 17179	Hypertension	24	0.999 (0.996 to 1.002)	0.523	1.000 (0.999 to 1.002)	0.672	1.000 (0.998 to 1.001)	0.489		
X - 19141	Hypertension	31	0.996 (0.992 to 1.000)	0.042	0.997 (0.996 to 0.998)	2.25e-05	1.000 (0.998 to 1.002)	0.801	0.998 (0.996 to 1.001)	0.161
X - 17438	Hypertension	10	1.006 (0.999 to 1.014)	0.140	1.002 (0.998 to 1.006)	0.393	1.002 (0.999 to 1.005)	0.141		
X - 21666	Hypertension	12	0.998 (0.992 to 1.004)	0.538	1.001 (0.997 to 1.004)	0.780	1.001 (0.998 to 1.004)	0.631		
X - 12855	Hypertension	17	0.996 (0.987 to 1.005)	0.384	0.998 (0.995 to 1.002)	0.394	0.998 (0.995 to 1.001)	0.204		

Exposure	Outcome	nSNP	MR Egger		Weighted median		Inverse variance weighted		MR_PRESSO	
			OR (95% CI)	pval	OR (95% CI)	pval	OR (95% CI)	pval	OR (95% CI)	pval
oleoyl-linoleoyl-glycerol (18:1/18:2) [1]	Hypertension	43	1.001 (0.995 to 1.008)	0.735	1.003 (1.001 to 1.006)	0.002	1.000 (0.998 to 1.003)	0.852	1.002 (1.000 to 1.003)	0.064
oleoyl-linoleoyl-glycerol (18:1/18:2) [2]	Hypertension	17	1.003 (0.995 to 1.012)	0.451	1.004 (1.001 to 1.008)	0.006	1.001 (0.997 to 1.005)	0.589	1.002 (0.998 to 1.005)	0.418
X - 12748	Hypertension	17	1.003 (0.997 to 1.008)	0.368	1.000 (0.996 to 1.003)	0.834	1.000 (0.997 to 1.003)	0.983	0.999 (0.997 to 1.002)	0.624
X - 21735	Hypertension	31	1.007 (1.001 to 1.013)	0.035	1.000 (0.998 to 1.003)	0.783	0.999 (0.997 to 1.001)	0.389	0.999 (0.997 to 1.001)	0.299
X - 21736	Hypertension	21	1.001 (0.995 to 1.007)	0.721	0.999 (0.996 to 1.003)	0.751	1.001 (0.998 to 1.004)	0.694	0.999 (0.997 to 1.002)	0.574
X - 21737	Hypertension	10	0.998 (0.991 to 1.005)	0.630	0.998 (0.994 to 1.002)	0.428	1.000 (0.997 to 1.004)	0.807		

Exposure	Outcome	nSNP	MR Egger		Weighted median		Inverse variance weighted		MR_PRESSO	
			OR (95% CI)	pval	OR (95% CI)	pval	OR (95% CI)	pval	OR (95% CI)	pval
X - 11522	Hypertension	20	1.001 (0.996 to 1.005)	0.810	0.998 (0.996 to 1.000)	0.106	0.999 (0.998 to 1.001)	0.269		
gulonic acid	Hypertension	12	1.005 (0.995 to 1.014)	0.363	0.998 (0.994 to 1.002)	0.418	0.997 (0.993 to 1.001)	0.182		
sulfate	Hypertension	17	0.999 (0.992 to 1.006)	0.828	1.001 (0.998 to 1.005)	0.389	1.001 (0.998 to 1.004)	0.690		
X - 15503	Hypertension	16	1.001 (0.994 to 1.009)	0.750	1.001 (0.998 to 1.005)	0.565	1.001 (0.998 to 1.004)	0.456		
X - 12822	Hypertension	21	1.002 (0.984 to 1.019)	0.852	0.990 (0.987 to 0.993)	1.44e-10	0.997 (0.992 to 1.002)	0.256	0.993 (0.990 to 0.996)	8.57e-05
X - 21821	Hypertension	14	0.995 (0.991 to 0.999)	0.028	0.999 (0.997 to 1.002)	0.573	0.999 (0.997 to 1.001)	0.165	1.000 (0.998 to 1.002)	0.861

Exposure	Outcome	nSNP	MR Egger		Weighted median		Inverse variance weighted		MR_PRESSO	
			OR (95% CI)	pval	OR (95% CI)	pval	OR (95% CI)	pval	OR (95% CI)	pval
X - 17327	Hypertension	26	1.000 (0.994 to 1.006)	0.949	0.998 (0.995 to 1.001)	0.184	0.999 (0.996 to 1.001)	0.195		
X - 16570	Hypertension	17	0.998 (0.983 to 1.014)	0.835	0.996 (0.992 to 1.000)	0.068	0.995 (0.991 to 1.000)	0.037	0.998 (0.994 to 1.001)	0.193
X - 21849	Hypertension	24	1.004 (0.998 to 1.010)	0.228	1.000 (0.998 to 1.003)	0.889	1.000 (0.998 to 1.002)	0.818		
X - 21892	Hypertension	25	1.003 (0.998 to 1.009)	0.285	0.998 (0.995 to 1.001)	0.195	0.999 (0.996 to 1.002)	0.460		
1-nonadecanoyl-GPC (19:0)	Hypertension	18	1.005 (0.998 to 1.011)	0.157	1.003 (0.999 to 1.006)	0.175	1.001 (0.998 to 1.004)	0.526	1.002 (1.000 to 1.005)	0.068
etiocholanolone glucuronide	Hypertension	25	1.001 (0.996 to 1.006)	0.626	0.998 (0.996 to 1.000)	0.107	1.001 (0.998 to 1.004)	0.479	1.001 (0.999 to 1.004)	0.307

Exposure	Outcome	nSNP	MR Egger		Weighted median		Inverse variance weighted		MR_PRESSO	
			OR (95% CI)	pval	OR (95% CI)	pval	OR (95% CI)	pval	OR (95% CI)	pval
2-palmitoleoyl-GPC (16:1)	Hypertension	17	0.993 (0.981 to 1.006)	0.302	0.998 (0.993 to 1.002)	0.338	0.995 (0.991 to 1.000)	0.043	0.998 (0.994 to 1.001)	0.184
sphingomyelin (d18:1/24:1, d18:2/24:0)	Hypertension	36	1.004 (1.001 to 1.007)	0.020	1.000 (0.999 to 1.002)	0.625	1.000 (0.998 to 1.001)	0.567	1.000 (0.999 to 1.001)	0.671
sphingomyelin (d18:2/14:0, d18:1/14:1)	Hypertension	14	1.005 (0.998 to 1.012)	0.183	1.002 (0.998 to 1.005)	0.444	1.000 (0.996 to 1.004)	0.999	1.001 (0.998 to 1.004)	0.374
X - 12680	Hypertension	16	1.005 (0.998 to 1.013)	0.158	1.001 (0.998 to 1.005)	0.533	0.998 (0.994 to 1.003)	0.482	1.000 (0.997 to 1.003)	0.932
X - 22147	Hypertension	14	1.000 (0.991 to 1.010)	0.938	0.996 (0.992 to 1.000)	0.040	0.997 (0.994 to 1.000)	0.059		
X - 22162	Hypertension	19	1.001 (0.999 to 1.003)	0.384	1.001 (0.999 to 1.002)	0.241	1.000 (0.999 to 1.001)	0.806		

Exposure	Outcome	nSNP	MR Egger		Weighted median		Inverse variance weighted		MR_PRESSO	
			OR (95% CI)	pval	OR (95% CI)	pval	OR (95% CI)	pval	OR (95% CI)	pval
X - 10458	Hypertension	4	1.001 (0.988 to 1.014)	0.901	1.002 (0.994 to 1.010)	0.652	0.999 (0.992 to 1.006)	0.812		
X - 11537	Hypertension	18	1.004 (0.998 to 1.010)	0.186	0.999 (0.996 to 1.003)	0.699	0.998 (0.995 to 1.001)	0.182		
X - 22379	Hypertension	31	0.999 (0.996 to 1.001)	0.252	0.999 (0.997 to 1.000)	0.098	1.000 (0.998 to 1.002)	0.973		
X - 11871	Hypertension	9	1.004 (0.995 to 1.014)	0.364	1.000 (0.996 to 1.005)	0.858	1.000 (0.995 to 1.004)	0.893		
X - 12101	Hypertension	26	0.995 (0.987 to 1.004)	0.312	0.997 (0.995 to 1.000)	0.033	0.999 (0.996 to 1.001)	0.318	0.998 (0.996 to 1.000)	0.122
X - 11483	Hypertension	10	1.003 (0.998 to 1.008)	0.227	1.002 (0.999 to 1.006)	0.207	1.003 (1.000 to 1.006)	0.032		

Exposure	Outcome	nSNP	MR Egger		Weighted median		Inverse variance weighted		MR_PRESSO	
			OR (95% CI)	pval	OR (95% CI)	pval	OR (95% CI)	pval	OR (95% CI)	pval
X - 11491	Hypertension	42	1.001 (0.999 to 1.003)	0.425	1.001 (1.000 to 1.002)	0.275	1.001 (1.000 to 1.001)	0.280		
X - 12231	Hypertension	18	0.998 (0.990 to 1.007)	0.685	0.998 (0.994 to 1.001)	0.257	1.001 (0.998 to 1.004)	0.605	1.000 (0.997 to 1.002)	0.835
X - 12830	Hypertension	12	1.005 (1.000 to 1.010)	0.097	1.002 (0.998 to 1.005)	0.381	1.001 (0.998 to 1.003)	0.485		
X - 18899	Hypertension	16	1.002 (0.998 to 1.007)	0.334	0.999 (0.997 to 1.002)	0.583	0.999 (0.996 to 1.002)	0.433	1.000 (0.997 to 1.002)	0.746
5-hydroxytryptophan	Hypertension	25	1.001 (0.994 to 1.008)	0.707	1.001 (0.998 to 1.005)	0.426	1.001 (0.998 to 1.004)	0.487	1.000 (0.997 to 1.002)	0.798
X - 12127	Hypertension	20	1.001 (0.990 to 1.012)	0.807	0.998 (0.995 to 1.001)	0.257	0.997 (0.994 to 1.000)	0.035		

Exposure	Outcome	nSNP	MR Egger		Weighted median		Inverse variance weighted		MR_PRESSO	
			OR (95% CI)	pval	OR (95% CI)	pval	OR (95% CI)	pval	OR (95% CI)	pval
X - 12339	Hypertension	16	0.988 (0.979 to 0.998)	0.028	0.999 (0.995 to 1.003)	0.550	0.998 (0.995 to 1.002)	0.369	0.997 (0.994 to 1.001)	0.140
X - 12681	Hypertension	27	0.993 (0.982 to 1.005)	0.272	0.998 (0.995 to 1.001)	0.120	0.995 (0.990 to 1.000)	0.068	0.998 (0.996 to 1.001)	0.200
X - 12686	Hypertension	25	0.991 (0.986 to 0.996)	0.001	0.998 (0.995 to 1.001)	0.201	0.998 (0.996 to 1.001)	0.165		
X - 12688	Hypertension	24	1.006 (1.001 to 1.011)	0.022	1.001 (0.998 to 1.004)	0.517	1.002 (1.000 to 1.004)	0.034		
X - 14838	Hypertension	26	1.004 (0.999 to 1.008)	0.106	1.003 (1.000 to 1.006)	0.073	1.002 (1.000 to 1.004)	0.110	1.000 (0.998 to 1.003)	0.782
nonanoylcarnitine	Hypertension	20	1.001 (0.998 to 1.004)	0.619	1.001 (0.999 to 1.002)	0.486	1.000 (0.999 to 1.002)	0.586		

Exposure	Outcome	nSNP	MR Egger		Weighted median		Inverse variance weighted		MR_PRESSO	
			OR (95% CI)	pval	OR (95% CI)	pval	OR (95% CI)	pval	OR (95% CI)	pval
X - 15666	Hypertension	17	1.002 (0.997 to 1.007)	0.473	1.002 (0.998 to 1.005)	0.296	1.002 (1.000 to 1.005)	0.078		
X - 16071	Hypertension	12	0.995 (0.990 to 1.000)	0.084	0.999 (0.996 to 1.002)	0.548	0.999 (0.997 to 1.001)	0.192		
X - 16580	Hypertension	16	1.001 (0.992 to 1.009)	0.842	1.004 (1.000 to 1.008)	0.059	1.003 (0.999 to 1.006)	0.150	1.004 (1.001 to 1.007)	0.032
X - 17337	Hypertension	31	1.001 (0.999 to 1.003)	0.311	1.000 (0.998 to 1.002)	0.960	1.000 (0.999 to 1.002)	0.497		
1-eicosapentaenoyl-GPE (20:5)	Hypertension	24	0.998 (0.993 to 1.004)	0.588	0.999 (0.996 to 1.002)	0.409	0.999 (0.997 to 1.001)	0.234		
X - 12026	Hypertension	15	1.003 (0.996 to 1.010)	0.362	1.002 (0.998 to 1.005)	0.436	1.001 (0.998 to 1.004)	0.549		

Exposure	Outcome	nSNP	MR Egger		Weighted median		Inverse variance weighted		MR_PRESSO	
			OR (95% CI)	pval	OR (95% CI)	pval	OR (95% CI)	pval	OR (95% CI)	pval
X - 12543	Hypertension	16	1.003 (0.990 to 1.016)	0.662	0.999 (0.994 to 1.004)	0.675	1.001 (0.995 to 1.007)	0.677	0.999 (0.996 to 1.003)	0.730
X - 17010	Hypertension	42	1.003 (0.999 to 1.006)	0.123	1.002 (0.999 to 1.004)	0.202	1.000 (0.999 to 1.002)	0.600		
X - 17351	Hypertension	12	1.002 (0.996 to 1.007)	0.565	1.000 (0.997 to 1.004)	0.878	1.002 (0.999 to 1.004)	0.131		
X - 22764	Hypertension	21	1.002 (0.997 to 1.006)	0.483	1.004 (1.001 to 1.007)	0.008	1.003 (1.001 to 1.006)	0.011	1.003 (1.001 to 1.005)	0.024
glucose	Hypertension	22	0.995 (0.990 to 1.001)	0.102	1.000 (0.997 to 1.004)	0.876	0.999 (0.996 to 1.001)	0.393		
mannose	Hypertension	19	1.000 (0.993 to 1.007)	0.913	1.002 (0.998 to 1.005)	0.269	1.000 (0.997 to 1.003)	0.976	1.000 (0.998 to 1.002)	0.860

Exposure	Outcome	nSNP	MR Egger		Weighted median		Inverse variance weighted		MR_PRESSO	
			OR (95% CI)	pval	OR (95% CI)	pval	OR (95% CI)	pval	OR (95% CI)	pval
X - 10358	Hypertension	17	1.003 (0.998 to 1.008)	0.312	1.001 (0.998 to 1.004)	0.525	1.001 (0.999 to 1.003)	0.404		
X - 22816	Hypertension	20	1.002 (0.997 to 1.008)	0.411	0.998 (0.995 to 1.001)	0.233	0.999 (0.997 to 1.001)	0.416		
X - 22822	Hypertension	13	1.000 (0.985 to 1.016)	0.964	1.001 (0.996 to 1.006)	0.587	1.000 (0.995 to 1.005)	0.996	1.000 (0.995 to 1.004)	0.912
myristoleoylcarnitine	Hypertension	18	1.000 (0.998 to 1.003)	0.789	1.000 (0.998 to 1.002)	0.742	0.999 (0.998 to 1.001)	0.458		
N-acetyltaurine	Hypertension	20	1.002 (0.996 to 1.009)	0.453	1.001 (0.998 to 1.004)	0.489	1.001 (0.998 to 1.004)	0.442	1.002 (0.999 to 1.004)	0.171
X - 12695	Hypertension	24	1.005 (1.001 to 1.009)	0.020	1.000 (0.998 to 1.001)	0.596	0.999 (0.997 to 1.000)	0.141	1.000 (0.999 to 1.000)	0.305

Exposure	Outcome	nSNP	MR Egger		Weighted median		Inverse variance weighted		MR_PRESSO	
			OR (95% CI)	pval	OR (95% CI)	pval	OR (95% CI)	pval	OR (95% CI)	pval
arabonate/xylonate	Hypertension	16	1.003 (0.997 to 1.009)	0.313	1.003 (0.999 to 1.006)	0.138	1.002 (1.000 to 1.005)	0.077		
2-oleoyl-GPC (18:1)	Hypertension	20	0.998 (0.992 to 1.004)	0.492	1.000 (0.996 to 1.003)	0.771	1.000 (0.997 to 1.002)	0.696		
X - 24435	Hypertension	23	1.001 (0.995 to 1.006)	0.824	0.999 (0.997 to 1.002)	0.557	0.999 (0.997 to 1.001)	0.437	1.000 (0.998 to 1.001)	0.684
dopamine sulfate (2)	Hypertension	28	1.002 (1.000 to 1.003)	0.034	1.001 (1.000 to 1.002)	0.184	1.002 (1.001 to 1.003)	0.002	1.001 (1.000 to 1.002)	0.091
tyramine O-sulfate	Hypertension	15	1.006 (1.001 to 1.011)	0.028	1.001 (0.998 to 1.004)	0.589	1.001 (0.998 to 1.003)	0.618		
methyl-4-hydroxybenzoate sulfate	Hypertension	6	1.000 (0.992 to 1.008)	0.931	1.001 (0.996 to 1.007)	0.606	1.002 (0.998 to 1.005)	0.386		

Exposure	Outcome	nSNP	MR Egger		Weighted median		Inverse variance weighted		MR_PRESSO	
			OR (95% CI)	pval	OR (95% CI)	pval	OR (95% CI)	pval	OR (95% CI)	pval
N-acetylcitrulline	Hypertension	29	1.000 (0.998 to 1.002)	0.776	1.002 (1.001 to 1.003)	1.12e-04	1.001 (1.000 to 1.002)	0.022	1.001 (1.001 to 1.002)	0.002
4-hydroxychlorothalonil	Hypertension	21	1.003 (0.997 to 1.008)	0.394	1.002 (0.999 to 1.005)	0.121	1.000 (0.998 to 1.002)	0.888		
3-hydroxypyridine sulfate	Hypertension	20	1.000 (0.997 to 1.003)	0.923	1.000 (0.999 to 1.002)	0.757	1.000 (0.998 to 1.001)	0.773	1.000 (0.999 to 1.002)	0.774
sphingomyelin (d18:1/20:0, d16:1/22:0)	Hypertension	22	0.989 (0.977 to 1.001)	0.086	1.001 (0.997 to 1.004)	0.737	0.999 (0.995 to 1.004)	0.767	1.001 (0.999 to 1.003)	0.338
sphingomyelin (d18:1/20:1, d18:2/20:0)	Hypertension	27	1.000 (0.995 to 1.004)	0.890	0.998 (0.996 to 1.001)	0.195	1.000 (0.998 to 1.001)	0.606		
behenoyl sphingomyelin (d18:1/22:0)	Hypertension	19	0.999 (0.990 to 1.007)	0.772	0.998 (0.995 to 1.002)	0.357	1.000 (0.995 to 1.004)	0.869	0.998 (0.995 to 1.001)	0.142

Exposure	Outcome	nSNP	MR Egger		Weighted median		Inverse variance weighted		MR_PRESSO	
			OR (95% CI)	pval	OR (95% CI)	pval	OR (95% CI)	pval	OR (95% CI)	pval
X - 23026	Hypertension	20	1.001 (0.993 to 1.009)	0.843	1.000 (0.997 to 1.003)	0.937	1.003 (1.000 to 1.006)	0.067	1.002 (0.999 to 1.005)	0.151
2-acetamidophenol sulfate	Hypertension	10	0.994 (0.976 to 1.012)	0.521	1.000 (0.995 to 1.005)	0.985	1.001 (0.997 to 1.005)	0.739		
1,2,3-benzenetriol sulfate (2)	Hypertension	9	1.000 (0.991 to 1.009)	0.963	1.002 (0.997 to 1.006)	0.470	1.004 (0.999 to 1.008)	0.096		
C-glycosyltryptophan	Hypertension	18	1.002 (0.997 to 1.008)	0.431	1.003 (1.000 to 1.007)	0.072	1.003 (0.999 to 1.006)	0.100	1.003 (1.001 to 1.006)	0.033
p-cresol-glucuronide	Hypertension	14	0.999 (0.991 to 1.007)	0.786	1.000 (0.995 to 1.004)	0.836	1.001 (0.997 to 1.004)	0.730		
arabitol/xylitol	Hypertension	24	1.009 (1.000 to 1.018)	0.064	1.002 (0.999 to 1.005)	0.206	1.001 (0.999 to 1.004)	0.344	1.003 (1.002 to 1.005)	0.001

Exposure	Outcome	nSNP	MR Egger		Weighted median		Inverse variance weighted		MR_PRESSO	
			OR (95% CI)	pval	OR (95% CI)	pval	OR (95% CI)	pval	OR (95% CI)	pval
X - 23293	Hypertension	33	1.003 (0.997 to 1.009)	0.371	1.000 (0.998 to 1.002)	0.949	1.000 (0.999 to 1.001)	0.773		
X - 23593	Hypertension	18	0.996 (0.990 to 1.002)	0.199	0.998 (0.995 to 1.002)	0.314	0.998 (0.995 to 1.001)	0.258		
X - 23639	Hypertension	18	1.001 (0.996 to 1.006)	0.641	1.001 (0.997 to 1.004)	0.719	0.999 (0.997 to 1.002)	0.693		
X - 23644	Hypertension	23	1.001 (0.997 to 1.006)	0.561	1.002 (0.999 to 1.004)	0.288	1.001 (0.998 to 1.003)	0.615		
X - 23705	Hypertension	43	0.996 (0.993 to 0.999)	0.011	0.999 (0.998 to 1.000)	0.024	0.999 (0.998 to 1.000)	0.030		
X - 11315	Hypertension	24	1.003 (0.998 to 1.008)	0.235	1.001 (0.999 to 1.004)	0.376	1.001 (0.999 to 1.003)	0.298		

Exposure	Outcome	nSNP	MR Egger		Weighted median		Inverse variance weighted		MR_PRESSO	
			OR (95% CI)	pval	OR (95% CI)	pval	OR (95% CI)	pval	OR (95% CI)	pval
X - 23765	Hypertension	19	0.997 (0.989 to 1.004)	0.422	0.998 (0.995 to 1.002)	0.338	0.999 (0.996 to 1.003)	0.661	1.000 (0.997 to 1.003)	0.939
X - 23782	Hypertension	13	1.002 (0.991 to 1.013)	0.747	0.999 (0.994 to 1.003)	0.477	1.001 (0.997 to 1.005)	0.644		
X - 23788	Hypertension	13	0.997 (0.992 to 1.002)	0.283	0.999 (0.996 to 1.003)	0.756	1.001 (0.998 to 1.004)	0.384		
anthranilate	Hypertension	16	0.997 (0.990 to 1.004)	0.388	1.001 (0.997 to 1.004)	0.687	1.000 (0.997 to 1.003)	0.848		
X - 23314	Hypertension	25	1.002 (0.998 to 1.006)	0.280	1.002 (0.999 to 1.004)	0.193	1.000 (0.998 to 1.002)	0.940	1.000 (0.999 to 1.002)	0.595
dimethylglycine	Hypertension	27	1.000 (0.998 to 1.003)	0.776	1.001 (0.999 to 1.002)	0.535	1.000 (0.999 to 1.002)	0.804	1.000 (0.999 to 1.001)	0.837

Exposure	Outcome	nSNP	MR Egger		Weighted median		Inverse variance weighted		MR_PRESSO	
			OR (95% CI)	pval	OR (95% CI)	pval	OR (95% CI)	pval	OR (95% CI)	pval
creatinine	Hypertension	22	0.999 (0.992 to 1.006)	0.747	1.001 (0.998 to 1.005)	0.345	0.999 (0.996 to 1.002)	0.433	1.000 (0.997 to 1.002)	0.811
X - 23997	Hypertension	17	0.998 (0.990 to 1.007)	0.690	1.002 (0.998 to 1.005)	0.330	0.999 (0.995 to 1.003)	0.586	1.001 (0.998 to 1.003)	0.549
X - 24021	Hypertension	15	0.990 (0.975 to 1.004)	0.191	0.997 (0.994 to 1.001)	0.192	0.997 (0.995 to 1.000)	0.054		
X - 24061	Hypertension	24	1.003 (1.000 to 1.007)	0.100	0.999 (0.996 to 1.002)	0.554	0.998 (0.996 to 1.000)	0.087		
X - 24065	Hypertension	15	0.995 (0.988 to 1.001)	0.119	0.998 (0.995 to 1.002)	0.333	1.000 (0.998 to 1.002)	0.991		
X - 24071	Hypertension	23	1.005 (0.996 to 1.014)	0.320	0.999 (0.995 to 1.002)	0.455	0.998 (0.994 to 1.001)	0.191	1.000 (0.996 to 1.003)	0.767

Exposure	Outcome	nSNP	MR Egger		Weighted median		Inverse variance weighted		MR_PRESSO	
			OR (95% CI)	pval	OR (95% CI)	pval	OR (95% CI)	pval	OR (95% CI)	pval
X - 24097	Hypertension	23	0.995 (0.989 to 1.001)	0.137	0.999 (0.995 to 1.002)	0.421	0.998 (0.995 to 1.001)	0.172		
X - 24125	Hypertension	24	1.003 (0.993 to 1.013)	0.596	1.002 (0.999 to 1.005)	0.193	1.000 (0.997 to 1.004)	0.892	1.002 (0.999 to 1.005)	0.222
X - 24240	Hypertension	29	1.003 (0.996 to 1.011)	0.382	1.001 (0.998 to 1.004)	0.461	1.002 (0.999 to 1.005)	0.179	1.002 (1.000 to 1.004)	0.138
X - 24241	Hypertension	26	1.000 (0.994 to 1.005)	0.893	1.002 (0.999 to 1.005)	0.111	1.001 (0.998 to 1.003)	0.627	1.001 (0.999 to 1.003)	0.400
2-hydroxybutyrate/2-hydroxyisobutyrate	Hypertension	15	0.996 (0.990 to 1.001)	0.166	1.002 (0.998 to 1.006)	0.353	1.001 (0.998 to 1.005)	0.391		
2-methylcitrate/homocitrate	Hypertension	14	0.998 (0.991 to 1.005)	0.606	1.002 (0.998 to 1.006)	0.334	1.001 (0.997 to 1.005)	0.688	1.000 (0.997 to 1.003)	0.876

Exposure	Outcome	nSNP	MR Egger		Weighted median		Inverse variance weighted		MR_PRESSO	
			OR (95% CI)	pval	OR (95% CI)	pval	OR (95% CI)	pval	OR (95% CI)	pval
X - 24242	Hypertension	16	0.997 (0.993 to 1.002)	0.292	0.997 (0.993 to 1.001)	0.202	0.997 (0.995 to 1.000)	0.026	0.999 (0.997 to 1.002)	0.673
oleate/vaccenate (18:1)	Hypertension	28	1.000 (0.998 to 1.002)	0.999	1.000 (0.998 to 1.002)	0.932	1.000 (0.999 to 1.001)	0.905		
2-hydroxyglutarate	Hypertension	18	1.004 (0.992 to 1.016)	0.497	1.001 (0.998 to 1.005)	0.472	1.000 (0.996 to 1.004)	0.993	1.000 (0.997 to 1.004)	0.787
X - 24293	Hypertension	12	0.998 (0.991 to 1.005)	0.566	0.999 (0.995 to 1.004)	0.732	0.998 (0.994 to 1.001)	0.122		
cis-aconitate	Hypertension	14	1.001 (0.991 to 1.010)	0.893	1.002 (0.997 to 1.006)	0.484	1.001 (0.997 to 1.005)	0.653	0.999 (0.996 to 1.002)	0.597
X - 24278	Hypertension	52	1.000 (0.999 to 1.002)	0.657	1.000 (0.999 to 1.001)	0.380	1.000 (0.999 to 1.001)	0.796	1.000 (0.999 to 1.001)	0.905

Exposure	Outcome	nSNP	MR Egger		Weighted median		Inverse variance weighted		MR_PRESSO	
			OR (95% CI)	pval	OR (95% CI)	pval	OR (95% CI)	pval	OR (95% CI)	pval
leucylphenylalanine/isoleucylphenylalanine	Hypertension	16	0.999 (0.993 to 1.005)	0.761	0.998 (0.995 to 1.001)	0.167	0.996 (0.993 to 0.999)	0.014	0.996 (0.992 to 1.000)	0.084
1-palmitoleoylglycerol (16:1)	Hypertension	17	1.001 (0.993 to 1.010)	0.768	1.002 (0.999 to 1.006)	0.226	1.002 (0.999 to 1.006)	0.241	1.001 (0.998 to 1.004)	0.452
sphingomyelin (d18:1/15:0, d16:1/17:0)	Hypertension	21	0.991 (0.985 to 0.998)	0.016	0.998 (0.994 to 1.001)	0.194	0.997 (0.994 to 0.999)	0.015		
palmitoyl dihydrosphingomyelin (d18:0/16:0)	Hypertension	15	1.000 (0.988 to 1.013)	0.941	1.000 (0.996 to 1.004)	0.967	1.003 (0.998 to 1.008)	0.311	1.000 (0.997 to 1.003)	0.981
sphingomyelin (d18:2/23:0, d18:1/23:1, d17:1/24:1)	Hypertension	21	0.999 (0.993 to 1.005)	0.727	1.002 (0.998 to 1.005)	0.296	1.001 (0.999 to 1.003)	0.413		
sphingomyelin (d18:2/24:1, d18:1/24:2)	Hypertension	23	0.997 (0.993 to 1.001)	0.155	1.000 (0.997 to 1.002)	0.791	1.000 (0.998 to 1.002)	0.965		

Exposure	Outcome	nSNP	MR Egger		Weighted median		Inverse variance weighted		MR_PRESSO	
			OR (95% CI)	pval	OR (95% CI)	pval	OR (95% CI)	pval	OR (95% CI)	pval
1-stearoyl-2-oleoyl-GPC (18:0/18:1)	Hypertension	18	0.998 (0.994 to 1.003)	0.513	1.000 (0.998 to 1.003)	0.675	1.001 (0.998 to 1.003)	0.557	1.000 (0.998 to 1.002)	0.969
1-stearoyl-2-linoleoyl-GPE (18:0/18:2)	Hypertension	64	1.001 (0.998 to 1.004)	0.459	1.001 (1.000 to 1.002)	0.009	1.001 (1.000 to 1.002)	0.012		
1-stearoyl-2-arachidonoyl-GPE (18:0/20:4)	Hypertension	29	1.001 (0.997 to 1.005)	0.627	1.000 (0.999 to 1.002)	0.896	1.000 (0.999 to 1.002)	0.438		
1-stearoyl-2-arachidonoyl-GPI (18:0/20:4)	Hypertension	50	1.000 (0.994 to 1.006)	0.965	0.998 (0.997 to 1.000)	0.010	0.999 (0.998 to 1.000)	0.075		
1-palmitoyl-2-linoleoyl-GPI (16:0/18:2)	Hypertension	53	0.996 (0.992 to 1.000)	0.085	1.002 (1.000 to 1.003)	0.013	1.001 (1.000 to 1.002)	0.034		
1-stearoyl-2-linoleoyl-GPC (18:0/18:2)	Hypertension	49	1.000 (0.996 to 1.004)	0.879	1.002 (1.000 to 1.004)	0.015	1.001 (1.000 to 1.003)	0.055		

Exposure	Outcome	nSNP	MR Egger		Weighted median		Inverse variance weighted		MR_PRESSO	
			OR (95% CI)	pval	OR (95% CI)	pval	OR (95% CI)	pval	OR (95% CI)	pval
1-oleoyl-2-linoleoyl-GPC (18:1/18:2)	Hypertension	49	0.995 (0.991 to 1.000)	0.078	1.002 (1.000 to 1.003)	0.016	1.001 (1.000 to 1.002)	0.132		
1-palmitoyl-2-dihomo-linolenoyl-GPC (16:0/20:3n3 or 6)	Hypertension	42	1.000 (0.999 to 1.001)	0.971	1.001 (1.000 to 1.002)	0.225	1.000 (1.000 to 1.001)	0.348		
1-palmitoyl-2-oleoyl-GPC (16:0/18:1)	Hypertension	18	1.007 (1.001 to 1.013)	0.036	1.001 (0.997 to 1.004)	0.771	1.001 (0.999 to 1.004)	0.341		
1-palmitoyl-2-arachidonoyl-GPC (16:0/20:4)	Hypertension	50	0.999 (0.997 to 1.001)	0.367	0.999 (0.999 to 1.000)	0.018	0.999 (0.999 to 1.000)	0.018		
1-palmitoyl-2-eicosapentaenoyl-GPC (16:0/20:5)	Hypertension	48	0.998 (0.993 to 1.003)	0.407	0.998 (0.996 to 1.000)	0.013	0.999 (0.997 to 1.000)	0.040		
1-palmitoyl-2-arachidonoyl-GPE (16:0/20:4)	Hypertension	26	1.000 (0.999 to 1.002)	0.736	1.000 (0.999 to 1.001)	0.855	1.000 (0.999 to 1.001)	0.830		

Exposure	Outcome	nSNP	MR Egger		Weighted median		Inverse variance weighted		MR_PRESSO	
			OR (95% CI)	pval	OR (95% CI)	pval	OR (95% CI)	pval	OR (95% CI)	pval
1-palmitoyl-2-docosahexaenoyl-GPE (16:0/22:6)	Hypertension	46	0.999 (0.996 to 1.001)	0.388	1.000 (0.998 to 1.001)	0.796	1.000 (0.999 to 1.001)	0.950	1.000 (0.999 to 1.001)	0.916
1-stearoyl-2-docosahexaenoyl-GPE (18:0/22:6)	Hypertension	35	1.000 (0.994 to 1.005)	0.863	1.000 (0.998 to 1.001)	0.612	0.999 (0.998 to 1.001)	0.526	0.999 (0.998 to 1.001)	0.392
1-palmitoyl-2-arachidonoyl-GPI (16:0/20:4)	Hypertension	38	1.000 (0.996 to 1.004)	0.852	0.999 (0.997 to 1.001)	0.388	1.000 (0.999 to 1.002)	0.893		
1-stearoyl-2-linoleoyl-GPI (18:0/18:2)	Hypertension	60	0.999 (0.995 to 1.003)	0.669	1.002 (1.000 to 1.003)	0.012	1.001 (1.000 to 1.002)	0.078		
1-palmitoyl-2-palmitoleoyl-GPC (16:0/16:1)	Hypertension	20	0.999 (0.989 to 1.008)	0.763	0.999 (0.995 to 1.003)	0.566	0.999 (0.995 to 1.002)	0.503		
gamma-tocopherol/beta-tocopherol	Hypertension	19	1.001 (0.992 to 1.010)	0.827	1.001 (0.997 to 1.005)	0.618	0.999 (0.996 to 1.002)	0.400		

Exposure	Outcome	nSNP	MR Egger		Weighted median		Inverse variance weighted		MR_PRESSO	
			OR (95% CI)	pval	OR (95% CI)	pval	OR (95% CI)	pval	OR (95% CI)	pval
1-(1-enyl-stearoyl)-2-arachidonoyl-GPE (P-18:0/20:4)	Hypertension	43	0.998 (0.993 to 1.003)	0.402	0.998 (0.997 to 1.000)	0.017	0.999 (0.998 to 1.000)	0.040		
1-(1-enyl-stearoyl)-2-docosahexaenoyl-GPE (P-18:0/22:6)	Hypertension	25	0.999 (0.995 to 1.003)	0.511	0.998 (0.996 to 1.001)	0.137	0.998 (0.996 to 1.000)	0.023		
1-(1-enyl-palmitoyl)-2-oleoyl-GPE (P-16:0/18:1)	Hypertension	29	0.995 (0.990 to 1.001)	0.090	0.998 (0.995 to 1.001)	0.124	0.999 (0.996 to 1.002)	0.433	0.998 (0.996 to 1.000)	0.098
1-(1-enyl-palmitoyl)-2-oleoyl-GPC (P-16:0/18:1)	Hypertension	28	0.998 (0.992 to 1.005)	0.628	0.998 (0.995 to 1.001)	0.215	0.999 (0.996 to 1.002)	0.345	0.998 (0.995 to 1.000)	0.097
sphingomyelin (d18:1/21:0, d17:1/22:0, d16:1/23:0)	Hypertension	30	0.994 (0.989 to 0.999)	0.030	0.999 (0.997 to 1.002)	0.669	0.998 (0.996 to 1.000)	0.111	0.998 (0.996 to 1.001)	0.176
1-pentadecanoyl-2-linoleoyl-GPC (15:0/18:2)	Hypertension	49	1.000 (0.995 to 1.004)	0.852	1.002 (1.001 to 1.004)	0.012	1.002 (1.000 to 1.003)	0.012		

Exposure	Outcome	nSNP	MR Egger		Weighted median		Inverse variance weighted		MR_PRESSO	
			OR (95% CI)	pval	OR (95% CI)	pval	OR (95% CI)	pval	OR (95% CI)	pval
1-pentadecanoyl-2-oleoyl-GPC (15:0/18:1)	Hypertension	11	1.005 (0.995 to 1.016)	0.353	1.000 (0.996 to 1.005)	0.849	1.002 (0.997 to 1.007)	0.449	1.002 (0.999 to 1.005)	0.170
1-margaroyl-2-oleoyl-GPC (17:0/18:1)	Hypertension	18	1.010 (1.002 to 1.018)	0.020	1.003 (1.000 to 1.007)	0.049	1.001 (0.998 to 1.003)	0.679		
1-margaroyl-2-linoleoyl-GPC (17:0/18:2)	Hypertension	30	0.999 (0.993 to 1.004)	0.576	1.003 (1.000 to 1.005)	0.039	1.001 (0.999 to 1.003)	0.384		
X - 24309	Hypertension	32	1.000 (0.999 to 1.002)	0.683	1.000 (1.000 to 1.001)	0.435	1.001 (1.000 to 1.002)	0.297	1.000 (1.000 to 1.001)	0.376
2'-deoxyuridine	Hypertension	24	1.000 (0.997 to 1.004)	0.861	1.003 (1.000 to 1.005)	0.038	1.002 (1.000 to 1.004)	0.075		
1,2-dilinoleoyl-GPC (18:2/18:2)	Hypertension	49	0.995 (0.991 to 1.000)	0.047	1.002 (1.000 to 1.003)	0.020	1.001 (1.000 to 1.002)	0.029		

Exposure	Outcome	nSNP	MR Egger		Weighted median		Inverse variance weighted		MR_PRESSO	
			OR (95% CI)	pval	OR (95% CI)	pval	OR (95% CI)	pval	OR (95% CI)	pval
1-palmitoyl-2-docosahexaenoyl-GPC (16:0/22:6)	Hypertension	23	1.000 (0.994 to 1.005)	0.884	0.999 (0.996 to 1.002)	0.627	0.999 (0.997 to 1.001)	0.367		
1-stearoyl-2-docosahexaenoyl-GPC (18:0/22:6)	Hypertension	21	0.998 (0.992 to 1.004)	0.557	0.999 (0.995 to 1.002)	0.493	0.999 (0.996 to 1.001)	0.271		
1-(1-enyl-stearoyl)-2-oleoyl-GPC (P-18:0/18:1)	Hypertension	21	1.000 (0.994 to 1.007)	0.895	1.002 (0.998 to 1.005)	0.305	1.002 (0.999 to 1.005)	0.257	1.001 (0.998 to 1.003)	0.528
1-(1-enyl-stearoyl)-2-arachidonoyl-GPC (P-18:0/20:4)	Hypertension	44	0.999 (0.996 to 1.001)	0.262	0.999 (0.998 to 1.000)	0.011	0.999 (0.998 to 1.000)	0.005		
1-(1-enyl-stearoyl)-2-oleoyl-GPE (P-18:0/18:1)	Hypertension	15	1.002 (0.999 to 1.004)	0.300	1.000 (0.998 to 1.002)	0.932	1.000 (0.999 to 1.002)	0.897	0.999 (0.998 to 1.001)	0.310
sphingomyelin (d18:1/17:0, d17:1/18:0, d19:1/16:0)	Hypertension	18	1.001 (0.995 to 1.007)	0.664	0.999 (0.995 to 1.002)	0.550	0.999 (0.997 to 1.001)	0.433		

Exposure	Outcome	nSNP	MR Egger		Weighted median		Inverse variance weighted		MR_PRESSO	
			OR (95% CI)	pval	OR (95% CI)	pval	OR (95% CI)	pval	OR (95% CI)	pval
1-palmitoyl-2-stearoyl-GPC (16:0/18:0)	Hypertension	39	1.000 (0.999 to 1.002)	0.701	1.001 (0.999 to 1.002)	0.503	1.000 (0.999 to 1.001)	0.375		
1-stearoyl-2-dihomo-linolenoyl-GPC (18:0/20:3n3 or 6)	Hypertension	23	1.001 (0.997 to 1.006)	0.574	1.002 (0.999 to 1.004)	0.233	1.001 (0.999 to 1.003)	0.365		
1-(1-enyl-palmitoyl)-2-docosahexaenoyl-GPC (P-16:0/22:6)	Hypertension	24	1.000 (0.994 to 1.005)	0.916	0.997 (0.994 to 1.000)	0.051	0.998 (0.996 to 1.001)	0.219		
palmitoleoyl-oleoyl-glycerol (16:1/18:1) [2]	Hypertension	36	1.004 (1.000 to 1.009)	0.054	1.003 (1.001 to 1.006)	0.004	1.003 (1.001 to 1.005)	0.013	1.003 (1.001 to 1.004)	8.38e-04
palmitoleoyl-oleoyl-glycerol (16:1/18:1) [1]	Hypertension	26	0.999 (0.995 to 1.002)	0.512	0.998 (0.996 to 1.001)	0.193	1.000 (0.998 to 1.002)	0.974		
palmitoyl-linoleoyl-glycerol (16:0/18:2) [1]	Hypertension	27	1.003 (0.992 to 1.014)	0.628	1.001 (0.998 to 1.005)	0.511	0.998 (0.993 to 1.002)	0.245	1.000 (0.996 to 1.003)	0.877

Exposure	Outcome	nSNP	MR Egger		Weighted median		Inverse variance weighted		MR_PRESSO	
			OR (95% CI)	pval	OR (95% CI)	pval	OR (95% CI)	pval	OR (95% CI)	pval
palmitoyl-linoleoyl-glycerol (16:0/18:2) [2]	Hypertension	22	0.997 (0.992 to 1.002)	0.206	1.000 (0.998 to 1.002)	0.892	0.998 (0.996 to 1.000)	0.128	0.999 (0.998 to 1.001)	0.505
X - 24425	Hypertension	16	1.004 (0.999 to 1.009)	0.138	1.000 (0.997 to 1.004)	0.823	1.000 (0.998 to 1.003)	0.816		
1-palmitoyl-2-oleoyl-GPI (16:0/18:1)	Hypertension	33	0.993 (0.987 to 0.998)	0.016	1.002 (1.000 to 1.004)	0.070	1.000 (0.999 to 1.002)	0.817		
1-(1-enyl-palmitoyl)-2-docosahexaenoyl-GPE (P-16:0/22:6)	Hypertension	29	0.998 (0.995 to 1.002)	0.421	0.999 (0.997 to 1.002)	0.665	0.999 (0.997 to 1.001)	0.269		
1-(1-enyl-palmitoyl)-2-arachidonoyl-GPE (P-16:0/20:4)	Hypertension	50	0.998 (0.994 to 1.002)	0.413	0.998 (0.997 to 1.000)	0.019	0.999 (0.998 to 1.000)	0.081		
1-(1-enyl-palmitoyl)-2-linoleoyl-GPE (P-16:0/18:2)	Hypertension	50	0.998 (0.994 to 1.003)	0.447	1.002 (1.001 to 1.004)	0.011	1.001 (1.000 to 1.003)	0.028		

Exposure	Outcome	nSNP	MR Egger		Weighted median		Inverse variance weighted		MR_PRESSO	
			OR (95% CI)	pval	OR (95% CI)	pval	OR (95% CI)	pval	OR (95% CI)	pval
1-(1-enyl-palmitoyl)-2-linoleoyl-GPC (P-16:0/18:2)	Hypertension	18	1.002 (0.996 to 1.008)	0.514	0.999 (0.995 to 1.003)	0.589	1.000 (0.997 to 1.003)	0.928		
1-palmitoleoyl-2-linoleoyl-GPC (16:1/18:2)	Hypertension	44	1.000 (0.995 to 1.004)	0.855	1.002 (1.000 to 1.003)	0.014	1.001 (1.000 to 1.002)	0.101		
1-palmitoyl-2-alpha-linolenoyl-GPC (16:0/18:3n3)	Hypertension	51	1.003 (0.999 to 1.006)	0.203	1.001 (1.000 to 1.003)	0.014	1.001 (1.000 to 1.002)	0.088		
1-(1-enyl-palmitoyl)-2-arachidonoyl-GPC (P-16:0/20:4)	Hypertension	49	0.997 (0.995 to 0.999)	0.015	0.999 (0.998 to 1.000)	0.016	0.999 (0.999 to 1.000)	0.022		
1-oleoyl-2-docosahexaenoyl-GPC (18:1/22:6)	Hypertension	15	1.003 (0.993 to 1.014)	0.575	1.000 (0.997 to 1.004)	0.817	1.000 (0.996 to 1.003)	0.861		
1-adrenoyl-GPC (22:4)	Hypertension	46	1.001 (0.996 to 1.005)	0.796	0.998 (0.997 to 1.000)	0.016	0.998 (0.997 to 1.000)	0.007		

Exposure	Outcome	nSNP	MR Egger		Weighted median		Inverse variance weighted		MR_PRESSO	
			OR (95% CI)	pval	OR (95% CI)	pval	OR (95% CI)	pval	OR (95% CI)	pval
1-stearoyl-2-docosapentaenoyl-GPC (18:0/22:5n3)	Hypertension	48	0.995 (0.991 to 1.000)	0.055	0.998 (0.997 to 1.000)	0.015	0.999 (0.998 to 1.000)	0.237		
lactate	Hypertension	21	1.003 (0.996 to 1.009)	0.408	0.998 (0.995 to 1.001)	0.194	0.997 (0.993 to 1.000)	0.048	0.998 (0.996 to 1.001)	0.219
1-stearoyl-2-docosapentaenoyl-GPC (18:0/22:5n6)	Hypertension	48	0.998 (0.994 to 1.002)	0.380	0.998 (0.996 to 1.000)	0.011	0.999 (0.998 to 1.000)	0.081		
1-(1-enyl-stearoyl)-GPC (P-18:0)	Hypertension	15	0.999 (0.992 to 1.005)	0.679	1.001 (0.997 to 1.004)	0.764	1.003 (1.000 to 1.006)	0.089		
1-stearyl-GPC (O-18:0)	Hypertension	14	0.999 (0.989 to 1.010)	0.908	1.002 (0.998 to 1.005)	0.408	1.004 (0.999 to 1.009)	0.097	1.005 (1.002 to 1.008)	0.013
1-(1-enyl-stearoyl)-2-docosahexaenoyl-GPC (P-18:0/22:6)	Hypertension	19	0.998 (0.988 to 1.008)	0.682	0.997 (0.994 to 1.001)	0.102	0.998 (0.994 to 1.001)	0.244	0.997 (0.993 to 1.000)	0.066

Exposure	Outcome	nSNP	MR Egger		Weighted median		Inverse variance weighted		MR_PRESSO	
			OR (95% CI)	pval	OR (95% CI)	pval	OR (95% CI)	pval	OR (95% CI)	pval
1-oleoyl-2-dihomo-linolenoyl-GPC (18:1/20:3)	Hypertension	44	0.999 (0.994 to 1.004)	0.834	1.002 (1.000 to 1.003)	0.011	1.001 (1.000 to 1.002)	0.012		
1-palmitoyl-2-adrenoyl-GPC (16:0/22:4)	Hypertension	16	1.002 (0.994 to 1.010)	0.636	0.996 (0.993 to 1.000)	0.051	0.997 (0.992 to 1.001)	0.168	0.997 (0.995 to 1.000)	0.066
1-myristoyl-2-linoleoyl-GPC (14:0/18:2)	Hypertension	50	1.001 (0.997 to 1.005)	0.652	1.002 (1.001 to 1.004)	0.007	1.002 (1.001 to 1.003)	0.003		
1-linoleoyl-2-arachidonoyl-GPC (18:2/20:4n6)	Hypertension	43	0.996 (0.993 to 1.000)	0.036	0.999 (0.998 to 1.000)	0.023	0.999 (0.998 to 1.000)	0.043		
1-oleoyl-2-eicosapentaenoyl-GPC (18:1/20:5)	Hypertension	47	1.000 (0.995 to 1.004)	0.852	0.998 (0.996 to 1.000)	0.014	0.999 (0.998 to 1.000)	0.038		
1-(1-enyl-palmitoyl)-2-palmitoyl-GPC (P-16:0/16:0)	Hypertension	18	1.007 (1.001 to 1.012)	0.027	1.001 (0.998 to 1.005)	0.384	1.001 (0.998 to 1.003)	0.593	0.999 (0.997 to 1.001)	0.401

Exposure	Outcome	nSNP	MR Egger		Weighted median		Inverse variance weighted		MR_PRESSO	
			OR (95% CI)	pval	OR (95% CI)	pval	OR (95% CI)	pval	OR (95% CI)	pval
phosphatidylcholine (16:0/22:5n3, 18:1/20:4)	Hypertension	50	0.997 (0.995 to 0.999)	0.015	0.999 (0.998 to 1.000)	0.019	0.999 (0.999 to 1.000)	0.068		
phosphatidylcholine (18:0/20:5, 16:0/22:5n6)	Hypertension	56	0.996 (0.992 to 1.000)	0.031	0.999 (0.997 to 1.000)	0.016	0.999 (0.998 to 1.000)	0.025		
1-palmitoyl-2-meadoyl-GPC (16:0/20:3n9)	Hypertension	45	1.001 (0.997 to 1.005)	0.634	0.999 (0.998 to 1.000)	0.018	0.999 (0.998 to 1.000)	0.018		
1-stearoyl-2-meadoyl-GPC (18:0/20:3n9)	Hypertension	41	0.997 (0.992 to 1.003)	0.303	0.999 (0.998 to 1.000)	0.025	0.999 (0.998 to 1.000)	0.020		
glutamine	Hypertension	17	1.002 (0.991 to 1.013)	0.781	1.000 (0.996 to 1.003)	0.927	0.999 (0.996 to 1.002)	0.483	0.999 (0.997 to 1.002)	0.684
3-hydroxy-3-methylglutarate	Hypertension	12	0.997 (0.991 to 1.004)	0.433	1.002 (0.998 to 1.006)	0.366	1.001 (0.998 to 1.004)	0.635		

Exposure	Outcome	nSNP	MR Egger		Weighted median		Inverse variance weighted		MR_PRESSO	
			OR (95% CI)	pval	OR (95% CI)	pval	OR (95% CI)	pval	OR (95% CI)	pval
tryptophan	Hypertension	19	0.999 (0.993 to 1.006)	0.849	1.001 (0.998 to 1.005)	0.514	1.003 (0.999 to 1.007)	0.093	1.002 (0.999 to 1.005)	0.230
beta-alanine	Hypertension	15	1.000 (0.996 to 1.004)	0.912	1.000 (0.997 to 1.003)	0.857	0.998 (0.996 to 1.000)	0.068		
adenosine	Hypertension	26	1.000 (0.998 to 1.002)	0.788	1.000 (0.999 to 1.001)	0.960	1.000 (0.999 to 1.001)	0.960		
caffeine	Hypertension	26	1.001 (0.995 to 1.008)	0.693	0.999 (0.996 to 1.002)	0.559	1.000 (0.997 to 1.004)	0.914	0.999 (0.996 to 1.001)	0.315
glutamate	Hypertension	15	1.003 (0.994 to 1.012)	0.548	1.002 (0.999 to 1.006)	0.242	1.002 (0.999 to 1.006)	0.228	1.001 (0.998 to 1.003)	0.548
glycine	Hypertension	13	0.990 (0.976 to 1.004)	0.173	0.998 (0.995 to 1.002)	0.399	0.999 (0.993 to 1.005)	0.817	0.998 (0.995 to 1.002)	0.349

Exposure	Outcome	nSNP	MR Egger		Weighted median		Inverse variance weighted		MR_PRESSO	
			OR (95% CI)	pval	OR (95% CI)	pval	OR (95% CI)	pval	OR (95% CI)	pval
gluconate	Hypertension	19	1.003 (0.998 to 1.007)	0.309	1.001 (0.997 to 1.004)	0.697	1.003 (1.000 to 1.005)	0.029		
histidine	Hypertension	15	0.999 (0.992 to 1.005)	0.694	0.999 (0.995 to 1.003)	0.536	1.000 (0.997 to 1.003)	0.967		
nicotinamide	Hypertension	16	1.003 (0.998 to 1.008)	0.272	0.998 (0.995 to 1.002)	0.370	0.999 (0.996 to 1.001)	0.356		
dihydroorotate	Hypertension	24	0.999 (0.996 to 1.001)	0.407	1.000 (0.998 to 1.001)	0.818	1.000 (0.998 to 1.001)	0.733	1.000 (0.999 to 1.002)	0.493
uridine	Hypertension	15	1.001 (0.989 to 1.014)	0.829	1.002 (0.999 to 1.006)	0.157	1.001 (0.997 to 1.004)	0.764	1.003 (1.001 to 1.005)	0.017
trans-urocanate	Hypertension	13	1.003 (0.996 to 1.009)	0.452	0.997 (0.994 to 1.001)	0.159	0.998 (0.996 to 1.001)	0.166		

Exposure	Outcome	nSNP	MR Egger		Weighted median		Inverse variance weighted		MR_PRESSO	
			OR (95% CI)	pval	OR (95% CI)	pval	OR (95% CI)	pval	OR (95% CI)	pval
2-aminoadipate	Hypertension	27	0.998 (0.993 to 1.004)	0.543	0.999 (0.996 to 1.002)	0.581	0.999 (0.996 to 1.001)	0.285	0.999 (0.997 to 1.002)	0.510
cholesterol	Hypertension	18	0.999 (0.992 to 1.005)	0.665	1.003 (0.999 to 1.007)	0.094	1.001 (0.998 to 1.005)	0.512	1.001 (0.999 to 1.004)	0.335
Carnitine	Hypertension	15	1.000 (0.990 to 1.009)	0.966	1.004 (1.000 to 1.008)	0.053	1.006 (1.000 to 1.012)	0.048	1.005 (1.000 to 1.010)	0.083
Benzoate	Hypertension	7	1.005 (0.987 to 1.023)	0.613	0.999 (0.989 to 1.010)	0.898	1.001 (0.993 to 1.009)	0.812		
Maltotriose	Hypertension	16	0.996 (0.988 to 1.004)	0.383	0.998 (0.992 to 1.003)	0.398	0.998 (0.994 to 1.002)	0.405		
Hippurate	Hypertension	10	1.005 (0.988 to 1.021)	0.598	1.001 (0.992 to 1.010)	0.828	1.001 (0.994 to 1.008)	0.872		

Exposure	Outcome	nSNP	MR Egger		Weighted median		Inverse variance weighted		MR_PRESSO	
			OR (95% CI)	pval	OR (95% CI)	pval	OR (95% CI)	pval	OR (95% CI)	pval
Methionine sulfoxide	Hypertension	10	0.977 (0.936 to 1.020)	0.327	1.002 (0.990 to 1.014)	0.769	1.007 (0.991 to 1.024)	0.393	1.005 (0.997 to 1.013)	0.284
Xanthurenate	Hypertension	17	0.999 (0.974 to 1.025)	0.968	0.997 (0.989 to 1.005)	0.446	0.998 (0.990 to 1.007)	0.694	0.997 (0.991 to 1.004)	0.413
N-alpha-acetylornithine	Hypertension	15	0.993 (0.980 to 1.006)	0.294	0.995 (0.988 to 1.002)	0.133	0.996 (0.990 to 1.001)	0.123	0.992 (0.987 to 0.996)	0.002
3-methylhistidine	Hypertension	15	0.994 (0.974 to 1.014)	0.566	1.004 (0.996 to 1.013)	0.322	1.002 (0.995 to 1.009)	0.571		
Glycerophosphorylcholine (GPC)	Hypertension	13	0.995 (0.978 to 1.013)	0.598	1.000 (0.992 to 1.009)	0.933	1.002 (0.995 to 1.009)	0.660		
Phenylacetate	Hypertension	14	1.011 (1.000 to 1.022)	0.073	1.003 (0.996 to 1.010)	0.417	1.007 (1.001 to 1.013)	0.033		

Exposure	Outcome	nSNP	MR Egger		Weighted median		Inverse variance weighted		MR_PRESSO	
			OR (95% CI)	pval	OR (95% CI)	pval	OR (95% CI)	pval	OR (95% CI)	pval
3-methyl-2-oxovalerate	Hypertension	8	0.992 (0.973 to 1.011)	0.437	0.999 (0.989 to 1.010)	0.918	1.001 (0.993 to 1.009)	0.815		
Tartarate	Hypertension	14	0.997 (0.983 to 1.011)	0.647	0.996 (0.988 to 1.004)	0.327	0.997 (0.990 to 1.004)	0.375		
Suberate (C8-DC)	Hypertension	14	0.992 (0.974 to 1.011)	0.417	0.998 (0.990 to 1.007)	0.721	1.001 (0.993 to 1.009)	0.775	0.999 (0.992 to 1.006)	0.746
5-hydroxylysine	Hypertension	10	0.999 (0.992 to 1.006)	0.773	1.004 (1.000 to 1.009)	0.046	1.006 (0.999 to 1.012)	0.073		
3-phenylpropionate hydrocinnamate	Hypertension	15	1.009 (0.990 to 1.027)	0.376	1.004 (0.996 to 1.013)	0.298	1.004 (0.997 to 1.012)	0.262	1.007 (1.000 to 1.014)	0.069
Kynurenine	Hypertension	14	0.952 (0.886 to 1.022)	0.202	1.002 (0.995 to 1.010)	0.507	1.023 (1.000 to 1.047)	0.049	1.012 (1.005 to 1.018)	0.007

Exposure	Outcome	nSNP	MR Egger		Weighted median		Inverse variance weighted		MR_PRESSO	
			OR (95% CI)	pval	OR (95% CI)	pval	OR (95% CI)	pval	OR (95% CI)	pval
N-acetylglutamate	Hypertension	12	0.999 (0.975 to 1.025)	0.969	0.999 (0.989 to 1.009)	0.831	1.006 (0.995 to 1.016)	0.274	1.002 (0.994 to 1.009)	0.640
Glycerol 3-phosphate	Hypertension	17	1.004 (0.994 to 1.015)	0.410	1.001 (0.995 to 1.007)	0.772	1.002 (0.997 to 1.008)	0.431		
Imidazole lactate	Hypertension	16	1.000 (0.992 to 1.008)	0.998	0.999 (0.995 to 1.003)	0.701	1.001 (0.996 to 1.006)	0.696	1.000 (0.997 to 1.003)	0.924
4-guanidinobutanoate	Hypertension	11	0.968 (0.888 to 1.056)	0.481	0.990 (0.980 to 1.001)	0.063	0.984 (0.959 to 1.010)	0.221	0.992 (0.984 to 1.000)	0.079
Glucuronate	Hypertension	7	0.999 (0.978 to 1.019)	0.894	1.007 (0.995 to 1.019)	0.271	1.006 (0.997 to 1.015)	0.190		
Ribitol	Hypertension	16	1.003 (0.997 to 1.010)	0.344	1.005 (0.999 to 1.011)	0.085	1.002 (0.998 to 1.006)	0.346		

Exposure	Outcome	nSNP	MR Egger		Weighted median		Inverse variance weighted		MR_PRESSO	
			OR (95% CI)	pval	OR (95% CI)	pval	OR (95% CI)	pval	OR (95% CI)	pval
Glycodeoxycholate	Hypertension	6	1.019 (1.003 to 1.035)	0.083	1.005 (0.994 to 1.016)	0.381	0.999 (0.988 to 1.011)	0.884		
Theobromine	Hypertension	13	0.998 (0.980 to 1.017)	0.856	0.999 (0.992 to 1.007)	0.855	0.996 (0.990 to 1.003)	0.260		
Quinate	Hypertension	11	0.999 (0.972 to 1.027)	0.954	0.996 (0.987 to 1.005)	0.400	0.993 (0.979 to 1.007)	0.317	0.993 (0.987 to 1.000)	0.083
Gentisate	Hypertension	19	1.001 (0.986 to 1.017)	0.887	1.002 (0.995 to 1.010)	0.531	1.000 (0.993 to 1.006)	0.944		
Theophylline	Hypertension	12	1.003 (0.990 to 1.017)	0.632	1.003 (0.994 to 1.012)	0.503	1.005 (0.998 to 1.011)	0.148		
1,5-anhydroglucitol (1,5-ag)	Hypertension	21	0.994 (0.981 to 1.008)	0.442	0.996 (0.989 to 1.002)	0.168	0.999 (0.992 to 1.007)	0.820	0.998 (0.994 to 1.002)	0.351

Exposure	Outcome	nSNP	MR Egger		Weighted median		Inverse variance weighted		MR_PRESSO	
			OR (95% CI)	pval	OR (95% CI)	pval	OR (95% CI)	pval	OR (95% CI)	pval
1-stearoyl-2-arachidonoyl-GPI (18:0/20:4)	Hypertension	16	0.999 (0.984 to 1.015)	0.931	0.999 (0.994 to 1.004)	0.587	1.002 (0.996 to 1.008)	0.544	0.998 (0.994 to 1.003)	0.513
Indolelactate	Hypertension	12	1.003 (0.991 to 1.015)	0.601	0.997 (0.989 to 1.005)	0.463	0.998 (0.993 to 1.004)	0.490		
Docosahexaenoate DHA; 22:6n3	Hypertension	9	0.990 (0.968 to 1.013)	0.418	1.001 (0.990 to 1.011)	0.907	1.006 (0.997 to 1.014)	0.194		
1-stearoyl-2-oleoyl-GPS (18:0/18:1)	Hypertension	9	0.988 (0.954 to 1.024)	0.535	1.013 (1.004 to 1.023)	0.007	1.014 (1.002 to 1.027)	0.027	1.012 (1.006 to 1.019)	0.007
1-myristoyl-2-palmitoyl-gpc (14:0/16:0)	Hypertension	9	0.987 (0.954 to 1.021)	0.470	1.000 (0.989 to 1.011)	0.956	0.995 (0.984 to 1.007)	0.456	1.000 (0.993 to 1.008)	0.937
Paraxanthine	Hypertension	17	0.995 (0.983 to 1.007)	0.406	1.001 (0.994 to 1.008)	0.714	1.002 (0.997 to 1.007)	0.451		

Exposure	Outcome	nSNP	MR Egger		Weighted median		Inverse variance weighted		MR_PRESSO	
			**OR (95% CI)**	**pval**	**OR (95% CI)**	**pval**	**OR (95% CI)**	**pval**	**OR (95% CI)**	**pval**
4-methyl-2-oxopentanoate	Hypertension	10	0.980 (0.956 to 1.006)	0.164	1.003 (0.993 to 1.014)	0.562	1.003 (0.994 to 1.013)	0.505		
3-indoxyl sulfate	Hypertension	11	0.998 (0.978 to 1.019)	0.849	1.002 (0.993 to 1.010)	0.668	0.999 (0.990 to 1.008)	0.750		
Sphingosine 1-phosphate	Hypertension	11	1.007 (0.988 to 1.026)	0.489	0.994 (0.985 to 1.004)	0.245	0.997 (0.989 to 1.005)	0.449		
Alpha-hydroxyisocaproate	Hypertension	14	1.006 (0.981 to 1.031)	0.655	1.003 (0.995 to 1.012)	0.437	0.999 (0.992 to 1.006)	0.833		
1-stearoyl-GPI (18:0)	Hypertension	11	1.009 (0.990 to 1.028)	0.387	1.004 (0.995 to 1.014)	0.384	1.008 (1.000 to 1.016)	0.047		
1,2-dipalmitoyl-gpc (16:0/16:0)	Hypertension	13	1.009 (0.987 to 1.032)	0.450	1.004 (0.996 to 1.012)	0.323	1.006 (0.997 to 1.015)	0.174	0.999 (0.989 to 1.010)	0.907

Exposure	Outcome	nSNP	MR Egger		Weighted median		Inverse variance weighted		MR_PRESSO	
			OR (95% CI)	pval	OR (95% CI)	pval	OR (95% CI)	pval	OR (95% CI)	pval
Maleate	Hypertension	12	0.990 (0.964 to 1.017)	0.466	1.001 (0.992 to 1.011)	0.774	0.994 (0.982 to 1.006)	0.359	1.002 (0.995 to 1.010)	0.536
Isovalerate (i5:0)	Hypertension	9	1.011 (0.984 to 1.039)	0.469	1.007 (0.996 to 1.019)	0.190	1.004 (0.993 to 1.014)	0.502		
4-acetylphenol sulfate	Hypertension	10	1.010 (0.988 to 1.032)	0.413	1.006 (0.996 to 1.015)	0.227	1.005 (0.995 to 1.015)	0.328	1.008 (1.000 to 1.016)	0.084
Phenyllactate (PLA)	Hypertension	20	0.994 (0.987 to 1.002)	0.172	0.997 (0.991 to 1.003)	0.347	0.998 (0.993 to 1.002)	0.307		
N-acetylaspartate (naa)	Hypertension	6	0.990 (0.943 to 1.040)	0.720	1.000 (0.988 to 1.013)	0.939	1.005 (0.989 to 1.021)	0.536	0.998 (0.989 to 1.007)	0.658
Palmitoylcarnitine	Hypertension	18	0.995 (0.979 to 1.010)	0.513	1.002 (0.995 to 1.010)	0.574	0.999 (0.993 to 1.005)	0.783		

Exposure	Outcome	nSNP	MR Egger		Weighted median		Inverse variance weighted		MR_PRESSO	
			OR (95% CI)	pval	OR (95% CI)	pval	OR (95% CI)	pval	OR (95% CI)	pval
Erythritol	Hypertension	17	0.988 (0.963 to 1.013)	0.361	0.993 (0.984 to 1.002)	0.131	1.003 (0.991 to 1.016)	0.633	0.998 (0.991 to 1.006)	0.684
Hexanoylcarnitine	Hypertension	13	1.002 (0.997 to 1.008)	0.413	1.003 (1.000 to 1.007)	0.080	1.004 (1.001 to 1.007)	0.012		
Acetylcarnitine	Hypertension	12	1.005 (0.990 to 1.019)	0.554	1.003 (0.995 to 1.011)	0.526	0.999 (0.992 to 1.006)	0.683		
Iminodiacetate (IDA)	Hypertension	10	1.006 (0.986 to 1.026)	0.569	0.998 (0.989 to 1.007)	0.666	0.999 (0.992 to 1.006)	0.713		
Cysteine s-sulfate	Hypertension	11	1.009 (0.990 to 1.027)	0.385	0.999 (0.990 to 1.009)	0.916	1.004 (0.995 to 1.013)	0.402		
Oxalate (ethanedioate)	Hypertension	14	0.993 (0.982 to 1.004)	0.233	0.995 (0.987 to 1.003)	0.209	0.998 (0.991 to 1.004)	0.468		

Exposure	Outcome	nSNP	MR Egger		Weighted median		Inverse variance weighted		MR_PRESSO	
			OR (95% CI)	pval	OR (95% CI)	pval	OR (95% CI)	pval	OR (95% CI)	pval
DHEAS	Hypertension	27	1.005 (0.985 to 1.026)	0.604	1.006 (0.998 to 1.013)	0.125	1.003 (0.993 to 1.013)	0.612	1.001 (0.995 to 1.007)	0.672
3-hydroxymyristate	Hypertension	7	1.005 (0.985 to 1.026)	0.626	1.001 (0.989 to 1.013)	0.868	0.996 (0.987 to 1.006)	0.444		
Homoarginine	Hypertension	18	1.004 (0.981 to 1.027)	0.755	0.995 (0.990 to 1.001)	0.115	1.000 (0.989 to 1.011)	0.970	1.000 (0.992 to 1.008)	0.954
1-oleoylglycerol (18:1)	Hypertension	17	1.007 (0.982 to 1.034)	0.580	0.995 (0.986 to 1.004)	0.265	0.993 (0.984 to 1.003)	0.187	0.991 (0.984 to 0.999)	0.040
3-methyl-2-oxobutyrate	Hypertension	13	1.008 (0.992 to 1.024)	0.366	1.000 (0.992 to 1.008)	0.972	1.004 (0.998 to 1.009)	0.214		
Tartronate (hydroxymalonate)	Hypertension	11	1.008 (0.989 to 1.027)	0.417	0.998 (0.989 to 1.007)	0.616	0.996 (0.989 to 1.004)	0.344		

Exposure	Outcome	nSNP	MR Egger		Weighted median		Inverse variance weighted		MR_PRESSO	
			OR (95% CI)	pval	OR (95% CI)	pval	OR (95% CI)	pval	OR (95% CI)	pval
2,3-dihydroxypyridine	Hypertension	11	1.015 (0.980 to 1.052)	0.427	0.999 (0.991 to 1.008)	0.884	0.996 (0.985 to 1.006)	0.401	1.000 (0.993 to 1.006)	0.931
3-hydroxyoctanoate	Hypertension	13	1.012 (0.993 to 1.031)	0.243	0.989 (0.981 to 0.996)	0.005	0.990 (0.984 to 0.996)	0.002		
2-hydroxyoctanoate	Hypertension	14	0.990 (0.977 to 1.004)	0.192	1.001 (0.994 to 1.009)	0.697	0.998 (0.992 to 1.004)	0.542		
Homocitrulline	Hypertension	16	0.998 (0.986 to 1.009)	0.711	1.000 (0.994 to 1.006)	0.889	1.000 (0.995 to 1.006)	0.933		
Indoleacetate	Hypertension	21	1.008 (0.997 to 1.019)	0.161	1.002 (0.996 to 1.009)	0.523	1.000 (0.995 to 1.005)	0.944		
1-linoleoylglycerol (18:2)	Hypertension	7	0.977 (0.955 to 1.000)	0.112	1.002 (0.990 to 1.013)	0.795	1.008 (0.997 to 1.019)	0.156		

Exposure	Outcome	nSNP	MR Egger		Weighted median		Inverse variance weighted		MR_PRESSO	
			OR (95% CI)	pval	OR (95% CI)	pval	OR (95% CI)	pval	OR (95% CI)	pval
2-linoleoylglycerol (18:2)	Hypertension	12	1.016 (0.991 to 1.041)	0.252	1.002 (0.994 to 1.010)	0.593	1.003 (0.993 to 1.014)	0.524	1.004 (0.997 to 1.011)	0.285
Indolepropionate	Hypertension	6	0.982 (0.963 to 1.001)	0.134	0.992 (0.983 to 1.001)	0.093	0.992 (0.985 to 0.999)	0.034		
Threonate	Hypertension	14	1.001 (0.997 to 1.004)	0.736	1.001 (0.997 to 1.004)	0.674	1.001 (0.998 to 1.004)	0.522		
N-acetylglycine	Hypertension	16	0.982 (0.974 to 0.990)	9.54e-04	0.986 (0.982 to 0.991)	1.79e-08	0.993 (0.986 to 0.999)	0.019	1.002 (0.995 to 1.008)	0.664
Glycolithocholate	Hypertension	12	1.016 (1.001 to 1.030)	0.061	0.999 (0.991 to 1.007)	0.869	0.999 (0.993 to 1.005)	0.776		
EDTA	Hypertension	8	1.001 (0.978 to 1.026)	0.908	1.006 (0.995 to 1.017)	0.289	1.007 (0.996 to 1.017)	0.204		

Exposure	Outcome	nSNP	MR Egger		Weighted median		Inverse variance weighted		MR_PRESSO	
			OR (95% CI)	pval	OR (95% CI)	pval	OR (95% CI)	pval	OR (95% CI)	pval
1-methylhistidine	Hypertension	19	0.999 (0.981 to 1.016)	0.878	1.001 (0.995 to 1.008)	0.730	1.000 (0.994 to 1.006)	0.899	1.001 (0.996 to 1.006)	0.654
Citramalate	Hypertension	11	1.038 (0.993 to 1.085)	0.130	1.007 (0.997 to 1.016)	0.155	1.001 (0.985 to 1.018)	0.884	1.005 (0.999 to 1.012)	0.161
3-hydroxydecanoate	Hypertension	8	1.002 (0.982 to 1.022)	0.872	0.997 (0.990 to 1.005)	0.471	0.998 (0.990 to 1.007)	0.692		
1,3-dimethylurate	Hypertension	12	1.002 (0.988 to 1.016)	0.801	1.009 (1.001 to 1.016)	0.026	1.008 (1.002 to 1.013)	0.010		
Galactonate	Hypertension	11	0.999 (0.973 to 1.025)	0.916	0.999 (0.989 to 1.009)	0.848	1.002 (0.992 to 1.012)	0.700	1.002 (0.995 to 1.009)	0.576
Trigonelline	Hypertension	9	0.990 (0.972 to 1.008)	0.315	0.991 (0.981 to 1.001)	0.078	0.992 (0.985 to 1.000)	0.050		

Exposure	Outcome	nSNP	MR Egger		Weighted median		Inverse variance weighted		MR_PRESSO	
			OR (95% CI)	pval	OR (95% CI)	pval	OR (95% CI)	pval	OR (95% CI)	pval
Dodecanedioate	Hypertension	16	0.997 (0.985 to 1.010)	0.690	1.002 (0.994 to 1.009)	0.665	0.999 (0.994 to 1.005)	0.750		
Isobutyrylcarnitine (c4)	Hypertension	20	1.000 (0.995 to 1.005)	0.949	1.000 (0.995 to 1.004)	0.877	1.003 (1.000 to 1.006)	0.066		
3-methylxanthine	Hypertension	15	1.000 (0.985 to 1.016)	0.995	0.998 (0.990 to 1.006)	0.618	1.002 (0.995 to 1.008)	0.616		
3-hydroxylaurate	Hypertension	7	1.002 (0.984 to 1.020)	0.832	1.008 (0.998 to 1.019)	0.103	1.004 (0.997 to 1.012)	0.264		
Pyridoxate	Hypertension	14	0.985 (0.966 to 1.005)	0.166	0.998 (0.989 to 1.007)	0.589	0.999 (0.989 to 1.010)	0.901	0.996 (0.988 to 1.004)	0.368
5-hydroxyhexanoate	Hypertension	11	1.007 (0.991 to 1.023)	0.428	0.997 (0.989 to 1.005)	0.489	0.997 (0.990 to 1.003)	0.290		

Exposure	Outcome	nSNP	MR Egger		Weighted median		Inverse variance weighted		MR_PRESSO	
			OR (95% CI)	pval	OR (95% CI)	pval	OR (95% CI)	pval	OR (95% CI)	pval
Gamma-glutamylvaline	Hypertension	15	0.999 (0.997 to 1.001)	0.343	0.999 (0.997 to 1.001)	0.492	1.000 (0.998 to 1.002)	0.860		
3-hydroxysebacate	Hypertension	12	1.005 (0.990 to 1.021)	0.504	0.998 (0.991 to 1.006)	0.648	0.996 (0.988 to 1.003)	0.276	0.993 (0.988 to 0.999)	0.040
Propionylglycine	Hypertension	14	0.985 (0.977 to 0.993)	0.003	0.986 (0.981 to 0.991)	9.89e-09	0.990 (0.986 to 0.995)	8.7e-05		
Butyrylglycine	Hypertension	12	0.999 (0.990 to 1.007)	0.731	0.998 (0.994 to 1.002)	0.237	0.992 (0.987 to 0.998)	0.009	0.989 (0.984 to 0.995)	0.005
Pro-hydroxy-pro	Hypertension	14	1.023 (1.007 to 1.039)	0.014	1.000 (0.991 to 1.008)	0.923	1.002 (0.995 to 1.009)	0.507		
Propionylcarnitine (c3)	Hypertension	11	1.000 (0.987 to 1.014)	0.983	1.006 (0.999 to 1.013)	0.071	1.008 (1.001 to 1.015)	0.031	1.005 (0.999 to 1.011)	0.154

Exposure	Outcome	nSNP	MR Egger		Weighted median		Inverse variance weighted		MR_PRESSO	
			OR (95% CI)	pval	OR (95% CI)	pval	OR (95% CI)	pval	OR (95% CI)	pval
Adrenate (22:4n6)	Hypertension	10	0.993 (0.975 to 1.011)	0.451	0.998 (0.990 to 1.007)	0.712	1.001 (0.992 to 1.009)	0.827		
Docosatrienoate (22:3n3)	Hypertension	14	0.998 (0.983 to 1.013)	0.814	1.004 (0.997 to 1.010)	0.317	1.007 (0.999 to 1.014)	0.077	1.003 (0.998 to 1.009)	0.299
Docosadienoate (22:2n6)	Hypertension	10	1.015 (0.985 to 1.045)	0.364	1.008 (0.998 to 1.018)	0.119	1.010 (1.001 to 1.019)	0.031		
3-carboxy-4-methyl-5-propyl-2-furanpropanoate (cmpf)	Hypertension	10	1.000 (0.975 to 1.027)	0.972	1.008 (0.997 to 1.018)	0.139	0.999 (0.989 to 1.009)	0.848	1.005 (0.999 to 1.012)	0.144
Docosapentaenoate n3 DPA; 22:5n3	Hypertension	12	1.018 (0.981 to 1.058)	0.366	1.004 (0.994 to 1.014)	0.462	0.999 (0.983 to 1.016)	0.940	1.004 (0.996 to 1.011)	0.403
3-hydroxy-2-ethylpropionate	Hypertension	20	0.996 (0.958 to 1.035)	0.836	0.999 (0.992 to 1.007)	0.893	1.006 (0.993 to 1.020)	0.368	1.001 (0.995 to 1.007)	0.817

Exposure	Outcome	nSNP	MR Egger		Weighted median		Inverse variance weighted		MR_PRESSO	
			OR (95% CI)	pval	OR (95% CI)	pval	OR (95% CI)	pval	OR (95% CI)	pval
Myristoleate (14:1n5)	Hypertension	12	0.994 (0.941 to 1.050)	0.828	1.009 (0.998 to 1.019)	0.107	0.991 (0.973 to 1.010)	0.338	1.004 (0.994 to 1.013)	0.461
10-undecenoate (11:1n1)	Hypertension	11	1.000 (0.994 to 1.006)	0.908	1.000 (0.996 to 1.005)	0.908	0.998 (0.994 to 1.002)	0.271		
1-methyl-4-imidazoleacetate	Hypertension	16	0.995 (0.988 to 1.002)	0.205	0.998 (0.994 to 1.003)	0.462	1.000 (0.996 to 1.004)	0.925		
Sebacate (C10-DC)	Hypertension	9	1.001 (0.983 to 1.020)	0.879	1.004 (0.995 to 1.013)	0.361	1.002 (0.996 to 1.008)	0.560		
Tauro-beta-muricholate	Hypertension	14	0.993 (0.988 to 0.998)	0.016	0.994 (0.990 to 0.998)	0.004	0.997 (0.993 to 1.000)	0.072		
Octanoylcarnitine (c8)	Hypertension	10	1.008 (1.001 to 1.015)	0.046	1.004 (0.999 to 1.008)	0.091	1.003 (1.000 to 1.007)	0.074		

Exposure	Outcome	nSNP	MR Egger		Weighted median		Inverse variance weighted		MR_PRESSO	
			OR (95% CI)	pval	OR (95% CI)	pval	OR (95% CI)	pval	OR (95% CI)	pval
Stearidonate (18:4n3)	Hypertension	14	1.011 (0.989 to 1.034)	0.351	1.009 (1.000 to 1.018)	0.054	1.007 (0.998 to 1.017)	0.141	1.010 (1.002 to 1.018)	0.025
Decanoylcarnitine (C10)	Hypertension	11	1.012 (1.002 to 1.021)	0.036	1.004 (0.999 to 1.010)	0.112	1.004 (0.999 to 1.009)	0.119		
5-dodecenoate (12:1n7)	Hypertension	8	0.987 (0.949 to 1.027)	0.550	0.993 (0.982 to 1.005)	0.259	0.988 (0.973 to 1.003)	0.127	0.996 (0.986 to 1.006)	0.452
N-acetylisoleucine	Hypertension	9	0.988 (0.953 to 1.023)	0.515	0.997 (0.986 to 1.009)	0.629	1.000 (0.986 to 1.013)	0.964	0.998 (0.988 to 1.008)	0.687
Campesterol	Hypertension	14	1.006 (0.988 to 1.024)	0.522	1.008 (1.003 to 1.012)	7.8e-04	1.003 (0.994 to 1.012)	0.509	1.006 (1.000 to 1.011)	0.065
N-acetylthreonine	Hypertension	15	1.001 (0.945 to 1.060)	0.970	1.007 (0.997 to 1.016)	0.161	1.015 (0.992 to 1.038)	0.202	1.006 (1.001 to 1.012)	0.055

Exposure	Outcome	nSNP	MR Egger		Weighted median		Inverse variance weighted		MR_PRESSO	
			OR (95% CI)	pval	OR (95% CI)	pval	OR (95% CI)	pval	OR (95% CI)	pval
1-oleoyl-GPC (18:1)	Hypertension	8	0.998 (0.980 to 1.017)	0.851	1.004 (0.995 to 1.014)	0.342	1.002 (0.995 to 1.009)	0.591		
10-nonadecenoate (19:1n9)	Hypertension	8	1.023 (0.963 to 1.086)	0.492	1.002 (0.989 to 1.015)	0.767	0.993 (0.968 to 1.018)	0.562	0.998 (0.985 to 1.011)	0.758
Piperine	Hypertension	14	0.991 (0.969 to 1.014)	0.461	1.006 (0.997 to 1.015)	0.179	1.001 (0.992 to 1.011)	0.785	1.001 (0.994 to 1.008)	0.850
Carnitine C14	Hypertension	11	1.006 (0.973 to 1.040)	0.721	1.007 (0.997 to 1.016)	0.173	1.012 (1.003 to 1.022)	0.009	1.006 (0.999 to 1.013)	0.104
10-heptadecenoate (17:1n7)	Hypertension	10	1.004 (0.956 to 1.054)	0.875	1.000 (0.989 to 1.011)	0.991	0.989 (0.972 to 1.006)	0.203	0.998 (0.989 to 1.006)	0.607
1-stearoyl-gpc (18:0)	Hypertension	17	0.995 (0.980 to 1.010)	0.521	1.004 (0.996 to 1.011)	0.319	1.002 (0.996 to 1.008)	0.575		

Exposure	Outcome	nSNP	MR Egger		Weighted median		Inverse variance weighted		MR_PRESSO	
			OR (95% CI)	pval	OR (95% CI)	pval	OR (95% CI)	pval	OR (95% CI)	pval
1-palmitoyl-GPC (16:0)	Hypertension	15	1.010 (0.991 to 1.028)	0.332	0.999 (0.992 to 1.006)	0.779	1.001 (0.995 to 1.007)	0.723		
Hyocholate	Hypertension	11	1.009 (0.999 to 1.020)	0.112	1.003 (0.995 to 1.011)	0.465	0.997 (0.989 to 1.005)	0.430	0.999 (0.993 to 1.006)	0.834
N-acetylhistidine	Hypertension	13	1.002 (0.995 to 1.009)	0.525	1.002 (0.999 to 1.006)	0.177	1.002 (0.998 to 1.006)	0.396	1.002 (0.999 to 1.005)	0.293
Alpha-hydroxyisovalerate	Hypertension	14	1.003 (0.988 to 1.018)	0.719	1.004 (0.999 to 1.010)	0.143	1.006 (1.000 to 1.013)	0.059	1.007 (1.002 to 1.013)	0.028
N-acetylproline	Hypertension	9	0.993 (0.973 to 1.013)	0.514	1.008 (0.998 to 1.018)	0.128	1.004 (0.996 to 1.013)	0.336		
Stachydrine	Hypertension	10	1.001 (0.986 to 1.016)	0.909	1.003 (0.993 to 1.012)	0.564	1.004 (0.997 to 1.011)	0.284		

Exposure	Outcome	nSNP	MR Egger		Weighted median		Inverse variance weighted		MR_PRESSO	
			OR (95% CI)	pval	OR (95% CI)	pval	OR (95% CI)	pval	OR (95% CI)	pval
Salicyluric glucuronide	Hypertension	5	0.996 (0.971 to 1.021)	0.764	0.996 (0.985 to 1.007)	0.502	0.998 (0.987 to 1.009)	0.708		
P-cresol sulfate	Hypertension	13	1.010 (0.986 to 1.035)	0.440	1.003 (0.993 to 1.013)	0.544	1.004 (0.994 to 1.013)	0.445	1.001 (0.993 to 1.009)	0.867
Gamma-glutamylglycine	Hypertension	18	0.986 (0.981 to 0.992)	1.61e-04	0.989 (0.985 to 0.992)	2.55e-10	0.991 (0.987 to 0.995)	1.55e-05		
1-linoleoyl-gpc (18:2)	Hypertension	11	0.990 (0.971 to 1.009)	0.332	1.001 (0.993 to 1.008)	0.858	0.998 (0.990 to 1.006)	0.649	0.998 (0.993 to 1.003)	0.491
Stearoylcarnitine	Hypertension	15	0.999 (0.971 to 1.029)	0.973	1.006 (0.998 to 1.013)	0.137	1.011 (1.002 to 1.020)	0.018	1.008 (1.001 to 1.016)	0.049
Eicosenoate (20:1)	Hypertension	13	1.025 (0.990 to 1.061)	0.190	0.998 (0.989 to 1.007)	0.682	0.991 (0.977 to 1.006)	0.236	0.995 (0.987 to 1.003)	0.217

Exposure	Outcome	nSNP	MR Egger		Weighted median		Inverse variance weighted		MR_PRESSO	
			OR (95% CI)	pval	OR (95% CI)	pval	OR (95% CI)	pval	OR (95% CI)	pval
Aconitate [cis or trans]	Hypertension	14	1.003 (0.988 to 1.018)	0.731	1.003 (0.995 to 1.011)	0.492	1.002 (0.996 to 1.008)	0.525		
Erythronate	Hypertension	11	0.989 (0.921 to 1.063)	0.772	0.990 (0.981 to 0.999)	0.033	1.002 (0.976 to 1.028)	0.910	0.998 (0.987 to 1.009)	0.691
Isovalerylcarnitine (C5)	Hypertension	10	1.007 (0.969 to 1.046)	0.739	1.012 (1.005 to 1.020)	0.002	1.008 (0.997 to 1.019)	0.160	1.006 (0.997 to 1.014)	0.227
Linolenate [alpha or gamma; (18:3n3 or 6)]	Hypertension	10	0.985 (0.959 to 1.012)	0.309	0.999 (0.988 to 1.010)	0.834	0.999 (0.987 to 1.010)	0.809	0.995 (0.986 to 1.004)	0.278
Gamma-glutamylthreonine	Hypertension	19	0.992 (0.972 to 1.013)	0.480	0.998 (0.991 to 1.006)	0.649	1.000 (0.991 to 1.009)	0.939	0.999 (0.993 to 1.005)	0.724
Laurylcarnitine	Hypertension	13	1.004 (0.986 to 1.022)	0.707	1.003 (0.995 to 1.011)	0.492	1.000 (0.993 to 1.007)	0.962		

Exposure	Outcome	nSNP	MR Egger		Weighted median		Inverse variance weighted		MR_PRESSO	
			OR (95% CI)	pval	OR (95% CI)	pval	OR (95% CI)	pval	OR (95% CI)	pval
Gamma-glutamylmethionine	Hypertension	13	1.008 (0.993 to 1.022)	0.334	0.999 (0.991 to 1.007)	0.756	0.998 (0.990 to 1.005)	0.492		
Epiandrosterone sulfate	Hypertension	17	1.005 (0.998 to 1.011)	0.186	1.005 (1.002 to 1.008)	0.004	1.004 (0.999 to 1.009)	0.136	1.003 (0.999 to 1.008)	0.170
Gamma-glutamyltryptophan	Hypertension	16	1.011 (0.983 to 1.040)	0.460	1.001 (0.993 to 1.010)	0.724	1.008 (0.999 to 1.017)	0.094	1.005 (0.997 to 1.014)	0.242
7-methylxanthine	Hypertension	10	1.016 (0.988 to 1.046)	0.301	0.994 (0.985 to 1.004)	0.231	0.991 (0.978 to 1.004)	0.184	0.996 (0.989 to 1.002)	0.246
3,7-dimethylurate	Hypertension	16	1.004 (0.990 to 1.018)	0.579	1.003 (0.995 to 1.010)	0.498	1.000 (0.992 to 1.008)	0.967	1.002 (0.994 to 1.009)	0.667
1,7-dimethyluric acid	Hypertension	17	1.001 (0.991 to 1.010)	0.902	1.000 (0.993 to 1.008)	0.995	1.001 (0.995 to 1.007)	0.804		

Exposure	Outcome	nSNP	MR Egger		Weighted median		Inverse variance weighted		MR_PRESSO	
			OR (95% CI)	pval	OR (95% CI)	pval	OR (95% CI)	pval	OR (95% CI)	pval
1,3,7-trimethylurate	Hypertension	14	1.010 (0.994 to 1.025)	0.249	0.999 (0.992 to 1.006)	0.786	1.001 (0.994 to 1.007)	0.827		
N4-acetylcytidine	Hypertension	25	0.995 (0.987 to 1.002)	0.183	0.996 (0.991 to 1.001)	0.100	0.999 (0.995 to 1.003)	0.730		
Indolebutyrate	Hypertension	15	0.995 (0.981 to 1.009)	0.475	1.000 (0.995 to 1.006)	0.900	0.999 (0.994 to 1.004)	0.798		
5-acetylamino-6-amino-3-methyluracil	Hypertension	19	1.005 (0.992 to 1.017)	0.473	1.005 (1.000 to 1.010)	0.040	1.000 (0.993 to 1.007)	0.935	0.997 (0.989 to 1.005)	0.503
N1-methylinosine	Hypertension	17	1.007 (0.985 to 1.030)	0.549	1.003 (0.994 to 1.011)	0.521	1.002 (0.994 to 1.009)	0.652	1.004 (0.998 to 1.010)	0.198
N6-carbamoylthreonyladenosine	Hypertension	14	0.985 (0.924 to 1.050)	0.657	0.989 (0.979 to 0.999)	0.027	0.999 (0.975 to 1.023)	0.919	0.992 (0.983 to 1.000)	0.076

Exposure	Outcome	nSNP	MR Egger		Weighted median		Inverse variance weighted		MR_PRESSO	
			OR (95% CI)	pval	OR (95% CI)	pval	OR (95% CI)	pval	OR (95% CI)	pval
1-methylxanthine	Hypertension	16	0.990 (0.977 to 1.004)	0.198	0.993 (0.988 to 0.998)	0.006	0.994 (0.987 to 1.001)	0.109	0.994 (0.988 to 0.999)	0.036
N2,n2-dimethylguanosine	Hypertension	9	1.001 (0.965 to 1.039)	0.942	0.995 (0.982 to 1.008)	0.442	1.000 (0.987 to 1.013)	0.966		
4-hydroxyhippurate	Hypertension	11	1.000 (0.983 to 1.018)	0.980	1.000 (0.991 to 1.009)	0.979	1.002 (0.995 to 1.009)	0.591		
3-(3-amino-3-carboxypropyl)uridine	Hypertension	8	1.064 (0.938 to 1.206)	0.375	0.992 (0.979 to 1.005)	0.224	1.020 (0.983 to 1.058)	0.294	1.004 (0.990 to 1.017)	0.632
5,6-dihydrouridine	Hypertension	17	1.000 (0.979 to 1.023)	0.966	0.996 (0.988 to 1.003)	0.261	0.998 (0.987 to 1.009)	0.758	1.000 (0.994 to 1.006)	0.949
Phenylacetylglutamine	Hypertension	12	1.006 (0.989 to 1.023)	0.537	1.004 (0.995 to 1.014)	0.390	1.002 (0.994 to 1.010)	0.599		

Exposure	Outcome	nSNP	MR Egger		Weighted median		Inverse variance weighted		MR_PRESSO	
			OR (95% CI)	pval	OR (95% CI)	pval	OR (95% CI)	pval	OR (95% CI)	pval
3-hydroxydodecanedioate	Hypertension	10	1.007 (0.991 to 1.023)	0.446	0.999 (0.990 to 1.008)	0.826	1.003 (0.996 to 1.009)	0.386		
1-arachidonylglycerol (20:4)	Hypertension	12	0.999 (0.988 to 1.011)	0.924	0.998 (0.992 to 1.004)	0.553	0.997 (0.992 to 1.003)	0.325		
7-methylguanine	Hypertension	12	0.985 (0.973 to 0.997)	0.040	0.995 (0.987 to 1.004)	0.269	0.994 (0.987 to 1.000)	0.039		
1-stearoyl-GPE (18:0)	Hypertension	26	0.995 (0.980 to 1.011)	0.562	1.001 (0.995 to 1.007)	0.739	1.004 (0.998 to 1.011)	0.186	1.002 (0.996 to 1.007)	0.515
Phenol sulfate	Hypertension	13	1.011 (0.994 to 1.028)	0.243	0.998 (0.989 to 1.006)	0.613	1.000 (0.994 to 1.006)	0.949		
Oleoylcarnitine	Hypertension	9	1.002 (0.986 to 1.017)	0.840	1.000 (0.992 to 1.008)	0.933	1.000 (0.994 to 1.006)	0.975		

Exposure	Outcome	nSNP	MR Egger		Weighted median		Inverse variance weighted		MR_PRESSO	
			OR (95% CI)	pval	OR (95% CI)	pval	OR (95% CI)	pval	OR (95% CI)	pval
Gamma-glutamylisoleucine	Hypertension	11	0.999 (0.983 to 1.015)	0.870	1.003 (0.994 to 1.012)	0.533	1.001 (0.994 to 1.008)	0.813		
Malonylcarnitine	Hypertension	12	0.997 (0.974 to 1.021)	0.799	1.008 (0.999 to 1.017)	0.083	1.009 (0.999 to 1.018)	0.064	1.006 (0.997 to 1.015)	0.202
1-palmitoleoyl-GPC (16:1)	Hypertension	18	0.985 (0.952 to 1.020)	0.411	0.998 (0.989 to 1.008)	0.736	0.997 (0.986 to 1.008)	0.600	1.000 (0.991 to 1.009)	0.943
N-methyl-2-pyridone-5-carboxamide	Hypertension	9	1.000 (0.973 to 1.028)	0.983	1.006 (0.995 to 1.017)	0.284	1.002 (0.993 to 1.012)	0.664		
Gamma-glutamyl-2-aminobutyrate	Hypertension	17	0.995 (0.984 to 1.007)	0.456	0.999 (0.992 to 1.006)	0.811	0.996 (0.991 to 1.001)	0.130		
1-stearoyl-GPG (18:0)	Hypertension	13	0.995 (0.979 to 1.011)	0.537	0.997 (0.990 to 1.003)	0.316	1.001 (0.994 to 1.007)	0.863	0.998 (0.993 to 1.003)	0.496

Exposure	Outcome	nSNP	MR Egger		Weighted median		Inverse variance weighted		MR_PRESSO	
			OR (95% CI)	pval	OR (95% CI)	pval	OR (95% CI)	pval	OR (95% CI)	pval
Hexanoylglycine	Hypertension	16	0.989 (0.974 to 1.003)	0.146	1.002 (0.995 to 1.009)	0.542	0.997 (0.991 to 1.003)	0.362	1.001 (0.996 to 1.006)	0.649
Glutamine degradant	Hypertension	18	1.003 (0.988 to 1.018)	0.741	0.999 (0.991 to 1.006)	0.746	1.005 (0.998 to 1.013)	0.159	1.000 (0.995 to 1.006)	0.918
5-methyluridine (ribothymidine)	Hypertension	20	0.997 (0.991 to 1.003)	0.298	0.998 (0.995 to 1.002)	0.264	0.997 (0.993 to 1.001)	0.176		
Cysteine-glutathione disulfide	Hypertension	14	0.998 (0.985 to 1.011)	0.784	0.999 (0.993 to 1.004)	0.623	0.997 (0.990 to 1.003)	0.307	0.998 (0.993 to 1.004)	0.499
Isovalerylglycine	Hypertension	13	0.974 (0.956 to 0.994)	0.025	0.997 (0.988 to 1.005)	0.464	0.990 (0.982 to 0.998)	0.020	0.998 (0.992 to 1.005)	0.600
2-hydroxy-3-methylvalerate	Hypertension	12	1.011 (0.985 to 1.037)	0.441	1.004 (0.996 to 1.012)	0.290	1.005 (0.997 to 1.013)	0.200		

Exposure	Outcome	nSNP	MR Egger		Weighted median		Inverse variance weighted		MR_PRESSO	
			OR (95% CI)	pval	OR (95% CI)	pval	OR (95% CI)	pval	OR (95% CI)	pval
Homostachydrine	Hypertension	12	1.012 (0.990 to 1.034)	0.315	0.998 (0.988 to 1.008)	0.694	1.004 (0.994 to 1.014)	0.431	0.994 (0.987 to 1.002)	0.175
1-arachidonoyl-gpc (20:4n6)	Hypertension	16	0.997 (0.988 to 1.005)	0.460	0.999 (0.996 to 1.002)	0.463	0.999 (0.993 to 1.005)	0.710	0.999 (0.996 to 1.001)	0.345
2-palmitoyl-GPC (16:0)	Hypertension	10	1.018 (0.991 to 1.047)	0.224	1.003 (0.994 to 1.012)	0.490	1.006 (0.996 to 1.015)	0.241		
2-palmitoleoyl-GPC (16:1)	Hypertension	19	0.998 (0.974 to 1.022)	0.844	0.999 (0.992 to 1.007)	0.858	0.997 (0.989 to 1.004)	0.419	1.001 (0.995 to 1.006)	0.794
1-arachidonoyl-GPE (20:4n6)	Hypertension	19	0.995 (0.985 to 1.005)	0.342	0.999 (0.995 to 1.003)	0.505	1.000 (0.994 to 1.005)	0.957	0.999 (0.994 to 1.003)	0.560
1-palmitoyl-GPE (16:0)	Hypertension	12	1.012 (0.984 to 1.041)	0.410	1.000 (0.993 to 1.006)	0.930	1.004 (0.995 to 1.012)	0.416	0.999 (0.994 to 1.004)	0.749

Exposure	Outcome	nSNP	MR Egger		Weighted median		Inverse variance weighted		MR_PRESSO	
			OR (95% CI)	pval	OR (95% CI)	pval	OR (95% CI)	pval	OR (95% CI)	pval
1-linoleoyl-GPE (18:2)	Hypertension	17	0.995 (0.985 to 1.005)	0.357	1.002 (0.996 to 1.007)	0.521	1.000 (0.995 to 1.005)	0.975		
1-oleoyl-GPE (18:1)	Hypertension	21	1.006 (0.991 to 1.022)	0.453	1.000 (0.993 to 1.006)	0.964	0.998 (0.993 to 1.003)	0.402		
2-hydroxypalmitate	Hypertension	14	1.000 (0.985 to 1.015)	0.972	1.000 (0.991 to 1.008)	0.982	1.002 (0.996 to 1.009)	0.505		
Docosapentaenoate (n6 DPA; 22:5n6)	Hypertension	10	1.000 (0.981 to 1.020)	0.975	0.997 (0.988 to 1.006)	0.518	0.999 (0.991 to 1.006)	0.701		
Gulonate	Hypertension	13	0.992 (0.964 to 1.021)	0.587	0.996 (0.987 to 1.006)	0.411	0.995 (0.985 to 1.006)	0.353	0.994 (0.988 to 1.000)	0.060
Isobutyrylglycine	Hypertension	18	0.988 (0.972 to 1.004)	0.168	0.999 (0.992 to 1.007)	0.889	0.996 (0.989 to 1.003)	0.292	1.002 (0.998 to 1.007)	0.322

Exposure	Outcome	nSNP	MR Egger		Weighted median		Inverse variance weighted		MR_PRESSO	
			OR (95% CI)	pval	OR (95% CI)	pval	OR (95% CI)	pval	OR (95% CI)	pval
Glutarylcarnitine (c5-dc)	Hypertension	25	1.003 (0.992 to 1.015)	0.567	1.003 (0.999 to 1.008)	0.102	1.005 (1.000 to 1.010)	0.049	1.004 (1.000 to 1.008)	0.080
Carnitine C5:1	Hypertension	13	0.992 (0.959 to 1.026)	0.654	0.995 (0.986 to 1.005)	0.339	0.998 (0.986 to 1.011)	0.803	0.995 (0.988 to 1.002)	0.171
Hydroquinone sulfate	Hypertension	10	0.983 (0.959 to 1.008)	0.218	0.999 (0.989 to 1.010)	0.900	1.000 (0.989 to 1.011)	0.995	0.999 (0.992 to 1.006)	0.809
Beta-hydroxyisovaleroylcarnitine	Hypertension	14	1.001 (0.983 to 1.019)	0.946	1.008 (1.001 to 1.014)	0.019	1.012 (1.003 to 1.020)	0.005	1.011 (1.005 to 1.018)	0.006
Catechol sulfate	Hypertension	19	1.002 (0.986 to 1.019)	0.792	1.005 (0.998 to 1.013)	0.154	1.001 (0.994 to 1.008)	0.784	1.004 (1.000 to 1.009)	0.090
7-alpha-hydroxy-3-oxo-4-cholestenoate (7-hoca)	Hypertension	15	1.006 (0.985 to 1.027)	0.615	1.003 (0.995 to 1.012)	0.480	0.999 (0.991 to 1.008)	0.826	1.002 (0.994 to 1.009)	0.681

Exposure	Outcome	nSNP	MR Egger		Weighted median		Inverse variance weighted		MR_PRESSO	
			OR (95% CI)	pval	OR (95% CI)	pval	OR (95% CI)	pval	OR (95% CI)	pval
Octadecanedioate	Hypertension	19	0.992 (0.976 to 1.009)	0.365	0.996 (0.989 to 1.004)	0.360	0.996 (0.990 to 1.003)	0.262	1.001 (0.996 to 1.006)	0.632
Tetradecanedioate (C14-DC)	Hypertension	9	0.999 (0.990 to 1.008)	0.826	0.998 (0.994 to 1.003)	0.461	0.998 (0.993 to 1.004)	0.559		
Hexadecanedioate (C16-DC)	Hypertension	11	1.000 (0.992 to 1.008)	0.934	0.998 (0.994 to 1.002)	0.411	0.997 (0.992 to 1.002)	0.270		
Glycerophosphoethanolamine	Hypertension	14	1.005 (0.992 to 1.018)	0.468	1.006 (0.999 to 1.014)	0.092	1.002 (0.996 to 1.008)	0.478		
Taurolithocholate 3-sulfate	Hypertension	14	0.991 (0.979 to 1.003)	0.186	0.993 (0.985 to 1.000)	0.053	0.997 (0.990 to 1.003)	0.312		
1-palmitoyl-GPI (16:0)	Hypertension	13	0.998 (0.982 to 1.014)	0.781	1.002 (0.993 to 1.010)	0.705	1.003 (0.996 to 1.010)	0.452		

Exposure	Outcome	nSNP	MR Egger		Weighted median		Inverse variance weighted		MR_PRESSO	
			OR (95% CI)	pval	OR (95% CI)	pval	OR (95% CI)	pval	OR (95% CI)	pval
Glycolithocholate sulfate	Hypertension	7	0.984 (0.963 to 1.004)	0.180	0.997 (0.989 to 1.004)	0.410	0.999 (0.988 to 1.009)	0.801	0.998 (0.992 to 1.004)	0.599
3-(3-hydroxyphenyl)propionate	Hypertension	14	1.007 (0.991 to 1.023)	0.428	1.004 (0.996 to 1.011)	0.324	1.007 (1.000 to 1.014)	0.036		
Deoxycarnitine	Hypertension	19	0.994 (0.985 to 1.004)	0.252	0.998 (0.993 to 1.003)	0.379	0.999 (0.994 to 1.005)	0.741	0.998 (0.993 to 1.002)	0.371
Alpha-hydroxycaproate	Hypertension	6	1.007 (0.979 to 1.035)	0.669	0.991 (0.983 to 1.001)	0.065	0.994 (0.986 to 1.002)	0.159		
1-ribosyl-imidazoleacetate	Hypertension	20	0.973 (0.950 to 0.997)	0.038	1.002 (0.995 to 1.009)	0.665	1.005 (0.995 to 1.015)	0.331	1.001 (0.995 to 1.008)	0.705
3,4-dihydroxybutyrate	Hypertension	11	1.004 (0.979 to 1.029)	0.766	1.005 (0.997 to 1.013)	0.205	1.001 (0.993 to 1.009)	0.776		

Exposure	Outcome	nSNP	MR Egger		Weighted median		Inverse variance weighted		MR_PRESSO	
			OR (95% CI)	pval	OR (95% CI)	pval	OR (95% CI)	pval	OR (95% CI)	pval
Indoleacetylglutamine	Hypertension	17	1.011 (1.000 to 1.022)	0.079	1.001 (0.996 to 1.007)	0.647	1.000 (0.996 to 1.005)	0.916		
Mannitol/sorbitol	Hypertension	13	0.998 (0.984 to 1.013)	0.835	1.003 (0.995 to 1.011)	0.492	1.005 (0.999 to 1.011)	0.131		
Tryptophan betaine	Hypertension	20	0.991 (0.968 to 1.015)	0.485	1.001 (0.994 to 1.007)	0.798	1.007 (0.994 to 1.020)	0.288	1.001 (0.997 to 1.005)	0.661
Dihomo-linolenate (20:3n3 or n6)	Hypertension	14	1.004 (0.990 to 1.018)	0.580	1.002 (0.993 to 1.011)	0.676	0.998 (0.992 to 1.004)	0.543		
1-linoleoyl-GPI (18:2)	Hypertension	18	1.013 (1.000 to 1.025)	0.061	1.000 (0.994 to 1.006)	0.959	0.998 (0.992 to 1.003)	0.400		
1-oleoyl-GPI (18:1)	Hypertension	19	1.012 (0.998 to 1.026)	0.107	1.002 (0.995 to 1.009)	0.527	1.003 (0.998 to 1.008)	0.220		

Exposure	Outcome	nSNP	MR Egger		Weighted median		Inverse variance weighted		MR_PRESSO	
			OR (95% CI)	pval	OR (95% CI)	pval	OR (95% CI)	pval	OR (95% CI)	pval
O-cresol sulfate	Hypertension	7	0.989 (0.941 to 1.040)	0.682	0.990 (0.978 to 1.002)	0.091	0.993 (0.981 to 1.005)	0.231		
4-vinylphenol sulfate	Hypertension	12	0.989 (0.970 to 1.008)	0.288	1.000 (0.991 to 1.009)	0.990	0.999 (0.992 to 1.007)	0.855		
Pyrraline	Hypertension	7	0.970 (0.915 to 1.029)	0.358	0.996 (0.984 to 1.009)	0.553	1.001 (0.980 to 1.022)	0.927	0.997 (0.986 to 1.009)	0.665
N6-acetyllysine	Hypertension	11	1.008 (0.996 to 1.020)	0.234	1.006 (1.001 to 1.011)	0.021	1.002 (0.994 to 1.011)	0.568	1.003 (0.995 to 1.010)	0.495
Thymol sulfate	Hypertension	10	0.993 (0.965 to 1.022)	0.666	1.002 (0.992 to 1.012)	0.670	1.004 (0.993 to 1.016)	0.451	0.998 (0.990 to 1.006)	0.622
N-acetylserine	Hypertension	11	0.997 (0.937 to 1.061)	0.926	0.996 (0.986 to 1.007)	0.502	1.005 (0.976 to 1.034)	0.733	0.998 (0.989 to 1.006)	0.610

Exposure	Outcome	nSNP	MR Egger		Weighted median		Inverse variance weighted		MR_PRESSO	
			OR (95% CI)	pval	OR (95% CI)	pval	OR (95% CI)	pval	OR (95% CI)	pval
3-methyladipate	Hypertension	9	1.003 (0.976 to 1.030)	0.839	1.000 (0.990 to 1.010)	0.977	0.992 (0.981 to 1.002)	0.121	0.995 (0.987 to 1.004)	0.340
4-ethylphenylsulfate	Hypertension	8	0.999 (0.957 to 1.043)	0.960	1.003 (0.991 to 1.015)	0.638	0.996 (0.981 to 1.011)	0.578	0.997 (0.983 to 1.010)	0.654
1-stearoyl-2-oleoyl-GPE (18:0/18:1)	Hypertension	18	1.002 (0.994 to 1.011)	0.613	1.001 (0.996 to 1.006)	0.619	1.003 (0.999 to 1.006)	0.177		
Dimethylarginine (sdma + adma)	Hypertension	9	0.999 (0.983 to 1.015)	0.881	1.005 (0.996 to 1.013)	0.277	1.002 (0.996 to 1.009)	0.520		
Hexanoylglutamine	Hypertension	13	1.011 (0.994 to 1.029)	0.235	1.007 (1.003 to 1.012)	0.003	1.005 (0.996 to 1.014)	0.310	1.001 (0.993 to 1.009)	0.846
Gamma-glutamylalanine	Hypertension	10	1.009 (0.946 to 1.076)	0.788	1.005 (0.995 to 1.015)	0.358	0.996 (0.978 to 1.014)	0.642	1.003 (0.995 to 1.012)	0.462

Exposure	Outcome	nSNP	MR Egger		Weighted median		Inverse variance weighted		MR_PRESSO	
			OR (95% CI)	pval	OR (95% CI)	pval	OR (95% CI)	pval	OR (95% CI)	pval
5alpha-androstan-3beta,17beta-diol disulfate	Hypertension	16	1.006 (0.995 to 1.017)	0.337	1.007 (1.001 to 1.013)	0.015	1.004 (0.997 to 1.011)	0.294	1.002 (0.996 to 1.008)	0.457
N-methylproline	Hypertension	10	1.003 (0.987 to 1.018)	0.729	1.008 (0.999 to 1.017)	0.092	1.005 (0.998 to 1.012)	0.155		
Carotenoid	Hypertension	14	1.014 (0.997 to 1.032)	0.129	1.006 (0.998 to 1.015)	0.161	1.005 (0.998 to 1.012)	0.171		
5alpha-pregnan-3beta,20alpha-diol disulfate	Hypertension	22	0.999 (0.990 to 1.009)	0.849	0.996 (0.991 to 1.002)	0.210	0.997 (0.992 to 1.001)	0.175		
Taurocholenate sulfate	Hypertension	27	0.997 (0.986 to 1.008)	0.569	0.997 (0.992 to 1.002)	0.229	1.001 (0.995 to 1.007)	0.801	1.000 (0.995 to 1.004)	0.907
Pregnenediol disulfate (C21H34O8S2)	Hypertension	22	1.001 (0.993 to 1.009)	0.800	1.001 (0.997 to 1.005)	0.663	0.997 (0.992 to 1.002)	0.235	0.994 (0.988 to 1.000)	0.052

Exposure	Outcome	nSNP	MR Egger		Weighted median		Inverse variance weighted		MR_PRESSO	
			OR (95% CI)	pval	OR (95% CI)	pval	OR (95% CI)	pval	OR (95% CI)	pval
Glycocholenate sulfate	Hypertension	24	0.999 (0.993 to 1.006)	0.867	0.999 (0.995 to 1.002)	0.408	0.997 (0.993 to 1.001)	0.120	0.998 (0.995 to 1.000)	0.115
Androstenediol (3beta,17beta) disulfate (1)	Hypertension	14	1.004 (0.996 to 1.013)	0.315	1.002 (0.997 to 1.008)	0.397	1.004 (0.999 to 1.009)	0.127		
Chiro-inositol	Hypertension	7	1.000 (0.983 to 1.016)	0.960	1.001 (0.993 to 1.009)	0.875	0.999 (0.993 to 1.005)	0.744		
1-palmitoyl-2-linoleoyl-GPE (16:0/18:2)	Hypertension	20	1.000 (0.992 to 1.009)	0.911	1.001 (0.998 to 1.005)	0.461	1.002 (0.998 to 1.007)	0.349	1.001 (0.998 to 1.004)	0.581
Sphinganine-1-phosphate	Hypertension	8	0.980 (0.928 to 1.035)	0.499	0.998 (0.988 to 1.008)	0.714	1.006 (0.989 to 1.022)	0.499	0.998 (0.991 to 1.005)	0.546
Glycosyl-N-stearoyl-sphingosine (d18:1/18:0)	Hypertension	19	0.995 (0.979 to 1.010)	0.490	0.998 (0.992 to 1.003)	0.414	1.002 (0.996 to 1.008)	0.591	1.000 (0.995 to 1.005)	0.903

Exposure	Outcome	nSNP	MR Egger		Weighted median		Inverse variance weighted		MR_PRESSO	
			OR (95% CI)	pval	OR (95% CI)	pval	OR (95% CI)	pval	OR (95% CI)	pval
4-allylphenol sulfate	Hypertension	12	0.996 (0.974 to 1.018)	0.723	0.998 (0.989 to 1.008)	0.734	1.003 (0.994 to 1.012)	0.482		
Succinylcarnitine	Hypertension	21	1.009 (1.002 to 1.016)	0.026	1.006 (1.001 to 1.011)	0.013	1.005 (1.001 to 1.008)	0.013		
21-hydroxypregnenolone disulfate	Hypertension	25	0.978 (0.954 to 1.003)	0.099	0.997 (0.990 to 1.003)	0.305	0.998 (0.988 to 1.008)	0.676	0.997 (0.993 to 1.001)	0.179
5alpha-androstan-3alpha,17alpha-diol monosulfate	Hypertension	14	1.004 (0.994 to 1.013)	0.477	1.005 (1.000 to 1.011)	0.070	1.001 (0.996 to 1.006)	0.720	1.002 (0.996 to 1.008)	0.531
Androstenediol (3beta,17beta) disulfate (2)	Hypertension	28	0.994 (0.974 to 1.014)	0.553	0.994 (0.987 to 1.001)	0.102	0.992 (0.984 to 1.000)	0.045	0.994 (0.988 to 1.000)	0.068
5alpha-androstan-3beta,17alpha-diol disulfate	Hypertension	13	1.016 (0.999 to 1.034)	0.087	1.007 (1.000 to 1.014)	0.056	1.008 (1.001 to 1.015)	0.019		

Exposure	Outcome	nSNP	MR Egger		Weighted median		Inverse variance weighted		MR_PRESSO	
			OR (95% CI)	pval	OR (95% CI)	pval	OR (95% CI)	pval	OR (95% CI)	pval
5alpha-pregnan-3beta,20beta-diol monosulfate (1)	Hypertension	16	0.999 (0.992 to 1.007)	0.823	0.995 (0.990 to 0.999)	0.013	0.996 (0.992 to 1.001)	0.125	0.995 (0.991 to 0.999)	0.026
5alpha-pregnan-3beta,20alpha-diol monosulfate (2)	Hypertension	20	0.999 (0.982 to 1.017)	0.915	0.994 (0.988 to 1.001)	0.095	0.998 (0.991 to 1.006)	0.652	0.997 (0.992 to 1.003)	0.386
5alpha-androstan-3beta,17beta-diol monosulfate (2)	Hypertension	20	1.006 (1.001 to 1.010)	0.015	1.005 (1.001 to 1.009)	0.008	1.004 (1.001 to 1.007)	0.011		
Androstenediol (3alpha, 17alpha) monosulfate (3)	Hypertension	18	1.003 (0.993 to 1.013)	0.585	1.003 (0.999 to 1.008)	0.147	1.002 (0.996 to 1.009)	0.531	1.004 (0.999 to 1.008)	0.115
5alpha-pregnan-diol disulfate	Hypertension	12	0.997 (0.982 to 1.013)	0.727	0.997 (0.992 to 1.003)	0.368	0.999 (0.994 to 1.004)	0.649		
5alpha-androstan-3alpha,17beta-diol monosulfate (1)	Hypertension	16	1.003 (0.999 to 1.008)	0.146	1.004 (1.001 to 1.007)	0.004	1.003 (1.000 to 1.006)	0.030		

Exposure	Outcome	nSNP	MR Egger		Weighted median		Inverse variance weighted		MR_PRESSO	
			OR (95% CI)	pval	OR (95% CI)	pval	OR (95% CI)	pval	OR (95% CI)	pval
5alpha-androstan-3alpha,17beta-diol disulfate	Hypertension	20	0.996 (0.986 to 1.006)	0.442	0.993 (0.987 to 0.999)	0.032	0.992 (0.988 to 0.996)	1.61e-04		
Andro steroid monosulfate C19H28O6S (1)	Hypertension	22	0.993 (0.985 to 1.001)	0.094	0.995 (0.990 to 1.000)	0.071	0.999 (0.993 to 1.004)	0.613	1.004 (0.998 to 1.010)	0.227
Pregnenediol sulfate (C21H34O5S)	Hypertension	24	0.976 (0.947 to 1.006)	0.131	0.994 (0.987 to 1.000)	0.064	1.000 (0.987 to 1.013)	0.972	0.999 (0.993 to 1.005)	0.852
4-hydroxycoumarin	Hypertension	11	0.995 (0.978 to 1.012)	0.566	1.003 (0.993 to 1.012)	0.566	1.002 (0.994 to 1.010)	0.686		
16a-hydroxy DHEA 3-sulfate	Hypertension	18	0.996 (0.987 to 1.004)	0.336	0.995 (0.991 to 0.999)	0.012	0.996 (0.990 to 1.003)	0.258	0.996 (0.993 to 1.000)	0.044
Gamma-CEHC	Hypertension	23	0.992 (0.983 to 1.001)	0.100	0.990 (0.985 to 0.996)	6.8e-04	0.997 (0.992 to 1.002)	0.216	1.000 (0.995 to 1.005)	0.947

Exposure	Outcome	nSNP	MR Egger		Weighted median		Inverse variance weighted		MR_PRESSO	
			OR (95% CI)	pval	OR (95% CI)	pval	OR (95% CI)	pval	OR (95% CI)	pval
2-hydroxyglutarate	Hypertension	14	1.002 (0.983 to 1.021)	0.836	1.007 (0.999 to 1.015)	0.067	1.009 (1.003 to 1.015)	0.003		
N-acetyl-beta-alanine	Hypertension	14	0.985 (0.977 to 0.994)	0.006	0.995 (0.988 to 1.003)	0.240	0.998 (0.992 to 1.004)	0.511		
Tridecenedioate (C13:1-DC)	Hypertension	13	0.992 (0.975 to 1.009)	0.386	1.001 (0.993 to 1.009)	0.759	1.001 (0.994 to 1.008)	0.790		
N-acetyl-3-methylhistidine	Hypertension	18	1.008 (1.001 to 1.014)	0.029	1.006 (1.001 to 1.010)	0.010	1.002 (0.999 to 1.006)	0.206		
2-hydroxyhippurate	Hypertension	11	0.987 (0.973 to 1.002)	0.122	0.996 (0.987 to 1.005)	0.405	1.002 (0.993 to 1.010)	0.713		
4-cholesten-3-one	Hypertension	16	0.991 (0.971 to 1.012)	0.413	1.001 (0.993 to 1.008)	0.847	1.004 (0.997 to 1.010)	0.247	1.006 (1.001 to 1.012)	0.045

Exposure	Outcome	nSNP	MR Egger		Weighted median		Inverse variance weighted		MR_PRESSO	
			OR (95% CI)	pval	OR (95% CI)	pval	OR (95% CI)	pval	OR (95% CI)	pval
Androstenediol (3beta,17beta) monosulfate (2)	Hypertension	20	1.013 (0.995 to 1.031)	0.171	1.004 (0.997 to 1.012)	0.231	1.004 (0.997 to 1.011)	0.267	1.006 (1.001 to 1.012)	0.041
Androstenediol (3beta,17beta) monosulfate (1)	Hypertension	19	1.000 (0.977 to 1.023)	0.977	1.010 (1.001 to 1.019)	0.032	1.006 (0.994 to 1.017)	0.362	1.003 (0.995 to 1.011)	0.471
Pregnenolone sulfate	Hypertension	20	0.978 (0.951 to 1.006)	0.137	0.997 (0.989 to 1.004)	0.421	1.002 (0.991 to 1.013)	0.704	1.002 (0.996 to 1.007)	0.622
S-methylmethionine	Hypertension	9	1.011 (0.994 to 1.029)	0.247	0.995 (0.986 to 1.004)	0.247	0.994 (0.987 to 1.002)	0.160		
13-HODE + 9-HODE	Hypertension	6	1.012 (0.994 to 1.031)	0.251	1.000 (0.989 to 1.012)	0.956	1.002 (0.993 to 1.011)	0.672		
Palmitoyl sphingomyelin (d18:1/16:0)	Hypertension	19	0.995 (0.977 to 1.013)	0.588	1.002 (0.994 to 1.010)	0.598	1.002 (0.995 to 1.010)	0.540	1.004 (0.998 to 1.011)	0.218

Exposure	Outcome	nSNP	MR Egger		Weighted median		Inverse variance weighted		MR_PRESSO	
			OR (95% CI)	pval	OR (95% CI)	pval	OR (95% CI)	pval	OR (95% CI)	pval
Indole-3-carboxylate	Hypertension	7	0.999 (0.981 to 1.017)	0.906	0.998 (0.989 to 1.007)	0.629	0.998 (0.990 to 1.006)	0.596		
Sphingomyelin (d18:1/18:1, d18:2/18:0)	Hypertension	19	1.035 (0.992 to 1.080)	0.126	0.997 (0.989 to 1.004)	0.411	0.992 (0.975 to 1.010)	0.402	0.995 (0.989 to 1.001)	0.124
Ergothioneine	Hypertension	11	0.998 (0.974 to 1.023)	0.879	0.997 (0.989 to 1.006)	0.521	0.992 (0.984 to 1.000)	0.064	0.998 (0.992 to 1.004)	0.481
Cis-4-decenoylcarnitine (C10:1)	Hypertension	11	1.008 (1.001 to 1.015)	0.046	1.004 (1.000 to 1.008)	0.082	1.003 (0.999 to 1.007)	0.169		
5-(galactosylhydroxy)-L-lysine	Hypertension	8	0.976 (0.958 to 0.993)	0.036	1.000 (0.989 to 1.011)	0.955	0.998 (0.989 to 1.007)	0.682		
2s,3R-dihydroxybutyrate	Hypertension	12	1.001 (0.986 to 1.017)	0.857	0.989 (0.981 to 0.998)	0.012	0.997 (0.989 to 1.006)	0.529		

Exposure	Outcome	nSNP	MR Egger		Weighted median		Inverse variance weighted		MR_PRESSO	
			OR (95% CI)	pval	OR (95% CI)	pval	OR (95% CI)	pval	OR (95% CI)	pval
Cysteinylglycine disulfide	Hypertension	10	1.017 (1.002 to 1.033)	0.059	1.019 (1.012 to 1.026)	1.52e-07	1.011 (1.004 to 1.019)	0.003	1.004 (0.995 to 1.013)	0.445
2,3-dihydroxyisovalerate	Hypertension	16	1.006 (0.991 to 1.021)	0.450	0.996 (0.988 to 1.004)	0.313	0.997 (0.990 to 1.003)	0.326		
Alpha-ketoglutaramate	Hypertension	6	1.016 (0.999 to 1.033)	0.135	1.013 (1.005 to 1.021)	0.001	1.011 (1.005 to 1.018)	7.64e-04		
3-methylglutaconate	Hypertension	13	0.991 (0.980 to 1.002)	0.125	0.998 (0.990 to 1.006)	0.667	0.997 (0.991 to 1.004)	0.457	1.007 (1.000 to 1.014)	0.063
(16 or 17)-methylstearate (a19:0 or i19:0)	Hypertension	11	1.017 (0.984 to 1.050)	0.343	1.000 (0.989 to 1.010)	0.935	0.989 (0.971 to 1.006)	0.209	0.996 (0.987 to 1.004)	0.364
Cinnamoylglycine	Hypertension	18	0.990 (0.976 to 1.004)	0.165	0.993 (0.986 to 1.000)	0.063	0.990 (0.984 to 0.996)	0.002	0.997 (0.992 to 1.001)	0.159

Exposure	Outcome	nSNP	MR Egger		Weighted median		Inverse variance weighted		MR_PRESSO	
			OR (95% CI)	pval	OR (95% CI)	pval	OR (95% CI)	pval	OR (95% CI)	pval
2R,3R-dihydroxybutyrate	Hypertension	20	1.019 (1.003 to 1.035)	0.030	1.004 (0.998 to 1.010)	0.215	1.003 (0.996 to 1.010)	0.446	1.001 (0.996 to 1.005)	0.833
4-hydroxyglutamate	Hypertension	17	1.000 (0.989 to 1.012)	0.945	1.001 (0.995 to 1.008)	0.672	1.005 (1.001 to 1.010)	0.030		
S-methylcysteine	Hypertension	9	1.008 (0.995 to 1.022)	0.261	1.002 (0.996 to 1.009)	0.489	0.999 (0.992 to 1.006)	0.858		
Isoursodeoxycholate	Hypertension	8	0.977 (0.942 to 1.014)	0.269	0.992 (0.980 to 1.003)	0.163	0.993 (0.980 to 1.006)	0.298	0.988 (0.979 to 0.996)	0.032
Cis-4-decenoate (10:1n6)	Hypertension	12	1.005 (0.998 to 1.012)	0.230	1.001 (0.997 to 1.005)	0.589	1.001 (0.996 to 1.005)	0.741		
4-oxo-retinoic acid	Hypertension	11	0.994 (0.967 to 1.022)	0.686	0.998 (0.989 to 1.006)	0.591	1.003 (0.991 to 1.016)	0.584	0.999 (0.991 to 1.008)	0.865

Exposure	Outcome	nSNP	MR Egger		Weighted median		Inverse variance weighted		MR_PRESSO	
			OR (95% CI)	pval	OR (95% CI)	pval	OR (95% CI)	pval	OR (95% CI)	pval
1-lignoceroyl-GPC (24:0)	Hypertension	8	1.012 (0.994 to 1.030)	0.253	0.998 (0.988 to 1.007)	0.620	0.996 (0.989 to 1.002)	0.209		
Sulfate	Hypertension	21	0.997 (0.977 to 1.017)	0.739	1.000 (0.992 to 1.008)	0.948	1.005 (0.998 to 1.013)	0.175	1.003 (0.996 to 1.010)	0.359
4-hydroxy-2-oxoglutaric acid	Hypertension	17	0.992 (0.981 to 1.002)	0.152	0.997 (0.989 to 1.004)	0.376	0.997 (0.992 to 1.002)	0.212		
Glycoursodeoxycholate	Hypertension	11	1.001 (0.980 to 1.022)	0.940	0.994 (0.984 to 1.004)	0.221	1.000 (0.991 to 1.009)	0.995		
1-(1-enyl-palmitoyl)-GPC (p-16:0)	Hypertension	14	0.995 (0.978 to 1.013)	0.617	1.001 (0.994 to 1.009)	0.716	1.002 (0.997 to 1.008)	0.441		
Argininate	Hypertension	14	0.974 (0.938 to 1.011)	0.195	0.997 (0.990 to 1.005)	0.498	0.986 (0.972 to 1.001)	0.071	0.993 (0.988 to 0.999)	0.031

Exposure	Outcome	nSNP	MR Egger		Weighted median		Inverse variance weighted		MR_PRESSO	
			OR (95% CI)	pval	OR (95% CI)	pval	OR (95% CI)	pval	OR (95% CI)	pval
1-methyl-5-imidazoleacetate	Hypertension	12	1.007 (0.983 to 1.030)	0.589	1.000 (0.990 to 1.009)	0.915	1.001 (0.992 to 1.010)	0.828		
Hydantoin-5-propionate	Hypertension	7	1.018 (0.983 to 1.053)	0.367	1.000 (0.990 to 1.010)	0.985	1.000 (0.991 to 1.009)	0.963		
2-oxoarginine	Hypertension	11	0.961 (0.889 to 1.039)	0.341	1.003 (0.993 to 1.012)	0.584	0.991 (0.966 to 1.016)	0.487	0.999 (0.993 to 1.005)	0.666
Pantoate	Hypertension	12	0.993 (0.969 to 1.017)	0.553	1.008 (0.999 to 1.017)	0.089	1.007 (0.997 to 1.016)	0.161	1.010 (1.003 to 1.017)	0.021
Formiminoglutamate	Hypertension	12	1.018 (0.990 to 1.047)	0.248	0.999 (0.990 to 1.008)	0.839	0.992 (0.977 to 1.007)	0.294	1.000 (0.994 to 1.006)	0.878
1-(1-enyl-palmitoyl)-GPE (p-16:0)	Hypertension	13	1.004 (0.990 to 1.020)	0.569	0.999 (0.991 to 1.007)	0.773	0.998 (0.992 to 1.005)	0.596		

Exposure	Outcome	nSNP	MR Egger		Weighted median		Inverse variance weighted		MR_PRESSO	
			OR (95% CI)	pval	OR (95% CI)	pval	OR (95% CI)	pval	OR (95% CI)	pval
S-methylcysteine sulfoxide	Hypertension	8	1.000 (0.977 to 1.023)	0.992	1.008 (0.997 to 1.020)	0.157	1.002 (0.992 to 1.012)	0.707		
2-o-methylascorbic acid	Hypertension	17	0.994 (0.989 to 1.000)	0.051	0.994 (0.991 to 0.998)	8.26e-04	0.995 (0.992 to 0.998)	0.001		
Eicosanedioate (C20-DC)	Hypertension	7	1.009 (0.985 to 1.034)	0.500	0.998 (0.988 to 1.007)	0.632	0.998 (0.991 to 1.006)	0.623		
1-(1-enyl-stearoyl)-GPE (p-18:0)	Hypertension	12	0.987 (0.928 to 1.050)	0.681	1.005 (0.996 to 1.014)	0.242	1.001 (0.986 to 1.016)	0.888	1.007 (1.000 to 1.014)	0.090
Beta-citrylglutamate	Hypertension	10	1.003 (0.996 to 1.011)	0.423	0.999 (0.995 to 1.003)	0.686	0.998 (0.994 to 1.003)	0.456		
N-oleoyltaurine	Hypertension	18	0.992 (0.983 to 1.002)	0.146	0.999 (0.993 to 1.005)	0.662	0.999 (0.995 to 1.003)	0.662		

Exposure	Outcome	nSNP	MR Egger		Weighted median		Inverse variance weighted		MR_PRESSO	
			OR (95% CI)	pval	OR (95% CI)	pval	OR (95% CI)	pval	OR (95% CI)	pval
Docosadioate (C22-DC)	Hypertension	10	1.006 (0.987 to 1.024)	0.577	0.993 (0.983 to 1.002)	0.141	0.995 (0.985 to 1.005)	0.307		
Oleoyl-linoleoyl-glycerol (18:1/18:2) [2]	Hypertension	21	1.003 (0.985 to 1.021)	0.740	1.009 (1.001 to 1.016)	0.018	1.007 (0.999 to 1.015)	0.068	1.007 (1.000 to 1.014)	0.055
Imidazole propionate	Hypertension	15	0.979 (0.955 to 1.004)	0.119	0.993 (0.986 to 1.001)	0.102	1.002 (0.992 to 1.011)	0.748	0.996 (0.990 to 1.001)	0.172
Carnitine C18:2	Hypertension	15	1.005 (0.991 to 1.020)	0.465	1.002 (0.996 to 1.008)	0.546	1.004 (0.998 to 1.011)	0.188	1.001 (0.997 to 1.006)	0.608
Pregnanediol-3-glucuronide	Hypertension	17	0.996 (0.980 to 1.011)	0.594	0.996 (0.991 to 1.002)	0.160	0.997 (0.990 to 1.003)	0.335	0.997 (0.992 to 1.001)	0.200
Carboxyethyl-gaba	Hypertension	12	1.017 (0.975 to 1.060)	0.460	1.013 (1.004 to 1.023)	0.007	1.008 (0.997 to 1.019)	0.157	1.011 (1.002 to 1.021)	0.035

Exposure	Outcome	nSNP	MR Egger		Weighted median		Inverse variance weighted		MR_PRESSO	
			OR (95% CI)	pval	OR (95% CI)	pval	OR (95% CI)	pval	OR (95% CI)	pval
Alliin	Hypertension	11	0.989 (0.980 to 0.997)	0.031	0.995 (0.991 to 0.999)	0.028	0.998 (0.992 to 1.005)	0.656		
Trimethylamine n-oxide	Hypertension	6	0.991 (0.969 to 1.013)	0.460	0.997 (0.986 to 1.008)	0.633	0.995 (0.986 to 1.004)	0.282		
Dihydroferulate	Hypertension	3	1.007 (0.985 to 1.029)	0.663	0.998 (0.984 to 1.014)	0.845	0.998 (0.986 to 1.010)	0.762		
Prolylglycine	Hypertension	12	1.000 (0.959 to 1.042)	0.997	1.002 (0.993 to 1.012)	0.605	1.011 (0.993 to 1.029)	0.224	1.002 (0.995 to 1.009)	0.609
N-palmitoylglycine	Hypertension	13	0.992 (0.977 to 1.008)	0.346	1.000 (0.994 to 1.007)	0.968	0.994 (0.987 to 1.002)	0.127	0.999 (0.991 to 1.007)	0.772
N-methyltaurine	Hypertension	15	1.010 (0.988 to 1.033)	0.385	0.998 (0.991 to 1.004)	0.484	0.996 (0.990 to 1.002)	0.217	0.998 (0.993 to 1.003)	0.452

Exposure	Outcome	nSNP	MR Egger		Weighted median		Inverse variance weighted		MR_PRESSO	
			OR (95% CI)	pval	OR (95% CI)	pval	OR (95% CI)	pval	OR (95% CI)	pval
Succinimide	Hypertension	8	0.999 (0.982 to 1.016)	0.916	1.001 (0.992 to 1.009)	0.887	0.999 (0.993 to 1.005)	0.789		
N-acetylcarnosine	Hypertension	16	1.010 (0.989 to 1.032)	0.366	1.000 (0.993 to 1.007)	0.963	0.996 (0.988 to 1.005)	0.427	0.999 (0.993 to 1.004)	0.674
Histidine betaine (hercynine)	Hypertension	13	0.996 (0.976 to 1.016)	0.714	0.994 (0.986 to 1.002)	0.123	0.994 (0.986 to 1.002)	0.115	0.998 (0.992 to 1.003)	0.448
2-stearoyl-GPE (18:0)	Hypertension	19	0.996 (0.981 to 1.013)	0.671	1.002 (0.995 to 1.009)	0.522	0.999 (0.993 to 1.005)	0.813	0.998 (0.993 to 1.003)	0.378
Margaroylcarnitine (C17)	Hypertension	12	0.988 (0.958 to 1.020)	0.481	0.999 (0.991 to 1.008)	0.877	1.003 (0.993 to 1.014)	0.554	0.998 (0.991 to 1.004)	0.495
(R)-3-hydroxybutyrylcarnitine	Hypertension	10	0.990 (0.959 to 1.022)	0.545	1.004 (0.995 to 1.014)	0.370	1.007 (0.996 to 1.018)	0.216	1.002 (0.995 to 1.009)	0.604

Exposure	Outcome	nSNP	MR Egger		Weighted median		Inverse variance weighted		MR_PRESSO	
			OR (95% CI)	pval	OR (95% CI)	pval	OR (95% CI)	pval	OR (95% CI)	pval
Lanthionine	Hypertension	16	0.986 (0.953 to 1.019)	0.409	0.994 (0.987 to 1.002)	0.132	1.001 (0.989 to 1.014)	0.876	0.995 (0.991 to 0.999)	0.024
Mannonate	Hypertension	10	0.980 (0.963 to 0.997)	0.054	0.989 (0.982 to 0.997)	0.005	0.992 (0.983 to 1.000)	0.059	0.990 (0.982 to 0.997)	0.026
3b-hydroxy-5-cholenoic acid	Hypertension	13	1.000 (0.972 to 1.030)	0.977	0.995 (0.987 to 1.003)	0.194	0.999 (0.988 to 1.011)	0.919	0.996 (0.992 to 1.001)	0.153
2-hydroxydecanoate	Hypertension	11	1.007 (0.985 to 1.030)	0.562	1.005 (0.996 to 1.014)	0.267	1.002 (0.993 to 1.011)	0.624		
Glycohyocholate	Hypertension	10	1.011 (0.984 to 1.038)	0.455	0.991 (0.983 to 1.000)	0.048	0.988 (0.974 to 1.001)	0.075	0.991 (0.985 to 0.997)	0.019
3-methyl catechol sulfate (2)	Hypertension	13	1.004 (0.976 to 1.034)	0.778	1.000 (0.991 to 1.009)	0.992	0.995 (0.983 to 1.008)	0.485	0.995 (0.987 to 1.003)	0.250

Exposure	Outcome	nSNP	MR Egger		Weighted median		Inverse variance weighted		MR_PRESSO	
			OR (95% CI)	pval	OR (95% CI)	pval	OR (95% CI)	pval	OR (95% CI)	pval
2-aminooctanoate	Hypertension	17	0.997 (0.981 to 1.013)	0.675	0.996 (0.992 to 0.999)	0.018	0.999 (0.989 to 1.008)	0.784	1.002 (0.995 to 1.009)	0.609
3-methyl catechol sulfate (1)	Hypertension	7	0.984 (0.937 to 1.032)	0.533	1.003 (0.990 to 1.016)	0.642	0.994 (0.975 to 1.013)	0.525	0.997 (0.984 to 1.011)	0.739
4-methylcatechol sulfate	Hypertension	14	0.983 (0.954 to 1.013)	0.294	0.994 (0.984 to 1.004)	0.216	0.992 (0.979 to 1.005)	0.251	0.993 (0.986 to 1.001)	0.108
Guaiacol sulfate	Hypertension	14	1.003 (0.982 to 1.024)	0.795	1.004 (0.995 to 1.013)	0.371	0.998 (0.988 to 1.008)	0.661	0.999 (0.994 to 1.005)	0.797
Gamma-CEHC glucuronide	Hypertension	14	0.994 (0.975 to 1.013)	0.557	0.997 (0.989 to 1.005)	0.432	1.000 (0.993 to 1.007)	0.997	1.004 (0.999 to 1.010)	0.164
Dimethyl sulfone	Hypertension	12	1.005 (0.993 to 1.017)	0.471	1.005 (0.998 to 1.013)	0.180	1.006 (0.999 to 1.012)	0.079		

Exposure	Outcome	nSNP	MR Egger		Weighted median		Inverse variance weighted		MR_PRESSO	
			OR (95% CI)	pval	OR (95% CI)	pval	OR (95% CI)	pval	OR (95% CI)	pval
2-piperidinone	Hypertension	12	0.991 (0.972 to 1.010)	0.354	1.001 (0.992 to 1.010)	0.849	1.006 (0.997 to 1.015)	0.202	1.003 (0.996 to 1.010)	0.447
Indolin-2-one	Hypertension	8	0.997 (0.976 to 1.017)	0.760	1.003 (0.995 to 1.011)	0.412	1.002 (0.996 to 1.008)	0.546		
Sphingomyelin (d18:1/14:0, d16:1/16:0)	Hypertension	18	1.001 (0.997 to 1.004)	0.724	1.001 (0.998 to 1.004)	0.444	1.000 (0.996 to 1.003)	0.760		
3-acetylphenol sulfate	Hypertension	11	0.992 (0.972 to 1.013)	0.468	1.001 (0.992 to 1.010)	0.847	1.006 (0.997 to 1.016)	0.205	1.002 (0.995 to 1.009)	0.611
Sphingomyelin (d18:2/16:0, d18:1/16:1)	Hypertension	14	1.010 (0.991 to 1.030)	0.318	0.997 (0.988 to 1.006)	0.537	0.997 (0.989 to 1.006)	0.546		
S-allylcysteine	Hypertension	10	0.989 (0.968 to 1.010)	0.337	1.004 (0.996 to 1.013)	0.350	1.000 (0.993 to 1.007)	0.981		

Exposure	Outcome	nSNP	MR Egger		Weighted median		Inverse variance weighted		MR_PRESSO	
			OR (95% CI)	pval	OR (95% CI)	pval	OR (95% CI)	pval	OR (95% CI)	pval
6-oxopiperidine-2-carboxylate	Hypertension	13	0.991 (0.983 to 0.999)	0.043	0.993 (0.988 to 0.997)	0.003	0.996 (0.990 to 1.001)	0.141	0.995 (0.990 to 0.999)	0.037
2-aminophenol sulfate	Hypertension	14	1.007 (0.991 to 1.024)	0.406	1.003 (0.995 to 1.012)	0.481	0.999 (0.992 to 1.006)	0.831		
N-acetylalliin	Hypertension	15	1.005 (0.992 to 1.018)	0.495	0.998 (0.992 to 1.005)	0.612	0.999 (0.994 to 1.004)	0.737	0.995 (0.991 to 1.000)	0.076
2-aminoheptanoate	Hypertension	10	1.003 (0.979 to 1.027)	0.834	0.999 (0.989 to 1.009)	0.860	1.000 (0.991 to 1.009)	0.948		
3-methoxytyramine sulfate	Hypertension	9	0.991 (0.972 to 1.010)	0.392	1.000 (0.991 to 1.010)	0.942	1.001 (0.994 to 1.009)	0.750		
N2,N5-diacetylornithine	Hypertension	16	1.003 (0.990 to 1.016)	0.673	1.005 (1.001 to 1.010)	0.016	1.003 (0.995 to 1.010)	0.496	1.002 (0.994 to 1.009)	0.626

Exposure	Outcome	nSNP	MR Egger		Weighted median		Inverse variance weighted		MR_PRESSO	
			OR (95% CI)	pval	OR (95% CI)	pval	OR (95% CI)	pval	OR (95% CI)	pval
Ferulic acid 4-sulfate	Hypertension	18	1.004 (0.992 to 1.016)	0.532	1.008 (1.003 to 1.013)	0.003	1.006 (1.000 to 1.012)	0.033	1.007 (1.002 to 1.012)	0.015
1H-indole-7-acetic acid	Hypertension	12	0.995 (0.982 to 1.009)	0.516	1.007 (0.999 to 1.015)	0.108	1.002 (0.995 to 1.009)	0.581		
3-(3-hydroxyphenyl)propionate sulfate	Hypertension	11	0.994 (0.973 to 1.015)	0.581	1.006 (0.997 to 1.015)	0.215	1.002 (0.994 to 1.011)	0.593	1.006 (1.000 to 1.012)	0.077
1-linolenoyl-GPC (18:3)	Hypertension	19	1.004 (0.985 to 1.023)	0.699	1.003 (0.996 to 1.011)	0.412	1.002 (0.996 to 1.008)	0.534	1.000 (0.994 to 1.006)	0.996
O-sulfo-l-tyrosine	Hypertension	17	0.997 (0.951 to 1.045)	0.891	0.995 (0.987 to 1.002)	0.178	1.009 (0.989 to 1.029)	0.365	0.997 (0.991 to 1.003)	0.296
N-formylanthranilic acid	Hypertension	16	1.004 (1.000 to 1.008)	0.100	1.003 (0.999 to 1.006)	0.100	1.002 (0.998 to 1.005)	0.330		

Exposure	Outcome	nSNP	MR Egger		Weighted median		Inverse variance weighted		MR_PRESSO	
			OR (95% CI)	pval	OR (95% CI)	pval	OR (95% CI)	pval	OR (95% CI)	pval
Methionine sulfone	Hypertension	24	0.994 (0.982 to 1.006)	0.340	0.995 (0.990 to 0.999)	0.018	0.993 (0.987 to 1.000)	0.053	0.996 (0.992 to 1.000)	0.049
Acisoga	Hypertension	19	1.006 (0.997 to 1.016)	0.222	1.004 (0.998 to 1.010)	0.149	1.006 (1.002 to 1.010)	0.008		
17alpha-hydroxypregnanolone glucuronide	Hypertension	18	0.996 (0.989 to 1.003)	0.313	0.998 (0.993 to 1.002)	0.298	0.999 (0.994 to 1.003)	0.576		
Etiocholanolone glucuronide	Hypertension	14	1.000 (0.994 to 1.005)	0.865	0.998 (0.994 to 1.002)	0.297	0.999 (0.996 to 1.003)	0.748		
Fructosyllysine	Hypertension	15	1.009 (0.991 to 1.028)	0.351	1.007 (0.998 to 1.016)	0.121	1.009 (1.002 to 1.017)	0.019	1.012 (1.005 to 1.019)	0.005
1-(1-enyl-oleoyl)-GPE (p-18:1)	Hypertension	6	1.038 (1.007 to 1.070)	0.071	1.009 (0.997 to 1.021)	0.155	1.006 (0.997 to 1.016)	0.207		

Exposure	Outcome	nSNP	MR Egger		Weighted median		Inverse variance weighted		MR_PRESSO	
			OR (95% CI)	pval	OR (95% CI)	pval	OR (95% CI)	pval	OR (95% CI)	pval
N-acetyltaurine	Hypertension	12	0.997 (0.960 to 1.036)	0.884	0.996 (0.988 to 1.004)	0.362	1.007 (0.986 to 1.028)	0.517	1.001 (0.993 to 1.009)	0.775
1-palmitoyl-GPG (16:0)	Hypertension	17	0.995 (0.984 to 1.005)	0.308	0.998 (0.991 to 1.005)	0.563	1.002 (0.998 to 1.007)	0.358		
1-oleoyl-GPG (18:1)	Hypertension	13	0.992 (0.982 to 1.003)	0.184	0.998 (0.994 to 1.003)	0.441	1.001 (0.995 to 1.007)	0.782	0.999 (0.996 to 1.002)	0.543
Octadecenedioylcarnitine (C18:1-DC)	Hypertension	14	0.996 (0.989 to 1.004)	0.374	0.998 (0.995 to 1.002)	0.375	0.997 (0.993 to 1.002)	0.231	0.998 (0.994 to 1.003)	0.477
3-methylglutarylcarnitine (2)	Hypertension	21	0.986 (0.975 to 0.996)	0.018	1.001 (0.994 to 1.007)	0.864	0.998 (0.991 to 1.004)	0.474	1.001 (0.994 to 1.007)	0.870
Sphingomyelin (d18:1/24:1, d18:2/24:0)	Hypertension	8	0.999 (0.970 to 1.029)	0.968	0.994 (0.983 to 1.005)	0.305	1.000 (0.990 to 1.010)	0.987		

Exposure	Outcome	nSNP	MR Egger		Weighted median		Inverse variance weighted		MR_PRESSO	
			OR (95% CI)	pval	OR (95% CI)	pval	OR (95% CI)	pval	OR (95% CI)	pval
Methyl glucopyranoside (alpha + beta)	Hypertension	16	0.996 (0.989 to 1.003)	0.237	0.998 (0.994 to 1.002)	0.259	0.999 (0.994 to 1.003)	0.590		
9-hydroxystearate	Hypertension	14	0.970 (0.925 to 1.017)	0.232	0.988 (0.978 to 0.998)	0.023	0.986 (0.974 to 0.998)	0.024	0.987 (0.978 to 0.995)	0.012
Sphingomyelin (d18:2/14:0, d18:1/14:1)	Hypertension	14	0.987 (0.955 to 1.021)	0.474	0.998 (0.988 to 1.009)	0.766	0.999 (0.984 to 1.014)	0.868	1.003 (0.994 to 1.012)	0.529
1-dihomo-linolenylglycerol (20:3)	Hypertension	13	1.002 (0.984 to 1.021)	0.833	1.000 (0.993 to 1.008)	0.899	1.000 (0.993 to 1.006)	0.965		
4-hydroxychlorothalonil	Hypertension	12	0.996 (0.990 to 1.002)	0.206	0.999 (0.994 to 1.004)	0.702	1.000 (0.996 to 1.004)	0.964		
Vanillactate	Hypertension	16	0.995 (0.983 to 1.008)	0.495	1.005 (0.997 to 1.013)	0.254	1.003 (0.997 to 1.010)	0.276		

Exposure	Outcome	nSNP	MR Egger		Weighted median		Inverse variance weighted		MR_PRESSO	
			OR (95% CI)	pval	OR (95% CI)	pval	OR (95% CI)	pval	OR (95% CI)	pval
Carnitine C14:1	Hypertension	14	1.014 (0.997 to 1.031)	0.132	1.000 (0.991 to 1.009)	0.999	1.003 (0.995 to 1.011)	0.478		
N-formylphenylalanine	Hypertension	14	0.998 (0.975 to 1.020)	0.832	1.004 (0.996 to 1.012)	0.318	1.002 (0.993 to 1.011)	0.680	1.002 (0.996 to 1.008)	0.504
2-acetamidophenol sulfate	Hypertension	8	1.020 (0.996 to 1.043)	0.148	0.998 (0.989 to 1.008)	0.688	1.001 (0.993 to 1.008)	0.890		
4-vinylguaiacol sulfate	Hypertension	6	1.026 (1.005 to 1.048)	0.074	1.006 (0.995 to 1.017)	0.267	1.008 (0.998 to 1.018)	0.100		
5alpha-androstan-3alpha,17beta-diol monosulfate (2)	Hypertension	16	0.977 (0.965 to 0.989)	0.002	0.996 (0.988 to 1.004)	0.340	0.999 (0.990 to 1.008)	0.814	0.995 (0.988 to 1.002)	0.161
Vanillic alcohol sulfate	Hypertension	13	0.999 (0.989 to 1.010)	0.904	1.000 (0.994 to 1.007)	0.938	0.997 (0.991 to 1.002)	0.194		

Exposure	Outcome	nSNP	MR Egger		Weighted median		Inverse variance weighted		MR_PRESSO	
			OR (95% CI)	pval	OR (95% CI)	pval	OR (95% CI)	pval	OR (95% CI)	pval
Methyl-4-hydroxybenzoate sulfate	Hypertension	13	1.003 (0.988 to 1.019)	0.680	1.001 (0.993 to 1.010)	0.782	1.004 (0.997 to 1.010)	0.257		
Eugenol sulfate	Hypertension	10	1.000 (0.985 to 1.016)	0.978	1.000 (0.991 to 1.008)	0.941	0.999 (0.993 to 1.005)	0.759		
2-methoxyresorcinol sulfate	Hypertension	13	0.999 (0.996 to 1.002)	0.614	0.998 (0.995 to 1.002)	0.316	0.998 (0.995 to 1.000)	0.108		
Tyramine O-sulfate	Hypertension	10	1.005 (0.993 to 1.016)	0.450	1.003 (0.995 to 1.012)	0.440	1.002 (0.996 to 1.008)	0.523		
4-methylguaiacol sulfate	Hypertension	9	1.020 (0.993 to 1.047)	0.187	0.999 (0.988 to 1.010)	0.818	1.001 (0.991 to 1.012)	0.816	0.998 (0.990 to 1.006)	0.614
3-hydroxypyridine sulfate	Hypertension	12	0.996 (0.964 to 1.029)	0.814	1.000 (0.989 to 1.011)	0.983	0.994 (0.981 to 1.007)	0.399	0.996 (0.986 to 1.006)	0.462

Exposure	Outcome	nSNP	MR Egger		Weighted median		Inverse variance weighted		MR_PRESSO	
			OR (95% CI)	pval	OR (95% CI)	pval	OR (95% CI)	pval	OR (95% CI)	pval
Arabonate/xylonate	Hypertension	15	1.002 (0.970 to 1.035)	0.892	1.000 (0.991 to 1.009)	0.969	1.000 (0.988 to 1.012)	0.992	1.001 (0.994 to 1.009)	0.725
Octadecanedioylcarnitine (C18-DC)	Hypertension	16	0.996 (0.990 to 1.003)	0.253	0.998 (0.994 to 1.002)	0.296	0.998 (0.995 to 1.002)	0.360		
Phenylacetylcarnitine	Hypertension	15	1.007 (0.991 to 1.023)	0.426	1.005 (0.998 to 1.012)	0.152	1.006 (1.000 to 1.012)	0.057		
Ethylparaben sulfate	Hypertension	11	1.001 (0.981 to 1.022)	0.891	1.003 (0.994 to 1.012)	0.499	0.997 (0.988 to 1.005)	0.467	1.000 (0.994 to 1.007)	0.960
Sphingomyelin (d18:1/20:2, d18:2/20:1, d16:1/22:2)	Hypertension	13	0.985 (0.967 to 1.002)	0.118	1.002 (0.995 to 1.010)	0.517	0.998 (0.991 to 1.005)	0.574	1.002 (0.996 to 1.007)	0.520
Sphingomyelin (d18:1/20:1, d18:2/20:0)	Hypertension	18	1.004 (0.985 to 1.022)	0.693	0.998 (0.991 to 1.004)	0.497	0.997 (0.989 to 1.006)	0.537	0.994 (0.989 to 1.000)	0.053

Exposure	Outcome	nSNP	MR Egger		Weighted median		Inverse variance weighted		MR_PRESSO	
			OR (95% CI)	pval	OR (95% CI)	pval	OR (95% CI)	pval	OR (95% CI)	pval
Behenoyl sphingomyelin (d18:1/22:0)	Hypertension	10	0.979 (0.955 to 1.005)	0.153	1.002 (0.992 to 1.012)	0.747	1.002 (0.991 to 1.012)	0.747	0.998 (0.991 to 1.004)	0.500
Propyl 4-hydroxybenzoate sulfate	Hypertension	10	1.004 (0.967 to 1.043)	0.833	1.000 (0.989 to 1.011)	0.985	1.000 (0.986 to 1.014)	0.979	1.001 (0.989 to 1.013)	0.874
4-methoxyphenol sulfate	Hypertension	12	1.014 (0.997 to 1.031)	0.140	0.993 (0.985 to 1.001)	0.088	0.996 (0.989 to 1.004)	0.299		
Umbelliferone sulfate	Hypertension	10	0.992 (0.980 to 1.004)	0.244	0.993 (0.987 to 1.000)	0.059	0.992 (0.986 to 0.998)	0.005		
Sphingomyelin (d18:1/20:0, d16:1/22:0)	Hypertension	18	0.997 (0.973 to 1.021)	0.810	0.997 (0.991 to 1.003)	0.271	0.999 (0.990 to 1.008)	0.863	0.999 (0.995 to 1.003)	0.658
6-hydroxyindole sulfate	Hypertension	9	0.996 (0.966 to 1.026)	0.787	1.006 (0.996 to 1.016)	0.248	0.997 (0.984 to 1.010)	0.650	1.006 (1.001 to 1.011)	0.045

Exposure	Outcome	nSNP	MR Egger		Weighted median		Inverse variance weighted		MR_PRESSO	
			OR (95% CI)	pval	OR (95% CI)	pval	OR (95% CI)	pval	OR (95% CI)	pval
P-cresol glucuronide	Hypertension	15	1.013 (0.992 to 1.035)	0.242	1.005 (0.997 to 1.014)	0.221	1.004 (0.996 to 1.013)	0.288	1.002 (0.995 to 1.009)	0.576
N-carbamoylalanine	Hypertension	9	0.993 (0.971 to 1.016)	0.571	0.992 (0.982 to 1.002)	0.123	0.996 (0.988 to 1.003)	0.240		
Citraconate/glutaconate	Hypertension	12	1.002 (0.981 to 1.024)	0.850	1.001 (0.992 to 1.009)	0.911	0.995 (0.985 to 1.004)	0.267	0.999 (0.994 to 1.004)	0.773
1,2,3-benzenetriol sulfate (2)	Hypertension	10	1.001 (0.984 to 1.019)	0.874	0.993 (0.985 to 1.002)	0.155	0.996 (0.989 to 1.003)	0.248		
3-methoxycatechol sulfate (2)	Hypertension	13	0.992 (0.977 to 1.006)	0.273	0.997 (0.989 to 1.006)	0.556	1.000 (0.993 to 1.007)	0.945		
C-glycosyltryptophan	Hypertension	23	1.009 (0.961 to 1.059)	0.723	0.994 (0.987 to 1.002)	0.150	1.004 (0.988 to 1.020)	0.636	0.999 (0.993 to 1.006)	0.878

Exposure	Outcome	nSNP	MR Egger		Weighted median		Inverse variance weighted		MR_PRESSO	
			OR (95% CI)	pval	OR (95% CI)	pval	OR (95% CI)	pval	OR (95% CI)	pval
3-methoxycatechol sulfate (1)	Hypertension	14	1.001 (0.988 to 1.015)	0.847	1.000 (0.992 to 1.008)	0.966	0.999 (0.993 to 1.005)	0.672		
Sphingomyelin (d18:1/22:1, d18:2/22:0, d16:1/24:1)	Hypertension	15	0.989 (0.967 to 1.010)	0.323	0.992 (0.983 to 1.002)	0.105	0.999 (0.990 to 1.009)	0.914	1.000 (0.992 to 1.009)	0.932
Arabitol/xylitol	Hypertension	15	0.994 (0.949 to 1.041)	0.802	1.003 (0.994 to 1.011)	0.535	1.012 (0.993 to 1.032)	0.207	1.002 (0.996 to 1.008)	0.485
3-hydroxyhexanoate	Hypertension	8	0.986 (0.956 to 1.018)	0.424	0.998 (0.988 to 1.009)	0.781	0.994 (0.984 to 1.004)	0.244		
Sphingomyelin (d18:1/22:2, d18:2/22:1, d16:1/24:2)	Hypertension	14	0.981 (0.965 to 0.998)	0.044	0.987 (0.979 to 0.995)	0.002	0.991 (0.984 to 0.998)	0.013		
3beta-hydroxy-5-cholestenoate	Hypertension	17	1.001 (0.995 to 1.008)	0.715	1.001 (0.996 to 1.006)	0.615	1.000 (0.996 to 1.003)	0.915		

Exposure	Outcome	nSNP	MR Egger		Weighted median		Inverse variance weighted		MR_PRESSO	
			OR (95% CI)	pval	OR (95% CI)	pval	OR (95% CI)	pval	OR (95% CI)	pval
N-acetylkynurenine (2)	Hypertension	14	1.003 (0.997 to 1.010)	0.323	1.004 (1.000 to 1.007)	0.024	1.004 (1.001 to 1.007)	0.014		
Dopamine 4-sulfate	Hypertension	16	0.997 (0.988 to 1.006)	0.528	0.999 (0.993 to 1.006)	0.838	0.998 (0.993 to 1.002)	0.325		
Sphingomyelin (d17:1/16:0, d18:1/15:0, d16:1/17:0)	Hypertension	15	0.994 (0.981 to 1.007)	0.366	0.991 (0.985 to 0.997)	0.004	0.996 (0.989 to 1.002)	0.186	0.999 (0.992 to 1.006)	0.798
Adipoylcarnitine (C6-DC)	Hypertension	14	0.992 (0.976 to 1.009)	0.372	0.997 (0.991 to 1.003)	0.353	0.997 (0.991 to 1.003)	0.303		
Dopamine 3-o-sulfate	Hypertension	9	1.003 (0.982 to 1.025)	0.773	1.012 (1.001 to 1.022)	0.025	1.007 (0.999 to 1.015)	0.089		
Lignoceroyl sphingomyelin (d18:1/24:0)	Hypertension	14	1.016 (0.979 to 1.054)	0.427	0.999 (0.991 to 1.007)	0.849	1.003 (0.990 to 1.017)	0.638	0.999 (0.993 to 1.006)	0.867

Exposure	Outcome	nSNP	MR Egger		Weighted median		Inverse variance weighted		MR_PRESSO	
			OR (95% CI)	pval	OR (95% CI)	pval	OR (95% CI)	pval	OR (95% CI)	pval
N-acetylglucosamine/n-acetylgalactosamine	Hypertension	13	0.998 (0.990 to 1.006)	0.665	0.999 (0.994 to 1.005)	0.850	0.998 (0.993 to 1.002)	0.267		
Glycochenodeoxycholate 3-sulfate	Hypertension	19	1.005 (0.990 to 1.019)	0.536	0.998 (0.992 to 1.004)	0.484	0.995 (0.988 to 1.003)	0.197	0.996 (0.992 to 1.000)	0.067
Trans 3,4-methyleneheptanoate	Hypertension	10	0.998 (0.974 to 1.023)	0.899	0.998 (0.988 to 1.007)	0.607	0.999 (0.991 to 1.007)	0.848		
Taurodeoxycholic acid 3-sulfate	Hypertension	10	0.997 (0.989 to 1.005)	0.500	0.997 (0.993 to 1.001)	0.163	0.998 (0.993 to 1.002)	0.336		
Phenol glucuronide	Hypertension	11	0.999 (0.987 to 1.010)	0.831	1.001 (0.995 to 1.008)	0.643	1.003 (0.998 to 1.007)	0.307		
Glycodeoxycholate 3-sulfate	Hypertension	17	0.997 (0.991 to 1.002)	0.271	0.998 (0.995 to 1.002)	0.441	0.999 (0.996 to 1.003)	0.710		

Exposure	Outcome	nSNP	MR Egger		Weighted median		Inverse variance weighted		MR_PRESSO	
			OR (95% CI)	pval	OR (95% CI)	pval	OR (95% CI)	pval	OR (95% CI)	pval
4-hydroxyphenylacetoylcarnitine	Hypertension	18	1.004 (0.991 to 1.017)	0.574	1.001 (0.995 to 1.008)	0.673	1.000 (0.994 to 1.006)	0.948	0.999 (0.993 to 1.004)	0.634
1-stearoyl-2-oleoyl-gpc (18:0/18:1)	Hypertension	21	0.991 (0.975 to 1.007)	0.261	1.001 (0.994 to 1.008)	0.779	0.998 (0.993 to 1.003)	0.432		
Linoleoyl ethanolamide	Hypertension	11	1.003 (0.989 to 1.017)	0.692	1.005 (0.997 to 1.012)	0.202	1.004 (0.999 to 1.010)	0.095		
1,2-dilinoleoyl-GPC (18:2/18:2)	Hypertension	10	1.004 (0.993 to 1.014)	0.512	1.001 (0.996 to 1.007)	0.635	0.999 (0.993 to 1.005)	0.663		
Nonanoylcarnitine (C9)	Hypertension	12	1.007 (1.000 to 1.013)	0.061	1.004 (0.999 to 1.008)	0.112	1.004 (1.000 to 1.008)	0.028		
2-hydroxybutyrate/2-hydroxyisobutyrate	Hypertension	6	0.994 (0.963 to 1.026)	0.727	1.003 (0.992 to 1.015)	0.566	1.007 (0.995 to 1.019)	0.228		

Exposure	Outcome	nSNP	MR Egger		Weighted median		Inverse variance weighted		MR_PRESSO	
			OR (95% CI)	pval	OR (95% CI)	pval	OR (95% CI)	pval	OR (95% CI)	pval
Sphingomyelin (d18:2/23:0, d18:1/23:1, d17:1/24:1)	Hypertension	11	0.993 (0.963 to 1.023)	0.644	0.999 (0.987 to 1.010)	0.799	0.996 (0.984 to 1.008)	0.501	0.998 (0.987 to 1.008)	0.659
Tricosanoyl sphingomyelin (d18:1/23:0)	Hypertension	15	1.008 (0.981 to 1.036)	0.589	0.996 (0.987 to 1.006)	0.436	1.001 (0.989 to 1.012)	0.903	0.996 (0.987 to 1.005)	0.423
Palmitoyl dihydrosphingomyelin (d18:0/16:0)	Hypertension	23	1.018 (0.996 to 1.040)	0.130	1.003 (0.995 to 1.010)	0.530	1.006 (0.998 to 1.015)	0.154	1.006 (0.999 to 1.014)	0.128
1-palmitoleoylglycerol (16:1)	Hypertension	20	0.999 (0.983 to 1.015)	0.877	1.001 (0.994 to 1.008)	0.794	0.995 (0.988 to 1.002)	0.160	0.997 (0.991 to 1.003)	0.361
1-stearoyl-2-linoleoyl-gpc (18:0/18:2)	Hypertension	17	0.992 (0.974 to 1.011)	0.425	1.002 (0.995 to 1.009)	0.544	0.999 (0.991 to 1.007)	0.800	1.002 (0.996 to 1.007)	0.590
1-palmitoyl-2-palmitoleoyl-gpc (16:0/16:1)	Hypertension	14	0.977 (0.963 to 0.991)	0.009	0.998 (0.990 to 1.007)	0.680	1.001 (0.992 to 1.009)	0.840	0.998 (0.991 to 1.005)	0.611

Exposure	Outcome	nSNP	MR Egger		Weighted median		Inverse variance weighted		MR_PRESSO	
			OR (95% CI)	pval	OR (95% CI)	pval	OR (95% CI)	pval	OR (95% CI)	pval
1-stearoyl-2-linoleoyl-GPE (18:0/18:2)	Hypertension	16	1.004 (0.994 to 1.014)	0.463	1.002 (0.998 to 1.006)	0.405	1.001 (0.997 to 1.006)	0.578	1.000 (0.996 to 1.004)	0.990
Sphingomyelin (d18:2/24:1, d18:1/24:2)	Hypertension	16	1.017 (0.980 to 1.055)	0.396	1.002 (0.993 to 1.012)	0.634	0.996 (0.984 to 1.007)	0.460	1.000 (0.991 to 1.008)	0.916
1-palmitoyl-2-stearoyl-gpc (16:0/18:0)	Hypertension	20	0.997 (0.985 to 1.008)	0.575	0.998 (0.992 to 1.004)	0.497	1.000 (0.995 to 1.006)	0.854		
Oleate/vaccenate (18:1)	Hypertension	13	1.021 (0.992 to 1.051)	0.189	1.000 (0.991 to 1.009)	0.972	0.998 (0.983 to 1.013)	0.796	1.001 (0.994 to 1.009)	0.739
1-palmitoyl-2-docosahexaenoyl-gpc (16:0/22:6)	Hypertension	12	0.983 (0.952 to 1.014)	0.298	0.999 (0.991 to 1.006)	0.729	1.007 (0.997 to 1.017)	0.176	1.004 (0.996 to 1.013)	0.335
1-(1-enyl-stearoyl)-2-oleoyl-GPE (p-18:0/18:1)	Hypertension	10	0.994 (0.962 to 1.027)	0.724	1.004 (0.994 to 1.015)	0.426	0.996 (0.983 to 1.010)	0.602	1.005 (0.996 to 1.013)	0.297

Exposure	Outcome	nSNP	MR Egger		Weighted median		Inverse variance weighted		MR_PRESSO	
			OR (95% CI)	pval	OR (95% CI)	pval	OR (95% CI)	pval	OR (95% CI)	pval
1-stearoyl-2-arachidonoyl-GPE (18:0/20:4)	Hypertension	18	0.995 (0.987 to 1.003)	0.270	1.001 (0.997 to 1.005)	0.649	1.003 (0.998 to 1.008)	0.236		
1-stearoyl-2-docosahexaenoyl-gpc (18:0/22:6)	Hypertension	18	0.993 (0.979 to 1.007)	0.326	0.998 (0.993 to 1.004)	0.608	1.003 (0.997 to 1.009)	0.361	1.001 (0.996 to 1.006)	0.659
Sphingomyelin (d18:1/17:0, d17:1/18:0, d19:1/16:0)	Hypertension	17	0.993 (0.970 to 1.016)	0.543	0.996 (0.988 to 1.005)	0.401	0.996 (0.987 to 1.004)	0.314	0.997 (0.988 to 1.005)	0.439
1-(1-enyl-palmitoyl)-2-arachidonoyl-GPE (p-16:0/20:4)	Hypertension	11	1.006 (0.993 to 1.020)	0.379	0.999 (0.991 to 1.007)	0.801	0.995 (0.989 to 1.002)	0.165		
Myristoyl dihydrosphingomyelin (d18:0/14:0)	Hypertension	13	0.989 (0.976 to 1.002)	0.123	0.999 (0.991 to 1.006)	0.727	1.000 (0.994 to 1.005)	0.857		
5-hydroxyindole sulfate	Hypertension	10	0.998 (0.983 to 1.013)	0.775	1.005 (0.997 to 1.013)	0.261	1.003 (0.997 to 1.009)	0.341		

Exposure	Outcome	nSNP	MR Egger		Weighted median		Inverse variance weighted		MR_PRESSO	
			OR (95% CI)	pval	OR (95% CI)	pval	OR (95% CI)	pval	OR (95% CI)	pval
1-palmitoyl-2-dihomo-linolenoyl-GPC (16:0/20:3n3 or 6)	Hypertension	15	1.000 (0.993 to 1.008)	0.972	1.001 (0.997 to 1.006)	0.561	1.001 (0.997 to 1.005)	0.692		
1-(1-enyl-palmitoyl)-2-arachidonoyl-gpc (p-16:0/20:4)	Hypertension	23	1.002 (0.992 to 1.011)	0.725	0.999 (0.995 to 1.002)	0.440	0.999 (0.993 to 1.004)	0.673	0.998 (0.995 to 1.002)	0.299
Sphingomyelin (d18:1/21:0, d17:1/22:0, d16:1/23:0)	Hypertension	12	0.996 (0.978 to 1.015)	0.696	0.991 (0.984 to 0.998)	0.008	0.992 (0.983 to 1.001)	0.096	0.992 (0.985 to 1.000)	0.075
1-palmitoyl-2-arachidonoyl-GPE (16:0/20:4)	Hypertension	14	1.002 (0.994 to 1.010)	0.637	1.001 (0.997 to 1.004)	0.666	1.001 (0.997 to 1.006)	0.524	1.001 (0.997 to 1.005)	0.724
Lactosyl-N-palmitoyl-sphingosine (d18:1/16:0)	Hypertension	14	0.994 (0.979 to 1.009)	0.458	1.000 (0.993 to 1.008)	0.954	0.998 (0.992 to 1.004)	0.484		
Sphingomyelin (d18:0/18:0, d19:0/17:0)	Hypertension	19	0.987 (0.963 to 1.012)	0.316	1.001 (0.993 to 1.008)	0.808	1.007 (0.997 to 1.016)	0.158	1.004 (0.999 to 1.009)	0.137

Exposure	Outcome	nSNP	MR Egger		Weighted median		Inverse variance weighted		MR_PRESSO	
			OR (95% CI)	pval	OR (95% CI)	pval	OR (95% CI)	pval	OR (95% CI)	pval
1-(1-enyl-palmitoyl)-2-oleoyl-gpc (p-16:0/18:1)	Hypertension	16	1.007 (0.985 to 1.030)	0.535	0.995 (0.987 to 1.004)	0.294	0.998 (0.990 to 1.006)	0.584	0.995 (0.988 to 1.002)	0.202
1-(1-enyl-stearoyl)-2-arachidonoyl-GPE (p-18:0/20:4)	Hypertension	13	0.999 (0.978 to 1.020)	0.910	0.997 (0.992 to 1.002)	0.297	0.992 (0.981 to 1.002)	0.127	0.992 (0.982 to 1.003)	0.189
Behenoyl dihydrosphingomyelin (d18:0/22:0)	Hypertension	23	1.003 (0.978 to 1.028)	0.819	1.007 (1.000 to 1.015)	0.056	1.005 (0.996 to 1.014)	0.320	1.002 (0.994 to 1.010)	0.631
1-(1-enyl-palmitoyl)-2-linoleoyl-GPC (p-16:0/18:2)	Hypertension	11	1.006 (0.974 to 1.039)	0.715	0.992 (0.982 to 1.002)	0.127	0.993 (0.983 to 1.004)	0.196	0.996 (0.986 to 1.006)	0.456
1-(1-enyl-palmitoyl)-2-oleoyl-GPE (p-16:0/18:1)	Hypertension	18	1.010 (0.989 to 1.031)	0.372	1.002 (0.995 to 1.010)	0.558	0.995 (0.986 to 1.005)	0.330	1.000 (0.993 to 1.007)	0.993
1-palmitoyl-2-arachidonoyl-GPI (16:0/20:4)	Hypertension	14	0.994 (0.981 to 1.007)	0.361	0.998 (0.991 to 1.005)	0.558	1.000 (0.995 to 1.005)	0.909		

Exposure	Outcome	nSNP	MR Egger		Weighted median		Inverse variance weighted		MR_PRESSO	
			OR (95% CI)	pval	OR (95% CI)	pval	OR (95% CI)	pval	OR (95% CI)	pval
1-palmitoyl-2-docosahexaenoyl-GPE (16:0/22:6)	Hypertension	13	0.997 (0.986 to 1.009)	0.654	1.001 (0.997 to 1.005)	0.600	1.004 (0.998 to 1.011)	0.219	1.005 (0.999 to 1.010)	0.160
N-palmitoyl-sphinganine (d18:0/16:0)	Hypertension	17	1.005 (0.968 to 1.043)	0.810	1.000 (0.992 to 1.008)	0.983	1.001 (0.989 to 1.014)	0.831	1.001 (0.995 to 1.008)	0.700
1-stearoyl-2-docosahexaenoyl-GPE (18:0/22:6)	Hypertension	17	0.998 (0.983 to 1.014)	0.832	1.002 (0.997 to 1.007)	0.453	1.005 (0.998 to 1.012)	0.196	1.003 (0.998 to 1.009)	0.275
Gamma-tocopherol/beta-tocopherol	Hypertension	12	1.005 (0.976 to 1.036)	0.734	0.995 (0.986 to 1.005)	0.329	1.002 (0.991 to 1.013)	0.753	0.997 (0.991 to 1.002)	0.269
1-stearoyl-2-linoleoyl-GPI (18:0/18:2)	Hypertension	16	1.011 (0.993 to 1.030)	0.240	1.001 (0.996 to 1.006)	0.688	1.000 (0.992 to 1.007)	0.910	1.001 (0.997 to 1.006)	0.517
1,2-dilinoleoyl-GPE (18:2/18:2)	Hypertension	10	0.993 (0.975 to 1.012)	0.505	1.002 (0.995 to 1.008)	0.620	1.003 (0.996 to 1.011)	0.376	1.001 (0.996 to 1.007)	0.636

Exposure	Outcome	nSNP	MR Egger		Weighted median		Inverse variance weighted		MR_PRESSO	
			OR (95% CI)	pval	OR (95% CI)	pval	OR (95% CI)	pval	OR (95% CI)	pval
Phenylacetylglutamate	Hypertension	13	0.986 (0.967 to 1.005)	0.181	0.996 (0.988 to 1.004)	0.312	0.995 (0.989 to 1.002)	0.181	1.000 (0.994 to 1.006)	0.965
1-(1-enyl-palmitoyl)-2-palmitoleoyl-GPC (P-16:0/16:1)	Hypertension	23	1.006 (0.992 to 1.020)	0.405	1.007 (1.002 to 1.012)	0.004	0.999 (0.992 to 1.007)	0.874	1.003 (0.996 to 1.010)	0.373
Palmitoylcholine	Hypertension	19	1.006 (0.993 to 1.020)	0.386	1.004 (0.996 to 1.011)	0.324	1.003 (0.997 to 1.009)	0.345		
Thioproline	Hypertension	10	0.985 (0.969 to 1.001)	0.097	1.000 (0.992 to 1.009)	0.971	1.001 (0.992 to 1.010)	0.833		
1-linoleoyl-2-arachidonoyl-GPC (18:2/20:4n6)	Hypertension	20	0.992 (0.982 to 1.003)	0.171	0.997 (0.992 to 1.002)	0.253	0.994 (0.989 to 0.999)	0.012	0.995 (0.991 to 1.000)	0.067
1-linoleoyl-GPG (18:2)	Hypertension	15	0.998 (0.991 to 1.004)	0.482	0.998 (0.993 to 1.003)	0.484	0.999 (0.995 to 1.003)	0.613		

Exposure	Outcome	nSNP	MR Egger		Weighted median		Inverse variance weighted		MR_PRESSO	
			OR (95% CI)	pval	OR (95% CI)	pval	OR (95% CI)	pval	OR (95% CI)	pval
1-(1-enyl-palmitoyl)-2-linoleoyl-GPE (p-16:0/18:2)	Hypertension	13	1.021 (0.978 to 1.066)	0.365	0.996 (0.989 to 1.003)	0.245	0.993 (0.982 to 1.004)	0.191	0.997 (0.992 to 1.001)	0.174
1-(1-enyl-stearoyl)-2-linoleoyl-GPE (p-18:0/18:2)	Hypertension	12	0.990 (0.954 to 1.026)	0.588	1.001 (0.993 to 1.009)	0.802	0.993 (0.982 to 1.005)	0.283	1.001 (0.995 to 1.007)	0.745
1-stearoyl-2-oleoyl-GPI (18:0/18:1)	Hypertension	14	0.995 (0.960 to 1.033)	0.812	1.000 (0.994 to 1.006)	0.973	1.000 (0.992 to 1.008)	0.971	1.001 (0.996 to 1.005)	0.766
1-myristoyl-2-arachidonoyl-GPC (14:0/20:4)	Hypertension	18	0.994 (0.982 to 1.005)	0.306	0.998 (0.993 to 1.002)	0.334	0.997 (0.990 to 1.003)	0.344	0.999 (0.995 to 1.002)	0.478
1-oleoyl-2-arachidonoyl-GPE (18:1/20:4)	Hypertension	18	1.000 (0.993 to 1.007)	0.968	1.001 (0.997 to 1.005)	0.640	1.002 (0.998 to 1.006)	0.251	1.002 (0.998 to 1.005)	0.339
1-palmitoyl-2-oleoyl-GPI (16:0/18:1)	Hypertension	15	0.995 (0.973 to 1.016)	0.625	1.002 (0.995 to 1.009)	0.571	1.001 (0.994 to 1.009)	0.737	1.004 (0.999 to 1.008)	0.121

Exposure	Outcome	nSNP	MR Egger		Weighted median		Inverse variance weighted		MR_PRESSO	
			OR (95% CI)	pval	OR (95% CI)	pval	OR (95% CI)	pval	OR (95% CI)	pval
1-oleoyl-2-docosahexaenoyl-GPC (18:1/22:6)	Hypertension	21	0.993 (0.969 to 1.018)	0.607	1.001 (0.994 to 1.008)	0.811	1.004 (0.995 to 1.013)	0.403	1.000 (0.995 to 1.006)	0.908
1-oleoyl-2-linoleoyl-GPE (18:1/18:2)	Hypertension	17	1.002 (0.995 to 1.010)	0.549	1.001 (0.998 to 1.005)	0.465	1.001 (0.997 to 1.005)	0.708	1.001 (0.998 to 1.004)	0.696
1-(1-enyl-palmitoyl)-2-palmitoyl-GPC (P-16:0/16:0)	Hypertension	21	1.010 (1.000 to 1.020)	0.068	1.007 (1.002 to 1.011)	0.005	1.001 (0.996 to 1.007)	0.688	0.996 (0.991 to 1.002)	0.196
Oleoylcholine	Hypertension	8	1.013 (0.985 to 1.042)	0.392	1.002 (0.991 to 1.013)	0.677	0.997 (0.986 to 1.008)	0.601	1.002 (0.994 to 1.010)	0.632
Glycosyl-N-palmitoyl-sphingosine (d18:1/16:0)	Hypertension	15	0.997 (0.986 to 1.007)	0.569	0.997 (0.992 to 1.003)	0.379	0.996 (0.991 to 1.001)	0.161		
Arachidonoylcholine	Hypertension	10	0.998 (0.982 to 1.015)	0.832	0.998 (0.990 to 1.006)	0.645	0.998 (0.992 to 1.004)	0.569		

Exposure	Outcome	nSNP	MR Egger		Weighted median		Inverse variance weighted		MR_PRESSO	
			OR (95% CI)	pval	OR (95% CI)	pval	OR (95% CI)	pval	OR (95% CI)	pval
2-methylserine	Hypertension	12	0.995 (0.962 to 1.030)	0.784	1.000 (0.991 to 1.009)	0.969	1.008 (0.993 to 1.023)	0.287	1.001 (0.995 to 1.007)	0.776
(S)-3-hydroxybutyrylcarnitine	Hypertension	13	1.007 (0.979 to 1.036)	0.653	1.004 (0.995 to 1.013)	0.388	0.998 (0.989 to 1.007)	0.656	0.996 (0.988 to 1.005)	0.426
Methylsuccinoylcarnitine	Hypertension	20	0.998 (0.989 to 1.007)	0.689	0.999 (0.994 to 1.004)	0.615	1.000 (0.996 to 1.004)	0.982		
Dihomo-linolenoyl-choline	Hypertension	15	1.004 (0.973 to 1.037)	0.794	1.001 (0.993 to 1.009)	0.745	1.002 (0.991 to 1.014)	0.706	1.000 (0.993 to 1.006)	0.927
Docosahexaenoylcholine	Hypertension	10	1.001 (0.975 to 1.028)	0.958	1.002 (0.992 to 1.012)	0.720	0.996 (0.986 to 1.007)	0.500	1.000 (0.991 to 1.009)	0.985
Glycocholate glucuronide (1)	Hypertension	10	1.001 (0.988 to 1.013)	0.929	0.999 (0.992 to 1.006)	0.779	1.000 (0.994 to 1.006)	0.995		

Exposure	Outcome	nSNP	MR Egger		Weighted median		Inverse variance weighted		MR_PRESSO	
			OR (95% CI)	pval	OR (95% CI)	pval	OR (95% CI)	pval	OR (95% CI)	pval
Caffeic acid sulfate	Hypertension	16	0.996 (0.978 to 1.014)	0.644	0.997 (0.989 to 1.005)	0.432	0.992 (0.986 to 0.999)	0.023		
Ascorbic acid 2-sulfate	Hypertension	14	1.001 (0.983 to 1.020)	0.878	1.001 (0.993 to 1.009)	0.824	1.000 (0.994 to 1.007)	0.925		
1-linoleoyl-2-linolenoyl-GPC (18:2/18:3)	Hypertension	13	1.007 (0.991 to 1.023)	0.427	1.002 (0.996 to 1.009)	0.525	1.000 (0.993 to 1.008)	0.897		
Catechol glucuronide	Hypertension	14	0.998 (0.983 to 1.013)	0.788	0.997 (0.991 to 1.004)	0.430	0.998 (0.993 to 1.003)	0.513		
Furaneol sulfate	Hypertension	9	0.997 (0.981 to 1.014)	0.723	0.995 (0.989 to 1.001)	0.125	0.994 (0.988 to 1.001)	0.113		
Ceramide (d18:1/24:1)	Hypertension	19	0.999 (0.988 to 1.011)	0.902	1.001 (0.996 to 1.006)	0.717	1.002 (0.996 to 1.008)	0.522	1.001 (0.996 to 1.006)	0.749

Exposure	Outcome	nSNP	MR Egger		Weighted median		Inverse variance weighted		MR_PRESSO	
			OR (95% CI)	pval	OR (95% CI)	pval	OR (95% CI)	pval	OR (95% CI)	pval
N-oleoylserine	Hypertension	11	1.001 (0.985 to 1.016)	0.946	1.001 (0.994 to 1.008)	0.719	0.998 (0.992 to 1.003)	0.385		
Perfluorooctanesulfonate (PFOS)	Hypertension	13	0.977 (0.954 to 1.002)	0.095	0.995 (0.986 to 1.004)	0.270	1.001 (0.990 to 1.011)	0.887	0.997 (0.990 to 1.004)	0.369
Pimeloylcarnitine/3-methyladipoylcarnitine (C7-DC)	Hypertension	14	0.994 (0.980 to 1.008)	0.383	0.999 (0.992 to 1.005)	0.755	1.000 (0.994 to 1.007)	0.928	0.999 (0.994 to 1.003)	0.580
1-palmitoleoyl-2-linolenoyl-GPC (16:1/18:3)	Hypertension	14	0.997 (0.985 to 1.009)	0.653	1.002 (0.997 to 1.008)	0.432	1.001 (0.996 to 1.007)	0.702		
Linoleoyl-arachidonoyl-glycerol (18:2/20:4) [1]	Hypertension	10	0.986 (0.966 to 1.006)	0.207	0.997 (0.992 to 1.002)	0.286	0.998 (0.987 to 1.008)	0.657	0.999 (0.995 to 1.002)	0.466
Hexadecadienoate (16:2n6)	Hypertension	7	0.992 (0.967 to 1.017)	0.546	1.003 (0.990 to 1.015)	0.667	1.000 (0.989 to 1.011)	0.978		

Exposure	Outcome	nSNP	MR Egger		Weighted median		Inverse variance weighted		MR_PRESSO	
			OR (95% CI)	pval	OR (95% CI)	pval	OR (95% CI)	pval	OR (95% CI)	pval
Glycosyl-N-behenoyl-sphingadienine (d18:2/22:0)	Hypertension	21	0.999 (0.992 to 1.007)	0.838	0.997 (0.992 to 1.002)	0.271	0.998 (0.994 to 1.001)	0.218		
4-hydroxyphenylacetylglutamine	Hypertension	14	1.007 (0.941 to 1.078)	0.846	0.999 (0.990 to 1.008)	0.810	1.009 (0.989 to 1.031)	0.381	0.999 (0.993 to 1.006)	0.797
Gamma-glutamyl-alpha-lysine	Hypertension	17	1.003 (0.991 to 1.014)	0.649	1.004 (0.997 to 1.010)	0.300	1.001 (0.995 to 1.006)	0.811		
N-palmitoyl-sphingadienine (d18:2/16:0)	Hypertension	11	0.965 (0.927 to 1.005)	0.118	0.994 (0.983 to 1.005)	0.258	0.996 (0.984 to 1.008)	0.488	0.997 (0.987 to 1.006)	0.502
Linoleoyl-arachidonoyl-glycerol (18:2/20:4) [2]	Hypertension	24	0.994 (0.985 to 1.002)	0.161	1.000 (0.995 to 1.005)	0.953	0.999 (0.995 to 1.004)	0.804	1.001 (0.998 to 1.004)	0.584
Palmitoleoylcarnitine (C16:1)	Hypertension	11	0.998 (0.956 to 1.042)	0.931	1.001 (0.991 to 1.011)	0.855	1.001 (0.986 to 1.016)	0.921	1.003 (0.996 to 1.012)	0.417

Exposure	Outcome	nSNP	MR Egger		Weighted median		Inverse variance weighted		MR_PRESSO	
			OR (95% CI)	pval	OR (95% CI)	pval	OR (95% CI)	pval	OR (95% CI)	pval
N-stearoyl-sphingadienine (d18:2/18:0)	Hypertension	12	1.010 (0.992 to 1.028)	0.308	0.997 (0.989 to 1.006)	0.523	0.995 (0.988 to 1.001)	0.111		
2,3-dihydroxy-2-methylbutyrate	Hypertension	8	0.988 (0.969 to 1.007)	0.245	0.998 (0.987 to 1.009)	0.720	0.998 (0.990 to 1.006)	0.646		
Stearoylcholine	Hypertension	10	0.981 (0.957 to 1.005)	0.157	1.003 (0.993 to 1.014)	0.571	1.012 (0.999 to 1.026)	0.080	1.006 (0.998 to 1.013)	0.183
Glycosyl-N-tricosanoyl-sphingadienine (d18:2/23:0)	Hypertension	11	1.003 (0.987 to 1.019)	0.733	1.002 (0.995 to 1.009)	0.610	1.005 (0.998 to 1.011)	0.153		
N-palmitoyl-heptadecasphingosine (d17:1/16:0)	Hypertension	17	0.987 (0.966 to 1.009)	0.261	0.999 (0.992 to 1.007)	0.873	0.994 (0.986 to 1.003)	0.218	0.998 (0.992 to 1.003)	0.403
Ceramide (d18:1/14:0, d16:1/16:0)	Hypertension	18	0.985 (0.974 to 0.997)	0.026	0.988 (0.982 to 0.995)	3.02e-04	0.992 (0.987 to 0.998)	0.004		

Exposure	Outcome	nSNP	MR Egger		Weighted median		Inverse variance weighted		MR_PRESSO	
			OR (95% CI)	pval	OR (95% CI)	pval	OR (95% CI)	pval	OR (95% CI)	pval
Glycosyl-N-(2-hydroxynervonoyl)-sphingosine (d18:1/24:1(2OH))	Hypertension	16	1.012 (0.991 to 1.034)	0.269	1.002 (0.996 to 1.007)	0.601	0.999 (0.990 to 1.009)	0.899	1.002 (0.996 to 1.007)	0.511
Glycosyl ceramide (d18:2/24:1, d18:1/24:2)	Hypertension	19	1.001 (0.992 to 1.010)	0.854	0.997 (0.991 to 1.003)	0.388	0.996 (0.992 to 1.001)	0.100		
Glycosyl ceramide (d18:1/23:1, d17:1/24:1)	Hypertension	10	0.991 (0.968 to 1.014)	0.458	1.000 (0.993 to 1.007)	0.965	0.999 (0.992 to 1.006)	0.711	1.002 (0.998 to 1.007)	0.376
Sphingadienine	Hypertension	11	1.016 (1.002 to 1.031)	0.055	1.011 (1.003 to 1.019)	0.008	1.005 (0.999 to 1.011)	0.116		
Ceramide (d18:1/17:0, d17:1/18:0)	Hypertension	13	1.000 (0.984 to 1.018)	0.957	1.004 (0.996 to 1.011)	0.368	1.001 (0.993 to 1.008)	0.889		
Nisinate (24:6n3)	Hypertension	10	0.988 (0.969 to 1.007)	0.251	0.995 (0.989 to 1.002)	0.172	0.996 (0.990 to 1.002)	0.207		

Exposure	Outcome	nSNP	MR Egger		Weighted median		Inverse variance weighted		MR_PRESSO	
			OR (95% CI)	pval	OR (95% CI)	pval	OR (95% CI)	pval	OR (95% CI)	pval
Sphingomyelin (d18:0/20:0, d16:0/22:0)	Hypertension	27	0.998 (0.987 to 1.010)	0.766	1.002 (0.996 to 1.007)	0.558	1.004 (0.998 to 1.010)	0.177	1.002 (0.998 to 1.006)	0.389
Sphingomyelin (d18:2/24:2)	Hypertension	14	0.998 (0.982 to 1.013)	0.767	0.997 (0.989 to 1.006)	0.525	0.992 (0.986 to 0.998)	0.013		
Sphingomyelin (d17:2/16:0, d18:2/15:0)	Hypertension	17	0.986 (0.969 to 1.004)	0.148	0.989 (0.981 to 0.998)	0.016	0.997 (0.989 to 1.005)	0.490	0.998 (0.991 to 1.004)	0.512
Sphingomyelin (d18:2/23:1)	Hypertension	15	0.992 (0.972 to 1.012)	0.439	0.997 (0.988 to 1.006)	0.506	0.995 (0.987 to 1.003)	0.240	0.997 (0.991 to 1.002)	0.242
Behenoylcarnitine (C22)	Hypertension	19	0.996 (0.979 to 1.014)	0.679	1.001 (0.995 to 1.007)	0.764	1.003 (0.995 to 1.010)	0.485	1.002 (0.997 to 1.007)	0.476
Sphingomyelin (d18:2/18:1)	Hypertension	8	1.007 (0.985 to 1.029)	0.576	0.998 (0.987 to 1.009)	0.713	0.997 (0.989 to 1.006)	0.526		

Exposure	Outcome	nSNP	MR Egger		Weighted median		Inverse variance weighted		MR_PRESSO	
			OR (95% CI)	pval	OR (95% CI)	pval	OR (95% CI)	pval	OR (95% CI)	pval
Sphingomyelin (d18:2/21:0, d16:2/23:0)	Hypertension	22	0.987 (0.966 to 1.009)	0.269	0.994 (0.986 to 1.003)	0.178	0.990 (0.981 to 0.998)	0.020	0.993 (0.986 to 0.999)	0.046
Linoleoylcholine	Hypertension	15	1.005 (0.992 to 1.017)	0.476	0.999 (0.991 to 1.007)	0.742	1.000 (0.994 to 1.006)	0.954		
Sphingomyelin (d18:1/19:0, d19:1/18:0)	Hypertension	15	0.989 (0.970 to 1.009)	0.289	0.998 (0.991 to 1.004)	0.452	0.999 (0.991 to 1.008)	0.858	1.001 (0.995 to 1.007)	0.728
Linolenoylcarnitine (C18:3)	Hypertension	17	1.003 (0.988 to 1.017)	0.727	0.997 (0.989 to 1.004)	0.379	1.000 (0.994 to 1.006)	0.932		
Heneicosapentaenoate (21:5n3)	Hypertension	8	1.004 (0.990 to 1.019)	0.573	1.000 (0.991 to 1.008)	0.947	0.999 (0.992 to 1.005)	0.669		
Sphingomyelin (d18:1/25:0, d19:0/24:1, d20:1/23:0, d19:1/24:0)	Hypertension	20	1.002 (0.995 to 1.008)	0.577	1.000 (0.996 to 1.004)	0.971	0.998 (0.994 to 1.002)	0.338		

Exposure	Outcome	nSNP	MR Egger		Weighted median		Inverse variance weighted		MR_PRESSO	
			OR (95% CI)	pval	OR (95% CI)	pval	OR (95% CI)	pval	OR (95% CI)	pval
Arachidoylcarnitine (C20)	Hypertension	16	0.998 (0.975 to 1.021)	0.874	1.006 (0.998 to 1.014)	0.150	1.005 (0.997 to 1.013)	0.220	0.998 (0.992 to 1.004)	0.577
Cerotoylcarnitine (C26)	Hypertension	22	1.009 (0.997 to 1.022)	0.167	1.002 (0.997 to 1.008)	0.391	1.003 (0.997 to 1.009)	0.360	0.999 (0.995 to 1.004)	0.778
Lignoceroylcarnitine (C24)	Hypertension	16	1.007 (0.997 to 1.018)	0.186	0.998 (0.992 to 1.003)	0.407	0.999 (0.994 to 1.004)	0.747	0.997 (0.993 to 1.001)	0.190
Docosatrienoate (22:3n6)	Hypertension	11	0.996 (0.983 to 1.009)	0.545	0.995 (0.989 to 1.002)	0.137	0.995 (0.990 to 1.000)	0.043		
Dihomo-linolenoylcarnitine (C20:3n3 or 6)	Hypertension	22	1.004 (0.997 to 1.010)	0.331	1.002 (0.998 to 1.006)	0.273	1.004 (1.000 to 1.007)	0.050	1.002 (0.999 to 1.005)	0.274
Carotene diol (2)	Hypertension	14	1.015 (0.990 to 1.041)	0.272	0.999 (0.993 to 1.006)	0.835	0.999 (0.989 to 1.010)	0.909	1.000 (0.993 to 1.007)	0.986

Exposure	Outcome	nSNP	MR Egger		Weighted median		Inverse variance weighted		MR_PRESSO	
			OR (95% CI)	pval	OR (95% CI)	pval	OR (95% CI)	pval	OR (95% CI)	pval
Ethyl alpha-glucopyranoside	Hypertension	15	0.978 (0.965 to 0.992)	0.008	0.996 (0.988 to 1.003)	0.241	0.997 (0.991 to 1.003)	0.350		
Carotene diol (1)	Hypertension	17	1.010 (0.989 to 1.031)	0.363	0.999 (0.993 to 1.005)	0.824	0.999 (0.990 to 1.007)	0.778	1.001 (0.994 to 1.008)	0.799
Nervonoylcarnitine (C24:1)	Hypertension	15	1.007 (0.988 to 1.027)	0.485	0.996 (0.989 to 1.003)	0.305	1.003 (0.995 to 1.010)	0.490	0.999 (0.994 to 1.005)	0.838
Dihomo-linoleoylcarnitine (C20:2)	Hypertension	18	1.005 (0.994 to 1.016)	0.402	1.000 (0.995 to 1.006)	0.877	1.001 (0.996 to 1.006)	0.673	0.998 (0.994 to 1.003)	0.443
Glycosyl ceramide (d18:1/20:0, d16:1/22:0)	Hypertension	21	0.986 (0.977 to 0.995)	0.008	0.997 (0.992 to 1.002)	0.307	0.997 (0.993 to 1.002)	0.258	0.998 (0.994 to 1.001)	0.258
Docosahexaenoylcarnitine (C22:6)	Hypertension	20	0.998 (0.976 to 1.020)	0.866	0.999 (0.993 to 1.006)	0.801	1.003 (0.995 to 1.012)	0.409	0.998 (0.994 to 1.002)	0.398

Exposure	Outcome	nSNP	MR Egger		Weighted median		Inverse variance weighted		MR_PRESSO	
			OR (95% CI)	pval	OR (95% CI)	pval	OR (95% CI)	pval	OR (95% CI)	pval
N,N,N-trimethyl-5-aminovalerate	Hypertension	23	0.992 (0.967 to 1.019)	0.578	1.008 (1.001 to 1.015)	0.025	1.008 (0.998 to 1.017)	0.127	1.001 (0.995 to 1.007)	0.700
Eicosenoylcarnitine (C20:1)	Hypertension	21	1.010 (0.987 to 1.034)	0.397	1.008 (1.000 to 1.015)	0.053	1.007 (0.999 to 1.015)	0.089	1.008 (1.001 to 1.015)	0.030
Ximenoylcarnitine (C26:1)	Hypertension	20	1.003 (0.993 to 1.012)	0.585	1.003 (0.997 to 1.009)	0.299	1.002 (0.998 to 1.007)	0.290	1.001 (0.997 to 1.004)	0.774
Arachidonoylcarnitine (C20:4)	Hypertension	23	1.000 (0.993 to 1.007)	0.991	1.001 (0.998 to 1.005)	0.452	1.004 (1.000 to 1.009)	0.048	1.002 (0.999 to 1.005)	0.274
5-dodecenoylcarnitine (C12:1)	Hypertension	14	0.985 (0.953 to 1.017)	0.371	0.996 (0.987 to 1.006)	0.486	0.993 (0.982 to 1.004)	0.223	0.996 (0.988 to 1.005)	0.424
2-butenoylglycine	Hypertension	15	0.979 (0.960 to 0.999)	0.061	0.990 (0.983 to 0.998)	0.011	0.993 (0.986 to 1.001)	0.084	1.001 (0.994 to 1.008)	0.739

Exposure	Outcome	nSNP	MR Egger		Weighted median		Inverse variance weighted		MR_PRESSO	
			OR (95% CI)	pval	OR (95% CI)	pval	OR (95% CI)	pval	OR (95% CI)	pval
(N(1) + N(8))-acetylspermidine	Hypertension	21	0.995 (0.985 to 1.006)	0.430	1.002 (0.996 to 1.008)	0.617	1.000 (0.994 to 1.006)	0.902	1.004 (0.999 to 1.010)	0.153
Hydroxy-cmpf	Hypertension	11	0.993 (0.964 to 1.024)	0.686	1.001 (0.991 to 1.010)	0.881	1.000 (0.989 to 1.010)	0.956	1.002 (0.997 to 1.008)	0.435
2-furoylcarnitine	Hypertension	7	1.011 (0.987 to 1.035)	0.416	0.998 (0.988 to 1.008)	0.668	0.989 (0.977 to 1.003)	0.113	0.995 (0.989 to 1.002)	0.204
Cortolone glucuronide (1)	Hypertension	17	1.000 (0.975 to 1.025)	0.973	0.998 (0.990 to 1.007)	0.681	1.004 (0.994 to 1.015)	0.437	1.001 (0.993 to 1.008)	0.890
Carotene diol (3)	Hypertension	15	1.007 (0.991 to 1.023)	0.428	0.999 (0.993 to 1.006)	0.791	0.998 (0.991 to 1.004)	0.479	1.000 (0.995 to 1.005)	0.915
3-hydroxyoleoylcarnitine	Hypertension	17	0.999 (0.974 to 1.025)	0.957	1.000 (0.992 to 1.009)	0.946	1.002 (0.994 to 1.011)	0.585	0.997 (0.990 to 1.004)	0.466

Exposure	Outcome	nSNP	MR Egger		Weighted median		Inverse variance weighted		MR_PRESSO	
			OR (95% CI)	pval	OR (95% CI)	pval	OR (95% CI)	pval	OR (95% CI)	pval
Dodecenedioate (C12:1-DC)	Hypertension	14	1.008 (0.988 to 1.029)	0.457	1.002 (0.995 to 1.009)	0.658	1.001 (0.993 to 1.008)	0.844	1.001 (0.995 to 1.007)	0.805
Trans-2-hexenoylglycine	Hypertension	14	0.976 (0.959 to 0.993)	0.019	0.990 (0.982 to 0.999)	0.021	0.989 (0.981 to 0.997)	0.008	0.997 (0.991 to 1.002)	0.272
2-hydroxyarachidate	Hypertension	6	0.979 (0.942 to 1.018)	0.342	1.001 (0.987 to 1.014)	0.931	0.998 (0.982 to 1.013)	0.774	0.992 (0.979 to 1.004)	0.251
3-hydroxyphenylacetoylglutamine	Hypertension	2					1.002 (0.989 to 1.016)	0.750		
Heptenedioate (C7:1-DC)	Hypertension	8	1.001 (0.982 to 1.021)	0.903	1.004 (0.994 to 1.014)	0.479	1.006 (0.998 to 1.014)	0.167		
Hexadecenedioate (C16:1-DC)	Hypertension	21	0.998 (0.993 to 1.003)	0.410	0.998 (0.995 to 1.001)	0.255	0.999 (0.995 to 1.002)	0.437		

Exposure	Outcome	nSNP	MR Egger		Weighted median		Inverse variance weighted		MR_PRESSO	
			OR (95% CI)	pval	OR (95% CI)	pval	OR (95% CI)	pval	OR (95% CI)	pval
Octadecenedioate (C18:1-DC)	Hypertension	15	0.998 (0.991 to 1.005)	0.553	0.998 (0.994 to 1.003)	0.417	0.997 (0.994 to 1.000)	0.079		
Dihydroferulic acid sulfate	Hypertension	16	1.007 (0.996 to 1.018)	0.248	0.996 (0.990 to 1.002)	0.219	0.999 (0.994 to 1.004)	0.722		
Glutamine conjugate of C7H12O2	Hypertension	14	0.992 (0.976 to 1.009)	0.377	1.000 (0.992 to 1.009)	0.920	1.001 (0.995 to 1.008)	0.694		
Hydroxyasparagine	Hypertension	12	1.008 (0.919 to 1.106)	0.873	0.994 (0.983 to 1.006)	0.310	1.004 (0.973 to 1.036)	0.794	0.998 (0.989 to 1.009)	0.774
Sphingomyelin (d17:1/14:0, d16:1/15:0)	Hypertension	17	0.984 (0.961 to 1.007)	0.199	0.998 (0.989 to 1.007)	0.617	0.994 (0.985 to 1.004)	0.260	1.003 (0.996 to 1.009)	0.455
Octadecadienedioate (C18:2-DC)	Hypertension	16	0.998 (0.993 to 1.003)	0.445	0.998 (0.994 to 1.002)	0.309	0.997 (0.994 to 1.000)	0.085		

Exposure	Outcome	nSNP	MR Egger		Weighted median		Inverse variance weighted		MR_PRESSO	
			OR (95% CI)	pval	OR (95% CI)	pval	OR (95% CI)	pval	OR (95% CI)	pval
Perfluorooctanoate (PFOA)	Hypertension	12	1.011 (0.982 to 1.041)	0.476	0.989 (0.980 to 0.999)	0.025	0.990 (0.980 to 1.001)	0.064	0.994 (0.986 to 1.002)	0.172
3-carboxy-4-methyl-5-pentyl-2-furanpropionate (3-CMPFP)	Hypertension	14	0.997 (0.985 to 1.009)	0.627	0.998 (0.990 to 1.005)	0.565	0.999 (0.993 to 1.005)	0.629	1.003 (0.998 to 1.009)	0.270
Glutamine conjugate of C6H10O2 (2)	Hypertension	8	0.988 (0.959 to 1.018)	0.467	0.988 (0.978 to 0.999)	0.025	0.994 (0.983 to 1.005)	0.269		
3-hydroxybutyroylglycine	Hypertension	15	0.980 (0.970 to 0.990)	0.002	0.984 (0.979 to 0.990)	6.41e-09	0.989 (0.983 to 0.995)	5.06e-04	0.996 (0.989 to 1.003)	0.317
Glycine conjugate of C10H14O2 (1)	Hypertension	11	0.992 (0.979 to 1.006)	0.291	0.998 (0.991 to 1.005)	0.597	1.000 (0.993 to 1.007)	0.932	0.998 (0.992 to 1.004)	0.530
Glutamine conjugate of C6H10O2 (1)	Hypertension	9	0.976 (0.936 to 1.018)	0.297	0.987 (0.977 to 0.997)	0.013	0.996 (0.983 to 1.009)	0.548	0.992 (0.981 to 1.003)	0.208

Exposure	Outcome	nSNP	MR Egger		Weighted median		Inverse variance weighted		MR_PRESSO	
			OR (95% CI)	pval	OR (95% CI)	pval	OR (95% CI)	pval	OR (95% CI)	pval
N-methylhydroxyproline	Hypertension	8	0.989 (0.968 to 1.011)	0.365	0.998 (0.989 to 1.007)	0.678	0.993 (0.985 to 1.002)	0.125		
Glucuronide of C10H18O2 (7)	Hypertension	10	0.993 (0.979 to 1.007)	0.356	0.999 (0.991 to 1.007)	0.742	1.002 (0.996 to 1.008)	0.538		
Glyco-beta-muricholate	Hypertension	13	0.993 (0.987 to 0.998)	0.022	0.993 (0.989 to 0.998)	0.005	0.994 (0.990 to 0.997)	7.89e-04		
Glucuronide of C12H22O4 (1)	Hypertension	9	1.003 (0.986 to 1.020)	0.776	1.000 (0.993 to 1.008)	0.936	0.998 (0.992 to 1.004)	0.520		
N,n,n-trimethyl-alanylproline betaine (tmap)	Hypertension	13	0.995 (0.918 to 1.078)	0.907	0.994 (0.984 to 1.004)	0.273	1.008 (0.984 to 1.032)	0.529	0.995 (0.986 to 1.003)	0.267
Glycine conjugate of C10H12O2	Hypertension	12	1.013 (0.977 to 1.050)	0.495	1.000 (0.992 to 1.009)	0.957	0.999 (0.988 to 1.010)	0.813	1.000 (0.992 to 1.007)	0.946

Exposure	Outcome	nSNP	MR Egger		Weighted median		Inverse variance weighted		MR_PRESSO	
			OR (95% CI)	pval	OR (95% CI)	pval	OR (95% CI)	pval	OR (95% CI)	pval
N-acetyl-2-aminooctanoate	Hypertension	17	1.001 (0.991 to 1.012)	0.803	1.004 (1.001 to 1.008)	0.015	1.007 (1.000 to 1.014)	0.040	1.009 (1.003 to 1.016)	0.017
3-formylindole	Hypertension	17	1.001 (0.996 to 1.007)	0.650	1.001 (0.997 to 1.005)	0.569	1.000 (0.997 to 1.003)	0.858		
Gamma-glutamylcitrulline	Hypertension	19	0.997 (0.968 to 1.028)	0.864	0.998 (0.989 to 1.006)	0.604	0.996 (0.988 to 1.005)	0.391	0.998 (0.990 to 1.006)	0.629
8-methoxykynurenate	Hypertension	11	1.022 (0.990 to 1.055)	0.210	0.997 (0.989 to 1.006)	0.521	0.992 (0.982 to 1.003)	0.156	0.997 (0.990 to 1.005)	0.491
Tetradecadienoate (14:2)	Hypertension	13	1.010 (0.974 to 1.047)	0.597	1.002 (0.994 to 1.011)	0.603	0.999 (0.987 to 1.011)	0.919	1.002 (0.995 to 1.008)	0.669
3-amino-2-piperidone	Hypertension	8	1.018 (0.999 to 1.036)	0.109	1.002 (0.993 to 1.011)	0.681	1.005 (0.997 to 1.012)	0.207		

Exposure	Outcome	nSNP	MR Egger		Weighted median		Inverse variance weighted		MR_PRESSO	
			OR (95% CI)	pval	OR (95% CI)	pval	OR (95% CI)	pval	OR (95% CI)	pval
Glucuronide of piperine metabolite C17H21NO3 (3)	Hypertension	11	0.989 (0.966 to 1.012)	0.381	1.002 (0.993 to 1.011)	0.676	1.004 (0.994 to 1.013)	0.440	1.006 (0.997 to 1.015)	0.198
Dodecadienoate (12:2)	Hypertension	15	1.002 (0.991 to 1.014)	0.706	1.006 (1.000 to 1.013)	0.065	1.005 (0.999 to 1.011)	0.101		
N-lactoyl tyrosine	Hypertension	10	0.998 (0.983 to 1.013)	0.788	1.001 (0.993 to 1.010)	0.731	0.999 (0.993 to 1.006)	0.842		
N,N-dimethylalanine	Hypertension	17	0.996 (0.979 to 1.013)	0.642	1.001 (0.994 to 1.008)	0.803	1.002 (0.994 to 1.010)	0.682	1.001 (0.996 to 1.006)	0.661
Ethyl beta-glucopyranoside	Hypertension	10	0.999 (0.994 to 1.004)	0.736	0.998 (0.993 to 1.002)	0.311	0.998 (0.994 to 1.002)	0.248		
N-lactoyl phenylalanine	Hypertension	8	0.983 (0.959 to 1.006)	0.201	0.994 (0.983 to 1.004)	0.256	0.995 (0.985 to 1.004)	0.278		

Exposure	Outcome	nSNP	MR Egger		Weighted median		Inverse variance weighted		MR_PRESSO	
			OR (95% CI)	pval	OR (95% CI)	pval	OR (95% CI)	pval	OR (95% CI)	pval
N-acetyl-isoputreanine	Hypertension	23	1.009 (1.002 to 1.017)	0.025	1.010 (1.005 to 1.014)	1.85e-05	1.007 (1.003 to 1.011)	1.96e-04	1.008 (1.005 to 1.011)	1.19e-04
2,2'-Methylenebis(6-tert-butyl-p-cresol)	Hypertension	11	0.987 (0.969 to 1.006)	0.216	0.994 (0.985 to 1.003)	0.166	0.994 (0.987 to 1.001)	0.117		
N-lactoyl isoleucine	Hypertension	8	0.996 (0.974 to 1.018)	0.706	0.996 (0.986 to 1.006)	0.433	0.995 (0.988 to 1.003)	0.250		
2-hydroxysebacate	Hypertension	11	1.004 (0.990 to 1.017)	0.595	1.002 (0.994 to 1.009)	0.698	1.001 (0.995 to 1.008)	0.640		
Ascorbic acid 3-sulfate	Hypertension	13	1.010 (0.994 to 1.027)	0.250	1.002 (0.994 to 1.010)	0.668	1.004 (0.997 to 1.011)	0.235		
N-lactoyl valine	Hypertension	11	0.996 (0.950 to 1.044)	0.862	0.995 (0.983 to 1.006)	0.344	1.001 (0.989 to 1.013)	0.843	0.995 (0.985 to 1.004)	0.293

Exposure	Outcome	nSNP	MR Egger		Weighted median		Inverse variance weighted		MR_PRESSO	
			OR (95% CI)	pval	OR (95% CI)	pval	OR (95% CI)	pval	OR (95% CI)	pval
6-bromotryptophan	Hypertension	16	1.000 (0.994 to 1.006)	0.970	1.000 (0.996 to 1.005)	0.841	1.000 (0.996 to 1.004)	0.974		
Delta-CEHC	Hypertension	10	0.996 (0.946 to 1.048)	0.882	0.998 (0.987 to 1.008)	0.658	1.000 (0.985 to 1.014)	0.966	1.003 (0.995 to 1.010)	0.554
Indoleacetoylcarnitine	Hypertension	27	1.002 (0.995 to 1.009)	0.580	1.001 (0.996 to 1.005)	0.727	1.002 (0.998 to 1.006)	0.390	1.001 (0.998 to 1.005)	0.564
N-acetyl leucine	Hypertension	9	0.998 (0.976 to 1.022)	0.895	0.999 (0.989 to 1.009)	0.850	0.997 (0.990 to 1.004)	0.435		
3-indoleglyoxylic acid	Hypertension	12	1.004 (0.997 to 1.012)	0.247	1.001 (0.997 to 1.006)	0.544	1.000 (0.996 to 1.004)	0.963		
Sulfate of piperine metabolite C18H21NO3 (1)	Hypertension	12	1.014 (0.987 to 1.042)	0.330	0.997 (0.989 to 1.006)	0.494	0.993 (0.982 to 1.004)	0.232	1.001 (0.995 to 1.007)	0.824

Exposure	Outcome	nSNP	MR Egger		Weighted median		Inverse variance weighted		MR_PRESSO	
			OR (95% CI)	pval	OR (95% CI)	pval	OR (95% CI)	pval	OR (95% CI)	pval
Methyl vanillate sulfate	Hypertension	10	0.989 (0.970 to 1.009)	0.320	1.002 (0.994 to 1.009)	0.641	0.999 (0.992 to 1.005)	0.692		
Glucuronide of piperine metabolite C17H21NO3 (4)	Hypertension	10	0.994 (0.974 to 1.015)	0.606	0.997 (0.990 to 1.004)	0.405	0.998 (0.991 to 1.005)	0.655		
Sulfate of piperine metabolite C16H19NO3 (3)	Hypertension	16	1.004 (0.988 to 1.021)	0.637	1.001 (0.993 to 1.009)	0.773	1.006 (1.000 to 1.012)	0.070		
Sulfate of piperine metabolite C16H19NO3 (2)	Hypertension	16	0.995 (0.979 to 1.010)	0.490	1.005 (0.998 to 1.013)	0.176	1.006 (1.000 to 1.012)	0.071		
3-hydroxypyridine glucuronide	Hypertension	13	1.012 (0.992 to 1.033)	0.273	1.006 (0.999 to 1.013)	0.099	1.000 (0.991 to 1.008)	0.925	1.003 (0.998 to 1.008)	0.219
Glucuronide of piperine metabolite C17H21NO3 (5)	Hypertension	10	0.994 (0.973 to 1.015)	0.595	0.991 (0.983 to 0.999)	0.030	0.998 (0.990 to 1.005)	0.524		

Exposure	Outcome	nSNP	MR Egger		Weighted median		Inverse variance weighted		MR_PRESSO	
			OR (95% CI)	pval	OR (95% CI)	pval	OR (95% CI)	pval	OR (95% CI)	pval
2-naphthol sulfate	Hypertension	12	1.001 (0.988 to 1.014)	0.924	1.000 (0.992 to 1.008)	0.992	1.004 (0.997 to 1.010)	0.255		
11beta-hydroxyandrosterone glucuronide	Hypertension	20	0.984 (0.970 to 0.998)	0.042	0.997 (0.991 to 1.004)	0.424	1.005 (0.995 to 1.014)	0.340	1.001 (0.996 to 1.005)	0.792
4-ethylcatechol sulfate	Hypertension	10	0.985 (0.962 to 1.009)	0.250	0.996 (0.985 to 1.007)	0.485	0.986 (0.975 to 0.996)	0.009	0.990 (0.982 to 0.998)	0.042
Sulfate of piperine metabolite C18H21NO3 (3)	Hypertension	13	0.989 (0.973 to 1.006)	0.238	0.999 (0.992 to 1.006)	0.778	1.000 (0.994 to 1.006)	0.924		
(2,4 or 2,5)-dimethylphenol sulfate	Hypertension	10	1.004 (0.986 to 1.023)	0.654	1.005 (0.998 to 1.012)	0.132	1.004 (0.999 to 1.009)	0.133		
11beta-hydroxyetiocholanolone glucuronide	Hypertension	12	0.997 (0.981 to 1.013)	0.712	0.994 (0.985 to 1.002)	0.126	0.995 (0.989 to 1.001)	0.077		

Exposure	Outcome	nSNP	MR Egger		Weighted median		Inverse variance weighted		MR_PRESSO	
			OR (95% CI)	pval	OR (95% CI)	pval	OR (95% CI)	pval	OR (95% CI)	pval
Cholic acid glucuronide	Hypertension	11	1.017 (1.005 to 1.028)	0.022	1.006 (1.001 to 1.011)	0.026	1.002 (0.995 to 1.009)	0.599	0.999 (0.993 to 1.006)	0.862
4-allylcatechol sulfate	Hypertension	11	1.015 (1.001 to 1.029)	0.064	1.007 (0.998 to 1.017)	0.118	1.006 (0.999 to 1.014)	0.100		
Glycoursodeoxycholic acid sulfate (1)	Hypertension	15	1.006 (0.995 to 1.018)	0.305	1.002 (0.996 to 1.009)	0.536	0.999 (0.995 to 1.004)	0.735		
Dihydrocaffeate sulfate (2)	Hypertension	10	1.010 (0.991 to 1.029)	0.331	0.996 (0.988 to 1.005)	0.423	0.997 (0.989 to 1.005)	0.454		
4-methylhexanoylglutamine	Hypertension	5	0.978 (0.954 to 1.002)	0.164	1.004 (0.997 to 1.011)	0.299	1.005 (0.992 to 1.019)	0.444	1.004 (0.996 to 1.011)	0.453
2-ketocaprylate	Hypertension	18	0.987 (0.975 to 0.999)	0.055	1.002 (0.996 to 1.008)	0.597	1.001 (0.996 to 1.007)	0.702	1.004 (0.999 to 1.009)	0.123

Exposure	Outcome	nSNP	MR Egger		Weighted median		Inverse variance weighted		MR_PRESSO	
			OR (95% CI)	pval	OR (95% CI)	pval	OR (95% CI)	pval	OR (95% CI)	pval
3-hydroxy-2-methylpyridine sulfate	Hypertension	12	0.991 (0.965 to 1.017)	0.500	1.000 (0.991 to 1.009)	0.994	0.992 (0.981 to 1.002)	0.118	0.996 (0.989 to 1.004)	0.393
5-hydroxy-2-methylpyridine sulfate	Hypertension	16	1.004 (0.988 to 1.021)	0.623	0.998 (0.991 to 1.006)	0.638	0.996 (0.989 to 1.003)	0.272	1.000 (0.995 to 1.005)	0.888
3-ethylcatechol sulfate (2)	Hypertension	10	0.999 (0.980 to 1.018)	0.892	1.004 (0.997 to 1.012)	0.268	1.005 (0.998 to 1.012)	0.168		
3-hydroxyhexanoylcarnitine (1)	Hypertension	12	1.008 (0.987 to 1.031)	0.464	1.009 (1.001 to 1.018)	0.036	1.005 (0.998 to 1.012)	0.141		
2,6-dihydroxybenzoic acid	Hypertension	14	1.003 (0.979 to 1.027)	0.838	1.011 (1.002 to 1.019)	0.010	1.009 (1.001 to 1.016)	0.028		
3-ethylcatechol sulfate (1)	Hypertension	13	1.002 (0.978 to 1.027)	0.855	0.998 (0.989 to 1.007)	0.618	0.993 (0.984 to 1.002)	0.139	0.994 (0.988 to 1.000)	0.067

Exposure	Outcome	nSNP	MR Egger		Weighted median		Inverse variance weighted		MR_PRESSO	
			OR (95% CI)	pval	OR (95% CI)	pval	OR (95% CI)	pval	OR (95% CI)	pval
Lithocholate sulfate (1)	Hypertension	18	0.988 (0.976 to 1.001)	0.093	1.000 (0.993 to 1.006)	0.890	0.998 (0.992 to 1.003)	0.420		
4-acetylcatechol sulfate (1)	Hypertension	5	1.008 (0.986 to 1.031)	0.517	0.995 (0.982 to 1.007)	0.401	0.995 (0.985 to 1.004)	0.287		
2,3-dihydroxy-5-methylthio-4-pentenoate (dmtpa)	Hypertension	18	0.998 (0.939 to 1.060)	0.941	1.002 (0.994 to 1.011)	0.576	1.010 (0.989 to 1.031)	0.350	1.003 (0.996 to 1.009)	0.449
Picolinoylglycine	Hypertension	11	1.004 (0.953 to 1.058)	0.872	1.000 (0.991 to 1.010)	0.981	0.989 (0.970 to 1.007)	0.231	1.003 (0.998 to 1.008)	0.342
Succinoyltaurine	Hypertension	16	0.992 (0.984 to 1.000)	0.077	0.996 (0.991 to 1.001)	0.143	0.998 (0.993 to 1.002)	0.330	1.000 (0.995 to 1.004)	0.834
Undecenoylcarnitine (C11:1)	Hypertension	17	1.001 (0.990 to 1.013)	0.813	1.000 (0.995 to 1.005)	0.878	0.998 (0.993 to 1.003)	0.399	0.997 (0.992 to 1.002)	0.214

Exposure	Outcome	nSNP	MR Egger		Weighted median		Inverse variance weighted		MR_PRESSO	
			OR (95% CI)	pval	OR (95% CI)	pval	OR (95% CI)	pval	OR (95% CI)	pval
Palmitoyl-sphingosine-phosphoethanolamine (d18:1/16:0)	Hypertension	16	1.009 (0.986 to 1.033)	0.467	1.001 (0.992 to 1.010)	0.794	0.996 (0.986 to 1.006)	0.453	0.997 (0.989 to 1.006)	0.525
Pregnenetriol disulfate	Hypertension	12	1.002 (0.997 to 1.007)	0.468	1.001 (0.998 to 1.005)	0.456	1.000 (0.997 to 1.004)	0.845		
Tetradecadienedioate (C14:2-DC)	Hypertension	20	0.999 (0.988 to 1.010)	0.831	1.001 (0.996 to 1.006)	0.620	0.997 (0.991 to 1.003)	0.292	0.998 (0.992 to 1.004)	0.428
Hydroxy-N6,N6,N6-trimethyllysine	Hypertension	15	1.004 (0.974 to 1.034)	0.820	0.994 (0.984 to 1.003)	0.180	0.989 (0.977 to 1.001)	0.066	0.988 (0.980 to 0.996)	0.017
Pregnenetriol sulfate	Hypertension	22	1.000 (0.974 to 1.027)	0.988	1.003 (0.996 to 1.010)	0.414	1.006 (0.994 to 1.018)	0.311	1.005 (0.999 to 1.011)	0.149
Taurochenodeoxycholic acid 3-sulfate	Hypertension	19	0.995 (0.981 to 1.010)	0.509	1.004 (0.998 to 1.010)	0.160	1.006 (0.999 to 1.012)	0.077	1.004 (0.999 to 1.008)	0.144

Exposure	Outcome	nSNP	MR Egger		Weighted median		Inverse variance weighted		MR_PRESSO	
			OR (95% CI)	pval	OR (95% CI)	pval	OR (95% CI)	pval	OR (95% CI)	pval
Hydroxypalmitoyl sphingomyelin (d18:1/16:0(OH))	Hypertension	20	1.006 (0.992 to 1.020)	0.440	0.999 (0.994 to 1.005)	0.825	0.997 (0.990 to 1.004)	0.408	0.999 (0.996 to 1.003)	0.760
5-hydroxymethyl-2-furoylcarnitine	Hypertension	10	0.974 (0.926 to 1.025)	0.338	1.000 (0.991 to 1.008)	0.919	1.001 (0.992 to 1.010)	0.812	0.998 (0.990 to 1.007)	0.717
Eicosenedioate (C20:1-DC)	Hypertension	11	0.997 (0.990 to 1.005)	0.520	0.998 (0.992 to 1.003)	0.400	0.999 (0.994 to 1.003)	0.634		
3-hydroxydecanoylcarnitine	Hypertension	17	0.998 (0.983 to 1.013)	0.802	1.000 (0.992 to 1.008)	0.971	1.004 (0.996 to 1.012)	0.352		
Levulinoylcarnitine	Hypertension	13	1.008 (0.995 to 1.021)	0.269	0.998 (0.992 to 1.005)	0.643	1.000 (0.994 to 1.006)	0.953		
Cis 3,4-methyleneheptanoate	Hypertension	18	0.993 (0.972 to 1.014)	0.522	1.007 (0.998 to 1.015)	0.114	1.010 (1.000 to 1.020)	0.062	1.005 (0.999 to 1.012)	0.129

Exposure	Outcome	nSNP	MR Egger		Weighted median		Inverse variance weighted		MR_PRESSO	
			OR (95% CI)	pval	OR (95% CI)	pval	OR (95% CI)	pval	OR (95% CI)	pval
Branched-chain, straight-chain, or cyclopropyl 12:1 fatty acid	Hypertension	15	1.000 (0.985 to 1.015)	0.988	1.012 (1.004 to 1.020)	0.003	1.006 (0.998 to 1.015)	0.152	1.011 (1.003 to 1.018)	0.014
Deoxycholic acid 12-sulfate	Hypertension	13	0.997 (0.984 to 1.009)	0.607	0.998 (0.994 to 1.003)	0.521	0.998 (0.992 to 1.004)	0.522	0.999 (0.995 to 1.004)	0.823
Branched-chain, straight-chain, or cyclopropyl 10:1 fatty acid (1)	Hypertension	6	0.986 (0.947 to 1.026)	0.521	0.995 (0.983 to 1.008)	0.441	0.996 (0.981 to 1.010)	0.544		
3,5-dichloro-2,6-dihydroxybenzoic acid	Hypertension	11	0.997 (0.989 to 1.006)	0.570	0.998 (0.992 to 1.004)	0.455	0.997 (0.992 to 1.002)	0.279		
Vanillic acid glycine	Hypertension	13	1.026 (0.999 to 1.053)	0.083	0.994 (0.985 to 1.002)	0.154	0.989 (0.978 to 0.999)	0.036	0.989 (0.982 to 0.996)	0.014
(S)-a-amino-omega-caprolactam	Hypertension	8	1.004 (0.914 to 1.104)	0.931	1.004 (0.993 to 1.016)	0.457	1.016 (0.981 to 1.053)	0.362	1.007 (1.002 to 1.011)	0.043

Exposure	Outcome	nSNP	MR Egger		Weighted median		Inverse variance weighted		MR_PRESSO	
			OR (95% CI)	pval	OR (95% CI)	pval	OR (95% CI)	pval	OR (95% CI)	pval
3-bromo-5-chloro-2,6-dihydroxybenzoic acid	Hypertension	11	0.994 (0.985 to 1.003)	0.213	0.999 (0.992 to 1.005)	0.687	0.999 (0.994 to 1.004)	0.726		
Dibutyl sulfosuccinate	Hypertension	19	1.000 (0.997 to 1.002)	0.910	1.000 (0.998 to 1.002)	0.999	1.000 (0.998 to 1.002)	0.938		
Metabolonic lactone sulfate	Hypertension	16	1.005 (0.999 to 1.012)	0.116	1.003 (1.000 to 1.007)	0.037	1.002 (0.998 to 1.006)	0.389	1.002 (0.999 to 1.005)	0.273
2-hydroxy-4-(methylthio)butanoic acid	Hypertension	15	1.003 (0.994 to 1.012)	0.501	1.005 (1.001 to 1.008)	0.011	1.004 (0.999 to 1.010)	0.109	1.005 (1.002 to 1.008)	0.015
Pentose acid	Hypertension	14	1.002 (0.988 to 1.017)	0.788	1.005 (0.997 to 1.014)	0.228	1.008 (1.002 to 1.014)	0.010		
Branched chain 14:0 dicarboxylic acid	Hypertension	8	1.020 (1.002 to 1.039)	0.072	1.002 (0.992 to 1.013)	0.646	1.001 (0.992 to 1.011)	0.824		

Exposure	Outcome	nSNP	MR Egger		Weighted median		Inverse variance weighted		MR_PRESSO	
			OR (95% CI)	pval	OR (95% CI)	pval	OR (95% CI)	pval	OR (95% CI)	pval
S-carboxyethylcysteine	Hypertension	13	0.990 (0.973 to 1.007)	0.252	0.998 (0.990 to 1.007)	0.710	0.998 (0.991 to 1.005)	0.608		
N-succinyl-phenylalanine	Hypertension	11	0.988 (0.975 to 1.000)	0.091	0.993 (0.988 to 0.997)	0.001	0.996 (0.990 to 1.002)	0.175	0.999 (0.990 to 1.007)	0.772
(2 or 3)-decenoate (10:1n7 or n8)	Hypertension	18	1.002 (0.990 to 1.013)	0.789	1.006 (0.999 to 1.013)	0.079	1.006 (1.000 to 1.011)	0.036		
Bilirubin degradation product, C17H20N2O5 (2)	Hypertension	13	0.998 (0.990 to 1.005)	0.561	0.998 (0.993 to 1.002)	0.245	0.997 (0.992 to 1.001)	0.117		
Bilirubin degradation product, C16H18N2O5 (4)	Hypertension	8	0.999 (0.991 to 1.007)	0.777	0.999 (0.996 to 1.003)	0.640	1.000 (0.994 to 1.005)	0.906		
Cis-3,4-methyleneheptanoylcarnitine	Hypertension	18	0.999 (0.977 to 1.022)	0.928	1.002 (0.995 to 1.009)	0.519	1.007 (0.997 to 1.017)	0.155	1.005 (1.000 to 1.010)	0.060

Exposure	Outcome	nSNP	MR Egger		Weighted median		Inverse variance weighted		MR_PRESSO	
			OR (95% CI)	pval	OR (95% CI)	pval	OR (95% CI)	pval	OR (95% CI)	pval
Tetrahydrocortisol glucuronide	Hypertension	19	0.996 (0.985 to 1.008)	0.530	0.998 (0.991 to 1.004)	0.466	0.998 (0.993 to 1.003)	0.467		
N-acetyl-2-aminoadipate	Hypertension	16	1.010 (1.001 to 1.018)	0.045	1.006 (1.001 to 1.010)	0.018	1.003 (0.998 to 1.009)	0.229	1.005 (1.000 to 1.009)	0.051
Bilirubin degradation product, C17H20N2O5 (1)	Hypertension	14	1.001 (0.994 to 1.007)	0.841	0.998 (0.993 to 1.002)	0.299	0.997 (0.994 to 1.001)	0.177		
2-methoxyhydroquinone sulfate (1)	Hypertension	9	1.016 (0.988 to 1.045)	0.307	1.011 (1.000 to 1.021)	0.054	1.010 (1.000 to 1.020)	0.049		
Bilirubin degradation product, C16H18N2O5 (3)	Hypertension	15	0.999 (0.993 to 1.006)	0.834	0.999 (0.996 to 1.003)	0.660	0.998 (0.994 to 1.002)	0.423		
3-hydroxyoctanoylcarnitine (1)	Hypertension	12	0.990 (0.963 to 1.018)	0.494	0.994 (0.984 to 1.005)	0.280	1.000 (0.989 to 1.010)	0.936	0.997 (0.988 to 1.006)	0.474

Exposure	Outcome	nSNP	MR Egger		Weighted median		Inverse variance weighted		MR_PRESSO	
			OR (95% CI)	pval	OR (95% CI)	pval	OR (95% CI)	pval	OR (95% CI)	pval
2-methoxyhydroquinone sulfate (2)	Hypertension	8	1.003 (0.986 to 1.021)	0.754	1.000 (0.991 to 1.008)	0.914	0.997 (0.989 to 1.004)	0.349		
Cis-3,4-methyleneheptanoylglycine	Hypertension	12	0.981 (0.973 to 0.989)	9.37e-04	0.987 (0.982 to 0.991)	3.54e-08	0.991 (0.985 to 0.996)	9.24e-04		
Bilirubin degradation product, C16H18N2O5 (2)	Hypertension	13	0.998 (0.990 to 1.005)	0.584	0.998 (0.994 to 1.001)	0.231	0.998 (0.993 to 1.003)	0.499		
2,4-di-tert-butylphenol	Hypertension	13	1.001 (0.987 to 1.014)	0.917	1.003 (0.995 to 1.011)	0.437	1.002 (0.996 to 1.008)	0.461		
Bilirubin degradation product, C16H18N2O5 (1)	Hypertension	11	1.002 (0.995 to 1.009)	0.664	0.999 (0.996 to 1.002)	0.588	0.999 (0.994 to 1.003)	0.574		
3-hydroxyoctanoylcarnitine (2)	Hypertension	13	0.997 (0.970 to 1.025)	0.860	1.001 (0.992 to 1.010)	0.799	1.001 (0.993 to 1.010)	0.746	0.999 (0.992 to 1.005)	0.710

Exposure	Outcome	nSNP	MR Egger		Weighted median		Inverse variance weighted		MR_PRESSO	
			OR (95% CI)	pval	OR (95% CI)	pval	OR (95% CI)	pval	OR (95% CI)	pval
Pantothenate	Hypertension	16	0.998 (0.979 to 1.017)	0.804	1.000 (0.992 to 1.009)	0.962	1.007 (0.999 to 1.015)	0.099		
5-oxoproline	Hypertension	11	1.007 (1.001 to 1.014)	0.044	1.005 (1.002 to 1.009)	0.003	1.004 (0.999 to 1.008)	0.134		
Phosphoethanolamine	Hypertension	15	1.004 (0.979 to 1.030)	0.769	1.005 (0.997 to 1.014)	0.195	1.004 (0.995 to 1.013)	0.342	1.005 (0.998 to 1.012)	0.216
Picolinate	Hypertension	16	0.994 (0.966 to 1.022)	0.665	1.003 (0.995 to 1.010)	0.443	0.995 (0.981 to 1.009)	0.448	0.999 (0.993 to 1.005)	0.834
3-ureidopropionate	Hypertension	10	1.000 (0.983 to 1.017)	0.980	1.000 (0.991 to 1.009)	0.997	1.001 (0.994 to 1.008)	0.716		
Erucate (22:1n9)	Hypertension	10	0.999 (0.980 to 1.019)	0.942	1.001 (0.991 to 1.011)	0.847	1.001 (0.992 to 1.010)	0.817		

Exposure	Outcome	nSNP	MR Egger		Weighted median		Inverse variance weighted		MR_PRESSO	
			OR (95% CI)	pval	OR (95% CI)	pval	OR (95% CI)	pval	OR (95% CI)	pval
Eicosenedioate (C20:1-DC)	Hypertension	10	0.988 (0.973 to 1.004)	0.183	1.000 (0.990 to 1.011)	0.955	1.003 (0.995 to 1.011)	0.497		
Allantoin	Hypertension	13	0.985 (0.971 to 0.999)	0.057	1.006 (0.996 to 1.015)	0.231	1.002 (0.995 to 1.010)	0.559		
N-acetylvaline	Hypertension	13	0.989 (0.947 to 1.033)	0.625	0.996 (0.987 to 1.005)	0.344	1.003 (0.981 to 1.026)	0.776	0.995 (0.989 to 1.001)	0.141
N-acetylleucine	Hypertension	16	1.004 (0.999 to 1.008)	0.131	1.003 (1.000 to 1.007)	0.062	1.002 (0.999 to 1.005)	0.183		
N-acetylmethionine	Hypertension	10	0.993 (0.973 to 1.013)	0.491	0.998 (0.988 to 1.007)	0.597	1.005 (0.994 to 1.017)	0.380	0.998 (0.991 to 1.006)	0.680
Glycerate	Hypertension	17	1.005 (0.994 to 1.017)	0.404	0.998 (0.991 to 1.005)	0.590	0.999 (0.994 to 1.004)	0.742		

Exposure	Outcome	nSNP	MR Egger		Weighted median		Inverse variance weighted		MR_PRESSO	
			OR (95% CI)	pval	OR (95% CI)	pval	OR (95% CI)	pval	OR (95% CI)	pval
Sarcosine	Hypertension	15	1.001 (0.979 to 1.022)	0.960	0.996 (0.987 to 1.005)	0.386	1.003 (0.994 to 1.012)	0.500		
Trans-4-hydroxyproline	Hypertension	10	0.995 (0.980 to 1.010)	0.524	1.004 (0.995 to 1.013)	0.349	1.005 (0.996 to 1.013)	0.284		
Thyroxine	Hypertension	13	0.999 (0.971 to 1.027)	0.919	1.002 (0.993 to 1.011)	0.614	1.013 (1.001 to 1.025)	0.041	1.001 (0.997 to 1.006)	0.549
Pipecolate	Hypertension	14	0.998 (0.985 to 1.012)	0.788	1.001 (0.993 to 1.009)	0.816	1.000 (0.994 to 1.006)	0.952		
Gamma-glutamyltyrosine	Hypertension	12	1.007 (0.988 to 1.027)	0.485	1.000 (0.992 to 1.008)	0.941	0.997 (0.989 to 1.006)	0.503	0.999 (0.993 to 1.006)	0.883
Methyl indole-3-acetate	Hypertension	8	1.014 (0.997 to 1.031)	0.150	1.003 (0.993 to 1.013)	0.607	1.003 (0.996 to 1.011)	0.404		

Exposure	Outcome	nSNP	MR Egger		Weighted median		Inverse variance weighted		MR_PRESSO	
			OR (95% CI)	pval	OR (95% CI)	pval	OR (95% CI)	pval	OR (95% CI)	pval
Ursodeoxycholate	Hypertension	13	1.008 (0.976 to 1.041)	0.640	0.999 (0.991 to 1.007)	0.774	1.001 (0.990 to 1.012)	0.924	0.998 (0.991 to 1.005)	0.568
3-hydroxy-3-methylglutarate	Hypertension	12	0.999 (0.972 to 1.027)	0.963	0.994 (0.983 to 1.004)	0.243	0.991 (0.981 to 1.001)	0.070		
Oleoyl ethanolamide	Hypertension	13	1.004 (0.995 to 1.014)	0.364	1.003 (0.996 to 1.009)	0.474	1.003 (0.998 to 1.008)	0.216		
4-hydroxyphenylpyruvate	Hypertension	9	1.008 (0.986 to 1.030)	0.505	0.998 (0.988 to 1.008)	0.710	0.999 (0.989 to 1.009)	0.881		
Gamma-glutamylglutamine	Hypertension	21	0.998 (0.980 to 1.015)	0.792	0.994 (0.988 to 1.000)	0.068	0.996 (0.990 to 1.003)	0.239	0.995 (0.989 to 1.000)	0.073
Alpha-tocopherol	Hypertension	17	1.007 (0.985 to 1.029)	0.567	1.001 (0.992 to 1.009)	0.875	1.005 (0.997 to 1.014)	0.216	1.007 (1.000 to 1.015)	0.079

Exposure	Outcome	nSNP	MR Egger		Weighted median		Inverse variance weighted		MR_PRESSO	
			OR (95% CI)	pval	OR (95% CI)	pval	OR (95% CI)	pval	OR (95% CI)	pval
N-acetyl-L-alanine	Hypertension	17	0.986 (0.938 to 1.037)	0.603	0.990 (0.982 to 0.999)	0.021	0.997 (0.977 to 1.017)	0.741	0.988 (0.983 to 0.993)	6.44e-04
Citrate	Hypertension	14	1.007 (0.988 to 1.025)	0.498	1.002 (0.994 to 1.009)	0.670	1.004 (0.996 to 1.011)	0.361	1.005 (0.998 to 1.012)	0.169
2-aminobutyrate	Hypertension	9	1.000 (0.985 to 1.016)	0.999	1.002 (0.994 to 1.010)	0.568	1.004 (0.998 to 1.010)	0.170		
Butyrate/isobutyrate (4:0)	Hypertension	11	0.993 (0.960 to 1.026)	0.685	0.997 (0.989 to 1.006)	0.570	0.995 (0.987 to 1.003)	0.211		
Urate	Hypertension	20	0.997 (0.987 to 1.007)	0.592	1.006 (1.000 to 1.012)	0.056	1.004 (0.998 to 1.009)	0.186	1.002 (0.998 to 1.007)	0.317
3-hydroxyisobutyrate	Hypertension	12	0.986 (0.960 to 1.012)	0.301	0.993 (0.985 to 1.001)	0.103	0.997 (0.987 to 1.007)	0.581	0.993 (0.988 to 0.997)	0.011

Exposure	Outcome	nSNP	MR Egger		Weighted median		Inverse variance weighted		MR_PRESSO	
			OR (95% CI)	pval	OR (95% CI)	pval	OR (95% CI)	pval	OR (95% CI)	pval
Vanillylmandelate (VMA)	Hypertension	22	1.000 (0.965 to 1.037)	0.990	1.001 (0.994 to 1.009)	0.699	1.013 (0.998 to 1.029)	0.091	1.004 (0.998 to 1.011)	0.198
3-aminoisobutyrate	Hypertension	14	1.000 (0.997 to 1.004)	0.918	1.001 (0.998 to 1.004)	0.431	1.001 (0.999 to 1.004)	0.377		
4-acetamidobutanoate	Hypertension	11	1.010 (1.000 to 1.021)	0.085	1.009 (1.003 to 1.015)	0.002	1.004 (0.998 to 1.011)	0.175		
2-hydroxystearate	Hypertension	15	1.010 (0.981 to 1.040)	0.504	1.003 (0.994 to 1.012)	0.545	1.004 (0.994 to 1.013)	0.418	1.006 (0.998 to 1.015)	0.157
N1-methyladenosine	Hypertension	20	0.996 (0.956 to 1.038)	0.855	1.007 (1.000 to 1.015)	0.066	1.011 (0.996 to 1.026)	0.158	1.004 (0.997 to 1.011)	0.251
N-acetylneuraminate	Hypertension	19	0.997 (0.969 to 1.025)	0.817	1.000 (0.993 to 1.007)	0.996	1.012 (0.997 to 1.027)	0.122	1.001 (0.998 to 1.005)	0.530

Exposure	Outcome	nSNP	MR Egger		Weighted median		Inverse variance weighted		MR_PRESSO	
			OR (95% CI)	pval	OR (95% CI)	pval	OR (95% CI)	pval	OR (95% CI)	pval
Gamma-glutamylhistidine	Hypertension	11	0.994 (0.965 to 1.023)	0.670	1.006 (0.996 to 1.015)	0.268	1.013 (0.998 to 1.027)	0.084	1.004 (0.999 to 1.009)	0.156
N-acetylglucosaminylasparagine	Hypertension	16	1.003 (0.995 to 1.012)	0.467	0.999 (0.995 to 1.004)	0.703	0.998 (0.992 to 1.003)	0.451	0.999 (0.994 to 1.004)	0.620
Homovanillate (hva)	Hypertension	15	0.991 (0.964 to 1.018)	0.520	1.003 (0.996 to 1.010)	0.407	0.997 (0.985 to 1.009)	0.633	1.002 (0.996 to 1.008)	0.546
Acetoacetate	Hypertension	11	1.009 (0.993 to 1.024)	0.303	1.001 (0.993 to 1.009)	0.829	1.002 (0.997 to 1.008)	0.418		
Cys-gly, oxidized	Hypertension	16	1.017 (1.010 to 1.024)	2.66e-04	1.015 (1.010 to 1.019)	1.34e-09	1.009 (1.004 to 1.013)	1.8e-04	1.003 (0.997 to 1.009)	0.304
Dihomo-linoleate (20:2n6)	Hypertension	10	1.022 (0.977 to 1.070)	0.372	1.001 (0.989 to 1.012)	0.919	0.997 (0.976 to 1.018)	0.747	0.998 (0.989 to 1.007)	0.653

Exposure	Outcome	nSNP	MR Egger		Weighted median		Inverse variance weighted		MR_PRESSO	
			OR (95% CI)	pval	OR (95% CI)	pval	OR (95% CI)	pval	OR (95% CI)	pval
Cholate	Hypertension	11	1.015 (0.993 to 1.037)	0.218	0.997 (0.987 to 1.007)	0.524	0.996 (0.988 to 1.005)	0.436		
Beta-hydroxyisovalerate	Hypertension	17	1.001 (0.988 to 1.013)	0.903	1.005 (0.999 to 1.011)	0.111	1.005 (0.999 to 1.010)	0.097		
Creatine	Hypertension	15	0.978 (0.953 to 1.004)	0.127	0.993 (0.983 to 1.002)	0.126	0.989 (0.979 to 1.000)	0.043	0.994 (0.986 to 1.001)	0.130
Anthranilate	Hypertension	12	1.005 (0.992 to 1.019)	0.444	1.003 (0.996 to 1.010)	0.420	1.003 (0.997 to 1.008)	0.350		
Glycerol	Hypertension	10	1.023 (0.985 to 1.061)	0.272	1.004 (0.993 to 1.016)	0.462	1.002 (0.991 to 1.013)	0.685		
Gamma-glutamylleucine	Hypertension	4	0.987 (0.888 to 1.096)	0.826	1.000 (0.985 to 1.014)	0.975	0.996 (0.978 to 1.015)	0.705		

Exposure	Outcome	nSNP	MR Egger		Weighted median		Inverse variance weighted		MR_PRESSO	
			OR (95% CI)	pval	OR (95% CI)	pval	OR (95% CI)	pval	OR (95% CI)	pval
Choline	Hypertension	14	0.997 (0.973 to 1.022)	0.832	1.003 (0.994 to 1.013)	0.474	1.010 (1.000 to 1.021)	0.055	1.006 (0.999 to 1.013)	0.140
3-methoxytyrosine	Hypertension	16	1.010 (0.993 to 1.026)	0.269	1.006 (0.999 to 1.013)	0.096	1.002 (0.995 to 1.009)	0.590	1.006 (1.000 to 1.011)	0.054
3-phosphoglycerate	Hypertension	13	0.982 (0.963 to 1.001)	0.083	0.994 (0.985 to 1.003)	0.201	0.999 (0.990 to 1.008)	0.795	0.996 (0.988 to 1.005)	0.425
1-palmitoyl-2-linoleoyl-gpc (16:0/18:2)	Hypertension	15	1.001 (0.990 to 1.012)	0.879	1.001 (0.995 to 1.007)	0.757	1.001 (0.996 to 1.006)	0.668		
1-palmitoyl-2-oleoyl-GPE (16:0/18:1)	Hypertension	21	1.003 (0.992 to 1.014)	0.654	1.002 (0.997 to 1.006)	0.517	1.003 (0.998 to 1.008)	0.241	1.002 (0.997 to 1.007)	0.429
1-palmitoyl-2-linoleoyl-GPI (16:0/18:2)	Hypertension	17	0.992 (0.967 to 1.017)	0.538	1.001 (0.995 to 1.008)	0.632	0.996 (0.987 to 1.005)	0.414	0.998 (0.993 to 1.004)	0.571

Exposure	Outcome	nSNP	MR Egger		Weighted median		Inverse variance weighted		MR_PRESSO	
			OR (95% CI)	pval	OR (95% CI)	pval	OR (95% CI)	pval	OR (95% CI)	pval
Taurocholic acid	Hypertension	15	0.992 (0.975 to 1.010)	0.410	1.003 (0.995 to 1.011)	0.476	1.005 (0.998 to 1.012)	0.172		
Taurodeoxycholate	Hypertension	9	0.996 (0.974 to 1.018)	0.727	1.000 (0.990 to 1.010)	0.926	1.004 (0.996 to 1.012)	0.289		
Stearoyl sphingomyelin (d18:1/18:0)	Hypertension	22	1.005 (0.982 to 1.028)	0.700	0.998 (0.991 to 1.006)	0.631	0.997 (0.987 to 1.007)	0.592	1.000 (0.993 to 1.007)	0.945
Ceramide (d18:1/16:0)	Hypertension	17	1.007 (0.992 to 1.023)	0.375	0.997 (0.989 to 1.005)	0.442	0.999 (0.992 to 1.007)	0.888	0.999 (0.994 to 1.003)	0.575
Taurochenodeoxycholate	Hypertension	11	1.021 (0.989 to 1.055)	0.237	1.004 (0.994 to 1.014)	0.460	1.002 (0.990 to 1.014)	0.756	1.006 (0.997 to 1.016)	0.210
4-hydroxyphenylacetate	Hypertension	12	1.003 (0.989 to 1.016)	0.700	1.000 (0.992 to 1.009)	0.956	1.001 (0.995 to 1.008)	0.664		

Exposure	Outcome	nSNP	MR Egger		Weighted median		Inverse variance weighted		MR_PRESSO	
			OR (95% CI)	pval	OR (95% CI)	pval	OR (95% CI)	pval	OR (95% CI)	pval
5,6-dihydrothymine	Hypertension	9	0.988 (0.948 to 1.029)	0.581	1.004 (0.991 to 1.016)	0.566	0.998 (0.983 to 1.014)	0.837	1.003 (0.996 to 1.011)	0.424
1-palmitoyl-2-oleoyl-gpc (16:0/18:1)	Hypertension	15	0.990 (0.974 to 1.007)	0.284	0.999 (0.991 to 1.008)	0.861	1.000 (0.993 to 1.007)	0.951		
N-stearoyl-sphingosine (d18:1/18:0)	Hypertension	15	0.989 (0.960 to 1.019)	0.477	0.997 (0.990 to 1.004)	0.464	0.997 (0.989 to 1.006)	0.544	0.998 (0.991 to 1.005)	0.546
Glycochenodeoxycholate	Hypertension	14	0.979 (0.954 to 1.006)	0.148	0.994 (0.985 to 1.003)	0.199	0.994 (0.984 to 1.004)	0.261	0.993 (0.986 to 1.001)	0.111
N-formylmethionine	Hypertension	14	0.997 (0.958 to 1.038)	0.885	1.012 (1.004 to 1.020)	0.002	1.014 (0.997 to 1.032)	0.113	1.012 (1.005 to 1.019)	0.007
N6,n6,n6-trimethyllysine	Hypertension	10	1.004 (0.994 to 1.013)	0.450	1.004 (0.997 to 1.011)	0.298	1.002 (0.996 to 1.007)	0.557		

Exposure	Outcome	nSNP	MR Egger		Weighted median		Inverse variance weighted		MR_PRESSO	
			OR (95% CI)	pval	OR (95% CI)	pval	OR (95% CI)	pval	OR (95% CI)	pval
S-adenosylhomocysteine (SAH)	Hypertension	13	0.999 (0.949 to 1.051)	0.961	1.005 (0.997 to 1.012)	0.203	1.011 (0.989 to 1.033)	0.339	1.007 (1.000 to 1.014)	0.071
4-acetaminophen sulfate	Hypertension	12	0.996 (0.983 to 1.009)	0.539	1.000 (0.993 to 1.006)	0.912	0.999 (0.994 to 1.004)	0.683		
2-hydroxyhippurate (salicylurate)	Hypertension	10	1.006 (0.989 to 1.024)	0.515	1.000 (0.990 to 1.009)	0.942	1.000 (0.993 to 1.008)	0.914		
Quinolinate	Hypertension	13	0.996 (0.935 to 1.059)	0.890	1.000 (0.991 to 1.008)	0.910	1.013 (0.992 to 1.034)	0.228	1.002 (0.996 to 1.009)	0.460
Eicosapentaenoate (EPA; 20:5n3)	Hypertension	12	0.993 (0.979 to 1.008)	0.409	1.005 (0.997 to 1.013)	0.210	1.004 (0.997 to 1.012)	0.276		
Laurate (12:0)	Hypertension	9	0.985 (0.968 to 1.001)	0.112	0.987 (0.977 to 0.998)	0.015	0.992 (0.985 to 1.000)	0.053		

Exposure	Outcome	nSNP	MR Egger		Weighted median		Inverse variance weighted		MR_PRESSO	
			OR (95% CI)	pval	OR (95% CI)	pval	OR (95% CI)	pval	OR (95% CI)	pval
N-acetylputrescine	Hypertension	17	1.006 (1.000 to 1.012)	0.072	1.005 (1.002 to 1.009)	0.002	1.004 (1.000 to 1.008)	0.036		
Methylsuccinate	Hypertension	13	0.996 (0.986 to 1.007)	0.522	0.997 (0.993 to 1.002)	0.227	0.998 (0.992 to 1.004)	0.579	0.997 (0.992 to 1.001)	0.173
9,10-DiHOME	Hypertension	12	0.988 (0.974 to 1.003)	0.143	1.003 (0.995 to 1.011)	0.501	1.006 (0.998 to 1.014)	0.130		
Linoleate (18:2n6)	Hypertension	12	1.013 (0.967 to 1.060)	0.606	1.000 (0.990 to 1.010)	0.972	0.996 (0.979 to 1.014)	0.684	1.001 (0.996 to 1.006)	0.707
Adenosine 5'-diphosphate (ADP)	Hypertension	15	1.000 (0.986 to 1.014)	0.980	1.007 (1.001 to 1.014)	0.022	1.006 (1.002 to 1.011)	0.008		
Adenosine 5'-monophosphate (AMP)	Hypertension	8	1.001 (0.981 to 1.020)	0.948	1.005 (0.995 to 1.015)	0.323	1.004 (0.996 to 1.012)	0.367		

Exposure	Outcome	nSNP	MR Egger		Weighted median		Inverse variance weighted		MR_PRESSO	
			OR (95% CI)	pval	OR (95% CI)	pval	OR (95% CI)	pval	OR (95% CI)	pval
Adenosine 3',5'-cyclic monophosphate (camp)	Hypertension	17	1.002 (0.988 to 1.016)	0.786	1.002 (0.996 to 1.009)	0.467	1.002 (0.997 to 1.007)	0.419		
Arachidonate (20:4n6)	Hypertension	14	1.000 (0.989 to 1.011)	0.995	0.998 (0.993 to 1.004)	0.513	1.000 (0.994 to 1.006)	0.994		
5-methylthioadenosine (mta)	Hypertension	14	1.006 (0.980 to 1.032)	0.657	0.999 (0.992 to 1.007)	0.880	1.006 (0.996 to 1.016)	0.223	1.000 (0.996 to 1.005)	0.870
Cortisone	Hypertension	8	0.995 (0.980 to 1.011)	0.554	0.997 (0.987 to 1.007)	0.609	0.998 (0.990 to 1.006)	0.579		
Beta-alanine	Hypertension	10	0.996 (0.978 to 1.014)	0.639	1.000 (0.992 to 1.007)	0.898	1.002 (0.994 to 1.011)	0.618	1.000 (0.994 to 1.005)	0.864
Succinate	Hypertension	9	0.979 (0.964 to 0.995)	0.035	0.988 (0.977 to 0.999)	0.033	0.988 (0.979 to 0.997)	0.007		

Exposure	Outcome	nSNP	MR Egger		Weighted median		Inverse variance weighted		MR_PRESSO	
			OR (95% CI)	pval	OR (95% CI)	pval	OR (95% CI)	pval	OR (95% CI)	pval
Phosphocholine	Hypertension	17	1.007 (0.995 to 1.020)	0.271	1.004 (0.997 to 1.011)	0.245	1.002 (0.996 to 1.007)	0.553		
Cysteinylglycine	Hypertension	12	1.029 (1.010 to 1.050)	0.015	1.010 (1.001 to 1.020)	0.034	1.009 (0.999 to 1.019)	0.070	1.000 (0.993 to 1.007)	0.988
Phenylpyruvate	Hypertension	17	0.999 (0.983 to 1.015)	0.857	1.003 (0.996 to 1.010)	0.370	1.005 (1.000 to 1.010)	0.073		
Creatinine	Hypertension	10	1.001 (0.916 to 1.093)	0.991	1.001 (0.988 to 1.014)	0.862	1.018 (0.985 to 1.051)	0.288	1.001 (0.993 to 1.009)	0.780
3-Hydroxybutyrate	Hypertension	9	0.998 (0.971 to 1.024)	0.860	1.004 (0.994 to 1.015)	0.421	1.002 (0.993 to 1.011)	0.698		
Cholesterol	Hypertension	13	0.984 (0.972 to 0.997)	0.029	0.998 (0.990 to 1.006)	0.624	0.997 (0.991 to 1.003)	0.366		

Exposure	Outcome	nSNP	MR Egger		Weighted median		Inverse variance weighted		MR_PRESSO	
			OR (95% CI)	pval	OR (95% CI)	pval	OR (95% CI)	pval	OR (95% CI)	pval
2-hydroxyphenylacetate	Hypertension	12	1.008 (0.995 to 1.021)	0.261	1.000 (0.993 to 1.008)	0.910	1.000 (0.995 to 1.006)	0.928		
Aspartate	Hypertension	12	1.014 (0.995 to 1.032)	0.184	1.013 (1.005 to 1.021)	0.001	1.005 (0.998 to 1.012)	0.149		
3-(4-hydroxyphenyl)lactate	Hypertension	12	1.026 (0.990 to 1.064)	0.193	0.996 (0.988 to 1.004)	0.336	0.990 (0.976 to 1.004)	0.156	0.995 (0.990 to 1.001)	0.136
Arginine	Hypertension	19	0.977 (0.953 to 1.000)	0.070	1.004 (0.997 to 1.011)	0.315	1.005 (0.996 to 1.014)	0.288	1.004 (0.999 to 1.010)	0.121
Gluconate	Hypertension	11	1.002 (0.992 to 1.012)	0.743	1.002 (0.994 to 1.009)	0.679	1.002 (0.996 to 1.007)	0.583		
Sphinganine	Hypertension	13	1.002 (0.975 to 1.030)	0.886	1.004 (0.995 to 1.013)	0.381	1.001 (0.992 to 1.010)	0.875	1.000 (0.991 to 1.008)	0.957

Exposure	Outcome	nSNP	MR Egger		Weighted median		Inverse variance weighted		MR_PRESSO	
			OR (95% CI)	pval	OR (95% CI)	pval	OR (95% CI)	pval	OR (95% CI)	pval
Gamma-glutamylglutamate	Hypertension	17	0.992 (0.965 to 1.019)	0.558	0.999 (0.990 to 1.007)	0.750	0.996 (0.986 to 1.007)	0.460	0.999 (0.992 to 1.006)	0.770
Flavin adenine dinucleotide (FAD)	Hypertension	19	1.011 (1.003 to 1.019)	0.018	1.005 (1.001 to 1.009)	0.017	1.002 (0.997 to 1.008)	0.418	1.004 (1.001 to 1.008)	0.032
Histidine	Hypertension	13	0.983 (0.946 to 1.021)	0.388	0.994 (0.985 to 1.004)	0.276	1.003 (0.990 to 1.016)	0.646	1.000 (0.992 to 1.008)	0.988
Cortisol	Hypertension	12	1.002 (0.983 to 1.022)	0.819	1.002 (0.993 to 1.011)	0.695	1.005 (0.997 to 1.012)	0.228		
Hypotaurine	Hypertension	23	1.018 (0.975 to 1.062)	0.427	0.997 (0.989 to 1.004)	0.399	1.004 (0.993 to 1.015)	0.499	1.001 (0.993 to 1.008)	0.852
Glutarate (C5-DC)	Hypertension	14	1.007 (0.993 to 1.021)	0.364	1.000 (0.992 to 1.008)	0.948	1.002 (0.996 to 1.008)	0.556		

Exposure	Outcome	nSNP	MR Egger		Weighted median		Inverse variance weighted		MR_PRESSO	
			OR (95% CI)	pval	OR (95% CI)	pval	OR (95% CI)	pval	OR (95% CI)	pval
S-1-pyrroline-5-carboxylate	Hypertension	9	1.002 (0.984 to 1.020)	0.856	1.004 (0.995 to 1.013)	0.404	0.998 (0.989 to 1.007)	0.700		
Cystine	Hypertension	10	1.001 (0.986 to 1.016)	0.942	1.004 (0.994 to 1.013)	0.468	1.003 (0.996 to 1.010)	0.417		
Cystathionine	Hypertension	16	1.014 (0.997 to 1.031)	0.135	1.003 (0.995 to 1.012)	0.426	1.002 (0.995 to 1.009)	0.623		
Guanidinoacetate	Hypertension	18	0.991 (0.966 to 1.016)	0.485	0.995 (0.989 to 1.002)	0.154	1.005 (0.993 to 1.017)	0.430	1.000 (0.995 to 1.006)	0.962
Sphingosine	Hypertension	7	1.031 (0.994 to 1.069)	0.166	1.005 (0.994 to 1.017)	0.373	1.004 (0.991 to 1.016)	0.562	1.002 (0.989 to 1.015)	0.773
Deoxycholate	Hypertension	15	0.975 (0.945 to 1.006)	0.141	0.996 (0.987 to 1.005)	0.439	1.001 (0.990 to 1.012)	0.856	0.997 (0.991 to 1.003)	0.308

Exposure	Outcome	nSNP	MR Egger		Weighted median		Inverse variance weighted		MR_PRESSO	
			OR (95% CI)	pval	OR (95% CI)	pval	OR (95% CI)	pval	OR (95% CI)	pval
Glycocholate	Hypertension	22	1.014 (0.998 to 1.030)	0.103	1.002 (0.995 to 1.009)	0.641	1.001 (0.994 to 1.008)	0.816	1.003 (0.997 to 1.009)	0.336
Palmitoleate (16:1n7)	Hypertension	14	1.010 (0.960 to 1.062)	0.706	1.006 (0.997 to 1.016)	0.194	0.993 (0.978 to 1.009)	0.408	1.007 (1.002 to 1.013)	0.027
Leucine	Hypertension	14	0.994 (0.972 to 1.017)	0.628	1.011 (1.002 to 1.020)	0.022	1.010 (1.001 to 1.020)	0.027	1.014 (1.007 to 1.021)	0.002
Stearate (18:0)	Hypertension	8	1.065 (0.981 to 1.156)	0.183	1.015 (1.001 to 1.029)	0.030	1.008 (0.977 to 1.039)	0.626	1.009 (0.999 to 1.020)	0.149
Methionine	Hypertension	12	1.002 (0.987 to 1.018)	0.811	0.995 (0.986 to 1.004)	0.252	0.994 (0.988 to 1.000)	0.067		
Orotate	Hypertension	19	0.999 (0.995 to 1.003)	0.728	1.000 (0.997 to 1.003)	0.853	1.000 (0.997 to 1.003)	0.980		

Exposure	Outcome	nSNP	MR Egger		Weighted median		Inverse variance weighted		MR_PRESSO	
			OR (95% CI)	pval	OR (95% CI)	pval	OR (95% CI)	pval	OR (95% CI)	pval
Phosphate	Hypertension	14	1.006 (0.984 to 1.028)	0.630	0.994 (0.987 to 1.002)	0.166	0.995 (0.986 to 1.004)	0.248	0.995 (0.989 to 1.002)	0.177
Inosine	Hypertension	10	0.996 (0.985 to 1.007)	0.492	1.004 (0.997 to 1.012)	0.271	1.005 (0.999 to 1.010)	0.103		
Inosine 5'-monophosphate (IMP)	Hypertension	13	0.990 (0.978 to 1.002)	0.118	1.001 (0.995 to 1.007)	0.741	1.001 (0.996 to 1.006)	0.643		
Phenylalanine	Hypertension	13	1.010 (0.998 to 1.023)	0.141	1.006 (0.998 to 1.014)	0.154	1.004 (0.998 to 1.009)	0.239		
Palmitate (16:0)	Hypertension	12	1.031 (1.000 to 1.064)	0.079	1.008 (0.998 to 1.017)	0.106	1.001 (0.986 to 1.017)	0.870	1.006 (0.999 to 1.012)	0.112
Malate	Hypertension	8	0.999 (0.979 to 1.020)	0.947	1.001 (0.993 to 1.010)	0.751	1.003 (0.996 to 1.010)	0.446		

Exposure	Outcome	nSNP	MR Egger		Weighted median		Inverse variance weighted		MR_PRESSO	
			OR (95% CI)	pval	OR (95% CI)	pval	OR (95% CI)	pval	OR (95% CI)	pval
Isoleucine	Hypertension	11	1.014 (0.984 to 1.044)	0.392	1.005 (0.996 to 1.015)	0.258	1.005 (0.996 to 1.013)	0.265		
Nicotinamide	Hypertension	12	1.001 (0.975 to 1.029)	0.918	1.004 (0.994 to 1.013)	0.456	0.996 (0.986 to 1.006)	0.391	0.999 (0.990 to 1.008)	0.842
Myo-inositol	Hypertension	18	1.013 (0.996 to 1.030)	0.144	1.002 (0.994 to 1.011)	0.560	1.000 (0.993 to 1.007)	0.944		
Lysine	Hypertension	18	0.997 (0.982 to 1.012)	0.685	0.997 (0.991 to 1.003)	0.366	0.998 (0.992 to 1.003)	0.381		
Citrulline	Hypertension	14	0.981 (0.962 to 1.001)	0.086	0.996 (0.987 to 1.004)	0.330	1.000 (0.990 to 1.009)	0.941	0.996 (0.990 to 1.002)	0.196
Ornithine	Hypertension	14	1.006 (0.994 to 1.019)	0.313	1.003 (0.996 to 1.011)	0.406	1.004 (0.998 to 1.010)	0.181		

Exposure	Outcome	nSNP	MR Egger		Weighted median		Inverse variance weighted		MR_PRESSO	
			OR (95% CI)	pval	OR (95% CI)	pval	OR (95% CI)	pval	OR (95% CI)	pval
Retinol (Vitamin A)	Hypertension	13	0.976 (0.946 to 1.007)	0.158	0.997 (0.987 to 1.007)	0.567	0.995 (0.982 to 1.008)	0.430	0.997 (0.990 to 1.004)	0.409
1-methylnicotinamide	Hypertension	15	0.996 (0.975 to 1.019)	0.746	0.998 (0.990 to 1.007)	0.714	1.002 (0.994 to 1.011)	0.562	1.000 (0.993 to 1.007)	0.975
Uridine	Hypertension	7	1.018 (0.997 to 1.039)	0.150	0.995 (0.986 to 1.003)	0.210	0.995 (0.988 to 1.001)	0.125		
Plasma lactate	Hypertension	8	0.998 (0.976 to 1.020)	0.856	0.999 (0.990 to 1.009)	0.900	1.000 (0.992 to 1.008)	0.943		
Pristanate	Hypertension	6	0.992 (0.970 to 1.014)	0.522	0.996 (0.985 to 1.006)	0.423	1.000 (0.991 to 1.009)	0.938		
Phytanate	Hypertension	12	1.007 (0.993 to 1.021)	0.374	1.001 (0.992 to 1.010)	0.760	1.003 (0.996 to 1.011)	0.407		

Exposure	Outcome	nSNP	MR Egger		Weighted median		Inverse variance weighted		MR_PRESSO	
			OR (95% CI)	pval	OR (95% CI)	pval	OR (95% CI)	pval	OR (95% CI)	pval
Plasma free proline	Hypertension	21	1.001 (0.993 to 1.008)	0.836	1.003 (0.997 to 1.009)	0.291	1.001 (0.997 to 1.005)	0.631		
Glutamate	Hypertension	13	1.020 (0.989 to 1.052)	0.228	1.009 (0.999 to 1.019)	0.095	1.004 (0.992 to 1.016)	0.513	1.007 (1.000 to 1.014)	0.078
2'-deoxyuridine	Hypertension	16	1.009 (0.980 to 1.039)	0.545	0.997 (0.991 to 1.003)	0.338	0.993 (0.979 to 1.008)	0.368	0.999 (0.995 to 1.003)	0.598
Myristate (14:0)	Hypertension	11	1.010 (0.961 to 1.062)	0.696	1.000 (0.990 to 1.010)	0.999	0.994 (0.977 to 1.011)	0.488	1.001 (0.993 to 1.010)	0.772
Serine	Hypertension	22	0.990 (0.973 to 1.007)	0.253	0.998 (0.992 to 1.005)	0.616	0.994 (0.987 to 1.001)	0.085	0.997 (0.992 to 1.002)	0.204
Trans-urocanate	Hypertension	10	0.996 (0.986 to 1.007)	0.516	0.997 (0.992 to 1.003)	0.342	0.998 (0.994 to 1.003)	0.501		

Exposure	Outcome	nSNP	MR Egger		Weighted median		Inverse variance weighted		MR_PRESSO	
			OR (95% CI)	pval	OR (95% CI)	pval	OR (95% CI)	pval	OR (95% CI)	pval
Urea	Hypertension	12	1.001 (0.973 to 1.028)	0.968	1.001 (0.990 to 1.011)	0.919	0.999 (0.989 to 1.009)	0.831	0.996 (0.987 to 1.005)	0.439
Pyridoxal	Hypertension	19	1.000 (0.990 to 1.010)	0.957	1.000 (0.994 to 1.007)	0.927	0.999 (0.994 to 1.004)	0.627		
Glutamine	Hypertension	14	0.995 (0.983 to 1.006)	0.372	0.992 (0.986 to 0.999)	0.020	0.991 (0.986 to 0.997)	0.003		
Taurine	Hypertension	6	0.998 (0.959 to 1.038)	0.925	1.004 (0.991 to 1.017)	0.565	0.999 (0.985 to 1.014)	0.936	1.005 (0.992 to 1.018)	0.496
Salicylate	Hypertension	14	1.007 (0.994 to 1.020)	0.332	1.001 (0.993 to 1.009)	0.739	1.002 (0.996 to 1.008)	0.527		
Serotonin	Hypertension	12	1.016 (1.000 to 1.033)	0.083	1.009 (1.000 to 1.017)	0.042	1.005 (0.999 to 1.011)	0.104		

Exposure	Outcome	nSNP	MR Egger		Weighted median		Inverse variance weighted		MR_PRESSO	
			OR (95% CI)	pval	OR (95% CI)	pval	OR (95% CI)	pval	OR (95% CI)	pval
Spermidine	Hypertension	14	1.005 (0.981 to 1.030)	0.669	1.000 (0.991 to 1.008)	0.915	0.999 (0.990 to 1.008)	0.881	1.002 (0.995 to 1.010)	0.531
Dimethylglycine	Hypertension	15	1.003 (0.991 to 1.014)	0.673	1.001 (0.996 to 1.006)	0.686	0.999 (0.992 to 1.006)	0.803	1.002 (0.997 to 1.007)	0.540
Betaine	Hypertension	15	1.008 (0.982 to 1.036)	0.542	1.002 (0.995 to 1.008)	0.613	1.005 (0.997 to 1.013)	0.265	1.003 (0.996 to 1.009)	0.445
Xylose	Hypertension	10	0.994 (0.980 to 1.008)	0.449	1.003 (0.995 to 1.012)	0.433	1.001 (0.994 to 1.009)	0.715		
Tyrosine	Hypertension	13	1.009 (0.999 to 1.020)	0.113	1.003 (0.995 to 1.011)	0.454	1.003 (0.997 to 1.008)	0.326		
Caprate (10:0)	Hypertension	11	1.009 (0.994 to 1.024)	0.257	1.006 (0.997 to 1.015)	0.213	1.005 (0.998 to 1.012)	0.138		

Exposure	Outcome	nSNP	MR Egger		Weighted median		Inverse variance weighted		MR_PRESSO	
			OR (95% CI)	pval	OR (95% CI)	pval	OR (95% CI)	pval	OR (95% CI)	pval
Cytidine	Hypertension	17	0.997 (0.991 to 1.003)	0.350	0.997 (0.990 to 1.003)	0.283	0.998 (0.994 to 1.002)	0.282		
Pyruvate	Hypertension	12	0.996 (0.972 to 1.020)	0.724	1.005 (0.995 to 1.015)	0.331	1.007 (0.997 to 1.017)	0.202		
Alanine	Hypertension	10	1.004 (0.977 to 1.031)	0.801	0.996 (0.986 to 1.006)	0.414	0.999 (0.988 to 1.011)	0.930	0.999 (0.990 to 1.009)	0.905
Threonine	Hypertension	20	1.021 (0.992 to 1.049)	0.170	1.002 (0.995 to 1.009)	0.668	1.003 (0.993 to 1.013)	0.552	0.998 (0.993 to 1.003)	0.488
Cytosine	Hypertension	8	1.016 (0.990 to 1.043)	0.285	1.000 (0.989 to 1.010)	0.934	1.001 (0.988 to 1.013)	0.925	1.002 (0.993 to 1.011)	0.704
12,13-DiHOME	Hypertension	15	1.004 (0.991 to 1.016)	0.554	1.004 (0.996 to 1.011)	0.351	1.003 (0.997 to 1.008)	0.363		

Exposure	Outcome	nSNP	MR Egger		Weighted median		Inverse variance weighted		MR_PRESSO	
			OR (95% CI)	pval	OR (95% CI)	pval	OR (95% CI)	pval	OR (95% CI)	pval
Mannose	Hypertension	11	0.991 (0.978 to 1.005)	0.249	0.993 (0.987 to 1.000)	0.037	0.997 (0.990 to 1.003)	0.322		
Caffeine	Hypertension	12	1.002 (0.983 to 1.022)	0.822	0.999 (0.990 to 1.008)	0.814	0.999 (0.990 to 1.007)	0.742	0.996 (0.989 to 1.003)	0.311
Fructose	Hypertension	13	0.990 (0.974 to 1.007)	0.263	1.001 (0.992 to 1.010)	0.863	1.002 (0.994 to 1.011)	0.568		
Alpha-ketobutyrate	Hypertension	7	0.988 (0.951 to 1.026)	0.553	1.005 (0.991 to 1.019)	0.492	1.007 (0.992 to 1.023)	0.364	1.002 (0.988 to 1.016)	0.769
Cysteine	Hypertension	9	1.006 (0.981 to 1.031)	0.665	0.995 (0.983 to 1.006)	0.372	0.994 (0.983 to 1.004)	0.242		
Blood sugar	Hypertension	11	1.009 (0.992 to 1.026)	0.334	1.003 (0.994 to 1.013)	0.485	1.002 (0.996 to 1.009)	0.503		

Exposure	Outcome	nSNP	MR Egger		Weighted median		Inverse variance weighted		MR_PRESSO	
			OR (95% CI)	pval	OR (95% CI)	pval	OR (95% CI)	pval	OR (95% CI)	pval
Tryptophan	Hypertension	11	0.980 (0.960 to 1.000)	0.086	0.995 (0.986 to 1.005)	0.323	0.998 (0.990 to 1.007)	0.706		
Valine	Hypertension	8	0.983 (0.961 to 1.005)	0.179	0.993 (0.983 to 1.003)	0.160	0.992 (0.985 to 1.000)	0.053		
Pseudouridine	Hypertension	15	0.984 (0.916 to 1.058)	0.673	0.996 (0.986 to 1.007)	0.484	1.009 (0.986 to 1.033)	0.432	0.999 (0.992 to 1.007)	0.902
Dihydroorotate	Hypertension	21	0.989 (0.970 to 1.007)	0.245	0.994 (0.989 to 1.000)	0.050	0.997 (0.989 to 1.006)	0.524	1.000 (0.994 to 1.006)	0.965
Caprylate (8:0)	Hypertension	14	1.006 (0.989 to 1.022)	0.522	0.998 (0.989 to 1.006)	0.563	0.999 (0.992 to 1.006)	0.870		
Arachidate (20:0)	Hypertension	7	1.018 (0.996 to 1.039)	0.168	0.996 (0.983 to 1.008)	0.485	0.995 (0.984 to 1.005)	0.323		

Exposure	Outcome	nSNP	MR Egger		Weighted median		Inverse variance weighted		MR_PRESSO	
			OR (95% CI)	pval	OR (95% CI)	pval	OR (95% CI)	pval	OR (95% CI)	pval
Maltose	Hypertension	11	0.999 (0.978 to 1.021)	0.938	0.996 (0.988 to 1.005)	0.384	0.999 (0.991 to 1.007)	0.770		
N-stearoyl-sphinganine (d18:0/18:0)	Hypertension	5	1.015 (0.995 to 1.035)	0.240	1.001 (0.991 to 1.011)	0.886	1.001 (0.993 to 1.009)	0.818		
Sucrose	Hypertension	8	1.013 (0.984 to 1.043)	0.418	1.004 (0.993 to 1.015)	0.502	1.005 (0.995 to 1.016)	0.332		
Caproate (6:0)	Hypertension	14	1.003 (0.991 to 1.016)	0.629	1.006 (1.000 to 1.013)	0.070	1.004 (0.999 to 1.009)	0.081		
Nonadecanoate (19:0)	Hypertension	13	1.001 (0.983 to 1.019)	0.913	1.007 (0.998 to 1.016)	0.107	1.006 (0.998 to 1.015)	0.124		
Plasma free asparagine	Hypertension	13	0.999 (0.988 to 1.011)	0.871	0.999 (0.996 to 1.003)	0.619	0.996 (0.989 to 1.004)	0.348	0.989 (0.982 to 0.996)	0.013

Exposure	Outcome	nSNP	MR Egger		Weighted median		Inverse variance weighted		MR_PRESSO	
			OR (95% CI)	pval	OR (95% CI)	pval	OR (95% CI)	pval	OR (95% CI)	pval
X-07765	Hypertension	16	0.998 (0.982 to 1.014)	0.800	1.005 (0.998 to 1.013)	0.157	1.003 (0.996 to 1.011)	0.374	1.005 (0.999 to 1.012)	0.127
Kynurenate	Hypertension	19	0.991 (0.970 to 1.013)	0.439	1.000 (0.995 to 1.006)	0.854	1.005 (0.993 to 1.017)	0.379	0.999 (0.996 to 1.003)	0.752
Alpha-ketoglutarate	Hypertension	14	1.011 (0.996 to 1.025)	0.168	1.002 (0.994 to 1.010)	0.579	0.999 (0.994 to 1.005)	0.860		
X-11308	Hypertension	17	0.991 (0.977 to 1.006)	0.247	0.995 (0.988 to 1.001)	0.101	1.000 (0.992 to 1.007)	0.904	0.997 (0.993 to 1.002)	0.222
Pentadecanoate (15:0)	Hypertension	9	1.031 (0.975 to 1.089)	0.318	1.001 (0.989 to 1.013)	0.885	1.000 (0.978 to 1.022)	0.972	1.006 (0.998 to 1.013)	0.189
X-11315	Hypertension	15	0.998 (0.990 to 1.006)	0.662	0.998 (0.992 to 1.003)	0.438	0.998 (0.994 to 1.002)	0.405		

Exposure	Outcome	nSNP	MR Egger		Weighted median		Inverse variance weighted		MR_PRESSO	
			OR (95% CI)	pval	OR (95% CI)	pval	OR (95% CI)	pval	OR (95% CI)	pval
Margarate (17:0)	Hypertension	11	1.007 (0.957 to 1.059)	0.800	1.000 (0.989 to 1.012)	0.953	0.997 (0.979 to 1.015)	0.735	0.998 (0.989 to 1.007)	0.687
X-11372	Hypertension	14	0.996 (0.977 to 1.016)	0.709	1.003 (0.995 to 1.011)	0.453	1.001 (0.994 to 1.008)	0.843		
X-10458	Hypertension	13	1.013 (1.000 to 1.025)	0.072	1.002 (0.995 to 1.009)	0.568	1.001 (0.995 to 1.006)	0.848		
X-11299	Hypertension	11	1.015 (1.000 to 1.030)	0.077	1.004 (0.995 to 1.012)	0.425	1.005 (0.998 to 1.012)	0.138		
X-11478	Hypertension	16	0.990 (0.969 to 1.012)	0.402	0.999 (0.992 to 1.007)	0.878	1.002 (0.993 to 1.011)	0.700	0.997 (0.991 to 1.003)	0.326
X-11849	Hypertension	16	1.002 (0.981 to 1.023)	0.875	0.993 (0.985 to 1.001)	0.075	0.993 (0.985 to 1.000)	0.055		

Exposure	Outcome	nSNP	MR Egger		Weighted median		Inverse variance weighted		MR_PRESSO	
			OR (95% CI)	pval	OR (95% CI)	pval	OR (95% CI)	pval	OR (95% CI)	pval
X-11787	Hypertension	10	0.998 (0.989 to 1.007)	0.663	0.998 (0.994 to 1.002)	0.320	0.997 (0.993 to 1.002)	0.267		
X-11632	Hypertension	15	0.995 (0.975 to 1.015)	0.641	1.008 (1.000 to 1.017)	0.050	1.000 (0.992 to 1.007)	0.930		
X-11852	Hypertension	12	0.995 (0.969 to 1.022)	0.715	0.999 (0.990 to 1.008)	0.860	1.001 (0.990 to 1.011)	0.872	0.997 (0.990 to 1.004)	0.426
X-11847	Hypertension	12	0.992 (0.978 to 1.007)	0.337	0.996 (0.989 to 1.004)	0.371	0.997 (0.990 to 1.003)	0.314		
X-11483	Hypertension	13	1.002 (0.986 to 1.019)	0.793	0.998 (0.990 to 1.007)	0.696	0.997 (0.990 to 1.004)	0.350		
X-11470	Hypertension	22	1.002 (0.995 to 1.008)	0.574	1.001 (0.996 to 1.007)	0.595	1.003 (0.999 to 1.007)	0.162		

Exposure	Outcome	nSNP	MR Egger		Weighted median		Inverse variance weighted		MR_PRESSO	
			OR (95% CI)	pval	OR (95% CI)	pval	OR (95% CI)	pval	OR (95% CI)	pval
X-11381	Hypertension	17	1.012 (0.999 to 1.025)	0.098	1.011 (1.004 to 1.017)	0.001	1.007 (1.001 to 1.014)	0.015	1.004 (0.999 to 1.009)	0.102
X-11850	Hypertension	13	1.003 (0.982 to 1.024)	0.789	0.992 (0.983 to 1.001)	0.100	0.993 (0.984 to 1.002)	0.113		
X-11795	Hypertension	8	1.002 (0.983 to 1.020)	0.875	0.997 (0.987 to 1.008)	0.614	0.997 (0.989 to 1.005)	0.471		
X-11444	Hypertension	21	0.998 (0.989 to 1.007)	0.625	0.998 (0.993 to 1.004)	0.546	1.003 (0.997 to 1.009)	0.261	1.000 (0.997 to 1.004)	0.883
X-11858	Hypertension	11	1.002 (0.983 to 1.021)	0.850	1.005 (0.997 to 1.013)	0.240	1.006 (0.999 to 1.012)	0.079		
X-11843	Hypertension	15	1.003 (0.993 to 1.013)	0.591	1.000 (0.993 to 1.008)	0.906	1.002 (0.996 to 1.008)	0.499		

Exposure	Outcome	nSNP	MR Egger		Weighted median		Inverse variance weighted		MR_PRESSO	
			OR (95% CI)	pval	OR (95% CI)	pval	OR (95% CI)	pval	OR (95% CI)	pval
X-12127	Hypertension	14	0.988 (0.958 to 1.019)	0.447	1.008 (1.000 to 1.016)	0.056	1.005 (0.996 to 1.014)	0.293	1.007 (1.001 to 1.014)	0.054
X-12221	Hypertension	7	1.018 (0.996 to 1.040)	0.166	0.997 (0.987 to 1.008)	0.580	1.001 (0.994 to 1.009)	0.747		
X-12283	Hypertension	14	1.013 (0.998 to 1.028)	0.111	1.006 (0.998 to 1.015)	0.140	1.000 (0.993 to 1.007)	0.976		
X-12410	Hypertension	13	1.010 (0.997 to 1.023)	0.161	1.007 (1.000 to 1.014)	0.050	1.001 (0.994 to 1.007)	0.855	0.995 (0.990 to 1.001)	0.146
X-12104	Hypertension	6	0.993 (0.973 to 1.014)	0.553	1.005 (0.995 to 1.015)	0.291	1.005 (0.997 to 1.014)	0.210		
X-12193	Hypertension	16	0.995 (0.985 to 1.006)	0.393	0.996 (0.989 to 1.003)	0.308	0.997 (0.991 to 1.002)	0.208		

Exposure	Outcome	nSNP	MR Egger		Weighted median		Inverse variance weighted		MR_PRESSO	
			OR (95% CI)	pval	OR (95% CI)	pval	OR (95% CI)	pval	OR (95% CI)	pval
X-12216	Hypertension	12	1.013 (0.973 to 1.055)	0.538	0.993 (0.984 to 1.002)	0.107	0.993 (0.978 to 1.007)	0.336	0.995 (0.987 to 1.002)	0.207
X-12306	Hypertension	13	1.001 (0.986 to 1.017)	0.865	1.005 (0.997 to 1.012)	0.215	1.002 (0.995 to 1.009)	0.595		
X-12100	Hypertension	11	0.967 (0.873 to 1.071)	0.537	0.999 (0.989 to 1.008)	0.770	1.024 (0.990 to 1.060)	0.171	1.011 (1.003 to 1.019)	0.051
X-12261	Hypertension	8	0.995 (0.979 to 1.011)	0.535	1.001 (0.993 to 1.008)	0.892	0.999 (0.993 to 1.005)	0.785		
X-11880	Hypertension	16	0.995 (0.979 to 1.012)	0.584	1.001 (0.993 to 1.009)	0.821	1.000 (0.994 to 1.007)	0.903		
X-12117	Hypertension	25	1.001 (0.994 to 1.009)	0.724	0.998 (0.993 to 1.002)	0.337	0.997 (0.992 to 1.002)	0.207	0.998 (0.994 to 1.002)	0.341

Exposure	Outcome	nSNP	MR Egger		Weighted median		Inverse variance weighted		MR_PRESSO	
			OR (95% CI)	pval	OR (95% CI)	pval	OR (95% CI)	pval	OR (95% CI)	pval
X-12407	Hypertension	7	1.012 (0.991 to 1.033)	0.320	0.990 (0.979 to 1.002)	0.090	0.993 (0.985 to 1.001)	0.086		
X-12101	Hypertension	13	0.982 (0.956 to 1.009)	0.214	0.990 (0.981 to 0.999)	0.030	0.999 (0.988 to 1.009)	0.816	0.996 (0.987 to 1.004)	0.345
X-12026	Hypertension	18	0.996 (0.977 to 1.015)	0.664	1.005 (0.997 to 1.013)	0.242	1.001 (0.993 to 1.009)	0.790		
X-12411	Hypertension	9	0.998 (0.983 to 1.012)	0.750	0.996 (0.988 to 1.004)	0.377	0.995 (0.987 to 1.004)	0.272		
X-12013	Hypertension	12	0.982 (0.969 to 0.995)	0.021	0.996 (0.988 to 1.004)	0.293	1.000 (0.993 to 1.006)	0.923		
X-12007	Hypertension	10	1.000 (0.978 to 1.023)	0.987	1.004 (0.995 to 1.013)	0.401	1.002 (0.992 to 1.012)	0.708	1.006 (0.999 to 1.013)	0.153

Exposure	Outcome	nSNP	MR Egger		Weighted median		Inverse variance weighted		MR_PRESSO	
			OR (95% CI)	pval	OR (95% CI)	pval	OR (95% CI)	pval	OR (95% CI)	pval
X-12462	Hypertension	6	0.987 (0.968 to 1.006)	0.241	0.990 (0.978 to 1.001)	0.073	0.993 (0.985 to 1.002)	0.152		
X-12714	Hypertension	7	1.001 (0.986 to 1.016)	0.874	1.005 (0.998 to 1.011)	0.142	1.005 (0.999 to 1.010)	0.102		
X-12740	Hypertension	10	1.001 (0.987 to 1.015)	0.908	0.992 (0.984 to 1.000)	0.047	0.995 (0.988 to 1.001)	0.084		
X-12730	Hypertension	8	1.000 (0.983 to 1.017)	0.990	1.001 (0.992 to 1.011)	0.791	1.000 (0.992 to 1.009)	0.953		
X-12701	Hypertension	8	1.011 (0.991 to 1.032)	0.328	0.998 (0.990 to 1.007)	0.712	1.002 (0.995 to 1.009)	0.547		
X-12816	Hypertension	17	1.010 (0.991 to 1.030)	0.330	1.001 (0.995 to 1.008)	0.674	0.999 (0.990 to 1.009)	0.888	1.001 (0.996 to 1.007)	0.689

Exposure	Outcome	nSNP	MR Egger		Weighted median		Inverse variance weighted		MR_PRESSO	
			OR (95% CI)	pval	OR (95% CI)	pval	OR (95% CI)	pval	OR (95% CI)	pval
X-12731	Hypertension	17	0.990 (0.978 to 1.003)	0.151	0.993 (0.988 to 0.999)	0.025	0.994 (0.990 to 0.999)	0.014		
X-12707	Hypertension	15	1.006 (0.995 to 1.017)	0.307	1.003 (0.996 to 1.010)	0.408	1.002 (0.996 to 1.009)	0.534	0.999 (0.993 to 1.006)	0.836
X-12729	Hypertension	14	1.005 (0.991 to 1.019)	0.484	1.000 (0.993 to 1.008)	0.940	1.001 (0.995 to 1.006)	0.835		
X-12830	Hypertension	15	0.989 (0.978 to 1.000)	0.079	1.003 (0.996 to 1.009)	0.385	1.002 (0.997 to 1.007)	0.404		
X-12818	Hypertension	8	0.993 (0.975 to 1.010)	0.440	0.998 (0.989 to 1.008)	0.732	0.997 (0.990 to 1.004)	0.411		
X-12812	Hypertension	10	1.002 (0.981 to 1.023)	0.855	0.997 (0.987 to 1.006)	0.463	1.001 (0.992 to 1.010)	0.844		

Exposure	Outcome	nSNP	MR Egger		Weighted median		Inverse variance weighted		MR_PRESSO	
			OR (95% CI)	pval	OR (95% CI)	pval	OR (95% CI)	pval	OR (95% CI)	pval
X-12822	Hypertension	9	0.997 (0.975 to 1.019)	0.801	1.011 (1.005 to 1.017)	7.17e-04	1.012 (1.002 to 1.021)	0.020	1.008 (1.002 to 1.015)	0.038
X-12738	Hypertension	8	0.999 (0.986 to 1.013)	0.903	1.002 (0.993 to 1.011)	0.657	1.001 (0.994 to 1.008)	0.749		
X-12544	Hypertension	14	0.997 (0.987 to 1.008)	0.650	1.001 (0.993 to 1.008)	0.870	1.002 (0.997 to 1.008)	0.448		
X-12680	Hypertension	8	1.012 (0.975 to 1.051)	0.548	0.998 (0.987 to 1.009)	0.693	1.003 (0.993 to 1.013)	0.561		
X-12906	Hypertension	9	1.002 (0.986 to 1.017)	0.852	1.003 (0.993 to 1.013)	0.537	1.004 (0.997 to 1.012)	0.294		
X-13684	Hypertension	21	0.996 (0.988 to 1.003)	0.268	0.998 (0.993 to 1.003)	0.390	1.000 (0.996 to 1.004)	0.988		

Exposure	Outcome	nSNP	MR Egger		Weighted median		Inverse variance weighted		MR_PRESSO	
			OR (95% CI)	pval	OR (95% CI)	pval	OR (95% CI)	pval	OR (95% CI)	pval
X-13507	Hypertension	6	0.990 (0.979 to 1.000)	0.120	0.995 (0.987 to 1.004)	0.267	0.998 (0.990 to 1.006)	0.615		
X-12851	Hypertension	9	0.966 (0.918 to 1.017)	0.227	0.994 (0.985 to 1.004)	0.253	1.005 (0.987 to 1.023)	0.578	0.997 (0.987 to 1.007)	0.615
X-12849	Hypertension	14	1.003 (0.988 to 1.018)	0.724	0.997 (0.988 to 1.005)	0.452	0.997 (0.990 to 1.003)	0.338		
X-12847	Hypertension	9	1.031 (1.004 to 1.059)	0.060	1.007 (0.996 to 1.019)	0.193	1.007 (0.998 to 1.016)	0.117		
X-13553	Hypertension	12	0.995 (0.964 to 1.028)	0.771	0.996 (0.986 to 1.006)	0.476	1.008 (0.993 to 1.024)	0.302	1.001 (0.994 to 1.007)	0.878
X-12839	Hypertension	17	1.000 (0.985 to 1.015)	0.998	0.998 (0.992 to 1.005)	0.584	1.000 (0.994 to 1.006)	0.950	0.999 (0.995 to 1.004)	0.674

Exposure	Outcome	nSNP	MR Egger		Weighted median		Inverse variance weighted		MR_PRESSO	
			OR (95% CI)	pval	OR (95% CI)	pval	OR (95% CI)	pval	OR (95% CI)	pval
X-13729	Hypertension	20	1.006 (0.994 to 1.017)	0.375	0.999 (0.992 to 1.006)	0.766	0.999 (0.993 to 1.005)	0.795		
X-13007	Hypertension	17	1.004 (0.992 to 1.016)	0.492	1.002 (0.996 to 1.009)	0.479	1.000 (0.996 to 1.005)	0.855		
X-13844	Hypertension	9	0.999 (0.977 to 1.021)	0.910	0.997 (0.985 to 1.009)	0.607	1.002 (0.992 to 1.012)	0.734		
X-13728	Hypertension	10	1.025 (1.000 to 1.051)	0.082	0.999 (0.989 to 1.010)	0.908	1.004 (0.994 to 1.013)	0.482		
X-12844	Hypertension	22	0.997 (0.983 to 1.011)	0.661	0.995 (0.990 to 1.000)	0.060	0.997 (0.992 to 1.003)	0.343	1.000 (0.995 to 1.004)	0.867
X-13695	Hypertension	9	0.997 (0.984 to 1.011)	0.716	0.999 (0.990 to 1.008)	0.848	0.998 (0.991 to 1.004)	0.534		

Exposure	Outcome	nSNP	MR Egger		Weighted median		Inverse variance weighted		MR_PRESSO	
			OR (95% CI)	pval	OR (95% CI)	pval	OR (95% CI)	pval	OR (95% CI)	pval
X-13723	Hypertension	7	1.003 (0.977 to 1.030)	0.820	1.003 (0.994 to 1.013)	0.457	1.003 (0.996 to 1.010)	0.392		
X-14056	Hypertension	5	1.101 (0.751 to 1.612)	0.656	1.006 (0.993 to 1.020)	0.347	1.010 (0.999 to 1.020)	0.063		
X-13866	Hypertension	15	0.998 (0.966 to 1.030)	0.890	0.998 (0.989 to 1.007)	0.634	1.003 (0.991 to 1.015)	0.599	1.001 (0.993 to 1.009)	0.834
X-17146	Hypertension	9	1.008 (0.985 to 1.032)	0.523	0.997 (0.987 to 1.008)	0.620	0.999 (0.989 to 1.009)	0.821	0.999 (0.993 to 1.005)	0.726
X-16397	Hypertension	10	0.997 (0.978 to 1.017)	0.798	0.996 (0.988 to 1.004)	0.347	0.999 (0.993 to 1.006)	0.810		
X-17335	Hypertension	6	1.011 (0.993 to 1.030)	0.291	1.001 (0.990 to 1.013)	0.804	0.996 (0.987 to 1.006)	0.424		

Exposure	Outcome	nSNP	MR Egger		Weighted median		Inverse variance weighted		MR_PRESSO	
			OR (95% CI)	pval	OR (95% CI)	pval	OR (95% CI)	pval	OR (95% CI)	pval
X-16964	Hypertension	11	1.014 (0.977 to 1.053)	0.472	1.006 (0.996 to 1.016)	0.240	1.006 (0.994 to 1.019)	0.323	1.007 (0.998 to 1.016)	0.150
X-16935	Hypertension	12	0.994 (0.980 to 1.008)	0.432	1.003 (0.998 to 1.008)	0.228	1.005 (0.997 to 1.012)	0.263	1.005 (0.999 to 1.011)	0.119
X-15486	Hypertension	7	1.033 (1.008 to 1.059)	0.050	1.013 (1.004 to 1.022)	0.004	1.008 (0.995 to 1.021)	0.250	1.009 (0.995 to 1.023)	0.273
X-16580	Hypertension	10	0.993 (0.972 to 1.015)	0.537	1.003 (0.993 to 1.012)	0.562	1.002 (0.995 to 1.009)	0.622		
X-17325	Hypertension	11	0.981 (0.951 to 1.012)	0.267	0.991 (0.982 to 1.001)	0.073	0.989 (0.979 to 0.998)	0.016	0.997 (0.991 to 1.004)	0.492
X-17328	Hypertension	12	0.997 (0.977 to 1.018)	0.814	1.006 (0.999 to 1.014)	0.085	1.007 (1.000 to 1.014)	0.042	1.005 (0.999 to 1.011)	0.121

Exposure	Outcome	nSNP	MR Egger		Weighted median		Inverse variance weighted		MR_PRESSO	
			OR (95% CI)	pval	OR (95% CI)	pval	OR (95% CI)	pval	OR (95% CI)	pval
X-16087	Hypertension	11	0.991 (0.978 to 1.005)	0.232	0.989 (0.983 to 0.994)	8.65e-05	0.991 (0.986 to 0.997)	0.002		
X-17010	Hypertension	9	0.995 (0.967 to 1.023)	0.720	1.000 (0.991 to 1.011)	0.923	1.001 (0.992 to 1.011)	0.771		
X-14939	Hypertension	11	0.993 (0.971 to 1.015)	0.525	1.000 (0.993 to 1.007)	0.963	1.001 (0.990 to 1.012)	0.852	0.998 (0.991 to 1.005)	0.558
X-15461	Hypertension	10	0.983 (0.960 to 1.007)	0.209	1.000 (0.989 to 1.010)	0.953	0.996 (0.986 to 1.006)	0.433		
X-15523	Hypertension	38	0.999 (0.998 to 1.000)	0.291	0.999 (0.998 to 1.000)	0.095	0.999 (0.999 to 1.000)	0.254		
X-15728	Hypertension	16	1.002 (0.989 to 1.016)	0.751	1.000 (0.993 to 1.007)	0.949	1.003 (0.997 to 1.008)	0.303		

Exposure	Outcome	nSNP	MR Egger		Weighted median		Inverse variance weighted		MR_PRESSO	
			OR (95% CI)	pval	OR (95% CI)	pval	OR (95% CI)	pval	OR (95% CI)	pval
X-15503	Hypertension	13	0.965 (0.915 to 1.019)	0.227	1.001 (0.993 to 1.008)	0.871	1.009 (0.987 to 1.033)	0.420	1.001 (0.997 to 1.005)	0.640
X-16124	Hypertension	15	0.999 (0.987 to 1.010)	0.831	1.000 (0.994 to 1.006)	0.939	1.003 (0.997 to 1.008)	0.309		
X-17438	Hypertension	10	1.004 (0.991 to 1.016)	0.594	1.004 (0.996 to 1.012)	0.348	1.004 (0.997 to 1.010)	0.238		
X-17354	Hypertension	6	1.013 (0.986 to 1.041)	0.401	0.997 (0.988 to 1.006)	0.565	0.996 (0.988 to 1.004)	0.315		
X-17654	Hypertension	13	0.998 (0.991 to 1.005)	0.577	1.000 (0.996 to 1.005)	0.916	0.998 (0.994 to 1.002)	0.316		
X-17676	Hypertension	20	1.006 (0.996 to 1.015)	0.256	1.000 (0.994 to 1.007)	0.876	1.000 (0.994 to 1.005)	0.915		

Exposure	Outcome	nSNP	MR Egger		Weighted median		Inverse variance weighted		MR_PRESSO	
			OR (95% CI)	pval	OR (95% CI)	pval	OR (95% CI)	pval	OR (95% CI)	pval
X-17367	Hypertension	16	0.979 (0.962 to 0.997)	0.037	0.990 (0.983 to 0.997)	0.006	0.993 (0.985 to 1.000)	0.065	0.997 (0.991 to 1.002)	0.222
X-17357	Hypertension	16	0.991 (0.980 to 1.002)	0.133	0.992 (0.987 to 0.998)	0.013	0.994 (0.989 to 0.999)	0.014		
X-18886	Hypertension	8	1.032 (0.978 to 1.088)	0.295	1.008 (0.996 to 1.020)	0.172	1.007 (0.991 to 1.024)	0.374	1.004 (0.994 to 1.015)	0.462
X-18887	Hypertension	7	1.014 (0.986 to 1.042)	0.374	1.006 (0.996 to 1.016)	0.256	1.003 (0.996 to 1.011)	0.385		
X-17653	Hypertension	15	0.989 (0.972 to 1.006)	0.237	0.997 (0.990 to 1.003)	0.260	1.002 (0.993 to 1.010)	0.690	0.999 (0.993 to 1.005)	0.807
X-17346	Hypertension	12	0.999 (0.993 to 1.005)	0.718	1.000 (0.995 to 1.006)	0.900	1.001 (0.997 to 1.005)	0.745		

Exposure	Outcome	nSNP	MR Egger		Weighted median		Inverse variance weighted		MR_PRESSO	
			OR (95% CI)	pval	OR (95% CI)	pval	OR (95% CI)	pval	OR (95% CI)	pval
X-17690	Hypertension	21	1.003 (0.991 to 1.016)	0.616	0.999 (0.993 to 1.005)	0.671	0.999 (0.994 to 1.004)	0.594	0.995 (0.990 to 0.999)	0.033
X-18345	Hypertension	13	0.990 (0.981 to 1.000)	0.065	1.001 (0.994 to 1.008)	0.877	0.999 (0.994 to 1.004)	0.584		
X-17685	Hypertension	10	1.006 (0.989 to 1.022)	0.525	0.997 (0.989 to 1.006)	0.540	0.994 (0.988 to 1.001)	0.077		
X-18888	Hypertension	11	1.006 (0.992 to 1.020)	0.436	1.006 (0.998 to 1.014)	0.170	1.007 (1.001 to 1.013)	0.017		
X-18901	Hypertension	14	1.000 (0.989 to 1.012)	0.936	0.997 (0.989 to 1.005)	0.472	1.000 (0.994 to 1.005)	0.883		
X-17351	Hypertension	13	1.008 (0.979 to 1.037)	0.610	1.001 (0.992 to 1.010)	0.852	0.999 (0.991 to 1.006)	0.712		

Exposure	Outcome	nSNP	MR Egger		Weighted median		Inverse variance weighted		MR_PRESSO	
			OR (95% CI)	pval	OR (95% CI)	pval	OR (95% CI)	pval	OR (95% CI)	pval
X-18779	Hypertension	5	0.993 (0.953 to 1.035)	0.758	1.013 (0.998 to 1.028)	0.090	1.020 (0.998 to 1.041)	0.071	1.009 (0.998 to 1.020)	0.211
X-21286	Hypertension	17	1.027 (0.992 to 1.063)	0.155	1.003 (0.996 to 1.011)	0.385	1.001 (0.993 to 1.010)	0.767	1.000 (0.994 to 1.006)	0.986
X-21355	Hypertension	6	0.989 (0.967 to 1.011)	0.387	0.999 (0.988 to 1.010)	0.879	0.997 (0.987 to 1.007)	0.541		
X-21319	Hypertension	9	0.962 (0.938 to 0.986)	0.019	1.001 (0.992 to 1.011)	0.784	0.998 (0.987 to 1.009)	0.700	1.005 (0.997 to 1.012)	0.239
X-21258	Hypertension	13	0.994 (0.979 to 1.010)	0.508	0.995 (0.987 to 1.003)	0.211	0.994 (0.986 to 1.002)	0.119	0.996 (0.989 to 1.003)	0.256
X-21364	Hypertension	11	0.975 (0.948 to 1.003)	0.116	0.994 (0.983 to 1.004)	0.222	0.992 (0.983 to 1.001)	0.086	0.997 (0.987 to 1.006)	0.493

Exposure	Outcome	nSNP	MR Egger		Weighted median		Inverse variance weighted		MR_PRESSO	
			OR (95% CI)	pval	OR (95% CI)	pval	OR (95% CI)	pval	OR (95% CI)	pval
X-21353	Hypertension	12	1.001 (0.987 to 1.014)	0.937	0.999 (0.992 to 1.006)	0.693	0.999 (0.993 to 1.004)	0.647		
X-19438	Hypertension	12	1.002 (0.984 to 1.022)	0.806	0.997 (0.988 to 1.005)	0.414	0.999 (0.992 to 1.007)	0.874		
X-18913	Hypertension	11	1.000 (0.984 to 1.017)	0.974	1.002 (0.994 to 1.011)	0.592	1.005 (0.996 to 1.013)	0.263		
X-18921	Hypertension	15	1.001 (0.993 to 1.009)	0.859	0.999 (0.995 to 1.003)	0.595	0.999 (0.994 to 1.003)	0.509		
X-21339	Hypertension	14	0.985 (0.955 to 1.016)	0.351	1.001 (0.992 to 1.010)	0.808	1.002 (0.988 to 1.016)	0.777	1.005 (0.998 to 1.011)	0.179
X-19299	Hypertension	11	0.992 (0.977 to 1.007)	0.327	1.000 (0.991 to 1.008)	0.920	0.999 (0.991 to 1.006)	0.695		

Exposure	Outcome	nSNP	MR Egger		Weighted median		Inverse variance weighted		MR_PRESSO	
			OR (95% CI)	pval	OR (95% CI)	pval	OR (95% CI)	pval	OR (95% CI)	pval
X-18935	Hypertension	17	0.999 (0.988 to 1.009)	0.805	1.000 (0.994 to 1.007)	0.891	1.001 (0.997 to 1.005)	0.662		
X-21310	Hypertension	13	1.002 (0.983 to 1.020)	0.856	1.004 (0.996 to 1.013)	0.331	1.003 (0.995 to 1.010)	0.502		
X-21283	Hypertension	11	0.998 (0.978 to 1.017)	0.812	1.001 (0.998 to 1.005)	0.483	1.005 (0.992 to 1.019)	0.433	1.004 (0.993 to 1.015)	0.470
X-21285	Hypertension	18	0.999 (0.989 to 1.009)	0.867	0.997 (0.990 to 1.003)	0.267	0.999 (0.994 to 1.003)	0.511		
X-21312	Hypertension	12	0.992 (0.978 to 1.006)	0.290	1.000 (0.994 to 1.007)	0.925	1.001 (0.995 to 1.007)	0.752		
X-18922	Hypertension	18	1.001 (0.993 to 1.010)	0.775	0.997 (0.992 to 1.003)	0.348	0.997 (0.993 to 1.001)	0.115		

Exposure	Outcome	nSNP	MR Egger		Weighted median		Inverse variance weighted		MR_PRESSO	
			OR (95% CI)	pval	OR (95% CI)	pval	OR (95% CI)	pval	OR (95% CI)	pval
X-21442	Hypertension	9	1.025 (0.985 to 1.065)	0.260	0.999 (0.990 to 1.008)	0.765	0.993 (0.982 to 1.004)	0.236	0.998 (0.993 to 1.004)	0.544
X-21796	Hypertension	15	0.984 (0.956 to 1.014)	0.313	0.996 (0.989 to 1.004)	0.360	1.002 (0.992 to 1.013)	0.661	0.998 (0.993 to 1.002)	0.363
X-21441	Hypertension	20	0.994 (0.986 to 1.001)	0.100	0.995 (0.991 to 0.999)	0.014	0.997 (0.993 to 1.002)	0.259	0.996 (0.994 to 0.998)	0.004
X-21752	Hypertension	10	1.011 (0.996 to 1.026)	0.203	1.001 (0.993 to 1.009)	0.858	1.003 (0.997 to 1.009)	0.373		
X-21807	Hypertension	10	1.007 (0.991 to 1.022)	0.419	0.995 (0.987 to 1.003)	0.245	0.994 (0.987 to 1.002)	0.121		
X-21736	Hypertension	19	0.994 (0.983 to 1.005)	0.315	1.004 (0.997 to 1.011)	0.236	1.004 (0.998 to 1.010)	0.174		

Exposure	Outcome	nSNP	MR Egger		Weighted median		Inverse variance weighted		MR_PRESSO	
			OR (95% CI)	pval	OR (95% CI)	pval	OR (95% CI)	pval	OR (95% CI)	pval
X-21829	Hypertension	17	1.004 (0.990 to 1.017)	0.585	1.000 (0.993 to 1.007)	0.969	1.002 (0.996 to 1.007)	0.607		
X-21471	Hypertension	18	0.996 (0.989 to 1.004)	0.346	0.997 (0.992 to 1.001)	0.126	0.998 (0.994 to 1.002)	0.402	0.998 (0.995 to 1.001)	0.136
X-21733	Hypertension	20	0.999 (0.980 to 1.018)	0.913	0.995 (0.988 to 1.001)	0.118	0.998 (0.991 to 1.005)	0.573	0.995 (0.990 to 1.001)	0.114
X-21742	Hypertension	7	0.999 (0.974 to 1.025)	0.949	0.999 (0.990 to 1.008)	0.823	1.000 (0.986 to 1.014)	0.965	1.000 (0.993 to 1.006)	0.894
X-21821	Hypertension	8	1.010 (0.994 to 1.026)	0.284	1.003 (0.992 to 1.013)	0.630	1.002 (0.995 to 1.010)	0.536		
X-21470	Hypertension	13	0.993 (0.982 to 1.005)	0.262	1.003 (0.997 to 1.008)	0.365	1.000 (0.994 to 1.005)	0.871	1.001 (0.996 to 1.006)	0.741

Exposure	Outcome	nSNP	MR Egger		Weighted median		Inverse variance weighted		MR_PRESSO	
			OR (95% CI)	pval	OR (95% CI)	pval	OR (95% CI)	pval	OR (95% CI)	pval
X-21383	Hypertension	13	1.009 (0.987 to 1.032)	0.428	1.002 (0.994 to 1.011)	0.582	0.999 (0.993 to 1.005)	0.725		
X-21607	Hypertension	19	0.992 (0.982 to 1.003)	0.180	1.002 (0.998 to 1.007)	0.344	1.006 (1.000 to 1.012)	0.052	1.005 (1.000 to 1.009)	0.063
X-21834	Hypertension	12	0.986 (0.973 to 1.000)	0.076	0.998 (0.990 to 1.006)	0.576	0.998 (0.991 to 1.004)	0.432		
X-21467	Hypertension	20	0.991 (0.982 to 1.001)	0.099	0.995 (0.991 to 1.000)	0.031	0.995 (0.990 to 1.001)	0.120	0.996 (0.993 to 1.000)	0.038
X-22776	Hypertension	10	1.012 (0.998 to 1.026)	0.145	1.000 (0.992 to 1.008)	0.987	1.001 (0.995 to 1.008)	0.683		
X-23655	Hypertension	13	1.003 (0.968 to 1.040)	0.859	0.996 (0.987 to 1.005)	0.349	0.995 (0.984 to 1.005)	0.311	0.999 (0.990 to 1.007)	0.754

Exposure	Outcome	nSNP	MR Egger		Weighted median		Inverse variance weighted		MR_PRESSO	
			OR (95% CI)	pval	OR (95% CI)	pval	OR (95% CI)	pval	OR (95% CI)	pval
X-22834	Hypertension	11	0.975 (0.958 to 0.992)	0.018	0.993 (0.984 to 1.001)	0.104	0.992 (0.985 to 1.000)	0.038		
X-22771	Hypertension	7	1.011 (0.971 to 1.052)	0.624	0.998 (0.986 to 1.009)	0.672	0.997 (0.988 to 1.006)	0.558		
X-23639	Hypertension	13	0.985 (0.946 to 1.025)	0.470	0.996 (0.987 to 1.005)	0.430	0.995 (0.983 to 1.007)	0.406	0.996 (0.988 to 1.004)	0.374
X-23648	Hypertension	10	1.026 (0.976 to 1.077)	0.342	1.003 (0.993 to 1.014)	0.576	1.008 (0.990 to 1.027)	0.393	1.002 (0.994 to 1.010)	0.617
X-23654	Hypertension	21	1.001 (0.994 to 1.007)	0.850	1.000 (0.995 to 1.006)	0.966	0.999 (0.995 to 1.003)	0.647		
X-22520	Hypertension	10	0.987 (0.971 to 1.004)	0.174	0.997 (0.990 to 1.004)	0.420	0.998 (0.992 to 1.005)	0.569		

Exposure	Outcome	nSNP	MR Egger		Weighted median		Inverse variance weighted		MR_PRESSO	
			OR (95% CI)	pval	OR (95% CI)	pval	OR (95% CI)	pval	OR (95% CI)	pval
X-23641	Hypertension	22	1.008 (0.992 to 1.023)	0.349	1.000 (0.995 to 1.005)	0.940	1.002 (0.996 to 1.007)	0.559	1.001 (0.998 to 1.003)	0.655
X-23276	Hypertension	13	0.989 (0.975 to 1.004)	0.178	0.995 (0.988 to 1.002)	0.137	0.996 (0.990 to 1.002)	0.220		
X-21845	Hypertension	13	1.005 (0.994 to 1.016)	0.425	0.998 (0.991 to 1.004)	0.532	0.997 (0.992 to 1.001)	0.164		
X-22509	Hypertension	10	1.002 (0.988 to 1.016)	0.765	1.000 (0.992 to 1.008)	0.974	1.001 (0.995 to 1.006)	0.806		
X-23644	Hypertension	8	1.010 (0.981 to 1.040)	0.528	1.002 (0.993 to 1.012)	0.639	1.000 (0.987 to 1.013)	0.987	1.005 (0.995 to 1.014)	0.390
X-22162	Hypertension	20	0.994 (0.983 to 1.005)	0.314	0.999 (0.995 to 1.003)	0.574	1.001 (0.992 to 1.010)	0.854	0.998 (0.995 to 1.001)	0.325

Exposure	Outcome	nSNP	MR Egger		Weighted median		Inverse variance weighted		MR_PRESSO	
			OR (95% CI)	pval	OR (95% CI)	pval	OR (95% CI)	pval	OR (95% CI)	pval
X-23587	Hypertension	8	1.002 (0.985 to 1.019)	0.806	0.994 (0.984 to 1.005)	0.281	0.995 (0.987 to 1.002)	0.176		
X-23593	Hypertension	12	1.004 (0.978 to 1.031)	0.762	0.999 (0.989 to 1.010)	0.887	1.007 (0.996 to 1.017)	0.209	1.003 (0.995 to 1.011)	0.480
X-23636	Hypertension	7	0.993 (0.965 to 1.022)	0.653	1.003 (0.991 to 1.015)	0.663	1.000 (0.991 to 1.010)	0.995		
X-23678	Hypertension	15	1.005 (0.989 to 1.021)	0.529	0.996 (0.988 to 1.003)	0.249	1.003 (0.995 to 1.010)	0.493	1.000 (0.994 to 1.007)	0.914
X-23680	Hypertension	12	1.008 (0.990 to 1.026)	0.423	1.002 (0.997 to 1.007)	0.487	1.002 (0.993 to 1.011)	0.703	1.004 (0.998 to 1.010)	0.190
X-23780	Hypertension	16	0.999 (0.991 to 1.008)	0.867	0.998 (0.992 to 1.005)	0.625	0.999 (0.994 to 1.003)	0.601		

Exposure	Outcome	nSNP	MR Egger		Weighted median		Inverse variance weighted		MR_PRESSO	
			OR (95% CI)	pval	OR (95% CI)	pval	OR (95% CI)	pval	OR (95% CI)	pval
X-23974	Hypertension	10	0.990 (0.973 to 1.008)	0.307	0.994 (0.986 to 1.003)	0.186	0.996 (0.990 to 1.003)	0.268		
X-23659	Hypertension	16	1.013 (1.001 to 1.025)	0.056	1.007 (1.000 to 1.014)	0.045	1.005 (0.999 to 1.011)	0.116	1.007 (1.001 to 1.012)	0.028
X-23665	Hypertension	7	1.004 (0.981 to 1.028)	0.733	1.007 (0.995 to 1.019)	0.263	1.007 (0.996 to 1.017)	0.208		
X-23782	Hypertension	12	0.994 (0.977 to 1.012)	0.523	1.001 (0.992 to 1.010)	0.877	1.001 (0.994 to 1.009)	0.775		
X-24295	Hypertension	13	0.978 (0.957 to 0.999)	0.060	0.996 (0.988 to 1.005)	0.379	1.000 (0.989 to 1.011)	0.999	0.993 (0.987 to 0.998)	0.028
X-24243	Hypertension	12	0.985 (0.967 to 1.003)	0.126	1.002 (0.993 to 1.011)	0.618	0.998 (0.989 to 1.008)	0.752	1.003 (0.995 to 1.012)	0.472

Exposure	Outcome	nSNP	MR Egger		Weighted median		Inverse variance weighted		MR_PRESSO	
			OR (95% CI)	pval	OR (95% CI)	pval	OR (95% CI)	pval	OR (95% CI)	pval
X-23739	Hypertension	14	0.998 (0.986 to 1.011)	0.767	0.999 (0.993 to 1.004)	0.672	0.995 (0.988 to 1.002)	0.195	0.997 (0.990 to 1.003)	0.326
X-23997	Hypertension	10	0.971 (0.953 to 0.989)	0.015	0.999 (0.990 to 1.009)	0.910	1.000 (0.991 to 1.010)	0.992		
X-24241	Hypertension	11	1.009 (0.986 to 1.031)	0.473	1.012 (1.004 to 1.020)	0.004	1.010 (1.002 to 1.019)	0.017	1.007 (1.000 to 1.014)	0.082
X-24328	Hypertension	23	0.995 (0.978 to 1.013)	0.579	1.003 (0.996 to 1.010)	0.451	1.003 (0.996 to 1.010)	0.423	0.999 (0.993 to 1.005)	0.835
X-24456	Hypertension	17	0.995 (0.983 to 1.008)	0.470	1.002 (0.996 to 1.008)	0.499	1.000 (0.996 to 1.005)	0.846		
X-24418	Hypertension	18	0.998 (0.987 to 1.010)	0.789	0.999 (0.995 to 1.003)	0.653	1.001 (0.995 to 1.006)	0.857	0.997 (0.994 to 1.000)	0.080

Exposure	Outcome	nSNP	MR Egger		Weighted median		Inverse variance weighted		MR_PRESSO	
			OR (95% CI)	pval	OR (95% CI)	pval	OR (95% CI)	pval	OR (95% CI)	pval
X-24344	Hypertension	10	0.998 (0.976 to 1.020)	0.844	1.000 (0.991 to 1.009)	0.984	1.005 (0.997 to 1.013)	0.251		
X-24556	Hypertension	13	0.979 (0.962 to 0.997)	0.043	1.000 (0.991 to 1.009)	0.976	1.000 (0.991 to 1.008)	0.964	1.004 (0.995 to 1.013)	0.416
X-24337	Hypertension	16	0.989 (0.972 to 1.007)	0.240	1.000 (0.994 to 1.006)	0.969	0.997 (0.988 to 1.005)	0.454	1.002 (0.995 to 1.008)	0.622
X-24546	Hypertension	18	0.998 (0.990 to 1.006)	0.619	0.998 (0.992 to 1.003)	0.457	0.997 (0.994 to 1.001)	0.165		
X-24306	Hypertension	13	0.983 (0.965 to 1.001)	0.099	0.998 (0.989 to 1.006)	0.588	0.997 (0.989 to 1.006)	0.530	1.000 (0.993 to 1.007)	0.963
X-24307	Hypertension	8	1.005 (0.970 to 1.041)	0.786	1.001 (0.989 to 1.012)	0.916	1.001 (0.991 to 1.011)	0.884		

Exposure	Outcome	nSNP	MR Egger		Weighted median		Inverse variance weighted		MR_PRESSO	
			OR (95% CI)	pval	OR (95% CI)	pval	OR (95% CI)	pval	OR (95% CI)	pval
X-24334	Hypertension	13	1.005 (0.973 to 1.039)	0.769	0.996 (0.988 to 1.004)	0.307	1.004 (0.992 to 1.016)	0.514	0.999 (0.992 to 1.005)	0.681
X-24518	Hypertension	15	0.999 (0.994 to 1.004)	0.742	0.997 (0.994 to 1.001)	0.117	0.997 (0.994 to 0.999)	0.014		
X-24494	Hypertension	11	0.994 (0.985 to 1.003)	0.223	0.997 (0.991 to 1.003)	0.360	1.000 (0.995 to 1.005)	0.985		
X-24544	Hypertension	21	0.994 (0.984 to 1.004)	0.232	0.996 (0.993 to 0.999)	0.019	0.998 (0.991 to 1.005)	0.509	0.997 (0.994 to 1.000)	0.037
X-24531	Hypertension	13	0.981 (0.965 to 0.998)	0.049	1.000 (0.992 to 1.009)	0.964	1.001 (0.992 to 1.010)	0.847	1.005 (0.999 to 1.012)	0.156
X-24811	Hypertension	14	1.018 (0.991 to 1.045)	0.224	0.997 (0.990 to 1.005)	0.446	0.998 (0.987 to 1.009)	0.712	0.998 (0.993 to 1.002)	0.348

Exposure	Outcome	nSNP	MR Egger		Weighted median		Inverse variance weighted		MR_PRESSO	
			OR (95% CI)	pval	OR (95% CI)	pval	OR (95% CI)	pval	OR (95% CI)	pval
X-24757	Hypertension	12	0.999 (0.970 to 1.028)	0.929	1.003 (0.996 to 1.011)	0.393	0.998 (0.986 to 1.009)	0.701	1.005 (0.999 to 1.011)	0.116
X-24978	Hypertension	12	0.997 (0.973 to 1.021)	0.806	1.001 (0.994 to 1.009)	0.717	0.997 (0.987 to 1.007)	0.604	0.999 (0.993 to 1.005)	0.779
X-24970	Hypertension	14	0.999 (0.981 to 1.016)	0.891	1.001 (0.993 to 1.009)	0.841	1.000 (0.994 to 1.006)	0.945		
X-24795	Hypertension	14	1.006 (0.985 to 1.029)	0.570	0.999 (0.993 to 1.006)	0.870	0.995 (0.987 to 1.003)	0.239	1.003 (0.999 to 1.006)	0.128
X-24951	Hypertension	11	1.011 (0.995 to 1.028)	0.207	1.005 (0.996 to 1.014)	0.278	1.002 (0.995 to 1.009)	0.569		
X-24728	Hypertension	16	1.015 (0.994 to 1.036)	0.194	1.002 (0.994 to 1.010)	0.692	0.998 (0.988 to 1.007)	0.625	1.002 (0.996 to 1.007)	0.577

Exposure	Outcome	nSNP	MR Egger		Weighted median		Inverse variance weighted		MR_PRESSO	
			OR (95% CI)	pval	OR (95% CI)	pval	OR (95% CI)	pval	OR (95% CI)	pval
X-24565	Hypertension	17	1.002 (0.991 to 1.012)	0.773	0.998 (0.993 to 1.003)	0.460	1.001 (0.997 to 1.006)	0.607		
X-24801	Hypertension	24	1.000 (0.989 to 1.011)	0.980	1.000 (0.994 to 1.006)	0.957	1.002 (0.997 to 1.008)	0.400	1.001 (0.996 to 1.006)	0.668
X-24736	Hypertension	20	0.995 (0.987 to 1.002)	0.183	1.001 (0.997 to 1.006)	0.564	1.004 (1.000 to 1.008)	0.031		
X-24585	Hypertension	6	1.000 (0.974 to 1.026)	0.988	0.994 (0.982 to 1.006)	0.296	0.994 (0.985 to 1.002)	0.147		
X-24588	Hypertension	24	0.995 (0.983 to 1.006)	0.383	0.998 (0.992 to 1.003)	0.402	0.999 (0.993 to 1.005)	0.697	0.998 (0.994 to 1.002)	0.423
X-25109	Hypertension	14	1.000 (0.984 to 1.017)	0.974	1.001 (0.994 to 1.009)	0.697	0.998 (0.992 to 1.005)	0.587	1.000 (0.995 to 1.005)	0.931

Exposure	Outcome	nSNP	MR Egger		Weighted median		Inverse variance weighted		MR_PRESSO	
			OR (95% CI)	pval	OR (95% CI)	pval	OR (95% CI)	pval	OR (95% CI)	pval
X-24947	Hypertension	19	1.003 (0.998 to 1.009)	0.251	1.003 (1.000 to 1.006)	0.069	1.004 (1.001 to 1.007)	0.019		
X-24949	Hypertension	17	0.995 (0.988 to 1.002)	0.210	0.998 (0.993 to 1.002)	0.334	1.001 (0.996 to 1.006)	0.704	1.001 (0.996 to 1.006)	0.719
X-24812	Hypertension	12	0.982 (0.949 to 1.016)	0.319	1.007 (0.997 to 1.016)	0.166	1.012 (0.999 to 1.024)	0.066	1.008 (1.001 to 1.016)	0.059
X-25217	Hypertension	10	1.016 (1.005 to 1.028)	0.025	1.000 (0.993 to 1.007)	0.951	0.997 (0.990 to 1.004)	0.364		
X-25828	Hypertension	5	1.000 (0.975 to 1.025)	0.990	1.007 (0.993 to 1.021)	0.343	1.006 (0.994 to 1.018)	0.321		
X-25371	Hypertension	20	1.002 (0.988 to 1.016)	0.766	0.995 (0.988 to 1.002)	0.196	0.995 (0.988 to 1.002)	0.193	1.002 (0.996 to 1.008)	0.542

Exposure	Outcome	nSNP	MR Egger		Weighted median		Inverse variance weighted		MR_PRESSO	
			OR (95% CI)	pval	OR (95% CI)	pval	OR (95% CI)	pval	OR (95% CI)	pval
X-25419	Hypertension	16	1.001 (0.985 to 1.018)	0.905	0.998 (0.991 to 1.006)	0.632	1.001 (0.994 to 1.007)	0.852		
X-25265	Hypertension	14	0.995 (0.971 to 1.021)	0.729	1.001 (0.991 to 1.010)	0.918	1.004 (0.991 to 1.016)	0.560	0.999 (0.989 to 1.009)	0.866
X-25172	Hypertension	9	1.025 (1.004 to 1.047)	0.051	1.004 (0.993 to 1.015)	0.467	1.003 (0.994 to 1.011)	0.532		
X-25957	Hypertension	8	1.028 (1.008 to 1.048)	0.032	1.001 (0.991 to 1.011)	0.785	1.002 (0.993 to 1.011)	0.734		
X-25422	Hypertension	16	1.009 (0.990 to 1.028)	0.362	1.001 (0.995 to 1.006)	0.851	0.996 (0.988 to 1.004)	0.316	0.999 (0.994 to 1.003)	0.568
X-25790	Hypertension	10	0.966 (0.927 to 1.006)	0.137	0.998 (0.988 to 1.009)	0.740	1.003 (0.991 to 1.016)	0.606	0.999 (0.989 to 1.010)	0.913

Exposure	Outcome	nSNP	MR Egger		Weighted median		Inverse variance weighted		MR_PRESSO	
			OR (95% CI)	pval	OR (95% CI)	pval	OR (95% CI)	pval	OR (95% CI)	pval
X-25420	Hypertension	10	0.971 (0.924 to 1.021)	0.281	0.991 (0.981 to 1.001)	0.072	1.009 (0.993 to 1.025)	0.276	0.999 (0.990 to 1.008)	0.825
X-25810	Hypertension	21	1.001 (0.990 to 1.012)	0.846	1.002 (0.995 to 1.009)	0.571	1.000 (0.995 to 1.005)	0.975		
X-25433	Hypertension	15	1.000 (0.985 to 1.015)	0.973	0.996 (0.991 to 1.002)	0.163	0.996 (0.988 to 1.003)	0.232	0.996 (0.992 to 1.001)	0.133
X-25343	Hypertension	8	0.999 (0.977 to 1.021)	0.928	1.005 (0.995 to 1.016)	0.313	1.008 (1.000 to 1.016)	0.043		
X-25271	Hypertension	12	1.005 (0.986 to 1.024)	0.651	1.003 (0.994 to 1.011)	0.578	1.003 (0.996 to 1.010)	0.377		
X-25520	Hypertension	8	0.999 (0.980 to 1.018)	0.904	0.994 (0.984 to 1.004)	0.267	0.997 (0.989 to 1.005)	0.443		

Exposure	Outcome	nSNP	MR Egger		Weighted median		Inverse variance weighted		MR_PRESSO	
			OR (95% CI)	pval	OR (95% CI)	pval	OR (95% CI)	pval	OR (95% CI)	pval
X-25519	Hypertension	9	1.023 (0.983 to 1.064)	0.296	1.001 (0.990 to 1.013)	0.812	1.010 (0.992 to 1.029)	0.276	1.007 (0.996 to 1.017)	0.248
X-26111	Hypertension	14	0.995 (0.981 to 1.011)	0.565	0.998 (0.991 to 1.004)	0.486	0.996 (0.989 to 1.004)	0.331	1.000 (0.996 to 1.005)	0.899
X-26109	Hypertension	17	1.006 (1.002 to 1.010)	0.014	1.003 (1.000 to 1.006)	0.026	1.001 (0.998 to 1.005)	0.460	1.000 (0.994 to 1.005)	0.861
X-26054	Hypertension	22	0.998 (0.986 to 1.010)	0.749	0.998 (0.994 to 1.002)	0.301	0.995 (0.988 to 1.002)	0.140	0.999 (0.995 to 1.002)	0.519
Carnitine C4	Hypertension	24	0.996 (0.989 to 1.003)	0.278	0.998 (0.994 to 1.001)	0.118	1.003 (0.998 to 1.008)	0.245	1.005 (0.999 to 1.011)	0.147
Androsterone sulfate	Hypertension	24	1.003 (0.997 to 1.009)	0.287	1.004 (1.001 to 1.007)	0.004	1.004 (0.999 to 1.009)	0.089	1.004 (1.001 to 1.007)	0.029

Exposure	Outcome	nSNP	MR Egger		Weighted median		Inverse variance weighted		MR_PRESSO	
			OR (95% CI)	pval	OR (95% CI)	pval	OR (95% CI)	pval	OR (95% CI)	pval
N-acetyltyrosine	Hypertension	21	1.001 (0.996 to 1.006)	0.579	1.003 (1.000 to 1.005)	0.048	1.002 (0.999 to 1.005)	0.255	1.002 (0.999 to 1.005)	0.178
N-acetyl-L-glutamine	Hypertension	13	1.002 (0.998 to 1.005)	0.441	1.003 (1.000 to 1.005)	0.019	1.003 (1.000 to 1.007)	0.031		
N-acetylphenylalanine	Hypertension	12	1.003 (0.999 to 1.008)	0.184	1.004 (1.001 to 1.007)	0.012	1.004 (1.001 to 1.007)	0.019		
N-acetylasparagine	Hypertension	12	1.001 (0.998 to 1.004)	0.593	1.002 (1.000 to 1.004)	0.016	1.003 (1.000 to 1.006)	0.054		
N-acetylarginine	Hypertension	15	1.001 (0.998 to 1.004)	0.586	1.003 (1.001 to 1.005)	0.012	1.004 (1.000 to 1.007)	0.024		
5-acetylamino-6-formylamino-3-methyluracil	Hypertension	16	1.006 (1.001 to 1.010)	0.044	1.003 (1.001 to 1.005)	0.010	1.001 (0.997 to 1.006)	0.524		

Exposure	Outcome	nSNP	MR Egger		Weighted median		Inverse variance weighted		MR_PRESSO	
			OR (95% CI)	pval	OR (95% CI)	pval	OR (95% CI)	pval	OR (95% CI)	pval
Orotidine	Hypertension	18	0.998 (0.986 to 1.010)	0.730	0.997 (0.994 to 1.000)	0.080	1.000 (0.990 to 1.010)	0.954	1.001 (0.994 to 1.009)	0.698
3-methylcytidine	Hypertension	9	1.000 (0.995 to 1.005)	0.907	0.999 (0.997 to 1.002)	0.592	0.999 (0.995 to 1.002)	0.469		
N-acetylcitrulline	Hypertension	12	1.000 (0.997 to 1.003)	0.959	1.003 (1.001 to 1.004)	0.011	1.003 (1.000 to 1.007)	0.067		
N-acetyl-aspartyl-glutamate (naag)	Hypertension	9	1.001 (0.990 to 1.012)	0.896	0.998 (0.993 to 1.003)	0.402	0.999 (0.992 to 1.006)	0.777	0.994 (0.988 to 1.000)	0.113
1-stearoyl-2-arachidonoyl-gpc (18:0/20:4)	Hypertension	18	0.998 (0.992 to 1.004)	0.599	0.999 (0.996 to 1.002)	0.468	0.998 (0.994 to 1.002)	0.333	0.998 (0.995 to 1.001)	0.221
Bilirubin (E,E)	Hypertension	15	1.001 (0.994 to 1.007)	0.858	0.998 (0.995 to 1.002)	0.378	0.998 (0.994 to 1.002)	0.426		

Exposure	Outcome	nSNP	MR Egger		Weighted median		Inverse variance weighted		MR_PRESSO	
			OR (95% CI)	pval	OR (95% CI)	pval	OR (95% CI)	pval	OR (95% CI)	pval
Bilirubin (E,Z or Z,E)	Hypertension	13	0.996 (0.990 to 1.003)	0.309	0.998 (0.995 to 1.002)	0.290	0.998 (0.994 to 1.002)	0.295		
Androsterone glucuronide	Hypertension	21	0.997 (0.991 to 1.004)	0.470	0.998 (0.995 to 1.001)	0.248	0.999 (0.994 to 1.004)	0.622	0.998 (0.995 to 1.002)	0.445
N6-methyllysine	Hypertension	16	0.999 (0.997 to 1.001)	0.557	0.999 (0.998 to 1.001)	0.261	0.999 (0.997 to 1.001)	0.226		
N-acetyl-1-methylhistidine	Hypertension	24	1.003 (0.998 to 1.008)	0.278	1.003 (1.000 to 1.005)	0.052	1.001 (0.998 to 1.005)	0.500	1.000 (0.994 to 1.005)	0.928
N-delta-acetylornithine	Hypertension	13	0.998 (0.995 to 1.001)	0.149	0.997 (0.995 to 1.000)	0.018	0.997 (0.995 to 0.999)	0.014		
1-palmitoyl-2-arachidonoyl-gpc (16:0/20:4n6)	Hypertension	15	0.999 (0.993 to 1.006)	0.841	0.999 (0.996 to 1.002)	0.390	0.998 (0.994 to 1.003)	0.431	0.998 (0.995 to 1.001)	0.302

Exposure	Outcome	nSNP	MR Egger		Weighted median		Inverse variance weighted		MR_PRESSO	
			OR (95% CI)	pval	OR (95% CI)	pval	OR (95% CI)	pval	OR (95% CI)	pval
Glycochenodeoxycholate glucuronide (1)	Hypertension	13	0.999 (0.995 to 1.003)	0.721	0.998 (0.996 to 1.001)	0.217	0.998 (0.995 to 1.001)	0.131		
2'-o-methylcytidine	Hypertension	11	0.998 (0.994 to 1.003)	0.528	0.998 (0.995 to 1.001)	0.114	0.998 (0.995 to 1.001)	0.182		
2'-o-methyluridine	Hypertension	12	0.997 (0.994 to 1.000)	0.095	0.998 (0.996 to 1.001)	0.122	0.999 (0.997 to 1.001)	0.345		
N6,N6-dimethyllysine	Hypertension	13	0.999 (0.996 to 1.002)	0.672	0.999 (0.997 to 1.001)	0.376	0.999 (0.997 to 1.001)	0.276		
N2-acetyl,N6,N6-dimethyllysine	Hypertension	16	1.001 (0.997 to 1.004)	0.692	1.000 (0.998 to 1.002)	0.866	1.001 (0.999 to 1.003)	0.462		
N2-acetyl,N6-methyllysine	Hypertension	17	1.000 (0.997 to 1.003)	0.898	1.000 (0.998 to 1.002)	0.934	1.001 (0.999 to 1.003)	0.265		

Exposure	Outcome	nSNP	MR Egger		Weighted median		Inverse variance weighted		MR_PRESSO	
			OR (95% CI)	pval	OR (95% CI)	pval	OR (95% CI)	pval	OR (95% CI)	pval
Deoxycholic acid glucuronide	Hypertension	16	1.004 (0.999 to 1.009)	0.155	1.005 (1.001 to 1.008)	0.014	1.003 (1.000 to 1.006)	0.022		
Decadienedioic acid (C10:2-DC)	Hypertension	16	1.002 (0.995 to 1.009)	0.579	1.001 (0.998 to 1.005)	0.424	0.998 (0.993 to 1.002)	0.316	0.996 (0.992 to 1.000)	0.083
Bilirubin degradation product, C17H18N2O4 (1)	Hypertension	14	0.998 (0.986 to 1.010)	0.721	0.997 (0.994 to 1.001)	0.125	0.998 (0.990 to 1.006)	0.664	0.997 (0.995 to 1.000)	0.082
Bilirubin degradation product, C17H18N2O4 (2)	Hypertension	13	0.997 (0.983 to 1.011)	0.693	0.998 (0.995 to 1.001)	0.271	0.999 (0.990 to 1.007)	0.752	0.997 (0.994 to 1.000)	0.061
Bilirubin degradation product, C17H18N2O4 (3)	Hypertension	12	0.998 (0.986 to 1.011)	0.817	0.997 (0.994 to 1.000)	0.077	0.996 (0.988 to 1.005)	0.371	0.996 (0.992 to 0.999)	0.032
Bilirubin (z,z)	Hypertension	17	0.999 (0.994 to 1.004)	0.746	0.998 (0.995 to 1.001)	0.238	0.998 (0.995 to 1.001)	0.152		

Exposure	Outcome	nSNP	MR Egger		Weighted median		Inverse variance weighted		MR_PRESSO	
			OR (95% CI)	pval	OR (95% CI)	pval	OR (95% CI)	pval	OR (95% CI)	pval
Ethylmalonate	Hypertension	22	0.997 (0.993 to 1.001)	0.178	0.998 (0.995 to 1.001)	0.149	0.999 (0.996 to 1.002)	0.466		
Biliverdin	Hypertension	17	0.998 (0.994 to 1.003)	0.406	0.997 (0.994 to 1.001)	0.130	0.996 (0.993 to 1.000)	0.024		
Glycine	Hypertension	13	0.991 (0.985 to 0.996)	0.007	0.991 (0.988 to 0.994)	4.93e-08	0.991 (0.988 to 0.995)	5.15e-06		
X-12112	Hypertension	20	1.000 (0.998 to 1.002)	0.946	0.999 (0.997 to 1.001)	0.322	0.999 (0.997 to 1.000)	0.157		
X-12798	Hypertension	18	0.998 (0.992 to 1.004)	0.509	0.997 (0.994 to 1.000)	0.084	0.997 (0.993 to 1.000)	0.084	0.997 (0.994 to 1.001)	0.146
X-13431	Hypertension	16	1.003 (0.988 to 1.018)	0.701	1.002 (0.995 to 1.008)	0.632	0.998 (0.992 to 1.003)	0.428		

Exposure	Outcome	nSNP	MR Egger		Weighted median		Inverse variance weighted		MR_PRESSO	
			OR (95% CI)	pval	OR (95% CI)	pval	OR (95% CI)	pval	OR (95% CI)	pval
X-19141	Hypertension	16	0.999 (0.989 to 1.008)	0.765	1.001 (0.996 to 1.006)	0.687	1.001 (0.996 to 1.006)	0.759	1.001 (0.995 to 1.008)	0.731
Glutamine	Hypertension	7	0.956 (0.809 to 1.129)	0.616	0.956 (0.865 to 1.056)	0.373	0.903 (0.826 to 0.987)	0.024		
Tryptophan	Hypertension	109	1.005 (0.704 to 1.434)	0.978	1.061 (1.006 to 1.120)	0.029	1.026 (0.975 to 1.079)	0.325	1.035 (0.988 to 1.085)	0.152
Histidine	Hypertension	5	0.703 (0.300 to 1.649)	0.477	0.966 (0.822 to 1.136)	0.678	1.123 (0.821 to 1.538)	0.468	1.064 (0.867 to 1.307)	0.611
Leucine	Hypertension	122	0.999 (0.883 to 1.129)	0.981	1.000 (0.937 to 1.066)	0.989	1.013 (0.967 to 1.062)	0.588	1.011 (0.969 to 1.055)	0.610
Cholesterol	Hypertension	30	1.064 (0.886 to 1.278)	0.514	1.023 (0.962 to 1.088)	0.463	1.058 (0.985 to 1.138)	0.124	1.027 (0.986 to 1.070)	0.208

Exposure	Outcome	nSNP	MR Egger		Weighted median		Inverse variance weighted		MR_PRESSO	
			OR (95% CI)	pval	OR (95% CI)	pval	OR (95% CI)	pval	OR (95% CI)	pval
Phenylalanine	Hypertension	4	0.884 (0.694 to 1.127)	0.424	0.972 (0.848 to 1.114)	0.680	1.055 (0.897 to 1.241)	0.516		
Creatinine	Hypertension	14	0.998 (0.825 to 1.206)	0.983	0.988 (0.893 to 1.094)	0.822	0.991 (0.920 to 1.069)	0.822		
Lactate	Hypertension	5	0.863 (0.559 to 1.333)	0.554	0.969 (0.885 to 1.061)	0.492	0.997 (0.876 to 1.135)	0.965	0.975 (0.866 to 1.097)	0.711
3-hydroxybutyrate (BHBA)	Hypertension	5	0.997 (0.980 to 1.014)	0.717	1.001 (0.990 to 1.012)	0.883	0.999 (0.990 to 1.008)	0.839		
Arabinose	Hypertension	4	0.976 (0.937 to 1.017)	0.371	0.992 (0.975 to 1.009)	0.334	0.995 (0.969 to 1.022)	0.706		
Fructose	Hypertension	2					0.982 (0.921 to 1.046)	0.573		

Exposure	Outcome	nSNP	MR Egger		Weighted median		Inverse variance weighted		MR_PRESSO	
			OR (95% CI)	pval	OR (95% CI)	pval	OR (95% CI)	pval	OR (95% CI)	pval
Mannose	Hypertension	8	0.887 (0.821 to 0.958)	0.022	0.967 (0.933 to 1.003)	0.072	0.978 (0.925 to 1.034)	0.430		
Pyruvate	Hypertension	11	1.032 (0.920 to 1.156)	0.605	0.994 (0.957 to 1.033)	0.765	0.991 (0.951 to 1.032)	0.658	1.003 (0.971 to 1.035)	0.877
Uridine	Hypertension	15	0.909 (0.707 to 1.169)	0.471	0.926 (0.858 to 0.999)	0.046	0.937 (0.873 to 1.006)	0.072	0.918 (0.870 to 0.969)	0.008
Linoleate (18:2n6)	Hypertension	9	0.993 (0.816 to 1.208)	0.943	1.002 (0.943 to 1.064)	0.949	1.011 (0.965 to 1.059)	0.649		
Allantoin	Hypertension	9	1.023 (0.990 to 1.057)	0.219	1.010 (0.983 to 1.038)	0.472	1.009 (0.988 to 1.029)	0.406		
Arachidonate (20:4n6)	Hypertension	11	0.966 (0.902 to 1.035)	0.355	0.987 (0.954 to 1.021)	0.437	0.999 (0.957 to 1.044)	0.974	0.990 (0.957 to 1.024)	0.572

Exposure	Outcome	nSNP	MR Egger		Weighted median		Inverse variance weighted		MR_PRESSO	
			**OR (95% CI)**	**pval**	**OR (95% CI)**	**pval**	**OR (95% CI)**	**pval**	**OR (95% CI)**	**pval**
Deoxycholate	Hypertension	6	1.012 (0.858 to 1.194)	0.893	0.981 (0.953 to 1.009)	0.181	0.989 (0.965 to 1.013)	0.354		
Margarate (17:0)	Hypertension	6	1.125 (0.753 to 1.680)	0.596	0.985 (0.924 to 1.050)	0.641	1.025 (0.926 to 1.134)	0.637	0.972 (0.925 to 1.022)	0.353
Inosine	Hypertension	5	1.027 (0.973 to 1.083)	0.404	1.002 (0.988 to 1.016)	0.749	1.002 (0.991 to 1.013)	0.737		
Isoleucine	Hypertension	11	0.916 (0.679 to 1.237)	0.582	1.025 (0.925 to 1.134)	0.640	0.991 (0.891 to 1.101)	0.861	1.014 (0.922 to 1.116)	0.779
Threonine	Hypertension	13	0.914 (0.681 to 1.228)	0.563	1.022 (0.951 to 1.099)	0.547	1.030 (0.941 to 1.128)	0.520	1.021 (0.958 to 1.087)	0.539
Tyrosine	Hypertension	21	1.008 (0.810 to 1.255)	0.941	0.966 (0.904 to 1.031)	0.295	0.990 (0.939 to 1.044)	0.709		

Exposure	Outcome	nSNP	MR Egger		Weighted median		Inverse variance weighted		MR_PRESSO	
			OR (95% CI)	pval	OR (95% CI)	pval	OR (95% CI)	pval	OR (95% CI)	pval
Lysine	Hypertension	15	0.916 (0.669 to 1.253)	0.592	0.959 (0.881 to 1.045)	0.339	0.975 (0.917 to 1.037)	0.429		
Methionine	Hypertension	13	0.905 (0.720 to 1.137)	0.411	0.976 (0.880 to 1.083)	0.651	0.995 (0.923 to 1.072)	0.890		
Malate	Hypertension	10	0.915 (0.660 to 1.268)	0.607	0.971 (0.920 to 1.026)	0.293	0.964 (0.925 to 1.005)	0.086		
Palmitate (16:0)	Hypertension	19	1.002 (0.824 to 1.218)	0.988	0.973 (0.918 to 1.031)	0.351	1.031 (0.957 to 1.111)	0.418	0.977 (0.941 to 1.013)	0.226
Nonadecanoate (19:0)	Hypertension	7	1.168 (0.801 to 1.702)	0.456	1.057 (0.987 to 1.132)	0.110	1.077 (0.977 to 1.187)	0.136	1.022 (0.978 to 1.068)	0.378
Stearate (18:0)	Hypertension	26	1.061 (0.924 to 1.217)	0.410	0.974 (0.926 to 1.025)	0.312	1.005 (0.953 to 1.059)	0.857	0.981 (0.948 to 1.015)	0.273

Exposure	Outcome	nSNP	MR Egger		Weighted median		Inverse variance weighted		MR_PRESSO	
			OR (95% CI)	pval	OR (95% CI)	pval	OR (95% CI)	pval	OR (95% CI)	pval
Oleate (18:1n9)	Hypertension	11	1.069 (0.785 to 1.457)	0.682	0.957 (0.899 to 1.019)	0.174	0.963 (0.916 to 1.012)	0.139		
Pentadecanoate (15:0)	Hypertension	10	0.874 (0.669 to 1.141)	0.350	1.018 (0.962 to 1.077)	0.543	0.965 (0.866 to 1.076)	0.521	1.014 (0.994 to 1.036)	0.216
Myristate (14:0)	Hypertension	12	1.020 (0.882 to 1.180)	0.792	1.021 (0.971 to 1.074)	0.416	1.056 (0.980 to 1.138)	0.151	1.020 (0.964 to 1.078)	0.512
Pipecolate	Hypertension	8	1.069 (0.992 to 1.153)	0.131	0.956 (0.916 to 0.998)	0.038	0.976 (0.932 to 1.021)	0.284	0.956 (0.915 to 0.998)	0.088
Ornithine	Hypertension	6	0.958 (0.806 to 1.139)	0.653	0.971 (0.904 to 1.042)	0.412	0.971 (0.922 to 1.023)	0.272		
5-oxoproline	Hypertension	16	1.084 (0.995 to 1.181)	0.087	1.071 (1.026 to 1.119)	0.002	1.058 (1.002 to 1.118)	0.042	1.069 (1.016 to 1.125)	0.022

Exposure	Outcome	nSNP	MR Egger		Weighted median		Inverse variance weighted		MR_PRESSO	
			OR (95% CI)	pval	OR (95% CI)	pval	OR (95% CI)	pval	OR (95% CI)	pval
Pantothenate	Hypertension	14	1.037 (0.914 to 1.177)	0.579	1.011 (0.959 to 1.066)	0.678	0.999 (0.935 to 1.069)	0.986	1.020 (0.976 to 1.065)	0.401
4-acetamidobutanoate	Hypertension	16	1.068 (0.906 to 1.260)	0.445	1.072 (1.008 to 1.141)	0.027	1.023 (0.950 to 1.102)	0.548	0.999 (0.945 to 1.055)	0.959
Alpha-tocopherol	Hypertension	5	1.064 (0.893 to 1.268)	0.538	0.998 (0.942 to 1.057)	0.943	1.015 (0.962 to 1.071)	0.583		
Citrate	Hypertension	20	1.042 (0.941 to 1.153)	0.443	1.056 (1.005 to 1.109)	0.030	1.026 (0.988 to 1.066)	0.186		
Glycerate	Hypertension	9	1.072 (0.872 to 1.318)	0.530	1.005 (0.937 to 1.078)	0.891	1.008 (0.946 to 1.074)	0.802		
Guanosine	Hypertension	8	0.999 (0.956 to 1.045)	0.981	1.003 (0.984 to 1.022)	0.779	1.003 (0.987 to 1.019)	0.724		

Exposure	Outcome	nSNP	MR Egger		Weighted median		Inverse variance weighted		MR_PRESSO	
			OR (95% CI)	pval	OR (95% CI)	pval	OR (95% CI)	pval	OR (95% CI)	pval
N-acetylalanine	Hypertension	12	4.633 (0.759 to 28.283)	0.128	1.103 (0.982 to 1.238)	0.099	1.058 (0.941 to 1.191)	0.346		
Urate	Hypertension	9	1.124 (1.035 to 1.221)	0.027	1.046 (0.992 to 1.104)	0.094	1.009 (0.950 to 1.072)	0.774		
Ursodeoxycholate	Hypertension	4	1.223 (1.045 to 1.432)	0.130	1.022 (0.987 to 1.059)	0.224	1.003 (0.948 to 1.060)	0.925	1.019 (0.970 to 1.070)	0.598
Arginine	Hypertension	11	1.095 (0.864 to 1.386)	0.473	1.017 (0.965 to 1.072)	0.534	1.030 (0.951 to 1.116)	0.469	1.010 (0.975 to 1.047)	0.596
Ascorbate (Vitamin C)	Hypertension	10	0.983 (0.940 to 1.028)	0.473	0.999 (0.986 to 1.012)	0.862	0.997 (0.984 to 1.009)	0.593		
Heptanoate (7:0)	Hypertension	19	1.030 (0.884 to 1.200)	0.710	0.979 (0.924 to 1.038)	0.477	0.979 (0.929 to 1.031)	0.417		

Exposure	Outcome	nSNP	MR Egger		Weighted median		Inverse variance weighted		MR_PRESSO	
			OR (95% CI)	pval	OR (95% CI)	pval	OR (95% CI)	pval	OR (95% CI)	pval
Laurate (12:0)	Hypertension	27	1.018 (0.884 to 1.173)	0.806	1.009 (0.962 to 1.058)	0.712	1.007 (0.960 to 1.057)	0.762	1.004 (0.971 to 1.038)	0.802
Valine	Hypertension	6	0.960 (0.863 to 1.068)	0.496	0.999 (0.926 to 1.079)	0.985	1.032 (0.932 to 1.142)	0.544		
Urea	Hypertension	8	1.084 (0.903 to 1.302)	0.418	1.040 (0.971 to 1.114)	0.262	1.018 (0.950 to 1.092)	0.612		
Cortisol	Hypertension	5	1.008 (0.912 to 1.115)	0.882	1.002 (0.952 to 1.055)	0.937	0.994 (0.948 to 1.042)	0.798		
Cortisone	Hypertension	24	1.014 (0.942 to 1.092)	0.714	1.021 (0.971 to 1.073)	0.425	0.987 (0.950 to 1.027)	0.526		
Proline	Hypertension	23	1.016 (0.963 to 1.072)	0.568	1.016 (0.979 to 1.055)	0.401	0.999 (0.967 to 1.033)	0.972	1.010 (0.980 to 1.042)	0.520

Exposure	Outcome	nSNP	MR Egger		Weighted median		Inverse variance weighted		MR_PRESSO	
			OR (95% CI)	pval	OR (95% CI)	pval	OR (95% CI)	pval	OR (95% CI)	pval
Citrulline	Hypertension	23	0.971 (0.834 to 1.131)	0.711	1.026 (0.964 to 1.092)	0.417	1.007 (0.952 to 1.066)	0.801	1.006 (0.957 to 1.057)	0.813
Biliverdin	Hypertension	12	1.000 (0.967 to 1.035)	0.989	0.995 (0.980 to 1.009)	0.451	0.995 (0.973 to 1.017)	0.641	1.000 (0.986 to 1.015)	0.953
Serotonin (5HT)	Hypertension	11	0.970 (0.857 to 1.097)	0.637	1.022 (0.976 to 1.071)	0.350	1.029 (0.980 to 1.082)	0.253	1.016 (0.970 to 1.064)	0.514
Gamma-glutamylglutamine	Hypertension	16	0.954 (0.829 to 1.098)	0.522	0.988 (0.934 to 1.046)	0.679	0.973 (0.912 to 1.039)	0.415	0.992 (0.946 to 1.041)	0.752
Gamma-glutamyltyrosine	Hypertension	23	1.024 (0.802 to 1.306)	0.853	1.064 (1.007 to 1.124)	0.026	1.032 (0.964 to 1.104)	0.363	1.011 (0.970 to 1.055)	0.604
Hypoxanthine	Hypertension	13	0.988 (0.876 to 1.114)	0.849	1.010 (0.967 to 1.055)	0.657	1.002 (0.960 to 1.045)	0.933		

Exposure	Outcome	nSNP	MR Egger		Weighted median		Inverse variance weighted		MR_PRESSO	
			OR (95% CI)	pval	OR (95% CI)	pval	OR (95% CI)	pval	OR (95% CI)	pval
Betaine	Hypertension	14	1.133 (0.936 to 1.371)	0.223	1.024 (0.967 to 1.084)	0.416	1.066 (0.999 to 1.136)	0.052	1.033 (0.975 to 1.095)	0.291
Xanthine	Hypertension	3	1.003 (0.864 to 1.165)	0.972	0.946 (0.892 to 1.004)	0.067	0.949 (0.900 to 1.000)	0.052		
Phosphate	Hypertension	2					0.982 (0.911 to 1.060)	0.646		
3-methoxytyrosine	Hypertension	9	0.976 (0.766 to 1.242)	0.846	0.967 (0.917 to 1.020)	0.218	0.982 (0.925 to 1.043)	0.557	0.968 (0.920 to 1.018)	0.246
Pelargonate (9:0)	Hypertension	20	1.078 (0.869 to 1.337)	0.504	1.005 (0.949 to 1.065)	0.856	0.992 (0.943 to 1.044)	0.770		
Undecanoate (11:0)	Hypertension	21	0.931 (0.844 to 1.027)	0.172	0.985 (0.925 to 1.049)	0.640	0.949 (0.901 to 0.999)	0.044	0.958 (0.922 to 0.996)	0.046

Exposure	Outcome	nSNP	MR Egger		Weighted median		Inverse variance weighted		MR_PRESSO	
			OR (95% CI)	pval	OR (95% CI)	pval	OR (95% CI)	pval	OR (95% CI)	pval
Beta-hydroxyisovalerate	Hypertension	17	0.987 (0.896 to 1.087)	0.792	1.008 (0.964 to 1.053)	0.735	1.049 (1.008 to 1.091)	0.019	1.062 (1.027 to 1.099)	0.003
Taurodeoxycholate	Hypertension	8	0.984 (0.947 to 1.022)	0.429	1.010 (0.994 to 1.027)	0.210	1.008 (0.994 to 1.022)	0.258		
X-02973	Hypertension	16	0.913 (0.609 to 1.369)	0.666	1.034 (0.948 to 1.128)	0.449	0.999 (0.917 to 1.088)	0.977	1.030 (0.961 to 1.103)	0.424
X-03003	Hypertension	3	0.022 (0.001 to 0.438)	0.242	0.969 (0.858 to 1.095)	0.613	1.052 (0.885 to 1.250)	0.568		
X-03088	Hypertension	7	1.005 (0.937 to 1.078)	0.896	1.010 (0.973 to 1.048)	0.610	1.017 (0.970 to 1.066)	0.477		
X-03094	Hypertension	17	1.072 (0.938 to 1.226)	0.322	0.955 (0.909 to 1.005)	0.074	0.987 (0.934 to 1.043)	0.647	0.936 (0.908 to 0.965)	7.7e-04

Exposure	Outcome	nSNP	MR Egger		Weighted median		Inverse variance weighted		MR_PRESSO	
			OR (95% CI)	pval	OR (95% CI)	pval	OR (95% CI)	pval	OR (95% CI)	pval
Glycerol	Hypertension	12	1.046 (0.972 to 1.126)	0.260	1.053 (1.001 to 1.107)	0.045	1.040 (1.002 to 1.081)	0.041		
Kynurenine	Hypertension	26	1.038 (0.858 to 1.257)	0.702	1.005 (0.962 to 1.048)	0.837	1.064 (0.966 to 1.173)	0.206	1.008 (0.974 to 1.044)	0.636
Mannitol	Hypertension	6	0.978 (0.919 to 1.041)	0.522	1.002 (0.985 to 1.018)	0.836	0.998 (0.974 to 1.022)	0.865	0.999 (0.982 to 1.016)	0.905
Glycerol 3-phosphate (G3P)	Hypertension	6	0.953 (0.834 to 1.089)	0.519	0.959 (0.896 to 1.027)	0.235	0.981 (0.927 to 1.038)	0.505		
Acetylphosphate	Hypertension	11	0.869 (0.477 to 1.583)	0.658	1.013 (0.921 to 1.114)	0.790	1.086 (0.970 to 1.217)	0.152	1.041 (0.960 to 1.129)	0.354
Carnitine	Hypertension	128	1.076 (0.939 to 1.234)	0.296	1.045 (0.983 to 1.110)	0.160	1.040 (0.992 to 1.090)	0.101	1.016 (0.978 to 1.054)	0.416

Exposure	Outcome	nSNP	MR Egger		Weighted median		Inverse variance weighted		MR_PRESSO	
			OR (95% CI)	pval	OR (95% CI)	pval	OR (95% CI)	pval	OR (95% CI)	pval
Choline	Hypertension	15	0.798 (0.503 to 1.266)	0.356	0.946 (0.848 to 1.056)	0.323	1.042 (0.907 to 1.198)	0.559	1.019 (0.923 to 1.125)	0.715
N-acetylornithine	Hypertension	10	0.982 (0.963 to 1.003)	0.125	0.984 (0.972 to 0.996)	0.010	0.984 (0.971 to 0.998)	0.024		
N1-methyladenosine	Hypertension	3	1.044 (0.421 to 2.589)	0.941	1.029 (0.878 to 1.207)	0.723	1.045 (0.889 to 1.229)	0.590		
3-methyl-2-oxovalerate	Hypertension	21	1.064 (0.950 to 1.193)	0.296	0.975 (0.923 to 1.030)	0.371	1.008 (0.965 to 1.054)	0.718	0.996 (0.959 to 1.035)	0.840
3-methylhistidine	Hypertension	4	0.962 (0.835 to 1.108)	0.642	1.008 (0.986 to 1.029)	0.495	1.019 (0.972 to 1.068)	0.440	1.010 (1.001 to 1.020)	0.286
3-phenylpropionate (hydrocinnamate)	Hypertension	4	0.979 (0.930 to 1.031)	0.504	0.980 (0.952 to 1.009)	0.177	0.980 (0.954 to 1.007)	0.142		

Exposure	Outcome	nSNP	MR Egger		Weighted median		Inverse variance weighted		MR_PRESSO	
			OR (95% CI)	pval	OR (95% CI)	pval	OR (95% CI)	pval	OR (95% CI)	pval
Phenylacetate	Hypertension	4	1.063 (0.880 to 1.285)	0.591	1.016 (0.959 to 1.076)	0.589	1.040 (0.989 to 1.093)	0.126		
Glycerophosphorylcholine (GPC)	Hypertension	8	0.982 (0.945 to 1.020)	0.374	0.986 (0.966 to 1.008)	0.209	0.986 (0.959 to 1.013)	0.307		
Aspartate	Hypertension	3	0.964 (0.874 to 1.063)	0.595	0.982 (0.934 to 1.031)	0.459	0.980 (0.942 to 1.019)	0.311		
X-04357	Hypertension	2					1.019 (0.979 to 1.061)	0.362		
X-04494	Hypertension	8	0.992 (0.837 to 1.176)	0.930	1.030 (0.967 to 1.096)	0.355	1.041 (0.989 to 1.095)	0.122		
X-04495	Hypertension	7	1.025 (0.934 to 1.125)	0.627	1.010 (0.969 to 1.054)	0.631	1.003 (0.972 to 1.035)	0.844		

Exposure	Outcome	nSNP	MR Egger		Weighted median		Inverse variance weighted		MR_PRESSO	
			OR (95% CI)	pval	OR (95% CI)	pval	OR (95% CI)	pval	OR (95% CI)	pval
X-04498	Hypertension	14	0.981 (0.895 to 1.076)	0.697	0.961 (0.917 to 1.008)	0.102	0.986 (0.948 to 1.025)	0.475		
X-04499--3,4-dihydroxybutyrate	Hypertension	12	1.076 (0.898 to 1.290)	0.445	1.047 (0.992 to 1.105)	0.098	1.015 (0.974 to 1.058)	0.482		
X-04500	Hypertension	5	1.018 (0.992 to 1.045)	0.272	1.013 (0.999 to 1.026)	0.072	1.012 (1.000 to 1.024)	0.052		
Dihomo-linoleate (20:2n6)	Hypertension	3	0.736 (0.561 to 0.966)	0.271	0.977 (0.899 to 1.062)	0.587	1.014 (0.899 to 1.143)	0.826		
X-18601	Hypertension	13	1.031 (0.956 to 1.112)	0.445	1.012 (0.990 to 1.035)	0.276	1.000 (0.961 to 1.040)	0.995	1.014 (0.996 to 1.032)	0.158
2-hydroxystearate	Hypertension	22	1.060 (0.953 to 1.179)	0.297	0.987 (0.935 to 1.043)	0.652	1.008 (0.961 to 1.057)	0.751		

Exposure	Outcome	nSNP	MR Egger		Weighted median		Inverse variance weighted		MR_PRESSO	
			OR (95% CI)	pval	OR (95% CI)	pval	OR (95% CI)	pval	OR (95% CI)	pval
X-05426	Hypertension	7	0.959 (0.835 to 1.102)	0.583	0.972 (0.940 to 1.006)	0.102	0.966 (0.928 to 1.005)	0.087	0.966 (0.942 to 0.991)	0.055
Indolelactate	Hypertension	11	1.010 (0.818 to 1.246)	0.929	0.989 (0.943 to 1.038)	0.663	1.015 (0.928 to 1.111)	0.744	0.985 (0.945 to 1.027)	0.500
Glycylvaline	Hypertension	3	1.073 (0.932 to 1.235)	0.505	0.993 (0.958 to 1.029)	0.696	1.009 (0.975 to 1.045)	0.596		
Gamma-glutamylleucine	Hypertension	17	0.986 (0.862 to 1.127)	0.838	1.034 (0.976 to 1.095)	0.257	1.044 (0.978 to 1.114)	0.194	1.019 (0.974 to 1.066)	0.428
Eicosapentaenoate (EPA; 20:5n3)	Hypertension	7	0.948 (0.873 to 1.030)	0.262	0.985 (0.948 to 1.024)	0.454	0.999 (0.967 to 1.032)	0.959		
Estrone 3-sulfate	Hypertension	8	0.999 (0.991 to 1.008)	0.896	0.994 (0.987 to 1.002)	0.137	0.996 (0.990 to 1.001)	0.122		

Exposure	Outcome	nSNP	MR Egger		Weighted median		Inverse variance weighted		MR_PRESSO	
			OR (95% CI)	pval	OR (95% CI)	pval	OR (95% CI)	pval	OR (95% CI)	pval
Glycocholate	Hypertension	6	1.006 (0.987 to 1.026)	0.579	1.006 (0.991 to 1.021)	0.438	1.010 (0.997 to 1.023)	0.148		
Glycodeoxycholate	Hypertension	5	1.151 (1.007 to 1.315)	0.132	0.998 (0.985 to 1.011)	0.723	1.013 (0.977 to 1.050)	0.473	0.998 (0.990 to 1.006)	0.627
Taurochenodeoxycholate	Hypertension	6	0.989 (0.963 to 1.015)	0.449	0.992 (0.973 to 1.011)	0.401	0.993 (0.978 to 1.008)	0.356		
Taurocholate	Hypertension	11	1.007 (0.992 to 1.023)	0.384	0.998 (0.986 to 1.011)	0.759	0.996 (0.988 to 1.005)	0.378		
X-05907	Hypertension	9	1.027 (0.934 to 1.131)	0.597	1.017 (0.967 to 1.070)	0.511	1.019 (0.979 to 1.060)	0.362		
Docosahexaenoate (DHA; 22:6n3)	Hypertension	1								
1-stearoylglycerophosphoinositol	Hypertension	4	0.998 (0.959 to 1.037)	0.915	1.001 (0.973 to 1.030)	0.935	1.001 (0.977 to 1.025)	0.957		

Exposure	Outcome	nSNP	MR Egger		Weighted median		Inverse variance weighted		MR_PRESSO	
			OR (95% CI)	pval	OR (95% CI)	pval	OR (95% CI)	pval	OR (95% CI)	pval
X-06226	Hypertension	15	1.038 (0.953 to 1.131)	0.411	1.044 (0.982 to 1.109)	0.167	1.041 (0.999 to 1.085)	0.057		
X-06246	Hypertension	13	1.016 (0.840 to 1.229)	0.874	1.024 (0.974 to 1.077)	0.351	1.037 (0.987 to 1.089)	0.153	1.012 (0.976 to 1.049)	0.537
X-06267	Hypertension	7	1.014 (0.816 to 1.260)	0.905	0.979 (0.914 to 1.049)	0.542	0.973 (0.912 to 1.038)	0.406		
X-06307	Hypertension	5	1.034 (0.887 to 1.205)	0.697	0.992 (0.952 to 1.033)	0.683	0.976 (0.926 to 1.028)	0.351		
X-06350	Hypertension	7	0.963 (0.892 to 1.040)	0.382	0.987 (0.944 to 1.033)	0.579	0.996 (0.960 to 1.032)	0.818		
X-06351	Hypertension	4	0.954 (0.903 to 1.007)	0.231	0.978 (0.938 to 1.020)	0.300	0.987 (0.952 to 1.024)	0.496		

Exposure	Outcome	nSNP	MR Egger		Weighted median		Inverse variance weighted		MR_PRESSO	
			OR (95% CI)	pval	OR (95% CI)	pval	OR (95% CI)	pval	OR (95% CI)	pval
Myo-inositol	Hypertension	27	0.997 (0.918 to 1.083)	0.938	1.009 (0.966 to 1.053)	0.703	1.024 (0.980 to 1.070)	0.299	1.006 (0.971 to 1.042)	0.750
Glucose	Hypertension	26	0.964 (0.865 to 1.074)	0.513	0.988 (0.932 to 1.048)	0.698	0.970 (0.916 to 1.028)	0.302	0.978 (0.927 to 1.033)	0.439
1,5-anhydroglucitol (1,5-AG)	Hypertension	16	1.013 (0.926 to 1.108)	0.789	0.987 (0.953 to 1.023)	0.482	0.992 (0.954 to 1.031)	0.685	0.983 (0.950 to 1.017)	0.341
2-hydroxybutyrate (AHB)	Hypertension	9	0.957 (0.866 to 1.058)	0.418	0.992 (0.946 to 1.040)	0.743	1.008 (0.954 to 1.065)	0.774	0.994 (0.949 to 1.041)	0.810
3-methyl-2-oxobutyrate	Hypertension	11	0.969 (0.828 to 1.134)	0.702	0.997 (0.932 to 1.068)	0.941	1.004 (0.958 to 1.052)	0.864		
1,6-anhydroglucose	Hypertension	4	1.000 (0.955 to 1.048)	0.988	1.008 (0.987 to 1.030)	0.451	1.014 (0.996 to 1.033)	0.127		

Exposure	Outcome	nSNP	MR Egger		Weighted median		Inverse variance weighted		MR_PRESSO	
			OR (95% CI)	pval	OR (95% CI)	pval	OR (95% CI)	pval	OR (95% CI)	pval
1-palmitoylglycerol (1-monopalmitin)	Hypertension	8	0.710 (0.476 to 1.057)	0.142	0.962 (0.901 to 1.028)	0.255	1.000 (0.920 to 1.088)	0.991	0.993 (0.932 to 1.059)	0.844
1-oleoylglycerol (1-monoolein)	Hypertension	10	0.960 (0.823 to 1.119)	0.616	1.004 (0.974 to 1.036)	0.784	0.997 (0.961 to 1.035)	0.884	0.994 (0.969 to 1.019)	0.635
1-stearoylglycerol (1-monostearin)	Hypertension	16	1.128 (0.955 to 1.331)	0.177	1.022 (0.973 to 1.073)	0.385	0.999 (0.951 to 1.050)	0.981	0.993 (0.947 to 1.042)	0.783
X-08402	Hypertension	12	0.955 (0.907 to 1.006)	0.115	0.945 (0.916 to 0.976)	4.73e-04	0.951 (0.923 to 0.980)	9.7e-04		
2-hydroxyisobutyrate	Hypertension	12	1.002 (0.807 to 1.243)	0.989	0.988 (0.938 to 1.040)	0.640	1.012 (0.944 to 1.085)	0.738	0.987 (0.945 to 1.031)	0.561
X-08766	Hypertension	11	1.032 (0.960 to 1.109)	0.414	1.015 (0.978 to 1.053)	0.437	1.028 (0.996 to 1.061)	0.087		

Exposure	Outcome	nSNP	MR Egger		Weighted median		Inverse variance weighted		MR_PRESSO	
			OR (95% CI)	pval	OR (95% CI)	pval	OR (95% CI)	pval	OR (95% CI)	pval
4-methyl-2-oxopentanoate	Hypertension	7	1.043 (0.876 to 1.243)	0.654	1.027 (0.955 to 1.104)	0.474	1.045 (0.990 to 1.104)	0.113		
Phenyllactate (PLA)	Hypertension	12	0.938 (0.792 to 1.112)	0.480	0.974 (0.921 to 1.029)	0.347	0.976 (0.921 to 1.034)	0.404	0.991 (0.942 to 1.043)	0.732
Homocitrulline	Hypertension	4	0.970 (0.786 to 1.196)	0.800	0.992 (0.949 to 1.036)	0.705	1.001 (0.958 to 1.046)	0.960		
Aspartylphenylalanine	Hypertension	3	1.122 (0.709 to 1.777)	0.709	1.090 (1.047 to 1.135)	2.68e-05	1.120 (1.024 to 1.227)	0.014		
Levulinate (4-oxovalerate)	Hypertension	25	1.227 (0.802 to 1.880)	0.356	0.968 (0.912 to 1.026)	0.275	0.956 (0.898 to 1.019)	0.166	0.962 (0.915 to 1.011)	0.138
Palmitoylcarnitine	Hypertension	5	1.038 (0.692 to 1.556)	0.869	1.047 (0.978 to 1.121)	0.191	1.088 (1.013 to 1.168)	0.020		

Exposure	Outcome	nSNP	MR Egger		Weighted median		Inverse variance weighted		MR_PRESSO	
			OR (95% CI)	pval	OR (95% CI)	pval	OR (95% CI)	pval	OR (95% CI)	pval
X-08988	Hypertension	22	0.902 (0.835 to 0.973)	0.015	0.944 (0.898 to 0.994)	0.028	0.932 (0.899 to 0.965)	8.87e-05	0.958 (0.925 to 0.991)	0.022
X-09026	Hypertension	11	0.977 (0.796 to 1.199)	0.829	0.958 (0.907 to 1.011)	0.122	0.961 (0.913 to 1.011)	0.125		
X-09108	Hypertension	6	1.628 (0.163 to 16.218)	0.699	0.904 (0.825 to 0.990)	0.030	0.925 (0.849 to 1.007)	0.072		
Cholate	Hypertension	2					0.980 (0.956 to 1.006)	0.126		
X-09706	Hypertension	12	0.951 (0.785 to 1.152)	0.618	0.979 (0.926 to 1.034)	0.445	0.997 (0.939 to 1.059)	0.918	1.011 (0.957 to 1.068)	0.703
X-10395	Hypertension	21	0.984 (0.961 to 1.009)	0.228	0.989 (0.963 to 1.015)	0.394	0.990 (0.971 to 1.008)	0.276		

Exposure	Outcome	nSNP	MR Egger		Weighted median		Inverse variance weighted		MR_PRESSO	
			OR (95% CI)	pval	OR (95% CI)	pval	OR (95% CI)	pval	OR (95% CI)	pval
X-10429	Hypertension	15	1.029 (0.990 to 1.070)	0.164	0.989 (0.954 to 1.025)	0.532	0.968 (0.932 to 1.006)	0.094	0.936 (0.913 to 0.960)	2.37e-04
X-10500	Hypertension	24	0.937 (0.720 to 1.218)	0.631	0.968 (0.908 to 1.032)	0.325	0.962 (0.892 to 1.037)	0.310	0.966 (0.916 to 1.019)	0.215
X-10506	Hypertension	6	0.957 (0.840 to 1.091)	0.550	0.978 (0.909 to 1.053)	0.554	0.997 (0.940 to 1.058)	0.930		
X-10510	Hypertension	16	0.900 (0.776 to 1.045)	0.188	0.984 (0.936 to 1.034)	0.514	0.968 (0.909 to 1.031)	0.315	1.026 (0.993 to 1.060)	0.146
1-linoleoylglycerol (1-monolinolein)	Hypertension	5	1.041 (0.910 to 1.191)	0.598	1.034 (1.003 to 1.067)	0.034	1.023 (0.993 to 1.055)	0.138		
Indoleacetate	Hypertension	12	1.028 (0.973 to 1.086)	0.345	0.994 (0.956 to 1.034)	0.762	0.997 (0.965 to 1.030)	0.853		

Exposure	Outcome	nSNP	MR Egger		Weighted median		Inverse variance weighted		MR_PRESSO	
			OR (95% CI)	pval	OR (95% CI)	pval	OR (95% CI)	pval	OR (95% CI)	pval
Hyodeoxycholate	Hypertension	10	1.001 (0.980 to 1.023)	0.896	1.016 (1.002 to 1.029)	0.023	1.011 (0.998 to 1.024)	0.090		
3-indoxyl sulfate	Hypertension	2					0.971 (0.885 to 1.066)	0.540		
N-acetylglycine	Hypertension	7	0.957 (0.910 to 1.007)	0.149	0.952 (0.929 to 0.976)	1.1e-04	0.969 (0.947 to 0.992)	0.007		
Bilirubin (Z,Z)	Hypertension	6	0.986 (0.961 to 1.011)	0.320	0.997 (0.988 to 1.007)	0.604	0.999 (0.985 to 1.013)	0.855		
Creatine	Hypertension	5	0.899 (0.808 to 1.000)	0.144	0.916 (0.881 to 0.952)	8.34e-06	0.913 (0.882 to 0.946)	4.17e-07		
Erythrose	Hypertension	3	1.063 (0.986 to 1.145)	0.357	1.000 (0.955 to 1.048)	0.990	1.000 (0.943 to 1.060)	0.997		

Exposure	Outcome	nSNP	MR Egger		Weighted median		Inverse variance weighted		MR_PRESSO	
			OR (95% CI)	pval	OR (95% CI)	pval	OR (95% CI)	pval	OR (95% CI)	pval
Threonate	Hypertension	9	1.012 (0.970 to 1.055)	0.601	1.006 (0.975 to 1.038)	0.724	1.000 (0.977 to 1.025)	0.969		
X-10810	Hypertension	12	1.000 (0.956 to 1.046)	0.999	1.002 (0.977 to 1.028)	0.863	1.004 (0.974 to 1.035)	0.791		
Cysteine	Hypertension	11	1.020 (0.937 to 1.110)	0.663	0.989 (0.953 to 1.027)	0.558	0.989 (0.937 to 1.044)	0.690	1.003 (0.975 to 1.032)	0.852
Pyroglutamylglycine	Hypertension	2					0.988 (0.939 to 1.040)	0.649		
N-(2-furoyl)glycine	Hypertension	17	1.004 (0.990 to 1.018)	0.606	0.999 (0.991 to 1.006)	0.706	1.003 (0.996 to 1.010)	0.436		
DSGEGDFXAEGGGVR*	Hypertension	8	0.919 (0.839 to 1.008)	0.122	1.028 (0.998 to 1.059)	0.064	1.006 (0.977 to 1.036)	0.684	1.016 (0.990 to 1.042)	0.281

Exposure	Outcome	nSNP	MR Egger		Weighted median		Inverse variance weighted		MR_PRESSO	
			OR (95% CI)	pval	OR (95% CI)	pval	OR (95% CI)	pval	OR (95% CI)	pval
Pyridoxate	Hypertension	6	1.051 (0.991 to 1.115)	0.173	1.035 (0.997 to 1.075)	0.072	1.019 (0.981 to 1.058)	0.332		
Androsterone sulfate	Hypertension	15	1.012 (0.994 to 1.031)	0.198	1.010 (1.002 to 1.019)	0.018	1.011 (0.999 to 1.023)	0.064	1.008 (0.998 to 1.018)	0.160
3-carboxy-4-methyl-5-propyl-2-furanpropanoate (CMPF)	Hypertension	10	1.010 (0.981 to 1.040)	0.525	1.001 (0.988 to 1.014)	0.919	0.993 (0.981 to 1.006)	0.298		
3-(4-hydroxyphenyl)lactate	Hypertension	13	1.037 (0.955 to 1.126)	0.405	1.024 (0.981 to 1.069)	0.281	1.027 (0.990 to 1.066)	0.150		
Acetylcarnitine	Hypertension	8	1.102 (0.965 to 1.259)	0.203	1.073 (1.028 to 1.121)	0.001	1.077 (1.020 to 1.136)	0.007	1.054 (1.015 to 1.095)	0.034
Serine	Hypertension	18	0.890 (0.764 to 1.037)	0.154	1.022 (0.964 to 1.083)	0.471	0.958 (0.899 to 1.022)	0.192	1.013 (0.968 to 1.060)	0.587

Exposure	Outcome	nSNP	MR Egger		Weighted median		Inverse variance weighted		MR_PRESSO	
			OR (95% CI)	pval	OR (95% CI)	pval	OR (95% CI)	pval	OR (95% CI)	pval
Trans-4-hydroxyproline	Hypertension	2					1.021 (0.975 to 1.068)	0.380		
Glutamate	Hypertension	2					1.013 (0.923 to 1.112)	0.792		
Hexanoylcarnitine	Hypertension	11	1.016 (0.959 to 1.078)	0.597	1.020 (0.997 to 1.044)	0.090	1.031 (1.000 to 1.062)	0.048		
Glycine	Hypertension	12	0.925 (0.889 to 0.963)	0.003	0.936 (0.915 to 0.958)	2.04e-08	0.945 (0.918 to 0.973)	1.21e-04		
Alanine	Hypertension	23	1.049 (0.879 to 1.251)	0.602	1.027 (0.965 to 1.092)	0.398	1.025 (0.974 to 1.077)	0.345	1.007 (0.960 to 1.056)	0.782
Glycochenodeoxycholate	Hypertension	9	1.016 (0.969 to 1.066)	0.529	0.998 (0.982 to 1.015)	0.851	0.997 (0.974 to 1.020)	0.778	1.004 (0.987 to 1.021)	0.690

Exposure	Outcome	nSNP	MR Egger		Weighted median		Inverse variance weighted		MR_PRESSO	
			OR (95% CI)	pval	OR (95% CI)	pval	OR (95% CI)	pval	OR (95% CI)	pval
2-aminobutyrate	Hypertension	30	1.020 (0.914 to 1.137)	0.730	1.034 (0.987 to 1.082)	0.157	1.037 (1.000 to 1.075)	0.047		
Scyllo-inositol	Hypertension	7	0.995 (0.962 to 1.029)	0.798	1.006 (0.982 to 1.030)	0.639	1.015 (0.988 to 1.041)	0.281		
Dodecanedioate	Hypertension	6	1.114 (0.997 to 1.245)	0.129	1.000 (0.966 to 1.034)	0.978	1.017 (0.982 to 1.053)	0.343		
Gamma-glutamylvaline	Hypertension	8	1.101 (0.664 to 1.826)	0.722	1.022 (0.956 to 1.093)	0.521	1.061 (0.962 to 1.170)	0.236	1.011 (0.962 to 1.064)	0.671
Indolepropionate	Hypertension	10	0.978 (0.903 to 1.059)	0.592	0.986 (0.956 to 1.018)	0.384	0.991 (0.964 to 1.018)	0.501		
Butyrylcarnitine	Hypertension	16	0.974 (0.945 to 1.004)	0.109	0.989 (0.977 to 1.001)	0.063	0.991 (0.972 to 1.010)	0.366	0.988 (0.973 to 1.003)	0.127

Exposure	Outcome	nSNP	MR Egger		Weighted median		Inverse variance weighted		MR_PRESSO	
			OR (95% CI)	pval	OR (95% CI)	pval	OR (95% CI)	pval	OR (95% CI)	pval
Myristoleate (14:1n5)	Hypertension	10	1.009 (0.925 to 1.101)	0.844	1.000 (0.966 to 1.035)	0.999	0.976 (0.936 to 1.018)	0.260	0.984 (0.955 to 1.013)	0.311
Dehydroisoandrosterone sulfate (DHEA-S)	Hypertension	12	1.038 (0.913 to 1.181)	0.581	1.030 (1.002 to 1.059)	0.036	1.037 (0.995 to 1.080)	0.085	1.037 (1.009 to 1.066)	0.029
Propionylcarnitine	Hypertension	24	0.982 (0.887 to 1.088)	0.738	0.998 (0.956 to 1.042)	0.938	1.005 (0.960 to 1.052)	0.821	1.010 (0.974 to 1.048)	0.589
Caproate (6:0)	Hypertension	17	1.049 (0.812 to 1.356)	0.719	1.031 (0.966 to 1.099)	0.357	0.983 (0.910 to 1.063)	0.668	0.991 (0.938 to 1.048)	0.757
Caprylate (8:0)	Hypertension	30	1.054 (0.956 to 1.163)	0.301	1.034 (0.983 to 1.088)	0.189	1.007 (0.961 to 1.055)	0.772	1.004 (0.967 to 1.043)	0.834
10-undecenoate (11:1n1)	Hypertension	12	1.019 (0.952 to 1.090)	0.607	1.002 (0.972 to 1.033)	0.886	0.991 (0.953 to 1.030)	0.653	0.999 (0.967 to 1.032)	0.953

Exposure	Outcome	nSNP	MR Egger		Weighted median		Inverse variance weighted		MR_PRESSO	
			OR (95% CI)	pval	OR (95% CI)	pval	OR (95% CI)	pval	OR (95% CI)	pval
Docosapentaenoate (n3 DPA; 22:5n3)	Hypertension	4	0.977 (0.883 to 1.082)	0.700	0.990 (0.950 to 1.033)	0.650	0.994 (0.960 to 1.029)	0.733		
X-11204	Hypertension	24	1.173 (0.745 to 1.846)	0.498	1.013 (0.946 to 1.085)	0.711	0.929 (0.823 to 1.049)	0.235	1.002 (0.948 to 1.059)	0.938
X-02269	Hypertension	8	1.061 (0.942 to 1.194)	0.369	1.001 (0.974 to 1.028)	0.965	1.007 (0.974 to 1.041)	0.697	1.009 (0.983 to 1.036)	0.518
Phenol sulfate	Hypertension	9	1.013 (0.874 to 1.173)	0.869	1.004 (0.972 to 1.038)	0.794	0.993 (0.955 to 1.032)	0.717	1.003 (0.969 to 1.039)	0.859
X-06126	Hypertension	10	0.999 (0.850 to 1.174)	0.986	0.989 (0.961 to 1.017)	0.435	1.005 (0.974 to 1.036)	0.762	1.006 (0.983 to 1.029)	0.624
X-07765	Hypertension	9	1.001 (0.959 to 1.044)	0.978	1.000 (0.984 to 1.016)	0.997	1.005 (0.986 to 1.024)	0.624	1.004 (0.991 to 1.016)	0.579

Exposure	Outcome	nSNP	MR Egger		Weighted median		Inverse variance weighted		MR_PRESSO	
			OR (95% CI)	pval	OR (95% CI)	pval	OR (95% CI)	pval	OR (95% CI)	pval
X-11247	Hypertension	11	1.005 (0.977 to 1.034)	0.737	0.987 (0.970 to 1.004)	0.146	0.986 (0.974 to 0.999)	0.030		
X-11261	Hypertension	7	0.981 (0.858 to 1.122)	0.790	1.013 (0.982 to 1.045)	0.424	1.028 (0.977 to 1.081)	0.285	1.022 (0.983 to 1.063)	0.339
Bilirubin (E,E)*	Hypertension	8	0.997 (0.977 to 1.018)	0.777	0.993 (0.981 to 1.005)	0.252	0.992 (0.981 to 1.003)	0.143		
X-02249	Hypertension	7	0.947 (0.797 to 1.126)	0.567	0.965 (0.921 to 1.011)	0.136	0.974 (0.931 to 1.019)	0.248		
Heme*	Hypertension	5	1.055 (0.942 to 1.183)	0.421	0.976 (0.936 to 1.017)	0.244	0.980 (0.949 to 1.012)	0.218		
X-11299	Hypertension	6	0.982 (0.884 to 1.091)	0.753	0.991 (0.969 to 1.012)	0.399	0.993 (0.972 to 1.014)	0.490		

Exposure	Outcome	nSNP	MR Egger		Weighted median		Inverse variance weighted		MR_PRESSO	
			OR (95% CI)	pval	OR (95% CI)	pval	OR (95% CI)	pval	OR (95% CI)	pval
X-11315	Hypertension	17	0.985 (0.930 to 1.044)	0.629	0.983 (0.952 to 1.014)	0.281	0.999 (0.966 to 1.033)	0.950	0.999 (0.975 to 1.023)	0.924
X-11317	Hypertension	28	1.134 (0.993 to 1.294)	0.074	1.025 (0.972 to 1.081)	0.363	0.987 (0.937 to 1.040)	0.624	1.000 (0.955 to 1.047)	0.997
1-linoleoylglycerophosphoethanolamine*	Hypertension	8	0.993 (0.874 to 1.128)	0.916	1.015 (0.982 to 1.049)	0.379	1.010 (0.968 to 1.055)	0.642	0.997 (0.965 to 1.031)	0.883
X-11327	Hypertension	24	1.082 (0.973 to 1.204)	0.161	1.004 (0.955 to 1.055)	0.872	0.985 (0.942 to 1.030)	0.514	0.996 (0.959 to 1.034)	0.824
X-11334	Hypertension	14	1.027 (0.809 to 1.304)	0.828	1.009 (0.960 to 1.061)	0.729	1.001 (0.942 to 1.063)	0.980	0.978 (0.942 to 1.016)	0.275
3-dehydrocarnitine*	Hypertension	16	1.243 (1.055 to 1.464)	0.021	1.023 (0.967 to 1.081)	0.435	1.060 (1.005 to 1.119)	0.032	1.030 (0.976 to 1.088)	0.304

Exposure	Outcome	nSNP	MR Egger		Weighted median		Inverse variance weighted		MR_PRESSO	
			OR (95% CI)	pval	OR (95% CI)	pval	OR (95% CI)	pval	OR (95% CI)	pval
Pyroglutamine*	Hypertension	11	0.995 (0.891 to 1.110)	0.924	0.989 (0.964 to 1.016)	0.424	1.009 (0.970 to 1.049)	0.671	0.987 (0.963 to 1.012)	0.327
C-glycosyltryptophan*	Hypertension	15	1.057 (0.909 to 1.228)	0.486	1.048 (0.978 to 1.124)	0.183	1.003 (0.930 to 1.082)	0.935	1.023 (0.965 to 1.085)	0.460
X-11374	Hypertension	12	1.019 (0.992 to 1.046)	0.195	1.003 (0.985 to 1.021)	0.777	1.000 (0.986 to 1.014)	0.975		
X-11381	Hypertension	9	1.052 (0.935 to 1.183)	0.427	0.987 (0.934 to 1.042)	0.636	0.990 (0.950 to 1.032)	0.638		
X-03056--N-[3-(2-Oxopyrrolidin-1-yl)propyl]acetamide	Hypertension	17	1.005 (0.963 to 1.048)	0.829	1.028 (0.996 to 1.062)	0.087	1.030 (1.005 to 1.055)	0.018		
X-11412	Hypertension	26	0.989 (0.741 to 1.322)	0.943	0.969 (0.920 to 1.021)	0.241	0.996 (0.947 to 1.048)	0.887	0.979 (0.945 to 1.014)	0.242

Exposure	Outcome	nSNP	MR Egger		Weighted median		Inverse variance weighted		MR_PRESSO	
			OR (95% CI)	pval	OR (95% CI)	pval	OR (95% CI)	pval	OR (95% CI)	pval
X-01911	Hypertension	9	1.003 (0.935 to 1.076)	0.936	0.997 (0.973 to 1.022)	0.840	0.994 (0.967 to 1.022)	0.679	0.999 (0.977 to 1.023)	0.957
X-11422--xanthine	Hypertension	3	1.179 (0.798 to 1.740)	0.560	0.991 (0.927 to 1.059)	0.792	0.987 (0.932 to 1.045)	0.655		
X-11423--O-sulfo-L-tyrosine	Hypertension	23	0.968 (0.809 to 1.158)	0.728	0.974 (0.919 to 1.032)	0.375	1.035 (0.967 to 1.108)	0.323	1.020 (0.979 to 1.062)	0.363
X-09789	Hypertension	5	1.256 (0.716 to 2.203)	0.485	1.001 (0.962 to 1.041)	0.963	1.013 (0.971 to 1.057)	0.551	1.011 (0.971 to 1.052)	0.657
X-11437	Hypertension	7	1.003 (0.985 to 1.021)	0.764	1.000 (0.985 to 1.015)	0.989	0.999 (0.987 to 1.010)	0.819		
X-11438	Hypertension	17	0.987 (0.917 to 1.062)	0.729	1.009 (0.982 to 1.037)	0.511	1.008 (0.978 to 1.040)	0.590	1.006 (0.990 to 1.022)	0.489

Exposure	Outcome	nSNP	MR Egger		Weighted median		Inverse variance weighted		MR_PRESSO	
			OR (95% CI)	pval	OR (95% CI)	pval	OR (95% CI)	pval	OR (95% CI)	pval
X-11440	Hypertension	12	0.998 (0.960 to 1.038)	0.916	1.006 (0.994 to 1.019)	0.315	1.012 (0.987 to 1.038)	0.349	1.005 (0.997 to 1.013)	0.235
X-11441	Hypertension	8	0.988 (0.954 to 1.023)	0.516	0.994 (0.978 to 1.009)	0.426	0.998 (0.976 to 1.019)	0.831		
X-11442	Hypertension	8	0.989 (0.955 to 1.024)	0.549	0.995 (0.980 to 1.010)	0.531	0.997 (0.976 to 1.017)	0.746		
X-11444	Hypertension	8	0.926 (0.828 to 1.034)	0.221	0.986 (0.946 to 1.028)	0.504	1.017 (0.951 to 1.087)	0.618	1.011 (0.960 to 1.065)	0.705
X-11445--5-alpha-pregnan-3beta,20alpha-disulfate	Hypertension	9	0.990 (0.944 to 1.038)	0.683	0.984 (0.969 to 1.000)	0.053	0.988 (0.976 to 1.000)	0.059		
X-11452	Hypertension	5	1.037 (0.825 to 1.304)	0.777	1.011 (0.969 to 1.055)	0.623	1.050 (0.994 to 1.109)	0.080	1.025 (0.978 to 1.074)	0.384

Exposure	Outcome	nSNP	MR Egger		Weighted median		Inverse variance weighted		MR_PRESSO	
			OR (95% CI)	pval	OR (95% CI)	pval	OR (95% CI)	pval	OR (95% CI)	pval
X-11469	Hypertension	8	1.083 (0.885 to 1.325)	0.470	1.012 (0.989 to 1.036)	0.298	1.006 (0.977 to 1.036)	0.693	1.010 (0.992 to 1.028)	0.334
X-11470	Hypertension	7	0.951 (0.705 to 1.283)	0.756	1.009 (0.959 to 1.062)	0.722	1.030 (0.914 to 1.161)	0.622	1.020 (0.980 to 1.061)	0.396
X-11478	Hypertension	8	0.964 (0.926 to 1.003)	0.123	0.984 (0.961 to 1.009)	0.206	0.985 (0.959 to 1.012)	0.278		
X-11483	Hypertension	7	1.006 (0.990 to 1.023)	0.474	1.003 (0.991 to 1.015)	0.623	1.004 (0.994 to 1.015)	0.396		
X-11485	Hypertension	2					1.009 (0.977 to 1.042)	0.591		
X-11491	Hypertension	10	1.019 (0.971 to 1.070)	0.466	0.993 (0.974 to 1.013)	0.505	0.983 (0.958 to 1.008)	0.182	0.986 (0.970 to 1.002)	0.135

Exposure	Outcome	nSNP	MR Egger		Weighted median		Inverse variance weighted		MR_PRESSO	
			OR (95% CI)	pval	OR (95% CI)	pval	OR (95% CI)	pval	OR (95% CI)	pval
X-11497	Hypertension	5	0.691 (0.509 to 0.938)	0.099	0.942 (0.866 to 1.026)	0.169	0.931 (0.867 to 1.000)	0.049		
HWESASXX*	Hypertension	6	1.107 (0.940 to 1.303)	0.290	0.998 (0.968 to 1.029)	0.898	0.997 (0.957 to 1.038)	0.868	0.992 (0.967 to 1.017)	0.568
X-11521	Hypertension	6	0.921 (0.715 to 1.186)	0.558	1.004 (0.958 to 1.052)	0.872	0.988 (0.947 to 1.031)	0.581		
X-11529	Hypertension	5	0.994 (0.984 to 1.005)	0.346	0.997 (0.989 to 1.005)	0.430	0.998 (0.990 to 1.005)	0.524		
X-11530	Hypertension	5	0.999 (0.975 to 1.024)	0.958	0.993 (0.978 to 1.009)	0.403	0.997 (0.983 to 1.011)	0.685		
X-11537	Hypertension	3	1.024 (0.791 to 1.327)	0.885	1.011 (0.982 to 1.042)	0.450	1.011 (0.984 to 1.039)	0.440		

Exposure	Outcome	nSNP	MR Egger		Weighted median		Inverse variance weighted		MR_PRESSO	
			OR (95% CI)	pval	OR (95% CI)	pval	OR (95% CI)	pval	OR (95% CI)	pval
X-11538	Hypertension	11	1.010 (0.979 to 1.042)	0.548	0.991 (0.973 to 1.009)	0.326	0.984 (0.964 to 1.004)	0.122		
X-11540	Hypertension	5	0.962 (0.891 to 1.039)	0.398	1.020 (0.987 to 1.055)	0.230	1.020 (0.992 to 1.048)	0.166		
X-11546	Hypertension	11	1.009 (0.994 to 1.024)	0.277	1.004 (0.994 to 1.014)	0.462	1.004 (0.997 to 1.012)	0.243		
X-11550	Hypertension	15	1.209 (0.730 to 2.000)	0.474	1.038 (0.931 to 1.157)	0.501	1.024 (0.893 to 1.175)	0.732	1.033 (0.933 to 1.143)	0.546
X-11552	Hypertension	8	0.959 (0.856 to 1.075)	0.500	0.993 (0.963 to 1.025)	0.672	0.989 (0.966 to 1.013)	0.371		
X-11593--O-methylascorbate*	Hypertension	24	0.944 (0.906 to 0.984)	0.012	0.945 (0.916 to 0.975)	3.76e-04	0.956 (0.933 to 0.981)	5.54e-04		

Exposure	Outcome	nSNP	MR Egger		Weighted median		Inverse variance weighted		MR_PRESSO	
			OR (95% CI)	pval	OR (95% CI)	pval	OR (95% CI)	pval	OR (95% CI)	pval
Adrenate (22:4n6)	Hypertension	5	1.062 (0.856 to 1.318)	0.622	0.989 (0.948 to 1.033)	0.622	0.988 (0.936 to 1.043)	0.667		
Homostachydrine*	Hypertension	4	0.949 (0.887 to 1.015)	0.265	0.986 (0.959 to 1.013)	0.303	0.991 (0.967 to 1.016)	0.477		
ADSGEGDFXAEGGGVR*	Hypertension	6	1.018 (0.938 to 1.105)	0.690	1.015 (0.986 to 1.044)	0.310	1.027 (0.992 to 1.063)	0.133		
X-11786--methylcysteine	Hypertension	8	0.992 (0.904 to 1.087)	0.863	1.012 (0.985 to 1.040)	0.388	1.003 (0.982 to 1.024)	0.803		
X-11787	Hypertension	15	0.965 (0.880 to 1.059)	0.469	0.992 (0.943 to 1.044)	0.755	0.973 (0.926 to 1.023)	0.287	0.981 (0.939 to 1.024)	0.399
X-11792	Hypertension	10	1.059 (1.006 to 1.115)	0.060	0.999 (0.984 to 1.016)	0.948	1.007 (0.987 to 1.028)	0.474	1.005 (0.987 to 1.024)	0.603

Exposure	Outcome	nSNP	MR Egger		Weighted median		Inverse variance weighted		MR_PRESSO	
			OR (95% CI)	pval	OR (95% CI)	pval	OR (95% CI)	pval	OR (95% CI)	pval
X-11793--oxidized bilirubin*	Hypertension	9	1.006 (0.951 to 1.065)	0.831	0.991 (0.974 to 1.009)	0.330	0.994 (0.962 to 1.026)	0.694	0.984 (0.967 to 1.001)	0.108
X-11795	Hypertension	7	1.012 (0.859 to 1.192)	0.892	1.063 (1.010 to 1.118)	0.019	1.048 (1.007 to 1.091)	0.021		
X-11799	Hypertension	4	0.989 (0.965 to 1.014)	0.482	0.995 (0.982 to 1.008)	0.427	0.997 (0.985 to 1.009)	0.586		
X-11805	Hypertension	3	0.959 (0.895 to 1.028)	0.448	1.006 (0.977 to 1.037)	0.671	0.997 (0.972 to 1.021)	0.783		
X-11818	Hypertension	13	0.972 (0.908 to 1.040)	0.425	0.981 (0.940 to 1.023)	0.363	0.996 (0.964 to 1.029)	0.810		
X-11820	Hypertension	7	1.015 (0.970 to 1.063)	0.545	1.016 (0.986 to 1.047)	0.302	1.003 (0.982 to 1.025)	0.769		
X-11843	Hypertension	1								

Exposure	Outcome	nSNP	MR Egger		Weighted median		Inverse variance weighted		MR_PRESSO	
			OR (95% CI)	pval	OR (95% CI)	pval	OR (95% CI)	pval	OR (95% CI)	pval
X-11845	Hypertension	12	0.999 (0.979 to 1.020)	0.951	1.004 (0.991 to 1.017)	0.541	1.005 (0.997 to 1.014)	0.237		
X-11847	Hypertension	7	1.057 (1.003 to 1.113)	0.091	1.003 (0.984 to 1.021)	0.783	1.009 (0.990 to 1.028)	0.377	1.003 (0.986 to 1.021)	0.734
X-11849	Hypertension	7	1.028 (1.001 to 1.056)	0.098	1.008 (0.994 to 1.023)	0.249	1.007 (0.993 to 1.022)	0.325	1.001 (0.987 to 1.016)	0.851
X-11850	Hypertension	5	1.001 (0.979 to 1.023)	0.928	0.999 (0.986 to 1.012)	0.854	0.999 (0.987 to 1.010)	0.806		
X-11852	Hypertension	7	0.981 (0.966 to 0.996)	0.056	0.992 (0.980 to 1.003)	0.159	0.996 (0.985 to 1.006)	0.416		
X-11858	Hypertension	6	1.014 (0.994 to 1.035)	0.237	1.009 (0.997 to 1.020)	0.128	1.013 (1.003 to 1.024)	0.014		

Exposure	Outcome	nSNP	MR Egger		Weighted median		Inverse variance weighted		MR_PRESSO	
			OR (95% CI)	pval	OR (95% CI)	pval	OR (95% CI)	pval	OR (95% CI)	pval
X-11859	Hypertension	7	0.940 (0.866 to 1.020)	0.198	0.967 (0.924 to 1.011)	0.141	0.971 (0.939 to 1.003)	0.079		
X-11876	Hypertension	8	0.985 (0.897 to 1.083)	0.771	1.006 (0.975 to 1.038)	0.694	0.997 (0.958 to 1.037)	0.864	0.985 (0.955 to 1.016)	0.386
1-arachidonoylglycerophosphocholine*	Hypertension	14	0.973 (0.929 to 1.018)	0.256	0.988 (0.961 to 1.017)	0.417	1.005 (0.979 to 1.031)	0.710		
1-palmitoleoylglycerophosphocholine*	Hypertension	8	0.746 (0.572 to 0.973)	0.074	1.053 (0.990 to 1.119)	0.099	0.985 (0.900 to 1.079)	0.750	1.037 (0.994 to 1.082)	0.153
X-11905	Hypertension	12	0.990 (0.961 to 1.019)	0.516	0.987 (0.970 to 1.005)	0.164	0.991 (0.978 to 1.004)	0.173		
X-12007	Hypertension	18	1.010 (0.996 to 1.024)	0.184	1.008 (0.999 to 1.017)	0.098	1.005 (0.998 to 1.013)	0.156		

Exposure	Outcome	nSNP	MR Egger		Weighted median		Inverse variance weighted		MR_PRESSO	
			OR (95% CI)	pval	OR (95% CI)	pval	OR (95% CI)	pval	OR (95% CI)	pval
X-12013	Hypertension	4	1.010 (0.995 to 1.025)	0.332	1.005 (0.993 to 1.018)	0.416	1.001 (0.986 to 1.016)	0.885		
Gamma-glutamylmethionine*	Hypertension	6	1.037 (0.961 to 1.120)	0.400	1.000 (0.964 to 1.038)	0.986	0.990 (0.962 to 1.020)	0.510		
Gamma-glutamylthreonine*	Hypertension	6	0.985 (0.888 to 1.093)	0.794	0.973 (0.925 to 1.023)	0.285	0.953 (0.914 to 0.993)	0.023		
X-12029	Hypertension	25	1.093 (0.919 to 1.299)	0.324	1.021 (0.957 to 1.089)	0.537	1.040 (0.992 to 1.091)	0.105		
X-12038	Hypertension	37	0.871 (0.701 to 1.083)	0.223	0.986 (0.944 to 1.030)	0.518	0.994 (0.951 to 1.040)	0.808	0.990 (0.956 to 1.024)	0.555
X-12039	Hypertension	12	1.003 (0.969 to 1.038)	0.865	1.002 (0.986 to 1.019)	0.806	1.001 (0.984 to 1.018)	0.922		

Exposure	Outcome	nSNP	MR Egger		Weighted median		Inverse variance weighted		MR_PRESSO	
			OR (95% CI)	pval	OR (95% CI)	pval	OR (95% CI)	pval	OR (95% CI)	pval
X-12040	Hypertension	9	1.022 (1.010 to 1.033)	0.007	1.005 (0.999 to 1.011)	0.081	1.005 (0.998 to 1.012)	0.189	1.007 (1.003 to 1.011)	0.010
X-12056	Hypertension	10	1.006 (0.972 to 1.042)	0.731	1.013 (0.997 to 1.029)	0.112	1.001 (0.983 to 1.019)	0.908	1.007 (0.996 to 1.017)	0.250
X-12063	Hypertension	11	1.010 (0.980 to 1.042)	0.519	1.004 (0.989 to 1.018)	0.626	1.005 (0.986 to 1.024)	0.624	0.992 (0.981 to 1.004)	0.244
Gamma-tocopherol	Hypertension	7	0.925 (0.821 to 1.042)	0.255	1.014 (0.978 to 1.051)	0.460	1.019 (0.992 to 1.046)	0.173		
Gamma-glutamylphenylalanine	Hypertension	21	0.990 (0.819 to 1.197)	0.917	0.954 (0.895 to 1.017)	0.145	0.977 (0.920 to 1.038)	0.459	0.963 (0.912 to 1.017)	0.188
Isobutyrylcarnitine	Hypertension	10	0.974 (0.912 to 1.039)	0.441	0.992 (0.968 to 1.017)	0.527	0.995 (0.964 to 1.028)	0.779	0.990 (0.963 to 1.018)	0.499

Exposure	Outcome	nSNP	MR Egger		Weighted median		Inverse variance weighted		MR_PRESSO	
			OR (95% CI)	pval	OR (95% CI)	pval	OR (95% CI)	pval	OR (95% CI)	pval
Pseudouridine	Hypertension	19	1.107 (0.847 to 1.447)	0.465	1.011 (0.937 to 1.091)	0.772	1.046 (0.979 to 1.117)	0.185		
Valerate	Hypertension	1								
Palmitoleate (16:1n7)	Hypertension	4	0.894 (0.618 to 1.293)	0.612	0.989 (0.914 to 1.071)	0.794	0.946 (0.846 to 1.058)	0.332	0.981 (NaN to NaN)	NaN
Alpha-ketoglutarate	Hypertension	11	0.986 (0.884 to 1.098)	0.798	1.000 (0.966 to 1.035)	0.997	0.998 (0.947 to 1.051)	0.926	0.992 (0.959 to 1.027)	0.661
Erythronate*	Hypertension	26	0.815 (0.409 to 1.624)	0.567	1.036 (0.977 to 1.098)	0.240	1.088 (0.979 to 1.208)	0.117	1.068 (1.023 to 1.115)	0.007
Lathosterol	Hypertension	10	0.987 (0.947 to 1.029)	0.567	0.997 (0.970 to 1.025)	0.845	1.009 (0.987 to 1.030)	0.428		
X-12092	Hypertension	11	0.995 (0.986 to 1.004)	0.267	0.996 (0.990 to 1.003)	0.240	0.996 (0.990 to 1.003)	0.240		

Exposure	Outcome	nSNP	MR Egger		Weighted median		Inverse variance weighted		MR_PRESSO	
			OR (95% CI)	pval	OR (95% CI)	pval	OR (95% CI)	pval	OR (95% CI)	pval
X-12093	Hypertension	7	1.020 (0.994 to 1.046)	0.191	1.009 (0.995 to 1.023)	0.222	1.004 (0.991 to 1.018)	0.520		
X-12094	Hypertension	5	0.952 (0.800 to 1.132)	0.614	0.994 (0.946 to 1.043)	0.798	1.008 (0.960 to 1.059)	0.738		
X-12095--N1-methyl-3-pyridone-4-carboxamide	Hypertension	15	1.003 (0.938 to 1.072)	0.932	1.002 (0.964 to 1.042)	0.921	1.015 (0.984 to 1.048)	0.350		
X-12100--hydroxytryptophan*	Hypertension	31	1.098 (0.906 to 1.331)	0.347	1.026 (0.978 to 1.076)	0.295	1.019 (0.976 to 1.065)	0.385	1.029 (0.988 to 1.071)	0.179
X-12116	Hypertension	4	1.003 (0.616 to 1.631)	0.992	0.985 (0.947 to 1.024)	0.435	1.002 (0.951 to 1.055)	0.950		
Eicosenoate (20:1n9 or 11)	Hypertension	6	0.958 (0.831 to 1.105)	0.588	0.983 (0.938 to 1.029)	0.455	0.980 (0.932 to 1.031)	0.435		

Exposure	Outcome	nSNP	MR Egger		Weighted median		Inverse variance weighted		MR_PRESSO	
			OR (95% CI)	pval	OR (95% CI)	pval	OR (95% CI)	pval	OR (95% CI)	pval
X-12188	Hypertension	11	0.999 (0.990 to 1.008)	0.793	1.000 (0.994 to 1.005)	0.869	0.998 (0.994 to 1.002)	0.367		
X-12189	Hypertension	20	0.999 (0.994 to 1.003)	0.559	1.001 (0.996 to 1.006)	0.709	1.000 (0.997 to 1.003)	0.997		
X-12206	Hypertension	5	1.045 (0.579 to 1.886)	0.894	0.961 (0.901 to 1.025)	0.229	1.006 (0.900 to 1.124)	0.919	0.982 (0.933 to 1.034)	0.559
X-12212	Hypertension	5	0.978 (0.900 to 1.063)	0.644	1.012 (0.984 to 1.040)	0.407	1.007 (0.984 to 1.031)	0.551		
X-12216	Hypertension	1								
X-12217	Hypertension	7	0.996 (0.896 to 1.108)	0.950	0.993 (0.960 to 1.029)	0.709	1.016 (0.956 to 1.080)	0.603	0.983 (0.953 to 1.014)	0.343
X-12230	Hypertension	2					0.992 (0.953 to 1.033)	0.703		

Exposure	Outcome	nSNP	MR Egger		Weighted median		Inverse variance weighted		MR_PRESSO	
			OR (95% CI)	pval	OR (95% CI)	pval	OR (95% CI)	pval	OR (95% CI)	pval
X-12231	Hypertension	7	0.938 (0.685 to 1.284)	0.706	0.968 (0.936 to 1.002)	0.061	0.984 (0.944 to 1.027)	0.462	0.964 (0.950 to 0.978)	0.004
X-12236	Hypertension	8	1.003 (0.962 to 1.046)	0.897	0.985 (0.967 to 1.004)	0.114	0.985 (0.968 to 1.003)	0.094		
X-12244--N-acetylcarnosine	Hypertension	14	1.123 (0.999 to 1.262)	0.076	0.993 (0.949 to 1.039)	0.748	0.999 (0.961 to 1.038)	0.950		
X-12253	Hypertension	4	1.010 (0.938 to 1.089)	0.811	0.989 (0.963 to 1.017)	0.438	0.987 (0.965 to 1.010)	0.271		
X-12261	Hypertension	7	1.005 (0.991 to 1.018)	0.537	1.002 (0.994 to 1.011)	0.584	1.002 (0.994 to 1.009)	0.660		
X-12329	Hypertension	18	1.001 (0.997 to 1.004)	0.773	1.000 (0.997 to 1.004)	0.814	1.001 (0.998 to 1.004)	0.473		

Exposure	Outcome	nSNP	MR Egger		Weighted median		Inverse variance weighted		MR_PRESSO	
			OR (95% CI)	pval	OR (95% CI)	pval	OR (95% CI)	pval	OR (95% CI)	pval
X-10346	Hypertension	9	0.999 (0.982 to 1.017)	0.931	0.998 (0.987 to 1.008)	0.681	0.998 (0.989 to 1.006)	0.559		
ADpSGEGDFXAEGGGVR*	Hypertension	4	0.814 (0.537 to 1.235)	0.436	0.935 (0.893 to 0.979)	0.004	0.978 (0.862 to 1.109)	0.724	0.961 (0.916 to 1.008)	0.349
1-eicosatrienoylglycerophosphocholine*	Hypertension	13	0.970 (0.894 to 1.054)	0.489	1.023 (0.989 to 1.059)	0.186	1.028 (0.997 to 1.060)	0.081		
1-docosahexaenoylglycerophosphocholine*	Hypertension	4	1.133 (0.880 to 1.460)	0.435	0.959 (0.899 to 1.024)	0.210	0.948 (0.893 to 1.007)	0.084		
X-12405	Hypertension	4	0.908 (0.804 to 1.025)	0.260	0.992 (0.959 to 1.025)	0.614	0.998 (0.971 to 1.025)	0.868		
X-12407	Hypertension	5	1.007 (0.960 to 1.057)	0.786	1.005 (0.990 to 1.021)	0.494	1.005 (0.991 to 1.019)	0.479		

Exposure	Outcome	nSNP	MR Egger		Weighted median		Inverse variance weighted		MR_PRESSO	
			OR (95% CI)	pval	OR (95% CI)	pval	OR (95% CI)	pval	OR (95% CI)	pval
X-12428	Hypertension	2					1.020 (0.989 to 1.052)	0.213		
1-eicosadienoylglycerophosphocholine*	Hypertension	6	1.086 (0.949 to 1.242)	0.295	1.018 (0.978 to 1.060)	0.387	0.999 (0.963 to 1.036)	0.936		
X-12435	Hypertension	16	0.999 (0.993 to 1.006)	0.844	1.001 (0.996 to 1.005)	0.729	1.000 (0.996 to 1.003)	0.829		
X-12441--12-hydroxyeicosatetraenoate (12-HETE)	Hypertension	6	0.998 (0.971 to 1.026)	0.887	1.004 (0.991 to 1.016)	0.577	1.006 (0.996 to 1.016)	0.242		
X-12442--5,8-tetradecadienoate	Hypertension	6	1.015 (0.967 to 1.065)	0.587	1.006 (0.979 to 1.033)	0.671	1.010 (0.988 to 1.033)	0.380		
X-12443	Hypertension	8	0.996 (0.972 to 1.020)	0.736	0.998 (0.984 to 1.013)	0.831	0.998 (0.987 to 1.009)	0.701		

Exposure	Outcome	nSNP	MR Egger		Weighted median		Inverse variance weighted		MR_PRESSO	
			OR (95% CI)	pval	OR (95% CI)	pval	OR (95% CI)	pval	OR (95% CI)	pval
X-12450	Hypertension	16	1.000 (0.925 to 1.081)	0.997	0.993 (0.955 to 1.032)	0.722	0.991 (0.963 to 1.020)	0.524		
X-12456	Hypertension	12	1.008 (0.973 to 1.045)	0.653	1.005 (0.983 to 1.027)	0.648	0.998 (0.983 to 1.014)	0.843		
X-12465	Hypertension	6	0.975 (0.939 to 1.013)	0.270	0.994 (0.966 to 1.023)	0.682	1.010 (0.981 to 1.039)	0.511		
Octanoylcarnitine	Hypertension	7	0.988 (0.934 to 1.045)	0.687	1.018 (0.998 to 1.039)	0.085	1.027 (0.991 to 1.064)	0.149	1.020 (1.001 to 1.039)	0.100
Alpha-hydroxyisovalerate	Hypertension	9	1.007 (0.941 to 1.078)	0.845	1.025 (0.995 to 1.057)	0.104	1.018 (0.993 to 1.044)	0.156		
N-acetylthreonine	Hypertension	6	1.032 (0.880 to 1.210)	0.718	1.015 (0.947 to 1.087)	0.675	1.018 (0.960 to 1.080)	0.549		

Exposure	Outcome	nSNP	MR Egger		Weighted median		Inverse variance weighted		MR_PRESSO	
			OR (95% CI)	pval	OR (95% CI)	pval	OR (95% CI)	pval	OR (95% CI)	pval
Decanoylcarnitine	Hypertension	9	1.001 (0.943 to 1.062)	0.975	1.015 (0.993 to 1.037)	0.189	1.016 (0.986 to 1.047)	0.310	1.009 (0.990 to 1.028)	0.399
1-palmitoylglycerophosphocholine	Hypertension	19	1.185 (0.908 to 1.546)	0.229	0.962 (0.898 to 1.031)	0.271	0.966 (0.907 to 1.029)	0.282	0.946 (0.896 to 1.000)	0.064
1-heptadecanoylglycerophosphocholine	Hypertension	4	1.106 (0.908 to 1.348)	0.422	1.012 (0.957 to 1.071)	0.678	1.000 (0.930 to 1.076)	0.996		
1-oleoylglycerophosphocholine	Hypertension	7	0.916 (0.734 to 1.143)	0.474	0.975 (0.908 to 1.046)	0.476	1.006 (0.921 to 1.100)	0.890	1.008 (0.944 to 1.075)	0.830
1-stearoylglycerophosphocholine	Hypertension	4	1.043 (0.786 to 1.385)	0.796	0.996 (0.936 to 1.060)	0.907	0.975 (0.912 to 1.043)	0.466		
5-dodecenoate (12:1n7)	Hypertension	11	0.976 (0.885 to 1.077)	0.639	0.991 (0.965 to 1.018)	0.495	0.979 (0.948 to 1.011)	0.198	0.994 (0.984 to 1.004)	0.261

Exposure	Outcome	nSNP	MR Egger		Weighted median		Inverse variance weighted		MR_PRESSO	
			OR (95% CI)	pval	OR (95% CI)	pval	OR (95% CI)	pval	OR (95% CI)	pval
Stearidonate (18:4n3)	Hypertension	5	0.947 (0.753 to 1.192)	0.676	0.992 (0.949 to 1.036)	0.709	0.990 (0.934 to 1.049)	0.726		
10-heptadecenoate (17:1n7)	Hypertension	2					0.968 (0.898 to 1.042)	0.385		
10-nonadecenoate (19:1n9)	Hypertension	4	0.948 (0.860 to 1.044)	0.393	1.000 (0.950 to 1.052)	0.990	1.002 (0.963 to 1.043)	0.923		
Epiandrosterone sulfate	Hypertension	9	1.012 (0.983 to 1.042)	0.455	1.014 (0.999 to 1.029)	0.062	1.016 (1.000 to 1.032)	0.057		
Linolenate [alpha or gamma; (18:3n3 or 6)]	Hypertension	3	0.709 (0.419 to 1.198)	0.421	0.961 (0.878 to 1.051)	0.383	0.937 (0.802 to 1.094)	0.410		
X-12510--2-aminooctanoic acid	Hypertension	11	0.987 (0.963 to 1.013)	0.349	0.982 (0.967 to 0.998)	0.027	0.980 (0.964 to 0.996)	0.014		

Exposure	Outcome	nSNP	MR Egger		Weighted median		Inverse variance weighted		MR_PRESSO	
			OR (95% CI)	pval	OR (95% CI)	pval	OR (95% CI)	pval	OR (95% CI)	pval
X-12524	Hypertension	15	1.006 (0.904 to 1.120)	0.913	1.027 (0.963 to 1.095)	0.423	1.044 (0.996 to 1.095)	0.072		
Bilirubin (E,Z or Z,E)*	Hypertension	8	0.977 (0.944 to 1.011)	0.234	0.991 (0.972 to 1.011)	0.379	0.998 (0.981 to 1.016)	0.850		
X-12544	Hypertension	10	1.006 (0.985 to 1.029)	0.582	1.003 (0.984 to 1.023)	0.752	1.005 (0.991 to 1.019)	0.465		
X-12556	Hypertension	13	1.049 (0.868 to 1.267)	0.633	0.984 (0.938 to 1.033)	0.517	0.968 (0.912 to 1.027)	0.276	0.986 (0.945 to 1.029)	0.532
1-arachidonoylglycerophosphoinositol*	Hypertension	14	0.945 (0.850 to 1.050)	0.311	0.975 (0.940 to 1.011)	0.165	0.998 (0.960 to 1.038)	0.935	0.987 (0.960 to 1.016)	0.399
X-12627	Hypertension	8	0.995 (0.918 to 1.078)	0.899	1.014 (0.982 to 1.047)	0.402	1.015 (0.991 to 1.040)	0.217		

Exposure	Outcome	nSNP	MR Egger		Weighted median		Inverse variance weighted		MR_PRESSO	
			OR (95% CI)	pval	OR (95% CI)	pval	OR (95% CI)	pval	OR (95% CI)	pval
X-12644	Hypertension	13	1.017 (0.943 to 1.096)	0.677	1.008 (0.970 to 1.048)	0.687	0.995 (0.967 to 1.023)	0.710		
X-12645	Hypertension	10	1.024 (0.948 to 1.107)	0.560	1.009 (0.977 to 1.041)	0.591	1.023 (0.993 to 1.055)	0.134	1.015 (0.991 to 1.039)	0.259
Asparagine	Hypertension	25	0.996 (0.922 to 1.076)	0.917	0.994 (0.949 to 1.041)	0.794	1.008 (0.968 to 1.050)	0.704	0.998 (0.968 to 1.029)	0.888
X-12680	Hypertension	9	0.988 (0.914 to 1.068)	0.769	0.993 (0.961 to 1.026)	0.688	0.985 (0.954 to 1.017)	0.352		
X-12696	Hypertension	13	1.010 (0.935 to 1.092)	0.802	0.984 (0.942 to 1.028)	0.475	0.985 (0.955 to 1.015)	0.323		
X-12704	Hypertension	4	0.965 (0.920 to 1.012)	0.279	0.983 (0.964 to 1.002)	0.084	0.981 (0.966 to 0.997)	0.018		

Exposure	Outcome	nSNP	MR Egger		Weighted median		Inverse variance weighted		MR_PRESSO	
			OR (95% CI)	pval	OR (95% CI)	pval	OR (95% CI)	pval	OR (95% CI)	pval
X-12712	Hypertension	20	1.001 (0.992 to 1.010)	0.835	0.999 (0.995 to 1.004)	0.794	0.999 (0.994 to 1.003)	0.525		
X-12717	Hypertension	4	1.328 (0.622 to 2.835)	0.540	0.991 (0.973 to 1.009)	0.321	0.995 (0.974 to 1.016)	0.650		
X-12719	Hypertension	4	1.009 (0.948 to 1.073)	0.813	0.995 (0.972 to 1.019)	0.682	0.983 (0.963 to 1.004)	0.108		
X-12726	Hypertension	12	1.008 (0.991 to 1.025)	0.381	1.006 (0.992 to 1.019)	0.428	1.006 (0.992 to 1.019)	0.417		
X-12728	Hypertension	32	1.000 (0.998 to 1.001)	0.838	1.000 (0.999 to 1.001)	0.965	1.000 (0.999 to 1.001)	0.964		
X-12729	Hypertension	13	0.992 (0.981 to 1.003)	0.190	0.999 (0.991 to 1.008)	0.890	0.997 (0.991 to 1.004)	0.413		

Exposure	Outcome	nSNP	MR Egger		Weighted median		Inverse variance weighted		MR_PRESSO	
			OR (95% CI)	pval	OR (95% CI)	pval	OR (95% CI)	pval	OR (95% CI)	pval
X-12734	Hypertension	4	1.007 (0.955 to 1.063)	0.816	1.004 (0.987 to 1.021)	0.677	1.003 (0.989 to 1.018)	0.682		
X-12740	Hypertension	10	1.012 (0.981 to 1.045)	0.460	0.999 (0.985 to 1.013)	0.872	0.992 (0.974 to 1.010)	0.390	0.992 (0.977 to 1.007)	0.307
X-12749	Hypertension	26	1.034 (0.896 to 1.194)	0.650	1.027 (0.974 to 1.083)	0.330	1.017 (0.965 to 1.072)	0.529	1.016 (0.972 to 1.063)	0.483
Stachydrine	Hypertension	2					0.990 (0.955 to 1.027)	0.594		
Isovalerylcarnitine	Hypertension	14	1.070 (1.005 to 1.138)	0.055	1.021 (0.985 to 1.057)	0.255	1.023 (0.986 to 1.061)	0.221	1.009 (0.985 to 1.034)	0.459
Stearoylcarnitine	Hypertension	4	1.643 (0.940 to 2.874)	0.224	1.036 (0.959 to 1.120)	0.373	1.085 (0.983 to 1.198)	0.105		

Exposure	Outcome	nSNP	MR Egger		Weighted median		Inverse variance weighted		MR_PRESSO	
			OR (95% CI)	pval	OR (95% CI)	pval	OR (95% CI)	pval	OR (95% CI)	pval
1-stearoylglycerophosphoethanolamine	Hypertension	9	1.026 (0.860 to 1.226)	0.782	1.007 (0.973 to 1.042)	0.694	0.991 (0.934 to 1.051)	0.755	0.997 (0.975 to 1.019)	0.771
1-linoleoylglycerophosphocholine	Hypertension	10	0.875 (0.749 to 1.023)	0.133	0.966 (0.908 to 1.029)	0.282	0.973 (0.926 to 1.021)	0.266		
Bradykinin, des-arg(9)	Hypertension	14	1.012 (0.985 to 1.040)	0.409	1.000 (0.990 to 1.011)	0.964	1.004 (0.991 to 1.016)	0.555	1.000 (0.990 to 1.010)	0.999
X-12771	Hypertension	9	1.005 (0.958 to 1.055)	0.847	0.999 (0.977 to 1.021)	0.915	0.996 (0.980 to 1.013)	0.651		
X-12776	Hypertension	2					1.141 (1.007 to 1.293)	0.039		
Gamma-glutamylisoleucine*	Hypertension	15	1.099 (1.017 to 1.189)	0.033	1.002 (0.961 to 1.045)	0.925	0.993 (0.959 to 1.027)	0.667		

Exposure	Outcome	nSNP	MR Egger		Weighted median		Inverse variance weighted		MR_PRESSO	
			OR (95% CI)	pval	OR (95% CI)	pval	OR (95% CI)	pval	OR (95% CI)	pval
X-12786	Hypertension	6	0.952 (0.878 to 1.032)	0.300	0.985 (0.953 to 1.019)	0.379	0.976 (0.933 to 1.021)	0.285	1.001 (0.955 to 1.049)	0.972
X-12798	Hypertension	6	1.007 (0.980 to 1.036)	0.635	0.996 (0.982 to 1.011)	0.607	0.989 (0.965 to 1.014)	0.406		
X-12816	Hypertension	3	0.998 (0.973 to 1.023)	0.905	1.006 (0.990 to 1.023)	0.459	1.008 (0.994 to 1.022)	0.266		
X-12830	Hypertension	8	0.991 (0.952 to 1.032)	0.686	0.993 (0.972 to 1.015)	0.519	0.997 (0.981 to 1.014)	0.754		
X-12833	Hypertension	9	0.994 (0.986 to 1.002)	0.206	0.998 (0.992 to 1.003)	0.443	0.999 (0.994 to 1.004)	0.698		
X-12844	Hypertension	7	0.963 (0.834 to 1.111)	0.627	0.970 (0.931 to 1.010)	0.141	0.984 (0.952 to 1.017)	0.325		

Exposure	Outcome	nSNP	MR Egger		Weighted median		Inverse variance weighted		MR_PRESSO	
			OR (95% CI)	pval	OR (95% CI)	pval	OR (95% CI)	pval	OR (95% CI)	pval
X-12847	Hypertension	2					0.991 (0.953 to 1.030)	0.642		
X-12850	Hypertension	8	0.967 (0.939 to 0.995)	0.064	1.008 (0.986 to 1.030)	0.475	1.000 (0.978 to 1.023)	0.987	1.009 (0.995 to 1.024)	0.268
Laurylcarnitine	Hypertension	7	0.939 (0.687 to 1.285)	0.712	1.038 (0.998 to 1.079)	0.063	1.042 (0.992 to 1.095)	0.102	1.020 (0.993 to 1.047)	0.210
X-12851	Hypertension	4	0.931 (0.673 to 1.290)	0.711	1.027 (0.994 to 1.060)	0.106	1.038 (1.003 to 1.075)	0.035		
X-12855	Hypertension	12	0.991 (0.851 to 1.155)	0.913	0.998 (0.965 to 1.032)	0.920	0.992 (0.958 to 1.027)	0.634	1.001 (0.971 to 1.032)	0.959
X-12990--docosapentaenoic acid (n6-DPA)	Hypertension	6	1.011 (0.912 to 1.121)	0.842	1.000 (0.970 to 1.031)	0.997	1.004 (0.979 to 1.028)	0.776		

Exposure	Outcome	nSNP	MR Egger		Weighted median		Inverse variance weighted		MR_PRESSO	
			OR (95% CI)	pval	OR (95% CI)	pval	OR (95% CI)	pval	OR (95% CI)	pval
Isovalerate	Hypertension	2					0.995 (0.921 to 1.075)	0.895		
X-13069	Hypertension	11	0.919 (0.809 to 1.044)	0.226	0.983 (0.948 to 1.019)	0.353	0.973 (0.934 to 1.014)	0.199	0.972 (0.944 to 1.000)	0.089
X-13183--stearamide	Hypertension	9	1.005 (0.972 to 1.040)	0.761	1.010 (0.988 to 1.032)	0.373	1.011 (0.996 to 1.026)	0.166		
X-13215	Hypertension	27	1.062 (0.989 to 1.141)	0.109	1.051 (1.003 to 1.102)	0.039	1.045 (1.014 to 1.076)	0.004		
7-methylguanine	Hypertension	4	1.073 (0.980 to 1.174)	0.268	1.006 (0.959 to 1.055)	0.814	1.007 (0.969 to 1.047)	0.725		
Phenylacetylglutamine	Hypertension	11	0.973 (0.901 to 1.052)	0.508	0.986 (0.961 to 1.012)	0.297	1.003 (0.964 to 1.044)	0.876	0.990 (0.971 to 1.010)	0.368

Exposure	Outcome	nSNP	MR Egger		Weighted median		Inverse variance weighted		MR_PRESSO	
			OR (95% CI)	pval	OR (95% CI)	pval	OR (95% CI)	pval	OR (95% CI)	pval
Pro-hydroxy-pro	Hypertension	11	0.987 (0.884 to 1.102)	0.820	1.002 (0.959 to 1.046)	0.940	0.997 (0.957 to 1.038)	0.872		
N2,N2-dimethylguanosine	Hypertension	35	0.954 (0.895 to 1.016)	0.154	0.958 (0.916 to 1.002)	0.059	0.972 (0.939 to 1.006)	0.102	0.977 (0.946 to 1.009)	0.169
Cysteine-glutathione disulfide	Hypertension	7	1.049 (0.937 to 1.174)	0.444	1.005 (0.981 to 1.029)	0.683	0.996 (0.977 to 1.016)	0.686		
Oleoylcarnitine	Hypertension	5	1.098 (0.460 to 2.618)	0.847	1.012 (0.953 to 1.075)	0.694	1.052 (0.971 to 1.140)	0.213	1.000 (0.971 to 1.030)	0.987
1-arachidonoylglycerophosphoethanolamine*	Hypertension	14	0.939 (0.874 to 1.009)	0.112	0.983 (0.945 to 1.022)	0.389	1.003 (0.975 to 1.033)	0.816		
X-13429	Hypertension	5	0.982 (0.949 to 1.016)	0.367	0.997 (0.984 to 1.011)	0.713	0.998 (0.986 to 1.009)	0.692		

Exposure	Outcome	nSNP	MR Egger		Weighted median		Inverse variance weighted		MR_PRESSO	
			OR (95% CI)	pval	OR (95% CI)	pval	OR (95% CI)	pval	OR (95% CI)	pval
X-13431--nonanoylcarnitine*	Hypertension	10	1.020 (0.967 to 1.077)	0.487	1.000 (0.985 to 1.015)	0.980	0.995 (0.965 to 1.026)	0.739	1.001 (0.987 to 1.016)	0.856
X-13435	Hypertension	11	0.960 (0.900 to 1.023)	0.240	0.988 (0.956 to 1.022)	0.495	1.009 (0.969 to 1.051)	0.656	1.018 (0.991 to 1.046)	0.236
X-13477	Hypertension	4	0.985 (0.700 to 1.387)	0.940	0.970 (0.891 to 1.055)	0.478	1.006 (0.887 to 1.142)	0.920	1.033 (0.944 to 1.129)	0.609
2-palmitoylglycerophosphocholine*	Hypertension	15	0.893 (0.768 to 1.039)	0.168	1.025 (0.972 to 1.081)	0.363	1.011 (0.958 to 1.067)	0.692	1.027 (0.978 to 1.078)	0.307
2-oleoylglycerophosphocholine*	Hypertension	10	1.150 (1.006 to 1.315)	0.075	0.980 (0.925 to 1.038)	0.485	0.974 (0.919 to 1.031)	0.365	0.951 (0.909 to 0.994)	0.059
2-stearoylglycerophosphocholine*	Hypertension	9	0.910 (0.762 to 1.087)	0.334	0.970 (0.932 to 1.009)	0.126	0.964 (0.933 to 0.995)	0.024		

Exposure	Outcome	nSNP	MR Egger		Weighted median		Inverse variance weighted		MR_PRESSO	
			OR (95% CI)	pval	OR (95% CI)	pval	OR (95% CI)	pval	OR (95% CI)	pval
2-linoleoylglycerophosphocholine*	Hypertension	13	1.001 (0.905 to 1.106)	0.992	1.020 (0.979 to 1.063)	0.338	1.028 (0.997 to 1.060)	0.077		
X-13496	Hypertension	29	1.180 (1.025 to 1.359)	0.029	1.025 (0.966 to 1.086)	0.416	1.078 (1.034 to 1.125)	4.9e-04	1.054 (1.012 to 1.097)	0.017
1-palmitoylglycerophosphoinositol*	Hypertension	5	0.984 (0.921 to 1.051)	0.659	1.001 (0.967 to 1.036)	0.954	0.986 (0.956 to 1.017)	0.374		
X-13548	Hypertension	28	1.034 (0.912 to 1.173)	0.604	1.028 (0.975 to 1.084)	0.300	1.017 (0.976 to 1.060)	0.414		
X-13549	Hypertension	11	0.911 (0.727 to 1.142)	0.441	0.963 (0.889 to 1.044)	0.363	0.985 (0.914 to 1.062)	0.689		
X-13553	Hypertension	2					0.990 (0.953 to 1.029)	0.612		

Exposure	Outcome	nSNP	MR Egger		Weighted median		Inverse variance weighted		MR_PRESSO	
			OR (95% CI)	pval	OR (95% CI)	pval	OR (95% CI)	pval	OR (95% CI)	pval
X-13619	Hypertension	17	1.067 (0.825 to 1.379)	0.629	1.013 (0.936 to 1.096)	0.754	0.979 (0.906 to 1.058)	0.594	1.003 (0.940 to 1.069)	0.939
2-methylbutyroylcarnitine	Hypertension	16	0.801 (0.610 to 1.050)	0.131	1.020 (0.966 to 1.077)	0.469	1.096 (1.005 to 1.197)	0.039	1.035 (0.986 to 1.087)	0.189
Hydroxyisovaleroyl carnitine	Hypertension	4	0.917 (0.859 to 0.979)	0.122	1.005 (0.950 to 1.064)	0.855	1.032 (0.951 to 1.120)	0.456	1.048 (0.999 to 1.100)	0.305
Glutaroyl carnitine	Hypertension	21	1.051 (0.977 to 1.132)	0.198	1.023 (0.995 to 1.051)	0.105	1.019 (0.987 to 1.052)	0.254	1.002 (0.982 to 1.023)	0.834
X-13658	Hypertension	9	1.003 (0.998 to 1.008)	0.316	1.001 (0.998 to 1.004)	0.432	1.001 (0.998 to 1.004)	0.699		
X-13671	Hypertension	10	1.027 (0.876 to 1.203)	0.752	1.006 (0.951 to 1.064)	0.843	0.995 (0.954 to 1.038)	0.816		

Exposure	Outcome	nSNP	MR Egger		Weighted median		Inverse variance weighted		MR_PRESSO	
			OR (95% CI)	pval	OR (95% CI)	pval	OR (95% CI)	pval	OR (95% CI)	pval
2-tetradecenoyl carnitine	Hypertension	11	0.961 (0.900 to 1.027)	0.273	0.982 (0.958 to 1.006)	0.142	0.998 (0.968 to 1.029)	0.910	0.986 (0.972 to 1.000)	0.082
4-hydroxyhippurate	Hypertension	3	0.972 (0.868 to 1.088)	0.706	1.033 (0.998 to 1.068)	0.064	1.025 (0.999 to 1.052)	0.064		
X-13741	Hypertension	8	0.893 (0.779 to 1.023)	0.155	0.987 (0.962 to 1.013)	0.324	1.019 (0.979 to 1.062)	0.356	1.012 (0.983 to 1.043)	0.460
1-myristoylglycerophosphocholine	Hypertension	3	0.978 (0.910 to 1.050)	0.647	0.999 (0.963 to 1.037)	0.968	1.016 (0.949 to 1.089)	0.642		
1-oleoylglycerophosphoethanolamine	Hypertension	4	0.919 (0.525 to 1.607)	0.795	0.994 (0.935 to 1.056)	0.834	1.020 (0.933 to 1.116)	0.658	0.980 (0.936 to 1.026)	0.476
1-palmitoylglycerophosphoethanolamine	Hypertension	14	0.919 (0.842 to 1.002)	0.080	1.023 (0.981 to 1.066)	0.294	1.023 (0.976 to 1.073)	0.345	1.054 (1.013 to 1.096)	0.024

Exposure	Outcome	nSNP	MR Egger		Weighted median		Inverse variance weighted		MR_PRESSO	
			OR (95% CI)	pval	OR (95% CI)	pval	OR (95% CI)	pval	OR (95% CI)	pval
3-(3-hydroxyphenyl)propionate	Hypertension	9	0.986 (0.921 to 1.056)	0.697	0.994 (0.982 to 1.005)	0.266	1.001 (0.976 to 1.027)	0.944	0.992 (0.982 to 1.002)	0.164
Tetradecanedioate	Hypertension	13	0.990 (0.969 to 1.012)	0.389	0.991 (0.976 to 1.007)	0.260	1.002 (0.988 to 1.016)	0.790		
2-hydroxypalmitate	Hypertension	30	1.022 (0.950 to 1.098)	0.569	1.012 (0.963 to 1.063)	0.636	1.005 (0.969 to 1.041)	0.803		
Hexadecanedioate	Hypertension	14	0.968 (0.921 to 1.017)	0.221	0.989 (0.969 to 1.009)	0.288	1.012 (0.984 to 1.040)	0.420	0.999 (0.984 to 1.015)	0.944
Dihomo-linolenate (20:3n3 or n6)	Hypertension	14	1.004 (0.910 to 1.107)	0.942	1.012 (0.970 to 1.057)	0.572	1.006 (0.973 to 1.041)	0.713		
X-13859	Hypertension	28	0.995 (0.914 to 1.084)	0.916	1.001 (0.957 to 1.048)	0.953	1.010 (0.971 to 1.050)	0.626		

Exposure	Outcome	nSNP	MR Egger		Weighted median		Inverse variance weighted		MR_PRESSO	
			OR (95% CI)	pval	OR (95% CI)	pval	OR (95% CI)	pval	OR (95% CI)	pval
Threitol	Hypertension	3	0.485 (0.179 to 1.310)	0.389	1.012 (0.944 to 1.084)	0.739	0.989 (0.938 to 1.043)	0.691		
X-14056	Hypertension	5	1.016 (0.906 to 1.140)	0.798	1.042 (0.996 to 1.089)	0.072	1.042 (0.988 to 1.098)	0.133		
X-14057	Hypertension	5	1.033 (0.927 to 1.152)	0.597	1.015 (0.977 to 1.055)	0.446	1.028 (0.989 to 1.068)	0.160		
X-14086	Hypertension	9	0.896 (0.799 to 1.004)	0.101	1.010 (0.983 to 1.038)	0.475	1.014 (0.974 to 1.056)	0.491	1.008 (1.000 to 1.017)	0.107
P-cresol sulfate	Hypertension	8	0.998 (0.963 to 1.035)	0.921	1.009 (0.987 to 1.032)	0.437	1.009 (0.987 to 1.031)	0.438		
X-14189--leucylalanine	Hypertension	8	0.993 (0.927 to 1.063)	0.844	1.010 (0.996 to 1.024)	0.147	1.017 (0.980 to 1.056)	0.369	1.005 (0.990 to 1.021)	0.524

Exposure	Outcome	nSNP	MR Egger		Weighted median		Inverse variance weighted		MR_PRESSO	
			OR (95% CI)	pval	OR (95% CI)	pval	OR (95% CI)	pval	OR (95% CI)	pval
X-14205--alpha-glutamyltyrosine	Hypertension	7	1.100 (0.979 to 1.236)	0.168	1.027 (0.992 to 1.065)	0.136	1.047 (1.004 to 1.091)	0.032	1.016 (0.997 to 1.036)	0.194
X-14208--phenylalanylserine	Hypertension	10	1.009 (0.952 to 1.069)	0.775	1.013 (0.995 to 1.031)	0.161	1.026 (0.996 to 1.058)	0.094	1.006 (0.993 to 1.020)	0.389
X-14304--leucylalanine	Hypertension	10	1.023 (0.962 to 1.089)	0.488	1.015 (0.993 to 1.037)	0.175	1.025 (1.003 to 1.046)	0.023	1.007 (1.001 to 1.014)	0.065
X-14374	Hypertension	6	0.944 (0.847 to 1.052)	0.359	1.024 (0.976 to 1.074)	0.335	1.006 (0.969 to 1.045)	0.747		
X-14450--phenylalanylleucine	Hypertension	4	1.031 (0.918 to 1.157)	0.659	0.990 (0.965 to 1.016)	0.443	0.994 (0.973 to 1.015)	0.559		
X-14473	Hypertension	9	1.011 (0.909 to 1.124)	0.850	0.981 (0.954 to 1.010)	0.199	0.992 (0.957 to 1.027)	0.639	0.975 (0.959 to 0.992)	0.022

Exposure	Outcome	nSNP	MR Egger		Weighted median		Inverse variance weighted		MR_PRESSO	
			OR (95% CI)	pval	OR (95% CI)	pval	OR (95% CI)	pval	OR (95% CI)	pval
X-14541	Hypertension	11	0.996 (0.949 to 1.045)	0.878	0.995 (0.977 to 1.013)	0.590	0.998 (0.984 to 1.012)	0.775		
X-14588	Hypertension	6	0.863 (0.671 to 1.109)	0.314	0.965 (0.821 to 1.135)	0.666	1.009 (0.891 to 1.143)	0.889		
X-14625	Hypertension	10	1.334 (1.115 to 1.595)	0.014	1.053 (0.963 to 1.151)	0.257	1.027 (0.919 to 1.148)	0.638	1.010 (0.935 to 1.091)	0.802
X-14626	Hypertension	10	0.945 (0.856 to 1.044)	0.302	0.992 (0.955 to 1.031)	0.697	0.979 (0.932 to 1.028)	0.390	0.993 (0.958 to 1.031)	0.734
X-14632	Hypertension	13	1.013 (0.994 to 1.033)	0.216	1.006 (0.991 to 1.022)	0.428	0.998 (0.984 to 1.013)	0.796		
X-14658	Hypertension	7	1.001 (0.908 to 1.103)	0.989	1.010 (0.980 to 1.040)	0.533	1.015 (0.983 to 1.049)	0.360	1.028 (1.002 to 1.054)	0.090

Exposure	Outcome	nSNP	MR Egger		Weighted median		Inverse variance weighted		MR_PRESSO	
			OR (95% CI)	pval	OR (95% CI)	pval	OR (95% CI)	pval	OR (95% CI)	pval
X-14662	Hypertension	11	1.004 (0.983 to 1.025)	0.714	1.002 (0.990 to 1.015)	0.735	1.003 (0.990 to 1.016)	0.623		
X-14745	Hypertension	7	0.958 (0.742 to 1.236)	0.754	0.997 (0.955 to 1.041)	0.894	0.990 (0.956 to 1.025)	0.563		
Gamma-glutamylglutamate	Hypertension	8	0.958 (0.884 to 1.038)	0.331	1.003 (0.977 to 1.029)	0.835	0.991 (0.967 to 1.015)	0.444		
Octadecanedioate	Hypertension	5	1.010 (0.865 to 1.180)	0.905	0.989 (0.948 to 1.032)	0.621	0.987 (0.928 to 1.050)	0.682	0.993 (0.945 to 1.043)	0.794
Leucylleucine	Hypertension	8	0.952 (0.801 to 1.130)	0.593	1.013 (0.971 to 1.056)	0.556	1.000 (0.958 to 1.045)	0.983	0.987 (0.952 to 1.023)	0.494
7-alpha-hydroxy-3-oxo-4-cholestenoate (7-Hoca)	Hypertension	8	1.156 (0.942 to 1.420)	0.214	1.020 (0.949 to 1.097)	0.585	1.010 (0.939 to 1.087)	0.785		

Exposure	Outcome	nSNP	MR Egger		Weighted median		Inverse variance weighted		MR_PRESSO	
			OR (95% CI)	pval	OR (95% CI)	pval	OR (95% CI)	pval	OR (95% CI)	pval
N-Butyl Oleate	Hypertension	5	1.222 (0.783 to 1.906)	0.442	0.957 (0.896 to 1.023)	0.196	0.971 (0.915 to 1.031)	0.337		
Dimethylarginine (SDMA + ADMA)	Hypertension	21	1.068 (0.867 to 1.314)	0.545	1.038 (0.968 to 1.113)	0.291	0.997 (0.925 to 1.075)	0.947	1.026 (0.964 to 1.093)	0.427
Taurolithocholate 3-sulfate	Hypertension	6	0.993 (0.967 to 1.018)	0.597	0.987 (0.967 to 1.007)	0.194	0.989 (0.974 to 1.004)	0.149		
X-14977--vanillin	Hypertension	2					1.031 (0.989 to 1.074)	0.149		
Succinylcarnitine	Hypertension	27	1.037 (0.939 to 1.145)	0.482	1.043 (1.002 to 1.087)	0.041	1.055 (1.008 to 1.105)	0.021	1.039 (1.003 to 1.076)	0.044
Tryptophan betaine	Hypertension	7	0.991 (0.935 to 1.050)	0.771	0.989 (0.975 to 1.004)	0.149	0.981 (0.964 to 0.998)	0.027	0.981 (0.972 to 0.991)	0.019

Exposure	Outcome	nSNP	MR Egger		Weighted median		Inverse variance weighted		MR_PRESSO	
			OR (95% CI)	pval	OR (95% CI)	pval	OR (95% CI)	pval	OR (95% CI)	pval
Cyclo(leu-pro)	Hypertension	10	0.971 (0.898 to 1.048)	0.469	0.980 (0.958 to 1.002)	0.076	1.003 (0.975 to 1.032)	0.831	0.992 (0.974 to 1.010)	0.410
Chiro-inositol	Hypertension	6	1.019 (0.995 to 1.043)	0.191	1.001 (0.991 to 1.011)	0.892	0.997 (0.989 to 1.005)	0.448		
5alpha-androstan-3beta,17beta-diol disulfate	Hypertension	10	1.017 (0.977 to 1.057)	0.437	1.012 (0.997 to 1.027)	0.127	1.009 (0.990 to 1.029)	0.341	1.004 (0.991 to 1.018)	0.561
4-androsten-3beta,17beta-diol disulfate 1*	Hypertension	14	1.012 (0.990 to 1.034)	0.309	1.004 (0.992 to 1.016)	0.489	1.003 (0.990 to 1.016)	0.692	1.006 (0.996 to 1.016)	0.285
4-androsten-3beta,17beta-diol disulfate 2*	Hypertension	13	0.995 (0.859 to 1.153)	0.951	0.983 (0.951 to 1.016)	0.305	0.972 (0.932 to 1.014)	0.195	0.981 (0.947 to 1.015)	0.295
2-hydroxyglutarate	Hypertension	5	1.111 (1.017 to 1.215)	0.102	1.040 (0.996 to 1.087)	0.077	1.026 (0.979 to 1.074)	0.280		
Ergothioneine	Hypertension	1								

Exposure	Outcome	nSNP	MR Egger		Weighted median		Inverse variance weighted		MR_PRESSO	
			OR (95% CI)	pval	OR (95% CI)	pval	OR (95% CI)	pval	OR (95% CI)	pval
Palmitoyl sphingomyelin	Hypertension	30	1.155 (0.973 to 1.371)	0.112	1.022 (0.968 to 1.079)	0.432	1.054 (1.002 to 1.108)	0.043	1.030 (0.983 to 1.079)	0.231
Phenylalanylphenylalanine	Hypertension	3	1.006 (0.798 to 1.268)	0.969	1.028 (0.980 to 1.078)	0.255	1.025 (0.975 to 1.078)	0.331		
Cis-4-decenoyl carnitine	Hypertension	6	1.033 (0.981 to 1.089)	0.284	1.023 (0.995 to 1.051)	0.111	1.019 (0.996 to 1.043)	0.104		
15-methylpalmitate (isobar with 2-methylpalmitate)	Hypertension	9	0.928 (0.813 to 1.060)	0.308	1.016 (0.960 to 1.075)	0.586	1.022 (0.981 to 1.064)	0.302		

n: 2187
